# Comparison of Epithor clinical national database and medico-administrative database to identify the influence of case-mix on the estimation of hospital outliers

**DOI:** 10.1371/journal.pone.0219672

**Published:** 2019-07-24

**Authors:** Alain Bernard, Pierre-Emmanuel Falcoz, Pascal Antoine Thomas, Caroline Rivera, Laurent Brouchet, Jean Marc Baste, Marc Puyraveau, Catherine Quantin, Pierre Benoit Pages, Marcel Dahan

**Affiliations:** 1 Department of Thoracic Surgery, Dijon University Hospital, Dijon, France; 2 Department of Thoracic Surgery, CHRU Strasbourg, Strasbourg, France; 3 Department of Thoracic Surgery, Hopital-Nord-APHM, Aix-Marseille University, Marseille, France; 4 Department of Thoracic Surgery, Bayonne Hospital, Bayonne, France; 5 Department of Thoracic Surgery, Hopital Larrey, CHU Toulouse, Toulouse, France; 6 Department of Thoracic Surgery, CHU Rouen, Rouen, France; 7 Department of Biostatistics and Epidemiology CHU Besançon, Besançon, France; 8 Department of Biostatistics and Medical Informatics, Dijon University Hospital, Dijon, France; 9 INSERM, CIC 1432, Clinical Investigation Center, clinical epidemiology/clinical trials unit, Dijon University Hospital, University of Burgundy, Dijon, France; 10 INSERM UMR 866, Dijon University Hospital, University of Burgundy, Dijon, France; Royal Infirmary of Edinburgh, UNITED KINGDOM

## Abstract

**Background:**

The national Epithor database was initiated in 2003 in France. Fifteen years on, a quality assessment of the recorded data seemed necessary. This study examines the completeness of the data recorded in Epithor through a comparison with the French PMSI database, which is the national medico-administrative reference database. The aim of this study was to demonstrate the influence of data quality with respect to identifying 30-day mortality hospital outliers.

**Methods:**

We used each hospital’s individual FINESS code to compare the number of pulmonary resections and deaths recorded in Epithor to the figures found in the PMSI. Centers were classified into either the good-quality data (GQD) group or the low-quality data (LQD) group. To demonstrate the influence of case-mix quality on the ranking of centers with low-quality data, we used 2 methods to estimate the standardized mortality rate (SMR). For the first (SMR1), the expected number of deaths per hospital was estimated with risk-adjustment models fitted with low-quality data. For the second (SMR2), the expected number of deaths per hospital was estimated with a linear predictor for the LQD group using the coefficients of a logistic regression model developed from the GQD group.

**Results:**

Of the hospitals that use Epithor, 25 were classified in the GQD group and 75 in the LQD group. The 30-day mortality rate was 2.8% (n = 300) in the GQD group vs. 1.9% (n = 181) in the LQD group (P <0.0001). The between-hospital differences in SMR1 appeared substantial (interquartile range (IQR) 0–1.036), and they were even higher in SMR2 (IQR 0–1.19). SMR1 identified 7 hospitals as high-mortality outliers. SMR2 identified 4 hospitals as high-mortality outliers. Some hospitals went from non-outlier to high mortality and vice-versa. Kappa values were roughly 0.46 and indicated moderate agreement.

**Conclusion:**

We found that most hospitals provided Epithor with high-quality data, but other hospitals needed to improve the quality of the information provided. Quality control is essential for this type of database and necessary for the unbiased adjustment of regression models.

## Introduction

Epithor, a French national database for thoracic surgery, has been in operation since 2003. Participation is on a voluntary basis. It has led to the publication of several research articles [1–6]) and, most notably, Falcoz et al. [7] used it to develop the Thoracoscore, a predictive score that is widely used by European surgeons according to the latest European recommendations [8].A number of existing publications have highlighted the importance of data quality in medical databases [9,10], particularly since missing or biased data can lead to erroneous conclusions regarding hospital quality [11,12]. At this stage of development, it seems to us that the quality of the data within the Epithor database needs to be assessed. We therefore used the "Programme de Medicalisation des Systèmes d’information" (PMSI), a French national medico-administrative database that collects exhaustive data regarding pulmonary resection for lung cancer, as a point of reference [13]. Comparing the two databases will make it possible to identify the hospitals participating in Epithor that provide insufficient data concerning the lung cancer patients treated in their facility. The aim of this study was to rank hospitals according to the completeness of the data and to estimate the influence of data quality with respect to identifying 30-day mortality outliers.

## Materials and methods

### National medico-administrative database

All the data for patients who underwent pulmonary resection for lung cancer (LC) from January 2016 to December 2017 were collected from the French national medico-administrative database. The completeness and validity of PMSI data have already been assessed [13]. Routinely collected medical information includes the principal diagnosis, secondary diagnoses and the procedure performed on the patient. Diagnoses identified during the hospital stay are coded according to the International Classification of Diseases, tenth revision (ICD-10) [14]. We selected patients with a principal discharge diagnosis of primary lung cancer (codes C34, C34.0, C34.1, C34.2, C34.3, and C34.9). Procedures were coded according to the CCAM (Classification Commune des Actes Médicaux). For all patients, LC was confirmed by pathology analyses according to the 2004 World Health Organization classification of LC [15]. Surgery-related variables included the surgical approach (thoracotomy or video assisted thoracic surgery), and the type of resection (limited resection, lobectomy, bi-lobectomy or pneumonectomy). In this study, data access was based on special permissions given by the National Health Insurance. All data were completely anonymous. Patients consent is not required. Ethics approval, use of this database was approved by the National Commission for Data protection (CNIL No 1576793) and this study adhered to the tenets of the declaration of Helsinsksi.

### The Epithor national clinical database

Epithor was modified in 2016 so that each surgeon could upload their patient data directly to a website called “web Epithor”. Between 1^st^ January 2016 and 31^st^ December 2017, 100 centers that operated patients for lung cancer provided data to Epithor. Baseline demographic data included sex, age, body mass index (BMI), performance status, American Society of Anesthesiologists (ASA) score, forced expiratory volume (FEV), dyspnea score, and smoking status. The comorbidities selected for our analysis were smoking status, chronic bronchitis, arrhythmia, chronic heart failure, peripheral artery disease, alcoholism, cirrhosis, stroke, diabetes, coagulopathy, hematologic disease, history of neoplasm, surgical history, severe malnutrition, pulmonary embolism, valvulopathy, neurological history, psychiatric history, asthma, respiratory failure, infectious disease, cardiac malformation, endocrine disorder, anemia, immunosuppression, and steroid treatment. The details of the surgery included surgical approach (open thoracotomy or video-assisted thoracoscopy) and type of surgery (segmentectomy, lobectomy, bilobectomy, or pneumonectomy). For clinical database, all data were completely anonymous. Patients consent is not required. Ethics approval, use of this database was approved by the National Commission for Data protection (CNIL No 809833) and this study adhered to the tenets of the declaration of Helsinki.

### Outcome definition

In-hospital mortality was defined as the death of a patient within 30 days of the surgery or at a later time but during the same hospital stay.

### Comparison of databases

The PMSI database was used to classify the hospitals that participated in data collection for the Epithor database. We used hospital FINESS codes to compare the number of pulmonary resections and related deaths recorded in Epithor to the figures recorded in the PMSI during the same period. We calculated the ratio of the number of lung resections in the PMSI to the number of lung resections in Epithor. For deaths, the calculation of the ratio was the same as for the number of pulmonary resections. The hospitals in the Epithor database were then divided into two groups according to the ratios obtained for pulmonary resections and deaths. Group 1was considered good-quality data (GQD) and included hospitals with ratios between 1 and 0.7 for both measures (75th percentile). Group 2 was defined as low-quality data (LQD) and included the hospitals with at least one of the two ratios below 0.7.

### Risk-adjustment models

We applied a multiple imputation framework to compensate for missing FEV data. For the variable TNM stage, we created a category for all cases with missing TNM data. We developed a logistic regression model from the centers in the GQD group. Variables with a level of significance of ≤0.1 in univariate analyses were included in multivariate analyses by means of logistic regression. Continuous or ranked variables were tested to ensure conformity with the linear gradient by using the likelihood-ratio chi-squared test. Interaction effects were sought for all variables included in the model. All models were constructed using backward stepwise variable selection. A step-down variable selection using Akaike’s information criterion was used as a stopping rule. The area under the receiver operating characteristic (ROC) curve, and the R2 value were used to measure the discriminatory ability of the model. The reliability of the model was assessed with the Hosmer Lemeshow goodness-of-fit test [16].

### Identification of quality outliers

The standardized mortality ratio (SMR) can be used as an indicator of quality for each hospital. SMR is defined as the ratio of O, number of deaths observed, to E, expected number of deaths per hospital estimated with the case-mix adjustment models. The estimated standard error of SMR is calculated with by Faris’ method [17]. A hospital whose SMR was significantly below 1 was considered a low mortality outlier, and when the SMR was significantly above 1 it was a high mortality outlier. We used the outlier detection method based on the test statistic [18].

To demonstrate the influence of case-mix quality on the classification of hospitals with low quality data (LQD group), we estimated SMR in two ways. For SMR1, the expected number (E1) of deaths per hospital was estimated with the risk-adjustment models fitted with low-quality data (LQD group). For SMR2, the expected number (E2) of deaths per hospital was estimated with the linear predictor for the low-quality data using the coefficients of a logistic regression model developed from the GQD group, as described by EW Steyerberg [16] (S1 Appendix). The effect of the different models on the overall between-hospital variation was quantified by calculating the interquartile ranges of SMR1 and SMR2. We used the kappa (k) statistic to assess the level of agreement between methods for outliers. The statistic measures the proportion of observed-to-expected agreement, and we adopted the principle that k>0.8 indicates excellent agreement, k = 0.6–0.8 indicates satisfactory agreement, k = 0.41–0.6 indicates moderate agreement, and k = 0.21–0.4 indicates low agreement [19].

Calculations were done with STATA 14 statistical software (StataCorp, College Station, Tex) and R statistical software for which we used Harrell’s Design library.

## Results

### Comparison of databases

**Fig 1:**
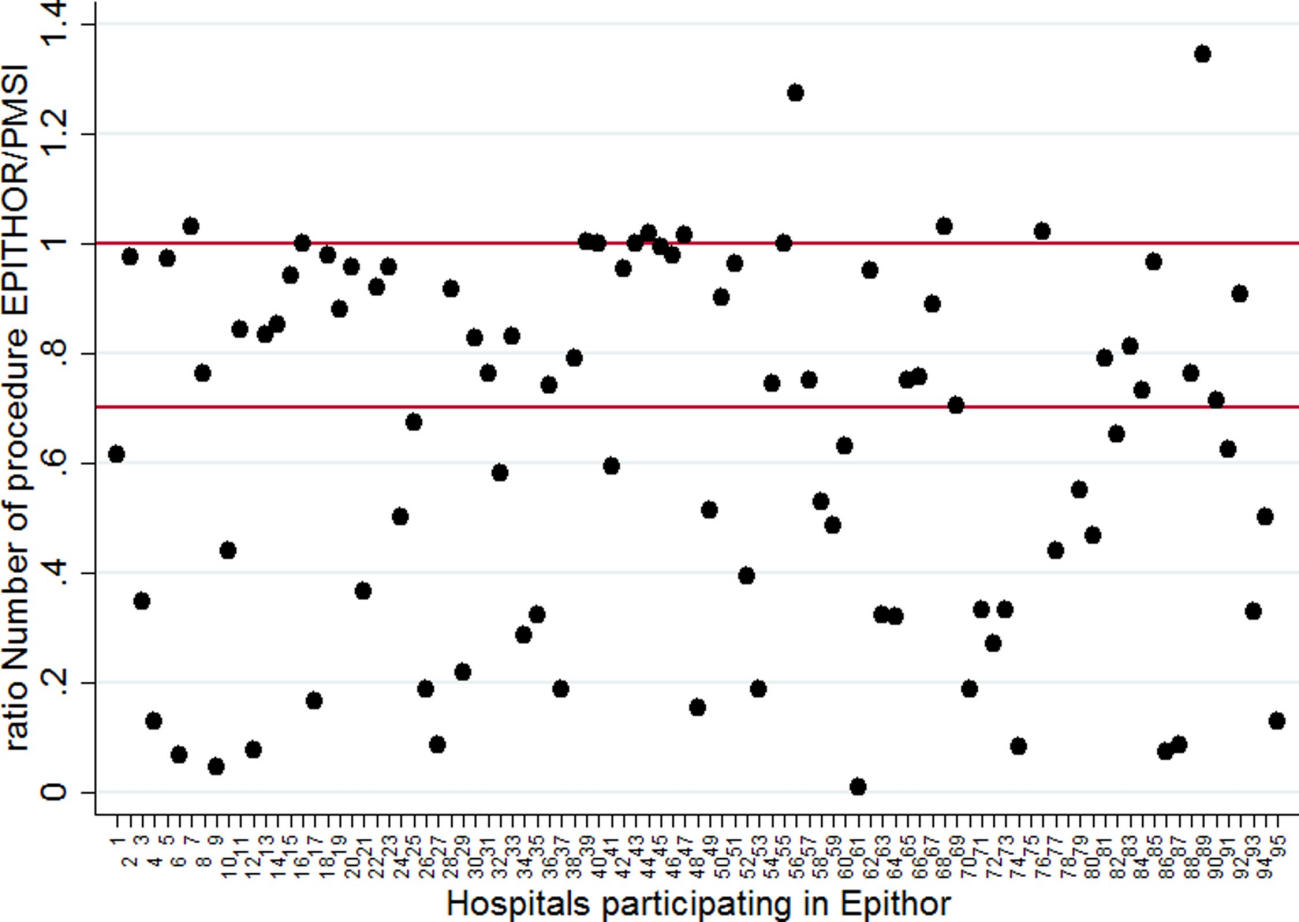
Ratio of the number of procedures in the Epithor National clinical database in comparison with the Medico-administrative database (PMSI).

**Fig 2:**
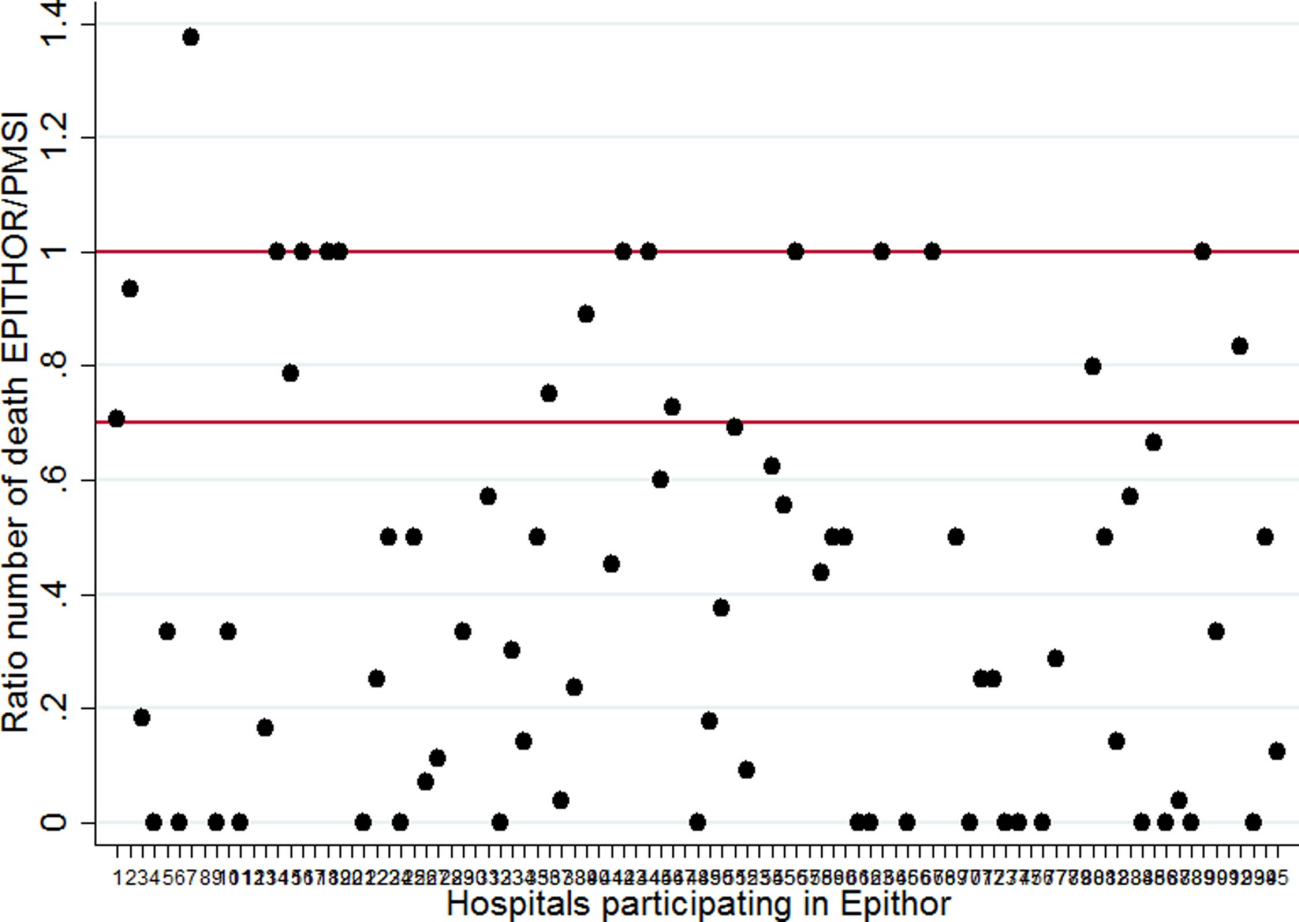
Ratio of the number of in-hospital deaths in the Epithor National clinical database in comparison with the Medico-administrative database (PMSI).

Twenty-five hospitals participating in Epithor were included in the good-quality data (GQD) group and 75 were classified in the low-quality data (LGD) group (Figs 1 and 2) (S1 Table). Hospital characteristics are presented in Table 1.

**Table 1:** Comparison hospital characteristics participating in Epithor with ratio of the number of pulmonary resections and the number of deaths greater than 0.7 vs hospital with ratio less than 0.7.

	LQD group	GQD group
Epithor National clinical database		
Number of hospitals	75	25
Number of patients	9616	10 597
Lung resection (n)	56^a^	167^a^
	17–138^b^	66–316^b^
30-day mortality (n)	1^a^	3^a^
	0–3^b^	2–8^b^
National medico-administrative database (PMSI)		
Lung resection (n)	121^a^	154^a^
	48–270^b^	51–323^b^
30-day mortality (n)	3^a^	3^a^
	1–9^b^	1–8^b^
Hospital type		
Non-teaching	14 (19%)	7 (28%)
Private	38 (51%)	8(32%)
Teaching	23(31%)	10(40%)

LQD group: Low-quality data group; GQD group: Good-quality data group;

a: median

b:IQR: interquartile range

### Patient characteristics

The 30-day mortality rate was 2.8% (n = 300) in the GQD group vs. 1.9% (n = 181) in the LQD group (p <0.0001). The comorbidities are compared in Table 2. In the GQD group, significantly more patients had a performance status of 1 and the dyspnea score was higher on average (Table 2). The TNM stage was missing significantly more often in the LQD group (Table 2).

**Table 2:** Comparison of patient characteristics in hospitals with good-quality data and low-quality data (Epithor database).

		LQD group (n = 9 616)	GQD group (n = 10 597)	P value
Sex	Male	6272 (65%)	6994 (66%)	0.25
	Female	3344 (35%)	3603 (34%)	
Age	Years^a^	64.5±9.7	64±10	0.002
American Society of Anesthesiologists score	1	1510(16%)	1640(15.5%)	0.06
	2	4849 (50%)	5512(52%)	
	≥3	3257 (34%)	3445(32.5%)	
Performance status	0	4995(52%)	4712(44%)	0.0001
	1	3689(38%)	4840(46%)	
	≥2	932(10%)	1045(10%)	
Dyspnea score	0	6386(66.5%)	4500(42.5%)	0.0001
	1	2424(25%)	3774(36%)	
	2	656(7%)	1944(18%)	
	≥3	150(1.5%)	379(3.5%)	
Forced expiratory volume	%	84.6±20.5	83±21	0.0001
Forced expiratory volume	Missing	792(8%)	1070(10%)	0.0001
Body mass index	Kg/m2	25.4±4.5	25.45±4.5	0.23
Procedure	Limited resection	1384(14%)	1482(14%)	0.3
	Lobectomy	7045(73%)	7822(74%)	
	Bilobectomy	397(4%)	393(3.5%)	
	Pneumonectomy	790(8%)	900(8.5%)	
Surgical approach	Video-assisted thoracoscopy	3536 (37%)	2952(28%)	0.0001
	Robotic Thoracotomy	6080(63%)	7645(72%)	
Coronary disease	yes	922(9.6%)	966(9%)	0.25
Tobacco user	Yes	2209(23%)	2708(25.5%)	0.0001
Chronic bronchitis	Yes	2183(23%)	2317(22%)	0.15
Arrhythmia	Yes	650(6.7%)	602(5.7%)	0.001
Chronic heart failure	Yes	168(1.75%)	255(2.4%)	0.001
Peripheral artery disease	Yes	161(1.7%)	206(1.9%)	0.15
Alcoholism	Yes	475(5%)	541(5%)	0.6
Cirrhosis	Yes	62(0.6%)	74(0.7%)	0.6
Stroke	Yes	325(3.4%)	327(3%)	0.23
Diabetes	Yes	1002(10.4%)	1095(10.3%)	0.8
Coagulation disorder	Yes	683(7%)	752(7%)	0.9
Hematologic disease	yes	147(1.53%)	198(1.9%)	0.06
History of neoplasm	Yes	2972(31%)	3087(29%)	0.006
Surgical history	Yes	735(7.6%)	891(8.4%)	0.04
Severe malnutrition	yes	67(0.7%)	73(0.7%)	0.9
Pulmonary embolism	Yes	114(1.2%)	136(1.3%)	0.5
Infectious disease	Yes	238(2.5%)	269(2.54%)	0.8
Endocrinology disease	Yes	389(4%)	373(3.5%)	0.05
Anemia	Yes	21(0.2%)	24(0.2%)	0.9
Steroids	Yes	41(0.4%)	45(0.4%)	0.9
Immunosuppression	Yes	38(0.4%)	64(0.6%)	0.04
Cardiac malformation	Yes	1(0.01%)	6(0.06%)	0.08
Chronic renal disease	Yes	161(1.7%)	193(1.82%)	0.4
Valvulopathy	Yes	86(0.9%)	81(0.8%)	0.3
Neurological disorder	Yes	160(1 .7%)	125(1.2%)	0.004
Psychiatric disorder	Yes	362(3.8%)	349(3.3%)	0.06
Asthma	Yes	151(1.6%)	132(1.25%)	0.05
Respiratory failure	Yes	617(6.4%)	641(6%)	0.28
pTumor	Ia or Ib or Ic	3417(35.5%)	4162(39%)	0.0001
	IIa or IIb	2484(26%)	3210(30%)	
	III	1114(11.5%)	1569(15%)	
	IV	453(5%)	576(5%)	
	Missing	2148(22%)	1080(10%)	
pNodes	0	5290(55%)	6651(63%)	0.0001
	1	909(9%)	1297(12%)	
	2	1084(11%)	1384(13%)	
	Missing	2333(24%)	1265(12%)	
Quality of resection	R0	7107(74%)	9128(86%)	0.0001
	R1	225(2%)	287(3%)	
	R2	86(1%)	166(2%)	
	Missing	2198(23%)	1016(10%)	

LQD group: Low-quality data group; GQD group: Good-quality data group

a: mean±standard deviation

### Risk-adjustment models

ASA scores were used as the linear variable. Performance status and dyspnea scores were categorized into 2 classes, and 3 classes were defined for BMI. Comorbidities were then selected in logistic regression using forward variable stepwise selection. The model was first developed from the GQD group and then applied to the LQD group (Tables 3 and S2). Variables such as performance status and dyspnea score had very different coefficients in the logistic model applied to the LQD group. The same can be said for comorbidities. The Hosmer-Lemeshow goodness-of-fit test was non-significant (Table 3). The C-statistic compared the discriminatory ability, which was found to be good for both models (HQD group 0.78 and LQD group 0.8) (Table 3).

**Table 3:** Logistic regression coefficients for the good-quality data group and low quality group for 30-day mortality (Epithor database).

		GQD group ^a^	P-value	LQD group^a^	P-value
Age	Age (minus mean) (year)	0.044(0.007)	0.0001	0.054 (0.009)	0.0001
Sex	Male	0	0.0001	0	0.0001
	Female	-0.944 (0.17)		-0.755(0.21)	
American Society of Anesthesiologist score	Linear	0.31(0.1)	0.003	0.67(0.14)	0.0001
Performance status classification	0 or 1	0	0.0001	0	0.9
	≥2	0.6(0.15)		-0.004 (0.22)	
Dyspnea score	0 or 1 or 2	0	0.005	0	0.0001
	≥3	0.62(0.22)		1.62(0.3)	
Procedure class	Other	0	0 0.0001	0	0.004
	Pneumonectomy	0.77(0.17)		0.63(0.22)	
Surgical approach	Thoracotomy	0	0.001	0	0.001
	VATS	-0.59(0.17)		-0.68(0.21)	
Body mass index	<24 kg/m2	0	0.0001	0	0.28
	24–28 kg/m2	-0.55(0.15)		-0.21(0.187)	
	>28 kg/m2	-0.67(0.15)		-0.27(0.19)	
Chronic heart failure	Yes	0.97(0.27)	0.0001	-0.07(0.62)	0.9
Alcoholism	Yes	0.58(0.24)	0.015	0.23(0.39)	0.5
Cirrhosis	Yes	0.94(0.57)	0.09	1.35(0.64)	0.035
History of neoplasm	Yes	0.33(0.14)	0.02	-0.03(0.21)	0.8
Respiratory failure	Yes	0.79(0.23)	0.001	0.71(0.28)	0.013
pTumor	Ia or Ib or Ic	0	0.0016	0	0.04
	IIa or IIb	0.04(0.17)		-0.17(0.22)	
	III	0.5(0.18)		0.33(0.24)	
	IV	0.75(0.23)		0.63(0.28)	
	Missing	0.79(0.41)		0.54(0.57)	
Quality of resection	R0	0	0.1	0	0.24
	R1	-0.11(0.31)		0.62(0.32)	
	R2	0.22(0.38)		0.26(0.63)	
	Missing	-0.74(0.32)		-0.19(0.355)	
Intercept		-4.4		-5.57	
Model performance					
R2		0.12		0.13	
C-statistic		0.78		0.8	
Hosmer Lemeshow test		4.47		6.4	
P-value		0.81		0.6	

VATS: Video-assisted thoracic surgery; LQD group: Low-quality data group; GQD group: Good-quality data group

a: coefficents of logistic regression (standard error)

### Effects of case-mix

The between-hospital differences were substantial in SMR1 (interquartile range (IQR) 0–1.036), and they were even higher in SMR2 (IQR 0–1.19). SMR1 and SMR2 for hospitals classified as LQD are compared in Table 4. Seven hospitals were identified as high-mortality outliers with SMR1. The methods used for SMR2 identified 4 hospitals as high-mortality outliers (Table 4). Some hospitals changed from non-outlier to high-mortality and vice-versa (Table 4). Kappa values were roughly 0.46 and indicated moderate agreement.

**Table 4:** Classification of hospitals with low-quality data according to standardized mortality rate (Epithor database).

	Low-outlier2^a^	Non-outlier2	High-outlier2	Total
Low-outlier1 ^b^	0	0	0	0
Non-outlier1	1	66	1	68
High-outlier1	0	4	3	7
Total	1	70	4	75

a: Hospital outlier detection based on SMR2 testing method

b: hospital outlier detection based on SMR1testing method

## Discussion

This study demonstrates the limitations of a clinical database with voluntary participation. We found that comorbidities were generally under-coded in the low-quality data group of hospitals and that the underestimation of observed mortality influenced the construction of the risk-adjustment model. These elements can lead to hospital misclassification [20–23]. However, the number of patients, specifically the number of patients who have a given event per hospital, have a much greater impact on accuracy [22]. The between-hospital difference in SMR was substantial (interquartile range) for both estimates. Our work shows the ability of the risk-adjustment model for identifying high-mortality outliers and low-mortality outliers with marginal agreement between the two methods.

Some work on the comparison of medico-administrative databases and clinical databases can be found in the literature; these studies emphasize that both types of database have their limits [21–24]. The observed mortality rates vary between databases for the same types of surgery (25). Co-morbidities can also vary significantly in two databases that include the same type of surgery [25]. These differences, which can be the result of non-standardized end-points or the misclassification of cases [25], have a direct influence on the construction of risk-adjustment models for estimating the standardized mortality ratio. One published study compared the European and the North American general thoracic surgery databases [26]. The authors revealed considerable disparities in the rates of certain comorbidities, including coronary artery disease. This poses a problem in the interpretation of results seeing as this discrepancy can hardly be explained by patient characteristics. Two potential reasons could be put forward: firstly, that the European database under-codes certain comorbidities, or, secondly, that the databases do not use the same definitions. Further in the article, missing data for the TNM stage are reported. Missing data were more numerous in the European database, corroborating the theory that certain variables were under-reported [26].

The definition of an outcome such as mortality, which is the most commonly used indicator in surgery, can vary across databases. For some, mortality means hospital mortality, defined as any death occurring during the same hospital stay as the surgery, but mortality can also be 30-day mortality. The reported rate depends on the definition used, leading to discrepancies in the logistic model [20,22,27,28].

Our work is different from other publications comparing databases. Contrary to one study that used the clinical database as a reference to validate the medico-administrative database, we used the medico-administrative database to validate the completeness of the data from the centers participating in Epithor [25]. We used the PMSI, the exhaustive medico-administrative database, as the gold standard against which we compared the number of pulmonary resections and deaths recorded in Epithor. All private and public centers in France are required to use the PMSI database to finance their activity. Death is systematically reported as one of reasons for patient discharge from the hospital. In addition, it is possible to follow up on patients to see whether they died later on in another hospital.

This work raises the essential question of data quality[23,25,27,28,29]. We showed that the estimation of logistic model coefficients differs considerably with the quality of the data. The differences between logistic regression models have an effect on the determination of hospital outliers as demonstrated by previous studies using different methodologies [11,20,30]. We used two logistic models to estimate the expected number of deaths in the low-quality data group. The first model, estimated from the LQD data, was responsible for the over-fitting of coefficients. In the second model, using the method proposed by Steyerberg, we applied the coefficients from logistic regression estimated from the data provided by the GQD group [16]. We showed that this changes the estimate of the standard mortality rate (SMR) (appendix) and consequently the determination of the outliers. This difference in case-mix can influence the comparison of standardized mortality ratios as demonstrated Manktelow et al. [11]. In addition, it has been shown that the death rate and the volume of activity have a significant influence on whether the hospital is considered a high mortality or low mortality outlier [11,20,30]. The observed mortality rate in the LQD group was significantly lower than that of the GQD group; this is explained by an under-reporting of events (as shown in Fig 2 where these hospitals had a ratio significantly lower than 0.7).

The main limit of our study was the use of the FINESS code of each hospital to link the two databases [31]. This code was used to identify the hospital in the PMSI data and then manually match it with the Epithor database. This method was a reliable means to compare the number of lung resections and the number of deaths. In France it is very difficult to link two databases using patient identifiers because personal data is strictly protected by the CNIL [32]. Our assessment of the Epithor database is limited, but it remains enlightening regarding the need to apply enhanced measures for quality control. Recently, dashboards have been put into place so that each center can see its fill rate for different items compared to a national reference. Future on-site audits are planned, similar to what is currently in place for the STS database [33].

This study was essential for improving the quality of the Epithor database and it has been used to select the hospitals and surgeons providing the most complete data. These hospitals will serve as references for teams with lower quality data. In parallel, on-site audits are being implemented with the aim of continuously improving the quality of the Epithor database.

## Conclusion

Epithor is an essential clinical database for measuring quality of care in France. There is a real interest in providing quality data, though some hospitals need to work on upgrading their participation. The systematic recording of data is an essential step in quality measurement, and it is necessary for the unbiased adjustment of regression models.

## Supporting information

S1 Appendix (PDF)

S1 Table Data that was used to make Figs 1 and 2. (PDF)

S2 Table Data used to perform the risk-adjustments models. (PDF)
