# Supplementary material for: Comparison of Epithor clinical national database and medico-administrative database to identify the influence of case-mix on the estimation of hospital outliers
Source: PLoS One. 2019 Jul 24;14(7):e0219672. doi: 10.1371/journal.pone.0219672 (PMC6655697; doi:10.1371/journal.pone.0219672)
Supplement: S1 Appendix — (PDF) [file pone.0219672.s001.pdf]

Appendix.

We report some values for Standardized Mortality Ratio 1 (SMR1) and Standardized Mortality Ratio 2 (SMR2) from hospitals with low-quality data (LQD).

For SMR1, the expected number (E1) of deaths per hospital was estimated with the risk-adjustment models (including age, sex, comorbidities, American Society of Anesthesiologists score, Performance status, dyspnea score) fitted with low-quality data (LQD group).

For SMR2, the expected number (E2) of deaths per hospital was estimated with a linear predictor using the coefficients of a logistic regression model (age, sex, comorbidities, American Society of Anesthesiologists score, Performance status, dyspnea score) developed from the good-quality data group. The coefficients for the logistic model were applied to the LQD group data to estimate expected number (E2) of deaths per hospital, as described by Steyerberg (16).

For SMR1 and SMR2, the number of deaths observed (O) for each hospital was estimated in the LQD group.

hospitals	SMR1	SMR2
1	2.67	5.12
2	1.710532	3.255
…….		
9	1.471635	0.93
……		
13	0.98	0.895
14	0.897	0.955
……		
67	1.23	2.177
68	0.515	0.55
69	2.96	3.2
70	0.51	0.52
…….		
73	3.16	2.8
74	1.537	1.3
75	0.78	0.706
