# Supplementary material for: Comparison of Epithor clinical national database and medico-administrative database to identify the influence of case-mix on the estimation of hospital outliers
Source: PLoS One. 2019 Jul 24;14(7):e0219672. doi: 10.1371/journal.pone.0219672 (PMC6655697; doi:10.1371/journal.pone.0219672)
Supplement: S1 Table — (PDF) [file pone.0219672.s002.pdf]

Volume_p	volume_e	number_deat	number_death_epit	ratio_volume	ratio_death	hospvalid
84	37	3	1	0.44047617912292	0.333333343267441	0
26	2	0	0	0.0769230797886	0	0
307	189	17	12	0.6156351566314	0.705882370471954	0
11	11	0	0	1	0	0
48	49	1	0	1.0208333730697	0	0
122	93	0	1	0.7622950673103	0	0
8	6	2	0	0.75	0	0
46	10	3	1	0.2173912972211	0.333333343267441	0
22	7	0	0	0.3181818127632	0	0
347	153	9	3	0.4409222006797	0.333333343267441	0
81	57	2	1	0.7037037014961	0.5	0
272	144	16	7	0.5294117927551	0.4375	0
185	177	4	2	0.9567567706108	0.5	0
75	35	5	4	0.4666666686534	0.800000011920928	0
303	252	10	3	0.8316831588745	0.300000011920929	0
16	10	0	0	0.625	0	0
62	59	2	0	0.9516128897666	0	0
264	22	9	0	0.0833333358168	0	0
41	15	1	0	0.3658536672592	0	0
282	36	7	0	0.1276595741510	0	0
19	12	2	1	0.6315789222717	0.5	0
105	1	1	0	0.0095238098874	0	0
653	516	17	4	0.7901991009712	0.235294118523598	0
305	120	11	1	0.3934426307678	0.0909090936183929	0
156	78	2	0	0.5	0	0
133	17	8	1	0.1278195530176	0.125	0
20	15	0	0	0.75	0	0
239	178	8	5	0.7447698712348	0.625	0
195	17	9	1	0.0871794894337	0.111111111938953	0
95	7	11	0	0.0736842080950	0	0
148	40	4	1	0.2702702581882	0.25	0
522	97	27	1	0.1858237534761	0.0370370373129845	0
47	43	0	0	0.9148936271667	0	0
45	43	0	0	0.9555555582046	0	0
66	68	0	1	1.0303030014038	0	0
270	193	9	3	0.7148148417472	0.333333343267441	0
295	285	6	4	0.9661017060279	0.666666686534881	1
44	2	3	0	0.0454545468091	0	0
107	72	2	1	0.6728972196578	0.5	0
365	177	6	3	0.4849314987659	0.5	0
699	456	14	2	0.6523604989051	0.142857149243355	0
48	24	2	1	0.5	0.5	0
106	20	1	0	0.1886792480945	0	0
12	2	0	0	0.1666666716337	0	0
148	120	7	4	0.8108108043670	0.571428596973419	0
237	138	4	0	0.5822784900665	0	0
114	90	2	1	0.7894737124443	0.5	0
133	38	7	1	0.2857142984867	0.142857149243355	0
255	82	6	3	0.3215686380863	0.5	0
3000	1038	82	15	0.3459999859333	0.182926833629608	0
15	1	1	0	0.0666666701436	0	0
111	102	4	1	0.9189189076423	0.25	0
120	66	0	0	0.5500000119209	0	0
474	177	13	2	0.3734177350997	0.15384615957737	0
24	8	4	1	0.3333333432674	0.25	0

2	2	0	0	1	0	0
244	186	7	4	0.76229506731033	0.571428596973419	0
435	37	26	1	0.08505747467279	0.0384615398943424	0
107	20	0	0	0.18691588938236	0	0
127	41	3	3	0.32283464074134	1	0
38	32	2	0	0.84210526943206	0	0
36	12	1	0	0.33333334326744	0	0
237	104	7	2	0.43881857395172	0.28571429848671	0
490	251	17	3	0.51224488019943	0.176470592617989	0
757	753	25	15	0.99471598863601	0.600000023841857	0
70	68	3	1	0.97142857313156	0.333333343267441	0
599	539	16	6	0.89983308315277	0.375	0
392	377	13	9	0.96173471212387	0.692307710647583	0
199	166	6	1	0.83417087793350	0.16666667163372	0
149	109	3	0	0.73154360055923	0	0
102	102	9	5	1	0.555555582046508	0
94	31	3	0	0.32978722453117	0	0
297	56	14	1	0.18855218589305	0.0714285746216774	0
160	122	3	0	0.76249998807907	0	0
572	339	31	14	0.59265732765197	0.451612889766693	0
59	9	2	0	0.15254236757755	0	0
98	177	1	2	1.80612242221832	2	1
118	105	3	3	0.88983052968978	1	1
399	376	14	11	0.94235587120056	0.785714268684387	1
323	316	11	8	0.97832816839218	0.727272748947143	1
27	23	1	1	0.85185188055038	1	1
44	43	2	2	0.97727274894714	1	1
323	316	11	8	0.97832816839218	0.727272748947143	1
51	65	1	1	1.2745097875595	1	1
41	31	1	3	0.75609755516052	3	1
279	283	5	8	1.0143369436264	1.60000002384185	1
162	167	8	11	1.03086423873901	1.375	1
556	460	4	7	0.82733815908432	1.75	1
795	775	30	28	0.97484278678894	0.933333337306976	1
75	66	1	1	0.87999999523162	1	1
28	296	2	5	10.5714282989501	2.5	1
110	105	2	2	0.95454543828964	1	1
118	105	3	3	0.88983052968978	1	1
154	114	4	3	0.74025976657867	0.75	1
16	16	1	1	1	1	1
174	158	6	5	0.90804594755172	0.833333313465118	1
350	356	11	11	1.01714289188385	1	1
189	254	3	3	1.3439153432846	1	1
458	459	9	8	1.00218343734741	0.888888895511627	1
556	460	4	7	0.82733815908432	1.75	1
