# Supplementary material for: Comparison of Epithor clinical national database and medico-administrative database to identify the influence of case-mix on the estimation of hospital outliers
Source: PLoS One. 2019 Jul 24;14(7):e0219672. doi: 10.1371/journal.pone.0219672 (PMC6655697; doi:10.1371/journal.pone.0219672)
Supplement: S2 Table — (PDF) [file pone.0219672.s003.pdf]

hospvalid	procedure	approach	number of	type	30-day mo	age	coronary d	tabacco us
0	0	VATS or ro	44	PRIVE	No	70	0	1
0	0	Thoracotoı	44	PRIVE	No	50	0	1
0	0	VATS or ro	44	PRIVE	No	70	0	1
0	0	VATS or ro	44	PRIVE	No	68	0	1
0	0	VATS or ro	44	PRIVE	No	77	0	1
0	0	VATS or ro	44	PRIVE	No	77	0	1
0	0	VATS or ro	44	PRIVE	No	56	0	0
0	1	VATS or ro	44	PRIVE	No	69	0	1
0	0	VATS or ro	44	PRIVE	No	73	0	1
0	1	VATS or ro	44	PRIVE	No	37	0	0
0	0	VATS or ro	44	PRIVE	No	65	0	1
0	0	VATS or ro	44	PRIVE	No	69	0	1
0	0	VATS or ro	44	PRIVE	No	47	0	1
0	0	VATS or ro	44	PRIVE	No	52	0	1
0	0	VATS or ro	44	PRIVE	No	70	0	1
0	0	VATS or ro	44	PRIVE	No	64	0	0
0	0	VATS or ro	44	PRIVE	No	60	0	0
0	0	VATS or ro	44	PRIVE	No	62	0	1
0	0	VATS or ro	44	PRIVE	No	60	0	1
0	0	VATS or ro	44	PRIVE	No	58	0	0
1	1	Thoracotoı	38	CHG	No	56	0	0
1	0	Thoracotoı	38	CHG	No	80	0	0
1	0	VATS or ro	38	CHG	No	72	0	0
1	0	Thoracotoı	38	CHG	No	68	0	0
1	0	Thoracotoı	38	CHG	No	70	0	0
1	1	Thoracotoı	38	CHG	No	69	0	0
1	0	Thoracotoı	38	CHG	No	54	0	0
1	0	Thoracotoı	38	CHG	No	56	0	0
1	0	Thoracotoı	38	CHG	No	79	0	0
1	0	Thoracotoı	38	CHG	No	66	0	0
1	0	Thoracotoı	38	CHG	No	85	0	0
1	1	Thoracotoı	38	CHG	No	57	0	0
1	0	Thoracotoı	38	CHG	No	64	0	0
1	0	Thoracotoı	38	CHG	No	59	0	0
1	0	VATS or ro	38	CHG	No	71	0	0
1	1	Thoracotoı	38	CHG	No	70	0	0
1	0	VATS or ro	38	CHG	No	62	0	0
1	0	Thoracotoı	38	CHG	No	77	0	0
1	0	Thoracotoı	38	CHG	No	56	0	0
1	0	Thoracotoı	38	CHG	No	77	0	0
1	1	Thoracotoı	38	CHG	No	67	0	1
1	1	Thoracotoı	38	CHG	Yes	52	0	1
1	0	VATS or ro	38	CHG	No	69	0	0
1	1	Thoracotoı	38	CHG	No	73	0	0
1	0	Thoracotoı	38	CHG	No	67	0	0
1	0	Thoracotoı	38	CHG	No	81	0	0
1	0	VATS or ro	38	CHG	No	71	0	1
1	0	Thoracotoı	38	CHG	No	68	0	0
1	0	VATS or ro	38	CHG	No	42	0	0
1	0	VATS or ro	38	CHG	No	76	0	0
1	0	VATS or ro	38	CHG	No	44	0	0
1	0	VATS or ro	38	CHG	No	70	0	0
1	0	Thoracotoı	38	CHG	No	66	0	0
1	0	VATS or ro	38	CHG	No	63	0	0
1	0	Thoracotoı	38	CHG	Yes	80	1	0

1	0	Thoracotoı	38	CHG	No	55	0	1
1	0	Thoracotoı	38	CHG	No	41	0	0
1	1	Thoracotoı	38	CHG	Yes	81	0	0
1	1	Thoracotoı	38	CHG	No	59	0	0
1	0	Thoracotoı	38	CHG	No	79	0	0
1	0	Thoracotoı	38	CHG	No	68	0	0
1	0	VATS or rc	38	CHG	No	62	0	0
1	0	Thoracotoı	38	CHG	No	50	0	0
1	0	Thoracotoı	38	CHG	No	79	0	0
1	0	Thoracotoı	38	CHG	No	52	0	0
1	0	VATS or rc	38	CHG	No	58	0	0
1	0	Thoracotoı	38	CHG	No	82	0	0
1	0	Thoracotoı	38	CHG	No	63	0	0
1	0	VATS or rc	38	CHG	No	63	0	0
1	0	VATS or rc	38	CHG	No	75	1	0
1	0	VATS or rc	38	CHG	No	72	0	0
1	0	VATS or rc	38	CHG	No	65	0	0
1	0	Thoracotoı	38	CHG	No	66	0	0
1	0	Thoracotoı	38	CHG	No	64	0	0
1	0	Thoracotoı	38	CHG	No	56	0	0
1	1	Thoracotoı	38	CHG	No	63	0	0
1	0	VATS or rc	38	CHG	No	78	1	0
1	0	Thoracotoı	38	CHG	No	43	0	0
1	0	Thoracotoı	38	CHG	No	63	0	0
1	0	Thoracotoı	38	CHG	No	71	0	0
1	0	Thoracotoı	38	CHG	No	47	0	0
1	0	Thoracotoı	38	CHG	No	61	0	1
1	0	Thoracotoı	38	CHG	No	66	0	0
1	0	VATS or rc	38	CHG	No	77	0	0
1	1	Thoracotoı	38	CHG	No	52	0	0
1	1	Thoracotoı	38	CHG	No	41	0	1
1	0	Thoracotoı	38	CHG	No	59	0	0
1	0	Thoracotoı	38	CHG	No	66	0	0
1	0	Thoracotoı	38	CHG	No	70	0	1
1	0	VATS or rc	38	CHG	No	66	0	1
0	0	VATS or rc	5	PRIVE	No	72	0	1
0	0	VATS or rc	5	PRIVE	No	67	0	0
0	0	VATS or rc	5	PRIVE	No	47	0	1
0	0	VATS or rc	5	PRIVE	No	54	0	1
0	0	Thoracotoı	5	PRIVE	No	56	0	1
0	0	VATS or rc	5	PRIVE	No	48	0	0
0	0	Thoracotoı	5	PRIVE	No	52	0	1
0	0	VATS or rc	5	PRIVE	No	61	0	1
0	0	VATS or rc	5	PRIVE	No	35	0	0
0	0	VATS or rc	5	PRIVE	No	65	0	1
0	0	VATS or rc	5	PRIVE	No	71	0	0
0	0	VATS or rc	5	PRIVE	No	69	0	0
0	0	VATS or rc	5	PRIVE	No	72	0	1
0	0	VATS or rc	5	PRIVE	No	60	0	1
0	0	VATS or rc	5	PRIVE	No	71	0	1
0	0	VATS or rc	5	PRIVE	No	72	0	0
0	0	VATS or rc	5	PRIVE	No	71	0	1
0	0	Thoracotoı	5	PRIVE	No	49	0	1
0	0	VATS or rc	5	PRIVE	No	78	0	1
0	0	Thoracotoı	5	PRIVE	No	48	0	0
0	0	Thoracotoı	5	PRIVE	No	69	0	1

0	0	VATS or rc	5	PRIVE	No	65	0	1
0	0	VATS or rc	5	PRIVE	No	58	0	1
0	0	VATS or rc	5	PRIVE	No	82	0	0
0	0	VATS or rc	5	PRIVE	No	74	0	1
0	0	VATS or rc	5	PRIVE	No	80	0	0
0	0	Thoracotoı	5	PRIVE	No	56	0	0
0	0	Thoracotoı	5	PRIVE	No	51	0	1
0	0	VATS or rc	5	PRIVE	Yes	78	1	0
0	0	VATS or rc	5	PRIVE	No	52	0	1
0	0	Thoracotoı	5	PRIVE	No	76	0	1
0	0	VATS or rc	166	CHU	No	56	0	0
0	0	VATS or rc	166	CHU	No	57	0	0
0	0	VATS or rc	166	CHU	No	65	0	0
0	0	Thoracotoı	166	CHU	No	74	0	0
0	0	VATS or rc	166	CHU	No	69	0	0
0	0	Thoracotoı	166	CHU	No	74	0	0
0	0	VATS or rc	166	CHU	No	61	0	0
0	0	Thoracotoı	166	CHU	No	48	0	0
0	0	VATS or rc	166	CHU	No	68	0	0
0	0	VATS or rc	166	CHU	No	59	0	0
0	0	Thoracotoı	166	CHU	No	53	0	0
0	0	VATS or rc	166	CHU	No	59	0	0
0	0	VATS or rc	166	CHU	No	58	0	0
0	0	Thoracotoı	166	CHU	No	53	0	0
0	0	VATS or rc	166	CHU	No	66	0	0
0	0	VATS or rc	166	CHU	No	71	0	0
0	0	VATS or rc	166	CHU	No	63	0	0
0	0	VATS or rc	166	CHU	No	58	0	0
0	0	VATS or rc	166	CHU	No	54	0	0
0	0	VATS or rc	166	CHU	No	70	0	0
0	0	VATS or rc	166	CHU	No	66	0	0
0	0	VATS or rc	166	CHU	No	62	0	0
0	0	VATS or rc	166	CHU	No	79	0	0
0	0	VATS or rc	166	CHU	No	70	0	0
0	0	VATS or rc	166	CHU	No	68	1	0
0	0	VATS or rc	166	CHU	No	64	0	0
0	0	VATS or rc	166	CHU	No	83	0	0
0	0	VATS or rc	166	CHU	No	63	0	0
0	0	VATS or rc	166	CHU	No	71	0	0
0	0	VATS or rc	166	CHU	No	72	0	0
0	0	VATS or rc	166	CHU	No	66	0	0
0	0	VATS or rc	166	CHU	No	60	0	1
0	0	VATS or rc	166	CHU	No	61	0	0
0	0	VATS or rc	166	CHU	No	81	0	0
0	0	VATS or rc	166	CHU	No	61	0	0
0	0	Thoracotoı	166	CHU	No	59	0	0
0	0	VATS or rc	166	CHU	No	62	0	0
0	0	VATS or rc	166	CHU	No	72	1	0
0	0	VATS or rc	166	CHU	No	54	0	0
0	0	Thoracotoı	166	CHU	No	57	0	0
0	0	VATS or rc	166	CHU	No	74	0	0
0	0	VATS or rc	166	CHU	No	68	0	0
0	0	VATS or rc	166	CHU	No	75	0	0
0	0	VATS or rc	166	CHU	No	65	0	0
0	0	VATS or rc	166	CHU	No	64	0	0
0	0	VATS or rc	166	CHU	No	77	0	0

0	0	VATS or ro	166	CHU	No	58	0	0
0	0	VATS or ro	166	CHU	No	61	0	0
0	0	VATS or ro	166	CHU	No	50	0	0
0	0	Thoracotom	166	CHU	No	65	0	0
0	0	VATS or ro	166	CHU	No	52	0	1
0	0	VATS or ro	166	CHU	No	62	0	0
0	0	VATS or ro	166	CHU	No	64	0	0
0	0	VATS or ro	166	CHU	No	63	0	0
0	1	Thoracotom	166	CHU	No	68	0	0
0	1	VATS or ro	166	CHU	No	71	1	1
0	0	VATS or ro	166	CHU	No	78	0	0
0	0	VATS or ro	166	CHU	No	74	0	0
0	0	VATS or ro	166	CHU	No	82	0	0
0	0	Thoracotom	166	CHU	No	42	0	0
0	1	Thoracotom	166	CHU	No	60	0	0
0	0	VATS or ro	166	CHU	No	71	0	0
0	0	VATS or ro	166	CHU	No	66	0	0
0	0	VATS or ro	166	CHU	No	61	0	0
0	0	VATS or ro	166	CHU	No	47	0	0
0	0	VATS or ro	166	CHU	No	66	0	0
0	0	VATS or ro	166	CHU	No	69	0	0
0	0	VATS or ro	166	CHU	No	67	0	0
0	0	VATS or ro	166	CHU	No	60	0	1
0	0	VATS or ro	166	CHU	No	70	0	0
0	0	Thoracotom	166	CHU	No	66	0	0
0	0	VATS or ro	166	CHU	No	61	0	0
0	0	VATS or ro	166	CHU	No	57	0	0
0	1	Thoracotom	166	CHU	No	50	0	1
0	0	VATS or ro	166	CHU	No	79	0	0
0	0	Thoracotom	166	CHU	No	61	0	0
0	0	VATS or ro	166	CHU	No	75	0	0
0	0	VATS or ro	166	CHU	No	32	0	0
0	0	VATS or ro	166	CHU	No	56	0	0
0	0	VATS or ro	166	CHU	No	67	0	0
0	0	VATS or ro	166	CHU	No	60	0	0
0	0	VATS or ro	166	CHU	No	53	0	0
0	0	VATS or ro	166	CHU	No	71	0	0
0	0	VATS or ro	166	CHU	No	72	0	0
0	0	Thoracotom	166	CHU	No	58	0	0
0	0	VATS or ro	166	CHU	No	67	0	0
0	0	VATS or ro	166	CHU	No	79	0	0
0	0	VATS or ro	166	CHU	No	79	0	0
0	0	VATS or ro	166	CHU	No	52	0	0
0	0	VATS or ro	166	CHU	No	46	0	1
0	0	VATS or ro	166	CHU	No	57	0	0
0	0	VATS or ro	166	CHU	No	69	0	0
0	0	VATS or ro	166	CHU	No	56	0	0
0	0	VATS or ro	166	CHU	No	62	0	0
0	0	VATS or ro	166	CHU	No	59	0	0
0	0	VATS or ro	166	CHU	No	59	0	1
0	0	VATS or ro	166	CHU	No	70	0	0
0	0	VATS or ro	166	CHU	No	71	0	0
0	0	Thoracotom	166	CHU	No	50	0	0
0	0	VATS or ro	166	CHU	No	49	0	1
0	1	Thoracotom	166	CHU	No	76	1	0
0	0	VATS or ro	166	CHU	No	56	0	0

0	0	VATS or rc	166	CHU	No	66	0	0
0	0	VATS or rc	166	CHU	No	66	0	0
0	0	VATS or rc	166	CHU	No	72	0	0
0	0	VATS or rc	166	CHU	No	62	0	0
0	0	VATS or rc	166	CHU	No	76	0	0
0	0	Thoracotoı	166	CHU	No	79	0	0
0	0	VATS or rc	166	CHU	No	42	0	0
0	0	VATS or rc	166	CHU	No	64	0	0
0	0	VATS or rc	166	CHU	No	69	0	1
0	0	VATS or rc	166	CHU	No	82	0	0
0	0	VATS or rc	166	CHU	No	68	0	0
0	0	VATS or rc	166	CHU	No	78	0	0
0	0	VATS or rc	166	CHU	No	67	1	0
0	0	Thoracotoı	166	CHU	No	62	0	0
0	0	VATS or rc	166	CHU	No	62	0	0
0	0	Thoracotoı	166	CHU	No	55	0	0
0	0	VATS or rc	166	CHU	No	65	0	0
0	0	VATS or rc	166	CHU	No	62	0	0
0	0	VATS or rc	166	CHU	No	61	0	0
0	0	VATS or rc	166	CHU	No	73	0	0
0	0	Thoracotoı	166	CHU	No	54	0	0
0	0	VATS or rc	166	CHU	No	68	0	0
0	0	VATS or rc	166	CHU	No	63	0	0
0	0	Thoracotoı	166	CHU	No	61	0	0
0	0	VATS or rc	166	CHU	No	51	0	0
0	0	Thoracotoı	166	CHU	No	74	0	0
0	0	VATS or rc	166	CHU	No	69	0	0
0	0	Thoracotoı	166	CHU	No	79	0	0
0	0	VATS or rc	166	CHU	No	65	0	0
0	0	VATS or rc	166	CHU	No	68	0	0
0	0	VATS or rc	166	CHU	No	83	1	0
0	0	VATS or rc	166	CHU	No	50	0	0
0	0	VATS or rc	166	CHU	No	71	0	0
0	0	VATS or rc	166	CHU	No	61	0	0
0	0	VATS or rc	166	CHU	No	74	0	0
0	0	VATS or rc	166	CHU	No	78	0	0
0	0	VATS or rc	166	CHU	No	59	0	0
0	0	VATS or rc	166	CHU	No	66	0	0
0	0	VATS or rc	166	CHU	No	63	0	0
0	0	Thoracotoı	166	CHU	No	59	0	0
0	0	VATS or rc	166	CHU	No	70	0	0
0	0	VATS or rc	166	CHU	No	57	0	0
0	0	VATS or rc	166	CHU	No	69	0	0
0	0	Thoracotoı	166	CHU	No	49	0	0
0	0	VATS or rc	166	CHU	No	70	0	0
0	0	Thoracotoı	166	CHU	No	68	0	0
0	0	VATS or rc	166	CHU	No	79	1	0
0	0	VATS or rc	166	CHU	No	56	0	0
0	0	VATS or rc	166	CHU	No	83	0	0
0	0	VATS or rc	166	CHU	No	69	0	0
0	0	VATS or rc	166	CHU	No	53	0	0
0	0	VATS or rc	166	CHU	No	70	0	0
0	0	VATS or rc	166	CHU	No	58	0	0
0	0	VATS or rc	166	CHU	No	61	0	0
0	0	VATS or rc	166	CHU	No	51	0	0
0	0	Thoracotoı	166	CHU	No	40	0	0

0	1	Thoracotor	166	CHU	No	65	0	0
0	1	Thoracotor	166	CHU	No	65	0	0
0	0	Thoracotor	166	CHU	No	61	0	1
0	0	VATS or rc	166	CHU	No	65	0	0
0	0	Thoracotor	166	CHU	No	70	1	0
0	0	VATS or rc	166	CHU	No	70	0	0
0	0	VATS or rc	166	CHU	No	68	0	0
0	0	VATS or rc	166	CHU	No	67	0	0
0	0	VATS or rc	166	CHU	No	55	0	0
0	1	Thoracotor	166	CHU	No	73	0	0
0	0	Thoracotor	166	CHU	No	71	0	0
0	0	Thoracotor	166	CHU	No	46	0	0
0	0	VATS or rc	166	CHU	No	63	0	1
0	0	VATS or rc	166	CHU	No	64	0	0
0	0	VATS or rc	166	CHU	No	70	0	0
0	0	VATS or rc	166	CHU	No	67	0	0
0	0	VATS or rc	166	CHU	No	66	0	0
0	0	Thoracotor	166	CHU	No	68	0	0
0	0	VATS or rc	166	CHU	No	62	0	1
0	1	VATS or rc	166	CHU	No	71	1	0
0	0	VATS or rc	166	CHU	No	63	0	0
0	0	VATS or rc	166	CHU	No	66	1	0
0	0	VATS or rc	166	CHU	No	53	0	0
0	1	Thoracotor	166	CHU	No	53	0	0
0	0	VATS or rc	166	CHU	No	69	0	0
0	0	VATS or rc	166	CHU	No	58	0	0
0	0	VATS or rc	166	CHU	No	71	0	0
0	0	Thoracotor	166	CHU	No	52	0	0
0	0	VATS or rc	166	CHU	No	76	0	0
0	0	VATS or rc	166	CHU	No	69	1	0
0	0	VATS or rc	166	CHU	No	57	0	0
0	0	VATS or rc	166	CHU	No	57	0	0
0	0	VATS or rc	166	CHU	No	77	0	0
0	0	VATS or rc	166	CHU	No	56	0	0
0	0	VATS or rc	166	CHU	No	66	0	0
0	0	VATS or rc	166	CHU	No	65	0	0
0	0	VATS or rc	166	CHU	No	58	0	0
0	0	VATS or rc	166	CHU	No	56	0	0
0	0	VATS or rc	166	CHU	No	48	0	0
0	0	Thoracotor	166	CHU	No	54	0	0
0	0	VATS or rc	166	CHU	No	74	0	0
0	0	VATS or rc	166	CHU	No	58	0	0
0	0	VATS or rc	166	CHU	No	49	0	0
0	0	Thoracotor	166	CHU	No	71	0	0
0	0	VATS or rc	166	CHU	No	78	0	0
0	0	VATS or rc	166	CHU	No	52	0	0
0	0	VATS or rc	166	CHU	No	47	0	0
0	0	Thoracotor	166	CHU	No	68	0	0
0	0	Thoracotor	166	CHU	No	58	0	1
0	0	VATS or rc	166	CHU	No	64	0	1
0	1	Thoracotor	166	CHU	No	62	0	0
0	0	VATS or rc	166	CHU	No	57	0	0
0	0	VATS or rc	166	CHU	No	57	0	0
0	0	VATS or rc	166	CHU	No	70	0	0
0	0	VATS or rc	166	CHU	No	65	0	0
0	0	VATS or rc	166	CHU	No	70	0	0

0	0	VATS or rc	166	CHU	No	83	0	0
0	0	VATS or rc	166	CHU	No	52	0	0
0	0	Thoracotoı	166	CHU	No	61	0	0
0	0	Thoracotoı	25	PRIVE	No	66	0	1
0	0	Thoracotoı	25	PRIVE	No	73	0	0
0	0	Thoracotoı	78	PRIVE	No	74	0	1
0	0	Thoracotoı	78	PRIVE	No	76	0	1
0	0	Thoracotoı	78	PRIVE	No	67	0	0
0	0	Thoracotoı	78	PRIVE	No	56	0	0
0	0	Thoracotoı	78	PRIVE	Yes	69	0	1
0	0	Thoracotoı	78	PRIVE	No	64	0	1
0	0	Thoracotoı	78	PRIVE	No	78	0	1
0	0	Thoracotoı	78	PRIVE	No	72	0	0
0	0	Thoracotoı	78	PRIVE	No	68	0	1
0	0	Thoracotoı	78	PRIVE	No	68	0	0
0	0	Thoracotoı	78	PRIVE	No	63	0	1
0	0	Thoracotoı	78	PRIVE	No	75	0	0
0	0	Thoracotoı	78	PRIVE	No	63	1	0
0	0	Thoracotoı	78	PRIVE	No	71	0	0
0	0	Thoracotoı	78	PRIVE	No	67	1	1
0	0	Thoracotoı	78	PRIVE	No	68	0	1
0	0	Thoracotoı	78	PRIVE	No	66	0	1
0	0	Thoracotoı	78	PRIVE	No	72	0	0
0	0	Thoracotoı	78	PRIVE	No	52	1	1
0	0	Thoracotoı	78	PRIVE	No	68	0	1
0	0	Thoracotoı	78	PRIVE	No	69	0	0
0	0	Thoracotoı	78	PRIVE	No	83	0	0
0	0	Thoracotoı	78	PRIVE	No	56	1	1
0	0	Thoracotoı	78	PRIVE	No	87	0	1
0	0	Thoracotoı	78	PRIVE	No	68	0	0
0	0	Thoracotoı	78	PRIVE	No	80	0	1
0	0	Thoracotoı	78	PRIVE	No	60	0	1
0	0	Thoracotoı	78	PRIVE	No	65	0	1
0	0	Thoracotoı	78	PRIVE	No	60	0	0
0	0	Thoracotoı	78	PRIVE	No	84	0	1
0	0	Thoracotoı	78	PRIVE	No	76	0	0
0	0	Thoracotoı	78	PRIVE	No	68	0	1
0	0	Thoracotoı	78	PRIVE	No	63	0	1
0	0	Thoracotoı	78	PRIVE	No	56	0	1
0	0	Thoracotoı	78	PRIVE	No	43	0	1
0	0	Thoracotoı	78	PRIVE	No	59	0	1
0	0	Thoracotoı	78	PRIVE	No	60	0	0
0	0	Thoracotoı	78	PRIVE	No	77	0	1
0	0	Thoracotoı	78	PRIVE	No	51	0	0
0	0	Thoracotoı	78	PRIVE	No	69	0	1
0	0	Thoracotoı	78	PRIVE	No	56	0	0
0	0	Thoracotoı	78	PRIVE	No	76	0	1
0	0	Thoracotoı	78	PRIVE	No	67	0	1
0	0	Thoracotoı	78	PRIVE	No	72	1	1
0	0	Thoracotoı	78	PRIVE	No	60	0	1
0	0	Thoracotoı	78	PRIVE	No	66	0	1
0	0	Thoracotoı	78	PRIVE	No	65	0	1
0	0	Thoracotoı	78	PRIVE	No	51	0	0
0	0	Thoracotoı	78	PRIVE	No	62	0	1
0	0	Thoracotoı	78	PRIVE	No	48	0	1
0	0	Thoracotoı	78	PRIVE	No	68	0	1

0	0	Thoracotoı	78	PRIVE	No	72	0	1
0	0	Thoracotoı	78	PRIVE	No	82	0	1
0	0	Thoracotoı	78	PRIVE	No	76	1	0
0	0	Thoracotoı	78	PRIVE	No	62	0	1
0	0	Thoracotoı	78	PRIVE	No	76	0	1
0	0	Thoracotoı	78	PRIVE	No	74	0	1
0	0	Thoracotoı	78	PRIVE	No	72	0	1
0	0	Thoracotoı	78	PRIVE	No	59	0	1
0	0	Thoracotoı	78	PRIVE	No	82	0	1
0	0	Thoracotoı	78	PRIVE	No	67	0	0
0	0	Thoracotoı	78	PRIVE	No	68	1	1
0	0	Thoracotoı	78	PRIVE	No	61	0	1
0	0	Thoracotoı	78	PRIVE	No	61	0	1
0	0	Thoracotoı	78	PRIVE	No	44	0	1
0	0	Thoracotoı	78	PRIVE	No	80	0	1
0	0	Thoracotoı	78	PRIVE	No	68	1	1
0	0	Thoracotoı	78	PRIVE	No	73	0	1
0	0	Thoracotoı	78	PRIVE	No	44	0	1
0	0	Thoracotoı	78	PRIVE	No	83	0	0
0	0	Thoracotoı	78	PRIVE	No	66	0	1
0	0	Thoracotoı	78	PRIVE	No	42	0	1
0	0	Thoracotoı	78	PRIVE	No	63	0	1
0	0	Thoracotoı	78	PRIVE	No	67	0	1
0	0	Thoracotoı	78	PRIVE	No	51	0	1
0	0	Thoracotoı	78	PRIVE	No	83	0	0
0	0	Thoracotoı	78	PRIVE	No	83	0	1
0	0	Thoracotoı	78	PRIVE	No	71	0	1
0	0	Thoracotoı	78	PRIVE	No	59	0	1
0	0	Thoracotoı	78	PRIVE	No	65	0	1
0	0	Thoracotoı	78	PRIVE	No	69	0	1
0	0	Thoracotoı	78	PRIVE	No	60	0	1
0	0	Thoracotoı	78	PRIVE	No	59	0	1
0	0	Thoracotoı	78	PRIVE	No	68	0	1
0	0	Thoracotoı	78	PRIVE	No	65	0	0
0	0	Thoracotoı	78	PRIVE	No	54	0	1
0	0	Thoracotoı	78	PRIVE	No	69	0	1
0	0	Thoracotoı	78	PRIVE	No	74	0	1
0	0	Thoracotoı	78	PRIVE	No	76	0	0
0	0	Thoracotoı	78	PRIVE	Yes	59	0	1
0	0	Thoracotoı	78	PRIVE	No	82	0	1
0	0	Thoracotoı	78	PRIVE	No	68	1	1
0	0	Thoracotoı	78	PRIVE	No	57	0	1
0	0	Thoracotoı	78	PRIVE	No	80	0	1
0	0	Thoracotoı	78	PRIVE	No	67	0	1
0	0	Thoracotoı	78	PRIVE	No	66	0	0
0	0	Thoracotoı	78	PRIVE	No	54	0	1
0	0	Thoracotoı	78	PRIVE	No	62	0	1
0	0	Thoracotoı	78	PRIVE	No	68	0	0
0	0	Thoracotoı	78	PRIVE	No	59	0	0
0	0	Thoracotoı	78	PRIVE	Yes	58	0	1
0	0	Thoracotoı	78	PRIVE	No	74	0	1
0	0	Thoracotoı	78	PRIVE	No	62	0	1
0	0	Thoracotoı	78	PRIVE	No	61	0	1
0	0	Thoracotoı	78	PRIVE	No	70	0	0
0	0	Thoracotoı	78	PRIVE	No	78	0	1
0	0	Thoracotoı	78	PRIVE	No	77	0	1

0	0	Thoracotoı	78	PRIVE	No	73	0	0
0	0	Thoracotoı	78	PRIVE	No	70	0	0
0	0	Thoracotoı	78	PRIVE	No	77	0	0
0	0	Thoracotoı	78	PRIVE	No	63	0	1
0	0	Thoracotoı	78	PRIVE	Yes	81	0	1
0	0	Thoracotoı	78	PRIVE	No	62	0	1
0	0	Thoracotoı	78	PRIVE	No	81	0	1
0	1	Thoracotoı	78	PRIVE	No	65	0	1
0	0	Thoracotoı	78	PRIVE	No	69	0	0
0	0	Thoracotoı	78	PRIVE	No	69	0	1
0	0	Thoracotoı	78	PRIVE	No	64	0	0
0	0	Thoracotoı	78	PRIVE	No	72	0	0
0	0	Thoracotoı	78	PRIVE	No	54	0	1
0	0	Thoracotoı	78	PRIVE	No	61	0	0
0	0	Thoracotoı	78	PRIVE	No	55	1	1
0	0	Thoracotoı	78	PRIVE	No	44	0	0
0	0	Thoracotoı	78	PRIVE	No	71	0	0
0	0	Thoracotoı	78	PRIVE	No	61	0	1
0	0	Thoracotoı	78	PRIVE	No	79	0	1
0	0	Thoracotoı	78	PRIVE	No	61	0	1
0	0	Thoracotoı	78	PRIVE	No	69	0	0
0	0	Thoracotoı	78	PRIVE	No	82	0	1
0	0	Thoracotoı	78	PRIVE	No	65	1	0
0	0	Thoracotoı	78	PRIVE	No	68	0	0
0	0	Thoracotoı	78	PRIVE	No	76	0	1
0	0	Thoracotoı	78	PRIVE	No	48	0	1
0	0	Thoracotoı	78	PRIVE	No	71	0	1
0	0	Thoracotoı	78	PRIVE	No	72	0	1
0	0	Thoracotoı	78	PRIVE	No	77	0	1
0	0	Thoracotoı	78	PRIVE	No	58	0	1
0	0	Thoracotoı	78	PRIVE	No	59	0	1
0	0	Thoracotoı	78	PRIVE	Yes	71	1	1
0	0	Thoracotoı	78	PRIVE	No	77	0	1
0	0	Thoracotoı	78	PRIVE	No	81	0	0
0	0	Thoracotoı	78	PRIVE	No	61	0	1
0	0	Thoracotoı	78	PRIVE	No	56	0	1
0	0	Thoracotoı	78	PRIVE	No	55	0	1
0	0	Thoracotoı	78	PRIVE	No	67	0	1
0	0	Thoracotoı	78	PRIVE	No	65	0	1
0	0	Thoracotoı	78	PRIVE	No	66	0	1
0	0	Thoracotoı	78	PRIVE	No	72	0	1
0	0	Thoracotoı	78	PRIVE	No	63	0	1
0	0	Thoracotoı	78	PRIVE	No	80	0	0
0	0	Thoracotoı	78	PRIVE	No	78	0	1
0	0	Thoracotoı	78	PRIVE	No	63	0	1
0	0	Thoracotoı	78	PRIVE	No	58	0	1
0	0	Thoracotoı	78	PRIVE	No	63	0	1
0	0	Thoracotoı	78	PRIVE	No	58	0	1
0	0	Thoracotoı	78	PRIVE	No	71	0	0
0	0	Thoracotoı	78	PRIVE	No	71	0	1
0	0	Thoracotoı	78	PRIVE	No	36	0	0
0	0	Thoracotoı	78	PRIVE	No	60	0	0
0	0	Thoracotoı	78	PRIVE	No	72	0	1
0	0	Thoracotoı	78	PRIVE	No	75	0	0
0	0	Thoracotoı	78	PRIVE	No	60	0	1
0	0	Thoracotoı	78	PRIVE	No	60	0	1

0	0	Thoracotoı	78	PRIVE	No	61	0	1
0	0	Thoracotoı	78	PRIVE	No	71	1	1
0	0	Thoracotoı	78	PRIVE	No	72	0	0
0	0	Thoracotoı	78	PRIVE	No	75	0	1
0	0	Thoracotoı	78	PRIVE	Yes	64	0	0
0	0	Thoracotoı	78	PRIVE	No	62	0	1
0	0	Thoracotoı	78	PRIVE	No	51	0	1
0	0	Thoracotoı	78	PRIVE	No	81	0	0
0	0	Thoracotoı	78	PRIVE	No	70	0	1
0	0	Thoracotoı	78	PRIVE	No	67	0	1
0	0	Thoracotoı	78	PRIVE	No	67	0	1
0	0	Thoracotoı	78	PRIVE	No	69	0	1
0	0	Thoracotoı	78	PRIVE	No	54	0	1
0	0	Thoracotoı	78	PRIVE	No	59	0	1
0	0	Thoracotoı	78	PRIVE	No	49	0	0
0	0	Thoracotoı	78	PRIVE	No	72	0	1
0	0	Thoracotoı	78	PRIVE	No	72	0	1
0	0	Thoracotoı	78	PRIVE	No	64	0	1
0	0	Thoracotoı	78	PRIVE	No	62	0	1
0	0	Thoracotoı	78	PRIVE	No	59	0	1
0	0	Thoracotoı	78	PRIVE	No	60	0	0
0	0	Thoracotoı	78	PRIVE	No	36	0	0
0	0	Thoracotoı	78	PRIVE	No	78	0	0
0	0	Thoracotoı	78	PRIVE	No	57	0	1
0	0	Thoracotoı	78	PRIVE	No	25	0	0
0	0	Thoracotoı	78	PRIVE	No	56	0	1
0	0	Thoracotoı	78	PRIVE	No	65	0	0
0	0	Thoracotoı	78	PRIVE	No	70	0	0
0	0	Thoracotoı	78	PRIVE	No	71	0	1
0	0	Thoracotoı	78	PRIVE	No	68	0	0
0	0	Thoracotoı	78	PRIVE	No	82	0	1
0	0	Thoracotoı	78	PRIVE	No	57	0	1
0	0	Thoracotoı	78	PRIVE	No	58	0	1
0	0	Thoracotoı	78	PRIVE	No	53	1	1
0	1	Thoracotoı	78	PRIVE	No	73	0	1
0	0	Thoracotoı	78	PRIVE	No	69	0	1
0	0	Thoracotoı	78	PRIVE	No	72	0	1
0	0	Thoracotoı	78	PRIVE	No	77	0	1
0	0	Thoracotoı	78	PRIVE	No	68	0	0
0	0	Thoracotoı	78	PRIVE	No	71	0	0
0	0	Thoracotoı	78	PRIVE	No	72	1	1
0	0	Thoracotoı	78	PRIVE	No	63	0	1
0	0	Thoracotoı	78	PRIVE	No	63	1	1
0	0	Thoracotoı	78	PRIVE	No	65	0	1
0	0	Thoracotoı	78	PRIVE	No	78	0	0
0	0	Thoracotoı	78	PRIVE	No	70	0	1
0	0	Thoracotoı	78	PRIVE	No	89	0	0
0	0	Thoracotoı	78	PRIVE	No	60	0	0
0	0	Thoracotoı	78	PRIVE	No	66	0	1
0	0	Thoracotoı	78	PRIVE	No	77	0	0
0	0	Thoracotoı	78	PRIVE	Yes	71	0	1
0	0	Thoracotoı	78	PRIVE	No	59	0	1
0	0	Thoracotoı	78	PRIVE	No	61	0	1
0	0	Thoracotoı	78	PRIVE	No	38	0	0
0	0	Thoracotoı	78	PRIVE	No	72	0	1
0	0	Thoracotoı	78	PRIVE	Yes	60	1	1

0	0	Thoracotoı	78	PRIVE	No	74	0	1
0	0	Thoracotoı	78	PRIVE	No	48	0	0
0	0	Thoracotoı	78	PRIVE	No	80	0	1
0	0	Thoracotoı	78	PRIVE	No	71	0	0
0	0	Thoracotoı	78	PRIVE	No	66	0	1
0	0	Thoracotoı	78	PRIVE	Yes	76	0	1
0	0	Thoracotoı	78	PRIVE	No	85	0	0
0	0	Thoracotoı	78	PRIVE	No	72	0	1
0	0	Thoracotoı	78	PRIVE	No	64	0	1
0	0	Thoracotoı	78	PRIVE	No	88	0	0
0	0	Thoracotoı	78	PRIVE	No	62	0	1
0	0	Thoracotoı	78	PRIVE	No	64	0	1
0	0	Thoracotoı	78	PRIVE	No	70	0	0
0	0	Thoracotoı	78	PRIVE	No	78	0	0
0	0	Thoracotoı	78	PRIVE	No	56	1	1
0	0	Thoracotoı	78	PRIVE	No	80	0	1
0	0	Thoracotoı	78	PRIVE	No	61	0	0
0	0	Thoracotoı	78	PRIVE	No	61	0	1
0	0	Thoracotoı	78	PRIVE	No	69	0	1
0	0	Thoracotoı	78	PRIVE	No	76	0	0
0	0	Thoracotoı	78	PRIVE	No	77	1	0
0	0	Thoracotoı	78	PRIVE	No	66	0	1
0	0	Thoracotoı	78	PRIVE	No	17	0	0
0	0	Thoracotoı	78	PRIVE	No	52	0	1
0	0	Thoracotoı	78	PRIVE	No	62	0	1
0	0	VATS or rc	44	CHG	No	68	0	0
0	0	VATS or rc	44	CHG	No	80	0	0
0	0	Thoracotoı	63	PRIVE	No	63	1	0
0	0	Thoracotoı	63	PRIVE	No	63	0	0
0	0	Thoracotoı	63	PRIVE	No	50	0	0
0	0	Thoracotoı	63	PRIVE	No	73	0	0
0	0	Thoracotoı	63	PRIVE	No	64	0	0
0	0	Thoracotoı	63	PRIVE	No	53	0	0
0	0	Thoracotoı	63	PRIVE	No	69	0	0
0	0	Thoracotoı	63	PRIVE	No	54	0	0
0	0	Thoracotoı	63	PRIVE	No	54	0	0
0	0	Thoracotoı	63	PRIVE	No	64	0	0
0	0	Thoracotoı	63	PRIVE	No	78	0	0
0	0	Thoracotoı	63	PRIVE	No	60	0	0
0	0	Thoracotoı	63	PRIVE	No	53	0	0
0	0	Thoracotoı	63	PRIVE	No	69	0	0
0	0	Thoracotoı	63	PRIVE	No	72	0	0
0	0	Thoracotoı	63	PRIVE	No	61	0	0
1	0	Thoracotoı	93	CHG	No	63	0	0
1	0	Thoracotoı	93	CHG	No	70	0	0
1	0	VATS or rc	93	CHG	No	68	0	1
1	0	Thoracotoı	93	CHG	No	55	0	1
1	0	Thoracotoı	93	CHG	No	66	0	1
1	0	Thoracotoı	93	CHG	No	75	0	0
1	0	VATS or rc	93	CHG	No	69	0	1
1	0	VATS or rc	93	CHG	No	60	0	0
1	0	Thoracotoı	93	CHG	No	59	0	0
1	0	VATS or rc	93	CHG	No	61	0	1
1	0	Thoracotoı	93	CHG	No	67	1	0
1	0	Thoracotoı	93	CHG	No	73	0	0
1	0	Thoracotoı	93	CHG	No	75	0	0

1	0	Thoracotoı	93	CHG	No	63	0	1
1	0	VATS or rc	93	CHG	No	61	0	0
1	0	Thoracotoı	93	CHG	No	40	0	0
1	0	VATS or rc	93	CHG	No	89	0	0
1	0	Thoracotoı	93	CHG	No	78	0	1
1	0	VATS or rc	93	CHG	No	70	1	0
1	0	Thoracotoı	93	CHG	No	54	0	0
1	0	VATS or rc	93	CHG	No	81	0	0
1	0	VATS or rc	93	CHG	No	65	0	0
1	0	Thoracotoı	93	CHG	No	59	0	1
1	0	VATS or rc	93	CHG	No	69	0	1
1	0	VATS or rc	93	CHG	No	60	0	1
1	0	Thoracotoı	93	CHG	No	65	1	0
1	0	Thoracotoı	93	CHG	No	71	0	0
1	0	Thoracotoı	93	CHG	No	64	1	0
1	0	Thoracotoı	93	CHG	No	67	0	1
1	0	Thoracotoı	93	CHG	No	71	0	0
1	0	VATS or rc	93	CHG	No	43	0	1
1	0	Thoracotoı	93	CHG	No	78	0	0
1	0	Thoracotoı	93	CHG	No	81	0	0
1	0	Thoracotoı	93	CHG	No	63	0	0
1	0	Thoracotoı	93	CHG	No	53	0	1
1	0	VATS or rc	93	CHG	No	76	0	0
1	0	Thoracotoı	93	CHG	No	68	0	1
1	0	Thoracotoı	93	CHG	No	74	0	1
1	0	Thoracotoı	93	CHG	No	74	0	0
1	0	Thoracotoı	93	CHG	No	67	0	1
1	0	Thoracotoı	93	CHG	No	74	0	0
1	0	Thoracotoı	93	CHG	No	65	0	1
1	0	VATS or rc	93	CHG	No	37	0	1
1	0	VATS or rc	93	CHG	No	82	0	0
1	0	VATS or rc	93	CHG	No	48	0	1
1	1	Thoracotoı	93	CHG	No	63	0	0
1	0	VATS or rc	93	CHG	No	77	0	0
1	0	VATS or rc	93	CHG	No	64	0	0
1	0	VATS or rc	93	CHG	No	65	0	0
1	0	Thoracotoı	93	CHG	No	66	0	0
1	0	Thoracotoı	93	CHG	No	57	0	1
1	0	VATS or rc	93	CHG	No	62	0	0
1	0	Thoracotoı	93	CHG	No	72	0	0
1	0	VATS or rc	93	CHG	No	78	0	0
1	0	VATS or rc	93	CHG	No	64	0	1
1	0	VATS or rc	93	CHG	No	57	0	1
1	0	VATS or rc	93	CHG	No	54	0	0
1	0	Thoracotoı	93	CHG	No	67	1	0
1	0	VATS or rc	93	CHG	No	71	0	0
1	0	VATS or rc	93	CHG	No	63	0	0
1	0	Thoracotoı	93	CHG	No	77	0	0
1	0	Thoracotoı	93	CHG	No	74	0	0
1	0	Thoracotoı	93	CHG	No	70	0	0
1	0	Thoracotoı	93	CHG	No	79	0	0
1	0	Thoracotoı	93	CHG	No	59	0	0
1	1	Thoracotoı	93	CHG	No	25	0	0
1	0	Thoracotoı	93	CHG	No	60	0	1
1	0	Thoracotoı	93	CHG	No	77	0	1
1	0	Thoracotoı	93	CHG	No	66	0	0

1	1	Thoracotoı	93	CHG	No	58	0	0
1	0	Thoracotoı	93	CHG	No	77	0	1
1	0	Thoracotoı	93	CHG	No	70	0	0
1	0	VATS or rc	93	CHG	No	61	0	1
1	0	Thoracotoı	93	CHG	No	61	0	0
1	0	Thoracotoı	93	CHG	Yes	61	0	1
1	0	Thoracotoı	93	CHG	No	43	0	1
1	0	Thoracotoı	93	CHG	No	59	0	0
1	0	VATS or rc	93	CHG	No	72	0	0
1	0	Thoracotoı	93	CHG	No	57	0	0
1	0	Thoracotoı	93	CHG	No	81	1	0
1	0	VATS or rc	93	CHG	No	26	0	0
1	0	VATS or rc	93	CHG	No	64	0	0
1	0	VATS or rc	93	CHG	No	59	0	1
1	0	VATS or rc	93	CHG	No	55	0	0
1	0	Thoracotoı	93	CHG	No	74	0	0
1	0	VATS or rc	93	CHG	No	82	0	0
1	0	VATS or rc	93	CHG	No	62	0	1
1	0	VATS or rc	93	CHG	No	64	0	0
1	0	Thoracotoı	93	CHG	No	78	0	0
1	0	Thoracotoı	93	CHG	No	70	0	0
1	0	VATS or rc	93	CHG	No	74	0	1
1	0	VATS or rc	93	CHG	No	57	0	1
1	0	Thoracotoı	93	CHG	No	73	0	0
1	0	Thoracotoı	93	CHG	No	69	0	0
1	0	VATS or rc	93	CHG	No	64	0	1
1	1	Thoracotoı	93	CHG	No	56	0	0
1	0	Thoracotoı	93	CHG	No	75	0	0
1	0	Thoracotoı	93	CHG	No	52	0	1
1	0	VATS or rc	93	CHG	No	69	0	0
1	0	VATS or rc	93	CHG	No	82	0	0
1	0	Thoracotoı	93	CHG	No	73	0	0
1	0	Thoracotoı	93	CHG	No	71	0	0
1	0	VATS or rc	93	CHG	No	68	0	0
1	0	Thoracotoı	93	CHG	No	80	0	0
1	0	Thoracotoı	93	CHG	No	66	0	0
1	0	Thoracotoı	93	CHG	No	73	0	0
1	0	Thoracotoı	93	CHG	No	49	0	1
1	0	Thoracotoı	93	CHG	No	40	0	0
1	0	Thoracotoı	93	CHG	No	39	0	1
1	0	Thoracotoı	93	CHG	No	67	0	0
1	0	VATS or rc	93	CHG	No	63	0	0
1	0	VATS or rc	93	CHG	No	69	0	0
1	0	Thoracotoı	93	CHG	No	74	1	0
1	0	Thoracotoı	93	CHG	No	73	0	0
1	0	Thoracotoı	93	CHG	No	78	0	1
1	0	VATS or rc	93	CHG	No	71	0	0
1	0	Thoracotoı	93	CHG	No	77	1	0
1	0	Thoracotoı	93	CHG	No	70	0	1
1	0	VATS or rc	93	CHG	No	61	0	0
1	0	VATS or rc	93	CHG	No	63	1	0
1	0	Thoracotoı	93	CHG	No	74	0	0
1	0	VATS or rc	93	CHG	No	59	0	1
1	0	Thoracotoı	93	CHG	No	51	0	1
1	0	VATS or rc	93	CHG	No	71	0	0
1	0	VATS or rc	93	CHG	No	70	1	1

1	0	Thoracotoı	93	CHG	No	69	0	0
1	1	Thoracotoı	93	CHG	No	62	0	0
1	0	Thoracotoı	93	CHG	No	66	0	1
1	0	Thoracotoı	93	CHG	No	62	0	0
1	0	VATS or rc	93	CHG	No	66	0	0
1	0	VATS or rc	93	CHG	No	81	0	0
1	0	VATS or rc	93	CHG	No	54	0	1
1	0	VATS or rc	93	CHG	No	58	0	1
1	0	VATS or rc	93	CHG	No	71	0	1
1	0	Thoracotoı	93	CHG	No	67	0	0
1	0	Thoracotoı	93	CHG	No	63	0	0
1	0	Thoracotoı	93	CHG	No	64	0	0
1	0	Thoracotoı	93	CHG	No	65	0	0
1	0	VATS or rc	93	CHG	No	64	0	0
1	0	Thoracotoı	93	CHG	No	71	0	0
1	0	Thoracotoı	93	CHG	No	71	0	1
1	0	Thoracotoı	93	CHG	No	64	1	0
1	0	Thoracotoı	93	CHG	No	64	0	0
1	0	VATS or rc	93	CHG	No	72	0	0
1	0	Thoracotoı	93	CHG	No	59	0	0
1	0	Thoracotoı	93	CHG	No	64	0	1
1	0	Thoracotoı	93	CHG	No	53	0	0
1	0	Thoracotoı	93	CHG	No	54	0	1
1	0	VATS or rc	93	CHG	Yes	82	0	0
1	0	Thoracotoı	93	CHG	No	71	0	0
1	1	VATS or rc	93	CHG	No	58	0	0
1	0	VATS or rc	93	CHG	No	74	0	0
1	0	Thoracotoı	93	CHG	No	72	1	0
1	0	VATS or rc	93	CHG	No	60	0	1
1	0	VATS or rc	93	CHG	No	72	0	0
1	0	Thoracotoı	93	CHG	No	75	0	1
1	0	Thoracotoı	93	CHG	No	61	0	0
1	0	VATS or rc	93	CHG	No	63	0	1
1	0	Thoracotoı	93	CHG	No	63	0	0
1	0	Thoracotoı	93	CHG	No	56	0	0
1	0	Thoracotoı	93	CHG	No	59	0	0
1	0	VATS or rc	93	CHG	No	85	0	0
1	0	Thoracotoı	93	CHG	No	67	0	1
1	0	VATS or rc	93	CHG	No	60	0	1
1	0	Thoracotoı	93	CHG	No	69	0	1
1	0	Thoracotoı	93	CHG	No	59	0	1
1	0	Thoracotoı	93	CHG	No	77	0	0
1	0	VATS or rc	93	CHG	No	69	0	0
1	0	Thoracotoı	93	CHG	No	59	0	0
1	0	VATS or rc	93	CHG	No	70	0	0
1	0	Thoracotoı	93	CHG	No	79	0	0
1	0	Thoracotoı	93	CHG	No	62	0	1
1	0	VATS or rc	93	CHG	No	81	0	1
1	0	VATS or rc	93	CHG	No	81	0	0
1	0	VATS or rc	93	CHG	No	73	0	1
1	0	Thoracotoı	93	CHG	No	61	1	0
1	0	Thoracotoı	93	CHG	No	73	0	0
1	0	VATS or rc	93	CHG	No	66	0	1
1	0	Thoracotoı	93	CHG	No	76	0	0
1	0	Thoracotoı	93	CHG	No	39	0	0
1	0	Thoracotoı	93	CHG	No	76	0	0

1	0	VATS or rc	93	CHG	No	65	0	0
1	0	Thoracotoı	93	CHG	No	74	0	0
1	1	Thoracotoı	93	CHG	No	67	1	0
1	0	VATS or rc	93	CHG	No	74	0	0
1	0	VATS or rc	93	CHG	No	72	0	1
1	0	Thoracotoı	93	CHG	No	61	1	1
1	0	Thoracotoı	93	CHG	No	74	0	0
1	0	VATS or rc	93	CHG	No	44	0	0
1	0	VATS or rc	93	CHG	No	55	0	0
1	0	VATS or rc	93	CHG	No	57	0	1
1	0	VATS or rc	93	CHG	No	74	0	0
1	0	VATS or rc	93	CHG	No	67	0	1
1	0	VATS or rc	93	CHG	No	69	0	0
1	0	Thoracotoı	93	CHG	No	57	0	0
1	0	Thoracotoı	93	CHG	No	59	0	1
1	0	Thoracotoı	93	CHG	No	66	0	0
1	0	Thoracotoı	93	CHG	No	71	0	0
1	0	Thoracotoı	93	CHG	No	57	0	0
1	0	VATS or rc	93	CHG	No	77	0	0
1	0	Thoracotoı	93	CHG	No	74	0	0
1	0	VATS or rc	93	CHG	No	52	0	1
1	0	VATS or rc	93	CHG	No	76	0	0
1	0	Thoracotoı	93	CHG	No	62	0	1
1	0	VATS or rc	93	CHG	No	73	0	0
1	0	VATS or rc	93	CHG	No	71	0	1
1	0	Thoracotoı	93	CHG	No	61	0	1
1	0	Thoracotoı	93	CHG	No	49	0	0
1	0	VATS or rc	93	CHG	No	68	0	0
1	0	Thoracotoı	93	CHG	No	63	0	1
1	0	VATS or rc	93	CHG	No	81	0	0
1	0	Thoracotoı	93	CHG	No	53	0	0
1	0	VATS or rc	93	CHG	No	46	0	1
1	0	VATS or rc	93	CHG	No	70	0	0
1	0	VATS or rc	93	CHG	No	66	0	0
1	0	VATS or rc	93	CHG	No	65	0	0
1	0	VATS or rc	93	CHG	No	81	1	0
1	1	Thoracotoı	93	CHG	No	63	0	0
1	0	Thoracotoı	93	CHG	No	70	0	0
1	0	VATS or rc	93	CHG	No	54	0	0
1	0	Thoracotoı	93	CHG	No	79	0	0
1	1	Thoracotoı	93	CHG	No	61	0	1
1	0	VATS or rc	93	CHG	No	66	0	1
1	0	Thoracotoı	93	CHG	No	71	0	0
1	0	Thoracotoı	93	CHG	No	58	0	1
1	0	VATS or rc	93	CHG	No	74	0	0
1	0	Thoracotoı	93	CHG	No	64	0	1
1	0	Thoracotoı	93	CHG	No	64	0	0
1	0	Thoracotoı	93	CHG	No	47	0	0
1	0	VATS or rc	93	CHG	Yes	76	1	0
1	0	Thoracotoı	93	CHG	No	77	0	0
1	0	Thoracotoı	93	CHG	No	56	0	0
1	0	VATS or rc	93	CHG	No	71	0	0
1	0	Thoracotoı	93	CHG	No	67	0	0
1	0	Thoracotoı	93	CHG	No	54	0	0
1	0	VATS or rc	93	CHG	No	74	0	0
1	0	Thoracotoı	93	CHG	No	43	0	0

1	0	Thoracotoı	93	CHG	No	61	0	0
1	0	Thoracotoı	93	CHG	No	65	0	0
1	0	VATS or rc	93	CHG	No	77	0	0
1	0	Thoracotoı	93	CHG	No	67	0	0
1	0	Thoracotoı	93	CHG	No	70	0	0
1	0	Thoracotoı	93	CHG	No	85	1	0
1	0	Thoracotoı	93	CHG	No	60	1	0
1	0	VATS or rc	93	CHG	No	64	0	1
1	0	VATS or rc	93	CHG	No	76	0	0
1	0	Thoracotoı	93	CHG	No	54	0	1
1	0	Thoracotoı	93	CHG	No	65	0	0
1	0	VATS or rc	93	CHG	No	66	0	0
1	0	VATS or rc	93	CHG	No	76	0	0
1	0	Thoracotoı	93	CHG	No	75	0	0
1	0	Thoracotoı	93	CHG	No	70	0	0
1	0	Thoracotoı	93	CHG	Yes	74	1	0
1	0	Thoracotoı	93	CHG	No	69	0	1
1	0	Thoracotoı	93	CHG	No	40	0	0
1	0	Thoracotoı	93	CHG	No	63	0	0
1	0	VATS or rc	93	CHG	No	50	0	1
1	0	Thoracotoı	93	CHG	No	69	1	1
1	0	Thoracotoı	93	CHG	No	69	1	0
1	0	Thoracotoı	93	CHG	No	65	0	0
1	0	Thoracotoı	93	CHG	Yes	56	0	1
1	0	VATS or rc	93	CHG	No	60	1	1
1	0	VATS or rc	93	CHG	No	66	0	1
1	1	VATS or rc	93	CHG	No	70	0	0
1	0	Thoracotoı	93	CHG	No	81	1	0
1	0	VATS or rc	93	CHG	No	68	0	1
1	0	Thoracotoı	93	CHG	No	79	0	0
1	0	VATS or rc	93	CHG	No	56	0	0
1	0	Thoracotoı	93	CHG	No	69	0	0
1	0	VATS or rc	93	CHG	No	63	0	0
1	0	Thoracotoı	93	CHG	No	62	0	1
1	0	Thoracotoı	93	CHG	No	70	0	1
1	0	Thoracotoı	93	CHG	No	71	0	1
1	0	VATS or rc	93	CHG	No	69	0	0
1	0	VATS or rc	93	CHG	No	55	0	1
1	0	Thoracotoı	93	CHG	No	58	0	0
1	0	Thoracotoı	93	CHG	No	61	0	0
1	0	Thoracotoı	93	CHG	No	62	0	0
1	0	Thoracotoı	93	CHG	No	81	0	0
1	0	Thoracotoı	93	CHG	No	67	1	0
1	0	Thoracotoı	93	CHG	No	61	0	0
1	0	Thoracotoı	93	CHG	No	78	0	0
1	0	Thoracotoı	93	CHG	No	67	0	0
1	0	VATS or rc	93	CHG	No	70	0	0
1	0	Thoracotoı	93	CHG	No	55	0	0
1	0	VATS or rc	93	CHG	No	64	0	0
1	0	Thoracotoı	93	CHG	No	69	0	0
1	0	VATS or rc	93	CHG	No	74	0	0
1	0	VATS or rc	93	CHG	No	68	0	0
1	0	Thoracotoı	93	CHG	No	47	0	0
1	0	Thoracotoı	93	CHG	No	61	0	0
1	0	VATS or rc	93	CHG	No	56	0	0
1	0	Thoracotoı	93	CHG	No	59	0	1

1	1	VATS or ro	93	CHG	No	58	0	0
1	0	Thoracotoı	93	CHG	No	55	0	0
1	0	Thoracotoı	93	CHG	No	66	0	0
1	0	Thoracotoı	93	CHG	No	66	0	0
1	0	Thoracotoı	93	CHG	No	71	0	0
1	0	Thoracotoı	93	CHG	No	69	0	1
1	1	Thoracotoı	93	CHG	No	68	0	1
1	0	VATS or ro	93	CHG	No	56	0	0
1	1	VATS or ro	93	CHG	No	70	0	0
1	1	Thoracotoı	93	CHG	No	72	0	0
1	0	VATS or ro	93	CHG	No	69	0	0
1	0	Thoracotoı	93	CHG	No	61	0	0
1	0	Thoracotoı	93	CHG	No	51	0	1
1	0	VATS or ro	93	CHG	No	74	0	0
0	0	Thoracotoı	13	CHG	No	52	0	0
0	0	VATS or ro	42	CHG	No	64	0	0
0	0	Thoracotoı	42	CHG	No	67	0	0
0	0	Thoracotoı	42	CHG	No	57	0	1
0	0	VATS or ro	42	CHG	No	67	0	1
0	0	Thoracotoı	42	CHG	Yes	68	0	1
0	0	VATS or ro	42	CHG	Yes	73	0	1
0	0	VATS or ro	42	CHG	No	69	0	1
0	0	Thoracotoı	42	CHG	No	70	0	1
0	0	Thoracotoı	42	CHG	No	52	0	1
0	0	Thoracotoı	42	CHG	No	74	1	0
0	0	VATS or ro	42	CHG	No	65	0	1
0	1	Thoracotoı	42	CHG	No	65	0	0
0	0	VATS or ro	42	CHG	No	68	1	1
0	0	VATS or ro	42	CHG	No	56	0	1
0	0	VATS or ro	42	CHG	No	72	0	1
0	0	Thoracotoı	42	CHG	No	78	0	0
0	0	Thoracotoı	42	CHG	No	79	0	0
0	0	VATS or ro	42	CHG	No	61	0	1
0	0	VATS or ro	42	CHG	No	60	0	1
0	0	Thoracotoı	42	CHG	No	71	0	0
0	0	VATS or ro	42	CHG	No	64	0	1
0	0	Thoracotoı	42	CHG	No	65	0	0
0	0	VATS or ro	42	CHG	No	59	0	0
0	0	Thoracotoı	42	CHG	No	77	0	0
0	0	Thoracotoı	42	CHG	No	66	0	1
0	0	Thoracotoı	42	CHG	No	65	0	1
0	0	Thoracotoı	42	CHG	No	61	0	1
0	0	Thoracotoı	42	CHG	No	50	1	1
0	0	Thoracotoı	42	CHG	No	67	0	0
0	0	VATS or ro	42	CHG	No	74	0	1
0	1	Thoracotoı	42	CHG	No	69	0	0
0	0	Thoracotoı	42	CHG	No	66	0	1
0	0	Thoracotoı	42	CHG	No	66	0	1
0	0	VATS or ro	42	CHG	No	83	1	1
0	0	VATS or ro	42	CHG	No	43	0	0
0	0	VATS or ro	42	CHG	No	73	0	1
0	0	VATS or ro	42	CHG	No	66	0	1
0	0	VATS or ro	42	CHG	No	81	1	1
0	0	VATS or ro	42	CHG	No	69	0	1
0	0	Thoracotoı	42	CHG	No	62	1	1
0	0	VATS or ro	42	CHG	No	61	0	0

0	0	VATS or ro	42	CHG	No	75	1	1
0	0	VATS or ro	42	CHG	No	64	0	1
0	1	Thoracotor	42	CHG	No	73	0	1
0	0	VATS or ro	42	CHG	No	69	0	0
0	0	VATS or ro	42	CHG	No	54	1	1
0	0	Thoracotor	42	CHG	No	67	0	1
0	0	Thoracotor	42	CHG	No	62	0	1
0	0	Thoracotor	42	CHG	No	72	1	1
0	0	VATS or ro	42	CHG	No	60	0	0
0	0	VATS or ro	42	CHG	No	52	0	0
0	0	Thoracotor	42	CHG	No	67	0	0
0	0	Thoracotor	42	CHG	No	55	0	0
0	0	VATS or ro	42	CHG	No	75	0	0
0	0	VATS or ro	42	CHG	No	69	0	1
0	0	Thoracotor	42	CHG	No	62	0	1
0	0	Thoracotor	42	CHG	No	35	0	0
0	0	Thoracotor	42	CHG	No	48	0	1
0	0	Thoracotor	42	CHG	No	58	0	0
0	0	Thoracotor	42	CHG	No	71	0	0
0	0	VATS or ro	42	CHG	No	58	0	0
0	0	Thoracotor	42	CHG	No	76	0	0
0	0	Thoracotor	42	CHG	No	59	0	0
0	0	Thoracotor	42	CHG	No	41	0	1
0	0	VATS or ro	42	CHG	No	59	0	1
0	0	VATS or ro	42	CHG	No	65	0	1
0	0	VATS or ro	42	CHG	No	75	1	0
0	0	Thoracotor	42	CHG	No	66	1	1
0	0	VATS or ro	42	CHG	No	76	0	0
0	0	Thoracotor	42	CHG	No	74	0	0
0	0	VATS or ro	42	CHG	No	61	0	1
0	0	VATS or ro	42	CHG	No	67	0	1
0	0	VATS or ro	42	CHG	No	75	0	1
0	0	VATS or ro	42	CHG	No	73	0	0
0	0	VATS or ro	42	CHG	No	64	0	1
0	0	VATS or ro	42	CHG	No	67	0	1
0	0	Thoracotor	42	CHG	No	67	0	1
0	0	VATS or ro	42	CHG	No	74	0	0
0	0	VATS or ro	42	CHG	No	81	1	0
0	0	VATS or ro	42	CHG	No	75	1	0
0	0	Thoracotor	42	CHG	No	74	0	0
0	0	VATS or ro	42	CHG	No	79	0	1
0	0	Thoracotor	42	CHG	No	78	0	1
0	0	VATS or ro	42	CHG	No	68	0	0
0	0	VATS or ro	42	CHG	No	41	0	1
0	0	VATS or ro	42	CHG	No	69	0	0
0	1	Thoracotor	42	CHG	No	65	0	0
0	0	Thoracotor	42	CHG	No	62	0	0
0	1	Thoracotor	42	CHG	No	69	1	0
0	0	VATS or ro	42	CHG	No	64	0	1
0	0	VATS or ro	42	CHG	No	74	1	1
0	0	Thoracotor	42	CHG	No	72	0	0
0	0	VATS or ro	42	CHG	No	54	0	1
0	0	Thoracotor	42	CHG	No	45	0	1
0	0	VATS or ro	42	CHG	No	75	0	0
0	0	Thoracotor	42	CHG	No	72	0	1
0	0	VATS or ro	42	CHG	No	64	0	1

0	0	VATS or ro	42	CHG	No	70	0	1
0	0	Thoracotoi	42	CHG	No	64	0	0
0	0	Thoracotoi	42	CHG	No	64	0	1
0	0	Thoracotoi	42	CHG	No	48	0	1
0	0	Thoracotoi	42	CHG	No	68	0	1
0	0	VATS or ro	42	CHG	No	57	0	1
0	0	VATS or ro	42	CHG	No	72	0	0
0	0	Thoracotoi	42	CHG	No	66	0	1
0	0	Thoracotoi	42	CHG	No	66	0	1
0	0	VATS or ro	42	CHG	No	58	0	0
0	0	VATS or ro	42	CHG	No	83	0	0
0	1	Thoracotoi	66,5	PRIVE	No	70	0	0
0	0	VATS or ro	66,5	PRIVE	No	76	0	0
0	0	VATS or ro	66,5	PRIVE	No	55	0	0
0	0	VATS or ro	66,5	PRIVE	No	56	0	0
0	0	VATS or ro	66,5	PRIVE	No	56	0	0
0	0	VATS or ro	66,5	PRIVE	No	55	0	0
0	0	VATS or ro	66,5	PRIVE	No	67	0	0
0	0	VATS or ro	66,5	PRIVE	No	53	0	1
0	0	Thoracotoi	113	CHU	No	68	0	0
0	0	Thoracotoi	113	CHU	No	48	0	1
0	0	Thoracotoi	113	CHU	No	69	0	0
0	0	VATS or ro	113	CHU	No	53	0	1
0	0	Thoracotoi	113	CHU	No	65	0	0
0	0	Thoracotoi	113	CHU	No	70	0	1
0	0	Thoracotoi	113	CHU	No	83	0	0
0	0	Thoracotoi	113	CHU	No	62	0	1
0	0	VATS or ro	113	CHU	No	82	0	0
0	0	Thoracotoi	113	CHU	No	60	0	0
0	0	Thoracotoi	113	CHU	No	60	0	0
0	0	VATS or ro	113	CHU	No	78	0	0
0	0	Thoracotoi	113	CHU	No	74	0	0
0	0	Thoracotoi	113	CHU	No	62	0	1
0	0	Thoracotoi	113	CHU	No	49	1	1
0	0	VATS or ro	113	CHU	No	75	0	0
0	0	Thoracotoi	113	CHU	No	74	0	0
0	0	VATS or ro	113	CHU	No	71	0	0
0	0	Thoracotoi	113	CHU	No	66	0	0
0	0	VATS or ro	113	CHU	No	77	0	0
0	0	Thoracotoi	113	CHU	No	70	1	0
0	0	VATS or ro	113	CHU	No	64	0	1
0	0	Thoracotoi	113	CHU	No	54	0	0
0	0	Thoracotoi	113	CHU	No	68	0	0
0	0	Thoracotoi	113	CHU	No	67	0	1
0	0	Thoracotoi	113	CHU	No	49	0	1
0	0	Thoracotoi	113	CHU	No	49	0	0
0	0	Thoracotoi	113	CHU	No	80	0	0
0	0	Thoracotoi	113	CHU	No	57	0	1
0	0	Thoracotoi	113	CHU	No	57	0	0
0	0	VATS or ro	113	CHU	No	73	0	0
0	0	Thoracotoi	113	CHU	No	45	0	0
0	0	VATS or ro	113	CHU	No	60	0	1
0	0	Thoracotoi	113	CHU	No	57	0	0
0	0	Thoracotoi	113	CHU	No	70	0	0
0	0	Thoracotoi	113	CHU	No	38	0	0
0	0	VATS or ro	113	CHU	No	59	0	1

0	0	Thoracotoı	113	CHU	No	73	1	0
0	0	Thoracotoı	113	CHU	No	80	0	0
0	0	VATS or rc	113	CHU	No	70	0	0
0	0	VATS or rc	113	CHU	No	76	0	0
0	0	VATS or rc	113	CHU	No	74	0	0
0	0	Thoracotoı	113	CHU	No	43	0	0
0	0	Thoracotoı	113	CHU	No	72	0	0
0	0	Thoracotoı	113	CHU	No	57	0	0
0	0	Thoracotoı	113	CHU	No	52	0	0
0	0	VATS or rc	113	CHU	No	60	0	0
0	0	Thoracotoı	113	CHU	No	64	0	1
0	0	Thoracotoı	113	CHU	No	78	0	0
0	0	Thoracotoı	113	CHU	No	58	0	0
0	0	VATS or rc	113	CHU	No	65	0	0
0	0	Thoracotoı	113	CHU	No	84	0	0
0	0	Thoracotoı	35	PRIVE	Yes	84	0	0
0	0	Thoracotoı	35	PRIVE	No	63	0	0
0	0	Thoracotoı	35	PRIVE	No	58	1	0
0	0	Thoracotoı	35	PRIVE	No	59	0	1
0	0	Thoracotoı	35	PRIVE	No	82	0	1
0	1	Thoracotoı	35	PRIVE	No	63	0	1
0	0	Thoracotoı	35	PRIVE	No	74	0	0
0	0	Thoracotoı	35	PRIVE	No	61	0	0
0	0	Thoracotoı	35	PRIVE	No	58	1	0
0	0	Thoracotoı	35	PRIVE	No	53	0	0
0	0	Thoracotoı	35	PRIVE	No	69	1	0
0	0	Thoracotoı	35	PRIVE	No	77	0	1
0	0	Thoracotoı	35	PRIVE	No	66	0	0
0	0	Thoracotoı	35	PRIVE	No	68	0	1
0	0	Thoracotoı	35	PRIVE	No	48	0	0
0	0	Thoracotoı	35	PRIVE	No	66	0	0
0	0	Thoracotoı	35	PRIVE	No	65	0	0
0	0	Thoracotoı	35	PRIVE	No	56	0	0
0	0	Thoracotoı	35	PRIVE	No	67	1	0
0	0	Thoracotoı	35	PRIVE	No	70	0	1
0	1	Thoracotoı	35	PRIVE	No	43	0	1
0	0	Thoracotoı	35	PRIVE	No	75	0	0
0	0	Thoracotoı	35	PRIVE	No	80	0	1
0	0	Thoracotoı	35	PRIVE	No	58	0	0
0	0	Thoracotoı	35	PRIVE	No	69	0	0
0	0	Thoracotoı	35	PRIVE	No	60	0	1
0	0	Thoracotoı	35	PRIVE	No	78	0	1
0	0	Thoracotoı	35	PRIVE	No	73	0	0
0	0	Thoracotoı	35	PRIVE	No	62	0	0
0	0	Thoracotoı	35	PRIVE	No	60	0	0
0	0	Thoracotoı	35	PRIVE	No	78	0	0
0	0	Thoracotoı	35	PRIVE	No	74	1	0
0	0	Thoracotoı	35	PRIVE	No	63	1	0
0	0	Thoracotoı	35	PRIVE	No	62	0	0
0	0	Thoracotoı	35	PRIVE	No	64	0	0
0	0	Thoracotoı	35	PRIVE	No	59	0	0
0	0	Thoracotoı	35	PRIVE	No	65	0	0
0	0	Thoracotoı	35	PRIVE	No	69	0	0
0	0	Thoracotoı	35	PRIVE	No	61	0	0
0	0	Thoracotoı	35	PRIVE	No	66	0	0
0	0	Thoracotoı	35	PRIVE	No	61	0	0

0	0	Thoracotoı	35	PRIVE	No	69	0	1
0	0	Thoracotoı	35	PRIVE	No	75	0	0
0	0	Thoracotoı	35	PRIVE	No	71	0	0
0	0	Thoracotoı	35	PRIVE	No	73	0	0
0	0	Thoracotoı	35	PRIVE	No	72	0	0
0	0	Thoracotoı	35	PRIVE	No	75	0	0
0	0	Thoracotoı	35	PRIVE	No	66	0	1
0	0	Thoracotoı	35	PRIVE	No	73	0	1
0	0	Thoracotoı	35	PRIVE	No	66	0	0
0	0	Thoracotoı	35	PRIVE	No	63	0	0
0	0	Thoracotoı	35	PRIVE	No	46	0	0
0	1	Thoracotoı	35	PRIVE	No	85	0	0
0	0	Thoracotoı	35	PRIVE	No	81	0	0
0	0	Thoracotoı	35	PRIVE	No	62	0	1
0	1	Thoracotoı	35	PRIVE	No	53	0	1
0	0	Thoracotoı	35	PRIVE	No	75	0	0
0	0	Thoracotoı	35	PRIVE	No	72	0	1
0	0	Thoracotoı	35	PRIVE	No	59	1	0
0	1	Thoracotoı	35	PRIVE	No	58	0	0
0	0	Thoracotoı	35	PRIVE	No	57	0	1
0	1	Thoracotoı	35	PRIVE	No	64	1	0
0	0	VATS or rc	35	PRIVE	No	70	0	1
0	0	Thoracotoı	35	PRIVE	No	65	1	0
0	0	Thoracotoı	35	PRIVE	No	61	0	0
0	0	Thoracotoı	35	PRIVE	No	69	0	0
0	0	Thoracotoı	35	PRIVE	No	68	0	0
0	0	Thoracotoı	35	PRIVE	No	73	0	0
0	0	Thoracotoı	35	PRIVE	No	72	0	0
0	0	Thoracotoı	35	PRIVE	No	58	0	1
0	0	Thoracotoı	35	PRIVE	No	61	0	1
0	0	Thoracotoı	35	PRIVE	No	67	0	0
0	0	Thoracotoı	35	PRIVE	No	47	0	1
0	0	Thoracotoı	35	PRIVE	No	58	0	1
0	0	Thoracotoı	35	PRIVE	No	62	0	0
0	0	Thoracotoı	35	PRIVE	No	68	0	0
0	0	Thoracotoı	35	PRIVE	No	60	0	1
0	0	Thoracotoı	35	PRIVE	No	68	0	0
0	0	Thoracotoı	35	PRIVE	No	56	0	0
0	0	Thoracotoı	35	PRIVE	No	76	0	0
0	0	Thoracotoı	35	PRIVE	No	74	1	1
0	0	Thoracotoı	35	PRIVE	No	55	0	0
0	0	Thoracotoı	35	PRIVE	No	50	0	0
0	0	Thoracotoı	35	PRIVE	No	67	0	1
0	0	Thoracotoı	35	PRIVE	No	62	0	1
0	0	Thoracotoı	35	PRIVE	No	56	0	0
0	0	Thoracotoı	35	PRIVE	No	61	0	1
0	0	Thoracotoı	35	PRIVE	No	74	0	0
0	0	Thoracotoı	35	PRIVE	No	75	0	0
0	0	Thoracotoı	1029	CHU	No	74	0	0
0	0	Thoracotoı	1029	CHU	No	72	1	0
0	1	Thoracotoı	1029	CHU	No	75	0	0
0	0	VATS or rc	1029	CHU	No	74	0	0
0	1	Thoracotoı	1029	CHU	No	53	0	1
0	0	Thoracotoı	1029	CHU	No	56	0	1
0	0	Thoracotoı	1029	CHU	No	80	0	0
0	0	Thoracotoı	1029	CHU	No	57	0	0

0	0	Thoracotoı	1029	CHU	No	66	0	0
0	0	Thoracotoı	1029	CHU	No	78	0	0
0	0	VATS or ro	1029	CHU	No	43	0	0
0	0	Thoracotoı	1029	CHU	No	62	0	0
0	0	VATS or ro	1029	CHU	No	69	0	0
0	0	VATS or ro	1029	CHU	Yes	63	0	0
0	0	VATS or ro	1029	CHU	No	28	0	0
0	0	VATS or ro	1029	CHU	No	54	0	1
0	0	VATS or ro	1029	CHU	No	80	0	0
0	0	VATS or ro	1029	CHU	No	58	0	0
0	0	Thoracotoı	1029	CHU	No	70	0	1
0	0	Thoracotoı	1029	CHU	No	76	0	0
0	0	Thoracotoı	1029	CHU	No	50	0	1
0	0	VATS or ro	1029	CHU	No	77	0	0
0	0	VATS or ro	1029	CHU	No	50	0	0
0	0	VATS or ro	1029	CHU	No	72	1	0
0	0	Thoracotoı	1029	CHU	No	46	0	0
0	0	Thoracotoı	1029	CHU	No	72	0	0
0	0	Thoracotoı	1029	CHU	No	65	0	1
0	0	Thoracotoı	1029	CHU	No	61	0	1
0	0	Thoracotoı	1029	CHU	No	81	0	0
0	0	VATS or ro	1029	CHU	No	45	0	0
0	0	Thoracotoı	1029	CHU	No	66	0	1
0	0	Thoracotoı	1029	CHU	No	61	0	1
0	0	Thoracotoı	1029	CHU	No	66	0	1
0	0	Thoracotoı	1029	CHU	No	54	0	0
0	0	VATS or ro	1029	CHU	No	62	0	0
0	0	VATS or ro	1029	CHU	No	70	0	0
0	0	Thoracotoı	1029	CHU	No	81	0	0
0	0	VATS or ro	1029	CHU	No	72	0	0
0	1	Thoracotoı	1029	CHU	No	75	0	0
0	0	Thoracotoı	1029	CHU	No	66	0	1
0	0	Thoracotoı	1029	CHU	No	70	1	0
0	0	VATS or ro	1029	CHU	No	61	1	0
0	0	VATS or ro	1029	CHU	No	70	0	0
0	0	Thoracotoı	1029	CHU	No	76	0	0
0	0	Thoracotoı	1029	CHU	No	65	0	1
0	1	Thoracotoı	1029	CHU	No	67	0	0
0	0	Thoracotoı	1029	CHU	No	79	0	1
0	0	Thoracotoı	1029	CHU	No	47	0	0
0	0	Thoracotoı	1029	CHU	No	52	0	0
0	0	Thoracotoı	1029	CHU	No	64	0	1
0	0	Thoracotoı	1029	CHU	No	64	0	0
0	0	VATS or ro	1029	CHU	No	68	0	0
0	0	Thoracotoı	1029	CHU	No	66	0	0
0	0	VATS or ro	1029	CHU	No	53	0	0
0	0	Thoracotoı	1029	CHU	No	82	0	0
0	0	Thoracotoı	1029	CHU	No	52	0	1
0	0	Thoracotoı	1029	CHU	No	76	0	0
0	0	Thoracotoı	1029	CHU	No	64	0	0
0	0	Thoracotoı	1029	CHU	No	54	0	1
0	0	Thoracotoı	1029	CHU	No	65	0	1
0	1	Thoracotoı	1029	CHU	No	71	0	1
0	0	Thoracotoı	1029	CHU	No	68	0	0
0	0	Thoracotoı	1029	CHU	No	60	0	1
0	0	Thoracotoı	1029	CHU	Yes	75	1	0

0	0	Thoracotoı	1029	CHU	No	81	0	0
0	0	Thoracotoı	1029	CHU	No	65	1	1
0	0	VATS or rc	1029	CHU	No	77	0	1
0	0	Thoracotoı	1029	CHU	No	65	0	1
0	0	VATS or rc	1029	CHU	No	74	0	0
0	0	Thoracotoı	1029	CHU	No	76	1	0
0	0	Thoracotoı	1029	CHU	No	75	0	1
0	0	VATS or rc	1029	CHU	No	46	0	0
0	0	Thoracotoı	1029	CHU	No	59	0	0
0	0	VATS or rc	1029	CHU	No	61	0	1
0	0	Thoracotoı	1029	CHU	No	78	1	0
0	0	Thoracotoı	1029	CHU	No	64	1	1
0	0	Thoracotoı	1029	CHU	No	74	0	0
0	0	Thoracotoı	1029	CHU	No	69	0	0
0	0	Thoracotoı	1029	CHU	No	79	0	0
0	0	Thoracotoı	1029	CHU	No	77	0	0
0	0	Thoracotoı	1029	CHU	No	51	1	1
0	0	Thoracotoı	1029	CHU	No	40	0	0
0	0	VATS or rc	1029	CHU	No	71	0	0
0	0	Thoracotoı	1029	CHU	No	72	0	0
0	0	VATS or rc	1029	CHU	No	57	0	0
0	0	Thoracotoı	1029	CHU	No	20	0	0
0	0	Thoracotoı	1029	CHU	No	88	0	0
0	0	Thoracotoı	1029	CHU	No	52	0	1
0	0	Thoracotoı	1029	CHU	No	68	0	0
0	0	Thoracotoı	1029	CHU	No	67	0	0
0	0	Thoracotoı	1029	CHU	No	57	0	1
0	0	Thoracotoı	1029	CHU	No	59	0	1
0	0	Thoracotoı	1029	CHU	No	61	0	0
0	0	Thoracotoı	1029	CHU	No	64	0	0
0	0	VATS or rc	1029	CHU	No	73	0	1
0	0	VATS or rc	1029	CHU	No	60	0	0
0	0	Thoracotoı	1029	CHU	No	70	0	0
0	0	Thoracotoı	1029	CHU	No	63	0	0
0	0	Thoracotoı	1029	CHU	No	65	0	0
0	0	VATS or rc	1029	CHU	No	70	0	0
0	0	Thoracotoı	1029	CHU	No	49	0	1
0	1	Thoracotoı	1029	CHU	No	49	0	1
0	0	VATS or rc	1029	CHU	No	63	0	1
0	0	Thoracotoı	1029	CHU	No	30	0	0
0	0	Thoracotoı	1029	CHU	No	71	1	1
0	0	VATS or rc	1029	CHU	No	72	0	1
0	0	Thoracotoı	1029	CHU	No	60	0	0
0	0	VATS or rc	1029	CHU	No	65	1	0
1	0	Thoracotoı	123	PRIVE	No	67	0	0
1	0	Thoracotoı	123	PRIVE	No	47	0	0
1	0	VATS or rc	123	PRIVE	No	74	0	0
1	0	VATS or rc	123	PRIVE	No	67	0	0
1	0	VATS or rc	123	PRIVE	No	69	0	0
1	1	Thoracotoı	123	PRIVE	No	56	1	0
1	0	Thoracotoı	123	PRIVE	No	76	1	0
1	0	Thoracotoı	123	PRIVE	No	74	0	0
1	0	Thoracotoı	123	PRIVE	No	58	0	0
1	0	VATS or rc	123	PRIVE	No	64	0	0
1	0	VATS or rc	123	PRIVE	No	56	0	1
1	0	VATS or rc	123	PRIVE	No	63	0	0

1	0	Thoracotor	123	PRIVE	No	67	0	0
1	0	Thoracotor	123	PRIVE	No	66	0	0
1	0	VATS or ro	123	PRIVE	No	82	0	0
1	0	Thoracotor	123	PRIVE	No	55	0	0
1	0	Thoracotor	123	PRIVE	No	60	0	1
1	0	Thoracotor	123	PRIVE	No	71	0	0
1	0	VATS or ro	123	PRIVE	No	59	0	0
1	0	VATS or ro	123	PRIVE	No	56	0	1
1	0	VATS or ro	123	PRIVE	No	81	0	0
1	0	Thoracotor	123	PRIVE	No	68	0	0
1	0	Thoracotor	123	PRIVE	No	73	0	1
1	0	Thoracotor	123	PRIVE	No	76	0	0
1	0	Thoracotor	123	PRIVE	No	60	0	0
1	0	VATS or ro	123	PRIVE	No	59	0	0
1	0	VATS or ro	123	PRIVE	No	75	0	0
1	0	VATS or ro	123	PRIVE	No	54	0	0
1	0	Thoracotor	123	PRIVE	No	66	0	0
1	0	Thoracotor	123	PRIVE	Yes	86	0	0
1	0	VATS or ro	123	PRIVE	No	45	0	1
1	0	VATS or ro	123	PRIVE	No	55	0	0
1	0	Thoracotor	123	PRIVE	No	49	0	1
1	0	Thoracotor	123	PRIVE	No	65	0	0
1	0	VATS or ro	123	PRIVE	No	54	0	1
1	0	VATS or ro	123	PRIVE	No	67	0	1
1	0	Thoracotor	123	PRIVE	No	57	0	0
1	0	VATS or ro	123	PRIVE	No	66	0	0
1	0	Thoracotor	123	PRIVE	No	60	0	1
1	0	VATS or ro	123	PRIVE	No	63	0	0
1	0	Thoracotor	123	PRIVE	No	59	0	0
1	0	Thoracotor	123	PRIVE	No	59	0	0
1	0	Thoracotor	123	PRIVE	No	70	0	0
1	0	VATS or ro	123	PRIVE	No	61	0	1
1	0	Thoracotor	123	PRIVE	No	65	0	0
1	0	Thoracotor	123	PRIVE	No	63	1	0
1	1	Thoracotor	123	PRIVE	No	66	0	0
1	0	Thoracotor	123	PRIVE	No	68	0	1
1	0	Thoracotor	123	PRIVE	No	68	1	0
1	0	Thoracotor	123	PRIVE	No	65	0	0
1	0	VATS or ro	123	PRIVE	No	66	0	0
1	0	VATS or ro	123	PRIVE	No	65	0	0
1	0	Thoracotor	123	PRIVE	No	81	0	0
1	0	VATS or ro	123	PRIVE	No	58	1	0
1	0	Thoracotor	123	PRIVE	No	82	0	1
1	1	Thoracotor	123	PRIVE	No	45	0	0
1	0	Thoracotor	123	PRIVE	No	63	0	0
1	0	Thoracotor	123	PRIVE	No	64	0	0
1	0	Thoracotor	123	PRIVE	No	78	1	0
1	1	Thoracotor	123	PRIVE	No	57	0	0
1	0	Thoracotor	123	PRIVE	No	78	0	0
1	0	VATS or ro	123	PRIVE	No	55	0	0
1	0	VATS or ro	123	PRIVE	No	72	0	0
1	0	VATS or ro	123	PRIVE	No	66	0	0
1	0	Thoracotor	123	PRIVE	No	65	0	0
1	0	VATS or ro	123	PRIVE	No	54	0	0
1	0	VATS or ro	123	PRIVE	No	60	0	0
1	0	VATS or ro	123	PRIVE	No	38	0	1

1	0	Thoracotoı	123	PRIVE	No	64	0	0
1	0	Thoracotoı	123	PRIVE	No	66	0	0
1	0	VATS or rc	123	PRIVE	No	59	0	0
1	0	VATS or rc	123	PRIVE	No	68	0	1
1	0	Thoracotoı	123	PRIVE	No	64	0	0
1	0	VATS or rc	123	PRIVE	No	55	0	0
1	0	Thoracotoı	123	PRIVE	No	68	0	0
1	0	VATS or rc	123	PRIVE	No	61	0	0
1	0	VATS or rc	123	PRIVE	No	75	0	0
1	0	Thoracotoı	123	PRIVE	No	59	0	0
1	0	VATS or rc	123	PRIVE	No	57	0	0
1	0	VATS or rc	123	PRIVE	No	71	0	0
1	1	Thoracotoı	123	PRIVE	No	59	0	0
1	0	VATS or rc	123	PRIVE	No	65	0	0
1	0	Thoracotoı	123	PRIVE	No	58	1	0
1	0	Thoracotoı	123	PRIVE	No	86	0	0
1	0	Thoracotoı	123	PRIVE	No	77	0	0
1	0	Thoracotoı	123	PRIVE	No	65	0	0
1	0	Thoracotoı	123	PRIVE	No	57	0	0
1	0	VATS or rc	123	PRIVE	No	60	0	0
1	0	VATS or rc	123	PRIVE	No	71	0	0
1	0	Thoracotoı	123	PRIVE	No	59	0	0
1	0	Thoracotoı	123	PRIVE	No	56	0	0
1	0	Thoracotoı	123	PRIVE	No	65	0	0
1	0	Thoracotoı	123	PRIVE	No	82	0	0
1	0	VATS or rc	123	PRIVE	No	58	0	0
1	0	Thoracotoı	123	PRIVE	No	35	0	0
1	0	VATS or rc	123	PRIVE	No	63	0	0
1	0	Thoracotoı	123	PRIVE	No	67	0	0
1	0	VATS or rc	123	PRIVE	No	45	0	0
1	0	Thoracotoı	123	PRIVE	No	57	0	0
1	0	Thoracotoı	123	PRIVE	No	69	0	0
1	0	Thoracotoı	123	PRIVE	No	77	1	0
1	0	Thoracotoı	123	PRIVE	No	70	1	0
1	0	VATS or rc	123	PRIVE	No	55	0	1
1	0	Thoracotoı	123	PRIVE	No	72	0	0
1	0	Thoracotoı	123	PRIVE	No	67	0	0
1	0	Thoracotoı	123	PRIVE	No	50	0	1
1	0	Thoracotoı	123	PRIVE	No	67	1	0
1	0	Thoracotoı	123	PRIVE	No	72	0	0
1	0	VATS or rc	123	PRIVE	No	52	0	0
1	0	VATS or rc	123	PRIVE	No	69	0	0
1	0	VATS or rc	123	PRIVE	No	46	0	1
1	0	VATS or rc	123	PRIVE	No	58	0	0
1	0	Thoracotoı	123	PRIVE	No	51	0	0
1	0	Thoracotoı	123	PRIVE	No	64	0	0
1	0	VATS or rc	123	PRIVE	No	76	0	0
1	0	VATS or rc	123	PRIVE	No	57	0	0
1	0	VATS or rc	123	PRIVE	No	63	0	0
1	0	VATS or rc	123	PRIVE	No	68	0	0
1	0	VATS or rc	123	PRIVE	No	69	0	0
1	0	Thoracotoı	123	PRIVE	No	33	0	0
1	0	Thoracotoı	123	PRIVE	No	64	0	0
1	0	Thoracotoı	123	PRIVE	No	58	0	0
1	0	VATS or rc	123	PRIVE	No	77	1	0
1	0	VATS or rc	123	PRIVE	No	64	0	0

1	0	Thoracotoı	123	PRIVE	No	36	0	0
1	0	VATS or rc	123	PRIVE	No	72	0	0
1	0	VATS or rc	123	PRIVE	No	70	0	0
1	0	Thoracotoı	123	PRIVE	No	64	0	0
1	0	VATS or rc	123	PRIVE	No	71	0	0
1	0	Thoracotoı	123	PRIVE	No	61	0	0
1	0	Thoracotoı	123	PRIVE	No	62	0	1
1	0	Thoracotoı	123	PRIVE	No	64	0	1
1	0	Thoracotoı	123	PRIVE	No	66	0	0
1	0	VATS or rc	123	PRIVE	No	50	0	0
1	0	Thoracotoı	123	PRIVE	No	61	0	0
1	1	Thoracotoı	123	PRIVE	No	64	0	0
1	1	Thoracotoı	123	PRIVE	No	57	0	0
1	0	VATS or rc	123	PRIVE	No	61	0	0
1	0	VATS or rc	123	PRIVE	No	73	0	0
1	0	VATS or rc	123	PRIVE	No	62	0	0
1	0	VATS or rc	123	PRIVE	No	76	0	0
1	0	VATS or rc	123	PRIVE	No	69	0	0
1	0	VATS or rc	123	PRIVE	No	67	0	0
1	0	VATS or rc	123	PRIVE	No	74	0	0
1	0	Thoracotoı	123	PRIVE	Yes	81	0	0
1	0	Thoracotoı	123	PRIVE	No	63	0	0
1	0	Thoracotoı	123	PRIVE	No	66	0	0
1	0	Thoracotoı	123	PRIVE	No	59	0	0
1	0	VATS or rc	123	PRIVE	No	84	1	0
1	0	VATS or rc	123	PRIVE	No	59	0	1
1	0	VATS or rc	123	PRIVE	No	64	0	0
1	0	VATS or rc	123	PRIVE	No	72	0	0
1	0	VATS or rc	123	PRIVE	No	59	0	0
1	0	Thoracotoı	123	PRIVE	No	54	0	1
1	0	VATS or rc	123	PRIVE	No	55	1	0
1	0	Thoracotoı	123	PRIVE	No	75	1	0
1	1	Thoracotoı	123	PRIVE	No	62	0	0
1	0	Thoracotoı	123	PRIVE	No	66	0	0
1	0	VATS or rc	123	PRIVE	No	64	0	0
1	0	Thoracotoı	123	PRIVE	No	64	0	1
1	0	Thoracotoı	123	PRIVE	No	66	0	0
1	0	VATS or rc	123	PRIVE	No	75	0	0
1	0	Thoracotoı	123	PRIVE	No	69	0	0
1	0	Thoracotoı	123	PRIVE	No	68	0	0
1	0	Thoracotoı	123	PRIVE	No	65	0	0
1	0	Thoracotoı	123	PRIVE	No	81	0	0
1	0	Thoracotoı	123	PRIVE	No	46	0	0
1	0	Thoracotoı	123	PRIVE	No	75	1	0
1	0	Thoracotoı	123	PRIVE	No	63	0	0
1	0	VATS or rc	123	PRIVE	No	77	1	0
1	0	VATS or rc	123	PRIVE	No	61	0	1
1	0	VATS or rc	123	PRIVE	No	74	0	0
1	1	Thoracotoı	123	PRIVE	No	76	0	0
1	0	Thoracotoı	123	PRIVE	No	63	0	0
1	0	VATS or rc	123	PRIVE	No	61	0	1
1	0	Thoracotoı	123	PRIVE	No	68	0	1
1	0	VATS or rc	123	PRIVE	No	72	0	0
1	0	Thoracotoı	123	PRIVE	No	51	0	0
1	0	Thoracotoı	123	PRIVE	No	62	0	0
1	0	Thoracotoı	123	PRIVE	No	56	0	0

1	0	VATS or ro	123	PRIVE	No	75	0	0
1	0	Thoracotoı	123	PRIVE	No	57	0	0
1	0	Thoracotoı	123	PRIVE	No	61	1	0
1	0	Thoracotoı	123	PRIVE	No	69	0	0
1	0	Thoracotoı	123	PRIVE	No	62	0	1
1	0	VATS or ro	123	PRIVE	No	67	1	0
1	0	Thoracotoı	123	PRIVE	No	75	1	0
1	0	Thoracotoı	123	PRIVE	No	81	1	0
1	0	VATS or ro	123	PRIVE	No	77	1	0
1	0	Thoracotoı	123	PRIVE	No	58	0	0
1	0	Thoracotoı	123	PRIVE	No	62	0	1
1	0	VATS or ro	123	PRIVE	No	53	0	1
1	0	Thoracotoı	123	PRIVE	No	56	0	0
1	0	Thoracotoı	123	PRIVE	No	63	0	0
1	0	Thoracotoı	123	PRIVE	No	72	0	0
1	0	VATS or ro	123	PRIVE	No	73	0	0
1	0	VATS or ro	123	PRIVE	No	51	0	0
1	0	Thoracotoı	123	PRIVE	No	60	0	1
1	0	Thoracotoı	123	PRIVE	No	60	0	0
1	0	VATS or ro	123	PRIVE	No	68	0	0
1	1	Thoracotoı	123	PRIVE	No	49	0	0
1	0	Thoracotoı	123	PRIVE	No	66	1	0
1	0	VATS or ro	123	PRIVE	No	70	0	0
1	0	VATS or ro	123	PRIVE	No	59	0	1
1	0	VATS or ro	123	PRIVE	No	67	0	0
1	0	VATS or ro	123	PRIVE	No	69	0	1
1	0	Thoracotoı	123	PRIVE	No	66	0	0
1	0	Thoracotoı	123	PRIVE	No	74	0	0
1	0	Thoracotoı	123	PRIVE	No	60	0	0
1	0	Thoracotoı	123	PRIVE	No	61	0	0
1	0	VATS or ro	123	PRIVE	No	66	0	1
1	0	Thoracotoı	123	PRIVE	No	61	0	0
1	0	VATS or ro	123	PRIVE	No	69	0	0
1	0	Thoracotoı	123	PRIVE	No	68	0	0
1	0	Thoracotoı	123	PRIVE	No	80	1	0
1	0	Thoracotoı	123	PRIVE	No	66	0	0
1	0	VATS or ro	123	PRIVE	No	71	0	0
1	0	VATS or ro	123	PRIVE	No	59	0	0
1	0	Thoracotoı	123	PRIVE	No	57	0	1
1	0	Thoracotoı	123	PRIVE	Yes	73	0	0
1	1	Thoracotoı	123	PRIVE	No	60	0	0
1	0	VATS or ro	123	PRIVE	No	72	0	0
1	0	Thoracotoı	123	PRIVE	No	78	0	0
1	0	Thoracotoı	123	PRIVE	No	65	0	0
1	0	VATS or ro	123	PRIVE	No	72	0	0
1	0	VATS or ro	123	PRIVE	No	74	0	0
1	0	VATS or ro	123	PRIVE	No	67	0	0
1	0	Thoracotoı	123	PRIVE	No	76	0	0
1	0	Thoracotoı	123	PRIVE	No	74	0	0
1	0	Thoracotoı	123	PRIVE	No	69	0	0
1	0	VATS or ro	123	PRIVE	No	56	0	0
1	0	VATS or ro	123	PRIVE	No	47	0	1
1	0	VATS or ro	123	PRIVE	No	69	0	0
1	0	Thoracotoı	123	PRIVE	No	52	0	0
1	0	VATS or ro	123	PRIVE	No	78	0	0
1	0	Thoracotoı	123	PRIVE	No	66	0	0

1	0	Thoracotoı	123	PRIVE	No	76	0	0
1	0	Thoracotoı	123	PRIVE	No	77	0	0
1	0	VATS or rc	123	PRIVE	No	53	0	0
1	0	Thoracotoı	123	PRIVE	No	76	1	0
1	0	VATS or rc	123	PRIVE	No	67	0	0
1	0	VATS or rc	123	PRIVE	No	62	0	0
1	0	Thoracotoı	123	PRIVE	No	64	0	0
1	0	VATS or rc	123	PRIVE	No	57	0	0
1	0	Thoracotoı	123	PRIVE	No	66	0	0
1	0	VATS or rc	123	PRIVE	No	70	1	0
1	0	Thoracotoı	123	PRIVE	No	75	0	0
1	0	VATS or rc	123	PRIVE	No	49	0	1
1	0	Thoracotoı	123	PRIVE	No	78	0	0
1	0	Thoracotoı	123	PRIVE	No	64	0	0
1	0	Thoracotoı	123	PRIVE	No	62	0	0
1	0	VATS or rc	123	PRIVE	No	77	0	0
1	0	Thoracotoı	123	PRIVE	No	59	0	0
1	0	VATS or rc	123	PRIVE	No	76	0	0
1	0	Thoracotoı	123	PRIVE	No	70	0	0
1	0	Thoracotoı	123	PRIVE	No	57	0	0
1	0	Thoracotoı	123	PRIVE	No	68	0	0
1	0	Thoracotoı	123	PRIVE	No	51	0	0
1	0	VATS or rc	123	PRIVE	No	55	0	0
1	0	Thoracotoı	123	PRIVE	No	59	0	1
1	0	VATS or rc	123	PRIVE	No	59	0	0
1	0	VATS or rc	123	PRIVE	No	59	0	0
1	0	VATS or rc	123	PRIVE	No	71	0	0
1	0	Thoracotoı	123	PRIVE	No	75	0	0
1	0	VATS or rc	123	PRIVE	No	55	0	0
1	0	Thoracotoı	123	PRIVE	No	65	0	0
1	0	VATS or rc	123	PRIVE	No	63	0	1
1	0	VATS or rc	123	PRIVE	No	57	0	0
1	0	VATS or rc	123	PRIVE	No	73	0	0
1	0	VATS or rc	123	PRIVE	No	55	0	0
1	0	Thoracotoı	123	PRIVE	No	69	0	0
1	0	Thoracotoı	123	PRIVE	No	53	0	0
1	0	Thoracotoı	123	PRIVE	No	81	0	0
1	0	VATS or rc	123	PRIVE	No	78	0	0
1	0	Thoracotoı	123	PRIVE	No	61	0	0
1	0	Thoracotoı	123	PRIVE	No	72	0	0
1	0	VATS or rc	123	PRIVE	No	69	0	0
1	0	VATS or rc	123	PRIVE	No	56	0	1
1	0	Thoracotoı	123	PRIVE	No	76	0	0
1	0	Thoracotoı	123	PRIVE	No	77	0	0
1	0	VATS or rc	123	PRIVE	No	60	0	0
1	0	VATS or rc	123	PRIVE	No	56	0	0
1	0	VATS or rc	123	PRIVE	No	73	0	0
1	0	VATS or rc	123	PRIVE	No	69	0	0
1	0	Thoracotoı	123	PRIVE	No	63	0	0
1	0	Thoracotoı	123	PRIVE	No	63	0	0
1	0	Thoracotoı	123	PRIVE	No	78	0	0
1	0	VATS or rc	123	PRIVE	No	81	0	0
1	1	Thoracotoı	123	PRIVE	No	69	0	0
1	0	Thoracotoı	123	PRIVE	No	72	0	0
1	0	VATS or rc	123	PRIVE	No	72	0	0
1	0	Thoracotoı	123	PRIVE	No	72	0	0

1	0	VATS or rc	123	PRIVE	No	62	0	1
1	0	VATS or rc	123	PRIVE	No	76	0	0
1	0	Thoracotor	123	PRIVE	No	62	0	0
1	0	VATS or rc	123	PRIVE	No	63	0	0
1	0	VATS or rc	123	PRIVE	No	63	1	0
1	0	VATS or rc	123	PRIVE	No	75	1	0
1	0	Thoracotor	123	PRIVE	No	75	0	0
1	0	Thoracotor	123	PRIVE	No	79	0	0
1	0	VATS or rc	123	PRIVE	No	82	0	0
1	0	Thoracotor	123	PRIVE	No	57	0	0
1	0	Thoracotor	123	PRIVE	No	59	0	0
1	0	VATS or rc	123	PRIVE	No	75	0	0
1	0	Thoracotor	123	PRIVE	No	54	0	0
0	0	Thoracotor	96	PRIVE	No	74	0	0
0	0	Thoracotor	96	PRIVE	No	53	0	0
0	0	Thoracotor	96	PRIVE	No	56	0	0
0	0	VATS or rc	96	PRIVE	No	70	0	0
0	0	Thoracotor	96	PRIVE	No	63	0	1
0	0	Thoracotor	96	PRIVE	No	79	0	0
0	0	VATS or rc	96	PRIVE	No	64	0	0
0	0	Thoracotor	96	PRIVE	No	75	0	0
0	0	Thoracotor	96	PRIVE	No	71	0	0
0	0	Thoracotor	96	PRIVE	No	57	0	1
0	0	Thoracotor	96	PRIVE	No	59	0	0
0	0	VATS or rc	96	PRIVE	No	54	0	0
0	0	Thoracotor	96	PRIVE	No	77	0	1
0	0	VATS or rc	96	PRIVE	No	63	0	0
0	0	VATS or rc	96	PRIVE	No	68	0	0
0	0	VATS or rc	96	PRIVE	No	69	0	0
0	0	Thoracotor	96	PRIVE	No	49	0	1
0	0	Thoracotor	96	PRIVE	No	77	0	0
0	0	Thoracotor	96	PRIVE	No	71	0	0
0	0	VATS or rc	96	PRIVE	No	57	0	0
0	0	Thoracotor	96	PRIVE	No	61	0	0
0	1	Thoracotor	96	PRIVE	No	54	0	1
0	0	VATS or rc	96	PRIVE	No	75	0	0
0	0	VATS or rc	96	PRIVE	No	68	0	0
0	0	Thoracotor	96	PRIVE	No	68	0	0
0	0	VATS or rc	96	PRIVE	No	70	0	0
0	0	Thoracotor	96	PRIVE	No	48	0	0
0	0	Thoracotor	96	PRIVE	No	63	0	0
0	0	VATS or rc	96	PRIVE	No	68	0	0
0	1	Thoracotor	96	PRIVE	No	60	0	0
0	1	Thoracotor	96	PRIVE	No	55	1	0
0	0	Thoracotor	96	PRIVE	No	66	0	0
0	0	Thoracotor	96	PRIVE	No	69	0	1
0	0	Thoracotor	96	PRIVE	No	71	1	0
0	0	VATS or rc	96	PRIVE	No	67	0	0
0	0	VATS or rc	96	PRIVE	No	53	0	0
0	0	Thoracotor	96	PRIVE	No	64	1	0
0	0	VATS or rc	96	PRIVE	No	64	0	0
0	0	VATS or rc	96	PRIVE	No	71	0	0
0	0	Thoracotor	96	PRIVE	No	56	0	0
0	0	Thoracotor	96	PRIVE	No	48	0	0
0	0	Thoracotor	96	PRIVE	No	62	0	0
0	0	VATS or rc	96	PRIVE	No	72	1	0

0	0	VATS or rc	96	PRIVE	No	64	0	0
0	0	Thoracotoı	96	PRIVE	No	57	0	1
0	0	Thoracotoı	96	PRIVE	No	73	0	0
0	1	VATS or rc	96	PRIVE	No	51	0	0
0	0	Thoracotoı	96	PRIVE	No	67	0	1
0	1	Thoracotoı	96	PRIVE	No	48	0	0
0	0	Thoracotoı	96	PRIVE	No	70	0	0
0	0	VATS or rc	96	PRIVE	No	56	0	0
0	0	VATS or rc	96	PRIVE	No	72	1	0
0	0	VATS or rc	96	PRIVE	No	73	1	0
0	0	VATS or rc	96	PRIVE	No	70	1	0
0	0	VATS or rc	96	PRIVE	No	64	0	0
0	0	VATS or rc	96	PRIVE	No	57	0	0
0	0	Thoracotoı	96	PRIVE	No	76	0	1
0	0	VATS or rc	96	PRIVE	No	64	0	0
0	0	Thoracotoı	96	PRIVE	No	51	0	1
0	1	Thoracotoı	96	PRIVE	No	77	0	0
0	0	Thoracotoı	96	PRIVE	No	69	1	0
0	0	VATS or rc	96	PRIVE	No	60	0	0
0	0	Thoracotoı	96	PRIVE	No	65	0	0
0	0	Thoracotoı	96	PRIVE	No	76	0	1
0	0	Thoracotoı	96	PRIVE	No	53	0	0
0	0	Thoracotoı	96	PRIVE	No	48	0	0
0	0	VATS or rc	96	PRIVE	No	65	0	0
0	0	VATS or rc	96	PRIVE	No	62	0	0
0	0	Thoracotoı	96	PRIVE	No	58	0	0
0	0	Thoracotoı	96	PRIVE	No	68	0	0
0	0	VATS or rc	96	PRIVE	No	61	0	0
0	0	Thoracotoı	96	PRIVE	No	62	0	0
0	1	Thoracotoı	96	PRIVE	No	71	0	0
0	0	Thoracotoı	96	PRIVE	No	63	1	0
0	0	Thoracotoı	96	PRIVE	No	75	0	0
0	0	Thoracotoı	96	PRIVE	No	57	0	0
0	0	VATS or rc	96	PRIVE	No	63	0	0
0	0	Thoracotoı	96	PRIVE	No	69	1	0
0	0	Thoracotoı	96	PRIVE	No	68	0	0
0	0	Thoracotoı	96	PRIVE	Yes	59	1	1
0	0	VATS or rc	96	PRIVE	No	69	0	0
0	0	Thoracotoı	96	PRIVE	No	75	0	0
0	0	VATS or rc	96	PRIVE	No	80	0	0
0	0	VATS or rc	96	PRIVE	No	71	0	0
0	0	Thoracotoı	96	PRIVE	No	67	0	1
0	0	VATS or rc	96	PRIVE	No	62	0	0
0	0	VATS or rc	96	PRIVE	No	74	0	0
0	0	VATS or rc	96	PRIVE	No	52	0	0
0	0	VATS or rc	96	PRIVE	No	58	0	0
0	0	Thoracotoı	96	PRIVE	No	59	0	0
0	0	Thoracotoı	96	PRIVE	No	88	0	0
0	0	VATS or rc	96	PRIVE	No	58	0	0
0	1	VATS or rc	96	PRIVE	No	51	0	0
0	0	VATS or rc	96	PRIVE	No	61	0	0
0	0	Thoracotoı	96	PRIVE	No	62	0	1
0	0	VATS or rc	96	PRIVE	No	76	0	0
0	0	VATS or rc	96	PRIVE	No	74	0	1
0	0	Thoracotoı	96	PRIVE	No	56	0	1
0	0	Thoracotoı	96	PRIVE	No	79	0	0

0	0	Thoracotoı	96	PRIVE	No	62	0	1
0	0	Thoracotoı	96	PRIVE	No	58	0	0
0	0	Thoracotoı	96	PRIVE	No	73	0	0
0	0	Thoracotoı	96	PRIVE	No	66	0	0
0	0	Thoracotoı	96	PRIVE	No	69	1	0
0	0	VATS or rc	96	PRIVE	No	76	0	0
0	0	VATS or rc	96	PRIVE	No	74	0	0
0	0	Thoracotoı	96	PRIVE	No	43	0	0
0	1	Thoracotoı	96	PRIVE	No	71	0	0
0	0	Thoracotoı	96	PRIVE	No	72	0	1
0	0	Thoracotoı	96	PRIVE	No	52	0	1
0	1	Thoracotoı	96	PRIVE	No	60	0	0
0	1	Thoracotoı	96	PRIVE	No	54	0	0
0	0	Thoracotoı	96	PRIVE	No	76	0	1
0	0	Thoracotoı	96	PRIVE	No	61	0	0
0	0	VATS or rc	96	PRIVE	No	58	0	0
0	0	Thoracotoı	96	PRIVE	No	69	0	0
0	0	Thoracotoı	96	PRIVE	No	56	0	0
0	0	Thoracotoı	96	PRIVE	No	60	0	0
0	0	VATS or rc	96	PRIVE	No	62	0	0
0	0	VATS or rc	96	PRIVE	No	70	0	0
0	1	Thoracotoı	96	PRIVE	No	59	0	0
0	0	VATS or rc	96	PRIVE	No	78	0	0
0	0	Thoracotoı	96	PRIVE	No	82	0	0
0	0	VATS or rc	96	PRIVE	No	65	0	0
0	0	Thoracotoı	96	PRIVE	No	68	1	0
0	0	VATS or rc	96	PRIVE	No	74	0	0
0	0	Thoracotoı	96	PRIVE	No	52	0	1
0	0	VATS or rc	96	PRIVE	No	66	0	0
0	0	VATS or rc	96	PRIVE	No	56	0	0
0	0	Thoracotoı	96	PRIVE	Yes	77	0	0
0	0	VATS or rc	96	PRIVE	No	67	0	0
0	0	Thoracotoı	96	PRIVE	No	61	0	0
0	0	VATS or rc	96	PRIVE	No	63	0	0
0	0	VATS or rc	96	PRIVE	No	60	0	0
0	0	Thoracotoı	96	PRIVE	No	59	1	0
0	0	Thoracotoı	96	PRIVE	No	75	0	0
0	0	VATS or rc	96	PRIVE	No	72	0	0
0	0	VATS or rc	96	PRIVE	No	72	0	0
0	1	Thoracotoı	96	PRIVE	No	75	0	0
0	0	Thoracotoı	96	PRIVE	No	63	0	0
0	0	VATS or rc	96	PRIVE	No	71	0	0
0	0	Thoracotoı	96	PRIVE	No	71	0	0
0	0	VATS or rc	96	PRIVE	No	66	0	0
0	0	Thoracotoı	96	PRIVE	No	67	0	0
0	0	VATS or rc	96	PRIVE	No	60	0	0
0	0	Thoracotoı	96	PRIVE	No	58	1	0
0	0	VATS or rc	96	PRIVE	No	48	0	0
0	1	VATS or rc	96	PRIVE	No	71	0	0
0	0	Thoracotoı	96	PRIVE	No	74	0	0
0	0	Thoracotoı	96	PRIVE	No	66	1	1
0	0	VATS or rc	96	PRIVE	No	55	0	0
0	0	VATS or rc	96	PRIVE	No	46	0	0
0	0	VATS or rc	96	PRIVE	No	60	0	0
0	0	VATS or rc	96	PRIVE	No	74	0	0
0	0	Thoracotoı	96	PRIVE	No	65	1	0

0	0	VATS or rc	96	PRIVE	No	75	0	0
0	0	VATS or rc	96	PRIVE	No	78	1	0
0	0	VATS or rc	96	PRIVE	No	66	0	0
0	0	Thoracotoı	96	PRIVE	No	69	0	0
0	0	VATS or rc	96	PRIVE	No	55	0	1
0	0	VATS or rc	96	PRIVE	No	64	0	0
0	0	Thoracotoı	96	PRIVE	No	68	0	0
0	0	Thoracotoı	96	PRIVE	No	47	0	0
0	0	VATS or rc	96	PRIVE	No	58	0	0
0	0	VATS or rc	96	PRIVE	No	76	1	0
0	0	VATS or rc	96	PRIVE	No	72	0	0
0	0	VATS or rc	96	PRIVE	No	54	0	0
0	0	Thoracotoı	96	PRIVE	No	59	0	0
0	0	VATS or rc	96	PRIVE	No	63	0	0
0	0	VATS or rc	96	PRIVE	No	78	0	0
0	0	VATS or rc	96	PRIVE	No	57	0	0
0	1	Thoracotoı	96	PRIVE	No	67	0	0
0	0	VATS or rc	96	PRIVE	No	74	0	0
0	0	Thoracotoı	96	PRIVE	No	64	0	0
0	0	Thoracotoı	96	PRIVE	Yes	60	0	0
0	0	VATS or rc	96	PRIVE	No	67	0	0
0	1	Thoracotoı	96	PRIVE	No	59	0	0
0	0	Thoracotoı	31	CHG	No	76	0	1
0	0	Thoracotoı	31	CHG	No	76	0	0
0	0	Thoracotoı	31	CHG	No	70	1	0
0	0	Thoracotoı	31	CHG	No	66	0	1
0	0	Thoracotoı	31	CHG	No	61	1	1
0	0	Thoracotoı	31	CHG	No	56	0	1
0	0	Thoracotoı	31	CHG	No	57	1	0
0	0	Thoracotoı	31	CHG	No	66	0	1
0	0	Thoracotoı	31	CHG	No	52	0	1
0	0	Thoracotoı	31	CHG	No	70	0	1
0	0	Thoracotoı	31	CHG	No	66	0	0
0	1	Thoracotoı	31	CHG	No	62	0	1
0	0	Thoracotoı	31	CHG	No	63	0	0
0	0	Thoracotoı	31	CHG	No	64	1	0
0	0	Thoracotoı	31	CHG	No	75	1	0
1	0	Thoracotoı	12,5	CHG	No	72	0	0
1	0	VATS or rc	12,5	CHG	No	65	0	0
1	0	Thoracotoı	12,5	CHG	No	81	0	0
1	0	Thoracotoı	12,5	CHG	No	68	0	0
1	0	VATS or rc	12,5	CHG	No	69	0	0
1	0	VATS or rc	12,5	CHG	No	59	0	0
1	0	Thoracotoı	12,5	CHG	No	68	1	0
1	0	VATS or rc	12,5	CHG	No	62	0	0
1	0	Thoracotoı	12,5	CHG	No	59	0	0
1	1	Thoracotoı	12,5	CHG	No	62	0	0
1	0	VATS or rc	12,5	CHG	No	72	0	0
1	0	VATS or rc	12,5	CHG	No	51	0	0
1	0	Thoracotoı	12,5	CHG	No	61	0	0
1	0	Thoracotoı	12,5	CHG	No	59	0	0
1	0	VATS or rc	12,5	CHG	No	59	0	0
1	0	VATS or rc	12,5	CHG	No	74	0	0
1	0	Thoracotoı	12,5	CHG	No	66	0	0
1	0	VATS or rc	12,5	CHG	No	51	0	0
1	0	VATS or rc	12,5	CHG	No	76	1	0

1	0	VATS or rc	12,5	CHG	No	46	0	0
1	0	VATS or rc	12,5	CHG	No	57	0	0
1	0	Thoracotoı	12,5	CHG	No	63	0	0
1	0	Thoracotoı	12,5	CHG	No	65	0	0
1	0	Thoracotoı	12,5	CHG	No	52	0	0
1	0	Thoracotoı	12,5	CHG	No	70	0	0
1	0	VATS or rc	12,5	CHG	No	63	0	0
1	0	VATS or rc	12,5	CHG	No	69	0	0
1	0	Thoracotoı	12,5	CHG	No	64	1	0
1	0	VATS or rc	12,5	CHG	No	32	0	0
1	0	VATS or rc	12,5	CHG	No	74	0	0
1	0	Thoracotoı	12,5	CHG	No	67	0	0
1	0	Thoracotoı	12,5	CHG	No	71	1	0
1	0	VATS or rc	12,5	CHG	No	68	0	0
1	0	Thoracotoı	12,5	CHG	No	58	0	0
1	0	VATS or rc	12,5	CHG	No	64	0	0
1	0	Thoracotoı	12,5	CHG	No	81	0	0
1	0	VATS or rc	12,5	CHG	No	70	0	0
1	0	Thoracotoı	12,5	CHG	No	43	0	0
1	0	Thoracotoı	12,5	CHG	No	75	1	0
1	0	VATS or rc	12,5	CHG	No	64	1	0
1	0	VATS or rc	12,5	CHG	No	42	0	0
1	0	Thoracotoı	12,5	CHG	No	60	0	0
1	0	Thoracotoı	12,5	CHG	No	65	0	0
1	0	VATS or rc	12,5	CHG	No	73	0	0
1	0	Thoracotoı	12,5	CHG	No	79	0	0
1	0	VATS or rc	12,5	CHG	Yes	80	1	0
1	0	Thoracotoı	12,5	CHG	No	54	0	0
1	0	Thoracotoı	12,5	CHG	No	52	0	0
1	0	VATS or rc	12,5	CHG	No	75	0	0
1	0	Thoracotoı	12,5	CHG	No	62	0	0
1	0	VATS or rc	12,5	CHG	No	70	0	0
1	0	VATS or rc	12,5	CHG	No	47	0	0
1	0	Thoracotoı	12,5	CHG	No	63	1	0
1	0	VATS or rc	12,5	CHG	No	77	1	0
1	0	VATS or rc	12,5	CHG	No	69	0	0
1	0	Thoracotoı	12,5	CHG	No	71	0	0
1	0	VATS or rc	12,5	CHG	No	56	0	0
1	0	Thoracotoı	12,5	CHG	No	54	0	0
1	0	Thoracotoı	12,5	CHG	No	59	0	0
1	0	Thoracotoı	12,5	CHG	No	52	0	0
1	0	Thoracotoı	12,5	CHG	No	80	0	0
1	0	VATS or rc	12,5	CHG	No	64	1	0
1	0	Thoracotoı	12,5	CHG	No	61	0	0
1	0	Thoracotoı	12,5	CHG	No	64	0	0
1	0	VATS or rc	12,5	CHG	No	56	0	0
0	0	VATS or rc	46	CHU	No	55	0	0
0	0	VATS or rc	46	CHU	No	68	0	0
0	0	VATS or rc	46	CHU	No	75	0	0
0	0	VATS or rc	46	CHU	No	71	0	0
0	0	VATS or rc	46	CHU	No	60	0	0
0	0	VATS or rc	46	CHU	No	66	0	0
0	0	VATS or rc	46	CHU	No	63	0	0
0	0	Thoracotoı	46	CHU	No	50	0	0
0	0	VATS or rc	46	CHU	No	83	0	0
0	0	Thoracotoı	46	CHU	No	52	0	0

0	0	VATS or rc	46	CHU	No	60	0	0
0	0	VATS or rc	46	CHU	No	60	0	0
0	0	Thoracotoı	46	CHU	No	66	0	0
0	0	VATS or rc	46	CHU	No	47	0	0
0	0	VATS or rc	46	CHU	No	73	0	0
0	0	VATS or rc	46	CHU	No	56	0	0
0	0	VATS or rc	46	CHU	No	65	0	0
0	0	Thoracotoı	46	CHU	No	52	0	0
0	0	Thoracotoı	46	CHU	No	54	0	0
0	0	VATS or rc	46	CHU	No	60	0	0
0	0	Thoracotoı	46	CHU	No	80	0	0
0	0	Thoracotoı	46	CHU	No	54	0	0
0	1	Thoracotoı	46	CHU	No	59	0	0
0	0	VATS or rc	46	CHU	No	66	0	0
0	0	Thoracotoı	46	CHU	No	55	1	1
0	0	Thoracotoı	46	CHU	No	77	0	1
0	0	Thoracotoı	46	CHU	No	69	0	0
0	0	VATS or rc	46	CHU	No	55	0	1
0	1	VATS or rc	46	CHU	No	72	0	1
0	0	Thoracotoı	46	CHU	No	67	1	0
0	1	Thoracotoı	46	CHU	No	75	0	0
0	0	VATS or rc	46	CHU	No	67	0	0
1	0	VATS or robotic		PRIVE	No	68	0	0
1	0	Thoracotomy		PRIVE	No	46	0	0
1	0	Thoracotomy		PRIVE	No	59	0	0
1	0	VATS or robotic		PRIVE	No	68	0	0
1	0	VATS or robotic		PRIVE	No	68	0	0
1	0	Thoracotomy		PRIVE	No	58	0	0
1	1	Thoracotomy		PRIVE	No	62	0	0
1	0	Thoracotomy		PRIVE	No	71	0	0
1	0	Thoracotomy		PRIVE	No	57	0	0
1	0	Thoracotomy		PRIVE	Yes	72	0	0
1	0	Thoracotomy		PRIVE	No	64	0	0
1	0	VATS or robotic		PRIVE	No	71	0	0
1	0	Thoracotomy		PRIVE	No	64	0	0
1	0	Thoracotomy		PRIVE	No	46	0	0
1	0	Thoracotomy		PRIVE	No	61	0	0
1	0	VATS or robotic		PRIVE	No	67	0	0
1	0	Thoracotomy		PRIVE	No	60	0	0
1	0	Thoracotomy		PRIVE	No	62	0	0
1	0	VATS or robotic		PRIVE	No	70	0	0
0	0	Thoracotoı	278	CHU	No	54	0	0
0	0	Thoracotoı	278	CHU	No	75	0	0
0	0	Thoracotoı	278	CHU	No	49	0	0
0	0	Thoracotoı	278	CHU	No	36	0	0
0	0	VATS or rc	278	CHU	No	67	0	0
0	0	VATS or rc	278	CHU	No	57	0	0
0	0	Thoracotoı	278	CHU	No	62	0	0
0	0	Thoracotoı	278	CHU	No	58	0	0
0	0	VATS or rc	278	CHU	No	58	0	0
0	1	Thoracotoı	278	CHU	No	63	1	0
0	0	Thoracotoı	278	CHU	No	72	0	0
0	1	Thoracotoı	278	CHU	No	60	0	0
0	0	Thoracotoı	278	CHU	No	73	0	0
0	0	Thoracotoı	278	CHU	No	71	0	0
0	1	Thoracotoı	278	CHU	No	67	0	0

0	0	VATS or ro	278	CHU	No	76	1	0
0	0	VATS or ro	278	CHU	No	75	0	0
0	0	Thoracotor	278	CHU	No	57	0	0
0	0	Thoracotor	278	CHU	No	49	0	0
0	0	Thoracotor	278	CHU	No	69	0	0
0	0	VATS or ro	278	CHU	No	71	0	0
0	0	Thoracotor	278	CHU	No	51	0	0
0	0	VATS or ro	278	CHU	No	60	0	0
0	0	VATS or ro	278	CHU	No	68	0	0
0	0	Thoracotor	278	CHU	No	55	0	0
0	0	VATS or ro	278	CHU	No	72	1	0
0	0	Thoracotor	278	CHU	No	52	0	0
0	0	Thoracotor	278	CHU	No	76	0	0
0	1	Thoracotor	278	CHU	No	61	0	0
0	0	Thoracotor	278	CHU	No	48	0	0
0	0	VATS or ro	278	CHU	No	65	0	0
0	0	VATS or ro	278	CHU	No	61	0	0
0	0	VATS or ro	278	CHU	No	79	0	0
0	0	Thoracotor	278	CHU	No	57	0	0
0	0	Thoracotor	278	CHU	No	71	0	0
0	0	Thoracotor	278	CHU	No	68	0	0
0	0	VATS or ro	278	CHU	No	60	0	0
0	0	VATS or ro	278	CHU	No	60	1	0
0	0	Thoracotor	278	CHU	No	61	0	0
0	0	Thoracotor	278	CHU	No	56	0	0
0	0	VATS or ro	278	CHU	No	61	0	1
0	0	VATS or ro	278	CHU	No	62	0	0
0	0	Thoracotor	278	CHU	No	68	0	0
0	0	VATS or ro	278	CHU	No	57	0	0
0	0	Thoracotor	278	CHU	No	81	0	0
0	0	VATS or ro	278	CHU	No	62	0	0
0	0	VATS or ro	278	CHU	No	64	0	0
0	0	Thoracotor	278	CHU	No	58	0	0
0	0	Thoracotor	278	CHU	No	59	0	0
0	0	VATS or ro	278	CHU	No	74	0	0
0	0	VATS or ro	278	CHU	No	68	0	0
0	0	VATS or ro	278	CHU	No	61	0	0
0	0	VATS or ro	278	CHU	No	71	0	0
0	1	Thoracotor	278	CHU	No	56	0	0
0	0	VATS or ro	278	CHU	No	70	0	0
0	0	VATS or ro	278	CHU	No	59	0	0
0	0	VATS or ro	278	CHU	No	58	0	0
0	0	Thoracotor	278	CHU	No	40	0	0
0	1	Thoracotor	278	CHU	No	73	0	0
0	0	VATS or ro	278	CHU	Yes	59	0	0
0	0	VATS or ro	278	CHU	No	62	1	0
0	0	Thoracotor	278	CHU	No	65	1	0
0	0	VATS or ro	278	CHU	No	67	0	0
0	0	VATS or ro	278	CHU	No	72	0	0
0	0	VATS or ro	278	CHU	No	51	0	0
0	0	Thoracotor	278	CHU	No	75	0	0
0	0	Thoracotor	278	CHU	No	70	0	0
0	0	VATS or ro	278	CHU	No	49	0	0
0	0	Thoracotor	278	CHU	No	61	0	0
0	0	Thoracotor	278	CHU	No	62	0	0
0	0	Thoracotor	278	CHU	No	45	0	0

0	0	VATS or rc	278	CHU	No	61	0	0
0	0	Thoracotoı	278	CHU	No	61	0	0
0	1	Thoracotoı	278	CHU	No	38	0	0
0	1	Thoracotoı	278	CHU	No	73	1	0
0	0	VATS or rc	278	CHU	No	71	0	0
0	0	VATS or rc	278	CHU	No	76	0	0
0	0	VATS or rc	278	CHU	No	52	0	0
0	0	VATS or rc	278	CHU	No	71	0	0
0	0	Thoracotoı	278	CHU	No	63	0	0
0	0	Thoracotoı	278	CHU	No	46	0	0
0	0	VATS or rc	278	CHU	No	72	1	0
0	0	Thoracotoı	278	CHU	No	59	0	0
0	0	Thoracotoı	278	CHU	No	72	0	0
0	0	VATS or rc	278	CHU	No	73	0	1
0	0	VATS or rc	278	CHU	No	55	0	0
0	0	VATS or rc	278	CHU	No	66	0	0
0	0	Thoracotoı	278	CHU	No	69	0	0
0	0	Thoracotoı	278	CHU	No	75	0	0
0	0	Thoracotoı	278	CHU	No	59	0	1
0	0	Thoracotoı	278	CHU	No	79	0	0
0	0	VATS or rc	278	CHU	No	71	0	0
0	0	VATS or rc	278	CHU	No	64	0	0
0	0	Thoracotoı	278	CHU	No	76	0	0
0	0	VATS or rc	278	CHU	No	55	0	0
0	0	VATS or rc	278	CHU	No	63	0	0
0	0	Thoracotoı	278	CHU	No	73	0	0
0	0	Thoracotoı	278	CHU	No	75	0	0
0	0	Thoracotoı	278	CHU	No	56	0	0
0	0	VATS or rc	278	CHU	No	82	0	0
0	0	Thoracotoı	278	CHU	No	51	0	0
0	0	Thoracotoı	278	CHU	No	80	1	0
0	0	Thoracotoı	278	CHU	Yes	61	0	0
0	0	VATS or rc	278	CHU	No	69	0	0
0	0	VATS or rc	278	CHU	No	56	0	0
0	0	VATS or rc	278	CHU	No	86	0	0
0	0	Thoracotoı	278	CHU	No	55	0	0
0	0	Thoracotoı	278	CHU	No	73	0	0
0	0	VATS or rc	278	CHU	No	69	0	0
0	0	Thoracotoı	278	CHU	No	52	1	0
0	0	Thoracotoı	278	CHU	No	63	0	0
0	0	VATS or rc	278	CHU	No	76	0	0
0	0	Thoracotoı	278	CHU	No	61	0	0
0	0	Thoracotoı	278	CHU	No	75	0	0
0	0	Thoracotoı	278	CHU	No	81	1	0
0	0	VATS or rc	278	CHU	No	71	1	0
0	0	VATS or rc	278	CHU	No	59	0	0
0	0	Thoracotoı	278	CHU	No	63	0	0
0	0	Thoracotoı	278	CHU	No	73	0	0
0	0	VATS or rc	278	CHU	No	76	0	0
0	0	VATS or rc	278	CHU	No	66	0	1
0	0	VATS or rc	278	CHU	No	69	0	0
0	0	Thoracotoı	278	CHU	No	79	0	0
0	0	Thoracotoı	278	CHU	No	81	0	0
0	0	Thoracotoı	278	CHU	No	61	0	0
0	0	VATS or rc	278	CHU	No	60	0	0
0	0	Thoracotoı	278	CHU	No	68	0	0

0	0	Thoracotoı	278	CHU	No	67	0	0
0	1	Thoracotoı	278	CHU	No	75	0	0
0	0	Thoracotoı	278	CHU	No	49	0	0
0	0	Thoracotoı	278	CHU	No	66	0	0
0	0	VATS or rc	278	CHU	No	63	1	0
0	0	VATS or rc	278	CHU	No	76	0	0
0	0	VATS or rc	278	CHU	No	72	1	0
0	0	Thoracotoı	278	CHU	No	61	0	0
0	0	VATS or rc	278	CHU	No	64	0	1
0	0	Thoracotoı	278	CHU	No	66	0	0
0	0	VATS or rc	278	CHU	No	71	0	0
0	0	Thoracotoı	278	CHU	No	65	0	0
0	0	Thoracotoı	278	CHU	No	62	1	0
0	0	Thoracotoı	278	CHU	No	50	0	0
0	0	VATS or rc	278	CHU	No	62	0	0
0	0	VATS or rc	278	CHU	No	64	1	0
0	0	Thoracotoı	278	CHU	No	68	0	0
0	0	Thoracotoı	278	CHU	No	51	1	1
0	0	VATS or rc	278	CHU	No	52	0	0
0	0	Thoracotoı	278	CHU	No	54	0	0
0	0	Thoracotoı	278	CHU	No	75	1	0
0	0	Thoracotoı	278	CHU	No	60	0	0
0	0	Thoracotoı	278	CHU	No	64	0	0
0	0	VATS or rc	278	CHU	No	45	0	0
0	0	VATS or rc	278	CHU	No	68	1	0
0	0	VATS or rc	278	CHU	No	62	0	1
0	0	Thoracotoı	278	CHU	No	72	0	0
0	0	Thoracotoı	278	CHU	No	60	0	0
0	0	Thoracotoı	278	CHU	No	68	0	0
0	0	VATS or rc	278	CHU	No	60	0	0
0	0	Thoracotoı	278	CHU	No	55	0	0
0	0	Thoracotoı	278	CHU	No	66	1	0
0	1	Thoracotoı	278	CHU	Yes	64	0	0
0	0	Thoracotoı	278	CHU	No	60	1	0
0	0	Thoracotoı	278	CHU	No	51	0	1
0	0	VATS or rc	278	CHU	No	61	0	0
1	0	Thoracotoı	147	CHU	No	73	0	0
1	0	VATS or rc	147	CHU	No	67	0	0
1	0	Thoracotoı	147	CHU	No	73	0	0
1	0	VATS or rc	147	CHU	No	50	0	0
1	0	VATS or rc	147	CHU	No	72	0	0
1	0	Thoracotoı	147	CHU	No	68	0	0
1	0	Thoracotoı	147	CHU	No	59	0	0
1	0	Thoracotoı	147	CHU	No	54	0	0
1	0	Thoracotoı	147	CHU	No	65	0	0
1	0	Thoracotoı	147	CHU	No	76	0	0
1	0	Thoracotoı	147	CHU	No	64	0	1
1	0	Thoracotoı	147	CHU	No	61	1	0
1	0	Thoracotoı	147	CHU	Yes	65	0	0
1	0	Thoracotoı	147	CHU	No	66	0	0
1	0	Thoracotoı	147	CHU	No	62	1	0
1	0	Thoracotoı	147	CHU	No	54	0	0
1	0	Thoracotoı	147	CHU	No	71	0	1
1	1	Thoracotoı	147	CHU	No	42	0	0
1	0	Thoracotoı	147	CHU	No	64	0	0
1	0	Thoracotoı	147	CHU	No	67	0	0

1	0	VATS or ro	147	CHU	No	64	0	1
1	0	VATS or ro	147	CHU	No	73	0	0
1	0	Thoracotor	147	CHU	No	61	1	0
1	0	Thoracotor	147	CHU	No	69	0	0
1	0	Thoracotor	147	CHU	No	79	1	0
1	0	Thoracotor	147	CHU	No	55	0	0
1	0	VATS or ro	147	CHU	No	68	0	0
1	0	VATS or ro	147	CHU	No	55	0	0
1	0	Thoracotor	147	CHU	No	61	0	0
1	0	VATS or ro	147	CHU	No	70	0	0
1	0	Thoracotor	147	CHU	No	68	0	0
1	0	Thoracotor	147	CHU	No	77	0	0
1	0	Thoracotor	147	CHU	No	81	1	0
1	0	Thoracotor	147	CHU	No	70	0	0
1	0	Thoracotor	147	CHU	No	53	0	0
1	1	Thoracotor	147	CHU	No	35	0	0
1	0	Thoracotor	147	CHU	No	58	0	0
1	0	Thoracotor	147	CHU	No	56	1	0
1	0	Thoracotor	147	CHU	No	71	0	0
1	0	Thoracotor	147	CHU	No	68	0	0
1	0	VATS or ro	147	CHU	No	61	0	0
1	0	Thoracotor	147	CHU	No	57	1	0
1	0	Thoracotor	147	CHU	No	54	0	0
1	0	Thoracotor	147	CHU	No	60	0	0
1	0	Thoracotor	147	CHU	No	85	1	0
1	0	Thoracotor	147	CHU	No	51	0	0
1	0	Thoracotor	147	CHU	No	76	0	0
1	0	VATS or ro	147	CHU	No	73	0	0
1	0	Thoracotor	147	CHU	No	70	0	0
1	0	VATS or ro	147	CHU	No	69	1	0
1	0	Thoracotor	147	CHU	No	59	0	0
1	0	Thoracotor	147	CHU	No	73	0	0
1	0	Thoracotor	147	CHU	No	68	0	0
1	0	Thoracotor	147	CHU	No	59	0	0
1	0	VATS or ro	147	CHU	No	62	0	0
1	0	Thoracotor	147	CHU	No	43	0	0
1	0	Thoracotor	147	CHU	No	43	0	0
1	0	Thoracotor	147	CHU	No	73	1	0
1	0	Thoracotor	147	CHU	No	55	0	0
1	0	Thoracotor	147	CHU	No	62	0	0
1	0	Thoracotor	147	CHU	No	64	1	0
1	0	Thoracotor	147	CHU	No	72	0	0
1	1	Thoracotor	147	CHU	No	59	0	0
1	0	Thoracotor	147	CHU	No	57	0	0
1	0	Thoracotor	147	CHU	No	66	0	0
1	0	Thoracotor	147	CHU	No	59	0	0
1	0	Thoracotor	147	CHU	No	72	0	0
1	0	Thoracotor	147	CHU	No	62	0	1
1	1	Thoracotor	147	CHU	Yes	76	0	0
1	0	VATS or ro	147	CHU	No	73	0	0
1	0	Thoracotor	147	CHU	No	68	0	0
1	0	Thoracotor	147	CHU	No	61	0	0
1	0	Thoracotor	147	CHU	No	66	0	0
1	0	VATS or ro	147	CHU	No	63	1	0
1	0	Thoracotor	147	CHU	No	67	1	0
1	0	Thoracotor	147	CHU	No	72	1	0

1	0	Thoracotoı	147	CHU	No	62	0	0
1	0	Thoracotoı	147	CHU	No	62	0	0
1	0	Thoracotoı	147	CHU	No	59	0	0
1	0	Thoracotoı	147	CHU	No	61	0	0
1	0	VATS or rc	147	CHU	No	60	0	1
1	0	Thoracotoı	147	CHU	No	59	0	0
1	0	Thoracotoı	147	CHU	No	71	0	0
1	0	Thoracotoı	147	CHU	No	76	1	0
1	1	Thoracotoı	147	CHU	No	79	1	0
1	1	Thoracotoı	147	CHU	No	69	0	0
1	0	Thoracotoı	147	CHU	No	62	0	0
1	0	Thoracotoı	147	CHU	No	66	1	0
1	0	Thoracotoı	147	CHU	No	63	0	0
1	0	VATS or rc	147	CHU	No	51	0	0
1	0	VATS or rc	147	CHU	No	66	0	0
1	0	Thoracotoı	147	CHU	No	69	1	0
1	0	Thoracotoı	147	CHU	No	59	0	0
1	0	Thoracotoı	147	CHU	No	77	0	0
1	0	Thoracotoı	147	CHU	No	63	0	0
1	0	Thoracotoı	147	CHU	No	61	0	0
1	0	Thoracotoı	147	CHU	No	72	0	0
1	0	VATS or rc	147	CHU	No	54	0	0
1	0	Thoracotoı	147	CHU	No	68	1	0
1	0	Thoracotoı	147	CHU	No	60	0	0
1	0	Thoracotoı	147	CHU	No	79	0	0
1	0	Thoracotoı	147	CHU	No	69	1	1
1	0	Thoracotoı	147	CHU	No	54	0	1
1	0	Thoracotoı	147	CHU	No	69	1	0
1	0	Thoracotoı	147	CHU	No	66	0	0
1	0	VATS or rc	147	CHU	No	73	0	0
1	0	Thoracotoı	147	CHU	No	58	0	1
1	0	VATS or rc	147	CHU	No	67	0	0
1	0	VATS or rc	147	CHU	No	61	0	0
1	0	VATS or rc	147	CHU	No	65	0	0
1	0	Thoracotoı	147	CHU	No	63	0	0
1	0	VATS or rc	147	CHU	No	78	0	0
1	0	Thoracotoı	147	CHU	No	59	0	0
1	0	Thoracotoı	147	CHU	No	63	0	0
1	0	VATS or rc	147	CHU	No	63	0	0
1	0	Thoracotoı	147	CHU	No	60	0	0
1	0	Thoracotoı	147	CHU	No	75	0	0
1	0	Thoracotoı	147	CHU	No	54	0	1
1	0	Thoracotoı	147	CHU	No	70	0	0
1	0	Thoracotoı	147	CHU	No	54	0	0
1	0	VATS or rc	147	CHU	No	52	0	0
1	0	VATS or rc	147	CHU	No	65	0	0
1	0	Thoracotoı	147	CHU	No	53	0	0
1	0	Thoracotoı	147	CHU	No	67	1	0
1	1	Thoracotoı	147	CHU	No	70	0	0
1	0	Thoracotoı	147	CHU	No	30	0	0
1	1	Thoracotoı	147	CHU	No	77	0	0
1	0	Thoracotoı	147	CHU	No	57	0	0
1	0	Thoracotoı	147	CHU	No	70	0	0
1	0	VATS or rc	147	CHU	No	61	0	0
1	0	Thoracotoı	147	CHU	No	59	0	0
1	0	Thoracotoı	147	CHU	No	55	0	0

1	0	Thoracotoı	147	CHU	No	68	0	0
1	0	Thoracotoı	147	CHU	No	35	1	0
1	0	Thoracotoı	147	CHU	No	62	0	0
1	0	Thoracotoı	147	CHU	No	66	0	0
1	0	Thoracotoı	147	CHU	No	79	0	0
1	0	Thoracotoı	147	CHU	No	78	0	0
1	0	VATS or rc	147	CHU	No	84	0	0
1	0	Thoracotoı	147	CHU	No	36	0	0
1	0	Thoracotoı	147	CHU	No	67	0	0
1	0	Thoracotoı	147	CHU	No	75	0	0
1	0	Thoracotoı	147	CHU	No	85	0	0
1	0	Thoracotoı	147	CHU	No	64	0	0
1	0	VATS or rc	147	CHU	No	77	0	0
1	0	VATS or rc	147	CHU	No	74	0	0
1	0	VATS or rc	147	CHU	No	74	0	0
1	0	Thoracotoı	147	CHU	No	67	0	1
1	0	Thoracotoı	147	CHU	No	81	0	0
1	0	Thoracotoı	147	CHU	No	66	0	0
1	0	VATS or rc	147	CHU	No	66	0	0
1	0	VATS or rc	147	CHU	No	80	0	0
1	0	VATS or rc	147	CHU	No	62	0	0
1	1	Thoracotoı	147	CHU	No	74	0	0
1	1	Thoracotoı	147	CHU	No	52	0	0
1	0	Thoracotoı	147	CHU	No	35	1	0
1	0	Thoracotoı	147	CHU	No	58	0	0
1	0	Thoracotoı	147	CHU	No	79	0	0
1	0	VATS or rc	147	CHU	No	72	0	0
1	0	Thoracotoı	147	CHU	Yes	62	0	1
1	0	VATS or rc	147	CHU	No	68	1	0
1	0	Thoracotoı	147	CHU	No	69	0	0
1	0	Thoracotoı	147	CHU	No	71	0	0
1	0	VATS or rc	147	CHU	No	44	0	0
1	0	VATS or rc	147	CHU	No	68	0	1
1	0	Thoracotoı	147	CHU	No	69	1	0
1	0	Thoracotoı	147	CHU	No	75	0	0
1	0	VATS or rc	147	CHU	No	59	0	0
1	0	Thoracotoı	147	CHU	No	64	0	0
1	0	VATS or rc	147	CHU	No	72	0	0
1	0	Thoracotoı	147	CHU	No	59	0	0
1	0	Thoracotoı	147	CHU	No	64	0	0
1	0	Thoracotoı	147	CHU	No	68	0	0
1	0	Thoracotoı	147	CHU	No	72	0	0
1	0	VATS or rc	147	CHU	No	55	0	0
1	0	Thoracotoı	147	CHU	No	69	0	0
1	0	Thoracotoı	147	CHU	No	66	1	0
1	0	Thoracotoı	147	CHU	No	67	0	0
1	0	VATS or rc	147	CHU	No	62	0	0
1	0	Thoracotoı	147	CHU	No	64	1	0
1	0	Thoracotoı	147	CHU	No	55	1	1
1	0	VATS or rc	147	CHU	No	58	0	0
1	0	Thoracotoı	147	CHU	No	63	0	0
1	0	Thoracotoı	147	CHU	Yes	79	0	0
1	0	Thoracotoı	147	CHU	No	52	0	0
1	1	Thoracotoı	147	CHU	No	53	0	0
1	0	Thoracotoı	147	CHU	No	54	0	0
1	0	Thoracotoı	147	CHU	No	65	0	1

1	0	Thoracotoı	147	CHU	No	76	0	0
1	0	Thoracotoı	147	CHU	No	52	0	1
1	1	Thoracotoı	147	CHU	No	64	0	0
1	0	Thoracotoı	147	CHU	No	69	1	0
1	0	VATS or rc	147	CHU	No	52	0	0
1	0	VATS or rc	147	CHU	No	55	0	0
1	0	Thoracotoı	147	CHU	No	47	0	0
1	0	Thoracotoı	147	CHU	No	56	0	0
1	0	Thoracotoı	147	CHU	No	61	0	0
1	0	Thoracotoı	147	CHU	No	59	1	0
1	0	Thoracotoı	147	CHU	No	60	0	0
1	0	Thoracotoı	147	CHU	No	66	0	0
1	0	VATS or rc	147	CHU	No	47	0	0
1	0	Thoracotoı	147	CHU	No	66	0	0
1	0	Thoracotoı	147	CHU	No	69	1	0
1	0	Thoracotoı	147	CHU	No	57	0	0
1	0	Thoracotoı	147	CHU	No	64	0	0
1	0	Thoracotoı	147	CHU	No	67	0	0
1	0	VATS or rc	147	CHU	No	57	0	0
1	0	VATS or rc	147	CHU	No	75	0	0
1	0	Thoracotoı	147	CHU	No	62	0	0
1	0	Thoracotoı	147	CHU	No	72	0	0
1	1	Thoracotoı	147	CHU	No	60	0	0
1	0	Thoracotoı	147	CHU	No	63	0	0
1	0	Thoracotoı	147	CHU	No	60	0	0
1	0	VATS or rc	147	CHU	No	62	0	0
1	0	Thoracotoı	147	CHU	No	69	0	0
1	0	Thoracotoı	147	CHU	No	65	0	0
1	0	VATS or rc	147	CHU	No	77	0	0
1	0	VATS or rc	147	CHU	No	53	0	0
1	1	Thoracotoı	147	CHU	No	71	0	0
1	0	VATS or rc	147	CHU	No	55	0	0
1	0	VATS or rc	147	CHU	No	63	0	0
1	0	VATS or rc	147	CHU	No	71	0	1
1	0	Thoracotoı	147	CHU	No	72	1	0
1	0	Thoracotoı	147	CHU	No	67	1	0
1	0	VATS or rc	147	CHU	No	63	0	0
1	0	VATS or rc	147	CHU	No	51	0	0
1	0	Thoracotoı	147	CHU	No	52	0	0
1	0	Thoracotoı	147	CHU	No	69	0	0
1	0	VATS or rc	147	CHU	No	63	0	0
1	0	VATS or rc	147	CHU	No	72	1	0
1	0	Thoracotoı	147	CHU	No	75	0	0
1	0	VATS or rc	147	CHU	No	74	0	0
1	0	VATS or rc	147	CHU	No	54	0	0
1	0	VATS or rc	147	CHU	No	82	0	0
1	0	Thoracotoı	147	CHU	No	69	1	0
1	0	Thoracotoı	147	CHU	No	66	0	0
1	0	VATS or rc	147	CHU	No	71	0	0
1	0	VATS or rc	147	CHU	No	61	0	0
1	0	Thoracotoı	147	CHU	No	67	0	0
1	0	Thoracotoı	147	CHU	No	66	0	0
1	0	Thoracotoı	147	CHU	No	69	0	0
1	0	Thoracotoı	147	CHU	No	56	0	0
1	0	Thoracotoı	147	CHU	No	61	0	0
1	0	Thoracotoı	147	CHU	No	56	1	0

1	0	Thoracotoı	147	CHU	No	84	0	0
1	0	Thoracotoı	147	CHU	No	69	0	0
1	0	Thoracotoı	147	CHU	No	66	0	0
1	0	Thoracotoı	147	CHU	No	56	1	0
1	1	Thoracotoı	147	CHU	No	54	0	0
1	0	Thoracotoı	147	CHU	No	74	0	0
1	0	VATS or rc	147	CHU	No	55	0	1
1	0	Thoracotoı	147	CHU	No	38	0	0
1	0	Thoracotoı	147	CHU	No	64	0	1
1	0	Thoracotoı	147	CHU	No	83	0	0
1	0	Thoracotoı	147	CHU	No	49	0	0
1	0	VATS or rc	147	CHU	No	64	0	0
1	0	Thoracotoı	147	CHU	No	56	0	0
1	0	Thoracotoı	147	CHU	No	55	0	0
1	0	Thoracotoı	147	CHU	No	58	0	0
1	0	Thoracotoı	147	CHU	No	66	0	0
1	0	Thoracotoı	147	CHU	No	57	0	0
1	0	Thoracotoı	147	CHU	No	74	1	0
1	0	Thoracotoı	147	CHU	No	59	0	0
1	0	VATS or rc	147	CHU	No	72	0	1
1	0	Thoracotoı	147	CHU	No	68	0	0
1	0	VATS or rc	147	CHU	No	76	1	0
1	0	Thoracotoı	147	CHU	No	53	0	0
1	0	Thoracotoı	147	CHU	No	79	0	0
1	0	Thoracotoı	147	CHU	No	78	0	0
1	0	Thoracotoı	147	CHU	No	45	0	0
1	0	Thoracotoı	147	CHU	Yes	59	0	0
1	0	VATS or rc	147	CHU	No	64	0	0
1	0	Thoracotoı	147	CHU	No	83	0	0
1	0	VATS or rc	147	CHU	No	82	0	0
1	0	VATS or rc	147	CHU	No	76	0	0
1	0	VATS or rc	147	CHU	No	61	1	0
1	0	Thoracotoı	147	CHU	No	47	0	0
1	0	Thoracotoı	147	CHU	No	47	0	0
1	0	Thoracotoı	147	CHU	No	61	0	0
1	0	Thoracotoı	147	CHU	No	65	0	0
1	0	Thoracotoı	147	CHU	No	58	0	1
1	0	Thoracotoı	147	CHU	No	70	1	0
1	0	Thoracotoı	147	CHU	No	67	0	0
1	0	VATS or rc	147	CHU	No	71	0	0
1	0	VATS or rc	147	CHU	No	64	0	0
1	0	VATS or rc	147	CHU	No	62	0	0
1	0	VATS or rc	147	CHU	No	60	0	0
1	0	Thoracotoı	147	CHU	No	64	0	0
1	0	Thoracotoı	147	CHU	No	71	0	0
1	0	VATS or rc	147	CHU	No	62	1	0
1	0	Thoracotoı	147	CHU	No	52	0	0
1	0	Thoracotoı	147	CHU	No	65	0	0
1	0	Thoracotoı	147	CHU	No	79	0	0
1	0	VATS or rc	147	CHU	No	55	0	0
1	0	Thoracotoı	147	CHU	No	77	1	0
1	0	VATS or rc	147	CHU	No	56	0	1
1	0	Thoracotoı	147	CHU	No	56	0	0
1	0	Thoracotoı	147	CHU	No	72	0	0
1	0	Thoracotoı	147	CHU	No	75	0	0
1	0	Thoracotoı	147	CHU	No	61	0	0

1	0	Thoracotoı	147	CHU	No	66	0	0
1	0	Thoracotoı	147	CHU	No	76	1	0
1	0	Thoracotoı	147	CHU	No	52	0	0
1	0	Thoracotoı	147	CHU	Yes	65	0	0
1	0	Thoracotoı	147	CHU	No	58	0	0
1	0	Thoracotoı	147	CHU	No	60	1	0
1	1	Thoracotoı	147	CHU	No	74	1	0
1	0	Thoracotoı	147	CHU	No	69	0	0
1	0	Thoracotoı	147	CHU	No	50	0	0
1	0	Thoracotoı	147	CHU	No	68	0	0
1	0	Thoracotoı	147	CHU	No	75	0	0
1	0	Thoracotoı	147	CHU	No	49	0	0
1	0	Thoracotoı	147	CHU	No	73	0	0
1	0	Thoracotoı	147	CHU	No	65	0	0
1	0	Thoracotoı	147	CHU	No	60	0	0
1	0	Thoracotoı	147	CHU	No	59	0	0
1	0	Thoracotoı	147	CHU	No	58	1	0
1	1	Thoracotoı	147	CHU	No	63	0	0
1	1	Thoracotoı	147	CHU	Yes	56	0	0
1	0	Thoracotoı	147	CHU	No	58	0	0
1	0	Thoracotoı	147	CHU	No	67	0	0
1	0	Thoracotoı	147	CHU	No	69	0	0
1	0	Thoracotoı	147	CHU	No	61	0	0
1	0	VATS or ro	147	CHU	No	64	0	0
1	0	VATS or ro	147	CHU	No	53	0	0
1	0	Thoracotoı	147	CHU	No	71	0	0
1	0	Thoracotoı	147	CHU	No	67	0	0
1	0	Thoracotoı	147	CHU	No	56	0	0
1	0	Thoracotoı	147	CHU	No	79	0	0
1	0	Thoracotoı	147	CHU	No	72	0	0
1	0	Thoracotoı	147	CHU	No	63	0	0
1	0	Thoracotoı	147	CHU	No	65	0	0
1	0	Thoracotoı	147	CHU	No	71	0	0
1	0	Thoracotoı	147	CHU	No	60	0	0
1	0	Thoracotoı	147	CHU	No	55	1	0
1	0	Thoracotoı	147	CHU	No	69	0	0
1	0	Thoracotoı	147	CHU	No	77	0	0
1	1	Thoracotoı	147	CHU	No	70	0	0
1	0	Thoracotoı	147	CHU	No	72	0	0
1	0	Thoracotoı	147	CHU	No	69	0	0
1	0	Thoracotoı	147	CHU	No	79	0	0
1	0	Thoracotoı	147	CHU	No	49	0	0
1	0	VATS or ro	147	CHU	No	64	0	0
1	0	Thoracotoı	147	CHU	No	42	0	1
1	0	Thoracotoı	147	CHU	No	62	0	0
1	0	Thoracotoı	147	CHU	No	72	0	0
1	0	Thoracotoı	147	CHU	No	52	0	0
1	0	Thoracotoı	147	CHU	No	67	0	0
0	0	VATS or ro	2	PRIVE	No	69	1	0
0	0	VATS or ro	2	PRIVE	No	52	0	1
0	0	Thoracotoı	2	PRIVE	No	78	0	0
0	0	Thoracotoı	2	PRIVE	No	78	0	0
0	0	VATS or ro	2	PRIVE	No	65	0	0
0	0	VATS or ro	2	PRIVE	No	54	0	1
0	0	VATS or ro	2	PRIVE	No	49	0	1
0	0	VATS or ro	2	PRIVE	No	74	0	0

0	0	VATS or ro	2	PRIVE	No	70	0	0
0	0	VATS or ro	2	PRIVE	No	68	0	1
0	0	VATS or ro	2	PRIVE	No	75	0	0
0	0	VATS or ro	2	PRIVE	No	71	0	0
0	0	VATS or ro	2	PRIVE	No	73	0	0
0	0	VATS or ro	2	PRIVE	No	70	0	0
0	1	Thoracotor	2	PRIVE	No	76	0	0
0	0	VATS or ro	2	PRIVE	No	77	0	0
0	0	VATS or ro	2	PRIVE	No	54	0	1
0	0	VATS or ro	2	PRIVE	No	66	1	1
0	0	VATS or ro	2	PRIVE	No	63	0	1
0	0	VATS or ro	2	PRIVE	No	65	0	0
0	0	VATS or ro	2	PRIVE	No	75	1	0
0	0	VATS or ro	2	PRIVE	No	69	0	0
0	0	VATS or ro	2	PRIVE	No	62	0	0
0	0	Thoracotor	2	PRIVE	No	61	0	1
0	1	VATS or ro	2	PRIVE	No	59	0	0
0	0	Thoracotor	2	PRIVE	No	69	0	0
0	0	Thoracotor	2	PRIVE	No	74	1	0
0	0	VATS or ro	2	PRIVE	No	65	0	0
0	0	Thoracotor	2	PRIVE	No	70	0	0
0	0	VATS or ro	2	PRIVE	No	69	0	1
0	0	Thoracotor	2	PRIVE	No	49	0	0
0	0	VATS or ro	2	PRIVE	No	64	0	1
0	0	VATS or ro	2	PRIVE	No	53	0	0
0	0	Thoracotor	2	PRIVE	No	68	0	0
0	0	VATS or ro	2	PRIVE	No	76	0	1
0	0	VATS or ro	2	PRIVE	No	61	0	0
0	0	VATS or ro	2	PRIVE	No	66	0	0
0	0	VATS or ro	2	PRIVE	No	68	0	0
0	0	VATS or ro	2	PRIVE	No	57	0	0
0	0	VATS or ro	2	PRIVE	No	66	0	0
0	0	VATS or ro	2	PRIVE	No	64	0	1
0	0	VATS or ro	2	PRIVE	No	61	0	1
0	0	VATS or ro	2	PRIVE	No	56	0	0
0	0	VATS or ro	2	PRIVE	No	65	0	1
0	0	VATS or ro	2	PRIVE	No	60	1	1
0	1	VATS or ro	2	PRIVE	No	62	0	0
0	0	VATS or ro	2	PRIVE	No	72	0	0
0	1	Thoracotor	2	PRIVE	Yes	72	0	0
0	0	VATS or ro	2	PRIVE	No	72	0	0
0	0	Thoracotor	2	PRIVE	No	58	0	0
0	0	VATS or ro	2	PRIVE	No	44	0	1
0	0	VATS or ro	2	PRIVE	No	66	1	0
0	0	VATS or ro	2	PRIVE	No	78	0	0
0	0	VATS or ro	2	PRIVE	No	72	0	0
0	0	Thoracotor	2	PRIVE	No	68	0	0
0	0	VATS or ro	2	PRIVE	No	54	0	0
0	0	VATS or ro	2	PRIVE	No	69	0	0
0	0	VATS or ro	2	PRIVE	No	61	1	0
0	0	VATS or ro	2	PRIVE	No	88	0	0
0	0	VATS or ro	2	PRIVE	No	50	0	0
0	0	VATS or ro	2	PRIVE	No	65	0	0
0	0	VATS or ro	2	PRIVE	No	84	0	0
0	0	VATS or ro	2	PRIVE	No	61	1	1
0	0	VATS or ro	2	PRIVE	No	60	0	1

0	0	VATS or rc	2	PRIVE	No	74	0	0
0	0	VATS or rc	2	PRIVE	No	66	0	0
0	0	VATS or rc	2	PRIVE	No	34	0	1
0	0	VATS or rc	2	PRIVE	No	66	0	0
0	0	VATS or rc	2	PRIVE	No	73	0	0
0	0	VATS or rc	2	PRIVE	No	60	0	0
0	0	VATS or rc	2	PRIVE	No	50	0	0
0	0	VATS or rc	2	PRIVE	No	57	0	0
0	0	Thoracotor	2	PRIVE	No	57	0	0
0	0	Thoracotor	2	PRIVE	No	44	0	1
0	0	VATS or rc	2	PRIVE	No	70	0	1
0	0	VATS or rc	2	PRIVE	Yes	69	1	1
0	0	VATS or rc	2	PRIVE	No	77	1	0
0	0	VATS or rc	2	PRIVE	No	64	0	1
0	0	VATS or rc	2	PRIVE	No	59	0	1
0	0	VATS or rc	2	PRIVE	No	67	0	0
0	0	VATS or rc	2	PRIVE	No	65	0	1
0	0	VATS or rc	2	PRIVE	No	62	0	0
0	0	VATS or rc	2	PRIVE	No	67	0	0
0	0	VATS or rc	2	PRIVE	No	68	0	0
0	0	Thoracotor	2	PRIVE	No	71	0	0
0	0	VATS or rc	2	PRIVE	No	54	0	0
0	0	VATS or rc	2	PRIVE	No	68	0	0
0	0	VATS or rc	2	PRIVE	No	76	0	0
0	0	VATS or rc	2	PRIVE	No	81	0	0
0	0	VATS or rc	2	PRIVE	No	74	0	0
0	0	Thoracotor	2	PRIVE	No	80	0	0
0	0	VATS or rc	2	PRIVE	No	54	0	0
0	0	VATS or rc	2	PRIVE	No	75	1	0
0	0	Thoracotor	2	PRIVE	No	68	0	0
0	0	VATS or rc	2	PRIVE	No	78	0	0
0	0	VATS or rc	2	PRIVE	No	64	0	0
0	0	VATS or rc	2	PRIVE	No	68	1	1
0	0	VATS or rc	2	PRIVE	No	68	1	0
0	0	Thoracotor	2	PRIVE	No	72	0	1
0	0	VATS or rc	2	PRIVE	No	72	0	0
0	0	VATS or rc	2	PRIVE	No	73	0	1
0	0	VATS or rc	2	PRIVE	No	67	0	0
0	0	VATS or rc	2	PRIVE	No	69	0	0
0	0	VATS or rc	2	PRIVE	No	57	0	0
0	0	VATS or rc	2	PRIVE	No	73	0	0
0	0	VATS or rc	2	PRIVE	No	69	0	0
0	0	VATS or rc	2	PRIVE	No	69	0	0
0	0	VATS or rc	2	PRIVE	No	82	0	0
0	0	VATS or rc	2	PRIVE	No	66	0	0
0	0	VATS or rc	2	PRIVE	No	83	0	0
0	0	VATS or rc	2	PRIVE	No	76	0	0
0	0	VATS or rc	2	PRIVE	No	77	0	0
0	0	VATS or rc	2	PRIVE	No	68	0	1
0	0	VATS or rc	2	PRIVE	No	54	0	0
0	0	VATS or rc	2	PRIVE	No	71	1	1
0	0	VATS or rc	2	PRIVE	No	73	0	1
0	0	VATS or rc	2	PRIVE	No	51	0	1
0	0	Thoracotor	2	PRIVE	No	52	0	0
0	0	Thoracotor	2	PRIVE	No	54	0	1
0	0	VATS or rc	2	PRIVE	No	61	1	0

0	0	VATS or rc	2	PRIVE	No	76	0	1
0	0	Thoracotoı	2	PRIVE	No	52	0	0
0	0	VATS or rc	2	PRIVE	No	67	0	0
0	0	VATS or rc	2	PRIVE	No	75	0	0
0	0	VATS or rc	2	PRIVE	No	66	0	0
0	0	VATS or rc	2	PRIVE	No	73	0	1
0	0	VATS or rc	2	PRIVE	No	62	0	0
0	0	VATS or rc	2	PRIVE	No	59	0	0
0	0	VATS or rc	2	PRIVE	No	74	0	0
0	0	VATS or rc	2	PRIVE	No	66	0	0
0	0	VATS or rc	2	PRIVE	No	68	1	0
0	0	VATS or rc	2	PRIVE	No	76	0	1
0	0	VATS or rc	2	PRIVE	No	67	0	0
0	0	VATS or rc	2	PRIVE	No	69	1	0
0	0	VATS or rc	2	PRIVE	No	69	0	0
0	0	VATS or rc	2	PRIVE	No	82	0	0
0	0	VATS or rc	2	PRIVE	No	68	0	0
0	0	VATS or rc	2	PRIVE	No	82	1	0
0	0	VATS or rc	2	PRIVE	No	75	0	1
0	0	VATS or rc	2	PRIVE	No	75	0	0
0	0	VATS or rc	2	PRIVE	No	60	0	0
0	0	VATS or rc	2	PRIVE	No	64	0	1
0	0	Thoracotoı	2	PRIVE	No	60	0	0
0	0	VATS or rc	2	PRIVE	No	57	0	1
0	0	VATS or rc	2	PRIVE	No	52	0	1
0	0	VATS or rc	2	PRIVE	No	70	0	0
0	0	VATS or rc	2	PRIVE	No	79	0	0
0	0	VATS or rc	2	PRIVE	No	76	0	0
0	0	VATS or rc	2	PRIVE	No	59	0	0
0	0	Thoracotoı	2	PRIVE	No	60	0	0
0	0	VATS or rc	2	PRIVE	No	63	0	1
0	0	VATS or rc	2	PRIVE	No	46	0	0
0	0	VATS or rc	2	PRIVE	No	70	0	0
0	0	Thoracotoı	2	PRIVE	No	48	0	1
0	0	VATS or rc	2	PRIVE	No	68	0	0
0	0	VATS or rc	2	PRIVE	No	56	1	1
0	0	VATS or rc	2	PRIVE	No	75	0	0
0	0	VATS or rc	2	PRIVE	No	88	1	0
0	0	VATS or rc	2	PRIVE	No	81	1	0
0	0	VATS or rc	2	PRIVE	No	75	1	0
0	0	VATS or rc	2	PRIVE	No	67	0	0
0	0	VATS or rc	2	PRIVE	No	67	0	0
0	0	VATS or rc	2	PRIVE	No	80	0	0
0	0	VATS or rc	2	PRIVE	No	74	1	0
0	0	VATS or rc	2	PRIVE	No	56	0	0
0	0	VATS or rc	2	PRIVE	No	68	0	0
0	0	Thoracotoı	2	PRIVE	No	48	0	1
0	0	Thoracotoı	2	PRIVE	No	60	0	0
0	1	VATS or rc	2	PRIVE	No	71	1	0
0	0	VATS or rc	2	PRIVE	No	72	0	1
0	0	VATS or rc	2	PRIVE	No	71	0	0
1	0	Thoracotoı	32	PRIVE	No	56	0	1
1	0	Thoracotoı	32	PRIVE	No	57	0	1
1	0	Thoracotoı	32	PRIVE	No	56	0	1
1	0	Thoracotoı	32	PRIVE	No	62	0	0
1	0	Thoracotoı	32	PRIVE	No	56	0	1

1	0	Thoracotoı	32	PRIVE	No	52	0	1
1	0	Thoracotoı	32	PRIVE	No	71	1	0
1	0	Thoracotoı	32	PRIVE	No	43	0	1
1	0	Thoracotoı	32	PRIVE	Yes	66	0	1
1	0	Thoracotoı	32	PRIVE	No	81	0	0
1	0	Thoracotoı	32	PRIVE	No	60	0	0
1	0	Thoracotoı	32	PRIVE	No	72	0	1
1	0	Thoracotoı	32	PRIVE	No	60	0	0
1	0	Thoracotoı	32	PRIVE	No	70	0	0
1	0	Thoracotoı	32	PRIVE	No	55	0	1
1	0	Thoracotoı	32	PRIVE	No	71	1	1
1	0	Thoracotoı	32	PRIVE	No	22	0	0
1	0	Thoracotoı	32	PRIVE	No	73	0	0
1	0	Thoracotoı	32	PRIVE	No	83	0	0
1	1	Thoracotoı	32	PRIVE	No	67	0	0
1	0	Thoracotoı	32	PRIVE	No	85	1	0
1	0	Thoracotoı	32	PRIVE	No	64	0	0
1	0	Thoracotoı	32	PRIVE	No	71	1	1
1	0	Thoracotoı	32	PRIVE	No	64	0	0
1	0	Thoracotoı	32	PRIVE	No	58	0	1
1	0	Thoracotoı	32	PRIVE	No	74	0	0
1	0	VATS or rc	32	PRIVE	No	72	0	0
1	0	Thoracotoı	32	PRIVE	No	64	0	0
1	0	Thoracotoı	56	CHG	No	57	0	0
1	0	Thoracotoı	56	CHG	No	83	0	0
1	0	Thoracotoı	56	CHG	No	70	0	0
1	0	Thoracotoı	56	CHG	No	53	0	1
1	1	Thoracotoı	56	CHG	No	74	0	0
1	0	Thoracotoı	56	CHG	No	75	0	0
1	0	Thoracotoı	56	CHG	No	61	0	1
1	0	Thoracotoı	56	CHG	No	52	0	0
1	0	Thoracotoı	56	CHG	No	57	1	0
1	0	Thoracotoı	56	CHG	No	72	1	0
1	0	Thoracotoı	56	CHG	No	67	0	1
1	0	Thoracotoı	56	CHG	No	49	0	1
1	0	Thoracotoı	56	CHG	No	55	0	1
1	0	Thoracotoı	56	CHG	No	61	0	1
1	1	Thoracotoı	56	CHG	No	75	0	0
1	0	Thoracotoı	56	CHG	No	84	0	0
1	0	Thoracotoı	56	CHG	No	70	0	0
1	0	Thoracotoı	56	CHG	No	58	0	0
1	0	Thoracotoı	56	CHG	No	80	0	0
1	0	Thoracotoı	56	CHG	No	78	1	0
1	0	Thoracotoı	56	CHG	No	58	0	0
1	0	Thoracotoı	56	CHG	No	53	0	0
1	1	Thoracotoı	56	CHG	No	57	0	0
1	0	VATS or rc	56	CHG	No	70	0	0
1	0	Thoracotoı	56	CHG	No	77	0	0
1	0	Thoracotoı	56	CHG	No	73	0	1
1	0	Thoracotoı	56	CHG	No	78	0	0
1	0	Thoracotoı	56	CHG	No	65	0	0
1	0	Thoracotoı	56	CHG	No	54	0	0
1	0	Thoracotoı	56	CHG	No	66	0	0
1	0	Thoracotoı	56	CHG	No	64	0	0
1	0	Thoracotoı	56	CHG	No	61	0	0
1	0	Thoracotoı	56	CHG	No	51	0	0

1	0	Thoracotoı	56	CHG	Yes	70	0	1
1	0	Thoracotoı	56	CHG	No	53	0	1
1	0	Thoracotoı	56	CHG	No	64	0	1
1	0	Thoracotoı	56	CHG	No	66	0	1
1	0	Thoracotoı	56	CHG	No	70	1	0
1	0	Thoracotoı	56	CHG	No	59	0	1
1	1	Thoracotoı	56	CHG	No	58	0	0
1	0	Thoracotoı	56	CHG	No	47	0	0
1	0	Thoracotoı	56	CHG	No	49	0	0
1	0	Thoracotoı	56	CHG	No	62	0	0
1	0	Thoracotoı	56	CHG	No	62	0	0
1	1	Thoracotoı	56	CHG	No	51	0	0
1	0	Thoracotoı	56	CHG	No	64	0	1
1	0	Thoracotoı	56	CHG	No	73	0	0
1	0	Thoracotoı	56	CHG	No	78	0	1
1	0	Thoracotoı	56	CHG	No	68	0	1
1	0	Thoracotoı	56	CHG	No	58	0	0
1	0	Thoracotoı	56	CHG	No	66	0	0
1	0	Thoracotoı	56	CHG	No	60	0	1
1	0	Thoracotoı	56	CHG	No	78	0	0
1	0	Thoracotoı	56	CHG	No	61	0	0
1	0	Thoracotoı	56	CHG	No	51	0	0
1	0	Thoracotoı	56	CHG	No	60	0	0
1	0	Thoracotoı	56	CHG	No	67	0	0
1	0	Thoracotoı	56	CHG	No	69	0	1
1	0	Thoracotoı	56	CHG	No	63	0	0
1	0	Thoracotoı	56	CHG	No	70	0	0
1	0	Thoracotoı	56	CHG	No	57	0	0
1	0	Thoracotoı	56	CHG	Yes	63	1	0
1	0	Thoracotoı	56	CHG	No	54	0	1
1	0	Thoracotoı	56	CHG	No	62	0	0
1	0	Thoracotoı	56	CHG	No	78	0	0
1	0	Thoracotoı	56	CHG	No	56	0	0
1	0	Thoracotoı	56	CHG	No	58	0	0
1	0	Thoracotoı	56	CHG	No	66	0	0
1	0	Thoracotoı	56	CHG	No	68	1	0
1	0	Thoracotoı	56	CHG	No	67	0	0
1	0	Thoracotoı	56	CHG	No	67	0	0
1	0	Thoracotoı	56	CHG	No	79	1	1
1	0	Thoracotoı	56	CHG	No	66	0	0
1	0	Thoracotoı	56	CHG	No	63	0	0
1	0	Thoracotoı	56	CHG	No	78	0	0
1	0	Thoracotoı	56	CHG	No	60	0	1
1	0	Thoracotoı	56	CHG	No	71	0	0
1	0	Thoracotoı	56	CHG	No	70	0	0
1	0	Thoracotoı	56	CHG	No	80	1	0
1	0	Thoracotoı	56	CHG	No	53	0	1
1	0	Thoracotoı	56	CHG	No	57	0	1
1	0	Thoracotoı	56	CHG	No	61	0	0
1	0	Thoracotoı	56	CHG	No	59	0	1
1	0	Thoracotoı	56	CHG	No	84	1	1
1	0	Thoracotoı	56	CHG	No	69	0	0
1	0	Thoracotoı	56	CHG	Yes	65	0	1
1	0	Thoracotoı	56	CHG	No	64	0	1
1	0	Thoracotoı	56	CHG	No	57	0	0
1	0	Thoracotoı	56	CHG	No	73	0	0

1	0	Thoracotoı	56	CHG	No	73	0	0
1	0	Thoracotoı	56	CHG	No	64	0	0
1	0	Thoracotoı	56	CHG	No	64	0	0
1	0	Thoracotoı	56	CHG	No	60	0	0
1	0	Thoracotoı	56	CHG	Yes	72	1	0
1	0	Thoracotoı	56	CHG	No	78	0	0
1	0	Thoracotoı	56	CHG	No	74	0	1
1	0	Thoracotoı	56	CHG	No	69	0	1
1	0	Thoracotoı	56	CHG	No	57	0	0
1	0	Thoracotoı	56	CHG	No	68	0	1
1	0	Thoracotoı	56	CHG	No	72	0	1
1	0	Thoracotoı	56	CHG	No	68	0	1
1	0	Thoracotoı	56	CHG	No	80	0	0
1	0	Thoracotoı	56	CHG	No	78	0	0
1	0	Thoracotoı	56	CHG	No	76	1	0
1	0	VATS or rc	56	CHG	No	81	0	0
1	0	Thoracotoı	56	CHG	No	54	0	0
1	0	Thoracotoı	56	CHG	No	75	0	0
1	0	Thoracotoı	56	CHG	No	73	0	0
1	0	Thoracotoı	56	CHG	No	77	0	0
1	0	Thoracotoı	56	CHG	No	66	0	1
1	0	Thoracotoı	56	CHG	No	56	0	0
1	0	Thoracotoı	56	CHG	No	52	0	1
1	0	Thoracotoı	56	CHG	No	56	0	0
1	0	Thoracotoı	56	CHG	No	58	0	1
1	0	Thoracotoı	56	CHG	No	65	0	0
1	0	Thoracotoı	56	CHG	No	71	0	1
1	0	Thoracotoı	56	CHG	No	66	1	0
1	1	Thoracotoı	56	CHG	No	51	0	0
1	0	VATS or rc	56	CHG	No	68	1	0
1	0	Thoracotoı	56	CHG	No	68	0	0
1	0	Thoracotoı	56	CHG	No	61	0	0
0	0	Thoracotoı	30	CHG	No	58	0	0
0	1	VATS or rc	30	CHG	No	66	0	1
0	0	Thoracotoı	30	CHG	No	70	1	0
0	1	Thoracotoı	249	PRIVE	No	51	0	0
0	0	Thoracotoı	249	PRIVE	No	56	0	0
0	0	VATS or rc	249	PRIVE	No	70	0	0
0	0	VATS or rc	249	PRIVE	No	67	0	0
0	1	Thoracotoı	249	PRIVE	No	83	0	0
0	0	Thoracotoı	249	PRIVE	Yes	75	0	0
0	0	Thoracotoı	249	PRIVE	No	66	0	0
0	0	Thoracotoı	249	PRIVE	No	75	0	0
0	0	Thoracotoı	249	PRIVE	No	70	0	0
0	0	Thoracotoı	249	PRIVE	No	72	0	0
0	1	Thoracotoı	249	PRIVE	No	56	0	0
0	1	Thoracotoı	249	PRIVE	No	60	0	1
0	0	VATS or rc	249	PRIVE	No	61	0	0
0	0	VATS or rc	249	PRIVE	No	72	1	1
0	1	Thoracotoı	249	PRIVE	No	66	0	0
0	0	Thoracotoı	249	PRIVE	No	55	0	0
0	1	Thoracotoı	249	PRIVE	No	77	0	0
0	0	Thoracotoı	249	PRIVE	No	56	0	1
0	1	Thoracotoı	249	PRIVE	No	74	0	0
0	1	Thoracotoı	249	PRIVE	No	72	0	0
0	0	Thoracotoı	249	PRIVE	No	55	0	0

0	0	VATS or rc	249	PRIVE	No	53	0	1
0	0	Thoracotoı	249	PRIVE	No	58	0	0
0	0	Thoracotoı	249	PRIVE	No	57	0	0
0	0	Thoracotoı	249	PRIVE	No	69	0	1
0	0	Thoracotoı	249	PRIVE	No	71	0	0
0	0	Thoracotoı	249	PRIVE	No	66	0	0
0	0	Thoracotoı	249	PRIVE	No	59	0	1
0	1	Thoracotoı	249	PRIVE	No	70	0	0
0	0	VATS or rc	249	PRIVE	No	65	0	0
0	0	Thoracotoı	249	PRIVE	No	74	0	0
0	0	Thoracotoı	249	PRIVE	No	70	0	0
0	0	Thoracotoı	249	PRIVE	No	78	0	0
0	0	Thoracotoı	249	PRIVE	No	65	0	0
0	0	Thoracotoı	249	PRIVE	No	62	0	0
0	0	Thoracotoı	249	PRIVE	No	54	0	0
0	0	Thoracotoı	249	PRIVE	No	51	0	0
0	1	Thoracotoı	249	PRIVE	No	73	0	0
0	0	VATS or rc	249	PRIVE	No	66	1	0
0	0	Thoracotoı	249	PRIVE	No	71	0	0
0	1	Thoracotoı	249	PRIVE	No	49	0	1
0	0	Thoracotoı	249	PRIVE	No	66	0	0
0	0	Thoracotoı	249	PRIVE	No	51	0	0
0	0	Thoracotoı	249	PRIVE	No	71	0	0
0	0	Thoracotoı	249	PRIVE	No	69	0	0
0	0	VATS or rc	249	PRIVE	No	66	0	0
0	0	Thoracotoı	249	PRIVE	No	57	0	0
0	1	Thoracotoı	249	PRIVE	No	62	0	0
0	0	Thoracotoı	249	PRIVE	No	79	0	1
0	0	Thoracotoı	249	PRIVE	No	62	0	1
0	0	Thoracotoı	249	PRIVE	No	65	0	0
0	0	Thoracotoı	249	PRIVE	No	60	0	0
0	0	Thoracotoı	249	PRIVE	No	46	0	0
0	0	Thoracotoı	249	PRIVE	No	66	0	0
0	0	Thoracotoı	249	PRIVE	No	60	0	1
0	0	VATS or rc	249	PRIVE	No	66	0	0
0	0	Thoracotoı	249	PRIVE	No	51	0	1
0	0	Thoracotoı	249	PRIVE	No	67	0	0
0	0	VATS or rc	249	PRIVE	No	53	0	0
0	0	Thoracotoı	249	PRIVE	No	37	0	0
0	1	Thoracotoı	249	PRIVE	No	51	0	0
0	0	Thoracotoı	249	PRIVE	No	64	0	0
0	0	Thoracotoı	249	PRIVE	No	50	0	0
0	0	Thoracotoı	249	PRIVE	No	60	0	1
0	0	VATS or rc	249	PRIVE	No	57	0	0
0	0	VATS or rc	249	PRIVE	No	79	0	1
0	0	Thoracotoı	249	PRIVE	No	62	0	1
0	0	VATS or rc	249	PRIVE	No	68	0	0
0	0	Thoracotoı	249	PRIVE	No	45	0	0
0	0	Thoracotoı	249	PRIVE	No	58	0	1
0	0	Thoracotoı	249	PRIVE	No	63	0	1
0	0	VATS or rc	249	PRIVE	No	59	0	0
0	0	Thoracotoı	249	PRIVE	No	52	0	0
0	0	VATS or rc	249	PRIVE	No	48	0	0
0	0	Thoracotoı	249	PRIVE	No	69	0	0
0	0	VATS or rc	249	PRIVE	No	61	0	0
0	0	Thoracotoı	249	PRIVE	No	53	0	1

0	0	Thoracotoı	249	PRIVE	Yes	65	0	0
0	0	Thoracotoı	249	PRIVE	No	61	0	0
0	0	Thoracotoı	249	PRIVE	No	44	1	1
0	0	VATS or rc	249	PRIVE	No	76	0	1
0	0	Thoracotoı	249	PRIVE	No	59	0	1
0	0	Thoracotoı	249	PRIVE	Yes	80	0	1
0	0	VATS or rc	249	PRIVE	No	66	0	1
0	0	Thoracotoı	249	PRIVE	No	53	0	0
0	1	Thoracotoı	249	PRIVE	No	64	0	0
0	0	Thoracotoı	249	PRIVE	No	73	0	0
0	0	VATS or rc	249	PRIVE	No	63	0	1
0	0	Thoracotoı	249	PRIVE	No	70	0	0
0	0	VATS or rc	249	PRIVE	No	67	0	0
0	0	Thoracotoı	249	PRIVE	No	62	0	0
0	1	Thoracotoı	249	PRIVE	No	58	0	0
0	0	VATS or rc	249	PRIVE	No	58	0	0
0	0	Thoracotoı	249	PRIVE	No	50	0	1
0	0	VATS or rc	249	PRIVE	No	59	0	0
0	0	VATS or rc	249	PRIVE	No	65	0	0
0	0	Thoracotoı	249	PRIVE	No	76	0	0
0	0	Thoracotoı	249	PRIVE	No	56	0	0
0	0	Thoracotoı	249	PRIVE	No	76	0	0
0	0	Thoracotoı	249	PRIVE	No	63	0	1
0	0	Thoracotoı	249	PRIVE	No	66	0	0
0	0	Thoracotoı	249	PRIVE	Yes	84	0	0
0	1	Thoracotoı	249	PRIVE	No	59	0	0
0	0	VATS or rc	249	PRIVE	No	74	0	0
0	0	Thoracotoı	249	PRIVE	No	61	0	0
0	1	Thoracotoı	249	PRIVE	No	56	0	0
0	0	VATS or rc	249	PRIVE	No	70	1	1
0	0	Thoracotoı	249	PRIVE	No	55	0	0
0	0	Thoracotoı	249	PRIVE	No	68	0	0
0	0	Thoracotoı	249	PRIVE	No	68	0	0
0	0	Thoracotoı	249	PRIVE	No	50	0	1
0	0	Thoracotoı	249	PRIVE	No	74	0	0
0	0	Thoracotoı	249	PRIVE	No	70	0	0
0	0	VATS or rc	249	PRIVE	No	63	0	0
0	0	Thoracotoı	249	PRIVE	No	66	0	0
0	0	Thoracotoı	249	PRIVE	No	54	0	0
0	0	VATS or rc	249	PRIVE	No	71	0	0
0	0	Thoracotoı	249	PRIVE	No	67	0	0
0	0	Thoracotoı	249	PRIVE	No	55	0	1
0	0	Thoracotoı	249	PRIVE	No	46	0	1
0	0	Thoracotoı	249	PRIVE	No	72	0	0
0	0	Thoracotoı	249	PRIVE	No	67	0	0
0	0	Thoracotoı	249	PRIVE	No	67	0	1
0	0	Thoracotoı	249	PRIVE	No	68	0	0
0	0	VATS or rc	249	PRIVE	No	63	0	0
0	0	Thoracotoı	249	PRIVE	No	53	0	1
0	0	Thoracotoı	249	PRIVE	No	70	0	0
0	0	Thoracotoı	249	PRIVE	No	64	0	0
0	0	Thoracotoı	249	PRIVE	No	54	0	1
0	0	Thoracotoı	249	PRIVE	No	66	0	1
0	0	Thoracotoı	249	PRIVE	No	74	0	0
0	0	Thoracotoı	249	PRIVE	No	60	0	0
0	1	Thoracotoı	249	PRIVE	No	55	0	0

0	0	VATS or ro	249	PRIVE	No	59	0	0
0	0	Thoracotor	249	PRIVE	No	75	0	0
0	0	VATS or ro	249	PRIVE	No	64	0	1
0	0	Thoracotor	249	PRIVE	No	61	0	0
0	0	Thoracotor	249	PRIVE	No	84	0	0
0	1	Thoracotor	249	PRIVE	No	74	0	0
0	0	Thoracotor	249	PRIVE	No	79	1	0
0	1	Thoracotor	249	PRIVE	No	57	0	0
0	0	VATS or ro	249	PRIVE	No	65	0	0
0	0	VATS or ro	249	PRIVE	No	81	0	0
0	0	Thoracotor	249	PRIVE	Yes	65	0	0
0	0	VATS or ro	249	PRIVE	No	79	0	1
0	0	Thoracotor	249	PRIVE	No	62	0	0
0	0	Thoracotor	249	PRIVE	No	53	0	1
0	1	Thoracotor	249	PRIVE	No	68	0	1
0	0	Thoracotor	249	PRIVE	No	55	0	0
0	0	VATS or ro	249	PRIVE	No	65	0	0
0	0	VATS or ro	249	PRIVE	No	52	0	0
0	0	Thoracotor	249	PRIVE	No	77	0	0
0	0	Thoracotor	249	PRIVE	No	66	0	0
0	0	VATS or ro	249	PRIVE	No	52	0	0
0	0	Thoracotor	249	PRIVE	No	53	0	0
0	0	VATS or ro	249	PRIVE	No	66	0	0
0	0	Thoracotor	249	PRIVE	No	71	0	0
0	0	Thoracotor	249	PRIVE	No	68	0	0
0	0	Thoracotor	249	PRIVE	No	71	0	0
0	0	Thoracotor	249	PRIVE	No	58	0	0
0	0	Thoracotor	249	PRIVE	No	54	0	0
0	0	Thoracotor	249	PRIVE	No	62	0	0
0	0	Thoracotor	249	PRIVE	No	53	0	0
0	0	Thoracotor	249	PRIVE	No	58	0	0
0	0	VATS or ro	249	PRIVE	No	57	0	0
0	0	Thoracotor	249	PRIVE	No	66	0	0
0	0	VATS or ro	249	PRIVE	No	63	0	1
0	0	VATS or ro	249	PRIVE	No	78	0	0
0	0	VATS or ro	249	PRIVE	No	69	0	1
0	1	Thoracotor	249	PRIVE	No	65	0	0
0	1	Thoracotor	249	PRIVE	No	67	0	0
0	0	Thoracotor	249	PRIVE	No	66	0	0
0	0	Thoracotor	249	PRIVE	No	64	0	0
0	0	Thoracotor	249	PRIVE	No	54	0	1
0	0	VATS or ro	249	PRIVE	No	69	0	0
0	0	Thoracotor	249	PRIVE	No	54	0	0
0	0	Thoracotor	249	PRIVE	No	54	0	1
0	0	Thoracotor	249	PRIVE	No	73	0	0
0	0	VATS or ro	249	PRIVE	No	65	0	0
0	1	Thoracotor	249	PRIVE	No	78	0	0
0	0	Thoracotor	249	PRIVE	No	58	0	0
0	0	VATS or ro	249	PRIVE	No	64	0	0
0	1	Thoracotor	249	PRIVE	No	52	0	1
0	0	Thoracotor	249	PRIVE	No	68	0	1
0	0	Thoracotor	249	PRIVE	No	75	0	0
0	0	VATS or ro	249	PRIVE	No	65	0	0
0	0	VATS or ro	249	PRIVE	No	79	0	0
0	0	VATS or ro	249	PRIVE	No	75	0	0
0	0	Thoracotor	249	PRIVE	No	65	0	0

0	0	VATS or rc	249	PRIVE	No	67	0	1
0	0	VATS or rc	249	PRIVE	No	56	0	0
0	0	Thoracotoı	249	PRIVE	No	57	0	0
0	0	Thoracotoı	249	PRIVE	No	45	0	0
0	0	Thoracotoı	249	PRIVE	No	57	0	0
0	0	Thoracotoı	249	PRIVE	No	54	0	1
0	0	Thoracotoı	249	PRIVE	No	52	0	0
0	0	Thoracotoı	249	PRIVE	No	54	1	0
0	1	Thoracotoı	249	PRIVE	Yes	79	0	0
0	0	VATS or rc	249	PRIVE	No	54	0	0
0	0	VATS or rc	249	PRIVE	No	67	0	0
0	0	Thoracotoı	249	PRIVE	No	52	0	0
0	0	VATS or rc	249	PRIVE	No	49	0	0
0	0	VATS or rc	249	PRIVE	No	61	0	1
0	0	Thoracotoı	249	PRIVE	No	49	0	0
0	0	VATS or rc	249	PRIVE	No	63	0	0
0	0	Thoracotoı	249	PRIVE	No	49	0	0
0	0	Thoracotoı	249	PRIVE	No	78	0	0
0	0	Thoracotoı	249	PRIVE	No	58	0	0
0	0	VATS or rc	249	PRIVE	Yes	78	0	0
0	1	Thoracotoı	249	PRIVE	No	56	0	1
0	0	VATS or rc	249	PRIVE	No	57	0	0
0	0	VATS or rc	249	PRIVE	No	58	0	1
0	0	Thoracotoı	249	PRIVE	No	44	0	0
0	0	Thoracotoı	249	PRIVE	No	75	0	0
0	1	Thoracotoı	249	PRIVE	No	63	0	0
0	0	Thoracotoı	249	PRIVE	No	72	0	1
0	0	Thoracotoı	249	PRIVE	No	60	0	0
0	0	Thoracotoı	249	PRIVE	No	69	0	1
0	0	Thoracotoı	249	PRIVE	No	48	0	0
0	0	Thoracotoı	249	PRIVE	No	44	0	0
0	0	Thoracotoı	249	PRIVE	No	71	0	0
0	0	Thoracotoı	249	PRIVE	No	64	0	0
0	0	VATS or rc	249	PRIVE	No	64	0	0
0	0	Thoracotoı	249	PRIVE	No	16	0	0
0	0	Thoracotoı	249	PRIVE	No	65	0	0
0	0	Thoracotoı	249	PRIVE	No	67	0	0
0	0	Thoracotoı	249	PRIVE	No	60	0	0
0	0	Thoracotoı	249	PRIVE	No	54	0	0
0	0	Thoracotoı	249	PRIVE	No	54	0	0
0	1	Thoracotoı	249	PRIVE	No	80	0	0
0	0	Thoracotoı	249	PRIVE	No	66	0	0
0	0	Thoracotoı	249	PRIVE	No	65	0	0
0	0	Thoracotoı	249	PRIVE	No	60	0	1
0	0	Thoracotoı	249	PRIVE	No	51	0	0
0	0	Thoracotoı	249	PRIVE	No	20	0	0
0	0	Thoracotoı	249	PRIVE	No	55	0	0
0	0	Thoracotoı	249	PRIVE	No	66	0	1
0	0	VATS or rc	249	PRIVE	No	64	0	0
0	0	Thoracotoı	249	PRIVE	No	73	0	0
0	0	Thoracotoı	249	PRIVE	No	84	0	0
0	0	Thoracotoı	249	PRIVE	No	62	0	0
0	0	VATS or rc	249	PRIVE	No	79	0	0
0	0	Thoracotoı	249	PRIVE	No	77	0	0
0	0	VATS or rc	249	PRIVE	No	76	0	0
0	0	VATS or rc	249	PRIVE	No	69	0	0

0	1	Thoracotoı	249	PRIVE	No	58	0	0
0	0	VATS or rc	249	PRIVE	No	52	0	1
0	0	Thoracotoı	249	PRIVE	No	61	0	0
0	0	Thoracotoı	249	PRIVE	No	78	0	0
0	0	Thoracotoı	249	PRIVE	No	79	0	0
0	0	VATS or rc	249	PRIVE	No	67	1	0
0	0	VATS or rc	249	PRIVE	No	65	1	0
0	0	Thoracotoı	249	PRIVE	No	74	0	1
0	0	Thoracotoı	249	PRIVE	No	49	0	1
0	0	Thoracotoı	249	PRIVE	No	53	1	0
0	0	Thoracotoı	249	PRIVE	No	52	0	1
0	0	VATS or rc	249	PRIVE	No	73	0	1
0	0	Thoracotoı	249	PRIVE	No	66	0	0
0	0	Thoracotoı	249	PRIVE	No	70	0	0
0	1	Thoracotoı	249	PRIVE	No	55	0	1
0	1	Thoracotoı	249	PRIVE	No	72	1	1
0	0	Thoracotoı	249	PRIVE	No	59	0	0
0	1	Thoracotoı	249	PRIVE	No	66	0	0
0	0	Thoracotoı	249	PRIVE	No	64	0	1
0	0	Thoracotoı	249	PRIVE	No	70	1	0
0	1	Thoracotoı	249	PRIVE	No	68	1	0
0	0	Thoracotoı	249	PRIVE	No	71	0	0
0	0	Thoracotoı	249	PRIVE	No	54	0	0
0	0	Thoracotoı	249	PRIVE	No	67	0	0
0	0	Thoracotoı	249	PRIVE	No	62	0	0
0	0	VATS or rc	249	PRIVE	No	61	0	1
0	0	VATS or rc	249	PRIVE	No	67	0	0
0	0	VATS or rc	249	PRIVE	No	76	0	0
0	1	Thoracotoı	249	PRIVE	No	62	0	0
0	0	Thoracotoı	249	PRIVE	No	73	0	0
0	0	Thoracotoı	249	PRIVE	No	63	0	0
0	0	Thoracotoı	249	PRIVE	No	60	0	0
0	0	Thoracotoı	249	PRIVE	No	52	0	1
0	0	Thoracotoı	249	PRIVE	No	69	0	0
0	0	Thoracotoı	249	PRIVE	No	67	0	1
0	0	Thoracotoı	249	PRIVE	No	69	0	0
0	0	Thoracotoı	249	PRIVE	No	59	0	0
0	1	Thoracotoı	249	PRIVE	No	60	0	0
0	0	VATS or rc	249	PRIVE	No	70	0	1
0	0	Thoracotoı	249	PRIVE	No	57	1	0
0	0	Thoracotoı	249	PRIVE	No	74	0	0
0	0	VATS or rc	249	PRIVE	No	76	0	0
0	0	Thoracotoı	249	PRIVE	No	66	0	0
0	1	Thoracotoı	249	PRIVE	No	67	0	0
0	1	Thoracotoı	249	PRIVE	No	64	0	0
0	0	VATS or rc	249	PRIVE	No	58	0	0
0	0	Thoracotoı	249	PRIVE	No	63	0	0
0	0	Thoracotoı	249	PRIVE	No	69	0	0
0	0	Thoracotoı	249	PRIVE	No	63	0	0
0	1	Thoracotoı	249	PRIVE	No	54	0	0
0	1	Thoracotoı	249	PRIVE	No	66	0	0
0	0	Thoracotoı	249	PRIVE	No	76	0	0
0	1	Thoracotoı	249	PRIVE	No	56	0	0
0	1	Thoracotoı	249	PRIVE	No	64	0	0
0	0	Thoracotoı	249	PRIVE	No	68	0	0
0	0	Thoracotoı	249	PRIVE	No	48	0	0

0	0	Thoracotoı	249	PRIVE	No	70	0	0
0	0	Thoracotoı	249	PRIVE	No	51	0	0
0	0	VATS or ro	249	PRIVE	No	55	0	1
0	0	Thoracotoı	249	PRIVE	No	62	0	0
0	0	Thoracotoı	249	PRIVE	No	70	0	0
0	0	VATS or ro	249	PRIVE	No	58	1	0
0	0	Thoracotoı	249	PRIVE	No	73	0	0
0	1	Thoracotoı	249	PRIVE	No	71	0	0
0	0	Thoracotoı	249	PRIVE	No	76	0	0
0	0	VATS or ro	249	PRIVE	No	61	0	0
0	0	Thoracotoı	249	PRIVE	No	67	0	0
0	0	VATS or ro	249	PRIVE	No	64	0	0
0	0	Thoracotoı	249	PRIVE	No	60	0	0
0	0	Thoracotoı	249	PRIVE	No	61	0	0
0	0	Thoracotoı	249	PRIVE	No	72	0	0
0	0	Thoracotoı	249	PRIVE	No	52	0	0
0	0	Thoracotoı	249	PRIVE	No	76	0	0
0	0	Thoracotoı	249	PRIVE	No	41	0	1
0	0	Thoracotoı	249	PRIVE	No	66	0	0
0	1	Thoracotoı	249	PRIVE	No	47	0	0
0	1	Thoracotoı	249	PRIVE	No	71	0	0
0	0	VATS or ro	249	PRIVE	No	45	0	0
0	0	Thoracotoı	249	PRIVE	No	69	0	0
0	1	Thoracotoı	249	PRIVE	No	71	0	0
0	0	VATS or ro	249	PRIVE	No	65	0	0
0	0	Thoracotoı	249	PRIVE	No	73	0	0
0	0	Thoracotoı	249	PRIVE	No	67	1	0
0	0	Thoracotoı	249	PRIVE	No	66	0	0
0	0	Thoracotoı	249	PRIVE	No	68	0	1
0	0	Thoracotoı	249	PRIVE	No	60	0	0
0	0	Thoracotoı	249	PRIVE	No	78	1	0
0	1	Thoracotoı	249	PRIVE	No	64	0	0
0	0	Thoracotoı	249	PRIVE	No	59	0	1
0	0	Thoracotoı	249	PRIVE	No	52	0	1
0	1	Thoracotoı	249	PRIVE	Yes	76	0	0
0	0	Thoracotoı	249	PRIVE	No	62	0	0
0	0	Thoracotoı	249	PRIVE	No	77	0	1
0	1	Thoracotoı	249	PRIVE	No	59	0	1
0	0	Thoracotoı	249	PRIVE	No	36	0	0
0	1	Thoracotoı	249	PRIVE	No	59	0	0
0	0	Thoracotoı	249	PRIVE	No	45	0	0
0	1	Thoracotoı	249	PRIVE	No	51	0	1
0	0	Thoracotoı	249	PRIVE	No	69	0	0
0	0	Thoracotoı	249	PRIVE	No	68	0	1
0	0	Thoracotoı	249	PRIVE	No	62	0	0
0	0	Thoracotoı	249	PRIVE	No	74	0	0
0	0	Thoracotoı	249	PRIVE	No	66	0	0
0	0	Thoracotoı	249	PRIVE	No	57	0	0
0	0	Thoracotoı	249	PRIVE	No	45	0	0
0	0	Thoracotoı	249	PRIVE	No	63	0	1
0	0	Thoracotoı	249	PRIVE	No	50	0	0
0	0	Thoracotoı	249	PRIVE	No	56	0	1
0	0	Thoracotoı	249	PRIVE	No	66	0	0
0	0	Thoracotoı	249	PRIVE	No	63	0	0
0	0	Thoracotoı	249	PRIVE	No	77	0	0
0	0	Thoracotoı	249	PRIVE	No	64	0	0

0	0	Thoracotoı	249	PRIVE	No	51	0	0
0	0	Thoracotoı	249	PRIVE	No	56	0	0
0	0	Thoracotoı	249	PRIVE	No	60	0	1
0	0	VATS or rc	249	PRIVE	No	64	1	1
0	0	Thoracotoı	249	PRIVE	No	80	0	0
0	1	Thoracotoı	249	PRIVE	No	63	0	0
0	0	Thoracotoı	249	PRIVE	No	61	0	0
0	1	Thoracotoı	249	PRIVE	No	61	0	0
0	0	VATS or rc	249	PRIVE	No	54	1	1
0	0	Thoracotoı	249	PRIVE	No	61	0	0
0	0	Thoracotoı	249	PRIVE	No	58	0	1
0	0	Thoracotoı	249	PRIVE	No	66	0	0
0	0	Thoracotoı	249	PRIVE	No	82	0	0
0	1	Thoracotoı	249	PRIVE	No	71	0	0
0	0	Thoracotoı	249	PRIVE	No	66	0	0
0	1	Thoracotoı	249	PRIVE	No	52	0	0
0	1	Thoracotoı	249	PRIVE	No	57	0	0
0	0	Thoracotoı	249	PRIVE	No	61	0	0
0	0	Thoracotoı	249	PRIVE	No	45	0	1
0	0	Thoracotoı	249	PRIVE	No	70	0	0
0	0	Thoracotoı	249	PRIVE	No	61	0	1
0	0	VATS or rc	249	PRIVE	No	84	0	0
0	0	VATS or rc	249	PRIVE	No	68	0	0
0	0	Thoracotoı	249	PRIVE	No	72	0	0
0	0	Thoracotoı	249	PRIVE	No	70	0	0
0	0	Thoracotoı	249	PRIVE	No	76	0	0
0	0	Thoracotoı	249	PRIVE	No	75	0	0
0	0	Thoracotoı	249	PRIVE	No	45	0	0
0	0	VATS or rc	249	PRIVE	No	70	0	1
0	0	VATS or rc	249	PRIVE	No	63	0	0
0	0	Thoracotoı	249	PRIVE	No	56	0	0
0	0	Thoracotoı	249	PRIVE	No	69	0	0
0	0	VATS or rc	249	PRIVE	No	62	0	0
0	0	Thoracotoı	249	PRIVE	No	49	0	0
0	0	VATS or rc	249	PRIVE	No	74	0	0
0	0	Thoracotoı	249	PRIVE	No	59	0	0
0	0	Thoracotoı	249	PRIVE	No	59	0	1
0	0	VATS or rc	249	PRIVE	No	55	1	0
0	0	Thoracotoı	249	PRIVE	No	76	0	0
0	0	VATS or rc	249	PRIVE	No	67	1	1
0	0	VATS or rc	249	PRIVE	No	65	0	0
0	1	Thoracotoı	249	PRIVE	No	72	0	1
0	0	Thoracotoı	249	PRIVE	No	65	0	0
0	0	VATS or rc	249	PRIVE	No	64	0	0
0	0	Thoracotoı	249	PRIVE	No	69	0	0
0	0	Thoracotoı	249	PRIVE	No	57	0	0
0	0	VATS or rc	249	PRIVE	No	71	0	0
0	0	Thoracotoı	249	PRIVE	No	54	0	1
0	0	Thoracotoı	249	PRIVE	No	53	0	1
0	0	Thoracotoı	249	PRIVE	No	68	0	0
0	0	Thoracotoı	249	PRIVE	No	57	0	0
0	0	VATS or rc	249	PRIVE	Yes	74	0	0
0	0	Thoracotoı	249	PRIVE	No	52	0	0
0	0	Thoracotoı	249	PRIVE	No	65	0	0
0	0	Thoracotoı	249	PRIVE	No	61	0	0
0	0	Thoracotoı	249	PRIVE	No	62	0	0

0	0	VATS or rc	249	PRIVE	No	69	0	0
0	0	VATS or rc	249	PRIVE	No	50	0	0
0	1	Thoracotoı	249	PRIVE	No	73	0	0
0	0	Thoracotoı	249	PRIVE	No	72	0	1
0	0	Thoracotoı	249	PRIVE	No	62	0	0
0	0	Thoracotoı	249	PRIVE	No	75	0	0
0	0	Thoracotoı	249	PRIVE	No	48	0	0
0	0	Thoracotoı	249	PRIVE	No	54	0	0
0	0	Thoracotoı	249	PRIVE	No	65	0	0
0	0	Thoracotoı	249	PRIVE	No	60	0	0
0	1	Thoracotoı	249	PRIVE	No	66	0	0
0	1	Thoracotoı	249	PRIVE	No	52	0	0
0	1	Thoracotoı	249	PRIVE	No	51	0	1
0	0	Thoracotoı	249	PRIVE	No	49	0	0
0	0	Thoracotoı	249	PRIVE	No	64	0	1
0	0	Thoracotoı	249	PRIVE	No	66	0	0
0	0	Thoracotoı	249	PRIVE	No	75	0	0
0	0	Thoracotoı	249	PRIVE	No	75	0	0
0	0	Thoracotoı	249	PRIVE	No	54	0	1
0	1	Thoracotoı	249	PRIVE	No	74	0	0
0	1	Thoracotoı	249	PRIVE	No	61	0	0
0	0	VATS or rc	249	PRIVE	No	76	0	0
0	0	Thoracotoı	249	PRIVE	No	62	0	0
0	1	Thoracotoı	249	PRIVE	No	57	0	1
0	0	Thoracotoı	249	PRIVE	No	80	0	0
0	0	VATS or rc	249	PRIVE	No	74	0	0
0	1	Thoracotoı	249	PRIVE	No	63	0	0
0	0	Thoracotoı	249	PRIVE	No	55	1	0
0	0	Thoracotoı	249	PRIVE	No	60	0	1
0	0	Thoracotoı	249	PRIVE	No	66	0	0
0	0	Thoracotoı	249	PRIVE	No	77	0	0
0	1	Thoracotoı	249	PRIVE	No	62	0	0
0	0	Thoracotoı	249	PRIVE	No	38	0	0
0	0	Thoracotoı	249	PRIVE	No	51	0	0
0	0	VATS or rc	249	PRIVE	No	79	0	0
0	0	Thoracotoı	249	PRIVE	No	65	0	0
0	0	Thoracotoı	249	PRIVE	No	59	0	1
0	0	Thoracotoı	249	PRIVE	No	64	0	0
0	0	Thoracotoı	249	PRIVE	No	77	0	0
0	0	Thoracotoı	249	PRIVE	No	61	0	0
0	1	Thoracotoı	249	PRIVE	No	64	0	0
0	0	Thoracotoı	249	PRIVE	No	43	0	0
0	0	Thoracotoı	249	PRIVE	No	72	0	0
0	0	Thoracotoı	249	PRIVE	No	82	0	0
0	0	Thoracotoı	249	PRIVE	No	45	0	0
0	0	Thoracotoı	249	PRIVE	No	75	0	0
0	0	VATS or rc	249	PRIVE	No	63	0	0
0	0	Thoracotoı	249	PRIVE	No	65	0	0
0	0	Thoracotoı	249	PRIVE	No	65	0	1
0	1	Thoracotoı	249	PRIVE	No	60	0	0
0	1	Thoracotoı	249	PRIVE	No	73	0	1
0	0	Thoracotoı	249	PRIVE	No	73	0	0
0	0	Thoracotoı	249	PRIVE	No	57	1	0
0	0	VATS or rc	249	PRIVE	No	69	0	0
0	0	Thoracotoı	249	PRIVE	No	61	0	0
0	1	Thoracotoı	249	PRIVE	Yes	70	0	1

0	1	Thoracotoı	249	PRIVE	No	61	0	0
0	1	Thoracotoı	249	PRIVE	No	53	0	1
0	0	Thoracotoı	249	PRIVE	No	52	0	0
0	0	Thoracotoı	249	PRIVE	No	60	0	0
0	1	Thoracotoı	249	PRIVE	No	64	0	0
0	0	Thoracotoı	249	PRIVE	No	62	0	0
0	0	VATS or rc	249	PRIVE	No	53	0	0
0	0	VATS or rc	249	PRIVE	No	73	0	0
0	0	VATS or rc	249	PRIVE	No	58	0	1
0	0	Thoracotoı	249	PRIVE	No	77	0	0
0	0	Thoracotoı	249	PRIVE	No	30	0	0
0	0	VATS or rc	249	PRIVE	No	66	0	0
0	0	Thoracotoı	249	PRIVE	Yes	73	0	1
0	1	Thoracotoı	249	PRIVE	No	75	0	1
0	0	Thoracotoı	249	PRIVE	No	67	0	0
0	0	Thoracotoı	249	PRIVE	No	69	0	0
0	0	Thoracotoı	249	PRIVE	No	62	0	0
0	0	VATS or rc	249	PRIVE	No	61	0	0
0	0	Thoracotoı	249	PRIVE	No	69	0	0
0	0	VATS or rc	249	PRIVE	No	70	0	0
0	0	VATS or rc	249	PRIVE	No	47	0	0
0	0	Thoracotoı	249	PRIVE	No	58	0	0
0	0	Thoracotoı	249	PRIVE	No	63	0	0
0	0	Thoracotoı	249	PRIVE	No	69	0	0
0	0	VATS or rc	249	PRIVE	No	39	0	1
0	0	VATS or rc	249	PRIVE	No	81	0	0
0	0	Thoracotoı	249	PRIVE	No	64	0	0
0	0	Thoracotoı	249	PRIVE	Yes	65	0	0
0	0	VATS or rc	249	PRIVE	No	61	0	0
0	0	Thoracotoı	249	PRIVE	No	67	0	0
0	0	Thoracotoı	249	PRIVE	No	58	0	1
0	1	Thoracotoı	249	PRIVE	No	46	0	0
0	0	Thoracotoı	249	PRIVE	No	67	0	0
0	0	Thoracotoı	249	PRIVE	No	66	0	0
0	0	Thoracotoı	249	PRIVE	No	66	0	0
0	1	Thoracotoı	249	PRIVE	No	68	0	1
0	0	Thoracotoı	249	PRIVE	No	65	0	0
0	0	Thoracotoı	249	PRIVE	No	68	1	0
0	0	VATS or rc	249	PRIVE	No	71	0	1
0	0	VATS or rc	249	PRIVE	No	50	0	1
0	0	Thoracotoı	249	PRIVE	No	73	0	0
0	0	Thoracotoı	249	PRIVE	No	73	0	0
0	0	Thoracotoı	249	PRIVE	No	47	0	0
0	0	Thoracotoı	249	PRIVE	No	42	0	0
0	0	Thoracotoı	249	PRIVE	No	73	0	0
0	0	Thoracotoı	249	PRIVE	No	69	0	0
0	0	Thoracotoı	249	PRIVE	No	51	0	0
0	0	Thoracotoı	249	PRIVE	No	55	0	0
0	0	Thoracotoı	249	PRIVE	No	64	0	0
0	1	Thoracotoı	249	PRIVE	No	52	0	0
0	0	Thoracotoı	249	PRIVE	No	54	0	0
0	0	VATS or rc	249	PRIVE	No	53	0	0
0	0	Thoracotoı	249	PRIVE	No	71	0	0
0	0	Thoracotoı	249	PRIVE	No	79	0	1
0	0	Thoracotoı	249	PRIVE	No	60	0	1
0	0	VATS or rc	249	PRIVE	No	67	0	0

0	0	VATS or ro	249	PRIVE	No	73	0	0
0	0	VATS or ro	249	PRIVE	No	70	0	0
0	0	Thoracotoı	249	PRIVE	No	77	0	0
0	0	Thoracotoı	249	PRIVE	No	24	0	1
0	0	VATS or ro	249	PRIVE	No	70	0	0
0	0	VATS or ro	249	PRIVE	No	64	0	1
0	0	Thoracotoı	249	PRIVE	No	55	0	0
0	1	Thoracotoı	249	PRIVE	No	58	0	0
0	0	VATS or ro	249	PRIVE	No	79	0	0
0	1	Thoracotoı	249	PRIVE	No	65	0	0
0	0	Thoracotoı	249	PRIVE	No	64	0	1
0	0	Thoracotoı	249	PRIVE	No	61	0	0
0	0	Thoracotoı	249	PRIVE	No	65	0	0
0	0	Thoracotoı	249	PRIVE	No	60	0	0
0	0	Thoracotoı	249	PRIVE	No	55	0	0
0	0	VATS or ro	249	PRIVE	No	58	0	0
0	0	VATS or ro	249	PRIVE	No	64	0	1
0	0	Thoracotoı	249	PRIVE	No	75	0	0
0	0	Thoracotoı	249	PRIVE	No	66	0	0
0	0	Thoracotoı	249	PRIVE	No	63	0	0
0	0	Thoracotoı	249	PRIVE	No	71	0	0
0	0	Thoracotoı	249	PRIVE	No	78	0	0
0	0	Thoracotoı	249	PRIVE	Yes	76	0	0
0	0	VATS or ro	249	PRIVE	No	64	0	0
0	0	Thoracotoı	249	PRIVE	No	63	0	0
0	1	Thoracotoı	249	PRIVE	No	52	0	1
0	0	Thoracotoı	249	PRIVE	No	57	0	1
0	0	Thoracotoı	249	PRIVE	No	63	0	0
0	0	Thoracotoı	249	PRIVE	No	54	0	0
0	0	Thoracotoı	249	PRIVE	No	60	0	0
0	0	Thoracotoı	249	PRIVE	No	64	0	0
0	0	Thoracotoı	249	PRIVE	No	86	0	0
0	0	Thoracotoı	249	PRIVE	No	58	0	0
0	0	Thoracotoı	249	PRIVE	No	65	0	0
0	0	Thoracotoı	249	PRIVE	No	73	0	0
0	0	Thoracotoı	249	PRIVE	No	41	0	0
0	0	Thoracotoı	249	PRIVE	No	72	0	0
0	1	Thoracotoı	249	PRIVE	No	52	0	0
0	0	Thoracotoı	249	PRIVE	No	52	0	0
0	0	VATS or ro	249	PRIVE	No	71	0	1
0	0	VATS or ro	249	PRIVE	No	83	0	0
0	0	Thoracotoı	249	PRIVE	No	57	0	1
0	0	Thoracotoı	249	PRIVE	No	56	0	0
0	0	Thoracotoı	249	PRIVE	No	58	1	0
0	0	Thoracotoı	249	PRIVE	No	51	0	1
0	0	VATS or ro	249	PRIVE	No	79	0	0
0	1	Thoracotoı	249	PRIVE	No	43	0	0
0	0	Thoracotoı	249	PRIVE	No	63	0	0
0	1	Thoracotoı	249	PRIVE	No	64	0	0
0	0	Thoracotoı	249	PRIVE	No	64	0	0
0	0	Thoracotoı	249	PRIVE	No	58	0	0
0	0	Thoracotoı	249	PRIVE	No	63	0	0
0	0	VATS or ro	249	PRIVE	No	75	0	1
0	0	VATS or ro	249	PRIVE	No	66	0	0
0	0	VATS or ro	249	PRIVE	No	62	0	1
0	0	Thoracotoı	249	PRIVE	No	81	0	0

0	0	Thoracotoı	249	PRIVE	No	82	0	0
0	0	VATS or rc	249	PRIVE	No	56	0	0
0	1	Thoracotoı	249	PRIVE	No	64	0	0
0	0	Thoracotoı	249	PRIVE	No	78	0	0
0	0	Thoracotoı	249	PRIVE	No	58	0	0
0	0	Thoracotoı	249	PRIVE	No	60	0	0
0	0	VATS or rc	249	PRIVE	No	54	1	0
0	0	Thoracotoı	249	PRIVE	No	48	0	1
0	0	VATS or rc	249	PRIVE	No	45	0	0
0	0	Thoracotoı	249	PRIVE	No	54	0	0
0	1	Thoracotoı	249	PRIVE	No	61	0	0
0	0	Thoracotoı	249	PRIVE	No	67	0	0
0	0	VATS or rc	249	PRIVE	No	70	0	0
0	0	Thoracotoı	249	PRIVE	No	75	0	0
0	1	Thoracotoı	249	PRIVE	No	76	0	0
0	0	Thoracotoı	249	PRIVE	No	69	0	0
0	0	Thoracotoı	249	PRIVE	No	64	0	0
0	0	Thoracotoı	249	PRIVE	No	58	0	1
0	0	Thoracotoı	249	PRIVE	No	65	0	0
0	0	VATS or rc	249	PRIVE	No	66	0	0
0	0	Thoracotoı	249	PRIVE	No	70	0	0
0	1	Thoracotoı	249	PRIVE	No	58	0	1
0	1	Thoracotoı	249	PRIVE	No	63	0	0
0	0	Thoracotoı	249	PRIVE	No	74	0	0
0	1	Thoracotoı	249	PRIVE	No	45	0	0
0	0	Thoracotoı	249	PRIVE	No	65	0	0
0	0	Thoracotoı	249	PRIVE	No	61	0	1
0	0	Thoracotoı	249	PRIVE	No	66	0	1
0	0	VATS or rc	249	PRIVE	No	57	0	1
0	0	Thoracotoı	249	PRIVE	No	57	0	1
0	0	VATS or rc	249	PRIVE	No	62	0	0
0	0	Thoracotoı	249	PRIVE	No	62	0	1
0	0	Thoracotoı	249	PRIVE	No	79	0	0
0	0	Thoracotoı	249	PRIVE	No	57	0	0
0	0	VATS or rc	249	PRIVE	No	67	0	1
0	1	Thoracotoı	249	PRIVE	No	68	0	0
0	0	VATS or rc	249	PRIVE	No	70	0	0
0	0	Thoracotoı	249	PRIVE	No	53	0	0
0	0	Thoracotoı	249	PRIVE	No	74	0	0
0	0	Thoracotoı	249	PRIVE	No	66	0	1
0	0	Thoracotoı	249	PRIVE	No	68	0	0
0	0	VATS or rc	249	PRIVE	No	70	0	0
0	0	Thoracotoı	249	PRIVE	No	50	0	0
0	0	VATS or rc	249	PRIVE	No	60	0	0
0	0	Thoracotoı	249	PRIVE	No	67	0	0
0	0	Thoracotoı	249	PRIVE	No	80	0	0
0	0	VATS or rc	249	PRIVE	No	54	0	0
0	0	Thoracotoı	249	PRIVE	No	62	0	0
0	0	Thoracotoı	249	PRIVE	No	65	1	0
0	1	Thoracotoı	249	PRIVE	No	62	0	1
0	0	Thoracotoı	249	PRIVE	No	78	0	0
0	0	Thoracotoı	249	PRIVE	No	69	0	0
0	0	VATS or rc	249	PRIVE	No	65	0	0
0	0	VATS or rc	249	PRIVE	No	44	0	0
0	0	Thoracotoı	249	PRIVE	No	73	0	0
0	0	Thoracotoı	249	PRIVE	No	62	0	0

0	0	VATS or rc	249	PRIVE	No	75	0	0
0	1	Thoracotoı	249	PRIVE	No	39	0	0
0	0	Thoracotoı	249	PRIVE	No	61	0	0
0	0	Thoracotoı	249	PRIVE	No	71	0	1
0	0	VATS or rc	249	PRIVE	No	65	0	0
0	1	Thoracotoı	249	PRIVE	No	68	0	0
0	0	Thoracotoı	249	PRIVE	No	66	0	0
0	0	Thoracotoı	249	PRIVE	No	81	0	0
0	0	Thoracotoı	249	PRIVE	No	69	0	0
0	0	Thoracotoı	249	PRIVE	No	69	0	0
0	0	Thoracotoı	249	PRIVE	No	78	1	0
0	0	Thoracotoı	249	PRIVE	No	60	1	0
0	0	VATS or rc	249	PRIVE	No	63	0	0
0	0	Thoracotoı	249	PRIVE	No	64	0	0
0	0	Thoracotoı	249	PRIVE	No	75	0	0
0	1	Thoracotoı	249	PRIVE	No	61	0	0
0	0	Thoracotoı	249	PRIVE	No	72	0	0
0	0	VATS or rc	249	PRIVE	No	61	1	0
0	0	VATS or rc	249	PRIVE	No	76	0	0
0	0	Thoracotoı	249	PRIVE	No	53	0	0
0	0	Thoracotoı	249	PRIVE	No	75	0	0
0	0	Thoracotoı	249	PRIVE	No	69	0	0
0	1	Thoracotoı	249	PRIVE	No	65	0	0
0	0	Thoracotoı	249	PRIVE	No	77	0	0
0	0	Thoracotoı	249	PRIVE	No	71	0	0
0	0	Thoracotoı	249	PRIVE	No	76	0	0
0	0	Thoracotoı	249	PRIVE	No	58	0	0
0	0	Thoracotoı	249	PRIVE	No	64	0	0
0	0	VATS or rc	249	PRIVE	No	81	0	0
0	0	Thoracotoı	249	PRIVE	No	67	0	0
0	0	Thoracotoı	249	PRIVE	No	44	0	1
0	0	VATS or rc	249	PRIVE	No	63	0	0
0	0	VATS or rc	249	PRIVE	No	61	0	0
0	0	Thoracotoı	249	PRIVE	No	47	0	1
0	0	Thoracotoı	249	PRIVE	No	61	0	0
0	0	Thoracotoı	249	PRIVE	No	73	0	0
0	0	Thoracotoı	249	PRIVE	No	62	0	0
0	1	Thoracotoı	249	PRIVE	No	67	0	0
0	0	VATS or rc	249	PRIVE	No	62	1	0
0	0	Thoracotoı	249	PRIVE	No	51	0	1
0	1	Thoracotoı	249	PRIVE	No	65	0	0
0	1	Thoracotoı	249	PRIVE	No	61	0	0
0	0	VATS or rc	249	PRIVE	No	59	0	0
0	0	Thoracotoı	249	PRIVE	No	55	0	0
0	0	Thoracotoı	249	PRIVE	No	80	0	0
0	0	Thoracotoı	249	PRIVE	No	76	0	0
0	0	Thoracotoı	249	PRIVE	No	58	0	1
0	0	Thoracotoı	249	PRIVE	No	74	0	0
0	1	Thoracotoı	249	PRIVE	No	61	0	0
0	0	VATS or rc	249	PRIVE	No	81	1	0
0	1	Thoracotoı	249	PRIVE	No	64	0	0
0	0	Thoracotoı	249	PRIVE	No	56	0	0
0	0	Thoracotoı	249	PRIVE	No	76	0	0
0	0	Thoracotoı	249	PRIVE	No	59	0	0
0	1	Thoracotoı	249	PRIVE	Yes	64	0	0
0	0	VATS or rc	249	PRIVE	No	75	0	0

0	0	Thoracotoı	249	PRIVE	No	61	0	0
0	0	Thoracotoı	249	PRIVE	No	57	0	0
0	0	Thoracotoı	249	PRIVE	No	68	0	0
0	0	Thoracotoı	249	PRIVE	No	57	0	0
0	0	Thoracotoı	249	PRIVE	No	56	0	0
0	0	VATS or rc	249	PRIVE	No	62	0	0
0	1	Thoracotoı	249	PRIVE	No	75	1	0
0	0	VATS or rc	249	PRIVE	No	77	0	0
0	0	VATS or rc	249	PRIVE	No	64	0	0
0	1	Thoracotoı	249	PRIVE	No	72	0	0
0	1	Thoracotoı	249	PRIVE	No	69	0	0
0	0	Thoracotoı	249	PRIVE	No	70	0	0
0	0	Thoracotoı	249	PRIVE	No	70	0	0
0	0	Thoracotoı	249	PRIVE	No	55	0	0
0	1	Thoracotoı	249	PRIVE	No	68	0	0
0	0	Thoracotoı	249	PRIVE	No	69	0	0
0	0	Thoracotoı	249	PRIVE	No	64	0	0
0	0	Thoracotoı	249	PRIVE	No	71	0	0
0	0	Thoracotoı	249	PRIVE	No	45	0	0
0	0	Thoracotoı	249	PRIVE	No	80	0	0
0	0	Thoracotoı	249	PRIVE	No	78	0	0
0	0	Thoracotoı	249	PRIVE	No	63	1	0
0	0	Thoracotoı	249	PRIVE	No	64	0	0
0	0	Thoracotoı	249	PRIVE	Yes	53	0	0
0	1	Thoracotoı	249	PRIVE	No	64	0	0
0	0	VATS or rc	249	PRIVE	No	65	0	0
0	0	Thoracotoı	249	PRIVE	No	67	0	0
0	0	Thoracotoı	249	PRIVE	No	75	0	0
0	0	VATS or rc	249	PRIVE	No	58	0	0
0	0	Thoracotoı	249	PRIVE	No	60	0	0
0	0	VATS or rc	249	PRIVE	No	63	0	1
0	0	Thoracotoı	249	PRIVE	No	78	0	1
0	0	VATS or rc	249	PRIVE	No	65	0	1
0	0	Thoracotoı	249	PRIVE	No	59	0	0
0	1	Thoracotoı	249	PRIVE	No	71	0	0
0	0	Thoracotoı	249	PRIVE	No	78	0	0
0	0	Thoracotoı	249	PRIVE	No	59	0	1
0	0	VATS or rc	249	PRIVE	No	53	0	0
0	0	VATS or rc	249	PRIVE	No	66	0	0
0	0	Thoracotoı	249	PRIVE	No	57	0	0
0	0	Thoracotoı	249	PRIVE	No	49	0	0
0	1	Thoracotoı	249	PRIVE	No	77	1	0
0	0	Thoracotoı	249	PRIVE	No	51	0	0
0	1	Thoracotoı	249	PRIVE	No	62	0	0
0	0	Thoracotoı	249	PRIVE	No	78	0	0
0	0	Thoracotoı	249	PRIVE	No	70	0	0
0	0	VATS or rc	249	PRIVE	No	77	0	0
0	0	VATS or rc	249	PRIVE	No	56	0	0
0	0	Thoracotoı	249	PRIVE	No	60	0	0
0	0	Thoracotoı	249	PRIVE	No	56	0	0
0	0	VATS or rc	249	PRIVE	No	71	0	0
0	0	Thoracotoı	249	PRIVE	No	44	0	0
0	0	Thoracotoı	249	PRIVE	No	65	0	0
0	0	VATS or rc	249	PRIVE	No	72	0	0
0	0	Thoracotoı	249	PRIVE	No	72	0	0
0	0	Thoracotoı	249	PRIVE	No	69	0	0

0	0	Thoracotoı	249	PRIVE	No	76	0	1
0	0	VATS or rc	249	PRIVE	No	61	0	0
0	0	Thoracotoı	249	PRIVE	No	61	0	0
0	0	VATS or rc	249	PRIVE	No	66	1	0
0	0	Thoracotoı	249	PRIVE	No	62	0	0
0	1	Thoracotoı	249	PRIVE	No	63	0	0
0	0	Thoracotoı	249	PRIVE	No	26	0	0
0	0	Thoracotoı	249	PRIVE	No	69	0	1
0	0	Thoracotoı	249	PRIVE	No	69	0	0
0	1	Thoracotoı	249	PRIVE	No	74	1	0
0	0	Thoracotoı	249	PRIVE	No	66	0	0
0	0	VATS or rc	249	PRIVE	No	48	0	1
0	0	Thoracotoı	249	PRIVE	No	59	0	0
0	0	Thoracotoı	249	PRIVE	No	61	0	1
0	0	Thoracotoı	249	PRIVE	No	57	0	0
0	0	Thoracotoı	249	PRIVE	No	83	0	0
0	0	Thoracotoı	249	PRIVE	No	72	0	0
0	0	Thoracotoı	249	PRIVE	No	77	0	0
0	0	Thoracotoı	249	PRIVE	No	52	0	1
0	0	VATS or rc	249	PRIVE	No	53	0	1
0	0	Thoracotoı	249	PRIVE	No	68	0	0
0	1	Thoracotoı	249	PRIVE	No	54	0	1
0	0	Thoracotoı	249	PRIVE	No	64	0	0
0	0	Thoracotoı	249	PRIVE	No	63	0	0
0	0	VATS or rc	249	PRIVE	No	60	0	1
0	0	Thoracotoı	249	PRIVE	No	65	0	0
0	0	VATS or rc	249	PRIVE	No	70	1	0
0	1	Thoracotoı	249	PRIVE	No	65	0	0
0	0	Thoracotoı	249	PRIVE	No	65	1	0
0	0	Thoracotoı	249	PRIVE	No	73	0	0
0	0	VATS or rc	249	PRIVE	No	57	0	0
0	0	Thoracotoı	249	PRIVE	No	59	0	0
0	0	Thoracotoı	249	PRIVE	No	74	0	0
0	0	Thoracotoı	249	PRIVE	No	74	0	0
0	0	VATS or rc	249	PRIVE	No	61	0	1
0	0	Thoracotoı	249	PRIVE	No	78	1	0
0	1	Thoracotoı	249	PRIVE	No	64	0	0
0	0	Thoracotoı	249	PRIVE	No	77	0	0
0	0	VATS or rc	249	PRIVE	No	63	0	1
0	0	Thoracotoı	249	PRIVE	No	79	0	0
0	0	Thoracotoı	249	PRIVE	No	49	0	0
0	0	Thoracotoı	249	PRIVE	No	82	0	0
0	0	VATS or rc	249	PRIVE	No	66	0	0
0	0	Thoracotoı	249	PRIVE	No	66	0	0
0	1	Thoracotoı	249	PRIVE	No	60	0	1
0	0	VATS or rc	249	PRIVE	No	71	0	1
0	1	Thoracotoı	249	PRIVE	No	64	0	0
0	1	Thoracotoı	249	PRIVE	No	58	0	0
0	1	Thoracotoı	249	PRIVE	No	55	0	1
0	0	VATS or rc	249	PRIVE	No	68	0	1
0	0	Thoracotoı	249	PRIVE	No	75	0	0
0	0	Thoracotoı	249	PRIVE	No	79	1	0
0	0	VATS or rc	249	PRIVE	No	71	1	1
0	0	VATS or rc	249	PRIVE	No	67	0	1
0	0	Thoracotoı	249	PRIVE	No	70	0	0
0	0	Thoracotoı	249	PRIVE	No	64	0	0

0	0	VATS or rc	249	PRIVE	No	79	0	1
0	0	VATS or rc	249	PRIVE	No	66	0	0
0	0	Thoracotoı	249	PRIVE	No	48	0	0
0	0	Thoracotoı	249	PRIVE	No	49	0	1
0	0	Thoracotoı	249	PRIVE	No	63	0	0
0	1	Thoracotoı	249	PRIVE	No	55	0	0
0	1	Thoracotoı	249	PRIVE	No	71	0	0
0	0	Thoracotoı	249	PRIVE	No	55	0	0
0	0	Thoracotoı	249	PRIVE	No	80	0	0
0	0	VATS or rc	249	PRIVE	No	61	0	1
0	0	Thoracotoı	249	PRIVE	Yes	66	1	0
0	0	VATS or rc	249	PRIVE	No	38	0	0
0	0	Thoracotoı	249	PRIVE	No	54	0	0
0	0	Thoracotoı	249	PRIVE	No	65	0	0
0	0	VATS or rc	249	PRIVE	No	73	0	0
0	0	VATS or rc	249	PRIVE	No	56	0	1
0	0	Thoracotoı	249	PRIVE	No	58	0	1
0	0	Thoracotoı	249	PRIVE	No	61	0	1
0	0	Thoracotoı	249	PRIVE	No	41	0	1
0	0	Thoracotoı	249	PRIVE	No	53	0	1
0	0	VATS or rc	249	PRIVE	No	67	0	0
0	0	Thoracotoı	249	PRIVE	No	53	0	0
0	0	VATS or rc	249	PRIVE	No	68	0	1
0	0	Thoracotoı	249	PRIVE	No	57	0	0
0	0	Thoracotoı	249	PRIVE	No	64	0	0
0	0	Thoracotoı	249	PRIVE	No	61	0	1
0	0	Thoracotoı	249	PRIVE	No	62	0	0
0	1	Thoracotoı	249	PRIVE	No	74	0	0
0	0	Thoracotoı	249	PRIVE	No	77	1	0
0	0	Thoracotoı	249	PRIVE	No	77	0	0
0	0	Thoracotoı	249	PRIVE	No	69	0	0
0	0	Thoracotoı	249	PRIVE	No	59	0	0
0	0	Thoracotoı	249	PRIVE	No	37	0	0
0	0	Thoracotoı	249	PRIVE	No	58	0	0
0	0	Thoracotoı	249	PRIVE	No	60	0	1
0	0	VATS or rc	249	PRIVE	No	71	0	0
0	0	Thoracotoı	249	PRIVE	No	63	0	0
0	1	Thoracotoı	249	PRIVE	No	46	0	0
0	0	VATS or rc	249	PRIVE	No	65	0	0
0	0	Thoracotoı	249	PRIVE	No	64	1	0
0	0	Thoracotoı	249	PRIVE	No	60	0	0
0	1	Thoracotoı	249	PRIVE	No	68	0	0
0	0	Thoracotoı	249	PRIVE	No	67	0	1
0	0	VATS or rc	249	PRIVE	No	62	1	0
0	0	Thoracotoı	249	PRIVE	No	73	0	0
0	0	Thoracotoı	249	PRIVE	No	58	0	0
0	0	Thoracotoı	249	PRIVE	No	40	0	0
0	0	Thoracotoı	249	PRIVE	No	56	0	1
0	0	Thoracotoı	249	PRIVE	No	56	0	0
0	1	Thoracotoı	249	PRIVE	No	71	0	0
0	0	Thoracotoı	249	PRIVE	No	60	1	0
0	0	VATS or rc	249	PRIVE	No	70	0	0
0	0	Thoracotoı	249	PRIVE	No	62	0	1
0	0	Thoracotoı	249	PRIVE	No	58	0	0
0	0	Thoracotoı	249	PRIVE	No	56	0	0
0	0	Thoracotoı	249	PRIVE	No	58	0	1

0	0	Thoracotoı	249	PRIVE	No	76	0	0
0	1	Thoracotoı	249	PRIVE	No	60	0	0
0	0	VATS or rc	249	PRIVE	No	76	0	0
0	0	Thoracotoı	249	PRIVE	No	65	0	0
0	0	Thoracotoı	249	PRIVE	No	76	0	0
0	0	Thoracotoı	249	PRIVE	No	56	0	1
0	0	Thoracotoı	249	PRIVE	No	57	0	0
0	1	Thoracotoı	249	PRIVE	No	46	0	0
0	0	VATS or rc	249	PRIVE	No	74	1	1
0	0	VATS or rc	249	PRIVE	No	63	0	0
0	0	Thoracotoı	249	PRIVE	No	49	0	0
0	0	Thoracotoı	249	PRIVE	No	73	0	0
0	0	Thoracotoı	249	PRIVE	No	74	0	0
0	0	Thoracotoı	249	PRIVE	No	64	0	0
0	0	VATS or rc	249	PRIVE	No	56	0	0
0	0	Thoracotoı	249	PRIVE	No	63	0	0
0	0	VATS or rc	249	PRIVE	No	71	1	0
0	0	Thoracotoı	249	PRIVE	No	63	0	0
0	0	VATS or rc	249	PRIVE	No	71	1	0
0	0	Thoracotoı	249	PRIVE	No	64	0	0
0	0	Thoracotoı	249	PRIVE	No	54	0	0
0	0	Thoracotoı	249	PRIVE	No	69	1	0
0	1	Thoracotoı	249	PRIVE	No	53	0	0
0	0	Thoracotoı	249	PRIVE	No	81	0	0
0	0	VATS or rc	249	PRIVE	No	76	0	0
0	0	Thoracotoı	249	PRIVE	No	57	0	1
0	0	VATS or rc	249	PRIVE	No	62	0	0
0	1	Thoracotoı	249	PRIVE	No	47	0	0
0	0	VATS or rc	249	PRIVE	No	69	0	0
0	0	Thoracotoı	249	PRIVE	No	39	0	1
0	0	VATS or rc	249	PRIVE	No	61	0	0
0	1	Thoracotoı	249	PRIVE	No	55	0	0
0	0	Thoracotoı	249	PRIVE	No	65	0	0
0	0	Thoracotoı	249	PRIVE	No	55	0	0
0	1	Thoracotoı	249	PRIVE	Yes	62	1	0
0	1	Thoracotoı	249	PRIVE	No	50	0	0
0	0	Thoracotoı	249	PRIVE	Yes	66	0	1
0	0	Thoracotoı	249	PRIVE	No	55	0	0
0	0	Thoracotoı	249	PRIVE	No	65	0	0
0	0	Thoracotoı	249	PRIVE	No	65	0	0
0	0	Thoracotoı	249	PRIVE	No	61	0	0
0	0	VATS or rc	249	PRIVE	No	76	0	0
0	1	Thoracotoı	249	PRIVE	No	58	0	0
0	0	Thoracotoı	249	PRIVE	No	49	0	0
0	0	Thoracotoı	249	PRIVE	No	66	0	0
0	0	Thoracotoı	249	PRIVE	No	71	0	0
0	0	Thoracotoı	249	PRIVE	No	78	0	0
0	0	Thoracotoı	249	PRIVE	No	56	0	0
0	0	VATS or rc	249	PRIVE	No	63	0	0
0	0	VATS or rc	249	PRIVE	No	71	1	0
0	1	Thoracotoı	249	PRIVE	No	72	0	0
0	0	Thoracotoı	249	PRIVE	No	61	0	0
0	0	Thoracotoı	249	PRIVE	No	63	0	0
0	0	VATS or rc	249	PRIVE	No	66	0	1
0	0	VATS or rc	249	PRIVE	No	66	0	0
0	0	VATS or rc	249	PRIVE	No	52	0	0

0	0	Thoracotoı	249	PRIVE	No	65	0	0
0	1	Thoracotoı	249	PRIVE	No	75	0	0
0	0	Thoracotoı	249	PRIVE	No	60	0	0
0	0	Thoracotoı	249	PRIVE	No	64	0	0
0	1	Thoracotoı	249	PRIVE	No	64	0	0
0	0	VATS or rc	249	PRIVE	No	71	0	0
0	1	Thoracotoı	249	PRIVE	No	79	0	0
0	0	VATS or rc	249	PRIVE	No	60	0	1
0	0	Thoracotoı	249	PRIVE	No	44	0	0
0	0	VATS or rc	249	PRIVE	No	70	0	0
0	0	VATS or rc	249	PRIVE	No	70	0	0
0	0	Thoracotoı	249	PRIVE	No	56	0	0
0	1	VATS or rc	249	PRIVE	No	66	0	0
0	1	Thoracotoı	249	PRIVE	No	47	0	0
0	0	VATS or rc	249	PRIVE	No	75	0	0
0	0	Thoracotoı	249	PRIVE	No	76	0	0
0	0	Thoracotoı	249	PRIVE	Yes	70	0	0
0	0	Thoracotoı	249	PRIVE	No	58	0	1
0	0	Thoracotoı	249	PRIVE	No	40	0	0
0	0	Thoracotoı	249	PRIVE	No	54	0	1
0	0	Thoracotoı	249	PRIVE	No	68	1	0
0	0	Thoracotoı	249	PRIVE	No	62	0	1
0	0	Thoracotoı	249	PRIVE	No	76	0	0
0	1	Thoracotoı	249	PRIVE	No	67	0	0
0	0	Thoracotoı	249	PRIVE	No	61	0	0
0	0	VATS or rc	249	PRIVE	No	63	0	0
0	0	Thoracotoı	249	PRIVE	No	52	0	0
0	0	Thoracotoı	249	PRIVE	No	74	0	0
0	0	Thoracotoı	249	PRIVE	No	40	0	0
0	0	Thoracotoı	249	PRIVE	No	69	1	0
0	0	Thoracotoı	249	PRIVE	No	57	0	0
0	0	Thoracotoı	249	PRIVE	No	59	0	1
0	0	Thoracotoı	249	PRIVE	No	69	0	1
0	0	Thoracotoı	249	PRIVE	No	66	0	0
0	0	Thoracotoı	249	PRIVE	No	77	0	0
0	0	Thoracotoı	249	PRIVE	No	74	0	0
0	0	Thoracotoı	249	PRIVE	No	60	0	0
0	1	Thoracotoı	249	PRIVE	No	67	0	0
0	0	Thoracotoı	249	PRIVE	No	66	0	0
0	0	Thoracotoı	249	PRIVE	No	64	0	1
0	0	VATS or rc	249	PRIVE	No	80	0	0
0	0	Thoracotoı	249	PRIVE	No	54	0	1
0	0	Thoracotoı	249	PRIVE	No	41	0	0
0	1	Thoracotoı	249	PRIVE	Yes	54	0	0
0	0	VATS or rc	249	PRIVE	No	67	0	1
0	0	Thoracotoı	249	PRIVE	Yes	79	0	0
0	0	Thoracotoı	249	PRIVE	No	84	1	0
0	0	Thoracotoı	249	PRIVE	No	59	0	0
0	1	Thoracotoı	249	PRIVE	No	77	0	0
0	0	Thoracotoı	249	PRIVE	No	76	1	0
0	0	Thoracotoı	249	PRIVE	No	65	0	0
0	0	Thoracotoı	249	PRIVE	No	63	0	0
0	0	Thoracotoı	249	PRIVE	No	54	0	0
0	0	Thoracotoı	249	PRIVE	No	57	0	0
0	0	VATS or rc	249	PRIVE	No	67	0	1
0	0	Thoracotoı	249	PRIVE	No	64	0	0

0	1	Thoracotoı	249	PRIVE	No	65	1	0
0	0	VATS or rc	249	PRIVE	No	64	0	0
0	0	Thoracotoı	249	PRIVE	No	56	0	0
0	0	Thoracotoı	249	PRIVE	No	52	0	0
0	0	Thoracotoı	249	PRIVE	No	60	0	1
0	0	Thoracotoı	249	PRIVE	No	59	0	0
0	0	VATS or rc	249	PRIVE	No	78	0	0
0	0	Thoracotoı	249	PRIVE	No	66	0	0
0	0	VATS or rc	249	PRIVE	No	60	0	0
0	0	Thoracotoı	249	PRIVE	No	63	0	0
0	0	Thoracotoı	249	PRIVE	No	69	0	0
0	1	Thoracotoı	249	PRIVE	No	59	0	0
0	0	Thoracotoı	249	PRIVE	No	58	0	1
0	0	Thoracotoı	249	PRIVE	No	53	0	0
0	0	Thoracotoı	249	PRIVE	No	69	0	0
0	0	Thoracotoı	249	PRIVE	No	67	0	0
0	1	Thoracotoı	249	PRIVE	No	57	0	0
0	0	VATS or rc	249	PRIVE	No	62	0	0
0	0	Thoracotoı	249	PRIVE	No	62	0	0
0	0	Thoracotoı	249	PRIVE	No	70	0	0
0	0	Thoracotoı	249	PRIVE	No	67	0	0
0	0	Thoracotoı	249	PRIVE	No	45	0	0
0	0	Thoracotoı	249	PRIVE	No	53	0	0
0	1	Thoracotoı	249	PRIVE	No	59	0	0
0	0	Thoracotoı	249	PRIVE	No	42	0	0
0	0	Thoracotoı	249	PRIVE	No	58	0	0
0	0	Thoracotoı	249	PRIVE	No	62	0	0
0	0	VATS or rc	249	PRIVE	No	71	0	0
0	0	Thoracotoı	249	PRIVE	No	66	0	0
0	0	Thoracotoı	249	PRIVE	No	56	0	1
0	0	Thoracotoı	249	PRIVE	Yes	64	0	0
0	1	Thoracotoı	249	PRIVE	No	76	0	0
0	0	Thoracotoı	249	PRIVE	No	61	0	1
0	0	Thoracotoı	249	PRIVE	No	56	0	0
0	0	Thoracotoı	249	PRIVE	No	60	0	1
0	0	VATS or rc	249	PRIVE	No	62	0	1
0	1	Thoracotoı	249	PRIVE	No	52	0	1
0	0	Thoracotoı	249	PRIVE	No	65	0	0
0	0	Thoracotoı	249	PRIVE	No	60	0	0
0	1	Thoracotoı	249	PRIVE	No	61	0	0
0	0	VATS or rc	249	PRIVE	No	48	0	0
0	1	Thoracotoı	249	PRIVE	No	61	1	0
0	0	Thoracotoı	249	PRIVE	No	61	0	1
0	0	Thoracotoı	249	PRIVE	No	72	0	0
0	0	Thoracotoı	249	PRIVE	No	53	0	1
0	0	Thoracotoı	249	PRIVE	No	65	0	0
0	1	Thoracotoı	249	PRIVE	No	64	1	0
0	0	Thoracotoı	249	PRIVE	No	65	0	0
0	1	Thoracotoı	249	PRIVE	No	55	0	0
0	0	VATS or rc	249	PRIVE	No	71	0	0
0	0	Thoracotoı	249	PRIVE	No	59	0	0
0	0	Thoracotoı	249	PRIVE	No	57	0	0
0	1	Thoracotoı	249	PRIVE	No	63	0	0
0	0	VATS or rc	249	PRIVE	No	47	0	0
0	0	Thoracotoı	249	PRIVE	No	76	0	0
0	1	Thoracotoı	249	PRIVE	No	50	0	0

0	0	Thoracotoı	249	PRIVE	No	64	0	0
0	0	Thoracotoı	249	PRIVE	No	80	0	0
0	0	Thoracotoı	249	PRIVE	No	68	0	0
0	0	Thoracotoı	249	PRIVE	No	76	0	0
0	0	Thoracotoı	249	PRIVE	No	69	0	0
0	0	Thoracotoı	249	PRIVE	No	53	0	0
0	0	Thoracotoı	249	PRIVE	No	67	0	0
0	0	VATS or rc	249	PRIVE	No	72	0	0
0	0	Thoracotoı	249	PRIVE	No	58	0	1
0	0	Thoracotoı	249	PRIVE	No	75	0	0
0	0	Thoracotoı	249	PRIVE	No	77	0	0
0	0	Thoracotoı	249	PRIVE	No	79	1	0
0	0	Thoracotoı	249	PRIVE	No	74	0	0
0	0	VATS or rc	249	PRIVE	No	71	0	0
0	0	Thoracotoı	249	PRIVE	No	48	0	0
0	0	VATS or rc	249	PRIVE	No	70	0	1
0	0	VATS or rc	249	PRIVE	No	57	0	0
0	0	Thoracotoı	249	PRIVE	No	45	0	0
0	0	Thoracotoı	249	PRIVE	No	72	0	0
0	0	Thoracotoı	249	PRIVE	No	68	0	0
0	0	VATS or rc	249	PRIVE	No	55	0	1
0	0	VATS or rc	249	PRIVE	No	67	0	0
0	0	Thoracotoı	249	PRIVE	No	74	0	0
0	0	Thoracotoı	249	PRIVE	No	35	0	0
0	0	Thoracotoı	249	PRIVE	No	57	0	0
0	0	Thoracotoı	249	PRIVE	No	74	0	0
0	0	Thoracotoı	249	PRIVE	No	64	0	0
0	0	Thoracotoı	249	PRIVE	No	57	0	0
0	0	Thoracotoı	249	PRIVE	Yes	45	0	0
0	1	Thoracotoı	249	PRIVE	No	63	0	0
0	0	Thoracotoı	249	PRIVE	No	65	0	1
0	0	Thoracotoı	249	PRIVE	No	49	0	1
0	0	Thoracotoı	249	PRIVE	No	53	0	0
0	0	Thoracotoı	249	PRIVE	No	66	0	0
0	0	Thoracotoı	249	PRIVE	No	71	0	0
0	0	Thoracotoı	249	PRIVE	No	53	0	0
0	0	Thoracotoı	249	PRIVE	No	45	0	0
0	1	Thoracotoı	249	PRIVE	No	67	0	0
0	0	Thoracotoı	249	PRIVE	No	59	0	0
0	0	VATS or rc	249	PRIVE	No	60	0	0
0	0	Thoracotoı	249	PRIVE	No	80	0	0
0	0	VATS or rc	249	PRIVE	No	71	0	1
0	0	Thoracotoı	249	PRIVE	No	75	0	0
0	0	Thoracotoı	249	PRIVE	No	67	0	0
0	0	Thoracotoı	249	PRIVE	No	74	0	0
0	0	Thoracotoı	249	PRIVE	No	68	0	0
0	0	Thoracotoı	249	PRIVE	No	56	0	0
0	0	Thoracotoı	249	PRIVE	No	79	0	0
0	0	Thoracotoı	249	PRIVE	No	75	0	0
0	1	Thoracotoı	249	PRIVE	No	69	0	0
0	1	Thoracotoı	249	PRIVE	No	58	0	1
0	0	Thoracotoı	249	PRIVE	No	74	0	0
0	0	Thoracotoı	249	PRIVE	No	57	0	0
0	1	Thoracotoı	249	PRIVE	No	57	0	0
0	0	Thoracotoı	249	PRIVE	No	61	0	0
0	0	Thoracotoı	249	PRIVE	No	52	0	1

0	0	Thoracotoı	249	PRIVE	No	61	0	0
0	0	VATS or rc	249	PRIVE	No	55	0	0
0	0	VATS or rc	249	PRIVE	No	81	0	0
0	0	Thoracotoı	249	PRIVE	No	49	0	0
0	0	Thoracotoı	249	PRIVE	No	57	0	0
0	0	VATS or rc	249	PRIVE	No	65	0	0
0	0	VATS or rc	249	PRIVE	No	70	0	0
0	0	VATS or rc	249	PRIVE	No	81	1	0
0	0	Thoracotoı	249	PRIVE	No	72	0	0
0	0	Thoracotoı	249	PRIVE	No	54	0	0
0	1	Thoracotoı	249	PRIVE	No	61	0	0
0	0	Thoracotoı	249	PRIVE	No	73	0	0
0	0	Thoracotoı	249	PRIVE	No	67	0	0
0	0	VATS or rc	249	PRIVE	No	56	0	1
0	0	VATS or rc	249	PRIVE	No	56	0	0
0	0	Thoracotoı	249	PRIVE	No	72	0	0
0	0	Thoracotoı	249	PRIVE	No	73	0	0
0	0	Thoracotoı	249	PRIVE	No	71	0	0
0	0	Thoracotoı	249	PRIVE	No	74	0	0
0	0	Thoracotoı	249	PRIVE	No	53	0	1
0	0	Thoracotoı	249	PRIVE	No	66	0	0
0	0	Thoracotoı	249	PRIVE	No	56	0	1
0	0	Thoracotoı	249	PRIVE	No	77	0	0
0	0	VATS or rc	249	PRIVE	No	50	0	0
0	0	Thoracotoı	249	PRIVE	No	57	0	0
0	0	VATS or rc	249	PRIVE	No	62	0	0
0	0	VATS or rc	249	PRIVE	No	66	0	0
0	0	VATS or rc	249	PRIVE	Yes	72	1	0
0	1	Thoracotoı	249	PRIVE	No	47	0	1
0	0	Thoracotoı	249	PRIVE	No	60	0	0
0	0	Thoracotoı	249	PRIVE	No	68	0	0
0	1	Thoracotoı	249	PRIVE	No	52	0	0
0	0	Thoracotoı	249	PRIVE	No	68	0	0
0	0	Thoracotoı	249	PRIVE	No	57	1	0
0	0	Thoracotoı	249	PRIVE	No	59	0	0
0	0	Thoracotoı	249	PRIVE	No	72	0	0
0	1	Thoracotoı	249	PRIVE	No	75	0	0
0	0	Thoracotoı	249	PRIVE	No	67	1	0
0	0	Thoracotoı	249	PRIVE	No	57	0	0
0	0	Thoracotoı	249	PRIVE	No	54	0	0
0	0	Thoracotoı	249	PRIVE	No	72	0	1
0	0	Thoracotoı	249	PRIVE	No	71	0	0
0	0	VATS or rc	249	PRIVE	No	46	0	0
0	0	Thoracotoı	249	PRIVE	No	53	0	0
0	0	Thoracotoı	249	PRIVE	No	76	1	0
0	1	Thoracotoı	249	PRIVE	No	59	0	0
0	1	Thoracotoı	249	PRIVE	No	79	0	0
0	0	Thoracotoı	249	PRIVE	No	68	1	0
0	0	Thoracotoı	249	PRIVE	No	56	0	0
0	1	Thoracotoı	249	PRIVE	No	74	0	1
0	1	Thoracotoı	249	PRIVE	No	78	0	0
0	1	Thoracotoı	249	PRIVE	No	62	0	1
0	0	VATS or rc	249	PRIVE	No	67	0	1
0	0	Thoracotoı	249	PRIVE	No	61	0	0
0	0	VATS or rc	249	PRIVE	No	64	0	1
0	0	Thoracotoı	249	PRIVE	No	70	0	0

0	1	Thoracotoı	249	PRIVE	No	54	0	0
0	0	Thoracotoı	249	PRIVE	No	64	0	0
0	0	Thoracotoı	249	PRIVE	No	50	0	0
0	0	Thoracotoı	249	PRIVE	No	70	0	0
0	0	VATS or rc	249	PRIVE	No	54	0	1
0	0	Thoracotoı	249	PRIVE	No	53	0	0
0	1	Thoracotoı	249	PRIVE	No	61	0	0
0	1	Thoracotoı	249	PRIVE	No	55	0	1
0	0	Thoracotoı	249	PRIVE	No	70	0	0
0	0	VATS or rc	249	PRIVE	No	72	0	0
0	0	Thoracotoı	249	PRIVE	No	64	0	0
0	0	Thoracotoı	249	PRIVE	No	56	0	0
0	0	Thoracotoı	249	PRIVE	No	74	0	0
0	0	Thoracotoı	249	PRIVE	No	68	0	1
0	0	Thoracotoı	249	PRIVE	No	70	0	1
0	0	Thoracotoı	249	PRIVE	No	51	0	0
0	0	VATS or rc	249	PRIVE	No	71	0	0
0	0	Thoracotoı	249	PRIVE	No	57	0	0
0	0	VATS or rc	249	PRIVE	No	53	0	1
0	0	Thoracotoı	249	PRIVE	No	65	0	0
0	1	Thoracotoı	249	PRIVE	No	63	0	0
0	0	Thoracotoı	249	PRIVE	No	57	0	0
0	0	Thoracotoı	249	PRIVE	No	67	0	0
0	0	Thoracotoı	249	PRIVE	Yes	44	0	0
0	1	Thoracotoı	249	PRIVE	No	56	0	0
0	0	Thoracotoı	249	PRIVE	No	51	0	1
0	0	VATS or rc	249	PRIVE	No	70	0	0
0	0	VATS or rc	249	PRIVE	No	61	0	0
0	0	Thoracotoı	249	PRIVE	No	74	0	0
0	1	Thoracotoı	249	PRIVE	No	66	0	0
0	0	Thoracotoı	249	PRIVE	No	65	0	0
0	0	Thoracotoı	249	PRIVE	No	66	0	0
0	0	Thoracotoı	249	PRIVE	No	55	0	0
0	0	Thoracotoı	249	PRIVE	No	64	0	0
0	0	Thoracotoı	249	PRIVE	No	64	0	0
0	0	Thoracotoı	249	PRIVE	Yes	55	0	0
0	0	Thoracotoı	249	PRIVE	No	60	0	0
0	0	VATS or rc	249	PRIVE	No	53	0	0
0	0	VATS or rc	249	PRIVE	No	64	0	0
0	0	Thoracotoı	249	PRIVE	No	58	0	0
0	0	Thoracotoı	249	PRIVE	No	46	0	0
0	0	VATS or rc	249	PRIVE	No	59	0	0
0	0	VATS or rc	249	PRIVE	No	42	0	0
0	0	Thoracotoı	249	PRIVE	No	72	0	0
0	0	VATS or rc	249	PRIVE	No	74	0	0
0	0	Thoracotoı	249	PRIVE	No	78	0	0
0	0	Thoracotoı	249	PRIVE	No	73	0	0
0	0	VATS or rc	249	PRIVE	No	63	0	1
0	0	Thoracotoı	249	PRIVE	No	62	0	0
0	1	Thoracotoı	249	PRIVE	No	74	1	1
0	1	Thoracotoı	249	PRIVE	No	72	0	0
0	0	Thoracotoı	249	PRIVE	No	70	1	0
0	0	Thoracotoı	249	PRIVE	No	55	0	0
0	0	Thoracotoı	249	PRIVE	No	60	0	0
0	0	Thoracotoı	249	PRIVE	No	61	0	0
0	0	Thoracotoı	249	PRIVE	No	53	0	0

0	0	Thoracotoı	249	PRIVE	No	74	0	0
0	0	Thoracotoı	249	PRIVE	No	64	0	0
0	0	VATS or rc	249	PRIVE	No	62	0	1
0	0	Thoracotoı	249	PRIVE	No	38	0	0
0	0	Thoracotoı	249	PRIVE	No	80	0	0
0	1	Thoracotoı	249	PRIVE	No	46	0	0
0	0	Thoracotoı	249	PRIVE	No	49	0	0
0	0	Thoracotoı	249	PRIVE	No	57	0	0
0	0	Thoracotoı	249	PRIVE	No	63	0	1
0	0	VATS or rc	249	PRIVE	No	70	0	0
0	0	Thoracotoı	249	PRIVE	No	76	0	1
0	0	Thoracotoı	249	PRIVE	No	45	0	1
0	1	Thoracotoı	249	PRIVE	No	55	0	0
0	0	Thoracotoı	249	PRIVE	No	71	0	1
0	0	VATS or rc	249	PRIVE	No	57	0	0
0	0	Thoracotoı	249	PRIVE	No	63	0	0
0	0	Thoracotoı	249	PRIVE	No	62	0	0
0	1	Thoracotoı	249	PRIVE	No	71	0	0
0	0	VATS or rc	249	PRIVE	No	72	0	1
0	0	Thoracotoı	249	PRIVE	No	73	0	0
0	0	Thoracotoı	249	PRIVE	No	59	0	1
0	0	Thoracotoı	249	PRIVE	No	57	0	0
0	0	Thoracotoı	249	PRIVE	No	56	0	0
0	0	Thoracotoı	249	PRIVE	No	57	0	1
0	0	Thoracotoı	249	PRIVE	No	62	0	0
0	1	Thoracotoı	249	PRIVE	No	70	0	0
0	1	Thoracotoı	249	PRIVE	No	74	0	0
0	1	Thoracotoı	249	PRIVE	No	69	0	1
0	0	VATS or rc	249	PRIVE	No	67	0	0
0	0	Thoracotoı	249	PRIVE	No	55	0	0
0	0	Thoracotoı	249	PRIVE	No	62	0	0
0	0	VATS or rc	249	PRIVE	No	60	0	0
0	0	Thoracotoı	249	PRIVE	No	69	0	1
0	0	Thoracotoı	249	PRIVE	No	62	0	0
0	0	Thoracotoı	249	PRIVE	No	61	0	0
0	1	Thoracotoı	249	PRIVE	No	61	0	0
0	0	Thoracotoı	249	PRIVE	No	74	1	0
0	0	VATS or rc	249	PRIVE	No	67	0	0
0	0	Thoracotoı	249	PRIVE	No	53	0	0
0	0	Thoracotoı	249	PRIVE	No	71	0	0
0	1	Thoracotoı	249	PRIVE	No	74	0	0
0	0	Thoracotoı	249	PRIVE	No	57	0	0
0	0	Thoracotoı	249	PRIVE	No	56	0	0
0	0	Thoracotoı	249	PRIVE	No	86	0	0
0	0	Thoracotoı	249	PRIVE	No	65	0	0
0	0	Thoracotoı	249	PRIVE	No	67	0	0
0	1	Thoracotoı	249	PRIVE	No	80	0	0
0	0	Thoracotoı	249	PRIVE	No	76	0	0
0	0	Thoracotoı	249	PRIVE	No	62	0	0
0	0	Thoracotoı	249	PRIVE	No	48	0	0
0	0	VATS or rc	249	PRIVE	No	49	0	0
0	0	VATS or rc	249	PRIVE	No	72	0	1
0	0	VATS or rc	249	PRIVE	No	57	0	0
0	0	VATS or rc	249	PRIVE	No	69	0	0
0	0	VATS or rc	249	PRIVE	No	69	0	0
0	0	Thoracotoı	249	PRIVE	No	59	0	1

0	0	Thoracotoı	249	PRIVE	No	59	0	1
0	0	Thoracotoı	249	PRIVE	No	73	0	0
0	1	Thoracotoı	249	PRIVE	No	47	0	0
0	0	VATS or rc	249	PRIVE	No	52	0	0
0	0	Thoracotoı	249	PRIVE	No	45	0	1
0	0	VATS or rc	249	PRIVE	No	66	0	0
0	0	Thoracotoı	249	PRIVE	No	73	0	0
0	0	Thoracotoı	249	PRIVE	No	59	0	0
0	0	Thoracotoı	249	PRIVE	No	57	0	0
0	1	Thoracotoı	249	PRIVE	No	58	0	0
0	0	VATS or rc	249	PRIVE	No	69	0	1
0	0	Thoracotoı	249	PRIVE	No	68	0	0
0	0	VATS or rc	249	PRIVE	No	50	0	1
0	0	Thoracotoı	249	PRIVE	No	57	0	1
0	0	VATS or rc	249	PRIVE	No	68	0	0
0	0	Thoracotoı	249	PRIVE	No	75	0	0
0	0	Thoracotoı	249	PRIVE	No	66	0	0
0	0	Thoracotoı	249	PRIVE	No	65	0	1
0	0	VATS or rc	249	PRIVE	No	60	0	1
0	0	Thoracotoı	249	PRIVE	No	63	0	0
0	0	Thoracotoı	249	PRIVE	No	67	1	0
0	0	Thoracotoı	249	PRIVE	No	54	0	0
0	1	Thoracotoı	249	PRIVE	No	74	0	0
0	1	Thoracotoı	249	PRIVE	Yes	71	0	0
0	0	VATS or rc	249	PRIVE	No	69	1	0
0	0	VATS or rc	249	PRIVE	No	75	0	0
0	0	Thoracotoı	249	PRIVE	No	59	0	1
0	0	Thoracotoı	249	PRIVE	No	73	1	0
0	1	Thoracotoı	249	PRIVE	No	71	0	0
0	0	Thoracotoı	249	PRIVE	No	59	0	1
0	0	VATS or rc	249	PRIVE	No	51	0	0
0	0	VATS or rc	249	PRIVE	No	54	0	1
0	0	VATS or rc	249	PRIVE	No	65	0	0
0	0	Thoracotoı	249	PRIVE	No	61	0	0
0	0	Thoracotoı	249	PRIVE	No	57	0	0
0	0	Thoracotoı	249	PRIVE	No	66	0	0
0	1	Thoracotoı	249	PRIVE	Yes	58	0	1
0	0	Thoracotoı	249	PRIVE	No	70	0	0
0	0	Thoracotoı	249	PRIVE	No	51	0	0
0	1	Thoracotoı	249	PRIVE	No	55	1	0
0	0	Thoracotoı	249	PRIVE	No	51	0	0
0	0	Thoracotoı	249	PRIVE	No	52	0	0
0	0	VATS or rc	249	PRIVE	No	68	0	0
0	0	VATS or rc	249	PRIVE	No	66	0	0
0	1	Thoracotoı	249	PRIVE	No	53	0	0
0	0	Thoracotoı	249	PRIVE	No	65	0	0
0	0	VATS or rc	249	PRIVE	No	74	0	0
0	0	Thoracotoı	249	PRIVE	No	79	0	0
0	0	VATS or rc	249	PRIVE	No	65	0	0
0	0	Thoracotoı	249	PRIVE	No	52	0	1
0	0	VATS or rc	249	PRIVE	No	70	0	0
0	0	Thoracotoı	249	PRIVE	No	77	0	0
0	0	Thoracotoı	249	PRIVE	No	74	0	0
0	0	VATS or rc	249	PRIVE	No	65	1	0
0	0	VATS or rc	249	PRIVE	No	72	0	0
0	0	Thoracotoı	249	PRIVE	No	57	0	0

0	0	Thoracotor	249	PRIVE	No	67	0	0
0	0	Thoracotor	249	PRIVE	No	55	0	0
0	0	Thoracotor	249	PRIVE	No	57	0	0
0	1	Thoracotor	249	PRIVE	No	71	0	0
0	0	VATS or rc	249	PRIVE	No	69	0	0
0	0	Thoracotor	249	PRIVE	No	60	0	0
0	0	Thoracotor	249	PRIVE	No	63	0	0
0	0	VATS or rc	249	PRIVE	No	63	0	0
0	0	Thoracotor	249	PRIVE	No	55	0	0
0	0	Thoracotor	249	PRIVE	No	65	0	0
0	0	Thoracotor	249	PRIVE	No	65	0	0
0	0	Thoracotor	249	PRIVE	No	53	0	0
0	0	VATS or rc	249	PRIVE	No	74	0	0
0	0	Thoracotor	249	PRIVE	No	60	0	0
0	0	Thoracotor	249	PRIVE	No	57	0	0
0	0	Thoracotor	249	PRIVE	No	65	0	0
0	0	Thoracotor	249	PRIVE	No	78	0	0
0	0	Thoracotor	249	PRIVE	No	64	0	1
0	0	Thoracotor	249	PRIVE	No	55	0	0
0	0	Thoracotor	249	PRIVE	No	64	0	0
0	0	Thoracotor	249	PRIVE	No	55	0	0
0	0	Thoracotor	249	PRIVE	No	75	0	0
0	0	VATS or rc	249	PRIVE	No	54	0	0
0	0	VATS or rc	249	PRIVE	No	52	0	0
0	1	Thoracotor	249	PRIVE	No	61	0	1
0	0	Thoracotor	249	PRIVE	No	73	0	0
0	0	Thoracotor	249	PRIVE	No	75	0	1
0	1	Thoracotor	249	PRIVE	No	60	0	1
0	1	Thoracotor	249	PRIVE	No	73	1	0
0	1	Thoracotor	249	PRIVE	No	55	0	0
0	0	Thoracotor	249	PRIVE	No	64	1	0
0	0	Thoracotor	249	PRIVE	No	76	0	1
0	0	VATS or rc	249	PRIVE	No	69	0	0
0	0	Thoracotor	249	PRIVE	No	64	0	0
0	0	Thoracotor	249	PRIVE	No	69	0	0
0	0	Thoracotor	249	PRIVE	No	67	0	0
0	1	Thoracotor	249	PRIVE	No	69	0	0
0	0	Thoracotor	249	PRIVE	No	57	0	0
0	0	Thoracotor	249	PRIVE	Yes	71	0	0
0	1	Thoracotor	249	PRIVE	No	63	0	1
0	0	Thoracotor	249	PRIVE	No	69	0	0
0	1	Thoracotor	249	PRIVE	No	62	0	0
0	0	Thoracotor	249	PRIVE	Yes	72	1	0
0	0	Thoracotor	249	PRIVE	No	80	0	1
0	0	VATS or rc	249	PRIVE	No	72	1	0
0	0	Thoracotor	249	PRIVE	No	69	1	0
0	0	VATS or rc	249	PRIVE	No	66	0	0
0	1	Thoracotor	249	PRIVE	No	76	0	0
0	0	VATS or rc	249	PRIVE	No	77	0	0
0	0	Thoracotor	249	PRIVE	No	82	0	0
0	0	Thoracotor	249	PRIVE	No	67	0	0
0	0	Thoracotor	249	PRIVE	No	54	0	0
0	0	Thoracotor	249	PRIVE	No	61	0	0
0	0	Thoracotor	249	PRIVE	No	70	0	0
0	1	Thoracotor	249	PRIVE	No	69	1	0
0	0	VATS or rc	249	PRIVE	No	67	0	1

0	0	Thoracotoı	249	PRIVE	No	63	0	1
0	0	Thoracotoı	249	PRIVE	No	60	0	0
0	0	VATS or rc	249	PRIVE	No	67	0	0
0	1	Thoracotoı	249	PRIVE	No	73	0	0
0	0	Thoracotoı	249	PRIVE	No	72	0	0
0	0	Thoracotoı	249	PRIVE	No	56	0	0
0	0	Thoracotoı	249	PRIVE	No	60	0	1
0	0	Thoracotoı	249	PRIVE	No	61	0	0
0	0	Thoracotoı	249	PRIVE	No	59	0	0
0	0	VATS or rc	249	PRIVE	No	74	0	0
0	0	Thoracotoı	249	PRIVE	No	71	0	0
0	0	Thoracotoı	249	PRIVE	No	67	0	0
0	0	Thoracotoı	249	PRIVE	No	80	1	0
0	1	Thoracotoı	249	PRIVE	No	65	0	0
0	0	VATS or rc	249	PRIVE	No	72	0	1
0	0	Thoracotoı	249	PRIVE	No	22	0	0
0	1	Thoracotoı	249	PRIVE	No	76	0	0
0	0	Thoracotoı	249	PRIVE	No	64	0	0
0	0	Thoracotoı	249	PRIVE	No	79	0	0
0	0	VATS or rc	249	PRIVE	No	57	0	1
0	1	Thoracotoı	249	PRIVE	No	61	0	0
0	0	Thoracotoı	249	PRIVE	No	62	1	0
0	1	Thoracotoı	249	PRIVE	No	63	0	0
0	0	Thoracotoı	249	PRIVE	No	78	0	0
0	0	Thoracotoı	249	PRIVE	No	52	0	0
0	0	Thoracotoı	249	PRIVE	No	61	0	1
0	0	Thoracotoı	249	PRIVE	No	50	0	0
0	0	Thoracotoı	249	PRIVE	No	75	0	0
0	0	VATS or rc	249	PRIVE	No	58	0	0
0	0	VATS or rc	249	PRIVE	No	58	0	1
0	0	Thoracotoı	249	PRIVE	No	80	0	0
0	1	Thoracotoı	249	PRIVE	No	58	0	0
0	0	Thoracotoı	249	PRIVE	No	73	0	0
0	0	VATS or rc	249	PRIVE	No	67	0	1
0	1	Thoracotoı	249	PRIVE	No	63	0	0
0	0	Thoracotoı	249	PRIVE	No	80	0	0
0	0	Thoracotoı	249	PRIVE	No	63	0	0
0	0	Thoracotoı	249	PRIVE	No	74	0	0
0	0	Thoracotoı	249	PRIVE	No	63	0	0
0	0	Thoracotoı	249	PRIVE	No	66	0	0
0	0	Thoracotoı	249	PRIVE	No	49	0	0
0	0	Thoracotoı	249	PRIVE	No	64	0	0
0	0	Thoracotoı	249	PRIVE	No	65	0	0
0	0	Thoracotoı	249	PRIVE	No	56	0	0
0	0	VATS or rc	249	PRIVE	No	63	0	0
0	1	Thoracotoı	249	PRIVE	No	66	0	0
0	0	Thoracotoı	249	PRIVE	No	46	0	1
0	0	VATS or rc	249	PRIVE	No	54	0	0
0	0	Thoracotoı	249	PRIVE	No	65	0	0
0	0	Thoracotoı	249	PRIVE	No	66	0	1
0	1	Thoracotoı	249	PRIVE	No	58	0	0
0	1	Thoracotoı	249	PRIVE	No	65	1	0
0	0	Thoracotoı	249	PRIVE	No	65	1	1
0	0	Thoracotoı	249	PRIVE	No	75	0	0
0	0	Thoracotoı	249	PRIVE	No	70	0	0
0	0	Thoracotoı	249	PRIVE	No	56	0	0

0	0	Thoracotoı	249	PRIVE	No	58	0	0
0	0	Thoracotoı	249	PRIVE	No	52	0	0
0	0	VATS or rc	249	PRIVE	No	67	0	0
0	0	VATS or rc	249	PRIVE	No	53	0	0
0	0	Thoracotoı	249	PRIVE	No	51	0	0
0	0	Thoracotoı	249	PRIVE	No	75	0	0
0	1	Thoracotoı	249	PRIVE	No	78	0	0
0	0	Thoracotoı	249	PRIVE	No	62	0	0
0	0	VATS or rc	249	PRIVE	No	79	0	0
0	0	Thoracotoı	249	PRIVE	No	72	0	0
0	0	Thoracotoı	249	PRIVE	No	62	0	0
0	1	Thoracotoı	249	PRIVE	No	69	0	0
0	0	Thoracotoı	249	PRIVE	No	48	0	1
0	0	Thoracotoı	249	PRIVE	No	49	0	0
0	0	Thoracotoı	249	PRIVE	No	70	0	0
0	0	Thoracotoı	249	PRIVE	No	62	0	0
0	0	Thoracotoı	249	PRIVE	No	79	0	0
0	0	Thoracotoı	249	PRIVE	No	75	0	0
0	0	Thoracotoı	249	PRIVE	No	64	0	1
0	0	VATS or rc	249	PRIVE	No	44	0	1
0	0	VATS or rc	249	PRIVE	No	60	0	0
0	0	Thoracotoı	249	PRIVE	No	76	0	0
0	0	Thoracotoı	249	PRIVE	No	53	0	0
0	0	Thoracotoı	249	PRIVE	No	50	0	0
0	0	Thoracotoı	249	PRIVE	No	60	0	0
0	0	Thoracotoı	249	PRIVE	No	60	0	1
0	0	VATS or rc	249	PRIVE	No	72	0	0
0	0	Thoracotoı	249	PRIVE	No	76	0	0
0	0	Thoracotoı	249	PRIVE	No	45	0	0
0	0	VATS or rc	249	PRIVE	No	52	1	1
0	0	Thoracotoı	249	PRIVE	No	58	0	0
0	0	Thoracotoı	249	PRIVE	No	50	0	0
0	0	Thoracotoı	249	PRIVE	No	58	0	0
0	0	Thoracotoı	249	PRIVE	No	58	0	0
0	0	VATS or rc	249	PRIVE	No	52	0	0
0	1	Thoracotoı	249	PRIVE	No	66	0	0
0	0	Thoracotoı	249	PRIVE	No	69	0	0
0	0	Thoracotoı	249	PRIVE	No	63	0	0
0	0	Thoracotoı	249	PRIVE	No	60	0	1
0	0	Thoracotoı	249	PRIVE	No	39	0	1
0	0	Thoracotoı	249	PRIVE	No	77	0	0
0	0	Thoracotoı	249	PRIVE	No	48	0	1
0	0	Thoracotoı	249	PRIVE	No	67	0	0
0	0	Thoracotoı	249	PRIVE	No	71	0	0
0	0	Thoracotoı	249	PRIVE	No	46	0	0
0	0	VATS or rc	249	PRIVE	No	68	0	0
0	0	Thoracotoı	249	PRIVE	No	61	0	0
0	1	Thoracotoı	249	PRIVE	No	57	0	1
0	0	Thoracotoı	249	PRIVE	No	43	0	0
0	0	Thoracotoı	249	PRIVE	No	59	0	1
0	0	Thoracotoı	249	PRIVE	No	80	0	0
0	0	VATS or rc	249	PRIVE	No	67	0	1
0	0	VATS or rc	249	PRIVE	No	45	0	1
0	0	Thoracotoı	249	PRIVE	No	74	0	0
0	0	Thoracotoı	249	PRIVE	No	65	0	0
0	0	VATS or rc	249	PRIVE	No	60	0	1

0	0	Thoracotoı	249	PRIVE	No	61	0	1
0	0	Thoracotoı	249	PRIVE	No	49	0	0
0	0	Thoracotoı	249	PRIVE	No	77	0	0
0	0	Thoracotoı	249	PRIVE	No	59	0	0
0	1	Thoracotoı	249	PRIVE	No	62	0	0
0	0	Thoracotoı	249	PRIVE	No	74	0	1
0	0	Thoracotoı	249	PRIVE	No	72	0	0
0	0	Thoracotoı	249	PRIVE	No	59	0	1
0	1	Thoracotoı	249	PRIVE	No	77	0	0
0	0	Thoracotoı	249	PRIVE	No	50	0	0
0	0	VATS or rc	249	PRIVE	No	50	0	1
0	0	Thoracotoı	249	PRIVE	No	66	0	0
0	1	Thoracotoı	249	PRIVE	No	73	0	0
0	0	Thoracotoı	249	PRIVE	No	73	0	0
0	0	Thoracotoı	249	PRIVE	No	50	0	1
0	0	Thoracotoı	249	PRIVE	No	69	0	0
0	0	VATS or rc	249	PRIVE	No	65	0	0
0	0	Thoracotoı	249	PRIVE	No	72	0	1
0	0	Thoracotoı	249	PRIVE	No	78	0	0
0	0	VATS or rc	249	PRIVE	No	59	0	0
0	1	Thoracotoı	249	PRIVE	No	59	0	1
0	0	Thoracotoı	249	PRIVE	No	44	1	0
0	0	Thoracotoı	249	PRIVE	No	54	0	0
0	0	Thoracotoı	249	PRIVE	No	56	0	1
0	0	Thoracotoı	249	PRIVE	No	51	0	1
0	1	Thoracotoı	249	PRIVE	Yes	62	0	0
0	0	VATS or rc	249	PRIVE	No	67	0	1
0	1	Thoracotoı	249	PRIVE	No	62	0	0
0	0	Thoracotoı	249	PRIVE	No	61	0	0
0	0	Thoracotoı	249	PRIVE	No	62	0	0
0	0	Thoracotoı	249	PRIVE	No	60	0	0
0	0	Thoracotoı	249	PRIVE	No	59	0	1
0	1	Thoracotoı	249	PRIVE	No	48	0	1
0	0	Thoracotoı	249	PRIVE	No	68	0	0
0	0	VATS or rc	249	PRIVE	No	71	0	0
0	0	Thoracotoı	249	PRIVE	No	54	0	0
0	0	Thoracotoı	249	PRIVE	No	57	0	0
0	0	Thoracotoı	249	PRIVE	No	49	0	1
0	0	Thoracotoı	249	PRIVE	No	51	0	0
0	0	Thoracotoı	249	PRIVE	No	77	0	0
0	0	Thoracotoı	249	PRIVE	No	67	0	0
0	0	Thoracotoı	249	PRIVE	No	72	0	0
0	0	Thoracotoı	249	PRIVE	No	54	0	0
0	0	Thoracotoı	249	PRIVE	No	78	0	0
0	0	VATS or rc	249	PRIVE	No	51	0	1
0	0	Thoracotoı	249	PRIVE	No	74	1	0
0	0	Thoracotoı	249	PRIVE	No	68	0	0
0	1	Thoracotoı	249	PRIVE	No	53	0	0
0	0	VATS or rc	249	PRIVE	No	63	0	1
0	0	Thoracotoı	249	PRIVE	No	72	0	1
0	0	VATS or rc	249	PRIVE	No	54	0	0
0	1	Thoracotoı	249	PRIVE	No	55	0	1
0	0	VATS or rc	249	PRIVE	No	69	0	1
0	0	VATS or rc	249	PRIVE	No	62	0	0
0	0	Thoracotoı	249	PRIVE	No	61	0	0
0	0	Thoracotoı	249	PRIVE	No	26	0	1

0	0	Thoracotoı	249	PRIVE	No	55	0	0
0	0	Thoracotoı	249	PRIVE	No	62	1	0
0	0	Thoracotoı	249	PRIVE	No	53	0	0
0	1	Thoracotoı	249	PRIVE	No	69	0	0
0	0	VATS or rc	249	PRIVE	No	44	0	1
0	0	Thoracotoı	249	PRIVE	No	67	0	0
0	0	Thoracotoı	249	PRIVE	No	61	0	0
0	0	Thoracotoı	249	PRIVE	No	62	0	0
0	0	VATS or rc	249	PRIVE	No	54	0	0
0	0	Thoracotoı	249	PRIVE	No	70	0	0
0	0	Thoracotoı	249	PRIVE	No	54	0	0
0	0	Thoracotoı	249	PRIVE	No	51	1	0
0	0	Thoracotoı	249	PRIVE	No	75	0	0
0	0	Thoracotoı	249	PRIVE	No	39	0	0
0	0	Thoracotoı	249	PRIVE	No	67	0	0
0	0	VATS or rc	249	PRIVE	No	66	0	1
0	0	VATS or rc	249	PRIVE	No	59	0	0
0	0	Thoracotoı	249	PRIVE	No	67	0	0
0	0	Thoracotoı	249	PRIVE	No	49	0	0
0	0	Thoracotoı	249	PRIVE	No	49	0	0
0	0	VATS or rc	249	PRIVE	No	60	0	0
0	0	Thoracotoı	249	PRIVE	No	54	0	0
0	1	Thoracotoı	249	PRIVE	No	45	0	0
0	0	Thoracotoı	249	PRIVE	No	51	0	0
0	1	Thoracotoı	249	PRIVE	No	55	0	0
0	0	Thoracotoı	249	PRIVE	No	77	0	0
0	0	Thoracotoı	249	PRIVE	No	69	0	0
0	0	Thoracotoı	249	PRIVE	No	57	0	0
0	1	Thoracotoı	249	PRIVE	No	56	0	0
0	0	VATS or rc	249	PRIVE	No	59	0	1
0	0	Thoracotoı	249	PRIVE	No	76	0	0
0	0	Thoracotoı	249	PRIVE	No	73	0	0
0	0	Thoracotoı	249	PRIVE	No	70	0	0
0	0	VATS or rc	249	PRIVE	No	75	0	0
0	0	VATS or rc	249	PRIVE	No	75	0	0
0	0	Thoracotoı	249	PRIVE	No	80	0	0
0	0	VATS or rc	249	PRIVE	No	50	0	1
0	0	Thoracotoı	249	PRIVE	No	51	0	0
0	1	Thoracotoı	249	PRIVE	No	67	0	0
0	0	VATS or rc	249	PRIVE	No	60	0	0
0	0	VATS or rc	249	PRIVE	No	42	0	1
0	1	Thoracotoı	249	PRIVE	No	48	0	0
0	0	Thoracotoı	249	PRIVE	No	72	0	0
0	0	Thoracotoı	249	PRIVE	No	77	0	0
0	0	Thoracotoı	249	PRIVE	No	54	0	0
0	0	Thoracotoı	249	PRIVE	No	71	0	0
0	1	Thoracotoı	249	PRIVE	No	64	0	0
0	0	VATS or rc	249	PRIVE	No	63	0	1
0	0	VATS or rc	249	PRIVE	No	49	0	1
0	0	Thoracotoı	249	PRIVE	No	61	0	0
0	0	Thoracotoı	249	PRIVE	No	73	0	0
0	0	Thoracotoı	249	PRIVE	No	72	0	0
0	0	Thoracotoı	249	PRIVE	No	71	0	1
0	0	VATS or rc	249	PRIVE	No	73	0	0
0	1	Thoracotoı	249	PRIVE	No	57	0	0
0	0	Thoracotoı	249	PRIVE	No	65	0	0

0	0	Thoracotoı	249	PRIVE	No	61	0	0
0	0	Thoracotoı	249	PRIVE	No	62	0	0
0	0	Thoracotoı	249	PRIVE	No	76	1	0
0	0	VATS or rc	249	PRIVE	No	73	0	0
0	0	Thoracotoı	249	PRIVE	No	77	1	0
0	0	VATS or rc	249	PRIVE	No	78	0	0
0	0	Thoracotoı	249	PRIVE	No	70	0	0
0	0	Thoracotoı	249	PRIVE	No	56	0	0
0	0	Thoracotoı	249	PRIVE	No	53	0	0
0	0	Thoracotoı	249	PRIVE	No	74	0	0
0	0	Thoracotoı	249	PRIVE	No	69	0	0
0	0	VATS or rc	249	PRIVE	No	45	0	1
0	0	Thoracotoı	249	PRIVE	No	58	0	0
0	0	Thoracotoı	249	PRIVE	No	73	0	0
0	1	Thoracotoı	249	PRIVE	No	73	0	0
0	1	Thoracotoı	249	PRIVE	No	68	0	0
0	0	VATS or rc	249	PRIVE	No	58	0	0
0	0	Thoracotoı	249	PRIVE	No	57	0	0
0	0	VATS or rc	249	PRIVE	No	65	0	0
0	0	Thoracotoı	249	PRIVE	No	63	0	0
0	0	Thoracotoı	249	PRIVE	No	36	0	0
0	0	Thoracotoı	249	PRIVE	No	75	0	0
0	0	Thoracotoı	249	PRIVE	No	59	0	0
0	1	Thoracotoı	249	PRIVE	No	62	0	0
0	0	VATS or rc	249	PRIVE	No	55	0	0
0	0	Thoracotoı	249	PRIVE	No	72	0	0
0	1	Thoracotoı	249	PRIVE	No	65	0	0
0	0	Thoracotoı	249	PRIVE	No	61	1	0
0	0	Thoracotoı	249	PRIVE	No	48	0	0
0	0	Thoracotoı	249	PRIVE	No	65	0	0
0	0	Thoracotoı	249	PRIVE	No	70	0	0
0	0	Thoracotoı	249	PRIVE	No	74	1	0
0	0	VATS or rc	249	PRIVE	No	81	0	0
0	0	Thoracotoı	249	PRIVE	No	65	0	0
0	0	Thoracotoı	249	PRIVE	No	56	0	0
0	0	Thoracotoı	249	PRIVE	No	60	0	0
0	0	VATS or rc	249	PRIVE	No	77	0	0
0	0	Thoracotoı	249	PRIVE	No	62	0	0
0	0	Thoracotoı	249	PRIVE	No	66	0	0
0	0	VATS or rc	249	PRIVE	No	60	0	1
0	0	Thoracotoı	249	PRIVE	No	78	1	0
0	0	Thoracotoı	249	PRIVE	No	64	0	0
0	0	VATS or rc	249	PRIVE	No	72	0	0
0	0	VATS or rc	249	PRIVE	No	67	0	0
0	1	Thoracotoı	249	PRIVE	No	52	0	0
0	0	VATS or rc	249	PRIVE	No	68	0	1
0	0	Thoracotoı	249	PRIVE	No	76	0	0
0	0	Thoracotoı	249	PRIVE	No	55	0	0
0	0	Thoracotoı	249	PRIVE	No	65	0	0
0	0	Thoracotoı	249	PRIVE	No	51	1	0
0	0	Thoracotoı	249	PRIVE	No	66	0	0
0	0	Thoracotoı	249	PRIVE	No	53	0	0
0	1	Thoracotoı	249	PRIVE	No	41	0	0
0	0	VATS or rc	249	PRIVE	No	64	0	0
0	0	Thoracotoı	249	PRIVE	No	75	0	0
0	1	Thoracotoı	249	PRIVE	No	58	0	0

0	1	Thoracotoı	249	PRIVE	No	59	0	0
0	0	Thoracotoı	249	PRIVE	No	60	0	0
0	0	Thoracotoı	249	PRIVE	No	81	0	0
0	0	VATS or rc	249	PRIVE	No	50	0	0
0	0	Thoracotoı	249	PRIVE	No	59	0	0
0	0	Thoracotoı	249	PRIVE	No	75	0	0
0	0	Thoracotoı	249	PRIVE	No	54	0	0
0	0	Thoracotoı	249	PRIVE	No	79	0	1
0	0	Thoracotoı	249	PRIVE	No	72	0	1
0	0	VATS or rc	249	PRIVE	No	66	0	1
0	0	VATS or rc	249	PRIVE	No	70	1	0
0	0	Thoracotoı	249	PRIVE	No	55	1	0
0	0	Thoracotoı	249	PRIVE	No	69	0	1
0	0	Thoracotoı	249	PRIVE	No	44	0	0
0	1	Thoracotoı	249	PRIVE	No	52	0	0
0	0	Thoracotoı	249	PRIVE	No	49	0	1
0	0	Thoracotoı	249	PRIVE	No	55	0	0
0	0	Thoracotoı	249	PRIVE	No	71	0	1
0	0	VATS or rc	249	PRIVE	No	59	0	0
0	0	Thoracotoı	249	PRIVE	No	63	0	0
0	1	Thoracotoı	249	PRIVE	No	45	0	0
0	0	Thoracotoı	249	PRIVE	No	80	0	0
0	0	Thoracotoı	249	PRIVE	No	58	0	0
0	0	Thoracotoı	249	PRIVE	No	81	0	0
0	0	Thoracotoı	249	PRIVE	No	57	0	0
0	0	Thoracotoı	249	PRIVE	No	76	0	0
0	0	Thoracotoı	249	PRIVE	No	71	0	0
0	0	Thoracotoı	249	PRIVE	No	64	1	0
0	0	VATS or rc	249	PRIVE	No	60	0	0
0	0	VATS or rc	249	PRIVE	No	67	0	0
0	0	Thoracotoı	249	PRIVE	No	67	0	1
0	0	VATS or rc	249	PRIVE	No	63	0	0
0	1	Thoracotoı	249	PRIVE	No	64	0	1
0	0	Thoracotoı	249	PRIVE	No	77	0	0
0	0	Thoracotoı	249	PRIVE	No	63	0	0
0	0	Thoracotoı	249	PRIVE	No	72	0	1
0	1	Thoracotoı	249	PRIVE	No	67	1	1
0	0	VATS or rc	249	PRIVE	No	78	0	0
0	0	Thoracotoı	249	PRIVE	No	52	0	0
0	1	Thoracotoı	249	PRIVE	No	55	0	0
0	0	Thoracotoı	249	PRIVE	No	64	0	0
0	0	VATS or rc	249	PRIVE	No	76	0	1
0	0	Thoracotoı	249	PRIVE	No	50	0	0
0	0	Thoracotoı	249	PRIVE	No	64	0	0
0	0	Thoracotoı	249	PRIVE	No	45	0	1
0	0	Thoracotoı	249	PRIVE	No	66	0	0
0	0	Thoracotoı	249	PRIVE	No	64	0	0
0	0	Thoracotoı	249	PRIVE	No	71	0	0
0	0	VATS or rc	249	PRIVE	No	59	0	0
0	0	VATS or rc	249	PRIVE	No	65	0	0
0	0	Thoracotoı	249	PRIVE	No	67	0	0
0	0	VATS or rc	249	PRIVE	No	81	0	0
0	0	Thoracotoı	249	PRIVE	No	67	0	1
0	0	VATS or rc	249	PRIVE	No	69	0	1
0	0	VATS or rc	249	PRIVE	No	64	0	0
0	0	Thoracotoı	249	PRIVE	No	74	0	0

0	0	Thoracotoı	249	PRIVE	No	77	0	0
0	0	Thoracotoı	249	PRIVE	No	46	0	0
0	0	Thoracotoı	249	PRIVE	No	69	0	0
0	1	Thoracotoı	249	PRIVE	No	53	0	0
0	0	Thoracotoı	249	PRIVE	No	57	0	0
0	0	Thoracotoı	249	PRIVE	No	53	0	0
0	0	Thoracotoı	249	PRIVE	No	71	0	0
0	0	Thoracotoı	249	PRIVE	No	58	0	0
0	0	Thoracotoı	249	PRIVE	No	62	0	0
0	1	Thoracotoı	249	PRIVE	No	57	0	0
0	0	Thoracotoı	249	PRIVE	No	67	0	0
0	0	VATS or rc	249	PRIVE	No	61	0	0
0	0	Thoracotoı	249	PRIVE	No	58	0	1
0	1	Thoracotoı	249	PRIVE	No	55	0	0
0	0	Thoracotoı	249	PRIVE	No	49	1	0
0	1	Thoracotoı	249	PRIVE	No	64	0	0
0	0	VATS or rc	249	PRIVE	No	71	0	0
0	0	Thoracotoı	249	PRIVE	No	59	0	0
0	0	Thoracotoı	249	PRIVE	No	59	0	1
0	0	VATS or rc	249	PRIVE	No	57	1	0
0	0	Thoracotoı	249	PRIVE	No	81	0	0
0	0	VATS or rc	249	PRIVE	No	64	0	1
0	0	Thoracotoı	249	PRIVE	No	65	0	1
0	0	Thoracotoı	249	PRIVE	No	60	0	0
0	0	Thoracotoı	249	PRIVE	No	70	0	0
0	0	VATS or rc	249	PRIVE	No	61	0	0
0	0	Thoracotoı	249	PRIVE	No	57	0	0
0	0	VATS or rc	249	PRIVE	No	60	1	1
0	0	Thoracotoı	249	PRIVE	No	56	0	0
0	0	VATS or rc	249	PRIVE	No	68	0	0
0	0	Thoracotoı	249	PRIVE	No	48	0	1
0	0	Thoracotoı	249	PRIVE	No	53	0	0
0	1	Thoracotoı	249	PRIVE	No	51	0	0
0	0	Thoracotoı	249	PRIVE	No	68	0	0
0	1	Thoracotoı	249	PRIVE	Yes	67	0	1
0	0	Thoracotoı	249	PRIVE	No	69	0	0
0	0	Thoracotoı	249	PRIVE	No	56	0	1
0	0	Thoracotoı	249	PRIVE	No	71	0	0
0	0	Thoracotoı	249	PRIVE	No	68	0	0
0	0	Thoracotoı	249	PRIVE	No	45	0	0
0	0	VATS or rc	249	PRIVE	No	64	0	0
0	0	Thoracotoı	249	PRIVE	No	68	0	0
0	0	VATS or rc	249	PRIVE	No	37	0	1
0	0	VATS or rc	249	PRIVE	No	81	1	0
0	0	Thoracotoı	249	PRIVE	No	58	0	1
0	0	Thoracotoı	249	PRIVE	No	77	0	1
0	0	Thoracotoı	249	PRIVE	No	60	0	0
0	0	Thoracotoı	249	PRIVE	No	58	0	0
0	0	Thoracotoı	249	PRIVE	No	68	0	0
0	0	Thoracotoı	249	PRIVE	No	40	0	0
0	1	Thoracotoı	249	PRIVE	No	59	0	0
0	0	VATS or rc	249	PRIVE	No	57	0	1
0	0	VATS or rc	249	PRIVE	Yes	73	0	0
0	0	Thoracotoı	249	PRIVE	No	62	0	0
0	0	Thoracotoı	249	PRIVE	No	55	0	1
0	1	Thoracotoı	249	PRIVE	No	52	0	0

0	0	Thoracotoı	249	PRIVE	No	72	0	0
0	0	Thoracotoı	249	PRIVE	No	70	0	0
0	0	Thoracotoı	249	PRIVE	No	61	0	0
0	1	Thoracotoı	249	PRIVE	No	57	0	0
0	1	Thoracotoı	249	PRIVE	No	50	0	0
0	1	Thoracotoı	249	PRIVE	No	55	0	1
0	0	Thoracotoı	249	PRIVE	No	71	0	0
0	0	VATS or rc	249	PRIVE	No	63	0	1
0	0	Thoracotoı	249	PRIVE	No	76	0	0
0	0	Thoracotoı	249	PRIVE	No	68	0	0
0	0	Thoracotoı	249	PRIVE	No	62	0	0
0	1	Thoracotoı	249	PRIVE	No	71	0	0
0	1	Thoracotoı	249	PRIVE	No	60	0	0
0	1	Thoracotoı	249	PRIVE	No	60	0	1
0	1	Thoracotoı	249	PRIVE	No	65	0	0
0	0	Thoracotoı	249	PRIVE	No	73	0	0
0	0	VATS or rc	249	PRIVE	No	75	0	0
0	0	Thoracotoı	249	PRIVE	No	72	0	0
0	0	Thoracotoı	249	PRIVE	No	64	0	0
0	0	Thoracotoı	249	PRIVE	No	53	0	0
0	0	Thoracotoı	249	PRIVE	No	81	0	0
0	0	VATS or rc	249	PRIVE	No	54	0	0
0	0	VATS or rc	249	PRIVE	No	71	0	0
0	1	Thoracotoı	249	PRIVE	No	62	0	0
0	0	Thoracotoı	249	PRIVE	No	54	0	0
0	0	Thoracotoı	249	PRIVE	No	64	0	0
0	0	Thoracotoı	249	PRIVE	No	63	0	0
0	0	VATS or rc	249	PRIVE	No	67	0	0
0	0	Thoracotoı	249	PRIVE	No	52	0	0
0	0	Thoracotoı	249	PRIVE	No	70	0	0
0	0	Thoracotoı	249	PRIVE	No	74	0	0
0	0	Thoracotoı	249	PRIVE	No	64	0	0
0	0	VATS or rc	249	PRIVE	No	69	0	0
0	0	Thoracotoı	249	PRIVE	No	68	0	0
0	0	Thoracotoı	249	PRIVE	No	67	0	0
0	0	Thoracotoı	249	PRIVE	No	52	0	0
0	1	Thoracotoı	249	PRIVE	No	60	0	0
0	0	Thoracotoı	249	PRIVE	No	75	0	0
0	0	VATS or rc	249	PRIVE	No	57	0	0
0	0	Thoracotoı	249	PRIVE	No	61	0	0
0	0	Thoracotoı	249	PRIVE	No	63	0	0
0	0	Thoracotoı	249	PRIVE	No	74	0	0
0	1	Thoracotoı	249	PRIVE	No	61	0	1
0	0	Thoracotoı	249	PRIVE	No	67	0	0
0	0	Thoracotoı	249	PRIVE	No	63	0	0
0	0	Thoracotoı	249	PRIVE	No	56	0	0
0	0	VATS or rc	249	PRIVE	No	64	0	1
0	0	VATS or rc	249	PRIVE	No	58	0	0
0	0	Thoracotoı	249	PRIVE	No	71	0	0
0	0	Thoracotoı	249	PRIVE	No	61	0	0
0	0	VATS or rc	249	PRIVE	No	40	0	0
0	1	Thoracotoı	249	PRIVE	No	67	0	0
0	0	Thoracotoı	249	PRIVE	No	66	0	0
0	0	Thoracotoı	249	PRIVE	No	61	0	1
0	0	Thoracotoı	249	PRIVE	No	60	0	0
0	0	Thoracotoı	249	PRIVE	No	40	0	0

0	1	Thoracotoı	249	PRIVE	No	53	0	0
0	0	Thoracotoı	249	PRIVE	No	61	0	0
0	0	Thoracotoı	249	PRIVE	No	54	0	0
0	0	Thoracotoı	249	PRIVE	No	69	0	0
0	0	VATS or ro	249	PRIVE	No	53	0	1
0	0	Thoracotoı	249	PRIVE	No	68	0	0
0	0	Thoracotoı	249	PRIVE	No	53	0	1
0	0	Thoracotoı	249	PRIVE	No	72	0	0
0	0	Thoracotoı	249	PRIVE	No	58	0	0
0	0	Thoracotoı	249	PRIVE	No	67	0	0
0	0	VATS or ro	249	PRIVE	No	64	0	0
0	0	Thoracotoı	249	PRIVE	No	63	0	0
0	0	Thoracotoı	249	PRIVE	No	78	0	0
0	0	Thoracotoı	249	PRIVE	No	79	0	0
0	0	Thoracotoı	249	PRIVE	No	65	0	0
0	0	Thoracotoı	249	PRIVE	No	54	0	0
0	0	Thoracotoı	249	PRIVE	No	67	0	0
0	0	VATS or ro	249	PRIVE	No	71	1	0
0	0	Thoracotoı	249	PRIVE	No	58	0	0
0	0	Thoracotoı	249	PRIVE	No	66	1	0
0	0	VATS or ro	249	PRIVE	No	69	0	1
0	0	Thoracotoı	249	PRIVE	No	62	0	0
0	0	Thoracotoı	249	PRIVE	No	60	0	1
0	0	Thoracotoı	249	PRIVE	No	56	0	1
0	0	Thoracotoı	249	PRIVE	No	81	1	0
0	1	Thoracotoı	249	PRIVE	Yes	79	0	0
0	0	Thoracotoı	249	PRIVE	No	54	0	0
0	0	Thoracotoı	249	PRIVE	No	75	0	0
0	0	Thoracotoı	249	PRIVE	No	56	0	1
0	0	Thoracotoı	249	PRIVE	No	75	0	0
0	0	Thoracotoı	249	PRIVE	No	65	0	0
0	0	Thoracotoı	249	PRIVE	No	73	0	0
0	0	Thoracotoı	249	PRIVE	No	54	0	0
0	0	Thoracotoı	249	PRIVE	No	64	0	0
0	0	Thoracotoı	249	PRIVE	No	63	0	0
0	0	Thoracotoı	249	PRIVE	No	49	0	0
0	0	VATS or ro	249	PRIVE	No	68	0	1
0	1	Thoracotoı	249	PRIVE	No	59	0	0
0	0	Thoracotoı	249	PRIVE	No	45	0	0
0	0	Thoracotoı	249	PRIVE	No	73	0	0
0	0	VATS or ro	249	PRIVE	No	69	0	0
0	0	Thoracotoı	249	PRIVE	No	75	1	0
0	0	Thoracotoı	249	PRIVE	No	64	0	0
0	1	Thoracotoı	249	PRIVE	No	61	0	0
0	0	Thoracotoı	249	PRIVE	No	56	0	1
0	0	VATS or ro	249	PRIVE	No	63	0	1
0	0	Thoracotoı	249	PRIVE	No	72	0	0
0	0	VATS or ro	249	PRIVE	No	69	0	0
0	1	Thoracotoı	249	PRIVE	No	52	0	0
0	0	Thoracotoı	249	PRIVE	No	62	0	0
0	0	Thoracotoı	249	PRIVE	No	20	0	0
0	0	Thoracotoı	249	PRIVE	No	56	0	0
0	0	Thoracotoı	249	PRIVE	No	69	0	1
0	0	Thoracotoı	249	PRIVE	No	75	0	0
0	0	VATS or ro	249	PRIVE	No	63	0	0
0	0	Thoracotoı	249	PRIVE	No	60	0	1

0	0	Thoracotoı	249	PRIVE	No	70	0	0
0	0	Thoracotoı	249	PRIVE	No	61	0	1
0	0	Thoracotoı	249	PRIVE	No	61	0	0
0	0	Thoracotoı	249	PRIVE	No	64	0	0
0	1	Thoracotoı	249	PRIVE	No	61	0	0
0	0	Thoracotoı	249	PRIVE	No	46	0	0
0	1	Thoracotoı	249	PRIVE	No	63	0	0
0	0	Thoracotoı	249	PRIVE	No	69	0	0
0	0	VATS or rc	249	PRIVE	No	80	0	0
0	0	Thoracotoı	249	PRIVE	No	56	1	0
0	0	Thoracotoı	249	PRIVE	No	50	0	1
0	0	Thoracotoı	249	PRIVE	No	59	0	0
0	0	Thoracotoı	249	PRIVE	No	54	0	0
0	0	Thoracotoı	249	PRIVE	No	63	0	0
0	0	Thoracotoı	249	PRIVE	No	78	0	0
0	0	Thoracotoı	249	PRIVE	No	58	0	0
0	0	VATS or rc	249	PRIVE	No	42	0	1
0	0	Thoracotoı	249	PRIVE	No	70	0	1
0	0	Thoracotoı	249	PRIVE	No	62	0	1
0	0	VATS or rc	249	PRIVE	No	50	0	0
0	1	Thoracotoı	249	PRIVE	Yes	73	0	0
0	0	Thoracotoı	249	PRIVE	No	80	0	0
0	0	Thoracotoı	249	PRIVE	No	74	0	0
0	1	Thoracotoı	249	PRIVE	No	56	0	0
0	0	Thoracotoı	249	PRIVE	No	62	0	0
0	0	Thoracotoı	249	PRIVE	No	60	1	0
0	0	Thoracotoı	249	PRIVE	No	65	0	0
0	0	Thoracotoı	249	PRIVE	No	72	0	1
0	0	Thoracotoı	249	PRIVE	No	75	0	0
0	1	Thoracotoı	249	PRIVE	No	58	0	0
0	0	VATS or rc	249	PRIVE	No	59	0	0
0	0	VATS or rc	249	PRIVE	No	71	0	0
0	0	Thoracotoı	249	PRIVE	No	59	0	1
0	1	Thoracotoı	249	PRIVE	No	67	0	0
0	0	Thoracotoı	249	PRIVE	No	60	0	0
0	0	Thoracotoı	249	PRIVE	No	56	0	0
0	0	VATS or rc	249	PRIVE	No	70	0	1
0	0	Thoracotoı	249	PRIVE	No	63	0	0
0	0	Thoracotoı	249	PRIVE	No	72	0	0
0	0	VATS or rc	249	PRIVE	No	74	0	0
0	0	Thoracotoı	249	PRIVE	No	65	0	0
0	0	Thoracotoı	249	PRIVE	No	54	0	0
0	0	Thoracotoı	249	PRIVE	No	82	0	0
0	0	Thoracotoı	249	PRIVE	No	76	0	0
0	0	Thoracotoı	249	PRIVE	No	63	0	0
0	0	Thoracotoı	249	PRIVE	No	76	0	0
0	0	Thoracotoı	249	PRIVE	No	56	0	0
0	0	VATS or rc	249	PRIVE	No	65	0	0
0	0	Thoracotoı	249	PRIVE	No	67	1	0
0	0	VATS or rc	249	PRIVE	No	65	0	0
0	0	Thoracotoı	249	PRIVE	No	50	0	0
0	0	Thoracotoı	249	PRIVE	No	71	0	1
0	0	Thoracotoı	249	PRIVE	No	65	0	0
0	0	Thoracotoı	249	PRIVE	No	49	0	1
0	0	Thoracotoı	249	PRIVE	No	62	0	0
0	0	Thoracotoı	249	PRIVE	No	69	0	0

0	0	Thoracotoı	249	PRIVE	No	53	0	0
0	0	Thoracotoı	249	PRIVE	No	58	0	1
0	1	Thoracotoı	249	PRIVE	No	50	0	0
0	0	Thoracotoı	249	PRIVE	No	81	0	0
0	0	Thoracotoı	249	PRIVE	No	66	0	0
0	0	Thoracotoı	249	PRIVE	No	68	0	0
0	0	Thoracotoı	249	PRIVE	No	30	0	0
0	0	VATS or rc	249	PRIVE	No	63	0	0
0	0	Thoracotoı	249	PRIVE	No	50	0	0
0	0	Thoracotoı	249	PRIVE	No	46	0	0
0	0	Thoracotoı	249	PRIVE	No	69	0	0
0	0	Thoracotoı	249	PRIVE	No	48	0	0
0	0	Thoracotoı	249	PRIVE	No	66	1	0
0	0	Thoracotoı	249	PRIVE	No	58	0	0
0	0	Thoracotoı	249	PRIVE	No	63	0	0
0	0	Thoracotoı	249	PRIVE	Yes	69	0	0
0	0	VATS or rc	249	PRIVE	No	58	1	1
0	0	Thoracotoı	249	PRIVE	No	58	0	0
0	0	Thoracotoı	249	PRIVE	No	68	1	0
0	0	Thoracotoı	249	PRIVE	No	51	0	0
0	0	VATS or rc	249	PRIVE	No	60	0	1
0	0	VATS or rc	249	PRIVE	No	54	0	1
0	0	Thoracotoı	249	PRIVE	No	57	0	0
0	0	Thoracotoı	249	PRIVE	No	59	1	0
0	0	Thoracotoı	249	PRIVE	No	58	0	0
0	0	Thoracotoı	249	PRIVE	No	67	0	0
0	0	VATS or rc	249	PRIVE	No	56	0	0
0	1	Thoracotoı	249	PRIVE	No	60	0	0
0	0	Thoracotoı	249	PRIVE	No	56	0	0
0	0	Thoracotoı	249	PRIVE	No	60	0	1
0	0	Thoracotoı	249	PRIVE	No	51	0	0
0	0	Thoracotoı	249	PRIVE	No	59	0	0
0	0	VATS or rc	249	PRIVE	No	56	0	0
0	1	Thoracotoı	249	PRIVE	No	56	0	0
0	0	Thoracotoı	249	PRIVE	No	63	0	0
0	0	Thoracotoı	249	PRIVE	No	59	0	1
0	0	Thoracotoı	249	PRIVE	No	58	0	1
0	0	Thoracotoı	249	PRIVE	No	58	0	0
0	0	VATS or rc	249	PRIVE	No	54	0	1
0	0	Thoracotoı	249	PRIVE	No	58	0	0
0	0	Thoracotoı	249	PRIVE	No	71	0	0
0	0	VATS or rc	249	PRIVE	No	53	0	1
0	0	Thoracotoı	249	PRIVE	No	59	0	1
0	0	Thoracotoı	249	PRIVE	No	68	0	0
0	0	VATS or rc	249	PRIVE	No	75	0	0
0	0	Thoracotoı	249	PRIVE	No	67	0	0
0	1	Thoracotoı	249	PRIVE	No	67	0	1
0	0	VATS or rc	249	PRIVE	No	57	0	1
0	0	VATS or rc	249	PRIVE	No	71	0	1
0	0	Thoracotoı	249	PRIVE	No	69	1	0
0	0	Thoracotoı	249	PRIVE	No	56	0	1
0	0	Thoracotoı	249	PRIVE	No	53	0	0
0	0	Thoracotoı	249	PRIVE	No	50	0	1
0	0	VATS or rc	249	PRIVE	No	63	0	0
0	0	Thoracotoı	249	PRIVE	No	67	0	1
0	0	Thoracotoı	249	PRIVE	No	78	0	0

0	0	Thoracotor	249	PRIVE	No	89	0	0
0	0	Thoracotor	249	PRIVE	No	75	0	0
0	0	VATS or ro	249	PRIVE	No	77	0	1
0	0	Thoracotor	249	PRIVE	No	53	0	0
0	0	Thoracotor	249	PRIVE	No	58	1	0
0	0	Thoracotor	249	PRIVE	No	78	0	0
0	0	VATS or ro	249	PRIVE	No	71	0	0
0	0	Thoracotor	249	PRIVE	No	69	0	0
0	0	VATS or ro	249	PRIVE	No	77	0	0
0	0	Thoracotor	249	PRIVE	No	60	0	0
0	0	Thoracotor	249	PRIVE	No	50	0	1
0	0	VATS or ro	249	PRIVE	No	67	0	0
0	0	Thoracotor	249	PRIVE	No	68	0	0
0	1	Thoracotor	249	PRIVE	No	48	1	0
0	0	Thoracotor	249	PRIVE	No	55	1	1
0	0	Thoracotor	249	PRIVE	No	59	0	0
0	0	Thoracotor	249	PRIVE	No	66	1	1
0	0	Thoracotor	249	PRIVE	No	51	0	0
0	0	Thoracotor	249	PRIVE	No	57	1	0
0	0	Thoracotor	249	PRIVE	No	71	0	1
0	1	Thoracotor	249	PRIVE	No	55	0	0
0	0	VATS or ro	249	PRIVE	No	60	0	0
0	0	Thoracotor	249	PRIVE	No	75	0	0
0	0	VATS or ro	249	PRIVE	No	70	0	1
0	0	Thoracotor	249	PRIVE	No	61	0	1
0	0	VATS or ro	249	PRIVE	No	61	1	1
0	0	VATS or ro	249	PRIVE	No	70	0	0
0	0	Thoracotor	249	PRIVE	No	74	0	1
0	0	Thoracotor	249	PRIVE	No	59	0	0
0	0	Thoracotor	249	PRIVE	No	72	0	0
0	0	Thoracotor	249	PRIVE	No	74	0	0
0	0	Thoracotor	249	PRIVE	No	78	0	0
0	0	Thoracotor	249	PRIVE	No	51	0	0
0	0	Thoracotor	249	PRIVE	No	50	0	1
0	0	Thoracotor	249	PRIVE	No	44	0	0
0	0	Thoracotor	249	PRIVE	No	69	0	1
0	0	Thoracotor	249	PRIVE	No	60	0	1
0	0	Thoracotor	249	PRIVE	No	61	0	1
0	1	Thoracotor	249	PRIVE	No	58	0	0
0	1	Thoracotor	249	PRIVE	No	46	0	0
0	0	Thoracotor	249	PRIVE	No	57	0	0
0	0	Thoracotor	249	PRIVE	No	64	1	0
0	0	Thoracotor	249	PRIVE	No	75	0	0
0	0	VATS or ro	249	PRIVE	No	62	0	0
0	0	VATS or ro	249	PRIVE	No	80	0	0
0	0	Thoracotor	249	PRIVE	No	78	0	0
0	0	Thoracotor	249	PRIVE	No	61	0	1
0	1	Thoracotor	249	PRIVE	No	78	0	1
0	1	Thoracotor	249	PRIVE	No	56	0	1
0	0	Thoracotor	249	PRIVE	No	62	0	0
0	1	Thoracotor	249	PRIVE	No	67	0	0
0	1	Thoracotor	249	PRIVE	No	66	0	0
0	0	Thoracotor	249	PRIVE	No	53	0	0
0	0	VATS or ro	249	PRIVE	No	71	0	0
0	0	Thoracotor	249	PRIVE	No	69	0	0
0	0	Thoracotor	249	PRIVE	No	67	0	0

0	0	Thoracotoı	249	PRIVE	No	64	1	0
0	1	Thoracotoı	249	PRIVE	No	56	0	0
0	0	Thoracotoı	249	PRIVE	No	66	0	0
0	0	Thoracotoı	249	PRIVE	No	54	0	0
0	0	Thoracotoı	249	PRIVE	No	75	0	1
0	0	Thoracotoı	249	PRIVE	No	66	0	0
0	0	VATS or rc	249	PRIVE	No	61	0	1
0	0	VATS or rc	249	PRIVE	No	56	0	0
0	0	VATS or rc	249	PRIVE	No	66	0	0
0	0	Thoracotoı	249	PRIVE	No	63	0	1
0	1	Thoracotoı	249	PRIVE	No	65	0	0
0	0	Thoracotoı	249	PRIVE	No	52	0	1
0	0	Thoracotoı	249	PRIVE	No	78	0	0
0	0	Thoracotoı	249	PRIVE	No	79	0	0
0	0	Thoracotoı	249	PRIVE	No	45	0	1
0	0	Thoracotoı	249	PRIVE	No	52	0	0
0	0	Thoracotoı	249	PRIVE	No	58	0	0
0	0	VATS or rc	249	PRIVE	No	65	0	0
0	0	Thoracotoı	249	PRIVE	No	77	0	0
0	0	Thoracotoı	249	PRIVE	No	62	0	1
0	1	Thoracotoı	249	PRIVE	No	60	0	0
0	0	Thoracotoı	249	PRIVE	No	54	0	0
0	0	Thoracotoı	249	PRIVE	No	53	0	0
0	1	Thoracotoı	249	PRIVE	No	44	0	0
0	0	VATS or rc	249	PRIVE	No	55	0	1
0	0	Thoracotoı	249	PRIVE	No	76	0	1
0	0	Thoracotoı	249	PRIVE	No	65	0	0
0	0	Thoracotoı	249	PRIVE	No	64	0	0
0	0	Thoracotoı	249	PRIVE	No	75	0	0
0	0	Thoracotoı	249	PRIVE	No	63	0	1
0	1	Thoracotoı	249	PRIVE	No	57	0	0
0	0	VATS or rc	249	PRIVE	No	66	0	0
0	0	Thoracotoı	249	PRIVE	No	74	0	0
0	0	VATS or rc	249	PRIVE	No	58	0	0
0	0	VATS or rc	249	PRIVE	No	56	0	0
0	1	Thoracotoı	249	PRIVE	No	62	0	0
0	1	Thoracotoı	249	PRIVE	No	76	0	0
0	0	VATS or rc	249	PRIVE	No	54	0	0
0	1	Thoracotoı	249	PRIVE	No	54	0	0
0	0	Thoracotoı	249	PRIVE	No	52	1	1
0	0	Thoracotoı	249	PRIVE	No	75	0	0
0	0	Thoracotoı	249	PRIVE	No	49	0	0
0	1	VATS or rc	249	PRIVE	No	54	0	0
0	0	VATS or rc	249	PRIVE	No	62	0	0
0	0	VATS or rc	249	PRIVE	No	69	0	1
0	0	Thoracotoı	249	PRIVE	No	59	0	0
0	0	Thoracotoı	249	PRIVE	No	49	0	0
0	0	Thoracotoı	249	PRIVE	No	77	0	0
0	0	Thoracotoı	249	PRIVE	No	53	1	0
0	0	Thoracotoı	249	PRIVE	No	74	0	0
0	0	Thoracotoı	249	PRIVE	No	40	0	0
0	0	Thoracotoı	249	PRIVE	No	77	0	0
0	1	Thoracotoı	249	PRIVE	No	70	0	0
0	0	Thoracotoı	249	PRIVE	No	78	0	1
0	0	VATS or rc	249	PRIVE	No	61	0	0
0	0	Thoracotoı	249	PRIVE	No	66	0	0

0	0	Thoracotoı	249	PRIVE	No	78	0	1
0	0	VATS or rc	249	PRIVE	No	54	0	0
0	0	Thoracotoı	249	PRIVE	No	46	0	0
0	0	VATS or rc	249	PRIVE	No	71	1	0
0	0	Thoracotoı	249	PRIVE	No	69	0	0
0	1	Thoracotoı	249	PRIVE	No	64	0	0
0	1	Thoracotoı	249	PRIVE	No	56	0	0
0	0	Thoracotoı	249	PRIVE	No	64	0	0
0	0	Thoracotoı	249	PRIVE	No	69	0	0
0	0	VATS or rc	249	PRIVE	No	72	1	0
0	0	Thoracotoı	249	PRIVE	No	67	0	0
0	0	Thoracotoı	249	PRIVE	No	59	0	1
0	0	Thoracotoı	249	PRIVE	No	79	0	0
0	0	VATS or rc	249	PRIVE	No	66	0	0
0	0	Thoracotoı	249	PRIVE	No	73	0	0
0	0	Thoracotoı	249	PRIVE	No	61	0	0
0	1	Thoracotoı	249	PRIVE	No	76	0	0
0	1	Thoracotoı	249	PRIVE	No	55	0	0
0	1	Thoracotoı	249	PRIVE	No	57	0	0
0	0	Thoracotoı	249	PRIVE	No	53	0	1
0	0	Thoracotoı	249	PRIVE	No	77	0	0
0	0	Thoracotoı	249	PRIVE	No	62	0	0
0	0	Thoracotoı	249	PRIVE	No	52	0	0
0	0	VATS or rc	249	PRIVE	No	71	0	0
0	0	Thoracotoı	249	PRIVE	No	60	1	0
0	1	Thoracotoı	249	PRIVE	No	51	0	1
0	0	Thoracotoı	249	PRIVE	No	66	0	0
0	0	Thoracotoı	249	PRIVE	No	64	0	1
0	1	Thoracotoı	249	PRIVE	No	46	0	1
0	0	Thoracotoı	249	PRIVE	No	40	0	0
0	0	Thoracotoı	249	PRIVE	No	68	0	1
0	0	VATS or rc	249	PRIVE	No	61	0	0
0	0	Thoracotoı	249	PRIVE	No	56	0	0
0	0	Thoracotoı	249	PRIVE	No	74	1	0
0	0	Thoracotoı	249	PRIVE	No	50	0	1
0	0	Thoracotoı	249	PRIVE	No	65	0	1
0	0	VATS or rc	249	PRIVE	No	60	0	0
0	0	Thoracotoı	249	PRIVE	No	59	0	1
0	0	Thoracotoı	249	PRIVE	No	63	0	0
0	1	Thoracotoı	249	PRIVE	No	48	0	0
0	0	Thoracotoı	249	PRIVE	No	74	0	0
0	0	Thoracotoı	249	PRIVE	No	75	0	0
0	0	VATS or rc	249	PRIVE	No	52	0	0
0	1	Thoracotoı	249	PRIVE	No	59	0	0
0	0	Thoracotoı	249	PRIVE	No	76	0	0
0	0	VATS or rc	35	CHG	No	68	0	0
0	0	Thoracotoı	35	CHG	No	73	1	0
0	0	Thoracotoı	35	CHG	No	63	0	0
0	0	Thoracotoı	35	CHG	No	65	0	0
0	0	Thoracotoı	35	CHG	No	62	1	0
0	0	Thoracotoı	35	CHG	No	60	0	0
0	0	Thoracotoı	35	CHG	No	63	0	0
0	0	Thoracotoı	35	CHG	No	66	0	0
0	0	Thoracotoı	35	CHG	No	53	0	0
0	0	Thoracotoı	35	CHG	No	67	0	0
0	0	Thoracotoı	35	CHG	No	73	1	0

0	0	Thoracotoı	84	PRIVE	No	50	0	0
0	0	VATS or rc	84	PRIVE	No	57	0	0
0	0	VATS or rc	84	PRIVE	No	60	0	0
0	0	VATS or rc	84	PRIVE	No	82	1	0
0	0	VATS or rc	84	PRIVE	No	76	0	0
0	0	Thoracotoı	84	PRIVE	Yes	66	0	0
0	0	VATS or rc	84	PRIVE	No	73	0	0
0	0	VATS or rc	84	PRIVE	No	64	0	0
0	0	VATS or rc	84	PRIVE	No	74	0	0
0	0	VATS or rc	84	PRIVE	No	66	0	0
0	0	VATS or rc	84	PRIVE	No	69	1	0
0	0	VATS or rc	84	PRIVE	No	56	1	0
0	1	Thoracotoı	84	PRIVE	No	63	0	0
0	0	VATS or rc	84	PRIVE	No	70	0	0
0	0	VATS or rc	84	PRIVE	No	74	0	0
0	0	VATS or rc	84	PRIVE	No	55	0	1
0	0	Thoracotoı	84	PRIVE	No	51	0	1
0	0	VATS or rc	84	PRIVE	No	73	0	0
0	0	VATS or rc	84	PRIVE	Yes	72	0	0
0	1	Thoracotoı	84	PRIVE	No	60	0	1
0	0	VATS or rc	84	PRIVE	No	60	0	1
0	0	VATS or rc	84	PRIVE	No	63	0	0
0	0	VATS or rc	84	PRIVE	No	62	1	0
0	0	VATS or rc	84	PRIVE	No	72	0	0
0	1	Thoracotoı	84	PRIVE	No	71	0	0
0	0	VATS or rc	84	PRIVE	No	66	0	0
0	0	VATS or rc	84	PRIVE	No	73	0	0
0	1	Thoracotoı	84	PRIVE	No	54	0	1
0	0	VATS or rc	84	PRIVE	No	71	0	0
0	0	Thoracotoı	84	PRIVE	No	62	1	0
0	0	VATS or rc	84	PRIVE	No	68	1	0
0	0	Thoracotoı	84	PRIVE	No	57	0	0
0	0	VATS or rc	84	PRIVE	No	63	0	0
0	0	VATS or rc	84	PRIVE	No	76	0	0
0	0	VATS or rc	84	PRIVE	No	67	0	0
0	0	VATS or rc	84	PRIVE	No	57	0	0
0	0	VATS or rc	84	PRIVE	No	61	0	0
0	0	VATS or rc	84	PRIVE	No	66	0	0
0	0	VATS or rc	84	PRIVE	No	67	0	0
0	1	Thoracotoı	84	PRIVE	No	54	0	0
0	0	VATS or rc	84	PRIVE	No	58	0	0
0	0	VATS or rc	84	PRIVE	No	57	0	0
0	0	VATS or rc	84	PRIVE	No	43	0	1
0	0	VATS or rc	84	PRIVE	No	64	1	0
0	0	VATS or rc	84	PRIVE	No	54	0	0
0	0	Thoracotoı	84	PRIVE	No	54	0	0
0	1	Thoracotoı	84	PRIVE	No	61	0	0
0	0	VATS or rc	84	PRIVE	No	47	0	0
0	0	VATS or rc	84	PRIVE	No	64	0	0
0	0	VATS or rc	84	PRIVE	No	76	0	0
0	0	VATS or rc	84	PRIVE	No	67	0	0
0	0	VATS or rc	84	PRIVE	No	72	0	0
0	0	VATS or rc	84	PRIVE	No	73	0	0
0	0	Thoracotoı	84	PRIVE	No	74	1	0
0	0	VATS or rc	84	PRIVE	No	51	0	1
0	0	VATS or rc	84	PRIVE	No	67	0	0

0	0	VATS or ro	84	PRIVE	No	65	0	1
0	0	VATS or ro	84	PRIVE	No	74	0	0
0	1	Thoracotoı	84	PRIVE	No	50	0	0
0	0	Thoracotoı	84	PRIVE	No	77	0	0
0	0	VATS or ro	84	PRIVE	No	65	1	0
0	0	VATS or ro	84	PRIVE	No	44	0	0
0	0	VATS or ro	84	PRIVE	No	63	0	0
0	0	Thoracotoı	84	PRIVE	No	41	0	0
0	0	Thoracotoı	84	PRIVE	No	71	0	0
0	0	VATS or ro	84	PRIVE	No	63	0	0
0	0	VATS or ro	84	PRIVE	No	57	0	0
0	0	VATS or ro	84	PRIVE	No	67	0	1
0	0	Thoracotoı	84	PRIVE	No	70	0	0
0	0	VATS or ro	84	PRIVE	No	68	0	0
0	0	VATS or ro	84	PRIVE	No	77	0	0
0	0	VATS or ro	84	PRIVE	No	81	1	0
0	0	VATS or ro	84	PRIVE	No	55	0	0
0	0	VATS or ro	84	PRIVE	No	62	0	0
0	0	Thoracotoı	84	PRIVE	No	67	1	0
0	0	VATS or ro	84	PRIVE	No	65	0	1
0	0	VATS or ro	84	PRIVE	No	50	1	0
0	0	VATS or ro	84	PRIVE	No	66	1	0
0	0	Thoracotoı	84	PRIVE	No	69	0	0
0	0	VATS or ro	84	PRIVE	No	51	0	0
0	0	Thoracotoı	84	PRIVE	No	66	0	0
0	0	VATS or ro	84	PRIVE	No	68	0	0
0	0	VATS or ro	84	PRIVE	No	59	1	0
0	0	VATS or ro	84	PRIVE	No	70	0	0
0	0	VATS or ro	84	PRIVE	No	72	0	0
0	0	VATS or ro	84	PRIVE	No	57	0	0
0	1	Thoracotoı	84	PRIVE	No	52	0	0
0	0	VATS or ro	84	PRIVE	No	84	0	0
0	0	Thoracotoı	84	PRIVE	No	44	0	0
0	0	VATS or ro	84	PRIVE	No	65	0	1
0	0	Thoracotoı	84	PRIVE	No	49	0	1
0	1	Thoracotoı	84	PRIVE	No	62	0	0
0	0	VATS or ro	84	PRIVE	No	71	1	0
0	0	VATS or ro	84	PRIVE	No	67	1	0
0	0	VATS or ro	84	PRIVE	No	62	0	0
0	0	VATS or ro	84	PRIVE	No	73	0	0
0	0	VATS or ro	84	PRIVE	No	58	0	0
0	0	VATS or ro	84	PRIVE	No	55	0	0
0	0	VATS or ro	84	PRIVE	No	65	0	0
0	0	VATS or ro	84	PRIVE	No	61	0	0
0	1	Thoracotoı	84	PRIVE	No	57	0	0
0	0	VATS or ro	84	PRIVE	No	53	0	0
0	0	VATS or ro	84	PRIVE	No	71	0	0
0	0	Thoracotoı	84	PRIVE	No	68	1	1
0	0	VATS or ro	84	PRIVE	No	71	0	0
0	0	VATS or ro	84	PRIVE	No	75	0	0
0	0	VATS or ro	84	PRIVE	No	76	0	0
0	1	Thoracotoı	84	PRIVE	No	67	0	0
0	1	Thoracotoı	84	PRIVE	No	77	0	0
0	0	VATS or ro	84	PRIVE	No	66	0	0
0	0	VATS or ro	84	PRIVE	No	65	0	0
0	0	VATS or ro	84	PRIVE	No	66	0	0

0	0	VATS or ro	84	PRIVE	No	66	0	0
0	0	Thoracotoı	84	PRIVE	No	62	0	0
0	0	Thoracotoı	84	PRIVE	No	46	0	0
0	0	Thoracotoı	84	PRIVE	No	50	0	0
0	0	VATS or ro	84	PRIVE	No	58	1	1
0	0	VATS or ro	84	PRIVE	No	65	0	0
0	0	VATS or ro	84	PRIVE	No	66	0	0
0	0	Thoracotoı	84	PRIVE	No	67	0	0
0	0	VATS or ro	84	PRIVE	No	60	0	0
0	0	VATS or ro	84	PRIVE	No	78	0	0
0	0	VATS or ro	84	PRIVE	No	68	0	1
0	0	VATS or ro	84	PRIVE	No	50	0	0
0	0	VATS or ro	84	PRIVE	No	63	0	0
0	0	Thoracotoı	84	PRIVE	No	52	0	0
0	0	VATS or ro	84	PRIVE	No	77	0	0
0	0	Thoracotoı	84	PRIVE	No	63	0	0
0	0	Thoracotoı	84	PRIVE	No	80	0	1
0	0	VATS or ro	84	PRIVE	No	62	0	0
0	0	VATS or ro	84	PRIVE	No	65	0	0
0	0	VATS or ro	84	PRIVE	No	69	0	0
0	0	VATS or ro	84	PRIVE	No	76	0	0
0	0	VATS or ro	84	PRIVE	No	75	0	0
0	0	VATS or ro	84	PRIVE	No	63	1	1
0	0	Thoracotoı	84	PRIVE	No	50	0	0
0	0	VATS or ro	84	PRIVE	No	40	0	0
0	0	VATS or ro	84	PRIVE	No	68	0	1
0	0	VATS or ro	84	PRIVE	No	59	0	1
0	0	VATS or ro	84	PRIVE	No	79	0	0
0	0	VATS or ro	84	PRIVE	No	64	0	0
0	0	VATS or ro	84	PRIVE	No	63	0	0
0	0	VATS or ro	84	PRIVE	No	47	0	1
0	0	VATS or ro	84	PRIVE	No	84	0	0
0	0	VATS or ro	84	PRIVE	No	56	0	0
0	0	VATS or ro	84	PRIVE	No	63	0	0
0	0	VATS or ro	84	PRIVE	No	51	1	1
0	0	VATS or ro	84	PRIVE	No	67	0	1
0	0	VATS or ro	84	PRIVE	No	74	0	0
0	0	Thoracotoı	84	PRIVE	No	77	0	0
0	0	VATS or ro	84	PRIVE	No	43	0	0
0	0	VATS or ro	84	PRIVE	No	76	0	0
1	0	Thoracotoı	144	CHU	No	74	1	0
1	0	Thoracotoı	144	CHU	No	59	1	0
1	0	Thoracotoı	144	CHU	No	65	1	0
1	0	VATS or ro	144	CHU	No	67	0	0
1	0	Thoracotoı	144	CHU	No	59	0	0
1	0	VATS or ro	144	CHU	No	67	0	0
1	0	Thoracotoı	144	CHU	No	79	0	0
1	0	VATS or ro	144	CHU	No	76	0	0
1	1	Thoracotoı	144	CHU	No	68	0	0
1	0	Thoracotoı	144	CHU	No	60	0	1
1	0	Thoracotoı	144	CHU	No	74	0	0
1	0	Thoracotoı	144	CHU	No	79	0	0
1	0	Thoracotoı	144	CHU	No	66	1	0
1	0	Thoracotoı	144	CHU	No	68	0	0
1	0	VATS or ro	144	CHU	No	45	0	0
1	0	Thoracotoı	144	CHU	No	79	1	1

1	0	Thoracotoı	144	CHU	No	80	0	0
1	0	Thoracotoı	144	CHU	No	79	0	0
1	0	VATS or rc	144	CHU	No	56	0	0
1	0	VATS or rc	144	CHU	No	68	0	0
1	0	Thoracotoı	144	CHU	No	68	0	0
1	0	VATS or rc	144	CHU	No	81	0	0
1	1	Thoracotoı	144	CHU	No	55	0	0
1	0	VATS or rc	144	CHU	No	74	0	0
1	0	Thoracotoı	144	CHU	No	59	0	0
1	0	VATS or rc	144	CHU	No	63	0	0
1	0	VATS or rc	144	CHU	No	66	1	0
1	0	Thoracotoı	144	CHU	No	63	0	0
1	0	VATS or rc	144	CHU	No	69	1	0
1	0	Thoracotoı	144	CHU	No	64	0	0
1	0	Thoracotoı	144	CHU	No	63	0	0
1	0	VATS or rc	144	CHU	No	70	1	0
1	0	Thoracotoı	144	CHU	No	64	0	0
1	0	VATS or rc	144	CHU	No	60	0	0
1	0	VATS or rc	144	CHU	No	66	0	1
1	1	Thoracotoı	144	CHU	No	67	0	0
1	0	VATS or rc	144	CHU	No	67	0	0
1	0	VATS or rc	144	CHU	No	72	1	0
1	0	Thoracotoı	144	CHU	No	61	1	1
1	1	Thoracotoı	144	CHU	No	59	0	1
1	0	VATS or rc	144	CHU	No	56	0	0
1	0	Thoracotoı	144	CHU	No	68	0	0
1	0	Thoracotoı	144	CHU	No	66	1	0
1	0	VATS or rc	144	CHU	No	73	0	0
1	0	Thoracotoı	144	CHU	No	69	1	0
1	0	Thoracotoı	144	CHU	No	66	0	0
1	0	VATS or rc	144	CHU	No	75	1	0
1	0	Thoracotoı	144	CHU	No	41	0	0
1	0	Thoracotoı	144	CHU	No	56	0	0
1	0	VATS or rc	144	CHU	No	72	0	0
1	0	Thoracotoı	144	CHU	No	61	0	1
1	0	VATS or rc	144	CHU	No	56	1	0
1	0	Thoracotoı	144	CHU	No	44	0	0
1	0	Thoracotoı	144	CHU	No	60	0	0
1	0	Thoracotoı	144	CHU	No	66	0	0
1	0	Thoracotoı	144	CHU	No	63	0	0
1	0	Thoracotoı	144	CHU	No	62	0	0
1	0	Thoracotoı	144	CHU	No	65	1	0
1	0	Thoracotoı	144	CHU	No	81	0	0
1	0	Thoracotoı	144	CHU	No	64	0	0
1	0	VATS or rc	144	CHU	No	60	0	0
1	0	Thoracotoı	144	CHU	No	59	0	0
1	0	VATS or rc	144	CHU	Yes	56	0	0
1	0	Thoracotoı	144	CHU	No	66	1	0
1	0	Thoracotoı	144	CHU	No	62	0	0
1	0	Thoracotoı	144	CHU	No	66	0	0
1	1	Thoracotoı	144	CHU	Yes	68	0	0
1	0	VATS or rc	144	CHU	No	59	1	0
1	0	Thoracotoı	144	CHU	No	60	1	0
1	0	VATS or rc	144	CHU	No	73	1	0
1	1	Thoracotoı	144	CHU	No	55	0	0
1	0	VATS or rc	144	CHU	No	63	1	0

1	0	Thoracotoı	144	CHU	No	80	0	0
1	0	Thoracotoı	144	CHU	No	73	0	0
1	0	VATS or rc	144	CHU	No	73	0	0
1	0	Thoracotoı	144	CHU	No	78	0	0
1	0	Thoracotoı	144	CHU	No	80	0	0
1	0	Thoracotoı	144	CHU	No	80	1	0
1	0	VATS or rc	144	CHU	No	65	1	0
1	0	VATS or rc	144	CHU	No	67	0	0
1	0	Thoracotoı	144	CHU	No	64	0	0
1	0	Thoracotoı	144	CHU	No	61	0	1
1	0	Thoracotoı	144	CHU	No	62	0	0
1	0	Thoracotoı	144	CHU	No	56	0	0
1	0	VATS or rc	144	CHU	No	74	0	0
1	0	VATS or rc	144	CHU	No	77	1	0
1	0	Thoracotoı	144	CHU	No	78	0	0
1	0	Thoracotoı	144	CHU	No	69	1	0
1	0	VATS or rc	144	CHU	Yes	81	0	0
1	1	Thoracotoı	144	CHU	No	59	0	0
1	0	Thoracotoı	144	CHU	No	68	0	0
1	0	Thoracotoı	144	CHU	No	70	0	0
1	0	Thoracotoı	144	CHU	No	59	1	0
1	0	Thoracotoı	144	CHU	No	60	0	0
1	0	Thoracotoı	144	CHU	No	50	0	1
1	0	Thoracotoı	144	CHU	No	55	0	0
1	0	Thoracotoı	144	CHU	No	65	1	0
1	0	Thoracotoı	144	CHU	No	54	0	0
1	0	Thoracotoı	144	CHU	No	80	0	0
1	0	Thoracotoı	144	CHU	No	59	1	0
1	0	Thoracotoı	144	CHU	No	47	0	0
1	0	VATS or rc	144	CHU	No	72	1	0
1	0	Thoracotoı	144	CHU	No	33	0	0
1	0	Thoracotoı	144	CHU	No	77	0	0
1	0	Thoracotoı	144	CHU	No	61	0	0
1	0	VATS or rc	144	CHU	No	53	0	0
1	0	Thoracotoı	144	CHU	No	78	0	1
1	0	Thoracotoı	144	CHU	No	79	0	0
1	0	Thoracotoı	144	CHU	No	60	0	0
1	0	Thoracotoı	144	CHU	No	50	0	0
1	0	VATS or rc	144	CHU	No	59	0	0
1	1	Thoracotoı	144	CHU	No	75	0	0
1	0	Thoracotoı	144	CHU	No	66	0	0
1	0	VATS or rc	144	CHU	No	81	1	0
1	0	Thoracotoı	144	CHU	No	67	0	0
1	0	Thoracotoı	144	CHU	No	55	0	0
1	0	VATS or rc	144	CHU	No	69	0	0
1	1	Thoracotoı	144	CHU	No	78	0	0
1	0	Thoracotoı	144	CHU	No	70	1	0
1	0	Thoracotoı	144	CHU	No	69	0	0
1	0	VATS or rc	144	CHU	No	73	0	0
1	0	VATS or rc	144	CHU	No	55	0	0
1	0	VATS or rc	144	CHU	No	70	0	0
1	0	Thoracotoı	144	CHU	No	67	0	0
1	1	Thoracotoı	144	CHU	No	64	0	0
1	0	Thoracotoı	144	CHU	No	33	0	0
1	0	VATS or rc	144	CHU	No	62	0	0
1	0	Thoracotoı	144	CHU	No	80	0	0

1	1	Thoracotoı	144	CHU	No	65	0	0
1	0	VATS or rc	144	CHU	No	65	0	0
1	0	VATS or rc	144	CHU	No	61	0	0
1	0	Thoracotoı	144	CHU	No	74	0	0
1	0	Thoracotoı	144	CHU	No	60	0	0
1	0	Thoracotoı	144	CHU	No	66	0	0
1	0	VATS or rc	144	CHU	No	70	0	0
1	0	Thoracotoı	144	CHU	No	79	0	0
1	0	VATS or rc	144	CHU	No	73	0	0
1	0	VATS or rc	144	CHU	No	57	0	0
1	0	Thoracotoı	144	CHU	No	63	0	0
1	0	Thoracotoı	144	CHU	No	66	0	0
1	0	Thoracotoı	144	CHU	No	64	0	0
1	0	Thoracotoı	144	CHU	No	64	0	0
1	0	Thoracotoı	144	CHU	No	72	0	0
1	1	Thoracotoı	144	CHU	No	64	0	0
1	0	Thoracotoı	144	CHU	No	67	0	0
1	0	Thoracotoı	144	CHU	No	74	1	0
1	0	Thoracotoı	144	CHU	No	65	0	1
1	0	VATS or rc	144	CHU	Yes	66	0	0
1	0	VATS or rc	144	CHU	No	57	0	0
1	0	Thoracotoı	144	CHU	No	66	0	0
1	0	Thoracotoı	144	CHU	No	76	0	0
1	0	Thoracotoı	144	CHU	No	71	1	0
1	0	Thoracotoı	144	CHU	No	52	0	0
1	1	Thoracotoı	144	CHU	No	49	0	0
1	0	VATS or rc	144	CHU	No	59	1	0
1	0	VATS or rc	144	CHU	No	58	0	0
1	0	Thoracotoı	144	CHU	No	62	0	0
1	0	Thoracotoı	144	CHU	No	64	1	0
1	0	Thoracotoı	144	CHU	No	70	0	0
1	0	Thoracotoı	144	CHU	No	59	0	0
1	1	Thoracotoı	144	CHU	No	60	0	0
1	0	Thoracotoı	144	CHU	No	74	0	0
1	0	Thoracotoı	144	CHU	No	60	0	0
1	0	VATS or rc	144	CHU	No	67	0	0
1	0	Thoracotoı	144	CHU	Yes	59	0	0
1	0	Thoracotoı	144	CHU	No	57	0	0
1	0	VATS or rc	144	CHU	No	78	0	0
1	1	Thoracotoı	144	CHU	No	75	0	0
1	0	Thoracotoı	144	CHU	No	63	0	0
1	0	Thoracotoı	144	CHU	No	51	0	0
1	0	Thoracotoı	144	CHU	No	64	0	0
1	0	VATS or rc	144	CHU	No	80	0	0
1	0	VATS or rc	144	CHU	No	51	0	0
1	0	Thoracotoı	144	CHU	No	45	0	0
1	0	VATS or rc	144	CHU	No	53	0	0
1	0	Thoracotoı	144	CHU	No	70	0	0
1	0	Thoracotoı	144	CHU	No	61	0	0
1	0	Thoracotoı	144	CHU	No	36	0	0
1	0	Thoracotoı	144	CHU	No	70	1	0
1	0	Thoracotoı	144	CHU	No	59	0	0
1	0	Thoracotoı	144	CHU	No	68	0	0
1	0	Thoracotoı	144	CHU	No	77	1	0
1	1	Thoracotoı	144	CHU	No	45	0	0
1	0	Thoracotoı	144	CHU	No	57	0	0

1	0	Thoracotoı	144	CHU	No	60	0	0
1	0	Thoracotoı	144	CHU	No	59	0	0
1	0	VATS or rc	144	CHU	No	67	0	0
1	0	VATS or rc	144	CHU	No	51	0	0
1	0	VATS or rc	144	CHU	No	54	1	0
1	0	Thoracotoı	144	CHU	No	78	0	0
1	0	Thoracotoı	144	CHU	No	67	0	0
1	0	Thoracotoı	144	CHU	No	70	0	0
1	0	Thoracotoı	144	CHU	No	41	0	0
1	0	Thoracotoı	144	CHU	No	82	0	0
1	0	VATS or rc	144	CHU	No	62	0	0
1	0	Thoracotoı	144	CHU	No	66	0	0
1	0	Thoracotoı	144	CHU	No	69	0	0
1	0	VATS or rc	144	CHU	No	78	1	0
1	0	VATS or rc	144	CHU	No	71	0	0
1	0	Thoracotoı	144	CHU	No	83	0	0
1	0	Thoracotoı	144	CHU	No	54	0	0
1	0	Thoracotoı	144	CHU	No	55	0	0
1	0	VATS or rc	144	CHU	No	61	0	0
1	0	Thoracotoı	144	CHU	No	65	1	0
1	0	VATS or rc	144	CHU	No	51	0	0
1	0	Thoracotoı	144	CHU	No	53	0	0
1	0	Thoracotoı	144	CHU	No	62	0	0
1	0	Thoracotoı	144	CHU	No	76	1	0
1	0	Thoracotoı	144	CHU	No	69	0	0
1	0	Thoracotoı	144	CHU	No	80	0	0
1	0	Thoracotoı	144	CHU	No	77	0	0
1	0	Thoracotoı	144	CHU	No	50	0	0
1	0	Thoracotoı	144	CHU	No	67	0	0
1	0	Thoracotoı	144	CHU	No	69	0	0
1	0	Thoracotoı	144	CHU	No	69	0	1
1	0	Thoracotoı	144	CHU	No	68	0	0
1	0	VATS or rc	144	CHU	No	57	0	1
1	0	VATS or rc	144	CHU	No	68	0	1
1	1	Thoracotoı	144	CHU	No	68	0	0
1	0	Thoracotoı	144	CHU	No	73	0	0
1	0	Thoracotoı	144	CHU	No	82	0	0
1	0	Thoracotoı	144	CHU	No	76	0	0
1	0	Thoracotoı	144	CHU	No	68	0	0
1	0	Thoracotoı	144	CHU	No	57	0	0
1	0	Thoracotoı	144	CHU	No	78	0	0
1	0	Thoracotoı	144	CHU	No	61	0	0
1	0	Thoracotoı	144	CHU	No	76	1	0
1	0	Thoracotoı	144	CHU	No	65	0	0
1	0	VATS or rc	144	CHU	No	61	0	0
1	1	Thoracotoı	144	CHU	No	70	0	0
1	0	Thoracotoı	144	CHU	No	69	1	0
1	0	Thoracotoı	144	CHU	No	72	0	0
1	0	Thoracotoı	144	CHU	No	56	0	0
1	0	VATS or rc	144	CHU	No	78	0	0
1	0	Thoracotoı	144	CHU	No	63	0	0
1	0	VATS or rc	144	CHU	No	73	1	0
1	0	Thoracotoı	144	CHU	No	72	0	0
1	0	VATS or rc	144	CHU	No	60	0	0
1	0	Thoracotoı	144	CHU	No	59	0	0
1	0	Thoracotoı	144	CHU	No	76	0	0

1	0	VATS or rc	144	CHU	No	75	0	0
1	0	Thoracotoı	144	CHU	No	66	0	0
1	0	Thoracotoı	144	CHU	No	65	0	0
1	0	VATS or rc	144	CHU	No	50	0	1
1	0	Thoracotoı	144	CHU	No	61	0	0
1	0	VATS or rc	144	CHU	No	71	0	0
1	0	VATS or rc	144	CHU	No	61	0	0
1	0	VATS or rc	144	CHU	No	56	0	0
1	0	VATS or rc	144	CHU	No	70	0	0
1	0	VATS or rc	144	CHU	No	55	0	0
1	0	VATS or rc	144	CHU	No	60	0	0
1	0	Thoracotoı	144	CHU	No	72	0	0
1	0	Thoracotoı	144	CHU	No	67	0	0
1	0	Thoracotoı	144	CHU	No	61	0	0
1	0	VATS or rc	144	CHU	No	50	0	0
1	0	Thoracotoı	144	CHU	No	67	0	0
1	0	VATS or rc	144	CHU	No	60	0	0
1	0	VATS or rc	144	CHU	No	68	0	0
1	0	Thoracotoı	144	CHU	No	64	0	0
1	0	Thoracotoı	144	CHU	No	67	1	0
1	0	Thoracotoı	144	CHU	No	77	0	0
1	0	Thoracotoı	144	CHU	No	66	0	0
1	0	VATS or rc	144	CHU	No	75	0	0
1	0	Thoracotoı	144	CHU	No	80	0	0
1	0	Thoracotoı	144	CHU	No	64	0	1
1	0	Thoracotoı	144	CHU	No	65	1	0
1	0	Thoracotoı	144	CHU	Yes	60	0	0
1	0	VATS or rc	144	CHU	No	64	0	0
1	0	Thoracotoı	144	CHU	No	58	0	0
1	0	VATS or rc	144	CHU	No	77	0	0
1	0	Thoracotoı	144	CHU	No	53	0	0
1	0	Thoracotoı	144	CHU	No	59	0	0
1	0	Thoracotoı	144	CHU	No	58	0	0
1	0	VATS or rc	144	CHU	No	60	0	0
1	0	Thoracotoı	144	CHU	No	56	0	0
1	0	VATS or rc	144	CHU	No	68	0	0
1	0	Thoracotoı	144	CHU	No	58	0	0
1	0	VATS or rc	144	CHU	No	60	0	0
1	0	VATS or rc	144	CHU	No	73	0	0
1	0	Thoracotoı	144	CHU	No	46	0	0
1	0	Thoracotoı	144	CHU	No	53	1	1
1	0	VATS or rc	144	CHU	No	54	0	0
1	0	Thoracotoı	144	CHU	No	80	1	0
1	0	Thoracotoı	144	CHU	No	64	0	0
1	0	Thoracotoı	144	CHU	No	58	1	0
1	0	VATS or rc	144	CHU	No	72	0	0
1	0	Thoracotoı	144	CHU	No	69	1	0
1	1	Thoracotoı	144	CHU	No	72	0	0
1	0	Thoracotoı	144	CHU	No	72	0	0
1	0	Thoracotoı	144	CHU	No	70	1	0
1	0	Thoracotoı	144	CHU	No	66	0	0
1	0	VATS or rc	144	CHU	No	59	0	0
1	0	VATS or rc	144	CHU	No	65	0	0
1	0	Thoracotoı	144	CHU	No	72	0	0
1	0	Thoracotoı	144	CHU	No	70	0	0
1	0	VATS or rc	144	CHU	No	64	0	0

1	0	Thoracotoı	144	CHU	No	74	0	0
1	0	Thoracotoı	144	CHU	No	59	0	0
1	0	VATS or rc	144	CHU	No	62	0	0
1	0	Thoracotoı	144	CHU	No	60	0	0
1	1	Thoracotoı	144	CHU	No	56	0	0
1	0	Thoracotoı	144	CHU	No	67	0	0
1	0	Thoracotoı	144	CHU	No	80	0	0
1	0	VATS or rc	144	CHU	No	74	0	0
1	1	Thoracotoı	144	CHU	No	74	0	0
1	1	Thoracotoı	144	CHU	No	73	0	0
1	0	VATS or rc	144	CHU	No	85	0	0
1	1	Thoracotoı	144	CHU	No	69	0	0
1	0	VATS or rc	144	CHU	No	62	0	0
1	0	Thoracotoı	144	CHU	No	56	0	0
1	1	Thoracotoı	144	CHU	No	55	0	0
1	0	Thoracotoı	144	CHU	No	52	0	0
1	0	Thoracotoı	144	CHU	No	80	0	0
1	0	VATS or rc	144	CHU	No	81	0	0
1	0	Thoracotoı	144	CHU	No	64	0	0
1	0	VATS or rc	144	CHU	No	66	0	0
1	0	Thoracotoı	144	CHU	No	53	0	0
1	0	Thoracotoı	144	CHU	No	59	0	0
1	0	VATS or rc	144	CHU	No	62	0	0
1	0	Thoracotoı	144	CHU	No	81	0	0
1	1	Thoracotoı	144	CHU	No	50	0	0
1	0	Thoracotoı	144	CHU	No	80	0	0
1	0	Thoracotoı	144	CHU	No	71	0	0
1	0	Thoracotoı	144	CHU	No	75	0	0
1	0	Thoracotoı	144	CHU	No	78	0	0
1	0	Thoracotoı	144	CHU	No	69	0	0
1	0	Thoracotoı	144	CHU	No	66	0	1
1	0	VATS or rc	144	CHU	No	70	0	0
1	0	Thoracotoı	144	CHU	No	64	0	0
1	0	Thoracotoı	144	CHU	No	73	1	0
1	0	Thoracotoı	144	CHU	No	47	0	0
1	0	Thoracotoı	144	CHU	No	59	0	0
1	1	Thoracotoı	144	CHU	No	55	0	0
1	1	Thoracotoı	144	CHU	No	78	0	0
1	0	Thoracotoı	144	CHU	No	65	0	0
1	0	Thoracotoı	144	CHU	No	48	0	0
1	0	VATS or rc	144	CHU	No	58	0	0
1	0	Thoracotoı	144	CHU	No	62	1	0
1	0	Thoracotoı	144	CHU	No	69	0	0
1	0	VATS or rc	144	CHU	No	73	0	0
1	0	VATS or rc	144	CHU	No	36	0	0
1	0	Thoracotoı	144	CHU	No	48	0	1
1	0	Thoracotoı	144	CHU	No	68	0	0
1	0	Thoracotoı	144	CHU	No	62	0	0
1	0	Thoracotoı	144	CHU	No	69	1	0
1	0	Thoracotoı	144	CHU	No	77	0	0
1	0	Thoracotoı	144	CHU	No	74	0	0
1	0	Thoracotoı	144	CHU	No	65	0	1
1	0	VATS or rc	144	CHU	No	79	0	0
1	0	VATS or rc	144	CHU	No	68	1	0
1	0	Thoracotoı	144	CHU	No	66	0	0
1	0	Thoracotoı	144	CHU	No	62	1	0

1	0	Thoracotoı	144	CHU	No	83	0	0
1	0	Thoracotoı	144	CHU	No	61	0	1
1	0	Thoracotoı	144	CHU	No	68	0	0
1	0	VATS or rc	144	CHU	No	50	0	0
1	0	Thoracotoı	144	CHU	No	73	0	0
1	0	VATS or rc	144	CHU	No	67	0	0
1	0	Thoracotoı	144	CHU	No	66	1	0
1	0	Thoracotoı	144	CHU	No	67	0	0
1	0	Thoracotoı	144	CHU	No	60	0	0
1	0	Thoracotoı	144	CHU	No	71	0	1
1	0	VATS or rc	144	CHU	No	70	0	0
1	0	Thoracotoı	144	CHU	No	39	0	0
1	0	VATS or rc	144	CHU	No	72	0	0
1	0	Thoracotoı	144	CHU	No	71	0	1
1	0	Thoracotoı	144	CHU	No	72	0	0
1	0	Thoracotoı	144	CHU	No	70	0	0
1	0	Thoracotoı	144	CHU	Yes	80	0	0
1	0	Thoracotoı	144	CHU	No	55	0	1
1	1	Thoracotoı	144	CHU	No	67	0	1
1	0	Thoracotoı	144	CHU	No	67	0	0
1	0	Thoracotoı	144	CHU	No	84	0	0
1	0	Thoracotoı	144	CHU	No	21	0	0
1	0	Thoracotoı	144	CHU	No	73	0	0
1	0	Thoracotoı	144	CHU	No	74	0	0
1	0	VATS or rc	144	CHU	No	74	0	0
1	0	Thoracotoı	144	CHU	No	46	0	0
1	0	Thoracotoı	144	CHU	No	55	0	0
1	0	VATS or rc	144	CHU	No	59	0	0
1	0	VATS or rc	144	CHU	No	77	0	0
1	0	Thoracotoı	144	CHU	No	75	0	0
1	0	Thoracotoı	144	CHU	No	64	0	0
1	0	Thoracotoı	144	CHU	No	60	0	1
1	1	Thoracotoı	144	CHU	Yes	55	0	1
1	0	VATS or rc	144	CHU	No	68	0	0
1	0	Thoracotoı	144	CHU	No	50	1	0
1	0	Thoracotoı	144	CHU	No	60	0	0
1	1	Thoracotoı	144	CHU	No	54	0	0
1	0	VATS or rc	144	CHU	No	43	0	0
1	0	Thoracotoı	144	CHU	No	74	0	0
1	0	Thoracotoı	144	CHU	No	75	0	0
1	0	Thoracotoı	144	CHU	No	59	0	0
1	0	Thoracotoı	144	CHU	No	71	0	0
1	0	Thoracotoı	144	CHU	No	51	0	0
1	0	VATS or rc	144	CHU	No	58	0	1
1	0	Thoracotoı	144	CHU	No	58	0	0
1	0	VATS or rc	144	CHU	No	66	0	0
1	0	Thoracotoı	144	CHU	No	77	0	0
1	0	Thoracotoı	144	CHU	No	58	0	1
1	1	Thoracotoı	144	CHU	No	51	0	1
1	0	Thoracotoı	144	CHU	No	61	0	0
1	1	Thoracotoı	144	CHU	No	77	1	0
1	0	Thoracotoı	144	CHU	No	57	0	0
1	0	VATS or rc	144	CHU	No	66	1	0
1	0	Thoracotoı	144	CHU	No	62	0	0
1	0	Thoracotoı	144	CHU	No	73	0	0
1	0	Thoracotoı	144	CHU	No	66	0	0

1	0	Thoracotoı	144	CHU	No	78	0	0
1	0	Thoracotoı	144	CHU	No	69	0	0
1	0	Thoracotoı	144	CHU	No	67	1	0
1	0	Thoracotoı	144	CHU	No	57	0	0
1	0	VATS or rc	144	CHU	No	68	0	0
1	0	Thoracotoı	144	CHU	No	60	0	0
1	0	VATS or rc	144	CHU	No	60	0	0
1	0	VATS or rc	144	CHU	No	64	0	0
1	1	Thoracotoı	144	CHU	No	64	0	0
1	0	Thoracotoı	144	CHU	No	51	0	0
1	0	VATS or rc	144	CHU	No	55	1	0
1	0	VATS or rc	144	CHU	No	75	0	0
1	0	VATS or rc	144	CHU	No	61	0	0
1	0	VATS or rc	144	CHU	No	52	0	0
1	1	Thoracotoı	144	CHU	No	64	0	0
1	0	VATS or rc	144	CHU	No	69	0	0
1	0	Thoracotoı	144	CHU	No	68	0	0
1	0	Thoracotoı	144	CHU	Yes	52	0	0
1	0	Thoracotoı	144	CHU	No	67	0	0
1	1	Thoracotoı	144	CHU	No	60	1	0
1	0	VATS or rc	144	CHU	No	67	0	0
1	0	Thoracotoı	144	CHU	No	57	0	0
1	0	VATS or rc	144	CHU	No	44	0	0
1	0	VATS or rc	144	CHU	No	67	1	0
1	0	Thoracotoı	144	CHU	No	55	0	0
1	0	Thoracotoı	144	CHU	No	54	0	0
1	0	VATS or rc	144	CHU	No	56	0	0
1	0	Thoracotoı	144	CHU	No	66	0	0
1	0	VATS or rc	144	CHU	No	65	0	1
1	1	Thoracotoı	144	CHU	No	68	0	0
1	0	Thoracotoı	144	CHU	No	74	0	0
1	0	VATS or rc	144	CHU	No	57	0	0
1	0	VATS or rc	144	CHU	No	64	0	0
1	0	Thoracotoı	144	CHU	No	72	0	0
1	0	VATS or rc	144	CHU	No	71	0	0
1	0	Thoracotoı	144	CHU	No	68	0	0
1	0	Thoracotoı	144	CHU	No	62	0	0
1	0	Thoracotoı	144	CHU	No	80	1	1
1	0	Thoracotoı	144	CHU	No	32	0	0
1	1	Thoracotoı	144	CHU	No	59	0	0
1	0	Thoracotoı	144	CHU	No	65	0	0
1	0	VATS or rc	144	CHU	No	58	0	0
1	0	Thoracotoı	144	CHU	No	63	0	0
1	0	Thoracotoı	144	CHU	No	85	0	0
1	0	Thoracotoı	144	CHU	No	72	0	0
1	1	Thoracotoı	144	CHU	No	66	0	0
1	0	VATS or rc	144	CHU	No	69	1	0
1	0	Thoracotoı	144	CHU	No	65	1	0
1	0	Thoracotoı	144	CHU	No	42	0	1
1	0	VATS or rc	144	CHU	No	56	0	0
1	0	Thoracotoı	144	CHU	No	47	0	0
1	0	Thoracotoı	144	CHU	No	68	0	0
1	0	Thoracotoı	144	CHU	No	70	0	0
1	1	Thoracotoı	144	CHU	No	79	0	0
1	0	VATS or rc	144	CHU	No	44	0	0
1	0	Thoracotoı	144	CHU	No	72	1	0

1	0	Thoracotoı	144	CHU	No	71	0	0
1	0	VATS or rc	144	CHU	No	52	0	0
1	0	Thoracotoı	144	CHU	No	72	0	0
1	0	Thoracotoı	144	CHU	No	62	0	0
1	0	Thoracotoı	144	CHU	No	57	0	1
1	0	Thoracotoı	144	CHU	No	69	0	0
1	0	VATS or rc	144	CHU	No	75	0	0
1	0	VATS or rc	144	CHU	No	80	1	0
1	0	Thoracotoı	144	CHU	No	69	0	0
1	0	Thoracotoı	144	CHU	No	67	0	0
1	1	Thoracotoı	144	CHU	No	66	1	0
1	0	VATS or rc	144	CHU	No	63	0	0
1	0	Thoracotoı	144	CHU	No	67	0	0
1	0	Thoracotoı	144	CHU	No	77	0	0
1	0	Thoracotoı	144	CHU	No	77	0	0
1	0	Thoracotoı	144	CHU	No	78	1	0
1	1	Thoracotoı	144	CHU	No	65	0	1
1	0	Thoracotoı	144	CHU	No	57	0	0
1	0	VATS or rc	144	CHU	No	56	0	0
1	0	Thoracotoı	144	CHU	No	72	1	0
1	0	Thoracotoı	144	CHU	No	55	0	0
1	0	VATS or rc	144	CHU	No	58	0	0
1	0	Thoracotoı	144	CHU	No	73	0	0
1	0	Thoracotoı	144	CHU	No	64	0	0
1	0	Thoracotoı	144	CHU	No	72	0	1
1	0	VATS or rc	144	CHU	No	49	0	1
1	0	VATS or rc	144	CHU	No	54	0	0
1	0	Thoracotoı	144	CHU	No	65	0	0
1	0	Thoracotoı	144	CHU	No	74	0	0
1	0	Thoracotoı	144	CHU	No	68	0	0
1	0	VATS or rc	144	CHU	No	69	0	0
1	0	Thoracotoı	144	CHU	No	58	0	0
1	0	Thoracotoı	144	CHU	No	72	0	0
1	0	VATS or rc	144	CHU	No	81	0	0
1	0	Thoracotoı	144	CHU	No	59	0	0
0	0	VATS or rc	28	PRIVE	No	48	0	0
0	0	VATS or rc	28	PRIVE	No	85	0	0
0	0	Thoracotoı	28	PRIVE	No	74	0	1
0	0	Thoracotoı	28	PRIVE	No	72	0	1
0	0	Thoracotoı	28	PRIVE	No	77	0	0
0	0	VATS or rc	28	PRIVE	No	63	0	0
0	0	VATS or rc	28	PRIVE	No	85	0	0
0	0	Thoracotoı	28	PRIVE	No	52	0	1
0	0	VATS or rc	28	PRIVE	No	52	0	0
0	0	Thoracotoı	28	PRIVE	No	72	0	1
0	0	Thoracotoı	28	PRIVE	No	79	1	0
0	0	VATS or rc	28	PRIVE	No	64	0	0
0	1	Thoracotoı	28	PRIVE	No	57	1	0
0	0	VATS or rc	28	PRIVE	No	64	0	0
0	0	VATS or rc	28	PRIVE	No	49	0	0
0	0	VATS or rc	28	PRIVE	No	69	1	0
0	0	Thoracotoı	28	PRIVE	No	79	1	0
0	0	VATS or rc	28	PRIVE	No	81	0	0
0	0	Thoracotoı	34	PRIVE	No	69	0	0
0	0	Thoracotoı	34	PRIVE	No	76	0	0
0	1	Thoracotoı	34	PRIVE	No	69	0	0

0	0	Thoracotoı	34	PRIVE	No	69	0	1
0	0	Thoracotoı	34	PRIVE	No	65	0	1
0	0	Thoracotoı	34	PRIVE	No	55	0	0
0	0	Thoracotoı	34	PRIVE	No	61	0	0
0	1	Thoracotoı	34	PRIVE	No	67	0	0
0	0	Thoracotoı	34	PRIVE	No	75	0	0
0	1	Thoracotoı	34	PRIVE	No	67	0	0
0	0	Thoracotoı	34	PRIVE	No	79	0	0
0	0	Thoracotoı	34	PRIVE	No	72	0	0
0	0	VATS or ro	34	PRIVE	No	58	0	0
0	0	Thoracotoı	34	PRIVE	No	70	0	0
0	0	VATS or ro	34	PRIVE	No	45	0	0
0	0	Thoracotoı	34	PRIVE	No	83	0	0
0	0	Thoracotoı	34	PRIVE	No	75	0	0
0	0	Thoracotoı	34	PRIVE	No	61	0	0
0	0	Thoracotoı	34	PRIVE	No	67	0	1
0	0	Thoracotoı	34	PRIVE	No	58	0	0
0	0	Thoracotoı	34	PRIVE	Yes	76	0	0
0	0	Thoracotoı	34	PRIVE	No	58	0	0
0	0	Thoracotoı	34	PRIVE	No	80	0	0
0	0	Thoracotoı	34	PRIVE	No	60	0	0
0	0	Thoracotoı	34	PRIVE	No	59	0	0
0	0	Thoracotoı	34	PRIVE	No	74	0	0
0	0	Thoracotoı	34	PRIVE	No	80	0	0
0	0	Thoracotoı	34	PRIVE	No	70	0	0
0	0	Thoracotoı	34	PRIVE	No	78	0	0
0	0	Thoracotoı	34	PRIVE	No	80	0	0
0	0	VATS or ro	34	PRIVE	No	78	0	0
0	0	Thoracotoı	34	PRIVE	No	72	0	0
0	0	Thoracotoı	34	PRIVE	No	72	0	0
0	0	Thoracotoı	34	PRIVE	No	79	0	0
0	1	Thoracotoı	34	PRIVE	No	78	0	0
0	0	Thoracotoı	34	PRIVE	No	61	0	0
0	0	Thoracotoı	34	PRIVE	No	67	0	0
0	0	Thoracotoı	34	PRIVE	No	64	0	0
0	0	VATS or ro	34	PRIVE	No	59	0	0
0	0	Thoracotoı	34	PRIVE	No	76	0	0
0	0	Thoracotoı	34	PRIVE	No	77	0	0
0	0	Thoracotoı	34	PRIVE	No	66	0	0
0	0	Thoracotoı	34	PRIVE	No	69	0	1
0	0	VATS or ro	34	PRIVE	No	62	0	0
0	0	Thoracotoı	34	PRIVE	No	56	0	0
0	0	Thoracotoı	34	PRIVE	No	59	0	0
0	0	Thoracotoı	34	PRIVE	No	70	0	0
0	0	Thoracotoı	34	PRIVE	No	61	0	1
0	1	Thoracotoı	34	PRIVE	No	67	0	0
0	0	Thoracotoı	34	PRIVE	No	56	0	0
0	0	Thoracotoı	34	PRIVE	No	71	0	0
0	0	Thoracotoı	34	PRIVE	No	58	0	0
0	0	Thoracotoı	34	PRIVE	No	69	0	0
0	0	VATS or ro	34	PRIVE	No	62	0	0
0	1	Thoracotoı	34	PRIVE	No	66	0	0
0	0	Thoracotoı	34	PRIVE	No	69	0	0
0	0	Thoracotoı	34	PRIVE	Yes	65	1	0
0	0	Thoracotoı	34	PRIVE	No	61	0	0
0	0	Thoracotoı	34	PRIVE	No	54	0	0

0	0	Thoracotoı	34	PRIVE	No	56	0	0
0	0	Thoracotoı	34	PRIVE	No	70	0	0
0	0	Thoracotoı	34	PRIVE	No	69	0	0
0	0	Thoracotoı	34	PRIVE	No	69	0	0
0	0	Thoracotoı	34	PRIVE	No	56	0	1
0	1	Thoracotoı	10	CHU	No	51	0	0
0	0	VATS or rc	10	CHU	No	80	0	0
0	0	Thoracotoı	10	CHU	No	37	0	0
0	0	VATS or rc	10	CHU	No	72	0	0
0	0	VATS or rc	10	CHU	No	70	0	0
0	0	VATS or rc	10	CHU	No	55	0	0
0	0	VATS or rc	10	CHU	No	56	0	0
0	0	Thoracotoı	10	CHU	No	70	0	0
0	0	VATS or rc	10	CHU	No	62	0	0
0	0	Thoracotoı	10	CHU	No	52	0	0
0	0	VATS or rc	10	CHU	No	56	0	0
0	0	VATS or rc	10	CHU	No	65	0	0
0	0	VATS or rc	10	CHU	No	76	0	0
0	0	Thoracotoı	10	CHU	No	66	0	0
0	0	Thoracotoı	10	CHU	No	60	0	0
0	0	VATS or rc	10	CHU	No	74	0	0
0	0	VATS or rc	10	CHU	No	77	0	0
0	0	Thoracotoı	10	CHU	No	53	0	0
0	0	Thoracotoı	10	CHU	No	80	0	0
0	0	VATS or rc	10	CHU	No	58	0	0
0	0	VATS or rc	10	CHU	No	73	0	0
0	0	VATS or rc	10	CHU	No	70	0	0
0	0	Thoracotoı	10	CHU	No	58	0	0
0	1	Thoracotoı	10	CHU	No	61	0	0
0	0	VATS or rc	10	CHU	No	35	0	0
0	0	Thoracotoı	10	CHU	No	54	0	0
0	0	Thoracotoı	10	CHU	No	78	0	0
0	1	Thoracotoı	10	CHU	No	66	0	0
0	0	Thoracotoı	10	CHU	No	74	0	0
0	0	VATS or rc	10	CHU	No	51	0	0
0	0	Thoracotoı	10	CHU	No	69	0	0
0	0	Thoracotoı	10	CHU	No	63	0	0
0	0	VATS or rc	10	CHU	No	51	0	0
0	0	VATS or rc	10	CHU	No	79	0	0
0	0	VATS or rc	10	CHU	No	46	0	0
0	0	VATS or rc	10	CHU	No	61	0	0
0	0	VATS or rc	10	CHU	No	52	0	0
0	0	Thoracotoı	10	CHU	No	63	0	0
0	0	VATS or rc	10	CHU	No	70	0	0
0	0	VATS or rc	10	CHU	No	83	0	0
0	0	VATS or rc	10	CHU	No	65	0	0
0	0	Thoracotoı	10	CHU	No	49	0	1
0	0	Thoracotoı	10	CHU	No	64	0	0
1	0	Thoracotoı	94	CHU	No	63	0	0
1	0	VATS or rc	94	CHU	No	72	0	0
1	0	VATS or rc	94	CHU	No	75	0	0
1	0	Thoracotoı	94	CHU	No	75	0	0
1	1	Thoracotoı	94	CHU	Yes	72	0	0
1	0	VATS or rc	94	CHU	No	72	0	0
1	0	VATS or rc	94	CHU	No	56	0	0
1	0	Thoracotoı	94	CHU	No	52	0	0

1	0	VATS or rc	94	CHU	No	61	0	0
1	0	Thoracotoı	94	CHU	No	59	0	0
1	0	VATS or rc	94	CHU	No	70	0	0
1	0	Thoracotoı	94	CHU	No	68	0	0
1	1	Thoracotoı	94	CHU	No	65	1	0
1	0	Thoracotoı	94	CHU	No	54	0	0
1	0	Thoracotoı	94	CHU	No	63	0	0
1	0	Thoracotoı	94	CHU	No	55	0	0
1	0	Thoracotoı	94	CHU	No	73	0	0
1	0	Thoracotoı	94	CHU	No	80	0	0
1	0	Thoracotoı	94	CHU	No	62	0	0
1	0	Thoracotoı	94	CHU	No	61	0	0
1	0	Thoracotoı	94	CHU	No	71	0	0
1	0	Thoracotoı	94	CHU	No	63	0	0
1	0	Thoracotoı	94	CHU	No	65	0	0
1	0	VATS or rc	94	CHU	No	81	0	0
1	0	Thoracotoı	94	CHU	No	59	0	0
1	0	VATS or rc	94	CHU	No	64	0	0
1	0	VATS or rc	94	CHU	No	61	0	0
1	0	Thoracotoı	94	CHU	No	60	0	0
1	1	Thoracotoı	94	CHU	No	71	0	0
1	1	Thoracotoı	94	CHU	No	74	0	0
1	0	Thoracotoı	94	CHU	No	71	0	0
1	0	VATS or rc	94	CHU	No	78	0	0
1	0	Thoracotoı	94	CHU	No	71	0	0
1	0	Thoracotoı	94	CHU	No	66	0	0
1	0	Thoracotoı	94	CHU	No	71	0	0
1	0	Thoracotoı	94	CHU	No	54	0	0
1	0	VATS or rc	94	CHU	No	78	0	0
1	0	Thoracotoı	94	CHU	No	67	0	0
1	0	Thoracotoı	94	CHU	No	57	1	0
1	0	VATS or rc	94	CHU	No	76	1	0
1	0	VATS or rc	94	CHU	No	74	0	0
1	0	VATS or rc	94	CHU	No	58	1	0
1	0	Thoracotoı	94	CHU	No	71	0	0
1	0	Thoracotoı	94	CHU	No	62	0	1
1	0	Thoracotoı	94	CHU	No	69	0	0
1	0	VATS or rc	94	CHU	No	67	0	0
1	0	VATS or rc	94	CHU	No	64	0	0
1	0	Thoracotoı	94	CHU	No	53	0	0
1	0	VATS or rc	94	CHU	No	64	0	0
1	1	Thoracotoı	94	CHU	No	78	0	0
1	1	Thoracotoı	94	CHU	No	57	0	0
1	0	VATS or rc	94	CHU	No	78	0	0
1	0	Thoracotoı	94	CHU	No	62	0	0
1	0	VATS or rc	94	CHU	No	59	0	0
1	0	Thoracotoı	94	CHU	No	71	0	1
1	0	VATS or rc	94	CHU	No	67	0	0
1	0	VATS or rc	94	CHU	No	76	0	0
1	0	Thoracotoı	94	CHU	No	82	0	0
1	0	VATS or rc	94	CHU	No	56	0	0
1	0	Thoracotoı	94	CHU	No	66	0	0
1	0	VATS or rc	94	CHU	Yes	61	0	0
1	0	VATS or rc	94	CHU	No	42	0	0
1	0	Thoracotoı	94	CHU	No	39	0	0
1	0	Thoracotoı	94	CHU	No	55	0	1

1	0	Thoracotoı	94	CHU	No	70	0	0
1	0	Thoracotoı	94	CHU	No	48	0	0
1	0	Thoracotoı	94	CHU	No	69	0	0
1	0	Thoracotoı	94	CHU	No	72	1	0
1	0	Thoracotoı	94	CHU	No	65	0	0
1	1	Thoracotoı	94	CHU	No	69	0	0
1	0	VATS or rc	94	CHU	No	61	0	0
1	0	Thoracotoı	94	CHU	No	68	0	0
1	0	Thoracotoı	94	CHU	No	58	0	0
1	0	Thoracotoı	94	CHU	No	70	0	0
1	0	Thoracotoı	94	CHU	No	54	0	0
1	0	Thoracotoı	94	CHU	No	61	0	0
1	0	VATS or rc	94	CHU	No	81	0	0
1	0	VATS or rc	94	CHU	No	46	0	0
1	0	VATS or rc	94	CHU	No	71	0	0
1	0	VATS or rc	94	CHU	No	63	0	0
1	0	VATS or rc	94	CHU	No	63	0	1
1	0	VATS or rc	94	CHU	No	70	1	0
1	0	Thoracotoı	94	CHU	No	65	0	0
1	0	Thoracotoı	94	CHU	No	54	0	0
1	0	Thoracotoı	94	CHU	No	78	0	0
1	0	VATS or rc	94	CHU	No	76	0	0
1	0	Thoracotoı	94	CHU	No	81	1	0
1	0	Thoracotoı	94	CHU	No	58	0	0
1	0	Thoracotoı	94	CHU	No	86	0	0
1	1	Thoracotoı	94	CHU	No	56	0	1
1	0	VATS or rc	94	CHU	No	61	0	0
1	0	Thoracotoı	94	CHU	Yes	80	1	0
1	0	Thoracotoı	94	CHU	No	69	0	0
1	0	Thoracotoı	94	CHU	No	78	0	0
1	1	Thoracotoı	94	CHU	No	83	0	0
1	0	VATS or rc	94	CHU	No	86	0	0
1	0	Thoracotoı	94	CHU	No	68	0	0
1	0	Thoracotoı	94	CHU	No	68	0	0
1	0	VATS or rc	94	CHU	No	71	0	0
1	0	VATS or rc	94	CHU	No	51	0	0
1	0	VATS or rc	94	CHU	No	59	0	0
1	0	VATS or rc	94	CHU	No	68	0	0
1	0	Thoracotoı	94	CHU	No	62	0	0
1	0	Thoracotoı	94	CHU	No	66	0	0
1	0	Thoracotoı	94	CHU	No	65	0	0
1	0	VATS or rc	94	CHU	No	53	0	0
1	0	Thoracotoı	94	CHU	No	77	0	0
1	0	VATS or rc	94	CHU	No	66	0	0
1	0	VATS or rc	94	CHU	No	64	0	0
1	0	VATS or rc	94	CHU	No	56	0	0
1	0	Thoracotoı	94	CHU	No	19	0	0
1	0	Thoracotoı	94	CHU	No	87	0	0
1	0	Thoracotoı	94	CHU	No	59	0	0
1	0	Thoracotoı	94	CHU	Yes	68	0	0
1	0	VATS or rc	94	CHU	No	74	0	0
1	0	Thoracotoı	94	CHU	No	67	0	0
1	0	Thoracotoı	94	CHU	No	73	0	0
1	0	Thoracotoı	94	CHU	Yes	69	0	0
1	0	VATS or rc	94	CHU	No	69	0	0
1	0	VATS or rc	94	CHU	No	54	0	0

1	0	Thoracotoı	94	CHU	No	59	0	0
1	0	Thoracotoı	94	CHU	No	61	0	0
1	0	VATS or rc	94	CHU	No	81	0	0
1	0	Thoracotoı	94	CHU	No	60	0	0
1	0	Thoracotoı	94	CHU	No	61	0	0
1	0	VATS or rc	94	CHU	No	54	0	0
1	0	VATS or rc	94	CHU	No	69	0	0
1	0	Thoracotoı	94	CHU	No	50	0	0
1	0	VATS or rc	94	CHU	No	54	0	0
1	0	Thoracotoı	94	CHU	No	71	0	0
1	0	Thoracotoı	94	CHU	No	62	0	0
1	0	Thoracotoı	94	CHU	No	48	0	1
1	0	Thoracotoı	94	CHU	No	59	0	0
1	0	Thoracotoı	94	CHU	No	78	0	0
1	0	VATS or rc	94	CHU	No	63	0	1
1	0	Thoracotoı	94	CHU	No	64	0	0
1	0	VATS or rc	94	CHU	No	70	0	0
1	0	Thoracotoı	94	CHU	No	57	0	0
1	0	VATS or rc	94	CHU	No	62	0	0
1	0	VATS or rc	94	CHU	No	75	0	1
1	0	Thoracotoı	94	CHU	No	53	0	0
1	0	Thoracotoı	94	CHU	No	62	0	0
1	0	Thoracotoı	94	CHU	No	70	1	0
1	0	Thoracotoı	94	CHU	No	60	0	0
1	0	Thoracotoı	94	CHU	No	71	0	0
1	0	Thoracotoı	94	CHU	No	71	0	0
1	0	VATS or rc	94	CHU	No	59	0	0
1	0	VATS or rc	94	CHU	No	80	0	0
1	0	Thoracotoı	94	CHU	No	64	0	0
1	0	VATS or rc	94	CHU	No	77	0	0
1	0	Thoracotoı	94	CHU	No	57	0	0
1	0	Thoracotoı	94	CHU	No	73	1	0
1	0	VATS or rc	94	CHU	No	81	0	0
1	0	VATS or rc	94	CHU	No	82	0	0
1	0	VATS or rc	94	CHU	No	75	0	0
1	0	Thoracotoı	94	CHU	No	70	0	0
1	1	Thoracotoı	94	CHU	No	60	0	0
1	0	VATS or rc	94	CHU	No	80	0	0
1	0	Thoracotoı	94	CHU	No	60	0	0
1	0	VATS or rc	94	CHU	No	85	0	0
1	0	VATS or rc	94	CHU	No	74	0	0
1	0	Thoracotoı	94	CHU	No	49	0	0
1	0	Thoracotoı	94	CHU	No	69	0	0
1	0	VATS or rc	94	CHU	No	57	0	0
1	1	Thoracotoı	94	CHU	No	57	0	0
1	0	Thoracotoı	94	CHU	No	69	0	0
1	0	Thoracotoı	94	CHU	Yes	49	0	0
1	0	VATS or rc	94	CHU	No	64	1	0
1	0	Thoracotoı	94	CHU	No	53	0	0
1	0	Thoracotoı	94	CHU	No	77	0	0
1	0	VATS or rc	94	CHU	No	55	0	0
1	0	Thoracotoı	94	CHU	No	57	0	0
1	0	VATS or rc	94	CHU	No	55	0	1
1	0	Thoracotoı	94	CHU	No	58	0	0
1	0	Thoracotoı	94	CHU	No	67	0	0
0	0	VATS or rc	65	CHG	No	71	0	0

0	0	Thoracotoı	65	CHG	No	71	0	1
0	0	Thoracotoı	65	CHG	No	60	0	0
0	0	Thoracotoı	65	CHG	No	73	0	1
0	0	Thoracotoı	65	CHG	No	79	0	1
0	0	Thoracotoı	65	CHG	No	73	0	0
0	0	VATS or rc	65	CHG	Yes	70	0	0
0	0	Thoracotoı	65	CHG	No	60	0	1
0	1	Thoracotoı	65	CHG	No	56	0	1
0	1	Thoracotoı	65	CHG	No	61	0	0
0	0	Thoracotoı	65	CHG	No	60	0	1
0	0	Thoracotoı	65	CHG	No	77	0	1
0	0	Thoracotoı	65	CHG	No	56	0	1
0	0	Thoracotoı	65	CHG	No	71	0	1
0	0	VATS or rc	65	CHG	No	68	0	1
0	0	Thoracotoı	65	CHG	No	74	1	0
0	0	Thoracotoı	65	CHG	No	66	0	1
0	0	Thoracotoı	65	CHG	No	56	0	0
0	0	VATS or rc	65	CHG	No	66	0	1
0	0	Thoracotoı	65	CHG	No	63	0	1
0	0	Thoracotoı	65	CHG	No	75	0	1
0	0	Thoracotoı	65	CHG	No	52	0	0
0	0	Thoracotoı	65	CHG	No	70	0	1
0	0	Thoracotoı	65	CHG	No	63	0	1
0	1	Thoracotoı	65	CHG	No	72	0	0
0	0	Thoracotoı	65	CHG	No	63	0	0
0	0	Thoracotoı	65	CHG	No	64	0	0
0	0	Thoracotoı	65	CHG	No	78	0	0
0	0	Thoracotoı	65	CHG	No	69	0	1
0	1	Thoracotoı	65	CHG	No	71	0	1
0	1	Thoracotoı	65	CHG	No	69	0	1
0	1	Thoracotoı	65	CHG	No	50	0	1
0	0	VATS or rc	65	CHG	No	71	0	0
0	0	Thoracotoı	65	CHG	No	67	0	1
0	0	Thoracotoı	65	CHG	No	66	0	0
0	0	Thoracotoı	65	CHG	No	68	0	1
0	1	Thoracotoı	65	CHG	No	66	0	1
0	0	Thoracotoı	65	CHG	No	77	0	0
0	0	Thoracotoı	65	CHG	No	61	0	0
0	0	Thoracotoı	65	CHG	No	67	0	0
0	0	Thoracotoı	65	CHG	No	68	0	0
0	0	Thoracotoı	65	CHG	No	67	0	1
0	0	Thoracotoı	65	CHG	No	57	0	1
0	0	Thoracotoı	65	CHG	No	74	1	0
0	0	Thoracotoı	65	CHG	No	53	1	1
0	0	Thoracotoı	65	CHG	No	78	0	1
0	0	Thoracotoı	65	CHG	No	63	0	1
0	0	Thoracotoı	65	CHG	No	74	0	1
0	0	Thoracotoı	65	CHG	No	56	1	1
0	0	Thoracotoı	65	CHG	No	64	0	0
0	0	Thoracotoı	65	CHG	No	67	0	1
0	1	Thoracotoı	65	CHG	No	72	0	1
0	0	Thoracotoı	65	CHG	No	64	0	0
0	0	Thoracotoı	65	CHG	No	46	0	0
0	0	Thoracotoı	65	CHG	No	56	0	1
0	1	Thoracotoı	65	CHG	No	77	0	0
0	0	VATS or rc	65	CHG	No	86	0	1

0	0	Thoracotoı	65	CHG	No	70	0	1
0	0	Thoracotoı	65	CHG	No	55	0	1
0	0	Thoracotoı	65	CHG	No	63	0	0
0	0	VATS or rc	65	CHG	No	67	0	0
0	0	Thoracotoı	65	CHG	No	56	0	0
0	0	Thoracotoı	65	CHG	No	65	0	0
0	0	Thoracotoı	65	CHG	No	73	1	0
0	0	VATS or rc	65	CHG	No	70	0	0
0	1	Thoracotoı	65	CHG	No	75	1	0
0	0	VATS or rc	65	CHG	No	66	0	1
0	0	Thoracotoı	65	CHG	No	59	0	1
0	0	Thoracotoı	65	CHG	No	53	0	0
0	0	Thoracotoı	65	CHG	No	62	0	1
0	0	Thoracotoı	65	CHG	No	54	0	1
0	1	Thoracotoı	65	CHG	No	70	0	0
0	0	Thoracotoı	65	CHG	No	86	0	1
0	0	Thoracotoı	65	CHG	No	53	0	0
0	0	VATS or rc	65	CHG	No	64	1	0
0	1	Thoracotoı	65	CHG	No	54	0	1
0	0	VATS or rc	65	CHG	No	52	0	1
0	0	Thoracotoı	65	CHG	No	59	0	0
0	0	Thoracotoı	65	CHG	No	74	0	0
0	0	Thoracotoı	65	CHG	No	72	1	1
0	0	Thoracotoı	65	CHG	No	64	0	0
0	0	VATS or rc	65	CHG	No	63	0	1
0	0	Thoracotoı	65	CHG	No	30	0	1
0	0	Thoracotoı	65	CHG	No	81	0	1
0	0	Thoracotoı	65	CHG	No	60	0	1
0	0	VATS or rc	65	CHG	No	68	0	0
0	0	VATS or rc	65	CHG	No	73	0	1
0	0	Thoracotoı	65	CHG	No	82	0	0
0	0	Thoracotoı	65	CHG	No	79	0	0
0	0	Thoracotoı	65	CHG	No	68	1	1
0	0	Thoracotoı	65	CHG	No	57	0	0
0	0	VATS or rc	65	CHG	No	56	0	0
0	0	Thoracotoı	65	CHG	No	88	0	1
0	1	Thoracotoı	65	CHG	No	71	0	0
0	0	VATS or rc	65	CHG	No	65	0	1
0	0	Thoracotoı	65	CHG	No	76	0	1
0	1	Thoracotoı	65	CHG	No	74	0	0
0	0	Thoracotoı	65	CHG	No	82	0	0
0	0	Thoracotoı	65	CHG	Yes	76	1	1
0	0	Thoracotoı	65	CHG	No	62	0	1
0	1	Thoracotoı	65	CHG	No	68	0	1
0	0	Thoracotoı	65	CHG	No	71	1	0
0	0	Thoracotoı	65	CHG	No	52	0	1
0	0	Thoracotoı	65	CHG	No	84	0	1
0	0	Thoracotoı	65	CHG	No	70	0	1
0	0	Thoracotoı	65	CHG	No	83	1	1
0	1	Thoracotoı	65	CHG	No	49	0	1
0	0	Thoracotoı	65	CHG	No	47	0	1
0	0	Thoracotoı	65	CHG	No	58	0	0
0	0	Thoracotoı	65	CHG	No	71	0	0
0	0	Thoracotoı	65	CHG	No	63	0	1
0	0	Thoracotoı	65	CHG	No	52	1	1
0	0	Thoracotoı	65	CHG	No	71	0	0

0	0	Thoracotoı	65	CHG	No	67	0	1
0	0	Thoracotoı	65	CHG	No	64	1	0
0	0	Thoracotoı	65	CHG	No	69	0	0
0	0	Thoracotoı	65	CHG	No	71	0	1
0	0	Thoracotoı	65	CHG	No	64	0	1
0	0	Thoracotoı	65	CHG	No	64	0	1
0	0	Thoracotoı	65	CHG	No	66	0	0
0	0	Thoracotoı	65	CHG	No	63	1	1
0	0	Thoracotoı	65	CHG	No	63	0	1
0	1	Thoracotoı	65	CHG	No	54	0	1
0	0	Thoracotoı	65	CHG	No	61	0	1
0	0	Thoracotoı	65	CHG	No	83	0	1
0	0	Thoracotoı	65	CHG	No	81	0	0
0	0	Thoracotoı	65	CHG	No	52	0	0
0	0	VATS or rc	65	CHG	No	49	0	1
0	0	Thoracotoı	65	CHG	No	65	0	0
1	0	VATS or rc	1	PRIVE	No	59	0	0
1	0	Thoracotoı	1	PRIVE	No	55	0	1
1	0	VATS or rc	1	PRIVE	No	67	0	1
1	0	VATS or rc	1	PRIVE	No	59	0	1
1	1	Thoracotoı	1	PRIVE	No	64	1	1
1	0	VATS or rc	1	PRIVE	No	59	0	1
1	0	Thoracotoı	1	PRIVE	No	60	0	1
1	0	Thoracotoı	1	PRIVE	No	50	0	1
1	0	VATS or rc	1	PRIVE	No	70	0	1
1	0	Thoracotoı	1	PRIVE	No	66	0	1
1	0	VATS or rc	1	PRIVE	No	77	0	1
1	0	Thoracotoı	1	PRIVE	No	56	0	1
1	0	VATS or rc	1	PRIVE	No	58	0	1
1	1	VATS or rc	1	PRIVE	No	57	0	1
1	0	Thoracotoı	1	PRIVE	No	58	0	1
1	0	Thoracotoı	1	PRIVE	No	63	0	1
1	0	Thoracotoı	1	PRIVE	No	60	0	0
1	0	VATS or rc	1	PRIVE	No	63	0	1
1	0	VATS or rc	1	PRIVE	No	59	0	1
1	0	Thoracotoı	1	PRIVE	No	76	0	1
1	0	Thoracotoı	1	PRIVE	No	66	0	0
1	0	Thoracotoı	1	PRIVE	No	69	0	1
1	0	Thoracotoı	1	PRIVE	No	61	0	1
1	0	VATS or rc	1	PRIVE	No	62	0	1
1	0	Thoracotoı	1	PRIVE	No	67	0	0
1	0	Thoracotoı	1	PRIVE	No	59	0	1
1	0	Thoracotoı	1	PRIVE	No	61	0	1
1	1	Thoracotoı	1	PRIVE	No	78	0	1
1	0	Thoracotoı	1	PRIVE	No	67	0	0
1	0	Thoracotoı	1	PRIVE	No	65	0	1
1	0	Thoracotoı	1	PRIVE	No	53	0	1
1	0	Thoracotoı	1	PRIVE	No	80	1	0
1	0	VATS or rc	1	PRIVE	No	64	0	1
1	0	Thoracotoı	1	PRIVE	No	62	0	0
1	0	VATS or rc	1	PRIVE	No	63	0	1
1	1	Thoracotoı	1	PRIVE	No	63	0	1
1	0	Thoracotoı	1	PRIVE	No	57	0	0
1	0	Thoracotoı	1	PRIVE	No	79	0	0
1	1	Thoracotoı	1	PRIVE	No	62	0	1
1	0	VATS or rc	1	PRIVE	No	74	0	1

1	0	VATS or rc	1	PRIVE	No	65	0	1
1	0	VATS or rc	1	PRIVE	No	58	0	1
1	0	Thoracotoı	1	PRIVE	No	69	0	1
1	0	Thoracotoı	1	PRIVE	No	51	0	1
1	0	Thoracotoı	1	PRIVE	No	56	0	1
1	0	VATS or rc	1	PRIVE	No	67	0	1
1	0	Thoracotoı	1	PRIVE	No	69	0	1
1	0	Thoracotoı	1	PRIVE	No	68	0	0
1	0	VATS or rc	1	PRIVE	No	66	0	1
1	0	Thoracotoı	1	PRIVE	No	49	0	1
1	0	Thoracotoı	1	PRIVE	No	71	0	1
1	0	Thoracotoı	1	PRIVE	No	75	1	1
1	0	Thoracotoı	1	PRIVE	No	57	0	1
1	0	Thoracotoı	1	PRIVE	No	62	0	1
1	0	Thoracotoı	1	PRIVE	No	71	0	1
1	0	VATS or rc	1	PRIVE	No	60	0	1
1	0	Thoracotoı	1	PRIVE	No	66	0	1
1	0	VATS or rc	1	PRIVE	No	67	0	0
1	0	Thoracotoı	1	PRIVE	No	74	1	1
1	0	Thoracotoı	1	PRIVE	No	85	0	0
1	0	Thoracotoı	1	PRIVE	No	75	1	1
1	0	Thoracotoı	1	PRIVE	No	74	1	1
1	0	Thoracotoı	1	PRIVE	No	64	0	1
1	0	Thoracotoı	1	PRIVE	No	73	0	1
1	0	VATS or rc	1	PRIVE	No	58	0	1
1	0	Thoracotoı	1	PRIVE	No	60	0	0
1	0	VATS or rc	1	PRIVE	No	68	1	1
1	0	Thoracotoı	1	PRIVE	Yes	78	0	1
1	1	VATS or rc	1	PRIVE	No	76	0	1
1	0	Thoracotoı	1	PRIVE	No	62	0	1
1	0	Thoracotoı	1	PRIVE	No	62	1	1
1	0	Thoracotoı	1	PRIVE	No	56	0	1
1	0	Thoracotoı	1	PRIVE	No	74	1	1
1	0	VATS or rc	1	PRIVE	No	61	0	0
1	0	VATS or rc	1	PRIVE	No	53	0	1
1	0	VATS or rc	1	PRIVE	Yes	70	0	1
1	1	Thoracotoı	1	PRIVE	No	55	0	1
1	0	Thoracotoı	1	PRIVE	No	69	0	1
1	0	Thoracotoı	1	PRIVE	No	65	0	1
1	0	VATS or rc	1	PRIVE	No	49	0	1
1	0	VATS or rc	1	PRIVE	No	61	0	1
1	0	Thoracotoı	1	PRIVE	No	54	0	1
1	0	Thoracotoı	1	PRIVE	No	73	0	1
1	1	Thoracotoı	1	PRIVE	No	59	0	0
1	0	VATS or rc	1	PRIVE	No	63	0	1
1	0	Thoracotoı	1	PRIVE	No	56	0	1
1	1	VATS or rc	1	PRIVE	No	63	1	1
1	0	Thoracotoı	1	PRIVE	No	63	0	1
1	0	Thoracotoı	1	PRIVE	No	69	0	1
1	0	Thoracotoı	1	PRIVE	No	56	0	1
1	0	Thoracotoı	1	PRIVE	No	63	0	1
1	0	VATS or rc	1	PRIVE	No	79	0	1
1	0	VATS or rc	1	PRIVE	No	62	0	0
1	1	Thoracotoı	1	PRIVE	No	60	0	1
1	1	Thoracotoı	1	PRIVE	No	44	0	1
1	0	VATS or rc	1	PRIVE	No	69	1	1

1	0	Thoracotoı	1	PRIVE	No	56	0	1
1	1	Thoracotoı	1	PRIVE	No	57	0	1
1	1	VATS or rc	1	PRIVE	No	64	0	1
1	0	VATS or rc	1	PRIVE	No	67	0	0
1	0	VATS or rc	1	PRIVE	No	63	0	1
1	0	VATS or rc	1	PRIVE	No	73	0	1
1	0	Thoracotoı	1	PRIVE	No	56	0	1
1	0	VATS or rc	1	PRIVE	No	79	0	0
1	0	VATS or rc	1	PRIVE	No	63	0	1
1	0	VATS or rc	1	PRIVE	No	68	0	0
1	1	VATS or rc	1	PRIVE	No	63	1	1
1	0	VATS or rc	1	PRIVE	No	68	0	1
1	0	Thoracotoı	1	PRIVE	No	74	0	1
1	0	Thoracotoı	1	PRIVE	No	65	0	1
1	0	Thoracotoı	1	PRIVE	No	85	0	0
1	0	VATS or rc	1	PRIVE	No	66	0	1
1	1	Thoracotoı	1	PRIVE	No	48	0	1
1	0	VATS or rc	1	PRIVE	No	73	0	1
1	0	Thoracotoı	1	PRIVE	No	63	0	1
1	0	Thoracotoı	1	PRIVE	No	83	0	1
1	0	Thoracotoı	1	PRIVE	No	59	0	1
1	0	VATS or rc	1	PRIVE	No	71	0	0
1	0	VATS or rc	1	PRIVE	Yes	55	0	1
1	0	VATS or rc	1	PRIVE	No	65	0	0
1	1	VATS or rc	1	PRIVE	No	64	0	1
1	0	VATS or rc	1	PRIVE	No	71	0	1
0	0	VATS or rc	2	CHG	No	57	0	0
0	0	Thoracotoı	2	CHG	No	64	0	0
0	0	Thoracotoı	2	CHG	No	51	0	1
0	0	VATS or rc	2	CHG	No	58	1	1
0	1	Thoracotoı	2	CHG	No	53	0	0
0	0	Thoracotoı	2	CHG	No	69	0	0
0	1	Thoracotoı	2	CHG	No	46	0	0
0	0	VATS or rc	2	CHG	No	60	0	0
0	0	VATS or rc	2	CHG	No	61	0	0
0	0	VATS or rc	2	CHG	No	56	0	1
0	0	VATS or rc	2	CHG	No	66	0	0
0	0	VATS or rc	2	CHG	No	62	0	1
0	0	VATS or rc	2	CHG	No	64	0	0
0	0	VATS or rc	2	CHG	No	63	1	0
0	0	Thoracotoı	2	CHG	No	77	0	1
0	0	VATS or rc	2	CHG	No	70	0	1
0	0	VATS or rc	2	CHG	No	69	0	0
0	0	VATS or rc	2	CHG	No	82	0	0
0	0	VATS or rc	2	CHG	No	51	0	0
0	0	Thoracotoı	2	CHG	No	68	0	1
0	0	VATS or rc	2	CHG	Yes	71	1	0
0	0	VATS or rc	2	CHG	No	75	0	1
0	0	Thoracotoı	2	CHG	No	71	0	0
0	0	VATS or rc	2	CHG	No	49	0	0
0	0	VATS or rc	2	CHG	No	70	1	0
0	0	Thoracotoı	2	CHG	No	74	0	0
0	0	Thoracotoı	2	CHG	No	65	0	1
0	0	Thoracotoı	2	CHG	No	45	0	0
0	0	VATS or rc	2	CHG	No	59	0	0
0	0	VATS or rc	2	CHG	No	82	0	0

0	1	Thoracotoı	2	CHG	No	69	0	0
0	0	VATS or rc	263	PRIVE	No	66	0	0
0	0	Thoracotoı	263	PRIVE	No	53	0	0
0	0	VATS or rc	263	PRIVE	No	77	1	0
0	0	VATS or rc	263	PRIVE	No	51	0	0
0	0	VATS or rc	263	PRIVE	No	51	0	0
0	0	Thoracotoı	263	PRIVE	No	18	0	0
0	0	Thoracotoı	263	PRIVE	No	56	0	0
0	0	Thoracotoı	263	PRIVE	No	70	0	0
0	0	VATS or rc	263	PRIVE	No	77	1	0
0	0	VATS or rc	263	PRIVE	No	65	0	0
0	1	Thoracotoı	263	PRIVE	No	57	0	0
0	0	VATS or rc	263	PRIVE	No	79	0	0
0	0	VATS or rc	263	PRIVE	No	45	0	0
0	0	Thoracotoı	263	PRIVE	No	56	0	0
0	0	VATS or rc	263	PRIVE	No	66	0	0
0	0	Thoracotoı	263	PRIVE	No	57	0	0
0	0	VATS or rc	263	PRIVE	No	58	0	0
0	0	VATS or rc	263	PRIVE	No	77	0	0
0	0	Thoracotoı	263	PRIVE	No	55	0	0
0	0	Thoracotoı	263	PRIVE	No	62	0	0
0	0	VATS or rc	263	PRIVE	No	54	0	0
0	0	Thoracotoı	263	PRIVE	No	77	0	0
0	0	VATS or rc	263	PRIVE	No	59	0	1
0	0	Thoracotoı	263	PRIVE	No	56	1	0
0	0	VATS or rc	263	PRIVE	No	66	0	0
0	0	Thoracotoı	263	PRIVE	No	69	1	0
0	0	Thoracotoı	263	PRIVE	No	47	0	0
0	0	VATS or rc	263	PRIVE	No	65	0	0
0	0	Thoracotoı	263	PRIVE	No	77	0	0
0	0	VATS or rc	263	PRIVE	No	62	0	0
0	0	VATS or rc	263	PRIVE	No	45	0	0
0	0	Thoracotoı	263	PRIVE	No	30	0	0
0	0	Thoracotoı	263	PRIVE	No	61	0	0
0	0	Thoracotoı	263	PRIVE	No	63	0	0
0	0	VATS or rc	263	PRIVE	No	75	0	0
0	0	VATS or rc	263	PRIVE	No	68	0	0
0	0	Thoracotoı	263	PRIVE	No	57	0	0
0	1	Thoracotoı	263	PRIVE	No	63	0	0
0	0	VATS or rc	263	PRIVE	No	63	0	1
0	0	VATS or rc	263	PRIVE	No	59	0	0
0	0	VATS or rc	263	PRIVE	No	60	0	0
0	1	Thoracotoı	263	PRIVE	No	60	0	0
0	0	Thoracotoı	263	PRIVE	No	57	0	0
0	0	Thoracotoı	263	PRIVE	No	69	0	0
0	0	Thoracotoı	263	PRIVE	No	57	0	0
0	0	Thoracotoı	263	PRIVE	No	48	0	0
0	0	Thoracotoı	263	PRIVE	No	50	0	0
0	0	Thoracotoı	263	PRIVE	No	63	0	0
0	0	Thoracotoı	263	PRIVE	No	60	0	0
0	0	Thoracotoı	263	PRIVE	No	73	0	0
0	0	Thoracotoı	263	PRIVE	No	65	0	0
0	0	Thoracotoı	263	PRIVE	No	59	0	0
0	0	VATS or rc	263	PRIVE	No	79	0	0
0	0	VATS or rc	263	PRIVE	No	56	0	0
0	0	VATS or rc	263	PRIVE	No	70	0	0

0	1	Thoracotoı	263	PRIVE	No	62	0	0
0	0	VATS or rc	263	PRIVE	No	62	1	1
0	0	VATS or rc	263	PRIVE	No	67	0	0
0	0	Thoracotoı	263	PRIVE	No	71	0	0
0	0	VATS or rc	263	PRIVE	No	59	0	0
0	0	Thoracotoı	263	PRIVE	No	62	0	0
0	0	VATS or rc	263	PRIVE	No	79	1	0
0	0	Thoracotoı	263	PRIVE	No	64	0	0
0	0	Thoracotoı	263	PRIVE	No	60	0	0
0	0	VATS or rc	263	PRIVE	No	68	0	1
0	0	Thoracotoı	263	PRIVE	No	51	0	0
0	0	VATS or rc	263	PRIVE	No	60	0	0
0	0	Thoracotoı	263	PRIVE	No	71	0	0
0	1	Thoracotoı	263	PRIVE	No	51	0	1
0	1	Thoracotoı	263	PRIVE	No	67	1	0
0	0	VATS or rc	263	PRIVE	No	56	0	0
0	0	Thoracotoı	263	PRIVE	No	73	0	0
0	0	Thoracotoı	263	PRIVE	No	61	0	0
0	0	Thoracotoı	263	PRIVE	No	71	0	0
0	0	Thoracotoı	263	PRIVE	No	75	0	1
0	0	Thoracotoı	263	PRIVE	No	46	0	0
0	0	Thoracotoı	263	PRIVE	No	38	0	0
0	0	Thoracotoı	263	PRIVE	No	53	0	0
0	0	VATS or rc	263	PRIVE	No	78	0	0
0	0	VATS or rc	263	PRIVE	No	64	0	0
0	0	VATS or rc	263	PRIVE	No	79	1	1
0	0	Thoracotoı	263	PRIVE	No	61	0	1
0	0	VATS or rc	263	PRIVE	No	66	0	0
0	0	VATS or rc	263	PRIVE	No	68	1	0
0	0	VATS or rc	263	PRIVE	No	71	0	0
0	1	Thoracotoı	263	PRIVE	No	56	0	0
0	0	VATS or rc	263	PRIVE	No	53	0	0
0	1	Thoracotoı	263	PRIVE	No	63	0	0
0	0	Thoracotoı	263	PRIVE	No	70	0	0
0	0	VATS or rc	263	PRIVE	No	76	0	0
0	0	Thoracotoı	263	PRIVE	No	70	0	0
0	0	VATS or rc	263	PRIVE	No	63	0	0
0	0	Thoracotoı	263	PRIVE	No	67	0	0
0	0	Thoracotoı	263	PRIVE	No	47	0	0
0	0	Thoracotoı	263	PRIVE	No	44	0	0
0	0	VATS or rc	263	PRIVE	No	61	0	0
0	0	VATS or rc	263	PRIVE	No	78	0	0
0	0	VATS or rc	263	PRIVE	No	53	0	0
0	0	Thoracotoı	263	PRIVE	No	64	0	0
0	0	VATS or rc	263	PRIVE	No	67	0	0
0	0	Thoracotoı	263	PRIVE	No	72	1	0
0	0	Thoracotoı	263	PRIVE	No	59	0	1
0	0	Thoracotoı	263	PRIVE	No	39	0	0
0	0	Thoracotoı	263	PRIVE	No	61	0	0
0	0	Thoracotoı	263	PRIVE	No	76	0	0
0	0	VATS or rc	263	PRIVE	No	53	0	0
0	0	VATS or rc	263	PRIVE	No	69	1	0
0	0	Thoracotoı	263	PRIVE	No	77	0	0
0	0	Thoracotoı	263	PRIVE	No	46	0	0
0	0	Thoracotoı	263	PRIVE	No	71	0	0
0	0	VATS or rc	263	PRIVE	No	74	0	0

0	0	VATS or ro	263	PRIVE	No	72	0	0
0	0	VATS or ro	263	PRIVE	No	83	1	0
0	0	Thoracotoı	263	PRIVE	No	63	0	0
0	0	VATS or ro	263	PRIVE	No	76	0	0
0	0	VATS or ro	263	PRIVE	No	75	0	0
0	0	Thoracotoı	263	PRIVE	Yes	83	0	0
0	0	Thoracotoı	263	PRIVE	No	55	1	1
0	0	VATS or ro	263	PRIVE	No	50	0	0
0	0	VATS or ro	263	PRIVE	No	66	0	0
0	0	Thoracotoı	263	PRIVE	No	68	1	0
0	0	Thoracotoı	263	PRIVE	No	57	0	0
0	0	Thoracotoı	263	PRIVE	No	70	0	0
0	0	VATS or ro	263	PRIVE	No	69	0	1
0	0	VATS or ro	263	PRIVE	No	72	0	0
0	0	Thoracotoı	263	PRIVE	No	63	0	0
0	0	VATS or ro	263	PRIVE	No	78	0	0
0	0	VATS or ro	263	PRIVE	No	34	0	0
0	0	VATS or ro	263	PRIVE	No	62	0	0
0	0	Thoracotoı	263	PRIVE	Yes	62	0	0
0	0	Thoracotoı	263	PRIVE	No	46	0	0
0	0	VATS or ro	263	PRIVE	No	52	0	0
0	0	Thoracotoı	263	PRIVE	No	73	0	0
0	0	VATS or ro	263	PRIVE	No	67	0	1
0	0	VATS or ro	263	PRIVE	No	58	0	1
0	0	VATS or ro	263	PRIVE	No	60	0	0
0	0	Thoracotoı	263	PRIVE	No	73	0	0
0	0	VATS or ro	263	PRIVE	No	60	0	0
0	0	Thoracotoı	263	PRIVE	No	62	0	0
0	0	VATS or ro	263	PRIVE	No	66	0	1
0	1	Thoracotoı	263	PRIVE	No	54	0	0
0	0	VATS or ro	263	PRIVE	No	60	0	0
0	0	VATS or ro	263	PRIVE	No	60	0	0
0	0	VATS or ro	263	PRIVE	No	72	0	0
0	0	Thoracotoı	263	PRIVE	No	57	0	0
0	0	VATS or ro	263	PRIVE	No	68	0	0
0	0	VATS or ro	263	PRIVE	No	71	0	0
0	0	Thoracotoı	263	PRIVE	No	63	0	0
0	0	Thoracotoı	263	PRIVE	No	63	0	0
0	0	VATS or ro	263	PRIVE	No	75	0	0
0	1	Thoracotoı	263	PRIVE	No	73	0	0
0	0	VATS or ro	263	PRIVE	No	51	0	0
0	0	Thoracotoı	263	PRIVE	No	60	0	0
0	0	VATS or ro	263	PRIVE	No	72	0	0
0	0	VATS or ro	263	PRIVE	No	67	0	0
0	0	VATS or ro	263	PRIVE	No	65	0	0
0	0	Thoracotoı	263	PRIVE	No	52	0	0
0	0	VATS or ro	263	PRIVE	No	73	0	0
0	0	VATS or ro	263	PRIVE	No	59	0	0
0	0	Thoracotoı	263	PRIVE	No	74	0	0
0	0	Thoracotoı	263	PRIVE	No	67	0	0
0	0	VATS or ro	263	PRIVE	No	76	0	0
0	0	VATS or ro	263	PRIVE	No	52	0	1
0	0	VATS or ro	263	PRIVE	No	52	0	1
0	0	VATS or ro	263	PRIVE	No	57	0	0
0	0	Thoracotoı	263	PRIVE	No	71	1	0
0	0	VATS or ro	263	PRIVE	No	67	1	0

0	0	VATS or rc	263	PRIVE	No	68	1	0
0	0	Thoracotoı	263	PRIVE	No	33	0	0
0	0	VATS or rc	263	PRIVE	No	65	0	1
0	1	Thoracotoı	263	PRIVE	No	63	0	0
0	0	VATS or rc	263	PRIVE	No	59	0	0
0	0	Thoracotoı	263	PRIVE	No	51	0	0
0	0	VATS or rc	263	PRIVE	No	85	0	0
0	0	Thoracotoı	263	PRIVE	No	59	0	0
0	0	VATS or rc	263	PRIVE	No	67	0	0
0	0	VATS or rc	263	PRIVE	No	61	0	0
0	0	VATS or rc	263	PRIVE	No	77	0	0
0	0	Thoracotoı	263	PRIVE	No	47	0	0
0	0	Thoracotoı	263	PRIVE	No	33	0	0
0	0	VATS or rc	263	PRIVE	No	67	0	0
0	0	VATS or rc	263	PRIVE	No	69	0	0
0	0	Thoracotoı	51	PRIVE	No	55	0	0
0	0	VATS or rc	51	PRIVE	No	69	0	0
0	0	VATS or rc	51	PRIVE	No	43	0	0
0	1	Thoracotoı	51	PRIVE	No	62	0	0
0	0	VATS or rc	51	PRIVE	No	52	0	0
0	0	VATS or rc	51	PRIVE	No	61	0	0
0	1	Thoracotoı	51	PRIVE	No	62	0	0
0	0	VATS or rc	51	PRIVE	No	65	0	0
0	0	Thoracotoı	51	PRIVE	Yes	64	0	0
0	1	Thoracotoı	51	PRIVE	No	64	0	0
0	0	Thoracotoı	51	PRIVE	No	67	0	0
0	0	Thoracotoı	51	PRIVE	No	66	0	0
0	0	Thoracotoı	51	PRIVE	No	66	0	0
0	0	Thoracotoı	51	PRIVE	No	75	1	0
0	0	Thoracotoı	51	PRIVE	No	58	0	0
0	0	Thoracotoı	51	PRIVE	No	73	0	0
0	0	Thoracotoı	51	PRIVE	No	41	0	0
0	0	VATS or rc	51	PRIVE	No	58	0	0
0	0	VATS or rc	51	PRIVE	No	61	0	0
0	0	VATS or rc	51	PRIVE	No	67	0	0
0	0	Thoracotoı	51	PRIVE	No	72	0	0
0	0	VATS or rc	51	PRIVE	No	60	0	0
0	0	VATS or rc	51	PRIVE	No	49	0	0
0	0	VATS or rc	51	PRIVE	No	64	0	0
0	0	Thoracotoı	51	PRIVE	No	59	1	0
0	0	Thoracotoı	51	PRIVE	No	71	0	0
0	0	VATS or rc	51	PRIVE	No	78	0	0
0	0	VATS or rc	51	PRIVE	No	32	0	0
0	0	Thoracotoı	51	PRIVE	No	70	0	0
0	0	Thoracotoı	51	PRIVE	No	72	1	0
0	1	Thoracotoı	51	PRIVE	No	56	0	0
0	0	VATS or rc	51	PRIVE	No	59	0	1
0	0	Thoracotoı	51	PRIVE	No	71	0	0
0	0	Thoracotoı	51	PRIVE	No	60	0	1
0	0	VATS or rc	51	PRIVE	No	54	0	0
0	0	Thoracotoı	51	PRIVE	No	74	0	0
0	0	Thoracotoı	51	PRIVE	No	70	0	1
0	0	VATS or rc	51	PRIVE	No	66	0	0
0	0	Thoracotoı	51	PRIVE	No	57	0	0
0	0	VATS or rc	51	PRIVE	No	78	1	0
0	0	Thoracotoı	51	PRIVE	No	53	0	1

0	0	Thoracotoı	51	PRIVE	No	75	0	0
0	0	VATS or rc	51	PRIVE	No	66	0	1
0	0	VATS or rc	51	PRIVE	No	77	0	1
0	0	VATS or rc	51	PRIVE	No	46	0	1
0	0	Thoracotoı	51	PRIVE	No	76	1	0
0	1	Thoracotoı	51	PRIVE	No	62	0	0
0	0	VATS or rc	51	PRIVE	No	69	0	0
0	0	VATS or rc	51	PRIVE	No	61	0	1
0	0	VATS or rc	51	PRIVE	No	63	1	0
0	0	Thoracotoı	51	PRIVE	No	61	0	0
0	0	Thoracotoı	51	PRIVE	No	57	0	0
0	0	VATS or rc	51	PRIVE	No	74	0	0
0	0	Thoracotoı	51	PRIVE	No	71	0	0
0	0	VATS or rc	51	PRIVE	No	58	0	0
0	0	VATS or rc	51	PRIVE	No	48	0	0
0	0	VATS or rc	51	PRIVE	No	84	0	0
0	0	Thoracotoı	51	PRIVE	No	67	0	0
0	0	VATS or rc	51	PRIVE	No	68	0	1
0	0	Thoracotoı	51	PRIVE	No	74	0	1
0	0	VATS or rc	51	PRIVE	No	80	0	0
0	0	VATS or rc	51	PRIVE	No	25	0	0
0	0	Thoracotoı	51	PRIVE	No	78	1	1
0	0	Thoracotoı	51	PRIVE	No	70	1	0
0	0	Thoracotoı	51	PRIVE	No	68	0	1
0	0	Thoracotoı	51	PRIVE	No	78	0	0
0	0	VATS or rc	51	PRIVE	No	66	0	0
0	0	VATS or rc	51	PRIVE	No	69	0	0
0	0	VATS or rc	51	PRIVE	No	61	0	0
0	0	Thoracotoı	51	PRIVE	No	72	0	0
0	0	Thoracotoı	51	PRIVE	No	65	0	0
0	0	VATS or rc	51	PRIVE	No	49	0	0
0	0	VATS or rc	51	PRIVE	No	67	1	0
0	0	Thoracotoı	51	PRIVE	No	60	0	1
0	1	Thoracotoı	51	PRIVE	No	53	0	1
0	0	Thoracotoı	51	PRIVE	No	56	0	1
0	0	Thoracotoı	51	PRIVE	No	69	0	0
0	0	VATS or rc	51	PRIVE	No	72	0	0
0	0	Thoracotoı	51	PRIVE	No	67	0	1
0	0	Thoracotoı	51	PRIVE	No	80	0	0
0	0	VATS or rc	51	PRIVE	No	48	0	1
0	0	VATS or rc	51	PRIVE	No	73	0	0
0	0	Thoracotoı	51	PRIVE	No	63	0	0
0	0	VATS or rc	51	PRIVE	No	58	0	0
0	0	Thoracotoı	51	PRIVE	No	75	0	0
0	0	VATS or rc	51	PRIVE	No	52	0	1
0	0	VATS or rc	51	PRIVE	No	78	0	0
0	0	Thoracotoı	51	PRIVE	No	70	0	0
0	0	VATS or rc	51	PRIVE	No	49	0	0
0	0	Thoracotoı	51	PRIVE	No	70	0	1
0	0	VATS or rc	51	PRIVE	No	69	0	0
0	0	VATS or rc	51	PRIVE	No	71	0	0
0	1	Thoracotoı	51	PRIVE	No	64	1	0
0	0	VATS or rc	51	PRIVE	No	61	0	0
0	0	Thoracotoı	51	PRIVE	No	36	0	0
0	0	Thoracotoı	51	PRIVE	No	72	1	0
0	0	VATS or rc	51	PRIVE	No	69	0	0

0	1	Thoracotoı	51	PRIVE	No	70	0	0
0	0	Thoracotoı	51	PRIVE	No	70	0	1
0	0	Thoracotoı	51	PRIVE	No	61	0	0
0	0	Thoracotoı	51	PRIVE	No	67	0	0
0	0	Thoracotoı	51	PRIVE	No	75	0	0
0	0	Thoracotoı	51	PRIVE	No	76	0	0
0	0	VATS or rc	51	PRIVE	No	58	0	0
0	0	Thoracotoı	51	PRIVE	No	76	0	0
0	0	VATS or rc	51	PRIVE	No	65	0	0
0	0	Thoracotoı	51	PRIVE	No	55	0	1
0	0	Thoracotoı	51	PRIVE	No	68	0	0
0	0	Thoracotoı	51	PRIVE	No	73	0	0
0	0	VATS or rc	51	PRIVE	No	70	0	0
0	0	Thoracotoı	51	PRIVE	No	60	0	1
0	0	Thoracotoı	51	PRIVE	No	60	0	0
0	0	VATS or rc	51	PRIVE	No	61	0	0
1	0	Thoracotoı	3	PRIVE	No	58	0	0
1	0	Thoracotoı	3	PRIVE	No	56	0	0
1	0	Thoracotoı	3	PRIVE	No	69	0	1
1	0	Thoracotoı	3	PRIVE	No	65	0	0
1	0	Thoracotoı	3	PRIVE	No	51	0	0
1	0	Thoracotoı	3	PRIVE	No	59	0	0
1	0	Thoracotoı	3	PRIVE	No	54	0	0
1	0	Thoracotoı	3	PRIVE	No	60	0	1
1	0	Thoracotoı	3	PRIVE	No	60	0	0
1	0	Thoracotoı	3	PRIVE	No	64	0	1
1	0	Thoracotoı	3	PRIVE	No	68	1	0
1	0	Thoracotoı	3	PRIVE	No	73	0	0
1	1	Thoracotoı	3	PRIVE	No	66	0	0
1	0	Thoracotoı	3	PRIVE	No	75	0	0
1	0	Thoracotoı	3	PRIVE	No	72	0	0
1	0	Thoracotoı	3	PRIVE	No	74	0	0
1	0	Thoracotoı	3	PRIVE	No	69	0	0
1	1	Thoracotoı	3	PRIVE	No	53	0	0
1	0	VATS or rc	3	PRIVE	No	57	0	0
1	0	VATS or rc	3	PRIVE	No	47	0	1
1	0	Thoracotoı	3	PRIVE	No	66	0	1
1	0	Thoracotoı	3	PRIVE	No	68	0	0
1	0	Thoracotoı	3	PRIVE	No	51	0	0
1	0	Thoracotoı	3	PRIVE	No	70	0	0
1	0	Thoracotoı	3	PRIVE	No	74	0	0
1	0	VATS or rc	3	PRIVE	No	56	0	0
1	0	Thoracotoı	3	PRIVE	No	49	0	0
1	0	Thoracotoı	3	PRIVE	No	72	0	0
1	0	Thoracotoı	3	PRIVE	No	42	0	1
1	0	Thoracotoı	3	PRIVE	No	45	0	0
1	0	Thoracotoı	3	PRIVE	No	52	0	1
1	0	Thoracotoı	3	PRIVE	No	60	0	0
1	0	Thoracotoı	3	PRIVE	No	47	0	0
1	1	Thoracotoı	3	PRIVE	No	55	0	0
1	0	Thoracotoı	3	PRIVE	No	74	0	0
1	0	Thoracotoı	3	PRIVE	No	66	0	0
1	0	Thoracotoı	3	PRIVE	No	76	0	0
1	0	Thoracotoı	3	PRIVE	No	73	0	0
1	0	Thoracotoı	3	PRIVE	No	71	0	0
1	0	Thoracotoı	3	PRIVE	No	65	0	0

1	0	Thoracotoı	3	PRIVE	No	65	0	0
1	0	VATS or rc	3	PRIVE	No	76	0	0
1	0	Thoracotoı	3	PRIVE	No	46	0	0
1	0	Thoracotoı	3	PRIVE	No	40	0	0
1	0	Thoracotoı	3	PRIVE	No	64	0	0
1	0	Thoracotoı	3	PRIVE	No	78	0	0
1	0	Thoracotoı	3	PRIVE	No	63	0	0
1	0	Thoracotoı	3	PRIVE	No	66	0	0
1	0	Thoracotoı	3	PRIVE	No	61	0	1
1	0	Thoracotoı	3	PRIVE	No	77	0	0
1	0	Thoracotoı	3	PRIVE	No	62	0	0
1	0	Thoracotoı	3	PRIVE	No	52	0	0
1	0	Thoracotoı	3	PRIVE	No	51	0	0
1	1	Thoracotoı	3	PRIVE	No	66	0	0
1	0	Thoracotoı	3	PRIVE	No	59	0	1
1	0	Thoracotoı	3	PRIVE	No	68	0	0
1	0	Thoracotoı	3	PRIVE	No	74	0	0
1	0	Thoracotoı	3	PRIVE	No	65	0	0
1	0	Thoracotoı	3	PRIVE	No	64	0	0
1	0	Thoracotoı	3	PRIVE	No	59	0	0
1	0	Thoracotoı	3	PRIVE	No	61	1	1
1	0	Thoracotoı	3	PRIVE	No	55	0	0
1	0	Thoracotoı	3	PRIVE	No	46	0	1
1	0	Thoracotoı	3	PRIVE	No	62	0	0
1	0	Thoracotoı	3	PRIVE	No	47	1	0
1	0	Thoracotoı	3	PRIVE	No	70	0	0
1	0	Thoracotoı	3	PRIVE	No	58	0	1
1	0	Thoracotoı	3	PRIVE	No	60	0	1
1	0	Thoracotoı	3	PRIVE	No	68	0	0
1	0	Thoracotoı	3	PRIVE	No	73	0	0
1	0	Thoracotoı	3	PRIVE	No	56	0	1
1	0	Thoracotoı	3	PRIVE	No	61	0	0
1	0	Thoracotoı	3	PRIVE	No	82	0	0
1	0	Thoracotoı	3	PRIVE	No	82	0	0
1	0	Thoracotoı	3	PRIVE	No	62	0	0
1	1	Thoracotoı	3	PRIVE	No	77	0	0
1	1	Thoracotoı	3	PRIVE	No	65	0	0
1	0	Thoracotoı	3	PRIVE	No	62	0	0
1	0	Thoracotoı	3	PRIVE	No	68	0	0
1	0	Thoracotoı	3	PRIVE	No	62	0	0
1	0	Thoracotoı	3	PRIVE	No	57	0	0
1	0	Thoracotoı	3	PRIVE	No	72	0	0
1	0	Thoracotoı	3	PRIVE	No	57	0	1
1	0	Thoracotoı	3	PRIVE	No	73	0	0
1	0	Thoracotoı	3	PRIVE	No	67	0	0
1	0	Thoracotoı	3	PRIVE	No	68	0	1
1	0	Thoracotoı	3	PRIVE	No	71	1	0
1	0	Thoracotoı	3	PRIVE	No	55	0	1
1	0	Thoracotoı	3	PRIVE	No	60	0	0
1	0	Thoracotoı	3	PRIVE	No	73	0	0
1	0	Thoracotoı	3	PRIVE	No	70	0	0
1	0	Thoracotoı	3	PRIVE	No	72	0	0
1	0	Thoracotoı	3	PRIVE	No	84	0	0
1	0	Thoracotoı	3	PRIVE	No	66	0	0
1	0	Thoracotoı	3	PRIVE	No	72	0	0
1	0	VATS or rc	3	PRIVE	No	78	0	0

1	1	Thoracotoı	3	PRIVE	No	62	0	0
1	1	Thoracotoı	3	PRIVE	No	68	0	0
1	0	Thoracotoı	3	PRIVE	No	50	0	0
1	0	Thoracotoı	3	PRIVE	No	55	0	0
1	0	VATS or rc	3	PRIVE	No	66	0	0
1	0	Thoracotoı	3	PRIVE	No	56	0	0
1	0	Thoracotoı	3	PRIVE	No	52	0	1
1	1	Thoracotoı	3	PRIVE	No	53	0	0
1	0	Thoracotoı	3	PRIVE	No	59	0	0
1	0	Thoracotoı	3	PRIVE	No	59	0	0
1	0	Thoracotoı	3	PRIVE	No	56	0	0
1	0	Thoracotoı	3	PRIVE	No	58	0	0
1	0	Thoracotoı	3	PRIVE	No	58	0	0
1	1	Thoracotoı	3	PRIVE	No	73	0	0
1	0	Thoracotoı	3	PRIVE	No	77	0	0
1	0	Thoracotoı	3	PRIVE	No	66	0	0
1	0	Thoracotoı	3	PRIVE	No	74	0	0
1	0	Thoracotoı	3	PRIVE	No	59	0	0
1	0	Thoracotoı	3	PRIVE	No	54	0	1
1	0	Thoracotoı	3	PRIVE	No	43	0	0
1	0	Thoracotoı	3	PRIVE	No	69	0	0
1	0	Thoracotoı	3	PRIVE	No	59	0	0
1	0	Thoracotoı	3	PRIVE	No	67	0	0
1	0	Thoracotoı	3	PRIVE	No	41	0	0
1	1	Thoracotoı	3	PRIVE	No	64	0	0
1	0	Thoracotoı	3	PRIVE	No	57	1	0
1	0	Thoracotoı	3	PRIVE	No	48	0	0
1	0	Thoracotoı	3	PRIVE	No	54	0	0
1	0	Thoracotoı	3	PRIVE	No	48	0	0
1	0	Thoracotoı	3	PRIVE	Yes	74	1	0
1	0	Thoracotoı	3	PRIVE	No	67	0	0
1	0	Thoracotoı	3	PRIVE	No	69	0	0
1	0	Thoracotoı	3	PRIVE	No	79	0	0
1	0	Thoracotoı	3	PRIVE	No	54	0	0
1	0	Thoracotoı	3	PRIVE	No	53	0	0
1	0	Thoracotoı	3	PRIVE	No	62	0	1
1	0	Thoracotoı	3	PRIVE	No	78	0	0
1	0	Thoracotoı	3	PRIVE	No	62	0	0
1	1	Thoracotoı	3	PRIVE	No	73	0	0
1	0	Thoracotoı	3	PRIVE	No	68	0	0
1	0	Thoracotoı	3	PRIVE	No	52	0	0
1	0	Thoracotoı	3	PRIVE	No	51	0	1
1	0	Thoracotoı	3	PRIVE	No	47	0	0
1	0	Thoracotoı	3	PRIVE	No	61	0	0
1	0	Thoracotoı	3	PRIVE	No	56	0	0
1	0	Thoracotoı	3	PRIVE	No	69	0	0
1	0	Thoracotoı	3	PRIVE	No	61	0	0
1	0	Thoracotoı	3	PRIVE	No	65	1	0
1	0	Thoracotoı	3	PRIVE	No	59	0	0
1	0	Thoracotoı	3	PRIVE	No	84	0	0
1	0	Thoracotoı	3	PRIVE	No	83	0	0
1	0	Thoracotoı	3	PRIVE	No	55	0	1
1	0	Thoracotoı	3	PRIVE	No	60	0	0
1	0	Thoracotoı	3	PRIVE	No	70	1	0
1	0	Thoracotoı	3	PRIVE	No	77	0	0
1	0	Thoracotoı	3	PRIVE	No	74	0	0

1	0	Thoracotoı	3	PRIVE	No	52	0	0
1	0	Thoracotoı	3	PRIVE	No	59	0	0
1	0	Thoracotoı	3	PRIVE	No	51	0	0
1	0	Thoracotoı	3	PRIVE	No	48	0	0
1	0	Thoracotoı	3	PRIVE	No	43	0	0
1	1	Thoracotoı	3	PRIVE	No	57	0	1
1	0	Thoracotoı	3	PRIVE	No	53	0	1
1	0	Thoracotoı	3	PRIVE	No	68	0	0
1	0	Thoracotoı	3	PRIVE	No	65	0	0
1	0	Thoracotoı	3	PRIVE	No	65	0	0
1	0	Thoracotoı	3	PRIVE	No	60	0	0
1	0	Thoracotoı	3	PRIVE	No	71	0	0
1	0	Thoracotoı	3	PRIVE	No	71	0	1
1	0	Thoracotoı	3	PRIVE	No	77	0	0
1	0	Thoracotoı	3	PRIVE	No	51	0	0
1	0	Thoracotoı	3	PRIVE	No	77	0	0
1	0	Thoracotoı	3	PRIVE	No	62	0	0
1	0	Thoracotoı	3	PRIVE	No	51	0	1
1	0	Thoracotoı	3	PRIVE	No	72	0	0
1	0	Thoracotoı	3	PRIVE	No	77	0	0
1	0	Thoracotoı	3	PRIVE	No	60	0	0
1	0	Thoracotoı	3	PRIVE	No	68	0	0
1	0	Thoracotoı	3	PRIVE	No	47	0	1
1	0	VATS or rc	3	PRIVE	No	81	0	0
1	0	Thoracotoı	3	PRIVE	No	66	0	0
1	0	Thoracotoı	3	PRIVE	No	60	0	0
1	0	Thoracotoı	3	PRIVE	No	61	0	0
1	0	Thoracotoı	3	PRIVE	No	57	0	0
1	0	VATS or rc	3	PRIVE	No	54	0	1
1	0	Thoracotoı	3	PRIVE	No	69	0	0
1	0	Thoracotoı	3	PRIVE	No	71	0	0
1	0	Thoracotoı	3	PRIVE	No	77	0	0
1	0	Thoracotoı	3	PRIVE	No	66	0	0
1	0	Thoracotoı	3	PRIVE	No	70	1	0
1	0	Thoracotoı	3	PRIVE	No	80	0	0
1	0	Thoracotoı	3	PRIVE	No	65	0	0
1	0	Thoracotoı	3	PRIVE	No	66	0	1
1	0	Thoracotoı	3	PRIVE	No	59	0	1
1	0	Thoracotoı	3	PRIVE	No	63	0	0
1	0	Thoracotoı	3	PRIVE	No	76	0	0
1	0	Thoracotoı	3	PRIVE	No	46	0	1
1	1	Thoracotoı	3	PRIVE	No	76	0	0
1	0	Thoracotoı	3	PRIVE	No	62	0	0
1	0	Thoracotoı	3	PRIVE	No	46	0	1
1	0	Thoracotoı	3	PRIVE	No	50	0	0
1	1	Thoracotoı	3	PRIVE	No	56	0	0
1	0	Thoracotoı	3	PRIVE	No	56	0	0
1	0	Thoracotoı	3	PRIVE	No	57	0	1
1	0	Thoracotoı	3	PRIVE	No	53	0	0
1	0	Thoracotoı	3	PRIVE	No	70	0	0
1	0	Thoracotoı	3	PRIVE	No	61	0	0
1	0	Thoracotoı	3	PRIVE	No	72	0	0
1	0	Thoracotoı	3	PRIVE	No	61	0	0
1	0	Thoracotoı	3	PRIVE	No	63	1	0
1	0	Thoracotoı	3	PRIVE	No	20	0	0
1	1	Thoracotoı	3	PRIVE	No	66	0	0

1	0	VATS or rc	3	PRIVE	No	67	0	0
1	0	Thoracotoı	3	PRIVE	No	66	0	0
1	0	Thoracotoı	3	PRIVE	No	56	0	0
1	0	VATS or rc	3	PRIVE	No	46	0	0
1	0	Thoracotoı	3	PRIVE	No	75	0	0
1	1	Thoracotoı	3	PRIVE	No	65	0	0
1	0	Thoracotoı	3	PRIVE	No	55	0	0
1	0	VATS or rc	3	PRIVE	No	74	0	0
1	0	Thoracotoı	3	PRIVE	No	64	0	0
1	0	Thoracotoı	3	PRIVE	No	59	0	0
1	0	Thoracotoı	3	PRIVE	No	68	0	0
1	0	Thoracotoı	3	PRIVE	No	84	0	0
1	1	Thoracotoı	3	PRIVE	No	58	0	0
0	0	Thoracotoı	218	CHU	No	78	0	1
0	0	Thoracotoı	218	CHU	No	60	0	1
0	0	Thoracotoı	218	CHU	No	55	0	0
0	0	Thoracotoı	218	CHU	No	58	0	1
0	0	Thoracotoı	218	CHU	No	71	1	1
0	1	Thoracotoı	218	CHU	No	61	0	1
0	0	Thoracotoı	218	CHU	No	62	0	1
0	0	Thoracotoı	218	CHU	No	71	1	0
0	0	Thoracotoı	218	CHU	No	60	0	1
0	0	Thoracotoı	218	CHU	No	56	0	1
0	1	Thoracotoı	218	CHU	No	53	0	1
0	0	Thoracotoı	218	CHU	No	60	0	1
0	0	Thoracotoı	218	CHU	Yes	83	0	0
0	0	Thoracotoı	218	CHU	Yes	73	0	1
0	0	Thoracotoı	218	CHU	No	63	0	0
0	0	Thoracotoı	218	CHU	No	58	0	0
0	0	Thoracotoı	218	CHU	No	44	0	0
0	0	Thoracotoı	218	CHU	No	79	0	1
0	0	Thoracotoı	218	CHU	No	73	1	1
0	0	Thoracotoı	218	CHU	No	67	0	1
0	0	Thoracotoı	218	CHU	No	75	0	1
0	0	Thoracotoı	218	CHU	No	58	0	0
0	0	Thoracotoı	218	CHU	No	69	0	0
0	0	Thoracotoı	218	CHU	No	54	0	1
0	0	Thoracotoı	218	CHU	No	66	0	1
0	0	Thoracotoı	218	CHU	No	67	1	1
0	0	Thoracotoı	218	CHU	Yes	60	0	0
0	0	Thoracotoı	218	CHU	No	57	0	1
0	0	Thoracotoı	218	CHU	No	81	1	0
0	0	Thoracotoı	218	CHU	No	72	1	0
0	0	Thoracotoı	218	CHU	No	62	0	1
0	0	Thoracotoı	218	CHU	No	64	0	0
0	0	Thoracotoı	218	CHU	No	60	0	1
0	0	Thoracotoı	218	CHU	No	40	0	1
0	0	Thoracotoı	218	CHU	No	63	1	1
0	0	Thoracotoı	218	CHU	No	63	0	1
0	0	Thoracotoı	218	CHU	No	56	0	0
0	0	Thoracotoı	218	CHU	No	57	1	1
0	0	Thoracotoı	218	CHU	No	50	0	0
0	0	Thoracotoı	218	CHU	No	81	1	0
0	0	Thoracotoı	218	CHU	No	70	0	1
0	0	Thoracotoı	218	CHU	No	62	0	1
0	0	VATS or rc	218	CHU	No	57	0	1

0	0	Thoracotoı	218	CHU	No	41	0	0
0	0	Thoracotoı	218	CHU	No	62	0	1
0	0	Thoracotoı	218	CHU	No	76	0	0
0	0	VATS or rc	218	CHU	No	63	0	1
0	0	Thoracotoı	218	CHU	No	56	0	1
0	0	Thoracotoı	218	CHU	No	69	0	0
0	0	Thoracotoı	218	CHU	No	69	0	0
0	0	Thoracotoı	218	CHU	No	78	1	1
0	0	Thoracotoı	218	CHU	No	73	1	1
0	0	Thoracotoı	218	CHU	No	68	0	1
0	0	VATS or rc	218	CHU	No	69	0	1
0	0	Thoracotoı	218	CHU	No	60	0	1
0	1	Thoracotoı	218	CHU	No	71	0	1
0	0	Thoracotoı	218	CHU	No	58	0	0
0	0	VATS or rc	218	CHU	No	57	0	1
0	1	Thoracotoı	218	CHU	No	65	0	1
0	0	Thoracotoı	218	CHU	No	71	0	0
0	0	Thoracotoı	218	CHU	No	52	0	1
0	0	Thoracotoı	218	CHU	No	68	0	1
0	0	Thoracotoı	218	CHU	No	51	0	1
0	0	Thoracotoı	218	CHU	No	67	0	1
0	0	Thoracotoı	218	CHU	No	56	0	0
0	0	Thoracotoı	218	CHU	No	71	1	0
0	0	Thoracotoı	218	CHU	No	45	0	1
0	0	VATS or rc	218	CHU	No	68	0	1
0	0	VATS or rc	218	CHU	No	71	1	1
0	0	Thoracotoı	218	CHU	No	60	0	1
0	0	Thoracotoı	218	CHU	No	64	0	1
0	0	VATS or rc	218	CHU	No	68	1	1
0	0	VATS or rc	218	CHU	No	61	0	0
0	0	Thoracotoı	218	CHU	No	62	0	1
0	1	Thoracotoı	218	CHU	No	81	0	0
0	0	Thoracotoı	218	CHU	No	73	0	1
0	0	Thoracotoı	218	CHU	No	66	0	1
0	0	Thoracotoı	218	CHU	No	62	0	0
0	0	Thoracotoı	218	CHU	No	57	0	1
0	0	Thoracotoı	218	CHU	No	68	0	0
0	0	Thoracotoı	218	CHU	No	75	0	0
0	0	Thoracotoı	218	CHU	No	75	0	1
0	0	Thoracotoı	218	CHU	No	50	0	0
0	0	Thoracotoı	218	CHU	No	68	1	0
0	0	VATS or rc	218	CHU	No	57	0	0
0	1	Thoracotoı	218	CHU	No	55	0	1
0	0	Thoracotoı	218	CHU	No	68	0	0
0	0	Thoracotoı	218	CHU	No	84	0	0
0	0	Thoracotoı	218	CHU	No	55	0	1
0	0	Thoracotoı	218	CHU	No	53	0	1
0	1	Thoracotoı	218	CHU	No	46	0	1
0	0	Thoracotoı	218	CHU	No	75	0	1
0	0	Thoracotoı	218	CHU	No	60	0	1
0	0	Thoracotoı	218	CHU	No	73	1	1
0	0	Thoracotoı	218	CHU	No	71	0	1
0	0	Thoracotoı	218	CHU	No	67	1	1
0	0	Thoracotoı	218	CHU	No	73	1	1
0	1	Thoracotoı	218	CHU	No	63	0	1
0	0	Thoracotoı	218	CHU	No	49	0	1

0	0	Thoracotoı	218	CHU	No	47	0	0
0	0	Thoracotoı	218	CHU	No	56	1	1
0	1	Thoracotoı	218	CHU	No	29	0	1
0	0	Thoracotoı	218	CHU	No	55	0	1
0	0	Thoracotoı	218	CHU	No	49	0	1
0	0	Thoracotoı	218	CHU	No	59	1	0
0	0	Thoracotoı	218	CHU	No	61	1	0
0	0	Thoracotoı	218	CHU	No	70	0	1
0	0	VATS or rc	218	CHU	No	74	0	1
0	0	Thoracotoı	218	CHU	No	40	0	1
0	0	Thoracotoı	218	CHU	No	69	0	0
0	1	Thoracotoı	218	CHU	No	59	0	1
0	0	Thoracotoı	218	CHU	No	64	0	1
0	0	Thoracotoı	218	CHU	No	72	0	1
0	0	Thoracotoı	218	CHU	No	58	0	1
0	0	Thoracotoı	218	CHU	No	69	0	0
0	0	Thoracotoı	218	CHU	No	61	0	0
0	0	Thoracotoı	218	CHU	No	67	0	0
0	0	Thoracotoı	218	CHU	No	62	0	1
0	0	Thoracotoı	218	CHU	No	55	0	1
0	0	VATS or rc	218	CHU	No	65	0	1
0	0	Thoracotoı	218	CHU	No	82	0	0
0	0	Thoracotoı	218	CHU	No	47	0	1
0	0	Thoracotoı	218	CHU	No	21	0	0
0	0	Thoracotoı	218	CHU	No	63	1	1
0	0	Thoracotoı	218	CHU	No	62	0	1
0	0	Thoracotoı	218	CHU	No	61	0	0
0	0	Thoracotoı	218	CHU	No	60	0	1
0	0	Thoracotoı	218	CHU	No	71	0	1
0	0	Thoracotoı	218	CHU	No	59	0	1
0	1	Thoracotoı	218	CHU	No	73	0	1
0	0	VATS or rc	218	CHU	No	58	0	1
0	0	Thoracotoı	218	CHU	No	62	0	0
0	0	Thoracotoı	218	CHU	No	80	1	0
0	0	Thoracotoı	218	CHU	No	65	0	1
0	0	Thoracotoı	218	CHU	No	58	0	1
0	0	Thoracotoı	218	CHU	No	70	0	0
0	0	Thoracotoı	218	CHU	No	58	0	1
0	0	Thoracotoı	218	CHU	No	65	1	1
0	1	Thoracotoı	218	CHU	No	49	0	1
0	0	Thoracotoı	218	CHU	No	65	0	1
0	0	Thoracotoı	218	CHU	No	70	0	1
0	0	Thoracotoı	218	CHU	No	56	0	1
0	0	Thoracotoı	218	CHU	No	75	0	1
0	0	Thoracotoı	218	CHU	No	47	0	0
0	0	Thoracotoı	218	CHU	No	68	0	1
0	0	Thoracotoı	218	CHU	No	60	0	1
0	0	Thoracotoı	218	CHU	No	66	0	1
0	1	Thoracotoı	218	CHU	No	52	0	1
0	0	Thoracotoı	218	CHU	No	64	0	1
0	0	Thoracotoı	218	CHU	No	60	0	0
0	0	Thoracotoı	218	CHU	No	37	0	0
0	0	Thoracotoı	218	CHU	No	51	0	0
0	0	Thoracotoı	218	CHU	No	47	0	0
0	0	Thoracotoı	218	CHU	No	55	0	1
0	0	Thoracotoı	218	CHU	No	79	0	0

0	0	Thoracotoı	218	CHU	No	44	0	1
0	0	Thoracotoı	218	CHU	No	56	1	1
0	0	Thoracotoı	218	CHU	No	66	0	1
0	0	Thoracotoı	218	CHU	No	52	0	1
0	0	Thoracotoı	218	CHU	No	69	0	1
0	0	Thoracotoı	218	CHU	No	66	0	1
0	0	Thoracotoı	218	CHU	No	67	0	1
0	1	Thoracotoı	218	CHU	No	57	0	1
0	0	Thoracotoı	218	CHU	No	62	1	0
0	0	Thoracotoı	218	CHU	No	68	1	1
0	0	Thoracotoı	218	CHU	No	69	0	1
0	0	VATS or rc	218	CHU	No	64	0	0
0	0	Thoracotoı	218	CHU	No	62	0	1
0	0	Thoracotoı	218	CHU	No	68	0	0
0	0	Thoracotoı	218	CHU	No	59	0	1
0	0	Thoracotoı	218	CHU	No	63	1	1
0	0	Thoracotoı	218	CHU	No	53	0	1
0	0	Thoracotoı	218	CHU	No	69	0	0
0	0	Thoracotoı	218	CHU	No	63	0	0
0	0	VATS or rc	218	CHU	No	45	0	0
0	0	Thoracotoı	218	CHU	No	60	0	1
0	0	Thoracotoı	218	CHU	No	71	1	1
0	0	VATS or rc	218	CHU	No	47	0	1
0	0	Thoracotoı	218	CHU	No	77	0	0
0	0	Thoracotoı	218	CHU	No	69	0	1
0	0	Thoracotoı	218	CHU	No	53	0	1
0	1	Thoracotoı	218	CHU	No	54	0	1
0	0	Thoracotoı	218	CHU	No	53	1	1
0	0	Thoracotoı	218	CHU	No	44	0	1
0	0	Thoracotoı	218	CHU	No	60	0	1
0	0	Thoracotoı	218	CHU	No	75	0	0
0	0	Thoracotoı	218	CHU	No	68	0	0
0	0	Thoracotoı	218	CHU	No	62	0	0
0	0	Thoracotoı	218	CHU	No	77	0	0
0	0	Thoracotoı	218	CHU	No	68	0	0
0	0	Thoracotoı	218	CHU	No	74	0	0
0	0	VATS or rc	218	CHU	No	69	0	1
0	0	Thoracotoı	218	CHU	No	68	0	0
0	0	Thoracotoı	218	CHU	No	43	0	0
0	0	Thoracotoı	218	CHU	No	55	0	0
0	0	Thoracotoı	218	CHU	No	62	0	1
0	0	Thoracotoı	218	CHU	No	58	0	1
0	0	Thoracotoı	218	CHU	No	60	0	0
0	0	Thoracotoı	218	CHU	No	66	0	1
0	0	Thoracotoı	218	CHU	No	71	0	1
0	1	Thoracotoı	218	CHU	No	64	0	0
0	0	Thoracotoı	218	CHU	No	55	0	1
0	0	Thoracotoı	218	CHU	No	64	0	0
0	0	Thoracotoı	218	CHU	No	69	0	1
0	0	Thoracotoı	218	CHU	No	71	0	0
0	0	Thoracotoı	218	CHU	No	56	0	1
0	0	Thoracotoı	218	CHU	No	73	0	0
0	0	Thoracotoı	218	CHU	No	67	0	0
0	0	Thoracotoı	218	CHU	No	67	0	1
0	0	Thoracotoı	218	CHU	No	62	0	1
0	0	VATS or rc	218	CHU	No	71	0	1

0	0	Thoracotoı	218	CHU	Yes	78	0	1
0	0	Thoracotoı	218	CHU	No	72	0	0
0	0	Thoracotoı	218	CHU	No	64	0	0
0	1	Thoracotoı	218	CHU	No	47	0	1
0	0	Thoracotoı	218	CHU	No	62	0	1
0	0	Thoracotoı	218	CHU	No	62	0	1
0	0	Thoracotoı	218	CHU	No	68	0	0
0	0	Thoracotoı	218	CHU	No	67	1	1
0	0	Thoracotoı	218	CHU	No	59	0	1
0	0	Thoracotoı	19	PRIVE	No	65	0	0
0	0	Thoracotoı	19	PRIVE	No	63	0	0
0	0	Thoracotoı	19	PRIVE	No	76	0	0
0	0	Thoracotoı	19	PRIVE	No	61	0	0
0	1	Thoracotoı	19	PRIVE	No	59	0	0
0	1	Thoracotoı	19	PRIVE	No	63	0	0
0	0	Thoracotoı	19	PRIVE	No	73	0	0
0	1	Thoracotoı	19	PRIVE	No	43	0	1
0	0	Thoracotoı	19	PRIVE	No	82	0	0
0	0	Thoracotoı	19	PRIVE	No	65	0	0
0	0	Thoracotoı	19	PRIVE	No	60	0	0
0	0	Thoracotoı	19	PRIVE	No	67	0	0
0	0	Thoracotoı	19	PRIVE	No	73	0	0
0	0	Thoracotoı	19	PRIVE	No	60	0	0
0	0	Thoracotoı	19	PRIVE	No	57	1	0
0	0	Thoracotoı	19	PRIVE	No	67	1	0
0	0	Thoracotoı	40	PRIVE	No	66	0	0
0	0	VATS or rc	40	PRIVE	No	75	0	0
0	0	VATS or rc	40	PRIVE	No	63	0	1
0	0	VATS or rc	40	PRIVE	No	49	0	0
0	0	VATS or rc	40	PRIVE	No	76	0	0
0	0	VATS or rc	40	PRIVE	No	68	1	0
0	0	Thoracotoı	40	PRIVE	No	69	0	0
0	0	VATS or rc	40	PRIVE	No	73	0	0
0	0	Thoracotoı	40	PRIVE	No	67	0	0
0	0	Thoracotoı	40	PRIVE	No	63	0	1
0	0	Thoracotoı	40	PRIVE	No	86	0	0
0	0	VATS or rc	40	PRIVE	No	71	0	1
0	0	VATS or rc	40	PRIVE	No	78	0	0
0	0	Thoracotoı	40	PRIVE	No	62	0	0
0	0	VATS or rc	40	PRIVE	No	51	0	0
0	0	VATS or rc	40	PRIVE	No	54	0	0
0	0	Thoracotoı	40	PRIVE	No	64	0	1
0	0	VATS or rc	40	PRIVE	No	88	0	0
0	0	Thoracotoı	40	PRIVE	No	85	0	0
0	0	VATS or rc	40	PRIVE	No	66	0	0
0	0	VATS or rc	40	PRIVE	No	70	0	0
0	0	Thoracotoı	40	PRIVE	No	53	0	0
0	0	VATS or rc	40	PRIVE	No	67	0	0
0	0	VATS or rc	40	PRIVE	No	83	0	0
0	0	VATS or rc	40	PRIVE	No	53	0	0
0	0	VATS or rc	40	PRIVE	No	60	0	0
0	0	VATS or rc	40	PRIVE	No	77	0	0
0	0	VATS or rc	40	PRIVE	No	50	0	0
0	0	VATS or rc	40	PRIVE	No	83	0	0
0	0	Thoracotoı	64	PRIVE	No	67	0	0
0	0	VATS or rc	64	PRIVE	No	72	0	0

0	1	VATS or rc	64	PRIVE	No	67	0	0
0	0	Thoracotoı	64	PRIVE	No	66	0	0
0	0	VATS or rc	64	PRIVE	No	66	0	0
0	0	VATS or rc	64	PRIVE	No	71	1	0
0	0	VATS or rc	64	PRIVE	No	67	0	0
0	0	VATS or rc	64	PRIVE	No	53	0	0
0	0	Thoracotoı	64	PRIVE	No	78	1	0
0	0	VATS or rc	64	PRIVE	No	61	0	0
0	0	Thoracotoı	64	PRIVE	No	61	0	0
0	0	VATS or rc	64	PRIVE	No	64	0	0
0	1	Thoracotoı	64	PRIVE	No	65	0	0
0	0	Thoracotoı	64	PRIVE	No	75	1	0
0	0	VATS or rc	64	PRIVE	No	64	0	0
0	0	VATS or rc	64	PRIVE	No	53	0	0
0	0	Thoracotoı	64	PRIVE	No	71	0	0
0	0	Thoracotoı	64	PRIVE	No	73	0	0
0	0	VATS or rc	64	PRIVE	No	65	0	0
0	0	Thoracotoı	64	PRIVE	No	46	0	0
0	0	Thoracotoı	64	PRIVE	No	66	0	0
0	0	VATS or rc	64	PRIVE	No	79	0	0
0	1	VATS or rc	64	PRIVE	No	37	0	0
0	0	VATS or rc	64	PRIVE	No	69	0	0
0	0	Thoracotoı	64	PRIVE	No	65	0	0
0	0	Thoracotoı	64	PRIVE	No	68	0	0
0	0	Thoracotoı	64	PRIVE	No	74	0	0
0	0	Thoracotoı	64	PRIVE	No	70	0	1
0	0	VATS or rc	64	PRIVE	No	58	0	0
0	0	VATS or rc	64	PRIVE	No	79	0	0
0	0	VATS or rc	64	PRIVE	No	65	0	0
0	0	VATS or rc	64	PRIVE	No	73	1	0
0	0	VATS or rc	64	PRIVE	No	63	0	0
0	1	VATS or rc	64	PRIVE	No	67	0	0
0	0	VATS or rc	64	PRIVE	No	57	0	0
0	0	Thoracotoı	64	PRIVE	No	53	0	0
0	0	VATS or rc	64	PRIVE	No	65	0	0
0	1	VATS or rc	64	PRIVE	No	37	0	0
0	0	VATS or rc	64	PRIVE	No	58	0	0
0	0	VATS or rc	64	PRIVE	No	75	0	0
0	0	VATS or rc	64	PRIVE	No	69	0	0
0	0	VATS or rc	64	PRIVE	No	51	0	0
0	0	Thoracotoı	64	PRIVE	No	54	0	0
0	0	VATS or rc	64	PRIVE	No	56	0	0
0	0	VATS or rc	64	PRIVE	No	60	0	0
0	1	Thoracotoı	64	PRIVE	No	74	0	0
0	0	VATS or rc	64	PRIVE	No	70	0	0
0	0	VATS or rc	64	PRIVE	No	70	0	0
0	0	VATS or rc	64	PRIVE	No	61	0	0
0	0	VATS or rc	64	PRIVE	No	73	0	0
0	0	VATS or rc	64	PRIVE	No	65	0	0
0	0	Thoracotoı	64	PRIVE	No	65	0	0
0	0	VATS or rc	64	PRIVE	No	66	0	0
0	0	VATS or rc	64	PRIVE	No	59	0	0
0	0	VATS or rc	64	PRIVE	No	59	0	0
0	1	Thoracotoı	64	PRIVE	No	66	0	0
0	0	VATS or rc	64	PRIVE	No	59	0	0
0	0	VATS or rc	64	PRIVE	No	46	0	0

0	0	VATS or rc	64	PRIVE	No	59	0	0
0	0	Thoracotoı	64	PRIVE	No	76	0	0
0	0	VATS or rc	64	PRIVE	No	79	0	0
0	0	Thoracotoı	64	PRIVE	No	63	0	0
0	1	Thoracotoı	64	PRIVE	No	61	0	0
0	0	Thoracotoı	64	PRIVE	No	73	0	0
0	0	VATS or rc	64	PRIVE	No	47	0	1
0	0	VATS or rc	64	PRIVE	No	75	0	0
0	0	VATS or rc	64	PRIVE	No	71	1	0
0	0	Thoracotoı	64	PRIVE	No	82	0	0
0	0	Thoracotoı	64	PRIVE	No	61	0	0
0	0	VATS or rc	64	PRIVE	No	60	0	0
0	0	VATS or rc	64	PRIVE	No	66	0	0
0	0	VATS or rc	64	PRIVE	No	57	0	0
0	0	VATS or rc	64	PRIVE	No	76	0	0
0	0	VATS or rc	64	PRIVE	No	54	0	1
0	0	VATS or rc	64	PRIVE	No	37	0	0
0	0	Thoracotoı	64	PRIVE	No	67	0	0
0	0	Thoracotoı	64	PRIVE	No	75	0	0
0	0	VATS or rc	64	PRIVE	No	63	0	1
0	1	Thoracotoı	64	PRIVE	No	67	0	0
0	0	VATS or rc	64	PRIVE	No	62	0	0
0	0	VATS or rc	64	PRIVE	No	67	0	0
0	0	VATS or rc	64	PRIVE	No	58	0	0
0	0	VATS or rc	64	PRIVE	No	64	0	0
0	0	VATS or rc	64	PRIVE	No	64	0	0
0	0	Thoracotoı	64	PRIVE	No	69	1	0
0	0	Thoracotoı	64	PRIVE	No	46	0	1
0	0	Thoracotoı	64	PRIVE	No	62	1	0
0	0	VATS or rc	64	PRIVE	No	65	0	0
0	0	VATS or rc	64	PRIVE	No	72	0	0
0	0	VATS or rc	64	PRIVE	No	66	0	0
0	0	VATS or rc	64	PRIVE	No	65	0	0
0	1	Thoracotoı	64	PRIVE	No	56	0	0
0	0	Thoracotoı	64	PRIVE	No	47	0	0
0	0	VATS or rc	64	PRIVE	No	70	1	0
0	0	Thoracotoı	64	PRIVE	No	63	1	0
0	0	Thoracotoı	64	PRIVE	No	51	0	1
0	0	VATS or rc	64	PRIVE	No	47	0	0
0	0	VATS or rc	64	PRIVE	No	54	0	1
0	0	VATS or rc	64	PRIVE	No	57	0	0
0	0	VATS or rc	64	PRIVE	No	50	0	0
0	0	VATS or rc	64	PRIVE	No	58	0	0
0	0	VATS or rc	64	PRIVE	No	62	1	0
0	0	VATS or rc	64	PRIVE	No	70	0	0
0	0	Thoracotoı	64	PRIVE	No	40	0	0
0	0	VATS or rc	64	PRIVE	No	62	0	0
0	0	VATS or rc	64	PRIVE	No	73	1	0
0	0	VATS or rc	64	PRIVE	No	51	1	0
0	0	VATS or rc	64	PRIVE	No	58	0	0
0	0	VATS or rc	64	PRIVE	No	69	0	0
0	0	VATS or rc	64	PRIVE	No	68	0	0
0	1	Thoracotoı	64	PRIVE	No	66	0	0
0	0	VATS or rc	64	PRIVE	No	74	0	0
0	0	Thoracotoı	64	PRIVE	No	71	0	1
0	0	VATS or rc	64	PRIVE	No	65	0	0

0	0	Thoracotoı	64	PRIVE	No	46	0	0
0	1	Thoracotoı	64	PRIVE	No	65	1	0
0	0	VATS or rc	64	PRIVE	No	44	0	0
0	0	VATS or rc	64	PRIVE	No	70	0	0
0	0	Thoracotoı	64	PRIVE	No	80	0	0
0	0	VATS or rc	64	PRIVE	No	55	0	0
0	0	Thoracotoı	64	PRIVE	No	58	0	0
0	0	VATS or rc	64	PRIVE	No	51	0	1
0	0	VATS or rc	64	PRIVE	No	80	0	0
0	0	VATS or rc	64	PRIVE	No	67	0	0
0	0	VATS or rc	64	PRIVE	No	67	1	0
0	0	VATS or rc	64	PRIVE	No	56	0	0
0	0	Thoracotoı	64	PRIVE	No	78	1	0
0	0	VATS or rc	64	PRIVE	No	61	0	0
0	0	Thoracotoı	64	PRIVE	No	63	0	0
0	0	Thoracotoı	64	PRIVE	No	60	0	0
0	0	VATS or rc	64	PRIVE	No	68	0	0
0	0	VATS or rc	64	PRIVE	No	73	0	0
0	0	VATS or rc	64	PRIVE	No	76	0	0
0	1	Thoracotoı	64	PRIVE	No	58	0	0
0	0	VATS or rc	64	PRIVE	No	59	1	0
0	0	VATS or rc	64	PRIVE	No	70	1	0
0	0	VATS or rc	64	PRIVE	No	68	0	0
0	0	Thoracotoı	64	PRIVE	No	67	0	0
0	0	VATS or rc	64	PRIVE	No	69	0	0
0	1	Thoracotoı	64	PRIVE	No	63	0	0
0	0	VATS or rc	64	PRIVE	No	52	0	0
0	0	Thoracotoı	64	PRIVE	No	65	0	0
0	0	VATS or rc	64	PRIVE	No	74	0	0
0	0	VATS or rc	64	PRIVE	No	70	0	0
0	0	VATS or rc	64	PRIVE	No	57	0	0
0	0	VATS or rc	64	PRIVE	Yes	77	0	0
0	0	VATS or rc	64	PRIVE	No	74	0	0
0	0	VATS or rc	64	PRIVE	No	71	0	0
0	0	VATS or rc	64	PRIVE	No	71	0	0
0	0	VATS or rc	64	PRIVE	No	80	0	0
0	0	Thoracotoı	64	PRIVE	No	63	0	0
0	0	VATS or rc	64	PRIVE	No	49	0	0
0	0	VATS or rc	64	PRIVE	No	57	0	0
0	0	VATS or rc	64	PRIVE	No	75	0	0
0	0	VATS or rc	64	PRIVE	No	70	0	0
0	1	Thoracotoı	64	PRIVE	No	58	0	1
0	0	VATS or rc	64	PRIVE	No	61	0	0
0	0	VATS or rc	64	PRIVE	No	58	0	0
0	0	VATS or rc	64	PRIVE	No	67	1	0
0	0	Thoracotoı	64	PRIVE	No	79	0	0
0	0	Thoracotoı	64	PRIVE	No	61	0	0
0	0	Thoracotoı	64	PRIVE	No	72	0	0
0	0	Thoracotoı	64	PRIVE	No	70	0	1
0	0	VATS or rc	64	PRIVE	No	48	0	1
0	0	VATS or rc	64	PRIVE	No	69	0	0
0	0	Thoracotoı	64	PRIVE	No	53	0	0
0	0	VATS or rc	64	PRIVE	No	66	0	0
0	0	Thoracotoı	64	PRIVE	No	27	0	0
0	0	Thoracotoı	64	PRIVE	No	68	0	0
0	1	Thoracotoı	64	PRIVE	No	47	0	1

0	1	Thoracotoı	64	PRIVE	No	56	0	0
0	0	VATS or rc	64	PRIVE	No	55	0	0
0	0	VATS or rc	64	PRIVE	No	62	1	0
0	0	Thoracotoı	64	PRIVE	No	54	0	0
0	1	Thoracotoı	64	PRIVE	No	57	0	0
0	0	VATS or rc	64	PRIVE	No	64	0	0
0	0	VATS or rc	64	PRIVE	No	59	0	0
0	0	Thoracotoı	64	PRIVE	No	65	0	0
0	0	VATS or rc	78	PRIVE	No	66	0	1
0	0	Thoracotoı	78	PRIVE	No	68	0	0
0	0	VATS or rc	78	PRIVE	No	62	0	0
0	1	Thoracotoı	78	PRIVE	No	63	0	0
0	0	VATS or rc	78	PRIVE	No	82	0	0
0	0	VATS or rc	78	PRIVE	No	68	1	0
0	0	VATS or rc	78	PRIVE	No	64	0	0
0	0	Thoracotoı	78	PRIVE	No	64	0	0
0	0	VATS or rc	78	PRIVE	No	65	0	1
0	0	VATS or rc	78	PRIVE	No	73	0	0
0	0	VATS or rc	78	PRIVE	No	70	1	0
0	0	VATS or rc	78	PRIVE	No	83	0	0
0	0	VATS or rc	78	PRIVE	No	72	0	0
0	1	Thoracotoı	78	PRIVE	No	48	0	1
0	0	VATS or rc	78	PRIVE	No	72	0	0
0	0	VATS or rc	78	PRIVE	No	49	0	1
0	0	VATS or rc	78	PRIVE	No	67	1	0
0	0	VATS or rc	78	PRIVE	No	82	0	0
0	0	VATS or rc	78	PRIVE	No	76	1	1
0	0	VATS or rc	78	PRIVE	No	66	0	0
0	0	VATS or rc	78	PRIVE	No	64	1	0
0	1	Thoracotoı	78	PRIVE	No	47	0	0
0	0	Thoracotoı	78	PRIVE	No	66	1	0
0	0	VATS or rc	78	PRIVE	No	67	0	1
0	0	VATS or rc	78	PRIVE	No	56	0	0
0	0	Thoracotoı	78	PRIVE	No	63	0	0
0	0	VATS or rc	78	PRIVE	No	71	0	1
0	0	Thoracotoı	78	PRIVE	No	78	0	0
0	0	VATS or rc	78	PRIVE	No	72	0	0
0	0	VATS or rc	78	PRIVE	No	58	0	0
0	0	VATS or rc	78	PRIVE	No	79	0	0
0	0	VATS or rc	78	PRIVE	No	65	1	1
0	0	VATS or rc	78	PRIVE	No	80	1	0
0	0	VATS or rc	78	PRIVE	No	76	0	0
0	0	Thoracotoı	78	PRIVE	No	73	0	0
0	0	VATS or rc	78	PRIVE	No	58	0	1
0	0	VATS or rc	78	PRIVE	No	70	0	0
0	0	Thoracotoı	78	PRIVE	No	76	0	0
0	0	VATS or rc	78	PRIVE	No	79	0	0
0	0	VATS or rc	78	PRIVE	No	61	0	0
0	0	VATS or rc	78	PRIVE	No	66	0	1
0	0	VATS or rc	78	PRIVE	No	59	0	0
0	0	VATS or rc	78	PRIVE	No	83	0	0
0	0	Thoracotoı	78	PRIVE	No	61	0	1
0	0	VATS or rc	78	PRIVE	No	69	0	0
0	0	Thoracotoı	78	PRIVE	No	51	0	0
0	0	VATS or rc	78	PRIVE	Yes	70	0	0
0	0	VATS or rc	78	PRIVE	No	77	1	0

0	0	Thoracotoı	78	PRIVE	No	54	0	1
0	0	Thoracotoı	78	PRIVE	No	73	1	0
0	0	VATS or ro	78	PRIVE	No	51	0	1
0	0	Thoracotoı	78	PRIVE	No	57	0	0
0	0	Thoracotoı	78	PRIVE	No	57	0	0
0	0	Thoracotoı	78	PRIVE	No	71	0	0
0	0	Thoracotoı	78	PRIVE	No	56	0	1
0	0	VATS or ro	78	PRIVE	No	61	1	0
0	0	VATS or ro	78	PRIVE	No	73	0	0
0	0	VATS or ro	78	PRIVE	No	57	0	0
0	0	VATS or ro	78	PRIVE	No	52	0	0
0	0	VATS or ro	78	PRIVE	No	44	0	0
0	0	VATS or ro	78	PRIVE	No	68	0	0
0	0	VATS or ro	78	PRIVE	No	66	0	0
0	0	VATS or ro	78	PRIVE	No	86	0	0
0	0	VATS or ro	78	PRIVE	No	81	0	0
0	0	VATS or ro	78	PRIVE	No	78	0	0
0	0	Thoracotoı	78	PRIVE	No	71	0	0
0	0	VATS or ro	78	PRIVE	No	54	0	0
0	0	VATS or ro	78	PRIVE	No	60	0	0
0	0	VATS or ro	78	PRIVE	No	57	0	1
0	0	VATS or ro	78	PRIVE	No	71	0	0
0	0	VATS or ro	78	PRIVE	No	63	0	0
0	0	Thoracotoı	41	PRIVE	No	47	0	0
0	0	VATS or ro	41	PRIVE	No	51	1	0
0	0	Thoracotoı	41	PRIVE	No	63	0	0
0	0	VATS or ro	41	PRIVE	No	73	0	0
0	0	Thoracotoı	41	PRIVE	No	79	0	0
0	0	Thoracotoı	41	PRIVE	No	84	0	0
0	0	Thoracotoı	41	PRIVE	No	54	1	0
0	0	Thoracotoı	41	PRIVE	No	52	0	0
0	0	Thoracotoı	41	PRIVE	No	63	0	0
0	0	Thoracotoı	41	PRIVE	No	77	0	0
0	0	Thoracotoı	41	PRIVE	No	63	0	0
0	0	Thoracotoı	41	PRIVE	No	48	0	0
0	0	Thoracotoı	41	PRIVE	No	68	0	0
0	0	VATS or ro	41	PRIVE	No	67	0	1
0	0	Thoracotoı	41	PRIVE	No	69	0	0
0	0	Thoracotoı	41	PRIVE	No	62	0	0
0	0	Thoracotoı	41	PRIVE	No	72	0	0
0	0	VATS or ro	41	PRIVE	No	57	0	0
0	0	Thoracotoı	41	PRIVE	No	80	0	0
0	0	Thoracotoı	41	PRIVE	No	63	0	0
0	0	Thoracotoı	41	PRIVE	No	70	0	0
0	1	Thoracotoı	41	PRIVE	No	64	0	0
0	0	Thoracotoı	41	PRIVE	No	61	0	0
0	0	VATS or ro	41	PRIVE	No	58	0	0
0	0	Thoracotoı	41	PRIVE	No	50	0	0
0	0	VATS or ro	41	PRIVE	No	74	0	1
0	0	Thoracotoı	41	PRIVE	No	57	0	1
0	0	VATS or ro	41	PRIVE	No	63	0	1
0	0	VATS or ro	41	PRIVE	No	77	0	0
0	0	Thoracotoı	41	PRIVE	No	51	0	0
0	0	Thoracotoı	41	PRIVE	No	72	0	0
0	0	Thoracotoı	41	PRIVE	No	68	0	0
0	0	Thoracotoı	41	PRIVE	No	62	0	0

0	0	Thoracotoı	41	PRIVE	No	58	0	0
0	1	Thoracotoı	41	PRIVE	No	48	0	0
0	0	VATS or rc	41	PRIVE	No	58	0	0
0	0	Thoracotoı	41	PRIVE	No	56	0	0
0	0	Thoracotoı	41	PRIVE	No	75	1	0
0	0	Thoracotoı	41	PRIVE	No	56	0	0
0	0	Thoracotoı	41	PRIVE	No	53	0	0
0	0	VATS or rc	41	PRIVE	No	63	0	0
0	0	Thoracotoı	41	PRIVE	No	68	1	1
0	0	Thoracotoı	41	PRIVE	No	56	0	0
0	0	VATS or rc	41	PRIVE	No	67	0	1
0	0	Thoracotoı	41	PRIVE	No	57	0	0
0	0	Thoracotoı	41	PRIVE	No	79	0	0
0	0	Thoracotoı	41	PRIVE	No	75	0	0
0	0	VATS or rc	41	PRIVE	No	74	0	0
0	0	Thoracotoı	41	PRIVE	No	70	0	0
0	0	Thoracotoı	41	PRIVE	No	59	0	0
0	0	Thoracotoı	41	PRIVE	No	56	0	0
0	0	Thoracotoı	41	PRIVE	No	62	0	0
0	0	Thoracotoı	41	PRIVE	No	51	0	0
0	0	Thoracotoı	41	PRIVE	No	64	0	0
0	0	Thoracotoı	41	PRIVE	No	55	0	0
0	0	Thoracotoı	41	PRIVE	No	67	0	0
0	0	Thoracotoı	41	PRIVE	No	60	0	0
0	0	VATS or rc	41	PRIVE	No	79	0	1
0	0	Thoracotoı	41	PRIVE	No	74	0	0
0	0	Thoracotoı	41	PRIVE	No	77	0	0
0	0	Thoracotoı	41	PRIVE	No	65	1	0
0	0	VATS or rc	41	PRIVE	No	75	1	0
0	0	Thoracotoı	41	PRIVE	No	64	0	0
0	0	Thoracotoı	41	PRIVE	No	64	1	0
0	0	Thoracotoı	41	PRIVE	No	43	0	0
0	0	Thoracotoı	41	PRIVE	No	63	0	0
0	0	Thoracotoı	41	PRIVE	No	62	0	0
0	0	Thoracotoı	41	PRIVE	No	59	1	0
0	0	Thoracotoı	41	PRIVE	No	66	0	0
0	0	Thoracotoı	41	PRIVE	No	66	0	0
0	0	Thoracotoı	41	PRIVE	No	41	0	0
0	0	Thoracotoı	41	PRIVE	No	55	0	0
0	0	Thoracotoı	41	PRIVE	No	74	1	0
0	0	Thoracotoı	41	PRIVE	No	59	0	0
0	0	Thoracotoı	41	PRIVE	No	75	0	0
0	0	Thoracotoı	41	PRIVE	No	75	0	0
0	0	Thoracotoı	41	PRIVE	No	66	1	0
0	0	Thoracotoı	41	PRIVE	No	65	0	0
0	0	Thoracotoı	41	PRIVE	No	72	0	0
0	0	Thoracotoı	41	PRIVE	No	55	0	0
0	0	VATS or rc	41	PRIVE	No	61	0	0
0	0	VATS or rc	41	PRIVE	No	70	0	0
0	0	Thoracotoı	41	PRIVE	No	61	1	0
0	0	Thoracotoı	41	PRIVE	No	75	0	0
0	0	Thoracotoı	41	PRIVE	No	72	1	0
0	0	VATS or rc	41	PRIVE	No	66	0	1
0	0	Thoracotoı	41	PRIVE	No	51	0	0
0	0	Thoracotoı	41	PRIVE	No	66	0	0
0	0	Thoracotoı	41	PRIVE	No	69	0	0

0	0	Thoracotor	41	PRIVE	No	57	0	0
0	0	Thoracotor	41	PRIVE	No	63	0	0
0	0	Thoracotor	41	PRIVE	No	64	0	0
0	0	Thoracotor	41	PRIVE	No	72	0	0
0	0	Thoracotor	41	PRIVE	No	72	0	0
0	0	Thoracotor	41	PRIVE	No	61	1	0
0	0	Thoracotor	41	PRIVE	No	56	0	0
0	0	Thoracotor	41	PRIVE	No	65	0	0
0	0	Thoracotor	41	PRIVE	No	70	0	0
0	0	Thoracotor	41	PRIVE	No	55	0	0
0	0	Thoracotor	41	PRIVE	No	71	0	0
0	0	Thoracotor	41	PRIVE	No	55	0	0
0	0	Thoracotor	41	PRIVE	No	71	0	0
0	0	Thoracotor	41	PRIVE	No	54	0	0
0	0	VATS or rc	41	PRIVE	No	80	0	0
0	0	Thoracotor	41	PRIVE	No	61	0	0
0	1	Thoracotor	41	PRIVE	No	49	0	0
0	0	Thoracotor	41	PRIVE	No	68	0	0
0	0	Thoracotor	41	PRIVE	No	67	0	0
0	0	Thoracotomy		PRIVE	No	78	1	0
0	0	Thoracotomy		PRIVE	No	64	0	1
0	0	Thoracotor	18	PRIVE	No	50	0	0
0	0	Thoracotor	18	PRIVE	No	75	0	0
0	0	Thoracotor	18	PRIVE	No	73	0	0
0	0	Thoracotor	18	PRIVE	No	52	0	0
0	0	Thoracotor	18	PRIVE	No	76	0	0
0	0	Thoracotor	18	PRIVE	No	72	0	0
0	0	Thoracotor	18	PRIVE	No	56	0	0
0	0	Thoracotor	18	PRIVE	No	75	0	0
0	0	Thoracotor	18	PRIVE	No	62	0	0
0	0	Thoracotor	18	PRIVE	No	74	0	0
0	0	Thoracotor	18	PRIVE	No	71	0	0
0	0	Thoracotor	18	PRIVE	No	73	0	0
0	0	Thoracotor	18	PRIVE	No	59	0	0
0	0	Thoracotor	18	PRIVE	No	71	0	0
0	0	Thoracotor	18	PRIVE	No	51	0	0
0	0	Thoracotor	18	PRIVE	No	67	0	0
0	0	Thoracotor	18	PRIVE	No	59	0	1
0	0	Thoracotor	18	PRIVE	No	60	0	0
0	0	Thoracotor	18	PRIVE	No	75	0	0
0	0	Thoracotor	18	PRIVE	No	69	0	0
0	0	Thoracotor	18	PRIVE	No	65	0	0
1	0	Thoracotor	265	CHU	No	58	0	0
1	0	Thoracotor	265	CHU	No	59	0	0
1	0	Thoracotor	265	CHU	No	81	0	0
1	0	VATS or rc	265	CHU	No	68	0	0
1	0	Thoracotor	265	CHU	No	57	0	1
1	0	Thoracotor	265	CHU	No	56	0	0
1	0	Thoracotor	265	CHU	No	61	0	1
1	0	VATS or rc	265	CHU	No	67	0	1
1	1	VATS or rc	265	CHU	No	56	0	1
1	0	Thoracotor	265	CHU	No	52	0	1
1	0	VATS or rc	265	CHU	No	61	0	1
1	0	Thoracotor	265	CHU	No	64	0	0
1	1	Thoracotor	265	CHU	No	68	0	1
1	0	Thoracotor	265	CHU	No	60	0	0

1	0	VATS or rc	265	CHU	No	69	0	1
1	0	Thoracotoı	265	CHU	No	84	0	1
1	0	Thoracotoı	265	CHU	No	58	0	0
1	0	Thoracotoı	265	CHU	No	60	0	1
1	0	VATS or rc	265	CHU	No	82	0	1
1	0	Thoracotoı	265	CHU	No	47	0	0
1	0	Thoracotoı	265	CHU	Yes	68	0	0
1	0	Thoracotoı	265	CHU	No	56	0	1
1	0	Thoracotoı	265	CHU	No	61	0	0
1	0	Thoracotoı	265	CHU	No	64	0	1
1	0	Thoracotoı	265	CHU	No	58	1	1
1	0	VATS or rc	265	CHU	No	57	0	0
1	0	VATS or rc	265	CHU	No	65	0	0
1	1	Thoracotoı	265	CHU	No	68	0	0
1	0	Thoracotoı	265	CHU	No	49	0	1
1	1	Thoracotoı	265	CHU	Yes	67	0	1
1	0	VATS or rc	265	CHU	No	35	0	0
1	0	Thoracotoı	265	CHU	No	79	0	1
1	0	Thoracotoı	265	CHU	No	64	0	1
1	0	Thoracotoı	265	CHU	No	67	0	0
1	0	Thoracotoı	265	CHU	No	67	1	1
1	0	VATS or rc	265	CHU	No	60	0	0
1	0	Thoracotoı	265	CHU	No	40	0	0
1	1	Thoracotoı	265	CHU	Yes	60	0	0
1	0	VATS or rc	265	CHU	No	66	0	0
1	0	VATS or rc	265	CHU	No	66	0	0
1	0	Thoracotoı	265	CHU	No	58	0	1
1	0	Thoracotoı	265	CHU	No	64	0	1
1	0	Thoracotoı	265	CHU	No	73	0	0
1	0	Thoracotoı	265	CHU	No	54	0	1
1	0	Thoracotoı	265	CHU	No	68	0	0
1	0	Thoracotoı	265	CHU	No	59	0	0
1	0	VATS or rc	265	CHU	No	72	0	0
1	0	Thoracotoı	265	CHU	No	57	0	0
1	0	VATS or rc	265	CHU	No	72	0	0
1	0	VATS or rc	265	CHU	No	54	0	0
1	0	Thoracotoı	265	CHU	No	73	0	0
1	0	Thoracotoı	265	CHU	No	67	0	0
1	0	Thoracotoı	265	CHU	No	55	0	0
1	0	Thoracotoı	265	CHU	No	78	0	1
1	0	Thoracotoı	265	CHU	No	43	0	0
1	0	Thoracotoı	265	CHU	No	78	0	0
1	0	VATS or rc	265	CHU	No	48	0	0
1	0	VATS or rc	265	CHU	No	55	0	1
1	0	VATS or rc	265	CHU	No	43	0	0
1	0	Thoracotoı	265	CHU	No	67	0	0
1	0	Thoracotoı	265	CHU	No	74	0	0
1	0	Thoracotoı	265	CHU	No	68	0	0
1	0	Thoracotoı	265	CHU	No	85	0	0
1	0	Thoracotoı	265	CHU	No	82	1	1
1	1	Thoracotoı	265	CHU	No	70	0	0
1	0	Thoracotoı	265	CHU	No	64	0	0
1	0	Thoracotoı	265	CHU	No	55	0	0
1	0	VATS or rc	265	CHU	No	65	1	1
1	0	Thoracotoı	265	CHU	No	43	0	1
1	0	VATS or rc	265	CHU	No	63	1	1

1	0	Thoracotoı	265	CHU	No	70	0	1
1	0	Thoracotoı	265	CHU	No	62	0	0
1	0	VATS or rc	265	CHU	No	66	0	0
1	0	Thoracotoı	265	CHU	No	63	0	0
1	1	Thoracotoı	265	CHU	No	59	0	0
1	0	VATS or rc	265	CHU	No	64	0	1
1	0	VATS or rc	265	CHU	No	57	0	1
1	0	VATS or rc	265	CHU	No	61	0	0
1	0	Thoracotoı	265	CHU	No	75	0	0
1	0	VATS or rc	265	CHU	No	65	0	0
1	0	VATS or rc	265	CHU	No	31	0	1
1	0	VATS or rc	265	CHU	No	65	1	1
1	0	Thoracotoı	265	CHU	No	16	0	0
1	0	VATS or rc	265	CHU	No	87	0	0
1	0	VATS or rc	265	CHU	No	42	1	1
1	0	Thoracotoı	265	CHU	No	61	0	0
1	0	VATS or rc	265	CHU	No	42	0	1
1	0	Thoracotoı	265	CHU	No	68	0	1
1	0	Thoracotoı	265	CHU	No	55	0	1
1	0	Thoracotoı	265	CHU	No	80	1	0
1	0	Thoracotoı	265	CHU	No	67	0	0
1	0	VATS or rc	265	CHU	No	79	1	0
1	0	Thoracotoı	265	CHU	No	59	1	0
1	0	VATS or rc	265	CHU	No	73	0	1
1	1	Thoracotoı	265	CHU	No	56	0	0
1	0	Thoracotoı	265	CHU	No	69	1	0
1	0	Thoracotoı	265	CHU	No	65	0	1
1	0	Thoracotoı	265	CHU	No	58	1	1
1	0	Thoracotoı	265	CHU	No	57	0	0
1	0	Thoracotoı	265	CHU	No	57	1	1
1	1	Thoracotoı	265	CHU	No	72	0	0
1	1	Thoracotoı	265	CHU	No	57	0	0
1	0	Thoracotoı	265	CHU	No	44	0	1
1	0	VATS or rc	265	CHU	No	81	0	0
1	0	Thoracotoı	265	CHU	No	25	0	0
1	0	Thoracotoı	265	CHU	No	51	0	1
1	1	Thoracotoı	265	CHU	No	60	0	0
1	0	Thoracotoı	265	CHU	No	69	0	0
1	0	Thoracotoı	265	CHU	No	42	0	1
1	0	VATS or rc	265	CHU	No	73	0	0
1	0	Thoracotoı	265	CHU	No	46	0	1
1	0	VATS or rc	265	CHU	No	74	1	0
1	0	Thoracotoı	265	CHU	No	47	0	1
1	0	Thoracotoı	265	CHU	No	55	1	0
1	0	Thoracotoı	265	CHU	No	60	0	1
1	1	Thoracotoı	265	CHU	No	76	0	0
1	0	VATS or rc	265	CHU	No	57	0	0
1	0	Thoracotoı	265	CHU	No	79	0	0
1	0	Thoracotoı	265	CHU	No	74	0	0
1	0	Thoracotoı	265	CHU	No	65	0	1
1	0	VATS or rc	265	CHU	No	69	0	0
1	0	Thoracotoı	265	CHU	No	74	0	0
1	0	VATS or rc	265	CHU	No	74	0	0
1	0	Thoracotoı	265	CHU	No	52	0	0
1	1	Thoracotoı	265	CHU	Yes	57	0	0
1	1	Thoracotoı	265	CHU	No	73	0	0

1	0	Thoracotoı	265	CHU	No	73	0	0
1	0	Thoracotoı	265	CHU	No	73	0	0
1	0	VATS or rc	265	CHU	No	85	0	0
1	0	Thoracotoı	265	CHU	No	52	0	1
1	0	Thoracotoı	265	CHU	No	82	0	1
1	0	Thoracotoı	265	CHU	No	58	0	0
1	0	Thoracotoı	265	CHU	No	72	0	0
1	0	Thoracotoı	265	CHU	No	68	0	0
1	0	Thoracotoı	265	CHU	No	52	0	0
1	0	Thoracotoı	265	CHU	No	57	1	0
1	0	Thoracotoı	265	CHU	No	45	0	0
1	0	VATS or rc	265	CHU	No	52	0	0
1	0	Thoracotoı	265	CHU	No	70	0	0
1	0	VATS or rc	265	CHU	No	62	0	1
1	0	Thoracotoı	265	CHU	No	58	0	1
1	0	Thoracotoı	265	CHU	No	60	0	1
1	0	Thoracotoı	265	CHU	No	76	0	0
1	0	Thoracotoı	265	CHU	No	51	0	0
1	0	Thoracotoı	265	CHU	No	83	1	0
1	0	VATS or rc	265	CHU	No	68	0	0
1	0	Thoracotoı	265	CHU	No	51	0	0
1	1	Thoracotoı	265	CHU	No	66	0	1
1	0	Thoracotoı	265	CHU	No	73	0	0
1	0	Thoracotoı	265	CHU	No	40	0	1
1	0	Thoracotoı	265	CHU	No	75	0	0
1	0	VATS or rc	265	CHU	No	71	0	1
1	0	Thoracotoı	265	CHU	No	74	0	1
1	0	Thoracotoı	265	CHU	No	74	1	0
1	0	VATS or rc	265	CHU	No	66	0	0
1	0	Thoracotoı	265	CHU	No	68	1	1
1	0	Thoracotoı	265	CHU	No	54	0	1
1	0	Thoracotoı	265	CHU	No	59	0	1
1	0	Thoracotoı	265	CHU	No	68	1	1
1	0	VATS or rc	265	CHU	No	29	0	1
1	0	Thoracotoı	265	CHU	No	74	0	0
1	0	Thoracotoı	265	CHU	No	49	0	1
1	0	Thoracotoı	265	CHU	No	76	0	0
1	0	VATS or rc	265	CHU	No	66	0	0
1	0	Thoracotoı	265	CHU	No	74	1	0
1	0	Thoracotoı	265	CHU	No	80	1	0
1	0	Thoracotoı	265	CHU	No	70	0	0
1	0	Thoracotoı	265	CHU	No	69	0	0
1	0	Thoracotoı	265	CHU	No	58	0	0
1	0	Thoracotoı	265	CHU	No	55	0	0
1	0	Thoracotoı	265	CHU	No	65	0	0
1	0	VATS or rc	265	CHU	No	56	0	1
1	0	VATS or rc	265	CHU	No	66	0	0
1	0	Thoracotoı	265	CHU	No	54	0	1
1	0	VATS or rc	265	CHU	No	80	0	0
1	0	Thoracotoı	265	CHU	No	79	0	0
1	0	Thoracotoı	265	CHU	No	69	0	0
1	0	Thoracotoı	265	CHU	No	59	0	0
1	0	Thoracotoı	265	CHU	No	71	0	0
1	1	Thoracotoı	265	CHU	No	61	0	0
1	0	Thoracotoı	265	CHU	No	72	0	1
1	0	VATS or rc	265	CHU	No	55	0	0

1	0	Thoracotoı	265	CHU	No	77	0	0
1	0	Thoracotoı	265	CHU	No	62	0	0
1	0	VATS or rc	265	CHU	No	69	1	0
1	0	VATS or rc	265	CHU	No	75	0	1
1	0	Thoracotoı	265	CHU	No	55	0	0
1	0	Thoracotoı	265	CHU	No	56	0	1
1	0	Thoracotoı	265	CHU	No	65	0	0
1	0	Thoracotoı	265	CHU	Yes	77	1	0
1	0	Thoracotoı	265	CHU	No	47	0	1
1	0	VATS or rc	265	CHU	No	66	0	0
1	0	Thoracotoı	265	CHU	No	50	0	1
1	0	Thoracotoı	265	CHU	No	81	0	0
1	0	Thoracotoı	265	CHU	No	52	0	1
1	0	VATS or rc	265	CHU	No	51	0	1
1	0	VATS or rc	265	CHU	No	58	0	1
1	0	VATS or rc	265	CHU	No	62	0	1
1	0	Thoracotoı	265	CHU	No	54	0	0
1	0	VATS or rc	265	CHU	No	78	0	0
1	0	VATS or rc	265	CHU	No	72	0	0
1	0	Thoracotoı	265	CHU	No	59	0	0
1	0	Thoracotoı	265	CHU	No	61	0	0
1	0	VATS or rc	265	CHU	No	56	0	1
1	0	VATS or rc	265	CHU	No	61	0	0
1	0	VATS or rc	265	CHU	No	66	0	0
1	0	Thoracotoı	265	CHU	No	60	0	0
1	0	VATS or rc	265	CHU	No	62	1	0
1	0	VATS or rc	265	CHU	No	56	0	1
1	1	Thoracotoı	265	CHU	No	65	1	0
1	0	Thoracotoı	265	CHU	No	59	0	0
1	0	VATS or rc	265	CHU	No	63	0	1
1	0	VATS or rc	265	CHU	No	73	0	0
1	0	VATS or rc	265	CHU	No	72	0	0
1	0	VATS or rc	265	CHU	No	31	0	0
1	0	Thoracotoı	265	CHU	No	62	1	0
1	0	VATS or rc	265	CHU	No	53	1	1
1	0	VATS or rc	265	CHU	No	62	0	0
1	0	Thoracotoı	265	CHU	No	71	0	1
1	0	Thoracotoı	265	CHU	No	55	0	1
1	0	Thoracotoı	265	CHU	No	50	0	1
1	0	VATS or rc	265	CHU	No	63	0	0
1	1	Thoracotoı	265	CHU	No	65	0	0
1	0	Thoracotoı	265	CHU	No	77	0	0
1	0	Thoracotoı	265	CHU	No	59	0	1
1	0	Thoracotoı	265	CHU	No	57	0	0
1	0	Thoracotoı	265	CHU	No	76	0	0
1	0	Thoracotoı	265	CHU	No	76	0	0
1	0	Thoracotoı	265	CHU	No	79	0	0
1	0	Thoracotoı	265	CHU	No	70	0	0
1	0	VATS or rc	265	CHU	No	60	0	0
1	0	VATS or rc	265	CHU	No	74	0	0
1	0	Thoracotoı	265	CHU	No	51	0	0
1	0	Thoracotoı	265	CHU	No	60	0	1
1	1	Thoracotoı	265	CHU	No	39	0	0
1	0	VATS or rc	265	CHU	No	68	0	1
1	0	VATS or rc	265	CHU	No	58	0	0
1	0	Thoracotoı	265	CHU	No	70	0	1

1	0	Thoracotoı	265	CHU	No	76	0	1
1	0	Thoracotoı	265	CHU	Yes	62	0	0
1	1	Thoracotoı	265	CHU	No	67	1	0
1	0	VATS or rc	265	CHU	No	68	0	0
1	0	VATS or rc	265	CHU	No	71	0	1
1	0	VATS or rc	265	CHU	No	73	0	1
1	0	Thoracotoı	265	CHU	No	50	0	1
1	0	Thoracotoı	265	CHU	No	68	0	1
1	0	Thoracotoı	265	CHU	No	67	0	0
1	0	Thoracotoı	265	CHU	No	74	0	1
1	0	Thoracotoı	265	CHU	No	52	0	0
1	0	VATS or rc	265	CHU	No	55	0	0
1	0	Thoracotoı	265	CHU	No	67	0	1
1	0	Thoracotoı	265	CHU	No	53	0	1
1	1	Thoracotoı	265	CHU	No	55	0	0
1	0	Thoracotoı	265	CHU	No	53	0	1
1	0	VATS or rc	265	CHU	No	75	0	0
1	0	Thoracotoı	265	CHU	No	74	0	0
1	0	VATS or rc	265	CHU	No	76	0	0
1	0	Thoracotoı	265	CHU	No	59	0	1
1	0	VATS or rc	265	CHU	No	52	0	1
1	0	Thoracotoı	265	CHU	No	76	0	0
1	0	Thoracotoı	265	CHU	No	42	0	0
1	1	Thoracotoı	265	CHU	No	61	0	0
1	1	Thoracotoı	265	CHU	No	57	0	1
1	0	Thoracotoı	265	CHU	No	56	0	0
1	0	VATS or rc	265	CHU	No	76	0	1
1	0	VATS or rc	265	CHU	No	56	0	1
1	0	Thoracotoı	265	CHU	No	69	0	0
1	0	Thoracotoı	265	CHU	No	68	0	0
1	0	VATS or rc	265	CHU	No	73	0	0
1	0	Thoracotoı	265	CHU	No	20	0	0
1	0	Thoracotoı	265	CHU	Yes	82	0	0
1	0	Thoracotoı	265	CHU	No	60	0	0
1	0	VATS or rc	265	CHU	No	47	0	0
1	0	Thoracotoı	265	CHU	No	75	0	0
1	0	Thoracotoı	265	CHU	No	75	0	1
1	0	Thoracotoı	265	CHU	No	68	0	0
1	0	VATS or rc	265	CHU	No	63	0	0
1	0	VATS or rc	265	CHU	No	59	0	0
1	0	Thoracotoı	265	CHU	No	70	0	0
1	0	Thoracotoı	265	CHU	No	65	0	0
1	1	Thoracotoı	265	CHU	No	57	0	1
1	0	VATS or rc	265	CHU	No	70	0	0
1	0	VATS or rc	265	CHU	No	48	1	1
1	0	Thoracotoı	265	CHU	No	65	0	1
1	0	VATS or rc	265	CHU	No	77	0	0
1	0	VATS or rc	265	CHU	No	62	0	1
1	0	VATS or rc	265	CHU	No	82	0	0
1	0	Thoracotoı	265	CHU	No	63	0	0
1	0	Thoracotoı	265	CHU	No	60	0	1
1	0	Thoracotoı	265	CHU	No	74	0	0
1	0	VATS or rc	265	CHU	No	70	0	0
1	0	Thoracotoı	265	CHU	No	57	1	1
1	0	Thoracotoı	265	CHU	No	76	0	0
1	0	Thoracotoı	265	CHU	No	53	0	0

1	0	Thoracotoı	265	CHU	No	48	0	1
1	0	Thoracotoı	265	CHU	No	62	0	0
1	1	Thoracotoı	265	CHU	No	56	0	0
1	0	Thoracotoı	265	CHU	No	71	0	0
1	0	Thoracotoı	265	CHU	No	60	0	0
1	0	Thoracotoı	265	CHU	No	63	0	0
1	0	VATS or rc	265	CHU	No	53	0	0
1	1	Thoracotoı	265	CHU	No	60	0	0
1	1	Thoracotoı	265	CHU	No	55	0	1
1	0	Thoracotoı	265	CHU	No	83	0	0
1	0	Thoracotoı	265	CHU	No	65	1	0
1	0	Thoracotoı	265	CHU	No	62	0	0
1	0	Thoracotoı	265	CHU	No	56	0	0
1	0	Thoracotoı	265	CHU	No	46	0	0
1	0	Thoracotoı	265	CHU	No	73	0	0
1	0	Thoracotoı	265	CHU	No	49	0	0
1	0	VATS or rc	265	CHU	No	63	0	1
1	0	VATS or rc	265	CHU	No	76	0	0
1	0	Thoracotoı	265	CHU	No	73	0	1
1	0	Thoracotoı	265	CHU	No	60	0	0
1	0	VATS or rc	265	CHU	No	82	1	0
1	0	Thoracotoı	265	CHU	No	85	0	0
1	0	VATS or rc	265	CHU	No	65	0	0
1	0	Thoracotoı	265	CHU	No	75	0	0
1	0	Thoracotoı	265	CHU	No	71	1	0
1	0	VATS or rc	265	CHU	No	59	0	1
1	0	Thoracotoı	265	CHU	No	56	0	1
1	0	Thoracotoı	265	CHU	No	59	0	1
1	0	Thoracotoı	265	CHU	No	66	0	0
1	0	Thoracotoı	265	CHU	No	62	0	1
1	0	Thoracotoı	265	CHU	No	53	0	1
1	0	Thoracotoı	265	CHU	No	54	0	1
1	0	Thoracotoı	265	CHU	No	62	0	0
1	0	VATS or rc	265	CHU	No	80	0	1
1	0	Thoracotoı	265	CHU	No	74	0	0
1	1	Thoracotoı	265	CHU	No	68	0	1
1	0	Thoracotoı	265	CHU	No	51	0	1
1	0	Thoracotoı	265	CHU	No	63	0	0
1	0	VATS or rc	265	CHU	No	61	0	1
1	0	Thoracotoı	265	CHU	No	64	0	0
1	0	Thoracotoı	265	CHU	No	61	0	1
1	0	Thoracotoı	265	CHU	No	77	0	0
1	0	Thoracotoı	265	CHU	No	49	0	1
1	0	Thoracotoı	265	CHU	No	69	0	1
1	0	Thoracotoı	265	CHU	No	68	0	0
1	0	Thoracotoı	265	CHU	No	67	0	1
1	0	VATS or rc	265	CHU	No	68	0	0
1	0	Thoracotoı	265	CHU	No	52	1	1
1	0	Thoracotoı	265	CHU	No	67	0	0
1	0	Thoracotoı	265	CHU	No	43	0	0
1	0	VATS or rc	265	CHU	No	67	0	1
1	0	Thoracotoı	265	CHU	No	55	0	0
1	0	Thoracotoı	265	CHU	No	65	0	1
1	0	VATS or rc	265	CHU	No	78	0	0
1	0	VATS or rc	265	CHU	No	59	0	0
1	0	Thoracotoı	265	CHU	No	69	0	0

1	0	Thoracotoı	265	CHU	No	51	0	0
1	0	VATS or rc	265	CHU	No	59	0	0
1	1	Thoracotoı	265	CHU	No	68	0	0
1	0	Thoracotoı	265	CHU	No	45	0	0
1	0	Thoracotoı	265	CHU	No	66	0	0
1	1	Thoracotoı	265	CHU	No	60	0	0
1	0	Thoracotoı	265	CHU	No	68	0	0
1	0	Thoracotoı	265	CHU	Yes	59	0	0
1	0	Thoracotoı	265	CHU	No	48	0	0
1	0	Thoracotoı	265	CHU	No	59	0	0
1	0	Thoracotoı	265	CHU	No	80	0	0
1	0	Thoracotoı	265	CHU	No	64	0	0
1	0	VATS or rc	265	CHU	No	74	0	0
1	0	Thoracotoı	265	CHU	No	76	0	0
1	0	VATS or rc	265	CHU	No	56	0	0
1	0	Thoracotoı	265	CHU	No	78	0	0
1	0	VATS or rc	265	CHU	No	71	0	1
1	0	Thoracotoı	265	CHU	No	75	0	1
1	0	Thoracotoı	265	CHU	No	48	0	1
1	0	VATS or rc	265	CHU	No	61	0	0
1	0	VATS or rc	265	CHU	No	57	0	1
1	0	Thoracotoı	265	CHU	No	81	0	0
1	0	VATS or rc	265	CHU	No	52	0	0
1	0	Thoracotoı	265	CHU	No	67	1	0
1	0	Thoracotoı	265	CHU	No	79	0	1
1	0	Thoracotoı	265	CHU	No	67	0	0
1	0	Thoracotoı	265	CHU	No	61	0	0
1	0	Thoracotoı	265	CHU	No	68	1	0
1	0	VATS or rc	265	CHU	No	70	0	1
1	0	Thoracotoı	265	CHU	No	71	0	0
1	0	Thoracotoı	265	CHU	No	56	0	0
1	0	Thoracotoı	265	CHU	No	67	0	1
1	0	Thoracotoı	265	CHU	No	68	0	0
1	0	VATS or rc	265	CHU	No	59	0	1
1	0	VATS or rc	265	CHU	No	70	0	0
1	0	Thoracotoı	265	CHU	No	68	0	1
1	0	Thoracotoı	265	CHU	No	56	0	0
1	1	Thoracotoı	265	CHU	No	81	0	0
1	0	Thoracotoı	265	CHU	No	65	0	0
1	0	Thoracotoı	265	CHU	No	19	0	0
1	0	Thoracotoı	265	CHU	No	59	0	0
1	0	Thoracotoı	265	CHU	No	80	1	0
1	0	Thoracotoı	265	CHU	No	59	0	0
1	0	Thoracotoı	265	CHU	No	70	0	0
1	0	Thoracotoı	265	CHU	No	59	0	1
1	0	VATS or rc	265	CHU	No	83	0	1
1	0	Thoracotoı	265	CHU	No	36	0	0
1	0	Thoracotoı	265	CHU	No	70	1	0
1	0	VATS or rc	265	CHU	No	67	0	1
1	0	Thoracotoı	265	CHU	No	54	0	0
1	0	Thoracotoı	265	CHU	No	81	0	0
1	1	Thoracotoı	265	CHU	No	64	0	1
1	0	Thoracotoı	265	CHU	No	52	0	0
1	0	Thoracotoı	265	CHU	No	65	0	0
1	0	Thoracotoı	265	CHU	No	73	0	0
1	0	VATS or rc	265	CHU	No	62	0	0

1	0	Thoracotoı	265	CHU	No	59	0	1
1	0	Thoracotoı	265	CHU	No	50	0	1
1	0	VATS or rc	265	CHU	No	56	0	0
1	0	Thoracotoı	265	CHU	No	58	0	0
1	0	Thoracotoı	265	CHU	No	61	0	0
1	0	Thoracotoı	265	CHU	No	72	0	1
1	0	Thoracotoı	265	CHU	No	61	0	0
1	0	Thoracotoı	265	CHU	No	77	0	0
1	1	Thoracotoı	265	CHU	No	40	0	0
1	0	Thoracotoı	265	CHU	No	67	0	0
1	0	VATS or rc	265	CHU	No	63	0	0
1	0	Thoracotoı	265	CHU	No	65	0	1
1	0	Thoracotoı	265	CHU	No	57	0	0
1	0	VATS or rc	265	CHU	No	71	0	0
1	0	Thoracotoı	265	CHU	No	68	1	0
1	0	Thoracotoı	265	CHU	No	66	0	0
1	0	Thoracotoı	265	CHU	No	51	0	0
1	0	Thoracotoı	265	CHU	No	54	0	0
1	0	VATS or rc	265	CHU	No	63	0	1
1	1	Thoracotoı	265	CHU	No	64	0	0
1	0	Thoracotoı	265	CHU	No	50	0	1
1	0	Thoracotoı	265	CHU	No	66	0	0
1	0	VATS or rc	265	CHU	No	59	0	1
1	0	VATS or rc	265	CHU	No	57	0	1
1	0	Thoracotoı	265	CHU	No	54	0	0
1	0	Thoracotoı	265	CHU	No	83	0	0
1	0	Thoracotoı	265	CHU	No	68	1	0
1	0	VATS or rc	265	CHU	No	74	0	0
1	1	Thoracotoı	265	CHU	No	37	0	1
1	0	Thoracotoı	265	CHU	No	58	0	0
1	0	VATS or rc	265	CHU	No	40	0	0
1	0	Thoracotoı	265	CHU	No	70	0	0
1	0	Thoracotoı	265	CHU	No	69	0	1
1	0	Thoracotoı	265	CHU	No	51	0	1
1	1	Thoracotoı	265	CHU	No	59	0	0
1	0	Thoracotoı	265	CHU	No	71	0	0
1	0	VATS or rc	265	CHU	No	78	0	0
1	0	Thoracotoı	265	CHU	No	66	0	0
1	0	VATS or rc	265	CHU	No	65	0	0
1	0	Thoracotoı	265	CHU	No	76	1	0
1	0	Thoracotoı	265	CHU	No	58	0	0
1	0	Thoracotoı	265	CHU	No	67	0	0
1	0	Thoracotoı	265	CHU	No	76	0	0
1	0	Thoracotoı	265	CHU	No	70	0	0
1	0	Thoracotoı	265	CHU	No	52	1	0
1	0	Thoracotoı	265	CHU	No	62	0	0
1	0	VATS or rc	265	CHU	No	62	0	1
1	0	Thoracotoı	265	CHU	No	55	0	1
1	0	VATS or rc	265	CHU	No	62	0	0
1	0	Thoracotoı	265	CHU	No	58	0	0
1	0	Thoracotoı	265	CHU	No	51	0	1
1	0	Thoracotoı	265	CHU	No	63	0	0
1	0	Thoracotoı	265	CHU	No	56	0	1
1	0	VATS or rc	265	CHU	No	57	0	1
1	0	Thoracotoı	265	CHU	No	49	0	0
1	0	VATS or rc	265	CHU	No	52	0	0

1	0	VATS or ro	265	CHU	No	68	1	0
1	0	Thoracotom	265	CHU	No	68	0	0
1	0	Thoracotom	265	CHU	No	60	1	0
1	0	VATS or ro	265	CHU	No	59	0	1
1	0	VATS or ro	265	CHU	No	47	0	0
1	0	Thoracotom	265	CHU	No	62	0	0
1	0	Thoracotom	265	CHU	No	61	0	0
1	0	VATS or ro	265	CHU	No	78	0	0
1	0	Thoracotom	265	CHU	No	76	0	0
1	0	Thoracotom	265	CHU	No	62	0	0
1	0	VATS or ro	265	CHU	No	78	0	0
1	1	Thoracotom	265	CHU	Yes	75	0	0
1	0	VATS or ro	265	CHU	No	54	0	0
1	0	Thoracotom	265	CHU	No	67	0	1
1	0	Thoracotom	265	CHU	No	72	1	0
1	0	Thoracotom	265	CHU	No	47	0	0
1	0	Thoracotom	265	CHU	No	49	0	0
1	0	Thoracotom	265	CHU	No	55	0	0
1	0	VATS or ro	265	CHU	No	52	0	0
1	0	Thoracotom	265	CHU	No	51	0	0
1	0	Thoracotom	265	CHU	No	60	0	1
1	0	Thoracotom	265	CHU	No	68	0	1
1	0	Thoracotom	265	CHU	No	40	1	0
1	0	Thoracotom	265	CHU	No	60	0	0
1	0	Thoracotom	265	CHU	No	65	0	0
1	0	Thoracotom	265	CHU	No	65	0	0
1	0	Thoracotom	265	CHU	No	60	0	0
1	0	VATS or ro	265	CHU	No	52	0	0
1	0	Thoracotom	265	CHU	No	55	0	0
1	0	VATS or ro	265	CHU	No	60	1	0
1	0	Thoracotom	265	CHU	No	74	1	0
1	0	Thoracotom	265	CHU	No	61	0	0
1	0	Thoracotom	265	CHU	No	75	1	0
1	0	Thoracotom	265	CHU	No	74	0	0
1	1	Thoracotom	265	CHU	Yes	46	0	0
1	0	Thoracotom	265	CHU	No	86	0	1
1	0	Thoracotom	265	CHU	No	71	0	1
1	0	Thoracotom	265	CHU	No	57	0	0
1	0	VATS or ro	265	CHU	No	78	0	0
1	0	Thoracotom	265	CHU	No	67	0	0
1	0	Thoracotom	265	CHU	No	83	0	0
1	0	Thoracotom	265	CHU	No	65	1	0
1	0	VATS or ro	265	CHU	No	81	0	0
1	0	Thoracotom	265	CHU	No	32	0	0
1	0	Thoracotom	265	CHU	No	79	0	1
1	0	Thoracotom	265	CHU	No	56	0	0
1	0	Thoracotom	265	CHU	No	69	0	1
1	0	Thoracotom	265	CHU	No	77	0	0
1	0	Thoracotom	265	CHU	No	74	0	0
1	1	Thoracotom	265	CHU	No	80	1	0
1	0	VATS or ro	265	CHU	No	69	0	0
1	0	Thoracotom	265	CHU	No	70	0	0
1	0	Thoracotom	265	CHU	No	40	0	0
1	0	Thoracotom	265	CHU	No	49	0	1
1	0	Thoracotom	265	CHU	No	62	0	0
1	0	Thoracotom	265	CHU	No	60	0	1

1	0	VATS or rc	265	CHU	No	65	0	0
1	0	VATS or rc	265	CHU	No	35	0	0
1	0	Thoracotoı	265	CHU	No	61	0	0
1	0	Thoracotoı	265	CHU	No	74	0	0
1	0	Thoracotoı	265	CHU	No	73	1	0
1	0	Thoracotoı	265	CHU	No	68	0	0
1	0	Thoracotoı	265	CHU	No	79	0	0
1	0	Thoracotoı	265	CHU	No	58	0	0
1	0	Thoracotoı	265	CHU	No	78	0	0
1	1	Thoracotoı	265	CHU	No	65	1	0
1	0	Thoracotoı	265	CHU	No	58	0	0
1	0	VATS or rc	265	CHU	No	80	0	0
1	0	Thoracotoı	265	CHU	No	70	0	0
1	0	Thoracotoı	265	CHU	No	50	0	0
1	0	VATS or rc	265	CHU	No	51	0	1
1	0	VATS or rc	265	CHU	No	68	0	1
1	0	Thoracotoı	265	CHU	No	80	1	0
1	0	Thoracotoı	265	CHU	No	72	0	0
1	1	Thoracotoı	265	CHU	No	60	0	0
1	1	Thoracotoı	265	CHU	No	58	0	1
1	1	Thoracotoı	265	CHU	No	76	0	0
1	0	Thoracotoı	265	CHU	No	72	0	0
1	0	Thoracotoı	265	CHU	No	73	0	0
1	0	Thoracotoı	265	CHU	No	54	0	1
1	0	VATS or rc	265	CHU	No	44	0	0
1	0	Thoracotoı	265	CHU	No	62	0	0
1	0	Thoracotoı	265	CHU	No	76	1	1
1	0	Thoracotoı	265	CHU	No	78	0	0
1	0	Thoracotoı	265	CHU	No	44	0	1
1	0	VATS or rc	265	CHU	No	63	0	0
1	0	Thoracotoı	265	CHU	No	67	0	0
1	0	VATS or rc	265	CHU	No	61	0	1
1	0	Thoracotoı	265	CHU	No	51	0	0
1	0	VATS or rc	265	CHU	No	56	1	0
1	0	VATS or rc	265	CHU	No	54	0	0
1	0	Thoracotoı	265	CHU	No	76	0	0
1	0	Thoracotoı	265	CHU	No	49	0	1
1	0	Thoracotoı	265	CHU	No	61	0	0
1	0	Thoracotoı	265	CHU	No	61	0	0
1	0	VATS or rc	265	CHU	No	74	0	0
1	0	Thoracotoı	265	CHU	No	65	0	1
1	0	VATS or rc	265	CHU	No	60	0	1
1	0	Thoracotoı	265	CHU	No	62	0	0
1	0	Thoracotoı	265	CHU	No	76	0	1
1	0	Thoracotoı	265	CHU	No	48	1	1
1	0	Thoracotoı	265	CHU	No	60	0	1
1	0	Thoracotoı	265	CHU	No	62	0	0
1	0	Thoracotoı	265	CHU	No	78	0	1
1	0	Thoracotoı	265	CHU	No	75	0	0
1	0	Thoracotoı	265	CHU	Yes	61	0	0
1	0	Thoracotoı	265	CHU	No	57	0	0
1	0	Thoracotoı	265	CHU	No	72	0	0
1	0	Thoracotoı	265	CHU	No	56	0	0
1	0	Thoracotoı	265	CHU	No	74	0	0
1	0	Thoracotoı	265	CHU	No	74	0	0
1	0	Thoracotoı	265	CHU	No	71	1	0

1	0	Thoracotoı	265	CHU	No	67	0	0
1	0	Thoracotoı	265	CHU	No	72	0	0
1	0	VATS or rc	265	CHU	No	70	0	1
1	0	VATS or rc	265	CHU	No	61	0	1
1	0	VATS or rc	265	CHU	No	55	0	0
1	0	VATS or rc	265	CHU	No	62	0	1
1	0	Thoracotoı	265	CHU	No	57	0	0
1	0	Thoracotoı	265	CHU	No	46	0	0
1	0	Thoracotoı	265	CHU	No	53	0	0
1	0	Thoracotoı	265	CHU	No	60	0	0
1	0	VATS or rc	265	CHU	No	57	0	1
1	0	Thoracotoı	265	CHU	No	69	0	0
1	0	VATS or rc	265	CHU	No	64	1	0
1	0	Thoracotoı	265	CHU	No	71	1	0
1	0	Thoracotoı	265	CHU	No	21	0	1
1	0	VATS or rc	265	CHU	No	57	0	1
1	0	VATS or rc	265	CHU	No	54	0	1
1	0	Thoracotoı	265	CHU	No	70	0	1
1	0	VATS or rc	265	CHU	No	60	0	0
1	0	Thoracotoı	265	CHU	No	57	1	0
1	0	Thoracotoı	265	CHU	No	58	0	0
1	0	Thoracotoı	265	CHU	No	71	0	0
1	0	VATS or rc	265	CHU	No	54	0	0
1	0	Thoracotoı	265	CHU	No	59	0	0
1	0	Thoracotoı	265	CHU	No	52	0	1
1	0	Thoracotoı	265	CHU	No	61	0	0
1	0	Thoracotoı	265	CHU	No	52	0	1
1	0	Thoracotoı	265	CHU	No	60	0	0
1	0	Thoracotoı	265	CHU	No	55	0	1
1	0	Thoracotoı	265	CHU	No	65	1	1
1	0	VATS or rc	265	CHU	No	60	0	1
1	0	Thoracotoı	265	CHU	No	64	0	0
1	0	VATS or rc	265	CHU	No	60	0	1
1	0	Thoracotoı	265	CHU	No	59	0	1
1	0	Thoracotoı	265	CHU	No	65	0	1
1	0	Thoracotoı	265	CHU	No	69	0	0
1	0	VATS or rc	265	CHU	No	56	0	0
1	0	Thoracotoı	265	CHU	No	53	0	0
1	0	Thoracotoı	265	CHU	No	59	0	0
1	1	Thoracotoı	265	CHU	No	47	0	1
1	0	Thoracotoı	265	CHU	No	60	0	1
1	0	Thoracotoı	265	CHU	No	34	0	0
1	1	Thoracotoı	265	CHU	No	72	0	0
1	0	VATS or rc	265	CHU	No	65	0	1
1	0	Thoracotoı	265	CHU	Yes	39	0	0
1	0	Thoracotoı	265	CHU	No	55	0	0
1	0	VATS or rc	265	CHU	No	62	1	1
1	0	Thoracotoı	265	CHU	No	63	0	1
1	0	VATS or rc	265	CHU	No	64	0	0
1	0	Thoracotoı	265	CHU	No	72	0	0
1	0	Thoracotoı	265	CHU	No	62	0	0
1	0	Thoracotoı	265	CHU	No	28	0	0
1	0	Thoracotoı	265	CHU	No	74	0	1
1	0	VATS or rc	265	CHU	No	69	0	0
1	0	Thoracotoı	265	CHU	Yes	80	0	0
1	0	VATS or rc	265	CHU	No	68	0	0

1	0	Thoracotoı	265	CHU	No	64	0	0
1	0	Thoracotoı	265	CHU	Yes	71	0	1
1	0	Thoracotoı	265	CHU	No	67	0	1
1	0	Thoracotoı	265	CHU	No	75	0	0
1	1	Thoracotoı	265	CHU	No	51	0	0
1	0	Thoracotoı	265	CHU	No	50	0	1
1	1	Thoracotoı	265	CHU	No	76	0	0
1	0	Thoracotoı	265	CHU	No	56	0	1
1	1	Thoracotoı	265	CHU	No	53	0	0
1	0	VATS or rc	265	CHU	No	81	0	0
1	0	Thoracotoı	265	CHU	No	54	0	0
1	0	Thoracotoı	265	CHU	No	56	0	1
1	1	Thoracotoı	265	CHU	No	59	0	1
1	0	VATS or rc	265	CHU	No	78	0	0
1	0	VATS or rc	265	CHU	No	31	0	0
1	0	Thoracotoı	265	CHU	No	75	0	0
1	0	Thoracotoı	265	CHU	No	61	0	0
1	0	Thoracotoı	265	CHU	No	69	0	0
1	0	VATS or rc	265	CHU	No	83	0	0
1	0	VATS or rc	265	CHU	No	49	0	0
1	0	Thoracotoı	265	CHU	No	64	0	1
1	0	Thoracotoı	265	CHU	No	68	0	0
1	0	Thoracotoı	265	CHU	No	42	0	1
1	0	Thoracotoı	265	CHU	No	51	0	0
1	0	VATS or rc	265	CHU	No	69	0	0
1	0	Thoracotoı	265	CHU	No	52	0	1
1	0	VATS or rc	265	CHU	No	63	0	0
1	0	Thoracotoı	265	CHU	No	39	0	1
1	0	Thoracotoı	265	CHU	No	73	0	0
1	0	Thoracotoı	265	CHU	No	72	0	0
1	0	Thoracotoı	265	CHU	No	60	0	0
1	0	Thoracotoı	265	CHU	No	73	0	0
1	0	Thoracotoı	265	CHU	No	54	0	1
1	1	Thoracotoı	265	CHU	Yes	66	0	0
1	0	Thoracotoı	265	CHU	No	57	0	0
1	0	VATS or rc	265	CHU	No	73	0	1
1	0	Thoracotoı	265	CHU	No	70	0	0
1	0	Thoracotoı	265	CHU	No	58	1	1
1	0	Thoracotoı	265	CHU	No	64	0	1
1	0	Thoracotoı	265	CHU	No	55	0	0
1	1	Thoracotoı	265	CHU	No	80	0	0
1	0	VATS or rc	265	CHU	No	56	0	0
1	0	VATS or rc	265	CHU	No	74	0	0
1	0	Thoracotoı	265	CHU	No	70	0	0
1	0	VATS or rc	265	CHU	No	71	1	1
1	0	Thoracotoı	265	CHU	No	76	0	0
1	0	Thoracotoı	265	CHU	No	70	1	0
1	0	VATS or rc	265	CHU	No	75	0	0
1	0	VATS or rc	265	CHU	No	73	0	0
1	0	Thoracotoı	265	CHU	No	28	0	0
1	0	VATS or rc	265	CHU	No	67	0	1
1	0	Thoracotoı	265	CHU	No	66	0	0
1	0	Thoracotoı	265	CHU	No	67	0	1
1	0	Thoracotoı	265	CHU	No	66	0	0
1	0	VATS or rc	265	CHU	No	58	1	0
1	0	Thoracotoı	265	CHU	Yes	54	0	0

1	0	Thoracotoı	265	CHU	No	65	1	1
1	0	Thoracotoı	265	CHU	No	74	0	1
1	0	Thoracotoı	265	CHU	No	72	0	0
1	1	Thoracotoı	265	CHU	No	51	0	1
1	0	VATS or rc	265	CHU	No	65	0	1
1	1	Thoracotoı	265	CHU	No	65	0	0
1	1	Thoracotoı	265	CHU	No	59	0	1
1	0	Thoracotoı	265	CHU	No	60	1	1
1	0	VATS or rc	265	CHU	No	45	0	0
1	0	Thoracotoı	265	CHU	No	54	0	0
1	0	VATS or rc	265	CHU	No	76	1	1
1	0	Thoracotoı	265	CHU	No	74	1	0
1	0	Thoracotoı	265	CHU	No	71	0	0
1	0	Thoracotoı	265	CHU	No	61	0	1
1	0	Thoracotoı	265	CHU	No	68	0	0
1	0	Thoracotoı	265	CHU	No	73	0	0
1	0	Thoracotoı	265	CHU	No	46	0	0
1	1	Thoracotoı	265	CHU	No	71	0	0
1	0	Thoracotoı	265	CHU	No	72	1	0
1	0	VATS or rc	265	CHU	Yes	75	0	0
1	0	VATS or rc	265	CHU	No	79	0	0
1	0	Thoracotoı	265	CHU	No	65	0	0
1	0	VATS or rc	265	CHU	No	69	0	1
1	0	Thoracotoı	265	CHU	No	56	0	0
1	0	VATS or rc	265	CHU	No	71	0	0
1	0	VATS or rc	265	CHU	No	60	0	0
1	0	Thoracotoı	265	CHU	No	66	0	0
1	0	Thoracotoı	265	CHU	No	59	0	1
1	0	Thoracotoı	265	CHU	No	67	0	0
1	0	VATS or rc	265	CHU	No	68	0	1
1	0	Thoracotoı	265	CHU	No	59	0	0
1	0	VATS or rc	265	CHU	No	66	0	0
1	0	Thoracotoı	265	CHU	No	62	0	0
1	0	Thoracotoı	265	CHU	No	53	0	1
1	0	Thoracotoı	265	CHU	No	50	0	0
1	0	VATS or rc	265	CHU	No	58	0	0
1	0	Thoracotoı	265	CHU	No	58	0	1
1	0	Thoracotoı	265	CHU	No	57	0	0
1	0	Thoracotoı	265	CHU	No	76	0	0
1	0	VATS or rc	265	CHU	No	52	0	1
1	0	Thoracotoı	265	CHU	No	81	0	0
1	0	Thoracotoı	265	CHU	No	60	0	1
1	0	VATS or rc	265	CHU	No	72	0	0
1	0	Thoracotoı	265	CHU	No	67	0	0
1	0	VATS or rc	265	CHU	No	63	0	0
1	1	Thoracotoı	265	CHU	Yes	74	0	0
1	0	Thoracotoı	265	CHU	No	63	0	1
1	0	VATS or rc	265	CHU	No	65	1	1
1	1	Thoracotoı	265	CHU	No	61	0	1
1	0	Thoracotoı	265	CHU	No	75	0	0
1	0	Thoracotoı	265	CHU	No	76	0	0
1	0	VATS or rc	265	CHU	No	61	0	0
1	0	VATS or rc	265	CHU	No	62	0	1
1	0	Thoracotoı	265	CHU	No	67	0	0
1	0	VATS or rc	265	CHU	No	77	0	0
1	0	Thoracotoı	265	CHU	No	67	0	0

1	0	Thoracotoı	265	CHU	No	60	0	1
1	0	VATS or rc	265	CHU	No	71	0	0
1	0	Thoracotoı	265	CHU	No	53	0	0
1	0	Thoracotoı	265	CHU	No	61	1	0
1	1	Thoracotoı	265	CHU	No	46	0	0
1	0	Thoracotoı	265	CHU	No	63	0	1
1	0	Thoracotoı	265	CHU	No	53	0	0
1	0	VATS or rc	265	CHU	No	51	0	1
1	0	VATS or rc	265	CHU	No	72	0	0
1	0	Thoracotoı	265	CHU	No	48	0	1
1	0	Thoracotoı	265	CHU	No	70	0	0
1	0	Thoracotoı	265	CHU	No	75	0	1
1	0	VATS or rc	265	CHU	No	65	0	0
1	0	VATS or rc	265	CHU	No	63	0	0
1	0	Thoracotoı	265	CHU	No	69	0	0
1	0	Thoracotoı	265	CHU	No	57	0	1
1	0	VATS or rc	265	CHU	No	66	0	0
1	0	VATS or rc	265	CHU	No	58	1	1
1	0	Thoracotoı	265	CHU	No	59	0	0
1	0	VATS or rc	265	CHU	No	56	0	1
1	0	VATS or rc	265	CHU	No	72	0	0
1	0	VATS or rc	265	CHU	No	54	0	0
1	0	Thoracotoı	265	CHU	No	78	0	0
1	0	VATS or rc	265	CHU	No	72	0	0
1	0	VATS or rc	265	CHU	No	66	0	1
1	0	VATS or rc	265	CHU	No	67	1	0
1	0	Thoracotoı	265	CHU	No	58	0	1
1	0	Thoracotoı	265	CHU	No	63	0	0
1	0	Thoracotoı	265	CHU	No	65	1	0
1	0	Thoracotoı	265	CHU	No	54	0	0
1	0	Thoracotoı	265	CHU	No	64	0	0
1	0	Thoracotoı	265	CHU	No	58	0	0
1	0	Thoracotoı	265	CHU	No	48	0	0
1	0	VATS or rc	265	CHU	No	68	0	0
1	0	Thoracotoı	265	CHU	No	67	0	0
1	0	VATS or rc	265	CHU	No	66	0	0
1	0	Thoracotoı	265	CHU	No	60	0	1
1	0	VATS or rc	265	CHU	No	60	0	0
1	0	Thoracotoı	265	CHU	No	53	0	0
1	0	Thoracotoı	265	CHU	No	67	0	1
1	0	VATS or rc	265	CHU	No	72	0	1
1	0	Thoracotoı	265	CHU	No	48	1	1
1	0	Thoracotoı	265	CHU	No	63	0	0
1	0	VATS or rc	265	CHU	No	61	1	0
1	1	Thoracotoı	265	CHU	No	58	0	1
1	0	Thoracotoı	265	CHU	No	48	0	1
1	0	Thoracotoı	265	CHU	No	79	0	0
1	0	Thoracotoı	265	CHU	No	80	1	0
1	0	Thoracotoı	265	CHU	No	42	0	0
1	0	VATS or rc	265	CHU	No	60	0	1
1	0	Thoracotoı	265	CHU	No	75	0	1
1	0	Thoracotoı	265	CHU	No	55	0	0
1	0	Thoracotoı	265	CHU	No	62	0	1
1	0	Thoracotoı	265	CHU	No	59	0	0
1	0	Thoracotoı	265	CHU	No	56	0	0
1	0	Thoracotoı	265	CHU	No	71	0	0

1	0	Thoracotoı	265	CHU	No	77	1	0
1	0	Thoracotoı	265	CHU	No	65	0	0
1	0	VATS or rc	265	CHU	No	67	1	0
1	0	Thoracotoı	265	CHU	No	79	0	0
1	0	Thoracotoı	265	CHU	No	66	0	0
1	0	Thoracotoı	265	CHU	No	61	0	0
1	0	Thoracotoı	265	CHU	No	59	0	0
1	0	Thoracotoı	265	CHU	No	61	0	0
1	0	Thoracotoı	265	CHU	No	80	1	0
1	1	Thoracotoı	265	CHU	No	61	0	1
1	0	VATS or rc	265	CHU	No	60	0	0
1	1	Thoracotoı	265	CHU	No	46	0	1
1	1	Thoracotoı	265	CHU	No	56	0	1
1	0	VATS or rc	265	CHU	No	69	0	1
1	0	VATS or rc	265	CHU	No	54	0	0
1	0	VATS or rc	265	CHU	No	72	0	0
1	0	Thoracotoı	265	CHU	No	60	0	1
1	0	Thoracotoı	265	CHU	No	83	0	0
1	0	Thoracotoı	265	CHU	No	52	1	0
1	0	Thoracotoı	265	CHU	No	61	0	0
1	0	Thoracotoı	265	CHU	No	46	0	1
1	0	Thoracotoı	265	CHU	No	57	0	0
1	0	Thoracotoı	265	CHU	No	49	0	1
1	0	Thoracotoı	265	CHU	No	71	0	0
1	0	Thoracotoı	265	CHU	No	74	0	0
1	0	Thoracotoı	265	CHU	No	66	0	1
1	1	Thoracotoı	265	CHU	No	56	0	0
1	0	Thoracotoı	265	CHU	No	72	1	0
1	0	Thoracotoı	265	CHU	No	69	0	0
1	0	Thoracotoı	265	CHU	No	76	0	0
1	0	Thoracotoı	265	CHU	No	58	0	0
1	0	VATS or rc	265	CHU	No	78	0	0
1	0	Thoracotoı	265	CHU	No	75	0	1
1	0	VATS or rc	265	CHU	No	65	0	1
1	0	Thoracotoı	265	CHU	No	67	0	0
1	0	Thoracotoı	265	CHU	No	74	1	0
1	0	Thoracotoı	265	CHU	No	73	0	0
1	0	Thoracotoı	265	CHU	No	48	1	0
1	0	Thoracotoı	265	CHU	No	59	0	0
1	0	Thoracotoı	265	CHU	No	59	0	0
1	0	Thoracotoı	265	CHU	No	76	0	1
1	0	Thoracotoı	265	CHU	No	63	0	1
1	1	Thoracotoı	265	CHU	Yes	63	0	1
1	0	Thoracotoı	265	CHU	No	48	0	0
1	0	Thoracotoı	265	CHU	No	75	1	0
1	0	VATS or rc	265	CHU	No	52	0	0
1	0	VATS or rc	265	CHU	No	70	0	0
1	0	VATS or rc	265	CHU	No	68	0	0
1	0	Thoracotoı	265	CHU	No	55	0	0
1	0	Thoracotoı	265	CHU	No	53	0	1
1	0	Thoracotoı	265	CHU	No	67	0	1
1	0	VATS or rc	265	CHU	No	73	0	0
1	0	Thoracotoı	265	CHU	No	62	0	0
1	0	Thoracotoı	265	CHU	No	86	0	1
1	0	Thoracotoı	265	CHU	No	64	1	0
1	0	Thoracotoı	265	CHU	No	72	0	0

1	1	Thoracotor	265	CHU	No	72	0	0
1	0	Thoracotor	265	CHU	No	56	0	0
1	0	Thoracotor	265	CHU	No	59	0	1
1	0	Thoracotor	265	CHU	No	58	0	1
1	0	VATS or ro	265	CHU	No	65	0	0
1	0	VATS or ro	265	CHU	No	68	0	0
1	0	VATS or ro	265	CHU	No	56	0	1
1	0	Thoracotor	265	CHU	No	68	0	0
1	0	Thoracotor	265	CHU	No	77	0	0
1	1	Thoracotor	265	CHU	No	52	0	1
1	0	Thoracotor	265	CHU	No	65	0	0
1	0	Thoracotor	265	CHU	No	66	0	1
1	0	VATS or ro	265	CHU	No	64	0	0
1	0	Thoracotor	265	CHU	No	73	1	1
1	0	Thoracotor	265	CHU	No	48	0	1
1	0	VATS or ro	265	CHU	No	77	1	0
1	0	VATS or ro	265	CHU	No	58	0	0
1	0	VATS or ro	265	CHU	No	75	0	1
1	0	Thoracotor	265	CHU	No	60	0	0
1	0	Thoracotor	265	CHU	No	80	1	0
1	0	Thoracotor	265	CHU	No	66	0	0
1	0	Thoracotor	265	CHU	No	69	0	0
1	0	Thoracotor	265	CHU	No	59	0	1
1	0	Thoracotor	265	CHU	No	70	0	0
1	0	Thoracotor	265	CHU	No	83	0	1
1	0	VATS or ro	265	CHU	No	71	0	1
1	1	Thoracotor	265	CHU	No	52	0	1
1	0	VATS or ro	265	CHU	No	56	0	0
1	1	Thoracotor	265	CHU	Yes	76	0	0
1	0	VATS or ro	265	CHU	No	72	0	0
1	0	Thoracotor	265	CHU	No	60	0	1
1	0	Thoracotor	265	CHU	No	61	0	0
1	0	Thoracotor	265	CHU	No	50	1	0
1	0	Thoracotor	265	CHU	No	61	0	0
1	0	Thoracotor	265	CHU	No	63	0	1
1	0	Thoracotor	265	CHU	No	55	0	1
1	0	Thoracotor	265	CHU	No	52	0	0
1	0	Thoracotor	265	CHU	No	58	0	0
1	0	Thoracotor	265	CHU	No	72	0	0
1	0	Thoracotor	265	CHU	No	54	0	0
1	0	Thoracotor	265	CHU	No	58	0	0
1	0	Thoracotor	265	CHU	No	65	0	0
1	0	Thoracotor	265	CHU	No	53	0	0
1	0	Thoracotor	265	CHU	No	70	1	0
1	0	Thoracotor	265	CHU	No	60	0	1
1	0	VATS or ro	265	CHU	No	67	0	0
1	0	Thoracotor	265	CHU	No	61	0	0
1	0	VATS or ro	265	CHU	Yes	65	0	0
1	0	Thoracotor	265	CHU	No	84	0	0
1	0	Thoracotor	265	CHU	No	73	0	0
1	0	Thoracotor	265	CHU	No	73	0	0
1	0	Thoracotor	265	CHU	No	67	0	0
1	0	VATS or ro	265	CHU	No	75	0	0
1	0	Thoracotor	265	CHU	No	66	0	1
1	1	Thoracotor	265	CHU	No	66	1	0
1	0	Thoracotor	265	CHU	No	63	1	1

1	0	Thoracotoı	265	CHU	No	72	0	0
1	0	Thoracotoı	265	CHU	No	54	0	1
1	0	VATS or rc	265	CHU	No	59	0	0
1	0	VATS or rc	265	CHU	No	69	0	1
1	0	Thoracotoı	265	CHU	No	60	0	1
1	0	Thoracotoı	265	CHU	No	75	0	0
1	0	VATS or rc	265	CHU	No	60	0	1
1	0	Thoracotoı	265	CHU	No	78	0	0
1	1	Thoracotoı	265	CHU	No	60	0	0
1	0	Thoracotoı	265	CHU	No	73	0	0
1	0	Thoracotoı	265	CHU	No	69	0	0
1	0	Thoracotoı	265	CHU	No	68	0	0
1	0	VATS or rc	265	CHU	No	58	0	0
1	1	Thoracotoı	265	CHU	Yes	55	0	0
1	0	Thoracotoı	265	CHU	No	75	0	0
1	0	Thoracotoı	265	CHU	No	59	0	0
1	0	Thoracotoı	265	CHU	No	81	1	0
1	0	VATS or rc	265	CHU	No	68	0	0
1	0	Thoracotoı	265	CHU	No	57	0	0
1	0	Thoracotoı	265	CHU	No	57	0	1
1	0	VATS or rc	265	CHU	No	45	0	0
1	0	VATS or rc	265	CHU	No	55	0	1
1	0	VATS or rc	265	CHU	No	57	0	0
1	0	Thoracotoı	265	CHU	No	75	0	0
1	0	VATS or rc	265	CHU	No	58	0	1
1	0	Thoracotoı	265	CHU	No	63	1	0
1	0	VATS or rc	265	CHU	No	57	0	0
1	0	VATS or rc	265	CHU	No	59	0	0
1	0	Thoracotoı	265	CHU	No	81	0	1
1	0	Thoracotoı	265	CHU	No	75	0	0
1	0	Thoracotoı	265	CHU	No	45	0	1
1	0	Thoracotoı	265	CHU	No	78	1	0
1	0	Thoracotoı	265	CHU	No	67	1	0
1	0	Thoracotoı	265	CHU	No	63	0	1
1	0	Thoracotoı	265	CHU	No	67	0	0
1	0	Thoracotoı	265	CHU	No	81	0	0
1	0	VATS or rc	265	CHU	No	69	0	0
1	1	Thoracotoı	265	CHU	No	67	0	0
1	0	Thoracotoı	265	CHU	No	65	0	0
1	0	VATS or rc	265	CHU	No	70	0	0
1	0	Thoracotoı	265	CHU	No	66	0	0
1	0	Thoracotoı	265	CHU	No	69	0	0
1	0	VATS or rc	265	CHU	No	69	0	1
1	0	Thoracotoı	265	CHU	No	81	0	1
1	0	Thoracotoı	265	CHU	No	59	1	0
1	0	VATS or rc	265	CHU	No	55	0	0
1	0	VATS or rc	265	CHU	No	74	0	0
1	0	Thoracotoı	265	CHU	No	72	0	0
1	0	Thoracotoı	265	CHU	No	51	0	1
1	0	Thoracotoı	265	CHU	No	63	0	0
1	0	Thoracotoı	265	CHU	No	30	0	0
1	0	Thoracotoı	265	CHU	No	54	0	1
1	0	VATS or rc	265	CHU	No	64	0	0
1	0	VATS or rc	265	CHU	No	83	0	0
1	0	Thoracotoı	265	CHU	No	55	0	1
1	0	Thoracotoı	265	CHU	No	59	0	1

1	0	Thoracotoı	265	CHU	No	68	0	1
1	1	Thoracotoı	265	CHU	No	29	0	0
1	0	Thoracotoı	265	CHU	No	71	0	1
1	0	VATS or rc	265	CHU	No	81	0	0
1	0	VATS or rc	265	CHU	No	46	0	0
1	0	Thoracotoı	265	CHU	No	40	0	0
1	0	VATS or rc	265	CHU	No	63	0	0
1	0	Thoracotoı	265	CHU	No	60	0	0
1	0	VATS or rc	265	CHU	No	58	1	0
1	0	Thoracotoı	265	CHU	No	68	0	0
1	0	Thoracotoı	265	CHU	No	76	0	0
1	0	Thoracotoı	265	CHU	No	39	0	0
1	1	Thoracotoı	265	CHU	No	62	0	0
1	0	Thoracotoı	265	CHU	No	60	0	1
1	0	VATS or rc	265	CHU	No	64	0	1
1	1	Thoracotoı	265	CHU	No	55	0	0
1	0	Thoracotoı	265	CHU	No	61	0	0
1	0	Thoracotoı	265	CHU	No	51	0	1
1	0	Thoracotoı	265	CHU	No	58	0	0
1	0	Thoracotoı	265	CHU	No	56	0	1
1	0	VATS or rc	265	CHU	No	59	0	0
1	0	VATS or rc	265	CHU	No	67	0	0
1	0	Thoracotoı	265	CHU	No	70	1	0
1	0	Thoracotoı	265	CHU	Yes	81	0	1
1	1	Thoracotoı	265	CHU	No	59	0	0
1	0	Thoracotoı	265	CHU	No	65	0	0
1	0	Thoracotoı	265	CHU	No	58	0	1
1	0	Thoracotoı	265	CHU	No	66	0	0
1	0	Thoracotoı	265	CHU	No	71	0	0
1	0	VATS or rc	265	CHU	No	63	0	0
1	0	Thoracotoı	265	CHU	No	68	0	0
1	0	VATS or rc	265	CHU	No	64	0	1
1	0	Thoracotoı	265	CHU	No	50	0	0
1	0	Thoracotoı	265	CHU	No	54	0	0
1	0	VATS or rc	265	CHU	No	54	0	1
1	0	Thoracotoı	265	CHU	No	70	0	1
1	0	VATS or rc	265	CHU	No	58	0	1
1	0	Thoracotoı	265	CHU	No	73	0	1
1	0	VATS or rc	265	CHU	No	66	0	0
1	0	Thoracotoı	265	CHU	No	78	0	0
1	0	VATS or rc	265	CHU	No	64	0	1
1	0	Thoracotoı	265	CHU	No	48	0	0
1	0	Thoracotoı	265	CHU	No	70	0	0
1	0	Thoracotoı	265	CHU	No	58	0	0
1	0	VATS or rc	265	CHU	No	58	0	0
1	0	Thoracotoı	265	CHU	Yes	70	0	1
1	0	Thoracotoı	265	CHU	No	59	0	1
1	0	VATS or rc	265	CHU	No	57	0	0
1	0	Thoracotoı	265	CHU	No	66	0	0
1	0	Thoracotoı	265	CHU	No	78	1	0
1	0	Thoracotoı	265	CHU	No	62	0	0
1	0	VATS or rc	265	CHU	No	63	0	0
1	0	Thoracotoı	265	CHU	No	57	1	0
1	1	Thoracotoı	265	CHU	No	43	0	0
1	0	Thoracotoı	265	CHU	No	68	0	0
1	0	Thoracotoı	265	CHU	No	70	0	1

1	0	VATS or rc	265	CHU	No	62	1	0
1	0	VATS or rc	265	CHU	No	72	0	1
1	0	Thoracotoı	265	CHU	No	21	0	0
1	0	VATS or rc	265	CHU	No	48	0	1
1	0	Thoracotoı	265	CHU	No	59	1	0
1	0	VATS or rc	265	CHU	No	71	0	1
1	0	Thoracotoı	265	CHU	No	54	0	0
1	0	VATS or rc	265	CHU	No	76	0	1
1	0	Thoracotoı	265	CHU	No	76	1	1
1	0	VATS or rc	265	CHU	No	74	0	0
1	0	Thoracotoı	265	CHU	No	67	0	1
1	0	Thoracotoı	265	CHU	No	70	1	0
1	0	VATS or rc	265	CHU	No	70	1	0
1	0	VATS or rc	265	CHU	No	80	0	1
1	0	Thoracotoı	265	CHU	No	73	0	1
1	1	Thoracotoı	265	CHU	No	74	0	0
1	0	VATS or rc	265	CHU	No	81	0	0
1	0	VATS or rc	265	CHU	No	70	0	0
1	0	Thoracotoı	265	CHU	No	80	0	0
1	0	Thoracotoı	265	CHU	No	75	0	1
1	0	Thoracotoı	265	CHU	No	64	0	1
1	0	Thoracotoı	265	CHU	No	67	0	0
1	0	Thoracotoı	265	CHU	No	65	0	0
1	0	Thoracotoı	265	CHU	No	52	1	0
1	0	Thoracotoı	265	CHU	No	66	0	1
1	0	Thoracotoı	265	CHU	No	58	0	0
1	0	VATS or rc	265	CHU	No	77	1	1
1	0	VATS or rc	265	CHU	No	74	0	0
1	0	Thoracotoı	265	CHU	No	73	0	0
1	0	Thoracotoı	265	CHU	No	71	0	1
1	0	Thoracotoı	265	CHU	No	71	0	1
1	0	Thoracotoı	265	CHU	Yes	75	0	0
1	0	Thoracotoı	265	CHU	Yes	69	0	0
1	0	Thoracotoı	265	CHU	No	49	0	0
1	0	VATS or rc	265	CHU	No	81	0	0
1	0	VATS or rc	265	CHU	No	62	0	0
1	0	VATS or rc	265	CHU	No	71	0	0
1	0	Thoracotoı	265	CHU	No	68	0	0
1	0	Thoracotoı	265	CHU	No	63	0	0
1	0	Thoracotoı	265	CHU	No	53	0	1
1	0	Thoracotoı	265	CHU	No	70	0	1
1	0	Thoracotoı	265	CHU	No	82	0	0
1	0	Thoracotoı	265	CHU	No	64	0	1
1	0	Thoracotoı	265	CHU	No	77	0	0
1	0	Thoracotoı	265	CHU	No	69	0	0
1	0	Thoracotoı	265	CHU	No	68	0	0
1	1	Thoracotoı	265	CHU	No	46	0	0
1	0	Thoracotoı	265	CHU	No	69	1	0
1	0	Thoracotoı	265	CHU	No	59	0	1
1	1	Thoracotoı	265	CHU	No	49	0	1
1	0	Thoracotoı	265	CHU	No	28	0	0
1	0	Thoracotoı	265	CHU	No	73	0	0
1	0	Thoracotoı	265	CHU	No	71	0	0
1	0	Thoracotoı	265	CHU	No	51	0	1
1	0	VATS or rc	265	CHU	No	56	0	1
1	0	Thoracotoı	265	CHU	No	71	0	1

1	0	VATS or ro	265	CHU	No	67	0	1
1	0	VATS or ro	265	CHU	No	54	0	0
1	0	Thoracotor	265	CHU	No	59	0	1
1	0	Thoracotor	265	CHU	No	41	0	1
1	0	Thoracotor	265	CHU	No	67	0	1
1	0	VATS or ro	265	CHU	No	81	0	0
1	0	VATS or ro	265	CHU	No	62	0	1
1	0	Thoracotor	265	CHU	No	72	0	0
1	0	VATS or ro	265	CHU	No	68	1	0
1	0	Thoracotor	265	CHU	No	49	0	0
1	0	Thoracotor	265	CHU	No	54	0	0
1	0	Thoracotor	265	CHU	No	73	0	1
1	0	Thoracotor	265	CHU	No	74	0	0
1	0	Thoracotor	265	CHU	No	74	1	1
1	0	Thoracotor	265	CHU	No	53	0	1
1	0	Thoracotor	265	CHU	No	55	1	1
1	0	VATS or ro	265	CHU	No	57	0	1
1	0	VATS or ro	265	CHU	No	75	1	1
1	0	Thoracotor	265	CHU	No	76	0	0
1	0	VATS or ro	265	CHU	No	65	0	0
1	0	Thoracotor	265	CHU	No	65	0	0
1	0	Thoracotor	265	CHU	No	48	0	0
1	0	Thoracotor	265	CHU	No	59	0	1
1	0	Thoracotor	265	CHU	No	78	0	0
1	0	VATS or ro	265	CHU	No	76	0	0
1	0	Thoracotor	265	CHU	No	61	0	0
1	0	Thoracotor	265	CHU	No	65	0	1
1	0	Thoracotor	265	CHU	No	58	0	0
1	0	Thoracotor	265	CHU	No	64	0	0
1	0	Thoracotor	265	CHU	No	75	0	0
1	0	Thoracotor	265	CHU	No	62	0	0
1	0	Thoracotor	265	CHU	No	54	0	1
1	0	Thoracotor	265	CHU	No	59	0	0
1	0	Thoracotor	265	CHU	No	69	1	0
1	0	Thoracotor	265	CHU	No	74	0	0
1	0	VATS or ro	265	CHU	No	66	0	1
1	0	Thoracotor	265	CHU	No	80	0	0
1	0	Thoracotor	265	CHU	No	63	0	0
1	0	VATS or ro	265	CHU	No	61	0	0
1	0	Thoracotor	265	CHU	No	61	0	0
1	0	Thoracotor	265	CHU	No	68	0	1
1	0	Thoracotor	265	CHU	No	61	0	0
1	0	Thoracotor	265	CHU	No	69	0	1
1	0	Thoracotor	265	CHU	No	67	0	0
1	1	Thoracotor	265	CHU	No	51	0	0
1	0	Thoracotor	265	CHU	No	60	0	0
1	0	VATS or ro	265	CHU	No	70	0	0
1	0	Thoracotor	265	CHU	No	68	1	0
1	0	Thoracotor	265	CHU	No	61	0	0
1	0	Thoracotor	265	CHU	No	79	0	0
1	0	Thoracotor	265	CHU	No	52	0	1
1	0	Thoracotor	265	CHU	No	75	0	1
1	0	Thoracotor	265	CHU	No	74	0	0
1	0	Thoracotor	265	CHU	No	64	0	0
1	0	Thoracotor	265	CHU	No	77	0	1
1	0	Thoracotor	265	CHU	No	78	0	0

1	0	Thoracotoı	265	CHU	No	72	0	1
1	0	Thoracotoı	265	CHU	No	77	0	0
1	0	Thoracotoı	265	CHU	No	54	0	0
1	0	Thoracotoı	265	CHU	No	74	0	0
1	0	Thoracotoı	265	CHU	No	60	0	0
1	0	Thoracotoı	265	CHU	No	72	0	0
1	0	Thoracotoı	265	CHU	No	71	0	0
1	1	Thoracotoı	265	CHU	No	71	0	0
1	0	Thoracotoı	265	CHU	No	67	1	0
1	0	Thoracotoı	265	CHU	No	68	0	1
1	0	VATS or ro	265	CHU	No	73	0	0
1	0	Thoracotoı	265	CHU	No	53	0	0
1	0	Thoracotoı	265	CHU	No	61	0	0
1	0	VATS or ro	265	CHU	No	62	0	0
1	0	VATS or ro	265	CHU	No	44	0	0
1	0	Thoracotoı	265	CHU	No	48	0	0
1	0	Thoracotoı	265	CHU	No	55	0	0
1	0	VATS or ro	265	CHU	No	60	0	0
1	0	Thoracotoı	265	CHU	No	68	0	0
1	0	VATS or ro	265	CHU	No	68	1	1
1	1	Thoracotoı	265	CHU	No	79	0	1
1	1	Thoracotoı	265	CHU	No	63	0	1
1	0	Thoracotoı	265	CHU	No	84	0	0
1	0	Thoracotoı	265	CHU	No	67	0	1
1	0	Thoracotoı	265	CHU	No	54	0	1
1	0	Thoracotoı	265	CHU	No	53	0	0
1	0	Thoracotoı	265	CHU	No	71	0	1
1	0	Thoracotoı	265	CHU	No	56	0	0
1	0	Thoracotoı	265	CHU	No	75	0	0
1	0	Thoracotoı	265	CHU	No	82	0	0
1	0	Thoracotoı	265	CHU	No	69	0	0
1	0	Thoracotoı	265	CHU	No	66	0	0
1	0	VATS or ro	265	CHU	No	55	0	1
1	0	Thoracotoı	265	CHU	No	51	0	0
1	0	Thoracotoı	265	CHU	No	68	0	0
1	0	Thoracotoı	265	CHU	No	67	0	0
1	0	Thoracotoı	265	CHU	No	69	0	0
1	0	VATS or ro	265	CHU	No	64	1	1
1	0	Thoracotoı	265	CHU	No	58	0	1
1	0	Thoracotoı	265	CHU	No	61	0	1
1	0	Thoracotoı	265	CHU	Yes	62	0	1
1	0	Thoracotoı	265	CHU	No	73	0	1
1	0	Thoracotoı	265	CHU	No	71	0	0
1	0	Thoracotoı	265	CHU	Yes	68	0	0
1	0	Thoracotoı	265	CHU	Yes	65	0	0
1	0	Thoracotoı	265	CHU	No	68	0	0
1	0	Thoracotoı	265	CHU	No	66	0	0
1	0	Thoracotoı	265	CHU	No	53	0	1
1	0	Thoracotoı	265	CHU	No	70	0	1
1	0	VATS or ro	265	CHU	No	65	0	0
1	0	VATS or ro	265	CHU	No	76	0	0
1	0	VATS or ro	265	CHU	No	71	1	0
1	0	Thoracotoı	265	CHU	No	60	0	0
1	0	Thoracotoı	265	CHU	No	72	0	1
1	0	Thoracotoı	265	CHU	Yes	66	0	0
1	0	Thoracotoı	265	CHU	No	64	1	1

1	0	VATS or ro	265	CHU	No	43	0	0
1	0	VATS or ro	265	CHU	No	83	0	0
1	0	Thoracotoı	265	CHU	No	70	0	0
1	0	Thoracotoı	265	CHU	No	57	0	1
1	0	Thoracotoı	265	CHU	No	58	0	1
1	0	Thoracotoı	265	CHU	No	66	0	0
1	0	Thoracotoı	265	CHU	No	67	0	1
1	0	VATS or ro	265	CHU	No	48	0	0
1	0	Thoracotoı	265	CHU	No	40	0	0
1	0	Thoracotoı	265	CHU	No	46	0	1
1	0	Thoracotoı	265	CHU	No	45	0	0
1	0	VATS or ro	265	CHU	No	62	1	0
1	0	VATS or ro	265	CHU	No	49	0	0
1	0	Thoracotoı	265	CHU	No	52	0	0
1	0	Thoracotoı	265	CHU	No	48	0	1
1	0	VATS or ro	265	CHU	No	74	0	0
1	0	Thoracotoı	265	CHU	No	70	0	0
1	0	Thoracotoı	265	CHU	No	66	0	0
1	0	Thoracotoı	265	CHU	No	63	0	1
1	0	Thoracotoı	265	CHU	No	61	0	0
1	0	VATS or ro	265	CHU	No	85	0	0
1	0	Thoracotoı	265	CHU	No	68	0	0
1	0	Thoracotoı	265	CHU	No	60	0	1
1	0	Thoracotoı	265	CHU	Yes	71	0	0
1	0	Thoracotoı	265	CHU	No	56	0	0
1	0	Thoracotoı	265	CHU	No	68	0	1
1	1	Thoracotoı	265	CHU	No	39	0	0
1	0	Thoracotoı	265	CHU	No	60	0	0
1	0	Thoracotoı	265	CHU	No	62	0	1
1	0	Thoracotoı	265	CHU	No	58	0	0
1	0	Thoracotoı	265	CHU	No	50	0	1
1	0	VATS or ro	265	CHU	No	47	0	1
1	0	Thoracotoı	265	CHU	No	63	0	0
1	0	Thoracotoı	265	CHU	No	70	1	0
1	0	Thoracotoı	265	CHU	No	76	0	0
1	0	Thoracotoı	265	CHU	No	65	0	0
1	0	Thoracotoı	265	CHU	No	47	0	1
1	0	Thoracotoı	265	CHU	No	69	0	1
1	0	Thoracotoı	265	CHU	No	62	0	0
1	1	Thoracotoı	265	CHU	No	48	0	0
1	0	Thoracotoı	265	CHU	No	63	0	1
1	0	Thoracotoı	265	CHU	No	51	0	1
1	0	Thoracotoı	265	CHU	No	74	0	0
1	0	Thoracotoı	265	CHU	No	81	0	0
1	0	VATS or ro	265	CHU	No	63	0	0
1	0	Thoracotoı	265	CHU	No	60	0	1
1	0	Thoracotoı	265	CHU	No	65	0	0
1	0	Thoracotoı	265	CHU	No	62	0	0
1	0	Thoracotoı	265	CHU	No	76	0	0
1	0	VATS or ro	265	CHU	No	72	0	0
1	0	Thoracotoı	265	CHU	No	74	0	0
1	0	Thoracotoı	265	CHU	No	55	0	1
1	0	Thoracotoı	265	CHU	No	67	0	0
1	0	VATS or ro	265	CHU	No	66	0	1
1	0	VATS or ro	265	CHU	No	57	0	1
1	0	Thoracotoı	265	CHU	No	62	0	1

1	0	VATS or rc	265	CHU	Yes	79	0	0
1	0	VATS or rc	265	CHU	No	70	1	0
1	0	Thoracotoı	265	CHU	Yes	72	0	1
1	0	Thoracotoı	265	CHU	No	59	1	1
1	0	VATS or rc	265	CHU	No	58	0	0
1	0	Thoracotoı	265	CHU	No	56	0	1
1	0	Thoracotoı	265	CHU	No	71	0	0
1	0	Thoracotoı	265	CHU	No	61	0	1
1	0	Thoracotoı	265	CHU	No	70	0	1
1	0	Thoracotoı	265	CHU	No	60	0	0
1	0	Thoracotoı	265	CHU	No	66	0	1
1	0	Thoracotoı	265	CHU	No	75	0	0
1	0	Thoracotoı	265	CHU	No	53	0	0
1	0	Thoracotoı	265	CHU	No	49	0	1
1	0	VATS or rc	265	CHU	No	64	0	0
1	0	Thoracotoı	265	CHU	No	59	0	0
1	0	VATS or rc	265	CHU	No	47	0	1
1	0	Thoracotoı	265	CHU	No	59	0	0
1	0	Thoracotoı	265	CHU	No	68	0	0
1	0	Thoracotoı	265	CHU	No	79	0	0
1	0	VATS or rc	265	CHU	No	73	0	0
1	0	Thoracotoı	265	CHU	No	54	0	0
1	0	VATS or rc	265	CHU	No	56	0	0
1	0	Thoracotoı	265	CHU	No	38	0	1
1	0	Thoracotoı	265	CHU	No	54	0	1
1	0	Thoracotoı	265	CHU	No	71	0	0
1	0	Thoracotoı	265	CHU	No	53	0	1
1	0	VATS or rc	265	CHU	No	60	0	0
1	0	Thoracotoı	265	CHU	No	77	0	0
1	0	Thoracotoı	265	CHU	No	55	0	0
1	0	VATS or rc	265	CHU	No	69	0	0
1	0	VATS or rc	265	CHU	No	67	0	0
1	0	Thoracotoı	265	CHU	No	70	1	0
1	0	Thoracotoı	265	CHU	No	74	0	0
1	0	Thoracotoı	265	CHU	No	59	0	0
1	1	Thoracotoı	265	CHU	No	63	1	0
1	0	Thoracotoı	265	CHU	No	66	1	0
1	1	Thoracotoı	265	CHU	No	66	0	0
1	1	Thoracotoı	265	CHU	No	52	0	0
1	0	VATS or rc	265	CHU	No	76	1	0
1	0	Thoracotoı	265	CHU	No	54	0	0
1	1	Thoracotoı	265	CHU	Yes	62	0	0
1	0	Thoracotoı	265	CHU	No	69	0	1
1	0	VATS or rc	265	CHU	No	49	0	1
1	0	Thoracotoı	265	CHU	No	59	0	0
1	0	Thoracotoı	265	CHU	No	49	0	0
1	0	Thoracotoı	265	CHU	No	43	0	0
1	0	Thoracotoı	265	CHU	No	79	0	0
1	0	Thoracotoı	265	CHU	No	48	1	1
1	0	Thoracotoı	265	CHU	No	63	0	0
1	0	Thoracotoı	265	CHU	No	65	0	0
1	0	Thoracotoı	265	CHU	No	58	0	0
1	0	VATS or rc	265	CHU	No	80	0	0
1	0	Thoracotoı	265	CHU	No	77	0	0
1	1	Thoracotoı	265	CHU	No	67	1	0
1	0	Thoracotoı	265	CHU	No	58	0	0

1	0	Thoracotoı	265	CHU	No	48	0	0
1	0	VATS or rc	265	CHU	No	80	0	1
1	0	VATS or rc	265	CHU	No	88	0	1
1	0	VATS or rc	265	CHU	No	59	0	0
1	0	Thoracotoı	265	CHU	No	79	0	0
1	0	Thoracotoı	265	CHU	No	51	0	1
1	0	VATS or rc	265	CHU	No	75	0	1
1	0	Thoracotoı	265	CHU	No	81	0	1
1	0	Thoracotoı	265	CHU	No	61	0	1
1	0	Thoracotoı	265	CHU	No	70	0	0
1	0	Thoracotoı	265	CHU	No	65	0	0
1	0	Thoracotoı	265	CHU	No	38	0	0
1	0	VATS or rc	265	CHU	No	74	0	0
1	0	Thoracotoı	265	CHU	Yes	77	1	0
1	1	Thoracotoı	265	CHU	No	61	0	0
1	0	Thoracotoı	265	CHU	No	73	0	0
1	0	VATS or rc	265	CHU	No	66	0	0
1	0	Thoracotoı	265	CHU	No	46	0	0
1	0	Thoracotoı	265	CHU	No	60	1	0
1	0	VATS or rc	265	CHU	No	80	0	0
1	0	Thoracotoı	265	CHU	No	62	0	1
1	0	Thoracotoı	265	CHU	No	68	0	0
1	0	Thoracotoı	265	CHU	No	69	0	0
1	0	Thoracotoı	265	CHU	No	61	1	0
1	0	Thoracotoı	265	CHU	No	72	0	0
1	0	Thoracotoı	265	CHU	No	71	0	0
1	0	VATS or rc	265	CHU	No	74	0	0
1	0	VATS or rc	265	CHU	No	63	0	1
1	0	VATS or rc	265	CHU	No	68	0	0
1	0	Thoracotoı	265	CHU	No	61	0	0
1	0	Thoracotoı	265	CHU	No	69	0	0
1	0	VATS or rc	265	CHU	No	64	0	1
1	0	Thoracotoı	265	CHU	No	72	1	0
1	1	Thoracotoı	265	CHU	No	60	1	1
1	0	Thoracotoı	265	CHU	No	45	0	0
1	1	Thoracotoı	265	CHU	No	68	0	0
1	0	VATS or rc	265	CHU	No	62	0	0
1	0	Thoracotoı	265	CHU	No	53	0	1
1	0	Thoracotoı	265	CHU	No	62	0	0
1	0	VATS or rc	265	CHU	No	73	0	0
1	0	VATS or rc	265	CHU	No	52	0	1
1	0	VATS or rc	265	CHU	No	53	0	0
1	0	Thoracotoı	265	CHU	No	61	0	1
1	0	Thoracotoı	265	CHU	No	61	0	0
1	0	Thoracotoı	265	CHU	No	71	0	1
1	0	Thoracotoı	265	CHU	No	67	0	0
1	0	Thoracotoı	265	CHU	No	65	0	0
1	0	VATS or rc	265	CHU	No	72	0	0
1	0	Thoracotoı	265	CHU	No	71	0	1
1	0	VATS or rc	265	CHU	No	82	0	0
1	0	Thoracotoı	265	CHU	No	68	0	0
1	0	VATS or rc	265	CHU	No	58	0	0
1	0	Thoracotoı	265	CHU	No	63	1	0
1	0	Thoracotoı	265	CHU	No	72	0	1
1	0	Thoracotoı	265	CHU	No	58	0	0
1	0	Thoracotoı	265	CHU	No	48	0	1

1	0	Thoracotoı	265	CHU	No	71	1	1
1	1	Thoracotoı	265	CHU	No	78	0	0
1	1	Thoracotoı	265	CHU	No	60	0	0
1	1	Thoracotoı	265	CHU	No	68	0	0
1	0	Thoracotoı	265	CHU	No	59	0	0
1	0	Thoracotoı	265	CHU	No	74	0	0
1	0	Thoracotoı	265	CHU	No	72	0	0
1	0	Thoracotoı	265	CHU	No	46	0	1
1	0	VATS or rc	265	CHU	No	70	1	0
1	0	Thoracotoı	265	CHU	No	53	0	1
1	0	Thoracotoı	265	CHU	No	47	0	0
1	0	Thoracotoı	265	CHU	No	60	0	0
1	0	Thoracotoı	265	CHU	No	70	0	0
1	0	VATS or rc	265	CHU	No	66	0	0
1	0	Thoracotoı	265	CHU	No	65	0	1
1	0	Thoracotoı	265	CHU	No	69	0	0
1	0	VATS or rc	265	CHU	No	80	0	0
1	0	Thoracotoı	265	CHU	No	48	0	1
1	0	Thoracotoı	265	CHU	Yes	78	0	0
1	0	Thoracotoı	265	CHU	No	70	0	0
1	0	VATS or rc	265	CHU	No	85	0	0
1	0	Thoracotoı	265	CHU	No	46	0	0
1	0	Thoracotoı	265	CHU	No	57	0	0
1	0	Thoracotoı	265	CHU	No	71	0	0
1	0	Thoracotoı	265	CHU	No	68	0	0
1	0	VATS or rc	265	CHU	No	54	1	0
1	1	Thoracotoı	265	CHU	No	56	0	1
1	0	Thoracotoı	265	CHU	No	62	0	1
1	0	Thoracotoı	265	CHU	No	53	0	0
1	1	Thoracotoı	265	CHU	No	63	0	1
1	0	Thoracotoı	265	CHU	No	64	0	1
1	0	Thoracotoı	265	CHU	No	48	0	0
1	0	VATS or rc	265	CHU	No	67	0	0
1	0	Thoracotoı	265	CHU	No	69	0	1
1	0	Thoracotoı	265	CHU	No	77	0	0
1	0	Thoracotoı	265	CHU	No	53	0	0
1	1	Thoracotoı	265	CHU	No	89	0	0
1	0	Thoracotoı	265	CHU	No	66	0	1
1	0	Thoracotoı	265	CHU	No	79	0	0
1	0	Thoracotoı	265	CHU	No	63	0	0
1	0	VATS or rc	265	CHU	No	77	0	0
1	0	VATS or rc	265	CHU	No	69	0	0
1	0	Thoracotoı	265	CHU	No	67	0	0
1	0	VATS or rc	265	CHU	No	75	0	0
1	0	Thoracotoı	265	CHU	No	79	0	1
1	0	Thoracotoı	265	CHU	No	73	1	0
1	0	Thoracotoı	265	CHU	No	78	0	0
1	0	Thoracotoı	265	CHU	No	63	0	0
1	0	Thoracotoı	265	CHU	No	47	0	1
1	0	Thoracotoı	265	CHU	No	70	0	1
1	0	Thoracotoı	265	CHU	No	43	0	1
1	0	Thoracotoı	265	CHU	No	56	0	0
1	0	VATS or rc	265	CHU	No	70	0	0
1	0	Thoracotoı	265	CHU	No	63	0	0
1	0	Thoracotoı	265	CHU	No	64	1	1
1	0	VATS or rc	265	CHU	No	82	0	0

1	0	VATS or rc	265	CHU	No	72	0	0
1	0	Thoracotoı	265	CHU	No	71	0	0
1	0	VATS or rc	265	CHU	No	70	1	1
1	0	VATS or rc	265	CHU	No	57	0	0
1	0	Thoracotoı	265	CHU	No	47	0	0
1	0	VATS or rc	265	CHU	No	78	0	0
1	0	Thoracotoı	265	CHU	No	62	0	1
1	0	VATS or rc	265	CHU	No	66	0	0
1	0	Thoracotoı	265	CHU	No	56	0	0
1	0	Thoracotoı	265	CHU	No	60	0	0
1	0	Thoracotoı	265	CHU	No	71	0	0
1	0	Thoracotoı	265	CHU	No	56	0	1
1	0	VATS or rc	265	CHU	No	74	0	0
1	0	Thoracotoı	265	CHU	No	71	0	1
1	0	Thoracotoı	265	CHU	No	40	0	0
1	1	Thoracotoı	265	CHU	No	72	0	0
1	0	VATS or rc	265	CHU	No	67	0	0
1	0	Thoracotoı	265	CHU	No	60	0	0
1	0	Thoracotoı	265	CHU	No	60	0	0
1	0	Thoracotoı	265	CHU	No	59	0	1
1	0	Thoracotoı	265	CHU	No	62	1	0
1	0	Thoracotoı	265	CHU	No	47	0	1
1	0	Thoracotoı	265	CHU	No	65	0	1
1	0	VATS or rc	265	CHU	No	75	0	0
1	0	Thoracotoı	265	CHU	Yes	77	0	0
1	0	Thoracotoı	265	CHU	No	61	0	1
1	0	Thoracotoı	265	CHU	No	62	0	1
1	1	Thoracotoı	265	CHU	No	79	0	0
1	0	VATS or rc	265	CHU	No	68	0	0
1	0	VATS or rc	265	CHU	No	70	1	0
1	0	Thoracotoı	265	CHU	No	79	0	1
1	0	VATS or rc	265	CHU	No	55	0	0
1	0	Thoracotoı	265	CHU	No	54	0	1
1	0	Thoracotoı	265	CHU	No	71	0	0
1	1	Thoracotoı	265	CHU	Yes	72	1	0
1	0	Thoracotoı	265	CHU	No	44	0	1
1	0	Thoracotoı	265	CHU	No	49	0	1
1	0	Thoracotoı	265	CHU	No	71	0	0
1	0	Thoracotoı	265	CHU	No	67	0	0
1	0	Thoracotoı	265	CHU	No	58	0	1
1	1	Thoracotoı	265	CHU	No	67	0	0
1	0	Thoracotoı	265	CHU	No	64	0	1
1	0	Thoracotoı	265	CHU	No	36	0	0
1	0	Thoracotoı	265	CHU	No	71	0	0
1	0	VATS or rc	265	CHU	No	57	0	1
1	0	VATS or rc	265	CHU	No	54	0	1
1	0	Thoracotoı	265	CHU	No	61	0	0
1	0	Thoracotoı	265	CHU	No	40	0	1
1	0	Thoracotoı	265	CHU	No	66	0	0
1	0	Thoracotoı	265	CHU	No	45	0	0
1	0	Thoracotoı	265	CHU	No	55	0	1
1	0	Thoracotoı	265	CHU	No	53	0	0
1	0	Thoracotoı	265	CHU	No	71	0	0
1	0	Thoracotoı	265	CHU	No	59	0	1
1	0	VATS or rc	265	CHU	No	65	0	0
1	0	VATS or rc	265	CHU	No	61	0	1

1	0	VATS or ro	265	CHU	No	83	0	0
1	0	Thoracotoı	265	CHU	No	56	0	1
1	0	Thoracotoı	265	CHU	No	74	0	1
1	0	Thoracotoı	265	CHU	No	63	0	0
1	1	Thoracotoı	265	CHU	No	63	0	1
1	0	Thoracotoı	265	CHU	No	79	0	0
1	0	Thoracotoı	265	CHU	No	54	0	1
1	0	Thoracotoı	265	CHU	No	61	0	1
1	0	Thoracotoı	265	CHU	No	60	1	0
1	0	Thoracotoı	265	CHU	No	64	0	1
1	0	VATS or ro	265	CHU	No	59	0	0
1	0	Thoracotoı	265	CHU	No	51	0	0
1	0	VATS or ro	265	CHU	No	65	0	0
1	0	Thoracotoı	265	CHU	Yes	71	0	0
1	0	Thoracotoı	265	CHU	No	53	0	0
1	0	Thoracotoı	265	CHU	No	58	1	0
1	0	Thoracotoı	265	CHU	No	58	1	0
1	0	Thoracotoı	265	CHU	No	82	0	0
1	0	Thoracotoı	265	CHU	No	46	0	1
1	0	Thoracotoı	265	CHU	No	63	0	1
1	0	VATS or ro	265	CHU	No	73	0	0
1	0	Thoracotoı	265	CHU	No	61	0	1
1	0	Thoracotoı	265	CHU	No	59	0	1
1	1	Thoracotoı	265	CHU	No	70	0	0
1	0	Thoracotoı	265	CHU	No	80	1	0
1	0	VATS or ro	265	CHU	No	86	0	0
1	0	VATS or ro	265	CHU	No	75	0	0
1	0	Thoracotoı	265	CHU	No	67	0	0
1	0	Thoracotoı	265	CHU	No	57	0	1
1	0	Thoracotoı	265	CHU	No	38	0	0
1	0	Thoracotoı	265	CHU	No	68	0	0
1	0	Thoracotoı	265	CHU	No	72	0	0
1	0	VATS or ro	265	CHU	No	43	0	0
1	0	Thoracotoı	265	CHU	No	53	0	0
1	0	VATS or ro	265	CHU	No	63	0	0
1	0	Thoracotoı	265	CHU	No	69	0	1
1	0	Thoracotoı	265	CHU	No	63	0	0
1	0	Thoracotoı	265	CHU	No	50	0	1
1	0	Thoracotoı	265	CHU	No	63	1	0
1	0	VATS or ro	265	CHU	No	71	0	1
1	0	Thoracotoı	265	CHU	No	72	0	1
1	0	Thoracotoı	265	CHU	No	56	0	0
1	0	Thoracotoı	265	CHU	No	73	0	0
1	0	VATS or ro	265	CHU	No	70	0	0
1	0	Thoracotoı	265	CHU	No	64	0	1
1	0	Thoracotoı	265	CHU	No	49	0	0
1	0	Thoracotoı	265	CHU	No	65	0	0
1	0	VATS or ro	265	CHU	No	66	0	1
1	0	Thoracotoı	265	CHU	No	58	0	1
1	0	Thoracotoı	265	CHU	No	70	0	0
1	0	VATS or ro	265	CHU	No	46	0	0
1	0	Thoracotoı	265	CHU	No	79	0	0
1	0	Thoracotoı	265	CHU	No	61	0	1
1	0	VATS or ro	265	CHU	No	75	0	0
1	0	VATS or ro	265	CHU	No	76	0	0
1	0	Thoracotoı	265	CHU	No	60	1	0

1	0	Thoracotoı	265	CHU	No	70	0	1
1	0	VATS or rc	265	CHU	No	57	1	1
1	0	Thoracotoı	265	CHU	Yes	61	0	0
1	1	Thoracotoı	265	CHU	No	72	0	0
1	0	Thoracotoı	265	CHU	No	72	0	0
1	0	Thoracotoı	265	CHU	No	61	0	0
1	0	VATS or rc	265	CHU	No	77	1	0
1	0	VATS or rc	265	CHU	No	65	1	1
1	0	Thoracotoı	265	CHU	No	47	0	1
1	0	Thoracotoı	265	CHU	No	60	1	1
1	0	VATS or rc	265	CHU	No	80	1	0
1	0	Thoracotoı	265	CHU	No	58	0	1
1	0	Thoracotoı	265	CHU	No	57	0	0
1	0	Thoracotoı	265	CHU	No	58	0	1
1	0	VATS or rc	265	CHU	No	63	1	0
1	0	Thoracotoı	265	CHU	No	75	0	0
1	0	Thoracotoı	265	CHU	No	54	0	0
1	0	Thoracotoı	265	CHU	No	83	1	0
1	0	Thoracotoı	265	CHU	No	56	0	1
1	0	Thoracotoı	265	CHU	No	72	0	0
1	0	VATS or rc	265	CHU	No	80	0	0
1	0	Thoracotoı	265	CHU	No	75	0	1
1	0	VATS or rc	265	CHU	No	80	0	1
1	0	Thoracotoı	265	CHU	No	68	0	0
1	0	Thoracotoı	265	CHU	No	52	0	0
1	0	Thoracotoı	265	CHU	No	53	0	1
1	0	Thoracotoı	265	CHU	No	52	0	1
1	1	Thoracotoı	265	CHU	No	67	0	1
1	0	VATS or rc	265	CHU	No	54	0	1
1	0	Thoracotoı	265	CHU	No	74	1	0
1	0	Thoracotoı	265	CHU	No	53	0	1
1	0	VATS or rc	265	CHU	No	59	0	0
1	0	Thoracotoı	265	CHU	No	77	0	1
1	0	Thoracotoı	265	CHU	No	65	0	0
1	1	Thoracotoı	265	CHU	Yes	63	0	0
1	0	VATS or rc	265	CHU	No	56	0	0
1	0	Thoracotoı	265	CHU	No	84	0	1
1	0	VATS or rc	265	CHU	No	63	0	0
1	1	Thoracotoı	265	CHU	No	68	0	1
1	0	Thoracotoı	265	CHU	No	80	0	0
1	0	Thoracotoı	265	CHU	No	59	0	0
1	0	Thoracotoı	265	CHU	No	74	0	1
1	0	Thoracotoı	265	CHU	No	68	1	0
1	0	Thoracotoı	265	CHU	No	78	0	0
1	0	Thoracotoı	265	CHU	No	76	0	0
1	0	Thoracotoı	265	CHU	No	62	0	1
1	0	Thoracotoı	265	CHU	No	57	0	1
1	0	VATS or rc	265	CHU	No	66	0	0
1	0	VATS or rc	265	CHU	No	66	0	0
1	0	Thoracotoı	265	CHU	No	43	0	1
1	0	Thoracotoı	265	CHU	No	83	0	0
1	0	VATS or rc	265	CHU	No	55	0	1
1	0	VATS or rc	265	CHU	No	73	0	0
1	0	Thoracotoı	265	CHU	No	78	0	0
1	0	Thoracotoı	265	CHU	No	54	0	1
1	0	Thoracotoı	265	CHU	No	54	0	0

1	0	VATS or rc	265	CHU	No	43	0	0
1	0	Thoracotoı	265	CHU	No	56	0	0
1	0	Thoracotoı	265	CHU	No	42	0	0
1	0	Thoracotoı	265	CHU	No	70	0	0
1	0	VATS or rc	265	CHU	No	65	0	1
1	0	Thoracotoı	265	CHU	No	71	0	1
1	0	Thoracotoı	265	CHU	No	56	0	1
1	0	Thoracotoı	265	CHU	No	76	0	0
1	0	Thoracotoı	265	CHU	No	53	0	1
1	1	Thoracotoı	265	CHU	No	74	0	0
1	0	Thoracotoı	265	CHU	No	74	0	0
1	0	VATS or rc	265	CHU	No	68	0	0
1	0	Thoracotoı	265	CHU	No	66	0	1
1	0	VATS or rc	265	CHU	No	71	0	0
1	1	Thoracotoı	265	CHU	No	74	0	0
1	0	Thoracotoı	265	CHU	No	46	0	1
1	0	VATS or rc	265	CHU	No	60	0	0
1	0	VATS or rc	265	CHU	No	72	0	0
1	0	Thoracotoı	265	CHU	No	61	0	1
1	0	Thoracotoı	265	CHU	No	81	0	0
1	0	Thoracotoı	265	CHU	No	53	0	1
1	0	Thoracotoı	265	CHU	No	58	0	0
1	1	Thoracotoı	265	CHU	No	62	0	0
1	0	Thoracotoı	265	CHU	No	59	0	0
1	1	Thoracotoı	265	CHU	No	79	0	0
1	0	Thoracotoı	265	CHU	No	59	0	1
1	0	Thoracotoı	265	CHU	No	66	0	0
1	0	Thoracotoı	265	CHU	No	78	0	0
1	0	VATS or rc	265	CHU	No	61	0	1
1	0	Thoracotoı	265	CHU	No	61	0	0
1	0	VATS or rc	265	CHU	No	64	1	0
1	0	VATS or rc	265	CHU	No	59	0	1
1	0	Thoracotoı	265	CHU	No	54	0	0
1	0	Thoracotoı	265	CHU	No	28	0	0
1	0	Thoracotoı	265	CHU	No	61	0	0
1	0	Thoracotoı	265	CHU	No	70	0	1
1	0	VATS or rc	265	CHU	No	28	0	0
1	0	Thoracotoı	265	CHU	No	55	0	1
1	0	Thoracotoı	265	CHU	No	59	0	1
1	1	Thoracotoı	265	CHU	No	57	0	1
1	0	VATS or rc	265	CHU	No	63	0	0
1	0	Thoracotoı	265	CHU	No	60	0	0
1	0	Thoracotoı	265	CHU	No	76	0	0
1	0	VATS or rc	265	CHU	No	73	0	0
1	0	Thoracotoı	265	CHU	No	72	0	1
1	0	VATS or rc	265	CHU	No	78	1	0
1	0	VATS or rc	265	CHU	No	60	0	1
1	0	Thoracotoı	265	CHU	No	51	0	1
1	1	Thoracotoı	265	CHU	No	76	0	0
1	0	VATS or rc	265	CHU	No	59	0	1
1	0	Thoracotoı	265	CHU	No	62	0	0
1	0	VATS or rc	265	CHU	No	84	0	1
1	0	Thoracotoı	265	CHU	No	68	0	1
1	0	Thoracotoı	265	CHU	No	80	0	0
1	1	Thoracotoı	265	CHU	No	74	0	0
1	0	Thoracotoı	265	CHU	No	66	0	1

1	0	VATS or rc	265	CHU	No	49	0	0
1	1	Thoracotoı	265	CHU	No	54	0	0
1	1	Thoracotoı	265	CHU	No	54	0	1
1	0	Thoracotoı	265	CHU	No	70	1	0
1	0	VATS or rc	265	CHU	No	51	0	0
1	0	Thoracotoı	265	CHU	No	69	0	0
1	0	VATS or rc	265	CHU	No	63	0	0
1	0	Thoracotoı	265	CHU	No	65	0	0
1	0	Thoracotoı	265	CHU	No	58	0	1
1	0	Thoracotoı	265	CHU	No	58	0	0
1	0	Thoracotoı	265	CHU	No	80	0	0
1	0	Thoracotoı	265	CHU	No	57	0	0
1	0	Thoracotoı	265	CHU	No	55	1	1
1	0	Thoracotoı	265	CHU	No	49	0	0
1	0	Thoracotoı	265	CHU	No	62	1	0
1	0	Thoracotoı	265	CHU	No	58	0	0
1	0	Thoracotoı	265	CHU	No	59	0	0
1	0	Thoracotoı	265	CHU	No	72	0	0
1	0	VATS or rc	265	CHU	No	77	0	1
1	0	Thoracotoı	265	CHU	No	63	0	0
1	0	Thoracotoı	265	CHU	No	74	0	0
1	0	Thoracotoı	265	CHU	No	59	0	0
1	0	Thoracotoı	265	CHU	No	65	0	0
1	0	Thoracotoı	265	CHU	No	74	0	0
1	1	Thoracotoı	265	CHU	No	62	0	0
1	0	Thoracotoı	265	CHU	No	67	0	0
1	0	Thoracotoı	265	CHU	No	77	0	1
1	0	VATS or rc	265	CHU	No	81	0	0
1	0	Thoracotoı	265	CHU	No	78	0	1
1	0	VATS or rc	265	CHU	No	59	0	0
1	0	Thoracotoı	265	CHU	No	61	0	1
1	0	Thoracotoı	265	CHU	No	61	0	1
1	0	Thoracotoı	265	CHU	No	22	0	1
1	0	Thoracotoı	265	CHU	No	80	1	0
1	0	VATS or rc	265	CHU	No	58	0	0
1	1	Thoracotoı	265	CHU	No	62	0	1
1	0	VATS or rc	265	CHU	No	79	0	0
1	0	Thoracotoı	265	CHU	No	59	0	0
1	0	Thoracotoı	265	CHU	No	44	0	0
1	0	Thoracotoı	265	CHU	No	68	1	0
1	0	Thoracotoı	265	CHU	No	66	1	0
1	0	Thoracotoı	265	CHU	No	67	0	1
1	0	VATS or rc	265	CHU	No	68	0	0
1	0	Thoracotoı	265	CHU	No	62	0	1
1	0	Thoracotoı	265	CHU	No	86	0	0
1	0	VATS or rc	265	CHU	No	73	0	0
1	0	Thoracotoı	265	CHU	No	62	0	0
1	0	VATS or rc	265	CHU	No	59	0	0
1	0	Thoracotoı	265	CHU	No	67	0	1
1	1	Thoracotoı	265	CHU	No	65	0	0
1	0	Thoracotoı	265	CHU	No	57	0	1
1	0	Thoracotoı	265	CHU	No	81	0	1
1	0	Thoracotoı	265	CHU	No	58	0	1
1	1	Thoracotoı	265	CHU	No	57	0	0
1	0	Thoracotoı	265	CHU	No	71	0	0
1	0	VATS or rc	265	CHU	No	46	0	1

1	0	Thoracotoı	265	CHU	No	53	0	0
1	0	VATS or rc	265	CHU	No	64	0	0
1	0	Thoracotoı	265	CHU	No	58	0	0
1	0	Thoracotoı	265	CHU	No	79	0	0
1	0	Thoracotoı	265	CHU	No	65	0	0
1	0	VATS or rc	265	CHU	No	66	0	1
1	0	Thoracotoı	265	CHU	No	64	1	0
1	0	Thoracotoı	265	CHU	No	62	0	0
1	0	VATS or rc	265	CHU	No	61	0	0
1	0	Thoracotoı	265	CHU	No	70	0	0
1	0	Thoracotoı	265	CHU	No	60	0	1
1	0	Thoracotoı	265	CHU	No	55	0	0
1	0	VATS or rc	265	CHU	No	58	0	0
1	1	Thoracotoı	265	CHU	No	64	0	0
1	0	Thoracotoı	265	CHU	No	70	0	1
1	0	Thoracotoı	265	CHU	No	73	0	0
1	0	VATS or rc	265	CHU	No	53	0	0
1	0	VATS or rc	265	CHU	No	78	0	1
1	0	VATS or rc	265	CHU	No	79	1	0
1	0	Thoracotoı	265	CHU	No	77	0	0
1	0	VATS or rc	265	CHU	No	72	0	0
1	0	VATS or rc	265	CHU	No	65	1	1
1	0	VATS or rc	265	CHU	No	61	0	1
1	0	Thoracotoı	265	CHU	No	82	0	1
1	0	Thoracotoı	265	CHU	No	76	0	0
1	0	Thoracotoı	265	CHU	No	67	1	1
1	0	Thoracotoı	265	CHU	No	63	0	0
1	0	Thoracotoı	265	CHU	No	62	0	0
1	0	Thoracotoı	265	CHU	No	59	1	0
1	0	VATS or rc	265	CHU	No	79	0	0
1	0	Thoracotoı	265	CHU	No	72	0	0
1	0	VATS or rc	265	CHU	No	80	0	0
1	0	Thoracotoı	265	CHU	No	64	0	0
1	1	Thoracotoı	265	CHU	No	70	0	0
1	0	VATS or rc	265	CHU	No	55	0	1
1	0	Thoracotoı	265	CHU	No	54	0	0
1	0	Thoracotoı	265	CHU	No	69	0	0
1	0	Thoracotoı	265	CHU	No	69	0	0
1	0	Thoracotoı	265	CHU	No	49	0	0
1	1	Thoracotoı	265	CHU	No	72	0	0
1	0	Thoracotoı	265	CHU	No	59	0	1
1	0	VATS or rc	265	CHU	No	75	0	0
1	0	VATS or rc	265	CHU	No	60	0	0
1	0	Thoracotoı	265	CHU	No	49	0	0
1	0	Thoracotoı	265	CHU	No	68	0	0
1	0	Thoracotoı	265	CHU	No	73	0	0
1	0	Thoracotoı	265	CHU	No	63	1	0
1	0	Thoracotoı	265	CHU	No	77	1	0
1	0	VATS or rc	265	CHU	No	71	0	1
1	0	VATS or rc	265	CHU	No	72	0	0
1	0	VATS or rc	265	CHU	No	76	0	0
1	0	Thoracotoı	265	CHU	No	57	0	0
1	1	Thoracotoı	265	CHU	No	57	0	1
1	0	Thoracotoı	265	CHU	No	58	1	0
1	0	Thoracotoı	265	CHU	No	61	1	0
1	0	Thoracotoı	265	CHU	No	56	1	0

1	0	Thoracotoı	265	CHU	No	24	0	0
1	0	Thoracotoı	265	CHU	No	53	0	0
1	0	Thoracotoı	265	CHU	No	46	0	1
1	0	VATS or rc	265	CHU	No	65	1	0
1	0	Thoracotoı	265	CHU	No	81	0	1
1	0	Thoracotoı	265	CHU	No	81	0	1
1	0	VATS or rc	265	CHU	No	80	0	0
1	0	Thoracotoı	265	CHU	No	71	0	1
1	0	Thoracotoı	265	CHU	No	63	0	0
1	0	VATS or rc	265	CHU	No	73	0	0
1	0	Thoracotoı	265	CHU	No	55	0	0
1	0	Thoracotoı	265	CHU	No	56	0	1
1	0	VATS or rc	265	CHU	No	75	0	0
1	0	Thoracotoı	265	CHU	No	62	0	0
1	0	VATS or rc	265	CHU	No	59	0	0
1	0	Thoracotoı	265	CHU	No	80	0	0
1	0	VATS or rc	265	CHU	No	73	0	1
1	0	Thoracotoı	265	CHU	No	56	0	1
1	0	VATS or rc	265	CHU	No	47	0	0
1	0	Thoracotoı	265	CHU	No	63	1	0
1	0	Thoracotoı	265	CHU	No	69	0	1
1	0	VATS or rc	265	CHU	No	54	0	1
1	0	VATS or rc	265	CHU	No	77	0	0
1	0	VATS or rc	265	CHU	No	58	0	0
1	0	VATS or rc	265	CHU	No	63	0	1
1	0	Thoracotoı	265	CHU	No	59	0	1
1	0	Thoracotoı	265	CHU	No	72	0	1
1	1	Thoracotoı	265	CHU	No	59	0	1
1	0	VATS or rc	265	CHU	No	65	0	0
1	0	Thoracotoı	265	CHU	No	55	0	1
1	0	Thoracotoı	265	CHU	No	40	0	1
1	0	Thoracotoı	265	CHU	No	65	1	0
1	0	VATS or rc	265	CHU	No	58	1	0
1	0	Thoracotoı	265	CHU	No	60	0	0
1	0	Thoracotoı	265	CHU	No	55	0	0
1	1	Thoracotoı	265	CHU	No	62	0	0
1	0	Thoracotoı	265	CHU	No	49	0	0
1	0	Thoracotoı	265	CHU	No	24	0	0
1	0	Thoracotoı	265	CHU	No	66	0	0
1	0	VATS or rc	265	CHU	No	35	0	1
1	0	Thoracotoı	265	CHU	No	46	0	1
1	0	Thoracotoı	265	CHU	No	75	0	1
1	0	VATS or rc	265	CHU	No	76	0	0
1	1	Thoracotoı	265	CHU	No	60	0	0
1	0	VATS or rc	265	CHU	No	70	0	0
1	0	Thoracotoı	265	CHU	No	74	0	0
1	0	VATS or rc	265	CHU	No	61	1	1
1	0	Thoracotoı	265	CHU	No	60	1	0
1	0	VATS or rc	265	CHU	No	72	0	0
1	0	Thoracotoı	265	CHU	No	65	0	0
1	0	VATS or rc	265	CHU	No	73	0	0
1	0	Thoracotoı	265	CHU	No	55	0	0
1	0	VATS or rc	265	CHU	No	68	0	0
1	0	VATS or rc	265	CHU	No	68	0	0
1	0	Thoracotoı	265	CHU	No	73	0	0
1	0	Thoracotoı	265	CHU	No	74	0	0

1	0	Thoracotoı	265	CHU	No	77	0	0
1	0	Thoracotoı	265	CHU	No	67	1	0
1	1	Thoracotoı	265	CHU	No	56	1	0
1	1	Thoracotoı	265	CHU	No	67	0	0
1	0	VATS or rc	265	CHU	No	70	0	0
1	0	VATS or rc	265	CHU	No	65	0	0
1	0	VATS or rc	265	CHU	No	58	0	1
1	0	Thoracotoı	265	CHU	No	58	0	1
1	0	Thoracotoı	265	CHU	No	64	0	0
1	0	Thoracotoı	265	CHU	No	66	1	1
1	0	Thoracotoı	265	CHU	No	59	0	1
1	0	VATS or rc	265	CHU	No	66	1	1
1	0	Thoracotoı	265	CHU	No	68	0	1
1	0	Thoracotoı	265	CHU	No	62	0	0
1	0	Thoracotoı	265	CHU	No	60	1	0
1	0	Thoracotoı	265	CHU	No	67	0	0
1	0	VATS or rc	265	CHU	No	62	1	0
1	0	Thoracotoı	265	CHU	No	51	0	1
1	0	Thoracotoı	265	CHU	No	60	1	0
1	0	Thoracotoı	265	CHU	No	56	0	1
1	0	VATS or rc	265	CHU	No	77	0	0
1	0	Thoracotoı	265	CHU	No	75	0	1
1	0	VATS or rc	265	CHU	No	68	0	0
1	1	Thoracotoı	265	CHU	No	53	0	0
1	0	VATS or rc	265	CHU	No	69	0	1
1	1	Thoracotoı	265	CHU	No	38	0	1
1	0	Thoracotoı	265	CHU	No	63	0	0
1	0	Thoracotoı	265	CHU	No	73	0	0
1	0	Thoracotoı	265	CHU	No	53	0	0
1	0	Thoracotoı	265	CHU	No	47	0	1
1	0	Thoracotoı	265	CHU	No	48	0	0
1	1	Thoracotoı	265	CHU	No	59	0	0
1	0	VATS or rc	265	CHU	No	57	1	0
1	1	Thoracotoı	265	CHU	No	36	0	0
1	1	Thoracotoı	265	CHU	Yes	62	0	0
1	0	Thoracotoı	265	CHU	No	54	0	0
1	0	VATS or rc	265	CHU	No	78	0	1
1	1	Thoracotoı	265	CHU	Yes	60	0	1
1	0	VATS or rc	265	CHU	No	69	0	0
1	0	VATS or rc	265	CHU	No	63	0	0
1	0	Thoracotoı	265	CHU	No	46	0	1
1	0	VATS or rc	265	CHU	No	56	0	1
1	0	Thoracotoı	265	CHU	No	71	0	1
1	0	Thoracotoı	265	CHU	No	60	1	0
1	0	Thoracotoı	265	CHU	No	62	0	0
1	1	Thoracotoı	265	CHU	No	67	0	0
1	0	VATS or rc	265	CHU	No	61	0	1
1	0	Thoracotoı	265	CHU	No	79	0	0
1	0	Thoracotoı	265	CHU	No	53	0	0
1	0	Thoracotoı	265	CHU	No	85	0	0
1	0	VATS or rc	265	CHU	No	65	0	0
1	0	Thoracotoı	265	CHU	No	69	0	1
1	0	Thoracotoı	265	CHU	No	46	0	0
1	0	Thoracotoı	265	CHU	No	44	0	1
1	0	VATS or rc	265	CHU	No	73	0	0
1	0	Thoracotoı	265	CHU	No	56	0	0

1	0	Thoracotoı	265	CHU	No	67	0	1
1	0	VATS or rc	265	CHU	No	61	0	1
1	0	VATS or rc	265	CHU	No	76	0	1
1	0	VATS or rc	265	CHU	No	64	0	0
1	0	Thoracotoı	265	CHU	No	44	0	1
1	0	VATS or rc	265	CHU	No	60	0	0
1	1	Thoracotoı	265	CHU	No	68	0	1
1	0	VATS or rc	265	CHU	No	71	0	0
1	0	Thoracotoı	265	CHU	No	57	0	1
1	0	Thoracotoı	265	CHU	No	59	1	1
1	0	Thoracotoı	265	CHU	No	67	0	0
1	0	VATS or rc	265	CHU	No	60	0	1
1	0	Thoracotoı	265	CHU	No	71	0	0
1	0	VATS or rc	265	CHU	No	71	0	1
1	0	VATS or rc	265	CHU	No	76	1	0
1	0	Thoracotoı	265	CHU	No	81	0	1
1	0	Thoracotoı	265	CHU	No	75	0	0
1	0	Thoracotoı	265	CHU	No	56	1	0
1	0	VATS or rc	265	CHU	No	64	0	1
1	0	Thoracotoı	265	CHU	No	61	0	1
1	0	VATS or rc	265	CHU	No	52	0	1
1	0	Thoracotoı	265	CHU	No	69	0	1
1	0	VATS or rc	265	CHU	No	72	1	0
1	0	VATS or rc	265	CHU	No	69	0	0
1	0	Thoracotoı	265	CHU	No	62	0	0
1	0	Thoracotoı	265	CHU	No	51	0	0
1	0	Thoracotoı	265	CHU	No	32	0	1
1	0	VATS or rc	265	CHU	No	70	0	0
1	0	Thoracotoı	265	CHU	No	58	1	0
1	0	Thoracotoı	265	CHU	No	51	0	0
1	0	VATS or rc	265	CHU	No	47	0	0
1	0	Thoracotoı	265	CHU	No	54	0	0
1	0	Thoracotoı	265	CHU	No	61	0	0
1	0	Thoracotoı	265	CHU	No	50	0	1
1	0	Thoracotoı	265	CHU	No	75	0	0
1	0	Thoracotoı	265	CHU	No	63	0	1
1	0	Thoracotoı	265	CHU	No	66	0	0
1	0	Thoracotoı	265	CHU	No	69	0	1
1	0	Thoracotoı	265	CHU	No	47	0	1
1	0	Thoracotoı	265	CHU	No	67	0	0
1	0	Thoracotoı	265	CHU	No	70	0	0
1	0	Thoracotoı	265	CHU	No	52	0	0
1	0	Thoracotoı	265	CHU	No	64	1	1
1	1	Thoracotoı	265	CHU	No	66	0	0
1	0	Thoracotoı	265	CHU	No	77	0	1
1	0	Thoracotoı	265	CHU	No	72	0	0
1	0	Thoracotoı	265	CHU	No	70	0	0
1	0	VATS or rc	265	CHU	No	27	0	0
1	1	Thoracotoı	265	CHU	No	49	0	0
1	0	Thoracotoı	265	CHU	No	75	0	1
1	0	VATS or rc	265	CHU	No	59	0	1
1	0	Thoracotoı	265	CHU	No	59	0	0
1	0	Thoracotoı	265	CHU	No	78	0	0
1	0	Thoracotoı	265	CHU	No	61	0	0
1	0	Thoracotoı	265	CHU	No	78	0	0
1	0	Thoracotoı	265	CHU	No	61	0	1

1	0	Thoracotoı	265	CHU	No	68	0	1
1	0	VATS or rc	265	CHU	No	75	0	1
1	0	Thoracotoı	265	CHU	No	33	0	0
1	0	Thoracotoı	265	CHU	No	70	0	0
1	0	VATS or rc	265	CHU	No	70	0	1
1	0	VATS or rc	265	CHU	No	75	0	0
1	0	Thoracotoı	265	CHU	No	48	0	0
1	0	Thoracotoı	265	CHU	No	62	0	0
1	0	Thoracotoı	265	CHU	No	74	0	0
1	0	VATS or rc	265	CHU	No	71	0	0
1	0	Thoracotoı	265	CHU	No	74	0	0
1	1	Thoracotoı	265	CHU	No	65	0	0
1	0	Thoracotoı	265	CHU	No	56	0	0
1	0	Thoracotoı	265	CHU	No	61	0	1
1	1	Thoracotoı	265	CHU	No	74	0	0
1	0	Thoracotoı	265	CHU	No	65	0	0
1	1	Thoracotoı	265	CHU	No	67	0	0
1	0	Thoracotoı	265	CHU	No	34	0	1
1	0	VATS or rc	265	CHU	No	55	0	0
1	0	Thoracotoı	265	CHU	Yes	57	0	1
1	0	Thoracotoı	265	CHU	No	74	0	0
1	0	Thoracotoı	265	CHU	No	73	0	0
1	0	Thoracotoı	265	CHU	No	57	0	0
1	0	VATS or rc	265	CHU	No	68	0	0
1	0	Thoracotoı	265	CHU	No	68	0	0
1	0	Thoracotoı	265	CHU	No	62	0	0
1	0	Thoracotoı	265	CHU	No	63	0	0
1	0	Thoracotoı	265	CHU	No	64	0	0
1	0	Thoracotoı	265	CHU	No	76	0	0
1	0	Thoracotoı	265	CHU	No	64	0	1
1	0	VATS or rc	265	CHU	No	71	0	1
1	0	Thoracotoı	265	CHU	No	16	0	0
1	1	Thoracotoı	265	CHU	Yes	72	0	0
1	0	Thoracotoı	265	CHU	No	72	0	1
1	0	Thoracotoı	265	CHU	No	65	1	0
1	0	VATS or rc	265	CHU	No	58	0	0
1	0	Thoracotoı	265	CHU	No	74	0	0
1	0	Thoracotoı	265	CHU	No	61	0	0
1	0	Thoracotoı	265	CHU	No	63	1	0
1	0	Thoracotoı	265	CHU	No	75	0	1
1	0	Thoracotoı	265	CHU	No	77	0	1
1	1	Thoracotoı	265	CHU	No	71	0	0
1	0	Thoracotoı	265	CHU	No	75	0	0
1	0	Thoracotoı	265	CHU	No	60	0	1
1	0	Thoracotoı	265	CHU	No	68	1	1
1	0	Thoracotoı	265	CHU	Yes	83	0	0
1	0	Thoracotoı	265	CHU	No	50	0	0
1	0	Thoracotoı	265	CHU	No	74	0	0
1	0	Thoracotoı	265	CHU	No	58	0	0
1	0	VATS or rc	265	CHU	No	38	0	1
1	0	VATS or rc	265	CHU	No	77	0	0
1	0	Thoracotoı	265	CHU	No	47	0	0
1	0	Thoracotoı	265	CHU	No	64	0	1
1	0	Thoracotoı	265	CHU	No	52	0	1
1	0	Thoracotoı	265	CHU	No	69	0	0
1	0	Thoracotoı	265	CHU	No	72	1	1

1	0	Thoracotoı	265	CHU	No	69	0	0
1	0	Thoracotoı	265	CHU	No	58	0	0
1	0	Thoracotoı	265	CHU	No	55	0	1
1	0	Thoracotoı	265	CHU	No	54	0	0
1	0	Thoracotoı	265	CHU	No	55	1	0
1	0	VATS or rc	265	CHU	No	67	0	0
1	0	VATS or rc	265	CHU	No	57	1	1
1	0	Thoracotoı	265	CHU	No	75	0	0
1	0	VATS or rc	265	CHU	No	58	0	0
1	0	Thoracotoı	265	CHU	No	59	0	0
1	0	VATS or rc	265	CHU	No	56	0	0
1	0	Thoracotoı	265	CHU	No	73	0	0
1	1	Thoracotoı	265	CHU	No	77	0	1
1	0	Thoracotoı	265	CHU	No	55	0	0
1	0	Thoracotoı	265	CHU	No	72	0	0
1	0	Thoracotoı	265	CHU	No	53	0	0
1	0	Thoracotoı	265	CHU	No	66	0	0
1	1	Thoracotoı	265	CHU	No	53	0	0
1	0	Thoracotoı	265	CHU	No	72	0	0
1	0	Thoracotoı	265	CHU	No	49	0	0
1	0	VATS or rc	265	CHU	No	58	0	0
1	0	Thoracotoı	265	CHU	No	64	0	0
1	1	Thoracotoı	265	CHU	No	47	0	1
1	0	Thoracotoı	265	CHU	No	58	0	0
1	0	Thoracotoı	265	CHU	No	75	0	0
1	0	Thoracotoı	265	CHU	No	73	0	0
1	0	Thoracotoı	265	CHU	No	64	0	1
1	0	Thoracotoı	265	CHU	No	53	0	0
1	0	Thoracotoı	265	CHU	No	71	1	1
1	0	VATS or rc	265	CHU	No	62	0	0
1	0	VATS or rc	265	CHU	No	60	0	0
1	0	VATS or rc	265	CHU	No	75	0	0
1	0	VATS or rc	265	CHU	No	82	0	0
1	0	Thoracotoı	265	CHU	No	74	1	0
1	0	VATS or rc	265	CHU	No	79	0	0
1	0	Thoracotoı	265	CHU	No	65	0	0
1	0	Thoracotoı	265	CHU	No	70	0	0
1	0	Thoracotoı	265	CHU	No	59	0	0
1	0	Thoracotoı	265	CHU	No	65	0	0
1	0	VATS or rc	265	CHU	No	66	0	0
1	0	VATS or rc	265	CHU	No	45	0	0
1	0	VATS or rc	265	CHU	No	62	0	0
1	0	Thoracotoı	265	CHU	No	75	0	1
1	0	Thoracotoı	265	CHU	No	57	0	1
1	1	Thoracotoı	265	CHU	No	51	0	0
1	0	Thoracotoı	265	CHU	No	69	0	0
1	0	VATS or rc	265	CHU	No	56	0	1
1	0	Thoracotoı	265	CHU	No	77	0	0
1	0	VATS or rc	265	CHU	No	51	0	0
1	0	Thoracotoı	265	CHU	No	55	0	1
1	0	VATS or rc	265	CHU	No	78	0	0
1	0	Thoracotoı	265	CHU	Yes	76	0	0
1	0	VATS or rc	265	CHU	No	52	0	1
1	0	Thoracotoı	265	CHU	No	67	1	0
1	0	VATS or rc	265	CHU	No	60	1	0
1	0	VATS or rc	265	CHU	No	64	1	1

1	0	VATS or rc	265	CHU	No	64	0	1
1	0	VATS or rc	265	CHU	No	72	0	0
1	0	Thoracotoı	265	CHU	No	77	0	0
1	0	Thoracotoı	265	CHU	No	69	1	1
1	0	Thoracotoı	265	CHU	No	70	0	0
1	0	Thoracotoı	265	CHU	No	63	0	0
1	1	Thoracotoı	265	CHU	No	66	0	0
1	0	Thoracotoı	265	CHU	No	58	0	0
1	0	Thoracotoı	265	CHU	No	77	1	0
1	0	Thoracotoı	265	CHU	No	72	0	0
1	0	Thoracotoı	265	CHU	No	52	0	0
1	0	VATS or rc	265	CHU	No	72	1	1
1	0	Thoracotoı	265	CHU	No	57	0	1
1	0	VATS or rc	265	CHU	No	58	0	1
1	0	Thoracotoı	265	CHU	No	62	0	0
1	1	Thoracotoı	265	CHU	No	75	0	0
1	0	Thoracotoı	265	CHU	No	64	0	1
1	0	Thoracotoı	265	CHU	No	67	0	0
1	0	Thoracotoı	265	CHU	No	42	0	0
1	0	Thoracotoı	265	CHU	No	74	0	0
1	0	Thoracotoı	265	CHU	No	68	0	0
1	1	Thoracotoı	265	CHU	No	72	0	0
1	0	VATS or rc	265	CHU	No	80	1	1
1	0	Thoracotoı	265	CHU	No	33	0	1
1	0	Thoracotoı	265	CHU	No	65	0	0
1	0	Thoracotoı	265	CHU	No	51	0	1
1	0	Thoracotoı	265	CHU	No	56	0	0
1	0	Thoracotoı	265	CHU	Yes	69	0	0
1	0	Thoracotoı	265	CHU	No	82	1	0
1	0	Thoracotoı	265	CHU	No	81	0	1
1	1	Thoracotoı	265	CHU	No	72	0	1
1	0	Thoracotoı	265	CHU	No	64	0	1
1	0	Thoracotoı	265	CHU	No	74	0	1
1	0	VATS or rc	265	CHU	No	65	0	0
1	1	Thoracotoı	265	CHU	No	61	0	0
1	0	VATS or rc	265	CHU	No	69	0	1
1	0	VATS or rc	265	CHU	No	58	0	0
1	0	VATS or rc	265	CHU	No	57	0	1
1	1	Thoracotoı	265	CHU	No	57	0	0
1	0	Thoracotoı	265	CHU	No	64	0	1
1	0	Thoracotoı	265	CHU	No	57	0	0
1	0	Thoracotoı	265	CHU	No	60	1	0
1	0	Thoracotoı	265	CHU	No	60	1	0
1	0	VATS or rc	265	CHU	No	72	0	0
1	0	Thoracotoı	265	CHU	No	72	0	1
1	0	Thoracotoı	265	CHU	No	74	0	0
1	0	VATS or rc	265	CHU	No	75	0	0
1	0	Thoracotoı	265	CHU	No	49	0	0
1	0	Thoracotoı	265	CHU	No	68	0	0
1	0	Thoracotoı	265	CHU	No	75	0	0
1	0	VATS or rc	265	CHU	No	55	0	1
1	0	VATS or rc	265	CHU	No	76	0	1
1	0	Thoracotoı	265	CHU	No	65	0	1
1	0	Thoracotoı	265	CHU	No	59	0	0
1	0	Thoracotoı	265	CHU	No	62	0	0
1	0	Thoracotoı	265	CHU	No	54	0	0

1	0	VATS or rc	265	CHU	No	49	0	0
1	0	Thoracotoı	265	CHU	No	57	1	1
1	0	Thoracotoı	265	CHU	No	58	0	1
1	0	Thoracotoı	265	CHU	No	63	0	0
1	0	Thoracotoı	265	CHU	No	64	0	0
1	0	VATS or rc	265	CHU	No	66	0	0
1	0	Thoracotoı	265	CHU	No	62	0	0
1	0	Thoracotoı	265	CHU	No	67	0	0
1	0	Thoracotoı	265	CHU	No	46	0	1
1	0	VATS or rc	265	CHU	No	48	0	0
1	0	Thoracotoı	265	CHU	No	64	0	0
1	0	Thoracotoı	265	CHU	No	47	0	0
1	0	Thoracotoı	265	CHU	No	80	0	1
1	0	Thoracotoı	265	CHU	No	53	0	0
1	0	Thoracotoı	265	CHU	No	77	0	1
1	0	Thoracotoı	265	CHU	No	61	0	0
1	1	Thoracotoı	265	CHU	No	58	0	0
1	0	VATS or rc	265	CHU	No	81	0	0
1	0	Thoracotoı	265	CHU	No	74	0	0
1	0	Thoracotoı	265	CHU	No	72	0	0
1	1	Thoracotoı	265	CHU	No	52	0	1
1	0	VATS or rc	265	CHU	No	58	0	1
1	0	VATS or rc	265	CHU	No	61	0	0
1	0	VATS or rc	265	CHU	No	66	0	0
1	0	Thoracotoı	265	CHU	No	41	0	0
1	0	VATS or rc	265	CHU	No	72	0	0
1	0	Thoracotoı	265	CHU	No	55	0	1
1	0	Thoracotoı	265	CHU	No	71	0	0
1	0	Thoracotoı	265	CHU	No	62	0	0
1	0	Thoracotoı	265	CHU	No	64	0	0
1	0	Thoracotoı	265	CHU	No	54	0	0
1	0	Thoracotoı	265	CHU	No	23	0	0
1	0	Thoracotoı	265	CHU	No	65	0	0
1	0	Thoracotoı	265	CHU	No	72	1	0
1	1	Thoracotoı	265	CHU	No	77	1	0
1	0	VATS or rc	265	CHU	No	64	0	0
1	0	VATS or rc	265	CHU	No	74	0	0
1	0	VATS or rc	265	CHU	No	62	0	0
1	0	VATS or rc	265	CHU	No	72	1	1
1	0	Thoracotoı	265	CHU	No	69	0	1
1	0	Thoracotoı	265	CHU	No	64	0	0
1	0	Thoracotoı	265	CHU	No	77	0	1
1	0	VATS or rc	265	CHU	No	45	0	0
1	0	VATS or rc	265	CHU	No	71	0	0
1	0	Thoracotoı	265	CHU	No	61	0	1
1	0	VATS or rc	265	CHU	No	73	0	0
1	0	Thoracotoı	265	CHU	No	35	0	0
1	1	Thoracotoı	265	CHU	No	59	0	0
1	0	Thoracotoı	265	CHU	No	69	0	0
1	0	Thoracotoı	265	CHU	No	53	0	1
1	0	Thoracotoı	265	CHU	No	77	0	0
1	0	Thoracotoı	265	CHU	No	39	0	0
1	0	Thoracotoı	265	CHU	No	61	0	0
1	0	Thoracotoı	265	CHU	No	70	0	0
1	0	Thoracotoı	265	CHU	Yes	84	0	0
1	0	Thoracotoı	265	CHU	No	58	0	0

1	0	Thoracotoı	265	CHU	No	79	0	0
1	0	Thoracotoı	265	CHU	No	52	0	1
1	0	VATS or rc	265	CHU	No	78	0	1
1	0	Thoracotoı	265	CHU	No	59	0	0
1	0	VATS or rc	265	CHU	Yes	71	1	0
1	0	VATS or rc	265	CHU	No	72	1	0
1	0	Thoracotoı	265	CHU	No	51	0	0
1	0	Thoracotoı	265	CHU	No	61	0	0
1	0	VATS or rc	265	CHU	No	62	0	1
1	0	VATS or rc	265	CHU	No	51	0	1
1	0	Thoracotoı	265	CHU	No	62	0	1
1	0	Thoracotoı	265	CHU	No	63	0	1
1	0	Thoracotoı	265	CHU	No	59	0	1
1	0	VATS or rc	265	CHU	No	57	0	1
1	0	Thoracotoı	265	CHU	No	66	0	0
1	0	VATS or rc	265	CHU	No	67	0	1
1	0	Thoracotoı	265	CHU	No	58	0	0
1	0	VATS or rc	265	CHU	No	69	0	0
1	0	Thoracotoı	265	CHU	No	52	0	0
1	0	Thoracotoı	265	CHU	No	65	0	0
1	0	VATS or rc	265	CHU	No	20	0	0
1	0	VATS or rc	265	CHU	No	66	0	0
1	0	Thoracotoı	265	CHU	No	82	0	1
1	0	Thoracotoı	265	CHU	No	78	0	1
1	0	VATS or rc	265	CHU	No	63	0	0
1	1	Thoracotoı	265	CHU	No	57	0	1
1	0	Thoracotoı	265	CHU	No	73	0	0
1	0	Thoracotoı	265	CHU	No	68	0	0
1	0	VATS or rc	265	CHU	No	58	0	0
1	0	VATS or rc	265	CHU	No	72	0	1
1	0	VATS or rc	265	CHU	No	68	0	0
1	0	Thoracotoı	265	CHU	No	57	0	0
1	0	Thoracotoı	265	CHU	No	72	0	0
1	0	Thoracotoı	265	CHU	No	81	0	0
1	1	Thoracotoı	265	CHU	No	63	0	0
1	0	Thoracotoı	265	CHU	No	67	0	0
1	0	Thoracotoı	265	CHU	No	70	0	0
1	0	Thoracotoı	265	CHU	No	58	0	0
1	0	Thoracotoı	265	CHU	No	56	0	0
1	0	Thoracotoı	265	CHU	No	55	0	1
1	0	Thoracotoı	265	CHU	No	71	0	0
1	0	Thoracotoı	265	CHU	No	67	0	1
1	1	Thoracotoı	265	CHU	No	62	0	0
1	0	Thoracotoı	265	CHU	No	82	0	0
1	0	VATS or rc	265	CHU	No	49	1	0
1	0	Thoracotoı	265	CHU	No	76	0	0
1	0	Thoracotoı	265	CHU	No	60	0	0
1	1	Thoracotoı	265	CHU	No	50	0	0
1	0	VATS or rc	265	CHU	No	69	0	0
1	0	Thoracotoı	265	CHU	No	56	0	1
1	0	VATS or rc	265	CHU	No	59	0	0
1	0	VATS or rc	265	CHU	No	73	1	1
1	0	Thoracotoı	265	CHU	No	57	0	0
1	0	Thoracotoı	265	CHU	No	78	0	0
1	1	Thoracotoı	265	CHU	No	17	0	0
1	0	VATS or rc	265	CHU	No	72	0	0

1	0	Thoracotoı	265	CHU	No	54	0	0
1	0	Thoracotoı	265	CHU	No	59	0	1
1	0	Thoracotoı	265	CHU	No	37	0	1
1	0	Thoracotoı	265	CHU	No	63	0	0
1	0	Thoracotoı	265	CHU	No	62	0	0
1	0	Thoracotoı	265	CHU	No	75	0	1
1	1	Thoracotoı	265	CHU	No	73	0	0
1	0	VATS or rc	265	CHU	No	73	0	0
1	0	Thoracotoı	265	CHU	No	63	0	0
1	0	Thoracotoı	265	CHU	No	80	0	0
1	0	Thoracotoı	265	CHU	No	65	0	0
1	0	Thoracotoı	265	CHU	No	75	0	0
1	0	VATS or rc	265	CHU	No	71	0	0
1	0	Thoracotoı	265	CHU	No	73	0	1
1	0	Thoracotoı	265	CHU	No	61	0	0
1	0	Thoracotoı	265	CHU	No	62	0	1
1	0	VATS or rc	265	CHU	No	60	0	0
1	0	VATS or rc	265	CHU	No	55	0	0
1	0	Thoracotoı	265	CHU	No	61	0	0
1	0	Thoracotoı	265	CHU	No	64	0	0
1	1	Thoracotoı	265	CHU	No	65	1	0
1	0	VATS or rc	265	CHU	No	62	0	0
1	0	Thoracotoı	265	CHU	No	73	0	0
1	0	Thoracotoı	265	CHU	No	43	0	0
1	0	Thoracotoı	265	CHU	No	58	0	0
1	0	VATS or rc	265	CHU	No	57	0	0
1	0	Thoracotoı	265	CHU	No	68	0	0
1	0	Thoracotoı	265	CHU	No	71	0	0
1	0	VATS or rc	265	CHU	No	64	0	1
1	1	Thoracotoı	265	CHU	No	57	0	0
1	0	Thoracotoı	265	CHU	No	50	0	0
1	0	Thoracotoı	265	CHU	No	76	0	0
1	0	Thoracotoı	265	CHU	No	42	0	0
1	0	Thoracotoı	265	CHU	No	72	0	0
1	0	Thoracotoı	265	CHU	No	61	0	0
1	0	Thoracotoı	265	CHU	No	47	0	0
1	0	VATS or rc	265	CHU	No	65	0	0
1	0	Thoracotoı	265	CHU	No	49	0	1
1	0	Thoracotoı	265	CHU	No	60	0	0
1	0	VATS or rc	265	CHU	No	68	1	1
1	0	Thoracotoı	265	CHU	No	62	0	0
1	0	Thoracotoı	265	CHU	No	46	0	0
1	0	VATS or rc	265	CHU	No	63	0	0
1	0	Thoracotoı	265	CHU	No	51	0	1
1	0	VATS or rc	265	CHU	No	72	1	1
1	0	Thoracotoı	265	CHU	No	74	0	0
1	0	VATS or rc	265	CHU	No	59	0	0
1	0	Thoracotoı	265	CHU	No	64	0	1
1	0	Thoracotoı	265	CHU	Yes	74	0	1
1	0	Thoracotoı	265	CHU	No	62	0	0
1	0	Thoracotoı	265	CHU	Yes	67	0	0
1	0	Thoracotoı	265	CHU	No	75	0	0
1	0	Thoracotoı	265	CHU	No	68	0	1
1	0	Thoracotoı	265	CHU	No	61	0	0
1	0	VATS or rc	265	CHU	No	79	0	0
1	0	Thoracotoı	265	CHU	No	55	0	1

1	0	Thoracotoı	265	CHU	No	71	0	0
1	0	VATS or rc	265	CHU	No	69	0	0
1	0	Thoracotoı	265	CHU	No	73	1	1
1	1	Thoracotoı	265	CHU	No	42	0	0
1	0	Thoracotoı	265	CHU	No	74	0	0
1	0	Thoracotoı	265	CHU	No	76	0	0
1	0	Thoracotoı	265	CHU	No	67	0	0
1	0	Thoracotoı	265	CHU	No	83	0	0
1	0	Thoracotoı	265	CHU	No	68	0	0
1	0	VATS or rc	265	CHU	No	55	0	1
1	0	Thoracotoı	265	CHU	No	63	0	0
1	0	Thoracotoı	265	CHU	No	83	0	0
1	0	VATS or rc	265	CHU	Yes	81	1	1
1	0	Thoracotoı	265	CHU	No	56	0	1
1	0	Thoracotoı	265	CHU	No	76	0	0
1	0	Thoracotoı	265	CHU	No	41	0	1
1	0	Thoracotoı	265	CHU	No	58	1	0
1	0	Thoracotoı	265	CHU	No	59	0	1
1	1	Thoracotoı	265	CHU	No	60	0	0
1	0	VATS or rc	265	CHU	No	69	0	0
1	0	VATS or rc	265	CHU	No	79	0	0
1	0	Thoracotoı	265	CHU	No	59	0	0
1	0	Thoracotoı	265	CHU	No	70	1	1
1	0	Thoracotoı	265	CHU	No	63	0	0
1	0	VATS or rc	265	CHU	No	69	0	1
1	0	Thoracotoı	265	CHU	No	65	1	1
1	0	VATS or rc	265	CHU	No	65	0	0
1	0	Thoracotoı	265	CHU	No	52	0	0
1	0	VATS or rc	265	CHU	No	45	0	0
1	0	Thoracotoı	265	CHU	No	58	0	0
1	0	VATS or rc	265	CHU	No	76	0	1
1	0	Thoracotoı	265	CHU	No	61	0	0
1	0	Thoracotoı	265	CHU	No	70	0	0
1	0	Thoracotoı	265	CHU	No	56	0	0
1	1	Thoracotoı	265	CHU	No	65	0	0
1	0	Thoracotoı	265	CHU	No	67	1	0
1	0	Thoracotoı	265	CHU	No	55	0	0
1	0	Thoracotoı	265	CHU	No	52	0	1
1	0	Thoracotoı	265	CHU	No	62	0	1
1	0	Thoracotoı	265	CHU	No	64	0	0
1	0	Thoracotoı	265	CHU	No	59	0	1
1	0	Thoracotoı	265	CHU	No	53	0	1
1	0	VATS or rc	265	CHU	No	58	0	1
1	0	Thoracotoı	265	CHU	No	75	0	1
1	0	Thoracotoı	265	CHU	No	61	1	0
1	0	VATS or rc	265	CHU	No	62	0	1
1	0	Thoracotoı	265	CHU	No	81	0	0
1	1	Thoracotoı	265	CHU	No	63	0	1
1	0	VATS or rc	265	CHU	No	45	0	0
1	1	Thoracotoı	265	CHU	No	72	0	0
1	0	VATS or rc	265	CHU	No	71	0	1
1	0	Thoracotoı	265	CHU	No	71	0	0
1	0	Thoracotoı	265	CHU	No	61	1	0
1	0	Thoracotoı	265	CHU	No	60	0	1
1	0	Thoracotoı	265	CHU	No	63	0	0
1	0	Thoracotoı	265	CHU	No	72	0	0

1	0	Thoracotoı	265	CHU	No	70	0	1
1	0	Thoracotoı	265	CHU	No	61	0	1
1	0	Thoracotoı	265	CHU	No	55	0	1
1	0	Thoracotoı	265	CHU	No	73	0	0
1	0	VATS or rc	265	CHU	No	70	0	0
1	1	Thoracotoı	265	CHU	No	60	0	1
1	0	Thoracotoı	265	CHU	No	66	0	0
1	0	Thoracotoı	265	CHU	No	61	0	0
1	0	Thoracotoı	265	CHU	No	52	0	0
1	0	Thoracotoı	265	CHU	No	57	0	0
1	0	VATS or rc	265	CHU	No	76	0	1
1	0	VATS or rc	265	CHU	No	66	0	1
1	0	Thoracotoı	265	CHU	No	75	0	0
1	1	Thoracotoı	265	CHU	No	61	1	1
1	0	Thoracotoı	265	CHU	No	59	0	0
1	0	Thoracotoı	265	CHU	No	65	0	0
1	0	Thoracotoı	265	CHU	No	76	0	0
1	0	VATS or rc	265	CHU	No	41	0	1
1	0	Thoracotoı	265	CHU	No	67	0	1
1	0	VATS or rc	265	CHU	No	81	0	0
1	0	VATS or rc	265	CHU	No	60	0	0
1	1	Thoracotoı	265	CHU	No	74	0	0
1	0	Thoracotoı	265	CHU	No	46	0	0
1	0	Thoracotoı	265	CHU	No	57	0	1
1	0	Thoracotoı	265	CHU	No	30	0	0
1	0	Thoracotoı	265	CHU	No	40	0	0
1	0	Thoracotoı	265	CHU	No	67	0	0
1	0	Thoracotoı	265	CHU	No	71	0	0
1	0	Thoracotoı	265	CHU	No	59	1	0
1	0	Thoracotoı	265	CHU	No	74	0	0
1	0	Thoracotoı	265	CHU	No	77	0	0
1	0	Thoracotoı	265	CHU	No	74	1	0
1	0	Thoracotoı	265	CHU	No	68	0	1
1	0	VATS or rc	265	CHU	No	71	0	1
1	0	VATS or rc	265	CHU	No	64	1	0
1	0	VATS or rc	265	CHU	No	74	0	1
1	0	Thoracotoı	265	CHU	No	64	0	0
1	0	Thoracotoı	265	CHU	No	77	0	0
1	1	Thoracotoı	265	CHU	No	65	0	0
1	1	Thoracotoı	265	CHU	No	61	0	0
1	0	Thoracotoı	265	CHU	No	70	0	0
1	0	VATS or rc	265	CHU	No	74	0	1
1	0	VATS or rc	265	CHU	No	62	0	1
1	0	Thoracotoı	265	CHU	No	69	0	1
1	0	Thoracotoı	265	CHU	No	53	0	1
1	0	Thoracotoı	265	CHU	No	64	0	0
1	0	Thoracotoı	265	CHU	No	63	0	0
1	0	Thoracotoı	265	CHU	No	66	0	0
1	0	Thoracotoı	265	CHU	No	49	0	0
1	0	VATS or rc	265	CHU	No	65	0	0
1	1	Thoracotoı	265	CHU	Yes	57	0	0
1	0	Thoracotoı	265	CHU	No	67	0	1
1	0	VATS or rc	265	CHU	No	45	0	0
1	0	Thoracotoı	265	CHU	No	61	0	1
1	0	Thoracotoı	265	CHU	No	72	0	0
1	0	Thoracotoı	265	CHU	No	81	1	0

1	0	VATS or rc	265	CHU	No	58	0	0
1	1	Thoracotoı	265	CHU	No	37	0	0
1	0	VATS or rc	265	CHU	No	40	0	1
1	0	Thoracotoı	265	CHU	No	59	0	0
1	0	Thoracotoı	265	CHU	No	71	0	0
1	0	Thoracotoı	265	CHU	No	65	0	0
1	0	VATS or rc	265	CHU	No	70	0	0
1	0	Thoracotoı	265	CHU	No	68	0	0
1	0	Thoracotoı	265	CHU	No	60	0	0
1	0	Thoracotoı	265	CHU	No	67	0	0
1	0	Thoracotoı	265	CHU	No	58	0	1
1	0	Thoracotoı	265	CHU	No	50	0	0
1	0	VATS or rc	265	CHU	No	73	1	1
1	0	Thoracotoı	265	CHU	No	48	0	1
1	0	VATS or rc	265	CHU	No	82	0	1
1	0	Thoracotoı	265	CHU	No	63	0	0
1	0	Thoracotoı	265	CHU	No	58	0	1
1	0	VATS or rc	265	CHU	No	46	0	1
1	0	Thoracotoı	265	CHU	No	66	0	0
1	0	Thoracotoı	265	CHU	No	67	0	1
1	0	VATS or rc	265	CHU	No	57	0	1
1	0	Thoracotoı	265	CHU	No	56	0	1
1	1	Thoracotoı	265	CHU	No	69	0	0
1	0	VATS or rc	265	CHU	No	56	1	0
1	0	Thoracotoı	265	CHU	No	74	0	0
1	0	Thoracotoı	265	CHU	No	67	0	0
1	1	Thoracotoı	265	CHU	No	80	0	0
1	0	Thoracotoı	265	CHU	No	62	0	0
1	0	Thoracotoı	265	CHU	No	57	0	0
1	0	Thoracotoı	265	CHU	No	67	0	0
1	0	Thoracotoı	265	CHU	No	53	0	1
1	1	Thoracotoı	265	CHU	Yes	56	0	0
1	0	Thoracotoı	265	CHU	No	59	1	1
1	0	Thoracotoı	265	CHU	No	63	0	0
1	0	Thoracotoı	265	CHU	No	67	0	0
1	0	VATS or rc	265	CHU	No	55	0	1
1	0	VATS or rc	265	CHU	No	49	0	0
1	0	Thoracotoı	265	CHU	No	54	0	0
1	1	Thoracotoı	265	CHU	No	74	0	1
1	0	Thoracotoı	265	CHU	No	65	0	0
1	0	Thoracotoı	265	CHU	No	72	0	0
1	0	Thoracotoı	265	CHU	No	65	0	1
1	0	Thoracotoı	265	CHU	No	53	0	1
1	0	VATS or rc	265	CHU	No	43	0	0
1	0	Thoracotoı	265	CHU	No	77	0	0
1	0	Thoracotoı	265	CHU	No	54	0	0
1	1	Thoracotoı	265	CHU	No	78	0	0
1	0	Thoracotoı	265	CHU	No	49	0	1
1	0	VATS or rc	265	CHU	No	64	0	1
1	1	Thoracotoı	265	CHU	No	66	0	1
1	1	Thoracotoı	265	CHU	No	51	0	0
1	0	VATS or rc	265	CHU	No	68	0	1
1	0	Thoracotoı	265	CHU	No	72	0	0
1	0	VATS or rc	265	CHU	No	62	1	0
1	1	Thoracotoı	265	CHU	No	64	0	0
1	1	Thoracotoı	265	CHU	No	70	0	1

1	0	Thoracotoı	265	CHU	No	63	0	0
1	0	Thoracotoı	265	CHU	No	62	0	0
1	0	Thoracotoı	265	CHU	No	54	0	0
1	0	Thoracotoı	265	CHU	No	67	0	1
1	0	Thoracotoı	265	CHU	Yes	62	0	0
1	0	Thoracotoı	265	CHU	No	70	0	0
1	0	Thoracotoı	265	CHU	No	63	0	0
1	0	VATS or rc	265	CHU	No	62	0	0
1	0	Thoracotoı	265	CHU	No	42	0	0
1	0	VATS or rc	265	CHU	No	73	0	0
1	0	Thoracotoı	265	CHU	No	51	0	0
1	0	Thoracotoı	265	CHU	No	73	0	0
1	0	VATS or rc	265	CHU	No	60	0	0
1	0	Thoracotoı	265	CHU	No	64	0	1
1	1	Thoracotoı	265	CHU	No	55	0	0
1	0	Thoracotoı	265	CHU	No	64	1	1
1	0	Thoracotoı	265	CHU	No	57	0	0
1	0	VATS or rc	265	CHU	No	76	0	0
1	0	Thoracotoı	265	CHU	No	51	0	0
1	0	Thoracotoı	265	CHU	No	45	0	0
1	0	VATS or rc	265	CHU	No	51	0	0
1	0	Thoracotoı	265	CHU	No	46	0	1
1	1	Thoracotoı	265	CHU	No	60	0	0
1	0	Thoracotoı	265	CHU	No	65	0	0
1	0	Thoracotoı	265	CHU	No	74	0	0
1	0	Thoracotoı	265	CHU	No	58	1	1
1	0	Thoracotoı	265	CHU	No	69	0	0
1	0	Thoracotoı	265	CHU	No	61	0	1
1	0	Thoracotoı	265	CHU	No	52	0	1
1	0	VATS or rc	265	CHU	No	77	0	0
1	1	Thoracotoı	265	CHU	No	48	0	0
1	0	Thoracotoı	265	CHU	No	58	0	1
1	0	VATS or rc	265	CHU	No	58	0	1
1	0	VATS or rc	265	CHU	No	63	1	0
1	0	Thoracotoı	265	CHU	No	45	0	0
1	0	Thoracotoı	265	CHU	No	76	0	0
1	0	Thoracotoı	265	CHU	No	69	0	0
1	0	Thoracotoı	265	CHU	No	39	0	1
1	0	Thoracotoı	265	CHU	No	58	0	1
1	0	Thoracotoı	265	CHU	No	55	0	1
1	0	Thoracotoı	265	CHU	No	63	0	1
1	0	VATS or rc	265	CHU	No	78	0	0
1	0	VATS or rc	265	CHU	No	44	0	1
1	1	Thoracotoı	265	CHU	Yes	70	0	0
1	0	Thoracotoı	265	CHU	No	71	0	0
1	0	VATS or rc	265	CHU	No	59	0	0
1	0	Thoracotoı	265	CHU	No	56	0	0
1	0	Thoracotoı	265	CHU	No	16	0	0
1	0	Thoracotoı	265	CHU	No	58	0	0
1	0	Thoracotoı	265	CHU	No	68	1	0
1	0	Thoracotoı	265	CHU	No	60	0	0
1	0	Thoracotoı	265	CHU	No	55	0	0
1	0	VATS or rc	265	CHU	No	62	0	0
1	1	Thoracotoı	265	CHU	No	62	0	1
1	0	VATS or rc	265	CHU	No	54	0	1
1	0	VATS or rc	265	CHU	No	38	0	1

1	0	Thoracotoı	265	CHU	No	62	0	0
1	0	VATS or rc	265	CHU	No	73	0	0
1	0	Thoracotoı	265	CHU	No	63	0	0
1	0	Thoracotoı	265	CHU	No	67	0	0
1	0	VATS or rc	265	CHU	No	70	0	0
1	0	VATS or rc	265	CHU	No	63	0	0
1	0	Thoracotoı	265	CHU	No	70	0	0
1	0	Thoracotoı	265	CHU	No	69	0	0
1	0	Thoracotoı	265	CHU	No	64	0	1
1	0	Thoracotoı	265	CHU	No	62	0	1
1	0	Thoracotoı	265	CHU	Yes	77	0	1
1	0	VATS or rc	265	CHU	No	62	0	1
1	0	Thoracotoı	265	CHU	No	70	0	0
1	0	Thoracotoı	265	CHU	No	54	0	0
1	0	Thoracotoı	265	CHU	No	58	0	0
1	0	Thoracotoı	265	CHU	No	70	1	1
1	0	VATS or rc	265	CHU	No	56	0	0
1	0	Thoracotoı	265	CHU	No	63	0	0
1	0	VATS or rc	265	CHU	No	59	0	0
1	0	Thoracotoı	265	CHU	No	66	0	1
1	0	VATS or rc	265	CHU	No	75	0	0
1	0	Thoracotoı	265	CHU	No	68	0	1
1	0	Thoracotoı	265	CHU	No	72	0	0
1	0	Thoracotoı	265	CHU	No	58	0	0
1	0	Thoracotoı	265	CHU	No	50	0	0
1	0	VATS or rc	265	CHU	No	65	0	0
1	0	Thoracotoı	265	CHU	No	54	0	0
1	0	Thoracotoı	265	CHU	No	71	0	1
1	0	Thoracotoı	265	CHU	No	79	0	1
1	0	Thoracotoı	265	CHU	No	75	0	0
1	0	Thoracotoı	265	CHU	No	76	0	0
1	0	Thoracotoı	265	CHU	No	51	0	1
1	0	VATS or rc	265	CHU	No	64	0	1
1	0	Thoracotoı	265	CHU	No	73	0	1
1	0	Thoracotoı	265	CHU	No	69	0	0
1	0	Thoracotoı	265	CHU	No	55	0	0
1	0	Thoracotoı	265	CHU	No	59	0	0
1	0	VATS or rc	265	CHU	No	56	0	0
1	0	Thoracotoı	265	CHU	No	79	0	1
1	0	VATS or rc	265	CHU	No	63	0	0
1	0	Thoracotoı	265	CHU	No	60	0	0
1	0	Thoracotoı	265	CHU	No	57	0	1
1	0	Thoracotoı	265	CHU	No	66	0	1
1	0	Thoracotoı	265	CHU	No	58	0	0
1	0	Thoracotoı	265	CHU	No	68	0	1
1	0	Thoracotoı	265	CHU	No	65	0	1
1	0	VATS or rc	265	CHU	No	51	0	0
1	1	Thoracotoı	265	CHU	No	50	0	0
1	0	VATS or rc	265	CHU	No	71	0	0
1	1	VATS or rc	265	CHU	No	56	0	1
1	1	Thoracotoı	265	CHU	No	61	0	0
1	0	Thoracotoı	265	CHU	No	64	0	0
1	0	VATS or rc	265	CHU	No	69	0	0
1	0	Thoracotoı	265	CHU	No	57	0	1
1	0	VATS or rc	265	CHU	No	62	0	0
1	0	VATS or rc	265	CHU	No	54	0	0

1	0	Thoracotoı	265	CHU	No	67	0	1
1	0	VATS or rc	265	CHU	No	73	0	0
1	0	VATS or rc	265	CHU	No	73	0	0
1	1	Thoracotoı	265	CHU	No	75	0	0
1	0	Thoracotoı	265	CHU	No	61	0	1
1	1	Thoracotoı	265	CHU	No	59	0	0
1	0	Thoracotoı	265	CHU	No	67	0	0
1	0	Thoracotoı	265	CHU	No	81	0	1
1	1	Thoracotoı	265	CHU	No	54	0	0
1	0	Thoracotoı	265	CHU	No	74	0	0
1	1	Thoracotoı	265	CHU	No	57	0	0
1	0	Thoracotoı	265	CHU	No	71	0	0
1	0	Thoracotoı	265	CHU	No	71	0	1
1	0	Thoracotoı	265	CHU	No	69	0	0
1	0	Thoracotoı	265	CHU	No	57	0	0
1	0	Thoracotoı	265	CHU	No	75	1	0
1	0	Thoracotoı	265	CHU	No	24	0	0
1	0	Thoracotoı	265	CHU	No	72	0	0
1	0	VATS or rc	265	CHU	No	53	0	1
1	1	Thoracotoı	265	CHU	No	54	0	0
1	0	Thoracotoı	265	CHU	No	51	0	0
1	0	Thoracotoı	265	CHU	No	63	0	1
1	0	Thoracotoı	265	CHU	No	51	0	0
1	0	Thoracotoı	265	CHU	No	48	0	1
1	0	Thoracotoı	265	CHU	No	72	0	0
1	0	Thoracotoı	265	CHU	No	60	0	1
1	0	Thoracotoı	265	CHU	No	52	0	0
1	0	Thoracotoı	265	CHU	No	59	0	0
1	0	Thoracotoı	265	CHU	No	67	0	0
1	0	VATS or rc	265	CHU	No	67	0	0
1	0	VATS or rc	265	CHU	No	58	0	1
1	0	VATS or rc	265	CHU	No	58	0	1
1	0	Thoracotoı	265	CHU	No	27	0	0
1	1	Thoracotoı	265	CHU	No	62	0	0
1	0	Thoracotoı	265	CHU	No	29	0	0
1	0	VATS or rc	265	CHU	No	70	0	1
1	0	Thoracotoı	265	CHU	No	73	1	1
1	0	Thoracotoı	265	CHU	No	35	0	0
1	0	Thoracotoı	265	CHU	No	76	0	0
1	0	Thoracotoı	265	CHU	No	67	0	0
1	0	Thoracotoı	265	CHU	No	82	0	0
1	1	Thoracotoı	265	CHU	No	53	0	1
1	0	Thoracotoı	265	CHU	No	65	0	1
1	0	VATS or rc	265	CHU	No	58	0	0
1	0	Thoracotoı	265	CHU	No	76	0	0
1	0	VATS or rc	265	CHU	No	56	0	1
1	0	Thoracotoı	265	CHU	No	61	0	1
1	0	VATS or rc	265	CHU	No	54	0	1
1	0	Thoracotoı	265	CHU	No	62	0	0
1	0	VATS or rc	265	CHU	No	77	0	0
1	0	VATS or rc	265	CHU	No	78	0	1
1	0	VATS or rc	265	CHU	No	60	0	0
1	0	Thoracotoı	265	CHU	No	65	0	0
1	1	Thoracotoı	265	CHU	No	68	0	0
1	0	VATS or rc	265	CHU	No	63	0	1
1	0	Thoracotoı	265	CHU	No	46	0	1

1	0	VATS or ro	265	CHU	No	51	0	0
1	0	VATS or ro	265	CHU	No	56	0	0
1	0	Thoracotoı	265	CHU	No	65	0	1
1	0	Thoracotoı	265	CHU	No	61	0	0
1	0	Thoracotoı	265	CHU	No	51	0	1
1	0	Thoracotoı	265	CHU	No	71	0	0
1	0	VATS or ro	265	CHU	No	37	0	0
1	0	Thoracotoı	265	CHU	No	44	0	0
1	0	VATS or ro	265	CHU	No	67	0	0
1	0	Thoracotoı	265	CHU	No	70	0	0
1	0	Thoracotoı	265	CHU	No	62	0	0
1	0	Thoracotoı	265	CHU	No	71	0	0
1	0	VATS or ro	265	CHU	No	70	0	1
1	0	Thoracotoı	265	CHU	No	53	0	1
1	0	Thoracotoı	265	CHU	No	51	0	1
1	0	Thoracotoı	265	CHU	No	65	0	0
1	0	Thoracotoı	265	CHU	No	83	0	0
1	0	Thoracotoı	265	CHU	No	51	0	1
1	0	Thoracotoı	265	CHU	No	45	0	1
1	0	VATS or ro	265	CHU	No	45	0	0
1	0	Thoracotoı	265	CHU	No	67	0	1
1	0	Thoracotoı	265	CHU	No	43	0	0
1	0	Thoracotoı	265	CHU	No	63	0	0
1	0	Thoracotoı	265	CHU	No	64	0	1
1	0	VATS or ro	265	CHU	No	60	0	0
1	0	Thoracotoı	265	CHU	No	72	0	0
1	0	VATS or ro	265	CHU	No	82	0	0
1	0	Thoracotoı	265	CHU	No	59	0	0
1	0	Thoracotoı	265	CHU	No	71	0	0
1	1	Thoracotoı	265	CHU	No	40	0	0
1	0	VATS or ro	265	CHU	No	58	0	1
1	0	Thoracotoı	265	CHU	Yes	52	0	1
1	0	Thoracotoı	265	CHU	No	77	0	0
1	0	Thoracotoı	265	CHU	No	76	0	1
1	0	Thoracotoı	265	CHU	No	55	0	1
1	0	Thoracotoı	265	CHU	No	61	1	0
1	1	Thoracotoı	265	CHU	No	65	0	0
1	0	VATS or ro	265	CHU	Yes	75	0	0
1	0	Thoracotoı	265	CHU	No	65	0	0
1	0	VATS or ro	265	CHU	No	41	0	0
1	0	VATS or ro	265	CHU	No	72	1	0
1	0	VATS or ro	265	CHU	No	79	1	1
1	0	Thoracotoı	265	CHU	No	69	0	1
1	0	Thoracotoı	265	CHU	No	52	0	0
1	0	Thoracotoı	265	CHU	No	72	0	1
1	0	VATS or ro	265	CHU	No	72	1	0
1	0	VATS or ro	265	CHU	No	70	0	0
1	0	Thoracotoı	265	CHU	No	60	0	0
1	0	Thoracotoı	265	CHU	No	44	0	1
1	0	Thoracotoı	265	CHU	Yes	48	0	1
1	0	Thoracotoı	265	CHU	No	45	0	1
1	1	Thoracotoı	265	CHU	No	64	0	1
1	0	Thoracotoı	265	CHU	No	66	0	0
1	0	Thoracotoı	265	CHU	Yes	76	0	1
1	0	Thoracotoı	265	CHU	No	46	0	1
1	0	Thoracotoı	265	CHU	No	55	0	1

1	0	VATS or ro	265	CHU	No	73	0	0
1	1	Thoracotor	265	CHU	No	62	0	0
1	0	Thoracotor	265	CHU	No	61	0	0
1	0	VATS or ro	265	CHU	No	57	0	0
1	0	VATS or ro	265	CHU	No	62	0	0
1	0	Thoracotor	265	CHU	No	54	1	0
1	0	Thoracotor	265	CHU	No	63	0	0
1	0	Thoracotor	265	CHU	No	54	0	0
1	0	VATS or ro	265	CHU	No	79	0	0
1	0	Thoracotor	265	CHU	No	64	0	0
1	0	Thoracotor	265	CHU	No	80	0	1
1	0	Thoracotor	265	CHU	Yes	72	0	0
1	0	Thoracotor	265	CHU	No	56	0	0
1	0	VATS or ro	265	CHU	No	67	0	0
1	0	Thoracotor	265	CHU	No	52	0	0
1	0	VATS or ro	265	CHU	No	74	0	0
1	1	Thoracotor	265	CHU	No	61	0	0
1	0	Thoracotor	265	CHU	No	45	0	1
1	0	Thoracotor	265	CHU	No	86	0	0
1	0	Thoracotor	265	CHU	No	78	1	0
1	0	Thoracotor	265	CHU	No	53	0	1
1	0	Thoracotor	265	CHU	No	55	0	1
1	0	Thoracotor	265	CHU	No	83	0	0
1	0	Thoracotor	265	CHU	No	48	0	0
1	0	VATS or ro	265	CHU	Yes	81	0	0
1	0	VATS or ro	265	CHU	No	68	0	0
1	0	Thoracotor	265	CHU	No	46	0	1
1	0	Thoracotor	265	CHU	No	67	1	0
1	0	VATS or ro	265	CHU	No	71	0	1
1	0	Thoracotor	265	CHU	No	61	0	0
1	0	Thoracotor	265	CHU	Yes	69	0	0
1	0	VATS or ro	265	CHU	No	70	0	1
1	0	Thoracotor	265	CHU	No	61	0	0
1	0	Thoracotor	265	CHU	No	59	1	1
1	0	Thoracotor	265	CHU	No	71	0	0
1	1	Thoracotor	265	CHU	No	60	0	0
1	0	Thoracotor	265	CHU	No	57	0	1
1	0	Thoracotor	265	CHU	No	80	0	1
1	0	Thoracotor	265	CHU	No	59	1	0
1	0	VATS or ro	265	CHU	No	52	0	0
1	0	VATS or ro	265	CHU	No	73	0	0
1	0	VATS or ro	265	CHU	No	72	0	1
1	1	Thoracotor	265	CHU	No	45	0	0
1	0	VATS or ro	265	CHU	No	73	1	0
1	0	Thoracotor	265	CHU	No	72	0	0
1	0	Thoracotor	265	CHU	No	76	0	0
1	0	Thoracotor	265	CHU	No	59	0	1
1	0	Thoracotor	265	CHU	No	68	0	1
1	0	VATS or ro	265	CHU	No	63	0	0
1	0	Thoracotor	265	CHU	No	53	0	0
1	0	Thoracotor	265	CHU	No	50	0	0
1	1	Thoracotor	265	CHU	No	74	1	1
1	0	Thoracotor	265	CHU	No	74	0	0
1	0	Thoracotor	265	CHU	No	63	1	0
1	0	Thoracotor	265	CHU	No	77	0	0
1	0	VATS or ro	265	CHU	No	64	0	1

1	0	Thoracotoı	265	CHU	No	60	0	0
1	0	VATS or rc	265	CHU	No	50	0	1
1	0	VATS or rc	265	CHU	Yes	53	0	1
1	0	VATS or rc	265	CHU	No	71	0	1
1	1	Thoracotoı	265	CHU	No	61	0	0
1	0	Thoracotoı	265	CHU	No	68	0	0
1	0	Thoracotoı	265	CHU	Yes	52	0	0
1	0	Thoracotoı	265	CHU	No	73	0	0
1	0	Thoracotoı	265	CHU	No	65	0	0
1	0	Thoracotoı	265	CHU	No	59	0	0
1	0	VATS or rc	265	CHU	No	46	0	0
1	0	VATS or rc	265	CHU	No	67	0	0
1	0	VATS or rc	265	CHU	No	68	0	0
1	0	Thoracotoı	265	CHU	No	39	0	1
1	0	Thoracotoı	265	CHU	No	46	0	0
1	0	Thoracotoı	265	CHU	No	65	0	1
1	0	VATS or rc	265	CHU	No	61	0	1
1	0	VATS or rc	265	CHU	No	63	0	1
1	0	VATS or rc	265	CHU	No	66	0	1
1	0	VATS or rc	265	CHU	No	82	0	0
1	0	VATS or rc	265	CHU	No	74	0	0
1	0	VATS or rc	265	CHU	No	85	0	0
1	1	Thoracotoı	265	CHU	No	37	0	0
1	0	Thoracotoı	265	CHU	No	57	0	1
1	0	Thoracotoı	265	CHU	No	63	0	1
1	0	Thoracotoı	265	CHU	No	50	0	0
1	0	Thoracotoı	265	CHU	No	57	0	0
1	0	Thoracotoı	265	CHU	No	62	0	0
1	0	VATS or rc	265	CHU	No	69	1	0
1	0	Thoracotoı	265	CHU	No	50	0	1
1	0	Thoracotoı	265	CHU	No	36	0	0
1	0	Thoracotoı	265	CHU	No	46	0	0
1	0	VATS or rc	265	CHU	No	73	0	1
1	0	Thoracotoı	265	CHU	No	70	0	0
1	0	VATS or rc	265	CHU	No	83	0	0
1	1	Thoracotoı	265	CHU	No	59	1	0
1	0	Thoracotoı	265	CHU	No	52	0	1
1	0	Thoracotoı	265	CHU	No	76	0	0
1	0	Thoracotoı	265	CHU	No	69	0	0
1	0	Thoracotoı	265	CHU	No	72	1	0
1	0	Thoracotoı	265	CHU	No	60	0	0
1	0	VATS or rc	265	CHU	No	71	0	0
1	0	Thoracotoı	265	CHU	No	64	1	0
1	0	Thoracotoı	265	CHU	No	66	0	1
1	0	VATS or rc	265	CHU	No	66	0	0
1	0	Thoracotoı	265	CHU	No	66	0	1
1	0	VATS or rc	265	CHU	No	78	0	0
1	0	Thoracotoı	265	CHU	No	36	0	0
1	0	Thoracotoı	265	CHU	No	62	0	0
1	0	Thoracotoı	265	CHU	No	59	0	1
1	0	VATS or rc	265	CHU	No	67	0	1
1	0	Thoracotoı	265	CHU	No	53	0	0
1	0	VATS or rc	265	CHU	No	67	0	0
1	1	Thoracotoı	265	CHU	No	59	0	1
1	0	Thoracotoı	265	CHU	No	53	0	0
1	0	VATS or rc	265	CHU	No	65	1	1

1	0	Thoracotoı	265	CHU	No	62	1	1
1	0	Thoracotoı	265	CHU	No	56	0	0
1	0	VATS or rc	265	CHU	No	43	0	1
1	0	VATS or rc	265	CHU	No	58	0	0
1	0	VATS or rc	265	CHU	No	57	0	1
1	0	VATS or rc	265	CHU	No	77	0	0
1	0	VATS or rc	265	CHU	No	68	0	0
1	0	VATS or rc	265	CHU	No	44	0	1
1	1	Thoracotoı	265	CHU	Yes	72	1	0
1	0	Thoracotoı	265	CHU	No	51	0	1
1	0	VATS or rc	265	CHU	No	61	0	0
1	0	Thoracotoı	265	CHU	No	58	0	1
1	0	VATS or rc	265	CHU	No	64	0	0
1	0	VATS or rc	265	CHU	No	61	0	1
1	0	Thoracotoı	265	CHU	No	75	0	0
1	0	Thoracotoı	265	CHU	No	77	0	1
1	0	Thoracotoı	265	CHU	No	58	0	0
1	0	VATS or rc	265	CHU	No	61	0	0
1	0	Thoracotoı	265	CHU	No	72	0	0
1	0	Thoracotoı	265	CHU	No	70	1	0
1	0	Thoracotoı	265	CHU	No	58	0	0
1	1	Thoracotoı	265	CHU	No	61	0	0
1	0	VATS or rc	265	CHU	No	73	0	0
1	0	VATS or rc	265	CHU	No	60	0	0
1	0	Thoracotoı	265	CHU	No	67	0	0
1	0	Thoracotoı	265	CHU	No	70	0	0
1	0	VATS or rc	265	CHU	No	60	1	0
1	0	Thoracotoı	265	CHU	No	69	0	0
1	0	Thoracotoı	265	CHU	No	64	0	0
1	0	VATS or rc	265	CHU	No	58	0	0
1	1	Thoracotoı	265	CHU	No	55	0	0
1	0	Thoracotoı	265	CHU	No	70	1	1
1	0	VATS or rc	265	CHU	No	52	0	0
1	0	VATS or rc	265	CHU	No	65	0	0
1	0	Thoracotoı	265	CHU	Yes	63	0	0
1	0	Thoracotoı	265	CHU	No	55	0	0
1	0	Thoracotoı	265	CHU	No	48	0	1
1	0	Thoracotoı	265	CHU	No	62	0	0
1	0	Thoracotoı	265	CHU	No	59	0	0
1	0	Thoracotoı	265	CHU	No	53	0	0
1	0	Thoracotoı	265	CHU	No	83	0	0
1	0	Thoracotoı	265	CHU	No	66	0	0
1	0	VATS or rc	265	CHU	No	72	0	0
1	0	Thoracotoı	265	CHU	No	67	0	1
1	0	Thoracotoı	265	CHU	No	61	0	0
1	0	Thoracotoı	265	CHU	No	71	0	1
1	0	Thoracotoı	265	CHU	No	62	0	0
1	0	VATS or rc	265	CHU	No	78	1	0
1	0	Thoracotoı	265	CHU	Yes	64	0	0
1	0	Thoracotoı	265	CHU	No	65	0	0
1	0	Thoracotoı	265	CHU	No	60	0	1
1	0	Thoracotoı	265	CHU	No	68	0	1
1	0	Thoracotoı	265	CHU	No	64	0	1
1	0	VATS or rc	265	CHU	No	86	1	1
1	0	Thoracotoı	265	CHU	No	64	0	0
1	0	VATS or rc	265	CHU	No	65	0	1

1	0	Thoracotoı	265	CHU	No	73	0	0
1	0	Thoracotoı	265	CHU	No	66	0	0
1	0	Thoracotoı	265	CHU	No	64	0	0
1	0	Thoracotoı	265	CHU	Yes	67	0	0
1	0	Thoracotoı	265	CHU	No	67	0	1
1	0	Thoracotoı	265	CHU	No	51	0	0
1	0	VATS or ro	265	CHU	No	67	0	0
1	0	Thoracotoı	265	CHU	No	47	0	0
1	0	Thoracotoı	265	CHU	No	60	0	1
1	0	Thoracotoı	265	CHU	No	69	1	0
1	0	Thoracotoı	265	CHU	Yes	74	0	1
1	0	Thoracotoı	265	CHU	No	57	0	1
1	0	VATS or ro	265	CHU	No	67	0	0
1	0	VATS or ro	265	CHU	No	62	0	1
1	0	Thoracotoı	265	CHU	Yes	75	0	0
1	0	Thoracotoı	265	CHU	No	67	0	0
1	0	VATS or ro	265	CHU	No	66	0	0
1	0	Thoracotoı	265	CHU	No	78	0	0
1	0	Thoracotoı	265	CHU	No	80	0	0
1	0	Thoracotoı	265	CHU	No	53	0	0
1	0	Thoracotoı	265	CHU	No	55	0	1
1	0	VATS or ro	265	CHU	No	75	0	0
1	0	Thoracotoı	265	CHU	No	78	0	0
1	0	Thoracotoı	265	CHU	No	59	0	1
1	0	Thoracotoı	265	CHU	No	66	0	0
1	1	Thoracotoı	265	CHU	No	42	0	1
1	0	Thoracotoı	265	CHU	No	58	0	1
1	0	Thoracotoı	265	CHU	No	78	0	0
1	0	VATS or ro	265	CHU	No	69	0	0
1	0	Thoracotoı	265	CHU	No	73	0	0
1	0	Thoracotoı	265	CHU	No	69	0	0
1	0	Thoracotoı	265	CHU	No	65	0	1
1	1	Thoracotoı	265	CHU	No	58	0	1
1	0	Thoracotoı	265	CHU	No	62	0	0
1	0	Thoracotoı	265	CHU	No	78	0	0
1	0	VATS or ro	265	CHU	No	63	0	0
1	0	Thoracotoı	265	CHU	No	70	0	1
1	0	Thoracotoı	265	CHU	No	65	1	0
1	0	VATS or ro	265	CHU	No	73	0	1
1	0	Thoracotoı	265	CHU	No	52	0	0
1	0	Thoracotoı	265	CHU	No	51	1	1
1	0	Thoracotoı	265	CHU	No	81	1	0
1	0	VATS or ro	265	CHU	No	63	0	0
1	0	Thoracotoı	265	CHU	No	54	1	1
1	0	VATS or ro	265	CHU	No	55	0	0
1	0	VATS or ro	265	CHU	No	75	0	1
1	0	Thoracotoı	265	CHU	No	71	0	0
1	0	Thoracotoı	265	CHU	No	66	0	1
1	0	Thoracotoı	265	CHU	No	67	0	0
1	1	Thoracotoı	265	CHU	No	55	0	1
1	0	Thoracotoı	265	CHU	No	78	0	0
1	0	VATS or ro	265	CHU	No	49	0	0
1	0	Thoracotoı	265	CHU	No	58	1	1
1	0	VATS or ro	265	CHU	No	69	0	0
1	0	Thoracotoı	265	CHU	No	51	0	1
1	0	Thoracotoı	265	CHU	No	73	0	0

1	0	Thoracotoı	265	CHU	No	58	0	1
1	0	Thoracotoı	265	CHU	No	74	0	1
1	0	Thoracotoı	265	CHU	No	79	0	0
1	0	Thoracotoı	265	CHU	No	51	0	1
1	0	Thoracotoı	265	CHU	No	62	0	0
1	0	Thoracotoı	265	CHU	No	60	0	0
1	0	Thoracotoı	265	CHU	No	47	0	0
1	0	Thoracotoı	265	CHU	No	77	0	1
1	0	VATS or rc	265	CHU	No	49	0	0
1	0	VATS or rc	265	CHU	No	56	1	1
1	0	VATS or rc	265	CHU	No	66	0	0
1	0	Thoracotoı	265	CHU	No	63	1	0
1	0	Thoracotoı	265	CHU	No	74	0	0
1	0	Thoracotoı	265	CHU	No	78	0	0
1	0	Thoracotoı	265	CHU	No	49	0	0
1	0	VATS or rc	265	CHU	No	62	0	1
1	0	Thoracotoı	265	CHU	No	73	0	0
1	0	VATS or rc	265	CHU	No	70	0	1
1	0	Thoracotoı	265	CHU	No	72	0	0
1	0	VATS or rc	265	CHU	No	58	0	1
1	0	Thoracotoı	265	CHU	Yes	67	1	0
1	0	Thoracotoı	265	CHU	No	64	0	0
1	0	Thoracotoı	265	CHU	No	56	0	0
1	0	VATS or rc	265	CHU	Yes	52	0	1
1	0	Thoracotoı	265	CHU	No	68	0	0
1	1	Thoracotoı	265	CHU	No	51	0	0
1	0	Thoracotoı	265	CHU	No	61	1	0
1	0	Thoracotoı	265	CHU	No	59	0	0
1	0	Thoracotoı	265	CHU	No	53	0	0
1	0	Thoracotoı	265	CHU	No	63	0	0
1	1	Thoracotoı	265	CHU	No	62	0	0
1	0	VATS or rc	265	CHU	No	70	0	0
1	0	VATS or rc	265	CHU	No	52	0	1
1	0	Thoracotoı	265	CHU	No	67	0	0
1	0	VATS or rc	265	CHU	No	46	0	1
1	0	VATS or rc	265	CHU	No	61	0	0
1	0	Thoracotoı	265	CHU	No	62	0	0
1	0	Thoracotoı	265	CHU	No	75	1	1
1	0	Thoracotoı	265	CHU	No	74	0	0
1	0	VATS or rc	265	CHU	No	56	0	0
1	0	Thoracotoı	265	CHU	No	74	0	0
1	0	VATS or rc	265	CHU	No	73	1	1
1	0	Thoracotoı	265	CHU	No	45	0	1
1	0	Thoracotoı	265	CHU	No	18	0	0
1	0	Thoracotoı	265	CHU	No	79	0	0
1	0	Thoracotoı	265	CHU	No	71	1	0
1	0	Thoracotoı	265	CHU	No	69	0	0
1	0	VATS or rc	265	CHU	No	48	0	1
1	1	Thoracotoı	265	CHU	No	55	0	0
1	0	VATS or rc	265	CHU	No	65	1	1
1	0	Thoracotoı	265	CHU	No	57	0	0
1	0	Thoracotoı	265	CHU	No	57	0	0
1	0	Thoracotoı	265	CHU	No	68	0	1
1	0	Thoracotoı	265	CHU	No	76	0	0
1	0	VATS or rc	265	CHU	No	62	0	0
1	0	Thoracotoı	265	CHU	No	70	1	0

1	0	Thoracotoı	265	CHU	No	68	0	1
1	0	VATS or rc	265	CHU	No	77	0	0
1	0	Thoracotoı	265	CHU	No	51	0	1
1	0	Thoracotoı	265	CHU	No	73	1	0
1	0	VATS or rc	265	CHU	No	56	0	0
1	0	VATS or rc	265	CHU	No	52	0	1
1	0	Thoracotoı	265	CHU	No	55	0	0
1	0	Thoracotoı	265	CHU	No	53	0	1
1	0	Thoracotoı	265	CHU	No	57	0	0
1	0	Thoracotoı	265	CHU	No	55	0	1
1	0	VATS or rc	265	CHU	No	79	0	0
1	0	VATS or rc	265	CHU	No	59	0	0
1	0	VATS or rc	265	CHU	No	59	0	0
1	1	Thoracotoı	265	CHU	No	42	0	0
1	0	Thoracotoı	265	CHU	No	70	0	0
1	0	Thoracotoı	265	CHU	No	80	0	0
1	0	Thoracotoı	265	CHU	No	57	0	0
1	1	Thoracotoı	265	CHU	No	56	0	0
1	1	Thoracotoı	265	CHU	No	76	1	0
1	0	VATS or rc	265	CHU	No	64	0	0
1	0	VATS or rc	265	CHU	No	57	0	1
1	0	Thoracotoı	265	CHU	No	73	0	0
1	0	VATS or rc	265	CHU	No	58	0	0
1	0	Thoracotoı	265	CHU	No	59	0	1
1	0	Thoracotoı	265	CHU	No	40	0	0
1	0	Thoracotoı	265	CHU	No	72	0	0
1	0	VATS or rc	265	CHU	No	63	0	1
1	0	VATS or rc	265	CHU	No	74	0	1
1	1	Thoracotoı	265	CHU	No	56	0	0
1	0	VATS or rc	265	CHU	No	63	0	1
1	1	Thoracotoı	265	CHU	No	50	0	0
1	0	VATS or rc	265	CHU	No	55	0	0
1	0	Thoracotoı	265	CHU	No	40	0	0
1	0	Thoracotoı	265	CHU	No	68	0	0
1	0	Thoracotoı	265	CHU	No	59	0	1
1	0	Thoracotoı	265	CHU	No	64	0	0
1	0	VATS or rc	265	CHU	No	77	1	0
1	0	VATS or rc	265	CHU	No	74	0	0
1	0	Thoracotoı	265	CHU	No	77	0	0
1	1	Thoracotoı	265	CHU	No	70	1	1
1	0	VATS or rc	265	CHU	No	55	0	0
1	0	VATS or rc	265	CHU	No	76	0	1
1	0	Thoracotoı	265	CHU	No	33	0	0
1	0	VATS or rc	265	CHU	No	56	0	0
1	0	Thoracotoı	265	CHU	Yes	64	0	1
1	0	Thoracotoı	265	CHU	No	69	0	1
1	0	Thoracotoı	265	CHU	No	65	0	0
1	0	VATS or rc	265	CHU	No	46	0	1
1	0	VATS or rc	265	CHU	No	65	0	1
1	0	Thoracotoı	265	CHU	No	64	0	1
1	0	Thoracotoı	265	CHU	No	62	0	0
1	0	Thoracotoı	265	CHU	No	79	0	1
1	0	Thoracotoı	265	CHU	No	71	0	1
1	0	VATS or rc	265	CHU	No	42	0	0
1	0	Thoracotoı	265	CHU	No	71	0	0
1	0	Thoracotoı	265	CHU	No	71	0	0

1	0	Thoracotoı	265	CHU	No	69	0	0
1	0	Thoracotoı	265	CHU	No	65	0	0
1	0	VATS or rc	265	CHU	No	67	0	0
1	0	Thoracotoı	265	CHU	No	55	0	1
1	0	Thoracotoı	265	CHU	No	58	0	0
1	0	Thoracotoı	265	CHU	No	46	0	1
1	0	Thoracotoı	265	CHU	No	58	0	0
1	0	Thoracotoı	265	CHU	No	53	0	0
1	0	Thoracotoı	265	CHU	No	74	1	0
1	0	Thoracotoı	265	CHU	No	47	0	0
1	0	Thoracotoı	265	CHU	No	69	0	0
1	0	Thoracotoı	265	CHU	No	63	0	1
1	0	Thoracotoı	265	CHU	Yes	75	0	0
1	0	Thoracotoı	265	CHU	No	76	0	0
1	0	VATS or rc	265	CHU	No	69	0	1
1	0	Thoracotoı	265	CHU	Yes	65	0	0
1	0	VATS or rc	265	CHU	No	47	0	0
1	0	Thoracotoı	265	CHU	No	63	0	0
1	0	VATS or rc	265	CHU	No	56	0	1
1	0	VATS or rc	265	CHU	No	49	1	0
1	0	VATS or rc	265	CHU	No	71	0	1
1	1	Thoracotoı	265	CHU	No	57	0	1
1	0	Thoracotoı	265	CHU	No	72	0	0
1	0	Thoracotoı	265	CHU	No	69	0	0
1	0	Thoracotoı	265	CHU	No	58	0	0
1	0	Thoracotoı	265	CHU	No	65	0	1
1	0	VATS or rc	265	CHU	No	62	0	0
1	0	VATS or rc	265	CHU	No	57	0	0
1	0	Thoracotoı	265	CHU	No	76	0	0
1	0	Thoracotoı	265	CHU	No	74	0	0
1	0	Thoracotoı	265	CHU	No	72	0	0
1	0	VATS or rc	265	CHU	No	68	1	0
1	0	VATS or rc	265	CHU	No	54	0	0
1	0	Thoracotoı	265	CHU	No	54	0	1
1	0	Thoracotoı	265	CHU	No	63	0	0
1	0	VATS or rc	265	CHU	No	58	0	0
1	0	Thoracotoı	265	CHU	No	69	0	0
1	0	Thoracotoı	265	CHU	No	68	0	0
1	0	VATS or rc	265	CHU	No	67	0	0
1	0	Thoracotoı	265	CHU	No	49	0	0
1	0	VATS or rc	265	CHU	No	61	0	0
1	0	VATS or rc	265	CHU	No	63	0	1
1	0	Thoracotoı	265	CHU	No	56	0	0
1	0	VATS or rc	265	CHU	No	66	0	1
1	0	Thoracotoı	265	CHU	No	64	0	0
1	0	Thoracotoı	265	CHU	No	58	0	1
1	0	VATS or rc	265	CHU	No	77	0	0
1	0	Thoracotoı	265	CHU	No	80	0	0
1	0	VATS or rc	265	CHU	No	64	0	0
1	0	Thoracotoı	265	CHU	No	68	0	0
1	0	VATS or rc	265	CHU	No	63	1	0
1	0	Thoracotoı	265	CHU	No	67	0	1
1	0	Thoracotoı	265	CHU	No	67	0	0
1	1	Thoracotoı	265	CHU	No	60	0	0
1	0	Thoracotoı	265	CHU	No	79	1	0
1	0	Thoracotoı	265	CHU	No	64	0	1

1	0	Thoracotoı	265	CHU	No	37	0	0
1	0	Thoracotoı	265	CHU	No	58	0	0
1	0	Thoracotoı	265	CHU	No	73	0	1
1	0	VATS or rc	265	CHU	No	83	0	1
1	1	Thoracotoı	265	CHU	No	71	0	0
1	0	Thoracotoı	265	CHU	No	72	0	0
1	0	Thoracotoı	265	CHU	No	18	0	0
1	0	VATS or rc	265	CHU	No	61	0	0
1	0	Thoracotoı	265	CHU	No	62	0	1
1	0	VATS or rc	265	CHU	No	63	0	0
1	0	VATS or rc	265	CHU	No	44	0	1
1	0	Thoracotoı	265	CHU	No	60	0	1
1	0	Thoracotoı	265	CHU	No	54	0	0
1	0	Thoracotoı	265	CHU	No	53	0	1
1	0	Thoracotoı	265	CHU	No	63	0	0
1	0	VATS or rc	265	CHU	No	81	0	0
1	0	Thoracotoı	265	CHU	No	56	0	0
1	0	Thoracotoı	265	CHU	No	65	0	1
1	1	Thoracotoı	265	CHU	No	73	0	0
1	0	Thoracotoı	265	CHU	Yes	70	1	0
1	0	VATS or rc	265	CHU	No	69	0	1
1	0	Thoracotoı	265	CHU	No	54	0	1
1	0	VATS or rc	265	CHU	No	68	0	0
1	1	Thoracotoı	265	CHU	No	72	0	1
1	0	Thoracotoı	265	CHU	No	80	1	0
1	0	Thoracotoı	265	CHU	No	67	0	0
1	0	Thoracotoı	265	CHU	No	65	0	0
1	0	VATS or rc	265	CHU	No	61	0	1
1	0	VATS or rc	265	CHU	No	72	0	0
1	0	VATS or rc	265	CHU	No	77	0	1
1	0	Thoracotoı	265	CHU	No	57	1	1
1	0	Thoracotoı	265	CHU	No	52	0	0
1	0	Thoracotoı	265	CHU	No	57	0	0
1	1	Thoracotoı	265	CHU	No	64	1	0
1	0	Thoracotoı	265	CHU	No	75	0	1
1	0	Thoracotoı	265	CHU	No	59	0	0
1	0	VATS or rc	265	CHU	No	49	0	1
1	0	Thoracotoı	265	CHU	No	78	0	1
1	0	Thoracotoı	265	CHU	No	69	0	1
1	0	VATS or rc	265	CHU	No	70	0	0
1	0	Thoracotoı	265	CHU	No	47	0	0
1	1	Thoracotoı	265	CHU	No	72	0	0
1	0	Thoracotoı	265	CHU	No	59	0	0
1	0	Thoracotoı	265	CHU	No	70	1	0
1	0	VATS or rc	265	CHU	No	74	0	1
1	0	VATS or rc	265	CHU	No	63	0	0
1	0	VATS or rc	265	CHU	No	70	1	0
1	1	Thoracotoı	265	CHU	No	65	0	0
1	0	Thoracotoı	265	CHU	No	72	0	0
1	1	Thoracotoı	265	CHU	No	43	0	1
1	0	Thoracotoı	265	CHU	No	54	0	1
1	0	Thoracotoı	265	CHU	No	69	1	0
1	0	Thoracotoı	265	CHU	No	51	0	0
1	1	Thoracotoı	265	CHU	No	61	0	1
1	0	VATS or rc	265	CHU	No	49	0	0
1	0	Thoracotoı	265	CHU	No	62	0	1

1	0	Thoracotoı	265	CHU	No	61	1	1
1	0	Thoracotoı	265	CHU	No	68	0	0
1	0	Thoracotoı	265	CHU	No	64	0	1
1	0	Thoracotoı	265	CHU	No	68	0	1
1	0	Thoracotoı	265	CHU	No	57	0	1
1	0	Thoracotoı	265	CHU	No	57	0	1
1	0	Thoracotoı	265	CHU	No	67	0	1
1	0	Thoracotoı	265	CHU	No	49	0	1
1	0	VATS or rc	265	CHU	No	82	0	0
1	0	VATS or rc	265	CHU	No	78	0	0
1	0	Thoracotoı	265	CHU	No	79	0	0
1	0	VATS or rc	265	CHU	Yes	64	0	0
1	0	Thoracotoı	265	CHU	No	59	0	0
1	0	Thoracotoı	265	CHU	No	74	0	0
1	0	Thoracotoı	265	CHU	No	46	0	0
1	0	Thoracotoı	265	CHU	No	64	0	1
1	0	Thoracotoı	265	CHU	No	73	1	1
1	0	VATS or rc	265	CHU	No	68	1	1
1	0	Thoracotoı	265	CHU	No	59	1	0
1	0	Thoracotoı	265	CHU	No	58	0	1
1	0	Thoracotoı	265	CHU	No	68	0	0
1	0	VATS or rc	265	CHU	No	74	0	0
1	0	Thoracotoı	265	CHU	No	57	0	1
0	0	Thoracotoı	26	CHG	No	78	0	0
0	0	Thoracotoı	26	CHG	No	62	0	0
0	0	Thoracotoı	26	CHG	No	57	0	0
0	0	Thoracotoı	26	CHG	No	68	0	0
1	0	Thoracotoı	64	PRIVE	No	68	0	0
1	0	Thoracotoı	64	PRIVE	No	62	0	0
1	0	VATS or rc	64	PRIVE	No	68	0	0
1	0	Thoracotoı	64	PRIVE	No	68	0	0
1	0	VATS or rc	64	PRIVE	No	70	0	0
1	0	VATS or rc	64	PRIVE	No	65	0	0
1	0	VATS or rc	64	PRIVE	No	68	0	0
1	0	VATS or rc	64	PRIVE	No	66	0	0
1	0	VATS or rc	64	PRIVE	No	72	0	1
1	0	VATS or rc	64	PRIVE	No	70	0	0
1	0	VATS or rc	64	PRIVE	No	71	0	0
1	0	Thoracotoı	64	PRIVE	No	72	0	0
1	0	Thoracotoı	64	PRIVE	No	51	0	0
1	0	VATS or rc	64	PRIVE	No	83	0	0
1	0	VATS or rc	64	PRIVE	No	77	0	0
1	0	VATS or rc	64	PRIVE	No	66	0	0
1	0	VATS or rc	64	PRIVE	No	65	0	0
1	0	VATS or rc	64	PRIVE	No	42	0	0
1	0	Thoracotoı	64	PRIVE	No	67	0	0
1	0	VATS or rc	64	PRIVE	No	63	0	0
1	0	Thoracotoı	64	PRIVE	No	53	0	1
1	0	VATS or rc	64	PRIVE	No	76	0	0
1	0	Thoracotoı	64	PRIVE	No	58	0	0
1	0	VATS or rc	64	PRIVE	No	75	0	0
1	0	Thoracotoı	64	PRIVE	No	62	0	0
1	0	VATS or rc	64	PRIVE	No	60	0	0
1	0	Thoracotoı	64	PRIVE	No	79	0	0
1	0	VATS or rc	64	PRIVE	No	83	0	0
1	0	Thoracotoı	64	PRIVE	No	75	0	0

1	0	VATS or rc	64	PRIVE	No	71	0	0
1	0	VATS or rc	64	PRIVE	No	57	0	0
1	0	VATS or rc	64	PRIVE	No	75	0	0
1	0	Thoracotoı	64	PRIVE	No	55	0	1
1	1	Thoracotoı	64	PRIVE	No	59	0	0
1	0	Thoracotoı	64	PRIVE	No	60	0	0
1	0	Thoracotoı	64	PRIVE	No	43	0	0
1	0	VATS or rc	64	PRIVE	No	72	0	0
1	0	VATS or rc	64	PRIVE	No	85	0	0
1	0	VATS or rc	64	PRIVE	No	71	0	0
1	0	VATS or rc	64	PRIVE	No	64	0	0
1	0	Thoracotoı	64	PRIVE	No	61	0	0
1	0	VATS or rc	64	PRIVE	No	66	0	1
1	0	VATS or rc	64	PRIVE	No	76	0	0
1	0	Thoracotoı	64	PRIVE	No	71	0	0
1	0	Thoracotoı	64	PRIVE	No	75	0	0
1	0	Thoracotoı	64	PRIVE	No	69	1	0
1	0	VATS or rc	64	PRIVE	No	48	0	0
1	0	VATS or rc	64	PRIVE	No	68	0	0
1	0	Thoracotoı	64	PRIVE	No	82	0	0
1	0	VATS or rc	64	PRIVE	No	59	0	0
1	1	Thoracotoı	64	PRIVE	No	47	0	0
1	0	VATS or rc	64	PRIVE	No	71	1	0
1	0	Thoracotoı	64	PRIVE	No	69	0	0
1	0	Thoracotoı	64	PRIVE	Yes	69	0	0
1	0	VATS or rc	64	PRIVE	No	79	0	0
1	0	Thoracotoı	64	PRIVE	No	76	0	0
1	0	Thoracotoı	64	PRIVE	No	67	0	0
1	0	VATS or rc	64	PRIVE	No	71	0	0
1	0	Thoracotoı	64	PRIVE	No	79	0	0
1	0	Thoracotoı	64	PRIVE	No	50	0	0
1	1	Thoracotoı	64	PRIVE	No	60	0	0
1	0	VATS or rc	64	PRIVE	No	68	0	0
1	0	VATS or rc	64	PRIVE	No	66	0	0
1	0	Thoracotoı	64	PRIVE	No	58	0	0
1	0	VATS or rc	64	PRIVE	No	72	0	0
1	0	Thoracotoı	64	PRIVE	No	64	0	0
1	0	Thoracotoı	64	PRIVE	No	75	0	0
1	0	VATS or rc	64	PRIVE	No	79	0	0
1	0	Thoracotoı	64	PRIVE	No	61	0	0
1	0	Thoracotoı	64	PRIVE	No	65	0	0
1	0	Thoracotoı	64	PRIVE	No	67	0	0
1	0	VATS or rc	64	PRIVE	No	55	0	0
1	0	Thoracotoı	64	PRIVE	No	56	0	0
1	0	Thoracotoı	64	PRIVE	No	74	1	0
1	0	VATS or rc	64	PRIVE	No	51	1	0
1	0	VATS or rc	64	PRIVE	No	81	0	0
1	0	VATS or rc	64	PRIVE	No	71	0	0
1	0	VATS or rc	64	PRIVE	No	74	0	0
1	0	VATS or rc	64	PRIVE	No	66	0	1
1	0	Thoracotoı	64	PRIVE	No	65	0	0
1	0	Thoracotoı	64	PRIVE	No	60	0	0
1	0	VATS or rc	64	PRIVE	No	60	0	0
1	0	Thoracotoı	64	PRIVE	No	49	0	0
1	0	VATS or rc	64	PRIVE	No	61	0	0
1	0	Thoracotoı	64	PRIVE	No	67	0	0

1	0	VATS or rc	64	PRIVE	No	72	0	0
1	0	VATS or rc	64	PRIVE	No	64	0	0
1	0	VATS or rc	64	PRIVE	No	51	0	0
1	0	VATS or rc	64	PRIVE	No	74	0	0
1	0	Thoracotoı	64	PRIVE	No	72	0	0
1	0	VATS or rc	64	PRIVE	No	61	0	0
1	0	VATS or rc	64	PRIVE	No	61	0	0
1	0	Thoracotoı	64	PRIVE	No	75	0	0
1	0	VATS or rc	64	PRIVE	No	76	0	0
1	0	Thoracotoı	64	PRIVE	No	74	0	0
1	0	Thoracotoı	64	PRIVE	No	71	0	0
1	1	Thoracotoı	64	PRIVE	No	65	0	0
1	0	VATS or rc	64	PRIVE	No	68	0	0
1	0	Thoracotoı	64	PRIVE	No	72	1	0
1	0	Thoracotoı	64	PRIVE	Yes	84	0	0
1	0	Thoracotoı	64	PRIVE	No	79	0	0
1	0	VATS or rc	64	PRIVE	No	74	0	0
1	0	VATS or rc	64	PRIVE	No	63	0	0
1	1	Thoracotoı	64	PRIVE	No	59	1	0
1	0	VATS or rc	64	PRIVE	No	84	0	0
1	0	Thoracotoı	64	PRIVE	No	72	0	0
1	0	Thoracotoı	64	PRIVE	No	75	0	0
1	0	VATS or rc	64	PRIVE	No	81	0	0
1	0	VATS or rc	64	PRIVE	No	67	0	0
1	0	VATS or rc	64	PRIVE	No	80	0	0
1	0	VATS or rc	64	PRIVE	No	80	0	0
1	0	Thoracotoı	64	PRIVE	No	67	0	0
1	0	Thoracotoı	64	PRIVE	No	71	0	0
1	0	VATS or rc	64	PRIVE	No	38	0	0
1	0	VATS or rc	64	PRIVE	No	63	0	0
1	0	Thoracotoı	64	PRIVE	No	71	0	0
1	0	VATS or rc	64	PRIVE	No	77	0	0
1	0	Thoracotoı	64	PRIVE	No	79	0	0
1	0	VATS or rc	64	PRIVE	No	69	1	0
1	0	Thoracotoı	64	PRIVE	No	79	1	0
1	0	VATS or rc	64	PRIVE	No	73	0	0
1	0	Thoracotoı	64	PRIVE	No	59	0	0
1	0	VATS or rc	64	PRIVE	No	72	0	0
1	0	Thoracotoı	64	PRIVE	No	64	0	0
1	0	Thoracotoı	64	PRIVE	No	31	0	0
1	0	Thoracotoı	64	PRIVE	No	75	0	0
1	0	Thoracotoı	64	PRIVE	No	72	0	0
1	0	Thoracotoı	64	PRIVE	No	72	0	0
1	0	Thoracotoı	64	PRIVE	No	70	0	0
1	0	Thoracotoı	64	PRIVE	No	70	0	0
1	0	VATS or rc	64	PRIVE	No	65	0	0
1	0	VATS or rc	64	PRIVE	No	67	0	0
1	0	Thoracotoı	64	PRIVE	No	72	0	0
1	0	VATS or rc	64	PRIVE	No	61	0	0
1	0	VATS or rc	64	PRIVE	No	70	0	0
1	0	VATS or rc	64	PRIVE	No	60	0	0
1	0	VATS or rc	64	PRIVE	No	75	0	0
1	0	Thoracotoı	64	PRIVE	No	66	0	0
1	0	VATS or rc	64	PRIVE	No	60	0	0
1	0	Thoracotoı	64	PRIVE	No	37	0	0
1	0	VATS or rc	64	PRIVE	No	62	0	1

1	0	Thoracotor	64	PRIVE	No	69	0	0
1	0	VATS or rc	64	PRIVE	No	72	0	0
1	1	Thoracotor	64	PRIVE	No	77	0	0
1	0	Thoracotor	64	PRIVE	No	74	0	0
1	0	VATS or rc	64	PRIVE	No	73	0	0
1	0	VATS or rc	64	PRIVE	No	54	0	0
1	1	Thoracotor	64	PRIVE	No	47	0	0
1	0	VATS or rc	64	PRIVE	No	75	0	0
1	0	VATS or rc	64	PRIVE	No	69	0	0
1	0	VATS or rc	64	PRIVE	No	67	0	0
1	0	VATS or rc	64	PRIVE	No	51	0	0
1	0	VATS or rc	64	PRIVE	No	63	1	0
1	0	VATS or rc	64	PRIVE	No	66	0	0
1	0	Thoracotor	64	PRIVE	No	77	0	0
1	0	VATS or rc	64	PRIVE	No	62	0	0
1	0	VATS or rc	64	PRIVE	No	79	0	0
1	0	VATS or rc	64	PRIVE	No	81	0	0
1	0	VATS or rc	64	PRIVE	No	77	0	0
1	0	VATS or rc	64	PRIVE	No	70	0	0
1	0	Thoracotor	64	PRIVE	No	65	0	0
1	0	Thoracotor	64	PRIVE	No	57	0	0
1	0	VATS or rc	64	PRIVE	No	59	0	0
1	0	VATS or rc	64	PRIVE	No	73	0	0
1	0	VATS or rc	64	PRIVE	No	64	0	0
1	0	VATS or rc	64	PRIVE	No	66	0	0
1	0	Thoracotor	64	PRIVE	No	66	1	0
1	0	Thoracotor	64	PRIVE	No	63	0	0
1	0	Thoracotor	64	PRIVE	No	65	0	0
1	0	VATS or rc	64	PRIVE	Yes	78	0	0
1	0	Thoracotor	64	PRIVE	No	67	1	0
1	0	VATS or rc	64	PRIVE	No	63	0	0
1	0	VATS or rc	64	PRIVE	No	72	0	0
1	0	VATS or rc	64	PRIVE	No	64	0	0
1	0	VATS or rc	64	PRIVE	No	81	0	0
1	0	VATS or rc	64	PRIVE	No	71	0	0
1	0	VATS or rc	64	PRIVE	No	67	0	0
1	0	VATS or rc	64	PRIVE	No	76	0	0
1	0	VATS or rc	64	PRIVE	No	67	0	0
1	0	Thoracotor	64	PRIVE	No	66	0	0
1	0	VATS or rc	64	PRIVE	No	74	0	0
1	0	VATS or rc	64	PRIVE	No	58	0	0
1	0	Thoracotor	64	PRIVE	No	63	0	0
1	0	VATS or rc	64	PRIVE	No	61	0	0
1	0	Thoracotor	64	PRIVE	No	64	0	0
1	0	Thoracotor	64	PRIVE	No	64	0	1
1	0	Thoracotor	64	PRIVE	No	71	0	0
1	0	VATS or rc	64	PRIVE	No	60	0	0
1	0	VATS or rc	64	PRIVE	No	78	0	0
1	0	VATS or rc	64	PRIVE	No	75	0	0
1	0	VATS or rc	64	PRIVE	No	56	0	0
1	0	VATS or rc	64	PRIVE	No	71	0	0
1	0	Thoracotor	64	PRIVE	No	65	1	0
1	0	VATS or rc	64	PRIVE	No	59	0	0
1	0	Thoracotor	64	PRIVE	No	60	0	0
1	0	VATS or rc	64	PRIVE	No	68	0	0
1	0	VATS or rc	64	PRIVE	No	78	0	0

1	0	VATS or rc	64	PRIVE	No	56	0	0
1	0	Thoracotoı	64	PRIVE	No	77	0	0
1	0	VATS or rc	64	PRIVE	No	79	0	0
1	0	Thoracotoı	64	PRIVE	No	71	0	0
1	1	Thoracotoı	64	PRIVE	No	74	0	0
1	0	VATS or rc	64	PRIVE	No	59	0	0
1	0	VATS or rc	64	PRIVE	No	77	0	0
1	0	Thoracotoı	64	PRIVE	No	62	0	0
1	0	VATS or rc	64	PRIVE	No	70	0	0
1	0	VATS or rc	64	PRIVE	No	67	0	0
0	0	VATS or rc	248	CHU	No	62	0	1
0	0	Thoracotoı	248	CHU	No	61	0	1
0	0	VATS or rc	248	CHU	No	60	0	1
0	0	VATS or rc	248	CHU	No	53	0	1
0	0	Thoracotoı	248	CHU	No	53	0	0
0	0	VATS or rc	248	CHU	No	77	0	0
0	0	VATS or rc	248	CHU	No	72	0	1
0	1	Thoracotoı	248	CHU	No	69	0	0
0	0	VATS or rc	248	CHU	No	71	0	1
0	0	VATS or rc	248	CHU	No	57	0	1
0	0	VATS or rc	248	CHU	No	64	0	1
0	0	Thoracotoı	248	CHU	No	47	0	0
0	0	Thoracotoı	248	CHU	No	74	1	1
0	0	VATS or rc	248	CHU	No	69	0	0
0	0	VATS or rc	248	CHU	No	71	0	1
0	0	VATS or rc	248	CHU	No	70	0	1
0	0	VATS or rc	248	CHU	No	79	0	1
0	0	VATS or rc	248	CHU	No	74	1	0
0	0	VATS or rc	248	CHU	No	75	0	0
0	0	Thoracotoı	248	CHU	No	78	0	0
0	0	Thoracotoı	248	CHU	No	69	0	1
0	0	VATS or rc	248	CHU	No	82	0	1
0	0	VATS or rc	248	CHU	No	68	0	1
0	0	VATS or rc	248	CHU	No	63	0	1
0	0	Thoracotoı	248	CHU	No	58	1	1
0	0	VATS or rc	248	CHU	No	62	0	1
0	0	VATS or rc	248	CHU	No	48	0	1
0	0	VATS or rc	248	CHU	No	60	0	1
0	0	VATS or rc	248	CHU	No	80	0	1
0	0	VATS or rc	248	CHU	No	69	1	1
0	0	VATS or rc	248	CHU	No	75	0	1
0	0	VATS or rc	248	CHU	No	62	0	0
0	1	Thoracotoı	248	CHU	No	53	0	1
0	0	VATS or rc	248	CHU	No	61	0	0
0	0	VATS or rc	248	CHU	No	79	1	1
0	0	VATS or rc	248	CHU	No	77	0	0
0	0	Thoracotoı	248	CHU	No	71	0	0
0	0	Thoracotoı	248	CHU	No	74	0	0
0	0	VATS or rc	248	CHU	No	57	0	0
0	0	VATS or rc	248	CHU	No	73	0	0
0	0	VATS or rc	248	CHU	No	70	0	0
0	0	VATS or rc	248	CHU	No	77	0	0
0	0	VATS or rc	248	CHU	No	57	0	0
0	0	VATS or rc	248	CHU	No	64	0	0
0	0	VATS or rc	248	CHU	No	70	0	1
0	0	VATS or rc	248	CHU	No	70	0	0

0	0	VATS or rc	248	CHU	No	78	0	0
0	0	VATS or rc	248	CHU	No	66	0	1
0	0	VATS or rc	248	CHU	No	65	0	1
0	0	VATS or rc	248	CHU	No	77	1	1
0	0	Thoracotoı	248	CHU	No	67	0	1
0	0	VATS or rc	248	CHU	No	67	0	0
0	0	VATS or rc	248	CHU	No	73	0	1
0	1	Thoracotoı	248	CHU	No	63	0	1
0	0	VATS or rc	248	CHU	No	71	0	1
0	0	VATS or rc	248	CHU	No	64	0	0
0	0	VATS or rc	248	CHU	No	75	0	1
0	1	Thoracotoı	248	CHU	No	71	0	0
0	0	VATS or rc	248	CHU	No	54	0	0
0	1	VATS or rc	248	CHU	No	78	0	0
0	0	VATS or rc	248	CHU	No	73	0	0
0	0	VATS or rc	248	CHU	No	41	0	0
0	0	VATS or rc	248	CHU	No	67	0	1
0	0	VATS or rc	248	CHU	No	77	0	0
0	0	Thoracotoı	248	CHU	No	70	0	1
0	0	VATS or rc	248	CHU	No	52	0	0
0	0	VATS or rc	248	CHU	No	49	0	0
0	0	VATS or rc	248	CHU	No	76	0	1
0	0	VATS or rc	248	CHU	No	69	0	1
0	0	VATS or rc	248	CHU	No	58	0	1
0	1	Thoracotoı	248	CHU	No	59	0	1
0	0	VATS or rc	248	CHU	No	57	0	1
0	0	VATS or rc	248	CHU	No	58	0	0
0	0	VATS or rc	248	CHU	No	48	0	1
0	0	VATS or rc	248	CHU	No	72	0	0
0	0	VATS or rc	248	CHU	No	55	0	0
0	0	VATS or rc	248	CHU	No	60	0	1
0	0	VATS or rc	248	CHU	No	60	0	1
0	0	Thoracotoı	248	CHU	No	53	0	1
0	0	Thoracotoı	248	CHU	No	32	0	0
0	0	VATS or rc	248	CHU	No	54	0	1
0	0	VATS or rc	248	CHU	No	66	0	0
0	0	VATS or rc	248	CHU	No	65	0	1
0	0	VATS or rc	248	CHU	No	74	0	0
0	0	VATS or rc	248	CHU	No	53	0	1
0	0	Thoracotoı	6	PRIVE	No	67	0	0
0	0	Thoracotoı	6	PRIVE	No	53	0	0
0	0	Thoracotoı	6	PRIVE	No	64	0	0
0	0	VATS or rc	6	PRIVE	No	71	0	1
0	0	Thoracotoı	6	PRIVE	No	52	0	0
0	0	Thoracotoı	6	PRIVE	No	74	0	0
0	0	Thoracotoı	6	PRIVE	No	55	0	1
0	0	Thoracotoı	6	PRIVE	No	49	0	1
0	0	Thoracotoı	6	PRIVE	No	51	0	1
0	0	Thoracotoı	6	PRIVE	No	53	0	0
0	0	Thoracotoı	6	PRIVE	No	79	0	0
0	0	Thoracotoı	6	PRIVE	No	53	0	0
0	0	Thoracotoı	6	PRIVE	No	52	0	1
0	0	Thoracotoı	6	PRIVE	No	72	1	0
0	0	Thoracotoı	6	PRIVE	No	62	0	1
0	0	Thoracotoı	49	PRIVE	No	60	0	0
0	0	VATS or rc	49	PRIVE	No	67	0	0

0	0	Thoracotoı	49	PRIVE	No	64	0	0
0	0	VATS or rc	49	PRIVE	No	65	0	0
0	0	Thoracotoı	49	PRIVE	No	68	1	0
0	0	Thoracotoı	49	PRIVE	No	69	0	0
0	0	Thoracotoı	49	PRIVE	No	71	0	0
0	0	Thoracotoı	49	PRIVE	No	59	0	0
0	0	Thoracotoı	49	PRIVE	No	64	0	0
0	0	Thoracotoı	49	PRIVE	No	78	0	0
0	0	Thoracotoı	49	PRIVE	No	75	0	0
0	0	Thoracotoı	49	PRIVE	No	63	0	0
0	0	Thoracotoı	49	PRIVE	No	83	0	0
0	0	Thoracotoı	49	PRIVE	No	61	1	0
0	0	Thoracotoı	49	PRIVE	No	54	0	0
0	1	Thoracotoı	49	PRIVE	No	81	0	0
0	0	VATS or rc	49	PRIVE	No	39	0	0
0	0	Thoracotoı	49	PRIVE	No	77	1	0
0	0	Thoracotoı	49	PRIVE	No	64	1	0
0	1	Thoracotoı	49	PRIVE	No	67	0	0
0	0	Thoracotoı	49	PRIVE	No	53	0	0
0	1	Thoracotoı	49	PRIVE	No	56	0	0
0	0	Thoracotoı	49	PRIVE	No	64	0	0
0	0	Thoracotoı	49	PRIVE	No	60	0	0
0	0	Thoracotoı	49	PRIVE	No	72	0	0
0	1	Thoracotoı	49	PRIVE	Yes	67	0	0
0	0	Thoracotoı	49	PRIVE	No	61	0	0
0	0	VATS or rc	49	PRIVE	No	74	0	0
0	0	Thoracotoı	49	PRIVE	No	75	0	0
0	0	Thoracotoı	49	PRIVE	No	62	0	0
0	0	VATS or rc	49	PRIVE	No	51	0	0
0	0	Thoracotoı	49	PRIVE	No	76	0	0
0	0	Thoracotoı	49	PRIVE	No	64	0	0
0	0	Thoracotoı	49	PRIVE	No	53	0	0
0	0	Thoracotoı	49	PRIVE	No	79	0	0
0	0	Thoracotoı	49	PRIVE	No	71	0	0
0	0	Thoracotoı	49	PRIVE	No	47	0	0
0	0	Thoracotoı	49	PRIVE	No	61	0	0
0	0	Thoracotoı	49	PRIVE	No	59	0	0
0	0	Thoracotoı	49	PRIVE	No	69	0	0
0	1	Thoracotoı	49	PRIVE	No	73	0	0
0	0	Thoracotoı	49	PRIVE	No	61	0	0
0	0	Thoracotoı	49	PRIVE	No	73	0	0
0	0	Thoracotoı	49	PRIVE	No	64	1	0
0	0	Thoracotoı	49	PRIVE	No	70	0	0
0	0	Thoracotoı	49	PRIVE	No	82	0	0
0	0	VATS or rc	49	PRIVE	No	67	0	0
0	0	Thoracotoı	49	PRIVE	No	81	0	0
0	0	Thoracotoı	49	PRIVE	No	57	0	0
0	0	Thoracotoı	49	PRIVE	No	63	0	0
0	1	Thoracotoı	49	PRIVE	No	70	0	0
0	0	Thoracotoı	49	PRIVE	No	58	0	0
0	0	VATS or rc	49	PRIVE	No	56	0	0
0	0	VATS or rc	49	PRIVE	No	69	0	0
0	0	Thoracotoı	49	PRIVE	No	69	0	0
0	0	Thoracotoı	49	PRIVE	No	57	0	0
0	0	Thoracotoı	49	PRIVE	No	85	0	0
0	0	Thoracotoı	49	PRIVE	No	65	0	0

0	0	Thoracotoı	49	PRIVE	No	63	1	0
0	0	Thoracotoı	49	PRIVE	No	57	0	0
0	0	Thoracotoı	49	PRIVE	No	64	0	0
0	0	Thoracotoı	49	PRIVE	No	67	0	0
0	0	VATS or rc	49	PRIVE	No	34	0	0
0	0	VATS or rc	49	PRIVE	No	74	0	0
0	1	Thoracotoı	49	PRIVE	No	79	0	0
0	0	Thoracotoı	49	PRIVE	No	69	0	0
0	0	Thoracotoı	49	PRIVE	No	68	0	0
0	0	VATS or rc	49	PRIVE	No	50	0	0
0	0	Thoracotoı	49	PRIVE	No	69	1	0
0	0	Thoracotoı	49	PRIVE	No	59	0	0
0	0	Thoracotoı	49	PRIVE	No	78	0	0
0	0	Thoracotoı	49	PRIVE	No	44	0	0
0	0	Thoracotoı	49	PRIVE	No	57	0	0
0	1	Thoracotoı	49	PRIVE	No	55	0	0
0	0	Thoracotoı	49	PRIVE	No	76	0	0
0	1	Thoracotoı	49	PRIVE	No	69	0	0
0	0	Thoracotoı	49	PRIVE	No	70	0	0
0	0	Thoracotoı	49	PRIVE	No	75	0	0
0	0	Thoracotoı	49	PRIVE	No	74	0	0
0	0	Thoracotoı	49	PRIVE	No	76	0	0
0	0	Thoracotoı	49	PRIVE	No	66	0	0
0	0	Thoracotoı	49	PRIVE	No	76	0	0
0	0	Thoracotoı	49	PRIVE	No	69	0	0
0	0	Thoracotoı	49	PRIVE	No	55	0	0
0	0	Thoracotoı	49	PRIVE	No	86	0	0
0	0	Thoracotoı	49	PRIVE	No	80	0	0
0	1	Thoracotoı	49	PRIVE	No	61	1	0
0	0	VATS or rc	49	PRIVE	No	52	0	0
0	0	Thoracotoı	49	PRIVE	No	63	0	0
0	0	Thoracotoı	49	PRIVE	No	84	1	0
0	0	Thoracotoı	49	PRIVE	No	66	0	0
0	0	Thoracotoı	49	PRIVE	No	66	0	0
0	1	Thoracotoı	49	PRIVE	No	62	0	0
0	0	Thoracotoı	49	PRIVE	No	65	0	0
0	0	Thoracotoı	49	PRIVE	No	69	0	0
0	0	Thoracotoı	49	PRIVE	No	73	0	0
0	0	Thoracotoı	49	PRIVE	No	69	0	0
0	0	Thoracotoı	49	PRIVE	No	50	0	0
0	0	Thoracotoı	49	PRIVE	No	54	1	0
0	1	Thoracotoı	49	PRIVE	No	65	1	0
0	0	VATS or rc	49	PRIVE	No	59	0	0
0	0	Thoracotoı	49	PRIVE	No	58	1	0
0	0	Thoracotoı	49	PRIVE	No	62	1	0
0	0	Thoracotoı	49	PRIVE	No	70	0	0
0	0	Thoracotoı	49	PRIVE	No	62	0	0
0	0	Thoracotoı	49	PRIVE	No	54	1	0
0	0	Thoracotoı	49	PRIVE	No	81	0	0
0	0	Thoracotoı	49	PRIVE	No	75	0	0
0	0	VATS or rc	49	PRIVE	No	67	0	0
0	0	Thoracotoı	49	PRIVE	No	71	0	0
0	0	VATS or rc	49	PRIVE	No	67	0	0
0	0	Thoracotoı	49	PRIVE	No	85	1	0
0	0	VATS or rc	49	PRIVE	No	63	0	0
0	0	Thoracotoı	49	PRIVE	No	62	0	0

0	0	Thoracotoı	49	PRIVE	No	54	0	0
0	0	Thoracotoı	49	PRIVE	No	61	0	0
0	0	VATS or rc	49	PRIVE	No	59	0	0
0	0	Thoracotoı	49	PRIVE	No	45	0	0
0	0	Thoracotoı	49	PRIVE	No	68	0	0
0	0	Thoracotoı	49	PRIVE	No	74	0	0
0	0	Thoracotoı	49	PRIVE	No	78	1	0
0	1	Thoracotoı	49	PRIVE	No	81	0	0
0	1	Thoracotoı	49	PRIVE	No	56	0	1
0	0	Thoracotoı	49	PRIVE	No	64	0	0
0	1	Thoracotoı	49	PRIVE	No	69	0	0
0	0	Thoracotoı	49	PRIVE	No	71	0	0
0	0	VATS or rc	49	PRIVE	No	48	0	0
0	0	Thoracotoı	49	PRIVE	No	78	1	0
0	0	Thoracotoı	49	PRIVE	No	70	0	0
0	0	VATS or rc	49	PRIVE	No	68	0	0
0	0	Thoracotoı	49	PRIVE	No	63	1	0
0	0	Thoracotoı	49	PRIVE	No	80	0	0
0	0	Thoracotoı	49	PRIVE	No	70	0	0
0	0	Thoracotoı	49	PRIVE	No	67	0	0
0	0	Thoracotoı	49	PRIVE	No	67	0	0
0	0	Thoracotoı	49	PRIVE	No	62	0	0
0	0	Thoracotoı	49	PRIVE	No	62	0	0
0	0	VATS or rc	49	PRIVE	No	78	0	0
0	0	Thoracotoı	49	PRIVE	No	72	0	0
0	0	Thoracotoı	49	PRIVE	No	62	0	0
0	0	Thoracotoı	49	PRIVE	No	59	0	0
0	0	VATS or rc	49	PRIVE	No	59	0	0
0	0	Thoracotoı	49	PRIVE	No	76	0	0
0	0	Thoracotoı	49	PRIVE	No	78	0	0
0	0	Thoracotoı	49	PRIVE	No	63	0	0
0	0	Thoracotoı	49	PRIVE	No	72	0	0
0	0	Thoracotoı	49	PRIVE	No	71	1	0
0	0	Thoracotoı	49	PRIVE	No	60	0	0
0	0	Thoracotoı	49	PRIVE	No	57	0	0
0	0	Thoracotoı	49	PRIVE	No	83	0	0
0	0	Thoracotoı	49	PRIVE	No	79	0	0
0	0	Thoracotoı	49	PRIVE	No	68	1	0
0	0	Thoracotoı	49	PRIVE	No	77	0	0
0	0	Thoracotoı	49	PRIVE	No	70	0	0
0	0	Thoracotoı	49	PRIVE	No	62	1	0
0	0	Thoracotoı	49	PRIVE	No	79	0	0
0	0	Thoracotoı	49	PRIVE	No	65	0	0
0	0	Thoracotoı	49	PRIVE	No	62	1	0
0	0	Thoracotoı	49	PRIVE	No	71	0	0
0	0	VATS or rc	49	PRIVE	No	57	1	0
0	0	Thoracotoı	49	PRIVE	No	74	0	0
0	0	VATS or rc	49	PRIVE	No	80	0	0
0	0	Thoracotoı	49	PRIVE	No	66	0	0
0	0	VATS or rc	49	PRIVE	No	79	0	0
0	0	Thoracotoı	49	PRIVE	No	75	0	0
0	0	Thoracotoı	49	PRIVE	No	61	0	0
0	0	Thoracotoı	49	PRIVE	No	75	0	0
0	0	Thoracotoı	49	PRIVE	No	74	0	0
0	0	VATS or rc	49	PRIVE	No	70	0	0
0	0	Thoracotoı	49	PRIVE	No	79	0	0

0	0	Thoracotoı	49	PRIVE	No	78	0	0
0	0	Thoracotoı	49	PRIVE	No	54	0	0
0	0	Thoracotoı	49	PRIVE	No	70	0	0
0	0	VATS or rc	49	PRIVE	No	59	0	0
0	0	Thoracotoı	49	PRIVE	No	64	0	0
0	0	Thoracotoı	49	PRIVE	No	71	0	0
0	0	Thoracotoı	49	PRIVE	No	61	0	0
0	0	Thoracotoı	49	PRIVE	No	61	0	0
0	0	Thoracotoı	49	PRIVE	No	70	0	0
0	0	Thoracotoı	49	PRIVE	No	61	0	0
0	0	VATS or rc	49	PRIVE	No	65	1	0
0	0	Thoracotoı	49	PRIVE	No	77	0	0
0	0	Thoracotoı	49	PRIVE	No	66	0	0
0	0	Thoracotoı	49	PRIVE	No	67	0	0
0	0	Thoracotoı	49	PRIVE	No	69	0	0
0	0	Thoracotoı	49	PRIVE	No	71	0	0
0	0	Thoracotoı	49	PRIVE	No	74	0	0
0	0	Thoracotoı	49	PRIVE	No	59	0	0
0	0	VATS or rc	49	PRIVE	No	53	0	0
0	0	VATS or rc	49	PRIVE	No	59	0	0
0	0	Thoracotoı	49	PRIVE	No	50	0	0
0	0	Thoracotoı	49	PRIVE	No	70	0	0
0	0	Thoracotoı	49	PRIVE	No	64	0	0
0	0	Thoracotoı	49	PRIVE	No	65	1	0
0	0	VATS or rc	49	PRIVE	No	67	0	0
0	0	Thoracotoı	49	PRIVE	No	60	1	0
0	0	Thoracotoı	49	PRIVE	No	62	0	0
0	1	Thoracotoı	49	PRIVE	No	37	0	0
0	0	VATS or rc	49	PRIVE	No	66	0	0
0	1	Thoracotoı	49	PRIVE	No	66	1	0
0	0	Thoracotoı	49	PRIVE	No	65	0	0
0	0	Thoracotoı	49	PRIVE	No	79	0	0
0	1	Thoracotoı	49	PRIVE	No	55	0	0
0	1	Thoracotoı	49	PRIVE	Yes	75	0	0
0	0	Thoracotoı	49	PRIVE	No	68	0	0
0	0	Thoracotoı	49	PRIVE	No	75	1	0
0	0	Thoracotoı	49	PRIVE	No	77	0	0
0	0	Thoracotoı	49	PRIVE	No	72	0	0
0	0	Thoracotoı	49	PRIVE	Yes	71	1	0
0	0	Thoracotoı	49	PRIVE	No	43	0	0
0	0	Thoracotoı	49	PRIVE	No	86	0	0
0	0	Thoracotoı	49	PRIVE	No	66	0	0
0	0	Thoracotoı	49	PRIVE	No	69	0	0
0	0	Thoracotoı	49	PRIVE	No	66	0	0
0	0	Thoracotoı	49	PRIVE	No	68	0	0
0	0	Thoracotoı	49	PRIVE	No	68	1	0
0	0	VATS or rc	49	PRIVE	No	69	0	0
0	0	Thoracotoı	49	PRIVE	No	74	1	0
0	0	Thoracotoı	49	PRIVE	No	67	1	0
0	0	Thoracotoı	49	PRIVE	No	66	1	0
0	1	Thoracotoı	49	PRIVE	No	80	0	0
0	0	Thoracotoı	49	PRIVE	No	77	0	0
0	0	Thoracotoı	49	PRIVE	No	68	0	0
0	0	VATS or rc	49	PRIVE	No	63	0	0
0	1	Thoracotoı	49	PRIVE	No	69	0	0
0	1	Thoracotoı	49	PRIVE	No	73	1	0

0	0	Thoracotoı	49	PRIVE	No	56	0	0
0	1	Thoracotoı	49	PRIVE	No	65	0	0
0	0	Thoracotoı	49	PRIVE	No	78	0	0
0	0	Thoracotoı	49	PRIVE	No	63	0	0
0	0	Thoracotoı	49	PRIVE	No	65	0	0
0	0	Thoracotoı	49	PRIVE	No	57	0	0
0	0	Thoracotoı	49	PRIVE	No	64	0	0
0	0	Thoracotoı	49	PRIVE	No	60	0	0
0	1	Thoracotoı	49	PRIVE	No	80	1	0
0	0	VATS or rc	49	PRIVE	No	75	0	0
0	0	Thoracotoı	49	PRIVE	No	87	0	0
0	1	Thoracotoı	49	PRIVE	No	60	0	0
0	0	Thoracotoı	49	PRIVE	No	54	1	0
0	0	Thoracotoı	49	PRIVE	No	44	0	0
0	1	Thoracotoı	49	PRIVE	No	44	0	0
0	0	Thoracotoı	49	PRIVE	No	68	0	0
0	1	Thoracotoı	49	PRIVE	No	67	1	0
0	0	Thoracotoı	49	PRIVE	No	70	0	0
0	0	Thoracotoı	49	PRIVE	No	62	1	0
0	0	Thoracotoı	49	PRIVE	No	50	0	0
0	0	Thoracotoı	49	PRIVE	No	47	0	0
0	0	Thoracotoı	49	PRIVE	No	68	0	0
0	0	Thoracotoı	49	PRIVE	No	49	0	0
0	0	VATS or rc	49	PRIVE	No	82	1	0
0	0	Thoracotoı	49	PRIVE	No	65	0	0
0	0	Thoracotoı	49	PRIVE	No	52	0	0
0	0	Thoracotoı	49	PRIVE	No	74	0	0
0	0	VATS or rc	49	PRIVE	No	72	0	0
0	0	Thoracotoı	49	PRIVE	No	57	1	0
0	0	Thoracotoı	49	PRIVE	No	57	1	0
0	1	Thoracotoı	49	PRIVE	No	71	0	0
0	0	VATS or rc	49	PRIVE	No	57	0	0
0	0	VATS or rc	49	PRIVE	No	40	0	0
0	0	Thoracotoı	49	PRIVE	No	64	0	0
0	0	VATS or rc	49	PRIVE	No	68	0	0
0	0	Thoracotoı	49	PRIVE	No	61	0	0
0	0	VATS or rc	49	PRIVE	No	40	0	0
0	0	Thoracotoı	49	PRIVE	No	58	0	0
0	0	VATS or rc	49	PRIVE	No	50	0	0
0	0	Thoracotoı	49	PRIVE	No	81	0	0
0	0	Thoracotoı	49	PRIVE	No	52	0	0
0	0	VATS or rc	49	PRIVE	No	64	0	0
0	0	Thoracotoı	49	PRIVE	No	83	0	0
0	0	Thoracotoı	49	PRIVE	No	67	0	0
0	0	Thoracotoı	49	PRIVE	No	59	0	0
0	1	Thoracotoı	49	PRIVE	No	60	0	0
0	0	VATS or rc	49	PRIVE	No	67	0	0
0	0	VATS or rc	49	PRIVE	No	56	0	0
0	0	Thoracotoı	49	PRIVE	No	54	0	0
0	0	Thoracotoı	49	PRIVE	No	58	0	0
0	0	Thoracotoı	49	PRIVE	Yes	61	1	0
0	0	Thoracotoı	49	PRIVE	No	71	0	0
0	0	VATS or rc	49	PRIVE	No	54	0	0
0	0	Thoracotoı	49	PRIVE	No	68	0	0
0	0	Thoracotoı	49	PRIVE	No	49	0	0
0	0	VATS or rc	49	PRIVE	No	65	0	0

0	0	Thoracotoı	49	PRIVE	No	66	1	0
0	0	Thoracotoı	49	PRIVE	No	70	0	0
0	0	Thoracotoı	49	PRIVE	No	66	0	0
0	0	Thoracotoı	49	PRIVE	No	64	0	0
0	0	Thoracotoı	49	PRIVE	No	67	0	0
0	0	Thoracotoı	49	PRIVE	No	77	0	0
0	0	VATS or rc	49	PRIVE	No	78	0	0
0	0	Thoracotoı	49	PRIVE	No	72	0	0
0	0	Thoracotoı	49	PRIVE	No	80	0	0
0	1	Thoracotoı	49	PRIVE	No	65	0	0
0	0	Thoracotoı	49	PRIVE	No	67	0	0
0	1	Thoracotoı	49	PRIVE	No	75	1	0
0	1	Thoracotoı	49	PRIVE	No	79	0	0
0	0	VATS or rc	49	PRIVE	No	63	0	0
0	0	VATS or rc	49	PRIVE	No	59	0	0
0	1	Thoracotoı	49	PRIVE	No	58	0	0
0	0	Thoracotoı	49	PRIVE	No	75	0	0
0	0	Thoracotoı	49	PRIVE	No	83	0	0
0	0	Thoracotoı	49	PRIVE	No	69	0	0
0	0	Thoracotoı	49	PRIVE	No	67	0	0
0	0	Thoracotoı	49	PRIVE	No	67	0	0
0	0	Thoracotoı	49	PRIVE	No	56	1	0
0	1	Thoracotoı	49	PRIVE	No	72	0	0
0	0	Thoracotoı	49	PRIVE	No	47	0	0
0	0	Thoracotoı	49	PRIVE	No	79	0	0
0	0	Thoracotoı	49	PRIVE	No	77	1	0
0	0	Thoracotoı	49	PRIVE	No	56	0	0
0	0	Thoracotoı	49	PRIVE	No	64	0	0
0	0	Thoracotoı	49	PRIVE	No	65	0	0
0	0	VATS or rc	49	PRIVE	No	63	0	0
0	1	Thoracotoı	49	PRIVE	No	75	0	0
0	0	VATS or rc	49	PRIVE	No	67	0	0
0	0	VATS or rc	49	PRIVE	No	76	0	0
0	0	Thoracotoı	49	PRIVE	No	59	0	0
0	0	VATS or rc	49	PRIVE	No	52	0	0
0	0	Thoracotoı	49	PRIVE	No	44	0	0
0	0	Thoracotoı	49	PRIVE	No	84	1	0
0	0	Thoracotoı	49	PRIVE	No	75	0	1
0	0	Thoracotoı	49	PRIVE	No	59	0	0
0	0	Thoracotoı	49	PRIVE	No	61	0	0
0	0	VATS or rc	49	PRIVE	No	81	0	0
0	0	Thoracotoı	49	PRIVE	No	68	0	0
0	0	Thoracotoı	49	PRIVE	No	58	0	0
0	0	Thoracotoı	49	PRIVE	No	72	0	0
0	0	Thoracotoı	49	PRIVE	No	64	0	0
0	0	Thoracotoı	49	PRIVE	No	79	1	0
0	0	Thoracotoı	49	PRIVE	No	73	0	0
0	0	Thoracotoı	49	PRIVE	No	55	0	0
0	0	Thoracotoı	49	PRIVE	No	82	0	0
0	1	Thoracotoı	49	PRIVE	No	73	1	0
0	0	VATS or rc	49	PRIVE	No	66	1	0
0	0	Thoracotoı	205	CHU	No	61	0	1
0	1	Thoracotoı	205	CHU	No	58	0	0
0	0	Thoracotoı	205	CHU	No	68	0	1
0	0	Thoracotoı	205	CHU	No	55	0	0
0	0	Thoracotoı	205	CHU	No	60	0	0

0	0	Thoracotoı	205	CHU	No	63	0	0
0	0	Thoracotoı	205	CHU	No	62	0	0
0	0	Thoracotoı	205	CHU	No	74	0	0
0	0	Thoracotoı	205	CHU	No	62	0	0
0	0	Thoracotoı	205	CHU	No	72	0	0
0	0	Thoracotoı	205	CHU	No	58	0	1
0	0	VATS or rc	205	CHU	No	61	0	1
0	0	Thoracotoı	205	CHU	No	68	0	0
0	0	Thoracotoı	205	CHU	No	66	1	0
0	0	Thoracotoı	205	CHU	No	52	1	1
0	1	Thoracotoı	205	CHU	No	65	0	0
0	0	Thoracotoı	205	CHU	No	59	0	1
0	0	VATS or rc	205	CHU	No	78	0	1
0	1	Thoracotoı	205	CHU	No	48	0	1
0	0	VATS or rc	205	CHU	No	65	0	1
0	0	Thoracotoı	205	CHU	No	50	0	1
0	0	Thoracotoı	205	CHU	No	33	0	1
0	0	Thoracotoı	205	CHU	No	69	0	0
0	0	Thoracotoı	205	CHU	No	63	0	1
0	0	Thoracotoı	205	CHU	No	53	0	0
0	0	VATS or rc	205	CHU	No	45	0	1
0	1	Thoracotoı	205	CHU	No	78	0	0
0	0	Thoracotoı	205	CHU	No	70	0	0
0	0	Thoracotoı	205	CHU	No	75	0	1
0	0	Thoracotoı	205	CHU	No	52	0	1
0	0	Thoracotoı	205	CHU	No	52	0	0
0	0	Thoracotoı	205	CHU	No	59	0	0
0	0	Thoracotoı	205	CHU	No	69	1	0
0	0	Thoracotoı	205	CHU	No	62	0	0
0	0	Thoracotoı	205	CHU	No	54	0	0
0	0	Thoracotoı	205	CHU	No	67	0	1
0	0	Thoracotoı	205	CHU	No	75	0	1
0	1	Thoracotoı	205	CHU	Yes	79	0	1
0	0	Thoracotoı	205	CHU	No	71	0	0
0	0	Thoracotoı	205	CHU	No	74	0	0
0	1	Thoracotoı	205	CHU	No	77	0	0
0	0	Thoracotoı	205	CHU	No	69	0	0
0	0	VATS or rc	205	CHU	No	58	0	0
0	0	Thoracotoı	205	CHU	No	67	1	1
0	1	Thoracotoı	205	CHU	No	63	1	0
0	0	Thoracotoı	205	CHU	No	48	0	1
0	0	Thoracotoı	205	CHU	No	55	1	1
0	0	Thoracotoı	205	CHU	No	63	0	0
0	0	Thoracotoı	205	CHU	No	55	0	0
0	0	Thoracotoı	205	CHU	No	44	0	0
0	0	Thoracotoı	205	CHU	No	33	0	1
0	0	Thoracotoı	205	CHU	No	86	0	0
0	0	Thoracotoı	205	CHU	No	64	1	1
0	0	Thoracotoı	205	CHU	No	69	0	0
0	0	Thoracotoı	205	CHU	No	61	0	0
0	0	Thoracotoı	205	CHU	No	46	0	0
0	0	Thoracotoı	205	CHU	No	42	0	1
0	0	Thoracotoı	205	CHU	No	63	0	0
0	0	Thoracotoı	205	CHU	No	82	0	0
0	0	Thoracotoı	205	CHU	No	59	0	0
0	0	Thoracotoı	205	CHU	No	57	0	0

0	0	Thoracotoı	205	CHU	No	57	0	0
0	1	Thoracotoı	205	CHU	No	68	0	0
0	0	Thoracotoı	205	CHU	No	68	0	0
0	1	Thoracotoı	205	CHU	No	46	0	0
0	0	Thoracotoı	205	CHU	No	70	0	0
0	1	Thoracotoı	205	CHU	No	67	0	0
0	0	Thoracotoı	205	CHU	No	69	0	1
0	0	Thoracotoı	205	CHU	No	67	0	0
0	0	Thoracotoı	205	CHU	No	56	0	0
0	0	Thoracotoı	205	CHU	No	56	0	0
0	0	Thoracotoı	205	CHU	No	48	1	1
0	0	Thoracotoı	205	CHU	No	69	0	0
0	0	Thoracotoı	205	CHU	No	76	0	1
0	0	VATS or rc	205	CHU	No	58	0	0
0	1	Thoracotoı	205	CHU	No	64	1	1
0	0	Thoracotoı	205	CHU	No	81	0	0
0	0	Thoracotoı	205	CHU	No	74	0	0
0	0	Thoracotoı	205	CHU	No	75	0	0
0	1	Thoracotoı	205	CHU	No	69	0	0
0	0	Thoracotoı	205	CHU	No	53	0	0
0	0	Thoracotoı	205	CHU	No	68	0	0
0	1	Thoracotoı	205	CHU	No	63	0	0
0	0	Thoracotoı	205	CHU	No	60	0	0
0	0	Thoracotoı	205	CHU	No	64	0	0
0	0	Thoracotoı	205	CHU	No	57	0	0
0	0	Thoracotoı	205	CHU	No	52	0	1
0	0	Thoracotoı	205	CHU	No	64	0	1
0	0	Thoracotoı	205	CHU	No	69	0	0
0	0	Thoracotoı	205	CHU	No	68	1	0
0	0	Thoracotoı	205	CHU	No	70	1	0
0	0	Thoracotoı	205	CHU	No	66	0	0
0	0	Thoracotoı	205	CHU	No	63	0	0
0	1	Thoracotoı	205	CHU	No	62	1	1
0	0	Thoracotoı	205	CHU	No	73	0	1
0	0	Thoracotoı	205	CHU	No	63	0	0
0	0	Thoracotoı	205	CHU	No	73	0	0
0	0	Thoracotoı	205	CHU	No	53	0	1
0	0	Thoracotoı	205	CHU	No	84	0	0
0	0	Thoracotoı	205	CHU	No	65	0	0
0	0	Thoracotoı	205	CHU	No	62	0	1
0	0	Thoracotoı	205	CHU	No	66	0	0
0	0	VATS or rc	205	CHU	No	49	0	0
0	0	Thoracotoı	205	CHU	No	70	0	0
0	0	Thoracotoı	205	CHU	No	65	0	0
0	0	Thoracotoı	205	CHU	No	65	0	0
0	0	Thoracotoı	205	CHU	No	57	0	0
0	0	Thoracotoı	205	CHU	No	65	0	1
0	0	Thoracotoı	205	CHU	No	72	0	0
0	0	Thoracotoı	205	CHU	No	61	0	0
0	0	Thoracotoı	205	CHU	No	38	0	1
0	0	Thoracotoı	205	CHU	No	68	0	0
0	0	Thoracotoı	205	CHU	No	56	0	0
0	0	Thoracotoı	205	CHU	No	63	1	0
0	0	VATS or rc	205	CHU	No	60	0	0
0	0	Thoracotoı	205	CHU	No	76	0	1
0	0	Thoracotoı	205	CHU	No	45	0	0

0	0	Thoracotoı	205	CHU	No	79	0	1
0	0	Thoracotoı	205	CHU	No	73	0	0
0	0	Thoracotoı	205	CHU	No	53	0	0
0	0	Thoracotoı	205	CHU	No	74	0	0
0	0	Thoracotoı	205	CHU	No	64	0	1
0	0	VATS or rc	205	CHU	No	65	0	1
0	0	Thoracotoı	205	CHU	No	66	0	0
0	1	Thoracotoı	205	CHU	No	57	0	1
0	0	Thoracotoı	205	CHU	No	57	0	0
0	0	Thoracotoı	205	CHU	No	73	0	1
0	0	Thoracotoı	205	CHU	No	65	0	0
0	0	Thoracotoı	205	CHU	No	67	0	1
0	0	VATS or rc	205	CHU	No	45	0	1
0	0	Thoracotoı	205	CHU	No	58	1	1
0	1	Thoracotoı	205	CHU	No	64	0	1
0	0	Thoracotoı	205	CHU	No	75	0	0
0	0	Thoracotoı	205	CHU	No	56	0	0
0	0	Thoracotoı	205	CHU	No	59	0	0
0	0	Thoracotoı	205	CHU	No	75	0	0
0	0	VATS or rc	205	CHU	No	79	1	0
0	0	Thoracotoı	205	CHU	No	60	0	0
0	0	Thoracotoı	205	CHU	No	78	1	0
0	1	Thoracotoı	205	CHU	No	58	0	0
0	0	Thoracotoı	205	CHU	No	58	0	1
0	0	Thoracotoı	205	CHU	No	59	0	1
0	0	Thoracotoı	205	CHU	No	63	0	0
0	0	Thoracotoı	205	CHU	No	72	0	1
0	0	Thoracotoı	205	CHU	No	62	0	0
0	0	Thoracotoı	205	CHU	No	61	1	0
0	0	Thoracotoı	205	CHU	No	61	0	0
0	0	Thoracotoı	205	CHU	No	56	0	0
0	0	Thoracotoı	205	CHU	No	81	0	0
0	0	Thoracotoı	205	CHU	No	60	0	1
0	0	Thoracotoı	205	CHU	No	56	0	1
0	0	Thoracotoı	205	CHU	No	70	1	1
0	0	VATS or rc	205	CHU	No	78	0	1
0	0	VATS or rc	205	CHU	No	74	0	0
0	0	Thoracotoı	205	CHU	No	69	0	0
0	0	Thoracotoı	205	CHU	No	62	0	0
0	0	Thoracotoı	205	CHU	Yes	70	0	0
0	0	Thoracotoı	205	CHU	No	89	0	0
0	0	Thoracotoı	205	CHU	No	65	0	0
0	0	Thoracotoı	205	CHU	No	63	1	0
0	0	VATS or rc	104	PRIVE	No	69	0	1
0	0	VATS or rc	104	PRIVE	No	54	0	0
0	0	Thoracotoı	104	PRIVE	No	52	0	1
0	0	Thoracotoı	104	PRIVE	Yes	76	0	0
0	0	VATS or rc	104	PRIVE	No	55	0	0
0	0	VATS or rc	104	PRIVE	No	52	0	1
0	0	VATS or rc	104	PRIVE	No	70	0	0
0	0	VATS or rc	104	PRIVE	No	60	1	0
0	0	Thoracotoı	104	PRIVE	No	65	0	1
0	0	VATS or rc	104	PRIVE	No	58	0	1
0	0	VATS or rc	104	PRIVE	Yes	81	0	0
0	0	Thoracotoı	104	PRIVE	No	62	0	0
0	0	VATS or rc	104	PRIVE	No	54	1	1

0	0	VATS or rc	104	PRIVE	No	76	0	0
0	0	VATS or rc	104	PRIVE	No	70	0	0
0	0	Thoracotoı	104	PRIVE	No	64	0	0
0	0	Thoracotoı	104	PRIVE	No	82	0	0
0	0	Thoracotoı	104	PRIVE	No	49	0	0
0	0	Thoracotoı	104	PRIVE	No	61	0	0
0	0	Thoracotoı	104	PRIVE	Yes	71	0	0
0	0	Thoracotoı	104	PRIVE	No	62	0	1
0	0	Thoracotoı	104	PRIVE	Yes	64	0	0
0	0	VATS or rc	104	PRIVE	No	59	0	0
0	0	VATS or rc	104	PRIVE	No	55	1	1
0	0	VATS or rc	104	PRIVE	No	66	0	0
0	0	Thoracotoı	104	PRIVE	No	53	0	0
0	0	Thoracotoı	104	PRIVE	No	62	0	0
0	0	VATS or rc	104	PRIVE	No	68	0	1
0	0	VATS or rc	104	PRIVE	No	67	0	0
0	0	VATS or rc	104	PRIVE	No	50	0	0
0	0	VATS or rc	104	PRIVE	No	62	0	1
0	0	VATS or rc	104	PRIVE	No	61	0	0
0	0	Thoracotoı	104	PRIVE	No	62	0	0
0	0	Thoracotoı	104	PRIVE	No	61	0	1
0	0	VATS or rc	104	PRIVE	No	71	0	0
0	0	VATS or rc	104	PRIVE	No	77	0	0
0	0	Thoracotoı	104	PRIVE	No	76	0	0
0	0	Thoracotoı	104	PRIVE	No	67	0	0
0	0	VATS or rc	104	PRIVE	No	68	0	0
0	0	VATS or rc	104	PRIVE	No	59	0	0
0	0	VATS or rc	104	PRIVE	No	49	0	0
0	0	VATS or rc	104	PRIVE	No	63	0	0
0	0	Thoracotoı	104	PRIVE	No	68	1	0
0	0	VATS or rc	104	PRIVE	No	76	0	0
0	0	VATS or rc	104	PRIVE	No	68	0	0
0	0	Thoracotoı	104	PRIVE	No	70	0	0
0	0	Thoracotoı	104	PRIVE	No	72	0	0
0	0	VATS or rc	104	PRIVE	No	72	0	0
0	0	VATS or rc	104	PRIVE	No	65	0	0
0	0	Thoracotoı	104	PRIVE	No	28	0	0
0	1	Thoracotoı	104	PRIVE	No	74	0	0
0	0	Thoracotoı	104	PRIVE	No	85	0	0
0	1	Thoracotoı	104	PRIVE	Yes	46	0	1
0	0	VATS or rc	104	PRIVE	No	88	1	0
0	0	VATS or rc	104	PRIVE	No	67	0	0
0	1	Thoracotoı	104	PRIVE	No	61	0	0
0	0	VATS or rc	104	PRIVE	No	73	0	0
0	0	Thoracotoı	104	PRIVE	No	56	0	1
0	0	Thoracotoı	104	PRIVE	No	66	0	1
0	0	VATS or rc	104	PRIVE	No	74	0	0
0	0	VATS or rc	104	PRIVE	No	66	0	0
0	0	VATS or rc	104	PRIVE	No	73	0	0
0	0	Thoracotoı	104	PRIVE	No	56	0	1
0	0	VATS or rc	104	PRIVE	No	64	1	1
0	0	Thoracotoı	104	PRIVE	No	67	0	0
0	0	VATS or rc	104	PRIVE	No	59	0	0
0	0	VATS or rc	104	PRIVE	No	79	0	0
0	0	VATS or rc	104	PRIVE	No	52	0	0
0	0	VATS or rc	104	PRIVE	No	68	0	0

0	0	Thoracotoı	104	PRIVE	No	64	0	0
0	0	VATS or rc	104	PRIVE	No	71	0	0
0	0	VATS or rc	104	PRIVE	No	69	0	0
0	0	Thoracotoı	104	PRIVE	No	62	1	0
0	0	Thoracotoı	104	PRIVE	No	63	0	0
0	0	VATS or rc	104	PRIVE	No	65	0	0
0	0	VATS or rc	104	PRIVE	No	69	0	0
0	1	Thoracotoı	104	PRIVE	No	61	0	0
0	0	VATS or rc	104	PRIVE	No	69	0	1
0	0	VATS or rc	104	PRIVE	No	63	1	1
0	0	VATS or rc	104	PRIVE	No	78	0	0
0	0	VATS or rc	104	PRIVE	No	52	0	0
0	0	Thoracotoı	104	PRIVE	No	76	1	0
0	0	Thoracotoı	104	PRIVE	No	58	0	0
0	0	VATS or rc	104	PRIVE	No	77	0	0
0	0	VATS or rc	104	PRIVE	No	45	0	1
0	0	VATS or rc	104	PRIVE	No	73	0	1
0	0	VATS or rc	104	PRIVE	No	70	0	0
0	0	VATS or rc	104	PRIVE	Yes	78	1	1
0	0	VATS or rc	104	PRIVE	No	81	1	0
0	0	VATS or rc	104	PRIVE	No	66	0	0
0	0	VATS or rc	104	PRIVE	No	70	0	0
0	0	VATS or rc	104	PRIVE	No	71	0	0
0	0	VATS or rc	104	PRIVE	No	56	0	0
0	0	VATS or rc	104	PRIVE	No	65	0	0
0	0	VATS or rc	104	PRIVE	No	65	1	0
0	0	VATS or rc	104	PRIVE	No	67	0	0
0	0	Thoracotoı	104	PRIVE	No	76	0	0
0	0	Thoracotoı	104	PRIVE	No	57	0	0
0	0	Thoracotoı	104	PRIVE	No	52	0	0
0	0	VATS or rc	104	PRIVE	No	61	0	0
0	0	VATS or rc	104	PRIVE	No	72	0	0
0	0	Thoracotoı	104	PRIVE	No	62	0	0
0	0	VATS or rc	104	PRIVE	No	71	0	1
0	0	Thoracotoı	104	PRIVE	No	64	0	0
0	0	VATS or rc	104	PRIVE	No	77	0	0
0	0	VATS or rc	104	PRIVE	No	74	1	0
0	0	Thoracotoı	104	PRIVE	No	56	0	1
0	0	Thoracotoı	104	PRIVE	No	69	0	0
0	1	Thoracotoı	104	PRIVE	No	66	0	0
0	0	Thoracotoı	104	PRIVE	No	59	0	1
0	0	VATS or rc	104	PRIVE	No	50	0	0
0	0	VATS or rc	104	PRIVE	No	57	0	0
0	0	Thoracotoı	104	PRIVE	No	78	0	0
0	0	VATS or rc	104	PRIVE	No	64	1	0
0	0	VATS or rc	104	PRIVE	No	74	0	0
0	0	Thoracotoı	104	PRIVE	No	68	0	0
0	0	Thoracotoı	104	PRIVE	No	66	0	1
0	0	Thoracotoı	104	PRIVE	No	67	0	0
0	0	VATS or rc	104	PRIVE	No	64	0	0
0	0	Thoracotoı	104	PRIVE	Yes	81	0	0
0	0	VATS or rc	104	PRIVE	Yes	71	0	0
0	0	VATS or rc	104	PRIVE	Yes	63	0	0
0	0	VATS or rc	104	PRIVE	No	76	0	0
0	0	VATS or rc	104	PRIVE	No	63	0	0
0	0	VATS or rc	104	PRIVE	No	68	0	0

0	0	Thoracotoı	104	PRIVE	No	65	1	0
0	0	VATS or rc	104	PRIVE	No	59	0	0
0	0	VATS or rc	104	PRIVE	No	65	0	1
0	0	VATS or rc	104	PRIVE	No	64	0	0
0	0	VATS or rc	104	PRIVE	No	55	0	0
0	0	Thoracotoı	104	PRIVE	No	61	1	1
0	0	VATS or rc	104	PRIVE	No	67	0	0
0	0	Thoracotoı	104	PRIVE	No	53	0	1
0	0	Thoracotoı	104	PRIVE	No	56	0	1
0	0	VATS or rc	104	PRIVE	No	65	0	0
0	0	VATS or rc	104	PRIVE	No	70	1	0
0	0	Thoracotoı	104	PRIVE	No	55	0	1
0	0	VATS or rc	104	PRIVE	No	62	0	1
0	0	Thoracotoı	104	PRIVE	No	70	0	0
0	0	VATS or rc	104	PRIVE	No	58	0	1
0	0	VATS or rc	104	PRIVE	No	66	0	0
0	0	VATS or rc	104	PRIVE	No	66	0	0
0	0	VATS or rc	104	PRIVE	No	59	0	0
0	0	VATS or rc	104	PRIVE	No	58	0	1
0	0	VATS or rc	104	PRIVE	No	67	0	0
0	0	Thoracotoı	104	PRIVE	No	52	0	0
0	0	VATS or rc	104	PRIVE	No	75	0	1
0	0	Thoracotoı	104	PRIVE	No	60	0	0
0	0	VATS or rc	104	PRIVE	No	50	0	0
0	0	VATS or rc	104	PRIVE	No	79	0	0
0	0	VATS or rc	104	PRIVE	No	60	0	0
0	0	VATS or rc	104	PRIVE	No	71	0	0
0	0	VATS or rc	104	PRIVE	No	60	0	0
0	0	VATS or rc	104	PRIVE	No	74	0	0
0	0	VATS or rc	104	PRIVE	No	46	0	1
0	0	VATS or rc	104	PRIVE	No	59	0	1
0	0	Thoracotoı	104	PRIVE	No	55	0	0
0	0	Thoracotoı	104	PRIVE	No	78	0	0
0	0	Thoracotoı	104	PRIVE	No	59	0	0
0	0	Thoracotoı	104	PRIVE	Yes	70	1	0
0	0	VATS or rc	104	PRIVE	No	84	0	0
0	0	VATS or rc	104	PRIVE	No	69	0	0
0	0	VATS or rc	104	PRIVE	No	62	0	1
0	0	VATS or rc	104	PRIVE	No	68	0	0
0	0	VATS or rc	104	PRIVE	No	66	0	0
0	0	VATS or rc	104	PRIVE	No	64	0	1
0	0	VATS or rc	104	PRIVE	No	77	0	0
0	0	Thoracotoı	104	PRIVE	No	58	0	0
0	0	VATS or rc	104	PRIVE	No	67	0	0
0	1	Thoracotoı	104	PRIVE	No	67	1	0
0	0	Thoracotoı	104	PRIVE	No	60	0	0
0	0	VATS or rc	104	PRIVE	No	80	0	0
0	0	VATS or rc	104	PRIVE	No	52	0	1
0	0	VATS or rc	104	PRIVE	No	61	0	0
0	0	VATS or rc	104	PRIVE	No	69	0	0
0	0	VATS or rc	104	PRIVE	No	59	0	1
0	0	VATS or rc	104	PRIVE	No	58	0	1
0	0	VATS or rc	104	PRIVE	No	71	0	0
0	0	VATS or rc	104	PRIVE	No	65	0	0
0	1	VATS or rc	104	PRIVE	No	77	1	0
0	0	Thoracotoı	104	PRIVE	No	44	0	1

0	0	Thoracotoı	104	PRIVE	No	70	0	0
0	0	VATS or rc	104	PRIVE	No	70	1	0
0	0	Thoracotoı	104	PRIVE	No	71	1	0
0	0	Thoracotoı	104	PRIVE	Yes	71	0	0
0	0	VATS or rc	54	PRIVE	No	58	0	0
0	0	VATS or rc	54	PRIVE	No	68	1	0
0	0	Thoracotoı	54	PRIVE	No	51	0	0
0	0	VATS or rc	54	PRIVE	No	58	0	0
0	0	VATS or rc	54	PRIVE	No	69	0	1
0	0	Thoracotoı	54	PRIVE	No	67	0	0
0	0	VATS or rc	54	PRIVE	No	64	0	0
0	0	Thoracotoı	54	PRIVE	No	71	1	0
0	0	VATS or rc	54	PRIVE	No	68	0	0
0	0	Thoracotoı	54	PRIVE	No	71	1	0
0	0	Thoracotoı	54	PRIVE	No	67	0	0
0	0	Thoracotoı	54	PRIVE	No	57	1	0
0	0	Thoracotoı	54	PRIVE	No	72	0	0
0	0	Thoracotoı	54	PRIVE	No	80	0	0
0	0	VATS or rc	54	PRIVE	No	68	1	0
0	0	VATS or rc	54	PRIVE	No	62	1	0
0	0	Thoracotoı	54	PRIVE	No	68	0	0
0	0	VATS or rc	54	PRIVE	No	57	0	0
0	0	VATS or rc	54	PRIVE	No	68	0	0
0	0	VATS or rc	54	PRIVE	No	65	0	0
0	0	VATS or rc	54	PRIVE	No	74	1	0
0	0	VATS or rc	54	PRIVE	No	71	0	0
0	1	VATS or rc	54	PRIVE	No	69	0	0
0	0	VATS or rc	54	PRIVE	No	61	0	1
0	0	Thoracotoı	54	PRIVE	No	55	0	0
0	0	Thoracotoı	54	PRIVE	No	79	0	0
0	0	VATS or rc	54	PRIVE	No	71	1	0
0	0	Thoracotoı	54	PRIVE	No	64	0	0
0	0	Thoracotoı	54	PRIVE	No	73	0	0
0	0	VATS or rc	54	PRIVE	No	77	0	0
0	0	VATS or rc	54	PRIVE	No	72	0	0
0	0	VATS or rc	54	PRIVE	No	58	0	0
0	0	VATS or rc	54	PRIVE	No	67	0	0
0	1	VATS or rc	54	PRIVE	No	61	0	0
0	0	VATS or rc	54	PRIVE	No	82	0	0
0	0	VATS or rc	54	PRIVE	No	68	0	0
0	0	Thoracotoı	54	PRIVE	No	67	0	0
0	0	VATS or rc	54	PRIVE	No	57	1	0
0	0	Thoracotoı	54	PRIVE	No	66	0	0
0	0	Thoracotoı	54	PRIVE	No	62	1	0
0	0	VATS or rc	54	PRIVE	No	70	0	0
0	0	Thoracotoı	54	PRIVE	No	45	0	0
0	0	Thoracotoı	54	PRIVE	No	69	1	0
0	1	Thoracotoı	54	PRIVE	No	49	0	0
0	0	VATS or rc	54	PRIVE	No	64	0	0
0	0	Thoracotoı	54	PRIVE	No	58	0	0
0	0	Thoracotoı	54	PRIVE	No	71	0	0
0	0	VATS or rc	54	PRIVE	No	67	0	0
0	0	VATS or rc	54	PRIVE	No	71	0	0
0	0	VATS or rc	54	PRIVE	No	66	1	0
0	0	VATS or rc	54	PRIVE	No	44	0	0
0	0	VATS or rc	54	PRIVE	No	77	1	0

0	0	VATS or ro	54	PRIVE	No	58	0	0
0	0	Thoracotoı	54	PRIVE	No	67	1	0
0	0	VATS or ro	54	PRIVE	No	51	0	0
0	0	Thoracotoı	54	PRIVE	No	63	0	0
0	0	Thoracotoı	54	PRIVE	No	63	1	0
0	0	VATS or ro	54	PRIVE	No	61	1	0
0	0	VATS or ro	54	PRIVE	No	55	0	1
0	0	Thoracotoı	54	PRIVE	No	58	0	0
0	0	VATS or ro	54	PRIVE	No	62	0	0
0	0	Thoracotoı	54	PRIVE	No	74	0	0
0	0	VATS or ro	54	PRIVE	No	74	1	0
0	0	VATS or ro	54	PRIVE	No	56	0	0
0	0	VATS or ro	54	PRIVE	No	53	0	0
0	0	Thoracotoı	54	PRIVE	No	64	0	0
0	0	Thoracotoı	54	PRIVE	No	52	0	0
0	0	Thoracotoı	54	PRIVE	No	53	0	0
0	0	VATS or ro	54	PRIVE	No	66	0	0
0	0	Thoracotoı	54	PRIVE	No	29	0	0
0	0	Thoracotoı	54	PRIVE	No	63	0	0
0	0	Thoracotoı	54	PRIVE	No	47	0	0
0	0	VATS or ro	54	PRIVE	No	71	0	0
0	1	Thoracotoı	54	PRIVE	No	45	0	0
0	0	Thoracotoı	54	PRIVE	No	65	0	0
0	0	Thoracotoı	54	PRIVE	No	79	0	0
0	0	Thoracotoı	54	PRIVE	No	50	0	1
0	0	VATS or ro	54	PRIVE	No	75	0	0
0	0	VATS or ro	54	PRIVE	No	58	1	0
0	0	VATS or ro	54	PRIVE	No	62	1	0
0	0	VATS or ro	54	PRIVE	No	61	0	0
0	1	Thoracotoı	54	PRIVE	No	57	0	0
0	0	VATS or ro	54	PRIVE	No	76	0	0
0	0	VATS or ro	54	PRIVE	No	67	0	0
0	0	Thoracotoı	54	PRIVE	No	60	0	1
0	0	VATS or ro	54	PRIVE	No	68	0	0
0	0	Thoracotoı	54	PRIVE	No	61	0	0
0	0	VATS or ro	54	PRIVE	No	59	0	0
0	0	VATS or ro	54	PRIVE	No	58	0	0
0	0	VATS or ro	54	PRIVE	No	57	0	1
0	0	Thoracotoı	54	PRIVE	No	55	0	0
0	0	Thoracotoı	54	PRIVE	No	68	0	0
0	0	Thoracotoı	54	PRIVE	No	55	0	1
0	0	Thoracotoı	54	PRIVE	No	60	0	0
0	0	VATS or ro	54	PRIVE	No	64	0	0
0	0	Thoracotoı	54	PRIVE	No	67	0	0
0	0	Thoracotoı	54	PRIVE	No	53	1	0
0	0	VATS or ro	54	PRIVE	No	78	0	0
0	1	Thoracotoı	54	PRIVE	No	75	0	0
0	0	VATS or ro	54	PRIVE	No	72	1	0
0	0	Thoracotoı	54	PRIVE	No	62	1	0
0	0	Thoracotoı	54	PRIVE	No	66	0	0
1	0	Thoracotoı	101	CHU	No	72	1	1
1	0	VATS or ro	101	CHU	No	52	0	1
1	0	Thoracotoı	101	CHU	No	71	0	0
1	0	Thoracotoı	101	CHU	No	80	1	0
1	0	VATS or ro	101	CHU	No	55	0	0
1	0	Thoracotoı	101	CHU	No	54	0	1

1	0	Thoracotoı	101	CHU	No	31	0	0
1	0	VATS or rc	101	CHU	No	56	0	0
1	0	VATS or rc	101	CHU	No	47	0	0
1	0	Thoracotoı	101	CHU	No	77	0	0
1	0	Thoracotoı	101	CHU	No	49	0	1
1	0	VATS or rc	101	CHU	No	62	0	0
1	0	VATS or rc	101	CHU	No	64	0	0
1	0	Thoracotoı	101	CHU	No	64	0	1
1	0	VATS or rc	101	CHU	No	63	0	0
1	0	VATS or rc	101	CHU	No	70	0	0
1	0	VATS or rc	101	CHU	No	84	0	1
1	0	VATS or rc	101	CHU	No	58	0	0
1	0	VATS or rc	101	CHU	No	78	1	0
1	0	Thoracotoı	101	CHU	No	68	0	0
1	0	VATS or rc	101	CHU	No	78	0	0
1	0	VATS or rc	101	CHU	No	64	0	0
1	0	Thoracotoı	101	CHU	No	73	0	0
1	0	VATS or rc	101	CHU	No	59	0	0
1	0	Thoracotoı	101	CHU	No	33	0	0
1	0	VATS or rc	101	CHU	No	72	0	0
1	0	Thoracotoı	101	CHU	No	54	0	0
1	0	VATS or rc	101	CHU	No	53	0	0
1	0	Thoracotoı	101	CHU	No	67	0	0
1	0	VATS or rc	101	CHU	No	71	0	0
1	0	VATS or rc	101	CHU	No	71	0	0
1	0	Thoracotoı	101	CHU	No	72	0	1
1	0	Thoracotoı	101	CHU	No	59	0	0
1	0	Thoracotoı	101	CHU	No	69	0	1
1	0	VATS or rc	101	CHU	No	42	0	0
1	0	VATS or rc	101	CHU	No	61	0	0
1	0	VATS or rc	101	CHU	No	70	1	1
1	0	VATS or rc	101	CHU	No	72	0	1
1	0	VATS or rc	101	CHU	No	70	0	0
1	0	VATS or rc	101	CHU	No	24	0	0
1	0	Thoracotoı	101	CHU	No	63	0	0
1	0	Thoracotoı	101	CHU	No	78	0	1
1	0	Thoracotoı	101	CHU	No	67	0	0
1	0	VATS or rc	101	CHU	No	74	0	1
1	0	VATS or rc	101	CHU	No	55	0	0
1	0	VATS or rc	101	CHU	No	35	0	0
1	0	Thoracotoı	101	CHU	No	63	0	1
1	0	VATS or rc	101	CHU	No	68	0	1
1	0	Thoracotoı	101	CHU	No	60	0	0
1	1	Thoracotoı	101	CHU	No	60	0	0
1	0	VATS or rc	101	CHU	No	72	0	0
1	0	VATS or rc	101	CHU	No	56	0	1
1	0	Thoracotoı	101	CHU	No	58	0	1
1	0	VATS or rc	101	CHU	No	71	0	1
1	1	Thoracotoı	101	CHU	No	67	1	1
1	0	Thoracotoı	101	CHU	No	73	0	0
1	0	Thoracotoı	101	CHU	No	28	0	0
1	0	Thoracotoı	101	CHU	No	70	0	1
1	0	Thoracotoı	101	CHU	No	68	0	0
1	1	Thoracotoı	101	CHU	No	50	0	1
1	0	Thoracotoı	101	CHU	No	66	0	0
1	0	VATS or rc	101	CHU	No	61	0	1

1	0	VATS or rc	101	CHU	No	72	0	0
1	1	Thoracotoı	101	CHU	No	53	0	0
1	0	Thoracotoı	101	CHU	No	50	0	0
1	0	VATS or rc	101	CHU	No	77	0	0
1	0	Thoracotoı	101	CHU	No	75	0	1
1	0	VATS or rc	101	CHU	No	73	1	0
1	0	VATS or rc	101	CHU	No	63	0	0
1	0	Thoracotoı	101	CHU	No	54	1	0
1	0	Thoracotoı	101	CHU	No	62	0	0
1	0	VATS or rc	101	CHU	No	63	0	0
1	0	VATS or rc	101	CHU	No	69	0	0
1	0	Thoracotoı	101	CHU	No	67	1	1
1	0	Thoracotoı	101	CHU	No	73	0	0
1	0	VATS or rc	101	CHU	No	67	0	0
1	0	Thoracotoı	101	CHU	No	63	0	1
1	1	Thoracotoı	101	CHU	No	61	0	0
1	0	VATS or rc	101	CHU	No	38	0	1
1	0	VATS or rc	101	CHU	No	74	0	0
1	0	VATS or rc	101	CHU	No	62	0	0
1	0	Thoracotoı	101	CHU	No	65	0	1
1	0	Thoracotoı	101	CHU	No	61	0	1
1	0	Thoracotoı	101	CHU	No	68	0	0
1	0	VATS or rc	101	CHU	No	67	0	0
1	0	VATS or rc	101	CHU	No	55	0	0
1	0	Thoracotoı	101	CHU	No	69	0	0
1	0	VATS or rc	101	CHU	No	67	1	0
1	1	Thoracotoı	101	CHU	No	44	0	1
1	0	Thoracotoı	101	CHU	No	73	0	0
1	0	VATS or rc	101	CHU	No	63	0	1
1	0	Thoracotoı	101	CHU	No	61	0	1
1	1	Thoracotoı	101	CHU	No	60	0	0
1	0	VATS or rc	101	CHU	No	70	0	1
1	0	Thoracotoı	101	CHU	No	78	0	0
1	0	VATS or rc	101	CHU	No	57	0	1
1	0	Thoracotoı	101	CHU	No	51	0	0
1	0	VATS or rc	101	CHU	No	57	0	1
1	0	Thoracotoı	101	CHU	No	68	0	1
1	0	VATS or rc	101	CHU	No	83	0	0
1	0	VATS or rc	101	CHU	No	63	0	0
1	0	VATS or rc	101	CHU	No	83	0	0
1	0	Thoracotoı	101	CHU	No	64	0	1
1	0	VATS or rc	101	CHU	No	63	0	0
1	0	Thoracotoı	101	CHU	No	54	0	0
1	0	Thoracotoı	101	CHU	Yes	77	0	0
1	0	Thoracotoı	101	CHU	No	67	0	1
1	0	VATS or rc	101	CHU	No	64	1	0
1	1	Thoracotoı	101	CHU	No	61	1	0
1	0	Thoracotoı	101	CHU	No	45	0	1
1	0	Thoracotoı	101	CHU	No	65	0	0
1	0	VATS or rc	101	CHU	No	66	1	1
1	0	Thoracotoı	101	CHU	No	67	1	0
1	1	Thoracotoı	101	CHU	No	68	0	0
1	0	VATS or rc	101	CHU	No	56	0	1
1	0	Thoracotoı	101	CHU	No	77	1	0
1	0	Thoracotoı	101	CHU	No	67	0	0
1	0	VATS or rc	101	CHU	No	64	0	1

1	0	Thoracotoı	101	CHU	No	77	0	0
1	0	Thoracotoı	101	CHU	No	52	0	1
1	0	VATS or rc	101	CHU	No	63	0	0
1	0	VATS or rc	101	CHU	No	59	0	1
1	0	Thoracotoı	101	CHU	No	74	0	1
1	0	Thoracotoı	101	CHU	No	59	0	1
1	0	VATS or rc	101	CHU	No	72	0	0
1	0	Thoracotoı	101	CHU	No	56	0	1
1	0	VATS or rc	101	CHU	No	70	0	0
1	0	VATS or rc	101	CHU	No	69	0	0
1	0	VATS or rc	101	CHU	No	71	0	0
1	0	Thoracotoı	101	CHU	No	56	0	1
1	0	VATS or rc	101	CHU	No	42	0	1
1	0	VATS or rc	101	CHU	No	80	0	0
1	1	Thoracotoı	101	CHU	No	46	0	0
1	0	VATS or rc	101	CHU	No	72	0	0
1	0	VATS or rc	101	CHU	No	74	0	0
1	0	Thoracotoı	101	CHU	No	69	0	0
1	0	VATS or rc	101	CHU	No	60	0	0
1	0	Thoracotoı	101	CHU	No	62	1	0
1	0	VATS or rc	101	CHU	No	63	0	1
1	0	Thoracotoı	101	CHU	No	82	0	1
1	0	Thoracotoı	101	CHU	No	51	0	0
1	0	Thoracotoı	101	CHU	Yes	74	0	0
1	0	VATS or rc	101	CHU	No	63	0	1
1	0	Thoracotoı	101	CHU	No	69	0	0
1	0	VATS or rc	101	CHU	No	62	1	0
1	1	Thoracotoı	101	CHU	No	55	0	1
1	0	Thoracotoı	101	CHU	No	55	0	1
1	0	Thoracotoı	101	CHU	No	61	0	1
1	1	Thoracotoı	101	CHU	No	59	0	0
1	0	VATS or rc	101	CHU	No	61	0	0
1	0	VATS or rc	101	CHU	No	52	0	1
1	0	VATS or rc	101	CHU	No	67	0	0
1	0	VATS or rc	101	CHU	No	66	0	1
1	0	Thoracotoı	101	CHU	No	62	0	1
1	0	Thoracotoı	101	CHU	No	59	1	1
1	0	VATS or rc	101	CHU	No	66	0	1
1	0	VATS or rc	101	CHU	No	73	0	0
1	0	Thoracotoı	101	CHU	No	68	0	0
1	0	VATS or rc	101	CHU	No	54	1	0
1	0	VATS or rc	101	CHU	No	72	0	0
1	0	VATS or rc	101	CHU	No	68	0	1
1	0	Thoracotoı	101	CHU	No	62	0	1
1	0	Thoracotoı	101	CHU	No	73	0	0
1	0	VATS or rc	101	CHU	No	60	0	1
1	0	VATS or rc	101	CHU	No	48	0	0
1	0	VATS or rc	101	CHU	No	61	0	0
1	0	VATS or rc	101	CHU	No	64	0	1
1	1	Thoracotoı	101	CHU	No	62	0	1
1	0	VATS or rc	101	CHU	No	70	0	1
1	1	Thoracotoı	101	CHU	No	54	0	0
1	0	VATS or rc	101	CHU	No	47	1	0
1	0	Thoracotoı	101	CHU	No	53	0	1
1	1	Thoracotoı	101	CHU	No	54	1	1
1	0	VATS or rc	101	CHU	No	71	0	0

1	0	VATS or rc	101	CHU	No	66	0	0
1	0	Thoracotoı	101	CHU	No	60	0	1
1	0	VATS or rc	101	CHU	No	62	1	0
1	0	Thoracotoı	101	CHU	No	68	0	0
1	0	VATS or rc	101	CHU	No	52	0	0
1	1	Thoracotoı	101	CHU	No	58	0	1
1	0	Thoracotoı	101	CHU	No	64	0	0
1	0	Thoracotoı	101	CHU	No	68	0	1
1	0	Thoracotoı	101	CHU	No	53	0	1
1	0	VATS or rc	101	CHU	No	63	0	1
1	0	VATS or rc	101	CHU	No	64	0	1
1	0	Thoracotoı	101	CHU	No	56	0	0
1	0	VATS or rc	101	CHU	No	65	0	0
1	1	Thoracotoı	101	CHU	No	76	0	0
1	0	Thoracotoı	101	CHU	No	71	0	0
1	0	VATS or rc	101	CHU	No	75	0	0
1	0	VATS or rc	101	CHU	No	74	0	0
1	0	VATS or rc	101	CHU	No	71	0	0
1	0	VATS or rc	101	CHU	No	66	0	1
1	0	Thoracotoı	101	CHU	No	62	0	0
1	0	VATS or rc	101	CHU	No	77	0	0
1	0	Thoracotoı	101	CHU	No	72	0	1
1	0	Thoracotoı	101	CHU	No	70	0	0
1	0	VATS or rc	101	CHU	No	68	0	0
1	0	VATS or rc	101	CHU	No	56	0	0
1	0	VATS or rc	101	CHU	No	74	0	1
1	0	VATS or rc	101	CHU	No	67	0	0
1	0	VATS or rc	101	CHU	No	69	0	0
1	0	Thoracotoı	101	CHU	No	50	0	0
1	0	VATS or rc	101	CHU	No	67	0	0
1	0	Thoracotoı	101	CHU	No	78	0	1
1	0	VATS or rc	101	CHU	No	61	0	0
1	0	VATS or rc	101	CHU	No	74	0	0
1	0	Thoracotoı	101	CHU	No	56	0	0
1	0	VATS or rc	101	CHU	No	50	0	0
1	0	VATS or rc	101	CHU	No	62	0	0
1	1	Thoracotoı	101	CHU	No	68	0	1
1	0	VATS or rc	101	CHU	No	62	0	1
1	1	Thoracotoı	101	CHU	No	70	0	0
1	0	VATS or rc	101	CHU	No	76	0	0
1	0	Thoracotoı	101	CHU	No	70	0	1
1	0	VATS or rc	101	CHU	No	62	0	1
1	0	VATS or rc	101	CHU	No	65	0	1
1	0	VATS or rc	101	CHU	No	67	0	0
1	0	Thoracotoı	101	CHU	No	48	0	1
1	0	VATS or rc	101	CHU	No	73	0	0
1	0	VATS or rc	101	CHU	No	69	0	0
1	0	VATS or rc	101	CHU	No	48	0	0
1	1	Thoracotoı	101	CHU	No	51	0	0
1	0	Thoracotoı	101	CHU	No	59	0	1
1	0	VATS or rc	101	CHU	No	62	0	1
1	0	VATS or rc	101	CHU	No	63	0	0
1	0	VATS or rc	101	CHU	No	62	0	0
1	0	VATS or rc	101	CHU	No	64	0	1
1	1	Thoracotoı	101	CHU	No	55	0	0
1	0	VATS or rc	101	CHU	No	69	1	0

1	1	Thoracotoı	101	CHU	No	69	0	0
1	0	VATS or rc	101	CHU	No	70	0	1
1	0	VATS or rc	101	CHU	No	54	0	0
1	0	VATS or rc	101	CHU	No	55	0	0
1	0	VATS or rc	101	CHU	No	63	0	0
1	0	VATS or rc	101	CHU	No	69	1	0
1	0	VATS or rc	101	CHU	No	59	0	0
1	0	VATS or rc	101	CHU	No	64	0	1
1	1	Thoracotoı	101	CHU	No	65	0	0
1	0	VATS or rc	101	CHU	No	67	0	0
1	0	Thoracotoı	101	CHU	No	68	0	1
1	0	Thoracotoı	101	CHU	No	77	0	0
1	0	VATS or rc	101	CHU	No	67	0	1
1	0	VATS or rc	101	CHU	No	75	0	0
1	0	VATS or rc	101	CHU	No	55	0	0
1	0	VATS or rc	101	CHU	No	81	0	1
1	0	VATS or rc	101	CHU	No	63	0	1
1	0	VATS or rc	101	CHU	No	52	0	1
1	0	VATS or rc	101	CHU	No	72	0	0
1	0	VATS or rc	101	CHU	No	62	0	0
1	0	VATS or rc	101	CHU	No	60	0	0
1	0	VATS or rc	101	CHU	No	60	0	0
1	0	VATS or rc	101	CHU	No	75	0	0
1	0	Thoracotoı	101	CHU	No	58	0	1
1	0	VATS or rc	101	CHU	No	57	1	0
1	0	Thoracotoı	101	CHU	No	72	0	0
1	0	Thoracotoı	101	CHU	No	60	0	0
1	0	Thoracotoı	101	CHU	No	70	0	0
1	0	VATS or rc	101	CHU	No	68	0	1
1	0	VATS or rc	101	CHU	No	61	0	1
1	0	VATS or rc	101	CHU	No	54	0	1
1	0	VATS or rc	101	CHU	No	73	0	1
1	0	VATS or rc	101	CHU	No	73	0	0
1	0	VATS or rc	101	CHU	No	74	0	1
0	1	Thoracotoı	199	CHU	No	71	1	0
0	0	VATS or rc	199	CHU	No	61	0	0
0	0	Thoracotoı	199	CHU	No	62	1	0
0	0	VATS or rc	199	CHU	No	50	0	0
0	0	Thoracotoı	199	CHU	No	59	1	0
0	0	Thoracotoı	199	CHU	No	67	0	0
0	0	Thoracotoı	199	CHU	No	74	0	0
0	0	VATS or rc	199	CHU	No	63	0	0
0	0	VATS or rc	199	CHU	No	59	0	0
0	0	VATS or rc	199	CHU	No	81	0	0
0	0	Thoracotoı	199	CHU	No	70	0	0
0	0	Thoracotoı	199	CHU	No	71	0	0
0	0	Thoracotoı	199	CHU	No	57	0	0
0	0	Thoracotoı	199	CHU	No	67	0	0
0	0	Thoracotoı	199	CHU	No	67	0	0
0	0	Thoracotoı	199	CHU	No	72	0	0
0	0	Thoracotoı	199	CHU	No	75	1	0
0	0	VATS or rc	199	CHU	No	62	0	0
0	0	Thoracotoı	199	CHU	No	57	0	0
0	1	Thoracotoı	199	CHU	No	58	1	0
0	1	Thoracotoı	199	CHU	No	76	0	0
0	0	VATS or rc	199	CHU	No	66	0	0

0	0	Thoracotoı	199	CHU	No	57	1	0
0	0	VATS or rc	199	CHU	No	59	0	0
0	0	VATS or rc	199	CHU	No	85	1	0
0	0	VATS or rc	199	CHU	No	66	1	0
0	0	Thoracotoı	199	CHU	No	74	1	0
0	0	Thoracotoı	199	CHU	No	63	0	0
0	1	Thoracotoı	199	CHU	No	73	0	0
0	0	Thoracotoı	199	CHU	No	60	1	0
0	0	VATS or rc	199	CHU	No	68	1	0
0	0	Thoracotoı	199	CHU	No	57	0	0
0	0	Thoracotoı	199	CHU	No	77	1	0
0	0	VATS or rc	199	CHU	No	77	1	0
0	1	Thoracotoı	199	CHU	No	52	0	0
0	0	Thoracotoı	199	CHU	No	59	0	0
0	0	VATS or rc	199	CHU	No	77	0	0
0	0	Thoracotoı	199	CHU	No	64	0	0
0	0	Thoracotoı	199	CHU	No	84	1	0
0	0	Thoracotoı	199	CHU	No	74	0	0
0	1	Thoracotoı	199	CHU	No	62	0	0
0	0	Thoracotoı	199	CHU	No	68	1	0
0	0	Thoracotoı	199	CHU	No	63	0	0
0	0	VATS or rc	199	CHU	No	69	0	0
0	0	Thoracotoı	199	CHU	No	68	0	0
0	0	VATS or rc	199	CHU	No	68	0	0
0	0	Thoracotoı	199	CHU	No	58	0	0
0	0	VATS or rc	199	CHU	No	67	0	0
0	0	VATS or rc	199	CHU	No	65	0	0
0	0	VATS or rc	199	CHU	No	53	0	0
0	0	VATS or rc	199	CHU	No	73	1	0
0	0	VATS or rc	199	CHU	No	56	0	0
0	0	Thoracotoı	199	CHU	No	61	0	0
0	0	Thoracotoı	199	CHU	No	80	0	0
0	0	VATS or rc	199	CHU	No	64	0	0
0	0	VATS or rc	199	CHU	No	73	0	0
0	0	Thoracotoı	199	CHU	No	66	0	0
0	0	Thoracotoı	199	CHU	No	61	0	0
0	0	VATS or rc	199	CHU	No	73	0	0
0	0	VATS or rc	199	CHU	No	63	0	0
0	0	VATS or rc	199	CHU	No	69	0	0
0	0	Thoracotoı	199	CHU	No	61	0	0
0	0	Thoracotoı	199	CHU	No	54	0	0
0	0	Thoracotoı	199	CHU	No	62	0	0
0	0	VATS or rc	199	CHU	No	72	0	0
0	0	VATS or rc	199	CHU	No	58	0	0
0	0	VATS or rc	199	CHU	No	84	0	0
0	0	Thoracotoı	199	CHU	No	74	0	0
0	0	Thoracotoı	199	CHU	No	65	0	0
0	0	Thoracotoı	199	CHU	No	61	0	0
0	0	Thoracotoı	199	CHU	No	57	0	0
0	0	Thoracotoı	199	CHU	No	46	0	0
0	0	Thoracotoı	199	CHU	No	61	0	0
0	0	Thoracotoı	199	CHU	No	68	0	0
0	0	VATS or rc	199	CHU	No	71	0	0
0	0	VATS or rc	199	CHU	No	57	0	0
0	0	Thoracotoı	199	CHU	No	85	0	1
0	0	VATS or rc	199	CHU	No	70	0	0

0	0	VATS or ro	199	CHU	No	66	1	0
0	0	Thoracotor	199	CHU	No	85	1	0
0	1	Thoracotor	199	CHU	No	64	0	0
0	0	VATS or ro	199	CHU	No	62	0	0
0	1	Thoracotor	199	CHU	Yes	68	0	0
0	0	VATS or ro	199	CHU	No	74	0	0
0	0	Thoracotor	199	CHU	No	60	0	0
0	0	Thoracotor	199	CHU	No	59	0	0
0	0	VATS or ro	199	CHU	No	60	0	0
0	0	Thoracotor	199	CHU	No	75	0	0
0	0	VATS or ro	199	CHU	No	75	0	0
0	0	Thoracotor	199	CHU	No	74	0	0
0	0	Thoracotor	199	CHU	No	51	0	0
0	0	VATS or ro	199	CHU	No	56	0	0
0	0	VATS or ro	199	CHU	No	66	0	0
0	0	VATS or ro	199	CHU	No	53	0	0
0	0	VATS or ro	199	CHU	No	74	0	0
0	0	VATS or ro	199	CHU	No	48	0	0
0	1	Thoracotor	199	CHU	No	64	0	0
0	0	Thoracotor	199	CHU	No	64	0	0
0	0	VATS or ro	199	CHU	No	42	0	0
0	0	VATS or ro	199	CHU	No	56	1	0
0	0	VATS or ro	199	CHU	No	62	0	0
0	0	Thoracotor	199	CHU	No	75	0	0
0	0	VATS or ro	199	CHU	No	70	0	0
0	0	Thoracotor	199	CHU	No	58	0	0
0	0	VATS or ro	199	CHU	No	65	0	0
0	0	Thoracotor	199	CHU	No	74	0	0
0	0	Thoracotor	199	CHU	No	42	0	0
0	0	Thoracotor	199	CHU	No	69	0	0
0	0	Thoracotor	199	CHU	No	68	0	0
0	0	VATS or ro	199	CHU	No	48	0	0
0	0	Thoracotor	199	CHU	No	66	1	0
0	0	VATS or ro	199	CHU	No	59	0	0
0	0	Thoracotor	199	CHU	No	74	1	0
0	0	Thoracotor	199	CHU	No	72	0	0
0	0	Thoracotor	199	CHU	No	73	0	0
0	1	Thoracotor	199	CHU	No	50	0	0
0	0	Thoracotor	199	CHU	No	71	0	0
0	0	Thoracotor	199	CHU	No	56	0	0
0	0	VATS or ro	199	CHU	No	81	0	0
0	1	Thoracotor	199	CHU	No	67	1	0
0	0	VATS or ro	199	CHU	No	71	0	0
0	0	VATS or ro	199	CHU	No	72	0	0
0	0	Thoracotor	199	CHU	No	50	0	0
0	1	Thoracotor	199	CHU	No	68	0	0
0	1	Thoracotor	199	CHU	No	60	0	0
0	0	VATS or ro	199	CHU	No	61	0	0
0	0	Thoracotor	199	CHU	No	61	0	0
0	0	VATS or ro	199	CHU	No	73	0	0
0	0	VATS or ro	199	CHU	No	59	0	0
0	0	VATS or ro	199	CHU	No	77	0	0
0	0	VATS or ro	199	CHU	No	69	0	0
0	0	Thoracotor	199	CHU	No	72	0	0
0	0	VATS or ro	199	CHU	No	71	0	0
0	0	VATS or ro	199	CHU	No	52	0	0

0	0	VATS or ro	199	CHU	No	65	0	0
0	0	VATS or ro	199	CHU	No	78	0	0
0	0	VATS or ro	199	CHU	No	65	0	0
0	0	Thoracotor	199	CHU	No	72	1	0
0	0	VATS or ro	199	CHU	No	70	0	0
0	0	Thoracotor	199	CHU	No	63	0	0
0	0	VATS or ro	199	CHU	No	77	0	0
0	0	Thoracotor	199	CHU	No	54	0	0
0	0	VATS or ro	199	CHU	No	58	0	0
0	0	Thoracotor	199	CHU	No	72	0	0
0	0	VATS or ro	199	CHU	No	49	0	0
0	0	VATS or ro	199	CHU	No	67	0	0
0	1	Thoracotor	199	CHU	No	57	0	0
0	0	VATS or ro	199	CHU	No	69	0	0
0	0	Thoracotor	199	CHU	No	63	0	0
0	0	Thoracotor	199	CHU	Yes	75	0	0
0	0	Thoracotor	199	CHU	No	73	0	0
0	0	VATS or ro	199	CHU	No	71	0	0
0	0	Thoracotor	199	CHU	No	60	0	0
0	0	Thoracotor	199	CHU	No	78	1	0
0	0	VATS or ro	199	CHU	No	63	0	0
0	0	Thoracotor	199	CHU	No	72	0	0
0	0	VATS or ro	199	CHU	No	61	0	0
0	0	Thoracotor	199	CHU	No	60	0	0
0	0	Thoracotor	199	CHU	No	68	0	0
0	0	Thoracotor	199	CHU	No	64	0	0
0	0	VATS or ro	199	CHU	No	75	0	0
0	0	Thoracotor	199	CHU	No	75	0	0
0	0	VATS or ro	199	CHU	No	62	1	0
0	0	Thoracotor	199	CHU	No	53	0	0
0	0	VATS or ro	199	CHU	No	63	0	0
0	0	VATS or ro	199	CHU	No	50	0	0
0	0	VATS or ro	199	CHU	No	61	0	0
0	0	VATS or ro	199	CHU	No	74	0	0
0	0	VATS or ro	199	CHU	No	68	0	0
0	0	VATS or ro	199	CHU	No	63	0	0
0	0	VATS or ro	199	CHU	No	74	0	0
0	0	VATS or ro	199	CHU	No	51	0	0
0	0	Thoracotor	199	CHU	No	46	1	0
0	0	VATS or ro	199	CHU	No	73	0	0
0	0	Thoracotor	199	CHU	No	61	0	0
0	0	VATS or ro	199	CHU	No	71	0	0
0	0	VATS or ro	199	CHU	No	59	0	0
0	0	Thoracotor	199	CHU	No	61	0	0
0	0	VATS or ro	199	CHU	No	79	0	0
0	0	Thoracotor	199	CHU	No	62	0	0
0	0	Thoracotor	199	CHU	No	70	0	0
0	0	Thoracotor	199	CHU	No	64	0	0
0	0	Thoracotor	199	CHU	No	77	0	0
0	0	VATS or ro	199	CHU	No	75	0	0
0	1	Thoracotor	199	CHU	No	49	0	0
0	0	VATS or ro	199	CHU	No	75	0	0
0	0	Thoracotor	199	CHU	No	58	0	0
0	0	Thoracotor	199	CHU	No	77	0	0
0	0	Thoracotor	199	CHU	No	60	1	0
0	0	Thoracotor	199	CHU	No	55	0	0

0	0	Thoracotoı	199	CHU	No	75	0	0
0	0	VATS or rc	199	CHU	No	79	0	0
0	0	VATS or rc	199	CHU	No	71	0	0
0	0	VATS or rc	199	CHU	No	63	0	0
0	0	Thoracotoı	199	CHU	No	63	0	0
0	0	VATS or rc	199	CHU	No	70	0	0
0	0	Thoracotoı	199	CHU	No	68	0	0
0	0	Thoracotoı	199	CHU	No	55	0	0
0	0	Thoracotoı	199	CHU	No	61	0	0
0	0	Thoracotoı	199	CHU	No	77	0	0
0	1	Thoracotoı	199	CHU	No	61	0	0
0	0	Thoracotoı	199	CHU	No	75	0	0
0	0	Thoracotoı	199	CHU	No	72	0	0
0	0	Thoracotoı	199	CHU	No	73	0	0
0	0	Thoracotoı	199	CHU	No	79	0	0
0	0	Thoracotoı	199	CHU	No	72	0	0
0	1	Thoracotoı	199	CHU	No	60	1	0
0	0	VATS or rc	199	CHU	No	60	0	0
0	0	Thoracotoı	199	CHU	No	49	0	0
0	0	Thoracotoı	199	CHU	No	63	0	0
0	0	VATS or rc	199	CHU	No	68	0	0
0	0	Thoracotoı	199	CHU	No	70	0	0
0	0	Thoracotoı	199	CHU	No	57	0	0
0	0	VATS or rc	199	CHU	No	67	0	0
0	1	Thoracotoı	199	CHU	Yes	84	0	0
0	0	Thoracotoı	199	CHU	No	65	0	0
0	0	Thoracotoı	199	CHU	No	68	1	0
0	0	Thoracotoı	199	CHU	No	57	0	0
0	0	Thoracotoı	199	CHU	No	68	0	0
0	0	Thoracotoı	199	CHU	No	56	0	0
0	0	Thoracotoı	199	CHU	No	68	0	0
0	0	VATS or rc	199	CHU	No	62	0	0
0	0	Thoracotoı	199	CHU	No	59	0	0
0	0	Thoracotoı	199	CHU	No	59	0	0
0	0	VATS or rc	199	CHU	No	83	0	0
0	0	VATS or rc	199	CHU	No	34	0	0
0	0	VATS or rc	199	CHU	No	74	0	0
0	0	Thoracotoı	199	CHU	No	80	0	0
0	0	Thoracotoı	199	CHU	No	60	0	0
0	0	Thoracotoı	199	CHU	No	59	0	0
0	0	Thoracotoı	199	CHU	No	54	0	0
0	0	VATS or rc	199	CHU	No	76	1	0
0	1	Thoracotoı	199	CHU	No	83	0	0
0	1	Thoracotoı	199	CHU	No	78	0	0
0	0	Thoracotoı	199	CHU	No	60	0	0
0	0	Thoracotoı	199	CHU	No	74	0	0
0	0	Thoracotoı	199	CHU	No	56	0	0
0	0	VATS or rc	199	CHU	No	65	0	0
0	0	VATS or rc	199	CHU	No	62	0	0
0	0	Thoracotoı	199	CHU	No	63	0	0
0	0	Thoracotoı	199	CHU	No	54	0	0
0	0	Thoracotoı	199	CHU	No	59	0	0
0	0	VATS or rc	199	CHU	No	71	0	0
0	0	VATS or rc	199	CHU	No	47	0	0
0	0	VATS or rc	199	CHU	No	79	0	0
0	0	Thoracotoı	199	CHU	No	69	0	0

0	1	Thoracotoı	199	CHU	No	77	0	0
0	0	VATS or rc	199	CHU	No	64	0	0
0	1	Thoracotoı	199	CHU	No	49	0	0
0	0	VATS or rc	199	CHU	No	68	0	0
0	0	Thoracotoı	199	CHU	No	66	0	0
0	0	Thoracotoı	199	CHU	No	74	0	0
0	0	VATS or rc	199	CHU	No	62	0	0
0	0	Thoracotoı	199	CHU	No	83	1	0
0	0	Thoracotoı	199	CHU	No	71	1	0
0	0	Thoracotoı	199	CHU	No	55	0	0
0	0	VATS or rc	199	CHU	No	70	0	0
0	1	Thoracotoı	199	CHU	No	59	0	0
0	0	Thoracotoı	199	CHU	No	81	0	0
0	0	VATS or rc	199	CHU	No	79	1	0
0	1	Thoracotoı	199	CHU	No	62	0	0
0	0	VATS or rc	199	CHU	No	60	0	0
0	0	VATS or rc	199	CHU	No	46	0	0
0	0	Thoracotoı	199	CHU	No	77	0	0
0	0	Thoracotoı	199	CHU	No	73	0	0
0	0	Thoracotoı	199	CHU	No	76	0	0
0	0	VATS or rc	199	CHU	No	68	0	0
0	0	Thoracotoı	199	CHU	No	38	0	0
0	0	Thoracotoı	199	CHU	No	75	0	0
0	0	VATS or rc	199	CHU	No	63	0	0
0	0	VATS or rc	199	CHU	No	74	0	0
0	0	Thoracotoı	199	CHU	No	59	0	0
0	0	Thoracotoı	199	CHU	No	54	0	0
0	1	Thoracotoı	199	CHU	No	65	1	0
0	1	Thoracotoı	199	CHU	No	44	1	0
0	0	Thoracotoı	199	CHU	No	63	0	0
0	0	VATS or rc	199	CHU	No	73	0	0
0	0	Thoracotoı	199	CHU	No	54	0	0
0	0	Thoracotoı	199	CHU	No	75	0	0
0	0	Thoracotoı	199	CHU	No	58	0	0
0	0	VATS or rc	199	CHU	No	70	0	0
0	0	Thoracotoı	199	CHU	No	73	1	0
0	0	Thoracotoı	199	CHU	No	57	0	0
0	1	Thoracotoı	199	CHU	No	59	0	0
0	0	VATS or rc	199	CHU	No	61	0	0
0	0	VATS or rc	199	CHU	No	67	1	0
0	1	Thoracotoı	199	CHU	No	56	0	0
0	0	Thoracotoı	199	CHU	No	61	0	0
0	1	Thoracotoı	199	CHU	No	62	0	0
0	0	Thoracotoı	199	CHU	No	35	0	0
0	0	Thoracotoı	199	CHU	No	73	0	0
0	0	VATS or rc	199	CHU	No	62	0	0
0	1	Thoracotoı	199	CHU	No	63	1	0
0	0	VATS or rc	199	CHU	No	67	0	0
0	0	Thoracotoı	199	CHU	No	52	0	0
0	0	Thoracotoı	199	CHU	No	70	0	0
0	0	Thoracotoı	199	CHU	No	62	0	0
0	0	Thoracotoı	199	CHU	No	78	0	0
0	0	Thoracotoı	199	CHU	No	64	0	0
0	1	Thoracotoı	199	CHU	No	61	0	0
0	0	Thoracotoı	199	CHU	No	73	0	0
0	0	Thoracotoı	199	CHU	No	79	0	0

0	0	Thoracotoı	199	CHU	No	76	0	0
0	0	VATS or rc	199	CHU	No	60	0	0
0	0	Thoracotoı	199	CHU	No	60	1	0
0	0	VATS or rc	199	CHU	No	72	0	0
0	0	Thoracotoı	199	CHU	No	65	0	0
0	0	Thoracotoı	199	CHU	No	66	0	0
0	0	Thoracotoı	199	CHU	No	72	0	0
0	0	Thoracotoı	199	CHU	No	65	0	0
0	0	VATS or rc	199	CHU	No	71	0	0
0	0	Thoracotoı	199	CHU	No	73	0	0
0	0	Thoracotoı	199	CHU	No	71	0	0
0	0	Thoracotoı	199	CHU	No	55	0	0
0	0	Thoracotoı	199	CHU	No	65	0	0
0	0	VATS or rc	199	CHU	No	63	1	0
0	0	Thoracotoı	199	CHU	No	54	0	0
0	1	Thoracotoı	199	CHU	No	61	0	0
0	0	VATS or rc	199	CHU	No	72	1	0
0	0	Thoracotoı	199	CHU	No	68	0	0
0	0	VATS or rc	199	CHU	No	71	0	0
0	0	Thoracotoı	199	CHU	No	66	0	0
0	0	Thoracotoı	199	CHU	No	51	0	0
0	0	Thoracotoı	199	CHU	No	61	1	0
0	0	VATS or rc	199	CHU	No	68	0	0
0	0	Thoracotoı	199	CHU	No	57	0	0
0	0	VATS or rc	199	CHU	No	63	1	0
0	0	VATS or rc	199	CHU	No	68	0	0
0	0	Thoracotoı	199	CHU	No	61	0	0
0	0	Thoracotoı	199	CHU	No	60	0	0
0	0	VATS or rc	199	CHU	No	68	0	0
0	0	Thoracotoı	199	CHU	No	55	0	0
0	0	Thoracotoı	199	CHU	No	70	0	0
0	0	Thoracotoı	199	CHU	No	44	0	0
0	0	VATS or rc	199	CHU	No	65	0	0
0	0	VATS or rc	32	PRIVE	No	69	1	0
0	0	Thoracotoı	32	PRIVE	No	61	0	0
0	0	VATS or rc	32	PRIVE	No	79	1	0
0	0	Thoracotoı	32	PRIVE	No	55	0	0
0	1	Thoracotoı	32	PRIVE	No	75	0	0
0	0	Thoracotoı	32	PRIVE	No	61	0	0
0	0	Thoracotoı	32	PRIVE	No	78	0	0
0	0	Thoracotoı	32	PRIVE	No	84	0	1
0	0	VATS or rc	32	PRIVE	No	86	0	0
0	0	Thoracotoı	32	PRIVE	No	68	0	0
0	1	VATS or rc	32	PRIVE	No	51	0	1
0	0	VATS or rc	32	PRIVE	No	70	0	0
0	1	VATS or rc	32	PRIVE	No	85	0	0
0	0	Thoracotoı	32	PRIVE	No	65	1	0
0	0	Thoracotoı	32	PRIVE	No	74	0	1
0	0	VATS or rc	32	PRIVE	No	87	0	0
0	0	VATS or rc	32	PRIVE	No	77	0	1
0	0	VATS or rc	32	PRIVE	No	63	0	0
0	1	VATS or rc	32	PRIVE	No	85	0	0
0	0	Thoracotoı	32	PRIVE	No	78	0	0
0	0	Thoracotoı	32	PRIVE	No	74	0	0
0	0	Thoracotoı	32	PRIVE	No	74	0	1
0	0	VATS or rc	32	PRIVE	No	53	0	0

0	1	Thoracotoı	32	PRIVE	No	75	0	0
0	0	VATS or rc	32	PRIVE	No	53	0	0
0	0	VATS or rc	32	PRIVE	No	86	0	0
0	0	Thoracotoı	32	PRIVE	No	76	0	0
0	0	Thoracotoı	32	PRIVE	No	68	0	0
0	0	VATS or rc	32	CHU	No	59	0	1
0	0	VATS or rc	32	CHU	No	48	0	0
0	0	VATS or rc	32	CHU	No	65	0	1
0	0	VATS or rc	32	CHU	No	75	0	0
0	0	Thoracotoı	32	CHU	No	77	0	0
0	0	VATS or rc	32	CHU	No	64	0	0
0	0	VATS or rc	32	CHU	No	59	0	0
0	0	VATS or rc	32	CHU	No	64	0	0
0	0	VATS or rc	32	CHU	No	73	1	1
0	0	Thoracotoı	32	CHU	No	55	0	1
0	0	VATS or rc	32	CHU	No	68	0	0
0	0	Thoracotoı	28	CHU	No	71	0	0
0	0	Thoracotoı	28	CHU	No	62	0	1
0	0	Thoracotoı	28	CHU	No	44	0	0
0	0	Thoracotoı	28	CHU	No	64	0	1
0	0	Thoracotoı	28	CHU	No	75	0	0
0	0	Thoracotoı	28	CHU	No	64	0	1
0	0	VATS or rc	28	CHU	No	72	0	1
0	0	VATS or rc	28	CHU	No	68	0	0
0	0	Thoracotoı	28	CHU	No	68	0	1
0	0	Thoracotoı	28	CHU	No	57	0	1
0	0	Thoracotoı	28	CHU	No	74	0	0
0	0	Thoracotoı	28	CHU	No	65	0	1
0	1	Thoracotoı	28	CHU	No	75	0	1
0	0	Thoracotoı	28	CHU	No	79	0	0
0	0	Thoracotoı	28	CHU	No	67	0	1
0	0	Thoracotoı	28	CHU	No	61	0	1
0	0	Thoracotoı	28	CHU	No	74	0	1
0	0	VATS or rc	28	CHU	No	66	0	1
0	0	VATS or rc	28	CHU	No	66	0	0
0	0	Thoracotoı	28	CHU	No	65	1	1
0	0	Thoracotoı	28	CHU	No	55	0	0
0	0	Thoracotoı	28	CHU	No	81	1	1
0	0	Thoracotoı	28	CHU	No	61	0	0
0	0	Thoracotoı	28	CHU	No	59	0	1
0	0	Thoracotoı	28	CHU	No	66	0	1
0	0	Thoracotoı	28	CHU	No	55	0	0
0	0	Thoracotoı	28	CHU	No	52	0	1
0	0	Thoracotoı	28	CHU	No	60	0	1
0	0	Thoracotoı	28	CHU	No	68	0	1
0	0	VATS or rc	28	CHU	No	68	0	0
0	0	Thoracotoı	28	CHU	No	58	0	1
0	0	Thoracotoı	28	CHU	No	49	0	1
0	0	Thoracotoı	28	CHU	No	62	0	0
0	0	VATS or rc	28	CHU	No	63	1	1
0	0	Thoracotoı	28	CHU	No	68	0	1
0	0	Thoracotoı	28	CHU	No	73	0	1
0	0	Thoracotoı	28	CHU	No	76	0	0
0	0	Thoracotoı	28	CHU	No	48	0	1
0	0	Thoracotoı	28	CHU	No	74	0	0
0	0	Thoracotoı	28	CHU	No	69	0	1

0	0	Thoracotoı	28	CHU	No	66	0	0
0	0	Thoracotoı	28	CHU	No	61	0	0
0	0	Thoracotoı	28	CHU	No	48	0	1
0	0	Thoracotoı	28	CHU	No	58	1	1
0	0	VATS or rc	28	CHU	No	81	1	1
0	0	Thoracotoı	28	CHU	No	60	0	1
0	0	Thoracotoı	28	CHU	No	64	0	1
0	0	Thoracotoı	28	CHU	No	70	1	1
0	0	Thoracotoı	28	CHU	No	53	0	1
0	1	Thoracotoı	28	CHU	No	62	0	1
0	0	VATS or rc	28	CHU	No	68	1	1
0	0	VATS or rc	28	CHU	No	62	0	1
0	0	Thoracotoı	28	CHU	No	81	0	1
0	0	Thoracotoı	28	CHU	No	62	1	1
0	0	Thoracotoı	28	CHU	No	62	0	1
0	0	Thoracotoı	28	CHU	No	71	0	0
0	0	Thoracotoı	28	CHU	No	68	0	1
0	0	Thoracotoı	28	CHU	No	50	0	0
0	0	VATS or rc	28	CHU	No	75	0	0
0	0	Thoracotoı	28	CHU	No	59	0	1
0	0	Thoracotoı	28	CHU	No	68	0	1
0	0	Thoracotoı	28	CHU	No	66	0	1
0	0	Thoracotoı	28	CHU	No	54	0	1
0	0	Thoracotoı	28	CHU	No	59	0	1
0	0	Thoracotoı	28	CHU	No	85	0	1
0	0	Thoracotoı	28	CHU	No	73	0	0
0	0	VATS or rc	28	CHU	No	81	0	1
0	1	Thoracotoı	28	CHU	No	75	0	1
0	0	VATS or rc	28	CHU	No	65	0	1
0	0	Thoracotoı	28	CHU	No	73	0	0
0	0	Thoracotoı	28	CHU	No	62	1	1
0	0	Thoracotoı	28	CHU	No	54	0	1
0	0	VATS or rc	28	CHU	No	69	0	0
0	0	Thoracotoı	28	CHU	No	45	0	0
0	0	Thoracotoı	28	CHU	No	79	0	1
0	0	VATS or rc	28	CHU	No	62	0	1
0	1	Thoracotoı	28	CHU	No	64	0	1
0	0	Thoracotoı	28	CHU	No	48	0	1
0	0	VATS or rc	28	CHU	No	83	0	0
0	0	Thoracotoı	28	CHU	No	54	0	1
0	0	Thoracotoı	28	CHU	No	48	0	1
0	0	Thoracotoı	28	CHU	No	68	0	1
0	0	Thoracotoı	28	CHU	No	71	0	1
0	0	Thoracotoı	28	CHU	No	56	0	1
0	0	Thoracotoı	28	CHU	No	58	0	1
0	0	Thoracotoı	28	CHU	No	59	0	0
0	0	VATS or rc	28	CHU	No	55	1	0
0	0	Thoracotoı	28	CHU	No	54	0	1
0	0	Thoracotoı	28	CHU	No	77	0	1
0	0	Thoracotoı	28	CHU	No	67	0	0
0	0	Thoracotoı	28	CHU	No	52	0	1
0	0	Thoracotoı	28	CHU	No	55	0	0
0	0	Thoracotoı	28	CHU	No	69	0	1
0	0	Thoracotoı	28	CHU	No	68	0	1
0	0	VATS or rc	28	CHU	No	66	0	0
0	0	Thoracotoı	28	CHU	No	64	0	0

0	0	Thoracotoı	28	CHU	No	62	0	1
0	0	Thoracotoı	28	CHU	No	69	0	0
0	0	VATS or rc	1029	CHU	No	60	1	0
0	0	VATS or rc	1029	CHU	No	50	0	0
0	0	VATS or rc	1029	CHU	No	60	0	0
0	0	VATS or rc	1029	CHU	No	69	0	1
0	0	VATS or rc	1029	CHU	No	77	0	0
0	0	Thoracotoı	1029	CHU	No	72	0	0
0	0	VATS or rc	1029	CHU	No	61	0	0
0	0	VATS or rc	1029	CHU	No	63	0	0
0	0	VATS or rc	1029	CHU	No	73	0	0
0	0	VATS or rc	1029	CHU	No	67	0	1
0	0	VATS or rc	1029	CHU	No	50	0	1
0	0	VATS or rc	1029	CHU	No	67	0	0
0	0	VATS or rc	1029	CHU	No	56	0	0
0	0	VATS or rc	1029	CHU	No	66	1	0
0	0	VATS or rc	1029	CHU	No	43	0	0
0	0	VATS or rc	1029	CHU	No	68	0	0
0	0	VATS or rc	1029	CHU	No	59	0	0
0	0	VATS or rc	1029	CHU	No	60	1	1
0	0	VATS or rc	1029	CHU	No	51	0	0
0	0	VATS or rc	1029	CHU	No	66	0	0
0	0	Thoracotoı	1029	CHU	No	73	0	0
0	0	VATS or rc	1029	CHU	No	64	0	0
0	0	VATS or rc	1029	CHU	No	75	0	0
0	0	VATS or rc	1029	CHU	No	50	1	1
0	0	VATS or rc	1029	CHU	No	69	0	0
0	1	Thoracotoı	1029	CHU	No	67	0	1
0	0	Thoracotoı	1029	CHU	No	67	0	0
0	0	Thoracotoı	1029	CHU	No	71	1	0
0	1	Thoracotoı	1029	CHU	No	82	0	0
0	0	Thoracotoı	1029	CHU	No	56	0	0
0	0	VATS or rc	1029	CHU	No	63	0	0
0	0	Thoracotoı	1029	CHU	No	74	0	1
0	0	Thoracotoı	1029	CHU	No	60	0	1
0	0	Thoracotoı	1029	CHU	No	64	0	0
0	0	Thoracotoı	1029	CHU	No	68	0	0
0	0	Thoracotoı	1029	CHU	No	64	0	0
0	0	Thoracotoı	1029	CHU	No	69	0	0
0	1	Thoracotoı	1029	CHU	No	61	0	0
0	0	Thoracotoı	1029	CHU	No	67	0	0
0	0	Thoracotoı	1029	CHU	No	59	1	1
0	0	Thoracotoı	1029	CHU	No	81	0	0
0	0	VATS or rc	1029	CHU	No	74	0	1
0	0	Thoracotoı	1029	CHU	No	68	0	0
0	0	Thoracotoı	1029	CHU	No	65	0	1
0	0	Thoracotoı	1029	CHU	No	56	0	0
0	0	Thoracotoı	1029	CHU	No	49	0	0
0	0	Thoracotoı	1029	CHU	No	66	1	0
0	0	Thoracotoı	1029	CHU	No	69	0	0
0	0	Thoracotoı	1029	CHU	No	81	0	0
0	0	Thoracotoı	1029	CHU	No	66	0	0
0	0	Thoracotoı	1029	CHU	No	63	0	0
0	0	Thoracotoı	1029	CHU	No	76	0	0
0	0	Thoracotoı	1029	CHU	No	62	0	0
0	0	Thoracotoı	1029	CHU	No	69	0	0

0	0	Thoracotoı	1029	CHU	No	47	0	0
0	0	VATS or rc	1029	CHU	No	72	0	0
0	1	Thoracotoı	1029	CHU	No	69	0	0
0	0	Thoracotoı	1029	CHU	No	48	0	0
0	0	Thoracotoı	1029	CHU	No	61	0	0
0	0	Thoracotoı	1029	CHU	No	72	0	0
0	0	Thoracotoı	1029	CHU	No	63	0	0
0	0	Thoracotoı	1029	CHU	No	67	0	1
0	0	Thoracotoı	1029	CHU	No	73	0	0
0	0	Thoracotoı	1029	CHU	No	75	0	0
0	0	Thoracotoı	1029	CHU	No	51	0	0
0	0	Thoracotoı	1029	CHU	No	22	0	0
0	0	Thoracotoı	1029	CHU	No	62	0	0
0	0	Thoracotoı	1029	CHU	No	64	0	0
0	0	Thoracotoı	1029	CHU	No	78	0	0
0	0	Thoracotoı	1029	CHU	No	70	1	0
0	1	Thoracotoı	1029	CHU	No	65	0	0
0	0	Thoracotoı	1029	CHU	No	80	0	0
0	1	Thoracotoı	1029	CHU	No	66	0	0
0	0	Thoracotoı	1029	CHU	No	67	0	0
0	0	Thoracotoı	1029	CHU	No	69	0	0
0	0	Thoracotoı	1029	CHU	No	77	0	0
0	0	Thoracotoı	1029	CHU	No	68	0	0
0	0	Thoracotoı	1029	CHU	No	84	1	0
0	1	Thoracotoı	1029	CHU	No	62	0	0
0	0	Thoracotoı	1029	CHU	No	57	0	1
0	0	Thoracotoı	1029	CHU	No	73	0	0
0	0	Thoracotoı	1029	CHU	No	62	0	0
0	0	Thoracotoı	1029	CHU	No	68	0	0
0	1	Thoracotoı	1029	CHU	No	69	0	0
0	0	Thoracotoı	1029	CHU	No	66	0	0
0	0	Thoracotoı	1029	CHU	No	78	0	0
0	0	VATS or rc	1029	CHU	No	63	0	0
0	0	Thoracotoı	1029	CHU	No	76	0	1
0	0	Thoracotoı	1029	CHU	No	78	0	0
0	0	Thoracotoı	1029	CHU	No	73	0	0
0	0	Thoracotoı	1029	CHU	No	81	0	0
0	0	Thoracotoı	1029	CHU	No	68	1	0
0	0	Thoracotoı	1029	CHU	No	73	0	0
0	0	Thoracotoı	1029	CHU	No	66	0	0
0	0	VATS or rc	1029	CHU	No	62	0	0
0	0	Thoracotoı	1029	CHU	No	75	0	0
0	1	Thoracotoı	1029	CHU	No	26	0	0
0	0	Thoracotoı	1029	CHU	No	72	0	0
0	1	Thoracotoı	1029	CHU	No	52	0	1
0	0	Thoracotoı	1029	CHU	No	56	0	1
0	0	Thoracotoı	1029	CHU	No	69	0	0
0	0	Thoracotoı	1029	CHU	No	75	0	0
0	0	Thoracotoı	1029	CHU	No	54	0	0
0	0	Thoracotoı	1029	CHU	No	73	0	0
0	0	Thoracotoı	1029	CHU	No	79	0	0
0	0	Thoracotoı	1029	CHU	No	63	0	0
0	0	Thoracotoı	1029	CHU	No	67	0	0
0	0	Thoracotoı	1029	CHU	No	49	0	0
0	0	Thoracotoı	1029	CHU	No	57	0	1
0	0	Thoracotoı	1029	CHU	No	58	0	0

0	0	Thoracotoı	1029	CHU	No	70	0	0
0	0	Thoracotoı	1029	CHU	No	70	0	1
0	0	Thoracotoı	1029	CHU	No	84	1	0
0	0	Thoracotoı	1029	CHU	No	77	0	0
0	0	Thoracotoı	1029	CHU	No	69	1	1
0	1	Thoracotoı	1029	CHU	No	58	0	0
0	0	Thoracotoı	1029	CHU	No	55	0	0
0	0	Thoracotoı	1029	CHU	No	82	0	0
0	0	Thoracotoı	1029	CHU	No	57	0	1
0	0	Thoracotoı	1029	CHU	No	61	0	0
0	0	Thoracotoı	1029	CHU	No	65	0	0
0	0	Thoracotoı	1029	CHU	No	69	1	0
0	0	Thoracotoı	1029	CHU	No	69	0	1
0	0	Thoracotoı	1029	CHU	No	57	0	1
0	0	Thoracotoı	1029	CHU	No	88	0	0
0	0	Thoracotoı	1029	CHU	No	55	0	0
0	0	Thoracotoı	1029	CHU	No	82	0	0
0	0	Thoracotoı	1029	CHU	No	71	0	0
0	0	Thoracotoı	1029	CHU	No	82	0	0
0	0	Thoracotoı	1029	CHU	No	60	0	0
0	0	Thoracotoı	1029	CHU	No	70	0	0
0	0	Thoracotoı	1029	CHU	No	51	0	0
0	0	Thoracotoı	1029	CHU	No	65	0	0
0	0	Thoracotoı	1029	CHU	No	70	0	0
0	0	Thoracotoı	1029	CHU	No	60	0	1
0	0	Thoracotoı	1029	CHU	No	72	0	0
0	0	Thoracotoı	1029	CHU	No	77	0	0
0	0	VATS or rc	1029	CHU	No	71	0	0
0	1	Thoracotoı	1029	CHU	No	76	0	0
0	0	Thoracotoı	1029	CHU	No	82	0	0
0	1	Thoracotoı	1029	CHU	No	67	0	1
0	0	Thoracotoı	1029	CHU	No	64	0	0
0	0	Thoracotoı	1029	CHU	No	57	0	0
0	0	Thoracotoı	1029	CHU	No	77	0	0
0	0	Thoracotoı	1029	CHU	No	74	1	0
0	0	Thoracotoı	1029	CHU	No	78	0	0
0	0	Thoracotoı	1029	CHU	No	82	0	0
0	0	Thoracotoı	1029	CHU	No	74	0	0
0	0	Thoracotoı	1029	CHU	No	58	0	0
0	0	Thoracotoı	1029	CHU	No	42	0	0
0	0	VATS or rc	1029	CHU	No	73	0	0
0	0	Thoracotoı	1029	CHU	No	59	0	0
0	0	Thoracotoı	1029	CHU	No	45	0	0
0	0	Thoracotoı	1029	CHU	No	71	0	0
0	0	Thoracotoı	1029	CHU	No	59	0	0
0	0	Thoracotoı	1029	CHU	No	62	0	1
0	0	Thoracotoı	1029	CHU	No	75	0	0
0	0	Thoracotoı	1029	CHU	No	81	0	0
0	0	Thoracotoı	1029	CHU	No	67	0	0
0	0	Thoracotoı	1029	CHU	No	70	0	0
0	0	Thoracotoı	1029	CHU	No	56	0	0
0	0	Thoracotoı	1029	CHU	No	83	0	0
0	0	Thoracotoı	1029	CHU	No	89	0	0
0	0	Thoracotoı	1029	CHU	No	59	0	0
0	0	Thoracotoı	1029	CHU	No	73	0	0
0	0	Thoracotoı	1029	CHU	No	54	0	0

0	0	Thoracotoı	1029	CHU	No	72	1	0
0	0	VATS or rc	1029	CHU	No	66	0	0
0	1	Thoracotoı	1029	CHU	No	69	0	0
0	0	Thoracotoı	1029	CHU	No	74	0	1
0	0	Thoracotoı	1029	CHU	No	54	0	0
0	0	Thoracotoı	1029	CHU	No	74	0	0
0	0	Thoracotoı	1029	CHU	No	50	0	0
0	0	Thoracotoı	1029	CHU	No	78	0	0
0	0	VATS or rc	1029	CHU	No	80	0	1
0	0	Thoracotoı	1029	CHU	No	65	0	0
0	0	Thoracotoı	1029	CHU	No	63	0	0
0	1	Thoracotoı	1029	CHU	Yes	72	0	0
0	0	Thoracotoı	1029	CHU	No	67	0	1
0	0	Thoracotoı	1029	CHU	No	81	0	0
0	0	Thoracotoı	1029	CHU	No	65	0	0
0	0	Thoracotoı	1029	CHU	No	57	0	1
0	0	Thoracotoı	1029	CHU	No	74	1	0
0	0	Thoracotoı	1029	CHU	No	42	0	0
0	0	Thoracotoı	1029	CHU	No	51	0	0
0	0	Thoracotoı	1029	CHU	No	65	0	0
0	0	Thoracotoı	1029	CHU	No	77	0	0
0	0	Thoracotoı	1029	CHU	No	78	0	0
0	0	Thoracotoı	1029	CHU	No	43	0	0
0	0	Thoracotoı	1029	CHU	No	65	0	0
0	0	Thoracotoı	1029	CHU	No	58	0	0
0	0	Thoracotoı	1029	CHU	No	76	1	0
0	0	Thoracotoı	1029	CHU	No	67	0	0
0	0	Thoracotoı	1029	CHU	No	65	0	0
0	0	Thoracotoı	1029	CHU	No	44	0	0
0	0	Thoracotoı	1029	CHU	No	67	0	0
0	0	Thoracotoı	1029	CHU	No	77	0	0
0	0	Thoracotoı	1029	CHU	No	53	0	0
0	0	Thoracotoı	1029	CHU	No	66	0	0
0	0	Thoracotoı	1029	CHU	No	83	0	0
0	1	Thoracotoı	1029	CHU	No	70	0	1
0	0	Thoracotoı	1029	CHU	No	68	0	0
0	0	Thoracotoı	1029	CHU	No	76	0	1
0	0	Thoracotoı	1029	CHU	No	66	0	0
0	0	Thoracotoı	1029	CHU	No	56	0	0
0	0	Thoracotoı	1029	CHU	No	76	0	0
0	0	Thoracotoı	1029	CHU	No	79	0	0
0	0	Thoracotoı	1029	CHU	No	58	0	0
0	0	Thoracotoı	1029	CHU	No	63	0	0
0	0	Thoracotoı	1029	CHU	No	78	0	0
0	0	Thoracotoı	1029	CHU	No	72	0	0
0	0	Thoracotoı	1029	CHU	No	71	0	0
0	0	Thoracotoı	1029	CHU	No	67	0	0
0	0	Thoracotoı	1029	CHU	No	75	0	0
0	0	Thoracotoı	1029	CHU	No	70	0	0
0	1	Thoracotoı	1029	CHU	No	39	0	0
0	0	Thoracotoı	1029	CHU	No	88	0	1
0	0	Thoracotoı	1029	CHU	No	61	0	0
0	0	Thoracotoı	1029	CHU	No	72	0	0
0	0	Thoracotoı	1029	CHU	No	86	1	0
0	0	Thoracotoı	1029	CHU	No	69	1	0
0	0	Thoracotoı	1029	CHU	No	74	0	0

0	0	Thoracotoı	1029	CHU	No	65	0	0
0	0	Thoracotoı	1029	CHU	No	76	0	0
0	0	Thoracotoı	1029	CHU	No	76	0	0
0	0	Thoracotoı	1029	CHU	No	63	1	0
0	0	Thoracotoı	1029	CHU	No	62	0	1
0	0	Thoracotoı	1029	CHU	No	71	0	0
0	0	Thoracotoı	1029	CHU	No	70	0	0
0	0	Thoracotoı	1029	CHU	No	61	0	0
0	0	VATS or rc	1029	CHU	No	74	0	0
0	0	Thoracotoı	1029	CHU	No	77	0	0
0	0	VATS or rc	1029	CHU	No	66	0	0
0	0	Thoracotoı	1029	CHU	No	59	0	0
0	0	Thoracotoı	1029	CHU	No	69	0	0
0	0	Thoracotoı	1029	CHU	No	68	0	0
0	0	Thoracotoı	1029	CHU	No	76	0	1
0	0	Thoracotoı	1029	CHU	No	78	0	0
0	0	Thoracotoı	1029	CHU	No	86	0	0
0	0	VATS or rc	1029	CHU	No	76	0	0
0	0	Thoracotoı	1029	CHU	No	71	0	0
0	0	VATS or rc	1029	CHU	No	59	0	0
0	0	Thoracotoı	1029	CHU	No	62	0	0
0	0	Thoracotoı	1029	CHU	No	61	0	0
0	0	Thoracotoı	1029	CHU	No	63	0	0
0	0	Thoracotoı	1029	CHU	No	80	0	0
0	0	Thoracotoı	1029	CHU	No	57	0	1
0	0	Thoracotoı	1029	CHU	No	62	0	1
0	0	Thoracotoı	1029	CHU	No	58	0	0
0	0	Thoracotoı	1029	CHU	No	60	0	0
0	0	Thoracotoı	1029	CHU	No	65	0	0
0	0	Thoracotoı	1029	CHU	No	74	0	1
0	0	Thoracotoı	1029	CHU	No	65	0	0
0	0	Thoracotoı	1029	CHU	No	64	0	0
0	0	Thoracotoı	1029	CHU	No	64	1	0
0	0	Thoracotoı	1029	CHU	No	55	0	0
0	0	Thoracotoı	1029	CHU	No	61	0	0
0	0	Thoracotoı	1029	CHU	No	61	1	0
0	0	Thoracotoı	1029	CHU	No	63	0	0
0	1	Thoracotoı	1029	CHU	No	71	0	0
0	0	Thoracotoı	1029	CHU	No	68	0	0
0	0	VATS or rc	1029	CHU	No	68	0	1
0	0	Thoracotoı	1029	CHU	No	69	0	0
0	0	Thoracotoı	1029	CHU	No	69	0	0
0	0	Thoracotoı	1029	CHU	No	75	0	0
0	0	Thoracotoı	1029	CHU	No	72	0	0
0	0	Thoracotoı	1029	CHU	No	45	0	0
0	0	VATS or rc	1029	CHU	No	61	0	1
0	0	Thoracotoı	1029	CHU	No	70	0	0
0	1	Thoracotoı	1029	CHU	No	78	0	0
0	0	Thoracotoı	1029	CHU	No	70	0	1
0	0	Thoracotoı	1029	CHU	No	58	1	1
0	0	Thoracotoı	1029	CHU	No	70	1	0
0	0	Thoracotoı	1029	CHU	No	70	0	0
0	0	Thoracotoı	1029	CHU	No	67	0	0
0	0	Thoracotoı	1029	CHU	No	67	0	0
0	0	Thoracotoı	1029	CHU	No	67	0	0
0	0	Thoracotoı	1029	CHU	No	73	0	0

0	1	Thoracotoı	1029	CHU	No	59	0	0
0	0	Thoracotoı	1029	CHU	No	68	0	0
0	0	Thoracotoı	1029	CHU	No	43	0	0
0	0	Thoracotoı	1029	CHU	No	59	0	0
0	0	Thoracotoı	1029	CHU	No	87	0	0
0	0	Thoracotoı	1029	CHU	No	58	0	1
0	0	Thoracotoı	1029	CHU	No	67	0	0
0	0	Thoracotoı	1029	CHU	No	60	1	0
0	0	Thoracotoı	1029	CHU	No	73	0	0
0	0	Thoracotoı	1029	CHU	No	80	0	0
0	0	Thoracotoı	1029	CHU	No	61	0	0
0	0	VATS or rc	1029	CHU	No	56	0	0
0	0	Thoracotoı	1029	CHU	No	21	0	0
0	0	VATS or rc	1029	CHU	No	58	0	0
0	0	Thoracotoı	1029	CHU	No	70	0	0
0	0	Thoracotoı	1029	CHU	No	49	0	0
0	0	Thoracotoı	1029	CHU	No	56	0	0
0	0	Thoracotoı	1029	CHU	No	64	0	1
0	0	Thoracotoı	1029	CHU	No	77	0	0
0	0	Thoracotoı	1029	CHU	No	69	0	0
0	0	Thoracotoı	1029	CHU	No	73	0	0
0	0	Thoracotoı	1029	CHU	No	60	0	0
0	0	VATS or rc	1029	CHU	No	72	1	0
0	1	Thoracotoı	1029	CHU	No	41	0	1
0	0	VATS or rc	1029	CHU	No	69	0	0
0	0	Thoracotoı	1029	CHU	No	57	0	0
0	0	Thoracotoı	1029	CHU	No	77	0	0
0	0	Thoracotoı	1029	CHU	No	78	0	0
0	0	Thoracotoı	1029	CHU	No	76	0	0
0	0	Thoracotoı	1029	CHU	No	45	0	0
0	0	Thoracotoı	1029	CHU	No	81	0	1
0	0	Thoracotoı	1029	CHU	No	64	0	0
0	0	Thoracotoı	1029	CHU	No	51	0	0
0	0	Thoracotoı	1029	CHU	No	67	0	1
0	0	Thoracotoı	1029	CHU	No	84	0	0
0	0	Thoracotoı	1029	CHU	No	67	0	0
0	0	Thoracotoı	1029	CHU	No	57	0	0
0	0	VATS or rc	1029	CHU	No	58	0	0
0	0	VATS or rc	1029	CHU	No	73	0	0
0	0	Thoracotoı	1029	CHU	No	73	0	0
0	0	Thoracotoı	1029	CHU	No	77	0	0
0	0	VATS or rc	1029	CHU	No	71	0	0
0	1	Thoracotoı	1029	CHU	No	64	0	0
0	0	VATS or rc	1029	CHU	No	71	0	0
0	0	VATS or rc	1029	CHU	No	74	0	1
0	0	Thoracotoı	1029	CHU	No	82	0	0
0	0	Thoracotoı	1029	CHU	No	66	0	0
0	0	Thoracotoı	1029	CHU	No	53	0	0
0	0	Thoracotoı	1029	CHU	No	70	0	1
0	0	VATS or rc	1029	CHU	No	78	0	0
0	0	Thoracotoı	1029	CHU	No	82	1	0
0	0	VATS or rc	1029	CHU	No	56	0	0
0	0	Thoracotoı	1029	CHU	No	59	0	0
0	0	Thoracotoı	1029	CHU	No	78	0	0
0	0	Thoracotoı	1029	CHU	No	78	0	0
0	1	Thoracotoı	1029	CHU	No	48	0	1

0	0	Thoracotoı	1029	CHU	No	74	0	0
0	0	Thoracotoı	1029	CHU	No	49	0	0
0	0	VATS or rc	1029	CHU	No	78	0	0
0	0	Thoracotoı	1029	CHU	No	70	0	0
0	0	VATS or rc	1029	CHU	No	47	0	0
0	0	Thoracotoı	1029	CHU	No	75	0	0
0	0	Thoracotoı	1029	CHU	No	70	0	0
0	0	Thoracotoı	1029	CHU	No	62	0	0
0	0	Thoracotoı	1029	CHU	No	63	0	0
0	0	VATS or rc	1029	CHU	No	80	0	0
0	0	VATS or rc	1029	CHU	No	67	0	0
0	0	Thoracotoı	1029	CHU	No	65	0	0
0	1	Thoracotoı	1029	CHU	No	69	1	0
0	0	Thoracotoı	1029	CHU	No	74	0	0
0	0	Thoracotoı	1029	CHU	No	70	0	0
0	0	Thoracotoı	1029	CHU	No	85	0	0
0	1	Thoracotoı	1029	CHU	No	72	0	0
0	0	Thoracotoı	1029	CHU	No	82	1	0
0	0	Thoracotoı	1029	CHU	No	65	0	0
0	0	VATS or rc	1029	CHU	No	79	0	0
0	0	Thoracotoı	1029	CHU	No	84	0	0
0	0	Thoracotoı	1029	CHU	No	21	0	0
0	0	Thoracotoı	1029	CHU	No	72	0	0
0	0	Thoracotoı	1029	CHU	No	79	0	1
0	0	Thoracotoı	1029	CHU	No	76	0	0
0	0	Thoracotoı	1029	CHU	No	69	0	0
0	0	Thoracotoı	1029	CHU	No	73	0	0
0	0	Thoracotoı	1029	CHU	No	65	0	0
0	0	Thoracotoı	1029	CHU	No	70	0	0
0	0	Thoracotoı	1029	CHU	No	73	0	0
0	0	Thoracotoı	1029	CHU	No	61	0	0
0	0	Thoracotoı	1029	CHU	No	60	0	0
0	0	Thoracotoı	1029	CHU	No	66	1	0
0	0	Thoracotoı	1029	CHU	No	75	0	0
0	0	Thoracotoı	1029	CHU	No	67	0	0
0	0	Thoracotoı	1029	CHU	No	57	0	0
0	1	Thoracotoı	1029	CHU	No	19	0	0
0	0	Thoracotoı	1029	CHU	No	69	0	0
0	0	Thoracotoı	1029	CHU	No	59	0	0
0	0	Thoracotoı	1029	CHU	No	30	0	0
0	0	Thoracotoı	1029	CHU	No	63	0	0
0	0	Thoracotoı	1029	CHU	No	59	0	0
0	0	Thoracotoı	1029	CHU	No	62	0	0
0	0	Thoracotoı	1029	CHU	No	69	0	0
0	0	VATS or rc	1029	CHU	No	62	0	0
0	0	Thoracotoı	1029	CHU	No	74	0	0
0	0	VATS or rc	1029	CHU	No	69	1	0
0	0	VATS or rc	1029	CHU	No	64	1	0
0	0	Thoracotoı	1029	CHU	No	54	0	0
0	0	Thoracotoı	1029	CHU	No	51	0	0
0	0	Thoracotoı	1029	CHU	No	68	0	0
0	0	VATS or rc	1029	CHU	No	70	0	0
0	0	Thoracotoı	1029	CHU	No	78	0	0
0	0	Thoracotoı	1029	CHU	No	66	1	0
0	0	Thoracotoı	1029	CHU	No	55	0	0
0	0	Thoracotoı	1029	CHU	No	53	0	0

0	0	VATS or rc	1029	CHU	No	78	0	0
0	0	Thoracotoı	1029	CHU	No	73	0	0
0	0	Thoracotoı	1029	CHU	No	59	0	1
0	0	Thoracotoı	1029	CHU	No	62	0	1
0	0	Thoracotoı	1029	CHU	No	62	0	0
0	0	Thoracotoı	1029	CHU	No	78	0	0
0	0	VATS or rc	1029	CHU	No	68	0	0
0	0	VATS or rc	1029	CHU	No	52	1	0
0	0	Thoracotoı	1029	CHU	No	67	0	0
0	0	VATS or rc	1029	CHU	No	76	0	0
0	0	Thoracotoı	1029	CHU	No	49	0	0
0	0	Thoracotoı	1029	CHU	No	48	0	0
0	1	Thoracotoı	1029	CHU	No	54	0	0
0	0	Thoracotoı	1029	CHU	No	82	0	0
0	0	Thoracotoı	1029	CHU	No	66	0	0
0	0	Thoracotoı	1029	CHU	No	76	0	0
0	0	Thoracotoı	1029	CHU	No	74	0	0
0	0	Thoracotoı	1029	CHU	No	67	0	0
0	0	Thoracotoı	1029	CHU	No	76	0	0
0	0	VATS or rc	1029	CHU	No	58	0	1
0	0	Thoracotoı	1029	CHU	No	68	0	0
0	0	Thoracotoı	1029	CHU	No	42	0	0
0	0	Thoracotoı	1029	CHU	No	66	0	0
0	0	Thoracotoı	1029	CHU	No	56	0	0
0	0	Thoracotoı	1029	CHU	No	59	0	0
0	0	Thoracotoı	1029	CHU	No	75	0	1
0	0	Thoracotoı	1029	CHU	No	65	0	0
0	1	Thoracotoı	1029	CHU	No	54	1	0
0	0	Thoracotoı	1029	CHU	No	62	0	1
0	0	Thoracotoı	1029	CHU	No	61	0	0
0	0	VATS or rc	1029	CHU	No	64	0	0
0	0	Thoracotoı	1029	CHU	No	66	0	1
0	0	Thoracotoı	1029	CHU	No	45	0	0
0	0	VATS or rc	1029	CHU	No	73	0	0
0	0	Thoracotoı	1029	CHU	No	69	0	0
0	0	Thoracotoı	1029	CHU	No	67	0	0
0	0	Thoracotoı	1029	CHU	No	73	0	0
0	0	VATS or rc	1029	CHU	No	77	0	0
0	0	Thoracotoı	1029	CHU	No	54	0	0
0	0	Thoracotoı	1029	CHU	No	44	0	0
0	0	Thoracotoı	1029	CHU	No	65	0	0
0	0	Thoracotoı	1029	CHU	No	69	0	0
0	0	Thoracotoı	1029	CHU	No	53	0	0
0	0	Thoracotoı	1029	CHU	No	47	0	0
0	0	Thoracotoı	1029	CHU	No	66	0	0
0	0	Thoracotoı	1029	CHU	No	58	0	0
0	0	Thoracotoı	1029	CHU	No	82	0	0
0	0	Thoracotoı	1029	CHU	No	76	0	1
0	0	Thoracotoı	1029	CHU	No	69	0	0
0	0	Thoracotoı	1029	CHU	No	75	0	0
0	1	Thoracotoı	1029	CHU	No	70	0	0
0	0	Thoracotoı	1029	CHU	No	69	0	0
0	0	Thoracotoı	1029	CHU	No	62	0	0
0	0	VATS or rc	1029	CHU	No	61	0	0
0	0	Thoracotoı	1029	CHU	No	72	1	0
0	0	Thoracotoı	1029	CHU	No	83	0	1

0	0	Thoracotoı	1029	CHU	No	52	0	0
0	0	Thoracotoı	1029	CHU	No	58	0	0
0	0	Thoracotoı	1029	CHU	No	79	0	0
0	0	Thoracotoı	1029	CHU	No	51	0	0
0	0	Thoracotoı	1029	CHU	No	74	0	1
0	0	Thoracotoı	1029	CHU	No	62	0	1
0	0	VATS or rc	1029	CHU	No	78	0	0
0	0	Thoracotoı	1029	CHU	No	69	0	0
0	0	Thoracotoı	1029	CHU	No	84	0	0
0	0	Thoracotoı	1029	CHU	No	64	0	0
0	0	Thoracotoı	1029	CHU	No	60	0	0
0	0	Thoracotoı	1029	CHU	No	74	0	0
0	0	Thoracotoı	1029	CHU	No	54	0	0
0	0	VATS or rc	1029	CHU	No	61	0	0
0	0	VATS or rc	1029	CHU	No	66	0	0
0	0	Thoracotoı	1029	CHU	No	70	0	0
0	0	VATS or rc	1029	CHU	No	63	0	0
0	0	Thoracotoı	1029	CHU	No	71	0	1
0	0	Thoracotoı	1029	CHU	No	55	0	0
0	0	Thoracotoı	1029	CHU	No	73	0	0
0	0	Thoracotoı	1029	CHU	No	84	0	0
0	0	Thoracotoı	1029	CHU	No	59	0	0
0	0	Thoracotoı	1029	CHU	No	59	0	0
0	0	Thoracotoı	1029	CHU	No	74	0	0
0	0	Thoracotoı	1029	CHU	No	58	0	0
0	0	Thoracotoı	1029	CHU	No	65	0	0
0	0	VATS or rc	1029	CHU	No	64	0	0
0	0	Thoracotoı	1029	CHU	No	59	1	0
0	0	Thoracotoı	1029	CHU	No	79	0	0
0	0	Thoracotoı	1029	CHU	No	63	0	0
0	0	Thoracotoı	1029	CHU	No	46	0	0
0	0	Thoracotoı	1029	CHU	No	76	0	0
0	0	Thoracotoı	1029	CHU	Yes	74	0	0
0	0	Thoracotoı	1029	CHU	No	65	0	0
0	0	VATS or rc	1029	CHU	No	63	0	0
0	0	Thoracotoı	1029	CHU	No	63	0	0
0	0	VATS or rc	1029	CHU	No	67	0	0
0	0	Thoracotoı	1029	CHU	No	68	0	0
0	0	Thoracotoı	1029	CHU	No	42	0	0
0	1	Thoracotoı	1029	CHU	No	63	0	1
0	0	VATS or rc	1029	CHU	No	65	0	0
0	0	Thoracotoı	1029	CHU	No	63	0	0
0	0	Thoracotoı	1029	CHU	No	58	0	0
0	0	Thoracotoı	1029	CHU	No	76	0	0
0	0	Thoracotoı	1029	CHU	No	60	1	0
0	0	Thoracotoı	1029	CHU	No	60	0	0
0	0	Thoracotoı	1029	CHU	No	48	1	0
0	0	Thoracotoı	1029	CHU	No	71	1	0
0	0	Thoracotoı	1029	CHU	No	60	0	0
0	0	Thoracotoı	1029	CHU	No	83	1	0
0	0	Thoracotoı	1029	CHU	No	68	0	0
0	0	VATS or rc	1029	CHU	No	66	0	0
0	0	VATS or rc	1029	CHU	No	78	0	0
0	0	Thoracotoı	1029	CHU	No	60	1	0
0	0	Thoracotoı	1029	CHU	No	61	0	1
0	0	Thoracotoı	1029	CHU	No	36	0	1

0	0	Thoracotoı	1029	CHU	No	63	0	1
0	0	Thoracotoı	1029	CHU	No	82	1	0
0	1	Thoracotoı	1029	CHU	No	70	1	0
0	0	Thoracotoı	1029	CHU	No	55	0	0
0	0	Thoracotoı	1029	CHU	No	52	0	1
0	0	Thoracotoı	1029	CHU	No	79	0	0
0	0	Thoracotoı	1029	CHU	No	62	0	0
0	0	Thoracotoı	1029	CHU	No	65	0	0
0	0	Thoracotoı	1029	CHU	No	58	0	0
0	0	VATS or ro	1029	CHU	No	65	1	0
0	0	VATS or ro	1029	CHU	No	55	0	0
0	0	Thoracotoı	1029	CHU	No	65	0	0
0	1	Thoracotoı	1029	CHU	No	66	0	0
0	0	Thoracotoı	1029	CHU	No	72	0	0
0	0	Thoracotoı	1029	CHU	No	60	1	0
0	0	Thoracotoı	1029	CHU	No	68	0	0
0	0	Thoracotoı	1029	CHU	No	25	0	0
0	0	Thoracotoı	1029	CHU	No	46	0	0
0	0	Thoracotoı	1029	CHU	No	64	0	0
0	0	Thoracotoı	1029	CHU	No	34	0	0
0	0	VATS or ro	1029	CHU	No	79	0	0
0	0	Thoracotoı	1029	CHU	No	64	1	0
0	0	Thoracotoı	1029	CHU	No	47	0	1
0	0	Thoracotoı	1029	CHU	No	50	0	0
0	0	Thoracotoı	1029	CHU	No	69	0	0
0	0	VATS or ro	1029	CHU	No	66	1	0
0	0	Thoracotoı	1029	CHU	No	48	0	0
0	0	Thoracotoı	1029	CHU	No	65	0	0
0	0	Thoracotoı	1029	CHU	No	58	0	0
0	0	Thoracotoı	1029	CHU	No	57	0	1
0	0	Thoracotoı	1029	CHU	No	69	0	0
0	0	Thoracotoı	1029	CHU	No	63	0	0
0	0	Thoracotoı	1029	CHU	No	59	0	0
0	0	Thoracotoı	1029	CHU	No	59	0	0
0	0	Thoracotoı	1029	CHU	No	71	0	0
0	0	VATS or ro	1029	CHU	No	61	0	0
0	0	Thoracotoı	1029	CHU	No	64	0	0
0	0	Thoracotoı	1029	CHU	No	74	0	1
0	0	Thoracotoı	1029	CHU	No	90	1	0
0	1	Thoracotoı	1029	CHU	No	61	0	0
0	0	Thoracotoı	1029	CHU	No	70	0	0
0	0	Thoracotoı	1029	CHU	No	76	0	1
0	0	Thoracotoı	1029	CHU	No	60	0	0
0	0	VATS or ro	1029	CHU	No	74	0	0
0	0	Thoracotoı	1029	CHU	No	56	0	1
0	0	Thoracotoı	1029	CHU	No	81	0	0
0	1	Thoracotoı	1029	CHU	No	54	0	0
0	0	Thoracotoı	1029	CHU	No	69	0	0
0	0	Thoracotoı	1029	CHU	No	83	0	0
0	0	Thoracotoı	1029	CHU	No	72	0	0
0	0	VATS or ro	1029	CHU	No	47	0	0
0	0	Thoracotoı	1029	CHU	No	70	0	0
0	0	VATS or ro	1029	CHU	No	71	0	0
0	0	Thoracotoı	1029	CHU	No	61	0	0
0	0	Thoracotoı	1029	CHU	No	70	0	0
0	0	Thoracotoı	1029	CHU	No	58	0	1

0	0	VATS or rc	1029	CHU	No	49	1	1
0	0	Thoracotoı	1029	CHU	No	69	0	0
0	0	Thoracotoı	1029	CHU	No	63	0	0
0	0	Thoracotoı	1029	CHU	No	69	0	0
0	0	Thoracotoı	1029	CHU	No	52	0	0
0	0	Thoracotoı	1029	CHU	No	65	0	0
0	0	Thoracotoı	1029	CHU	No	75	0	1
0	0	VATS or rc	1029	CHU	No	67	0	0
0	0	Thoracotoı	1029	CHU	No	65	0	0
0	0	Thoracotoı	1029	CHU	No	82	0	0
0	0	VATS or rc	1029	CHU	No	68	0	0
0	0	VATS or rc	1029	CHU	No	49	0	1
0	0	Thoracotoı	1029	CHU	No	58	0	0
0	0	Thoracotoı	1029	CHU	No	66	0	0
0	0	VATS or rc	1029	CHU	No	69	1	0
0	0	Thoracotoı	1029	CHU	No	64	0	1
0	0	Thoracotoı	1029	CHU	No	64	0	0
0	0	Thoracotoı	1029	CHU	No	58	0	0
0	0	Thoracotoı	1029	CHU	No	63	0	0
0	0	Thoracotoı	1029	CHU	Yes	78	0	1
0	0	VATS or rc	1029	CHU	No	57	0	0
0	0	Thoracotoı	1029	CHU	No	62	0	0
0	0	Thoracotoı	1029	CHU	No	77	0	0
0	1	Thoracotoı	1029	CHU	No	49	0	0
0	0	Thoracotoı	1029	CHU	No	68	0	0
0	0	Thoracotoı	1029	CHU	No	61	1	0
0	0	VATS or rc	1029	CHU	No	74	1	0
0	0	Thoracotoı	1029	CHU	No	72	0	0
0	0	Thoracotoı	1029	CHU	No	71	0	1
0	0	Thoracotoı	1029	CHU	No	63	0	0
0	0	Thoracotoı	1029	CHU	No	38	0	0
0	0	Thoracotoı	1029	CHU	No	79	0	0
0	0	Thoracotoı	1029	CHU	No	66	0	1
0	1	Thoracotoı	1029	CHU	No	64	0	0
0	0	Thoracotoı	1029	CHU	No	53	0	0
0	0	Thoracotoı	1029	CHU	No	67	0	0
0	0	Thoracotoı	1029	CHU	No	60	0	0
0	0	VATS or rc	1029	CHU	No	67	1	0
0	0	Thoracotoı	1029	CHU	No	56	0	0
0	0	Thoracotoı	1029	CHU	No	56	0	0
0	0	Thoracotoı	1029	CHU	No	74	0	0
0	0	Thoracotoı	1029	CHU	No	69	0	0
0	0	Thoracotoı	1029	CHU	No	61	0	1
0	0	VATS or rc	1029	CHU	No	61	0	0
0	0	Thoracotoı	1029	CHU	No	59	0	0
0	0	Thoracotoı	1029	CHU	No	60	0	0
0	0	VATS or rc	1029	CHU	No	67	0	0
0	0	Thoracotoı	1029	CHU	No	72	0	0
0	0	Thoracotoı	1029	CHU	No	69	0	0
0	0	Thoracotoı	1029	CHU	No	65	0	0
0	0	Thoracotoı	1029	CHU	No	62	0	0
0	0	VATS or rc	232	PRIVE	No	71	0	0
0	0	VATS or rc	232	PRIVE	No	73	0	0
0	0	VATS or rc	232	PRIVE	No	64	0	0
0	0	VATS or rc	232	PRIVE	No	64	0	0
0	0	Thoracotoı	232	PRIVE	No	62	0	0

0	0	VATS or rc	232	PRIVE	No	56	0	1
0	0	VATS or rc	232	PRIVE	No	57	0	0
0	0	VATS or rc	232	PRIVE	No	64	0	0
0	0	VATS or rc	232	PRIVE	No	68	0	0
0	0	Thoracotoı	232	PRIVE	No	67	0	0
0	0	VATS or rc	232	PRIVE	No	69	1	1
0	0	VATS or rc	232	PRIVE	No	65	0	0
0	0	VATS or rc	232	PRIVE	No	71	0	0
0	0	VATS or rc	232	PRIVE	No	61	0	0
0	0	VATS or rc	232	PRIVE	No	68	1	0
0	0	VATS or rc	232	PRIVE	No	51	0	0
0	0	VATS or rc	232	PRIVE	No	44	0	0
0	0	VATS or rc	232	PRIVE	No	79	0	0
0	0	VATS or rc	232	PRIVE	No	57	0	0
0	0	VATS or rc	232	PRIVE	No	69	0	0
0	0	Thoracotoı	232	PRIVE	No	76	0	0
0	0	Thoracotoı	232	PRIVE	No	69	1	0
0	0	VATS or rc	232	PRIVE	No	51	0	0
0	0	VATS or rc	232	PRIVE	No	61	0	0
0	0	Thoracotoı	232	PRIVE	No	46	1	0
0	0	VATS or rc	232	PRIVE	No	71	1	0
0	0	VATS or rc	232	PRIVE	No	80	0	0
0	0	Thoracotoı	232	PRIVE	No	57	0	0
0	0	Thoracotoı	232	PRIVE	No	73	0	0
0	0	Thoracotoı	232	PRIVE	No	74	0	0
0	0	VATS or rc	232	PRIVE	No	69	0	0
0	0	Thoracotoı	232	PRIVE	No	75	0	0
0	0	VATS or rc	232	PRIVE	No	67	0	0
0	0	VATS or rc	232	PRIVE	No	84	0	0
0	0	VATS or rc	232	PRIVE	No	73	0	0
0	0	VATS or rc	232	PRIVE	No	78	0	0
0	0	VATS or rc	232	PRIVE	No	64	0	0
0	0	VATS or rc	232	PRIVE	No	49	0	0
0	0	Thoracotoı	232	PRIVE	No	37	0	0
0	1	Thoracotoı	232	PRIVE	No	68	0	0
0	0	VATS or rc	232	PRIVE	No	67	0	0
0	0	Thoracotoı	232	PRIVE	No	73	0	0
0	1	Thoracotoı	232	PRIVE	No	66	1	0
0	0	VATS or rc	232	PRIVE	No	69	0	0
0	0	VATS or rc	232	PRIVE	No	73	0	1
0	0	Thoracotoı	232	PRIVE	No	68	0	0
0	0	VATS or rc	232	PRIVE	No	60	0	1
0	0	VATS or rc	232	PRIVE	No	70	0	0
0	0	VATS or rc	232	PRIVE	No	57	0	0
0	0	Thoracotoı	232	PRIVE	No	66	0	0
0	0	VATS or rc	232	PRIVE	No	66	0	0
0	0	VATS or rc	232	PRIVE	No	72	0	0
0	0	VATS or rc	232	PRIVE	No	69	0	0
0	0	VATS or rc	232	PRIVE	No	72	0	0
0	0	VATS or rc	232	PRIVE	No	57	0	1
0	0	Thoracotoı	232	PRIVE	No	54	0	0
0	0	VATS or rc	232	PRIVE	No	71	0	0
0	0	Thoracotoı	232	PRIVE	No	47	0	1
0	0	VATS or rc	232	PRIVE	No	58	0	0
0	1	Thoracotoı	232	PRIVE	No	63	0	0
0	0	Thoracotoı	232	PRIVE	No	64	0	0

0	0	Thoracotoı	232	PRIVE	No	74	0	0
0	0	Thoracotoı	232	PRIVE	No	70	0	0
0	0	VATS or rc	232	PRIVE	No	61	0	0
0	0	VATS or rc	232	PRIVE	No	55	0	0
0	0	VATS or rc	232	PRIVE	No	81	0	0
0	0	Thoracotoı	232	PRIVE	No	50	0	0
0	0	VATS or rc	232	PRIVE	No	82	0	0
0	0	VATS or rc	232	PRIVE	No	62	0	0
0	0	VATS or rc	232	PRIVE	No	69	0	0
0	0	Thoracotoı	232	PRIVE	No	68	0	0
0	0	VATS or rc	232	PRIVE	No	81	0	0
0	0	VATS or rc	232	PRIVE	No	62	0	0
0	1	Thoracotoı	232	PRIVE	No	64	0	0
0	0	VATS or rc	232	PRIVE	No	54	0	0
0	0	VATS or rc	232	PRIVE	No	75	0	0
0	0	VATS or rc	232	PRIVE	No	56	0	0
0	0	VATS or rc	232	PRIVE	No	56	0	0
0	0	VATS or rc	232	PRIVE	No	77	0	0
0	0	VATS or rc	232	PRIVE	No	63	0	0
0	1	Thoracotoı	232	PRIVE	No	64	0	0
0	0	VATS or rc	232	PRIVE	No	55	0	0
0	0	VATS or rc	232	PRIVE	No	64	0	0
0	0	VATS or rc	232	PRIVE	No	60	0	0
0	0	VATS or rc	232	PRIVE	No	64	0	1
0	0	VATS or rc	232	PRIVE	No	68	0	0
0	0	Thoracotoı	232	PRIVE	No	72	0	0
0	0	VATS or rc	232	PRIVE	No	80	0	0
0	0	VATS or rc	232	PRIVE	No	57	0	0
0	0	Thoracotoı	232	PRIVE	No	64	0	0
0	0	VATS or rc	232	PRIVE	No	69	0	0
0	0	Thoracotoı	232	PRIVE	No	64	1	0
0	0	VATS or rc	232	PRIVE	No	73	0	0
0	0	VATS or rc	232	PRIVE	No	63	0	0
0	0	VATS or rc	232	PRIVE	No	72	0	0
0	0	Thoracotoı	232	PRIVE	No	81	0	0
0	0	VATS or rc	232	PRIVE	No	62	0	0
0	0	VATS or rc	232	PRIVE	No	69	0	1
0	0	Thoracotoı	232	PRIVE	No	82	0	0
0	0	VATS or rc	232	PRIVE	No	72	0	0
0	0	VATS or rc	232	PRIVE	No	65	0	0
0	0	VATS or rc	232	PRIVE	No	69	0	0
0	0	Thoracotoı	232	PRIVE	No	54	0	0
0	0	VATS or rc	232	PRIVE	No	84	0	0
0	1	Thoracotoı	232	PRIVE	No	72	0	0
0	0	VATS or rc	232	PRIVE	No	63	0	0
0	0	VATS or rc	232	PRIVE	No	57	0	0
0	0	Thoracotoı	232	PRIVE	No	53	0	0
0	0	Thoracotoı	232	PRIVE	No	72	1	0
0	0	Thoracotoı	232	PRIVE	No	82	0	0
0	1	Thoracotoı	232	PRIVE	No	85	0	0
0	0	Thoracotoı	232	PRIVE	No	68	0	0
0	0	VATS or rc	232	PRIVE	No	62	1	0
0	0	VATS or rc	232	PRIVE	No	68	0	0
0	0	VATS or rc	232	PRIVE	No	63	0	0
0	0	VATS or rc	232	PRIVE	No	60	0	0
0	0	Thoracotoı	232	PRIVE	No	48	0	0

0	0	VATS or ro	232	PRIVE	No	48	0	0
0	0	VATS or ro	232	PRIVE	No	44	0	0
0	0	VATS or ro	232	PRIVE	No	69	0	0
0	0	VATS or ro	232	PRIVE	No	75	0	0
0	0	VATS or ro	232	PRIVE	No	72	0	0
0	0	VATS or ro	232	PRIVE	No	60	0	0
0	0	VATS or ro	232	PRIVE	No	74	0	0
0	0	VATS or ro	232	PRIVE	No	61	0	0
0	0	VATS or ro	232	PRIVE	No	73	0	0
0	0	VATS or ro	232	PRIVE	No	77	0	0
0	0	VATS or ro	232	PRIVE	No	68	0	0
0	0	VATS or ro	232	PRIVE	No	73	0	0
0	0	VATS or ro	232	PRIVE	No	64	0	0
0	0	VATS or ro	232	PRIVE	No	60	0	0
0	0	Thoracotor	232	PRIVE	No	69	0	0
0	0	Thoracotor	232	PRIVE	No	61	0	0
0	0	VATS or ro	232	PRIVE	No	54	0	0
0	0	VATS or ro	232	PRIVE	No	76	0	0
0	0	VATS or ro	232	PRIVE	No	69	0	0
0	0	Thoracotor	232	PRIVE	No	67	0	0
0	0	VATS or ro	232	PRIVE	No	73	0	0
0	0	Thoracotor	232	PRIVE	No	59	0	0
0	0	VATS or ro	232	PRIVE	No	74	0	0
0	0	VATS or ro	232	PRIVE	No	64	0	0
0	0	VATS or ro	232	PRIVE	No	79	0	0
0	0	VATS or ro	232	PRIVE	No	59	0	0
0	0	VATS or ro	232	PRIVE	No	84	0	0
0	0	VATS or ro	232	PRIVE	No	56	0	0
0	0	Thoracotor	232	PRIVE	No	57	0	1
0	0	Thoracotor	232	PRIVE	No	66	0	0
0	0	VATS or ro	232	PRIVE	No	76	0	0
0	0	VATS or ro	232	PRIVE	No	71	0	1
0	0	VATS or ro	232	PRIVE	No	66	0	0
0	0	VATS or ro	232	PRIVE	No	68	0	0
0	0	VATS or ro	232	PRIVE	No	78	0	0
0	0	Thoracotor	232	PRIVE	No	72	0	0
0	0	Thoracotor	232	PRIVE	No	53	0	1
0	0	VATS or ro	232	PRIVE	No	81	0	0
0	0	VATS or ro	232	PRIVE	No	53	0	0
0	0	VATS or ro	232	PRIVE	No	58	0	0
0	0	VATS or ro	232	PRIVE	No	45	0	0
0	0	VATS or ro	232	PRIVE	No	76	0	0
0	0	Thoracotor	232	PRIVE	No	55	0	0
0	0	VATS or ro	232	PRIVE	No	54	0	0
0	0	VATS or ro	232	PRIVE	No	69	0	1
0	0	VATS or ro	232	PRIVE	No	57	0	0
0	0	VATS or ro	232	PRIVE	No	74	0	0
0	0	VATS or ro	232	PRIVE	No	62	0	0
0	0	VATS or ro	232	PRIVE	No	63	0	0
0	0	VATS or ro	232	PRIVE	No	69	0	0
0	0	VATS or ro	232	PRIVE	No	67	0	0
0	0	Thoracotor	232	PRIVE	No	68	0	0
0	0	VATS or ro	232	PRIVE	No	59	0	0
0	0	Thoracotor	232	PRIVE	No	69	0	0
0	0	Thoracotor	232	PRIVE	No	46	0	0
0	0	Thoracotor	232	PRIVE	No	72	0	0

0	0	Thoracotoı	232	PRIVE	No	63	0	0
0	0	Thoracotoı	232	PRIVE	No	70	0	0
0	0	Thoracotoı	232	PRIVE	No	68	0	0
0	0	VATS or rc	232	PRIVE	No	73	0	0
0	0	VATS or rc	232	PRIVE	No	46	0	0
0	0	VATS or rc	232	PRIVE	No	78	0	0
0	0	VATS or rc	232	PRIVE	No	50	0	0
0	0	Thoracotoı	232	PRIVE	No	73	0	0
0	0	Thoracotoı	232	PRIVE	No	53	0	0
0	0	Thoracotoı	232	PRIVE	No	59	1	0
0	0	VATS or rc	232	PRIVE	No	67	0	0
0	0	VATS or rc	232	PRIVE	No	37	0	0
0	0	VATS or rc	232	PRIVE	No	60	0	0
0	0	VATS or rc	232	PRIVE	No	61	0	0
0	0	VATS or rc	232	PRIVE	No	57	0	0
0	0	Thoracotoı	232	PRIVE	No	60	0	0
0	0	VATS or rc	232	PRIVE	No	69	0	0
0	0	VATS or rc	232	PRIVE	No	76	0	0
0	0	Thoracotoı	232	PRIVE	No	74	0	0
0	0	VATS or rc	232	PRIVE	No	52	0	0
0	0	VATS or rc	232	PRIVE	No	69	1	0
0	0	VATS or rc	232	PRIVE	No	71	0	0
0	0	VATS or rc	232	PRIVE	No	76	1	0
0	0	VATS or rc	232	PRIVE	No	68	0	1
0	0	VATS or rc	232	PRIVE	No	77	1	0
0	0	VATS or rc	232	PRIVE	No	53	1	0
0	0	VATS or rc	232	PRIVE	No	77	0	0
0	0	VATS or rc	232	PRIVE	No	82	0	0
0	0	VATS or rc	232	PRIVE	No	72	0	0
0	0	VATS or rc	232	PRIVE	No	73	0	0
0	0	VATS or rc	232	PRIVE	No	78	0	0
0	0	Thoracotoı	232	PRIVE	No	69	0	0
0	0	VATS or rc	232	PRIVE	No	66	1	0
0	0	VATS or rc	232	PRIVE	No	61	0	0
0	0	VATS or rc	232	PRIVE	No	58	0	1
0	0	Thoracotoı	232	PRIVE	No	41	0	0
0	0	VATS or rc	232	PRIVE	No	71	0	0
0	0	VATS or rc	232	PRIVE	No	65	0	0
0	0	VATS or rc	232	PRIVE	No	70	0	0
0	0	VATS or rc	232	PRIVE	No	48	0	0
0	0	Thoracotoı	232	PRIVE	No	46	0	0
0	0	VATS or rc	232	PRIVE	No	59	0	0
0	0	Thoracotoı	232	PRIVE	No	49	0	0
0	0	VATS or rc	232	PRIVE	No	60	0	0
0	0	Thoracotoı	232	PRIVE	No	60	0	0
0	0	VATS or rc	232	PRIVE	No	57	0	0
0	0	VATS or rc	232	PRIVE	No	65	0	1
0	0	VATS or rc	232	PRIVE	No	46	0	0
0	0	VATS or rc	232	PRIVE	No	63	0	1
0	0	VATS or rc	232	PRIVE	No	50	0	0
0	0	VATS or rc	232	PRIVE	No	60	0	0
0	0	Thoracotoı	232	PRIVE	No	68	0	0
0	0	Thoracotoı	232	PRIVE	No	61	1	0
0	0	Thoracotoı	232	PRIVE	No	60	0	0
0	0	VATS or rc	232	PRIVE	No	71	0	0
0	0	VATS or rc	232	PRIVE	No	72	0	0

0	0	VATS or rc	232	PRIVE	No	74	0	0
0	0	Thoracotoı	232	PRIVE	No	43	0	0
0	0	VATS or rc	232	PRIVE	No	68	0	1
0	0	VATS or rc	232	PRIVE	No	59	1	0
0	0	VATS or rc	232	PRIVE	No	47	0	1
0	0	VATS or rc	232	PRIVE	No	68	0	0
0	0	VATS or rc	232	PRIVE	No	86	0	0
0	0	VATS or rc	232	PRIVE	No	66	0	0
0	0	VATS or rc	232	PRIVE	No	79	0	0
0	0	VATS or rc	232	PRIVE	No	78	0	0
0	0	VATS or rc	232	PRIVE	No	73	0	0
0	0	Thoracotoı	232	PRIVE	No	72	1	0
0	0	Thoracotoı	232	PRIVE	No	69	0	0
0	0	VATS or rc	232	PRIVE	No	61	0	0
0	0	Thoracotoı	232	PRIVE	No	66	1	0
0	0	Thoracotoı	232	PRIVE	No	74	0	0
0	0	VATS or rc	232	PRIVE	No	52	0	0
0	0	Thoracotoı	232	PRIVE	No	71	0	0
0	0	Thoracotoı	232	PRIVE	No	82	0	0
0	0	Thoracotoı	232	PRIVE	No	71	1	0
0	0	Thoracotoı	232	PRIVE	No	66	0	0
0	0	VATS or rc	232	PRIVE	No	60	0	0
0	0	VATS or rc	232	PRIVE	No	72	0	0
0	0	VATS or rc	232	PRIVE	No	67	0	1
0	0	Thoracotoı	232	PRIVE	No	52	0	0
0	0	VATS or rc	232	PRIVE	No	68	0	0
0	0	VATS or rc	232	PRIVE	No	63	0	0
0	0	VATS or rc	232	PRIVE	No	66	0	0
0	0	VATS or rc	232	PRIVE	No	72	0	1
0	0	VATS or rc	232	PRIVE	No	77	1	0
0	0	VATS or rc	232	PRIVE	No	67	0	1
0	0	VATS or rc	232	PRIVE	No	35	0	0
0	0	VATS or rc	232	PRIVE	No	68	0	0
0	0	VATS or rc	232	PRIVE	No	61	0	0
0	0	VATS or rc	232	PRIVE	No	55	0	0
0	0	VATS or rc	232	PRIVE	No	24	0	0
0	0	VATS or rc	232	PRIVE	No	72	0	0
0	0	VATS or rc	232	PRIVE	No	80	0	0
0	0	Thoracotoı	232	PRIVE	No	62	0	0
0	0	VATS or rc	232	PRIVE	No	51	0	0
0	0	Thoracotoı	232	PRIVE	No	48	0	1
0	0	VATS or rc	232	PRIVE	No	67	0	0
0	0	Thoracotoı	232	PRIVE	No	55	0	0
0	0	VATS or rc	232	PRIVE	No	69	0	0
0	0	VATS or rc	232	PRIVE	No	45	0	0
0	0	VATS or rc	232	PRIVE	No	50	0	1
0	1	Thoracotoı	232	PRIVE	No	74	0	0
0	0	Thoracotoı	232	PRIVE	No	71	0	0
0	0	Thoracotoı	232	PRIVE	No	53	0	0
0	0	VATS or rc	232	PRIVE	No	59	0	0
0	0	VATS or rc	232	PRIVE	No	66	1	0
0	0	VATS or rc	232	PRIVE	No	60	0	0
0	0	VATS or rc	232	PRIVE	No	71	0	0
0	0	VATS or rc	232	PRIVE	No	83	0	0
0	0	VATS or rc	232	PRIVE	No	60	0	0
0	0	VATS or rc	232	PRIVE	No	72	0	0

0	0	VATS or rc	232	PRIVE	No	70	0	0
0	0	VATS or rc	232	PRIVE	No	46	0	0
0	0	VATS or rc	232	PRIVE	No	58	0	0
0	0	Thoracotor	232	PRIVE	No	66	1	1
0	0	Thoracotor	232	PRIVE	No	68	0	0
0	0	VATS or rc	232	PRIVE	No	68	0	0
0	0	VATS or rc	232	PRIVE	No	49	0	0
0	0	VATS or rc	232	PRIVE	No	69	0	0
0	1	Thoracotor	232	PRIVE	No	72	0	0
0	0	VATS or rc	232	PRIVE	No	77	0	0
0	0	VATS or rc	232	PRIVE	No	53	0	0
0	0	VATS or rc	232	PRIVE	No	68	0	0
0	0	VATS or rc	232	PRIVE	No	52	0	0
0	0	Thoracotor	232	PRIVE	No	80	0	0
0	1	Thoracotor	232	PRIVE	No	72	1	1
0	0	VATS or rc	232	PRIVE	No	58	0	0
0	0	VATS or rc	232	PRIVE	No	72	0	0
0	0	VATS or rc	232	PRIVE	No	82	0	0
0	0	VATS or rc	232	PRIVE	No	65	0	0
0	0	VATS or rc	232	PRIVE	No	57	0	0
0	0	VATS or rc	232	PRIVE	No	73	0	0
0	0	VATS or rc	232	PRIVE	No	67	0	0
0	1	Thoracotor	232	PRIVE	No	67	0	0
0	0	VATS or rc	232	PRIVE	No	76	0	0
0	0	VATS or rc	232	PRIVE	No	71	0	0
0	0	Thoracotor	232	PRIVE	No	83	0	0
0	0	Thoracotor	232	PRIVE	No	67	0	0
0	0	VATS or rc	232	PRIVE	No	67	0	0
0	0	VATS or rc	232	PRIVE	No	71	0	0
0	0	VATS or rc	232	PRIVE	No	37	0	0
0	0	Thoracotor	232	PRIVE	No	64	1	0
0	0	VATS or rc	232	PRIVE	No	73	0	0
0	0	VATS or rc	232	PRIVE	No	47	0	0
0	0	Thoracotor	232	PRIVE	No	76	0	0
0	0	VATS or rc	232	PRIVE	No	65	0	0
0	0	VATS or rc	232	PRIVE	No	67	0	0
0	0	Thoracotor	232	PRIVE	No	79	0	0
0	0	VATS or rc	232	PRIVE	No	62	0	0
0	0	VATS or rc	232	PRIVE	No	62	1	1
0	1	Thoracotor	232	PRIVE	No	69	0	0
0	0	Thoracotor	232	PRIVE	No	78	1	0
0	0	VATS or rc	232	PRIVE	No	70	0	0
0	0	VATS or rc	232	PRIVE	No	84	0	1
0	0	VATS or rc	232	PRIVE	No	50	0	0
0	0	Thoracotor	232	PRIVE	No	70	0	0
0	0	Thoracotor	232	PRIVE	No	64	0	0
0	0	VATS or rc	232	PRIVE	No	69	0	0
0	0	VATS or rc	232	PRIVE	No	66	0	0
0	0	Thoracotor	232	PRIVE	Yes	51	0	0
0	0	VATS or rc	232	PRIVE	No	51	0	0
0	0	Thoracotor	232	PRIVE	No	76	0	0
0	1	Thoracotor	232	PRIVE	No	56	0	0
0	0	VATS or rc	232	PRIVE	No	64	0	0
0	0	VATS or rc	232	PRIVE	No	70	0	0
0	0	VATS or rc	232	PRIVE	No	56	0	0
0	0	VATS or rc	232	PRIVE	No	51	0	0

0	0	VATS or ro	232	PRIVE	No	53	0	0
0	0	VATS or ro	232	PRIVE	No	62	0	0
0	0	VATS or ro	232	PRIVE	No	65	0	0
0	0	VATS or ro	232	PRIVE	No	69	0	0
0	0	VATS or ro	232	PRIVE	No	62	0	0
0	0	VATS or ro	232	PRIVE	No	66	0	0
0	0	VATS or ro	232	PRIVE	No	69	0	0
0	0	Thoracotor	232	PRIVE	No	67	0	0
0	0	VATS or ro	232	PRIVE	No	71	0	0
0	0	VATS or ro	232	PRIVE	No	67	0	0
0	0	Thoracotor	232	PRIVE	No	72	0	0
0	0	VATS or ro	232	PRIVE	No	71	0	0
0	0	VATS or ro	232	PRIVE	No	68	0	0
0	0	VATS or ro	232	PRIVE	No	66	0	0
0	0	VATS or ro	232	PRIVE	No	68	0	0
0	0	VATS or ro	232	PRIVE	No	61	0	1
0	0	VATS or ro	232	PRIVE	No	59	0	0
0	0	VATS or ro	232	PRIVE	No	57	0	1
0	0	Thoracotor	232	PRIVE	No	83	0	0
0	0	VATS or ro	232	PRIVE	No	52	0	0
0	0	VATS or ro	232	PRIVE	No	68	0	0
0	0	VATS or ro	232	PRIVE	No	67	0	0
0	0	Thoracotor	232	PRIVE	No	66	0	0
0	0	Thoracotor	232	PRIVE	No	75	0	0
0	0	VATS or ro	232	PRIVE	No	76	0	0
0	0	VATS or ro	232	PRIVE	No	46	0	0
0	0	Thoracotor	232	PRIVE	No	58	0	0
0	0	VATS or ro	232	PRIVE	No	59	0	0
0	0	VATS or ro	232	PRIVE	No	48	0	0
0	1	Thoracotor	232	PRIVE	No	50	0	0
0	0	Thoracotor	232	PRIVE	No	70	0	0
0	0	VATS or ro	232	PRIVE	No	70	0	0
0	0	VATS or ro	232	PRIVE	No	70	1	0
0	0	VATS or ro	232	PRIVE	No	55	1	0
0	0	VATS or ro	232	PRIVE	No	61	0	0
0	0	VATS or ro	232	PRIVE	No	64	0	0
0	0	Thoracotor	232	PRIVE	No	52	0	0
0	0	Thoracotor	232	PRIVE	No	71	0	0
0	0	VATS or ro	232	PRIVE	No	60	0	0
0	0	Thoracotor	232	PRIVE	No	51	0	0
0	0	Thoracotor	232	PRIVE	No	66	0	0
0	0	VATS or ro	232	PRIVE	No	76	0	0
0	0	Thoracotor	232	PRIVE	No	78	0	1
0	0	VATS or ro	232	PRIVE	No	71	0	0
0	0	VATS or ro	232	PRIVE	No	56	0	0
0	0	Thoracotor	232	PRIVE	No	79	1	0
0	0	VATS or ro	232	PRIVE	No	68	0	0
0	0	Thoracotor	232	PRIVE	No	61	0	0
0	0	VATS or ro	232	PRIVE	No	58	0	0
0	0	VATS or ro	232	PRIVE	No	58	0	0
0	0	VATS or ro	232	PRIVE	No	76	0	0
0	0	VATS or ro	232	PRIVE	No	50	0	0
0	0	VATS or ro	232	PRIVE	No	53	0	0
0	0	VATS or ro	232	PRIVE	No	29	0	0
0	0	VATS or ro	232	PRIVE	No	75	0	0
0	0	VATS or ro	232	PRIVE	No	60	0	0

0	0	VATS or rc	232	PRIVE	No	62	0	0
0	0	Thoracotoı	232	PRIVE	No	62	0	0
0	0	VATS or rc	232	PRIVE	No	56	0	0
0	0	VATS or rc	232	PRIVE	No	76	0	0
0	0	VATS or rc	232	PRIVE	No	71	1	0
0	0	VATS or rc	232	PRIVE	No	56	0	0
0	0	VATS or rc	232	PRIVE	No	73	1	0
0	0	VATS or rc	232	PRIVE	No	73	0	0
0	0	Thoracotoı	232	PRIVE	No	45	1	0
0	0	VATS or rc	232	PRIVE	No	50	0	0
0	0	VATS or rc	232	PRIVE	No	65	0	0
0	0	VATS or rc	232	PRIVE	No	69	0	0
0	0	VATS or rc	232	PRIVE	No	54	0	0
0	0	VATS or rc	232	PRIVE	No	57	0	1
0	0	VATS or rc	232	PRIVE	No	53	0	0
0	0	VATS or rc	232	PRIVE	No	68	0	0
0	0	VATS or rc	232	PRIVE	No	82	1	1
0	0	VATS or rc	232	PRIVE	No	57	0	1
0	0	VATS or rc	232	PRIVE	No	66	0	0
0	0	Thoracotoı	232	PRIVE	No	65	0	0
0	0	Thoracotoı	232	PRIVE	No	77	0	0
0	0	VATS or rc	232	PRIVE	No	74	0	0
0	0	VATS or rc	232	PRIVE	No	75	0	0
0	0	VATS or rc	232	PRIVE	No	64	0	0
0	0	VATS or rc	232	PRIVE	No	58	0	0
0	0	VATS or rc	232	PRIVE	No	77	0	0
0	0	VATS or rc	232	PRIVE	No	63	0	1
0	0	VATS or rc	232	PRIVE	No	52	0	1
0	0	VATS or rc	232	PRIVE	No	59	0	0
0	0	VATS or rc	232	PRIVE	No	62	0	0
0	0	VATS or rc	232	PRIVE	No	78	0	0
0	0	Thoracotoı	232	PRIVE	No	79	0	0
0	0	VATS or rc	232	PRIVE	No	75	0	0
0	0	VATS or rc	232	PRIVE	No	50	0	0
0	0	VATS or rc	232	PRIVE	No	79	0	0
0	0	VATS or rc	232	PRIVE	No	71	0	0
0	0	Thoracotoı	232	PRIVE	No	69	0	0
0	0	Thoracotoı	232	PRIVE	No	61	1	0
0	0	VATS or rc	232	PRIVE	No	54	0	0
0	0	VATS or rc	232	PRIVE	No	73	0	0
0	0	Thoracotoı	232	PRIVE	No	68	0	0
0	0	Thoracotoı	232	PRIVE	No	77	1	0
0	0	VATS or rc	232	PRIVE	No	78	0	0
0	0	Thoracotoı	232	PRIVE	No	32	0	0
0	0	VATS or rc	232	PRIVE	No	72	0	0
0	0	VATS or rc	232	PRIVE	No	60	0	0
0	0	VATS or rc	232	PRIVE	No	51	0	0
0	0	VATS or rc	232	PRIVE	No	73	0	0
0	0	Thoracotoı	232	PRIVE	No	56	0	1
0	0	VATS or rc	232	PRIVE	No	63	0	0
0	0	Thoracotoı	232	PRIVE	No	67	0	0
0	0	VATS or rc	232	PRIVE	No	63	0	0
0	1	Thoracotoı	232	PRIVE	No	76	0	0
0	0	VATS or rc	232	PRIVE	No	66	0	0
0	0	Thoracotoı	232	PRIVE	No	66	0	0
0	0	VATS or rc	232	PRIVE	No	74	0	0

0	0	VATS or rc	232	PRIVE	No	67	0	0
0	0	Thoracotoı	232	PRIVE	No	67	1	0
0	0	VATS or rc	232	PRIVE	No	74	0	1
0	0	VATS or rc	232	PRIVE	No	69	0	0
0	0	VATS or rc	232	PRIVE	No	46	0	0
0	0	VATS or rc	232	PRIVE	No	48	0	0
0	0	VATS or rc	232	PRIVE	No	74	1	0
0	1	Thoracotoı	232	PRIVE	No	59	1	0
0	0	Thoracotoı	232	PRIVE	No	76	0	0
0	0	VATS or rc	232	PRIVE	No	72	0	0
0	0	Thoracotoı	232	PRIVE	No	70	0	0
0	0	Thoracotoı	232	PRIVE	No	65	0	0
0	0	VATS or rc	232	PRIVE	No	66	0	0
0	0	Thoracotoı	232	PRIVE	No	66	0	0
0	0	VATS or rc	232	PRIVE	No	69	0	0
0	0	VATS or rc	232	PRIVE	No	64	0	0
0	0	VATS or rc	232	PRIVE	No	62	0	0
0	0	VATS or rc	232	PRIVE	No	53	0	0
0	0	VATS or rc	232	PRIVE	No	79	0	0
0	0	VATS or rc	232	PRIVE	No	64	0	0
0	0	VATS or rc	232	PRIVE	No	74	0	0
0	0	VATS or rc	232	PRIVE	No	74	0	0
0	0	VATS or rc	232	PRIVE	No	59	0	0
0	0	VATS or rc	232	PRIVE	No	68	0	0
0	0	VATS or rc	232	PRIVE	No	48	0	0
0	0	VATS or rc	232	PRIVE	No	61	0	0
0	0	VATS or rc	232	PRIVE	No	54	0	0
0	0	VATS or rc	232	PRIVE	No	85	0	0
0	0	Thoracotoı	232	PRIVE	No	78	0	0
0	0	VATS or rc	232	PRIVE	No	65	0	0
0	0	VATS or rc	232	PRIVE	No	65	0	0
0	0	VATS or rc	1029	CHU	No	69	1	1
0	0	Thoracotoı	1029	CHU	No	47	0	1
0	0	VATS or rc	1029	CHU	No	65	0	1
0	0	Thoracotoı	1029	CHU	No	71	0	0
0	0	Thoracotoı	1029	CHU	No	71	0	0
0	0	Thoracotoı	1029	CHU	No	51	0	0
0	0	VATS or rc	1029	CHU	No	63	0	0
0	0	Thoracotoı	1029	CHU	No	44	0	0
0	0	VATS or rc	1029	CHU	No	76	0	0
0	0	VATS or rc	1029	CHU	No	76	0	0
0	0	VATS or rc	1029	CHU	No	68	0	1
0	0	VATS or rc	1029	CHU	No	79	1	1
0	1	VATS or rc	1029	CHU	No	43	0	0
0	0	VATS or rc	1029	CHU	No	74	0	0
0	0	VATS or rc	1029	CHU	No	68	0	0
0	0	VATS or rc	1029	CHU	No	80	0	0
0	0	Thoracotoı	1029	CHU	No	64	0	0
0	0	VATS or rc	1029	CHU	No	61	0	0
0	0	VATS or rc	1029	CHU	No	75	0	1
0	0	VATS or rc	1029	CHU	No	66	0	0
0	0	Thoracotoı	1029	CHU	No	43	0	0
0	1	Thoracotoı	1029	CHU	No	60	0	0
0	1	Thoracotoı	1029	CHU	No	81	0	0
0	0	Thoracotoı	1029	CHU	No	70	0	0
0	0	Thoracotoı	1029	CHU	No	78	0	0

0	0	Thoracotoı	1029	CHU	No	79	1	0
0	0	Thoracotoı	1029	CHU	No	62	0	0
0	0	Thoracotoı	1029	CHU	No	72	0	0
0	0	VATS or rc	1029	CHU	No	46	0	0
0	0	Thoracotoı	1029	CHU	No	68	1	1
0	0	VATS or rc	1029	CHU	No	58	0	0
0	0	Thoracotoı	1029	CHU	No	60	0	0
0	1	Thoracotoı	1029	CHU	No	65	0	0
0	0	VATS or rc	1029	CHU	No	76	0	0
0	0	VATS or rc	1029	CHU	No	57	0	1
0	0	VATS or rc	1029	CHU	No	67	0	0
0	0	VATS or rc	1029	CHU	No	53	0	1
0	1	Thoracotoı	1029	CHU	No	77	0	0
0	0	VATS or rc	1029	CHU	No	75	1	0
0	0	VATS or rc	1029	CHU	No	63	0	0
0	0	VATS or rc	1029	CHU	No	71	0	0
0	0	VATS or rc	1029	CHU	No	62	0	0
0	0	Thoracotoı	1029	CHU	No	75	0	0
0	0	VATS or rc	1029	CHU	No	73	0	0
0	0	VATS or rc	1029	CHU	No	45	0	0
0	0	Thoracotoı	1029	CHU	No	46	0	0
0	1	Thoracotoı	1029	CHU	No	62	0	0
0	0	Thoracotoı	1029	CHU	No	47	0	0
0	0	VATS or rc	1029	CHU	No	80	0	1
0	0	VATS or rc	1029	CHU	No	58	0	0
0	0	Thoracotoı	1029	CHU	No	72	0	0
0	0	VATS or rc	1029	CHU	No	55	1	1
0	0	VATS or rc	1029	CHU	No	63	0	0
0	0	VATS or rc	1029	CHU	No	65	0	0
0	0	VATS or rc	1029	CHU	No	61	0	0
0	0	VATS or rc	1029	CHU	No	73	0	0
0	0	Thoracotoı	1029	CHU	No	66	1	0
0	0	Thoracotoı	1029	CHU	No	62	0	1
0	1	Thoracotoı	1029	CHU	No	50	0	0
0	1	Thoracotoı	1029	CHU	No	73	1	0
0	0	VATS or rc	1029	CHU	No	57	0	0
0	0	VATS or rc	1029	CHU	No	75	0	0
0	0	Thoracotoı	1029	CHU	No	65	1	0
0	0	VATS or rc	1029	CHU	No	74	0	0
0	0	VATS or rc	1029	CHU	No	65	0	1
0	0	VATS or rc	1029	CHU	No	68	0	0
0	0	VATS or rc	1029	CHU	No	45	0	0
0	0	VATS or rc	1029	CHU	No	40	0	0
0	0	Thoracotoı	1029	CHU	No	56	1	0
0	0	VATS or rc	1029	CHU	No	73	1	0
0	0	Thoracotoı	1029	CHU	No	72	0	0
0	0	Thoracotoı	1029	CHU	No	76	0	0
0	0	Thoracotoı	1029	CHU	No	73	0	0
0	0	Thoracotoı	1029	CHU	No	56	0	1
0	0	VATS or rc	1029	CHU	No	74	1	0
0	0	VATS or rc	1029	CHU	No	63	0	1
0	0	VATS or rc	1029	CHU	No	64	0	1
0	0	Thoracotoı	1029	CHU	No	44	0	0
0	0	VATS or rc	1029	CHU	No	67	0	0
0	0	Thoracotoı	1029	CHU	No	62	0	0
0	0	VATS or rc	1029	CHU	No	67	0	0

0	0	VATS or ro	1029	CHU	No	42	0	0
0	0	VATS or ro	1029	CHU	No	64	1	0
0	0	VATS or ro	1029	CHU	No	67	0	1
0	0	VATS or ro	1029	CHU	No	53	0	0
0	0	VATS or ro	1029	CHU	No	69	1	0
0	0	Thoracotoı	1029	CHU	No	70	0	0
0	0	Thoracotoı	1029	CHU	No	73	0	1
0	0	VATS or ro	1029	CHU	No	74	1	0
0	0	VATS or ro	1029	CHU	No	42	0	0
0	0	Thoracotoı	1029	CHU	No	61	0	1
0	0	Thoracotoı	1029	CHU	No	66	0	0
0	0	Thoracotoı	1029	CHU	No	57	0	1
0	0	Thoracotoı	1029	CHU	No	63	0	0
0	0	Thoracotoı	1029	CHU	No	72	0	0
0	0	Thoracotoı	1029	CHU	No	58	0	1
0	0	VATS or ro	1029	CHU	No	62	0	0
0	0	VATS or ro	1029	CHU	No	73	0	0
0	0	Thoracotoı	1029	CHU	No	54	0	0
0	0	Thoracotoı	1029	CHU	No	79	0	0
0	0	VATS or ro	1029	CHU	No	62	0	0
0	0	VATS or ro	1029	CHU	No	57	0	1
0	0	VATS or ro	1029	CHU	No	65	0	1
0	0	Thoracotoı	1029	CHU	No	71	1	1
0	0	VATS or ro	1029	CHU	No	57	1	1
0	1	Thoracotoı	1029	CHU	No	76	0	1
0	1	Thoracotoı	1029	CHU	No	68	1	1
0	0	Thoracotoı	1029	CHU	No	59	0	0
0	0	VATS or ro	1029	CHU	No	61	0	1
0	0	Thoracotoı	1029	CHU	No	77	0	0
0	0	VATS or ro	1029	CHU	No	58	0	0
0	0	Thoracotoı	1029	CHU	No	69	0	0
0	0	Thoracotoı	1029	CHU	No	46	0	0
0	0	Thoracotoı	1029	CHU	No	57	0	0
0	0	VATS or ro	1029	CHU	No	76	0	0
0	0	VATS or ro	1029	CHU	No	63	0	0
0	0	Thoracotoı	1029	CHU	No	59	0	0
0	0	VATS or ro	1029	CHU	No	69	0	1
0	0	VATS or ro	1029	CHU	No	70	0	1
0	0	VATS or ro	1029	CHU	No	58	0	0
0	0	Thoracotoı	1029	CHU	No	73	0	0
0	0	VATS or ro	1029	CHU	No	73	0	0
0	0	VATS or ro	1029	CHU	No	61	0	0
0	0	Thoracotoı	1029	CHU	No	61	0	0
0	0	VATS or ro	1029	CHU	No	63	0	0
0	0	VATS or ro	1029	CHU	No	70	1	1
0	0	Thoracotoı	1029	CHU	No	50	0	1
0	0	VATS or ro	1029	CHU	No	75	0	0
0	0	VATS or ro	1029	CHU	No	60	0	0
0	0	VATS or ro	1029	CHU	No	74	0	0
0	1	Thoracotoı	1029	CHU	No	66	0	0
0	0	VATS or ro	1029	CHU	No	77	0	0
0	1	Thoracotoı	1029	CHU	No	74	0	1
0	0	VATS or ro	1029	CHU	No	77	0	1
0	0	Thoracotoı	1029	CHU	No	80	0	1
0	0	VATS or ro	1029	CHU	No	63	0	0
0	0	VATS or ro	1029	CHU	No	43	1	0

0	0	VATS or rc	1029	CHU	No	44	0	0
0	0	VATS or rc	1029	CHU	No	71	0	1
0	0	Thoracotoı	1029	CHU	No	66	0	0
0	0	Thoracotoı	1029	CHU	No	63	0	1
0	0	Thoracotoı	1029	CHU	No	70	0	1
0	1	Thoracotoı	1029	CHU	No	76	0	0
0	1	Thoracotoı	1029	CHU	No	43	0	0
0	0	VATS or rc	1029	CHU	No	68	0	0
0	1	Thoracotoı	1029	CHU	No	43	0	0
0	0	VATS or rc	1029	CHU	No	52	0	0
0	1	Thoracotoı	1029	CHU	No	25	0	0
0	0	VATS or rc	1029	CHU	No	77	0	0
0	0	Thoracotoı	1029	CHU	No	50	0	0
0	0	Thoracotoı	1029	CHU	No	65	1	1
0	0	Thoracotoı	1029	CHU	No	74	0	1
0	0	Thoracotoı	1029	CHU	No	61	0	0
0	0	VATS or rc	1029	CHU	No	80	0	0
0	0	VATS or rc	1029	CHU	No	70	0	0
0	0	Thoracotoı	1029	CHU	No	58	0	0
0	1	Thoracotoı	1029	CHU	No	51	0	0
0	0	Thoracotoı	1029	CHU	No	68	0	0
0	1	Thoracotoı	1029	CHU	No	87	0	0
0	0	VATS or rc	1029	CHU	No	61	0	0
0	1	Thoracotoı	1029	CHU	No	55	0	1
0	0	VATS or rc	1029	CHU	No	72	0	0
0	0	Thoracotoı	1029	CHU	No	75	0	1
0	0	VATS or rc	1029	CHU	No	59	0	0
0	0	VATS or rc	1029	CHU	No	61	0	0
0	0	VATS or rc	1029	CHU	No	68	0	0
0	1	Thoracotoı	1029	CHU	No	63	0	0
0	0	VATS or rc	1029	CHU	No	63	1	1
0	1	Thoracotoı	1029	CHU	No	78	0	0
0	0	Thoracotoı	1029	CHU	No	73	0	0
0	0	Thoracotoı	1029	CHU	No	61	0	0
0	0	Thoracotoı	1029	CHU	No	78	0	1
0	0	VATS or rc	1029	CHU	No	28	0	0
0	0	VATS or rc	1029	CHU	No	67	0	0
0	0	VATS or rc	1029	CHU	No	60	0	0
0	0	Thoracotoı	1029	CHU	No	59	0	0
0	0	VATS or rc	1029	CHU	No	60	0	0
0	0	Thoracotoı	1029	CHU	No	63	0	0
0	1	VATS or rc	1029	CHU	No	61	0	0
0	0	VATS or rc	1029	CHU	No	56	0	0
0	0	Thoracotoı	1029	CHU	No	72	1	0
0	0	Thoracotoı	1029	CHU	No	65	0	0
0	0	Thoracotoı	1029	CHU	No	64	0	0
0	0	Thoracotoı	1029	CHU	No	66	1	0
0	0	Thoracotoı	1029	CHU	No	77	0	1
0	0	Thoracotoı	1029	CHU	No	64	0	0
0	0	VATS or rc	1029	CHU	No	42	0	0
0	0	VATS or rc	1029	CHU	No	63	0	0
0	0	VATS or rc	1029	CHU	No	68	0	1
0	1	Thoracotoı	1029	CHU	No	74	0	0
0	0	VATS or rc	1029	CHU	No	79	0	0
0	0	VATS or rc	1029	CHU	No	62	0	1
0	0	Thoracotoı	1029	CHU	No	65	0	0

0	0	VATS or ro	1029	CHU	No	85	0	1
0	0	VATS or ro	1029	CHU	No	68	0	0
0	0	VATS or ro	1029	CHU	No	59	0	1
0	0	VATS or ro	1029	CHU	No	67	0	0
0	0	Thoracotor	1029	CHU	No	69	0	0
0	0	Thoracotor	1029	CHU	No	66	0	0
0	0	VATS or ro	1029	CHU	No	63	0	0
0	0	Thoracotor	1029	CHU	No	65	0	0
0	0	VATS or ro	1029	CHU	No	68	0	0
0	0	VATS or ro	1029	CHU	No	75	0	0
0	0	VATS or ro	1029	CHU	No	68	0	0
0	0	VATS or ro	1029	CHU	No	76	0	0
0	0	Thoracotor	1029	CHU	No	59	0	0
0	0	Thoracotor	1029	CHU	No	68	0	0
0	0	Thoracotor	1029	CHU	No	74	0	1
0	0	Thoracotor	1029	CHU	No	62	0	0
0	0	VATS or ro	1029	CHU	No	72	0	0
0	0	VATS or ro	1029	CHU	No	72	0	0
0	1	Thoracotor	1029	CHU	No	63	0	1
0	0	Thoracotor	1029	CHU	No	49	0	0
0	0	VATS or ro	1029	CHU	No	66	0	0
0	0	Thoracotor	1029	CHU	No	72	0	0
0	0	VATS or ro	1029	CHU	No	69	0	0
0	0	VATS or ro	1029	CHU	No	74	0	0
0	1	Thoracotor	1029	CHU	No	65	0	0
1	0	VATS or ro	44	CHG	No	70	0	1
1	0	Thoracotor	44	CHG	No	80	0	1
1	0	Thoracotor	44	CHG	No	78	0	0
1	0	Thoracotor	44	CHG	No	80	0	1
1	0	Thoracotor	44	CHG	No	77	0	0
1	0	Thoracotor	44	CHG	No	75	1	0
1	1	Thoracotor	44	CHG	No	59	0	0
1	0	Thoracotor	44	CHG	No	60	0	1
1	0	VATS or ro	44	CHG	No	49	0	1
1	0	VATS or ro	44	CHG	No	71	0	0
1	0	Thoracotor	44	CHG	No	80	0	0
1	0	Thoracotor	44	CHG	No	67	0	0
1	0	VATS or ro	44	CHG	No	54	0	1
1	0	Thoracotor	44	CHG	No	74	0	1
1	1	Thoracotor	44	CHG	Yes	64	1	1
1	0	Thoracotor	44	CHG	No	65	0	1
1	0	Thoracotor	44	CHG	No	72	0	1
1	0	Thoracotor	44	CHG	No	65	0	0
1	0	Thoracotor	44	CHG	No	54	0	0
1	0	Thoracotor	44	CHG	No	58	0	1
1	0	Thoracotor	44	CHG	No	59	0	1
1	0	VATS or ro	44	CHG	No	60	0	1
1	0	Thoracotor	44	CHG	No	72	0	0
1	0	Thoracotor	44	CHG	No	55	0	1
1	0	VATS or ro	44	CHG	No	67	0	1
1	1	Thoracotor	44	CHG	No	59	0	0
1	0	Thoracotor	44	CHG	No	52	0	1
1	0	Thoracotor	44	CHG	No	79	0	0
1	0	Thoracotor	44	CHG	No	72	0	0
1	0	Thoracotor	44	CHG	No	73	0	1
1	0	Thoracotor	44	CHG	No	60	0	0

1	0	Thoracotoı	44	CHG	No	73	0	1
1	0	VATS or rc	44	CHG	No	71	0	1
1	0	VATS or rc	44	CHG	No	54	0	0
1	0	VATS or rc	44	CHG	No	29	0	0
1	0	Thoracotoı	44	CHG	No	52	0	0
1	0	Thoracotoı	44	CHG	No	73	0	0
1	0	Thoracotoı	44	CHG	No	77	0	1
1	0	Thoracotoı	44	CHG	No	57	0	1
1	0	Thoracotoı	44	CHG	No	65	0	1
1	0	Thoracotoı	44	CHG	No	60	0	1
1	0	Thoracotoı	44	CHG	No	80	0	0
1	0	Thoracotoı	44	CHG	No	52	0	0
1	0	Thoracotoı	44	CHG	No	59	0	0
1	0	Thoracotoı	44	CHG	No	76	0	0
1	0	Thoracotoı	44	CHG	No	79	0	0
1	0	VATS or rc	44	CHG	No	63	0	1
1	0	Thoracotoı	44	CHG	No	52	0	0
1	0	Thoracotoı	44	CHG	No	70	0	0
1	0	VATS or rc	44	CHG	No	74	0	0
1	0	Thoracotoı	44	CHG	No	67	0	0
1	0	VATS or rc	44	CHG	No	66	0	1
1	0	Thoracotoı	44	CHG	No	68	0	1
1	0	Thoracotoı	44	CHG	No	65	0	0
1	0	VATS or rc	44	CHG	No	60	0	1
1	0	Thoracotoı	44	CHG	No	72	0	0
1	0	Thoracotoı	44	CHG	No	70	0	0
1	0	Thoracotoı	44	CHG	No	47	0	1
1	0	Thoracotoı	44	CHG	No	63	0	1
1	0	Thoracotoı	44	CHG	No	64	0	0
1	0	Thoracotoı	44	CHG	No	58	0	0
1	0	Thoracotoı	44	CHG	No	78	0	0
1	0	Thoracotoı	44	CHG	No	59	0	0
1	0	Thoracotoı	44	CHG	No	75	0	0
1	0	VATS or rc	44	CHG	No	55	0	0
1	0	Thoracotoı	44	CHG	No	48	0	1
1	0	VATS or rc	44	CHG	No	63	0	1
1	0	Thoracotoı	44	CHG	Yes	80	0	0
1	0	Thoracotoı	44	CHG	No	65	0	1
1	0	Thoracotoı	44	CHG	No	62	0	0
1	0	Thoracotoı	44	CHG	No	57	0	1
1	0	Thoracotoı	44	CHG	No	58	0	1
1	0	Thoracotoı	44	CHG	No	66	0	0
1	1	Thoracotoı	44	CHG	No	59	0	0
1	0	Thoracotoı	44	CHG	No	56	0	0
1	0	Thoracotoı	44	CHG	No	65	0	0
1	1	Thoracotoı	44	CHG	No	59	0	1
1	0	Thoracotoı	44	CHG	No	59	0	1
1	0	Thoracotoı	44	CHG	No	65	0	1
1	1	Thoracotoı	44	CHG	No	59	0	1
1	0	Thoracotoı	44	CHG	No	55	0	0
1	0	VATS or rc	44	CHG	No	74	1	0
1	0	Thoracotoı	44	CHG	No	59	0	1
1	0	Thoracotoı	44	CHG	No	67	0	1
1	0	Thoracotoı	44	CHG	No	87	0	1
1	0	Thoracotoı	44	CHG	No	54	0	0
1	0	VATS or rc	44	CHG	No	74	0	0

1	0	Thoracotoı	44	CHG	No	66	0	1
1	0	VATS or rc	44	CHG	No	68	0	1
1	0	Thoracotoı	44	CHG	No	53	0	0
1	0	Thoracotoı	25	CHG	No	74	0	0
1	0	Thoracotoı	25	CHG	No	72	1	0
1	0	Thoracotoı	25	CHG	No	67	0	0
1	0	Thoracotoı	25	CHG	No	82	0	1
1	1	Thoracotoı	25	CHG	No	59	0	0
1	0	Thoracotoı	25	CHG	No	54	0	0
1	0	Thoracotoı	25	CHG	No	71	0	0
1	0	Thoracotoı	25	CHG	No	68	1	0
1	0	Thoracotoı	25	CHG	No	70	0	0
1	0	Thoracotoı	25	CHG	No	53	0	0
1	0	Thoracotoı	25	CHG	No	62	0	1
1	0	Thoracotoı	25	CHG	No	67	0	0
1	0	Thoracotoı	25	CHG	No	73	0	0
1	0	Thoracotoı	25	CHG	No	57	0	0
1	0	Thoracotoı	25	CHG	No	69	1	0
1	0	Thoracotoı	25	CHG	No	80	0	0
1	0	Thoracotoı	25	CHG	Yes	67	0	0
1	0	Thoracotoı	25	CHG	No	64	0	0
1	0	Thoracotoı	25	CHG	No	62	0	1
1	0	Thoracotoı	25	CHG	No	58	0	0
1	0	Thoracotoı	25	CHG	No	74	0	0
1	0	Thoracotoı	25	CHG	No	73	0	0
1	0	Thoracotoı	25	CHG	No	57	0	0
1	0	Thoracotoı	25	CHG	No	57	0	0
1	0	Thoracotoı	25	CHG	No	83	0	0
1	0	Thoracotoı	25	CHG	No	66	0	0
1	1	Thoracotoı	25	CHG	No	67	0	0
1	1	Thoracotoı	25	CHG	No	58	0	0
1	0	Thoracotoı	25	CHG	No	64	0	0
1	0	Thoracotoı	25	CHG	No	50	0	0
1	0	Thoracotoı	25	CHG	No	68	0	1
1	0	Thoracotoı	25	CHG	No	62	0	1
1	0	Thoracotoı	25	CHG	No	74	0	0
1	1	Thoracotoı	25	CHG	No	42	0	0
1	0	Thoracotoı	25	CHG	No	76	0	0
1	0	Thoracotoı	25	CHG	No	48	1	0
1	0	Thoracotoı	25	CHG	No	62	0	0
1	0	Thoracotoı	25	CHG	No	77	0	1
1	0	Thoracotoı	25	CHG	No	72	0	0
1	0	Thoracotoı	25	CHG	No	70	0	0
1	0	Thoracotoı	25	CHG	No	62	0	0
1	0	Thoracotoı	25	CHG	No	78	0	0
1	0	Thoracotoı	25	CHG	No	62	0	1
1	0	Thoracotoı	25	CHG	No	67	0	0
0	0	VATS or rc	41	PRIVE	No	67	1	0
0	0	Thoracotoı	41	PRIVE	No	59	0	0
0	0	Thoracotoı	41	PRIVE	No	71	0	0
0	0	Thoracotoı	41	PRIVE	No	70	0	0
0	0	Thoracotoı	41	PRIVE	No	75	0	0
0	0	Thoracotoı	41	PRIVE	No	57	0	0
0	0	Thoracotoı	41	PRIVE	No	72	1	0
0	0	Thoracotoı	41	PRIVE	No	71	0	0
0	0	Thoracotoı	41	PRIVE	No	77	0	0

0	0	Thoracotoı	41	PRIVE	No	64	1	0
0	1	Thoracotoı	41	PRIVE	No	62	0	0
0	0	Thoracotoı	41	PRIVE	No	69	1	0
0	0	Thoracotoı	41	PRIVE	No	55	0	0
0	0	Thoracotoı	41	PRIVE	No	70	0	0
0	0	Thoracotoı	41	PRIVE	No	66	0	0
0	0	Thoracotoı	41	PRIVE	No	61	0	0
0	0	Thoracotoı	41	PRIVE	No	56	0	0
0	0	Thoracotoı	41	PRIVE	No	71	0	0
0	0	Thoracotoı	41	PRIVE	No	72	1	0
0	0	Thoracotoı	41	PRIVE	No	65	0	0
0	0	Thoracotoı	41	PRIVE	No	69	0	0
0	0	Thoracotoı	41	PRIVE	No	45	0	0
0	0	Thoracotoı	41	PRIVE	No	63	0	0
0	0	Thoracotoı	41	PRIVE	No	79	0	0
0	0	Thoracotoı	41	PRIVE	No	44	0	0
0	0	VATS or rc	41	PRIVE	No	72	0	0
0	0	Thoracotoı	41	PRIVE	No	64	1	0
0	0	Thoracotoı	41	PRIVE	No	58	1	0
0	0	Thoracotoı	41	PRIVE	No	65	0	0
0	0	Thoracotoı	41	PRIVE	No	54	0	0
0	0	Thoracotoı	41	PRIVE	No	58	1	0
0	1	Thoracotoı	41	PRIVE	No	73	0	0
0	0	Thoracotoı	41	PRIVE	No	70	1	0
0	0	Thoracotoı	41	PRIVE	No	56	1	0
0	0	Thoracotoı	41	PRIVE	No	64	0	0
0	0	Thoracotoı	41	PRIVE	No	65	0	0
0	0	VATS or rc	41	PRIVE	No	61	0	0
0	0	Thoracotoı	41	PRIVE	No	75	0	0
0	0	Thoracotoı	41	PRIVE	No	76	0	0
0	0	Thoracotoı	41	PRIVE	No	77	0	0
0	0	Thoracotoı	41	PRIVE	No	65	1	0
0	0	Thoracotoı	41	PRIVE	No	56	0	0
0	0	Thoracotoı	41	PRIVE	No	64	0	0
0	0	Thoracotoı	41	PRIVE	No	77	0	0
0	0	Thoracotoı	41	PRIVE	No	65	1	0
0	0	Thoracotoı	41	PRIVE	No	74	0	0
0	0	Thoracotoı	41	PRIVE	No	45	0	0
0	0	Thoracotoı	41	PRIVE	No	68	1	0
0	1	Thoracotoı	41	PRIVE	No	59	0	0
0	0	Thoracotoı	41	PRIVE	No	53	0	0
0	0	Thoracotoı	41	PRIVE	No	72	0	0
0	0	Thoracotoı	41	PRIVE	No	70	0	0
0	0	Thoracotoı	41	PRIVE	No	59	1	0
0	0	Thoracotoı	41	PRIVE	No	61	0	0
0	0	Thoracotoı	41	PRIVE	No	67	0	0
0	0	Thoracotoı	41	PRIVE	No	70	0	0
0	0	Thoracotoı	41	PRIVE	No	77	0	0
0	0	Thoracotoı	41	PRIVE	No	78	0	0
0	0	Thoracotoı	41	PRIVE	No	54	0	0
0	0	Thoracotoı	41	PRIVE	No	66	0	0
0	0	Thoracotoı	41	PRIVE	No	61	0	0
0	0	Thoracotoı	41	PRIVE	No	75	0	0
0	0	Thoracotoı	41	PRIVE	No	84	1	0
0	0	Thoracotoı	41	PRIVE	No	70	0	0
0	0	Thoracotoı	41	PRIVE	No	62	0	0

0	0	Thoracotoı	41	PRIVE	No	61	0	0
0	0	VATS or rc	41	PRIVE	No	78	0	0
0	0	Thoracotoı	41	PRIVE	No	81	0	0
0	0	VATS or rc	41	PRIVE	No	68	0	0
0	0	VATS or rc	41	PRIVE	No	55	0	0
0	0	Thoracotoı	41	PRIVE	No	67	0	0
0	1	Thoracotoı	41	PRIVE	No	66	1	0
0	0	Thoracotoı	41	PRIVE	No	57	0	0
0	0	Thoracotoı	41	PRIVE	No	48	0	0
0	0	Thoracotoı	41	PRIVE	No	63	0	0
0	0	Thoracotoı	41	PRIVE	No	72	0	0
0	0	Thoracotoı	41	PRIVE	No	47	0	0
0	0	Thoracotoı	41	PRIVE	No	70	0	0
0	0	Thoracotoı	41	PRIVE	No	66	0	0
0	0	Thoracotoı	41	PRIVE	No	79	0	0
0	0	Thoracotoı	41	PRIVE	No	64	0	0
0	0	Thoracotoı	41	PRIVE	No	73	1	0
0	0	VATS or rc	41	PRIVE	No	70	0	0
0	0	VATS or rc	41	PRIVE	No	64	0	0
0	0	Thoracotoı	41	PRIVE	No	48	0	0
0	0	Thoracotoı	41	PRIVE	No	65	1	0
0	0	Thoracotoı	41	PRIVE	No	73	0	0
0	0	Thoracotoı	41	PRIVE	No	78	0	0
0	0	VATS or rc	41	PRIVE	No	60	0	0
0	0	Thoracotoı	41	PRIVE	No	61	0	0
0	0	VATS or rc	41	PRIVE	No	59	0	0
0	0	VATS or rc	41	PRIVE	No	57	0	0
0	0	VATS or rc	41	PRIVE	No	62	0	0
0	0	Thoracotoı	41	PRIVE	No	67	0	0
0	0	Thoracotoı	41	PRIVE	No	61	0	0
0	0	Thoracotoı	41	PRIVE	No	74	0	0
0	1	Thoracotoı	41	PRIVE	No	63	0	0
0	0	Thoracotoı	41	PRIVE	No	62	0	0
0	0	Thoracotoı	41	PRIVE	No	60	0	0
0	0	Thoracotoı	41	PRIVE	No	51	1	0
0	0	VATS or rc	41	PRIVE	No	70	0	0
0	0	Thoracotoı	41	PRIVE	No	65	0	1
0	0	Thoracotoı	41	PRIVE	No	63	0	0
0	0	Thoracotoı	41	PRIVE	No	78	0	0
0	0	Thoracotoı	41	PRIVE	No	32	0	0
0	0	Thoracotoı	41	PRIVE	No	74	0	0
0	0	VATS or rc	215	CHU	No	68	0	0
0	0	VATS or rc	215	CHU	No	57	0	1
0	1	Thoracotoı	215	CHU	No	56	0	0
0	0	Thoracotoı	215	CHU	No	69	0	0
0	0	VATS or rc	215	CHU	No	67	0	0
0	0	Thoracotoı	215	CHU	No	69	1	0
0	0	VATS or rc	215	CHU	No	71	0	0
0	0	Thoracotoı	215	CHU	No	59	0	1
0	0	Thoracotoı	215	CHU	No	77	0	0
0	0	Thoracotoı	215	CHU	No	61	0	0
0	0	VATS or rc	215	CHU	No	62	0	1
0	0	Thoracotoı	215	CHU	No	58	0	1
0	0	Thoracotoı	215	CHU	No	67	0	0
0	0	Thoracotoı	215	CHU	No	55	0	1
0	0	VATS or rc	215	CHU	No	61	0	0

0	0	VATS or rc	215	CHU	No	67	0	0
0	0	VATS or rc	215	CHU	No	49	0	0
0	0	VATS or rc	215	CHU	No	67	0	0
0	0	VATS or rc	215	CHU	No	52	0	0
0	0	Thoracotoı	215	CHU	No	60	1	0
0	0	Thoracotoı	215	CHU	No	76	0	0
0	0	Thoracotoı	215	CHU	No	50	0	0
0	0	Thoracotoı	215	CHU	No	74	0	1
0	0	Thoracotoı	215	CHU	No	50	0	0
0	0	Thoracotoı	215	CHU	No	67	0	0
0	0	Thoracotoı	215	CHU	Yes	75	0	1
0	0	Thoracotoı	215	CHU	No	53	0	0
0	0	Thoracotoı	215	CHU	No	64	0	0
0	0	Thoracotoı	215	CHU	No	66	1	0
0	0	Thoracotoı	215	CHU	No	74	1	1
0	0	Thoracotoı	215	CHU	No	58	0	1
0	0	VATS or rc	215	CHU	No	64	0	1
0	0	Thoracotoı	215	CHU	No	70	0	0
0	0	Thoracotoı	215	CHU	No	51	0	0
0	0	Thoracotoı	215	CHU	No	64	0	0
0	1	Thoracotoı	215	CHU	No	38	0	1
0	0	VATS or rc	215	CHU	No	54	0	0
0	0	Thoracotoı	215	CHU	No	67	0	1
0	0	VATS or rc	215	CHU	No	36	0	1
0	0	VATS or rc	215	CHU	No	54	0	0
0	0	VATS or rc	215	CHU	No	62	0	0
0	0	VATS or rc	215	CHU	No	67	0	0
0	0	VATS or rc	215	CHU	No	68	0	0
0	0	Thoracotoı	215	CHU	No	55	0	1
0	0	VATS or rc	215	CHU	No	84	0	0
0	0	VATS or rc	215	CHU	No	76	0	1
0	0	VATS or rc	215	CHU	No	57	1	1
0	0	VATS or rc	215	CHU	No	64	0	1
0	0	VATS or rc	215	CHU	No	69	0	0
0	0	VATS or rc	215	CHU	No	68	1	0
0	0	VATS or rc	215	CHU	No	55	0	0
0	0	Thoracotoı	215	CHU	No	44	0	0
0	0	VATS or rc	215	CHU	No	68	0	0
0	0	Thoracotoı	215	CHU	No	58	0	0
0	0	VATS or rc	215	CHU	No	59	0	0
0	0	Thoracotoı	215	CHU	No	47	0	0
0	0	VATS or rc	215	CHU	No	52	0	1
0	0	Thoracotoı	215	CHU	No	60	0	1
0	0	VATS or rc	215	CHU	No	70	1	0
0	0	Thoracotoı	215	CHU	No	52	0	1
0	0	VATS or rc	215	CHU	No	78	0	0
0	0	Thoracotoı	215	CHU	No	72	0	0
0	0	VATS or rc	215	CHU	No	53	0	0
0	0	VATS or rc	215	CHU	No	54	0	0
0	0	VATS or rc	215	CHU	No	62	0	0
0	0	VATS or rc	215	CHU	No	68	0	1
0	0	Thoracotoı	215	CHU	No	73	1	0
0	0	VATS or rc	215	CHU	No	60	0	0
0	0	VATS or rc	215	CHU	No	74	0	0
0	0	VATS or rc	215	CHU	No	46	0	0
0	0	Thoracotoı	215	CHU	No	68	0	0

0	0	VATS or rc	215	CHU	No	70	0	0
0	0	Thoracotoı	215	CHU	No	55	0	0
0	0	VATS or rc	215	CHU	No	60	0	0
0	0	Thoracotoı	215	CHU	No	77	0	0
0	0	VATS or rc	215	CHU	No	70	0	1
0	0	VATS or rc	215	CHU	No	50	0	0
0	0	Thoracotoı	215	CHU	No	61	0	0
0	0	Thoracotoı	215	CHU	No	56	0	0
0	0	Thoracotoı	215	CHU	No	68	0	1
0	0	Thoracotoı	215	CHU	No	68	1	0
0	0	Thoracotoı	215	CHU	No	67	0	1
0	0	VATS or rc	215	CHU	No	61	0	0
0	0	Thoracotoı	215	CHU	No	56	0	1
0	0	VATS or rc	215	CHU	No	60	0	0
0	0	VATS or rc	215	CHU	No	58	0	0
0	0	Thoracotoı	215	CHU	No	66	0	1
0	0	Thoracotoı	215	CHU	No	70	0	1
0	0	Thoracotoı	215	CHU	No	73	0	0
0	0	Thoracotoı	215	CHU	No	61	0	0
0	0	Thoracotoı	215	CHU	No	59	0	1
0	0	VATS or rc	215	CHU	No	56	0	0
0	0	VATS or rc	215	CHU	No	66	0	0
0	0	Thoracotoı	215	CHU	No	73	0	0
0	0	VATS or rc	215	CHU	No	71	0	0
0	0	VATS or rc	215	CHU	No	67	0	0
0	0	Thoracotoı	215	CHU	No	66	0	0
0	0	Thoracotoı	215	CHU	No	64	0	0
0	0	VATS or rc	215	CHU	No	56	1	1
0	0	VATS or rc	215	CHU	No	66	0	0
0	0	Thoracotoı	215	CHU	No	62	0	1
0	0	VATS or rc	215	CHU	No	69	0	0
0	0	Thoracotoı	215	CHU	No	71	0	1
0	0	VATS or rc	215	CHU	No	73	0	0
0	0	Thoracotoı	215	CHU	No	39	0	1
0	0	Thoracotoı	215	CHU	No	54	0	0
0	0	VATS or rc	215	CHU	No	62	0	0
0	0	Thoracotoı	215	CHU	No	68	0	0
0	0	VATS or rc	215	CHU	No	63	0	1
0	0	VATS or rc	215	CHU	No	67	0	0
0	0	VATS or rc	215	CHU	No	69	0	1
0	0	VATS or rc	215	CHU	No	63	0	1
0	0	VATS or rc	215	CHU	No	64	0	0
0	0	Thoracotoı	215	CHU	No	61	0	0
0	0	VATS or rc	215	CHU	No	73	0	0
0	0	Thoracotoı	215	CHU	No	78	1	1
0	0	Thoracotoı	215	CHU	No	59	0	0
0	0	VATS or rc	215	CHU	No	76	0	0
0	0	Thoracotoı	215	CHU	No	63	0	0
0	0	VATS or rc	215	CHU	No	71	0	0
0	0	Thoracotoı	215	CHU	No	77	0	0
0	0	Thoracotoı	215	CHU	No	68	1	0
0	0	Thoracotoı	215	CHU	No	52	0	1
0	0	Thoracotoı	215	CHU	No	66	0	0
0	0	Thoracotoı	215	CHU	No	59	0	0
0	0	Thoracotoı	215	CHU	No	69	0	1
0	0	Thoracotoı	215	CHU	No	55	0	0

0	0	Thoracotoı	215	CHU	No	66	0	0
0	0	VATS or rc	215	CHU	No	47	0	1
0	0	Thoracotoı	215	CHU	No	66	0	0
0	0	VATS or rc	215	CHU	No	53	0	0
0	0	VATS or rc	215	CHU	No	55	0	1
0	0	Thoracotoı	215	CHU	No	69	0	0
0	0	VATS or rc	215	CHU	No	65	0	0
0	0	Thoracotoı	215	CHU	No	67	0	0
0	0	VATS or rc	215	CHU	No	69	0	0
0	0	Thoracotoı	215	CHU	No	48	0	0
0	0	Thoracotoı	215	CHU	No	74	1	0
0	0	Thoracotoı	215	CHU	No	57	0	0
0	0	VATS or rc	215	CHU	No	56	0	0
0	0	VATS or rc	215	CHU	No	60	0	1
0	0	VATS or rc	215	CHU	No	69	0	0
0	0	Thoracotoı	215	CHU	No	54	1	0
0	0	VATS or rc	215	CHU	No	66	0	1
0	0	VATS or rc	215	CHU	No	69	1	1
0	0	Thoracotoı	215	CHU	No	53	0	0
0	0	VATS or rc	215	CHU	No	72	0	0
0	0	Thoracotoı	215	CHU	No	58	0	0
0	0	Thoracotoı	215	CHU	No	61	0	0
0	0	Thoracotoı	215	CHU	No	52	0	1
0	0	Thoracotoı	215	CHU	No	65	0	0
0	0	VATS or rc	215	CHU	No	66	0	0
0	1	Thoracotoı	215	CHU	No	45	0	0
0	0	VATS or rc	215	CHU	No	63	0	0
0	0	Thoracotoı	215	CHU	No	62	0	0
0	0	VATS or rc	215	CHU	No	72	0	0
0	0	Thoracotoı	215	CHU	No	58	0	0
0	0	VATS or rc	215	CHU	No	68	0	0
0	0	Thoracotoı	215	CHU	No	78	0	1
0	0	VATS or rc	215	CHU	No	77	0	0
0	0	VATS or rc	215	CHU	No	63	0	0
0	0	VATS or rc	215	CHU	No	59	0	1
0	0	VATS or rc	215	CHU	No	36	0	1
0	0	VATS or rc	215	CHU	No	60	0	0
0	0	Thoracotoı	215	CHU	No	63	0	0
0	0	Thoracotoı	215	CHU	No	58	0	1
0	0	VATS or rc	215	CHU	No	68	0	0
0	0	VATS or rc	215	CHU	No	76	0	0
0	0	Thoracotoı	215	CHU	No	73	0	1
0	0	Thoracotoı	215	CHU	No	73	0	1
0	0	VATS or rc	215	CHU	No	61	0	1
0	0	VATS or rc	215	CHU	No	69	0	0
0	0	VATS or rc	215	CHU	No	56	0	1
0	0	Thoracotoı	215	CHU	No	65	0	0
0	0	VATS or rc	215	CHU	No	71	0	0
0	0	Thoracotoı	215	CHU	No	61	0	0
0	0	Thoracotoı	215	CHU	No	65	0	1
0	0	VATS or rc	215	CHU	No	68	0	0
0	0	Thoracotoı	215	CHU	No	57	0	0
0	0	VATS or rc	215	CHU	No	81	0	0
0	0	VATS or rc	215	CHU	No	72	1	1
0	0	Thoracotoı	215	CHU	No	57	0	0
0	0	VATS or rc	215	CHU	No	64	0	0

0	0	VATS or rc	215	CHU	No	61	0	0
0	0	Thoracotor	215	CHU	No	70	0	0
0	0	VATS or rc	215	CHU	No	70	0	1
0	0	Thoracotor	215	CHU	No	70	0	0
0	0	Thoracotor	215	CHU	No	42	0	0
0	0	VATS or rc	215	CHU	No	69	0	0
0	0	Thoracotor	215	CHU	No	79	0	1
0	0	Thoracotor	215	CHU	No	77	0	0
0	0	VATS or rc	215	CHU	No	59	0	0
0	0	VATS or rc	215	CHU	No	64	0	0
0	0	Thoracotor	215	CHU	No	74	0	0
0	0	Thoracotor	215	CHU	No	63	0	0
0	0	Thoracotor	215	CHU	No	67	1	1
0	0	Thoracotor	215	CHU	No	57	0	0
0	1	Thoracotor	215	CHU	No	70	0	0
0	0	Thoracotor	215	CHU	No	67	0	0
0	0	VATS or rc	215	CHU	No	71	0	0
0	0	VATS or rc	215	CHU	No	66	0	1
0	0	VATS or rc	215	CHU	No	75	0	0
0	0	VATS or rc	215	CHU	No	60	0	1
0	1	Thoracotor	215	CHU	No	66	0	0
0	0	VATS or rc	215	CHU	No	62	0	1
0	0	VATS or rc	215	CHU	No	63	1	0
0	0	Thoracotor	215	CHU	No	51	0	1
0	0	VATS or rc	215	CHU	No	79	0	0
0	0	Thoracotor	215	CHU	No	61	0	1
0	0	VATS or rc	215	CHU	No	71	0	0
0	0	Thoracotor	215	CHU	No	70	0	0
0	0	VATS or rc	215	CHU	No	73	0	0
0	0	VATS or rc	215	CHU	No	71	0	0
0	0	Thoracotor	215	CHU	No	58	0	0
0	0	Thoracotor	215	CHU	No	67	0	0
0	0	VATS or rc	215	CHU	No	56	1	1
0	1	Thoracotor	215	CHU	No	73	0	0
0	0	VATS or rc	215	CHU	No	61	0	1
0	0	VATS or rc	215	CHU	No	70	0	0
0	0	Thoracotor	215	CHU	No	70	0	0
0	0	Thoracotor	215	CHU	No	65	0	0
0	0	VATS or rc	215	CHU	No	54	0	1
0	0	Thoracotor	215	CHU	No	68	0	1
0	0	Thoracotor	215	CHU	No	60	0	1
0	0	VATS or rc	215	CHU	No	63	0	0
0	0	VATS or rc	215	CHU	No	69	0	0
0	0	VATS or rc	215	CHU	No	69	0	0
0	0	Thoracotor	215	CHU	No	63	0	1
0	0	VATS or rc	215	CHU	No	76	0	0
0	0	VATS or rc	215	CHU	No	68	0	1
0	0	VATS or rc	215	CHU	No	70	0	0
0	0	Thoracotor	215	CHU	No	57	0	0
0	0	VATS or rc	215	CHU	No	70	0	0
0	0	VATS or rc	215	CHU	No	52	0	0
0	0	Thoracotor	215	CHU	No	65	0	0
0	0	VATS or rc	215	CHU	No	68	0	0
0	0	Thoracotor	215	CHU	No	68	0	0
0	0	Thoracotor	215	CHU	No	60	0	0
0	0	Thoracotor	215	CHU	No	74	0	1

0	0	VATS or rc	215	CHU	No	76	0	1
0	0	Thoracotoı	215	CHU	No	63	0	0
0	0	Thoracotoı	215	CHU	No	74	0	0
0	0	VATS or rc	215	CHU	No	54	0	1
0	0	Thoracotoı	215	CHU	No	79	0	0
0	0	Thoracotoı	215	CHU	No	66	1	0
0	0	VATS or rc	215	CHU	No	66	0	0
0	0	Thoracotoı	215	CHU	No	62	0	0
0	0	Thoracotoı	215	CHU	No	54	0	0
0	0	Thoracotoı	215	CHU	No	53	0	0
0	0	VATS or rc	215	CHU	No	80	0	1
0	0	Thoracotoı	215	CHU	No	59	1	0
0	0	Thoracotoı	215	CHU	No	50	0	1
0	0	VATS or rc	215	CHU	No	74	0	0
0	0	Thoracotoı	215	CHU	No	53	0	0
0	0	VATS or rc	215	CHU	No	64	0	0
0	0	VATS or rc	215	CHU	No	70	0	0
0	0	VATS or rc	215	CHU	No	62	0	1
0	0	Thoracotoı	215	CHU	No	59	0	0
0	1	VATS or rc	215	CHU	No	75	0	0
0	0	Thoracotoı	215	CHU	No	54	0	1
0	0	VATS or rc	215	CHU	No	57	0	0
0	0	Thoracotoı	215	CHU	No	70	0	1
0	0	Thoracotoı	215	CHU	No	72	1	1
0	0	Thoracotoı	215	CHU	No	60	0	0
0	0	Thoracotoı	215	CHU	No	51	0	1
0	0	VATS or rc	215	CHU	No	52	0	1
0	0	VATS or rc	215	CHU	No	73	0	1
0	0	VATS or rc	215	CHU	No	70	0	0
0	0	VATS or rc	215	CHU	No	40	0	1
0	0	Thoracotoı	215	CHU	No	51	0	1
0	0	VATS or rc	215	CHU	No	61	0	0
0	0	Thoracotoı	215	CHU	No	72	0	0
0	0	VATS or rc	215	CHU	No	65	0	0
0	0	Thoracotoı	215	CHU	No	85	0	0
0	0	Thoracotoı	215	CHU	No	57	0	0
0	0	VATS or rc	215	CHU	No	60	0	1
0	0	VATS or rc	215	CHU	No	70	0	0
0	0	Thoracotoı	215	CHU	No	62	0	0
0	1	Thoracotoı	215	CHU	No	71	0	1
0	0	Thoracotoı	215	CHU	No	54	0	1
0	0	Thoracotoı	215	CHU	No	65	0	0
0	0	VATS or rc	215	CHU	No	71	0	0
0	0	VATS or rc	215	CHU	No	74	0	1
0	0	VATS or rc	215	CHU	No	71	1	0
0	0	VATS or rc	215	CHU	No	46	0	0
0	0	Thoracotoı	215	CHU	No	67	0	0
0	0	Thoracotoı	215	CHU	No	58	0	1
0	0	VATS or rc	215	CHU	No	68	0	1
0	0	VATS or rc	215	CHU	No	73	0	0
0	0	Thoracotoı	215	CHU	No	70	0	0
0	0	Thoracotoı	215	CHU	No	73	1	1
0	0	VATS or rc	215	CHU	No	70	0	0
0	0	Thoracotoı	215	CHU	No	58	0	1
0	0	Thoracotoı	215	CHU	No	69	0	0
0	0	Thoracotoı	215	CHU	No	62	0	0

0	0	Thoracotoı	215	CHU	No	76	0	1
0	0	VATS or rc	215	CHU	No	67	0	1
0	0	VATS or rc	215	CHU	No	72	0	0
0	0	VATS or rc	215	CHU	No	62	1	1
0	0	VATS or rc	215	CHU	No	70	1	1
0	0	VATS or rc	215	CHU	No	41	0	0
0	0	Thoracotoı	215	CHU	No	64	0	0
0	0	Thoracotoı	215	CHU	No	65	0	1
0	0	VATS or rc	215	CHU	No	72	0	0
0	0	VATS or rc	215	CHU	No	67	0	0
0	0	Thoracotoı	215	CHU	No	50	0	0
0	0	VATS or rc	215	CHU	No	67	0	0
0	0	VATS or rc	215	CHU	No	67	0	0
0	0	Thoracotoı	215	CHU	No	70	0	1
0	0	VATS or rc	215	CHU	No	62	0	1
0	0	Thoracotoı	215	CHU	No	59	0	0
0	0	Thoracotoı	215	CHU	No	71	0	0
0	0	Thoracotoı	215	CHU	No	64	0	0
0	0	VATS or rc	215	CHU	No	76	0	0
0	0	Thoracotoı	215	CHU	No	54	0	1
0	0	Thoracotoı	215	CHU	No	55	0	1
0	0	Thoracotoı	215	CHU	No	59	0	1
0	0	VATS or rc	215	CHU	No	76	0	0
0	0	VATS or rc	215	CHU	No	72	1	0
0	0	VATS or rc	215	CHU	No	66	0	0
0	0	Thoracotoı	215	CHU	No	77	0	0
0	0	Thoracotoı	215	CHU	No	68	0	0
0	0	Thoracotoı	215	CHU	Yes	53	0	1
0	0	Thoracotoı	215	CHU	No	58	0	0
0	0	VATS or rc	215	CHU	No	74	0	1
0	0	VATS or rc	215	CHU	No	60	0	1
0	0	VATS or rc	215	CHU	No	64	0	0
0	0	Thoracotoı	215	CHU	No	48	0	1
0	0	Thoracotoı	215	CHU	No	57	0	1
0	0	VATS or rc	215	CHU	No	71	0	1
0	0	VATS or rc	215	CHU	No	60	0	0
0	0	VATS or rc	215	CHU	No	59	0	0
0	0	VATS or rc	215	CHU	No	73	0	0
0	0	VATS or rc	215	CHU	No	66	0	0
0	0	VATS or rc	215	CHU	No	64	0	1
0	0	VATS or rc	215	CHU	No	70	0	0
0	1	Thoracotoı	215	CHU	No	69	0	1
0	0	VATS or rc	215	CHU	No	60	0	1
0	0	Thoracotoı	215	CHU	No	35	0	0
0	0	Thoracotoı	215	CHU	No	72	0	0
0	0	Thoracotoı	215	CHU	No	64	1	0
0	0	VATS or rc	215	CHU	No	61	0	0
0	0	Thoracotoı	215	CHU	No	41	0	0
0	0	Thoracotoı	215	CHU	No	68	0	1
0	0	Thoracotoı	215	CHU	No	62	1	0
0	0	VATS or rc	215	CHU	No	57	0	1
0	0	Thoracotoı	215	CHU	No	66	0	0
0	0	Thoracotoı	215	CHU	No	64	1	0
0	0	Thoracotoı	215	CHU	No	59	0	0
0	0	VATS or rc	215	CHU	No	63	0	0
0	0	Thoracotoı	215	CHU	No	66	0	0

0	0	Thoracotoı	215	CHU	No	58	0	0
0	0	VATS or rc	215	CHU	No	56	1	1
0	0	Thoracotoı	215	CHU	No	62	0	1
0	0	Thoracotoı	215	CHU	No	79	0	0
0	0	Thoracotoı	215	CHU	No	82	0	0
0	0	VATS or rc	215	CHU	No	71	0	0
0	0	VATS or rc	215	CHU	No	72	0	0
0	0	Thoracotoı	215	CHU	No	62	0	0
0	0	VATS or rc	215	CHU	No	68	0	0
0	0	VATS or rc	215	CHU	No	60	0	0
0	0	Thoracotoı	215	CHU	No	45	0	0
0	0	VATS or rc	215	CHU	No	62	0	0
0	0	VATS or rc	215	CHU	No	71	0	1
0	0	Thoracotoı	215	CHU	No	62	0	0
0	0	VATS or rc	215	CHU	No	77	0	0
0	0	Thoracotoı	215	CHU	No	57	0	0
0	0	Thoracotoı	215	CHU	No	63	0	0
0	0	VATS or rc	215	CHU	No	53	0	0
0	0	Thoracotoı	215	CHU	No	52	0	0
0	0	VATS or rc	215	CHU	No	66	1	1
0	0	VATS or rc	215	CHU	No	65	0	1
0	0	Thoracotoı	215	CHU	No	67	1	0
0	0	Thoracotoı	215	CHU	No	54	0	1
0	1	Thoracotoı	215	CHU	No	83	0	0
0	0	Thoracotoı	215	CHU	No	59	1	0
0	0	VATS or rc	215	CHU	No	69	0	1
0	0	VATS or rc	215	CHU	No	56	0	0
0	0	Thoracotoı	215	CHU	No	71	1	0
0	0	Thoracotoı	215	CHU	No	73	0	0
0	0	VATS or rc	215	CHU	No	65	1	1
0	0	Thoracotoı	215	CHU	No	70	0	0
0	0	Thoracotoı	215	CHU	No	64	0	0
0	0	Thoracotoı	215	CHU	No	59	0	0
0	0	VATS or rc	215	CHU	No	71	0	0
0	0	VATS or rc	215	CHU	No	78	0	0
0	0	Thoracotoı	215	CHU	No	62	0	0
0	0	Thoracotoı	215	CHU	No	42	0	1
0	0	VATS or rc	215	CHU	No	80	0	0
0	0	VATS or rc	215	CHU	No	74	0	0
0	0	VATS or rc	215	CHU	No	56	0	0
0	0	Thoracotoı	215	CHU	No	71	0	1
0	0	VATS or rc	215	CHU	No	61	0	0
0	0	Thoracotoı	215	CHU	No	68	0	0
0	0	VATS or rc	215	CHU	No	82	0	0
0	0	VATS or rc	215	CHU	No	81	0	0
0	0	Thoracotoı	215	CHU	No	66	0	0
0	0	VATS or rc	215	CHU	No	59	0	0
0	0	VATS or rc	215	CHU	No	60	0	1
0	0	VATS or rc	215	CHU	No	64	0	0
0	0	Thoracotoı	215	CHU	No	64	0	1
0	0	Thoracotoı	215	CHU	No	60	0	0
0	0	Thoracotoı	215	CHU	No	50	0	1
0	0	VATS or rc	215	CHU	No	55	1	1
0	0	Thoracotoı	215	CHU	No	45	0	1
0	0	Thoracotoı	215	CHU	No	50	0	0
0	0	Thoracotoı	215	CHU	No	74	0	0

0	0	Thoracotoı	215	CHU	No	45	0	0
0	0	Thoracotoı	215	CHU	No	66	0	0
0	0	VATS or rc	215	CHU	No	63	0	0
0	0	VATS or rc	215	CHU	No	55	1	0
0	0	VATS or rc	215	CHU	No	73	0	0
0	0	VATS or rc	215	CHU	No	77	1	0
0	0	VATS or rc	215	CHU	No	86	0	0
0	0	VATS or rc	215	CHU	No	72	0	1
0	0	VATS or rc	215	CHU	No	71	0	0
0	0	VATS or rc	215	CHU	No	62	0	1
0	0	VATS or rc	215	CHU	No	73	0	1
0	0	Thoracotoı	215	CHU	Yes	59	0	1
0	0	Thoracotoı	215	CHU	No	67	0	0
0	0	VATS or rc	215	CHU	No	60	0	0
0	0	VATS or rc	215	CHU	No	79	0	0
0	0	Thoracotoı	215	CHU	No	75	0	0
0	0	VATS or rc	215	CHU	No	60	0	0
0	0	Thoracotoı	215	CHU	No	48	0	0
0	0	VATS or rc	215	CHU	No	59	0	1
0	0	VATS or rc	215	CHU	No	63	0	0
0	0	Thoracotoı	215	CHU	No	29	0	1
0	0	VATS or rc	215	CHU	No	75	0	0
0	0	VATS or rc	215	CHU	No	79	0	1
0	0	VATS or rc	215	CHU	No	72	0	1
0	0	Thoracotoı	215	CHU	No	62	0	0
0	0	Thoracotoı	215	CHU	No	57	0	0
0	0	VATS or rc	215	CHU	No	75	0	0
0	0	VATS or rc	215	CHU	No	68	0	0
0	0	VATS or rc	215	CHU	No	53	1	0
0	0	Thoracotoı	215	CHU	No	73	0	0
0	0	Thoracotoı	215	CHU	No	62	0	1
0	0	Thoracotoı	215	CHU	No	61	0	0
0	0	VATS or rc	215	CHU	No	47	0	0
0	0	VATS or rc	215	CHU	No	70	0	0
0	0	Thoracotoı	215	CHU	No	71	0	0
0	0	Thoracotoı	215	CHU	No	71	0	1
0	0	VATS or rc	215	CHU	No	73	0	1
0	0	VATS or rc	215	CHU	No	63	0	1
0	0	Thoracotoı	215	CHU	No	71	0	0
0	0	VATS or rc	215	CHU	No	70	0	0
0	0	VATS or rc	215	CHU	No	60	0	0
0	0	VATS or rc	215	CHU	No	70	0	1
0	0	VATS or rc	215	CHU	No	60	0	1
0	0	VATS or rc	215	CHU	No	62	0	0
0	0	VATS or rc	215	CHU	No	78	0	0
0	0	Thoracotoı	215	CHU	No	61	0	1
0	0	Thoracotoı	215	CHU	No	66	0	0
0	0	VATS or rc	215	CHU	No	61	1	1
0	0	VATS or rc	215	CHU	No	66	0	1
0	0	VATS or rc	215	CHU	No	76	0	0
0	0	Thoracotoı	215	CHU	No	65	0	0
0	1	Thoracotoı	215	CHU	No	66	0	1
0	0	Thoracotoı	215	CHU	No	58	0	0
0	0	VATS or rc	215	CHU	No	69	0	0
0	1	Thoracotoı	215	CHU	No	70	0	0
0	0	VATS or rc	55	PRIVE	No	66	0	0

0	0	VATS or rc	55	PRIVE	No	75	1	0
0	0	VATS or rc	55	PRIVE	No	56	0	0
0	1	Thoracotoı	55	PRIVE	No	71	0	0
0	0	VATS or rc	55	PRIVE	No	64	0	0
0	0	Thoracotoı	55	PRIVE	No	69	0	0
0	0	Thoracotoı	55	PRIVE	No	63	0	0
0	0	Thoracotoı	55	PRIVE	No	54	0	0
0	0	VATS or rc	55	PRIVE	No	76	0	0
0	0	VATS or rc	55	PRIVE	No	66	0	0
0	0	VATS or rc	55	PRIVE	No	51	0	0
0	0	Thoracotoı	55	PRIVE	No	76	1	0
0	0	VATS or rc	55	PRIVE	No	67	0	0
0	0	Thoracotoı	55	PRIVE	No	76	0	0
0	0	Thoracotoı	55	PRIVE	No	70	1	0
0	0	VATS or rc	55	PRIVE	No	53	0	0
0	0	VATS or rc	55	PRIVE	No	54	0	0
0	0	Thoracotoı	55	PRIVE	No	76	0	0
0	0	Thoracotoı	55	PRIVE	No	77	0	0
0	0	VATS or rc	55	PRIVE	No	58	0	0
0	0	Thoracotoı	55	PRIVE	No	70	0	0
0	0	Thoracotoı	55	PRIVE	No	54	0	0
0	0	VATS or rc	55	PRIVE	No	51	0	0
0	0	VATS or rc	55	PRIVE	No	77	0	0
0	0	Thoracotoı	55	PRIVE	No	65	0	0
0	0	Thoracotoı	55	PRIVE	No	74	0	0
0	0	VATS or rc	55	PRIVE	No	53	0	0
0	0	VATS or rc	55	PRIVE	No	67	0	0
0	0	Thoracotoı	55	PRIVE	No	59	0	0
0	0	VATS or rc	55	PRIVE	No	73	0	0
0	0	Thoracotoı	55	PRIVE	No	69	0	0
0	0	VATS or rc	55	PRIVE	No	60	0	0
0	0	VATS or rc	55	PRIVE	No	81	0	0
0	0	VATS or rc	55	PRIVE	No	70	0	0
0	0	VATS or rc	55	PRIVE	No	49	0	0
0	0	VATS or rc	55	PRIVE	No	61	0	0
0	0	VATS or rc	55	PRIVE	No	74	1	0
0	0	VATS or rc	55	PRIVE	No	46	0	0
0	0	VATS or rc	55	PRIVE	No	80	1	0
0	1	Thoracotoı	55	PRIVE	No	66	0	0
0	0	Thoracotoı	55	PRIVE	No	69	0	0
0	0	VATS or rc	55	PRIVE	No	58	0	0
0	0	VATS or rc	55	PRIVE	No	63	0	0
0	0	VATS or rc	55	PRIVE	No	62	0	0
0	0	VATS or rc	55	PRIVE	No	69	1	0
0	0	Thoracotoı	55	PRIVE	No	66	0	0
0	0	Thoracotoı	55	PRIVE	No	73	0	0
0	0	Thoracotoı	55	PRIVE	No	67	0	0
0	0	Thoracotoı	55	PRIVE	No	61	0	0
0	0	VATS or rc	55	PRIVE	No	61	1	0
0	0	VATS or rc	55	PRIVE	No	65	0	0
0	0	VATS or rc	55	PRIVE	No	64	0	0
0	0	VATS or rc	55	PRIVE	No	48	0	0
0	0	VATS or rc	55	PRIVE	No	79	0	0
0	0	VATS or rc	55	PRIVE	No	58	0	0
0	0	Thoracotoı	55	PRIVE	No	69	0	0
0	1	Thoracotoı	55	PRIVE	No	56	0	0

0	0	Thoracotoı	55	PRIVE	No	76	0	0
0	0	Thoracotoı	55	PRIVE	No	72	1	0
0	0	Thoracotoı	55	PRIVE	No	82	0	0
0	0	VATS or rc	55	PRIVE	No	75	0	0
0	0	VATS or rc	55	PRIVE	No	69	0	0
0	0	VATS or rc	55	PRIVE	No	56	0	0
0	0	VATS or rc	55	PRIVE	No	70	0	0
0	0	Thoracotoı	55	PRIVE	No	65	0	0
0	1	Thoracotoı	55	PRIVE	No	71	0	0
0	0	VATS or rc	55	PRIVE	No	73	0	0
0	0	VATS or rc	55	PRIVE	No	60	0	0
0	0	Thoracotoı	55	PRIVE	No	62	0	0
0	0	Thoracotoı	55	PRIVE	No	58	0	0
0	0	VATS or rc	55	PRIVE	No	68	0	0
0	0	VATS or rc	55	PRIVE	No	54	0	0
0	0	Thoracotoı	55	PRIVE	No	69	0	0
0	1	Thoracotoı	55	PRIVE	No	57	0	0
0	0	VATS or rc	55	PRIVE	No	61	0	0
0	1	Thoracotoı	55	PRIVE	No	71	0	0
0	0	VATS or rc	55	PRIVE	No	55	0	0
0	0	Thoracotoı	55	PRIVE	No	64	0	0
0	1	Thoracotoı	55	PRIVE	No	71	0	0
0	0	Thoracotoı	55	PRIVE	No	77	0	0
0	0	VATS or rc	55	PRIVE	No	68	0	0
0	0	Thoracotoı	55	PRIVE	No	73	0	0
0	0	VATS or rc	55	PRIVE	No	63	0	0
0	0	VATS or rc	55	PRIVE	No	61	0	0
0	0	VATS or rc	55	PRIVE	No	76	0	0
0	0	VATS or rc	55	PRIVE	No	72	0	0
0	0	Thoracotoı	55	PRIVE	No	57	0	0
0	0	VATS or rc	55	PRIVE	No	71	0	0
0	0	Thoracotoı	55	PRIVE	No	60	0	0
0	0	VATS or rc	55	PRIVE	No	52	0	0
0	0	Thoracotoı	55	PRIVE	No	71	0	0
0	0	Thoracotoı	55	PRIVE	No	76	0	0
0	0	VATS or rc	55	PRIVE	No	55	0	0
0	0	Thoracotoı	55	PRIVE	No	51	0	0
0	0	VATS or rc	55	PRIVE	No	53	0	0
0	0	VATS or rc	55	PRIVE	No	71	0	0
0	0	Thoracotoı	55	PRIVE	No	66	0	0
0	0	VATS or rc	55	PRIVE	No	72	0	0
0	0	VATS or rc	55	PRIVE	No	58	1	0
0	0	VATS or rc	55	PRIVE	No	75	0	0
0	0	VATS or rc	55	PRIVE	No	63	0	0
0	0	Thoracotoı	55	PRIVE	No	58	0	0
0	0	VATS or rc	55	PRIVE	No	73	0	0
0	0	VATS or rc	55	PRIVE	No	72	0	0
0	0	Thoracotoı	55	PRIVE	No	75	1	0
0	1	Thoracotoı	55	PRIVE	No	60	0	0
0	0	Thoracotoı	55	PRIVE	No	55	0	0
0	0	VATS or rc	55	PRIVE	No	59	0	0
0	0	VATS or rc	55	PRIVE	No	64	0	0
0	0	VATS or rc	55	PRIVE	No	66	0	0
0	1	Thoracotoı	55	PRIVE	No	61	0	0
0	0	VATS or rc	55	PRIVE	No	76	0	0
0	0	Thoracotoı	55	PRIVE	No	88	0	0

0	0	Thoracotoı	55	PRIVE	No	72	0	0
0	0	Thoracotoı	55	PRIVE	No	45	0	0
0	1	Thoracotoı	55	PRIVE	No	62	0	0
0	0	Thoracotoı	55	PRIVE	No	69	0	0
0	0	Thoracotoı	55	PRIVE	No	56	0	0
0	0	VATS or rc	55	PRIVE	No	66	0	0
0	0	Thoracotoı	55	PRIVE	No	76	0	0
0	0	VATS or rc	55	PRIVE	No	39	0	0
0	0	Thoracotoı	55	PRIVE	No	44	0	0
0	0	Thoracotoı	55	PRIVE	No	71	0	0
0	0	Thoracotoı	55	PRIVE	No	55	0	0
0	0	VATS or rc	55	PRIVE	No	77	0	0
0	0	VATS or rc	55	PRIVE	No	74	0	0
0	0	VATS or rc	55	PRIVE	No	64	0	0
0	0	VATS or rc	55	PRIVE	No	44	0	0
0	0	VATS or rc	55	PRIVE	No	48	0	0
0	0	Thoracotoı	55	PRIVE	No	66	0	0
0	0	VATS or rc	55	PRIVE	No	71	0	0
0	0	Thoracotoı	55	PRIVE	No	56	0	0
0	0	Thoracotoı	55	PRIVE	No	57	0	0
0	1	Thoracotoı	55	PRIVE	No	63	0	0
0	0	VATS or rc	55	PRIVE	No	63	0	0
0	0	Thoracotoı	55	PRIVE	No	66	0	0
0	0	VATS or rc	55	PRIVE	No	64	0	1
0	0	Thoracotoı	55	PRIVE	No	53	0	0
0	0	Thoracotoı	55	PRIVE	No	60	0	0
0	0	VATS or rc	55	PRIVE	No	56	0	0
0	0	Thoracotoı	55	PRIVE	No	52	0	0
0	0	VATS or rc	55	PRIVE	No	58	0	0
0	0	Thoracotoı	55	PRIVE	No	74	0	0
0	0	Thoracotoı	55	PRIVE	No	73	0	0
0	0	Thoracotoı	55	PRIVE	No	68	0	0
0	0	VATS or rc	55	PRIVE	No	67	0	0
0	0	VATS or rc	55	PRIVE	No	69	0	0
0	0	VATS or rc	55	PRIVE	No	58	0	0
0	0	VATS or rc	55	PRIVE	No	73	0	0
0	0	VATS or rc	55	PRIVE	No	72	0	0
0	0	Thoracotoı	55	PRIVE	No	68	0	0
0	0	Thoracotoı	55	PRIVE	No	67	0	0
0	0	VATS or rc	55	PRIVE	No	70	0	0
0	0	VATS or rc	55	PRIVE	No	79	0	0
0	1	Thoracotoı	55	PRIVE	No	52	0	0
0	0	VATS or rc	55	PRIVE	No	71	0	0
0	0	VATS or rc	55	PRIVE	No	66	0	0
0	0	VATS or rc	55	PRIVE	No	82	0	0
0	0	Thoracotoı	55	PRIVE	No	79	1	0
0	0	Thoracotoı	2	CHU	No	57	0	0
0	1	Thoracotoı	2	CHU	No	71	0	0
1	0	Thoracotoı	110	CHU	No	76	0	0
1	1	Thoracotoı	110	CHU	No	68	0	1
1	0	Thoracotoı	110	CHU	No	62	0	0
1	0	Thoracotoı	110	CHU	No	69	0	0
1	0	Thoracotoı	110	CHU	No	59	0	0
1	0	Thoracotoı	110	CHU	No	64	0	0
1	0	Thoracotoı	110	CHU	No	49	0	0
1	0	Thoracotoı	110	CHU	No	71	0	0

1	0	Thoracotoı	110	CHU	No	66	0	1
1	0	Thoracotoı	110	CHU	No	72	1	0
1	0	Thoracotoı	110	CHU	Yes	53	0	1
1	1	Thoracotoı	110	CHU	No	43	0	0
1	0	Thoracotoı	110	CHU	No	63	0	1
1	0	Thoracotoı	110	CHU	No	73	0	0
1	0	Thoracotoı	110	CHU	No	67	0	1
1	0	Thoracotoı	110	CHU	No	61	0	0
1	0	Thoracotoı	110	CHU	No	54	0	1
1	0	Thoracotoı	110	CHU	No	78	0	0
1	1	Thoracotoı	110	CHU	No	67	0	0
1	0	Thoracotoı	110	CHU	No	48	0	0
1	0	Thoracotoı	110	CHU	No	54	0	0
1	0	Thoracotoı	110	CHU	No	53	0	1
1	0	Thoracotoı	110	CHU	No	63	0	1
1	0	Thoracotoı	110	CHU	No	52	0	0
1	0	Thoracotoı	110	CHU	No	70	1	1
1	0	Thoracotoı	110	CHU	No	75	0	0
1	0	Thoracotoı	110	CHU	No	55	0	0
1	0	Thoracotoı	110	CHU	No	62	1	0
1	0	Thoracotoı	110	CHU	No	55	0	0
1	0	Thoracotoı	110	CHU	No	83	0	0
1	0	Thoracotoı	110	CHU	No	73	1	0
1	1	Thoracotoı	110	CHU	No	66	0	0
1	0	Thoracotoı	110	CHU	No	59	0	1
1	0	Thoracotoı	110	CHU	No	69	0	0
1	0	Thoracotoı	110	CHU	No	82	1	0
1	1	Thoracotoı	110	CHU	No	52	0	0
1	0	Thoracotoı	110	CHU	No	73	0	0
1	0	Thoracotoı	110	CHU	No	68	1	0
1	0	Thoracotoı	110	CHU	No	70	0	0
1	0	Thoracotoı	110	CHU	No	62	1	0
1	0	Thoracotoı	110	CHU	No	69	0	0
1	0	Thoracotoı	110	CHU	No	60	0	0
1	0	Thoracotoı	110	CHU	No	69	0	0
1	0	VATS or rc	110	CHU	No	71	0	0
1	0	Thoracotoı	110	CHU	No	65	0	0
1	0	Thoracotoı	110	CHU	No	54	0	0
1	0	Thoracotoı	110	CHU	No	54	0	1
1	0	Thoracotoı	110	CHU	No	56	0	0
1	0	Thoracotoı	110	CHU	No	86	0	0
1	0	Thoracotoı	110	CHU	No	57	0	0
1	0	Thoracotoı	110	CHU	No	58	0	1
1	0	Thoracotoı	110	CHU	No	72	0	0
1	0	Thoracotoı	110	CHU	No	64	1	0
1	0	Thoracotoı	110	CHU	No	78	0	0
1	1	Thoracotoı	110	CHU	Yes	69	0	0
1	0	Thoracotoı	110	CHU	No	74	0	0
1	0	Thoracotoı	110	CHU	No	64	0	1
1	0	Thoracotoı	110	CHU	No	85	0	0
1	0	Thoracotoı	110	CHU	No	75	0	1
1	0	Thoracotoı	110	CHU	No	73	1	0
1	0	Thoracotoı	110	CHU	No	75	0	0
1	0	Thoracotoı	110	CHU	No	77	0	0
1	0	Thoracotoı	110	CHU	No	72	0	0
1	0	Thoracotoı	110	CHU	No	60	0	1

1	0	Thoracotoı	110	CHU	No	57	0	0
1	0	Thoracotoı	110	CHU	No	58	0	0
1	0	Thoracotoı	110	CHU	No	88	0	0
1	0	Thoracotoı	110	CHU	No	75	1	0
1	0	Thoracotoı	110	CHU	No	60	0	0
1	1	Thoracotoı	110	CHU	No	47	0	1
1	0	Thoracotoı	110	CHU	No	62	0	1
1	0	Thoracotoı	110	CHU	No	72	0	1
1	0	Thoracotoı	110	CHU	No	66	0	0
1	0	Thoracotoı	110	CHU	No	66	1	0
1	0	Thoracotoı	110	CHU	No	79	0	0
1	0	Thoracotoı	110	CHU	No	70	0	0
1	0	Thoracotoı	110	CHU	No	77	0	0
1	0	Thoracotoı	110	CHU	No	51	0	1
1	0	Thoracotoı	110	CHU	No	78	0	1
1	0	Thoracotoı	110	CHU	No	64	0	0
1	1	Thoracotoı	110	CHU	No	56	0	0
1	0	Thoracotoı	110	CHU	No	58	0	1
1	0	Thoracotoı	110	CHU	No	54	0	1
1	0	Thoracotoı	110	CHU	No	79	0	0
1	0	Thoracotoı	110	CHU	No	58	0	0
1	0	Thoracotoı	110	CHU	No	57	1	0
1	0	Thoracotoı	110	CHU	No	65	0	0
1	0	Thoracotoı	110	CHU	No	41	0	0
1	0	VATS or rc	110	CHU	No	59	0	0
1	0	Thoracotoı	110	CHU	No	62	0	0
1	0	Thoracotoı	110	CHU	No	66	0	0
1	1	Thoracotoı	110	CHU	No	54	0	0
1	0	Thoracotoı	110	CHU	No	73	0	0
1	0	VATS or rc	110	CHU	No	81	0	0
1	0	Thoracotoı	110	CHU	No	68	0	0
1	0	Thoracotoı	110	CHU	No	73	0	0
1	0	Thoracotoı	110	CHU	No	74	1	0
1	0	Thoracotoı	110	CHU	No	71	0	0
1	0	Thoracotoı	110	CHU	Yes	62	0	0
1	0	Thoracotoı	110	CHU	No	77	0	0
1	0	VATS or rc	110	CHU	No	56	0	1
1	0	Thoracotoı	110	CHU	No	77	1	1
1	1	Thoracotoı	110	CHU	No	68	1	0
1	0	Thoracotoı	110	CHU	No	78	1	0
1	0	Thoracotoı	110	CHU	No	71	0	0
1	0	Thoracotoı	110	CHU	No	58	0	0
1	0	Thoracotoı	110	CHU	Yes	88	0	0
1	0	Thoracotoı	110	CHU	No	49	0	0
1	0	Thoracotoı	110	CHU	No	68	0	0
1	0	Thoracotoı	110	CHU	No	71	0	0
1	0	Thoracotoı	110	CHU	No	56	0	0
1	0	Thoracotoı	110	CHU	No	83	1	0
1	0	Thoracotoı	110	CHU	No	70	0	0
1	0	Thoracotoı	110	CHU	No	65	0	0
1	0	Thoracotoı	110	CHU	No	66	0	0
1	0	Thoracotoı	110	CHU	No	56	0	1
1	0	Thoracotoı	110	CHU	No	65	0	1
1	0	Thoracotoı	110	CHU	No	59	0	0
1	0	Thoracotoı	110	CHU	No	82	0	0
1	0	VATS or rc	110	CHU	No	57	0	0

1	0	Thoracotoı	110	CHU	Yes	66	0	0
1	0	Thoracotoı	110	CHU	No	78	1	1
1	0	Thoracotoı	110	CHU	No	69	1	0
1	0	VATS or rc	110	CHU	No	65	0	0
1	1	Thoracotoı	110	CHU	No	62	1	0
1	0	Thoracotoı	110	CHU	No	64	0	0
1	0	Thoracotoı	110	CHU	No	58	1	1
1	1	Thoracotoı	110	CHU	No	78	0	0
1	0	Thoracotoı	110	CHU	No	74	0	0
1	0	VATS or rc	110	CHU	No	63	0	0
1	0	Thoracotoı	110	CHU	No	57	0	0
1	1	Thoracotoı	110	CHU	No	72	0	1
1	0	Thoracotoı	110	CHU	No	66	0	0
1	1	Thoracotoı	110	CHU	No	65	0	0
1	0	Thoracotoı	110	CHU	No	57	0	1
1	0	Thoracotoı	110	CHU	No	55	0	0
1	0	Thoracotoı	110	CHU	No	59	0	0
1	0	Thoracotoı	110	CHU	No	75	0	0
1	0	Thoracotoı	110	CHU	No	53	0	1
1	0	Thoracotoı	110	CHU	No	74	1	0
1	0	Thoracotoı	110	CHU	No	61	0	0
1	0	Thoracotoı	110	CHU	No	57	0	0
1	0	Thoracotoı	110	CHU	No	62	0	0
1	0	Thoracotoı	110	CHU	No	55	0	0
1	0	Thoracotoı	110	CHU	No	67	0	0
1	0	Thoracotoı	110	CHU	No	61	0	0
1	0	Thoracotoı	110	CHU	No	57	0	0
1	1	Thoracotoı	110	CHU	No	70	0	0
1	0	Thoracotoı	110	CHU	No	68	0	1
1	0	Thoracotoı	110	CHU	No	57	0	0
1	0	Thoracotoı	110	CHU	No	76	1	1
1	0	Thoracotoı	110	CHU	No	63	0	0
1	0	Thoracotoı	110	CHU	No	77	0	0
1	0	Thoracotoı	110	CHU	No	70	0	0
1	0	Thoracotoı	110	CHU	No	60	0	0
1	0	Thoracotoı	110	CHU	No	43	0	0
1	0	Thoracotoı	110	CHU	No	75	0	0
1	0	Thoracotoı	110	CHU	No	53	0	0
1	0	Thoracotoı	110	CHU	Yes	63	1	1
1	0	Thoracotoı	110	CHU	No	78	1	1
1	0	VATS or rc	110	CHU	No	66	0	0
1	0	Thoracotoı	110	CHU	No	63	0	0
1	0	Thoracotoı	110	CHU	No	72	1	0
1	0	Thoracotoı	110	CHU	No	73	0	0
1	0	Thoracotoı	110	CHU	No	69	0	0
1	0	Thoracotoı	110	CHU	No	74	0	0
1	0	Thoracotoı	110	CHU	No	78	0	0
1	0	Thoracotoı	110	CHU	No	65	0	0
1	0	Thoracotoı	110	CHU	No	62	0	1
1	0	Thoracotoı	110	CHU	No	76	1	0
1	0	Thoracotoı	110	CHU	No	70	0	0
1	0	Thoracotoı	110	CHU	No	55	1	1
1	0	Thoracotoı	110	CHU	No	62	1	0
1	0	Thoracotoı	110	CHU	No	55	0	0
1	0	Thoracotoı	110	CHU	No	54	0	1
1	0	Thoracotoı	110	CHU	No	59	1	1

1	0	Thoracotoı	110	CHU	No	59	1	1
1	0	Thoracotoı	110	CHU	No	82	0	0
1	0	Thoracotoı	110	CHU	No	68	0	0
1	0	Thoracotoı	110	CHU	No	82	0	0
1	0	Thoracotoı	110	CHU	No	73	0	0
1	0	Thoracotoı	110	CHU	No	50	1	1
1	0	Thoracotoı	110	CHU	No	80	0	0
1	0	Thoracotoı	110	CHU	No	71	0	0
1	0	Thoracotoı	110	CHU	No	80	1	0
1	0	Thoracotoı	110	CHU	No	55	0	1
1	0	Thoracotoı	110	CHU	No	58	0	0
1	0	Thoracotoı	110	CHU	No	57	0	0
1	0	VATS or rc	110	CHU	No	73	0	0
1	0	Thoracotoı	110	CHU	No	64	0	0
1	0	Thoracotoı	110	CHU	No	64	0	0
1	0	Thoracotoı	110	CHU	No	60	0	0
1	0	Thoracotoı	110	CHU	No	70	0	0
1	0	Thoracotoı	110	CHU	No	63	0	0
1	0	Thoracotoı	110	CHU	No	68	0	0
1	0	Thoracotoı	110	CHU	No	75	0	0
1	0	Thoracotoı	110	CHU	No	57	0	0
1	0	Thoracotoı	110	CHU	No	81	0	0
1	0	Thoracotoı	110	CHU	No	68	1	0
1	0	VATS or rc	110	CHU	No	63	0	0
1	1	Thoracotoı	110	CHU	No	68	0	0
1	0	Thoracotoı	110	CHU	Yes	62	0	0
1	0	Thoracotoı	110	CHU	No	59	0	1
1	0	Thoracotoı	110	CHU	No	66	0	0
1	0	Thoracotoı	110	CHU	No	51	0	0
1	0	Thoracotoı	110	CHU	No	44	0	1
1	0	Thoracotoı	110	CHU	No	50	0	0
1	0	Thoracotoı	110	CHU	No	64	1	0
1	0	Thoracotoı	110	CHU	No	62	0	0
1	0	Thoracotoı	110	CHU	No	79	0	0
1	0	Thoracotoı	110	CHU	No	57	1	1
1	1	Thoracotoı	110	CHU	No	79	0	0
1	0	Thoracotoı	110	CHU	No	60	1	1
1	0	Thoracotoı	110	CHU	No	78	1	0
1	0	Thoracotoı	110	CHU	No	87	0	0
1	0	Thoracotoı	110	CHU	No	54	0	0
1	0	Thoracotoı	110	CHU	No	58	0	0
1	0	Thoracotoı	110	CHU	No	78	0	1
1	1	Thoracotoı	110	CHU	No	68	0	0
1	0	Thoracotoı	110	CHU	No	63	0	0
1	0	Thoracotoı	110	CHU	No	64	1	0
1	0	Thoracotoı	110	CHU	No	67	0	0
1	0	Thoracotoı	110	CHU	No	66	0	0
1	0	Thoracotoı	110	CHU	No	51	0	0
1	1	Thoracotoı	110	CHU	No	48	0	1
1	0	Thoracotoı	110	CHU	No	65	0	0
1	0	Thoracotoı	110	CHU	No	58	0	0
1	0	Thoracotoı	110	CHU	No	63	1	1
1	0	Thoracotoı	110	CHU	No	56	0	0
1	0	Thoracotoı	110	CHU	No	63	0	0
1	0	Thoracotoı	110	CHU	No	76	0	0
1	1	Thoracotoı	110	CHU	No	56	0	0

1	0	Thoracotor	110	CHU	No	64	0	0
1	0	Thoracotor	110	CHU	No	54	0	0
1	1	Thoracotor	110	CHU	Yes	77	1	0
1	0	Thoracotor	110	CHU	No	66	0	0
1	1	Thoracotor	110	CHU	No	60	0	0
1	0	Thoracotor	110	CHU	No	62	0	1
1	0	VATS or rc	110	CHU	No	44	0	0
1	0	Thoracotor	110	CHU	No	62	0	0
1	0	Thoracotor	110	CHU	No	68	0	1
1	0	Thoracotor	110	CHU	No	67	0	0
1	0	Thoracotor	110	CHU	No	53	0	0
1	0	Thoracotor	110	CHU	No	60	0	0
1	0	Thoracotor	110	CHU	No	72	0	0
1	0	Thoracotor	110	CHU	No	72	1	0
1	0	Thoracotor	110	CHU	No	50	0	0
1	0	Thoracotor	110	CHU	No	72	0	0
1	0	Thoracotor	110	CHU	No	61	0	0
1	0	Thoracotor	110	CHU	No	50	0	1
1	0	Thoracotor	110	CHU	No	55	0	0
1	0	Thoracotor	110	CHU	No	74	0	0
1	0	Thoracotor	110	CHU	No	70	0	0
1	0	Thoracotor	110	CHU	No	66	0	1
1	0	Thoracotor	110	CHU	No	70	0	0
1	0	Thoracotor	110	CHU	No	63	0	0
1	0	Thoracotor	110	CHU	No	53	0	1
1	0	Thoracotor	110	CHU	No	60	0	0
1	0	Thoracotor	110	CHU	No	64	0	0
1	0	VATS or rc	110	CHU	No	76	1	1
1	0	Thoracotor	110	CHU	No	69	0	0
1	1	Thoracotor	110	CHU	No	69	0	0
1	0	Thoracotor	110	CHU	No	54	0	0
1	0	Thoracotor	110	CHU	No	86	0	0
1	0	Thoracotor	110	CHU	No	59	0	0
1	0	Thoracotor	110	CHU	No	43	0	0
1	0	Thoracotor	110	CHU	No	58	0	0
1	0	VATS or rc	110	CHU	No	75	0	0
1	0	Thoracotor	110	CHU	No	69	0	0
1	0	Thoracotor	110	CHU	No	61	0	0
1	0	VATS or rc	110	CHU	No	69	0	0
1	0	Thoracotor	110	CHU	No	64	0	0
1	0	Thoracotor	110	CHU	No	71	0	0
1	0	Thoracotor	110	CHU	No	69	0	0
1	0	Thoracotor	110	CHU	No	66	0	0
1	0	Thoracotor	110	CHU	No	56	0	1
1	0	Thoracotor	110	CHU	No	56	0	0
1	0	Thoracotor	110	CHU	No	51	0	0
1	0	Thoracotor	110	CHU	No	62	0	0
1	0	Thoracotor	110	CHU	No	71	0	0
1	0	Thoracotor	110	CHU	No	67	0	0
1	0	Thoracotor	110	CHU	No	57	0	0
1	0	Thoracotor	110	CHU	No	68	0	0
1	0	Thoracotor	110	CHU	No	62	0	1
1	0	Thoracotor	110	CHU	No	81	0	0
1	0	Thoracotor	110	CHU	No	67	1	0
1	0	Thoracotor	110	CHU	No	84	0	0
1	0	Thoracotor	110	CHU	No	64	0	0

1	0	Thoracotoı	110	CHU	No	67	0	0
1	0	Thoracotoı	110	CHU	No	78	1	0
1	0	Thoracotoı	110	CHU	No	62	0	0
1	0	Thoracotoı	110	CHU	No	59	1	0
1	0	VATS or rc	110	CHU	No	56	0	1
1	0	Thoracotoı	110	CHU	No	58	0	0
1	0	Thoracotoı	110	CHU	No	54	0	0
1	0	Thoracotoı	110	CHU	No	53	0	0
1	0	Thoracotoı	110	CHU	No	60	0	0
1	1	Thoracotoı	110	CHU	No	70	0	0
1	0	Thoracotoı	110	CHU	No	67	0	1
1	0	Thoracotoı	110	CHU	No	72	0	0
1	0	Thoracotoı	110	CHU	No	77	0	0
1	0	Thoracotoı	110	CHU	No	51	0	0
1	0	Thoracotoı	110	CHU	No	70	0	0
1	0	Thoracotoı	110	CHU	No	60	0	0
1	0	Thoracotoı	110	CHU	No	70	0	1
1	0	Thoracotoı	110	CHU	No	53	0	0
1	0	Thoracotoı	110	CHU	No	48	0	0
0	1	Thoracotoı	73	CHG	No	65	0	1
0	0	Thoracotoı	73	CHG	No	68	1	1
0	0	Thoracotoı	73	CHG	No	77	0	1
0	0	Thoracotoı	73	CHG	No	71	1	1
0	0	Thoracotoı	73	CHG	No	72	0	0
0	0	Thoracotoı	73	CHG	No	81	0	1
0	0	Thoracotoı	73	CHG	No	65	0	1
0	0	Thoracotoı	73	CHG	No	52	0	1
0	0	Thoracotoı	73	CHG	No	56	0	0
0	0	Thoracotoı	73	CHG	No	76	0	1
0	0	Thoracotoı	73	CHG	No	75	0	1
0	0	Thoracotoı	73	CHG	No	82	0	1
0	0	Thoracotoı	73	CHG	Yes	67	0	1
0	0	Thoracotoı	73	CHG	No	71	0	1
0	0	Thoracotoı	73	CHG	No	45	0	1
0	1	Thoracotoı	73	CHG	No	51	0	1
0	0	Thoracotoı	73	CHG	No	58	0	1
0	0	Thoracotoı	73	CHG	No	62	0	1
0	0	VATS or rc	73	CHG	No	66	0	1
0	0	Thoracotoı	73	CHG	No	66	0	1
0	0	VATS or rc	73	CHG	No	59	0	1
0	1	Thoracotoı	73	CHG	No	74	0	1
0	0	Thoracotoı	73	CHG	No	56	1	1
0	0	Thoracotoı	73	CHG	No	65	0	1
0	0	Thoracotoı	73	CHG	No	68	0	1
0	0	Thoracotoı	73	CHG	No	64	1	1
0	0	Thoracotoı	73	CHG	No	75	0	1
0	1	Thoracotoı	73	CHG	No	59	0	1
0	1	Thoracotoı	73	CHG	No	66	0	1
0	0	Thoracotoı	73	CHG	No	57	0	1
0	1	Thoracotoı	73	CHG	No	68	0	1
0	0	Thoracotoı	73	CHG	No	58	0	1
0	0	Thoracotoı	73	CHG	No	80	0	1
0	0	Thoracotoı	73	CHG	No	63	0	1
0	0	VATS or rc	73	CHG	No	75	0	1
0	0	VATS or rc	73	CHG	No	57	1	1
0	0	Thoracotoı	73	CHG	No	74	1	0

0	0	Thoracotoı	73	CHG	No	66	0	1
0	1	Thoracotoı	73	CHG	No	62	0	1
0	0	Thoracotoı	73	CHG	No	69	0	1
0	0	Thoracotoı	73	CHG	No	67	0	1
0	0	Thoracotoı	73	CHG	No	76	0	1
0	0	Thoracotoı	73	CHG	No	76	0	1
0	0	Thoracotoı	73	CHG	No	56	0	1
0	0	Thoracotoı	73	CHG	No	64	1	1
0	1	Thoracotoı	73	CHG	No	66	0	1
0	0	Thoracotoı	73	CHG	No	76	0	1
0	1	Thoracotoı	73	CHG	No	78	0	1
0	1	Thoracotoı	73	CHG	No	68	0	1
0	0	Thoracotoı	73	CHG	No	70	0	1
0	0	Thoracotoı	73	CHG	No	47	0	1
0	0	Thoracotoı	73	CHG	No	62	0	1
0	1	Thoracotoı	73	CHG	No	65	0	1
0	0	VATS or rc	73	CHG	No	46	0	1
0	0	Thoracotoı	73	CHG	No	57	0	1
0	0	Thoracotoı	73	CHG	Yes	71	1	1
0	0	Thoracotoı	73	CHG	No	62	0	0
0	1	Thoracotoı	73	CHG	No	72	0	1
0	0	Thoracotoı	73	CHG	No	36	0	1
0	0	Thoracotoı	73	CHG	No	74	0	1
0	0	Thoracotoı	73	CHG	No	73	0	1
0	0	Thoracotoı	73	CHG	No	67	0	0
0	0	Thoracotoı	73	CHG	No	57	0	1
0	0	Thoracotoı	73	CHG	No	61	0	1
0	0	VATS or rc	73	CHG	No	66	0	1
0	0	Thoracotoı	73	CHG	No	58	0	1
0	0	Thoracotoı	73	CHG	No	63	1	1
0	0	Thoracotoı	73	CHG	No	61	1	1
0	1	Thoracotoı	73	CHG	No	65	0	1
0	0	VATS or rc	73	CHG	No	72	0	0
0	0	VATS or rc	73	CHG	No	68	0	1
0	0	VATS or rc	73	CHG	No	72	0	0
0	0	Thoracotoı	73	CHG	No	66	0	1
0	0	Thoracotoı	73	CHG	No	60	0	1
0	1	Thoracotoı	73	CHG	No	53	0	1
0	0	Thoracotoı	73	CHG	No	65	0	1
0	0	Thoracotoı	73	CHG	No	50	0	1
0	0	Thoracotoı	73	CHG	No	64	0	1
0	0	VATS or rc	73	CHG	No	71	0	1
0	0	Thoracotoı	73	CHG	No	63	0	1
0	0	VATS or rc	73	CHG	No	63	0	1
0	0	Thoracotoı	73	CHG	No	55	0	1
0	0	Thoracotoı	73	CHG	No	62	0	1
0	0	Thoracotoı	73	CHG	No	62	0	1
0	0	Thoracotoı	73	CHG	No	58	0	1
0	1	Thoracotoı	73	CHG	No	77	0	1
0	0	Thoracotoı	73	CHG	No	68	1	1
0	0	Thoracotoı	73	CHG	No	66	0	1
0	0	Thoracotoı	73	CHG	No	77	0	1
0	0	VATS or robotic		CHU	No	65	0	0
0	0	VATS or robotic		CHU	No	62	0	0
1	0	Thoracotoı	59	PRIVE	No	70	0	0
1	0	VATS or rc	59	PRIVE	No	79	0	1

1	0	VATS or rc	59	PRIVE	No	69	0	0
1	0	Thoracotoı	59	PRIVE	No	66	0	0
1	0	VATS or rc	59	PRIVE	No	59	0	0
1	0	Thoracotoı	59	PRIVE	No	66	0	0
1	0	VATS or rc	59	PRIVE	Yes	63	1	1
1	0	VATS or rc	59	PRIVE	No	70	0	0
1	0	VATS or rc	59	PRIVE	No	73	0	0
1	0	Thoracotoı	59	PRIVE	No	60	0	0
1	0	VATS or rc	59	PRIVE	No	77	0	0
1	0	VATS or rc	59	PRIVE	No	68	0	0
1	0	Thoracotoı	59	PRIVE	No	69	0	0
1	0	Thoracotoı	59	PRIVE	No	55	0	0
1	0	VATS or rc	59	PRIVE	No	50	0	0
1	0	Thoracotoı	59	PRIVE	No	60	0	0
1	0	VATS or rc	59	PRIVE	No	64	0	1
1	0	Thoracotoı	59	PRIVE	No	73	0	0
1	0	VATS or rc	59	PRIVE	No	66	0	0
1	0	Thoracotoı	59	PRIVE	No	67	0	0
1	0	VATS or rc	59	PRIVE	No	70	0	0
1	0	VATS or rc	59	PRIVE	No	58	0	0
1	0	Thoracotoı	59	PRIVE	No	62	0	0
1	0	VATS or rc	59	PRIVE	No	78	0	0
1	0	Thoracotoı	59	PRIVE	No	68	0	0
1	0	Thoracotoı	59	PRIVE	No	61	0	0
1	0	VATS or rc	59	PRIVE	No	81	0	0
1	0	VATS or rc	59	PRIVE	No	63	0	1
1	0	Thoracotoı	59	PRIVE	No	73	0	0
1	0	VATS or rc	59	PRIVE	No	57	0	0
1	1	Thoracotoı	59	PRIVE	No	56	0	0
1	0	Thoracotoı	59	PRIVE	No	57	0	0
1	0	VATS or rc	59	PRIVE	No	57	0	1
1	0	VATS or rc	59	PRIVE	No	55	0	0
1	0	Thoracotoı	59	PRIVE	No	74	0	1
1	0	Thoracotoı	59	PRIVE	No	71	0	0
1	0	Thoracotoı	59	PRIVE	No	73	0	0
1	0	VATS or rc	59	PRIVE	No	73	0	0
1	0	VATS or rc	59	PRIVE	No	53	0	0
1	0	Thoracotoı	59	PRIVE	No	52	0	1
1	1	Thoracotoı	59	PRIVE	No	56	0	0
1	0	VATS or rc	59	PRIVE	No	48	0	0
1	0	Thoracotoı	59	PRIVE	No	60	0	0
1	0	VATS or rc	59	PRIVE	No	53	0	0
1	0	Thoracotoı	59	PRIVE	No	33	0	0
1	1	Thoracotoı	59	PRIVE	No	59	0	0
1	1	VATS or rc	59	PRIVE	No	47	0	0
1	0	Thoracotoı	59	PRIVE	No	73	0	1
1	1	Thoracotoı	59	PRIVE	No	61	0	0
1	0	VATS or rc	59	PRIVE	No	42	0	0
1	0	VATS or rc	59	PRIVE	No	74	1	0
1	1	Thoracotoı	59	PRIVE	No	69	0	0
1	0	VATS or rc	59	PRIVE	No	47	0	0
1	0	Thoracotoı	59	PRIVE	Yes	62	0	0
1	1	Thoracotoı	59	PRIVE	No	70	0	0
1	0	VATS or rc	59	PRIVE	No	53	0	0
1	1	Thoracotoı	59	PRIVE	No	63	1	0
1	0	VATS or rc	59	PRIVE	No	69	0	0

1	0	Thoracotoı	59	PRIVE	No	71	0	0
1	0	Thoracotoı	59	PRIVE	No	67	0	0
1	0	Thoracotoı	59	PRIVE	No	74	0	0
1	0	VATS or rc	59	PRIVE	No	50	0	0
1	0	Thoracotoı	59	PRIVE	No	71	0	0
1	0	Thoracotoı	59	PRIVE	No	75	1	0
1	0	VATS or rc	59	PRIVE	No	63	0	0
1	0	Thoracotoı	59	PRIVE	No	65	0	0
1	0	Thoracotoı	59	PRIVE	No	67	0	0
1	0	VATS or rc	59	PRIVE	No	59	0	1
1	0	Thoracotoı	59	PRIVE	No	81	0	0
1	0	Thoracotoı	59	PRIVE	No	71	0	1
1	0	Thoracotoı	59	PRIVE	No	55	0	1
1	0	Thoracotoı	59	PRIVE	No	63	0	0
1	0	VATS or rc	59	PRIVE	No	69	0	0
1	0	Thoracotoı	59	PRIVE	No	74	1	0
1	0	Thoracotoı	59	PRIVE	No	73	0	0
1	0	Thoracotoı	59	PRIVE	No	57	0	0
1	1	Thoracotoı	59	PRIVE	No	66	0	0
1	1	Thoracotoı	59	PRIVE	No	77	1	0
1	0	Thoracotoı	59	PRIVE	No	59	1	0
1	0	Thoracotoı	59	PRIVE	Yes	71	0	0
1	0	Thoracotoı	59	PRIVE	No	60	0	0
1	1	Thoracotoı	59	PRIVE	No	51	0	1
1	0	VATS or rc	59	PRIVE	No	67	0	0
1	1	Thoracotoı	59	PRIVE	No	47	0	0
1	0	Thoracotoı	59	PRIVE	No	73	0	0
1	0	VATS or rc	59	PRIVE	No	80	0	0
1	0	Thoracotoı	59	PRIVE	No	77	0	0
1	0	VATS or rc	59	PRIVE	No	74	0	0
1	0	Thoracotoı	59	PRIVE	No	77	0	0
1	0	Thoracotoı	59	PRIVE	No	55	0	1
1	0	Thoracotoı	59	PRIVE	No	78	1	0
1	0	Thoracotoı	59	PRIVE	No	71	0	0
1	0	VATS or rc	59	PRIVE	No	70	0	0
1	0	VATS or rc	59	PRIVE	No	50	0	1
1	0	Thoracotoı	59	PRIVE	No	59	0	1
1	0	Thoracotoı	59	PRIVE	No	75	0	0
1	0	VATS or rc	59	PRIVE	No	42	0	0
1	0	Thoracotoı	59	PRIVE	No	61	0	0
1	0	Thoracotoı	59	PRIVE	No	69	0	0
1	0	Thoracotoı	59	PRIVE	No	60	0	0
1	0	Thoracotoı	59	PRIVE	No	67	0	0
1	0	Thoracotoı	59	PRIVE	No	82	0	0
1	0	Thoracotoı	59	PRIVE	No	75	1	0
1	0	VATS or rc	59	PRIVE	No	67	0	1
1	0	VATS or rc	59	PRIVE	No	48	0	0
1	0	Thoracotoı	59	PRIVE	No	67	0	0
1	0	Thoracotoı	59	PRIVE	No	62	0	0
1	0	Thoracotoı	59	PRIVE	No	65	0	0
1	1	Thoracotoı	59	PRIVE	No	59	0	0
1	0	Thoracotoı	59	PRIVE	No	60	0	0
1	0	Thoracotoı	59	PRIVE	No	71	0	0
1	1	Thoracotoı	59	PRIVE	No	73	0	0
1	0	Thoracotoı	59	PRIVE	No	71	0	0
1	0	VATS or rc	59	PRIVE	No	61	1	0

1	0	Thoracotoı	59	PRIVE	No	74	0	1
1	0	Thoracotoı	59	PRIVE	No	59	0	0
1	0	Thoracotoı	59	PRIVE	Yes	68	0	0
1	0	Thoracotoı	59	PRIVE	No	55	0	0
1	0	VATS or rc	59	PRIVE	No	50	0	0
1	0	VATS or rc	59	PRIVE	No	71	0	0
1	0	Thoracotoı	59	PRIVE	No	65	0	0
1	0	Thoracotoı	59	PRIVE	No	69	0	0
1	0	Thoracotoı	59	PRIVE	No	40	0	1
1	0	VATS or rc	59	PRIVE	No	53	0	0
1	1	Thoracotoı	59	PRIVE	No	66	0	0
1	0	Thoracotoı	59	PRIVE	No	71	0	0
1	0	VATS or rc	59	PRIVE	No	67	0	1
1	0	VATS or rc	59	PRIVE	No	56	0	0
1	0	Thoracotoı	59	PRIVE	No	76	0	0
1	0	VATS or rc	59	PRIVE	No	78	0	0
1	0	Thoracotoı	59	PRIVE	No	70	0	0
1	0	Thoracotoı	59	PRIVE	No	46	0	1
1	1	Thoracotoı	59	PRIVE	No	59	0	0
1	0	VATS or rc	59	PRIVE	No	69	0	0
1	0	VATS or rc	59	PRIVE	No	71	0	1
1	0	Thoracotoı	59	PRIVE	No	60	0	0
1	0	Thoracotoı	59	PRIVE	No	64	0	0
1	0	VATS or rc	59	PRIVE	No	53	0	0
1	0	VATS or rc	59	PRIVE	No	63	0	0
1	0	VATS or rc	59	PRIVE	No	70	0	0
1	0	Thoracotoı	59	PRIVE	No	67	0	0
1	0	Thoracotoı	59	PRIVE	No	70	0	0
1	0	Thoracotoı	59	PRIVE	No	62	0	0
1	0	Thoracotoı	59	PRIVE	No	73	0	0
1	0	VATS or rc	59	PRIVE	No	72	0	0
1	1	Thoracotoı	59	PRIVE	Yes	50	0	1
1	0	Thoracotoı	59	PRIVE	No	71	0	0
1	0	Thoracotoı	59	PRIVE	No	59	1	0
1	0	Thoracotoı	59	PRIVE	No	55	0	1
1	0	Thoracotoı	59	PRIVE	No	71	0	0
1	0	Thoracotoı	59	PRIVE	No	79	0	0
1	0	Thoracotoı	59	PRIVE	No	74	0	0
1	0	VATS or rc	59	PRIVE	No	60	0	0
1	1	Thoracotoı	59	PRIVE	No	77	1	0
1	0	Thoracotoı	59	PRIVE	No	64	0	0
1	0	VATS or rc	59	PRIVE	No	69	0	0
1	0	Thoracotoı	59	PRIVE	No	67	0	0
1	0	Thoracotoı	59	PRIVE	No	55	0	1
1	0	VATS or rc	59	PRIVE	No	63	0	0
1	0	Thoracotoı	59	PRIVE	No	70	0	0
1	0	VATS or rc	59	PRIVE	No	63	0	1
1	0	Thoracotoı	59	PRIVE	No	64	0	0
1	0	Thoracotoı	59	PRIVE	No	72	0	0
1	1	Thoracotoı	59	PRIVE	No	69	0	0
0	0	VATS or rc	36	PRIVE	No	76	0	0
0	1	Thoracotoı	92	CHU	No	49	0	0
0	0	Thoracotoı	92	CHU	No	54	0	0
0	0	Thoracotoı	92	CHU	No	66	0	0
0	0	Thoracotoı	92	CHU	No	72	1	1
0	0	Thoracotoı	92	CHU	No	63	0	1

0	0	Thoracotoı	92	CHU	No	57	0	1
0	0	Thoracotoı	92	CHU	No	65	0	0
0	0	Thoracotoı	92	CHU	No	75	0	0
0	0	VATS or rc	92	CHU	No	77	0	0
0	0	Thoracotoı	92	CHU	No	66	0	0
0	0	VATS or rc	92	CHU	No	76	0	1
0	0	VATS or rc	92	CHU	No	63	0	1
0	0	VATS or rc	92	CHU	No	68	0	0
0	1	Thoracotoı	92	CHU	No	49	0	0
0	0	Thoracotoı	92	CHU	No	70	0	1
0	0	Thoracotoı	92	CHU	No	74	1	0
0	0	VATS or rc	92	CHU	No	51	0	0
0	0	VATS or rc	92	CHU	No	48	0	1
0	0	VATS or rc	92	CHU	No	80	1	0
0	0	VATS or rc	92	CHU	No	61	0	1
0	0	Thoracotoı	92	CHU	No	61	0	1
0	1	Thoracotoı	92	CHU	Yes	83	0	1
0	0	Thoracotoı	92	CHU	No	74	0	0
0	0	VATS or rc	92	CHU	No	57	0	1
0	0	Thoracotoı	92	CHU	No	70	0	0
0	0	Thoracotoı	92	CHU	No	71	1	0
0	0	Thoracotoı	92	CHU	No	69	0	1
0	1	Thoracotoı	92	CHU	No	76	0	0
0	0	VATS or rc	92	CHU	No	55	0	1
0	0	Thoracotoı	92	CHU	No	63	0	0
0	0	Thoracotoı	92	CHU	No	79	0	0
0	0	VATS or rc	92	CHU	No	61	0	0
0	0	Thoracotoı	92	CHU	No	72	0	0
0	0	Thoracotoı	92	CHU	No	79	0	0
0	0	Thoracotoı	92	CHU	No	66	0	1
0	0	VATS or rc	92	CHU	No	47	0	0
0	0	Thoracotoı	92	CHU	No	63	0	1
0	1	Thoracotoı	92	CHU	No	73	0	1
0	0	Thoracotoı	92	CHU	No	70	0	0
0	0	Thoracotoı	92	CHU	No	68	0	0
0	0	Thoracotoı	92	CHU	No	66	1	0
0	0	VATS or rc	92	CHU	No	68	0	1
0	0	Thoracotoı	92	CHU	No	63	0	0
0	0	Thoracotoı	92	CHU	No	73	0	1
0	0	VATS or rc	92	CHU	No	68	0	1
0	0	VATS or rc	92	CHU	No	57	0	0
0	0	Thoracotoı	92	CHU	No	58	0	1
0	0	VATS or rc	92	CHU	No	58	0	1
0	0	Thoracotoı	92	CHU	No	85	0	1
0	0	Thoracotoı	92	CHU	No	72	1	1
0	0	Thoracotoı	92	CHU	No	63	0	1
0	1	Thoracotoı	92	CHU	No	63	0	1
0	1	Thoracotoı	92	CHU	No	68	0	1
0	0	Thoracotoı	92	CHU	No	76	0	0
0	0	Thoracotoı	92	CHU	No	71	0	0
0	1	Thoracotoı	92	CHU	No	61	0	1
0	0	Thoracotoı	92	CHU	No	82	0	1
0	0	Thoracotoı	92	CHU	No	62	0	1
0	0	VATS or rc	92	CHU	No	72	0	1
0	0	VATS or rc	92	CHU	No	78	0	0
0	0	Thoracotoı	92	CHU	No	50	0	1

0	1	Thoracotoı	92	CHU	No	74	0	0
0	0	VATS or rc	92	CHU	No	70	0	0
0	0	Thoracotoı	92	CHU	No	56	0	1
0	0	VATS or rc	92	CHU	No	64	0	1
0	0	Thoracotoı	92	CHU	No	60	0	0
0	0	VATS or rc	92	CHU	No	82	0	0
0	0	Thoracotoı	92	CHU	No	61	0	0
0	0	Thoracotoı	92	CHU	No	58	0	1
0	0	Thoracotoı	92	CHU	No	75	0	1
0	0	Thoracotoı	92	CHU	No	53	0	0
0	0	VATS or rc	92	CHU	No	78	0	0
0	0	Thoracotoı	92	CHU	No	71	0	0
0	0	Thoracotoı	92	CHU	No	67	0	0
0	0	Thoracotoı	92	CHU	No	66	1	1
0	0	VATS or rc	92	CHU	No	70	0	1
0	0	Thoracotoı	92	CHU	No	56	0	1
0	0	Thoracotoı	92	CHU	No	62	0	1
0	0	Thoracotoı	92	CHU	No	80	0	1
0	0	Thoracotoı	92	CHU	No	61	0	0
0	0	Thoracotoı	92	CHU	No	43	0	0
0	1	Thoracotoı	92	CHU	No	59	0	0
0	0	Thoracotoı	92	CHU	No	74	0	0
0	0	Thoracotoı	92	CHU	No	74	0	1
0	0	Thoracotoı	92	CHU	No	60	0	0
0	0	Thoracotoı	92	CHU	No	50	0	1
0	0	VATS or rc	92	CHU	No	65	1	1
0	0	Thoracotoı	92	CHU	No	66	0	1
0	0	Thoracotoı	92	CHU	No	87	0	0
0	0	Thoracotoı	92	CHU	No	52	0	1
0	0	Thoracotoı	92	CHU	No	55	0	1
0	0	Thoracotoı	92	CHU	No	70	0	0
0	0	Thoracotoı	92	CHU	No	60	0	1
0	0	VATS or rc	92	CHU	No	69	0	0
0	0	Thoracotoı	92	CHU	No	72	0	0
0	0	Thoracotoı	92	CHU	No	65	1	0
0	0	VATS or rc	92	CHU	No	54	0	0
0	0	Thoracotoı	92	CHU	No	74	0	1
0	0	Thoracotoı	92	CHU	Yes	87	0	0
0	0	VATS or rc	92	CHU	No	62	0	0
0	0	Thoracotoı	92	CHU	No	66	0	0
0	0	Thoracotoı	92	CHU	No	73	0	1
0	0	VATS or rc	92	CHU	No	53	0	1
0	0	Thoracotoı	92	CHU	No	84	0	0
0	0	VATS or rc	92	CHU	No	74	1	0
0	0	Thoracotoı	92	CHU	No	66	0	1
0	1	Thoracotoı	92	CHU	No	60	0	1
0	0	Thoracotoı	92	CHU	No	81	0	1
0	0	VATS or rc	92	CHU	No	73	0	1
0	0	VATS or rc	92	CHU	No	70	0	0
0	0	Thoracotoı	92	CHU	No	58	0	0
0	0	VATS or rc	92	CHU	No	67	1	0
0	0	Thoracotoı	92	CHU	No	69	0	1
0	0	VATS or rc	92	CHU	No	68	1	0
0	0	Thoracotoı	92	CHU	No	68	1	0
0	0	Thoracotoı	92	CHU	No	56	0	1
0	0	VATS or rc	92	CHU	Yes	76	0	1

0	0	Thoracotoı	92	CHU	No	51	0	1
0	0	VATS or rc	92	CHU	No	61	0	1
0	0	Thoracotoı	92	CHU	No	81	0	0
0	0	Thoracotoı	92	CHU	No	76	0	0
0	0	Thoracotoı	92	CHU	No	52	0	0
0	0	Thoracotoı	92	CHU	No	58	1	1
0	1	Thoracotoı	92	CHU	No	70	0	0
0	0	Thoracotoı	92	CHU	No	70	0	0
0	0	Thoracotoı	92	CHU	No	67	0	0
0	0	VATS or rc	92	CHU	No	68	0	0
0	0	VATS or rc	92	CHU	No	69	0	0
0	1	Thoracotoı	92	CHU	No	62	1	1
0	0	Thoracotoı	92	CHU	No	60	0	0
0	0	VATS or rc	92	CHU	No	67	0	0
0	0	Thoracotoı	92	CHU	No	66	0	1
0	1	Thoracotoı	92	CHU	No	62	0	1
0	0	Thoracotoı	92	CHU	No	75	1	1
0	0	VATS or rc	92	CHU	No	68	1	0
0	0	VATS or rc	92	CHU	No	61	0	1
0	0	VATS or rc	92	CHU	No	48	0	1
0	0	VATS or rc	80	CHU	No	57	0	0
0	0	VATS or rc	80	CHU	No	68	0	0
0	0	VATS or rc	80	CHU	No	56	0	0
0	0	VATS or rc	80	CHU	No	68	0	0
0	0	VATS or rc	80	CHU	No	57	0	0
0	0	VATS or rc	80	CHU	No	59	0	0
0	0	VATS or rc	80	CHU	No	69	0	0
0	0	VATS or rc	80	CHU	No	59	0	0
0	0	VATS or rc	80	CHU	No	46	0	0
0	0	VATS or rc	80	CHU	No	58	0	0
0	0	Thoracotoı	80	CHU	No	74	0	0
0	0	VATS or rc	80	CHU	No	70	0	1
0	1	Thoracotoı	80	CHU	No	63	0	0
0	0	VATS or rc	80	CHU	No	59	0	0
0	0	VATS or rc	80	CHU	No	71	0	0
0	0	VATS or rc	80	CHU	No	73	0	0
0	0	Thoracotoı	80	CHU	No	65	1	0
0	0	VATS or rc	80	CHU	No	71	0	0
0	0	VATS or rc	80	CHU	No	64	0	0
0	0	Thoracotoı	80	CHU	No	69	0	0
0	0	Thoracotoı	80	CHU	No	65	0	0
0	0	VATS or rc	80	CHU	No	69	0	1
0	0	VATS or rc	80	CHU	No	67	1	1
0	0	VATS or rc	80	CHU	No	63	0	0
0	0	VATS or rc	80	CHU	No	70	0	0
0	0	VATS or rc	80	CHU	No	51	0	0
0	0	VATS or rc	80	CHU	No	84	0	0
0	0	VATS or rc	80	CHU	No	58	0	1
0	0	Thoracotoı	80	CHU	No	68	0	1
0	0	Thoracotoı	80	CHU	No	75	0	0
0	1	Thoracotoı	80	CHU	No	61	0	0
0	0	VATS or rc	80	CHU	No	75	0	0
0	0	Thoracotoı	80	CHU	No	59	0	0
0	0	VATS or rc	80	CHU	No	63	0	0
0	0	VATS or rc	80	CHU	No	63	0	0
0	0	VATS or rc	80	CHU	No	68	0	1

0	1	Thoracotor	80	CHU	No	63	0	0
0	0	VATS or rc	80	CHU	No	58	0	1
0	0	VATS or rc	80	CHU	No	61	0	1
0	1	Thoracotor	80	CHU	No	48	0	1
0	0	VATS or rc	80	CHU	No	52	0	0
0	0	VATS or rc	80	CHU	No	75	0	0
0	0	Thoracotor	80	CHU	No	58	0	0
0	0	VATS or rc	80	CHU	No	64	0	0
0	0	VATS or rc	80	CHU	No	82	0	0
0	0	VATS or rc	80	CHU	No	69	0	0
0	0	VATS or rc	80	CHU	No	66	0	0
0	0	VATS or rc	80	CHU	No	70	0	0
0	0	VATS or rc	80	CHU	No	77	0	0
0	0	VATS or rc	80	CHU	No	68	0	1
0	0	VATS or rc	80	CHU	No	51	0	0
0	0	Thoracotor	80	CHU	No	62	0	1
0	0	Thoracotor	80	CHU	No	67	0	0
0	0	VATS or rc	80	CHU	No	52	0	0
0	0	Thoracotor	80	CHU	No	65	0	0
0	0	Thoracotor	80	CHU	No	49	0	1
0	0	VATS or rc	80	CHU	No	68	0	0
0	0	VATS or rc	80	CHU	No	59	0	0
0	0	VATS or rc	80	CHU	No	73	1	1
0	0	VATS or rc	80	CHU	No	48	0	0
0	0	Thoracotor	80	CHU	No	69	0	0
0	0	VATS or rc	80	CHU	No	79	0	0
0	0	VATS or rc	80	CHU	No	66	0	0
0	0	VATS or rc	80	CHU	No	67	0	0
0	0	Thoracotor	80	CHU	No	22	0	0
0	0	Thoracotor	80	CHU	No	72	0	0
0	0	VATS or rc	80	CHU	No	57	0	1
0	0	VATS or rc	80	CHU	No	42	0	0
0	0	VATS or rc	80	CHU	No	49	0	1
0	0	VATS or rc	80	CHU	No	70	0	0
0	0	VATS or rc	80	CHU	No	62	0	0
0	0	VATS or rc	80	CHU	No	57	0	1
0	0	VATS or rc	80	CHU	No	53	0	1
0	0	VATS or rc	80	CHU	No	67	0	0
0	0	VATS or rc	80	CHU	No	54	0	0
0	0	Thoracotor	80	CHU	No	64	0	0
0	0	VATS or rc	80	CHU	No	79	0	0
0	0	VATS or rc	80	CHU	No	70	0	0
0	0	VATS or rc	80	CHU	No	52	0	0
0	0	VATS or rc	80	CHU	No	74	0	0
0	0	VATS or rc	80	CHU	No	77	0	1
0	0	VATS or rc	80	CHU	No	42	0	0
0	0	VATS or rc	80	CHU	No	68	0	0
0	0	Thoracotor	80	CHU	No	52	0	0
0	0	VATS or rc	80	CHU	No	63	0	0
0	0	Thoracotor	80	CHU	No	78	0	0
0	1	VATS or rc	80	CHU	No	63	0	0
0	0	VATS or rc	80	CHU	No	69	0	0
0	0	VATS or rc	80	CHU	No	53	0	0
0	0	Thoracotor	80	CHU	No	70	0	0
0	0	VATS or rc	80	CHU	No	62	0	0
0	0	VATS or rc	80	CHU	No	61	0	0

0	0	Thoracotoı	80	CHU	No	57	0	1
0	0	Thoracotoı	80	CHU	No	75	0	0
0	0	VATS or rc	80	CHU	No	56	0	0
0	0	VATS or rc	80	CHU	No	68	0	0
0	0	VATS or rc	80	CHU	No	57	0	0
0	0	VATS or rc	80	CHU	No	42	0	0
0	0	VATS or rc	80	CHU	No	50	0	1
0	0	VATS or rc	80	CHU	No	62	0	1
0	0	VATS or rc	80	CHU	No	68	0	0
0	0	VATS or rc	80	CHU	No	71	0	0
0	0	VATS or rc	80	CHU	No	64	0	0
0	0	VATS or rc	80	CHU	No	65	0	0
0	0	VATS or rc	80	CHU	No	74	0	0
0	0	VATS or rc	80	CHU	No	54	0	0
0	0	VATS or rc	80	CHU	No	57	0	0
0	0	VATS or rc	80	CHU	No	75	0	0
0	0	VATS or rc	80	CHU	No	78	0	0
0	0	VATS or rc	80	CHU	No	63	0	0
0	0	VATS or rc	80	CHU	No	63	0	0
0	0	VATS or rc	80	CHU	No	60	1	1
0	0	VATS or rc	80	CHU	No	72	0	0
0	0	VATS or rc	80	CHU	No	71	1	0
0	0	Thoracotoı	80	CHU	No	64	0	0
0	0	VATS or rc	80	CHU	No	60	0	0
0	0	Thoracotoı	80	CHU	No	58	0	0
0	1	VATS or rc	80	CHU	No	55	0	0
0	1	Thoracotoı	80	CHU	No	70	0	0
0	0	VATS or rc	80	CHU	No	46	0	0
0	0	Thoracotoı	80	CHU	No	63	0	0
0	0	VATS or rc	80	CHU	No	54	0	0
0	0	Thoracotoı	80	CHU	No	44	0	0
0	0	VATS or rc	80	CHU	No	76	0	0
0	0	Thoracotoı	80	CHU	No	77	0	1
0	1	Thoracotoı	80	CHU	No	77	0	0
0	0	Thoracotoı	46	CHU	No	68	0	0
0	0	VATS or rc	46	CHU	No	58	0	1
0	0	Thoracotoı	46	CHU	No	53	0	0
0	1	Thoracotoı	46	CHU	No	69	0	0
0	0	VATS or rc	46	CHU	No	63	0	1
0	0	VATS or rc	46	CHU	No	72	0	0
0	0	VATS or rc	46	CHU	No	65	0	1
0	0	VATS or rc	46	CHU	No	68	0	0
0	0	Thoracotoı	46	CHU	No	38	0	0
0	0	Thoracotoı	46	CHU	No	65	0	1
0	1	Thoracotoı	46	CHU	No	73	0	0
0	0	VATS or rc	46	CHU	No	60	0	1
0	0	Thoracotoı	46	CHU	No	59	0	0
0	0	Thoracotoı	46	CHU	No	44	0	1
1	0	Thoracotoı	122	CHU	No	57	0	0
1	1	Thoracotoı	122	CHU	No	54	0	1
1	0	VATS or rc	122	CHU	No	51	0	0
1	0	Thoracotoı	122	CHU	No	56	0	0
1	1	Thoracotoı	122	CHU	No	68	0	0
1	0	Thoracotoı	122	CHU	No	61	0	0
1	0	Thoracotoı	122	CHU	No	62	0	0
1	0	Thoracotoı	122	CHU	No	63	0	0

1	0	Thoracotoı	122	CHU	No	65	0	0
1	0	Thoracotoı	122	CHU	No	62	0	0
1	0	Thoracotoı	122	CHU	No	65	0	0
1	0	Thoracotoı	122	CHU	No	63	0	1
1	0	Thoracotoı	122	CHU	No	63	0	0
1	0	VATS or rc	122	CHU	No	64	0	0
1	0	VATS or rc	122	CHU	No	59	0	0
1	0	Thoracotoı	122	CHU	No	70	1	0
1	0	VATS or rc	122	CHU	No	58	0	0
1	0	VATS or rc	122	CHU	No	77	0	0
1	0	Thoracotoı	122	CHU	No	56	0	0
1	0	VATS or rc	122	CHU	No	61	0	0
1	0	VATS or rc	122	CHU	No	60	0	0
1	0	VATS or rc	122	CHU	No	53	0	1
1	0	Thoracotoı	122	CHU	No	74	0	0
1	0	VATS or rc	122	CHU	No	74	1	0
1	0	Thoracotoı	122	CHU	No	61	0	0
1	0	Thoracotoı	122	CHU	No	79	0	0
1	0	Thoracotoı	122	CHU	No	63	0	0
1	0	Thoracotoı	122	CHU	No	81	1	0
1	0	VATS or rc	122	CHU	No	61	0	0
1	0	Thoracotoı	122	CHU	No	55	0	1
1	0	Thoracotoı	122	CHU	No	44	0	1
1	0	VATS or rc	122	CHU	No	79	0	0
1	0	Thoracotoı	122	CHU	No	52	0	0
1	0	VATS or rc	122	CHU	No	66	0	0
1	0	Thoracotoı	122	CHU	No	79	0	0
1	0	Thoracotoı	122	CHU	No	78	0	0
1	0	Thoracotoı	122	CHU	No	62	0	0
1	0	Thoracotoı	122	CHU	No	55	0	0
1	0	VATS or rc	122	CHU	No	69	0	0
1	0	Thoracotoı	122	CHU	No	62	0	0
1	0	VATS or rc	122	CHU	No	50	0	0
1	0	Thoracotoı	122	CHU	No	78	0	0
1	0	Thoracotoı	122	CHU	No	67	0	1
1	0	Thoracotoı	122	CHU	No	70	0	1
1	0	Thoracotoı	122	CHU	No	64	0	1
1	0	Thoracotoı	122	CHU	No	52	0	1
1	0	Thoracotoı	122	CHU	No	59	0	0
1	0	Thoracotoı	122	CHU	No	68	0	0
1	0	VATS or rc	122	CHU	No	67	0	0
1	0	VATS or rc	122	CHU	No	75	0	0
1	0	Thoracotoı	122	CHU	No	74	0	0
1	0	Thoracotoı	122	CHU	No	81	0	0
1	0	VATS or rc	122	CHU	No	67	0	0
1	0	VATS or rc	122	CHU	Yes	54	0	0
1	0	Thoracotoı	122	CHU	No	78	1	1
1	0	Thoracotoı	122	CHU	No	59	1	0
1	1	Thoracotoı	122	CHU	No	63	0	0
1	0	Thoracotoı	122	CHU	No	59	0	1
1	0	Thoracotoı	122	CHU	No	75	0	0
1	0	Thoracotoı	122	CHU	No	54	0	0
1	0	VATS or rc	122	CHU	No	65	0	0
1	0	Thoracotoı	122	CHU	Yes	80	0	0
1	0	Thoracotoı	122	CHU	No	67	0	0
1	0	VATS or rc	122	CHU	No	61	0	0

1	0	VATS or rc	122	CHU	No	65	0	1
1	0	Thoracotoı	122	CHU	No	63	0	0
1	0	VATS or rc	122	CHU	No	60	0	0
1	0	Thoracotoı	122	CHU	No	59	0	0
1	0	Thoracotoı	122	CHU	No	76	0	0
1	0	Thoracotoı	122	CHU	No	63	0	0
1	0	Thoracotoı	122	CHU	No	75	0	0
1	0	VATS or rc	122	CHU	No	64	0	0
1	0	VATS or rc	122	CHU	No	78	0	0
1	0	VATS or rc	122	CHU	No	75	1	0
1	0	Thoracotoı	122	CHU	No	59	0	0
1	0	VATS or rc	122	CHU	No	53	0	0
1	0	VATS or rc	122	CHU	No	62	0	0
1	0	Thoracotoı	122	CHU	No	58	0	0
1	0	VATS or rc	122	CHU	No	56	0	0
1	0	Thoracotoı	122	CHU	No	68	0	0
1	0	VATS or rc	122	CHU	No	80	0	0
1	0	Thoracotoı	122	CHU	No	51	0	0
1	1	VATS or rc	122	CHU	No	45	0	0
1	0	Thoracotoı	122	CHU	No	51	0	1
1	0	VATS or rc	122	CHU	No	82	0	0
1	0	Thoracotoı	122	CHU	No	52	0	0
1	0	Thoracotoı	122	CHU	No	60	0	0
1	0	Thoracotoı	122	CHU	No	67	0	0
1	0	Thoracotoı	122	CHU	No	70	0	0
1	0	Thoracotoı	122	CHU	No	29	0	0
1	0	VATS or rc	122	CHU	No	87	0	0
1	0	Thoracotoı	122	CHU	No	48	0	0
1	0	Thoracotoı	122	CHU	No	37	0	0
1	0	VATS or rc	122	CHU	No	68	1	0
1	0	Thoracotoı	122	CHU	No	53	0	0
1	0	Thoracotoı	122	CHU	No	67	0	0
1	0	Thoracotoı	122	CHU	No	33	0	1
1	0	Thoracotoı	122	CHU	No	61	0	1
1	0	Thoracotoı	122	CHU	No	58	0	0
1	0	Thoracotoı	122	CHU	Yes	85	0	0
1	0	Thoracotoı	122	CHU	No	83	0	0
1	0	VATS or rc	122	CHU	No	65	0	0
1	0	Thoracotoı	122	CHU	No	62	0	1
1	1	Thoracotoı	122	CHU	No	66	0	0
1	0	Thoracotoı	122	CHU	No	77	0	0
1	0	Thoracotoı	122	CHU	No	61	0	1
1	0	Thoracotoı	122	CHU	No	58	0	0
1	0	Thoracotoı	122	CHU	No	68	0	1
1	0	Thoracotoı	122	CHU	No	67	0	0
1	0	Thoracotoı	122	CHU	No	68	0	0
1	1	Thoracotoı	122	CHU	No	59	0	0
1	0	Thoracotoı	122	CHU	No	48	0	0
1	0	VATS or rc	122	CHU	No	66	0	0
1	0	Thoracotoı	122	CHU	No	60	0	0
1	0	VATS or rc	122	CHU	No	46	0	0
1	0	Thoracotoı	122	CHU	No	69	0	1
1	0	VATS or rc	122	CHU	No	62	0	0
1	0	Thoracotoı	122	CHU	No	71	0	0
1	0	Thoracotoı	122	CHU	No	72	0	0
1	0	Thoracotoı	122	CHU	No	73	1	0

1	0	Thoracotor	122	CHU	No	75	0	1
1	0	Thoracotor	122	CHU	No	61	0	0
1	0	VATS or rc	122	CHU	No	57	0	0
1	0	Thoracotor	122	CHU	No	51	0	0
1	0	Thoracotor	122	CHU	No	43	0	1
1	0	Thoracotor	122	CHU	No	76	0	0
1	0	Thoracotor	122	CHU	No	56	0	1
1	1	VATS or rc	122	CHU	No	62	0	1
1	0	Thoracotor	122	CHU	No	69	0	0
1	0	Thoracotor	122	CHU	No	62	0	0
1	0	Thoracotor	122	CHU	No	60	0	0
1	0	VATS or rc	122	CHU	No	52	0	1
1	1	VATS or rc	122	CHU	No	52	0	1
1	0	Thoracotor	122	CHU	No	80	0	0
1	0	VATS or rc	122	CHU	No	65	0	0
1	0	VATS or rc	122	CHU	No	70	0	1
1	0	Thoracotor	122	CHU	No	69	0	0
1	0	Thoracotor	122	CHU	No	73	0	0
1	0	Thoracotor	122	CHU	No	57	0	0
1	0	Thoracotor	122	CHU	No	71	0	0
1	0	Thoracotor	122	CHU	No	51	0	0
1	0	Thoracotor	122	CHU	No	58	0	0
1	0	Thoracotor	122	CHU	No	55	0	1
1	0	Thoracotor	122	CHU	No	75	1	0
1	0	Thoracotor	122	CHU	No	66	0	0
1	1	Thoracotor	122	CHU	No	69	0	0
1	0	Thoracotor	122	CHU	No	59	0	0
1	0	Thoracotor	122	CHU	No	79	0	0
1	0	VATS or rc	122	CHU	Yes	59	0	0
1	1	VATS or rc	122	CHU	No	79	0	0
1	0	VATS or rc	122	CHU	No	47	0	0
1	0	VATS or rc	122	CHU	No	55	0	0
1	0	VATS or rc	122	CHU	Yes	71	0	0
1	0	VATS or rc	122	CHU	No	74	1	0
1	0	VATS or rc	122	CHU	No	74	0	0
1	0	Thoracotor	122	CHU	No	60	0	0
1	0	Thoracotor	122	CHU	No	55	0	0
1	0	VATS or rc	122	CHU	No	64	0	0
1	0	Thoracotor	122	CHU	No	69	0	1
1	0	VATS or rc	122	CHU	No	63	0	0
1	1	VATS or rc	122	CHU	No	59	0	0
1	0	Thoracotor	122	CHU	No	47	0	0
1	0	VATS or rc	122	CHU	No	70	0	0
1	0	VATS or rc	122	CHU	No	60	0	1
1	0	VATS or rc	122	CHU	No	70	0	0
1	0	VATS or rc	122	CHU	No	60	0	0
1	0	VATS or rc	122	CHU	No	58	0	0
1	1	Thoracotor	122	CHU	No	57	0	0
1	0	VATS or rc	122	CHU	No	64	0	1
1	1	VATS or rc	122	CHU	No	78	1	0
1	0	Thoracotor	122	CHU	No	58	0	0
1	0	Thoracotor	122	CHU	No	75	0	0
1	0	Thoracotor	122	CHU	No	66	0	1
1	0	VATS or rc	122	CHU	No	63	0	0
1	0	Thoracotor	122	CHU	Yes	78	0	0
1	0	VATS or rc	122	CHU	No	55	0	0

1	0	Thoracotoı	122	CHU	No	72	0	0
1	0	VATS or rc	122	CHU	No	60	0	1
1	0	Thoracotoı	122	CHU	No	67	0	0
1	0	Thoracotoı	122	CHU	No	63	0	1
1	0	VATS or rc	122	CHU	No	79	0	0
1	0	Thoracotoı	122	CHU	No	68	0	0
1	0	Thoracotoı	122	CHU	No	16	0	0
1	1	VATS or rc	122	CHU	No	64	0	0
1	0	VATS or rc	122	CHU	No	68	0	0
1	0	Thoracotoı	122	CHU	No	66	0	0
1	0	Thoracotoı	122	CHU	No	68	0	0
1	0	Thoracotoı	122	CHU	No	66	0	0
1	0	VATS or rc	122	CHU	No	49	0	0
1	0	Thoracotoı	122	CHU	No	76	0	1
1	1	VATS or rc	122	CHU	No	69	0	0
1	0	Thoracotoı	122	CHU	No	62	0	0
1	0	Thoracotoı	122	CHU	No	72	0	0
1	1	Thoracotoı	122	CHU	No	63	1	0
1	0	VATS or rc	122	CHU	No	45	0	0
1	0	Thoracotoı	122	CHU	No	50	0	1
1	0	Thoracotoı	122	CHU	No	46	0	1
1	0	VATS or rc	122	CHU	No	63	0	0
1	0	Thoracotoı	122	CHU	No	59	0	0
1	0	Thoracotoı	122	CHU	No	64	0	0
1	0	Thoracotoı	122	CHU	No	70	0	0
1	0	Thoracotoı	122	CHU	No	33	0	0
1	0	Thoracotoı	122	CHU	No	63	0	1
1	0	VATS or rc	122	CHU	No	69	0	0
1	0	Thoracotoı	122	CHU	No	49	0	0
1	0	Thoracotoı	122	CHU	No	69	0	0
1	0	VATS or rc	122	CHU	No	50	0	0
1	0	Thoracotoı	122	CHU	No	39	0	1
1	0	Thoracotoı	122	CHU	No	52	0	0
1	0	Thoracotoı	122	CHU	No	59	0	0
1	0	Thoracotoı	122	CHU	No	50	0	0
1	0	VATS or rc	122	CHU	No	45	0	1
1	0	VATS or rc	122	CHU	No	68	1	1
1	0	Thoracotoı	122	CHU	No	42	0	0
1	0	Thoracotoı	122	CHU	No	66	0	0
1	0	Thoracotoı	122	CHU	No	65	0	0
1	0	VATS or rc	122	CHU	No	77	1	0
1	0	Thoracotoı	122	CHU	No	55	0	0
1	0	Thoracotoı	122	CHU	No	83	0	0
1	0	Thoracotoı	122	CHU	No	55	1	0
1	1	VATS or rc	122	CHU	No	71	0	1
1	0	Thoracotoı	122	CHU	No	59	0	0
1	0	Thoracotoı	122	CHU	No	68	0	0
1	0	Thoracotoı	122	CHU	No	57	0	0
1	0	Thoracotoı	122	CHU	No	75	1	1
1	0	Thoracotoı	122	CHU	No	64	0	0
1	0	VATS or rc	122	CHU	No	57	0	0
1	1	VATS or rc	122	CHU	No	79	0	0
1	0	Thoracotoı	122	CHU	No	59	0	0
1	0	VATS or rc	122	CHU	No	64	0	0
1	0	Thoracotoı	122	CHU	No	47	0	1
1	0	Thoracotoı	122	CHU	No	56	0	1

1	1	Thoracotoı	122	CHU	No	64	0	1
1	0	Thoracotoı	122	CHU	No	55	0	0
1	0	Thoracotoı	122	CHU	No	71	0	0
1	0	Thoracotoı	122	CHU	No	67	0	1
1	0	Thoracotoı	122	CHU	No	77	0	0
1	0	Thoracotoı	122	CHU	No	74	0	0
1	0	Thoracotoı	122	CHU	No	70	0	1
1	0	VATS or rc	122	CHU	No	65	0	0
1	0	Thoracotoı	122	CHU	No	62	0	0
1	0	Thoracotoı	122	CHU	No	72	0	0
1	0	VATS or rc	122	CHU	No	69	0	0
1	0	Thoracotoı	122	CHU	No	64	0	0
1	0	VATS or rc	122	CHU	No	57	0	1
1	0	Thoracotoı	122	CHU	No	77	0	0
1	0	VATS or rc	122	CHU	No	70	0	0
1	0	Thoracotoı	122	CHU	No	56	0	1
1	0	VATS or rc	122	CHU	No	67	0	0
1	0	VATS or rc	122	CHU	No	65	0	0
1	0	Thoracotoı	122	CHU	No	45	0	0
1	0	Thoracotoı	122	CHU	No	58	0	1
1	0	Thoracotoı	122	CHU	No	76	0	0
1	0	VATS or rc	122	CHU	No	67	0	0
1	0	VATS or rc	122	CHU	No	32	0	0
1	0	Thoracotoı	122	CHU	No	63	0	0
1	0	VATS or rc	122	CHU	No	41	0	0
1	0	VATS or rc	122	CHU	No	65	0	0
1	0	VATS or rc	122	CHU	No	64	0	1
1	0	Thoracotoı	122	CHU	No	34	0	0
1	1	Thoracotoı	122	CHU	No	69	0	0
1	0	Thoracotoı	122	CHU	No	70	0	0
1	0	Thoracotoı	122	CHU	No	60	0	0
1	0	Thoracotoı	122	CHU	No	77	0	1
1	0	Thoracotoı	122	CHU	No	61	0	0
1	0	Thoracotoı	122	CHU	No	62	0	0
1	0	Thoracotoı	122	CHU	No	49	0	0
1	0	Thoracotoı	122	CHU	No	65	0	0
1	0	Thoracotoı	122	CHU	No	67	0	0
1	0	Thoracotoı	122	CHU	No	55	0	0
1	0	Thoracotoı	122	CHU	No	68	0	0
1	0	VATS or rc	122	CHU	No	60	0	1
1	0	VATS or rc	122	CHU	No	70	0	0
1	0	VATS or rc	122	CHU	No	67	0	0
1	0	Thoracotoı	122	CHU	No	84	0	0
1	0	Thoracotoı	122	CHU	No	57	0	0
1	0	Thoracotoı	122	CHU	No	68	0	0
1	0	Thoracotoı	122	CHU	No	74	0	0
1	0	Thoracotoı	122	CHU	No	53	0	0
1	0	Thoracotoı	122	CHU	No	67	0	0
1	0	Thoracotoı	122	CHU	No	75	0	0
1	0	Thoracotoı	122	CHU	No	54	0	0
1	0	Thoracotoı	122	CHU	No	66	0	1
1	0	Thoracotoı	122	CHU	No	48	0	0
1	0	Thoracotoı	122	CHU	No	78	1	0
1	0	VATS or rc	122	CHU	No	58	0	0
1	0	VATS or rc	122	CHU	No	73	0	0
1	0	Thoracotoı	122	CHU	No	70	0	1

1	0	VATS or ro	122	CHU	No	61	0	0
1	0	Thoracotor	122	CHU	No	67	0	0
1	1	VATS or ro	122	CHU	No	50	0	0
1	0	VATS or ro	122	CHU	No	60	0	0
1	0	VATS or ro	122	CHU	No	59	0	0
1	0	Thoracotor	122	CHU	No	63	0	0
1	0	VATS or ro	122	CHU	No	72	0	0
1	0	VATS or ro	122	CHU	No	62	0	0
1	0	VATS or ro	122	CHU	No	68	0	1
1	0	VATS or ro	122	CHU	No	76	0	0
1	0	VATS or ro	122	CHU	No	74	0	0
1	0	Thoracotor	122	CHU	No	53	0	0
1	0	Thoracotor	122	CHU	No	76	0	0
1	1	VATS or ro	122	CHU	No	64	0	0
1	0	Thoracotor	122	CHU	No	68	0	0
1	0	Thoracotor	122	CHU	No	64	0	0
1	0	VATS or ro	122	CHU	No	53	0	0
1	0	Thoracotor	122	CHU	No	75	0	1
1	0	VATS or ro	122	CHU	No	59	0	0
1	0	VATS or ro	122	CHU	No	61	0	0
1	0	Thoracotor	122	CHU	No	51	0	0
1	0	Thoracotor	122	CHU	No	73	0	0
1	1	VATS or ro	122	CHU	No	33	0	0
1	0	VATS or ro	122	CHU	No	63	0	0
1	0	Thoracotor	122	CHU	No	62	0	0
1	0	Thoracotor	122	CHU	No	83	0	0
1	0	Thoracotor	122	CHU	No	84	0	1
1	0	Thoracotor	122	CHU	No	76	0	0
1	0	VATS or ro	122	CHU	No	67	0	0
1	0	Thoracotor	122	CHU	No	55	0	0
1	0	VATS or ro	122	CHU	No	44	0	0
1	0	Thoracotor	122	CHU	No	56	0	1
1	0	Thoracotor	122	CHU	No	64	0	0
1	0	VATS or ro	122	CHU	No	59	0	0
1	0	VATS or ro	122	CHU	No	55	0	0
1	0	Thoracotor	122	CHU	No	46	0	0
1	0	Thoracotor	122	CHU	No	74	0	0
1	0	VATS or ro	122	CHU	No	63	0	0
1	0	Thoracotor	122	CHU	No	60	0	0
1	0	VATS or ro	122	CHU	No	56	0	0
1	0	VATS or ro	122	CHU	No	52	0	0
1	0	Thoracotor	122	CHU	No	70	0	0
1	0	Thoracotor	122	CHU	No	56	0	1
1	0	VATS or ro	122	CHU	No	63	0	0
1	1	VATS or ro	122	CHU	No	57	0	0
1	0	Thoracotor	122	CHU	No	57	0	1
1	0	Thoracotor	122	CHU	No	51	0	0
1	0	Thoracotor	122	CHU	No	60	0	0
1	1	Thoracotor	122	CHU	No	68	0	0
1	0	Thoracotor	122	CHU	No	63	1	1
1	1	Thoracotor	122	CHU	No	62	0	0
1	0	VATS or ro	122	CHU	No	65	0	0
1	0	Thoracotor	122	CHU	No	59	0	0
1	0	Thoracotor	122	CHU	No	59	0	0
1	0	Thoracotor	122	CHU	No	61	0	1
1	0	Thoracotor	122	CHU	No	58	0	0

1	0	VATS or rc	122	CHU	No	57	0	0
1	0	Thoracotoı	122	CHU	No	58	0	0
1	0	Thoracotoı	122	CHU	No	66	0	0
1	0	Thoracotoı	122	CHU	No	72	0	0
1	0	VATS or rc	122	CHU	No	54	0	1
1	0	Thoracotoı	122	CHU	No	59	0	1
1	1	VATS or rc	122	CHU	No	73	0	0
1	0	Thoracotoı	122	CHU	No	58	0	0
1	0	VATS or rc	122	CHU	No	76	0	0
1	0	Thoracotoı	122	CHU	No	27	0	0
1	1	Thoracotoı	122	CHU	No	65	0	0
1	0	Thoracotoı	122	CHU	No	73	0	0
1	0	Thoracotoı	122	CHU	No	55	0	0
1	0	Thoracotoı	122	CHU	No	63	0	0
1	0	Thoracotoı	122	CHU	No	58	0	1
1	0	Thoracotoı	122	CHU	No	59	0	1
1	0	Thoracotoı	122	CHU	No	73	0	0
1	0	Thoracotoı	122	CHU	No	59	0	1
1	1	VATS or rc	122	CHU	No	59	0	0
1	0	Thoracotoı	122	CHU	No	73	0	0
1	0	Thoracotoı	122	CHU	No	55	0	1
1	0	Thoracotoı	122	CHU	No	70	0	0
1	0	VATS or rc	122	CHU	No	79	1	0
1	1	VATS or rc	122	CHU	No	63	0	1
1	0	VATS or rc	122	CHU	No	52	0	0
1	0	Thoracotoı	122	CHU	No	70	0	0
1	0	Thoracotoı	122	CHU	No	64	0	0
1	0	Thoracotoı	122	CHU	No	68	0	0
1	0	VATS or rc	122	CHU	No	62	0	1
1	0	VATS or rc	122	CHU	No	45	0	1
1	0	Thoracotoı	122	CHU	No	58	0	1
1	1	Thoracotoı	122	CHU	No	50	0	1
1	0	Thoracotoı	122	CHU	No	66	0	0
1	0	Thoracotoı	122	CHU	No	77	0	0
1	0	VATS or rc	122	CHU	No	74	1	0
1	1	VATS or rc	122	CHU	No	55	0	0
1	0	Thoracotoı	122	CHU	No	66	0	0
1	0	Thoracotoı	122	CHU	No	59	0	1
1	0	Thoracotoı	122	CHU	No	50	0	0
1	0	VATS or rc	122	CHU	No	64	0	0
1	0	VATS or rc	122	CHU	No	69	0	0
1	0	Thoracotoı	122	CHU	No	78	0	0
1	0	Thoracotoı	122	CHU	No	55	0	0
1	0	Thoracotoı	122	CHU	No	56	0	0
1	0	Thoracotoı	122	CHU	No	70	0	0
1	0	Thoracotoı	122	CHU	No	67	0	0
1	0	Thoracotoı	122	CHU	No	61	1	1
1	0	VATS or rc	122	CHU	No	71	0	0
1	0	Thoracotoı	122	CHU	No	66	0	0
1	0	Thoracotoı	122	CHU	No	57	0	0
1	0	Thoracotoı	122	CHU	No	59	0	0
1	0	Thoracotoı	122	CHU	No	56	0	0
1	0	Thoracotoı	122	CHU	No	72	1	0
1	1	Thoracotoı	122	CHU	No	50	0	1
1	1	Thoracotoı	122	CHU	No	63	0	1
1	0	VATS or rc	122	CHU	No	64	0	0

1	0	Thoracotor	122	CHU	No	73	0	0
1	0	Thoracotor	122	CHU	No	66	0	0
1	0	Thoracotor	122	CHU	No	53	0	0
1	0	Thoracotor	122	CHU	No	40	0	1
1	0	VATS or ro	122	CHU	No	45	0	1
1	0	VATS or ro	122	CHU	No	48	0	0
1	0	Thoracotor	122	CHU	No	65	0	0
1	0	Thoracotor	122	CHU	No	75	0	0
1	0	VATS or ro	122	CHU	No	58	0	0
1	0	VATS or ro	122	CHU	No	57	0	0
1	0	VATS or ro	122	CHU	No	73	0	0
1	0	Thoracotor	122	CHU	No	66	0	0
1	0	VATS or ro	122	CHU	No	61	0	1
1	0	Thoracotor	122	CHU	No	80	0	0
1	0	Thoracotor	122	CHU	No	80	0	0
1	0	VATS or ro	122	CHU	No	69	0	0
1	0	Thoracotor	122	CHU	No	62	0	1
1	0	VATS or ro	122	CHU	No	60	0	1
1	0	VATS or ro	122	CHU	No	71	0	0
1	0	VATS or ro	122	CHU	No	55	0	0
1	0	VATS or ro	122	CHU	No	54	0	0
1	0	Thoracotor	122	CHU	No	69	0	0
1	0	Thoracotor	122	CHU	No	74	0	1
1	0	VATS or ro	122	CHU	No	67	0	0
1	0	Thoracotor	122	CHU	No	69	0	0
1	0	Thoracotor	122	CHU	No	68	0	1
1	0	Thoracotor	122	CHU	No	64	0	0
1	0	Thoracotor	122	CHU	No	62	0	0
1	0	VATS or ro	122	CHU	No	81	0	0
1	1	Thoracotor	122	CHU	No	63	0	0
1	0	Thoracotor	122	CHU	No	72	0	0
1	0	Thoracotor	122	CHU	No	66	0	0
1	0	Thoracotor	122	CHU	No	65	0	0
1	0	Thoracotor	122	CHU	No	61	0	0
1	0	VATS or ro	122	CHU	No	50	0	0
1	0	Thoracotor	122	CHU	No	69	0	0
1	0	Thoracotor	122	CHU	No	68	0	0
1	0	Thoracotor	122	CHU	No	69	0	0
1	0	Thoracotor	122	CHU	No	76	0	0
1	0	VATS or ro	122	CHU	No	59	0	0
1	0	VATS or ro	122	CHU	No	74	0	0
1	0	VATS or ro	122	CHU	No	68	0	0
1	0	VATS or ro	122	CHU	No	77	1	0
1	0	Thoracotor	122	CHU	No	64	0	0
1	0	VATS or ro	122	CHU	No	57	1	0
1	1	Thoracotor	122	CHU	No	68	0	0
1	0	Thoracotor	122	CHU	No	69	0	0
1	1	Thoracotor	122	CHU	No	64	1	0
1	0	VATS or ro	122	CHU	No	79	0	0
1	0	Thoracotor	122	CHU	No	60	0	0
1	0	VATS or ro	122	CHU	No	73	0	0
1	0	VATS or ro	122	CHU	No	81	0	0
1	0	Thoracotor	122	CHU	No	59	0	0
1	0	Thoracotor	122	CHU	No	69	0	0
1	0	Thoracotor	122	CHU	No	52	0	1
1	0	Thoracotor	122	CHU	No	70	0	0

1	0	Thoracotor	122	CHU	No	59	0	0
1	0	VATS or rc	122	CHU	No	76	1	0
1	0	VATS or rc	122	CHU	No	77	0	0
1	1	Thoracotor	122	CHU	No	64	0	0
1	0	VATS or rc	122	CHU	No	72	0	0
1	0	Thoracotor	122	CHU	No	55	0	0
1	0	VATS or rc	122	CHU	No	50	0	0
1	0	Thoracotor	122	CHU	No	62	0	0
1	0	VATS or rc	122	CHU	No	56	0	0
1	0	Thoracotor	122	CHU	No	64	0	1
1	0	Thoracotor	122	CHU	No	71	0	1
1	0	Thoracotor	122	CHU	No	45	0	0
1	0	Thoracotor	122	CHU	No	59	0	0
1	1	Thoracotor	122	CHU	No	68	0	0
1	1	Thoracotor	122	CHU	No	69	0	0
1	0	Thoracotor	122	CHU	No	60	0	0
1	0	Thoracotor	122	CHU	No	49	0	1
1	0	Thoracotor	122	CHU	No	68	0	0
1	0	VATS or rc	122	CHU	No	55	0	0
1	0	Thoracotor	122	CHU	No	64	0	0
1	0	VATS or rc	122	CHU	No	76	0	0
1	0	Thoracotor	122	CHU	No	56	0	0
1	0	Thoracotor	122	CHU	No	80	0	0
1	0	Thoracotor	122	CHU	No	78	0	0
1	0	Thoracotor	122	CHU	No	62	0	0
1	0	VATS or rc	122	CHU	No	81	0	0
1	0	Thoracotor	122	CHU	No	67	0	0
1	0	VATS or rc	122	CHU	No	82	0	0
1	0	Thoracotor	122	CHU	No	65	0	0
1	0	VATS or rc	122	CHU	No	49	0	0
1	0	Thoracotor	122	CHU	No	68	0	0
1	0	Thoracotor	122	CHU	No	53	0	0
1	0	Thoracotor	122	CHU	No	57	0	0
1	0	Thoracotor	122	CHU	No	73	1	1
1	0	Thoracotor	122	CHU	No	68	1	1
1	0	VATS or rc	122	CHU	No	61	0	0
1	0	Thoracotor	122	CHU	No	70	0	1
1	0	VATS or rc	122	CHU	No	70	0	0
1	0	VATS or rc	122	CHU	No	74	0	0
1	0	Thoracotor	122	CHU	No	63	0	0
1	0	VATS or rc	122	CHU	No	82	0	0
1	0	Thoracotor	122	CHU	No	75	0	0
1	0	VATS or rc	122	CHU	No	76	0	0
1	0	Thoracotor	122	CHU	No	60	0	0
1	0	Thoracotor	122	CHU	No	68	0	0
1	0	Thoracotor	122	CHU	No	74	0	0
1	1	Thoracotor	122	CHU	No	66	0	0
1	0	Thoracotor	122	CHU	No	73	1	0
1	0	Thoracotor	122	CHU	No	77	0	1
1	0	VATS or rc	122	CHU	No	66	0	0
1	0	Thoracotor	122	CHU	No	89	0	0
1	0	VATS or rc	122	CHU	No	57	0	0
1	0	Thoracotor	122	CHU	No	55	0	0
1	0	VATS or rc	122	CHU	No	61	0	0
1	0	Thoracotor	122	CHU	No	72	0	0
1	0	Thoracotor	122	CHU	No	79	0	1

1	0	Thoracotoı	122	CHU	No	77	0	0
1	0	VATS or rc	122	CHU	No	44	0	1
1	0	Thoracotoı	122	CHU	No	63	0	1
1	0	VATS or rc	122	CHU	No	64	0	0
1	0	Thoracotoı	122	CHU	No	77	0	0
1	0	Thoracotoı	122	CHU	No	52	0	0
1	1	VATS or rc	122	CHU	No	51	0	0
1	0	VATS or rc	122	CHU	No	65	0	1
1	1	Thoracotoı	122	CHU	No	64	0	0
1	0	Thoracotoı	122	CHU	No	62	0	0
1	0	VATS or rc	122	CHU	No	56	0	0
0	0	Thoracotoı	75	CHG	No	79	0	0
0	0	VATS or rc	75	CHG	No	66	0	0
0	0	VATS or rc	75	CHG	No	60	0	0
0	0	VATS or rc	75	CHG	No	74	0	0
0	0	VATS or rc	75	CHG	No	64	0	1
0	0	VATS or rc	75	CHG	No	61	0	0
0	0	Thoracotoı	75	CHG	No	64	0	1
0	0	VATS or rc	75	CHG	No	64	0	0
0	0	Thoracotoı	75	CHG	No	40	0	1
0	1	VATS or rc	75	CHG	No	73	0	0
0	0	VATS or rc	75	CHG	No	65	0	0
0	0	Thoracotoı	75	CHG	No	60	0	0
0	0	VATS or rc	75	CHG	No	67	0	0
0	0	VATS or rc	75	CHG	No	72	0	0
0	1	Thoracotoı	75	CHG	No	50	0	1
0	0	VATS or rc	75	CHG	No	64	0	0
0	0	Thoracotoı	75	CHG	No	69	1	1
0	0	VATS or rc	75	CHG	No	66	0	0
0	0	VATS or rc	75	CHG	No	52	0	0
0	0	VATS or rc	75	CHG	No	58	0	0
0	0	Thoracotoı	75	CHG	No	55	0	0
0	0	Thoracotoı	75	CHG	No	63	0	0
0	0	VATS or rc	75	CHG	No	66	1	0
0	0	Thoracotoı	75	CHG	No	39	0	1
0	0	VATS or rc	75	CHG	No	65	0	0
0	0	Thoracotoı	75	CHG	No	46	0	1
0	0	VATS or rc	75	CHG	No	68	0	0
0	0	VATS or rc	75	CHG	No	54	0	0
0	1	VATS or rc	75	CHG	No	73	0	0
0	0	Thoracotoı	75	CHG	No	40	0	1
0	0	Thoracotoı	75	CHG	No	36	0	1
0	0	VATS or rc	75	CHG	No	53	0	0
0	0	VATS or rc	75	CHG	No	75	0	0
0	0	Thoracotoı	75	CHG	No	54	0	0
0	0	Thoracotoı	75	CHG	No	61	0	0
0	0	VATS or rc	75	CHG	No	58	0	0
0	0	Thoracotoı	75	CHG	No	69	0	0
0	0	VATS or rc	75	CHG	No	63	0	0
0	0	VATS or rc	75	CHG	No	67	1	0
0	0	VATS or rc	75	CHG	No	48	0	0
0	0	VATS or rc	75	CHG	No	74	0	0
0	1	Thoracotoı	75	CHG	No	50	0	1
0	0	VATS or rc	75	CHG	No	74	0	0
0	0	VATS or rc	75	CHG	No	68	0	0
0	0	VATS or rc	75	CHG	No	73	0	0

0	1	VATS or ro	75	CHG	No	52	0	0
0	0	VATS or ro	75	CHG	No	67	0	0
0	1	Thoracotoı	75	CHG	No	54	0	0
0	0	VATS or ro	75	CHG	No	53	0	0
0	0	VATS or ro	75	CHG	No	73	0	0
0	0	Thoracotoı	75	CHG	No	56	0	0
0	0	VATS or ro	75	CHG	No	47	0	0
0	0	Thoracotoı	75	CHG	No	72	0	0
0	1	Thoracotoı	75	CHG	No	68	0	0
0	0	Thoracotoı	75	CHG	No	67	0	1
0	1	VATS or ro	75	CHG	No	76	0	0
0	0	VATS or ro	75	CHG	No	79	0	0
0	0	Thoracotoı	75	CHG	No	72	0	0
0	0	VATS or ro	75	CHG	No	63	0	0
0	0	VATS or ro	75	CHG	No	72	0	0
0	0	VATS or ro	75	CHG	No	54	0	0
0	0	VATS or ro	75	CHG	No	55	0	0
0	0	Thoracotoı	75	CHG	No	71	0	0
0	0	Thoracotoı	75	CHG	No	78	1	0
0	0	VATS or ro	75	CHG	No	75	0	0
0	0	VATS or ro	75	CHG	No	71	0	0
0	0	Thoracotoı	75	CHG	No	67	0	1
0	0	Thoracotoı	75	CHG	No	70	0	1
0	0	VATS or ro	75	CHG	No	60	0	0
0	0	Thoracotoı	75	CHG	No	77	0	0
0	0	VATS or ro	75	CHG	No	76	0	0
0	0	VATS or ro	75	CHG	No	55	0	0
0	0	Thoracotoı	75	CHG	No	54	0	0
0	0	VATS or ro	75	CHG	No	77	0	0
0	0	VATS or ro	75	CHG	No	62	0	0
0	0	VATS or ro	75	CHG	No	69	0	0
0	1	VATS or ro	75	CHG	No	73	0	0
0	0	VATS or ro	75	CHG	No	64	0	0
0	1	VATS or ro	75	CHG	No	67	0	0
0	1	Thoracotoı	75	CHG	No	55	0	1
0	0	Thoracotoı	75	CHG	No	73	0	0
0	0	Thoracotoı	75	CHG	No	64	0	0
0	0	VATS or ro	75	CHG	No	66	0	0
0	0	VATS or ro	75	CHG	No	66	0	0
0	0	VATS or ro	75	CHG	No	63	0	1
0	0	VATS or ro	75	CHG	No	64	0	1
0	0	Thoracotoı	75	CHG	No	71	0	1
0	0	Thoracotoı	75	CHG	No	56	0	0
0	0	VATS or ro	75	CHG	No	65	0	0
0	0	VATS or ro	75	CHG	No	64	0	0
0	0	VATS or ro	75	CHG	No	54	0	0
0	0	VATS or ro	75	CHG	No	61	0	1
0	0	VATS or ro	75	CHG	No	60	0	0
0	0	Thoracotoı	75	CHG	No	64	0	1
0	0	VATS or ro	75	CHG	No	65	0	0
0	0	VATS or ro	75	CHG	No	60	0	0
0	0	VATS or ro	75	CHG	No	82	0	0
0	0	Thoracotoı	75	CHG	No	53	0	0
0	0	VATS or ro	75	CHG	No	69	0	1
0	0	VATS or ro	75	CHG	No	48	0	0
0	0	VATS or ro	75	CHG	No	77	0	0

0	0	Thoracotoı	75	CHG	No	75	0	0
0	0	VATS or rc	75	CHG	No	82	0	0
0	0	Thoracotoı	75	CHG	No	64	0	0
0	0	VATS or rc	75	CHG	No	68	0	0
0	0	VATS or rc	75	CHG	No	67	0	0
0	0	Thoracotoı	75	CHG	No	71	0	0
0	0	Thoracotoı	75	CHG	No	68	0	0
0	0	VATS or rc	75	CHG	No	75	1	0
0	0	VATS or rc	75	CHG	No	69	0	1
0	0	VATS or rc	75	CHG	No	72	0	0
0	0	VATS or rc	75	CHG	No	59	0	0
0	0	VATS or rc	75	CHG	No	62	0	0
0	0	VATS or rc	75	CHG	No	61	0	0
0	0	VATS or rc	75	CHG	No	67	1	1
0	0	VATS or rc	75	CHG	No	70	1	1
0	0	VATS or rc	106	CHU	No	66	0	0
0	0	VATS or rc	106	CHU	No	77	1	0
0	0	Thoracotoı	106	CHU	No	68	0	0
0	0	Thoracotoı	106	CHU	No	84	0	0
0	0	VATS or rc	106	CHU	No	70	0	0
0	0	VATS or rc	106	CHU	No	70	0	0
0	0	VATS or rc	106	CHU	No	66	0	0
0	0	VATS or rc	106	CHU	No	49	0	0
0	0	VATS or rc	106	CHU	No	66	0	0
0	0	VATS or rc	106	CHU	No	68	0	0
0	0	Thoracotoı	106	CHU	No	65	0	0
0	0	VATS or rc	106	CHU	No	67	0	0
0	0	VATS or rc	106	CHU	No	84	0	0
0	0	Thoracotoı	106	CHU	No	61	0	0
0	0	VATS or rc	106	CHU	No	70	0	0
0	0	VATS or rc	106	CHU	No	77	0	1
0	0	VATS or rc	106	CHU	No	51	0	0
0	1	Thoracotoı	106	CHU	No	37	0	0
0	0	VATS or rc	106	CHU	No	69	1	0
0	0	VATS or rc	106	CHU	No	73	0	0
0	0	VATS or rc	106	CHU	No	60	0	0
0	0	VATS or rc	106	CHU	No	66	0	0
0	0	VATS or rc	106	CHU	No	49	0	0
0	0	VATS or rc	106	CHU	No	65	0	0
0	0	VATS or rc	106	CHU	Yes	62	0	0
0	0	VATS or rc	106	CHU	No	64	0	0
0	0	VATS or rc	106	CHU	No	79	0	0
0	1	Thoracotoı	106	CHU	No	76	0	0
0	0	Thoracotoı	106	CHU	No	64	0	0
0	0	Thoracotoı	106	CHU	No	64	1	0
0	0	Thoracotoı	106	CHU	No	73	0	0
0	0	VATS or rc	106	CHU	No	69	0	0
0	0	Thoracotoı	106	CHU	No	83	0	0
0	0	VATS or rc	106	CHU	No	71	0	0
0	0	VATS or rc	106	CHU	No	66	0	0
0	0	VATS or rc	106	CHU	No	65	0	0
0	0	VATS or rc	106	CHU	No	54	0	0
0	0	VATS or rc	106	CHU	No	65	0	0
0	0	Thoracotoı	106	CHU	No	54	0	0
0	0	VATS or rc	106	CHU	No	64	0	0
0	0	VATS or rc	106	CHU	No	76	0	0

0	0	Thoracotoı	106	CHU	No	67	0	0
0	0	VATS or rc	106	CHU	No	66	0	0
0	0	VATS or rc	106	CHU	No	47	0	0
0	0	VATS or rc	106	CHU	No	77	0	0
0	1	Thoracotoı	106	CHU	No	68	0	0
0	0	VATS or rc	106	CHU	No	66	0	1
0	0	Thoracotoı	106	CHU	No	62	0	0
0	0	VATS or rc	106	CHU	No	59	0	0
0	0	VATS or rc	106	CHU	No	76	0	0
0	0	Thoracotoı	106	CHU	No	73	0	0
0	0	Thoracotoı	106	CHU	No	78	0	0
0	0	Thoracotoı	106	CHU	No	62	0	0
0	0	VATS or rc	106	CHU	No	67	0	0
0	0	VATS or rc	106	CHU	No	66	0	0
0	0	VATS or rc	106	CHU	No	77	0	0
0	0	VATS or rc	106	CHU	No	65	0	1
0	0	VATS or rc	106	CHU	No	61	1	0
0	0	VATS or rc	106	CHU	No	83	0	0
0	0	VATS or rc	106	CHU	No	82	0	0
0	1	Thoracotoı	106	CHU	No	55	0	0
0	0	VATS or rc	106	CHU	No	67	0	0
0	0	VATS or rc	106	CHU	No	49	0	0
0	0	VATS or rc	106	CHU	No	79	0	0
0	0	Thoracotoı	106	CHU	No	55	0	0
0	0	Thoracotoı	106	CHU	No	78	0	0
0	0	VATS or rc	106	CHU	No	58	0	0
0	0	VATS or rc	106	CHU	No	63	0	1
0	0	Thoracotoı	106	CHU	No	69	0	0
0	1	Thoracotoı	106	CHU	No	61	1	0
0	0	VATS or rc	106	CHU	No	73	0	0
0	0	VATS or rc	106	CHU	No	58	0	0
0	0	VATS or rc	106	CHU	No	75	0	0
0	0	Thoracotoı	106	CHU	No	81	0	0
0	0	VATS or rc	106	CHU	No	64	0	0
0	0	VATS or rc	106	CHU	No	85	0	0
0	0	VATS or rc	36	PRIVE	No	71	0	0
0	0	Thoracotoı	36	PRIVE	No	67	0	0
0	1	Thoracotoı	36	PRIVE	No	49	0	1
0	0	Thoracotoı	36	PRIVE	No	83	1	0
0	0	VATS or rc	36	PRIVE	No	59	0	0
0	0	VATS or rc	36	PRIVE	No	71	0	0
0	0	Thoracotoı	36	PRIVE	No	76	0	0
0	0	Thoracotoı	36	PRIVE	No	80	0	0
0	0	VATS or rc	40	PRIVE	No	59	0	0
0	0	Thoracotoı	40	PRIVE	No	55	0	1
0	0	VATS or rc	40	PRIVE	No	78	0	0
0	0	VATS or rc	40	PRIVE	No	81	0	0
0	0	VATS or rc	40	PRIVE	No	80	0	0
0	0	VATS or rc	40	PRIVE	No	62	0	1
0	0	VATS or rc	40	PRIVE	No	69	0	1
0	0	VATS or rc	40	PRIVE	No	70	0	0
0	0	VATS or rc	40	PRIVE	No	57	0	0
0	1	Thoracotoı	40	PRIVE	No	79	0	0
0	0	VATS or rc	40	PRIVE	No	49	0	1
0	0	VATS or rc	40	PRIVE	No	69	0	0
0	0	Thoracotoı	40	PRIVE	No	53	0	1

0	0	VATS or rc	40	PRIVE	No	54	0	1
0	0	Thoracotoı	40	PRIVE	No	73	0	0
0	0	VATS or rc	40	PRIVE	No	59	0	1
0	0	VATS or rc	40	PRIVE	No	61	0	1
0	0	VATS or rc	40	PRIVE	No	63	0	0
0	0	Thoracotoı	40	PRIVE	No	64	0	1
0	0	VATS or rc	40	PRIVE	No	68	0	1
0	0	VATS or rc	40	PRIVE	No	65	0	1
0	0	VATS or rc	40	PRIVE	No	72	0	1
0	1	VATS or rc	40	PRIVE	No	74	0	0
0	0	VATS or rc	40	PRIVE	No	55	0	0
0	0	Thoracotoı	40	PRIVE	No	80	0	1
0	0	VATS or rc	40	PRIVE	No	72	0	0
0	0	VATS or rc	40	PRIVE	No	77	0	0
0	0	VATS or rc	40	PRIVE	No	63	0	1
0	0	VATS or rc	40	PRIVE	No	65	0	0
0	0	VATS or rc	40	PRIVE	No	78	0	0
0	0	VATS or rc	40	PRIVE	No	63	0	0
0	0	VATS or rc	40	PRIVE	No	74	0	0
0	0	VATS or rc	40	PRIVE	Yes	78	0	0
0	0	VATS or rc	40	PRIVE	No	68	0	0
0	0	VATS or rc	40	PRIVE	No	87	0	0
0	0	VATS or rc	40	PRIVE	No	74	0	0
0	0	VATS or rc	40	PRIVE	No	63	0	0
0	0	VATS or rc	40	PRIVE	No	68	0	0
0	0	VATS or rc	40	PRIVE	No	65	0	0
0	0	VATS or rc	40	PRIVE	No	77	0	0
0	0	VATS or rc	40	PRIVE	No	57	0	0
0	0	VATS or rc	40	PRIVE	No	63	0	1
0	0	VATS or rc	40	PRIVE	No	52	0	0
0	0	Thoracotoı	55	PRIVE	No	72	0	1
0	0	VATS or rc	55	PRIVE	No	86	0	0
0	0	Thoracotoı	55	PRIVE	No	57	1	1
0	0	Thoracotoı	55	PRIVE	No	65	1	1
0	0	Thoracotoı	55	PRIVE	No	61	0	0
0	0	Thoracotoı	55	PRIVE	No	42	0	1
0	0	VATS or rc	55	PRIVE	No	82	0	0
0	0	Thoracotoı	55	PRIVE	No	75	0	1
0	0	Thoracotoı	55	PRIVE	No	55	0	1
0	0	VATS or rc	55	PRIVE	No	71	0	0
0	0	VATS or rc	55	PRIVE	No	72	0	0
0	0	Thoracotoı	55	PRIVE	No	63	0	1
0	0	VATS or rc	55	PRIVE	No	74	0	0
0	0	VATS or rc	55	PRIVE	No	71	0	1
0	0	Thoracotoı	55	PRIVE	No	82	0	1
0	0	VATS or rc	55	PRIVE	No	43	0	1
0	0	VATS or rc	55	PRIVE	No	70	0	0
0	0	Thoracotoı	55	PRIVE	No	49	0	0
0	0	Thoracotoı	55	PRIVE	No	57	0	0
0	0	VATS or rc	55	PRIVE	No	68	0	0
0	1	Thoracotoı	55	PRIVE	No	64	0	1
0	0	VATS or rc	55	PRIVE	No	65	1	0
0	0	Thoracotoı	55	PRIVE	No	69	0	1
0	0	Thoracotoı	55	PRIVE	No	74	0	0
0	0	Thoracotoı	55	PRIVE	No	77	0	0
0	0	Thoracotoı	55	PRIVE	No	66	0	1

0	0	Thoracotoı	55	PRIVE	No	65	0	0
0	0	VATS or rc	55	PRIVE	No	75	1	0
0	1	VATS or rc	55	PRIVE	No	73	0	0
0	0	Thoracotoı	55	PRIVE	No	63	0	0
0	0	Thoracotoı	55	PRIVE	No	77	0	0
0	0	VATS or rc	55	PRIVE	No	57	0	1
0	0	VATS or rc	55	PRIVE	No	71	1	0
0	0	Thoracotoı	55	PRIVE	No	57	0	1
0	0	Thoracotoı	55	PRIVE	No	72	0	0
0	0	VATS or rc	55	PRIVE	No	53	0	1
0	0	Thoracotoı	55	PRIVE	No	56	1	1
0	0	Thoracotoı	55	PRIVE	No	54	0	1
0	0	Thoracotoı	55	PRIVE	No	66	0	0
0	0	Thoracotoı	55	PRIVE	No	62	0	0
0	0	VATS or rc	55	PRIVE	No	73	0	0
0	0	VATS or rc	55	PRIVE	No	64	0	0
0	0	Thoracotoı	55	PRIVE	No	71	0	0
0	0	Thoracotoı	55	PRIVE	No	55	0	1
0	0	Thoracotoı	55	PRIVE	No	73	0	0
0	0	Thoracotoı	55	PRIVE	No	68	1	0
0	0	Thoracotoı	55	PRIVE	No	61	0	1
0	0	Thoracotoı	55	PRIVE	No	56	0	0
0	0	VATS or rc	55	PRIVE	No	78	0	0
0	0	VATS or rc	55	PRIVE	No	77	0	0
0	0	Thoracotoı	55	PRIVE	No	82	0	1
0	0	VATS or rc	55	PRIVE	No	67	0	0
0	0	Thoracotoı	55	PRIVE	No	62	1	0
0	0	Thoracotoı	55	PRIVE	No	70	0	1
0	0	Thoracotoı	55	PRIVE	No	59	0	1
0	0	VATS or rc	55	PRIVE	No	63	0	0
0	0	Thoracotoı	55	PRIVE	No	62	1	0
0	0	VATS or rc	55	PRIVE	No	37	0	1
0	0	Thoracotoı	55	PRIVE	No	66	0	0
0	0	Thoracotoı	55	PRIVE	No	56	0	1
0	0	VATS or rc	55	PRIVE	No	56	0	1
0	0	Thoracotoı	55	PRIVE	No	70	0	0
0	0	Thoracotoı	55	PRIVE	No	57	0	1
0	0	Thoracotoı	55	PRIVE	No	62	0	0
0	0	Thoracotoı	55	PRIVE	No	70	0	1
0	0	VATS or rc	55	PRIVE	No	43	1	0
0	0	VATS or rc	55	PRIVE	No	64	0	0
0	0	Thoracotoı	55	PRIVE	No	66	0	1
0	0	Thoracotoı	55	PRIVE	No	66	0	1
0	0	Thoracotoı	55	PRIVE	No	82	0	0
0	1	Thoracotoı	55	PRIVE	No	49	0	0
0	0	Thoracotoı	55	PRIVE	No	84	0	0
0	0	Thoracotoı	55	PRIVE	No	58	0	0
0	0	Thoracotoı	55	PRIVE	No	64	0	0
0	1	Thoracotoı	55	PRIVE	No	81	0	0
0	0	Thoracotoı	55	PRIVE	No	54	0	0
0	0	VATS or rc	55	PRIVE	No	85	0	0
0	0	Thoracotoı	55	PRIVE	No	79	0	1
0	0	VATS or rc	55	PRIVE	No	79	0	0
0	0	VATS or rc	55	PRIVE	No	64	0	0
0	0	Thoracotoı	55	PRIVE	No	70	0	0
0	0	VATS or rc	55	PRIVE	No	73	0	0

0	0	Thoracotoı	55	PRIVE	No	74	0	0
0	0	Thoracotoı	55	PRIVE	No	60	1	1
0	0	Thoracotoı	55	PRIVE	No	79	0	0
0	0	Thoracotoı	55	PRIVE	No	58	0	1
0	0	VATS or rc	55	PRIVE	No	58	0	0
0	0	Thoracotoı	55	PRIVE	No	83	0	0
0	0	VATS or rc	55	PRIVE	No	72	0	0
0	0	Thoracotoı	55	PRIVE	No	77	0	0
0	0	Thoracotoı	55	PRIVE	No	68	0	0
0	0	Thoracotoı	55	PRIVE	No	62	0	0
0	0	Thoracotoı	55	PRIVE	No	60	0	1
0	0	Thoracotoı	55	PRIVE	No	68	0	0
0	0	VATS or rc	55	PRIVE	No	76	0	0
0	0	VATS or rc	55	PRIVE	No	72	0	0
0	0	Thoracotoı	55	PRIVE	No	59	0	1
0	0	VATS or rc	55	PRIVE	No	66	0	0
0	0	Thoracotoı	55	PRIVE	No	59	0	1
0	0	Thoracotoı	55	PRIVE	No	71	0	0
0	0	Thoracotoı	55	PRIVE	No	75	0	0
0	0	Thoracotoı	55	PRIVE	No	61	0	1
0	0	VATS or rc	55	PRIVE	No	78	1	0
1	0	VATS or rc	77	PRIVE	No	70	0	0
1	0	VATS or rc	77	PRIVE	No	56	0	0
1	0	Thoracotoı	77	PRIVE	No	71	0	0
1	0	Thoracotoı	77	PRIVE	No	70	1	0
1	0	Thoracotoı	77	PRIVE	No	66	1	0
1	0	Thoracotoı	77	PRIVE	No	70	0	0
1	0	Thoracotoı	77	PRIVE	No	64	0	0
1	0	Thoracotoı	77	PRIVE	No	78	0	0
1	0	Thoracotoı	77	PRIVE	No	44	0	1
1	0	Thoracotoı	77	PRIVE	No	56	0	0
1	0	Thoracotoı	77	PRIVE	No	61	0	1
1	0	VATS or rc	77	PRIVE	No	84	0	0
1	0	VATS or rc	77	PRIVE	No	72	0	0
1	0	Thoracotoı	77	PRIVE	No	72	0	0
1	0	VATS or rc	77	PRIVE	No	75	0	0
1	0	VATS or rc	77	PRIVE	No	65	0	0
1	0	VATS or rc	77	PRIVE	No	59	1	0
1	0	Thoracotoı	77	PRIVE	No	82	1	0
1	0	Thoracotoı	77	PRIVE	No	85	0	0
1	0	VATS or rc	77	PRIVE	No	83	0	0
1	0	VATS or rc	77	PRIVE	No	59	0	1
1	0	Thoracotoı	77	PRIVE	No	78	0	0
1	0	Thoracotoı	77	PRIVE	No	66	0	0
1	0	VATS or rc	77	PRIVE	No	64	0	1
1	0	Thoracotoı	77	PRIVE	No	59	0	0
1	0	VATS or rc	77	PRIVE	No	61	0	1
1	0	VATS or rc	77	PRIVE	No	75	0	0
1	0	VATS or rc	77	PRIVE	No	75	0	0
1	0	VATS or rc	77	PRIVE	No	69	0	1
1	0	Thoracotoı	77	PRIVE	No	67	0	0
1	0	Thoracotoı	77	PRIVE	No	75	0	0
1	0	VATS or rc	77	PRIVE	No	69	0	0
1	0	VATS or rc	77	PRIVE	No	60	0	0
1	0	Thoracotoı	77	PRIVE	No	76	0	0
1	0	Thoracotoı	77	PRIVE	No	78	0	0

1	0	VATS or rc	77	PRIVE	No	50	0	0
1	0	VATS or rc	77	PRIVE	No	71	0	0
1	0	Thoracotoı	77	PRIVE	No	73	0	0
1	0	VATS or rc	77	PRIVE	No	53	0	1
1	0	VATS or rc	77	PRIVE	No	83	0	0
1	0	VATS or rc	77	PRIVE	No	72	0	0
1	1	Thoracotoı	77	PRIVE	No	64	0	0
1	0	Thoracotoı	77	PRIVE	No	68	0	0
1	1	Thoracotoı	77	PRIVE	No	63	0	1
1	0	Thoracotoı	77	PRIVE	No	72	0	0
1	0	VATS or rc	77	PRIVE	No	66	0	0
1	0	Thoracotoı	77	PRIVE	No	51	0	0
1	0	VATS or rc	77	PRIVE	No	62	0	0
1	0	VATS or rc	77	PRIVE	No	74	0	0
1	0	VATS or rc	77	PRIVE	No	67	0	0
1	0	VATS or rc	77	PRIVE	No	65	0	0
1	0	VATS or rc	77	PRIVE	No	65	0	0
1	0	Thoracotoı	77	PRIVE	No	58	0	0
1	0	VATS or rc	77	PRIVE	No	61	0	0
1	0	VATS or rc	77	PRIVE	No	53	0	1
1	0	VATS or rc	77	PRIVE	No	61	0	0
1	0	VATS or rc	77	PRIVE	No	65	0	0
1	0	VATS or rc	77	PRIVE	No	60	0	1
1	0	VATS or rc	77	PRIVE	No	55	0	0
1	0	Thoracotoı	77	PRIVE	No	56	0	0
1	0	Thoracotoı	77	PRIVE	No	64	0	0
1	0	Thoracotoı	77	PRIVE	No	64	0	0
1	0	VATS or rc	77	PRIVE	No	69	0	0
1	0	VATS or rc	77	PRIVE	No	69	0	0
1	0	VATS or rc	77	PRIVE	No	77	0	0
1	0	VATS or rc	77	PRIVE	No	55	0	0
1	0	VATS or rc	77	PRIVE	No	69	0	1
1	0	Thoracotoı	77	PRIVE	No	69	1	0
1	0	Thoracotoı	77	PRIVE	No	59	0	0
1	0	Thoracotoı	77	PRIVE	No	60	0	0
1	0	Thoracotoı	77	PRIVE	No	71	0	0
1	0	VATS or rc	77	PRIVE	No	64	0	0
1	0	Thoracotoı	77	PRIVE	No	45	0	0
1	0	Thoracotoı	77	PRIVE	No	78	0	0
1	1	Thoracotoı	77	PRIVE	No	57	0	1
1	0	Thoracotoı	77	PRIVE	No	51	0	0
1	0	VATS or rc	77	PRIVE	No	63	0	0
1	0	VATS or rc	77	PRIVE	No	61	0	0
1	1	Thoracotoı	77	PRIVE	No	59	0	0
1	0	VATS or rc	77	PRIVE	No	77	0	0
1	0	Thoracotoı	77	PRIVE	No	77	0	0
1	0	Thoracotoı	77	PRIVE	No	64	0	0
1	0	Thoracotoı	77	PRIVE	No	77	0	0
1	0	Thoracotoı	77	PRIVE	No	45	0	0
1	0	Thoracotoı	77	PRIVE	No	66	1	0
1	0	VATS or rc	77	PRIVE	No	62	0	0
1	0	Thoracotoı	77	PRIVE	No	72	0	0
1	0	Thoracotoı	77	PRIVE	No	77	0	0
1	0	VATS or rc	77	PRIVE	No	80	0	0
1	0	Thoracotoı	77	PRIVE	No	69	0	0
1	0	Thoracotoı	77	PRIVE	No	63	0	0

1	0	VATS or ro	77	PRIVE	No	66	1	0
1	0	Thoracotoı	77	PRIVE	No	64	0	0
1	0	Thoracotoı	77	PRIVE	No	51	0	0
1	0	Thoracotoı	77	PRIVE	No	59	0	0
1	0	VATS or ro	77	PRIVE	No	65	1	0
1	0	Thoracotoı	77	PRIVE	No	56	0	0
1	1	Thoracotoı	77	PRIVE	No	77	0	0
1	0	Thoracotoı	77	PRIVE	No	65	0	0
1	0	Thoracotoı	77	PRIVE	No	51	0	0
1	0	VATS or ro	77	PRIVE	No	68	0	0
1	0	Thoracotoı	77	PRIVE	No	74	0	0
1	0	Thoracotoı	77	PRIVE	Yes	54	0	0
1	0	VATS or ro	77	PRIVE	No	76	0	0
1	0	VATS or ro	77	PRIVE	No	79	0	1
1	0	Thoracotoı	77	PRIVE	No	65	0	0
1	0	VATS or ro	77	PRIVE	No	68	0	0
1	0	VATS or ro	77	PRIVE	No	75	0	0
1	1	Thoracotoı	77	PRIVE	No	64	0	0
1	0	VATS or ro	77	PRIVE	No	61	0	1
1	0	VATS or ro	77	PRIVE	No	77	0	0
1	0	Thoracotoı	77	PRIVE	No	54	0	0
1	0	VATS or ro	77	PRIVE	No	81	0	0
1	0	Thoracotoı	77	PRIVE	No	72	0	0
1	0	VATS or ro	77	PRIVE	No	70	0	0
1	0	VATS or ro	77	PRIVE	No	70	0	0
1	0	VATS or ro	77	PRIVE	No	71	0	0
1	0	Thoracotoı	77	PRIVE	No	61	0	1
1	0	Thoracotoı	77	PRIVE	No	67	0	1
1	0	VATS or ro	77	PRIVE	No	84	1	0
1	0	Thoracotoı	77	PRIVE	No	64	0	0
1	1	Thoracotoı	77	PRIVE	No	59	0	0
1	0	Thoracotoı	77	PRIVE	No	68	0	0
1	0	VATS or ro	77	PRIVE	No	75	0	0
1	0	Thoracotoı	77	PRIVE	No	72	0	0
1	1	Thoracotoı	77	PRIVE	No	74	1	0
1	0	VATS or ro	77	PRIVE	No	78	0	0
1	1	Thoracotoı	77	PRIVE	No	70	0	0
1	0	VATS or ro	250	CHU	No	52	0	0
1	0	Thoracotoı	250	CHU	No	78	0	0
1	0	VATS or ro	250	CHU	No	58	0	0
1	0	Thoracotoı	250	CHU	No	61	0	0
1	0	Thoracotoı	250	CHU	No	59	0	0
1	0	Thoracotoı	250	CHU	No	54	0	0
1	0	Thoracotoı	250	CHU	No	81	0	0
1	0	VATS or ro	250	CHU	No	81	0	0
1	0	Thoracotoı	250	CHU	No	59	0	0
1	0	Thoracotoı	250	CHU	No	61	0	0
1	0	Thoracotoı	250	CHU	No	60	0	1
1	0	Thoracotoı	250	CHU	No	46	0	0
1	0	Thoracotoı	250	CHU	No	57	0	0
1	0	Thoracotoı	250	CHU	No	61	0	0
1	0	Thoracotoı	250	CHU	No	79	0	0
1	0	VATS or ro	250	CHU	No	64	0	0
1	0	Thoracotoı	250	CHU	No	60	0	0
1	0	Thoracotoı	250	CHU	No	67	0	1
1	0	Thoracotoı	250	CHU	No	65	0	1

1	1	Thoracotoı	250	CHU	No	57	0	1
1	0	Thoracotoı	250	CHU	Yes	54	0	1
1	1	Thoracotoı	250	CHU	No	42	0	0
1	0	Thoracotoı	250	CHU	No	62	0	0
1	0	Thoracotoı	250	CHU	No	53	0	0
1	0	Thoracotoı	250	CHU	No	64	0	0
1	0	VATS or rc	250	CHU	No	70	0	0
1	0	Thoracotoı	250	CHU	No	57	0	0
1	1	Thoracotoı	250	CHU	No	49	0	0
1	0	Thoracotoı	250	CHU	No	82	0	0
1	0	VATS or rc	250	CHU	No	56	0	0
1	0	Thoracotoı	250	CHU	No	60	0	1
1	0	Thoracotoı	250	CHU	No	73	0	0
1	1	Thoracotoı	250	CHU	No	79	0	0
1	0	Thoracotoı	250	CHU	No	83	1	0
1	0	VATS or rc	250	CHU	No	60	0	1
1	0	Thoracotoı	250	CHU	No	55	0	1
1	0	Thoracotoı	250	CHU	No	68	0	0
1	0	Thoracotoı	250	CHU	No	60	0	0
1	0	Thoracotoı	250	CHU	No	72	0	1
1	0	Thoracotoı	250	CHU	No	72	0	1
1	1	Thoracotoı	250	CHU	No	66	0	0
1	0	Thoracotoı	250	CHU	No	60	0	0
1	0	Thoracotoı	250	CHU	No	67	0	0
1	0	VATS or rc	250	CHU	No	77	0	0
1	0	Thoracotoı	250	CHU	No	72	0	0
1	0	Thoracotoı	250	CHU	No	56	0	0
1	0	VATS or rc	250	CHU	No	75	0	0
1	0	VATS or rc	250	CHU	No	60	0	0
1	0	VATS or rc	250	CHU	No	66	0	1
1	0	Thoracotoı	250	CHU	No	69	0	0
1	0	Thoracotoı	250	CHU	No	65	0	1
1	0	Thoracotoı	250	CHU	No	71	0	1
1	1	Thoracotoı	250	CHU	No	61	0	0
1	0	Thoracotoı	250	CHU	No	57	0	1
1	0	Thoracotoı	250	CHU	No	60	0	0
1	0	Thoracotoı	250	CHU	No	60	0	0
1	0	Thoracotoı	250	CHU	No	60	0	1
1	1	Thoracotoı	250	CHU	No	53	0	1
1	1	Thoracotoı	250	CHU	No	69	0	0
1	0	Thoracotoı	250	CHU	No	56	0	0
1	0	Thoracotoı	250	CHU	No	55	0	0
1	0	Thoracotoı	250	CHU	No	38	0	1
1	0	Thoracotoı	250	CHU	No	69	0	0
1	0	Thoracotoı	250	CHU	No	61	0	0
1	0	Thoracotoı	250	CHU	No	62	0	1
1	0	Thoracotoı	250	CHU	No	70	0	0
1	0	VATS or rc	250	CHU	No	81	0	0
1	0	VATS or rc	250	CHU	No	73	0	0
1	1	Thoracotoı	250	CHU	No	26	0	0
1	0	VATS or rc	250	CHU	No	60	0	0
1	0	Thoracotoı	250	CHU	No	78	0	0
1	0	Thoracotoı	250	CHU	No	68	0	0
1	0	Thoracotoı	250	CHU	No	59	0	0
1	0	VATS or rc	250	CHU	No	78	0	0
1	0	Thoracotoı	250	CHU	No	69	0	0

1	0	Thoracotoı	250	CHU	No	50	0	0
1	1	Thoracotoı	250	CHU	No	58	0	1
1	0	VATS or rc	250	CHU	No	56	0	1
1	0	Thoracotoı	250	CHU	No	58	0	0
1	0	Thoracotoı	250	CHU	No	57	0	1
1	0	VATS or rc	250	CHU	No	59	0	0
1	0	VATS or rc	250	CHU	No	53	0	0
1	0	VATS or rc	250	CHU	No	73	1	0
1	0	Thoracotoı	250	CHU	No	72	0	0
1	0	Thoracotoı	250	CHU	No	68	0	0
1	0	Thoracotoı	250	CHU	No	76	0	0
1	0	VATS or rc	250	CHU	No	76	0	0
1	0	Thoracotoı	250	CHU	No	58	0	1
1	0	Thoracotoı	250	CHU	No	70	1	0
1	0	VATS or rc	250	CHU	No	60	0	0
1	0	Thoracotoı	250	CHU	No	68	1	0
1	0	Thoracotoı	250	CHU	No	53	0	0
1	0	Thoracotoı	250	CHU	No	68	0	0
1	0	VATS or rc	250	CHU	No	72	0	0
1	0	Thoracotoı	250	CHU	No	67	0	0
1	0	Thoracotoı	250	CHU	No	62	0	0
1	0	Thoracotoı	250	CHU	No	75	0	0
1	0	VATS or rc	250	CHU	No	68	0	0
1	1	Thoracotoı	250	CHU	No	52	0	0
1	0	VATS or rc	250	CHU	No	64	0	0
1	0	Thoracotoı	250	CHU	No	76	0	0
1	0	Thoracotoı	250	CHU	No	48	0	0
1	0	Thoracotoı	250	CHU	No	56	0	1
1	0	Thoracotoı	250	CHU	No	49	0	0
1	1	Thoracotoı	250	CHU	No	74	1	0
1	0	Thoracotoı	250	CHU	No	63	0	0
1	1	Thoracotoı	250	CHU	No	62	0	0
1	0	Thoracotoı	250	CHU	No	65	0	0
1	0	Thoracotoı	250	CHU	No	69	1	0
1	1	Thoracotoı	250	CHU	No	54	0	0
1	0	VATS or rc	250	CHU	No	71	0	0
1	0	VATS or rc	250	CHU	No	63	0	1
1	0	Thoracotoı	250	CHU	No	56	0	0
1	0	Thoracotoı	250	CHU	No	55	0	0
1	0	Thoracotoı	250	CHU	No	74	0	1
1	0	VATS or rc	250	CHU	No	59	0	1
1	0	VATS or rc	250	CHU	No	59	0	0
1	0	Thoracotoı	250	CHU	No	63	0	1
1	0	Thoracotoı	250	CHU	No	60	0	0
1	0	Thoracotoı	250	CHU	No	48	0	0
1	0	Thoracotoı	250	CHU	No	53	0	1
1	0	VATS or rc	250	CHU	No	70	0	0
1	0	Thoracotoı	250	CHU	No	62	0	0
1	0	Thoracotoı	250	CHU	No	64	0	0
1	1	Thoracotoı	250	CHU	No	68	0	0
1	0	Thoracotoı	250	CHU	No	72	0	0
1	0	VATS or rc	250	CHU	No	73	0	1
1	0	Thoracotoı	250	CHU	No	78	0	1
1	0	VATS or rc	250	CHU	No	79	0	0
1	0	Thoracotoı	250	CHU	No	58	0	1
1	0	Thoracotoı	250	CHU	No	69	0	0

1	0	Thoracotoı	250	CHU	No	58	0	0
1	0	Thoracotoı	250	CHU	No	43	0	1
1	0	Thoracotoı	250	CHU	No	79	0	0
1	0	VATS or rc	250	CHU	No	78	0	0
1	0	Thoracotoı	250	CHU	No	71	0	0
1	1	Thoracotoı	250	CHU	No	61	1	0
1	0	Thoracotoı	250	CHU	No	52	1	0
1	1	Thoracotoı	250	CHU	No	60	0	1
1	0	Thoracotoı	250	CHU	No	54	0	0
1	0	Thoracotoı	250	CHU	No	61	0	0
1	0	Thoracotoı	250	CHU	No	72	0	1
1	1	Thoracotoı	250	CHU	No	55	0	1
1	0	Thoracotoı	250	CHU	No	71	0	0
1	0	Thoracotoı	250	CHU	No	49	0	0
1	0	Thoracotoı	250	CHU	No	73	0	0
1	1	Thoracotoı	250	CHU	No	47	0	1
1	0	Thoracotoı	250	CHU	No	61	0	0
1	0	Thoracotoı	250	CHU	No	80	0	0
1	0	Thoracotoı	250	CHU	No	56	0	1
1	0	Thoracotoı	250	CHU	No	54	0	0
1	1	Thoracotoı	250	CHU	No	52	0	0
1	0	Thoracotoı	250	CHU	No	55	0	0
1	0	Thoracotoı	250	CHU	No	84	0	0
1	0	VATS or rc	250	CHU	No	48	0	0
1	0	Thoracotoı	250	CHU	No	63	0	0
1	1	Thoracotoı	250	CHU	No	56	1	0
1	0	Thoracotoı	250	CHU	No	65	0	1
1	0	VATS or rc	250	CHU	No	55	0	1
1	1	Thoracotoı	250	CHU	No	68	0	1
1	0	Thoracotoı	250	CHU	No	70	0	0
1	0	VATS or rc	250	CHU	No	74	1	0
1	0	Thoracotoı	250	CHU	No	58	0	0
1	0	VATS or rc	250	CHU	No	69	0	1
1	0	Thoracotoı	250	CHU	No	74	0	0
1	0	VATS or rc	250	CHU	No	61	0	0
1	0	Thoracotoı	250	CHU	No	63	0	0
1	0	VATS or rc	250	CHU	No	68	0	0
1	0	Thoracotoı	250	CHU	No	61	0	0
1	0	VATS or rc	250	CHU	No	73	0	0
1	0	Thoracotoı	250	CHU	No	65	0	0
1	0	Thoracotoı	250	CHU	No	59	0	0
1	0	Thoracotoı	250	CHU	No	64	0	0
1	0	Thoracotoı	250	CHU	No	56	0	0
1	0	Thoracotoı	250	CHU	No	58	0	0
1	0	VATS or rc	250	CHU	No	75	0	0
1	1	Thoracotoı	250	CHU	No	61	0	1
1	0	Thoracotoı	250	CHU	No	65	0	0
1	0	Thoracotoı	250	CHU	No	62	0	0
1	0	VATS or rc	250	CHU	No	50	0	0
1	0	Thoracotoı	250	CHU	No	68	0	0
1	0	Thoracotoı	250	CHU	No	27	0	0
1	0	VATS or rc	250	CHU	No	66	0	0
1	0	Thoracotoı	250	CHU	No	81	0	0
1	0	Thoracotoı	250	CHU	No	76	0	0
1	0	VATS or rc	250	CHU	No	78	0	0
1	0	Thoracotoı	250	CHU	No	68	0	0

1	0	VATS or rc	250	CHU	No	55	0	0
1	0	VATS or rc	250	CHU	No	58	0	0
1	0	Thoracotor	250	CHU	No	75	0	0
1	0	Thoracotor	250	CHU	No	70	1	1
1	0	VATS or rc	250	CHU	No	55	0	1
1	1	Thoracotor	250	CHU	No	68	0	1
1	0	VATS or rc	250	CHU	No	70	0	0
1	0	Thoracotor	250	CHU	No	57	0	1
1	0	VATS or rc	250	CHU	No	58	1	0
1	0	VATS or rc	250	CHU	No	69	0	1
1	0	Thoracotor	250	CHU	No	59	0	0
1	0	Thoracotor	250	CHU	No	82	0	0
1	0	Thoracotor	250	CHU	No	75	0	0
1	0	Thoracotor	250	CHU	No	76	0	0
1	0	Thoracotor	250	CHU	No	81	0	0
1	0	VATS or rc	250	CHU	No	73	0	0
1	0	Thoracotor	250	CHU	No	58	0	0
1	0	Thoracotor	250	CHU	No	76	0	0
1	0	VATS or rc	250	CHU	No	68	0	0
1	0	VATS or rc	250	CHU	No	72	0	0
1	0	Thoracotor	250	CHU	No	68	0	0
1	0	VATS or rc	250	CHU	No	38	0	0
1	0	Thoracotor	250	CHU	No	44	0	1
1	0	Thoracotor	250	CHU	No	69	0	1
1	0	Thoracotor	250	CHU	No	62	0	0
1	0	VATS or rc	250	CHU	No	68	0	0
1	1	Thoracotor	250	CHU	No	68	0	0
1	0	Thoracotor	250	CHU	No	76	0	1
1	0	Thoracotor	250	CHU	No	74	0	0
1	0	Thoracotor	250	CHU	No	70	0	0
1	0	Thoracotor	250	CHU	No	65	0	1
1	1	Thoracotor	250	CHU	No	57	0	0
1	0	Thoracotor	250	CHU	No	76	0	0
1	0	Thoracotor	250	CHU	No	49	0	0
1	0	Thoracotor	250	CHU	No	68	0	0
1	0	Thoracotor	250	CHU	No	74	0	1
1	0	VATS or rc	250	CHU	No	73	1	0
1	0	Thoracotor	250	CHU	No	72	0	0
1	0	Thoracotor	250	CHU	No	54	0	0
1	0	Thoracotor	250	CHU	No	57	1	0
1	0	Thoracotor	250	CHU	No	46	0	0
1	0	VATS or rc	250	CHU	No	70	0	0
1	0	Thoracotor	250	CHU	No	73	0	0
1	0	Thoracotor	250	CHU	No	69	0	0
1	0	Thoracotor	250	CHU	No	56	0	0
1	0	Thoracotor	250	CHU	No	67	0	0
1	0	Thoracotor	250	CHU	No	55	0	0
1	0	Thoracotor	250	CHU	No	69	0	0
1	0	Thoracotor	250	CHU	No	66	0	0
1	0	VATS or rc	250	CHU	No	60	0	1
1	0	Thoracotor	250	CHU	No	71	0	0
1	0	VATS or rc	250	CHU	No	59	0	0
1	0	Thoracotor	250	CHU	No	67	0	0
1	0	Thoracotor	250	CHU	No	60	1	0
1	0	VATS or rc	250	CHU	No	56	0	0
1	0	Thoracotor	250	CHU	No	63	0	0

1	0	VATS or ro	250	CHU	No	47	0	1
1	0	Thoracotor	250	CHU	No	55	0	0
1	0	Thoracotor	250	CHU	No	59	1	0
1	0	Thoracotor	250	CHU	No	76	1	0
1	0	Thoracotor	250	CHU	No	66	0	0
1	0	VATS or ro	250	CHU	No	82	0	0
1	0	Thoracotor	250	CHU	No	60	0	1
1	0	VATS or ro	250	CHU	No	71	0	1
1	1	Thoracotor	250	CHU	No	60	0	0
1	0	Thoracotor	250	CHU	No	68	0	0
1	1	Thoracotor	250	CHU	No	63	0	0
1	0	Thoracotor	250	CHU	No	59	0	0
1	0	Thoracotor	250	CHU	Yes	59	0	0
1	0	Thoracotor	250	CHU	No	61	0	0
1	0	Thoracotor	250	CHU	No	56	0	0
1	0	Thoracotor	250	CHU	No	70	0	0
1	0	VATS or ro	250	CHU	No	76	0	0
1	1	Thoracotor	250	CHU	No	58	0	0
1	0	VATS or ro	250	CHU	No	63	0	1
1	0	Thoracotor	250	CHU	No	81	0	0
1	0	Thoracotor	250	CHU	No	55	0	1
1	0	Thoracotor	250	CHU	No	55	1	0
1	0	VATS or ro	250	CHU	No	61	0	0
1	0	Thoracotor	250	CHU	No	81	0	0
1	0	VATS or ro	250	CHU	No	57	0	0
1	1	Thoracotor	250	CHU	No	63	0	1
1	1	Thoracotor	250	CHU	Yes	55	0	1
1	0	VATS or ro	250	CHU	No	64	0	1
1	0	Thoracotor	250	CHU	No	53	0	0
1	0	Thoracotor	250	CHU	No	59	0	1
1	0	Thoracotor	250	CHU	No	69	0	0
1	1	Thoracotor	250	CHU	No	73	0	0
1	0	VATS or ro	250	CHU	No	62	0	0
1	0	VATS or ro	250	CHU	No	67	0	1
1	0	Thoracotor	250	CHU	No	67	0	0
1	0	Thoracotor	250	CHU	No	66	0	0
1	0	Thoracotor	250	CHU	No	69	0	0
1	0	Thoracotor	250	CHU	No	64	0	1
1	0	Thoracotor	250	CHU	Yes	71	0	1
1	1	Thoracotor	250	CHU	No	60	0	0
1	0	Thoracotor	250	CHU	No	58	0	0
1	0	Thoracotor	250	CHU	No	49	0	0
1	0	Thoracotor	250	CHU	No	56	0	0
1	0	Thoracotor	250	CHU	No	45	0	0
1	0	Thoracotor	250	CHU	Yes	56	0	0
1	0	Thoracotor	250	CHU	No	77	1	0
1	0	Thoracotor	250	CHU	No	41	0	0
1	0	Thoracotor	250	CHU	No	74	0	0
1	0	Thoracotor	250	CHU	No	50	0	1
1	0	Thoracotor	250	CHU	No	61	0	0
1	1	Thoracotor	250	CHU	No	63	0	1
1	0	VATS or ro	250	CHU	No	62	0	1
1	0	Thoracotor	250	CHU	No	49	0	0
1	0	Thoracotor	250	CHU	No	48	0	0
1	0	Thoracotor	250	CHU	No	76	0	1
1	0	Thoracotor	250	CHU	No	60	0	0

1	0	VATS or ro	250	CHU	No	68	0	0
1	1	Thoracotoı	250	CHU	No	74	0	0
1	0	Thoracotoı	250	CHU	No	48	0	0
1	0	Thoracotoı	250	CHU	No	76	0	0
1	1	Thoracotoı	250	CHU	No	86	0	1
1	0	Thoracotoı	250	CHU	No	68	0	0
1	0	Thoracotoı	250	CHU	No	77	1	0
1	0	Thoracotoı	250	CHU	No	57	0	1
1	0	Thoracotoı	250	CHU	No	83	0	0
1	1	Thoracotoı	250	CHU	No	68	0	1
1	0	VATS or ro	250	CHU	No	52	0	0
1	1	Thoracotoı	250	CHU	No	66	0	1
1	0	Thoracotoı	250	CHU	No	60	0	1
1	0	Thoracotoı	250	CHU	No	50	0	1
1	0	Thoracotoı	250	CHU	No	61	0	0
1	0	VATS or ro	250	CHU	No	56	0	0
1	0	Thoracotoı	250	CHU	No	79	0	0
1	0	VATS or ro	250	CHU	No	72	0	0
1	0	Thoracotoı	250	CHU	No	71	0	0
1	0	Thoracotoı	250	CHU	No	59	0	0
1	0	Thoracotoı	250	CHU	No	56	1	0
1	0	Thoracotoı	250	CHU	No	79	0	0
1	0	Thoracotoı	250	CHU	No	53	0	0
1	0	VATS or ro	250	CHU	No	68	0	0
1	0	Thoracotoı	250	CHU	No	65	0	1
1	1	Thoracotoı	250	CHU	No	76	0	0
1	0	Thoracotoı	250	CHU	No	57	0	0
1	0	Thoracotoı	250	CHU	No	64	0	0
1	1	Thoracotoı	250	CHU	No	55	0	0
1	0	Thoracotoı	250	CHU	No	73	0	0
1	0	Thoracotoı	250	CHU	No	64	0	1
1	0	Thoracotoı	250	CHU	No	74	0	0
1	0	VATS or ro	250	CHU	No	74	0	0
1	0	Thoracotoı	250	CHU	No	67	0	1
1	0	VATS or ro	250	CHU	No	56	0	0
1	0	Thoracotoı	250	CHU	No	46	0	0
1	0	Thoracotoı	250	CHU	No	53	0	0
1	0	Thoracotoı	250	CHU	No	57	0	0
1	0	VATS or ro	250	CHU	No	47	0	1
1	0	VATS or ro	250	CHU	No	78	0	0
1	0	Thoracotoı	250	CHU	No	58	0	0
1	0	Thoracotoı	250	CHU	No	50	0	1
1	0	Thoracotoı	250	CHU	No	51	0	0
1	0	Thoracotoı	250	CHU	No	69	0	1
1	0	VATS or ro	250	CHU	No	43	0	0
1	0	Thoracotoı	250	CHU	No	57	0	0
1	0	Thoracotoı	250	CHU	No	82	0	0
1	0	VATS or ro	250	CHU	No	82	0	0
1	0	Thoracotoı	250	CHU	No	30	0	0
1	0	Thoracotoı	250	CHU	No	60	1	1
1	0	VATS or ro	250	CHU	No	72	0	1
1	1	Thoracotoı	250	CHU	No	57	0	1
1	0	VATS or ro	250	CHU	No	82	0	0
1	0	Thoracotoı	250	CHU	No	50	0	1
1	0	Thoracotoı	250	CHU	No	69	0	1
1	0	VATS or ro	250	CHU	No	80	1	0

1	1	Thoracotoı	250	CHU	No	38	0	0
1	0	VATS or rc	250	CHU	No	52	0	1
1	0	Thoracotoı	250	CHU	No	60	0	0
1	0	VATS or rc	250	CHU	No	78	0	1
1	0	Thoracotoı	250	CHU	No	53	0	0
1	0	Thoracotoı	250	CHU	No	70	0	1
1	0	VATS or rc	250	CHU	No	65	0	0
1	0	Thoracotoı	250	CHU	No	73	0	0
1	0	Thoracotoı	250	CHU	No	70	1	0
1	0	Thoracotoı	250	CHU	No	74	1	0
1	1	Thoracotoı	250	CHU	No	61	0	0
1	0	VATS or rc	250	CHU	No	68	0	1
1	0	VATS or rc	250	CHU	No	65	0	0
1	0	VATS or rc	250	CHU	No	54	0	0
1	0	Thoracotoı	250	CHU	No	67	0	0
1	0	Thoracotoı	250	CHU	No	69	0	0
1	1	Thoracotoı	250	CHU	No	51	0	0
1	0	Thoracotoı	250	CHU	No	65	0	0
1	1	Thoracotoı	250	CHU	No	75	0	0
1	0	Thoracotoı	250	CHU	Yes	55	0	1
1	1	Thoracotoı	250	CHU	No	59	0	0
1	0	Thoracotoı	250	CHU	No	70	0	0
1	0	Thoracotoı	250	CHU	No	75	0	0
1	0	Thoracotoı	250	CHU	No	65	0	1
1	0	Thoracotoı	250	CHU	No	52	0	1
1	0	VATS or rc	250	CHU	No	67	0	0
1	0	Thoracotoı	250	CHU	No	68	0	1
1	0	Thoracotoı	250	CHU	No	64	0	1
1	0	Thoracotoı	250	CHU	No	73	1	0
1	0	Thoracotoı	250	CHU	No	63	0	0
1	0	Thoracotoı	250	CHU	No	64	1	0
1	0	Thoracotoı	250	CHU	No	61	0	0
1	0	Thoracotoı	250	CHU	Yes	62	0	0
1	1	Thoracotoı	250	CHU	No	55	0	0
1	1	Thoracotoı	250	CHU	No	58	1	1
1	0	VATS or rc	250	CHU	No	71	0	1
1	0	Thoracotoı	250	CHU	No	65	0	1
1	0	VATS or rc	250	CHU	No	72	0	1
1	0	VATS or rc	250	CHU	No	81	0	1
1	0	Thoracotoı	250	CHU	No	74	1	1
1	0	Thoracotoı	250	CHU	No	65	0	0
1	0	Thoracotoı	250	CHU	No	56	0	0
1	0	Thoracotoı	250	CHU	No	57	1	0
1	0	Thoracotoı	250	CHU	No	77	0	0
1	0	Thoracotoı	250	CHU	No	66	0	0
1	0	Thoracotoı	250	CHU	No	59	0	1
1	0	VATS or rc	250	CHU	No	66	0	0
1	0	VATS or rc	250	CHU	No	64	0	0
1	0	Thoracotoı	250	CHU	No	43	0	0
1	0	Thoracotoı	250	CHU	No	66	0	0
1	0	Thoracotoı	250	CHU	No	61	0	1
1	0	Thoracotoı	250	CHU	No	66	0	1
1	0	Thoracotoı	250	CHU	No	53	0	0
1	0	Thoracotoı	250	CHU	No	53	0	0
1	0	Thoracotoı	250	CHU	No	70	0	0
1	0	Thoracotoı	250	CHU	No	60	0	0

1	0	Thoracotoı	250	CHU	No	65	0	0
1	0	Thoracotoı	250	CHU	No	59	0	0
1	1	Thoracotoı	250	CHU	No	67	0	0
1	1	VATS or rc	250	CHU	No	58	0	1
1	0	Thoracotoı	250	CHU	No	77	1	0
1	0	Thoracotoı	250	CHU	No	70	1	1
1	0	VATS or rc	250	CHU	No	59	0	0
1	1	Thoracotoı	250	CHU	No	65	0	0
1	0	Thoracotoı	250	CHU	No	66	0	1
1	1	Thoracotoı	250	CHU	Yes	78	0	0
1	0	Thoracotoı	250	CHU	No	73	0	0
1	0	Thoracotoı	250	CHU	No	68	0	0
1	0	Thoracotoı	250	CHU	No	70	0	0
1	0	Thoracotoı	250	CHU	No	79	0	0
1	0	Thoracotoı	250	CHU	No	61	0	0
1	0	Thoracotoı	250	CHU	No	54	0	1
1	0	Thoracotoı	250	CHU	No	73	0	1
1	1	Thoracotoı	250	CHU	No	51	0	0
1	0	VATS or rc	250	CHU	No	68	0	0
1	0	Thoracotoı	250	CHU	No	51	0	1
1	0	Thoracotoı	250	CHU	No	48	0	0
1	0	VATS or rc	250	CHU	No	23	0	0
1	0	Thoracotoı	250	CHU	No	79	0	1
1	0	Thoracotoı	250	CHU	No	82	0	0
1	0	Thoracotoı	250	CHU	No	74	0	0
1	0	VATS or rc	250	CHU	No	62	0	0
1	0	VATS or rc	250	CHU	No	61	0	0
1	1	Thoracotoı	250	CHU	No	71	0	0
1	0	Thoracotoı	250	CHU	No	68	0	0
1	0	VATS or rc	250	CHU	No	54	0	1
1	1	Thoracotoı	250	CHU	No	57	0	0
1	0	Thoracotoı	250	CHU	No	64	0	0
1	0	Thoracotoı	250	CHU	No	53	0	0
1	0	Thoracotoı	250	CHU	No	49	1	0
1	0	Thoracotoı	250	CHU	Yes	66	0	0
1	0	VATS or rc	250	CHU	No	79	0	1
1	0	Thoracotoı	250	CHU	No	73	1	0
1	1	Thoracotoı	250	CHU	No	57	0	1
1	0	VATS or rc	250	CHU	No	76	0	0
1	0	Thoracotoı	250	CHU	No	52	0	0
1	0	Thoracotoı	250	CHU	No	66	0	1
1	0	Thoracotoı	250	CHU	No	55	0	1
1	0	Thoracotoı	250	CHU	No	57	0	0
1	0	VATS or rc	250	CHU	No	58	0	0
1	0	VATS or rc	250	CHU	No	86	0	0
1	0	VATS or rc	250	CHU	No	54	0	0
1	0	Thoracotoı	250	CHU	No	64	0	0
1	0	VATS or rc	250	CHU	No	61	0	1
1	0	Thoracotoı	250	CHU	No	83	0	0
1	0	Thoracotoı	250	CHU	No	68	0	0
1	0	Thoracotoı	250	CHU	No	73	0	0
1	0	VATS or rc	250	CHU	No	55	0	1
1	0	Thoracotoı	250	CHU	No	72	0	0
1	1	Thoracotoı	250	CHU	No	60	0	0
1	0	Thoracotoı	250	CHU	No	66	0	0
1	0	Thoracotoı	250	CHU	No	81	0	0

1	0	Thoracotoı	250	CHU	No	57	0	0
1	0	Thoracotoı	250	CHU	No	72	0	0
1	0	Thoracotoı	250	CHU	No	62	0	0
1	0	Thoracotoı	250	CHU	No	69	0	0
1	1	Thoracotoı	250	CHU	No	51	0	0
1	0	Thoracotoı	250	CHU	No	68	0	0
1	0	Thoracotoı	250	CHU	No	64	0	1
1	1	Thoracotoı	250	CHU	No	53	0	1
1	0	VATS or rc	250	CHU	No	77	0	1
1	0	Thoracotoı	250	CHU	No	41	0	1
1	0	Thoracotoı	250	CHU	No	70	0	0
1	0	VATS or rc	250	CHU	No	68	1	0
1	0	VATS or rc	250	CHU	No	73	0	0
1	0	Thoracotoı	250	CHU	No	73	0	0
1	0	Thoracotoı	250	CHU	No	52	0	1
1	0	VATS or rc	250	CHU	No	57	1	1
1	0	Thoracotoı	250	CHU	No	60	0	0
1	1	Thoracotoı	250	CHU	No	62	0	0
1	0	Thoracotoı	250	CHU	No	58	0	0
1	0	Thoracotoı	250	CHU	No	63	0	0
1	0	Thoracotoı	250	CHU	No	54	0	0
1	0	Thoracotoı	250	CHU	No	75	0	0
1	0	Thoracotoı	250	CHU	No	82	1	0
1	0	Thoracotoı	250	CHU	No	73	0	1
1	1	Thoracotoı	250	CHU	No	62	0	0
1	0	Thoracotoı	250	CHU	No	74	0	0
1	0	VATS or rc	250	CHU	No	65	0	0
1	0	Thoracotoı	250	CHU	No	28	0	0
1	0	Thoracotoı	250	CHU	No	73	0	1
1	0	Thoracotoı	250	CHU	No	62	0	0
1	0	Thoracotoı	250	CHU	No	58	0	0
1	0	Thoracotoı	250	CHU	No	57	1	0
1	0	Thoracotoı	250	CHU	No	53	0	0
1	1	Thoracotoı	250	CHU	No	66	0	1
1	0	Thoracotoı	250	CHU	No	60	0	0
1	0	VATS or rc	250	CHU	No	74	0	0
1	0	Thoracotoı	250	CHU	No	54	0	0
1	1	Thoracotoı	250	CHU	No	56	1	0
1	0	Thoracotoı	250	CHU	No	69	0	0
1	0	Thoracotoı	250	CHU	No	55	0	1
1	0	Thoracotoı	250	CHU	No	55	0	0
1	0	VATS or rc	250	CHU	No	78	0	0
1	1	Thoracotoı	250	CHU	No	57	0	0
1	0	Thoracotoı	250	CHU	No	79	0	1
1	0	Thoracotoı	250	CHU	No	69	0	0
1	0	Thoracotoı	250	CHU	No	66	0	0
1	0	Thoracotoı	250	CHU	No	57	0	0
1	0	VATS or rc	250	CHU	No	55	0	1
1	0	Thoracotoı	250	CHU	No	53	0	0
1	0	Thoracotoı	250	CHU	No	56	0	1
1	0	Thoracotoı	250	CHU	No	70	0	0
1	0	VATS or rc	250	CHU	No	44	0	0
1	0	VATS or rc	250	CHU	No	65	1	0
1	0	Thoracotoı	250	CHU	No	61	0	1
1	0	VATS or rc	250	CHU	No	77	0	1
1	0	Thoracotoı	250	CHU	No	45	0	0

1	1	Thoracotoı	250	CHU	No	64	0	0
1	0	Thoracotoı	250	CHU	No	66	0	1
1	0	Thoracotoı	250	CHU	No	85	0	0
1	0	Thoracotoı	250	CHU	No	73	0	0
1	1	Thoracotoı	250	CHU	No	58	0	0
1	0	Thoracotoı	250	CHU	No	74	0	0
1	1	Thoracotoı	250	CHU	No	64	0	1
1	0	VATS or rc	250	CHU	No	28	0	0
1	0	Thoracotoı	250	CHU	No	57	0	0
1	0	Thoracotoı	250	CHU	No	69	0	0
1	0	VATS or rc	250	CHU	No	72	0	0
1	0	Thoracotoı	250	CHU	No	53	0	1
1	0	Thoracotoı	250	CHU	No	69	0	1
1	0	Thoracotoı	250	CHU	No	56	0	1
1	0	Thoracotoı	250	CHU	No	63	0	0
1	0	Thoracotoı	250	CHU	No	69	0	0
1	0	Thoracotoı	250	CHU	No	65	0	0
1	0	Thoracotoı	250	CHU	No	47	0	0
1	0	Thoracotoı	250	CHU	No	68	0	0
1	1	Thoracotoı	250	CHU	No	79	1	0
1	0	Thoracotoı	250	CHU	No	46	0	0
1	0	Thoracotoı	250	CHU	No	80	0	0
1	0	Thoracotoı	250	CHU	No	78	0	0
1	1	Thoracotoı	250	CHU	No	67	0	0
1	0	Thoracotoı	250	CHU	No	55	0	1
1	1	Thoracotoı	250	CHU	No	53	0	1
1	0	Thoracotoı	250	CHU	No	74	0	0
1	0	Thoracotoı	250	CHU	No	76	0	0
1	1	Thoracotoı	250	CHU	No	59	0	0
1	0	Thoracotoı	250	CHU	No	64	0	0
1	0	Thoracotoı	250	CHU	No	62	0	1
1	0	Thoracotoı	250	CHU	No	75	1	0
1	0	Thoracotoı	250	CHU	No	64	0	1
1	0	Thoracotoı	250	CHU	No	50	0	0
1	0	Thoracotoı	250	CHU	Yes	48	0	0
1	0	Thoracotoı	250	CHU	No	59	0	1
1	1	Thoracotoı	250	CHU	No	79	0	1
1	0	VATS or rc	250	CHU	No	86	0	0
1	0	VATS or rc	250	CHU	No	59	0	0
1	0	Thoracotoı	250	CHU	No	65	0	0
1	0	Thoracotoı	250	CHU	No	77	0	1
1	0	VATS or rc	250	CHU	No	67	0	0
1	0	Thoracotoı	250	CHU	No	57	0	0
1	1	Thoracotoı	250	CHU	No	50	0	0
1	0	Thoracotoı	250	CHU	No	68	0	0
1	0	Thoracotoı	250	CHU	No	55	0	0
1	0	Thoracotoı	250	CHU	No	63	0	0
1	0	Thoracotoı	250	CHU	No	53	0	0
1	0	VATS or rc	250	CHU	No	63	0	0
1	0	Thoracotoı	250	CHU	No	72	0	1
1	0	VATS or rc	250	CHU	No	62	0	1
1	0	Thoracotoı	250	CHU	No	63	0	0
1	1	Thoracotoı	250	CHU	No	65	0	0
1	1	Thoracotoı	250	CHU	No	76	0	0
1	0	Thoracotoı	250	CHU	No	70	0	0
1	0	VATS or rc	250	CHU	No	51	0	0

1	0	VATS or ro	250	CHU	No	85	0	0
1	0	Thoracotoı	250	CHU	No	80	0	0
1	0	Thoracotoı	250	CHU	No	75	0	0
1	0	Thoracotoı	250	CHU	Yes	57	0	0
1	0	Thoracotoı	250	CHU	No	51	0	1
1	0	Thoracotoı	250	CHU	No	63	0	0
1	0	Thoracotoı	250	CHU	No	62	0	0
1	0	VATS or ro	250	CHU	No	68	0	0
1	0	Thoracotoı	250	CHU	No	81	0	0
1	0	VATS or ro	250	CHU	No	65	0	0
1	0	VATS or ro	250	CHU	No	82	1	0
1	0	Thoracotoı	250	CHU	No	30	0	0
1	0	Thoracotoı	250	CHU	No	81	0	0
1	0	VATS or ro	250	CHU	No	61	0	0
1	0	Thoracotoı	250	CHU	No	64	0	0
1	0	Thoracotoı	250	CHU	No	63	0	1
1	0	Thoracotoı	250	CHU	No	54	0	1
1	0	Thoracotoı	250	CHU	No	69	0	0
1	0	Thoracotoı	250	CHU	No	61	0	1
1	1	Thoracotoı	250	CHU	No	59	0	1
1	0	Thoracotoı	250	CHU	No	60	0	0
1	0	VATS or ro	250	CHU	No	56	0	1
1	0	Thoracotoı	250	CHU	No	81	0	0
1	0	VATS or ro	250	CHU	No	64	1	0
1	0	VATS or ro	250	CHU	No	63	1	0
1	1	Thoracotoı	250	CHU	No	59	0	0
1	0	Thoracotoı	250	CHU	No	71	0	0
1	1	Thoracotoı	250	CHU	No	59	0	0
1	0	Thoracotoı	250	CHU	No	54	0	0
1	0	Thoracotoı	250	CHU	No	47	0	0
1	0	Thoracotoı	250	CHU	No	52	0	0
1	0	Thoracotoı	250	CHU	No	68	0	0
1	0	Thoracotoı	250	CHU	No	59	0	0
1	0	Thoracotoı	250	CHU	No	40	0	1
1	1	Thoracotoı	250	CHU	No	60	0	0
1	0	Thoracotoı	250	CHU	No	21	0	0
1	1	Thoracotoı	250	CHU	No	42	0	0
1	1	Thoracotoı	250	CHU	No	51	0	1
1	0	Thoracotoı	250	CHU	No	68	1	1
1	0	Thoracotoı	250	CHU	No	82	0	0
1	0	Thoracotoı	250	CHU	No	72	0	0
1	0	Thoracotoı	250	CHU	No	42	0	0
1	0	VATS or ro	250	CHU	No	68	0	0
1	0	Thoracotoı	250	CHU	No	53	0	0
1	0	Thoracotoı	250	CHU	No	57	0	1
1	0	Thoracotoı	250	CHU	No	63	0	0
1	0	Thoracotoı	250	CHU	No	65	0	0
1	0	Thoracotoı	250	CHU	No	56	0	0
1	0	Thoracotoı	250	CHU	No	63	0	0
1	0	Thoracotoı	250	CHU	No	52	0	0
1	0	Thoracotoı	250	CHU	No	78	0	0
1	0	VATS or ro	250	CHU	No	73	0	1
1	0	Thoracotoı	250	CHU	No	51	0	0
1	0	VATS or ro	250	CHU	No	80	0	0
1	0	Thoracotoı	250	CHU	No	47	0	0
1	0	Thoracotoı	250	CHU	No	56	0	0

1	0	VATS or rc	250	CHU	No	66	0	0
1	1	Thoracotoı	250	CHU	No	68	0	0
1	1	Thoracotoı	250	CHU	No	74	0	0
1	0	VATS or rc	250	CHU	No	70	1	1
1	0	Thoracotoı	250	CHU	No	53	0	0
1	0	VATS or rc	250	CHU	No	82	0	0
1	0	Thoracotoı	250	CHU	No	80	0	0
1	0	Thoracotoı	250	CHU	No	58	0	1
1	0	Thoracotoı	250	CHU	No	54	0	1
1	0	Thoracotoı	250	CHU	No	73	1	0
1	0	Thoracotoı	250	CHU	No	55	0	0
1	0	VATS or rc	250	CHU	No	67	1	0
1	1	Thoracotoı	250	CHU	No	56	0	1
1	0	Thoracotoı	250	CHU	No	68	0	0
1	0	Thoracotoı	250	CHU	No	50	0	1
1	0	VATS or rc	250	CHU	No	72	0	0
1	0	Thoracotoı	250	CHU	No	69	0	0
1	0	VATS or rc	250	CHU	No	62	0	0
1	0	Thoracotoı	250	CHU	No	68	0	0
1	1	Thoracotoı	250	CHU	No	57	0	0
1	0	VATS or rc	250	CHU	No	81	0	0
1	0	Thoracotoı	250	CHU	No	61	0	0
1	0	Thoracotoı	250	CHU	No	55	0	0
1	0	Thoracotoı	250	CHU	No	61	0	1
1	0	Thoracotoı	250	CHU	No	56	0	0
1	0	Thoracotoı	250	CHU	No	62	0	0
1	0	Thoracotoı	250	CHU	No	51	0	0
1	0	Thoracotoı	250	CHU	No	65	0	0
1	0	Thoracotoı	250	CHU	No	72	0	0
1	0	Thoracotoı	250	CHU	No	62	1	0
1	0	VATS or rc	250	CHU	No	70	0	0
1	0	Thoracotoı	250	CHU	No	56	0	1
1	0	Thoracotoı	250	CHU	No	58	0	0
1	1	Thoracotoı	250	CHU	No	64	0	0
1	0	Thoracotoı	250	CHU	No	62	0	0
1	0	Thoracotoı	250	CHU	No	79	0	0
1	0	VATS or rc	250	CHU	No	76	0	0
1	0	VATS or rc	250	CHU	No	53	0	0
1	0	Thoracotoı	250	CHU	No	72	0	0
1	0	Thoracotoı	250	CHU	No	70	0	0
1	0	Thoracotoı	250	CHU	No	73	0	1
1	1	Thoracotoı	250	CHU	No	49	0	0
1	0	Thoracotoı	250	CHU	No	71	0	0
1	0	Thoracotoı	250	CHU	No	64	0	0
1	0	Thoracotoı	250	CHU	No	51	0	1
1	0	Thoracotoı	250	CHU	No	60	0	0
1	0	Thoracotoı	250	CHU	No	57	0	1
1	0	Thoracotoı	250	CHU	No	68	0	0
1	0	Thoracotoı	250	CHU	No	55	0	1
1	0	Thoracotoı	250	CHU	No	46	0	0
1	0	Thoracotoı	250	CHU	No	70	0	0
1	1	Thoracotoı	250	CHU	No	68	0	1
1	0	Thoracotoı	250	CHU	No	60	0	0
1	1	Thoracotoı	250	CHU	No	60	0	0
1	0	Thoracotoı	250	CHU	No	64	0	1
1	0	Thoracotoı	250	CHU	No	63	0	0

1	1	Thoracotor	250	CHU	No	62	0	0
1	0	Thoracotor	250	CHU	No	73	0	1
1	0	Thoracotor	250	CHU	No	50	0	1
1	0	VATS or rc	250	CHU	No	63	0	0
1	0	VATS or rc	250	CHU	No	75	1	0
1	1	Thoracotor	250	CHU	No	62	0	0
1	0	Thoracotor	250	CHU	No	74	0	0
1	0	Thoracotor	250	CHU	No	69	0	0
1	0	VATS or rc	250	CHU	No	67	0	1
1	0	Thoracotor	250	CHU	No	59	0	0
1	0	Thoracotor	250	CHU	No	61	0	0
1	0	Thoracotor	250	CHU	No	73	0	0
1	0	Thoracotor	250	CHU	No	54	0	1
1	0	Thoracotor	250	CHU	No	56	0	0
1	0	VATS or rc	250	CHU	No	52	0	0
1	0	Thoracotor	250	CHU	No	58	0	1
1	0	Thoracotor	250	CHU	No	69	0	0
1	0	Thoracotor	250	CHU	No	62	0	0
1	0	Thoracotor	250	CHU	No	69	0	0
1	0	Thoracotor	250	CHU	No	61	0	0
1	0	Thoracotor	250	CHU	No	81	0	0
1	0	Thoracotor	250	CHU	No	56	0	0
1	1	Thoracotor	250	CHU	No	67	0	0
1	0	VATS or rc	250	CHU	No	64	0	1
1	0	Thoracotor	250	CHU	No	63	0	0
1	0	Thoracotor	250	CHU	No	62	0	0
1	0	Thoracotor	250	CHU	No	78	0	0
1	0	VATS or rc	250	CHU	No	65	0	1
1	1	Thoracotor	250	CHU	No	59	1	0
1	0	Thoracotor	250	CHU	No	60	0	0
1	0	Thoracotor	250	CHU	No	68	0	0
1	0	Thoracotor	250	CHU	No	70	0	0
1	0	VATS or rc	250	CHU	No	57	0	1
1	0	Thoracotor	250	CHU	No	52	0	1
1	1	Thoracotor	250	CHU	No	59	0	0
1	1	Thoracotor	250	CHU	No	47	0	0
1	0	Thoracotor	250	CHU	No	75	0	0
1	0	Thoracotor	250	CHU	No	70	1	0
1	0	VATS or rc	250	CHU	No	78	0	0
1	0	Thoracotor	250	CHU	No	56	0	0
1	0	Thoracotor	250	CHU	No	49	0	0
1	1	Thoracotor	250	CHU	No	51	0	0
1	0	Thoracotor	250	CHU	No	72	0	0
1	0	Thoracotor	250	CHU	No	67	0	1
1	1	Thoracotor	250	CHU	No	80	0	0
1	0	Thoracotor	250	CHU	No	50	0	1
1	0	Thoracotor	250	CHU	No	63	0	0
1	0	Thoracotor	250	CHU	No	68	0	0
1	0	VATS or rc	250	CHU	No	71	0	0
1	0	Thoracotor	250	CHU	No	46	0	0
1	0	Thoracotor	250	CHU	No	76	0	0
1	1	Thoracotor	250	CHU	No	39	0	0
1	0	Thoracotor	250	CHU	No	54	0	0
1	0	Thoracotor	250	CHU	No	75	0	0
1	0	Thoracotor	250	CHU	No	50	0	0
1	0	VATS or rc	250	CHU	No	70	0	0

1	0	Thoracotoı	250	CHU	No	47	0	0
1	0	VATS or rc	250	CHU	No	67	0	0
1	0	VATS or rc	250	CHU	No	71	0	0
1	0	Thoracotoı	250	CHU	No	66	0	0
1	0	Thoracotoı	250	CHU	No	63	0	0
1	1	Thoracotoı	250	CHU	No	62	0	0
1	0	Thoracotoı	250	CHU	No	72	0	0
1	0	Thoracotoı	250	CHU	Yes	53	0	1
1	0	Thoracotoı	250	CHU	No	80	0	0
1	0	Thoracotoı	250	CHU	No	59	0	1
1	1	Thoracotoı	250	CHU	No	70	0	0
1	0	VATS or rc	250	CHU	No	78	0	0
1	0	Thoracotoı	250	CHU	No	70	0	0
1	0	Thoracotoı	250	CHU	No	72	0	1
1	0	VATS or rc	250	CHU	No	44	0	0
1	1	Thoracotoı	250	CHU	No	58	0	1
1	0	Thoracotoı	250	CHU	No	86	0	0
1	0	Thoracotoı	250	CHU	No	70	0	0
1	0	Thoracotoı	250	CHU	No	71	0	0
1	0	Thoracotoı	250	CHU	No	82	0	0
1	0	Thoracotoı	250	CHU	No	74	0	0
1	1	Thoracotoı	250	CHU	No	58	0	0
1	0	Thoracotoı	250	CHU	No	77	0	0
1	0	Thoracotoı	250	CHU	No	77	0	0
1	0	Thoracotoı	250	CHU	No	58	0	0
1	0	Thoracotoı	250	CHU	No	66	0	0
1	0	Thoracotoı	250	CHU	No	74	0	0
1	0	Thoracotoı	250	CHU	No	64	0	0
1	1	Thoracotoı	250	CHU	No	74	0	0
1	0	Thoracotoı	250	CHU	No	62	0	0
1	0	Thoracotoı	250	CHU	No	66	0	0
1	0	Thoracotoı	250	CHU	No	66	0	0
1	0	Thoracotoı	250	CHU	No	44	0	0
1	0	Thoracotoı	250	CHU	No	64	0	0
1	1	Thoracotoı	250	CHU	No	79	0	0
1	1	Thoracotoı	250	CHU	No	47	0	0
1	0	Thoracotoı	250	CHU	No	67	1	1
1	0	Thoracotoı	250	CHU	No	83	0	0
1	0	Thoracotoı	250	CHU	No	64	0	1
1	0	Thoracotoı	250	CHU	No	76	0	0
1	0	Thoracotoı	250	CHU	No	76	0	0
1	0	Thoracotoı	250	CHU	No	53	0	1
1	0	Thoracotoı	250	CHU	No	52	0	0
1	0	Thoracotoı	250	CHU	No	47	0	1
1	1	Thoracotoı	250	CHU	No	59	0	0
1	0	Thoracotoı	250	CHU	No	66	0	0
1	0	Thoracotoı	250	CHU	No	69	0	0
1	0	Thoracotoı	250	CHU	No	68	0	1
1	0	Thoracotoı	250	CHU	No	69	0	0
1	0	Thoracotoı	250	CHU	No	59	1	1
1	0	VATS or rc	250	CHU	No	80	0	0
1	0	VATS or rc	250	CHU	No	63	0	0
1	0	Thoracotoı	250	CHU	No	70	0	0
1	0	Thoracotoı	250	CHU	No	53	0	0
1	0	Thoracotoı	250	CHU	No	57	0	1
1	0	Thoracotoı	250	CHU	No	70	0	1

1	0	Thoracotor	250	CHU	No	66	0	0
1	0	Thoracotor	250	CHU	No	66	0	0
1	0	Thoracotor	250	CHU	No	50	0	0
1	0	Thoracotor	250	CHU	No	38	0	0
1	0	VATS or ro	250	CHU	No	52	0	1
1	0	Thoracotor	250	CHU	No	62	0	0
1	0	Thoracotor	250	CHU	No	59	0	0
1	0	Thoracotor	250	CHU	No	73	1	0
1	0	Thoracotor	250	CHU	No	55	0	0
1	0	VATS or ro	250	CHU	No	77	0	0
1	0	Thoracotor	250	CHU	No	80	0	0
1	0	Thoracotor	250	CHU	No	65	0	1
1	0	Thoracotor	250	CHU	No	73	0	0
1	0	Thoracotor	250	CHU	No	65	0	0
1	0	Thoracotor	250	CHU	No	60	0	0
1	0	Thoracotor	250	CHU	No	80	0	1
1	0	Thoracotor	250	CHU	No	70	0	0
1	0	Thoracotor	250	CHU	No	71	0	1
1	0	Thoracotor	250	CHU	No	69	0	0
1	0	Thoracotor	250	CHU	No	74	1	0
1	1	Thoracotor	250	CHU	Yes	73	0	0
1	0	Thoracotor	250	CHU	No	67	0	0
1	0	Thoracotor	250	CHU	No	74	1	0
1	0	Thoracotor	250	CHU	No	82	0	0
1	0	Thoracotor	250	CHU	No	66	1	0
1	0	VATS or ro	250	CHU	No	55	0	0
1	0	VATS or ro	250	CHU	No	58	0	1
1	0	VATS or ro	250	CHU	No	66	0	0
1	0	VATS or ro	250	CHU	No	62	0	0
1	0	Thoracotor	250	CHU	No	64	0	1
1	0	VATS or ro	250	CHU	No	52	0	0
1	0	Thoracotor	250	CHU	No	66	0	1
1	0	VATS or ro	250	CHU	No	75	0	0
1	0	Thoracotor	250	CHU	No	64	0	0
1	1	Thoracotor	250	CHU	No	47	0	0
1	0	Thoracotor	250	CHU	No	77	0	1
1	0	Thoracotor	250	CHU	No	50	0	0
1	0	VATS or ro	250	CHU	No	72	1	1
1	0	Thoracotor	250	CHU	No	69	0	0
1	0	Thoracotor	250	CHU	No	64	0	0
1	0	VATS or ro	250	CHU	No	57	0	0
1	1	Thoracotor	250	CHU	No	67	0	0
1	1	Thoracotor	250	CHU	No	76	0	1
1	0	VATS or ro	250	CHU	No	71	0	1
1	0	Thoracotor	250	CHU	No	56	0	1
1	0	VATS or ro	250	CHU	No	46	0	1
1	0	Thoracotor	250	CHU	No	60	0	0
1	0	Thoracotor	250	CHU	No	62	0	1
1	0	Thoracotor	250	CHU	No	59	0	0
1	0	Thoracotor	250	CHU	No	68	1	1
1	0	Thoracotor	250	CHU	No	83	0	0
1	0	Thoracotor	250	CHU	No	60	0	0
1	0	Thoracotor	250	CHU	No	65	0	0
1	0	Thoracotor	250	CHU	No	78	0	0
1	0	Thoracotor	250	CHU	No	62	0	0
1	0	Thoracotor	250	CHU	No	66	0	0

1	0	Thoracotoı	250	CHU	No	51	0	0
1	1	Thoracotoı	250	CHU	Yes	69	0	0
1	0	Thoracotoı	250	CHU	No	60	0	0
1	1	Thoracotoı	250	CHU	No	57	0	0
1	0	VATS or rc	250	CHU	No	81	0	0
1	0	Thoracotoı	250	CHU	No	76	0	0
1	0	Thoracotoı	250	CHU	No	75	0	0
1	0	VATS or rc	250	CHU	No	52	0	1
1	0	Thoracotoı	250	CHU	No	78	0	0
1	0	Thoracotoı	250	CHU	No	65	0	0
1	1	Thoracotoı	250	CHU	No	62	0	0
1	0	VATS or rc	250	CHU	No	48	0	0
1	0	Thoracotoı	250	CHU	No	61	1	0
1	0	VATS or rc	250	CHU	No	75	0	0
1	0	Thoracotoı	250	CHU	No	57	0	1
1	0	Thoracotoı	250	CHU	No	61	0	0
1	0	VATS or rc	250	CHU	No	61	0	0
1	0	VATS or rc	250	CHU	No	68	0	0
1	0	Thoracotoı	250	CHU	No	46	0	0
1	0	Thoracotoı	250	CHU	No	80	0	1
1	1	Thoracotoı	250	CHU	No	50	0	0
1	0	Thoracotoı	250	CHU	No	52	0	0
1	0	Thoracotoı	250	CHU	No	62	0	0
1	0	VATS or rc	250	CHU	No	70	0	1
1	0	Thoracotoı	250	CHU	No	72	0	0
1	0	Thoracotoı	250	CHU	No	62	0	1
1	0	Thoracotoı	250	CHU	No	79	0	0
1	0	Thoracotoı	250	CHU	No	57	0	1
1	0	VATS or rc	250	CHU	No	61	0	0
1	0	VATS or rc	250	CHU	No	65	0	1
1	0	VATS or rc	250	CHU	No	69	0	0
1	0	Thoracotoı	250	CHU	No	72	0	0
1	0	Thoracotoı	250	CHU	No	56	1	0
1	0	Thoracotoı	250	CHU	No	45	0	1
1	0	Thoracotoı	250	CHU	No	69	0	1
1	0	Thoracotoı	250	CHU	No	63	0	1
1	0	Thoracotoı	250	CHU	No	79	0	1
1	0	VATS or rc	250	CHU	No	71	0	0
1	0	VATS or rc	250	CHU	No	53	0	0
1	0	Thoracotoı	250	CHU	No	67	0	0
1	0	Thoracotoı	250	CHU	No	55	0	1
1	0	Thoracotoı	250	CHU	No	67	0	1
1	0	Thoracotoı	250	CHU	No	53	0	0
1	0	Thoracotoı	250	CHU	No	76	1	0
1	0	Thoracotoı	250	CHU	No	79	0	1
1	0	VATS or rc	250	CHU	No	68	0	0
1	0	VATS or rc	250	CHU	No	81	0	0
1	1	Thoracotoı	250	CHU	No	51	0	0
1	1	Thoracotoı	250	CHU	No	59	0	0
1	1	Thoracotoı	250	CHU	No	82	0	0
1	0	Thoracotoı	250	CHU	No	53	0	0
1	0	Thoracotoı	250	CHU	No	73	0	0
1	0	VATS or rc	250	CHU	No	83	0	0
1	0	Thoracotoı	250	CHU	No	57	0	1
1	0	Thoracotoı	250	CHU	No	70	0	0
1	0	Thoracotoı	250	CHU	No	77	0	1

1	0	Thoracotoı	250	CHU	No	55	0	1
1	0	Thoracotoı	250	CHU	No	46	0	1
1	1	Thoracotoı	250	CHU	Yes	74	0	0
1	0	VATS or rc	250	CHU	No	76	0	0
1	0	VATS or rc	250	CHU	No	60	0	1
1	0	Thoracotoı	250	CHU	No	82	0	0
1	1	Thoracotoı	250	CHU	No	73	0	0
1	0	Thoracotoı	250	CHU	No	49	0	1
1	0	Thoracotoı	250	CHU	No	37	0	0
1	0	Thoracotoı	250	CHU	No	49	0	1
1	0	Thoracotoı	250	CHU	No	67	0	1
1	0	Thoracotoı	250	CHU	No	56	1	0
1	0	Thoracotoı	250	CHU	No	55	0	0
1	0	Thoracotoı	250	CHU	No	56	0	0
1	1	Thoracotoı	250	CHU	No	30	0	0
1	0	Thoracotoı	250	CHU	No	74	0	0
1	0	Thoracotoı	250	CHU	No	67	0	0
1	0	Thoracotoı	250	CHU	No	58	0	0
1	1	Thoracotoı	250	CHU	No	66	0	0
1	0	VATS or rc	250	CHU	No	63	0	1
1	0	Thoracotoı	250	CHU	No	57	0	1
1	0	Thoracotoı	250	CHU	No	70	0	1
1	0	Thoracotoı	250	CHU	No	73	1	0
1	0	Thoracotoı	250	CHU	No	68	0	0
1	0	Thoracotoı	250	CHU	No	62	0	0
1	0	VATS or rc	250	CHU	No	65	0	1
1	0	Thoracotoı	250	CHU	No	57	0	0
1	0	Thoracotoı	250	CHU	No	75	0	1
1	1	Thoracotoı	250	CHU	No	66	0	0
1	0	Thoracotoı	250	CHU	No	64	0	0
1	0	VATS or rc	250	CHU	No	66	0	1
1	0	Thoracotoı	250	CHU	No	75	0	0
1	0	Thoracotoı	250	CHU	No	67	0	0
1	0	VATS or rc	250	CHU	No	63	0	0
1	0	Thoracotoı	250	CHU	No	76	0	0
1	0	Thoracotoı	250	CHU	No	30	0	1
1	0	VATS or rc	250	CHU	No	82	0	0
1	0	Thoracotoı	250	CHU	No	69	0	0
1	0	Thoracotoı	250	CHU	No	62	0	0
1	0	Thoracotoı	250	CHU	Yes	65	0	0
1	0	VATS or rc	250	CHU	No	68	1	0
1	0	Thoracotoı	250	CHU	No	75	0	0
1	0	Thoracotoı	250	CHU	No	80	0	0
1	0	Thoracotoı	250	CHU	No	62	0	0
1	0	VATS or rc	250	CHU	No	35	0	0
1	0	Thoracotoı	250	CHU	No	59	0	0
1	1	Thoracotoı	250	CHU	No	49	0	1
1	0	VATS or rc	250	CHU	No	63	0	0
1	0	Thoracotoı	250	CHU	No	55	0	0
1	0	Thoracotoı	250	CHU	No	64	0	1
1	0	Thoracotoı	250	CHU	No	68	0	0
1	0	Thoracotoı	250	CHU	Yes	70	0	0
1	0	Thoracotoı	250	CHU	No	74	1	0
1	0	Thoracotoı	250	CHU	No	57	0	0
1	0	Thoracotoı	250	CHU	No	65	0	0
1	0	Thoracotoı	250	CHU	No	33	0	0

1	0	Thoracotoı	250	CHU	No	67	0	0
1	0	Thoracotoı	250	CHU	No	59	0	0
1	0	Thoracotoı	250	CHU	No	69	0	0
1	0	VATS or rc	250	CHU	No	66	0	0
1	1	Thoracotoı	250	CHU	No	53	0	1
1	0	Thoracotoı	250	CHU	No	69	1	0
1	0	Thoracotoı	250	CHU	No	50	0	0
1	0	Thoracotoı	250	CHU	No	69	1	0
1	0	Thoracotoı	250	CHU	No	70	0	0
1	1	Thoracotoı	250	CHU	No	61	0	1
1	1	Thoracotoı	250	CHU	No	71	0	0
1	0	Thoracotoı	250	CHU	No	66	0	0
1	0	VATS or rc	250	CHU	No	58	0	1
1	0	Thoracotoı	250	CHU	No	57	0	0
1	0	Thoracotoı	250	CHU	No	56	0	0
1	1	Thoracotoı	250	CHU	No	56	0	0
1	0	Thoracotoı	250	CHU	No	65	0	0
1	0	Thoracotoı	250	CHU	No	74	0	0
1	1	Thoracotoı	250	CHU	No	66	0	1
1	0	Thoracotoı	250	CHU	No	78	0	0
1	0	Thoracotoı	250	CHU	No	73	0	0
1	1	Thoracotoı	250	CHU	No	70	0	1
1	0	Thoracotoı	250	CHU	No	68	0	0
1	0	Thoracotoı	250	CHU	No	48	0	0
1	0	Thoracotoı	250	CHU	No	60	0	0
1	1	Thoracotoı	250	CHU	Yes	63	0	0
1	0	VATS or rc	250	CHU	No	50	0	1
1	0	Thoracotoı	250	CHU	No	59	0	0
1	0	Thoracotoı	250	CHU	No	79	0	0
1	0	VATS or rc	250	CHU	No	55	0	0
1	0	Thoracotoı	250	CHU	No	51	0	0
1	1	Thoracotoı	250	CHU	No	48	0	0
1	0	Thoracotoı	250	CHU	No	67	0	0
1	0	Thoracotoı	250	CHU	No	66	0	1
1	0	VATS or rc	250	CHU	No	64	0	0
1	0	Thoracotoı	250	CHU	No	66	0	0
1	0	Thoracotoı	250	CHU	No	66	0	1
1	0	Thoracotoı	250	CHU	No	54	0	0
1	0	Thoracotoı	250	CHU	No	70	0	0
1	1	Thoracotoı	250	CHU	No	53	0	0
1	0	Thoracotoı	250	CHU	No	57	0	1
1	1	Thoracotoı	250	CHU	No	50	0	0
1	0	Thoracotoı	250	CHU	No	69	0	0
1	0	Thoracotoı	250	CHU	No	79	0	0
1	0	Thoracotoı	250	CHU	No	71	0	0
1	0	Thoracotoı	250	CHU	No	55	0	0
1	1	Thoracotoı	250	CHU	No	53	0	1
1	1	Thoracotoı	250	CHU	No	72	0	0
1	0	Thoracotoı	250	CHU	No	57	0	1
1	0	Thoracotoı	250	CHU	No	57	0	0
1	0	Thoracotoı	250	CHU	No	51	0	0
1	0	Thoracotoı	250	CHU	No	59	0	0
1	0	Thoracotoı	250	CHU	No	48	0	0
1	0	VATS or rc	250	CHU	No	60	0	0
1	0	Thoracotoı	250	CHU	No	48	0	0
1	1	Thoracotoı	250	CHU	No	62	0	0

1	0	Thoracotoı	250	CHU	No	70	0	0
1	1	Thoracotoı	250	CHU	No	55	0	0
1	0	Thoracotoı	250	CHU	No	75	0	0
1	0	Thoracotoı	250	CHU	No	62	0	0
1	0	Thoracotoı	250	CHU	No	63	0	0
1	0	Thoracotoı	250	CHU	No	56	1	0
1	1	Thoracotoı	250	CHU	No	59	0	0
1	0	Thoracotoı	250	CHU	No	52	0	1
1	0	Thoracotoı	250	CHU	No	67	0	0
1	0	Thoracotoı	250	CHU	No	81	0	0
1	1	Thoracotoı	250	CHU	Yes	81	0	1
1	0	Thoracotoı	250	CHU	No	63	0	0
1	0	Thoracotoı	250	CHU	No	75	0	0
1	0	Thoracotoı	250	CHU	No	61	0	0
1	0	Thoracotoı	250	CHU	No	79	0	0
1	0	Thoracotoı	250	CHU	No	68	1	0
1	0	Thoracotoı	250	CHU	No	76	0	0
1	0	Thoracotoı	250	CHU	No	71	1	0
1	0	Thoracotoı	250	CHU	Yes	54	0	0
1	0	Thoracotoı	250	CHU	No	68	0	1
1	0	VATS or rc	250	CHU	No	69	0	0
1	0	VATS or rc	250	CHU	No	57	0	0
1	0	VATS or rc	250	CHU	No	63	0	0
1	0	VATS or rc	250	CHU	No	72	0	0
1	0	Thoracotoı	250	CHU	No	76	0	0
1	0	Thoracotoı	250	CHU	No	49	0	0
1	0	Thoracotoı	250	CHU	No	65	0	1
1	0	Thoracotoı	250	CHU	No	60	0	0
1	1	Thoracotoı	250	CHU	No	54	0	1
1	0	Thoracotoı	250	CHU	No	69	0	0
1	0	Thoracotoı	250	CHU	No	52	0	0
1	0	VATS or rc	250	CHU	No	51	0	0
1	0	Thoracotoı	250	CHU	No	60	0	0
1	0	Thoracotoı	250	CHU	No	72	0	0
1	1	Thoracotoı	250	CHU	No	48	0	1
1	0	Thoracotoı	250	CHU	No	63	0	0
1	0	Thoracotoı	250	CHU	No	24	0	0
1	0	Thoracotoı	250	CHU	No	63	0	1
1	0	Thoracotoı	250	CHU	No	79	0	0
1	0	VATS or rc	250	CHU	No	62	0	0
1	0	Thoracotoı	250	CHU	No	67	0	1
1	0	Thoracotoı	250	CHU	No	55	0	0
1	0	VATS or rc	250	CHU	No	79	0	0
1	0	Thoracotoı	250	CHU	No	71	0	0
1	0	Thoracotoı	250	CHU	No	37	0	0
1	0	Thoracotoı	250	CHU	No	65	0	0
1	0	VATS or rc	250	CHU	No	73	0	0
1	0	Thoracotoı	250	CHU	No	60	0	0
1	0	VATS or rc	250	CHU	No	64	0	0
1	0	Thoracotoı	250	CHU	No	54	1	1
1	0	VATS or rc	250	CHU	No	76	0	0
1	0	Thoracotoı	250	CHU	No	77	0	0
1	0	Thoracotoı	250	CHU	No	63	0	0
1	1	Thoracotoı	250	CHU	Yes	70	0	0
1	0	VATS or rc	250	CHU	No	80	0	0
1	0	VATS or rc	250	CHU	Yes	58	0	0

1	1	Thoracotoı	250	CHU	No	62	0	0
1	0	Thoracotoı	250	CHU	No	78	0	0
1	0	Thoracotoı	250	CHU	No	47	0	0
1	1	Thoracotoı	250	CHU	No	65	1	0
1	0	Thoracotoı	250	CHU	No	69	0	1
1	0	Thoracotoı	250	CHU	No	64	0	0
1	0	Thoracotoı	250	CHU	No	55	0	1
1	0	VATS or rı	250	CHU	No	66	0	1
1	0	Thoracotoı	250	CHU	No	66	0	0
1	0	VATS or rı	250	CHU	No	64	0	0
1	1	Thoracotoı	250	CHU	No	64	0	1
1	0	Thoracotoı	250	CHU	No	78	0	0
1	0	Thoracotoı	250	CHU	No	76	1	0
1	0	Thoracotoı	250	CHU	No	70	0	0
1	0	Thoracotoı	250	CHU	No	79	0	0
1	0	Thoracotoı	250	CHU	No	65	0	0
1	0	Thoracotoı	250	CHU	No	79	0	0
1	0	Thoracotoı	250	CHU	No	69	0	0
1	0	VATS or rı	250	CHU	No	68	0	0
1	0	Thoracotoı	250	CHU	No	71	0	0
1	0	Thoracotoı	250	CHU	No	78	1	0
1	0	VATS or rı	250	CHU	No	52	0	0
1	0	Thoracotoı	250	CHU	Yes	68	0	0
1	0	Thoracotoı	250	CHU	No	71	0	0
1	0	Thoracotoı	250	CHU	No	43	0	1
1	0	Thoracotoı	250	CHU	No	61	0	1
1	0	Thoracotoı	250	CHU	No	58	0	1
1	0	Thoracotoı	250	CHU	No	72	0	1
1	0	VATS or rı	250	CHU	No	76	0	0
1	0	Thoracotoı	250	CHU	No	68	0	0
1	0	VATS or rı	250	CHU	No	74	0	0
1	1	Thoracotoı	250	CHU	No	85	0	0
1	0	VATS or rı	250	CHU	No	68	0	0
1	1	Thoracotoı	250	CHU	No	55	0	0
1	0	Thoracotoı	250	CHU	No	56	0	0
1	0	Thoracotoı	250	CHU	No	57	0	0
1	0	Thoracotoı	250	CHU	No	79	0	0
1	0	Thoracotoı	250	CHU	No	76	0	0
1	1	Thoracotoı	250	CHU	No	69	0	0
1	0	VATS or rı	250	CHU	No	55	0	1
1	0	Thoracotoı	250	CHU	No	56	0	0
1	0	Thoracotoı	250	CHU	No	51	0	0
1	0	VATS or rı	250	CHU	No	67	0	0
1	0	Thoracotoı	250	CHU	No	44	0	1
1	0	Thoracotoı	250	CHU	Yes	70	0	0
1	0	Thoracotoı	250	CHU	No	65	0	0
1	0	Thoracotoı	250	CHU	No	78	0	0
1	1	Thoracotoı	250	CHU	No	54	0	1
1	0	Thoracotoı	250	CHU	No	59	0	1
1	0	Thoracotoı	250	CHU	No	58	1	0
1	0	Thoracotoı	250	CHU	No	73	0	0
1	1	Thoracotoı	250	CHU	No	52	0	0
1	0	Thoracotoı	250	CHU	No	72	0	1
1	0	Thoracotoı	250	CHU	No	55	0	1
1	0	Thoracotoı	250	CHU	No	67	0	1
1	0	Thoracotoı	250	CHU	No	50	0	0

1	0	Thoracotoı	250	CHU	No	59	0	0
1	0	Thoracotoı	250	CHU	No	52	0	1
1	0	VATS or rc	250	CHU	No	50	0	0
1	1	Thoracotoı	250	CHU	No	68	1	0
1	0	Thoracotoı	250	CHU	No	75	0	0
1	1	Thoracotoı	250	CHU	No	84	0	0
1	0	Thoracotoı	250	CHU	No	49	0	1
1	0	VATS or rc	250	CHU	No	58	0	0
1	0	Thoracotoı	250	CHU	No	75	0	0
1	0	Thoracotoı	250	CHU	No	53	0	0
1	0	Thoracotoı	250	CHU	No	61	0	1
1	0	Thoracotoı	250	CHU	No	55	0	0
1	0	VATS or rc	250	CHU	No	67	0	0
1	1	Thoracotoı	250	CHU	No	63	0	1
1	0	VATS or rc	250	CHU	No	69	0	0
1	0	VATS or rc	250	CHU	No	52	0	0
1	0	VATS or rc	250	CHU	No	57	0	1
1	0	Thoracotoı	250	CHU	No	72	0	0
1	0	Thoracotoı	250	CHU	No	63	0	0
1	0	VATS or rc	250	CHU	No	71	0	0
1	0	Thoracotoı	250	CHU	No	75	0	0
1	0	Thoracotoı	250	CHU	No	60	0	0
1	0	Thoracotoı	250	CHU	No	63	0	0
1	1	Thoracotoı	250	CHU	No	73	0	1
1	0	Thoracotoı	250	CHU	No	71	0	0
1	0	Thoracotoı	250	CHU	No	60	0	1
1	0	Thoracotoı	250	CHU	No	53	0	0
1	0	Thoracotoı	250	CHU	No	76	0	0
1	0	Thoracotoı	250	CHU	No	81	0	0
1	0	Thoracotoı	250	CHU	No	61	0	0
1	0	Thoracotoı	250	CHU	No	66	0	1
1	0	Thoracotoı	250	CHU	No	74	1	0
1	0	Thoracotoı	250	CHU	No	50	0	0
1	1	Thoracotoı	250	CHU	No	60	0	0
1	0	Thoracotoı	250	CHU	No	78	0	1
1	0	Thoracotoı	250	CHU	No	69	0	0
1	1	Thoracotoı	250	CHU	No	75	0	1
1	0	Thoracotoı	250	CHU	No	51	0	0
1	0	VATS or rc	250	CHU	No	78	1	1
1	0	Thoracotoı	250	CHU	No	80	0	0
1	0	VATS or rc	250	CHU	No	71	0	1
1	0	Thoracotoı	250	CHU	No	66	1	1
1	0	Thoracotoı	250	CHU	No	74	0	0
1	0	Thoracotoı	250	CHU	No	58	0	0
1	0	Thoracotoı	250	CHU	No	62	0	0
1	0	Thoracotoı	250	CHU	No	57	0	0
1	0	VATS or rc	250	CHU	No	53	0	0
1	0	Thoracotoı	250	CHU	No	66	0	0
1	0	Thoracotoı	250	CHU	No	58	0	0
1	0	VATS or rc	250	CHU	No	73	0	1
1	0	Thoracotoı	250	CHU	No	73	0	0
1	0	Thoracotoı	250	CHU	No	55	0	0
1	0	Thoracotoı	250	CHU	No	64	0	0
1	0	Thoracotoı	250	CHU	No	63	0	0
1	0	Thoracotoı	250	CHU	No	58	0	0
1	1	Thoracotoı	250	CHU	No	54	0	0

1	0	VATS or rc	250	CHU	No	68	0	0
1	0	Thoracotoı	250	CHU	No	76	1	0
1	0	Thoracotoı	250	CHU	No	52	0	0
1	0	Thoracotoı	250	CHU	No	62	0	0
1	0	Thoracotoı	250	CHU	No	66	0	1
1	0	Thoracotoı	250	CHU	No	55	0	0
1	0	Thoracotoı	250	CHU	No	75	1	0
1	0	Thoracotoı	250	CHU	No	81	0	0
1	1	Thoracotoı	250	CHU	No	72	0	0
1	0	Thoracotoı	250	CHU	No	59	0	0
1	0	VATS or rc	250	CHU	No	68	0	0
1	0	Thoracotoı	250	CHU	No	70	0	0
1	0	Thoracotoı	250	CHU	No	52	0	1
1	0	Thoracotoı	250	CHU	No	72	0	1
1	0	Thoracotoı	250	CHU	No	60	0	1
1	0	VATS or rc	250	CHU	No	44	0	0
1	0	VATS or rc	250	CHU	No	72	0	1
1	0	Thoracotoı	250	CHU	No	64	0	0
1	1	Thoracotoı	250	CHU	No	68	0	0
1	1	Thoracotoı	250	CHU	No	62	0	1
1	0	Thoracotoı	250	CHU	No	76	0	0
1	0	Thoracotoı	250	CHU	No	71	0	0
1	0	Thoracotoı	250	CHU	No	79	0	0
1	0	VATS or rc	250	CHU	No	83	0	1
1	0	Thoracotoı	250	CHU	No	76	0	0
1	0	VATS or rc	250	CHU	No	62	0	1
1	0	Thoracotoı	250	CHU	No	60	0	0
1	0	Thoracotoı	250	CHU	No	72	0	0
1	0	VATS or rc	250	CHU	No	75	0	0
1	0	VATS or rc	250	CHU	No	64	0	0
1	0	Thoracotoı	250	CHU	No	77	0	0
1	0	Thoracotoı	250	CHU	No	69	0	0
1	0	Thoracotoı	250	CHU	No	62	0	1
1	0	Thoracotoı	250	CHU	No	72	0	0
1	1	Thoracotoı	250	CHU	No	46	0	1
1	0	Thoracotoı	250	CHU	No	63	0	0
1	0	Thoracotoı	250	CHU	No	74	0	0
1	0	Thoracotoı	250	CHU	No	77	0	1
1	1	Thoracotoı	250	CHU	No	68	0	0
1	0	VATS or rc	250	CHU	No	64	1	1
1	1	Thoracotoı	250	CHU	No	60	0	1
1	0	Thoracotoı	250	CHU	No	69	0	0
1	0	VATS or rc	250	CHU	No	46	0	0
1	0	Thoracotoı	250	CHU	Yes	78	0	0
1	0	VATS or rc	250	CHU	No	60	0	0
1	0	Thoracotoı	250	CHU	No	80	0	0
1	0	Thoracotoı	250	CHU	No	60	0	0
1	0	Thoracotoı	250	CHU	No	72	1	0
1	0	Thoracotoı	250	CHU	No	76	0	0
1	1	Thoracotoı	250	CHU	No	75	0	0
1	0	Thoracotoı	250	CHU	No	59	0	1
1	1	Thoracotoı	250	CHU	No	58	0	0
1	0	Thoracotoı	250	CHU	No	70	0	0
1	0	VATS or rc	250	CHU	No	66	0	0
1	0	Thoracotoı	250	CHU	No	57	0	0
1	0	VATS or rc	250	CHU	No	48	0	0

1	0	Thoracotor	250	CHU	No	59	1	1
1	0	Thoracotor	250	CHU	No	77	0	0
1	0	Thoracotor	250	CHU	No	62	0	0
1	0	Thoracotor	250	CHU	No	72	0	0
1	0	Thoracotor	250	CHU	No	75	0	0
1	0	VATS or ro	250	CHU	No	80	0	0
1	1	Thoracotor	250	CHU	No	59	0	0
1	1	Thoracotor	250	CHU	No	49	0	0
1	0	VATS or ro	250	CHU	No	78	0	0
1	0	Thoracotor	250	CHU	No	59	0	0
1	0	Thoracotor	250	CHU	No	69	0	0
1	0	Thoracotor	250	CHU	No	53	0	0
1	0	Thoracotor	250	CHU	No	62	0	0
1	0	Thoracotor	250	CHU	No	56	0	0
1	0	Thoracotor	250	CHU	No	74	1	0
1	0	Thoracotor	250	CHU	No	60	0	1
1	1	Thoracotor	250	CHU	No	70	0	0
1	0	Thoracotor	250	CHU	No	59	0	1
1	0	Thoracotor	250	CHU	No	79	0	0
1	0	Thoracotor	250	CHU	No	57	0	1
1	0	Thoracotor	250	CHU	No	52	0	1
1	0	Thoracotor	250	CHU	No	74	0	0
1	1	Thoracotor	250	CHU	No	62	0	1
1	0	Thoracotor	250	CHU	No	65	0	1
1	0	Thoracotor	250	CHU	No	76	0	0
1	1	Thoracotor	250	CHU	No	81	0	0
1	0	Thoracotor	250	CHU	No	77	0	0
1	1	Thoracotor	250	CHU	No	48	0	1
1	0	Thoracotor	250	CHU	No	67	0	0
1	0	VATS or ro	250	CHU	No	65	0	0
1	1	Thoracotor	250	CHU	No	60	0	1
1	0	VATS or ro	250	CHU	No	72	1	0
1	0	VATS or ro	250	CHU	No	68	0	0
1	0	Thoracotor	250	CHU	No	78	0	0
1	0	VATS or ro	250	CHU	No	59	0	1
1	0	VATS or ro	250	CHU	No	59	0	0
1	0	Thoracotor	250	CHU	No	53	0	1
1	0	Thoracotor	250	CHU	No	58	0	0
1	0	Thoracotor	250	CHU	No	77	0	0
1	0	VATS or ro	250	CHU	No	76	0	0
1	0	Thoracotor	250	CHU	No	68	0	0
1	0	VATS or ro	250	CHU	No	49	0	1
1	0	Thoracotor	250	CHU	No	60	0	0
1	0	Thoracotor	250	CHU	No	80	0	1
1	0	Thoracotor	250	CHU	No	69	1	0
1	0	VATS or ro	250	CHU	No	62	0	0
1	0	Thoracotor	250	CHU	No	69	0	0
1	0	Thoracotor	250	CHU	No	69	0	0
1	0	Thoracotor	250	CHU	No	81	0	0
1	0	Thoracotor	250	CHU	No	83	0	0
1	0	VATS or ro	250	CHU	No	72	1	0
1	1	Thoracotor	250	CHU	No	84	0	0
1	0	VATS or ro	250	CHU	No	67	0	0
1	0	Thoracotor	250	CHU	No	64	0	1
1	0	Thoracotor	250	CHU	No	51	0	0
1	0	Thoracotor	250	CHU	No	65	0	0

1	0	Thoracotoı	250	CHU	No	56	0	1
1	0	VATS or rc	250	CHU	No	68	0	0
1	0	Thoracotoı	250	CHU	No	72	0	0
1	0	Thoracotoı	250	CHU	No	63	0	1
1	1	Thoracotoı	250	CHU	No	61	0	0
1	0	Thoracotoı	250	CHU	No	60	0	0
1	0	Thoracotoı	250	CHU	No	57	0	0
1	0	Thoracotoı	250	CHU	No	60	0	0
1	0	Thoracotoı	250	CHU	No	55	0	1
1	0	Thoracotoı	250	CHU	No	74	0	0
1	0	Thoracotoı	250	CHU	No	67	0	0
1	0	Thoracotoı	250	CHU	No	47	0	0
1	0	Thoracotoı	250	CHU	No	72	0	1
1	0	Thoracotoı	250	CHU	No	69	1	0
1	0	VATS or rc	250	CHU	No	70	0	0
1	1	Thoracotoı	250	CHU	No	68	0	0
1	1	Thoracotoı	250	CHU	No	69	0	0
1	0	Thoracotoı	250	CHU	No	51	0	0
1	1	Thoracotoı	250	CHU	No	63	0	0
1	0	Thoracotoı	250	CHU	No	65	0	1
1	0	Thoracotoı	250	CHU	No	72	0	0
1	1	Thoracotoı	250	CHU	No	75	0	0
1	0	Thoracotoı	250	CHU	No	80	0	1
1	0	Thoracotoı	250	CHU	No	65	0	0
1	0	Thoracotoı	250	CHU	No	59	0	0
1	0	Thoracotoı	250	CHU	No	64	0	0
1	0	Thoracotoı	250	CHU	No	66	0	1
1	1	Thoracotoı	250	CHU	No	62	0	0
1	0	Thoracotoı	250	CHU	No	62	1	0
1	0	Thoracotoı	250	CHU	No	62	0	0
1	0	Thoracotoı	250	CHU	No	59	0	1
1	0	Thoracotoı	250	CHU	No	76	0	0
1	0	VATS or rc	250	CHU	No	80	1	0
1	0	Thoracotoı	250	CHU	No	76	0	0
1	0	Thoracotoı	250	CHU	No	54	0	1
1	0	VATS or rc	250	CHU	No	65	0	0
1	0	Thoracotoı	250	CHU	No	78	0	0
1	1	Thoracotoı	250	CHU	No	61	0	0
1	0	Thoracotoı	250	CHU	No	83	0	0
1	1	Thoracotoı	250	CHU	No	63	0	0
1	0	Thoracotoı	250	CHU	No	59	0	1
1	0	Thoracotoı	250	CHU	No	76	0	0
1	0	VATS or rc	250	CHU	No	63	0	0
1	0	Thoracotoı	250	CHU	No	64	0	0
1	0	Thoracotoı	250	CHU	No	62	0	0
1	0	VATS or rc	250	CHU	No	69	1	0
1	0	VATS or rc	250	CHU	No	41	0	0
1	0	Thoracotoı	250	CHU	No	76	0	0
1	0	VATS or rc	250	CHU	No	64	0	0
1	0	VATS or rc	250	CHU	No	64	0	1
1	0	Thoracotoı	250	CHU	No	55	0	0
1	0	Thoracotoı	250	CHU	No	56	0	1
1	0	Thoracotoı	250	CHU	No	69	0	0
1	0	VATS or rc	250	CHU	No	62	0	1
1	0	Thoracotoı	250	CHU	No	69	0	0
1	0	Thoracotoı	250	CHU	No	58	0	0

1	0	Thoracotoı	250	CHU	No	43	0	1
1	1	Thoracotoı	250	CHU	No	67	0	0
1	0	Thoracotoı	250	CHU	No	58	0	0
1	0	Thoracotoı	250	CHU	No	51	0	0
1	1	Thoracotoı	250	CHU	No	56	0	0
1	0	Thoracotoı	250	CHU	No	78	0	0
1	0	Thoracotoı	250	CHU	No	54	0	1
1	0	Thoracotoı	250	CHU	No	62	0	0
1	0	Thoracotoı	250	CHU	No	55	1	0
1	0	Thoracotoı	250	CHU	No	60	0	0
1	0	Thoracotoı	250	CHU	No	79	0	1
1	0	Thoracotoı	250	CHU	No	68	1	0
1	1	Thoracotoı	250	CHU	No	72	0	0
1	0	VATS or rc	250	CHU	No	54	0	0
1	0	Thoracotoı	250	CHU	No	56	0	0
1	0	Thoracotoı	250	CHU	No	78	0	0
1	1	Thoracotoı	250	CHU	No	59	0	1
1	0	Thoracotoı	250	CHU	No	62	0	0
1	0	Thoracotoı	250	CHU	No	53	0	1
1	0	Thoracotoı	250	CHU	No	69	0	0
1	0	Thoracotoı	250	CHU	No	62	0	0
1	0	Thoracotoı	250	CHU	No	66	0	1
1	0	Thoracotoı	250	CHU	No	61	0	1
1	1	Thoracotoı	250	CHU	No	64	0	0
1	0	Thoracotoı	250	CHU	No	62	0	0
1	1	Thoracotoı	250	CHU	No	59	0	0
1	1	Thoracotoı	250	CHU	No	52	1	1
1	0	Thoracotoı	250	CHU	No	66	0	1
1	0	Thoracotoı	250	CHU	No	63	0	0
1	0	Thoracotoı	250	CHU	No	67	0	1
1	0	Thoracotoı	250	CHU	No	58	0	1
1	0	VATS or rc	250	CHU	No	68	0	1
1	0	Thoracotoı	250	CHU	No	73	0	1
1	0	Thoracotoı	250	CHU	No	55	0	0
1	0	VATS or rc	250	CHU	No	74	0	0
1	0	Thoracotoı	250	CHU	No	72	0	0
1	1	Thoracotoı	250	CHU	No	57	0	0
1	0	Thoracotoı	250	CHU	No	75	0	0
1	0	Thoracotoı	250	CHU	No	51	0	0
1	0	Thoracotoı	250	CHU	No	55	0	1
1	0	Thoracotoı	250	CHU	No	60	0	0
1	0	VATS or rc	250	CHU	No	67	0	0
1	0	Thoracotoı	250	CHU	No	52	0	1
1	0	Thoracotoı	250	CHU	No	49	0	0
1	0	Thoracotoı	250	CHU	No	77	0	0
1	0	Thoracotoı	250	CHU	No	59	0	0
1	0	Thoracotoı	250	CHU	No	64	0	1
1	0	Thoracotoı	250	CHU	No	69	0	0
1	0	Thoracotoı	250	CHU	No	65	0	1
1	0	Thoracotoı	250	CHU	No	60	0	1
1	1	Thoracotoı	250	CHU	No	49	0	0
1	0	Thoracotoı	250	CHU	No	53	0	0
1	0	Thoracotoı	250	CHU	No	81	0	1
1	0	VATS or rc	250	CHU	No	70	0	0
1	1	Thoracotoı	250	CHU	No	52	0	0
1	0	Thoracotoı	250	CHU	No	63	0	1

1	0	Thoracotoı	250	CHU	No	70	0	0
1	0	Thoracotoı	250	CHU	No	57	0	1
1	0	Thoracotoı	250	CHU	No	58	0	1
1	1	Thoracotoı	250	CHU	No	59	0	1
1	0	VATS or rc	250	CHU	No	82	0	0
1	0	Thoracotoı	250	CHU	No	73	0	1
1	0	VATS or rc	250	CHU	No	69	0	1
1	0	Thoracotoı	250	CHU	No	57	0	0
1	1	Thoracotoı	250	CHU	No	62	0	0
1	0	Thoracotoı	250	CHU	No	73	0	0
1	0	Thoracotoı	250	CHU	No	66	0	0
1	0	Thoracotoı	250	CHU	No	57	0	0
1	0	VATS or rc	250	CHU	No	80	0	0
1	0	Thoracotoı	250	CHU	No	78	0	0
1	1	Thoracotoı	250	CHU	No	65	0	0
1	0	Thoracotoı	250	CHU	No	56	0	0
1	0	VATS or rc	250	CHU	No	68	0	0
1	0	Thoracotoı	250	CHU	No	54	0	0
1	0	Thoracotoı	250	CHU	No	64	0	0
1	1	Thoracotoı	250	CHU	No	69	0	0
1	0	Thoracotoı	250	CHU	No	71	0	0
1	0	Thoracotoı	250	CHU	No	65	0	0
1	1	Thoracotoı	250	CHU	No	51	0	0
1	0	Thoracotoı	250	CHU	No	67	0	0
1	0	Thoracotoı	250	CHU	No	61	0	0
1	0	Thoracotoı	250	CHU	No	57	0	0
1	0	Thoracotoı	250	CHU	No	66	1	0
1	1	Thoracotoı	250	CHU	No	70	0	0
1	0	VATS or rc	250	CHU	No	66	1	0
1	0	Thoracotoı	250	CHU	No	60	0	1
1	0	Thoracotoı	250	CHU	No	79	0	0
1	0	Thoracotoı	250	CHU	No	62	0	0
1	0	Thoracotoı	250	CHU	No	58	0	1
1	0	VATS or rc	250	CHU	No	58	0	0
1	1	Thoracotoı	250	CHU	No	68	0	1
1	0	Thoracotoı	250	CHU	No	54	0	0
1	0	VATS or rc	250	CHU	No	67	1	0
1	0	Thoracotoı	250	CHU	No	63	0	0
1	1	Thoracotoı	250	CHU	No	58	1	1
1	0	Thoracotoı	250	CHU	No	50	0	1
1	0	Thoracotoı	250	CHU	No	68	0	1
1	0	Thoracotoı	250	CHU	No	49	0	0
1	0	Thoracotoı	250	CHU	No	82	0	0
1	0	Thoracotoı	250	CHU	No	82	0	0
1	0	Thoracotoı	250	CHU	No	58	0	0
1	0	VATS or rc	250	CHU	No	70	0	0
1	0	Thoracotoı	250	CHU	No	75	0	1
1	0	Thoracotoı	250	CHU	No	68	0	0
1	0	VATS or rc	250	CHU	No	64	0	0
1	0	Thoracotoı	250	CHU	No	51	0	0
1	0	Thoracotoı	250	CHU	No	69	0	0
1	0	Thoracotoı	250	CHU	No	64	0	0
1	0	Thoracotoı	250	CHU	No	57	0	1
1	0	Thoracotoı	250	CHU	No	65	0	0
1	0	VATS or rc	250	CHU	No	61	0	0
1	0	Thoracotoı	250	CHU	No	80	0	0

1	0	Thoracotoı	250	CHU	No	48	0	0
1	0	Thoracotoı	250	CHU	No	67	0	0
1	1	Thoracotoı	250	CHU	Yes	40	0	1
1	0	Thoracotoı	250	CHU	No	43	0	1
1	0	Thoracotoı	250	CHU	No	58	0	0
1	0	Thoracotoı	250	CHU	No	72	0	0
1	1	Thoracotoı	250	CHU	No	63	0	1
1	1	Thoracotoı	250	CHU	No	73	0	0
1	0	VATS or rc	250	CHU	No	67	0	0
1	0	Thoracotoı	250	CHU	No	77	0	0
1	0	Thoracotoı	250	CHU	No	75	0	0
1	0	Thoracotoı	250	CHU	No	68	0	0
1	0	Thoracotoı	250	CHU	No	77	0	0
1	0	Thoracotoı	250	CHU	No	65	0	0
1	0	Thoracotoı	250	CHU	No	78	0	0
1	0	Thoracotoı	250	CHU	No	61	0	0
1	0	Thoracotoı	250	CHU	No	64	0	0
1	0	VATS or rc	250	CHU	No	50	0	1
1	0	Thoracotoı	250	CHU	No	70	0	0
1	1	Thoracotoı	250	CHU	No	51	0	1
1	0	Thoracotoı	250	CHU	No	66	0	0
1	0	Thoracotoı	250	CHU	No	50	0	1
1	1	Thoracotoı	250	CHU	No	65	0	1
1	0	Thoracotoı	250	CHU	No	69	0	1
1	0	Thoracotoı	250	CHU	No	55	0	0
1	0	Thoracotoı	250	CHU	No	59	0	0
1	0	Thoracotoı	250	CHU	No	67	0	1
1	0	Thoracotoı	250	CHU	No	73	0	1
1	0	Thoracotoı	250	CHU	No	63	0	0
1	0	Thoracotoı	250	CHU	No	69	0	0
1	0	Thoracotoı	250	CHU	No	42	0	0
1	0	Thoracotoı	250	CHU	No	71	0	0
1	0	Thoracotoı	250	CHU	Yes	63	0	1
1	0	Thoracotoı	250	CHU	No	62	0	0
1	0	Thoracotoı	250	CHU	No	63	0	0
1	0	Thoracotoı	250	CHU	No	67	0	0
1	0	Thoracotoı	250	CHU	No	55	0	1
1	0	Thoracotoı	250	CHU	No	60	0	0
1	0	VATS or rc	250	CHU	No	63	0	0
1	0	Thoracotoı	250	CHU	No	62	0	0
1	0	Thoracotoı	250	CHU	No	59	0	1
1	0	VATS or rc	250	CHU	No	70	0	0
1	0	Thoracotoı	250	CHU	No	48	0	1
1	0	VATS or rc	250	CHU	No	62	0	0
1	0	VATS or rc	250	CHU	No	75	0	0
1	0	Thoracotoı	250	CHU	No	67	0	0
1	0	Thoracotoı	250	CHU	No	69	0	0
1	0	VATS or rc	250	CHU	No	51	0	0
1	0	Thoracotoı	250	CHU	No	53	0	0
1	1	Thoracotoı	250	CHU	No	67	0	0
1	1	Thoracotoı	250	CHU	No	62	0	0
1	0	Thoracotoı	250	CHU	No	62	0	0
1	0	Thoracotoı	250	CHU	No	73	0	0
1	0	Thoracotoı	250	CHU	No	69	0	1
1	0	Thoracotoı	250	CHU	No	67	0	0
1	0	Thoracotoı	250	CHU	No	77	0	0

1	0	Thoracotoı	250	CHU	No	76	0	0
1	0	Thoracotoı	250	CHU	No	67	0	0
1	0	Thoracotoı	250	CHU	No	53	0	0
1	0	Thoracotoı	250	CHU	No	65	0	0
1	0	Thoracotoı	250	CHU	No	66	0	0
1	0	VATS or rc	250	CHU	No	58	0	1
1	0	Thoracotoı	250	CHU	No	51	0	1
1	0	Thoracotoı	250	CHU	No	67	0	0
1	0	Thoracotoı	250	CHU	No	59	0	0
1	0	Thoracotoı	250	CHU	No	53	0	0
1	0	VATS or rc	250	CHU	No	76	0	1
1	0	VATS or rc	250	CHU	No	58	0	0
1	1	Thoracotoı	250	CHU	No	66	0	0
1	0	Thoracotoı	250	CHU	No	75	0	0
1	0	Thoracotoı	250	CHU	No	69	0	0
1	0	VATS or rc	250	CHU	No	65	0	0
1	0	VATS or rc	250	CHU	No	62	0	0
1	0	Thoracotoı	250	CHU	No	57	0	0
1	0	Thoracotoı	250	CHU	No	73	0	0
1	0	Thoracotoı	250	CHU	No	62	0	0
1	0	VATS or rc	250	CHU	No	74	0	0
1	0	Thoracotoı	250	CHU	No	56	0	1
1	1	Thoracotoı	250	CHU	No	71	0	1
1	0	Thoracotoı	250	CHU	No	62	0	0
1	0	Thoracotoı	250	CHU	No	67	0	1
1	1	Thoracotoı	250	CHU	No	52	0	1
1	0	Thoracotoı	250	CHU	No	67	0	0
1	0	Thoracotoı	250	CHU	No	64	0	0
1	0	Thoracotoı	250	CHU	No	58	0	0
1	0	VATS or rc	250	CHU	No	75	1	0
1	0	VATS or rc	250	CHU	No	69	0	1
1	0	Thoracotoı	250	CHU	No	44	0	1
1	0	Thoracotoı	250	CHU	No	56	0	0
1	0	Thoracotoı	250	CHU	No	71	0	0
1	0	Thoracotoı	250	CHU	No	64	0	1
1	0	Thoracotoı	250	CHU	No	62	0	0
1	1	Thoracotoı	250	CHU	No	68	0	0
1	0	Thoracotoı	250	CHU	No	54	0	0
1	0	Thoracotoı	250	CHU	No	64	0	1
1	0	Thoracotoı	250	CHU	No	57	0	0
1	0	Thoracotoı	250	CHU	No	58	0	0
1	0	Thoracotoı	250	CHU	No	62	0	1
1	0	Thoracotoı	250	CHU	No	74	0	0
1	0	Thoracotoı	250	CHU	No	71	0	0
1	0	Thoracotoı	250	CHU	No	71	0	0
1	0	Thoracotoı	250	CHU	No	61	0	0
1	0	VATS or rc	250	CHU	No	52	0	0
1	0	Thoracotoı	250	CHU	No	74	0	0
1	1	Thoracotoı	250	CHU	No	67	0	0
1	0	Thoracotoı	250	CHU	No	58	0	0
1	0	Thoracotoı	250	CHU	No	62	0	1
1	0	Thoracotoı	250	CHU	No	60	1	0
1	1	Thoracotoı	250	CHU	No	63	0	0
1	0	Thoracotoı	250	CHU	No	71	0	0
1	0	VATS or rc	250	CHU	No	68	0	0
1	0	Thoracotoı	250	CHU	No	45	0	0

1	1	Thoracotoı	250	CHU	No	66	0	0
1	1	Thoracotoı	250	CHU	No	64	0	0
1	1	Thoracotoı	250	CHU	No	57	0	0
1	0	Thoracotoı	250	CHU	Yes	82	0	0
1	0	Thoracotoı	250	CHU	No	68	0	0
1	0	VATS or rc	250	CHU	No	58	0	0
1	0	Thoracotoı	250	CHU	No	73	0	0
1	0	Thoracotoı	250	CHU	No	68	0	0
1	0	Thoracotoı	250	CHU	No	44	0	1
1	0	Thoracotoı	250	CHU	No	72	0	1
1	0	Thoracotoı	250	CHU	No	60	0	0
1	0	Thoracotoı	250	CHU	No	71	0	1
1	1	Thoracotoı	250	CHU	No	60	0	1
1	0	Thoracotoı	250	CHU	No	25	0	1
1	0	Thoracotoı	250	CHU	No	75	0	0
1	0	Thoracotoı	250	CHU	No	65	0	0
1	0	Thoracotoı	250	CHU	No	55	1	0
1	0	VATS or rc	250	CHU	No	66	0	0
1	0	Thoracotoı	250	CHU	No	52	0	1
1	0	Thoracotoı	250	CHU	No	65	0	0
1	0	Thoracotoı	250	CHU	No	67	0	0
1	0	Thoracotoı	250	CHU	No	62	0	0
1	0	Thoracotoı	250	CHU	No	71	0	0
1	0	Thoracotoı	250	CHU	No	64	0	0
1	0	Thoracotoı	250	CHU	No	60	0	0
1	0	Thoracotoı	250	CHU	No	59	0	0
1	0	Thoracotoı	250	CHU	No	47	0	0
1	0	Thoracotoı	250	CHU	No	72	0	1
1	1	Thoracotoı	250	CHU	No	64	0	0
1	0	Thoracotoı	250	CHU	No	66	0	1
1	0	Thoracotoı	250	CHU	No	74	0	0
1	0	Thoracotoı	250	CHU	No	58	0	1
1	0	VATS or rc	250	CHU	No	70	0	0
1	0	Thoracotoı	250	CHU	No	78	0	1
1	0	VATS or rc	250	CHU	No	73	0	0
1	0	VATS or rc	250	CHU	No	60	0	0
1	0	Thoracotoı	250	CHU	No	70	0	0
1	0	VATS or rc	250	CHU	No	75	0	0
1	0	VATS or rc	250	CHU	No	68	0	0
1	0	Thoracotoı	250	CHU	No	78	0	0
1	0	Thoracotoı	250	CHU	No	60	0	0
1	0	VATS or rc	250	CHU	No	71	1	1
1	0	VATS or rc	250	CHU	No	73	1	0
1	0	Thoracotoı	250	CHU	No	78	0	1
1	0	Thoracotoı	250	CHU	No	77	0	0
1	0	Thoracotoı	250	CHU	No	54	0	0
1	0	Thoracotoı	250	CHU	No	58	0	0
1	0	VATS or rc	250	CHU	No	70	0	0
1	0	VATS or rc	250	CHU	No	57	0	0
1	1	Thoracotoı	250	CHU	No	71	0	0
1	1	Thoracotoı	250	CHU	No	56	0	0
1	0	VATS or rc	250	CHU	No	52	0	0
1	0	VATS or rc	250	CHU	No	62	0	0
1	0	Thoracotoı	250	CHU	No	57	0	0
1	1	Thoracotoı	250	CHU	No	62	0	0
1	0	Thoracotoı	250	CHU	No	54	0	0

1	0	Thoracotoı	250	CHU	No	73	1	0
1	0	Thoracotoı	250	CHU	No	66	0	0
1	1	Thoracotoı	250	CHU	No	69	0	0
1	0	Thoracotoı	250	CHU	No	75	0	0
1	0	Thoracotoı	250	CHU	Yes	60	0	0
1	0	VATS or rc	250	CHU	No	66	0	1
1	0	Thoracotoı	250	CHU	No	64	0	0
1	0	VATS or rc	250	CHU	No	75	0	0
1	0	VATS or rc	250	CHU	No	76	0	0
1	0	VATS or rc	250	CHU	No	69	0	1
1	0	Thoracotoı	250	CHU	No	55	0	0
1	0	Thoracotoı	250	CHU	No	63	0	0
1	1	Thoracotoı	250	CHU	No	53	0	0
1	0	Thoracotoı	250	CHU	No	71	0	0
1	0	Thoracotoı	250	CHU	No	66	0	0
1	0	VATS or rc	250	CHU	No	66	0	0
1	1	Thoracotoı	250	CHU	No	68	0	0
1	0	Thoracotoı	250	CHU	No	70	1	0
1	0	Thoracotoı	250	CHU	No	61	1	1
1	0	Thoracotoı	250	CHU	No	33	0	0
1	0	Thoracotoı	250	CHU	No	65	0	0
1	1	Thoracotoı	250	CHU	No	56	0	0
1	0	VATS or rc	250	CHU	No	58	0	0
1	0	Thoracotoı	250	CHU	No	47	0	0
1	0	Thoracotoı	250	CHU	No	65	0	1
1	0	Thoracotoı	250	CHU	No	68	1	0
1	1	Thoracotoı	250	CHU	No	58	0	0
1	0	Thoracotoı	250	CHU	No	56	0	0
1	1	Thoracotoı	250	CHU	No	70	0	0
1	0	Thoracotoı	250	CHU	No	29	0	1
1	0	Thoracotoı	250	CHU	No	66	0	0
1	0	Thoracotoı	250	CHU	No	55	0	0
1	0	VATS or rc	250	CHU	No	72	1	0
1	0	Thoracotoı	250	CHU	No	64	1	0
1	0	Thoracotoı	250	CHU	No	58	0	1
1	0	Thoracotoı	250	CHU	No	82	0	0
1	0	Thoracotoı	250	CHU	No	63	0	0
1	0	Thoracotoı	250	CHU	No	75	0	0
1	0	VATS or rc	250	CHU	No	42	0	0
1	0	Thoracotoı	250	CHU	No	69	0	1
1	0	Thoracotoı	250	CHU	No	75	0	0
1	0	Thoracotoı	250	CHU	No	60	0	0
1	0	Thoracotoı	250	CHU	No	79	0	0
1	0	Thoracotoı	250	CHU	No	68	0	0
1	1	Thoracotoı	250	CHU	No	45	0	1
1	0	Thoracotoı	250	CHU	No	66	0	0
1	0	VATS or rc	250	CHU	No	56	0	1
1	0	Thoracotoı	250	CHU	No	52	1	0
1	0	Thoracotoı	250	CHU	No	75	0	0
1	0	Thoracotoı	250	CHU	No	68	1	1
1	0	VATS or rc	250	CHU	No	63	0	0
1	0	Thoracotoı	250	CHU	No	63	0	1
1	0	Thoracotoı	250	CHU	No	67	1	0
1	0	Thoracotoı	250	CHU	No	43	0	0
1	0	Thoracotoı	250	CHU	No	75	0	0
1	1	Thoracotoı	250	CHU	No	59	1	0

1	1	Thoracotoı	250	CHU	No	46	0	1
1	0	Thoracotoı	250	CHU	No	67	0	0
1	0	VATS or rc	250	CHU	No	72	1	0
1	1	Thoracotoı	250	CHU	No	50	0	0
1	0	Thoracotoı	250	CHU	No	63	0	0
1	0	VATS or rc	250	CHU	No	39	0	0
1	0	Thoracotoı	250	CHU	No	67	0	0
1	0	Thoracotoı	250	CHU	No	48	0	0
1	0	Thoracotoı	250	CHU	No	73	0	0
1	0	Thoracotoı	250	CHU	No	64	0	1
1	0	Thoracotoı	250	CHU	No	76	0	0
1	0	Thoracotoı	250	CHU	No	65	0	0
1	0	Thoracotoı	250	CHU	No	84	0	0
1	0	Thoracotoı	250	CHU	No	60	0	1
1	0	Thoracotoı	250	CHU	No	65	0	0
1	0	VATS or rc	250	CHU	No	52	0	1
1	0	VATS or rc	250	CHU	No	66	0	1
1	0	Thoracotoı	250	CHU	No	60	0	0
1	0	Thoracotoı	250	CHU	No	44	0	1
1	0	Thoracotoı	250	CHU	No	70	1	1
1	0	Thoracotoı	250	CHU	No	63	0	0
1	0	VATS or rc	250	CHU	No	59	0	1
1	0	Thoracotoı	250	CHU	No	78	0	0
1	0	Thoracotoı	250	CHU	No	58	1	0
1	1	Thoracotoı	250	CHU	No	71	0	0
1	0	Thoracotoı	250	CHU	No	77	0	0
1	0	Thoracotoı	250	CHU	No	60	0	0
1	0	Thoracotoı	250	CHU	No	45	0	1
1	0	Thoracotoı	250	CHU	No	72	0	0
1	0	VATS or rc	250	CHU	No	72	0	1
1	0	Thoracotoı	250	CHU	No	50	0	0
1	0	Thoracotoı	250	CHU	No	65	0	0
1	0	VATS or rc	250	CHU	No	71	0	0
1	0	VATS or rc	250	CHU	No	64	0	0
1	0	Thoracotoı	250	CHU	No	61	0	0
1	0	Thoracotoı	250	CHU	No	49	0	0
1	0	VATS or rc	250	CHU	No	73	0	0
1	0	Thoracotoı	250	CHU	No	53	0	0
1	0	Thoracotoı	250	CHU	No	67	0	0
1	0	VATS or rc	250	CHU	No	69	0	0
1	0	Thoracotoı	250	CHU	No	63	0	0
1	0	VATS or rc	250	CHU	No	72	0	1
1	0	Thoracotoı	250	CHU	Yes	76	0	0
1	0	VATS or rc	250	CHU	No	58	0	1
1	0	Thoracotoı	250	CHU	No	66	0	0
1	0	Thoracotoı	250	CHU	No	52	0	0
1	0	Thoracotoı	250	CHU	No	57	0	0
1	0	VATS or rc	250	CHU	No	68	0	0
1	0	Thoracotoı	250	CHU	No	72	0	0
1	0	Thoracotoı	250	CHU	No	53	0	1
1	0	Thoracotoı	250	CHU	Yes	62	0	0
1	0	Thoracotoı	250	CHU	No	53	0	1
1	0	Thoracotoı	250	CHU	No	68	0	0
1	0	Thoracotoı	250	CHU	No	74	0	0
1	1	Thoracotoı	250	CHU	No	74	0	0
1	0	Thoracotoı	250	CHU	No	68	0	0

1	0	Thoracotoı	250	CHU	No	64	0	1
1	0	Thoracotoı	250	CHU	No	44	0	1
1	0	Thoracotoı	250	CHU	No	79	0	0
1	0	VATS or rc	250	CHU	No	56	0	1
1	0	Thoracotoı	250	CHU	No	57	0	0
1	0	Thoracotoı	250	CHU	No	57	0	1
1	0	Thoracotoı	250	CHU	No	77	0	0
1	1	Thoracotoı	250	CHU	No	54	0	0
1	0	Thoracotoı	250	CHU	No	72	0	0
1	0	Thoracotoı	250	CHU	No	64	0	0
1	0	Thoracotoı	250	CHU	No	78	0	1
1	0	Thoracotoı	250	CHU	No	49	0	0
1	0	VATS or rc	250	CHU	No	61	0	0
1	0	Thoracotoı	250	CHU	No	50	0	0
1	0	Thoracotoı	250	CHU	No	51	0	0
1	0	VATS or rc	250	CHU	No	76	0	0
1	0	VATS or rc	250	CHU	No	67	0	0
1	0	Thoracotoı	250	CHU	No	57	0	1
1	0	Thoracotoı	250	CHU	No	61	0	1
1	0	Thoracotoı	250	CHU	No	61	0	0
1	1	Thoracotoı	250	CHU	No	61	0	0
1	0	Thoracotoı	250	CHU	No	61	0	0
1	0	Thoracotoı	250	CHU	No	77	0	0
1	0	Thoracotoı	250	CHU	No	69	0	1
1	0	Thoracotoı	250	CHU	No	54	0	0
1	0	Thoracotoı	250	CHU	No	72	0	0
1	0	VATS or rc	250	CHU	No	63	0	0
1	1	VATS or rc	250	CHU	No	55	0	1
1	0	Thoracotoı	250	CHU	No	72	0	0
1	1	Thoracotoı	250	CHU	No	59	0	0
1	1	Thoracotoı	250	CHU	No	67	0	0
1	0	Thoracotoı	250	CHU	No	52	0	0
1	0	Thoracotoı	250	CHU	No	77	0	0
1	0	Thoracotoı	250	CHU	No	62	0	0
1	0	Thoracotoı	250	CHU	No	69	1	0
1	0	Thoracotoı	250	CHU	No	76	0	0
1	0	Thoracotoı	250	CHU	No	70	0	0
1	0	VATS or rc	250	CHU	No	67	0	0
1	0	Thoracotoı	250	CHU	No	41	0	1
1	1	Thoracotoı	250	CHU	No	73	0	0
1	1	Thoracotoı	250	CHU	No	69	0	0
1	0	Thoracotoı	250	CHU	No	72	0	0
1	0	Thoracotoı	250	CHU	No	66	0	1
1	0	Thoracotoı	250	CHU	No	66	0	0
1	0	VATS or rc	250	CHU	No	63	0	1
1	0	Thoracotoı	250	CHU	No	48	0	0
1	0	Thoracotoı	250	CHU	No	54	0	0
1	1	Thoracotoı	250	CHU	No	52	0	1
1	0	Thoracotoı	250	CHU	No	56	0	0
1	0	Thoracotoı	250	CHU	No	67	0	0
1	0	Thoracotoı	250	CHU	No	59	0	0
1	1	Thoracotoı	250	CHU	No	66	0	0
1	0	VATS or rc	250	CHU	No	66	0	0
1	0	Thoracotoı	250	CHU	No	77	0	0
1	0	Thoracotoı	250	CHU	No	75	0	0
1	0	Thoracotoı	250	CHU	No	73	0	1

1	0	Thoracotoı	250	CHU	No	62	0	0
1	0	VATS or rc	250	CHU	No	49	0	0
1	0	Thoracotoı	250	CHU	No	65	0	0
1	0	VATS or rc	250	CHU	No	73	0	0
1	1	Thoracotoı	250	CHU	No	57	0	1
1	0	VATS or rc	250	CHU	No	67	0	0
1	0	Thoracotoı	250	CHU	Yes	58	0	0
1	0	Thoracotoı	250	CHU	No	64	0	0
1	1	Thoracotoı	250	CHU	Yes	58	0	0
1	0	Thoracotoı	250	CHU	No	71	0	0
1	0	Thoracotoı	250	CHU	No	73	0	0
1	0	Thoracotoı	250	CHU	No	70	0	0
1	0	Thoracotoı	250	CHU	No	66	1	0
1	0	VATS or rc	250	CHU	No	71	0	0
1	0	Thoracotoı	250	CHU	No	54	0	0
1	0	VATS or rc	250	CHU	No	62	0	0
1	0	VATS or rc	250	CHU	No	76	0	1
1	0	Thoracotoı	250	CHU	No	75	0	0
1	0	Thoracotoı	250	CHU	No	80	0	0
1	0	VATS or rc	250	CHU	No	52	0	0
1	0	Thoracotoı	250	CHU	No	53	0	0
1	0	Thoracotoı	250	CHU	No	69	0	0
1	0	Thoracotoı	250	CHU	No	63	0	0
1	0	Thoracotoı	250	CHU	No	64	0	0
1	0	Thoracotoı	250	CHU	No	63	1	0
1	1	Thoracotoı	250	CHU	No	60	0	0
1	0	Thoracotoı	250	CHU	No	57	0	0
1	0	Thoracotoı	250	CHU	No	71	0	0
1	0	VATS or rc	250	CHU	No	77	0	0
1	0	Thoracotoı	250	CHU	No	69	0	1
1	0	Thoracotoı	250	CHU	No	64	0	0
1	0	Thoracotoı	250	CHU	No	61	0	0
1	0	Thoracotoı	250	CHU	No	68	0	0
1	1	Thoracotoı	250	CHU	No	61	0	0
1	0	VATS or rc	250	CHU	No	66	0	0
1	0	Thoracotoı	250	CHU	No	68	0	0
1	1	Thoracotoı	250	CHU	No	60	0	0
1	0	Thoracotoı	250	CHU	No	60	0	1
1	1	Thoracotoı	250	CHU	No	58	0	0
1	0	VATS or rc	250	CHU	No	80	0	0
1	0	VATS or rc	250	CHU	No	72	0	0
1	0	Thoracotoı	250	CHU	No	66	0	0
1	0	Thoracotoı	250	CHU	No	61	0	1
1	0	Thoracotoı	250	CHU	No	65	0	0
1	0	Thoracotoı	250	CHU	No	61	0	0
1	0	Thoracotoı	250	CHU	No	67	0	1
1	0	Thoracotoı	250	CHU	No	63	0	0
1	0	VATS or rc	250	CHU	No	52	0	0
1	1	Thoracotoı	250	CHU	No	57	0	0
1	0	Thoracotoı	250	CHU	No	57	0	0
1	0	Thoracotoı	250	CHU	No	71	0	0
1	0	VATS or rc	250	CHU	No	63	0	0
1	1	Thoracotoı	250	CHU	No	75	0	0
1	0	VATS or rc	250	CHU	No	54	0	0
1	0	Thoracotoı	250	CHU	No	55	0	1
1	0	VATS or rc	250	CHU	No	65	0	1

1	0	Thoracotoı	250	CHU	No	42	0	0
1	1	Thoracotoı	250	CHU	No	59	1	0
1	0	Thoracotoı	250	CHU	No	66	0	0
1	0	Thoracotoı	250	CHU	No	71	0	0
1	0	VATS or rc	250	CHU	No	66	0	0
1	1	Thoracotoı	250	CHU	No	62	0	0
1	0	VATS or rc	250	CHU	No	65	0	1
1	1	Thoracotoı	250	CHU	No	70	0	0
1	1	Thoracotoı	250	CHU	No	50	0	0
1	1	Thoracotoı	250	CHU	No	68	0	0
1	0	Thoracotoı	250	CHU	No	59	0	0
1	0	Thoracotoı	250	CHU	No	83	1	0
1	0	Thoracotoı	250	CHU	No	77	1	0
1	0	VATS or rc	250	CHU	No	48	1	0
1	1	Thoracotoı	250	CHU	No	51	0	0
1	0	VATS or rc	250	CHU	No	65	0	0
1	0	Thoracotoı	250	CHU	No	62	0	0
1	0	Thoracotoı	250	CHU	No	44	0	0
1	0	Thoracotoı	250	CHU	No	72	0	0
1	0	Thoracotoı	250	CHU	No	63	1	0
1	0	VATS or rc	250	CHU	No	59	0	0
1	0	Thoracotoı	250	CHU	No	71	0	0
1	0	VATS or rc	250	CHU	No	69	0	1
1	0	Thoracotoı	250	CHU	No	47	0	0
1	1	Thoracotoı	250	CHU	No	52	0	0
1	0	Thoracotoı	250	CHU	No	73	0	0
1	0	Thoracotoı	250	CHU	No	70	0	0
1	0	Thoracotoı	250	CHU	No	69	0	0
1	0	Thoracotoı	250	CHU	No	68	1	0
1	0	VATS or rc	250	CHU	No	62	1	0
1	0	Thoracotoı	250	CHU	No	41	0	1
1	0	Thoracotoı	250	CHU	No	72	0	0
1	0	VATS or rc	250	CHU	No	55	0	1
1	0	Thoracotoı	250	CHU	No	51	0	0
1	0	Thoracotoı	250	CHU	No	71	0	0
1	0	Thoracotoı	250	CHU	No	63	0	0
1	0	VATS or rc	250	CHU	No	76	0	0
1	0	Thoracotoı	250	CHU	No	77	0	0
1	0	Thoracotoı	250	CHU	No	48	0	0
1	0	VATS or rc	250	CHU	No	66	0	0
1	0	Thoracotoı	250	CHU	No	71	0	0
1	0	Thoracotoı	250	CHU	No	68	0	0
1	0	Thoracotoı	250	CHU	No	73	0	0
1	0	VATS or rc	250	CHU	No	58	1	0
1	0	Thoracotoı	250	CHU	No	72	0	0
1	0	Thoracotoı	250	CHU	No	74	0	0
1	0	Thoracotoı	250	CHU	No	66	0	1
1	0	Thoracotoı	250	CHU	No	57	0	0
1	0	VATS or rc	250	CHU	No	57	0	1
1	0	VATS or rc	250	CHU	No	67	0	1
1	0	Thoracotoı	250	CHU	No	75	0	0
1	0	Thoracotoı	250	CHU	No	74	0	0
1	0	VATS or rc	250	CHU	No	55	0	1
1	1	Thoracotoı	250	CHU	No	57	0	1
1	0	Thoracotoı	250	CHU	No	62	0	1
1	0	Thoracotoı	250	CHU	No	57	1	0

1	0	Thoracotoı	250	CHU	No	58	0	1
1	1	Thoracotoı	250	CHU	No	65	0	0
1	0	VATS or rc	250	CHU	No	59	0	0
1	0	VATS or rc	250	CHU	No	62	0	1
1	0	Thoracotoı	250	CHU	No	68	0	0
1	0	Thoracotoı	250	CHU	No	58	0	1
1	0	Thoracotoı	250	CHU	No	49	0	1
1	0	Thoracotoı	250	CHU	No	46	0	0
1	0	Thoracotoı	250	CHU	No	56	0	0
1	0	Thoracotoı	250	CHU	No	70	0	0
1	0	Thoracotoı	250	CHU	No	60	0	0
1	0	Thoracotoı	250	CHU	No	59	0	1
1	0	Thoracotoı	250	CHU	No	86	0	0
1	0	Thoracotoı	250	CHU	No	64	0	1
1	0	VATS or rc	250	CHU	No	68	0	0
1	0	Thoracotoı	250	CHU	No	69	0	0
1	0	Thoracotoı	250	CHU	No	60	0	1
1	0	Thoracotoı	250	CHU	No	74	0	0
1	0	Thoracotoı	250	CHU	No	81	0	0
1	0	Thoracotoı	250	CHU	No	71	0	0
1	0	VATS or rc	250	CHU	No	80	0	0
1	0	VATS or rc	250	CHU	No	71	0	0
1	0	Thoracotoı	250	CHU	No	66	0	0
1	0	VATS or rc	250	CHU	No	81	1	0
1	0	VATS or rc	250	CHU	No	69	0	1
1	0	VATS or rc	250	CHU	No	72	0	1
1	0	Thoracotoı	250	CHU	No	67	0	0
1	0	Thoracotoı	250	CHU	No	40	0	0
1	0	Thoracotoı	250	CHU	No	82	0	0
1	0	Thoracotoı	250	CHU	No	52	1	0
1	0	VATS or rc	250	CHU	No	79	0	0
1	0	VATS or rc	250	CHU	No	76	0	1
1	0	Thoracotoı	250	CHU	No	66	0	0
1	0	Thoracotoı	250	CHU	No	63	1	0
1	0	Thoracotoı	250	CHU	No	73	0	1
1	0	Thoracotoı	250	CHU	No	74	1	0
1	0	VATS or rc	250	CHU	No	54	0	1
1	0	Thoracotoı	250	CHU	No	67	0	1
1	0	Thoracotoı	250	CHU	No	57	0	0
1	0	Thoracotoı	250	CHU	No	57	0	0
1	0	VATS or rc	250	CHU	No	68	0	0
1	0	Thoracotoı	250	CHU	No	63	0	0
1	0	Thoracotoı	250	CHU	No	68	0	0
1	0	Thoracotoı	250	CHU	No	69	0	1
1	1	Thoracotoı	250	CHU	No	66	0	0
1	0	Thoracotoı	250	CHU	No	72	0	0
1	0	Thoracotoı	250	CHU	No	56	0	1
1	1	VATS or rc	250	CHU	No	57	0	0
1	0	Thoracotoı	250	CHU	No	61	0	0
1	0	VATS or rc	250	CHU	No	72	0	0
1	0	Thoracotoı	250	CHU	No	79	0	0
1	0	Thoracotoı	250	CHU	No	74	0	0
1	1	Thoracotoı	250	CHU	No	74	0	0
1	0	Thoracotoı	250	CHU	No	52	0	1
1	0	Thoracotoı	250	CHU	No	65	0	0
1	0	Thoracotoı	250	CHU	No	65	1	0

1	1	Thoracotor	250	CHU	No	56	0	0
1	0	VATS or ro	250	CHU	No	65	0	0
1	1	Thoracotor	250	CHU	No	75	0	0
1	0	Thoracotor	250	CHU	No	74	0	0
1	0	Thoracotor	250	CHU	No	73	0	0
1	0	VATS or ro	250	CHU	No	57	0	0
1	0	Thoracotor	250	CHU	No	69	1	0
1	0	Thoracotor	250	CHU	No	62	0	1
1	0	Thoracotor	250	CHU	No	57	0	1
1	0	Thoracotor	250	CHU	No	76	0	0
1	0	VATS or ro	250	CHU	No	73	0	1
1	0	Thoracotor	250	CHU	No	56	0	1
1	0	Thoracotor	250	CHU	No	77	0	0
1	0	Thoracotor	250	CHU	No	70	0	0
1	0	Thoracotor	250	CHU	No	71	0	0
1	1	Thoracotor	250	CHU	No	74	0	0
1	0	Thoracotor	250	CHU	No	61	0	0
1	0	Thoracotor	250	CHU	No	66	0	1
1	0	VATS or ro	250	CHU	No	47	0	0
1	0	Thoracotor	250	CHU	No	50	0	0
1	0	Thoracotor	250	CHU	Yes	72	0	0
1	0	VATS or ro	250	CHU	No	77	0	0
1	0	VATS or ro	250	CHU	No	59	0	0
1	0	Thoracotor	250	CHU	No	61	0	0
1	0	Thoracotor	250	CHU	No	53	0	0
1	0	Thoracotor	250	CHU	No	55	0	0
1	0	Thoracotor	250	CHU	No	50	0	0
1	0	Thoracotor	250	CHU	No	61	0	0
1	0	Thoracotor	250	CHU	No	25	0	0
1	0	VATS or ro	250	CHU	No	67	0	1
1	0	Thoracotor	250	CHU	No	61	0	0
1	0	Thoracotor	250	CHU	No	60	0	0
1	0	VATS or ro	250	CHU	No	67	0	0
1	0	Thoracotor	250	CHU	No	73	0	0
1	0	Thoracotor	250	CHU	No	70	0	1
1	0	Thoracotor	250	CHU	No	70	0	0
1	0	Thoracotor	250	CHU	Yes	61	0	1
1	0	Thoracotor	250	CHU	No	65	0	0
1	0	Thoracotor	250	CHU	No	52	0	1
1	0	Thoracotor	250	CHU	No	55	0	0
1	0	Thoracotor	250	CHU	Yes	80	0	0
1	0	VATS or ro	250	CHU	No	68	0	0
1	0	VATS or ro	250	CHU	No	63	0	0
1	0	Thoracotor	250	CHU	No	76	0	0
1	0	Thoracotor	250	CHU	No	60	0	0
1	0	Thoracotor	250	CHU	No	81	0	0
1	0	Thoracotor	250	CHU	No	61	0	0
1	0	VATS or ro	250	CHU	No	59	0	1
1	0	Thoracotor	250	CHU	No	69	0	0
1	1	Thoracotor	250	CHU	No	52	0	0
1	0	Thoracotor	250	CHU	No	70	0	1
1	0	Thoracotor	250	CHU	No	43	0	0
1	0	Thoracotor	250	CHU	No	65	0	0
1	0	Thoracotor	250	CHU	No	73	0	0
1	0	Thoracotor	250	CHU	No	68	0	0
1	0	Thoracotor	250	CHU	No	61	0	0

1	0	VATS or rc	250	CHU	No	80	1	1
1	0	Thoracotoı	250	CHU	No	69	0	0
1	0	Thoracotoı	250	CHU	No	79	0	0
1	0	VATS or rc	250	CHU	No	67	0	0
1	0	Thoracotoı	250	CHU	No	76	0	0
1	0	Thoracotoı	250	CHU	No	67	1	0
1	0	Thoracotoı	250	CHU	No	73	0	0
1	0	Thoracotoı	250	CHU	No	51	0	0
1	0	Thoracotoı	250	CHU	No	68	0	0
1	0	Thoracotoı	250	CHU	No	82	0	0
1	0	Thoracotoı	250	CHU	No	63	0	0
1	0	VATS or rc	250	CHU	No	49	0	0
1	1	Thoracotoı	250	CHU	No	68	0	0
1	0	Thoracotoı	250	CHU	No	81	0	0
1	1	Thoracotoı	250	CHU	No	71	0	0
1	0	Thoracotoı	250	CHU	No	64	0	1
1	0	Thoracotoı	250	CHU	No	72	0	1
1	0	Thoracotoı	250	CHU	No	60	0	1
1	0	VATS or rc	250	CHU	No	69	0	0
1	0	Thoracotoı	250	CHU	No	58	0	1
1	0	Thoracotoı	250	CHU	No	73	0	0
1	0	Thoracotoı	250	CHU	No	67	0	0
1	0	Thoracotoı	250	CHU	No	55	1	0
1	0	Thoracotoı	250	CHU	No	70	0	1
1	0	Thoracotoı	250	CHU	No	62	0	1
1	0	Thoracotoı	250	CHU	No	50	0	1
1	0	Thoracotoı	250	CHU	No	77	0	1
1	0	VATS or rc	250	CHU	No	64	0	0
1	0	Thoracotoı	250	CHU	No	68	0	0
1	0	VATS or rc	250	CHU	Yes	81	0	0
1	0	VATS or rc	250	CHU	No	67	0	0
1	0	VATS or rc	250	CHU	No	62	0	0
1	0	Thoracotoı	250	CHU	No	76	0	0
1	0	Thoracotoı	250	CHU	No	69	0	0
1	0	Thoracotoı	250	CHU	Yes	66	0	0
1	0	VATS or rc	250	CHU	No	67	0	0
1	0	Thoracotoı	250	CHU	No	67	0	0
1	0	Thoracotoı	250	CHU	No	58	0	0
1	0	Thoracotoı	250	CHU	No	71	0	1
1	1	Thoracotoı	250	CHU	No	78	0	0
1	0	Thoracotoı	250	CHU	No	49	0	1
1	0	VATS or rc	250	CHU	No	64	0	0
1	0	Thoracotoı	250	CHU	No	73	0	0
1	0	VATS or rc	250	CHU	No	28	0	0
1	0	Thoracotoı	250	CHU	No	50	0	0
1	0	Thoracotoı	250	CHU	No	69	0	0
1	1	Thoracotoı	250	CHU	No	43	0	1
1	0	Thoracotoı	250	CHU	No	60	0	0
1	0	Thoracotoı	250	CHU	No	77	0	0
1	0	Thoracotoı	250	CHU	No	67	1	0
1	0	VATS or rc	250	CHU	No	82	1	0
1	0	Thoracotoı	250	CHU	No	54	0	0
1	0	Thoracotoı	250	CHU	No	66	0	0
1	0	Thoracotoı	250	CHU	No	59	1	0
1	0	Thoracotoı	250	CHU	No	67	0	0
1	0	Thoracotoı	250	CHU	No	72	0	0

1	0	Thoracotoı	250	CHU	No	69	0	0
1	0	Thoracotoı	250	CHU	No	70	0	1
1	0	VATS or rc	250	CHU	No	75	0	0
1	0	Thoracotoı	250	CHU	No	56	0	1
1	0	VATS or rc	250	CHU	No	75	0	0
1	1	Thoracotoı	250	CHU	No	58	0	0
1	0	Thoracotoı	250	CHU	No	73	1	1
1	0	VATS or rc	250	CHU	No	62	0	0
1	0	Thoracotoı	250	CHU	No	60	0	0
1	0	Thoracotoı	250	CHU	No	56	0	0
1	0	Thoracotoı	250	CHU	No	61	0	1
1	0	Thoracotoı	250	CHU	No	56	0	0
1	0	Thoracotoı	250	CHU	No	60	0	1
1	0	Thoracotoı	250	CHU	No	58	0	1
1	1	Thoracotoı	250	CHU	No	64	0	0
1	1	Thoracotoı	250	CHU	No	53	0	0
1	0	VATS or rc	250	CHU	No	65	1	1
1	0	VATS or rc	250	CHU	No	78	0	0
1	0	VATS or rc	250	CHU	No	77	1	0
1	0	Thoracotoı	250	CHU	No	72	0	1
1	1	Thoracotoı	250	CHU	No	60	1	0
1	0	Thoracotoı	250	CHU	No	74	1	1
1	0	Thoracotoı	250	CHU	No	64	0	0
1	0	Thoracotoı	250	CHU	No	70	0	0
1	1	Thoracotoı	250	CHU	No	45	0	1
1	0	Thoracotoı	250	CHU	No	58	0	1
1	1	Thoracotoı	250	CHU	No	47	0	0
1	0	Thoracotoı	250	CHU	No	65	0	0
1	0	Thoracotoı	250	CHU	No	77	0	0
1	0	VATS or rc	250	CHU	No	73	0	0
1	0	Thoracotoı	250	CHU	No	64	0	0
1	0	VATS or rc	250	CHU	No	58	0	0
1	0	VATS or rc	250	CHU	No	71	0	0
1	0	Thoracotoı	250	CHU	No	62	0	0
1	0	VATS or rc	250	CHU	No	64	0	0
1	0	Thoracotoı	250	CHU	No	50	0	0
1	1	Thoracotoı	250	CHU	No	52	0	0
1	0	Thoracotoı	250	CHU	No	78	0	0
1	0	Thoracotoı	250	CHU	No	73	0	0
1	0	Thoracotoı	250	CHU	No	80	0	0
1	0	Thoracotoı	250	CHU	No	76	1	0
1	0	Thoracotoı	250	CHU	No	70	0	0
1	0	Thoracotoı	250	CHU	No	68	0	0
1	0	Thoracotoı	250	CHU	No	63	0	0
1	0	Thoracotoı	250	CHU	No	72	0	0
1	0	Thoracotoı	250	CHU	No	66	0	1
1	0	VATS or rc	250	CHU	No	69	1	1
1	0	Thoracotoı	250	CHU	No	78	0	0
1	0	VATS or rc	250	CHU	No	84	0	0
1	1	Thoracotoı	250	CHU	No	65	0	0
1	0	Thoracotoı	250	CHU	No	57	0	1
1	0	Thoracotoı	250	CHU	No	67	0	1
1	0	Thoracotoı	250	CHU	No	46	0	0
1	0	Thoracotoı	250	CHU	No	69	0	0
1	0	Thoracotoı	250	CHU	No	51	0	0
1	0	Thoracotoı	250	CHU	No	54	0	0

1	0	VATS or rc	250	CHU	No	59	0	0
1	0	VATS or rc	250	CHU	No	77	0	0
1	0	Thoracotoı	250	CHU	No	66	0	0
1	0	Thoracotoı	250	CHU	No	58	0	1
1	0	Thoracotoı	250	CHU	No	62	0	0
1	0	Thoracotoı	250	CHU	No	61	0	0
1	0	Thoracotoı	250	CHU	No	59	0	1
1	0	Thoracotoı	250	CHU	No	68	1	0
1	1	Thoracotoı	250	CHU	No	56	0	1
1	1	Thoracotoı	250	CHU	No	53	0	0
1	0	Thoracotoı	250	CHU	No	57	0	0
1	1	Thoracotoı	250	CHU	No	46	0	1
1	0	Thoracotoı	250	CHU	No	47	0	0
1	0	Thoracotoı	250	CHU	No	55	0	1
1	0	Thoracotoı	250	CHU	No	67	0	0
1	0	Thoracotoı	250	CHU	No	59	0	0
1	0	Thoracotoı	250	CHU	No	69	0	0
1	0	Thoracotoı	250	CHU	No	69	0	0
1	0	Thoracotoı	250	CHU	No	71	0	1
1	0	VATS or rc	250	CHU	No	73	0	0
1	0	Thoracotoı	250	CHU	No	72	0	0
1	0	Thoracotoı	250	CHU	No	54	0	1
1	0	VATS or rc	250	CHU	No	62	0	0
1	0	Thoracotoı	250	CHU	No	75	0	0
1	0	Thoracotoı	250	CHU	No	65	0	0
1	0	Thoracotoı	250	CHU	No	52	0	1
1	0	Thoracotoı	250	CHU	No	69	1	0
1	1	Thoracotoı	250	CHU	No	76	0	0
1	0	VATS or rc	250	CHU	No	66	0	0
1	0	Thoracotoı	250	CHU	No	64	0	0
1	1	Thoracotoı	250	CHU	No	58	0	0
1	0	Thoracotoı	250	CHU	No	65	0	0
1	0	Thoracotoı	250	CHU	No	71	0	0
1	0	Thoracotoı	250	CHU	No	63	0	0
1	0	Thoracotoı	250	CHU	No	69	0	1
1	0	Thoracotoı	250	CHU	No	74	0	0
1	1	Thoracotoı	250	CHU	No	58	0	0
1	1	Thoracotoı	250	CHU	No	64	0	0
1	0	Thoracotoı	250	CHU	No	64	0	0
1	0	Thoracotoı	250	CHU	No	67	1	0
1	0	Thoracotoı	250	CHU	No	72	0	1
1	0	Thoracotoı	250	CHU	No	63	0	0
1	0	Thoracotoı	250	CHU	No	41	0	0
1	0	VATS or rc	250	CHU	No	66	0	0
1	0	VATS or rc	250	CHU	No	63	0	0
1	0	Thoracotoı	250	CHU	No	54	0	0
1	0	VATS or rc	250	CHU	No	62	1	0
1	0	VATS or rc	250	CHU	No	66	0	0
1	0	VATS or rc	250	CHU	No	60	0	0
1	0	Thoracotoı	250	CHU	No	57	0	0
1	1	Thoracotoı	250	CHU	No	49	0	0
1	0	VATS or rc	250	CHU	No	80	0	0
1	1	Thoracotoı	250	CHU	No	42	0	0
1	0	Thoracotoı	250	CHU	No	54	0	1
1	0	Thoracotoı	250	CHU	No	56	0	0
1	0	Thoracotoı	250	CHU	No	77	0	0

1	0	Thoracotoı	250	CHU	No	66	0	0
1	0	Thoracotoı	250	CHU	No	68	0	0
1	0	Thoracotoı	250	CHU	No	72	0	1
1	0	Thoracotoı	250	CHU	No	67	0	0
1	0	Thoracotoı	250	CHU	Yes	89	0	0
1	0	Thoracotoı	250	CHU	No	64	0	1
1	0	VATS or rc	250	CHU	No	53	0	1
1	0	Thoracotoı	250	CHU	No	66	0	1
1	0	Thoracotoı	250	CHU	No	64	0	0
1	0	Thoracotoı	250	CHU	No	66	0	1
1	0	Thoracotoı	250	CHU	No	70	0	1
1	0	Thoracotoı	250	CHU	No	58	0	0
1	0	Thoracotoı	250	CHU	No	79	0	0
1	0	VATS or rc	250	CHU	No	76	0	0
1	1	Thoracotoı	250	CHU	No	60	0	0
1	0	Thoracotoı	250	CHU	No	67	1	0
1	0	VATS or rc	250	CHU	No	57	0	1
1	0	VATS or rc	250	CHU	No	54	0	0
1	0	VATS or rc	250	CHU	Yes	75	0	0
1	0	VATS or rc	250	CHU	No	71	1	1
1	0	Thoracotoı	250	CHU	No	77	0	1
1	1	Thoracotoı	250	CHU	Yes	59	0	0
1	0	Thoracotoı	250	CHU	No	50	0	0
1	0	Thoracotoı	250	CHU	No	67	0	0
1	0	VATS or rc	250	CHU	No	69	0	0
1	0	Thoracotoı	250	CHU	No	56	0	0
1	0	VATS or rc	250	CHU	No	58	0	0
1	0	Thoracotoı	250	CHU	No	60	0	0
1	0	Thoracotoı	250	CHU	No	50	0	0
1	0	Thoracotoı	250	CHU	No	59	0	0
1	0	Thoracotoı	250	CHU	No	60	0	0
1	0	Thoracotoı	250	CHU	No	83	0	0
1	0	VATS or rc	250	CHU	No	65	0	1
1	0	Thoracotoı	250	CHU	No	58	0	0
1	0	VATS or rc	250	CHU	No	85	0	0
1	0	Thoracotoı	250	CHU	No	68	0	0
1	0	Thoracotoı	250	CHU	No	76	0	1
1	0	Thoracotoı	250	CHU	No	63	0	0
1	0	Thoracotoı	250	CHU	No	67	0	0
1	0	Thoracotoı	250	CHU	No	51	0	0
1	0	Thoracotoı	250	CHU	No	80	0	0
1	1	Thoracotoı	250	CHU	No	44	0	0
1	0	Thoracotoı	250	CHU	No	62	0	1
1	0	Thoracotoı	250	CHU	No	52	0	1
1	0	Thoracotoı	250	CHU	No	76	0	0
1	0	Thoracotoı	250	CHU	No	74	1	0
1	0	Thoracotoı	250	CHU	No	54	0	0
1	0	VATS or rc	250	CHU	No	58	0	0
1	0	Thoracotoı	250	CHU	No	72	0	0
1	0	VATS or rc	250	CHU	No	64	0	1
1	0	Thoracotoı	250	CHU	No	65	0	0
1	0	VATS or rc	250	CHU	No	80	0	0
1	0	Thoracotoı	250	CHU	No	41	0	0
1	0	Thoracotoı	250	CHU	No	71	0	0
1	0	Thoracotoı	250	CHU	No	63	0	0
1	0	VATS or rc	250	CHU	No	54	0	0

1	0	Thoracotoı	250	CHU	No	66	0	1
1	0	VATS or rc	250	CHU	No	73	0	0
1	0	VATS or rc	250	CHU	No	72	0	0
1	0	VATS or rc	250	CHU	No	70	0	0
1	0	Thoracotoı	250	CHU	No	75	1	0
1	0	Thoracotoı	250	CHU	No	44	0	0
1	0	Thoracotoı	250	CHU	No	64	1	1
1	1	Thoracotoı	250	CHU	No	63	0	1
1	1	Thoracotoı	250	CHU	No	42	0	1
1	0	Thoracotoı	250	CHU	No	67	0	1
1	0	VATS or rc	250	CHU	No	71	0	1
1	0	Thoracotoı	250	CHU	No	48	0	0
1	0	VATS or rc	250	CHU	No	63	0	0
1	0	Thoracotoı	250	CHU	No	54	0	0
1	0	VATS or rc	250	CHU	No	84	0	0
1	0	Thoracotoı	250	CHU	No	57	0	0
1	0	Thoracotoı	250	CHU	No	34	0	0
1	0	Thoracotoı	250	CHU	No	75	0	0
1	0	Thoracotoı	250	CHU	No	58	0	0
1	0	Thoracotoı	250	CHU	Yes	76	0	0
1	0	Thoracotoı	250	CHU	No	50	0	0
1	0	Thoracotoı	250	CHU	No	73	0	0
1	0	VATS or rc	250	CHU	No	77	0	0
1	0	Thoracotoı	250	CHU	No	78	0	0
1	1	Thoracotoı	250	CHU	No	69	0	1
1	0	Thoracotoı	250	CHU	No	67	1	0
1	0	VATS or rc	250	CHU	No	57	0	0
1	0	VATS or rc	250	CHU	No	65	0	0
1	0	Thoracotoı	250	CHU	No	63	1	1
1	0	VATS or rc	250	CHU	No	68	0	0
1	0	VATS or rc	250	CHU	No	82	1	0
1	0	VATS or rc	250	CHU	No	54	0	0
1	0	Thoracotoı	250	CHU	No	58	0	0
1	0	VATS or rc	250	CHU	No	76	1	1
1	0	Thoracotoı	250	CHU	No	86	1	0
1	0	Thoracotoı	250	CHU	No	79	1	0
1	0	Thoracotoı	250	CHU	No	58	0	0
1	1	Thoracotoı	250	CHU	No	67	0	0
1	0	Thoracotoı	250	CHU	No	60	0	0
1	0	VATS or rc	250	CHU	No	51	0	0
1	0	Thoracotoı	250	CHU	No	73	0	0
1	0	Thoracotoı	250	CHU	No	42	0	0
1	0	Thoracotoı	250	CHU	No	71	0	0
1	0	Thoracotoı	250	CHU	No	79	0	0
1	0	Thoracotoı	250	CHU	No	55	0	1
1	0	VATS or rc	250	CHU	No	72	0	0
1	0	Thoracotoı	250	CHU	No	70	0	1
1	0	Thoracotoı	250	CHU	No	59	0	0
1	0	Thoracotoı	250	CHU	No	59	0	1
1	0	VATS or rc	250	CHU	No	75	1	0
1	1	Thoracotoı	250	CHU	No	53	0	0
1	0	Thoracotoı	250	CHU	No	82	0	0
1	0	VATS or rc	250	CHU	No	73	0	1
1	0	Thoracotoı	250	CHU	No	63	0	0
1	0	Thoracotoı	250	CHU	No	71	0	0
1	0	Thoracotoı	250	CHU	No	59	0	1

1	0	VATS or rc	250	CHU	No	69	0	0
1	0	Thoracotoı	250	CHU	No	52	1	0
1	0	Thoracotoı	250	CHU	No	65	0	0
1	0	Thoracotoı	250	CHU	No	58	0	0
1	0	Thoracotoı	250	CHU	No	66	0	0
1	0	Thoracotoı	250	CHU	No	65	0	0
1	0	VATS or rc	250	CHU	No	76	0	0
1	0	Thoracotoı	250	CHU	No	55	1	1
1	1	Thoracotoı	250	CHU	No	72	0	0
1	0	Thoracotoı	250	CHU	No	64	0	0
1	0	Thoracotoı	250	CHU	No	43	0	0
1	1	Thoracotoı	250	CHU	No	55	0	0
1	0	Thoracotoı	250	CHU	No	64	0	0
1	0	Thoracotoı	250	CHU	No	58	0	1
1	0	VATS or rc	250	CHU	No	82	0	0
1	0	VATS or rc	250	CHU	No	80	0	0
1	0	Thoracotoı	250	CHU	No	63	0	0
1	0	Thoracotoı	250	CHU	No	70	0	0
1	0	Thoracotoı	250	CHU	No	40	0	0
1	0	Thoracotoı	250	CHU	No	51	0	1
1	0	Thoracotoı	250	CHU	No	69	0	0
1	0	Thoracotoı	250	CHU	No	54	0	1
1	0	VATS or rc	250	CHU	No	64	0	0
1	0	Thoracotoı	250	CHU	No	65	0	0
1	0	Thoracotoı	250	CHU	No	57	0	1
1	0	Thoracotoı	250	CHU	No	62	0	0
1	0	Thoracotoı	250	CHU	No	77	0	0
1	0	Thoracotoı	250	CHU	No	74	0	0
1	0	Thoracotoı	250	CHU	No	73	0	0
1	0	Thoracotoı	250	CHU	No	66	0	0
1	0	Thoracotoı	250	CHU	No	59	0	1
1	0	Thoracotoı	250	CHU	No	54	0	0
1	0	VATS or rc	250	CHU	No	59	0	0
1	0	Thoracotoı	250	CHU	No	76	0	0
1	0	Thoracotoı	250	CHU	No	67	0	1
1	0	Thoracotoı	250	CHU	No	75	0	0
1	1	Thoracotoı	250	CHU	No	58	0	0
1	0	Thoracotoı	250	CHU	No	68	0	0
1	0	VATS or rc	250	CHU	No	64	0	0
1	0	Thoracotoı	250	CHU	No	58	0	0
1	0	Thoracotoı	250	CHU	No	71	0	0
1	0	VATS or rc	250	CHU	No	64	0	0
1	0	Thoracotoı	250	CHU	No	37	0	0
1	0	Thoracotoı	250	CHU	No	71	0	0
1	0	Thoracotoı	250	CHU	No	52	0	1
1	0	Thoracotoı	250	CHU	No	74	0	0
1	1	Thoracotoı	250	CHU	No	58	0	0
1	0	Thoracotoı	250	CHU	No	65	0	1
1	0	Thoracotoı	250	CHU	No	55	0	0
1	1	Thoracotoı	250	CHU	Yes	59	0	1
1	0	Thoracotoı	250	CHU	No	74	0	0
1	0	Thoracotoı	250	CHU	No	69	0	1
1	0	Thoracotoı	250	CHU	No	63	0	1
1	0	Thoracotoı	250	CHU	No	59	0	0
1	0	Thoracotoı	250	CHU	No	78	0	1
1	0	Thoracotoı	250	CHU	No	64	0	0

1	0	Thoracotoı	250	CHU	No	54	0	0
1	0	VATS or rc	250	CHU	No	64	0	0
1	0	Thoracotoı	250	CHU	No	70	0	0
1	0	Thoracotoı	250	CHU	No	78	0	0
1	0	Thoracotoı	250	CHU	No	55	0	0
1	0	Thoracotoı	250	CHU	No	75	0	0
1	0	Thoracotoı	250	CHU	No	60	0	0
1	0	VATS or rc	250	CHU	No	68	0	0
1	0	Thoracotoı	250	CHU	No	62	0	0
1	0	Thoracotoı	250	CHU	No	54	0	0
1	1	Thoracotoı	250	CHU	No	62	0	1
1	0	Thoracotoı	250	CHU	No	60	0	0
1	0	Thoracotoı	250	CHU	No	69	0	1
1	0	Thoracotoı	250	CHU	No	75	0	0
1	0	Thoracotoı	250	CHU	No	76	1	1
1	0	VATS or rc	250	CHU	No	68	0	0
1	1	Thoracotoı	250	CHU	No	80	0	0
1	0	VATS or rc	250	CHU	No	62	0	1
1	0	Thoracotoı	250	CHU	No	52	0	1
1	0	Thoracotoı	250	CHU	No	61	0	0
1	0	Thoracotoı	250	CHU	No	55	0	1
0	0	VATS or rc	50	PRIVE	No	47	0	0
0	0	Thoracotoı	50	PRIVE	No	71	0	0
0	0	Thoracotoı	50	PRIVE	No	64	0	0
0	0	Thoracotoı	50	PRIVE	No	66	1	0
0	1	Thoracotoı	50	PRIVE	No	53	0	0
0	0	Thoracotoı	50	PRIVE	No	77	0	0
0	0	Thoracotoı	50	PRIVE	No	68	1	0
0	0	Thoracotoı	50	PRIVE	No	58	0	0
0	0	Thoracotoı	50	PRIVE	No	60	0	0
0	0	Thoracotoı	50	PRIVE	No	80	0	0
0	0	Thoracotoı	50	PRIVE	No	64	0	0
0	0	Thoracotoı	50	PRIVE	No	62	1	0
0	0	Thoracotoı	50	PRIVE	No	79	0	0
0	1	Thoracotoı	50	PRIVE	No	54	0	0
0	0	Thoracotoı	50	PRIVE	No	55	0	0
0	1	Thoracotoı	50	PRIVE	No	62	1	0
0	1	Thoracotoı	50	PRIVE	No	75	0	0
0	1	Thoracotoı	50	PRIVE	No	55	0	0
0	0	Thoracotoı	50	PRIVE	No	62	0	0
0	0	Thoracotoı	50	PRIVE	No	79	1	0
0	0	Thoracotoı	50	PRIVE	No	61	0	0
0	0	Thoracotoı	50	PRIVE	No	78	0	0
0	0	VATS or rc	50	PRIVE	No	61	0	0
0	0	Thoracotoı	50	PRIVE	No	80	0	0
0	0	Thoracotoı	50	PRIVE	Yes	77	0	0
0	0	Thoracotoı	50	PRIVE	No	61	0	0
0	0	Thoracotoı	50	PRIVE	No	67	0	0
0	0	Thoracotoı	50	PRIVE	No	61	0	0
0	0	Thoracotoı	50	PRIVE	No	72	0	0
0	0	Thoracotoı	50	PRIVE	No	81	0	0
0	0	Thoracotoı	50	PRIVE	No	71	0	0
0	0	Thoracotoı	50	PRIVE	No	76	0	0
0	0	Thoracotoı	50	PRIVE	No	59	0	0
0	0	Thoracotoı	50	PRIVE	No	63	0	0
0	0	Thoracotoı	50	PRIVE	No	66	0	0

0	0	Thoracotoı	50	PRIVE	No	65	0	0
0	0	Thoracotoı	226	PRIVE	No	59	0	0
0	0	Thoracotoı	226	PRIVE	No	76	0	0
0	0	VATS or rc	226	PRIVE	No	58	0	1
0	0	Thoracotoı	226	PRIVE	No	66	0	1
0	0	Thoracotoı	226	PRIVE	No	72	0	1
0	0	Thoracotoı	226	PRIVE	No	52	0	0
0	1	Thoracotoı	226	PRIVE	No	76	0	0
0	0	Thoracotoı	226	PRIVE	No	55	0	1
0	0	Thoracotoı	226	PRIVE	No	79	1	0
0	0	Thoracotoı	226	PRIVE	No	62	0	1
0	0	Thoracotoı	226	PRIVE	No	73	1	1
0	0	Thoracotoı	226	PRIVE	No	77	0	0
0	0	VATS or rc	226	PRIVE	Yes	63	1	0
0	0	Thoracotoı	226	PRIVE	No	77	0	0
0	0	Thoracotoı	226	PRIVE	No	61	0	1
0	0	Thoracotoı	226	PRIVE	No	59	0	1
0	0	Thoracotoı	226	PRIVE	No	54	1	1
0	0	VATS or rc	226	PRIVE	No	62	0	1
0	0	Thoracotoı	226	PRIVE	No	73	0	0
0	0	Thoracotoı	226	PRIVE	No	79	0	1
0	0	VATS or rc	226	PRIVE	No	64	0	1
0	0	VATS or rc	226	PRIVE	No	44	0	0
0	0	Thoracotoı	226	PRIVE	No	73	0	0
0	0	Thoracotoı	226	PRIVE	No	64	0	0
0	0	Thoracotoı	226	PRIVE	No	65	0	1
0	0	Thoracotoı	226	PRIVE	No	60	0	1
0	0	VATS or rc	226	PRIVE	No	68	0	1
0	0	VATS or rc	226	PRIVE	No	60	0	1
0	0	VATS or rc	226	PRIVE	No	75	1	1
0	0	VATS or rc	226	PRIVE	No	71	0	1
0	0	Thoracotoı	226	PRIVE	No	76	0	0
0	0	Thoracotoı	226	PRIVE	No	68	1	1
0	0	Thoracotoı	226	PRIVE	No	56	0	1
0	0	VATS or rc	226	PRIVE	No	46	0	0
0	0	Thoracotoı	226	PRIVE	No	78	0	0
0	0	Thoracotoı	226	PRIVE	No	74	0	1
0	0	VATS or rc	226	PRIVE	No	82	0	0
0	0	Thoracotoı	226	PRIVE	No	75	0	1
0	0	Thoracotoı	226	PRIVE	No	68	0	0
0	0	Thoracotoı	226	PRIVE	No	71	0	0
0	1	Thoracotoı	226	PRIVE	No	63	0	0
0	0	VATS or rc	226	PRIVE	No	82	0	1
0	0	Thoracotoı	226	PRIVE	No	50	0	0
0	0	Thoracotoı	226	PRIVE	No	57	0	1
0	0	Thoracotoı	226	PRIVE	No	69	0	1
0	0	Thoracotoı	226	PRIVE	No	66	0	0
0	0	Thoracotoı	226	PRIVE	No	57	0	1
0	0	Thoracotoı	226	PRIVE	No	69	1	1
0	0	VATS or rc	226	PRIVE	No	66	0	0
0	1	Thoracotoı	226	PRIVE	No	68	0	1
0	0	Thoracotoı	226	PRIVE	No	59	0	0
0	0	Thoracotoı	226	PRIVE	No	68	0	0
0	0	Thoracotoı	226	PRIVE	No	60	0	0
0	0	Thoracotoı	226	PRIVE	No	59	0	0
0	0	VATS or rc	226	PRIVE	No	75	0	1

0	0	VATS or ro	226	PRIVE	No	66	0	0
0	0	Thoracotoı	226	PRIVE	No	61	0	1
0	0	Thoracotoı	226	PRIVE	No	68	0	1
0	0	Thoracotoı	226	PRIVE	No	82	0	0
0	1	Thoracotoı	226	PRIVE	No	25	0	0
0	0	VATS or ro	226	PRIVE	No	70	0	0
0	0	Thoracotoı	226	PRIVE	No	56	0	0
0	0	Thoracotoı	226	PRIVE	No	61	1	1
0	0	VATS or ro	226	PRIVE	No	68	0	0
0	0	Thoracotoı	226	PRIVE	No	76	0	1
0	0	Thoracotoı	226	PRIVE	No	78	0	1
0	0	VATS or ro	226	PRIVE	No	70	0	1
0	0	Thoracotoı	226	PRIVE	No	61	0	0
0	0	VATS or ro	226	PRIVE	No	56	0	1
0	0	Thoracotoı	226	PRIVE	No	69	0	1
0	0	Thoracotoı	226	PRIVE	No	59	0	0
0	0	Thoracotoı	226	PRIVE	No	56	0	0
0	0	Thoracotoı	226	PRIVE	No	55	0	1
0	1	Thoracotoı	226	PRIVE	No	63	0	1
0	0	Thoracotoı	226	PRIVE	No	85	0	0
0	0	Thoracotoı	226	PRIVE	No	75	1	0
0	0	Thoracotoı	226	PRIVE	No	73	0	1
0	0	Thoracotoı	226	PRIVE	No	67	0	0
0	0	VATS or ro	226	PRIVE	No	66	0	0
0	0	Thoracotoı	226	PRIVE	No	57	0	1
0	0	Thoracotoı	226	PRIVE	No	61	0	1
0	0	Thoracotoı	226	PRIVE	No	67	0	0
0	0	Thoracotoı	226	PRIVE	No	57	0	1
0	0	VATS or ro	226	PRIVE	No	70	1	0
0	0	Thoracotoı	226	PRIVE	No	61	0	1
0	0	VATS or ro	226	PRIVE	No	70	0	0
0	0	Thoracotoı	226	PRIVE	No	60	0	0
0	0	VATS or ro	226	PRIVE	No	71	1	0
0	0	Thoracotoı	226	PRIVE	No	78	0	0
0	0	Thoracotoı	226	PRIVE	No	73	0	0
0	0	Thoracotoı	226	PRIVE	No	67	0	1
0	0	Thoracotoı	226	PRIVE	No	70	0	0
0	0	VATS or ro	226	PRIVE	No	64	0	1
0	0	Thoracotoı	226	PRIVE	No	50	0	1
0	0	VATS or ro	226	PRIVE	No	67	0	1
0	0	Thoracotoı	226	PRIVE	No	66	1	1
0	0	Thoracotoı	226	PRIVE	No	64	0	1
0	1	Thoracotoı	226	PRIVE	No	68	0	1
0	0	VATS or ro	226	PRIVE	No	70	0	0
0	0	VATS or ro	226	PRIVE	No	61	0	1
0	0	Thoracotoı	226	PRIVE	No	59	0	0
0	0	Thoracotoı	226	PRIVE	No	70	0	1
0	0	Thoracotoı	226	PRIVE	No	70	0	1
0	0	VATS or ro	226	PRIVE	No	80	0	1
0	0	VATS or ro	11	PRIVE	No	59	0	0
0	0	Thoracotoı	93	CHG	No	75	1	0
0	0	VATS or ro	93	CHG	No	80	1	0
0	0	VATS or ro	93	CHG	No	62	0	1
0	0	VATS or ro	93	CHG	No	71	1	0
0	0	Thoracotoı	93	CHG	No	85	0	0
0	0	VATS or ro	93	CHG	No	61	0	0

0	0	VATS or ro	93	CHG	No	56	0	0
0	0	VATS or ro	93	CHG	No	68	0	0
0	0	Thoracotom	93	CHG	No	65	0	1
0	0	VATS or ro	93	CHG	No	71	0	0
0	0	Thoracotom	93	CHG	No	74	1	0
0	0	Thoracotom	93	CHG	No	62	0	0
0	0	Thoracotom	93	CHG	Yes	77	1	0
0	0	VATS or ro	93	CHG	No	65	0	1
0	0	VATS or ro	93	CHG	No	74	0	0
0	0	Thoracotom	93	CHG	No	64	0	0
0	0	VATS or ro	93	CHG	No	56	0	0
0	0	VATS or ro	93	CHG	Yes	72	0	1
0	0	VATS or ro	93	CHG	No	62	0	1
0	0	VATS or ro	93	CHG	No	59	1	1
0	0	VATS or ro	93	CHG	No	80	0	0
0	0	Thoracotom	93	CHG	No	60	0	0
0	0	VATS or ro	93	CHG	No	68	0	0
0	0	VATS or ro	93	CHG	No	56	0	0
0	0	Thoracotom	93	CHG	No	53	0	0
0	0	VATS or ro	93	CHG	No	71	0	1
0	0	VATS or ro	93	CHG	No	50	0	0
0	0	Thoracotom	93	CHG	No	79	0	0
0	0	VATS or ro	93	CHG	No	68	0	0
0	0	Thoracotom	93	CHG	No	61	0	0
0	0	Thoracotom	93	CHG	No	66	0	0
0	0	Thoracotom	93	CHG	No	66	0	0
0	0	VATS or ro	93	CHG	No	66	0	0
0	0	VATS or ro	93	CHG	No	62	0	0
0	0	VATS or ro	93	CHG	No	71	0	1
0	0	VATS or ro	93	CHG	No	58	0	0
0	0	VATS or ro	93	CHG	No	49	0	0
0	0	Thoracotom	19	CHG	No	64	0	1
0	0	VATS or ro	19	CHG	No	63	0	1
0	0	VATS or ro	19	CHG	No	60	0	0
0	0	Thoracotom	19	CHG	No	68	0	1
0	1	Thoracotom	19	CHG	No	68	0	0
0	0	VATS or ro	19	CHG	No	65	1	1
0	0	VATS or ro	19	CHG	No	68	0	1
0	0	VATS or ro	19	CHG	No	51	0	1
0	0	VATS or ro	19	CHG	No	69	1	0
0	0	VATS or ro	19	CHG	No	85	0	1
0	0	VATS or ro	19	CHG	No	75	1	1
0	0	VATS or ro	19	CHG	No	64	0	0
0	0	VATS or ro	19	CHG	No	75	0	0
0	0	Thoracotom	19	CHG	No	73	0	0
0	0	VATS or ro	19	CHG	No	87	0	1
0	0	Thoracotom	19	CHG	No	58	0	1
0	0	Thoracotom	19	CHG	No	67	1	1
0	0	VATS or ro	19	CHG	Yes	42	0	0
0	0	VATS or ro	19	CHG	No	56	1	0
0	0	Thoracotom	19	CHG	No	69	1	1
0	0	Thoracotom	19	CHG	No	60	0	0
0	0	VATS or ro	19	CHG	No	81	1	0
0	0	Thoracotom	19	CHG	No	57	0	0
0	0	Thoracotom	19	CHG	No	73	0	0
0	0	Thoracotom	19	CHG	No	66	0	0

0	0	VATS or ro	19	CHG	No	64	0	0
0	0	VATS or ro	19	CHG	No	81	1	0
0	0	Thoracotor	19	CHG	No	56	0	1
0	0	Thoracotor	19	CHG	No	62	0	1
0	1	Thoracotor	19	CHG	No	73	0	1
0	0	Thoracotor	19	CHG	No	74	0	0
0	0	VATS or ro	19	CHG	No	67	0	0
0	0	VATS or ro	19	CHG	No	74	0	1
0	0	Thoracotor	19	CHG	No	63	0	0
0	0	Thoracotor	19	CHG	No	64	0	0
0	0	VATS or ro	19	CHG	No	63	0	0
0	0	VATS or ro	19	CHG	No	58	0	1
0	0	Thoracotor	19	CHG	No	61	0	1
0	0	Thoracotor	19	CHG	No	77	0	1
0	0	VATS or ro	19	CHG	No	61	0	0
0	0	VATS or ro	19	CHG	No	82	0	1
0	0	Thoracotor	19	CHG	No	60	0	0
0	0	VATS or ro	19	CHG	No	64	1	1
0	0	Thoracotor	19	CHG	No	82	0	0
0	1	Thoracotor	19	CHG	No	71	0	1
0	0	VATS or ro	19	CHG	No	55	0	0
0	0	VATS or ro	19	CHG	No	60	0	1
0	0	VATS or ro	19	CHG	No	76	1	0
0	0	VATS or ro	19	CHG	No	75	1	0
0	0	VATS or ro	19	CHG	No	84	0	0
0	0	VATS or ro	19	CHG	No	67	1	1
0	0	Thoracotor	19	CHG	No	58	0	1
0	0	Thoracotor	19	CHG	No	60	0	0
0	0	Thoracotor	19	CHG	No	75	0	0
0	0	VATS or ro	19	CHG	No	77	0	0
0	0	VATS or ro	19	CHG	No	56	1	0
0	0	Thoracotor	19	CHG	No	61	0	1
0	0	VATS or ro	19	CHG	No	73	1	0
0	1	Thoracotor	19	CHG	No	63	0	1
0	0	Thoracotor	19	CHG	No	52	0	1
0	0	VATS or ro	19	CHG	No	69	1	0
0	0	VATS or ro	19	CHG	No	86	0	1
0	0	VATS or ro	19	CHG	No	76	0	0
0	0	VATS or ro	19	CHG	No	60	0	1
0	0	Thoracotor	19	CHG	No	62	0	1
0	0	Thoracotor	19	CHG	No	56	0	0
0	0	VATS or ro	19	CHG	No	63	0	1
0	0	VATS or ro	19	CHG	No	75	0	1
0	0	VATS or ro	19	CHG	No	78	0	1
0	0	VATS or ro	19	CHG	No	73	0	1
0	0	VATS or ro	19	CHG	No	67	0	0
0	0	VATS or ro	19	CHG	No	86	0	0
0	0	VATS or ro	19	CHG	No	53	0	0
0	0	VATS or ro	19	CHG	No	67	0	0
0	0	Thoracotor	19	CHG	No	68	0	1
0	0	VATS or ro	19	CHG	No	69	0	0
0	0	VATS or ro	19	CHG	No	62	0	0
0	0	VATS or ro	19	CHG	No	79	1	0
0	0	Thoracotor	19	CHG	No	62	0	1
0	0	Thoracotor	19	CHG	No	73	1	1
0	0	VATS or ro	19	CHG	No	73	0	1

0	0	VATS or rc	19	CHG	No	79	0	0
0	0	VATS or rc	19	CHG	No	76	0	1
0	1	Thoracotoı	19	CHG	Yes	69	0	0
0	0	Thoracotoı	19	CHG	No	35	0	1
0	0	Thoracotoı	19	CHG	Yes	75	0	1
0	0	VATS or rc	19	CHG	No	63	0	1
0	0	VATS or rc	19	CHG	No	71	0	1
0	0	Thoracotoı	19	CHG	No	74	0	1
0	0	VATS or rc	19	CHG	No	70	1	1
0	0	VATS or rc	19	CHG	No	67	0	1
0	0	VATS or rc	19	CHG	No	81	0	0
0	0	VATS or rc	19	CHG	No	70	1	1
0	0	VATS or rc	19	CHG	No	73	0	1
0	0	Thoracotoı	19	CHG	No	65	0	1
0	0	VATS or rc	19	CHG	No	64	0	1
0	1	Thoracotoı	19	CHG	No	55	0	1
0	0	Thoracotoı	19	CHG	No	63	1	0
0	0	VATS or rc	19	CHG	No	67	0	0
0	0	VATS or rc	19	CHG	No	89	0	0
0	0	VATS or rc	19	CHG	No	84	0	0
1	0	Thoracotoı	187	CHU	No	47	0	1
1	0	VATS or rc	187	CHU	No	65	0	0
1	0	Thoracotoı	187	CHU	No	56	0	0
1	0	VATS or rc	187	CHU	No	60	1	0
1	0	Thoracotoı	187	CHU	No	66	0	0
1	0	VATS or rc	187	CHU	No	44	0	0
1	0	Thoracotoı	187	CHU	No	65	0	0
1	0	VATS or rc	187	CHU	No	68	0	1
1	0	VATS or rc	187	CHU	No	70	0	0
1	0	Thoracotoı	187	CHU	No	80	0	0
1	0	Thoracotoı	187	CHU	No	58	0	1
1	0	VATS or rc	187	CHU	No	62	0	0
1	0	Thoracotoı	187	CHU	No	48	1	0
1	0	VATS or rc	187	CHU	No	44	0	1
1	0	Thoracotoı	187	CHU	No	65	0	0
1	0	Thoracotoı	187	CHU	No	74	0	1
1	0	VATS or rc	187	CHU	No	74	0	0
1	0	Thoracotoı	187	CHU	No	60	1	1
1	0	Thoracotoı	187	CHU	No	63	0	0
1	0	Thoracotoı	187	CHU	No	57	1	1
1	0	Thoracotoı	187	CHU	No	61	0	1
1	0	VATS or rc	187	CHU	No	71	0	0
1	0	Thoracotoı	187	CHU	No	73	0	0
1	0	Thoracotoı	187	CHU	No	52	0	1
1	0	Thoracotoı	187	CHU	No	51	0	1
1	0	Thoracotoı	187	CHU	No	72	0	1
1	0	Thoracotoı	187	CHU	No	66	0	1
1	0	VATS or rc	187	CHU	No	50	0	0
1	0	VATS or rc	187	CHU	No	70	0	1
1	0	Thoracotoı	187	CHU	No	60	0	1
1	0	VATS or rc	187	CHU	No	60	0	1
1	0	VATS or rc	187	CHU	No	64	0	0
1	0	Thoracotoı	187	CHU	No	80	0	1
1	0	VATS or rc	187	CHU	No	58	1	0
1	0	Thoracotoı	187	CHU	No	67	0	1
1	0	Thoracotoı	187	CHU	No	72	0	0

1	0	VATS or rc	187	CHU	No	52	0	1
1	0	Thoracotoı	187	CHU	No	67	0	1
1	0	Thoracotoı	187	CHU	No	65	0	1
1	0	VATS or rc	187	CHU	No	62	0	0
1	1	Thoracotoı	187	CHU	No	58	0	0
1	0	Thoracotoı	187	CHU	No	66	0	0
1	0	Thoracotoı	187	CHU	No	60	0	1
1	0	Thoracotoı	187	CHU	No	69	0	0
1	1	Thoracotoı	187	CHU	No	70	0	1
1	1	Thoracotoı	187	CHU	No	57	1	1
1	0	Thoracotoı	187	CHU	No	69	1	0
1	0	VATS or rc	187	CHU	No	54	0	0
1	0	VATS or rc	187	CHU	No	59	0	1
1	1	Thoracotoı	187	CHU	No	53	0	1
1	0	Thoracotoı	187	CHU	No	71	0	0
1	0	Thoracotoı	187	CHU	No	79	0	0
1	0	Thoracotoı	187	CHU	No	58	1	0
1	0	VATS or rc	187	CHU	No	68	0	0
1	0	Thoracotoı	187	CHU	No	47	0	1
1	0	Thoracotoı	187	CHU	No	56	1	0
1	0	Thoracotoı	187	CHU	No	56	0	0
1	0	VATS or rc	187	CHU	No	74	0	1
1	0	Thoracotoı	187	CHU	No	60	0	1
1	0	Thoracotoı	187	CHU	No	68	0	0
1	1	Thoracotoı	187	CHU	No	69	0	0
1	0	Thoracotoı	187	CHU	No	74	0	1
1	0	Thoracotoı	187	CHU	No	65	1	0
1	0	Thoracotoı	187	CHU	No	63	0	1
1	0	Thoracotoı	187	CHU	No	80	0	0
1	0	VATS or rc	187	CHU	No	61	0	1
1	1	Thoracotoı	187	CHU	No	66	0	1
1	0	VATS or rc	187	CHU	No	60	0	1
1	0	VATS or rc	187	CHU	No	76	0	0
1	0	Thoracotoı	187	CHU	No	68	0	0
1	0	VATS or rc	187	CHU	No	70	0	0
1	0	Thoracotoı	187	CHU	No	72	0	0
1	0	Thoracotoı	187	CHU	No	67	0	1
1	0	Thoracotoı	187	CHU	No	69	0	0
1	0	VATS or rc	187	CHU	No	55	0	1
1	0	Thoracotoı	187	CHU	No	68	0	0
1	0	Thoracotoı	187	CHU	No	68	0	0
1	0	VATS or rc	187	CHU	No	54	0	1
1	0	Thoracotoı	187	CHU	No	77	0	0
1	1	Thoracotoı	187	CHU	No	69	0	0
1	0	VATS or rc	187	CHU	No	65	0	0
1	0	Thoracotoı	187	CHU	No	72	0	0
1	0	VATS or rc	187	CHU	No	74	0	1
1	0	Thoracotoı	187	CHU	No	61	0	0
1	1	Thoracotoı	187	CHU	No	66	0	0
1	0	Thoracotoı	187	CHU	No	65	0	1
1	0	VATS or rc	187	CHU	No	73	0	0
1	0	Thoracotoı	187	CHU	No	68	0	0
1	0	VATS or rc	187	CHU	No	59	0	1
1	0	Thoracotoı	187	CHU	No	75	0	0
1	0	VATS or rc	187	CHU	No	60	0	1
1	0	VATS or rc	187	CHU	No	55	0	1

1	0	VATS or rc	187	CHU	No	69	0	1
1	0	Thoracotoı	187	CHU	No	70	0	0
1	0	VATS or rc	187	CHU	No	69	1	0
1	0	Thoracotoı	187	CHU	No	55	0	0
1	0	Thoracotoı	187	CHU	No	70	0	1
1	0	Thoracotoı	187	CHU	No	50	0	1
1	0	VATS or rc	187	CHU	No	72	0	0
1	0	VATS or rc	187	CHU	No	58	0	1
1	0	Thoracotoı	187	CHU	No	73	0	0
1	0	VATS or rc	187	CHU	No	45	0	1
1	1	Thoracotoı	187	CHU	No	50	0	0
1	0	VATS or rc	187	CHU	No	72	0	0
1	0	Thoracotoı	187	CHU	No	54	0	1
1	0	Thoracotoı	187	CHU	No	58	0	0
1	0	Thoracotoı	187	CHU	No	75	0	1
1	1	Thoracotoı	187	CHU	No	62	0	1
1	0	Thoracotoı	187	CHU	No	58	0	0
1	0	VATS or rc	187	CHU	No	52	0	0
1	0	VATS or rc	187	CHU	No	75	0	0
1	0	Thoracotoı	187	CHU	No	72	0	1
1	0	Thoracotoı	187	CHU	No	65	0	0
1	0	Thoracotoı	187	CHU	No	75	0	0
1	0	Thoracotoı	187	CHU	No	66	0	0
1	0	Thoracotoı	187	CHU	No	78	1	0
1	0	VATS or rc	187	CHU	No	66	1	1
1	0	VATS or rc	187	CHU	No	64	0	1
1	0	Thoracotoı	187	CHU	No	65	1	0
1	0	VATS or rc	187	CHU	No	60	0	0
1	0	Thoracotoı	187	CHU	No	66	0	1
1	0	Thoracotoı	187	CHU	No	53	0	1
1	0	Thoracotoı	187	CHU	No	67	0	0
1	0	VATS or rc	187	CHU	No	79	1	0
1	0	VATS or rc	187	CHU	No	70	0	0
1	0	Thoracotoı	187	CHU	No	69	0	0
1	0	VATS or rc	187	CHU	No	66	0	0
1	0	Thoracotoı	187	CHU	No	50	0	1
1	0	Thoracotoı	187	CHU	No	69	0	0
1	0	Thoracotoı	187	CHU	No	50	0	1
1	0	Thoracotoı	187	CHU	No	65	0	0
1	0	VATS or rc	187	CHU	No	46	0	1
1	0	Thoracotoı	187	CHU	No	55	0	1
1	0	Thoracotoı	187	CHU	No	71	1	1
1	0	Thoracotoı	187	CHU	No	74	1	1
1	0	Thoracotoı	187	CHU	No	72	0	0
1	0	Thoracotoı	187	CHU	No	86	1	0
1	0	Thoracotoı	187	CHU	No	58	0	1
1	0	Thoracotoı	187	CHU	No	33	0	0
1	1	Thoracotoı	187	CHU	No	68	0	0
1	0	Thoracotoı	187	CHU	No	58	0	1
1	0	Thoracotoı	187	CHU	No	72	0	1
1	0	Thoracotoı	187	CHU	No	67	0	0
1	0	Thoracotoı	187	CHU	No	63	0	1
1	0	VATS or rc	187	CHU	No	55	0	0
1	0	Thoracotoı	187	CHU	No	78	0	0
1	0	VATS or rc	187	CHU	No	57	0	1
1	0	VATS or rc	187	CHU	No	66	0	0

1	0	Thoracotoı	187	CHU	No	57	0	1
1	0	Thoracotoı	187	CHU	No	63	0	0
1	0	Thoracotoı	187	CHU	No	46	0	0
1	0	Thoracotoı	187	CHU	No	63	0	0
1	0	Thoracotoı	187	CHU	No	46	0	1
1	0	Thoracotoı	187	CHU	No	61	0	0
1	0	Thoracotoı	187	CHU	No	58	0	1
1	0	Thoracotoı	187	CHU	No	67	0	0
1	0	VATS or rc	187	CHU	No	52	0	0
1	0	Thoracotoı	187	CHU	No	69	0	1
1	0	VATS or rc	187	CHU	No	47	0	1
1	0	Thoracotoı	187	CHU	No	61	0	0
1	0	VATS or rc	187	CHU	No	51	0	0
1	0	Thoracotoı	187	CHU	No	71	1	0
1	0	VATS or rc	187	CHU	No	61	0	0
1	0	VATS or rc	187	CHU	No	63	0	1
1	0	VATS or rc	187	CHU	No	67	0	0
1	1	Thoracotoı	187	CHU	No	68	0	0
1	1	Thoracotoı	187	CHU	No	48	0	1
1	0	Thoracotoı	187	CHU	No	80	0	0
1	0	VATS or rc	187	CHU	No	71	0	1
1	0	VATS or rc	187	CHU	No	70	0	0
1	0	VATS or rc	187	CHU	No	65	0	0
1	0	Thoracotoı	187	CHU	No	70	0	1
1	0	VATS or rc	187	CHU	No	72	0	0
1	0	VATS or rc	187	CHU	No	68	0	1
1	0	VATS or rc	187	CHU	No	58	0	1
1	0	Thoracotoı	187	CHU	No	65	0	0
1	0	VATS or rc	187	CHU	No	74	0	0
1	0	Thoracotoı	187	CHU	No	55	0	0
1	0	Thoracotoı	187	CHU	No	53	0	1
1	0	VATS or rc	187	CHU	No	67	1	0
1	0	Thoracotoı	187	CHU	No	50	0	1
1	0	Thoracotoı	187	CHU	No	63	0	1
1	0	VATS or rc	187	CHU	No	73	1	1
1	0	VATS or rc	187	CHU	No	57	0	1
1	0	VATS or rc	187	CHU	No	67	0	0
1	0	Thoracotoı	187	CHU	No	71	0	1
1	0	Thoracotoı	187	CHU	No	63	0	0
1	0	VATS or rc	187	CHU	No	70	0	0
1	0	Thoracotoı	187	CHU	No	72	1	1
1	0	Thoracotoı	187	CHU	No	69	0	0
1	0	Thoracotoı	187	CHU	No	63	0	1
1	0	Thoracotoı	187	CHU	No	63	0	0
1	0	Thoracotoı	187	CHU	No	63	0	1
1	0	VATS or rc	187	CHU	No	55	0	1
1	0	VATS or rc	187	CHU	No	60	1	0
1	0	Thoracotoı	187	CHU	No	79	0	0
1	0	Thoracotoı	187	CHU	No	70	0	1
1	0	Thoracotoı	187	CHU	No	61	0	1
1	0	Thoracotoı	187	CHU	No	78	0	0
1	0	VATS or rc	187	CHU	No	71	0	0
1	0	Thoracotoı	187	CHU	No	69	0	0
1	0	Thoracotoı	187	CHU	No	71	0	1
1	0	Thoracotoı	187	CHU	No	76	0	1
1	0	VATS or rc	187	CHU	No	61	0	0

1	0	VATS or rc	187	CHU	No	63	0	0
1	0	Thoracotoı	187	CHU	No	68	0	0
1	0	Thoracotoı	187	CHU	No	74	0	0
1	0	Thoracotoı	187	CHU	No	64	0	1
1	0	Thoracotoı	187	CHU	No	57	0	1
1	0	Thoracotoı	187	CHU	No	49	0	0
1	0	Thoracotoı	187	CHU	No	47	0	0
1	0	Thoracotoı	187	CHU	No	70	0	1
1	0	Thoracotoı	187	CHU	No	69	0	1
1	0	Thoracotoı	187	CHU	No	59	0	1
1	0	VATS or rc	187	CHU	No	60	0	0
1	0	Thoracotoı	187	CHU	No	70	0	1
1	0	VATS or rc	187	CHU	No	75	0	0
1	0	Thoracotoı	187	CHU	No	72	0	0
1	0	VATS or rc	187	CHU	No	63	0	1
1	0	VATS or rc	187	CHU	No	56	0	0
1	0	Thoracotoı	187	CHU	No	68	0	0
1	0	VATS or rc	187	CHU	No	57	0	1
1	0	Thoracotoı	187	CHU	No	59	1	1
1	0	VATS or rc	187	CHU	No	70	0	0
1	0	Thoracotoı	187	CHU	No	48	0	1
1	0	Thoracotoı	187	CHU	No	71	0	0
1	0	Thoracotoı	187	CHU	No	56	0	1
1	0	VATS or rc	187	CHU	No	60	0	1
1	0	Thoracotoı	187	CHU	No	49	0	1
1	0	Thoracotoı	187	CHU	No	57	1	0
1	0	Thoracotoı	187	CHU	No	57	0	1
1	0	VATS or rc	187	CHU	No	69	0	0
1	0	VATS or rc	187	CHU	No	67	1	0
1	0	Thoracotoı	187	CHU	No	59	0	0
1	0	Thoracotoı	187	CHU	No	58	0	0
1	0	Thoracotoı	187	CHU	No	59	0	1
1	0	VATS or rc	187	CHU	No	47	0	1
1	0	VATS or rc	187	CHU	No	65	0	0
1	0	Thoracotoı	187	CHU	No	52	0	1
1	0	VATS or rc	187	CHU	No	67	0	0
1	0	VATS or rc	187	CHU	No	66	0	0
1	0	Thoracotoı	187	CHU	No	46	0	0
1	0	Thoracotoı	187	CHU	No	65	0	0
1	0	VATS or rc	187	CHU	No	54	0	1
1	0	VATS or rc	187	CHU	No	74	0	1
1	1	Thoracotoı	187	CHU	No	66	0	0
1	1	Thoracotoı	187	CHU	No	66	0	0
1	1	Thoracotoı	187	CHU	No	52	0	1
1	0	Thoracotoı	187	CHU	No	62	0	1
1	0	Thoracotoı	187	CHU	No	47	0	0
1	0	Thoracotoı	187	CHU	No	75	0	1
1	0	VATS or rc	187	CHU	No	62	0	1
1	0	Thoracotoı	187	CHU	No	51	0	1
1	0	Thoracotoı	187	CHU	No	67	0	1
1	0	Thoracotoı	187	CHU	No	63	0	1
1	0	Thoracotoı	187	CHU	No	75	0	0
1	0	VATS or rc	187	CHU	No	67	1	1
1	0	Thoracotoı	187	CHU	No	66	0	0
1	0	Thoracotoı	187	CHU	No	73	0	0
1	0	Thoracotoı	187	CHU	No	72	0	0

1	0	VATS or rc	187	CHU	No	69	0	0
1	0	Thoracotoı	187	CHU	No	66	0	1
1	0	VATS or rc	187	CHU	No	66	0	0
1	0	Thoracotoı	187	CHU	No	70	0	0
1	0	Thoracotoı	187	CHU	No	72	0	0
1	1	Thoracotoı	187	CHU	No	53	0	1
1	0	Thoracotoı	187	CHU	No	62	0	1
1	0	Thoracotoı	187	CHU	No	67	0	1
1	0	VATS or rc	187	CHU	No	76	0	0
1	0	Thoracotoı	187	CHU	No	69	0	1
1	0	Thoracotoı	187	CHU	No	65	0	1
1	0	Thoracotoı	187	CHU	No	58	0	0
1	0	Thoracotoı	187	CHU	No	71	0	0
1	0	VATS or rc	187	CHU	No	75	0	0
1	0	Thoracotoı	187	CHU	No	85	0	0
1	0	Thoracotoı	187	CHU	No	65	0	0
1	0	Thoracotoı	187	CHU	No	64	0	0
1	0	Thoracotoı	187	CHU	No	60	0	1
1	0	Thoracotoı	187	CHU	No	78	0	1
1	0	Thoracotoı	187	CHU	No	58	0	0
1	0	VATS or rc	187	CHU	No	53	0	1
1	0	Thoracotoı	187	CHU	No	61	0	0
1	0	Thoracotoı	187	CHU	No	72	0	0
1	0	VATS or rc	187	CHU	No	76	0	0
1	0	Thoracotoı	187	CHU	No	65	0	1
1	0	Thoracotoı	187	CHU	No	65	0	1
1	0	Thoracotoı	187	CHU	No	57	0	0
1	0	Thoracotoı	187	CHU	Yes	86	0	1
1	0	Thoracotoı	187	CHU	No	71	0	1
1	0	Thoracotoı	187	CHU	No	53	0	0
1	0	VATS or rc	187	CHU	Yes	66	0	1
1	0	Thoracotoı	187	CHU	No	69	0	0
1	0	Thoracotoı	187	CHU	No	48	0	0
1	0	Thoracotoı	187	CHU	No	57	0	1
1	0	VATS or rc	187	CHU	No	70	0	1
1	0	Thoracotoı	187	CHU	No	47	0	1
1	0	Thoracotoı	187	CHU	No	71	0	0
1	0	VATS or rc	187	CHU	No	53	0	1
1	0	Thoracotoı	187	CHU	No	67	0	0
1	1	Thoracotoı	187	CHU	No	43	0	1
1	0	VATS or rc	187	CHU	No	68	0	0
1	0	Thoracotoı	187	CHU	No	56	0	1
1	0	Thoracotoı	187	CHU	No	66	0	0
1	0	VATS or rc	187	CHU	No	59	0	1
1	0	Thoracotoı	187	CHU	No	66	1	0
1	0	Thoracotoı	187	CHU	No	67	0	0
1	0	VATS or rc	187	CHU	No	75	0	0
1	0	VATS or rc	187	CHU	No	69	0	1
1	0	Thoracotoı	187	CHU	No	51	0	1
1	0	Thoracotoı	187	CHU	No	77	1	1
1	0	VATS or rc	187	CHU	No	66	0	1
1	0	Thoracotoı	187	CHU	No	70	0	0
1	0	VATS or rc	187	CHU	No	59	0	0
1	0	VATS or rc	187	CHU	No	62	0	0
1	0	Thoracotoı	187	CHU	No	65	1	0
1	0	Thoracotoı	187	CHU	No	76	1	0

1	0	Thoracotoı	187	CHU	No	65	0	1
1	0	Thoracotoı	187	CHU	No	76	0	1
1	0	VATS or rc	187	CHU	No	71	0	0
1	0	Thoracotoı	187	CHU	No	76	0	0
1	0	Thoracotoı	187	CHU	No	65	0	0
1	0	Thoracotoı	187	CHU	No	79	0	0
1	0	Thoracotoı	187	CHU	No	50	0	1
1	0	Thoracotoı	187	CHU	No	62	0	0
1	0	Thoracotoı	187	CHU	No	64	0	1
1	0	VATS or rc	187	CHU	No	52	0	1
1	0	VATS or rc	187	CHU	No	66	1	1
1	0	Thoracotoı	187	CHU	No	56	0	0
1	0	Thoracotoı	187	CHU	No	76	0	0
1	0	Thoracotoı	187	CHU	No	72	0	0
1	0	VATS or rc	187	CHU	No	72	1	0
1	0	VATS or rc	187	CHU	No	81	0	0
1	0	VATS or rc	187	CHU	No	69	0	1
1	1	Thoracotoı	187	CHU	No	62	0	0
1	0	VATS or rc	187	CHU	No	57	0	1
1	0	VATS or rc	187	CHU	No	53	0	0
1	0	Thoracotoı	187	CHU	No	69	0	1
1	0	Thoracotoı	187	CHU	No	59	0	0
1	0	Thoracotoı	187	CHU	No	76	0	1
1	0	Thoracotoı	187	CHU	No	53	0	1
1	0	Thoracotoı	187	CHU	No	64	0	1
1	0	Thoracotoı	187	CHU	No	72	0	0
1	0	Thoracotoı	187	CHU	No	79	1	0
1	0	Thoracotoı	187	CHU	No	67	0	0
1	0	VATS or rc	187	CHU	No	58	0	0
1	1	Thoracotoı	187	CHU	Yes	77	0	0
1	0	Thoracotoı	187	CHU	No	29	0	0
1	0	Thoracotoı	187	CHU	No	69	0	1
1	0	Thoracotoı	187	CHU	No	55	0	1
1	0	Thoracotoı	187	CHU	No	30	0	0
1	0	VATS or rc	187	CHU	No	74	0	0
1	0	Thoracotoı	187	CHU	No	63	0	0
1	0	VATS or rc	187	CHU	No	66	0	0
1	0	Thoracotoı	187	CHU	No	57	0	0
1	0	VATS or rc	187	CHU	No	59	0	1
1	0	Thoracotoı	187	CHU	Yes	55	0	1
1	1	Thoracotoı	187	CHU	No	69	0	0
1	0	Thoracotoı	187	CHU	No	54	0	1
1	0	VATS or rc	187	CHU	No	64	0	0
1	0	Thoracotoı	187	CHU	No	72	0	1
1	0	Thoracotoı	187	CHU	No	64	0	1
1	0	VATS or rc	187	CHU	No	59	0	0
1	0	Thoracotoı	187	CHU	Yes	60	0	1
1	0	VATS or rc	187	CHU	No	67	1	0
1	0	Thoracotoı	187	CHU	No	56	0	0
1	0	VATS or rc	187	CHU	No	61	0	0
1	0	VATS or rc	187	CHU	No	49	0	1
1	0	VATS or rc	187	CHU	No	78	0	0
1	0	Thoracotoı	187	CHU	No	49	0	1
1	0	Thoracotoı	187	CHU	No	72	0	0
1	0	Thoracotoı	187	CHU	No	69	0	0
1	0	Thoracotoı	187	CHU	No	48	0	1

1	0	Thoracotoı	187	CHU	No	65	0	1
1	0	Thoracotoı	187	CHU	No	54	0	1
1	0	Thoracotoı	187	CHU	No	77	0	1
1	0	Thoracotoı	187	CHU	No	60	0	1
1	0	VATS or rc	187	CHU	No	81	0	1
1	0	Thoracotoı	187	CHU	No	52	0	1
1	0	VATS or rc	187	CHU	No	62	0	0
1	0	Thoracotoı	187	CHU	No	64	0	1
1	1	Thoracotoı	187	CHU	No	74	0	1
1	0	Thoracotoı	187	CHU	No	71	0	1
1	0	Thoracotoı	187	CHU	No	54	0	1
1	0	VATS or rc	187	CHU	No	78	0	0
1	0	Thoracotoı	187	CHU	No	73	0	0
1	0	Thoracotoı	187	CHU	No	51	0	1
1	0	VATS or rc	187	CHU	No	69	0	0
1	0	VATS or rc	187	CHU	No	64	0	1
1	0	VATS or rc	187	CHU	No	63	0	0
1	0	Thoracotoı	187	CHU	No	76	0	0
1	0	Thoracotoı	187	CHU	No	61	1	0
1	0	Thoracotoı	187	CHU	No	39	0	0
1	0	Thoracotoı	187	CHU	No	67	0	1
1	1	Thoracotoı	187	CHU	No	75	0	0
1	0	Thoracotoı	187	CHU	No	59	0	0
1	0	Thoracotoı	187	CHU	No	50	0	1
1	0	Thoracotoı	187	CHU	No	76	0	0
1	0	Thoracotoı	187	CHU	No	62	0	0
1	0	VATS or rc	187	CHU	No	78	1	0
1	0	Thoracotoı	187	CHU	No	57	0	1
1	0	Thoracotoı	187	CHU	No	82	0	1
1	0	Thoracotoı	187	CHU	No	82	1	0
1	0	Thoracotoı	187	CHU	No	67	0	1
1	0	Thoracotoı	187	CHU	No	68	0	0
1	0	Thoracotoı	187	CHU	No	62	0	1
1	0	Thoracotoı	187	CHU	Yes	67	0	0
1	0	VATS or rc	187	CHU	No	55	0	0
1	1	Thoracotoı	187	CHU	No	67	0	0
1	0	VATS or rc	187	CHU	No	69	0	0
1	0	Thoracotoı	187	CHU	No	73	0	1
1	0	Thoracotoı	187	CHU	No	72	0	1
1	0	Thoracotoı	187	CHU	No	68	0	1
1	0	Thoracotoı	187	CHU	No	53	0	1
1	0	VATS or rc	187	CHU	No	79	0	0
1	1	Thoracotoı	187	CHU	No	64	0	1
1	0	Thoracotoı	187	CHU	No	53	1	1
1	0	VATS or rc	187	CHU	No	66	0	0
1	0	Thoracotoı	187	CHU	No	57	1	1
1	0	VATS or rc	187	CHU	No	78	0	0
1	1	Thoracotoı	187	CHU	No	73	0	1
1	0	Thoracotoı	187	CHU	No	48	0	0
1	0	Thoracotoı	187	CHU	No	58	0	1
1	0	VATS or rc	187	CHU	No	58	1	1
1	0	VATS or rc	187	CHU	No	69	0	1
1	0	Thoracotoı	187	CHU	No	62	0	0
1	0	VATS or rc	187	CHU	No	67	0	0
1	0	Thoracotoı	187	CHU	No	77	0	0
1	0	Thoracotoı	187	CHU	No	69	0	1

1	0	Thoracotoı	187	CHU	No	59	0	1
1	0	VATS or rc	187	CHU	No	60	0	0
1	0	Thoracotoı	187	CHU	No	65	0	0
1	0	Thoracotoı	187	CHU	No	59	0	1
1	1	Thoracotoı	187	CHU	No	66	0	0
1	0	Thoracotoı	187	CHU	No	75	0	1
1	0	VATS or rc	187	CHU	No	64	1	1
1	0	VATS or rc	187	CHU	No	69	1	1
1	0	VATS or rc	187	CHU	No	77	0	0
1	0	Thoracotoı	187	CHU	No	60	0	0
1	0	Thoracotoı	187	CHU	No	58	0	1
1	0	VATS or rc	187	CHU	No	69	0	0
1	0	Thoracotoı	187	CHU	No	64	0	0
1	0	Thoracotoı	187	CHU	No	64	0	1
1	0	Thoracotoı	187	CHU	No	72	0	0
1	0	Thoracotoı	187	CHU	No	60	1	1
1	0	Thoracotoı	187	CHU	Yes	62	0	1
1	0	VATS or rc	187	CHU	No	63	0	1
1	0	VATS or rc	187	CHU	No	69	0	0
1	0	Thoracotoı	187	CHU	No	66	0	1
1	0	Thoracotoı	187	CHU	No	43	0	1
1	0	Thoracotoı	187	CHU	No	58	0	1
1	0	Thoracotoı	187	CHU	No	63	0	1
1	0	VATS or rc	187	CHU	No	76	0	0
1	0	Thoracotoı	187	CHU	No	71	0	1
1	1	Thoracotoı	187	CHU	No	73	0	1
1	0	Thoracotoı	187	CHU	No	74	0	0
1	0	Thoracotoı	187	CHU	No	74	0	0
1	0	VATS or rc	187	CHU	No	55	0	0
1	0	VATS or rc	187	CHU	No	74	0	0
1	0	Thoracotoı	187	CHU	No	57	0	0
1	0	Thoracotoı	187	CHU	No	43	0	0
1	1	Thoracotoı	187	CHU	No	64	0	0
1	0	VATS or rc	187	CHU	No	78	0	0
1	0	VATS or rc	187	CHU	No	67	0	1
1	0	Thoracotoı	187	CHU	No	63	0	0
1	0	VATS or rc	187	CHU	No	75	0	0
1	0	Thoracotoı	187	CHU	No	74	0	1
1	1	Thoracotoı	187	CHU	No	68	1	1
1	0	Thoracotoı	187	CHU	No	58	0	1
1	0	Thoracotoı	187	CHU	No	70	0	0
1	1	Thoracotoı	187	CHU	No	70	0	0
1	0	Thoracotoı	187	CHU	No	75	0	1
1	1	Thoracotoı	187	CHU	No	65	0	0
1	0	VATS or rc	187	CHU	No	68	0	0
1	0	Thoracotoı	187	CHU	No	73	0	0
1	0	VATS or rc	187	CHU	No	66	0	0
1	0	Thoracotoı	187	CHU	No	61	0	0
1	0	Thoracotoı	102	CHU	No	62	0	0
1	0	Thoracotoı	102	CHU	No	54	1	1
1	0	Thoracotoı	102	CHU	No	57	0	0
1	0	Thoracotoı	102	CHU	No	62	1	1
1	0	Thoracotoı	102	CHU	No	40	0	1
1	0	Thoracotoı	102	CHU	No	72	0	0
1	0	Thoracotoı	102	CHU	No	67	0	0
1	0	Thoracotoı	102	CHU	No	69	0	1

1	0	Thoracotoı	102	CHU	No	61	0	0
1	0	VATS or rc	102	CHU	No	64	0	1
1	0	Thoracotoı	102	CHU	No	74	0	0
1	0	Thoracotoı	102	CHU	No	64	0	0
1	0	VATS or rc	102	CHU	No	82	0	0
1	0	Thoracotoı	102	CHU	No	69	0	0
1	0	VATS or rc	102	CHU	No	59	0	1
1	0	Thoracotoı	102	CHU	No	67	0	0
1	0	Thoracotoı	102	CHU	No	56	0	0
1	0	Thoracotoı	102	CHU	No	81	0	0
1	0	VATS or rc	102	CHU	No	67	0	0
1	0	Thoracotoı	102	CHU	No	68	0	0
1	0	Thoracotoı	102	CHU	No	69	0	0
1	0	Thoracotoı	102	CHU	No	59	0	1
1	0	VATS or rc	102	CHU	No	60	0	0
1	0	Thoracotoı	102	CHU	No	59	0	0
1	0	Thoracotoı	102	CHU	No	67	0	1
1	0	Thoracotoı	102	CHU	No	58	0	1
1	0	Thoracotoı	102	CHU	No	65	0	0
1	0	Thoracotoı	102	CHU	No	72	0	0
1	1	Thoracotoı	102	CHU	No	70	0	0
1	0	VATS or rc	102	CHU	No	77	0	0
1	1	VATS or rc	102	CHU	No	69	0	0
1	0	Thoracotoı	102	CHU	No	43	0	0
1	0	Thoracotoı	102	CHU	No	53	0	1
1	0	VATS or rc	102	CHU	No	56	0	0
1	0	VATS or rc	102	CHU	No	64	0	1
1	0	Thoracotoı	102	CHU	No	55	0	1
1	0	VATS or rc	102	CHU	No	48	0	0
1	0	VATS or rc	102	CHU	No	78	0	0
1	0	Thoracotoı	102	CHU	No	62	1	0
1	0	Thoracotoı	102	CHU	No	68	0	0
1	0	Thoracotoı	102	CHU	No	53	0	1
1	0	Thoracotoı	102	CHU	No	62	0	0
1	0	VATS or rc	102	CHU	No	75	0	0
1	0	Thoracotoı	102	CHU	No	66	0	0
1	0	Thoracotoı	102	CHU	No	60	0	0
1	0	Thoracotoı	102	CHU	No	72	0	0
1	0	Thoracotoı	102	CHU	No	69	0	0
1	0	Thoracotoı	102	CHU	No	67	0	1
1	0	Thoracotoı	102	CHU	No	64	0	0
1	0	Thoracotoı	102	CHU	No	75	0	0
1	0	Thoracotoı	102	CHU	No	58	0	0
1	0	Thoracotoı	102	CHU	No	78	1	0
1	0	Thoracotoı	102	CHU	No	68	0	0
1	0	Thoracotoı	102	CHU	No	56	0	0
1	0	Thoracotoı	102	CHU	No	69	0	0
1	1	Thoracotoı	102	CHU	No	49	0	0
1	0	VATS or rc	102	CHU	No	61	0	1
1	0	VATS or rc	102	CHU	No	70	0	0
1	0	VATS or rc	102	CHU	No	54	0	0
1	0	Thoracotoı	102	CHU	No	77	1	0
1	0	Thoracotoı	102	CHU	No	62	0	0
1	1	Thoracotoı	102	CHU	No	77	0	0
1	0	Thoracotoı	102	CHU	No	45	0	1
1	0	VATS or rc	102	CHU	No	74	0	0

1	0	Thoracotoı	102	CHU	No	63	0	0
1	0	Thoracotoı	102	CHU	No	66	0	0
1	0	Thoracotoı	102	CHU	No	65	0	0
1	0	Thoracotoı	102	CHU	No	75	0	0
1	0	Thoracotoı	102	CHU	No	70	0	0
1	0	VATS or ro	102	CHU	No	50	0	0
1	0	Thoracotoı	102	CHU	No	29	0	0
1	0	VATS or ro	102	CHU	No	77	0	0
1	0	Thoracotoı	102	CHU	No	63	0	1
1	0	VATS or ro	102	CHU	No	71	0	0
1	0	VATS or ro	102	CHU	No	63	1	0
1	0	VATS or ro	102	CHU	No	63	0	0
1	0	Thoracotoı	102	CHU	No	65	0	0
1	0	Thoracotoı	102	CHU	No	67	0	0
1	0	Thoracotoı	102	CHU	No	76	0	0
1	0	VATS or ro	102	CHU	No	60	0	0
1	0	Thoracotoı	102	CHU	No	52	0	1
1	0	Thoracotoı	102	CHU	No	82	0	1
1	1	Thoracotoı	102	CHU	No	67	0	1
1	0	Thoracotoı	102	CHU	No	72	0	0
1	0	Thoracotoı	102	CHU	No	71	0	1
1	0	Thoracotoı	102	CHU	No	58	0	0
1	1	Thoracotoı	102	CHU	No	44	0	1
1	0	VATS or ro	102	CHU	No	66	0	0
1	0	Thoracotoı	102	CHU	No	64	0	0
1	0	Thoracotoı	102	CHU	No	74	0	0
1	0	Thoracotoı	102	CHU	No	50	0	1
1	0	VATS or ro	102	CHU	No	61	0	0
1	0	Thoracotoı	102	CHU	No	73	0	0
1	0	Thoracotoı	102	CHU	No	56	0	0
1	0	VATS or ro	102	CHU	No	70	0	0
1	0	Thoracotoı	102	CHU	No	73	1	0
1	0	Thoracotoı	102	CHU	No	64	0	0
1	0	Thoracotoı	102	CHU	No	64	1	0
1	0	VATS or ro	102	CHU	No	61	0	0
1	0	Thoracotoı	102	CHU	No	60	0	0
1	1	Thoracotoı	102	CHU	No	78	0	1
1	0	Thoracotoı	102	CHU	No	70	0	0
1	0	Thoracotoı	102	CHU	No	69	0	0
1	0	Thoracotoı	102	CHU	No	45	0	0
1	0	Thoracotoı	102	CHU	No	66	0	0
1	0	Thoracotoı	102	CHU	No	47	0	0
1	0	Thoracotoı	102	CHU	No	54	0	0
1	0	Thoracotoı	102	CHU	No	55	0	0
1	0	Thoracotoı	102	CHU	No	56	0	0
1	0	VATS or ro	102	CHU	No	67	1	0
1	0	VATS or ro	102	CHU	No	68	0	1
1	0	Thoracotoı	102	CHU	No	70	1	0
1	0	VATS or ro	102	CHU	No	45	0	1
1	0	Thoracotoı	102	CHU	No	70	0	1
1	0	VATS or ro	102	CHU	No	75	0	0
1	0	VATS or ro	102	CHU	No	61	0	0
1	0	Thoracotoı	102	CHU	No	48	0	0
1	0	Thoracotoı	102	CHU	No	58	0	0
1	0	Thoracotoı	102	CHU	No	67	1	0
1	0	Thoracotoı	102	CHU	No	74	0	0

1	0	VATS or ro	102	CHU	No	70	0	0
1	0	VATS or ro	102	CHU	No	74	0	1
1	0	Thoracotoı	102	CHU	No	63	0	0
1	1	Thoracotoı	102	CHU	No	82	0	0
1	0	Thoracotoı	102	CHU	No	75	0	0
1	0	VATS or ro	102	CHU	No	75	0	0
1	0	VATS or ro	102	CHU	No	59	0	1
1	0	Thoracotoı	102	CHU	No	67	0	0
1	0	VATS or ro	102	CHU	No	73	0	0
1	0	Thoracotoı	102	CHU	No	62	0	1
1	0	Thoracotoı	102	CHU	No	59	0	0
1	0	Thoracotoı	102	CHU	No	75	0	0
1	0	Thoracotoı	102	CHU	No	71	0	0
1	0	Thoracotoı	102	CHU	No	75	0	0
1	0	Thoracotoı	102	CHU	No	69	1	0
1	0	VATS or ro	102	CHU	No	77	0	0
1	0	Thoracotoı	102	CHU	No	63	0	0
1	0	VATS or ro	102	CHU	No	64	1	0
1	0	VATS or ro	102	CHU	No	72	1	0
1	0	Thoracotoı	102	CHU	No	40	0	1
1	0	Thoracotoı	102	CHU	No	60	0	1
1	0	Thoracotoı	102	CHU	Yes	77	1	0
1	0	Thoracotoı	102	CHU	No	73	0	1
1	0	VATS or ro	102	CHU	No	61	0	1
1	0	Thoracotoı	102	CHU	No	46	0	0
1	0	Thoracotoı	102	CHU	No	65	0	0
1	0	VATS or ro	102	CHU	No	58	0	1
1	0	Thoracotoı	102	CHU	No	68	0	0
1	0	Thoracotoı	102	CHU	No	54	0	0
1	0	Thoracotoı	102	CHU	No	80	0	0
1	0	VATS or ro	102	CHU	No	74	0	0
1	0	Thoracotoı	102	CHU	No	75	0	0
1	0	Thoracotoı	102	CHU	No	73	0	0
1	0	VATS or ro	102	CHU	Yes	79	0	0
1	0	Thoracotoı	102	CHU	No	66	0	0
1	0	Thoracotoı	102	CHU	Yes	65	1	0
1	0	Thoracotoı	102	CHU	No	74	0	0
1	0	VATS or ro	102	CHU	No	67	0	0
1	0	Thoracotoı	102	CHU	No	63	0	0
1	0	Thoracotoı	102	CHU	No	60	0	1
1	0	Thoracotoı	102	CHU	No	69	0	0
1	1	Thoracotoı	102	CHU	No	67	0	0
1	0	Thoracotoı	102	CHU	Yes	87	0	0
1	0	Thoracotoı	102	CHU	No	57	0	1
1	0	Thoracotoı	102	CHU	No	47	0	1
1	0	Thoracotoı	102	CHU	No	57	0	1
1	0	VATS or ro	102	CHU	No	48	0	0
1	0	Thoracotoı	102	CHU	No	75	0	0
1	0	Thoracotoı	102	CHU	No	58	0	1
1	0	Thoracotoı	102	CHU	No	60	0	0
1	0	VATS or ro	102	CHU	Yes	75	0	0
1	0	Thoracotoı	102	CHU	No	63	0	1
1	0	Thoracotoı	102	CHU	No	59	0	1
1	0	Thoracotoı	102	CHU	Yes	83	0	0
1	1	Thoracotoı	102	CHU	No	62	0	1
1	0	Thoracotoı	102	CHU	No	56	0	1

1	0	VATS or rc	102	CHU	No	73	0	0
1	0	Thoracotoı	102	CHU	No	55	0	0
1	0	VATS or rc	102	CHU	No	72	1	0
1	1	Thoracotoı	102	CHU	No	63	0	0
1	0	Thoracotoı	102	CHU	No	46	1	1
1	0	Thoracotoı	102	CHU	No	79	0	0
1	0	VATS or rc	102	CHU	No	62	0	0
1	0	Thoracotoı	102	CHU	No	70	0	1
1	0	Thoracotoı	102	CHU	No	76	0	1
1	0	Thoracotoı	102	CHU	No	61	0	0
1	0	Thoracotoı	102	CHU	No	60	0	1
1	0	Thoracotoı	102	CHU	No	69	0	1
1	0	VATS or rc	102	CHU	No	46	0	1
1	0	Thoracotoı	102	CHU	No	56	0	0
1	0	Thoracotoı	102	CHU	No	77	0	1
1	0	Thoracotoı	102	CHU	No	69	0	0
1	0	VATS or rc	102	CHU	No	75	0	1
1	0	Thoracotoı	102	CHU	No	65	1	0
1	0	Thoracotoı	102	CHU	No	74	0	0
1	0	VATS or rc	102	CHU	No	70	0	1
1	0	Thoracotoı	102	CHU	No	74	0	0
1	0	VATS or rc	102	CHU	No	81	0	0
1	0	VATS or rc	102	CHU	No	68	0	0
1	0	Thoracotoı	102	CHU	No	57	0	0
1	0	Thoracotoı	102	CHU	No	42	0	0
1	0	Thoracotoı	102	CHU	No	64	0	0
1	0	VATS or rc	102	CHU	No	73	1	1
1	1	Thoracotoı	102	CHU	No	69	0	0
1	0	Thoracotoı	102	CHU	No	81	0	0
1	0	Thoracotoı	102	CHU	No	53	0	0
1	0	Thoracotoı	102	CHU	No	56	0	0
1	0	Thoracotoı	102	CHU	No	72	0	0
1	0	Thoracotoı	102	CHU	No	60	0	0
1	0	VATS or rc	102	CHU	No	67	0	0
1	0	Thoracotoı	102	CHU	No	68	0	0
1	0	VATS or rc	102	CHU	No	65	1	1
1	1	Thoracotoı	102	CHU	No	54	1	0
1	0	VATS or rc	102	CHU	No	78	0	0
1	0	Thoracotoı	102	CHU	No	67	0	0
1	0	Thoracotoı	102	CHU	No	72	0	0
1	0	Thoracotoı	102	CHU	No	46	0	0
1	0	Thoracotoı	102	CHU	No	61	0	1
1	0	Thoracotoı	102	CHU	No	73	0	1
1	0	Thoracotoı	102	CHU	No	80	0	0
1	0	Thoracotoı	102	CHU	No	69	0	0
1	0	Thoracotoı	102	CHU	No	52	0	1
1	1	Thoracotoı	102	CHU	No	64	0	0
1	0	Thoracotoı	102	CHU	No	61	0	0
1	0	Thoracotoı	102	CHU	No	83	0	0
1	0	Thoracotoı	102	CHU	No	66	0	0
1	0	Thoracotoı	102	CHU	No	64	0	0
1	0	Thoracotoı	102	CHU	No	48	0	1
1	0	Thoracotoı	102	CHU	No	48	0	1
1	0	Thoracotoı	102	CHU	No	69	0	1
1	0	Thoracotoı	102	CHU	No	71	0	1
1	0	Thoracotoı	102	CHU	No	81	0	0

1	0	Thoracotoı	102	CHU	No	72	0	0
1	0	Thoracotoı	102	CHU	No	53	0	1
1	0	Thoracotoı	102	CHU	No	70	1	1
1	0	Thoracotoı	102	CHU	No	76	0	0
1	0	VATS or rc	102	CHU	No	65	0	0
1	0	Thoracotoı	102	CHU	No	68	0	0
1	0	Thoracotoı	102	CHU	No	83	1	0
1	0	Thoracotoı	102	CHU	No	71	0	0
1	0	VATS or rc	102	CHU	No	76	0	0
1	0	VATS or rc	102	CHU	No	41	0	1
1	0	VATS or rc	102	CHU	No	60	0	0
1	0	Thoracotoı	102	CHU	No	55	0	1
1	0	Thoracotoı	102	CHU	No	78	0	0
1	0	VATS or rc	102	CHU	No	53	0	0
1	0	Thoracotoı	102	CHU	No	59	0	0
1	0	Thoracotoı	102	CHU	No	74	1	0
1	1	Thoracotoı	102	CHU	Yes	71	0	0
1	0	VATS or rc	102	CHU	No	78	0	0
1	0	Thoracotoı	102	CHU	No	56	0	0
1	0	Thoracotoı	102	CHU	No	59	0	0
1	0	VATS or rc	102	CHU	No	56	0	0
1	0	Thoracotoı	102	CHU	No	65	0	0
1	0	VATS or rc	102	CHU	No	61	0	1
1	0	Thoracotoı	102	CHU	No	61	0	0
1	0	Thoracotoı	102	CHU	No	42	0	0
1	0	VATS or rc	102	CHU	No	60	0	1
1	0	Thoracotoı	102	CHU	No	60	0	0
1	0	VATS or rc	102	CHU	No	70	0	0
1	0	VATS or rc	102	CHU	No	80	0	0
1	0	VATS or rc	102	CHU	No	72	0	0
1	0	Thoracotoı	102	CHU	No	72	0	0
1	0	VATS or rc	102	CHU	No	59	0	0
1	0	VATS or rc	102	CHU	No	62	0	0
1	0	Thoracotoı	102	CHU	No	75	0	0
1	0	Thoracotoı	102	CHU	No	74	0	1
1	0	Thoracotoı	102	CHU	No	45	0	1
1	0	VATS or rc	102	CHU	No	78	0	0
1	0	Thoracotoı	102	CHU	No	56	0	0
1	0	Thoracotoı	102	CHU	No	59	0	0
1	0	Thoracotoı	102	CHU	No	76	0	0
0	0	Thoracotoı	112	CHG	No	56	0	1
0	0	Thoracotoı	112	CHG	No	57	1	0
0	0	VATS or rc	112	CHG	No	62	0	0
0	0	Thoracotoı	112	CHG	No	59	0	0
0	0	Thoracotoı	112	CHG	No	59	0	1
0	0	Thoracotoı	112	CHG	No	69	0	0
0	0	VATS or rc	112	CHG	No	64	1	0
0	0	Thoracotoı	112	CHG	No	55	0	0
0	0	Thoracotoı	112	CHG	No	73	0	0
0	0	Thoracotoı	112	CHG	No	72	0	0
0	0	Thoracotoı	112	CHG	No	75	1	1
0	0	Thoracotoı	112	CHG	No	61	0	0
0	0	VATS or rc	112	CHG	No	75	0	0
0	0	Thoracotoı	112	CHG	No	58	0	1
0	0	VATS or rc	112	CHG	No	66	0	0
0	0	Thoracotoı	112	CHG	No	68	0	1

0	0	Thoracotor	112	CHG	No	69	0	1
0	0	Thoracotor	112	CHG	No	70	0	1
0	0	Thoracotor	112	CHG	No	70	0	0
0	0	Thoracotor	112	CHG	No	63	0	0
0	0	Thoracotor	112	CHG	No	60	0	1
0	0	Thoracotor	112	CHG	No	61	0	1
0	0	Thoracotor	112	CHG	No	59	1	0
0	0	Thoracotor	112	CHG	No	67	0	0
0	0	Thoracotor	112	CHG	No	76	0	0
0	0	Thoracotor	112	CHG	No	66	0	0
0	0	Thoracotor	112	CHG	No	62	0	1
0	0	Thoracotor	112	CHG	No	67	0	0
0	0	Thoracotor	112	CHG	No	73	0	0
0	0	VATS or ro	112	CHG	No	57	0	0
0	1	Thoracotor	112	CHG	No	51	0	1
0	0	VATS or ro	112	CHG	No	64	0	0
0	0	Thoracotor	112	CHG	No	73	0	0
0	0	Thoracotor	112	CHG	No	50	0	0
0	0	Thoracotor	112	CHG	No	73	1	1
0	0	VATS or ro	112	CHG	No	64	0	1
0	1	Thoracotor	112	CHG	Yes	69	0	1
0	0	VATS or ro	112	CHG	No	64	0	1
0	0	Thoracotor	112	CHG	No	56	0	1
0	0	Thoracotor	112	CHG	No	77	0	0
0	0	Thoracotor	112	CHG	No	69	0	0
0	0	Thoracotor	112	CHG	No	66	0	0
0	0	Thoracotor	112	CHG	No	53	0	0
0	0	Thoracotor	112	CHG	No	84	0	0
0	0	VATS or ro	112	CHG	No	73	1	0
0	0	Thoracotor	112	CHG	No	35	0	1
0	0	Thoracotor	112	CHG	No	71	0	0
0	0	Thoracotor	112	CHG	No	66	0	0
0	0	Thoracotor	112	CHG	No	70	0	0
0	0	Thoracotor	112	CHG	No	69	0	0
0	0	VATS or ro	112	CHG	No	48	0	1
0	0	Thoracotor	112	CHG	No	63	0	1
0	0	Thoracotor	112	CHG	No	59	0	0
0	0	Thoracotor	112	CHG	No	65	0	0
0	0	Thoracotor	112	CHG	No	57	0	1
0	0	Thoracotor	112	CHG	No	58	0	0
0	0	Thoracotor	112	CHG	No	61	0	0
0	0	Thoracotor	112	CHG	No	73	0	0
0	0	Thoracotor	112	CHG	No	61	0	1
0	0	Thoracotor	112	CHG	No	78	0	0
0	0	Thoracotor	112	CHG	No	57	1	0
0	0	Thoracotor	112	CHG	No	64	0	1
0	0	Thoracotor	112	CHG	No	64	0	0
0	0	Thoracotor	112	CHG	No	67	0	1
0	0	Thoracotor	112	CHG	No	59	0	1
0	0	Thoracotor	112	CHG	No	68	0	1
0	0	Thoracotor	112	CHG	No	71	0	0
0	0	Thoracotor	112	CHG	No	59	0	0
0	0	Thoracotor	112	CHG	No	79	0	0
0	0	Thoracotor	112	CHG	No	71	0	0
0	0	VATS or ro	112	CHG	No	81	1	0
0	0	Thoracotor	112	CHG	No	52	0	0

0	0	Thoracotoı	112	CHG	No	70	0	0
0	0	Thoracotoı	112	CHG	No	53	0	1
0	0	Thoracotoı	112	CHG	No	50	0	1
0	0	Thoracotoı	112	CHG	Yes	78	0	0
0	0	Thoracotoı	112	CHG	No	78	0	0
0	0	VATS or rc	112	CHG	No	57	1	0
0	1	Thoracotoı	112	CHG	No	47	0	1
0	0	VATS or rc	112	CHG	No	65	0	0
0	0	Thoracotoı	112	CHG	No	58	0	1
0	0	Thoracotoı	112	CHG	No	58	1	0
0	0	Thoracotoı	112	CHG	No	65	0	0
0	0	Thoracotoı	112	CHG	No	64	0	0
0	0	Thoracotoı	112	CHG	No	47	0	1
0	0	Thoracotoı	112	CHG	No	71	0	0
0	0	Thoracotoı	112	CHG	No	67	0	0
0	0	Thoracotoı	112	CHG	No	68	0	0
0	0	Thoracotoı	112	CHG	No	53	0	0
0	0	Thoracotoı	112	CHG	No	56	0	0
0	0	Thoracotoı	112	CHG	No	50	0	0
0	0	Thoracotoı	112	CHG	No	55	0	1
0	0	Thoracotoı	112	CHG	No	52	0	1
0	0	Thoracotoı	112	CHG	No	63	0	0
0	0	Thoracotoı	112	CHG	No	46	0	1
0	0	VATS or rc	112	CHG	No	57	0	1
0	0	Thoracotoı	112	CHG	No	46	0	0
0	0	Thoracotoı	112	CHG	No	62	0	1
0	0	Thoracotoı	112	CHG	No	64	0	1
0	0	Thoracotoı	112	CHG	No	58	0	1
0	0	Thoracotoı	112	CHG	No	60	0	1
0	0	VATS or rc	112	CHG	No	67	0	0
0	1	Thoracotoı	112	CHG	No	55	0	1
0	0	Thoracotoı	112	CHG	No	65	0	0
0	0	Thoracotoı	112	CHG	No	59	0	1
0	0	VATS or rc	112	CHG	No	61	0	0
0	0	VATS or rc	112	CHG	No	65	0	0
0	0	Thoracotoı	112	CHG	No	53	0	1
0	0	Thoracotoı	112	CHG	No	67	0	0
0	0	Thoracotoı	112	CHG	No	60	0	1
0	0	VATS or rc	112	CHG	No	55	0	0
0	0	Thoracotoı	112	CHG	No	70	0	1
0	0	Thoracotoı	112	CHG	No	45	0	1
0	0	Thoracotoı	112	CHG	No	74	0	0
0	0	VATS or rc	112	CHG	No	68	0	0
0	0	VATS or rc	112	CHG	No	65	0	0
0	0	Thoracotoı	112	CHG	No	67	0	1
0	0	Thoracotoı	112	CHG	No	73	0	0
0	0	Thoracotoı	112	CHG	No	68	1	0
0	0	Thoracotoı	112	CHG	No	62	0	0
0	0	Thoracotoı	112	CHG	No	68	0	0
0	0	Thoracotoı	112	CHG	Yes	52	1	1
0	0	Thoracotoı	112	CHG	No	65	0	1
0	0	VATS or rc	112	CHG	No	64	0	0
0	0	Thoracotoı	112	CHG	No	69	0	1
0	0	Thoracotoı	112	CHG	No	65	0	0
0	0	Thoracotoı	112	CHG	No	59	0	1
0	0	VATS or rc	112	CHG	No	60	0	0

0	0	Thoracotoı	112	CHG	No	78	0	0
0	0	Thoracotoı	112	CHG	No	56	0	0
0	0	Thoracotoı	112	CHG	No	64	0	1
0	0	Thoracotoı	112	CHG	No	57	0	1
0	0	Thoracotoı	112	CHG	No	64	1	0
0	0	Thoracotoı	112	CHG	No	68	0	0
0	0	Thoracotoı	112	CHG	No	58	0	1
0	0	Thoracotoı	112	CHG	No	57	0	1
0	0	Thoracotoı	112	CHG	No	65	0	1
0	0	Thoracotoı	112	CHG	No	57	0	0
0	0	Thoracotoı	112	CHG	No	56	1	0
0	0	Thoracotoı	112	CHG	No	57	0	1
0	0	Thoracotoı	112	CHG	No	66	1	1
0	0	Thoracotoı	112	CHG	No	57	0	1
0	0	VATS or rc	112	CHG	No	55	0	1
0	0	Thoracotoı	112	CHG	No	67	0	0
0	1	Thoracotoı	112	CHG	No	46	0	1
0	0	VATS or rc	112	CHG	No	76	0	0
0	0	Thoracotoı	112	CHG	No	49	0	1
0	0	Thoracotoı	112	CHG	No	81	0	0
0	0	Thoracotoı	112	CHG	No	53	1	1
0	0	Thoracotoı	112	CHG	No	57	0	1
0	1	Thoracotoı	112	CHG	No	67	1	0
0	0	Thoracotoı	112	CHG	No	57	0	1
0	0	Thoracotoı	112	CHG	No	66	0	0
0	0	Thoracotoı	112	CHG	No	69	0	1
0	0	Thoracotoı	112	CHG	No	45	0	1
0	0	Thoracotoı	112	CHG	No	72	1	0
0	1	Thoracotoı	112	CHG	No	58	0	0
0	0	Thoracotoı	112	CHG	No	81	0	0
0	0	Thoracotoı	112	CHG	No	41	0	0
0	0	VATS or rc	112	CHG	No	58	0	0
0	0	Thoracotoı	112	CHG	No	67	0	1
0	0	Thoracotoı	112	CHG	No	55	0	1
0	0	Thoracotoı	112	CHG	No	54	1	0
0	0	Thoracotoı	112	CHG	No	62	0	1
0	0	VATS or rc	112	CHG	No	63	1	1
0	0	VATS or rc	112	CHG	No	72	0	0
0	0	VATS or rc	112	CHG	No	59	0	0
0	0	Thoracotoı	112	CHG	No	74	0	0
0	0	Thoracotoı	112	CHG	No	80	0	1
0	1	Thoracotoı	112	CHG	No	68	0	1
0	0	Thoracotoı	112	CHG	No	78	1	0
0	0	Thoracotoı	112	CHG	No	60	0	1
0	0	Thoracotoı	112	CHG	No	68	1	0
0	0	Thoracotoı	41	PRIVE	No	67	0	0
0	0	Thoracotoı	41	PRIVE	No	61	0	0
0	0	Thoracotoı	41	PRIVE	No	71	0	0
0	0	Thoracotoı	41	PRIVE	No	64	0	0
0	0	Thoracotoı	41	PRIVE	No	49	0	0
0	0	Thoracotoı	41	PRIVE	No	78	1	0
0	0	Thoracotoı	41	PRIVE	No	78	0	0
0	0	Thoracotoı	41	PRIVE	No	79	0	0
0	0	VATS or rc	41	PRIVE	No	67	0	0
0	0	VATS or rc	41	PRIVE	No	71	0	0
0	0	Thoracotoı	41	PRIVE	No	80	0	0

0	0	Thoracotoı	41	PRIVE	No		63	0	0
0	0	Thoracotoı	41	PRIVE	No		52	0	1
0	0	Thoracotoı	41	PRIVE	No		60	0	0
0	1	Thoracotoı	41	PRIVE	No		59	0	0
0	0	VATS or rc	41	PRIVE	No		61	0	0
0	0	Thoracotoı	41	PRIVE	No		82	1	0
0	0	Thoracotoı	41	PRIVE	No		69	1	0
0	0	VATS or rc	41	PRIVE	No		63	0	0
0	0	Thoracotoı	41	PRIVE	No		65	0	0
0	0	Thoracotoı	41	PRIVE	No		69	0	0
0	0	Thoracotoı	41	PRIVE	No		65	0	0
0	0	Thoracotoı	41	PRIVE	No		63	1	0
0	0	Thoracotoı	41	PRIVE	No		65	0	0
0	0	Thoracotoı	41	PRIVE	No		77	0	0
0	0	VATS or rc	41	PRIVE	No		69	0	1
0	0	Thoracotoı	41	PRIVE	No		77	0	0
0	0	Thoracotoı	41	PRIVE	No		77	0	0
0	0	Thoracotoı	41	PRIVE	No		67	0	0
0	0	Thoracotoı	41	PRIVE	No		69	1	0
0	0	Thoracotoı	41	PRIVE	No		52	0	0
0	0	VATS or rc	41	PRIVE	No		77	0	0
0	0	Thoracotoı	41	PRIVE	No		59	0	0
0	0	VATS or rc	41	PRIVE	No		65	0	0
0	0	Thoracotoı	41	PRIVE	No		59	0	0
0	0	Thoracotoı	41	PRIVE	No		61	0	0
0	0	Thoracotoı	41	PRIVE	No		56	0	1
0	0	Thoracotoı	41	PRIVE	No		84	0	0
0	0	Thoracotoı	41	PRIVE	No		66	0	0
0	0	Thoracotoı	41	PRIVE	No		71	0	0
0	0	Thoracotoı	41	PRIVE	Yes		66	0	1
0	0	Thoracotoı	41	PRIVE	No		68	0	0
0	0	VATS or rc	41	PRIVE	No		77	0	0
0	0	Thoracotoı	41	PRIVE	No		63	0	0
0	0	VATS or rc	41	PRIVE	No		65	0	1
0	0	Thoracotoı	41	PRIVE	No		58	1	0
0	0	Thoracotoı	41	PRIVE	No		65	0	0
0	0	Thoracotoı	41	PRIVE	No		58	0	1
0	0	Thoracotoı	41	PRIVE	No		67	0	0
0	0	Thoracotoı	41	PRIVE	No		73	0	1
0	0	Thoracotoı	41	PRIVE	No		74	0	0
0	0	VATS or rc	41	PRIVE	No		61	0	0
0	0	VATS or rc	41	PRIVE	No		63	0	1
0	0	VATS or rc	41	PRIVE	No		75	0	0
0	0	Thoracotoı	41	PRIVE	No		78	0	0
0	0	Thoracotoı	41	PRIVE	No		76	1	0
0	0	VATS or rc	41	PRIVE	No		60	0	0
0	0	Thoracotoı	41	PRIVE	No		78	0	1
0	0	Thoracotoı	41	PRIVE	No		55	0	0
0	0	VATS or rc	41	PRIVE	No		74	0	0
0	0	Thoracotoı	41	PRIVE	No		68	0	0
0	0	Thoracotoı	41	PRIVE	No		77	0	0
0	0	VATS or rc	41	PRIVE	No		72	0	1
0	0	VATS or rc	41	PRIVE	No		67	0	0
0	0	Thoracotoı	41	PRIVE	No		74	0	0
0	0	Thoracotoı	41	PRIVE	No		60	0	1
0	0	Thoracotoı	41	PRIVE	No		64	1	1

0	0	Thoracotoı	41	PRIVE	No	59	0	0
1	0	VATS or rc	148	CHG	No	62	0	0
1	0	Thoracotoı	148	CHG	No	65	0	1
1	0	VATS or rc	148	CHG	No	55	0	0
1	1	Thoracotoı	148	CHG	No	56	0	1
1	0	Thoracotoı	148	CHG	No	63	0	0
1	0	Thoracotoı	148	CHG	No	67	0	0
1	0	Thoracotoı	148	CHG	No	74	0	0
1	0	VATS or rc	148	CHG	No	60	1	0
1	0	Thoracotoı	148	CHG	No	65	0	0
1	0	Thoracotoı	148	CHG	No	74	0	1
1	0	Thoracotoı	148	CHG	No	75	0	1
1	0	Thoracotoı	148	CHG	No	53	1	1
1	0	Thoracotoı	148	CHG	No	71	0	0
1	0	Thoracotoı	148	CHG	No	54	0	1
1	0	Thoracotoı	148	CHG	No	78	0	1
1	0	VATS or rc	148	CHG	No	46	0	0
1	0	Thoracotoı	148	CHG	No	61	0	0
1	0	VATS or rc	148	CHG	No	66	0	0
1	0	VATS or rc	148	CHG	No	55	0	0
1	0	VATS or rc	148	CHG	No	66	0	0
1	0	VATS or rc	148	CHG	No	73	0	0
1	0	Thoracotoı	148	CHG	No	73	0	0
1	0	Thoracotoı	148	CHG	No	68	0	0
1	1	Thoracotoı	148	CHG	No	68	0	0
1	0	VATS or rc	148	CHG	No	69	0	1
1	0	VATS or rc	148	CHG	No	65	0	0
1	0	VATS or rc	148	CHG	No	66	0	0
1	0	VATS or rc	148	CHG	No	67	0	0
1	0	VATS or rc	148	CHG	No	51	1	0
1	0	VATS or rc	148	CHG	No	61	1	0
1	0	Thoracotoı	148	CHG	No	49	0	1
1	1	Thoracotoı	148	CHG	No	61	0	1
1	0	VATS or rc	148	CHG	No	64	0	0
1	0	Thoracotoı	148	CHG	No	64	0	0
1	0	Thoracotoı	148	CHG	No	48	0	1
1	1	Thoracotoı	148	CHG	No	61	0	0
1	0	VATS or rc	148	CHG	No	53	0	0
1	0	VATS or rc	148	CHG	No	63	0	0
1	0	Thoracotoı	148	CHG	No	75	0	0
1	0	Thoracotoı	148	CHG	No	71	0	0
1	0	Thoracotoı	148	CHG	No	57	0	1
1	0	VATS or rc	148	CHG	No	74	0	0
1	0	VATS or rc	148	CHG	No	61	0	0
1	0	Thoracotoı	148	CHG	No	66	0	1
1	0	VATS or rc	148	CHG	No	59	0	0
1	0	VATS or rc	148	CHG	No	67	0	0
1	0	Thoracotoı	148	CHG	No	58	0	1
1	1	Thoracotoı	148	CHG	No	67	0	1
1	0	VATS or rc	148	CHG	No	71	0	0
1	0	VATS or rc	148	CHG	No	75	0	0
1	0	Thoracotoı	148	CHG	No	48	0	1
1	0	Thoracotoı	148	CHG	No	72	0	0
1	0	Thoracotoı	148	CHG	No	68	0	1
1	0	VATS or rc	148	CHG	No	58	0	0
1	0	Thoracotoı	148	CHG	No	48	0	1

1	0	Thoracotoı	148	CHG	No	76	0	0
1	0	Thoracotoı	148	CHG	No	63	1	1
1	0	VATS or rc	148	CHG	No	60	0	0
1	0	VATS or rc	148	CHG	No	65	0	1
1	1	Thoracotoı	148	CHG	No	44	0	1
1	0	Thoracotoı	148	CHG	No	54	0	1
1	0	VATS or rc	148	CHG	No	67	1	0
1	0	Thoracotoı	148	CHG	No	72	0	1
1	0	Thoracotoı	148	CHG	No	61	0	0
1	0	VATS or rc	148	CHG	No	58	1	0
1	0	Thoracotoı	148	CHG	No	61	0	0
1	0	VATS or rc	148	CHG	No	75	0	0
1	0	Thoracotoı	148	CHG	No	46	0	1
1	0	Thoracotoı	148	CHG	No	64	0	1
1	0	VATS or rc	148	CHG	No	57	0	0
1	0	Thoracotoı	148	CHG	No	57	0	1
1	0	VATS or rc	148	CHG	No	74	0	0
1	0	Thoracotoı	148	CHG	No	64	0	0
1	1	Thoracotoı	148	CHG	No	56	0	0
1	0	Thoracotoı	148	CHG	No	68	0	0
1	0	Thoracotoı	148	CHG	No	76	1	0
1	0	Thoracotoı	148	CHG	No	61	0	0
1	0	VATS or rc	148	CHG	No	59	0	0
1	0	Thoracotoı	148	CHG	No	62	0	0
1	1	Thoracotoı	148	CHG	No	74	0	0
1	0	Thoracotoı	148	CHG	No	54	0	0
1	0	VATS or rc	148	CHG	No	75	0	0
1	0	Thoracotoı	148	CHG	No	73	0	0
1	0	Thoracotoı	148	CHG	No	64	0	0
1	0	Thoracotoı	148	CHG	No	56	0	1
1	0	VATS or rc	148	CHG	No	56	0	0
1	0	Thoracotoı	148	CHG	No	58	0	1
1	0	VATS or rc	148	CHG	No	63	0	0
1	0	VATS or rc	148	CHG	No	52	0	0
1	0	Thoracotoı	148	CHG	No	73	0	0
1	0	Thoracotoı	148	CHG	No	54	0	1
1	0	Thoracotoı	148	CHG	No	64	0	1
1	0	Thoracotoı	148	CHG	No	54	0	1
1	0	Thoracotoı	148	CHG	No	51	0	0
1	0	Thoracotoı	148	CHG	No	72	0	0
1	0	Thoracotoı	148	CHG	No	75	0	1
1	0	VATS or rc	148	CHG	No	72	0	0
1	0	Thoracotoı	148	CHG	No	54	0	0
1	0	Thoracotoı	148	CHG	No	47	0	1
1	0	Thoracotoı	148	CHG	No	66	0	0
1	0	Thoracotoı	148	CHG	No	62	0	1
1	0	Thoracotoı	148	CHG	No	64	0	0
1	0	Thoracotoı	148	CHG	No	70	0	0
1	0	Thoracotoı	148	CHG	No	78	0	0
1	0	VATS or rc	148	CHG	No	66	0	1
1	0	VATS or rc	148	CHG	No	59	0	0
1	0	VATS or rc	148	CHG	No	64	0	0
1	0	Thoracotoı	148	CHG	No	67	0	0
1	0	Thoracotoı	148	CHG	No	66	0	1
1	0	VATS or rc	148	CHG	No	79	0	0
1	0	Thoracotoı	148	CHG	No	66	1	0

1	0	VATS or rc	148	CHG	No	62	1	0
1	0	VATS or rc	148	CHG	No	71	0	0
1	0	Thoracotoı	148	CHG	No	59	0	1
1	0	Thoracotoı	148	CHG	No	64	0	0
1	0	VATS or rc	148	CHG	No	60	0	0
1	0	Thoracotoı	148	CHG	No	62	0	0
1	0	Thoracotoı	148	CHG	No	74	1	0
1	0	Thoracotoı	148	CHG	No	62	0	1
1	0	Thoracotoı	148	CHG	No	58	1	0
1	0	VATS or rc	148	CHG	No	47	0	0
1	0	Thoracotoı	148	CHG	No	65	0	1
1	0	Thoracotoı	148	CHG	No	77	0	0
1	0	VATS or rc	148	CHG	No	74	0	0
1	0	Thoracotoı	148	CHG	No	59	0	0
1	0	Thoracotoı	148	CHG	No	74	0	1
1	0	VATS or rc	148	CHG	No	71	0	0
1	0	Thoracotoı	148	CHG	No	74	0	1
1	0	Thoracotoı	148	CHG	No	78	0	1
1	0	VATS or rc	148	CHG	No	66	1	0
1	0	Thoracotoı	148	CHG	No	65	0	0
1	0	Thoracotoı	148	CHG	No	77	0	0
1	0	VATS or rc	148	CHG	No	61	1	0
1	0	VATS or rc	148	CHG	No	69	0	0
1	0	Thoracotoı	148	CHG	No	58	0	0
1	0	VATS or rc	148	CHG	No	74	0	0
1	0	VATS or rc	148	CHG	No	79	0	0
1	0	Thoracotoı	148	CHG	No	57	0	1
1	0	VATS or rc	148	CHG	No	65	0	1
1	0	Thoracotoı	148	CHG	No	56	0	0
1	0	Thoracotoı	148	CHG	No	58	0	0
1	0	Thoracotoı	148	CHG	No	77	0	1
1	0	Thoracotoı	148	CHG	No	71	0	0
1	0	Thoracotoı	148	CHG	No	59	0	1
1	0	Thoracotoı	148	CHG	No	64	0	1
1	1	Thoracotoı	148	CHG	No	59	0	0
1	1	Thoracotoı	148	CHG	No	67	0	0
1	0	VATS or rc	148	CHG	No	78	0	0
1	0	VATS or rc	148	CHG	No	84	0	0
1	0	Thoracotoı	148	CHG	No	71	0	0
1	0	Thoracotoı	148	CHG	No	57	0	1
1	0	VATS or rc	148	CHG	No	71	0	0
1	0	VATS or rc	148	CHG	No	73	1	0
1	0	Thoracotoı	148	CHG	No	55	0	0
1	0	Thoracotoı	148	CHG	No	67	0	0
1	0	Thoracotoı	148	CHG	No	79	0	1
1	0	Thoracotoı	148	CHG	No	65	1	0
1	0	VATS or rc	148	CHG	No	76	1	0
1	0	Thoracotoı	148	CHG	No	67	0	0
1	0	Thoracotoı	148	CHG	No	58	1	0
1	0	VATS or rc	148	CHG	No	66	0	0
1	0	VATS or rc	148	CHG	No	59	0	0
1	0	Thoracotoı	148	CHG	No	49	0	1
1	0	VATS or rc	148	CHG	No	70	0	0
1	0	VATS or rc	148	CHG	No	84	0	0
1	0	Thoracotoı	148	CHG	No	46	0	0
1	0	VATS or rc	148	CHG	No	59	0	0

1	0	VATS or rc	148	CHG	No	64	0	0
1	0	Thoracotoı	148	CHG	No	68	0	0
1	0	Thoracotoı	148	CHG	No	67	1	0
1	0	VATS or rc	148	CHG	No	63	0	0
1	0	Thoracotoı	148	CHG	No	73	0	0
1	0	Thoracotoı	148	CHG	No	56	0	0
1	1	Thoracotoı	148	CHG	No	56	0	0
1	0	Thoracotoı	148	CHG	No	72	0	0
1	0	Thoracotoı	148	CHG	Yes	73	0	0
1	1	Thoracotoı	148	CHG	No	49	0	0
1	0	VATS or rc	148	CHG	No	53	0	0
1	0	VATS or rc	148	CHG	No	86	0	0
1	0	VATS or rc	148	CHG	No	64	0	0
1	0	Thoracotoı	148	CHG	No	69	1	0
1	0	Thoracotoı	148	CHG	No	64	0	1
1	0	Thoracotoı	148	CHG	No	68	0	1
1	1	Thoracotoı	148	CHG	No	68	0	0
1	0	VATS or rc	148	CHG	No	78	0	0
1	0	Thoracotoı	148	CHG	No	66	0	0
1	0	Thoracotoı	148	CHG	No	70	0	0
1	0	VATS or rc	148	CHG	No	73	0	0
1	0	VATS or rc	148	CHG	No	71	0	0
1	0	Thoracotoı	148	CHG	No	62	0	1
1	0	Thoracotoı	148	CHG	No	54	0	1
1	0	Thoracotoı	148	CHG	No	77	0	1
1	0	Thoracotoı	148	CHG	No	71	0	0
1	0	VATS or rc	148	CHG	No	74	0	0
1	0	VATS or rc	148	CHG	No	60	0	0
1	0	VATS or rc	148	CHG	No	57	0	0
1	0	Thoracotoı	148	CHG	No	51	0	0
1	0	VATS or rc	148	CHG	No	71	0	0
1	0	VATS or rc	148	CHG	No	77	0	0
1	0	VATS or rc	148	CHG	No	68	1	0
1	0	Thoracotoı	148	CHG	No	52	0	0
1	0	Thoracotoı	148	CHG	No	65	0	1
1	0	Thoracotoı	148	CHG	No	66	1	0
1	0	Thoracotoı	148	CHG	No	74	0	1
1	0	Thoracotoı	148	CHG	No	57	0	1
1	0	VATS or rc	148	CHG	No	67	0	0
1	0	Thoracotoı	148	CHG	No	64	0	0
1	0	Thoracotoı	148	CHG	No	52	0	0
1	0	Thoracotoı	148	CHG	No	72	0	1
1	0	VATS or rc	148	CHG	No	70	0	0
1	1	Thoracotoı	148	CHG	No	61	0	0
1	0	Thoracotoı	148	CHG	No	78	0	1
1	0	Thoracotoı	148	CHG	No	49	0	1
1	1	Thoracotoı	148	CHG	No	26	0	0
1	0	Thoracotoı	148	CHG	No	55	0	0
1	0	Thoracotoı	148	CHG	No	54	0	0
1	0	VATS or rc	148	CHG	No	65	0	1
1	1	Thoracotoı	148	CHG	No	57	0	0
1	0	Thoracotoı	148	CHG	No	66	0	1
1	0	Thoracotoı	148	CHG	No	72	0	0
1	0	Thoracotoı	148	CHG	No	57	0	1
1	0	Thoracotoı	148	CHG	No	50	0	0
1	0	Thoracotoı	148	CHG	No	57	0	1

1	0	VATS or rc	148	CHG	No	60	0	1
1	0	Thoracotoı	148	CHG	No	71	0	1
1	0	VATS or rc	148	CHG	No	79	1	0
1	0	Thoracotoı	148	CHG	No	41	0	0
1	0	Thoracotoı	148	CHG	No	60	0	0
1	0	Thoracotoı	148	CHG	No	56	0	1
1	0	Thoracotoı	148	CHG	No	50	0	1
1	0	Thoracotoı	148	CHG	No	54	0	1
1	0	Thoracotoı	148	CHG	No	65	0	0
1	0	VATS or rc	148	CHG	No	77	0	0
1	0	Thoracotoı	148	CHG	No	48	0	1
1	0	Thoracotoı	148	CHG	No	64	0	0
1	0	Thoracotoı	148	CHG	No	71	0	0
1	0	VATS or rc	148	CHG	No	73	0	0
1	0	VATS or rc	148	CHG	No	82	1	0
1	0	VATS or rc	148	CHG	No	66	0	0
1	0	Thoracotoı	148	CHG	No	60	0	1
1	0	VATS or rc	148	CHG	No	49	0	0
1	0	Thoracotoı	148	CHG	No	63	1	1
1	0	Thoracotoı	148	CHG	No	48	0	1
1	0	Thoracotoı	148	CHG	No	60	1	0
1	0	VATS or rc	148	CHG	No	78	0	0
1	0	VATS or rc	148	CHG	No	60	1	0
1	0	VATS or rc	148	CHG	No	57	0	0
1	0	Thoracotoı	148	CHG	No	59	0	0
1	0	Thoracotoı	148	CHG	No	52	0	1
1	0	VATS or rc	148	CHG	No	69	0	0
1	0	Thoracotoı	148	CHG	No	72	0	0
1	0	VATS or rc	148	CHG	No	78	0	0
1	0	Thoracotoı	148	CHG	No	66	0	1
1	0	Thoracotoı	148	CHG	No	65	1	1
1	0	VATS or rc	148	CHG	No	46	0	0
1	1	Thoracotoı	148	CHG	No	63	0	0
1	0	VATS or rc	148	CHG	No	83	0	0
1	0	VATS or rc	148	CHG	No	69	0	0
1	0	Thoracotoı	148	CHG	No	58	0	1
1	0	Thoracotoı	148	CHG	No	68	0	0
1	0	Thoracotoı	148	CHG	No	59	0	1
1	0	VATS or rc	148	CHG	No	56	0	0
1	0	Thoracotoı	148	CHG	No	67	0	0
1	0	VATS or rc	148	CHG	No	63	0	0
1	0	Thoracotoı	148	CHG	No	69	0	0
1	0	VATS or rc	148	CHG	No	55	0	1
1	0	VATS or rc	148	CHG	No	46	0	0
1	0	Thoracotoı	148	CHG	No	67	0	0
1	0	VATS or rc	148	CHG	No	76	0	0
1	0	Thoracotoı	148	CHG	No	77	0	1
1	0	Thoracotoı	148	CHG	No	57	0	1
1	0	VATS or rc	148	CHG	No	62	0	0
1	0	Thoracotoı	148	CHG	No	62	0	0
1	0	VATS or rc	148	CHG	No	63	0	1
1	0	VATS or rc	148	CHG	No	75	1	0
1	1	Thoracotoı	148	CHG	No	67	0	1
1	0	Thoracotoı	148	CHG	No	54	0	0
1	0	Thoracotoı	148	CHG	No	68	0	0
1	0	VATS or rc	148	CHG	No	55	0	0

1	0	VATS or rc	148	CHG	No	71	0	0
1	0	Thoracotoı	148	CHG	No	62	0	0
1	0	VATS or rc	148	CHG	No	78	0	0
1	0	VATS or rc	148	CHG	No	53	1	0
1	0	Thoracotoı	148	CHG	No	82	0	0
1	0	Thoracotoı	148	CHG	No	55	0	1
1	0	VATS or rc	148	CHG	No	75	0	0
1	0	VATS or rc	148	CHG	No	56	0	0
1	0	Thoracotoı	148	CHG	No	77	0	0
1	0	Thoracotoı	148	CHG	No	60	0	0
1	0	VATS or rc	148	CHG	No	75	0	0
1	0	Thoracotoı	148	CHG	No	65	0	0
1	0	Thoracotoı	148	CHG	No	56	0	1
1	0	Thoracotoı	148	CHG	No	61	0	0
1	0	VATS or rc	148	CHG	No	67	0	0
1	0	Thoracotoı	148	CHG	No	63	0	1
1	0	Thoracotoı	148	CHG	No	55	0	1
1	0	Thoracotoı	148	CHG	No	74	0	0
1	0	Thoracotoı	148	CHG	No	72	0	0
1	0	Thoracotoı	148	CHG	No	55	0	1
1	0	Thoracotoı	148	CHG	No	73	0	1
1	0	Thoracotoı	148	CHG	No	73	0	0
1	0	Thoracotoı	148	CHG	No	68	1	0
1	0	VATS or rc	148	CHG	No	71	0	0
1	0	Thoracotoı	148	CHG	No	53	0	0
1	1	Thoracotoı	148	CHG	No	69	0	0
1	0	Thoracotoı	148	CHG	No	68	0	0
1	0	VATS or rc	148	CHG	No	60	0	0
1	1	Thoracotoı	148	CHG	No	57	0	0
1	0	Thoracotoı	148	CHG	No	54	0	1
1	0	Thoracotoı	148	CHG	No	64	0	1
1	0	Thoracotoı	148	CHG	No	53	0	0
1	0	Thoracotoı	148	CHG	No	64	0	0
1	0	Thoracotoı	148	CHG	No	66	1	0
1	0	VATS or rc	148	CHG	No	67	0	1
1	0	Thoracotoı	148	CHG	No	74	1	0
1	0	Thoracotoı	148	CHG	No	77	0	0
1	0	VATS or rc	148	CHG	No	77	1	0
1	0	Thoracotoı	148	CHG	No	65	0	1
1	1	Thoracotoı	148	CHG	No	57	0	0
1	0	VATS or rc	148	CHG	No	62	0	0
1	0	Thoracotoı	148	CHG	No	49	0	0
1	0	Thoracotoı	148	CHG	No	61	0	0
1	0	VATS or rc	148	CHG	No	66	0	0
1	0	Thoracotoı	148	CHG	No	76	0	0
1	0	Thoracotoı	148	CHG	No	71	0	0
1	0	Thoracotoı	148	CHG	No	58	0	0
1	0	VATS or rc	148	CHG	No	63	0	0
1	0	VATS or rc	148	CHG	No	78	0	0
1	0	Thoracotoı	148	CHG	No	65	0	1
1	0	Thoracotoı	148	CHG	No	69	0	0
1	0	Thoracotoı	148	CHG	No	64	0	0
1	0	VATS or rc	148	CHG	No	59	0	0
1	0	Thoracotoı	148	CHG	No	79	1	0

chronic bro	arrhythmia	chronic he	peripheral	alcoholism	cirrhosis	stroke	diabetes	chronic ren
0	0	0	0	0	0	0	0	0
0	0	0	0	1	0	0	0	0
0	0	0	0	0	0	0	0	0
0	0	0	1	1	0	1	0	0
0	0	0	0	0	0	0	0	0
1	0	0	0	0	0	0	0	0
1	0	0	0	0	0	0	0	0
0	0	0	0	0	0	0	0	0
0	0	0	0	0	0	0	1	0
0	0	0	0	0	0	0	0	0
0	0	0	0	0	0	0	0	0
0	0	0	0	0	0	0	0	0
0	0	0	0	0	0	0	0	0
0	0	0	0	0	0	0	0	0
0	0	0	0	0	0	0	0	0
0	0	0	0	0	0	0	0	0
0	0	0	0	0	0	0	0	0
0	0	0	0	0	0	0	0	0
1	0	0	0	0	0	0	0	0
0	0	0	0	0	0	0	0	0
0	0	0	0	0	0	0	0	0
1	0	0	0	0	0	0	0	1
0	0	0	0	0	0	0	0	0
0	0	0	0	0	0	0	0	0
1	0	0	0	0	0	0	0	0
1	0	0	0	0	0	0	1	0
1	0	0	0	0	0	0	0	0
0	0	0	0	0	0	0	0	0
0	0	0	0	0	0	1	1	0
1	0	0	0	1	0	0	1	0
0	0	0	0	0	0	0	0	0
0	0	0	0	0	0	0	0	0
0	0	0	0	0	0	0	1	0
0	0	0	0	0	0	0	0	0
0	0	0	0	0	0	0	0	0
0	0	0	0	0	0	0	0	0
1	0	0	0	1	0	0	0	0
0	0	0	0	0	0	0	0	0
0	0	0	0	0	0	0	0	0
0	0	0	0	0	0	0	0	0
1	0	0	0	1	0	0	0	0
0	0	0	0	1	0	0	0	0
0	0	0	0	0	0	0	0	0
0	0	0	0	0	0	0	0	0
0	0	0	0	0	0	0	0	0
0	1	0	0	0	0	0	1	1
1	0	0	0	1	0	0	0	0
1	0	0	0	0	0	0	0	0
0	0	0	0	0	0	0	0	0
0	0	0	0	0	0	0	0	0
0	0	0	0	0	0	0	0	0
0	0	0	0	0	0	1	1	0
0	0	0	0	0	0	0	1	0
1	0	0	0	0	0	0	0	0
0	0	0	0	0	0	0	0	0

0	0	0	0	1	0	0	0	0
0	0	0	0	0	0	0	0	0
1	0	0	0	0	0	0	0	0
0	0	0	0	0	0	0	0	0
0	0	0	0	0	0	0	0	0
1	0	0	0	0	0	0	1	0
0	0	0	1	0	0	1	0	1
0	0	0	0	0	0	0	0	0
0	0	0	0	0	0	1	0	0
0	0	0	0	0	0	0	0	0
0	0	0	0	0	0	0	0	0
1	0	0	0	0	0	0	1	0
0	0	1	0	0	0	0	1	0
0	0	0	0	0	0	0	0	0
0	0	0	0	0	0	0	0	0
0	0	0	0	0	0	0	0	0
0	0	0	0	0	0	0	0	0
1	0	0	0	0	0	0	0	0
0	0	0	0	0	0	0	0	0
1	0	0	0	0	0	0	0	0
0	0	0	0	1	0	0	0	0
0	1	0	0	0	0	0	1	1
0	0	0	0	0	0	0	0	0
0	0	0	0	0	0	0	0	0
1	0	0	0	0	0	0	0	0
0	0	0	0	0	0	0	0	0
1	0	0	0	0	0	0	0	0
0	0	0	0	0	0	0	0	0
1	0	0	0	0	0	0	0	0
0	0	0	0	0	0	0	0	0
0	0	0	0	0	0	0	0	0
1	0	0	0	1	0	0	0	0
0	0	0	0	0	0	0	0	0
0	0	0	0	0	0	0	0	0
1	0	0	0	0	0	0	1	0
1	0	0	0	0	0	0	0	0
0	0	0	0	0	0	0	0	0
1	0	0	0	0	0	0	0	0
0	0	0	0	0	0	0	0	0
0	0	0	0	0	0	0	0	0
0	0	0	0	0	0	0	0	0
0	0	0	0	0	0	0	0	0
0	0	0	0	0	0	0	1	0
0	0	0	0	0	0	0	0	0
0	0	0	0	0	0	0	0	0
1	0	0	0	0	0	0	0	0
0	0	0	0	0	0	0	0	0
0	0	0	0	1	0	0	0	0
0	0	0	0	0	0	0	0	0
0	0	0	0	0	0	0	0	0
0	0	0	0	0	0	0	0	0
0	0	0	0	1	0	0	0	0
1	0	0	0	0	0	0	0	0
0	0	0	0	0	0	0	0	0
0	0	0	0	0	0	0	0	0
0	0	0	0	0	0	0	0	0

0	0	0	0	0	0	0	0	0
0	0	0	0	0	0	0	0	0
0	0	0	0	0	0	1	0	0
1	0	0	0	0	0	0	0	0
0	0	0	0	0	0	1	0	0
0	0	0	0	0	0	0	0	0
0	0	0	0	0	0	0	0	0
0	0	0	0	0	0	0	0	0
0	0	0	0	0	0	0	0	0
0	0	0	0	0	0	0	0	0
0	0	0	0	0	0	0	0	0
0	0	0	0	0	0	0	0	0
0	0	0	0	0	0	0	0	0
0	0	0	0	0	0	0	0	0
1	0	0	0	0	0	0	0	0
0	0	0	0	0	0	0	0	0
0	0	0	0	0	0	0	0	0
0	0	0	0	0	0	0	0	0
0	1	0	0	0	0	0	0	0
0	0	0	0	0	0	0	0	0
0	0	0	0	0	0	0	0	0
0	0	0	0	0	0	0	0	0
0	0	0	0	0	0	0	0	0
0	0	0	0	0	0	0	0	0
0	0	0	0	0	0	0	0	0
0	0	0	0	0	0	0	0	0
0	0	0	0	0	0	0	0	0
0	0	0	0	0	0	0	0	0
0	0	0	0	0	0	0	1	0
0	0	0	0	0	0	0	1	0
1	1	0	0	0	0	0	0	0
1	0	0	0	0	0	0	0	0
1	0	0	0	0	0	0	0	0
0	0	0	1	0	0	0	0	0
0	0	0	0	0	0	0	0	0
0	0	0	0	0	0	0	0	0
0	0	0	0	0	0	0	0	0
0	0	0	0	0	0	0	0	0
0	0	0	0	0	0	0	0	0
0	0	0	0	0	0	0	0	0
1	0	0	0	0	0	0	0	0
0	0	0	0	0	0	0	0	0
0	1	0	0	0	0	0	0	0
0	0	0	0	0	0	1	1	0
0	0	0	0	0	0	0	0	0
0	0	0	0	0	0	0	0	0
1	0	0	0	0	0	0	0	0
0	1	0	0	0	0	0	0	0
0	0	0	0	0	0	1	0	0
0	0	0	0	0	0	0	0	0
0	0	0	0	0	0	0	1	0
0	0	0	0	0	0	0	1	1
1	0	0	0	0	0	0	0	0
1	0	0	0	0	0	0	0	0
0	0	0	0	0	0	0	0	0
0	1	0	0	0	0	0	1	0

1	0	0	0	0	0	0	0	0
0	0	0	0	0	0	0	0	0
0	0	0	0	0	0	0	0	0
0	0	0	0	0	0	0	0	0
0	0	0	0	0	0	0	0	0
0	0	0	0	0	0	0	0	0
0	1	0	0	0	0	0	0	0
0	0	0	0	0	0	0	0	0
1	0	0	0	0	0	0	0	0
0	0	0	0	0	0	0	0	0
0	0	0	0	0	0	0	0	0
0	0	0	0	0	0	0	0	0
0	0	0	0	0	0	0	0	0
0	0	0	0	0	0	0	0	0
1	0	0	0	0	0	0	0	0
0	0	0	0	0	0	0	0	0
0	0	0	0	0	0	0	0	0
0	0	0	0	0	0	0	0	0
0	0	0	0	0	0	0	0	0
0	0	0	0	0	0	0	0	0
0	0	0	0	0	0	0	0	0
0	0	0	0	0	0	0	0	0
0	0	0	0	0	0	0	1	0
1	0	0	0	0	0	0	0	0
1	0	0	0	0	0	0	0	0
0	0	0	0	0	0	0	0	0
0	0	0	0	0	0	0	0	0
0	1	0	0	1	0	0	0	0
0	0	0	0	0	0	0	0	0
1	0	0	0	0	0	0	0	0
0	0	0	0	0	0	0	0	0
0	0	0	0	0	0	0	0	0
0	0	0	0	0	0	0	0	0
0	1	0	0	0	0	0	0	0
0	0	0	0	0	0	0	0	0
0	0	0	0	0	0	0	0	0
0	0	0	0	0	0	0	0	0
0	0	0	0	0	0	0	0	0
0	0	0	0	0	0	0	0	0
1	0	0	0	0	0	0	0	0
0	0	0	0	0	0	0	0	0
0	0	0	0	0	0	0	0	0
0	0	0	0	0	0	0	0	0
1	0	0	0	0	0	0	0	0
0	0	0	0	0	0	0	0	0
0	0	0	0	0	0	1	0	0
1	0	0	0	0	0	0	1	1
0	0	0	0	0	0	0	0	0
1	0	0	0	0	0	0	0	0
0	0	0	0	1	0	0	1	1
0	0	0	0	0	0	0	0	0
0	0	0	0	0	0	0	0	0
0	0	0	0	0	0	0	0	0
1	0	0	0	0	0	0	0	0
0	0	0	0	0	0	0	0	0
0	0	0	0	0	0	0	0	0

0	0	0	0	0	0	0	0	0
0	0	0	0	0	0	0	0	0
0	0	0	1	0	0	0	0	0
0	0	0	0	0	0	0	0	0
0	0	0	0	0	0	0	0	0
0	0	0	0	0	0	0	1	1
0	0	0	0	0	0	0	0	0
0	0	0	0	0	0	0	0	0
0	0	0	0	0	0	0	0	0
0	1	0	0	0	0	0	0	0
0	0	0	0	0	0	0	1	1
0	0	0	0	0	0	0	0	0
0	0	0	0	0	0	0	0	0
1	0	0	0	0	0	0	1	0
0	0	0	0	0	0	0	0	0
0	0	0	0	0	0	0	0	0
0	0	0	0	0	0	0	0	0
0	0	0	0	0	0	0	0	0
0	0	0	0	0	0	0	0	0
0	0	0	0	0	0	1	0	0
0	0	0	0	0	0	0	0	0
0	0	0	0	0	0	0	0	0
0	0	0	0	0	0	0	0	0
1	0	0	0	0	0	0	0	0
0	0	0	0	1	0	0	0	0
0	0	0	0	0	0	0	0	0
0	0	0	0	0	0	0	0	0
0	0	0	0	0	0	0	0	0
0	0	0	0	0	0	0	0	0
1	0	0	0	0	0	0	0	0
0	1	0	0	0	1	0	0	0
0	0	0	0	1	0	1	0	0
0	1	0	0	0	0	0	0	0
0	0	0	0	0	0	0	0	0
0	0	0	0	0	0	0	0	0
0	0	0	0	0	0	0	0	0
0	1	0	0	0	0	1	0	0
0	0	0	0	0	0	0	0	0
0	0	0	0	0	0	0	0	0
1	1	0	0	0	0	0	0	0
0	0	0	0	0	0	0	0	0
0	0	0	0	0	0	0	0	0
0	0	0	0	0	0	0	1	0
0	0	0	0	0	0	0	0	0
0	0	0	0	1	0	0	0	0
0	0	0	0	0	0	0	1	0
0	0	0	0	0	0	0	0	0
0	0	0	0	0	0	0	0	0
1	0	0	0	0	0	0	0	0
0	0	0	0	0	0	0	0	0
0	0	0	0	0	0	0	0	0
0	0	0	0	0	0	1	0	0
0	0	0	0	0	0	0	0	0
0	0	0	0	0	0	0	0	0
0	0	0	0	0	0	0	0	0
0	0	0	0	0	0	0	0	0

0	0	0	0	0	0	0	0	0
0	0	0	0	0	0	0	0	0
0	0	0	0	0	0	0	0	0
1	0	0	0	0	0	0	0	0
1	0	0	0	0	0	0	0	0
0	0	0	0	0	0	1	0	0
0	0	0	0	0	0	0	0	1
0	0	0	0	0	0	0	0	0
1	0	0	0	0	0	0	0	0
0	1	0	0	0	0	0	0	0
0	0	0	0	0	0	0	0	0
0	0	0	0	1	0	0	0	0
0	0	0	0	0	0	0	0	0
0	0	0	0	0	0	0	0	0
0	1	0	0	0	0	0	0	0
0	0	0	0	0	0	0	0	0
0	0	0	0	0	0	0	0	0
1	0	0	0	0	0	0	0	0
0	0	0	0	0	0	0	0	0
0	0	0	0	0	0	0	0	0
0	0	0	0	0	0	0	0	0
1	0	0	0	0	0	0	0	0
0	0	0	0	0	0	0	0	0
1	0	0	0	0	0	0	0	0
0	0	0	0	0	0	0	0	0
0	0	0	0	0	0	0	0	0
0	0	0	0	0	0	0	0	0
0	0	0	0	0	0	0	0	0
0	0	0	0	0	0	0	0	0
1	0	0	0	0	0	0	1	0
1	0	0	0	0	0	0	0	0
0	0	0	0	0	0	0	0	0
0	0	0	0	0	0	0	0	0
1	0	0	0	0	0	1	0	0
1	0	0	0	0	0	0	0	0
1	0	0	0	0	0	0	0	0
0	0	0	0	0	0	0	0	0
0	1	0	0	0	0	0	0	0
0	0	0	0	0	0	0	0	0
0	0	0	0	0	0	0	0	0
0	0	0	0	0	0	0	0	0
0	0	0	0	0	0	0	0	0
1	0	0	0	0	0	0	0	0
1	0	0	0	0	0	0	0	0
0	0	0	0	0	0	0	0	0
0	0	0	0	0	0	0	0	0
0	0	0	0	0	0	0	0	0
0	0	0	0	0	0	0	1	0
0	0	0	0	0	0	0	0	0
0	1	0	0	1	0	0	0	0
0	0	0	0	0	0	0	0	0
0	0	0	0	0	0	0	0	0
1	0	0	0	0	0	0	0	0
1	0	0	0	0	0	0	0	0
0	0	0	0	0	0	0	0	0
0	0	0	0	0	0	0	0	0

0	0	0	0	0	0	0	1	0
0	0	0	0	0	0	0	0	0
1	0	0	0	0	0	0	0	0
0	0	0	0	0	0	0	0	0
0	0	0	0	0	0	0	0	0
0	0	0	0	0	0	0	0	0
1	0	1	0	0	0	0	1	0
0	0	0	0	0	0	0	0	0
0	0	0	0	0	0	0	0	0
0	0	0	0	0	0	0	0	0
0	0	0	0	0	0	0	0	0
0	0	0	0	0	0	0	0	0
0	0	0	0	0	0	0	0	0
1	0	0	0	0	0	1	0	1
1	0	0	0	0	0	0	0	0
0	0	0	0	0	0	0	0	0
1	0	0	0	0	0	0	0	1
1	0	0	0	0	0	0	0	0
0	0	0	0	0	0	0	0	0
0	0	0	0	0	0	0	0	1
0	0	0	0	0	0	0	0	0
0	0	0	0	1	0	0	0	0
1	0	0	0	0	0	0	1	0
0	0	0	0	0	0	0	0	0
0	0	0	0	0	0	0	0	0
0	0	0	0	0	0	0	0	0
0	0	0	0	0	0	0	0	0
0	0	0	0	0	0	0	0	0
0	0	0	0	0	0	0	0	0
0	1	0	0	0	0	1	0	0
0	0	1	0	0	0	0	0	0
1	0	0	0	0	0	0	0	0
1	0	0	0	0	0	0	0	0
0	0	0	0	0	0	0	0	0
1	1	0	0	0	0	0	1	0
0	0	0	0	0	0	0	0	0
1	0	0	0	0	0	0	0	0
0	0	0	0	0	0	0	0	0
0	0	0	0	0	0	0	1	0
0	0	0	0	1	0	0	0	0
1	0	0	0	0	0	0	0	0
1	0	0	0	0	0	0	0	0
0	0	0	0	0	0	0	0	0
0	0	0	0	0	0	0	0	0
0	0	0	0	0	0	0	0	0
0	0	0	0	0	0	0	0	0
0	0	0	0	0	0	0	0	0
0	0	0	0	0	0	0	1	0
1	0	0	0	0	0	0	0	0
0	0	0	0	0	0	0	0	0
0	0	0	0	1	0	0	0	0
1	0	0	0	0	0	0	0	0
0	0	0	0	0	0	0	0	0
0	0	0	0	0	0	0	0	0
0	0	0	0	0	0	0	0	0
1	0	0	0	0	0	0	0	0

0	0	0	0	0	0	0	0	0
0	0	0	0	0	0	0	0	0
0	0	0	0	0	0	0	0	0
1	0	0	0	0	0	0	1	0
0	1	0	0	0	0	0	0	0
0	0	0	0	0	0	0	0	0
0	0	0	0	0	0	0	0	0
0	0	0	0	0	0	0	0	0
0	1	0	0	0	0	0	0	1
0	0	0	0	0	0	0	0	0
0	0	0	0	1	0	0	1	0
1	0	0	0	0	0	0	0	0
1	0	0	0	1	0	0	0	0
0	0	0	0	0	0	0	0	0
0	0	0	0	0	0	0	0	0
0	0	0	0	0	0	0	0	0
1	0	0	0	0	0	0	1	1
0	0	0	0	0	0	0	0	0
0	1	0	0	0	0	0	0	0
0	0	0	0	0	0	0	0	0
0	0	0	0	0	0	0	0	0
0	1	0	0	0	0	0	0	0
0	0	0	0	0	0	0	0	0
0	0	0	0	1	0	0	0	0
0	0	0	0	0	0	0	0	0
0	0	0	0	0	0	0	0	0
1	0	0	0	0	0	0	0	0
1	0	0	0	0	0	0	0	0
0	0	0	0	0	0	0	0	0
0	0	0	0	0	0	0	0	0
1	0	0	0	0	0	0	0	0
0	0	0	0	0	0	0	0	0
0	0	0	0	0	0	0	0	0
0	0	0	0	0	0	0	0	0
0	0	0	0	0	0	0	0	0
0	0	0	0	0	0	0	0	0
0	0	0	0	0	0	0	0	0
0	0	0	0	0	0	0	0	0
0	0	0	0	0	0	0	0	0
0	0	0	0	0	0	0	0	0
0	0	0	0	0	0	0	0	0
0	0	0	0	0	0	0	0	0
1	0	0	0	0	0	0	0	0
0	0	0	0	1	0	0	0	0
0	0	0	0	0	0	0	0	0
0	0	0	0	0	0	0	0	0
0	0	0	0	0	0	0	0	0
0	0	0	0	0	0	0	0	0
0	0	0	0	0	0	0	0	0
0	0	0	0	1	0	0	0	0
1	0	0	0	0	0	0	0	0
0	0	0	0	0	0	0	1	0
0	0	0	0	1	0	0	0	0
1	0	0	0	0	0	0	0	0
1	0	0	0	0	0	0	0	0
0	0	0	1	0	0	0	0	0

0	0	0	0	1	0	0	0	0
0	0	0	0	1	0	0	0	0
0	0	1	0	0	0	0	0	0
1	0	0	0	0	0	0	0	0
0	0	0	0	0	0	0	0	0
0	0	0	0	0	0	0	0	0
1	0	0	0	0	0	0	0	0
1	0	0	0	0	0	0	0	0
0	0	0	0	0	0	0	1	0
0	0	0	0	0	0	0	0	0
0	0	0	0	0	0	0	0	0
0	0	0	0	0	0	0	0	0
0	0	0	0	0	0	0	0	0
1	0	0	0	1	0	0	0	0
1	0	0	0	0	0	0	0	0
0	0	0	0	0	0	0	1	0
1	0	0	0	0	0	0	1	0
0	0	0	0	0	0	0	1	0
0	0	0	0	0	0	0	0	0
0	0	0	0	0	0	0	0	0
0	0	0	0	0	0	0	0	0
0	0	0	0	0	0	0	0	0
1	0	0	1	0	0	0	0	0
0	0	0	0	0	0	0	0	0
0	0	0	0	0	0	0	0	0
0	0	0	0	1	0	0	0	0
0	0	0	0	0	0	0	0	0
0	0	0	0	0	0	0	1	0
0	0	0	0	0	0	0	0	0
0	0	0	0	0	0	0	0	0
1	0	0	0	0	0	0	0	0
0	0	0	0	0	0	0	0	0
1	0	0	0	0	0	0	0	0
0	0	0	0	0	0	0	0	0
0	0	0	0	0	0	0	0	0
0	0	0	0	0	0	1	0	0
0	0	0	0	1	0	0	0	0
0	0	0	0	1	0	0	0	0
1	0	0	0	0	0	0	1	0
1	0	0	0	0	0	0	0	0
1	0	0	0	0	0	0	0	0
0	0	0	0	0	0	0	0	0
0	0	0	0	0	0	0	0	0
0	0	0	0	0	0	0	0	0
0	0	0	0	0	0	0	1	0
0	0	0	0	0	0	0	0	0
0	0	0	0	0	0	0	0	0
1	1	0	0	0	0	0	0	0
0	0	0	0	0	0	0	0	0
0	1	0	0	0	0	0	0	0
0	0	0	0	0	0	0	0	0
0	0	0	0	0	0	0	1	0
1	0	0	0	0	0	0	0	0
0	0	0	0	0	0	0	0	0
1	0	0	1	0	0	0	0	0
0	0	0	0	0	0	0	0	0

0	0	0	0	0	0	0	0	0
0	0	0	0	0	0	0	1	0
0	0	0	0	0	0	0	0	0
1	0	0	0	1	0	0	0	0
0	0	0	0	0	0	0	1	0
0	0	0	0	0	0	0	0	0
0	0	0	0	0	0	0	0	0
0	0	0	0	0	0	1	0	0
0	0	0	0	0	0	0	0	0
1	0	0	0	0	0	0	0	0
0	0	0	0	0	0	0	0	0
0	0	0	0	0	0	0	0	0
0	0	0	1	0	0	0	0	0
1	0	0	0	0	0	0	0	0
0	0	0	0	0	0	0	1	0
1	0	0	0	0	0	0	0	0
1	0	0	0	0	0	0	0	0
0	0	0	0	0	0	0	0	0
0	0	0	0	0	0	0	0	0
0	0	0	0	0	0	0	1	0
0	0	0	0	0	0	0	0	0
0	0	0	0	0	0	0	0	0
0	0	0	0	0	0	1	0	0
0	0	0	0	0	0	0	0	0
0	0	0	0	0	0	0	0	0
0	0	0	0	0	0	0	0	0
1	0	1	0	0	0	0	1	0
0	0	0	0	0	0	0	0	0
1	0	0	0	0	0	0	0	0
0	0	0	0	0	0	0	1	0
0	0	0	0	0	0	0	0	0
0	0	0	0	0	0	0	0	0
1	1	0	0	0	0	0	0	0
0	0	0	0	0	0	0	0	0
0	0	0	0	0	0	0	0	0
0	0	0	0	0	0	0	0	0
0	0	0	0	0	0	0	0	0
0	0	1	0	0	0	0	1	0
0	0	0	0	0	0	0	0	0
0	0	0	0	0	0	0	0	0
1	0	0	0	0	0	0	0	0
0	0	0	0	0	0	0	0	0
0	0	0	0	0	0	0	0	0
1	0	0	0	0	0	0	0	0
0	0	1	0	0	0	0	0	1
0	0	0	0	0	0	0	0	0
0	1	0	0	0	0	0	0	0
0	0	1	0	0	0	0	0	0
0	0	0	0	1	0	0	0	0
0	0	0	0	0	0	0	0	0
0	0	0	0	0	0	0	1	0
0	0	1	0	0	0	0	0	0
0	0	0	0	1	0	0	0	0
0	0	0	0	0	0	0	0	0
1	0	0	0	0	0	0	0	0
1	0	0	0	0	0	0	0	0

0	0	0	0	0	0	0	0	0
0	0	0	0	0	0	0	0	0
0	0	0	0	0	0	0	0	0
0	0	0	0	0	0	0	0	0
1	0	0	0	0	0	0	0	0
0	0	0	0	0	0	0	0	0
0	0	0	0	0	0	0	0	0
0	0	0	0	0	0	0	0	0
1	0	0	0	0	0	0	0	0
0	1	0	0	0	0	0	0	0
0	0	0	0	1	0	0	0	0
0	1	0	0	0	0	0	1	0
0	0	0	0	0	0	0	0	0
0	1	0	0	0	0	0	0	0
0	0	0	0	0	0	1	0	0
0	0	1	0	0	0	0	0	0
0	0	0	0	0	0	0	1	0
1	0	0	0	1	0	0	0	0
0	0	0	0	0	0	0	0	0
1	0	0	0	0	0	0	0	0
0	0	0	0	0	0	0	0	0
1	0	0	0	0	0	0	0	0
0	0	0	0	0	0	0	0	0
0	0	0	0	0	0	0	0	0
1	0	0	0	0	0	0	0	0
1	0	0	0	0	0	0	0	0
0	0	0	0	1	0	0	0	0
0	0	0	0	0	0	0	0	0
0	0	0	0	0	0	0	0	0
0	0	0	0	0	0	0	0	0
0	0	0	0	0	0	0	0	0
0	0	0	0	0	0	1	0	0
0	0	0	0	0	0	0	0	0
0	0	0	0	0	0	0	0	0
0	0	0	0	0	0	0	0	0
0	0	0	0	0	0	0	0	0
0	0	0	0	0	0	0	1	0
0	0	0	0	0	0	0	0	0
1	0	0	0	0	0	0	0	0
0	0	0	0	0	0	0	0	0
0	0	0	0	0	0	0	0	1
0	0	0	0	0	0	0	0	0
1	0	0	0	0	0	0	0	0
0	0	0	0	0	0	0	0	0
1	0	0	0	0	0	0	0	0
0	0	0	0	0	0	0	0	0
0	0	0	0	0	0	0	0	0
0	0	0	0	0	0	0	0	0
0	0	0	0	0	0	0	1	0
0	0	0	0	0	0	0	0	0
0	0	0	0	0	0	0	0	0
0	0	0	0	0	0	0	0	0
0	0	0	0	0	0	0	0	0
0	0	0	0	0	0	0	0	0
0	0	0	0	0	0	0	0	0
0	0	0	0	0	0	0	0	0

0	0	0	0	0	0	0	0	0
0	0	0	0	0	0	0	0	0
0	0	0	0	0	0	0	0	0
0	1	0	0	0	0	0	0	0
0	0	0	0	0	0	0	0	0
0	1	0	0	0	0	0	0	0
0	0	0	0	0	0	0	0	0
0	0	0	0	0	0	0	0	0
0	0	0	0	0	0	0	0	0
0	0	1	0	0	0	0	0	0
0	0	0	0	0	0	0	0	0
0	0	0	0	0	0	0	0	0
0	0	0	0	0	0	0	0	0
0	0	0	0	0	0	0	0	0
0	0	0	0	0	0	0	0	0
0	0	0	0	0	0	0	0	0
0	0	0	0	0	0	0	0	0
0	0	0	0	0	0	0	0	0
1	0	0	0	0	0	0	0	0
0	1	0	0	0	0	0	0	0
0	0	0	0	0	0	0	0	0
0	0	0	0	0	0	0	0	0
0	0	0	0	0	0	0	0	0
0	0	0	0	0	0	0	1	0
0	0	0	0	0	0	0	0	0
1	0	0	0	0	0	0	0	0
0	0	0	0	0	0	0	0	0
1	0	0	0	0	0	0	0	0
0	0	0	0	0	0	0	0	0
0	0	0	0	0	0	0	0	0
0	1	0	0	0	0	0	0	0
0	0	0	0	0	0	0	0	0
0	0	1	0	0	0	0	1	0
0	0	0	0	0	0	0	0	0
0	0	0	0	0	0	0	0	0
0	0	0	0	0	0	0	0	0
0	0	0	0	0	0	0	1	0
1	0	0	0	0	0	0	0	0
0	1	0	0	0	0	0	0	0
0	0	0	0	0	0	0	0	0
0	0	0	0	0	0	0	0	0
0	0	0	0	0	0	0	0	0
0	0	0	0	0	0	0	0	0
0	0	0	0	0	1	0	0	0
0	0	0	0	0	0	0	0	0
0	0	0	0	0	0	0	0	0
0	0	0	0	0	0	0	0	0
0	0	0	0	0	0	0	0	0
0	0	0	0	0	0	0	0	0
1	0	0	0	0	0	0	0	0
0	1	1	0	0	0	0	0	0
0	0	0	0	0	0	0	0	0
0	0	0	0	0	0	0	0	0
1	0	0	0	0	0	0	1	0
0	0	0	0	0	0	0	0	0
0	1	0	0	0	0	0	0	0

0	0	0	0	0	0	0	0	0
0	0	0	0	0	0	0	0	0
1	0	0	0	0	0	0	1	0
0	0	0	0	0	0	0	0	0
0	0	0	0	0	0	0	1	0
0	0	0	0	0	0	0	0	0
0	0	0	0	0	0	0	0	0
0	0	0	0	0	0	0	1	0
1	0	0	0	0	0	0	0	0
0	0	0	0	0	0	0	0	0
0	0	0	0	0	0	0	1	0
0	0	0	0	0	0	0	0	0
0	0	0	0	0	0	0	0	0
0	0	0	0	0	0	0	0	0
0	0	0	0	0	0	0	0	0
0	0	0	0	0	0	0	0	0
0	0	0	0	0	0	0	0	0
0	0	0	0	0	0	0	0	0
0	0	0	0	0	0	0	0	0
0	0	0	0	0	0	0	0	0
1	0	0	0	0	0	0	0	0
1	0	0	0	0	0	0	0	0
0	0	0	0	0	0	0	0	0
0	0	0	0	0	0	0	0	0
0	0	0	0	0	0	0	0	0
0	0	0	0	0	0	0	1	0
0	0	0	0	0	0	0	1	0
1	0	0	0	0	0	0	1	0
0	0	0	0	0	0	0	0	0
1	0	0	0	0	0	0	0	0
0	0	0	0	0	0	0	0	0
0	0	0	0	0	0	0	0	0
0	1	0	0	0	0	0	0	0
0	0	0	0	0	0	0	0	0
0	0	0	0	0	0	0	0	0
0	0	0	0	0	0	0	0	0
0	0	0	0	0	0	0	1	0
0	0	0	0	0	0	0	0	0
0	0	0	0	0	0	0	0	0
1	0	0	0	0	0	0	0	0
0	0	0	0	0	0	0	0	0
0	0	0	0	0	0	0	0	0
0	0	0	0	0	0	0	0	0
0	1	0	0	0	0	0	0	0
1	0	0	0	0	0	0	0	0
1	0	0	0	0	0	0	0	0
0	0	0	0	0	0	0	0	0
0	0	0	0	0	0	0	1	0
1	0	0	0	0	0	0	0	0
0	0	0	0	0	0	0	0	0
0	0	0	0	0	0	0	0	0
0	0	0	0	0	0	0	0	0
0	0	0	0	0	0	0	0	0
0	0	0	0	0	0	0	0	0
0	0	0	0	0	0	0	0	0
0	0	0	0	0	0	0	0	0

0	0	0	0	0	0	0	1	0
0	0	0	0	0	0	0	0	0
0	0	0	0	0	0	0	0	0
0	0	0	0	0	0	0	0	0
0	0	0	0	0	0	0	0	0
0	1	0	0	0	0	0	0	0
0	0	0	0	0	0	0	0	0
1	0	0	0	0	0	0	0	0
0	0	0	0	0	0	0	0	0
0	0	0	0	0	0	0	0	0
1	0	0	0	0	0	0	0	0
0	0	0	0	0	0	0	0	0
0	0	0	0	0	0	0	0	0
0	0	0	0	0	0	0	0	0
0	0	0	0	0	0	0	0	0
0	0	0	0	0	0	0	0	0
1	0	0	0	0	0	0	1	0
0	0	0	0	0	0	0	0	0
0	0	0	0	0	0	0	0	0
0	0	0	0	0	0	0	0	0
0	0	0	0	0	0	0	0	0
0	0	0	0	0	0	0	1	0
0	0	0	0	0	0	0	0	1
0	1	1	0	0	0	0	0	0
0	0	0	0	0	0	0	0	0
0	0	0	0	0	0	0	1	0
1	0	0	0	0	0	0	0	0
0	0	0	0	0	0	0	0	0
0	0	0	0	1	0	0	0	0
0	0	0	0	0	0	0	0	0
0	0	0	0	0	0	0	0	0
0	0	0	0	0	0	0	0	0
0	0	0	0	0	0	0	0	0
0	0	0	0	0	0	0	0	0
1	0	0	0	0	0	0	0	0
1	0	0	0	0	0	0	0	0
0	0	0	0	0	0	0	0	0
0	0	0	0	0	0	0	0	0
1	0	0	0	0	0	0	0	0
0	0	0	0	0	0	0	0	0
1	0	0	0	0	0	0	0	0
0	0	0	0	0	0	0	0	0
0	0	0	0	0	0	0	0	0
0	0	0	0	0	0	0	0	0
0	0	0	0	0	0	0	0	0
0	0	0	0	0	0	0	0	0
0	0	0	0	0	0	0	1	0
1	0	0	0	0	0	0	0	0
0	1	0	0	0	0	0	0	0
0	0	0	0	0	0	0	0	0
0	0	0	0	0	0	0	0	0
0	0	0	0	0	0	0	0	0
0	0	0	0	0	0	0	0	0
1	0	0	0	0	0	0	1	0
0	0	0	0	0	0	0	0	0
0	0	0	0	0	0	0	0	0

0	0	0	0	0	0	0	0	0
0	0	0	0	0	0	0	1	0
0	0	0	0	0	0	0	1	0
0	0	0	0	0	0	0	0	0
1	0	0	0	0	0	0	0	0
0	0	0	0	0	0	0	0	0
0	0	0	0	0	0	0	0	0
0	0	0	0	0	0	0	0	0
0	0	0	0	0	0	0	0	0
0	0	0	0	1	0	0	1	0
0	0	0	0	0	0	0	1	0
0	0	0	0	0	0	0	0	0
0	0	0	0	0	0	0	0	0
0	0	0	0	0	0	0	1	0
1	0	0	0	0	0	0	0	0
0	0	0	0	0	0	0	0	0
0	0	0	0	0	0	0	0	0
0	0	0	0	0	0	0	0	0
1	0	0	0	0	0	0	1	0
0	0	0	0	0	0	0	0	0
0	0	0	0	0	0	0	0	0
1	0	0	0	0	0	0	0	0
0	0	0	0	0	0	0	0	0
0	0	0	0	0	0	0	1	0
0	0	0	0	0	0	0	0	0
0	0	0	0	0	0	0	0	0
0	1	0	0	0	0	0	0	0
0	0	0	0	0	0	0	0	0
0	0	0	0	0	1	0	1	0
0	0	0	0	0	0	0	0	0
0	0	0	0	0	0	0	0	0
0	0	0	0	1	0	0	0	0
0	0	0	0	0	0	0	1	0
0	0	0	0	0	0	0	0	0
0	0	0	0	0	0	0	0	0
0	0	0	0	0	0	0	0	0
0	0	1	0	0	0	0	1	0
0	0	0	0	0	0	0	0	0
0	0	0	0	0	0	0	0	0
0	0	0	0	0	0	0	0	0
0	0	0	0	0	0	0	0	0
0	0	0	0	0	0	0	0	0
0	0	0	0	0	0	0	0	0
1	0	0	0	0	0	0	0	0
0	0	0	0	0	0	0	0	0
0	0	0	0	0	0	0	0	0
0	0	0	0	0	0	0	0	0
1	0	0	0	0	0	0	0	0
1	0	0	0	0	0	0	0	0
0	0	0	0	0	0	0	0	0
0	0	0	0	0	0	0	0	0
0	0	0	0	0	0	0	0	0
0	0	0	0	0	0	0	0	0
0	0	0	0	0	0	0	0	0
0	0	0	0	0	0	0	0	0
0	0	0	0	0	0	0	0	0

0	0	0	0	0	0	0	0	0
0	0	0	0	0	0	0	0	0
0	1	0	0	0	0	0	0	0
0	0	0	0	0	0	0	0	0
0	0	0	0	0	0	0	0	0
0	0	0	0	0	0	0	0	0
0	0	0	0	0	0	0	0	0
0	0	0	0	0	0	0	0	0
0	0	0	0	0	0	0	0	0
0	0	0	0	0	0	0	0	0
1	0	0	0	0	0	0	0	0
0	0	0	0	0	0	0	0	0
0	1	0	0	0	0	0	1	0
0	0	0	0	0	0	0	0	0
1	0	0	0	0	0	0	0	0
0	0	0	0	0	0	0	1	0
0	0	0	0	0	0	0	0	0
0	0	0	0	0	0	0	0	0
0	0	0	0	0	0	0	0	0
0	0	0	0	0	0	0	0	0
0	0	0	0	0	0	0	0	0
0	0	0	0	0	0	0	0	0
0	0	0	0	0	0	0	1	0
0	0	0	0	0	0	0	0	0
0	0	0	0	0	0	0	0	0
0	0	0	0	0	0	0	0	0
0	0	0	0	0	0	0	1	0
0	1	0	0	0	0	0	0	0
0	0	0	0	0	0	0	0	0
0	0	0	0	0	0	0	0	0
0	0	0	0	0	0	0	0	0
0	0	0	0	0	0	0	0	0
0	0	0	0	0	0	0	0	0
0	0	0	0	0	0	0	1	0
0	0	0	0	0	0	0	0	0
0	0	0	0	0	0	0	0	0
0	0	0	0	0	0	0	0	0
0	0	0	0	1	0	0	0	0
0	0	0	0	0	0	0	0	0
0	0	0	0	0	0	0	0	0
0	0	0	0	0	0	0	0	0
1	1	0	0	0	0	0	0	0
1	0	0	0	0	0	0	0	0
1	0	0	0	0	0	0	0	0
1	0	0	0	0	0	0	0	0
0	0	0	0	0	0	0	1	0
0	0	0	0	0	0	0	0	0
0	0	0	0	0	0	0	0	0
0	0	0	0	0	0	0	0	0
0	0	0	0	0	0	0	0	0
0	0	0	0	0	0	0	1	0
0	1	0	0	0	0	0	1	0
1	0	0	0	0	0	0	0	0
1	0	0	0	0	0	0	0	0
0	0	0	0	0	0	0	0	1
1	0	0	0	1	0	0	0	0

0	0	0	0	0	0	0	1	0
1	0	0	0	1	1	0	0	0
0	0	0	0	0	0	0	0	0
0	0	0	0	0	0	0	0	0
0	1	0	0	0	0	0	1	0
0	0	0	0	0	0	0	0	0
0	0	0	0	0	0	0	0	0
0	0	0	0	0	0	0	0	0
0	0	0	0	0	0	0	1	0
0	0	0	0	0	0	0	0	0
0	0	0	0	0	0	0	0	0
0	0	0	0	0	0	0	0	0
0	0	0	0	0	0	0	0	0
0	0	0	0	0	0	0	0	0
0	0	0	0	0	0	0	1	1
0	0	1	0	0	0	0	0	0
0	0	0	0	0	0	0	0	0
1	0	0	0	0	0	0	0	0
1	0	0	0	0	0	1	0	0
0	1	0	0	0	0	0	0	0
1	0	0	0	0	0	0	0	0
0	0	0	0	1	0	0	0	0
0	1	0	0	0	0	1	0	0
0	0	0	0	0	0	0	0	0
0	0	0	0	0	0	0	1	0
1	1	0	0	0	0	0	0	0
0	1	0	0	0	0	0	0	0
0	1	0	0	1	0	0	1	0
0	0	0	0	0	0	0	0	0
1	0	0	0	0	0	0	0	0
0	0	0	0	0	0	0	0	0
0	1	0	0	0	0	0	0	0
1	0	0	0	0	0	0	0	0
0	0	0	0	0	0	0	0	0
0	1	0	0	0	0	0	0	0
0	0	0	0	0	0	0	0	0
0	0	0	0	0	0	0	1	1
0	0	0	0	0	0	0	0	0
0	1	0	0	0	0	0	0	0
1	0	0	0	0	0	0	0	0
0	0	0	0	1	0	0	1	0
0	0	0	0	1	0	0	0	1
1	0	0	0	0	0	0	0	0
0	0	0	0	0	0	0	0	0
0	0	0	0	0	0	0	0	0
0	0	0	0	0	0	0	0	0
1	0	0	0	0	0	0	1	0
1	0	0	0	0	0	0	0	0
0	0	0	0	0	0	0	0	0
0	0	0	0	0	0	0	0	0
1	0	0	0	0	0	0	0	0
1	0	0	0	0	0	0	0	0
0	1	1	1	0	0	1	0	0
1	0	0	0	1	0	0	0	0
0	0	0	0	0	0	0	0	0
0	0	0	0	0	0	0	0	0

0	0	0	1	0	0	0	0	0
0	0	0	0	0	0	0	1	0
0	0	0	0	0	0	0	0	0
0	0	0	0	0	0	0	0	0
0	0	0	0	0	0	0	0	0
0	0	0	0	0	0	0	0	0
1	0	0	0	0	0	0	0	0
1	0	0	0	1	0	0	0	0
0	0	0	0	0	0	0	0	1
0	1	0	0	0	0	0	0	0
0	0	0	0	0	0	0	0	0
0	0	0	0	0	0	0	0	0
0	0	0	0	0	0	0	0	0
1	0	0	0	0	0	0	1	0
1	0	0	0	0	0	0	0	0
0	0	0	0	0	0	0	0	0
0	0	0	0	1	0	0	0	0
0	0	0	0	0	0	0	0	0
1	1	0	0	0	0	0	1	0
1	0	0	0	0	0	0	1	1
0	0	0	0	0	0	0	0	0
0	0	0	0	0	0	0	0	0
1	0	0	0	0	0	0	0	0
1	0	0	0	0	0	0	0	0
0	0	0	0	0	0	0	0	0
0	1	0	0	0	0	0	0	1
0	0	1	0	0	0	0	1	0
1	0	0	0	0	0	0	1	0
0	0	0	0	0	0	0	1	0
1	0	0	0	0	0	0	0	0
1	0	0	0	0	0	0	0	0
0	0	0	0	0	0	0	1	0
0	0	0	0	0	0	0	0	0
1	0	0	0	0	0	0	0	0
0	0	0	0	0	0	0	0	0
0	0	0	0	1	1	1	0	0
0	1	0	0	1	0	0	0	0
0	0	0	0	0	0	0	0	0
0	0	0	0	0	0	0	1	1
0	1	0	0	0	0	0	0	0
1	0	0	0	0	0	0	1	0
1	0	0	0	1	0	0	0	0
0	0	0	1	0	0	0	0	0
0	0	0	0	0	0	0	0	0
0	0	0	1	0	0	0	0	0
0	0	0	0	0	0	0	0	0
0	0	0	0	0	0	0	0	0
0	0	0	0	0	0	0	0	0
0	0	0	0	0	0	1	0	0
1	1	0	0	0	0	1	1	0
0	0	0	0	0	0	0	0	0
0	0	0	0	0	0	0	1	0
0	0	0	0	0	0	0	0	0
0	0	0	0	0	0	0	0	0
0	0	0	0	0	0	0	0	0
1	0	0	0	0	0	0	1	0

0	0	0	0	0	0	0	0	0
0	0	0	0	0	0	0	0	0
1	0	0	0	0	0	0	0	0
0	0	0	0	0	0	0	0	0
0	0	0	0	0	0	0	0	0
1	0	0	0	1	0	0	0	0
1	0	0	0	0	0	1	0	0
0	0	0	0	0	0	0	1	0
1	0	0	0	0	0	0	0	0
0	0	0	0	0	0	0	0	0
0	0	0	0	0	0	0	0	0
0	0	0	0	1	0	0	0	0
0	0	0	0	0	0	0	1	0
0	0	0	0	0	0	0	0	0
0	0	0	0	0	0	0	0	0
0	0	0	0	0	0	0	0	0
0	0	0	0	0	0	0	0	0
0	0	0	0	0	0	0	0	0
0	0	0	0	0	0	0	0	0
1	0	0	0	0	0	0	0	0
1	0	0	0	0	0	0	0	0
0	1	0	0	0	0	1	0	0
1	0	0	0	0	0	0	0	0
0	0	0	0	0	0	0	0	0
1	0	0	0	0	0	0	0	0
0	0	0	0	0	0	0	1	0
0	0	0	0	0	0	0	0	0
0	1	0	0	0	0	1	0	0
0	0	0	0	0	0	0	0	0
0	0	0	0	0	0	0	0	0
0	0	0	0	0	0	0	0	0
1	0	0	0	0	0	0	0	0
0	0	0	0	0	0	0	0	0
0	0	0	0	0	0	0	0	0
0	0	0	0	0	0	0	0	0
0	0	0	0	0	0	0	1	0
1	0	0	0	0	0	0	0	0
0	0	0	0	0	0	0	0	0
0	0	0	0	0	0	0	0	0
0	0	0	0	0	0	0	0	0
0	0	0	0	0	0	1	0	0
0	0	0	0	0	0	0	0	1
0	0	0	0	0	0	0	0	0
1	0	0	0	0	0	0	0	0
1	0	0	0	0	0	0	0	0
0	0	0	0	0	0	0	0	0
0	0	0	0	0	0	0	0	0
1	0	0	0	0	0	0	0	0
1	0	0	0	0	0	0	0	0
0	0	0	0	0	0	0	0	0
0	0	0	0	0	0	0	0	0
1	0	0	0	0	0	0	0	0
0	0	0	0	0	0	0	0	0
0	0	0	0	0	0	0	0	0
0	0	0	0	0	0	0	0	0
0	0	0	0	0	0	1	0	0

0	0	0	0	0	0	0	1	0
0	1	0	0	0	0	0	0	0
1	0	0	0	0	0	0	0	0
0	0	0	0	0	0	0	1	0
0	0	0	0	0	0	0	0	0
0	0	0	0	0	0	0	0	0
0	0	0	0	0	0	0	0	0
0	0	0	0	0	0	0	0	0
0	0	0	0	1	0	0	0	0
1	0	0	0	0	0	0	0	0
0	0	0	0	0	0	0	0	0
0	0	0	0	0	0	0	1	0
0	1	0	0	0	0	0	0	0
0	0	0	0	0	0	0	0	0
1	0	0	0	0	0	0	0	0
0	1	0	0	0	0	0	0	0
1	0	0	1	0	0	0	0	0
0	0	0	0	0	0	0	0	1
1	0	0	0	0	0	0	0	0
1	0	0	0	0	0	0	0	0
0	0	0	0	0	0	0	0	0
0	0	0	0	0	0	0	0	0
1	0	0	0	0	0	0	0	0
1	0	0	0	0	0	0	0	0
1	0	0	0	0	0	0	0	0
0	0	0	0	0	0	0	0	0
0	0	0	0	0	0	0	0	0
0	0	0	0	0	1	0	0	1
1	0	0	0	0	0	0	0	0
0	0	0	0	0	0	0	0	0
1	0	0	0	0	0	0	0	0
1	0	1	0	0	0	0	0	0
1	0	1	0	0	0	0	0	0
0	0	0	0	0	0	0	1	0
1	0	0	0	0	0	1	0	0
1	0	0	0	0	0	0	0	0
0	0	0	0	0	0	1	0	0
1	0	0	0	0	0	1	0	0
1	1	0	0	0	0	0	0	1
1	0	0	0	0	0	0	0	0
0	0	0	0	1	1	0	1	0
0	0	0	0	0	0	0	1	0
1	0	0	0	0	0	1	0	0
1	0	0	0	0	0	0	0	0
1	0	0	0	0	0	0	0	0
0	0	0	0	0	0	0	1	0
1	0	0	0	0	0	0	0	0
0	0	0	0	0	0	0	0	0
1	0	0	0	0	0	0	0	0
1	1	0	0	0	0	1	0	0
1	0	0	0	0	0	0	0	0
1	0	0	0	0	0	0	0	0
1	1	0	0	0	0	0	0	0
1	0	0	0	0	0	0	0	0
1	0	1	0	0	0	0	0	0
1	0	0	0	0	0	0	0	0

1	0	0	0	0	0	0	0	0
0	1	0	0	0	0	0	0	1
1	0	0	0	0	0	0	0	0
0	0	0	0	0	0	0	0	0
0	0	0	0	0	0	0	0	0
1	0	0	1	0	0	0	0	0
1	0	0	0	0	0	0	0	0
0	0	0	0	0	0	0	0	0
1	1	0	0	0	0	0	0	0
0	0	0	0	0	0	0	0	0
0	0	0	0	0	0	0	1	0
1	0	0	0	0	0	0	0	0
1	1	0	0	0	0	0	0	0
1	0	0	0	0	0	0	0	0
0	0	0	0	0	0	0	0	0
0	1	1	0	0	0	0	0	0
0	0	0	0	0	0	0	1	0
0	1	0	0	0	0	0	0	0
1	0	0	0	0	0	0	0	0
1	0	0	0	0	0	0	0	0
0	0	0	0	0	0	0	0	0
1	0	0	0	0	0	0	0	0
1	0	0	0	0	0	0	0	0
0	0	0	0	0	0	0	0	0
1	0	0	0	0	0	0	0	0
1	0	0	0	0	0	0	1	0
0	0	0	0	0	0	0	0	0
0	0	0	0	0	0	0	0	0
1	0	0	0	1	0	0	0	0
0	0	0	0	0	0	0	0	0
1	1	0	0	0	0	0	0	0
1	0	0	0	1	0	0	0	0
0	0	0	0	0	0	0	0	0
0	0	0	0	0	0	0	0	0
1	1	0	0	0	0	0	0	0
0	0	0	0	0	0	1	1	0
0	0	0	0	0	0	0	0	0
1	0	0	0	0	0	1	0	0
1	0	0	0	0	0	0	1	0
1	0	0	0	0	0	0	1	0
0	0	0	0	0	0	0	1	0
0	0	0	0	0	0	0	0	0
1	0	0	0	0	0	0	0	0
1	0	0	0	0	0	0	0	0
1	0	0	0	1	0	1	1	0
1	0	0	0	0	0	0	0	0
1	0	0	0	0	0	0	0	0
0	1	1	0	0	0	0	0	0
0	0	0	0	0	0	0	0	0
0	0	0	0	0	0	0	0	0
0	0	0	0	0	0	0	0	0
0	0	0	0	0	0	0	0	0
1	0	0	0	0	0	0	0	0
1	0	0	0	0	0	0	0	1
0	0	0	0	0	0	0	1	0
0	0	0	0	0	0	0	0	0

0	0	0	0	0	0	0	1	0
0	0	0	0	0	0	0	1	0
0	1	0	0	0	0	0	0	0
0	0	0	0	0	0	0	0	0
1	0	0	0	0	0	0	0	0
0	0	0	0	0	1	0	1	0
0	0	0	0	0	0	0	0	0
0	0	0	0	0	0	0	0	0
0	0	0	0	0	0	0	0	0
0	0	0	0	0	0	0	0	0
1	0	0	0	0	0	0	0	0
0	0	0	0	0	0	0	0	0
1	0	0	0	0	0	0	0	0
1	0	1	0	0	0	0	0	0
0	0	0	0	0	0	0	0	0
0	0	0	0	0	0	0	1	0
1	1	0	0	0	0	0	0	0
1	0	0	0	0	0	0	0	0
1	0	0	0	0	0	0	1	0
0	0	0	0	0	0	0	1	0
1	0	0	0	0	0	0	0	0
0	0	0	0	0	0	0	0	0
1	0	0	0	0	0	0	0	0
0	0	0	0	0	0	0	0	0
1	0	0	0	0	0	0	0	0
0	0	0	0	0	0	0	0	0
0	0	0	0	0	0	0	0	0
0	0	0	0	0	0	0	0	0
1	0	0	0	0	0	1	0	0
0	0	0	0	0	0	0	0	0
0	0	0	0	0	0	0	1	0
0	0	0	0	1	0	0	0	0
0	0	0	0	0	0	0	1	1
0	0	0	0	0	0	0	1	0
1	0	0	0	0	0	0	0	0
0	0	0	0	0	0	0	0	0
1	0	0	0	0	0	0	1	0
0	0	0	0	0	0	0	0	0
1	0	0	0	0	0	0	0	0
0	0	0	0	0	0	0	0	0
0	0	0	0	0	0	0	0	0
0	0	0	0	0	0	0	0	0
0	0	1	0	0	0	0	0	0
1	0	0	0	0	0	0	0	0
0	0	0	0	0	0	0	1	0
0	0	0	0	0	0	0	0	0
1	1	0	0	0	0	0	0	0
1	0	0	0	0	0	0	0	0
0	0	0	0	0	0	0	0	0
0	0	0	0	0	0	0	0	0
1	0	0	0	0	0	0	1	0
0	0	0	0	0	0	0	0	0
1	0	0	0	0	0	0	0	0
0	0	0	0	0	0	0	0	0
1	0	0	0	0	0	0	0	0
0	0	0	0	0	0	0	0	0

0	0	0	0	0	0	0	0	0
0	0	0	0	0	0	0	1	0
1	0	0	0	0	0	0	0	0
1	0	0	0	0	0	0	0	0
0	1	0	1	0	0	0	0	0
0	0	0	0	0	0	0	0	0
1	0	0	0	0	0	0	0	0
0	0	0	0	0	0	0	0	0
1	0	0	0	1	0	0	1	0
1	0	0	0	0	0	0	0	0
1	0	0	0	0	0	0	0	0
1	0	0	0	0	0	0	0	0
0	0	0	0	0	0	0	0	0
1	0	0	1	0	0	0	0	0
1	0	0	0	0	0	0	0	0
0	0	0	0	0	0	0	1	0
1	0	0	0	0	0	0	0	0
0	0	0	0	0	0	0	0	0
0	0	0	0	0	0	0	0	0
1	0	0	0	0	0	0	0	0
0	0	0	0	0	0	0	0	0
1	0	0	0	0	0	0	0	0
0	1	0	0	0	0	0	0	0
1	0	0	0	0	0	0	0	0
1	0	0	0	0	0	0	0	0
1	0	0	0	0	0	0	0	0
1	0	0	0	0	0	0	0	0
0	0	0	0	0	0	0	0	0
0	0	0	0	0	0	0	0	0
0	0	0	0	0	0	0	0	0
0	0	0	0	0	0	0	0	0
1	0	0	0	0	0	0	0	0
0	0	0	0	0	0	0	0	0
0	0	0	0	0	0	0	0	0
0	0	0	0	0	0	0	0	0
0	0	0	0	0	0	0	0	0
1	0	0	0	0	0	0	0	0
0	0	0	0	0	0	0	1	0
0	0	0	0	1	0	0	1	0
0	0	0	0	0	0	0	0	0
1	0	0	0	0	0	0	0	0
1	0	0	0	0	0	0	0	0
1	0	0	0	0	0	0	0	0
1	0	0	0	0	0	0	0	0
1	0	0	0	0	0	0	0	0
0	0	0	0	0	0	0	0	0
0	0	0	0	0	0	0	0	0
0	0	0	0	0	0	0	0	0
0	0	0	0	0	0	0	0	0
0	0	0	0	0	0	0	0	0
0	0	0	0	0	0	0	0	0
1	0	0	0	0	0	0	1	0
0	0	0	0	0	0	0	0	0
1	0	0	0	0	0	0	0	0
0	0	0	0	0	0	1	0	0
0	0	0	0	0	0	0	1	0

1	0	0	0	0	0	0	0	0
1	0	0	0	0	0	0	0	0
0	0	0	0	0	0	0	0	0
1	0	0	0	0	0	0	0	0
1	0	0	0	1	0	0	0	0
0	1	0	0	0	0	0	0	0
1	0	0	0	0	0	0	0	0
1	0	0	0	0	0	0	0	0
0	0	0	0	0	0	0	0	0
1	0	0	0	0	0	0	0	0
0	0	0	0	0	0	0	0	0
1	0	0	0	0	0	0	0	0
1	0	0	0	0	0	0	0	0
1	0	0	0	0	0	0	0	0
0	0	0	0	0	0	0	1	0
1	0	0	0	0	0	0	0	0
0	0	0	0	0	0	0	0	0
1	0	0	0	0	0	0	0	0
0	0	0	0	0	0	0	0	0
1	0	0	0	0	0	0	0	0
1	0	0	0	0	0	0	0	0
0	0	0	0	0	0	0	0	0
1	0	0	0	0	0	0	1	0
1	0	0	0	0	0	0	0	0
1	0	0	0	0	0	0	0	0
0	0	0	0	0	0	0	0	0
0	0	0	0	0	0	0	0	0
0	0	0	0	0	0	0	0	0
1	0	0	0	0	0	0	1	0
1	0	0	0	0	0	0	0	0
0	0	0	0	0	0	0	0	0
1	0	0	0	0	0	0	0	0
1	0	0	0	0	0	0	0	0
1	0	0	0	0	0	0	0	0
1	0	0	0	0	0	0	0	0
1	0	0	0	0	0	0	0	0
1	0	0	0	0	0	0	0	0
0	0	0	0	0	0	0	0	0
0	0	0	0	0	0	0	0	0
0	0	0	0	0	0	0	0	0
0	0	0	0	0	0	0	0	0
0	0	0	0	0	0	0	0	0
1	0	0	0	0	0	0	0	0
1	0	0	0	0	0	0	0	0
0	0	0	0	0	0	1	0	0
0	0	0	0	0	0	0	0	0
0	0	0	0	0	0	0	0	0
1	0	0	0	0	0	0	0	0
1	0	0	0	0	0	0	0	0
1	0	0	0	0	0	0	0	0
1	0	0	0	0	0	0	0	0
1	0	0	0	0	0	0	0	0
1	0	0	0	0	0	1	0	0
0	0	0	0	0	0	0	0	0
0	0	0	0	0	0	0	0	0
1	0	0	0	0	0	0	0	0

1	0	0	0	0	0	0	1	0
0	0	0	0	0	0	0	0	0
0	0	0	0	0	0	0	0	0
0	0	0	0	0	0	0	1	0
1	0	0	0	0	0	0	0	0
0	0	0	0	0	0	0	0	0
1	0	0	0	0	0	0	0	0
0	0	0	0	0	0	0	0	0
1	0	0	0	0	0	0	0	0
0	0	0	0	0	0	0	1	0
1	0	0	0	0	0	0	0	0
0	0	0	0	0	0	0	0	0
1	0	0	0	0	0	0	0	0
1	0	0	0	0	0	0	0	0
0	0	0	0	0	0	0	1	0
1	0	0	0	0	0	1	0	0
1	0	0	0	0	0	0	0	0
0	0	0	0	0	0	0	0	0
1	0	0	0	0	0	0	0	0
0	0	0	0	0	0	0	0	0
0	0	0	0	0	0	0	0	0
0	0	0	0	0	0	0	0	0
0	0	0	0	0	0	0	0	0
1	0	0	0	1	0	0	0	0
0	0	0	0	0	0	0	0	0
1	0	0	0	0	0	0	0	0
0	0	0	0	0	0	0	0	0
1	0	0	0	0	0	0	0	0
1	0	0	0	1	0	0	1	0
0	0	0	0	0	0	0	0	0
1	0	0	0	0	0	0	0	0
0	0	0	0	0	0	0	0	0
0	0	0	0	0	0	0	1	0
0	0	0	0	0	0	0	0	0
1	0	0	0	1	0	0	0	0
0	0	0	0	0	0	1	0	0
0	0	0	0	0	0	0	0	0
0	0	0	0	0	0	0	0	0
0	0	0	0	0	0	0	1	0
1	0	0	0	0	0	0	0	0
0	0	0	0	0	0	0	0	0
1	0	0	0	0	0	0	1	0
0	0	0	0	0	0	0	0	0
1	0	0	0	0	0	0	0	0
1	0	0	0	0	0	0	0	0
1	0	0	0	0	0	1	0	0
1	0	0	0	0	0	0	0	0
0	0	0	0	0	0	0	0	0
0	0	0	0	0	0	0	0	0
1	0	0	0	0	0	0	0	0
0	0	0	0	0	0	0	0	0
1	0	0	0	0	0	0	0	0
0	0	0	0	0	0	0	0	0
0	0	0	0	0	0	0	0	0
0	0	0	0	0	0	0	0	0
1	0	0	0	0	0	0	1	0

1	0	0	0	0	0	0	0	0
1	0	0	0	0	0	0	0	0
1	0	0	0	0	0	0	0	0
0	0	0	0	0	0	0	0	0
1	0	0	0	0	0	0	0	0
1	0	0	0	0	0	0	0	0
0	0	0	0	0	0	0	0	0
1	0	0	0	0	0	0	0	0
0	0	0	0	0	0	0	0	0
0	0	0	0	0	0	0	0	0
0	0	0	0	0	0	0	1	0
0	0	0	0	0	0	0	1	0
1	0	0	0	0	0	0	0	0
0	0	0	0	0	0	0	0	0
0	0	0	0	0	0	0	0	0
0	0	0	0	0	0	0	0	1
0	0	0	0	0	0	0	0	0
1	0	0	0	0	0	0	0	0
0	0	0	0	0	0	0	0	0
0	0	0	0	0	0	0	0	0
1	1	0	0	0	0	0	0	0
0	0	0	0	0	0	0	0	0
0	1	0	0	0	0	0	0	0
0	0	0	0	0	0	0	0	0
0	1	0	0	0	0	0	0	0
1	0	0	0	0	0	0	0	0
1	0	0	0	0	0	0	0	0
0	1	0	0	0	0	0	0	0
1	0	0	0	0	0	0	0	0
0	0	0	0	1	0	0	0	0
1	0	0	0	0	0	0	0	0
1	0	0	0	0	0	0	0	0
1	0	0	0	0	0	0	0	0
0	0	0	0	0	0	0	0	0
0	0	0	0	0	0	0	0	0
1	0	0	0	0	0	0	1	0
0	0	0	0	0	0	0	0	1
0	0	0	0	0	0	0	1	0
0	0	0	0	0	0	0	0	0
0	0	0	0	0	0	0	0	0
1	0	0	0	1	0	0	0	0
0	0	0	0	0	0	0	0	0
0	0	0	0	0	0	0	0	0
0	0	0	0	0	0	0	0	0
1	0	0	0	0	0	0	0	0
0	0	0	0	0	0	0	0	0
1	0	0	0	0	0	0	0	0
0	0	0	0	0	0	0	0	0
0	0	0	0	0	0	0	0	0
1	0	0	0	0	0	0	0	0
0	0	0	0	0	0	0	0	0
0	0	0	0	0	0	0	0	0
0	1	0	0	0	0	0	0	0
0	0	0	0	0	0	0	0	0
0	0	0	0	0	0	0	0	0
1	0	0	0	0	0	0	0	0

0	0	0	0	0	0	0	1	0
1	0	0	0	0	0	0	0	0
0	0	0	0	0	0	0	0	0
1	0	0	0	0	0	0	0	0
1	0	0	0	0	0	0	0	0
0	0	0	0	0	0	0	0	0
1	0	0	0	0	0	0	0	0
0	0	0	0	0	0	0	0	0
0	0	0	0	0	0	0	0	0
1	0	0	0	0	0	0	0	0
1	0	0	0	1	0	0	0	0
1	0	0	0	0	0	0	0	0
1	0	0	0	0	0	0	1	0
1	0	0	0	0	0	1	1	0
1	0	0	0	0	0	0	0	0
0	0	0	0	0	0	0	0	0
0	0	0	0	0	0	0	0	0
0	0	0	0	0	0	0	0	0
1	0	0	0	0	0	0	0	0
1	0	0	0	0	0	0	0	0
1	0	0	0	0	0	0	0	0
1	0	0	0	0	0	0	0	0
1	0	0	0	0	0	0	0	0
0	0	0	0	0	0	0	0	0
0	0	0	0	0	0	0	0	0
1	0	0	0	0	0	0	0	0
0	0	0	0	0	0	0	0	0
0	0	0	0	0	0	0	0	0
0	0	0	0	0	0	0	0	0
1	1	0	0	0	0	0	0	0
1	0	0	0	0	0	0	0	0
0	0	0	0	0	0	0	0	0
0	0	0	0	0	0	0	0	0
0	0	0	0	0	0	0	1	0
1	0	0	0	0	0	0	0	0
0	0	0	0	0	0	0	0	0
0	0	0	0	0	0	0	0	0
0	0	0	0	0	0	0	0	0
0	0	0	0	0	0	0	0	0
1	1	0	0	0	0	0	0	0
1	0	0	0	0	0	0	0	0
1	0	0	0	0	0	0	0	0
1	0	0	0	0	0	0	0	0
1	0	0	0	0	0	0	0	0
0	0	0	0	0	0	0	0	0
1	0	0	0	0	0	0	0	0
1	0	0	0	0	0	0	0	0
1	0	0	0	0	0	0	0	0
1	0	0	0	0	0	0	0	0
1	0	0	0	0	0	0	0	0
1	0	0	0	0	0	0	0	0
1	0	0	0	0	0	0	0	0
0	0	0	0	0	0	0	0	0
0	0	0	0	0	0	0	0	0
0	1	0	0	0	0	0	0	0
1	0	0	0	0	0	0	0	0

0	0	0	0	0	0	0	0	0
0	0	0	0	0	0	0	1	0
0	0	0	0	0	0	0	0	0
0	0	0	0	0	0	0	0	0
0	0	0	0	0	0	0	0	0
1	0	0	0	0	0	0	0	0
0	0	0	0	0	0	0	0	0
0	0	0	0	0	0	0	0	0
1	0	0	0	0	0	0	0	0
0	0	0	0	0	0	0	0	1
0	0	0	0	0	0	0	0	0
1	0	0	0	0	0	0	0	0
1	0	0	0	0	0	0	0	0
1	0	0	0	0	0	0	0	0
1	0	0	0	0	0	0	0	0
1	0	0	0	0	0	0	0	0
1	0	0	0	0	0	0	0	0
0	0	0	0	0	0	0	0	0
1	0	0	0	0	0	0	0	0
0	0	0	0	0	0	0	0	0
0	0	0	0	0	0	0	1	0
1	0	0	0	0	0	0	0	0
1	0	0	0	0	0	0	0	0
1	0	0	0	0	0	0	0	0
1	0	0	0	0	0	0	0	0
1	0	0	0	0	0	0	0	0
1	0	0	0	0	0	0	0	0
1	1	0	0	0	0	0	0	0
0	1	0	0	0	0	0	0	0
0	0	0	0	0	0	0	0	0
1	0	0	0	0	0	0	0	0
0	0	0	0	0	0	0	1	0
0	0	0	0	0	0	0	0	0
1	0	0	0	0	0	0	0	0
1	0	0	0	0	0	0	0	0
1	0	0	0	0	0	0	0	0
0	0	0	0	0	0	1	0	0
0	0	0	0	0	0	0	0	0
1	0	0	0	0	0	0	0	0
1	0	0	0	0	0	0	1	0
1	0	0	0	0	0	0	0	0
1	0	0	0	0	0	0	0	0
1	0	0	0	0	0	0	1	0
0	1	0	0	0	0	0	0	0
0	0	0	0	0	0	0	0	0
0	1	0	0	0	0	0	0	0
0	0	0	0	0	0	0	1	1
1	0	0	0	0	0	0	1	0
0	0	0	0	0	0	0	0	0
0	0	0	0	0	0	0	0	0
0	0	0	0	0	0	0	0	0
0	0	0	0	0	0	0	0	0
1	0	0	0	0	0	0	0	0
1	0	0	0	0	0	0	1	0
1	0	0	0	0	0	0	0	0
0	0	0	0	0	0	0	0	0

1	0	0	0	0	0	0	0	0
0	0	0	0	0	0	0	0	0
1	0	0	0	0	0	0	0	0
0	0	0	0	0	0	0	1	0
0	0	0	0	0	0	0	0	0
1	0	0	0	0	0	0	0	1
1	0	0	0	0	0	0	0	0
1	0	0	0	0	0	0	0	0
1	0	0	0	0	0	0	1	0
1	0	0	0	0	0	0	0	0
1	1	0	0	0	0	0	1	0
0	1	0	0	0	0	1	0	0
0	0	0	0	0	0	0	0	0
1	0	0	0	0	0	0	0	0
0	0	0	0	0	0	0	0	0
0	0	0	0	0	0	0	0	0
0	0	0	0	0	0	0	0	0
0	0	0	0	0	0	0	1	0
0	0	0	0	0	0	0	1	0
0	0	0	0	1	0	0	0	0
0	0	0	0	0	0	0	0	0
0	0	0	0	0	0	0	0	0
0	0	0	0	0	0	0	0	0
1	0	0	0	0	0	0	0	0
0	0	0	0	0	0	0	0	0
1	0	0	0	0	0	0	0	0
0	0	0	0	0	0	0	0	0
0	0	0	0	0	0	0	0	0
0	0	0	0	0	0	0	0	0
0	0	0	0	0	0	0	0	0
0	0	0	0	0	0	0	0	0
0	0	0	0	0	0	0	0	0
0	0	0	0	0	0	0	0	0
0	1	0	0	0	0	0	0	0
1	0	0	0	0	0	0	0	0
0	0	0	0	0	0	0	0	0
0	0	0	0	0	0	0	0	0
0	1	0	0	0	0	0	0	0
0	0	0	0	0	0	0	0	0
0	0	0	0	0	0	0	0	0
1	0	0	0	0	0	0	0	0
0	0	0	0	0	0	0	0	0
0	1	0	0	0	0	0	0	0
0	0	0	0	0	0	0	0	0
1	0	0	0	0	0	0	0	0
0	0	0	0	0	0	0	0	0
1	0	0	0	0	0	0	0	0
0	0	0	0	0	0	0	0	0
1	0	0	0	0	0	0	0	0
0	0	0	0	0	0	0	0	0
1	0	0	0	0	0	0	0	0
1	0	0	0	0	0	0	0	0
0	0	0	0	0	0	0	0	0
0	0	0	0	0	0	0	0	0
0	0	0	0	0	0	0	0	0
0	0	0	0	0	0	0	0	0

1	0	0	0	0	0	0	0	0
0	0	0	0	0	0	0	0	0
1	0	0	0	0	0	0	0	0
0	0	0	0	0	0	0	1	0
1	0	0	0	0	0	0	0	0
1	0	0	0	0	0	0	0	0
0	0	0	0	0	0	0	0	0
0	0	0	0	0	0	0	0	0
0	0	0	0	0	0	0	0	0
0	0	0	0	0	0	0	0	0
1	0	0	0	0	0	0	0	0
0	0	0	0	0	0	1	0	0
1	0	0	0	0	0	0	0	0
0	0	0	0	0	0	0	1	0
0	0	0	0	0	0	0	0	0
1	0	0	0	0	0	0	0	0
0	0	0	0	0	0	0	1	0
0	0	0	0	0	0	0	1	0
0	0	0	0	0	0	0	0	0
0	0	0	0	0	0	0	0	0
0	0	0	0	0	0	0	1	0
0	0	0	0	0	0	0	0	0
0	0	0	0	0	0	0	0	0
0	0	0	0	0	0	0	0	0
0	0	0	0	0	0	0	0	0
0	0	0	0	0	0	1	0	0
0	0	0	0	0	0	0	0	0
1	0	0	0	0	0	0	0	0
0	0	0	0	0	0	0	0	0
0	0	0	0	0	0	0	0	0
1	0	0	0	0	0	0	0	0
0	0	0	0	0	0	0	0	0
1	0	0	0	0	0	0	0	0
1	0	0	0	0	0	0	0	0
0	0	0	0	0	0	0	0	0
1	0	0	0	0	0	0	0	0
0	0	0	0	1	0	0	0	0
0	0	0	0	0	0	0	0	0
1	0	0	0	1	0	0	0	0
0	0	0	0	0	0	0	0	0
0	0	0	0	0	0	0	0	0
0	0	0	0	0	0	1	0	0
0	0	0	0	0	0	0	1	0
0	0	1	0	0	0	0	0	0
1	0	0	0	0	0	0	0	0
1	0	0	0	0	0	0	0	0
1	0	0	0	0	0	0	0	0
0	0	0	0	0	0	0	1	0
0	0	0	0	0	0	0	1	0
0	0	0	0	0	0	0	1	0
0	0	0	0	0	0	0	0	0
1	0	0	0	0	0	0	0	0
0	1	0	0	0	0	0	0	0
0	0	0	0	1	0	0	0	0
0	0	0	0	0	0	0	1	0
1	1	0	0	0	0	0	0	0

1	0	0	0	0	0	0	0	0
0	0	0	0	0	0	0	0	0
0	0	0	0	0	0	0	0	0
1	1	0	0	0	0	0	1	0
1	0	0	0	0	0	0	0	1
0	0	0	0	0	0	0	0	0
1	0	0	0	0	0	0	0	0
1	0	0	0	0	0	0	0	0
0	0	0	0	0	0	0	0	0
1	1	0	0	0	0	0	0	0
0	0	0	0	0	0	0	0	0
0	0	0	0	0	0	0	0	0
0	0	0	0	0	0	0	0	0
1	0	0	0	0	0	0	0	0
0	0	0	0	0	0	0	0	0
0	0	0	0	0	0	0	0	0
1	0	0	0	0	0	0	0	0
0	0	0	0	0	0	0	0	0
0	0	0	0	0	0	0	0	0
0	0	0	0	0	0	0	0	0
0	0	0	0	0	0	0	0	0
0	0	0	0	0	0	0	0	0
0	0	0	0	0	0	1	0	0
0	0	0	0	0	0	0	0	0
0	0	0	0	0	0	0	0	0
0	0	0	0	0	0	0	0	0
1	0	0	0	0	0	0	0	0
1	0	0	0	0	0	0	0	0
1	0	0	0	0	0	0	1	0
1	0	0	0	0	0	0	0	0
0	0	0	0	0	0	0	0	0
1	0	0	0	0	0	0	0	0
0	0	0	0	0	0	0	0	0
0	0	0	0	0	0	0	0	0
0	0	0	0	0	0	0	0	0
0	0	0	0	0	0	0	0	0
0	0	0	0	0	0	0	0	0
0	0	0	0	0	0	0	0	0
0	0	0	0	0	0	0	0	0
0	1	0	0	0	0	0	0	0
1	0	0	0	0	0	0	0	0
1	0	0	0	0	0	0	0	0
0	0	0	0	0	0	0	0	0
0	0	0	0	0	0	0	0	0
0	0	0	0	0	0	0	0	0
0	0	0	0	0	0	0	0	0
1	0	0	0	0	0	0	0	0
0	0	0	0	0	0	0	0	0
0	0	0	0	0	0	0	0	0
0	0	0	0	0	0	0	0	0
1	0	0	0	0	0	0	0	0
0	0	0	0	0	0	0	0	0
0	0	0	0	0	0	0	0	0
1	0	0	0	0	0	0	0	0
0	0	0	0	0	0	1	0	0
0	0	0	0	0	0	0	0	0

0	0	0	0	0	0	0	0	0
0	0	0	0	0	0	1	0	0
1	0	0	0	0	0	0	0	0
0	0	0	0	0	0	0	1	0
0	0	0	0	0	0	0	0	0
0	0	0	0	0	0	0	0	0
0	0	0	0	0	0	0	0	0
1	0	0	0	0	0	0	0	0
1	0	0	0	0	0	0	0	0
0	0	0	0	0	0	0	0	0
0	0	0	0	0	0	0	1	0
0	0	0	0	0	0	0	0	0
0	0	0	0	0	0	0	0	0
0	0	0	0	0	0	1	0	0
0	0	0	0	0	0	1	1	0
1	0	0	0	0	0	0	0	0
0	0	0	0	0	0	0	1	0
0	0	0	0	0	0	1	1	0
0	0	0	0	0	0	0	0	0
0	0	0	0	0	0	0	0	0
0	0	0	0	0	0	0	0	0
0	0	0	0	0	0	0	0	0
0	1	0	0	0	0	0	0	0
1	0	0	0	0	0	0	1	0
0	0	0	0	0	0	0	0	0
0	0	0	0	1	0	0	0	0
0	0	0	0	1	0	0	0	0
0	0	0	0	1	0	0	0	0
0	0	0	0	0	0	0	0	0
0	0	0	0	1	0	0	0	0
0	0	0	0	0	0	0	0	0
1	0	0	0	0	0	0	0	0
0	0	0	0	1	1	0	1	0
0	0	0	0	0	0	0	0	0
0	0	0	0	0	0	0	0	0
0	0	0	0	0	0	0	0	0
0	0	0	0	0	0	0	0	0
0	1	0	0	0	0	0	1	0
0	0	0	0	0	0	0	0	0
0	0	0	0	0	0	0	0	0
0	0	0	0	0	0	0	0	0
0	0	0	0	0	0	0	0	0
0	0	0	0	0	0	0	0	0
0	0	0	0	0	0	0	0	0
0	0	0	0	0	0	0	0	0
0	0	0	0	0	0	0	0	0
0	0	0	0	0	0	0	0	0
0	0	0	0	0	0	0	0	0
0	0	0	0	0	0	0	0	0
0	0	0	0	0	0	0	0	0
0	0	0	0	0	0	0	0	0
0	0	0	0	0	0	0	0	0
0	0	0	0	0	0	0	0	0
0	0	0	0	0	0	0	0	0
0	0	0	0	0	0	0	0	0
0	0	0	0	0	0	0	0	0

0	0	0	0	0	0	0	0	0
0	0	0	0	0	0	0	0	0
0	0	0	0	0	0	0	0	0
0	0	0	0	0	0	0	0	0
0	0	0	0	1	0	0	0	1
0	0	0	0	0	0	0	0	0
0	0	0	0	0	0	0	0	0
1	0	0	0	0	0	0	0	0
1	0	0	0	0	0	0	0	0
0	0	0	0	0	0	0	0	0
0	0	0	0	0	0	0	0	0
0	0	0	0	0	0	0	0	0
0	0	0	0	0	0	0	0	0
0	0	0	0	0	0	0	0	0
0	0	0	0	1	1	0	0	0
0	0	0	0	0	0	0	0	0
0	0	0	0	0	0	0	0	0
0	0	0	0	0	0	0	0	0
0	0	0	0	0	0	0	0	0
0	0	0	0	0	0	0	0	0
0	0	0	0	0	0	0	0	0
1	0	0	0	0	0	0	0	0
0	0	0	0	0	0	0	0	0
0	0	0	0	0	0	0	0	0
0	0	0	0	0	0	0	0	0
0	0	0	0	0	0	0	0	0
0	0	0	0	0	0	0	0	0
0	0	0	0	0	0	0	0	0
1	0	0	0	0	0	0	0	0
0	0	0	0	0	0	0	0	0
0	0	0	0	0	0	0	0	0
1	0	0	0	0	0	0	0	0
0	0	0	0	0	0	0	0	0
0	0	0	0	0	0	0	0	0
0	0	0	0	0	0	0	0	0
1	0	0	0	0	0	0	0	0
0	0	0	0	0	0	0	0	0
0	0	0	0	0	0	0	0	0
0	0	0	0	0	0	0	0	0
0	0	0	0	0	0	0	0	0
0	0	0	0	0	0	0	0	0
0	0	0	0	0	0	0	0	0
1	0	0	0	0	0	0	0	0
0	0	0	0	0	0	0	0	0
0	0	0	0	0	0	0	0	0
0	0	0	0	0	0	0	0	0
0	0	0	0	0	0	0	0	0
0	0	0	0	0	0	0	0	0
0	0	0	0	0	0	0	0	0
0	0	0	0	0	0	0	0	0
0	0	0	0	0	0	0	0	0
0	0	0	0	0	0	0	0	0
0	0	0	0	0	0	0	0	0
0	0	0	0	1	0	0	0	0
0	0	0	0	0	0	0	0	0
0	0	0	0	0	0	0	0	0

0	0	0	0	0	0	0	0	0
0	0	0	0	1	0	0	0	0
0	0	0	0	1	0	0	0	0
0	0	0	0	0	0	0	0	0
0	0	0	0	0	0	0	0	0
0	0	0	0	0	0	0	0	0
0	0	0	0	0	0	0	0	0
0	0	0	0	0	0	0	0	0
0	0	0	0	0	0	0	0	0
0	0	0	0	1	0	0	0	0
0	0	1	0	1	0	0	0	0
0	0	0	0	1	0	0	0	0
0	0	0	0	0	0	0	0	0
0	0	0	0	0	0	0	0	0
0	0	0	0	0	0	0	0	0
0	0	0	0	0	0	0	0	0
0	1	1	0	0	0	0	0	0
0	0	0	0	0	0	0	0	0
0	0	0	0	0	0	0	0	0
0	0	0	0	0	0	0	0	0
0	0	0	0	0	0	0	0	0
0	0	0	0	0	0	0	0	0
0	0	0	0	0	0	0	0	0
0	0	0	0	1	0	0	0	0
0	0	0	0	0	0	0	0	0
0	0	0	0	0	0	0	0	0
0	0	0	0	1	0	0	0	0
0	0	0	0	0	0	0	0	0
0	1	0	0	0	0	1	0	0
0	0	0	0	0	0	0	0	0
0	0	0	0	0	0	0	0	0
1	0	1	0	0	0	0	0	0
0	0	0	0	0	0	0	0	0
0	0	0	0	0	0	0	0	0
0	0	0	0	0	0	0	0	0
0	0	0	0	0	0	0	0	0
0	0	0	0	0	0	0	0	0
0	0	1	0	0	0	0	0	0
0	0	0	0	0	0	0	0	0
0	0	0	0	1	0	0	0	0
0	0	0	0	0	0	0	0	0
0	0	0	0	1	0	1	0	0
0	0	0	0	0	0	0	0	1
0	0	0	0	0	0	0	0	0
0	0	0	0	0	0	0	0	0
1	0	0	0	0	0	0	0	0
1	0	0	0	0	0	0	0	0
0	0	0	0	0	0	0	0	0
1	0	0	0	0	0	0	0	0
1	0	0	0	0	0	0	0	0
1	0	0	0	0	0	0	0	0
0	0	0	0	0	0	0	0	0
1	0	0	0	0	0	0	0	0
1	0	0	0	0	0	0	0	0
1	0	0	0	0	0	0	0	0
0	0	0	0	0	0	0	1	0

1	0	0	0	0	0	0	0	0
0	0	0	0	0	0	0	0	0
0	0	0	0	0	0	0	0	0
1	0	0	0	0	1	0	1	0
0	0	0	0	0	0	0	1	0
0	0	0	0	0	0	0	1	0
0	0	0	0	0	0	0	1	0
0	0	0	0	1	0	0	0	0
1	0	0	0	0	0	0	0	0
0	0	0	0	0	0	0	0	0
1	0	0	0	0	0	0	1	0
1	0	0	0	1	0	0	1	0
0	0	0	0	0	0	0	0	0
0	0	0	0	0	0	0	0	0
0	0	0	0	0	0	0	0	0
0	1	0	0	0	0	0	0	0
0	0	0	0	0	0	0	0	0
0	1	0	0	0	0	0	0	0
1	0	0	0	0	0	0	0	0
1	0	0	0	1	0	0	0	0
0	0	0	0	0	0	0	0	0
0	0	0	0	0	0	0	0	0
0	0	0	0	0	0	0	0	0
0	0	0	0	0	0	0	0	0
0	0	0	0	0	0	0	1	0
0	0	0	0	0	0	0	0	0
0	0	1	0	0	0	0	0	0
1	0	0	0	0	0	0	0	0
0	0	0	0	0	0	0	0	0
0	0	0	0	0	0	0	1	1
1	0	0	0	0	0	0	0	0
0	0	0	0	0	0	0	0	0
0	0	0	0	0	0	0	0	0
0	0	0	0	0	0	0	0	0
0	0	0	0	0	0	0	1	0
0	0	0	0	0	0	0	0	0
0	0	0	0	0	0	0	0	0
0	0	0	1	0	0	0	0	0
0	0	0	0	0	1	0	0	0
0	0	0	0	0	0	0	0	0
0	0	0	0	0	0	0	0	0
0	0	0	0	0	0	0	0	0
0	0	0	0	0	0	0	0	0
1	0	0	0	0	0	0	1	0
0	0	0	0	0	0	0	0	0
0	1	0	0	0	0	0	0	0
1	0	0	0	0	0	1	0	0
1	0	0	0	0	0	0	0	0
0	0	0	0	0	0	0	1	0
0	0	1	0	0	0	0	0	0
0	0	0	0	0	0	0	0	0
1	0	0	0	0	0	0	0	0
0	0	0	0	0	0	0	0	0
0	0	0	0	0	0	0	0	0
0	0	0	0	0	0	0	0	1
0	0	0	0	0	0	0	0	0

0	0	0	0	0	0	0	0	0
0	0	0	0	0	0	0	0	0
0	0	0	0	0	0	0	0	0
1	0	0	0	0	0	0	0	0
1	0	0	0	0	0	0	0	0
0	0	0	0	0	0	0	0	0
0	0	0	0	0	0	0	0	0
0	0	0	0	0	0	1	0	0
1	0	0	0	1	0	0	0	0
0	0	0	0	0	0	0	0	0
0	0	0	0	0	0	0	0	0
0	0	0	0	0	0	0	0	0
0	0	0	0	0	0	0	0	0
0	0	0	0	0	0	0	0	0
1	0	0	0	0	0	0	0	0
0	0	0	0	0	0	0	0	0
0	0	0	0	0	0	0	0	0
0	0	0	0	0	0	0	0	0
0	0	0	0	0	0	0	0	0
0	0	0	0	0	0	0	0	0
1	0	0	0	0	0	0	0	0
1	0	0	0	0	0	0	0	0
0	1	0	0	0	0	0	1	0
1	0	0	0	0	0	0	0	0
1	0	0	0	0	0	0	0	0
1	0	0	0	0	0	0	1	0
1	0	0	0	0	0	0	0	0
1	0	0	0	0	0	0	0	0
0	0	0	0	0	0	0	0	0
0	0	0	0	0	0	0	0	0
0	0	0	0	0	0	0	0	0
1	0	0	0	0	0	0	0	0
0	0	0	0	0	0	0	0	0
1	0	0	0	0	0	0	0	0
0	0	0	0	0	0	0	0	0
0	0	0	0	0	0	0	0	0
0	0	0	0	0	0	0	1	0
0	0	0	0	0	0	0	0	0
0	1	0	0	0	0	0	0	0
0	0	0	0	0	0	1	0	0
0	0	0	0	0	0	0	0	0
0	0	0	0	0	0	0	0	0
0	0	0	0	0	0	0	0	0
0	0	0	0	0	0	0	0	1
0	0	0	0	0	0	0	0	0
0	0	0	0	0	0	0	1	0
0	0	0	0	0	0	0	0	0
0	1	0	0	0	0	0	0	0
0	0	0	0	0	0	0	0	0
0	0	0	0	0	0	0	0	0
1	0	0	0	0	0	0	0	0
0	1	0	0	0	0	0	0	0
0	1	1	0	0	1	0	1	0
0	0	0	0	0	0	0	0	0
0	0	0	0	0	0	0	0	0
0	0	0	0	0	0	0	0	0

0	0	0	0	0	0	0	0	0
1	0	0	0	0	0	0	0	0
0	0	0	0	0	0	0	0	0
0	0	0	0	0	0	0	0	0
0	0	0	0	0	0	0	1	0
0	0	0	0	0	0	0	0	0
0	0	0	0	0	0	0	1	1
0	0	0	0	0	0	1	0	0
0	0	0	0	0	0	0	0	0
0	0	0	0	0	0	0	0	0
1	0	0	0	0	0	0	1	0
0	0	0	0	0	0	0	0	0
0	1	0	0	0	0	0	0	0
1	0	0	0	0	0	0	0	0
0	0	0	0	0	0	0	0	0
0	0	0	0	0	0	0	0	0
0	1	0	0	0	0	0	0	0
0	0	0	0	0	0	0	0	0
0	0	0	0	0	0	0	0	0
0	0	0	0	0	0	0	0	0
1	0	0	0	0	0	0	0	0
0	0	0	0	0	0	0	0	0
1	0	0	0	0	0	0	0	0
0	0	0	0	0	0	0	0	0
0	0	0	0	0	0	1	0	0
1	0	0	0	0	0	0	0	0
1	0	0	0	0	0	0	0	0
0	0	0	0	0	0	0	0	0
0	1	0	0	0	0	0	0	0
0	0	0	0	0	0	0	0	0
1	0	0	0	0	0	0	0	0
0	0	0	0	0	0	0	1	0
0	0	0	0	0	0	0	0	0
0	0	0	0	0	0	0	0	0
1	0	0	0	0	0	0	0	0
0	0	0	0	0	0	0	0	0
0	0	0	0	0	0	0	0	0
0	0	0	0	0	1	0	0	0
0	0	0	0	1	0	0	0	0
1	0	0	0	1	0	0	0	0
0	0	0	0	0	0	0	0	0
0	0	0	0	0	0	0	0	0
1	0	0	0	1	0	0	0	0
0	0	0	0	1	0	0	0	0
0	0	0	0	0	0	0	0	0
0	0	0	0	0	0	0	0	0
0	0	0	0	0	0	0	0	0
0	0	0	0	0	0	0	1	0
0	0	0	0	1	0	0	0	0
0	0	0	0	0	0	1	0	0
1	0	0	0	0	0	0	0	0
0	0	0	0	0	0	0	0	0
0	0	0	0	1	0	0	0	0
0	0	0	0	0	0	0	0	0
0	0	0	0	0	0	0	0	0
0	0	0	0	0	0	0	0	0

0	0	0	0	0	0	0	0	0
0	0	0	0	0	0	0	0	0
0	0	0	0	0	0	0	0	0
0	0	0	0	0	0	0	0	0
0	0	0	0	0	0	0	1	0
0	0	0	0	0	0	0	0	0
0	0	0	0	0	0	0	0	0
0	0	0	0	0	0	1	0	0
0	0	0	0	0	0	0	0	0
0	0	0	0	0	0	0	0	0
0	0	0	0	0	0	0	0	0
0	0	0	0	0	0	0	1	0
0	0	0	0	0	0	0	0	0
0	0	0	0	0	0	0	0	0
0	0	0	0	0	0	0	0	0
0	0	0	0	0	0	0	0	0
0	0	0	0	0	0	0	1	0
0	1	0	0	1	0	0	0	0
0	0	0	0	0	0	0	0	0
0	0	0	0	0	0	0	1	0
1	0	0	0	1	0	0	0	0
0	0	0	1	0	0	0	0	0
0	0	0	0	0	0	0	0	0
0	0	0	0	1	0	0	0	0
0	0	0	0	0	0	1	0	0
0	0	0	0	0	0	0	0	0
0	0	0	0	0	0	0	0	0
0	1	0	0	0	0	0	1	0
0	0	0	0	0	0	0	0	0
1	0	0	0	0	0	0	0	0
0	0	0	0	0	0	0	0	0
0	0	0	0	0	0	0	1	0
0	0	0	0	0	0	0	0	0
0	0	0	0	0	0	0	0	0
0	0	0	0	1	0	0	0	0
0	0	0	0	0	0	0	0	0
0	0	0	0	0	0	0	0	0
0	0	1	0	0	0	0	0	0
1	0	0	0	1	0	0	0	0
0	0	0	1	0	0	0	1	0
0	0	0	0	0	0	0	0	0
0	0	0	0	0	0	0	0	0
0	0	0	0	0	0	0	0	0
0	0	0	0	0	0	0	0	0
1	0	0	0	0	0	0	0	0
0	0	0	1	0	0	0	0	0
0	0	0	0	0	0	0	1	1
0	0	0	0	0	0	0	0	0
0	1	0	0	0	0	0	0	0
0	0	0	0	0	0	0	0	0
0	0	0	0	0	0	0	0	0
0	0	0	0	0	0	0	0	0
0	0	0	0	0	0	0	1	0
0	0	0	0	0	0	0	1	0
0	0	0	0	0	0	1	0	0
0	0	1	0	0	0	0	0	0

1	0	0	0	0	0	0	0	0
1	0	0	0	0	0	0	0	0
0	0	0	0	0	0	0	0	0
0	0	0	0	0	0	0	0	1
0	0	0	0	0	0	0	1	0
0	0	0	0	0	0	0	0	0
0	0	0	0	0	0	0	1	0
0	0	0	0	0	0	0	0	0
0	0	0	0	0	0	0	0	0
0	0	0	0	0	0	0	0	0
0	0	0	0	0	0	0	0	0
0	0	0	1	0	1	0	0	0
0	0	0	0	0	0	1	1	0
0	0	0	0	0	0	0	0	0
0	0	0	0	0	0	0	0	0
0	0	0	0	0	0	0	1	0
0	0	0	0	0	0	0	0	0
0	0	0	0	0	0	0	0	0
0	0	0	0	0	0	0	0	0
0	0	0	0	0	0	0	0	0
0	0	0	0	0	0	0	0	0
1	0	0	0	0	0	0	0	0
0	0	0	0	0	0	0	0	0
0	0	0	0	0	0	0	0	0
0	0	0	0	0	0	0	0	0
0	0	0	0	0	0	0	0	0
0	0	0	0	0	0	0	0	0
1	0	0	0	0	0	0	0	0
0	0	0	0	0	0	0	0	0
0	0	0	0	0	0	0	0	0
0	0	0	0	1	0	0	0	0
0	0	0	0	0	1	0	1	0
0	0	0	0	0	0	0	0	0
0	0	0	0	0	0	0	0	0
0	0	0	0	0	0	0	0	0
0	0	0	0	0	0	0	0	0
0	0	0	0	0	0	0	0	0
0	0	0	0	0	0	0	0	0
0	0	0	0	0	0	0	0	0
0	1	0	0	0	0	0	0	0
0	0	0	0	0	0	0	0	0
1	0	0	0	0	0	0	0	0
0	0	0	0	0	0	0	0	0
0	0	0	0	0	0	0	0	0
0	0	0	0	0	0	0	0	0
0	0	0	1	0	0	0	0	0
0	0	0	0	0	0	0	0	0
0	0	0	0	0	0	1	0	0
0	0	0	0	0	0	0	0	0
0	0	0	0	0	0	0	0	0
0	0	0	0	0	0	0	0	0
0	0	0	0	0	0	0	0	0
0	0	0	0	0	0	0	0	0
1	0	0	0	0	0	0	0	0
0	0	0	0	0	0	0	0	0
1	0	0	0	0	0	0	0	0

0	0	0	0	0	0	0	1	0
0	0	0	0	0	0	0	0	0
0	0	0	0	0	0	0	0	0
0	0	0	0	0	0	0	0	0
0	0	0	0	0	0	1	0	0
0	1	0	0	0	0	0	1	0
0	0	0	0	0	0	0	0	1
0	0	0	0	0	0	0	0	0
0	0	0	0	0	0	0	0	0
0	0	0	0	0	0	0	0	0
0	0	0	0	0	0	0	0	0
0	0	0	0	0	0	0	0	0
0	0	0	0	0	0	0	0	0
0	0	0	0	0	0	0	0	0
0	0	0	0	0	0	0	0	0
0	0	0	0	1	0	0	0	0
0	0	0	0	0	0	0	0	0
0	0	0	0	0	0	0	0	0
0	0	0	0	0	0	0	0	0
0	1	0	0	0	0	0	0	0
0	0	0	0	1	0	0	1	0
0	0	0	0	0	0	0	0	0
0	0	0	0	0	0	0	0	0
0	0	0	0	0	0	0	0	0
0	0	0	0	0	0	0	0	0
1	0	0	0	0	0	1	0	0
0	0	0	1	0	0	0	0	0
0	0	0	0	1	0	0	0	0
0	0	0	0	0	0	0	1	0
0	0	0	0	0	0	0	0	0
0	0	0	0	0	0	0	0	0
0	0	0	0	0	0	0	0	0
1	0	0	0	0	0	0	0	0
0	0	0	0	0	0	0	0	0
0	0	0	0	0	0	0	0	0
0	0	0	0	0	0	0	0	0
0	0	0	0	0	0	0	0	0
0	0	0	0	0	0	0	0	0
0	0	0	0	0	0	0	1	0
1	0	0	0	0	0	0	0	0
0	0	1	0	0	0	0	0	0
0	0	0	0	0	0	0	0	0
0	0	0	0	1	0	0	0	0
0	0	0	0	1	0	0	0	0
0	0	0	1	0	0	0	1	0
0	0	0	0	0	0	0	0	0
0	0	0	0	0	0	0	0	0
0	0	0	1	0	0	0	0	0
0	0	0	0	0	0	0	0	0
0	0	0	0	0	0	0	0	1
0	0	0	0	1	0	0	0	0
0	0	0	0	0	0	0	0	0
0	0	0	0	0	0	0	0	0
0	0	0	0	0	0	0	0	0
0	1	1	0	0	0	0	0	0
0	0	0	0	0	0	0	0	0

0	0	0	0	0	0	0	0	0
0	0	0	0	0	0	0	0	0
0	0	0	0	0	0	0	0	0
0	0	0	0	0	0	0	0	0
0	0	0	0	0	0	0	0	0
0	0	0	0	0	0	0	0	0
0	0	0	0	0	0	0	0	0
0	0	0	0	0	0	0	0	0
0	1	0	0	0	0	0	0	0
0	0	0	0	0	0	0	0	0
0	0	0	0	0	0	0	0	0
1	0	0	0	0	0	0	0	0
0	0	0	0	0	0	0	0	0
0	0	0	0	0	0	0	0	0
0	0	0	1	0	0	0	0	0
0	0	0	0	0	0	0	0	0
0	0	0	0	0	0	0	0	0
0	0	0	0	0	0	0	0	0
0	0	0	0	1	0	0	0	0
0	0	0	0	0	0	0	0	0
0	0	0	0	0	0	0	0	0
1	0	0	0	0	0	0	0	0
0	0	0	0	0	0	0	1	0
0	0	0	0	0	0	0	0	0
0	0	0	0	0	0	0	1	0
0	0	0	1	0	0	0	0	0
0	0	0	0	1	0	0	0	0
0	0	0	0	0	0	0	1	0
0	0	0	0	0	0	0	0	0
0	0	0	0	0	0	0	0	0
0	0	0	0	0	0	0	0	0
0	0	0	0	0	0	0	0	0
0	1	0	0	0	0	0	0	0
0	0	0	0	0	0	0	0	0
0	0	1	0	0	0	0	0	1
0	0	1	0	0	0	0	0	0
0	0	0	0	0	0	0	0	0
0	0	0	0	0	0	0	0	0
1	0	0	0	0	0	0	0	0
0	0	0	0	0	0	0	0	0
1	0	0	1	1	0	0	0	0
0	0	0	0	0	0	0	0	0
0	1	0	0	0	0	0	0	0
0	0	0	0	0	0	0	0	0
0	0	0	0	0	0	0	0	0
0	1	0	0	0	0	0	0	0
0	0	0	0	0	0	0	0	0
0	0	0	0	0	0	0	1	0
0	0	0	0	0	0	0	0	0
0	0	0	1	0	0	0	0	0
0	0	0	0	0	0	0	0	0
1	0	0	0	0	0	0	0	0
0	1	0	0	0	0	0	0	0
0	0	0	0	0	0	0	0	0
0	0	0	0	0	0	0	0	0
0	0	0	0	0	0	0	0	1

0	0	0	0	0	0	1	0	0
0	0	0	0	0	0	0	0	0
0	0	0	0	0	0	0	0	0
0	0	0	0	0	0	0	0	0
0	0	0	0	0	0	0	0	0
0	0	0	0	0	0	0	0	0
1	0	0	0	0	0	0	0	0
0	0	0	0	0	0	0	0	0
0	0	0	0	0	0	0	0	0
0	0	0	0	0	0	0	0	0
0	0	0	0	0	0	1	0	0
0	0	0	0	0	0	0	0	0
0	0	0	0	0	0	0	0	0
0	0	0	0	0	0	0	1	0
0	0	0	0	1	0	0	0	0
0	0	0	0	0	0	1	0	0
0	0	0	0	0	0	0	0	0
0	0	0	0	0	0	0	0	0
0	0	0	0	0	0	0	0	0
0	0	0	1	0	0	0	0	1
0	0	0	0	0	0	0	0	0
0	0	0	1	0	0	0	0	0
0	0	0	0	0	0	0	0	0
0	0	0	1	0	0	0	0	0
0	0	0	0	0	0	0	0	0
0	0	0	0	0	0	0	0	0
0	1	1	0	0	0	0	0	0
1	0	0	0	0	0	0	0	0
0	1	0	0	0	0	0	0	0
0	0	0	0	0	0	0	0	0
0	0	0	0	0	0	0	0	0
0	0	0	0	0	0	0	1	0
0	0	0	0	0	0	0	0	0
0	0	0	0	0	0	0	0	0
0	1	0	0	0	0	0	0	0
0	1	0	0	0	0	0	0	0
0	0	0	0	0	0	0	0	0
0	0	0	0	0	0	0	1	0
0	0	0	1	0	0	0	0	0
0	0	0	0	0	0	0	0	0
1	0	0	0	0	0	0	0	0
0	0	0	0	0	0	0	0	0
0	0	0	0	0	0	0	0	0
1	0	0	0	0	0	0	0	0
0	1	0	0	0	0	1	0	1
0	0	0	0	0	0	0	1	0
0	0	0	0	0	0	0	0	0
0	1	0	0	0	0	0	0	0
0	0	0	0	0	0	0	0	0
0	0	0	0	0	0	0	0	0
0	0	0	1	1	0	1	0	0
0	0	0	0	0	0	0	1	0
0	0	0	0	0	0	0	0	0
0	0	0	0	0	0	0	0	0
0	0	0	0	1	0	0	1	0
1	0	0	0	0	0	0	0	0

0	0	0	0	0	0	0	0	0
0	1	0	0	0	0	0	1	0
0	0	0	0	0	0	0	0	0
1	0	0	0	1	0	0	0	0
0	0	0	0	0	0	0	0	0
0	0	0	0	0	0	0	0	0
0	0	0	0	0	0	0	1	1
0	1	0	0	0	0	0	0	0
0	0	0	0	0	0	0	0	0
0	0	0	0	1	0	0	0	1
0	0	0	0	0	0	0	0	0
0	0	0	0	0	0	0	0	0
0	0	0	0	0	0	0	0	0
0	0	0	0	0	0	0	0	0
0	0	0	0	0	0	0	0	0
0	0	0	0	0	0	0	0	0
0	0	0	0	0	0	0	0	0
0	0	0	0	0	0	0	0	0
1	0	0	0	1	0	0	0	0
1	0	0	0	0	0	0	0	0
1	0	0	0	0	0	0	0	0
0	0	0	0	0	0	0	0	0
0	0	0	0	0	0	0	0	0
0	0	0	0	1	0	0	0	0
0	0	0	0	0	0	0	1	0
0	0	0	0	0	0	0	0	0
0	0	0	0	0	0	0	0	0
0	0	0	0	0	0	0	0	0
0	1	0	1	0	0	0	0	1
0	0	0	0	0	0	0	0	0
0	0	0	0	0	0	0	0	0
0	0	0	0	0	0	0	0	0
1	0	0	0	0	0	0	0	0
0	0	0	0	0	0	0	0	0
0	0	0	0	1	0	0	0	0
0	0	0	0	0	0	0	0	0
0	0	0	0	0	0	0	0	0
0	0	0	0	0	0	0	0	0
0	0	0	0	0	0	0	1	1
0	0	0	0	0	0	0	0	0
0	1	0	0	0	0	0	0	0
0	0	0	0	0	0	0	0	0
0	0	0	0	0	0	0	0	0
1	0	0	0	0	0	0	0	0
0	0	0	0	1	0	0	0	0
0	0	0	0	0	0	0	0	0
0	0	0	0	0	0	0	0	0
0	0	0	0	0	0	0	1	0
0	0	0	0	0	0	0	0	0
0	0	0	0	1	1	0	0	0
1	0	0	0	0	0	0	1	0
1	0	0	0	0	0	0	1	0
0	0	0	0	0	0	0	0	0
0	0	0	0	0	0	0	0	0
1	1	0	0	0	0	0	0	0
0	0	0	0	0	0	0	0	0

1	1	0	0	0	0	1	0	0
0	0	0	0	0	0	0	0	0
0	0	0	0	0	0	0	0	0
0	0	0	0	0	0	0	1	0
0	0	0	0	0	0	0	0	0
1	0	0	0	0	0	0	1	0
0	0	0	0	0	0	0	0	0
1	0	0	1	0	0	0	0	0
1	0	0	0	0	0	0	0	0
0	0	0	0	0	0	0	0	0
0	0	0	0	0	0	0	1	0
0	0	0	0	1	0	0	1	0
1	0	0	1	0	0	0	0	1
1	0	0	0	1	0	0	0	1
0	1	0	0	0	0	0	0	0
1	0	0	0	1	0	0	0	0
1	0	0	0	0	0	0	0	0
1	0	0	0	0	0	0	0	0
1	0	0	0	0	0	0	0	0
0	0	0	0	0	0	0	0	0
1	0	0	0	0	0	0	0	0
0	0	0	0	0	0	0	0	0
0	0	0	0	0	0	0	0	0
1	0	0	0	0	0	0	0	0
1	0	0	0	0	0	0	0	0
0	1	0	0	0	0	0	0	0
0	0	0	1	0	0	0	0	0
1	1	0	0	0	0	0	0	0
1	0	0	0	0	0	0	0	0
0	0	0	0	0	0	0	0	0
0	0	0	0	0	0	1	0	0
1	0	0	0	0	0	0	0	0
0	0	0	0	0	0	0	0	0
0	0	0	0	0	0	0	0	0
1	0	0	0	0	0	0	0	0
1	0	0	0	1	0	0	0	0
1	1	0	0	0	0	0	1	0
0	0	0	0	0	0	0	0	0
0	1	0	0	0	0	0	0	0
0	0	0	0	0	0	0	0	0
1	0	0	0	0	0	0	0	0
1	0	0	0	0	0	0	1	0
0	0	0	0	0	0	0	0	0
1	1	0	0	0	0	0	0	0
1	0	0	0	0	0	0	0	0
1	0	0	0	0	0	0	0	0
1	1	0	0	1	0	0	0	0
1	0	0	0	0	0	0	0	0
1	0	0	0	1	0	0	0	1
0	0	0	0	0	0	1	0	0
0	0	0	0	0	0	0	0	0
0	0	0	0	0	0	0	0	0
1	0	0	0	0	0	0	1	0
0	0	0	0	0	0	0	0	0
0	0	0	0	0	0	0	0	0
0	0	0	0	0	0	0	0	0

0	0	0	0	0	0	0	0	0
1	0	0	0	0	0	0	0	0
0	0	0	0	0	0	0	0	0
1	0	0	0	0	0	0	0	0
1	0	0	0	1	1	0	1	0
0	0	0	0	0	0	0	0	1
1	0	0	0	1	0	0	0	0
1	0	0	0	0	0	0	0	0
0	0	0	0	0	0	0	0	0
1	0	0	0	0	0	0	0	0
1	0	0	0	0	0	0	0	0
1	0	0	0	0	0	0	0	0
0	0	0	1	0	0	0	0	0
1	0	0	0	0	0	0	0	0
0	0	0	0	0	0	0	0	0
1	0	0	0	0	0	1	0	0
1	0	0	0	0	0	0	0	0
1	0	0	0	0	0	0	0	0
1	0	0	0	1	0	0	0	0
1	0	0	0	0	0	0	0	0
1	1	0	0	0	0	0	1	0
0	0	0	0	0	0	0	0	0
1	0	0	0	0	0	0	0	0
0	0	0	0	0	0	0	0	0
0	0	0	0	0	0	0	0	0
0	1	0	0	0	0	0	0	0
0	0	0	0	0	0	0	0	0
0	0	0	0	0	0	0	0	0
0	1	0	0	0	0	0	0	0
0	1	0	0	0	0	0	0	0
1	1	0	0	0	0	0	1	0
0	0	0	0	0	0	0	0	0
1	0	0	0	0	0	0	0	0
1	0	0	0	0	0	0	1	0
1	0	0	0	0	0	0	0	0
1	0	0	0	0	0	0	0	0
1	0	0	0	0	0	0	0	0
1	0	0	0	0	0	0	0	0
1	1	1	0	0	0	0	1	0
0	0	0	0	0	0	0	0	0
0	1	0	0	0	0	1	0	0
0	0	0	0	0	0	0	0	0
1	0	0	0	1	0	0	0	1
0	0	0	0	0	0	0	0	0
0	0	0	0	0	0	0	0	0
0	0	0	0	0	0	0	0	0
0	0	0	0	0	0	0	0	0
0	0	0	0	0	0	0	0	1
1	0	0	0	0	0	0	0	0
1	0	0	0	0	0	0	0	0
1	1	0	0	0	0	0	1	0
1	0	0	0	0	0	1	0	0
1	0	0	0	0	0	0	0	0
0	0	0	0	0	0	0	0	1
1	0	0	0	1	0	0	0	0
0	0	0	0	0	0	0	0	0

1	0	0	0	0	0	0	0	0
0	0	0	0	0	0	0	0	1
0	0	0	0	0	0	0	1	0
0	0	0	0	0	0	0	1	0
0	0	0	0	0	0	0	0	0
1	0	0	0	1	0	0	0	0
0	1	0	0	0	0	0	0	0
1	0	0	0	0	0	0	0	0
1	0	0	0	0	0	0	1	0
0	0	0	0	0	0	0	0	0
1	0	0	0	0	0	0	0	0
1	0	0	1	0	0	0	0	0
1	0	0	1	0	0	0	0	0
0	0	0	0	0	0	0	0	0
0	0	0	0	0	0	0	0	0
0	0	0	0	0	0	0	0	0
1	0	0	0	0	0	0	0	0
1	0	0	0	0	0	0	0	0
1	0	0	0	0	0	0	0	0
0	0	0	0	0	0	0	1	0
1	0	0	0	0	0	0	0	0
1	0	0	0	0	0	0	1	0
0	0	0	0	0	0	0	0	0
1	0	0	0	0	0	0	0	0
1	0	0	0	0	0	0	0	0
1	1	0	0	0	0	0	0	0
0	1	1	0	0	0	0	0	0
0	1	0	0	0	0	0	0	0
1	0	0	0	0	0	0	0	0
0	0	0	0	0	0	0	0	0
1	0	0	0	0	0	0	0	0
1	0	0	0	0	0	0	0	0
0	0	0	0	0	0	0	1	0
0	0	0	0	0	0	0	0	0
0	0	0	0	0	0	0	0	0
1	0	0	0	0	0	0	0	0
1	1	0	0	0	0	0	0	0
0	0	0	0	0	0	0	0	0
0	0	0	0	0	1	0	1	0
0	1	0	0	0	0	0	0	0
1	0	0	0	0	0	0	0	0
0	0	0	0	0	0	0	0	0
0	0	0	0	0	0	0	1	0
1	0	0	0	0	0	0	1	0
0	0	0	0	0	0	0	0	0
1	0	0	0	0	0	0	0	0
0	0	0	0	0	0	0	0	0
0	0	0	0	0	0	0	0	0
0	0	0	0	0	0	0	1	0
1	0	0	0	0	0	0	0	1
0	0	0	0	0	0	0	0	0
0	0	0	0	1	0	0	0	0
1	0	0	0	0	0	0	0	0
0	0	0	0	0	0	0	0	0
0	0	0	0	0	0	0	0	0
0	0	0	0	0	0	0	0	0

0	0	0	0	1	0	0	0	0
0	0	0	0	0	0	0	0	0
0	0	0	0	1	0	0	0	0
0	0	1	0	1	0	0	0	0
1	1	0	0	0	0	0	0	0
0	0	0	0	0	0	0	0	0
0	0	0	0	0	0	0	0	0
0	0	0	0	0	0	0	0	0
0	0	0	0	0	0	0	0	1
0	0	0	0	1	0	0	0	0
1	1	0	0	1	0	0	0	1
0	0	0	0	0	0	0	0	0
0	0	0	0	0	0	0	0	0
0	0	0	1	0	0	1	0	0
0	0	0	0	0	0	0	0	0
0	0	0	0	0	0	0	0	0
0	0	0	0	0	0	0	0	0
0	0	0	0	0	0	0	0	0
0	0	0	0	0	0	0	0	0
0	0	0	0	0	0	0	0	0
1	0	0	0	0	0	0	0	0
0	0	0	0	0	0	0	0	0
0	0	0	0	0	0	0	0	0
0	0	0	0	1	1	0	0	0
0	1	0	1	0	0	0	0	0
1	0	0	0	1	0	0	0	0
1	0	0	0	0	0	0	0	0
0	0	0	0	0	0	0	0	0
1	0	0	0	0	0	0	0	0
1	0	0	0	0	0	0	0	0
1	0	0	0	0	0	0	0	0
1	0	0	0	0	0	0	0	0
0	0	0	0	0	0	0	1	0
0	0	0	0	0	0	0	0	0
1	0	0	0	0	0	0	0	0
0	0	0	0	0	0	0	0	0
1	0	0	0	0	0	0	0	0
0	0	0	0	0	0	0	0	0
0	1	0	0	0	0	0	0	0
0	0	0	0	0	0	0	0	0
1	0	0	0	1	0	0	0	0
0	0	1	0	0	0	1	0	0
0	0	0	0	0	0	0	1	0
1	0	0	0	1	0	0	0	0
0	0	0	0	0	0	0	0	0
0	0	0	0	0	0	1	0	0
0	0	0	0	0	0	1	0	0
0	0	0	0	0	0	0	0	0
1	0	0	0	0	0	0	0	0
0	0	0	0	0	0	0	0	0
1	0	0	1	0	0	0	0	0
0	0	0	0	0	0	0	0	0
0	0	0	0	0	0	0	0	0
1	0	0	0	0	0	0	0	0
0	0	0	0	0	0	0	0	0
0	0	0	0	0	0	0	0	0

1	1	0	0	0	0	0	0	0
1	0	0	0	0	0	0	0	0
0	0	0	0	0	0	0	0	0
1	0	0	0	0	0	0	0	0
0	1	0	0	0	0	0	0	0
1	0	0	0	0	0	0	0	0
0	0	0	0	0	0	0	0	0
0	1	0	0	0	0	0	0	0
0	0	0	0	0	0	0	0	0
0	0	0	0	0	0	0	0	0
0	0	0	0	0	0	0	0	0
0	0	0	0	0	0	0	0	0
1	0	0	0	1	0	0	0	0
0	0	0	0	0	0	0	0	0
0	1	1	0	0	0	0	0	1
0	0	0	0	1	0	0	0	0
1	0	0	0	0	0	0	0	0
0	1	0	0	0	0	0	1	0
1	0	0	0	0	0	0	0	0
0	0	0	0	0	0	0	0	0
1	0	0	0	0	0	0	0	0
1	0	0	0	1	0	0	0	0
0	1	0	0	0	0	0	0	0
0	0	0	0	0	0	0	0	0
0	0	0	0	0	0	0	0	0
0	0	0	0	0	0	0	0	0
0	0	0	0	0	0	0	0	0
0	0	0	0	1	1	0	0	0
1	0	0	0	0	0	1	0	0
0	0	0	0	0	0	0	0	0
1	0	0	0	0	0	0	0	0
1	1	0	0	0	0	0	0	0
1	0	0	0	0	0	0	0	0
0	0	0	0	0	0	0	1	0
0	0	0	0	0	0	0	0	0
1	0	0	0	0	0	0	0	0
0	0	0	0	0	0	0	0	0
0	0	0	0	0	0	0	0	0
1	0	0	0	0	0	0	0	0
0	0	0	0	0	0	0	1	0
1	0	0	0	0	0	0	0	0
0	0	0	0	0	0	0	0	0
0	0	0	0	0	0	0	0	0
0	0	0	0	0	0	0	0	0
1	0	0	0	0	0	0	0	0
0	0	0	0	0	0	0	1	0
1	0	0	0	0	0	0	0	0
1	0	0	0	0	0	0	0	0
0	0	0	0	0	0	0	0	0
0	0	0	0	0	0	0	0	0
0	1	0	0	0	0	0	0	0
0	0	0	0	0	0	0	0	0
1	0	0	0	0	0	0	0	0
0	0	0	0	0	0	0	0	0
1	0	0	0	0	0	0	0	0
1	0	0	0	0	0	0	0	0

1	0	0	0	0	0	0	0	0
1	0	0	0	0	0	0	0	0
1	0	0	0	0	0	0	0	0
0	0	0	0	0	0	0	0	0
1	0	0	0	0	0	0	0	0
0	0	0	0	0	0	0	0	0
0	0	0	1	0	0	0	1	0
1	0	0	0	0	0	0	0	0
0	0	0	0	0	0	1	0	0
0	0	0	0	0	0	0	0	1
0	0	0	0	0	0	0	0	0
1	0	0	0	0	0	0	0	0
1	0	0	0	0	0	0	1	1
1	0	0	0	0	0	0	1	0
1	0	0	0	0	0	0	0	0
0	1	1	0	0	0	0	0	0
0	0	0	0	1	0	0	0	0
1	0	0	0	0	0	0	0	0
1	0	0	0	0	0	0	0	0
0	0	0	0	0	0	0	1	0
1	0	0	0	0	0	0	0	0
0	0	0	0	0	0	0	1	0
0	0	0	0	0	0	0	0	0
1	0	0	0	0	0	0	0	0
0	0	0	0	0	0	0	0	0
0	0	0	0	0	0	0	0	0
1	0	0	0	0	0	1	0	0
1	0	0	0	0	0	0	0	0
0	0	0	0	0	0	0	0	0
1	0	0	0	0	0	1	0	0
0	0	0	0	0	0	0	0	0
0	0	0	0	0	0	0	0	0
0	0	0	0	0	0	0	0	0
0	0	0	0	0	0	0	0	0
0	0	0	0	0	0	0	0	0
0	0	0	0	1	0	0	0	0
0	0	0	0	0	0	0	0	0
0	0	0	0	0	0	0	0	0
0	0	0	0	0	0	0	1	0
0	1	0	0	0	0	0	0	0
0	0	0	0	0	0	0	0	0
0	0	0	0	0	0	0	0	0
0	0	0	0	0	0	0	0	0
1	0	0	0	0	0	0	0	0
0	0	0	0	0	0	0	0	0
1	0	0	0	0	0	0	0	0
0	0	0	0	0	0	0	0	0
0	0	0	0	0	0	0	0	0
0	0	0	0	0	0	0	0	0
1	0	0	0	0	0	0	0	0
0	0	0	0	0	0	0	0	0
1	1	0	0	0	0	0	0	0
0	0	0	0	1	0	0	0	0
0	0	0	0	0	0	0	0	0
0	0	0	0	0	0	0	0	0
1	0	0	0	1	0	0	0	0

0	0	0	0	0	0	0	0	0
1	0	1	0	0	0	0	0	0
0	0	0	0	0	0	0	0	0
1	0	0	0	0	0	0	0	0
0	0	0	0	0	0	0	1	0
0	0	0	0	0	0	0	0	0
0	0	0	0	0	0	1	0	0
0	1	0	0	0	0	0	0	0
1	0	0	0	0	0	0	0	1
0	0	0	0	0	0	0	0	0
0	0	0	0	0	0	0	0	0
1	0	1	0	0	0	0	0	0
1	0	0	0	0	0	0	0	0
0	0	0	0	0	0	0	0	0
1	0	0	0	0	0	0	1	0
0	0	0	0	0	0	0	0	0
0	0	0	0	0	0	0	1	0
0	0	0	0	0	0	0	0	0
1	1	0	0	0	0	0	0	0
0	0	0	0	0	0	0	0	0
1	0	0	0	0	0	0	0	0
1	0	1	0	0	0	0	1	0
0	0	0	0	0	0	0	0	0
0	0	0	0	0	0	0	0	1
1	0	0	0	0	0	0	0	0
0	0	0	0	0	0	0	0	0
0	0	0	0	0	0	0	0	0
0	0	0	0	0	0	0	0	0
0	0	0	0	0	0	0	0	0
0	0	0	0	0	0	0	0	0
1	0	0	0	0	0	0	0	0
0	0	0	0	0	0	0	0	0
0	1	0	0	0	0	0	0	0
1	0	0	0	0	0	1	0	0
0	0	0	0	0	0	0	0	0
0	0	0	0	0	0	0	0	0
0	0	0	0	0	0	0	0	0
0	0	0	0	0	0	0	0	0
0	0	0	0	0	0	0	0	0
1	0	0	0	0	0	0	0	0
0	0	0	0	0	0	0	0	0
0	1	1	0	0	0	0	0	1
0	0	0	0	0	0	0	0	0
0	0	0	0	0	0	0	0	0
0	0	0	0	0	0	0	0	0
1	0	0	0	0	0	0	0	0
0	0	0	0	0	0	0	1	0
1	0	0	0	0	0	0	0	0
0	0	0	0	0	0	0	0	0
1	0	0	0	0	0	0	0	0
0	0	0	0	0	0	0	0	0
0	0	0	0	0	0	0	0	0
0	0	0	0	0	0	0	0	0
0	0	0	0	0	0	0	0	0
0	0	0	0	0	0	0	0	0
0	0	0	0	0	0	1	0	0

0	0	0	0	0	0	0	0	0
1	0	0	0	1	0	0	0	0
0	0	0	0	0	0	0	0	0
1	0	0	0	0	0	0	1	0
1	0	0	0	0	0	0	0	0
0	0	0	0	0	0	0	0	0
1	0	0	0	0	0	0	0	0
1	0	0	0	0	0	0	0	0
1	0	0	0	0	0	0	0	0
0	0	0	0	0	0	0	0	0
0	0	0	0	0	0	0	0	0
1	1	0	0	0	0	0	0	0
0	0	0	0	0	0	0	0	0
1	0	1	0	0	0	0	0	0
0	0	0	0	0	0	0	0	0
0	0	0	0	0	0	0	0	0
0	0	0	0	1	0	0	0	0
1	0	0	0	0	1	0	0	0
0	0	0	0	0	0	0	0	0
0	0	0	0	0	0	0	0	0
0	0	0	0	0	0	0	0	0
0	0	0	0	0	0	1	0	0
0	0	0	0	0	0	0	0	0
1	0	0	0	0	0	0	0	0
1	0	1	0	0	0	0	0	0
1	0	0	0	0	0	0	0	0
1	1	0	0	0	0	0	0	0
0	0	0	0	0	0	0	0	0
1	0	0	0	1	0	0	0	0
0	0	0	0	0	0	0	0	0
1	0	0	0	0	0	0	0	0
0	0	0	0	0	0	0	0	0
1	0	0	0	0	0	0	0	0
0	0	0	0	1	0	0	0	0
0	0	0	0	0	0	0	0	0
0	0	0	0	0	0	0	0	0
0	0	0	0	0	0	0	1	0
1	0	0	0	0	0	0	0	0
1	0	0	0	0	0	0	0	0
0	0	0	0	0	0	0	0	0
1	0	1	0	0	0	0	0	0
1	0	0	0	0	0	0	0	0
1	0	0	0	0	0	0	0	0
0	0	0	0	0	0	0	0	0
0	0	0	0	0	0	0	0	0
0	0	0	0	0	0	0	0	0
0	0	0	0	0	0	0	1	0
1	0	0	0	0	0	0	0	0
0	0	0	0	1	0	0	0	0
0	0	0	0	0	0	0	0	0
0	0	0	0	0	0	0	0	0
0	0	0	0	0	0	0	0	0
1	0	0	0	0	0	0	0	0
0	0	0	0	0	0	0	0	0
1	0	0	0	0	0	0	0	0
0	0	0	0	0	0	0	0	0

0	0	0	0	0	0	0	0	0
1	0	1	0	0	0	0	0	0
0	0	0	0	1	0	0	0	0
0	0	0	0	0	0	0	0	0
0	0	0	0	0	0	0	0	0
0	0	0	0	0	0	0	0	0
0	0	0	0	0	0	0	0	0
1	1	0	0	0	0	0	0	0
0	0	0	1	0	0	0	0	0
0	1	0	0	0	0	0	0	0
0	0	0	0	0	0	0	0	0
0	0	0	0	0	0	0	0	0
0	0	0	0	0	0	0	0	0
0	0	0	0	0	0	0	0	0
0	0	0	0	0	0	0	0	0
0	0	0	0	0	0	0	0	0
1	1	0	0	0	0	0	0	0
1	0	0	0	1	0	0	0	0
0	0	0	0	0	0	0	0	0
0	0	0	0	0	0	0	0	0
1	0	0	0	0	0	0	0	0
0	0	0	0	0	0	0	0	0
1	0	0	0	0	0	0	0	0
1	0	0	0	0	0	0	0	0
0	1	0	0	0	0	0	0	0
1	0	0	0	0	0	0	0	0
0	0	0	1	0	0	0	0	0
0	0	0	0	0	0	0	0	0
0	0	0	0	0	0	0	0	0
1	0	0	0	0	0	0	0	0
0	0	0	0	0	0	0	0	0
0	1	0	0	0	0	0	0	0
0	0	0	0	0	0	0	1	0
1	0	0	0	0	0	0	0	0
0	0	0	0	0	0	0	1	0
0	0	0	0	1	1	0	0	0
0	0	0	0	0	0	0	0	0
1	0	0	0	0	0	0	1	0
1	0	0	0	0	0	0	0	0
0	0	0	1	0	0	0	0	0
0	0	0	0	0	0	0	0	0
0	0	0	0	0	0	0	0	0
1	0	0	0	0	0	0	0	0
0	0	0	0	0	0	0	0	0
0	0	0	0	0	0	0	0	0
1	0	0	0	0	0	0	0	0
0	0	0	0	0	0	0	0	0
0	0	0	0	0	0	0	0	0
0	0	0	0	0	0	0	0	0
0	0	0	0	0	0	0	0	0
1	0	0	0	0	0	0	0	0
0	0	0	0	0	0	0	1	0
0	0	0	0	0	0	0	0	0
0	0	0	0	0	0	0	0	0
0	0	0	0	0	0	0	0	0
0	0	0	0	0	0	0	0	0

1	0	0	0	0	0	0	0	0
0	0	0	0	0	0	0	0	0
1	0	0	0	0	0	0	0	0
1	0	0	0	0	0	0	0	0
0	0	0	0	0	0	0	0	0
1	0	0	0	0	0	0	0	0
1	0	0	0	0	0	0	1	0
0	0	0	0	0	0	0	0	0
0	0	0	0	0	0	0	0	0
0	0	0	0	0	1	0	0	0
0	0	0	0	0	0	0	0	0
1	0	0	0	0	0	0	0	0
0	0	0	0	0	0	0	0	0
0	0	0	1	0	0	0	0	0
0	0	0	0	0	0	0	0	0
0	0	0	0	0	0	0	0	0
0	0	0	0	0	0	0	0	0
0	0	0	0	0	0	0	0	0
1	0	0	0	0	0	0	0	0
0	0	0	0	0	0	0	0	0
0	0	0	0	0	0	0	0	0
0	0	0	0	0	0	0	0	0
1	0	0	0	0	0	0	0	0
0	0	0	0	1	0	0	0	0
0	0	0	0	0	0	0	0	0
0	0	0	1	0	0	1	0	0
0	0	0	0	0	0	0	0	0
1	0	0	0	0	0	0	0	0
0	0	0	0	0	0	1	0	0
0	0	0	0	0	0	0	0	0
1	1	0	0	0	0	0	0	0
0	0	0	0	0	0	0	0	0
1	0	0	0	0	0	0	0	0
0	0	0	0	0	0	0	0	0
0	0	0	0	0	0	0	0	0
0	1	0	0	0	0	0	0	0
0	0	0	0	0	0	0	0	0
1	0	0	0	1	0	0	0	0
1	0	1	0	0	0	0	0	0
0	0	0	0	0	0	0	0	0
0	0	0	0	0	0	0	0	0
0	0	0	0	0	0	0	0	0
1	0	0	0	0	0	0	0	0
0	0	0	0	0	0	0	0	0
0	1	0	0	0	0	0	0	0
0	0	0	0	0	0	0	0	0
0	0	0	0	0	0	0	0	0
0	0	0	0	0	0	0	0	0
0	0	0	0	0	0	0	1	0
0	0	0	0	0	0	0	0	0
0	0	0	0	0	0	0	0	0
1	0	0	0	0	0	0	0	0
0	1	0	0	0	0	0	0	0
1	0	0	0	0	0	0	0	0
0	0	0	0	0	0	0	0	0
0	0	0	0	0	0	0	0	0

0	0	0	0	0	0	0	0	0
0	0	0	0	0	0	0	0	0
0	0	0	0	0	0	0	0	0
0	0	0	0	0	0	0	0	0
0	0	0	0	0	0	0	0	0
0	0	0	0	0	0	0	1	0
0	0	0	0	0	0	1	0	0
0	0	0	0	0	0	0	0	0
0	0	0	0	0	0	0	0	0
0	0	0	0	0	0	0	0	0
0	0	0	0	0	0	0	0	0
1	0	0	0	0	0	0	0	0
0	0	0	0	0	1	0	0	0
1	0	0	0	0	0	0	1	0
0	0	0	0	0	0	0	0	0
0	0	0	1	0	0	0	0	0
0	0	0	0	0	0	0	0	0
0	0	0	0	0	0	0	0	0
0	0	0	0	0	0	0	0	0
0	0	0	0	0	0	0	0	0
1	0	0	0	0	0	0	0	0
0	0	0	1	0	0	0	0	0
0	0	0	0	0	0	0	0	0
0	0	0	0	0	0	0	0	0
0	0	0	0	0	0	0	0	0
1	0	0	0	0	0	0	0	0
0	0	0	0	0	0	0	0	0
0	0	0	0	0	0	1	0	0
0	0	0	0	0	0	0	0	0
0	0	0	0	0	0	0	0	0
1	0	0	0	0	0	0	0	0
0	0	0	0	0	0	0	0	0
0	0	0	0	0	0	0	0	0
1	0	0	0	0	0	0	0	1
0	0	0	0	0	0	0	0	0
0	1	0	0	0	0	0	0	0
1	0	0	0	0	0	0	0	0
1	0	0	0	0	0	0	0	0
0	0	0	0	0	0	0	0	0
0	0	0	0	0	0	0	0	0
1	0	0	0	0	0	0	0	0
0	0	0	0	0	0	0	0	0
0	0	0	0	0	0	0	0	0
0	0	0	0	0	0	0	0	0
1	0	0	0	0	0	0	0	0
0	0	0	0	0	0	0	1	0
0	0	0	0	0	0	0	0	0
1	0	0	0	0	0	0	0	0
0	0	0	0	0	0	0	0	0
0	0	0	0	0	0	0	0	0
0	0	0	0	0	0	0	0	0
0	0	0	0	0	0	0	0	0
0	0	0	0	0	0	0	0	1
0	0	0	0	0	0	0	0	0
1	0	0	0	0	0	0	1	0
0	0	0	0	0	0	0	0	0

0	0	0	0	0	0	0	0	0
1	0	0	0	0	0	0	0	0
0	0	0	0	0	0	0	0	0
0	0	0	0	0	0	0	0	0
1	0	0	0	0	0	0	0	0
0	0	0	0	0	0	0	1	0
0	0	0	0	0	0	0	0	0
0	0	0	0	0	0	0	1	0
0	0	0	0	0	0	0	0	0
0	0	0	0	0	0	0	0	0
0	0	0	0	0	0	0	1	0
0	0	0	0	0	0	0	0	0
1	0	0	0	0	0	0	1	0
0	0	0	0	0	0	0	0	0
0	0	0	0	0	0	0	0	0
1	0	0	0	0	0	0	0	0
0	0	0	0	0	0	0	0	0
0	0	0	0	0	0	0	0	0
1	0	0	0	0	0	0	0	0
0	0	0	0	0	0	0	0	0
0	0	0	0	0	0	0	0	0
0	0	0	0	0	0	0	0	0
1	0	0	0	0	0	0	0	0
0	0	0	0	0	0	0	0	0
1	0	0	0	0	0	0	0	0
0	0	0	0	0	0	0	1	0
0	0	0	0	0	0	0	0	0
0	0	0	0	0	0	0	0	0
0	0	0	0	0	0	0	0	0
0	0	0	0	0	0	0	0	0
0	0	0	0	0	0	0	1	0
1	0	0	0	0	0	0	0	0
1	0	0	0	0	0	0	0	0
0	0	0	0	0	0	0	0	0
0	0	0	0	0	0	0	0	0
0	0	0	0	0	0	0	1	0
0	0	0	0	0	0	0	0	0
0	0	0	0	1	0	0	0	0
0	0	0	0	0	0	0	0	0
0	0	0	0	0	0	0	0	0
0	0	0	0	0	0	0	0	0
0	0	0	0	0	0	0	0	0
0	1	0	0	0	0	0	0	0
0	0	0	0	0	0	0	0	0
0	0	0	0	0	0	0	0	0
0	0	0	0	0	0	0	0	0
0	0	0	0	0	0	0	0	0
0	0	0	0	0	0	0	0	0
0	0	0	0	0	0	0	0	0
0	0	0	0	0	0	0	0	0
0	0	0	0	0	0	0	0	0
0	0	0	0	0	0	0	0	0
0	0	0	0	0	0	0	0	0
1	0	0	0	0	0	0	0	0
1	0	0	0	0	0	0	0	0
0	0	0	0	0	0	0	1	0

1	0	0	0	0	0	0	0	0
0	0	0	0	0	0	0	0	0
0	0	0	0	0	0	0	0	0
0	0	0	1	0	0	0	0	0
0	0	0	0	0	0	0	1	0
1	0	1	0	0	0	0	0	0
0	0	0	0	0	0	0	0	0
0	0	0	0	0	0	0	0	0
0	0	0	0	0	0	0	0	0
1	0	0	0	0	0	0	0	0
0	0	0	0	0	0	0	0	0
0	0	0	0	0	0	0	0	0
0	0	0	0	0	0	0	0	0
0	0	0	0	0	0	0	0	0
0	0	0	0	0	0	0	0	0
0	0	0	0	1	0	0	0	0
1	0	0	0	0	0	0	0	0
0	0	0	0	0	0	0	0	0
0	0	0	0	0	0	0	0	0
1	0	0	0	0	0	1	0	0
0	0	0	0	0	0	0	1	0
0	0	0	0	0	0	0	1	0
0	0	0	0	0	0	0	0	0
1	0	0	0	0	0	0	1	0
0	1	0	0	1	0	0	0	0
0	0	0	0	0	0	0	1	0
1	0	0	0	0	0	0	0	0
0	0	0	0	0	0	0	0	0
0	1	0	0	0	0	0	0	0
0	0	0	0	0	0	0	1	0
1	0	0	0	0	0	0	0	0
0	0	0	0	0	0	0	1	0
0	0	0	0	0	0	0	0	0
0	0	0	0	0	0	0	0	0
0	0	0	0	0	0	0	0	0
0	0	0	0	0	0	0	0	0
1	0	0	0	0	0	0	0	0
0	0	0	0	0	0	0	0	0
0	0	0	0	0	0	0	0	0
0	0	0	0	0	0	1	0	0
0	0	0	0	0	0	0	0	0
0	0	0	0	0	0	0	0	0
0	0	0	0	0	0	0	0	0
1	0	0	0	0	0	0	0	0
0	0	0	0	0	0	0	0	0
0	0	0	0	0	0	0	0	0
1	0	0	0	0	0	0	0	0
0	0	0	0	0	0	0	0	0
0	0	0	0	1	0	0	0	0
1	0	0	0	0	0	0	0	0
1	0	0	0	0	0	0	0	0
0	0	0	0	0	0	0	0	0
0	0	0	0	0	0	0	0	0
1	0	0	0	0	0	0	0	0
0	0	0	0	0	0	0	1	0
1	0	0	0	0	0	0	1	0

0	0	0	0	0	0	0	0	0
1	0	0	0	0	0	0	0	0
0	0	0	0	0	0	0	0	0
1	0	0	0	0	0	0	0	0
1	0	0	0	0	0	0	0	0
1	0	0	0	0	0	0	0	0
1	0	0	0	0	0	0	0	0
1	0	0	0	0	0	0	0	0
0	0	0	0	0	0	0	1	0
1	0	0	0	0	0	0	0	0
0	0	0	0	0	0	0	0	0
0	0	0	0	0	0	0	0	0
0	0	0	0	0	0	0	0	0
0	1	0	0	0	0	0	0	0
1	0	0	0	0	0	0	0	0
1	0	0	0	0	0	0	0	0
0	0	0	0	0	0	0	0	0
1	0	0	0	0	0	0	0	0
0	0	0	0	0	0	0	0	0
0	0	0	0	0	0	0	0	0
0	0	0	0	0	0	0	0	0
0	0	0	0	0	0	0	0	0
1	0	0	0	0	0	0	0	0
0	0	0	0	0	0	0	0	0
0	0	0	0	0	0	0	0	0
0	0	0	0	0	0	0	0	0
0	0	0	0	0	0	0	0	1
0	0	0	0	0	0	0	1	0
0	0	0	0	0	0	0	0	0
0	0	0	0	0	0	0	0	0
0	0	0	0	0	0	0	0	0
0	0	0	0	0	0	0	0	0
0	0	0	0	0	0	0	0	0
1	0	0	0	0	0	0	0	0
0	0	0	0	0	0	0	0	0
0	0	1	0	0	0	0	0	1
0	0	0	0	0	0	0	0	0
0	0	0	0	0	0	0	0	0
1	0	0	0	0	0	0	1	0
1	0	0	0	0	0	0	0	0
1	0	0	0	0	0	0	0	0
0	0	0	0	0	0	0	0	0
0	0	0	0	0	0	0	0	0
0	0	0	0	0	0	0	0	0
1	0	0	0	0	0	0	0	0
1	0	0	0	0	0	0	0	0
0	0	0	0	0	0	0	0	0
1	0	0	0	0	0	0	0	0
1	0	0	0	0	0	0	0	0
1	0	0	0	0	0	0	0	0
0	0	0	0	0	0	0	0	0
0	0	0	0	0	0	0	0	0
0	0	0	0	0	0	1	0	0
0	0	0	0	0	0	0	0	0
0	0	0	0	0	0	1	1	0
0	1	0	0	0	0	0	0	0

1	0	0	0	0	0	0	0	0
0	0	0	0	0	0	0	0	0
0	0	0	0	0	0	0	0	0
0	0	0	0	0	0	0	0	0
0	0	0	0	0	0	0	0	0
1	0	0	0	0	0	0	0	0
0	0	0	0	0	0	0	0	0
1	0	0	0	0	0	0	0	0
0	0	0	0	0	0	0	0	0
0	0	0	0	0	0	0	0	0
0	0	0	0	0	0	0	0	0
0	0	0	0	0	0	0	0	0
0	0	0	0	0	0	0	0	0
0	0	0	0	0	0	0	0	0
0	0	0	0	0	1	0	0	0
0	0	0	0	0	0	0	0	0
0	0	0	0	0	0	0	0	0
0	0	0	0	0	0	0	1	0
1	0	0	0	0	0	0	0	0
1	0	0	0	0	0	0	0	0
1	0	0	0	0	0	0	0	0
1	0	0	0	0	0	0	0	0
0	0	0	0	0	0	0	0	0
1	0	1	0	0	0	0	0	0
0	0	0	0	1	0	0	0	0
0	0	0	0	0	0	0	1	0
1	0	0	0	0	0	0	0	0
0	0	0	0	0	0	1	0	0
0	0	0	0	0	0	1	0	0
1	0	0	0	0	0	0	0	0
0	0	0	0	1	0	0	0	0
0	0	0	0	0	0	0	0	0
0	0	0	0	0	0	0	0	0
1	0	0	0	0	0	0	0	0
0	1	0	0	0	0	0	0	0
0	0	0	0	0	0	0	0	0
0	0	0	0	0	0	0	0	0
0	0	0	0	0	0	0	0	0
1	0	0	0	0	0	0	0	0
0	0	0	0	0	0	0	0	0
0	0	0	0	0	0	0	0	0
1	0	0	0	0	0	0	0	0
1	0	0	0	0	0	0	0	0
0	0	0	0	0	0	0	1	0
1	0	1	0	0	0	0	0	0
0	1	0	0	0	0	1	0	0
1	0	0	0	0	0	0	0	0
0	0	0	0	0	0	0	0	0
0	0	0	0	0	0	1	1	0
1	0	0	0	0	0	0	0	0
0	0	0	0	1	0	1	0	0
0	0	0	0	0	0	0	0	0
0	0	0	0	0	0	0	0	0
0	0	0	0	0	0	0	0	0
0	0	0	0	0	0	0	0	0
0	0	0	0	0	0	0	0	0

0	0	0	0	0	0	0	0	0
0	0	0	0	0	0	0	0	0
1	0	0	1	0	0	0	0	0
0	0	0	0	0	0	0	0	0
0	0	0	0	0	0	0	0	0
1	0	0	0	0	0	0	0	0
1	0	0	0	0	0	0	0	0
1	0	0	0	0	0	0	0	0
0	0	0	0	0	0	0	0	0
1	0	0	0	0	0	0	0	0
0	0	0	0	0	0	0	0	0
1	0	0	0	0	0	0	0	0
0	0	0	0	0	0	0	1	0
0	0	0	0	0	0	0	0	0
1	0	0	0	0	0	0	0	0
0	0	0	0	0	0	0	1	0
0	0	0	0	0	0	0	0	0
0	0	0	0	0	1	0	0	0
0	0	0	0	0	0	0	0	0
1	0	0	0	0	0	0	1	0
1	0	0	0	0	0	0	0	0
0	0	0	0	0	0	0	0	0
0	0	0	0	0	0	1	0	0
1	0	0	0	0	0	0	0	0
0	0	0	0	0	0	0	1	0
0	0	0	0	0	0	0	0	0
0	0	0	0	0	0	0	0	0
1	0	0	0	0	0	0	0	0
1	0	0	0	0	0	0	0	0
1	0	0	0	0	0	0	0	0
0	0	0	0	0	0	0	0	0
0	0	0	0	0	0	0	0	0
0	0	0	0	0	0	0	0	0
0	0	0	0	0	0	0	0	0
1	0	0	0	0	0	0	0	0
0	0	0	0	0	0	0	0	0
1	0	1	0	0	0	0	1	0
0	0	0	0	0	0	0	0	0
0	0	0	0	0	0	0	0	0
0	0	0	0	0	0	0	0	0
0	0	0	0	0	0	0	0	0
0	0	0	0	0	0	0	0	0
1	0	0	0	0	0	0	0	0
0	0	1	0	0	0	0	0	0
0	0	0	0	0	0	0	0	0
0	0	0	0	0	0	0	0	0
0	0	0	0	0	0	0	0	0
0	0	0	0	0	0	1	0	0
0	0	0	0	0	0	1	0	0
0	0	0	0	0	0	0	0	0
0	0	0	0	0	0	0	0	0
0	0	0	0	0	0	0	0	0
0	0	1	0	0	0	0	0	0
0	0	0	0	0	0	0	0	0
0	0	0	0	0	0	0	0	0
0	1	0	0	0	0	0	0	0

0	1	0	0	0	0	1	0	0
0	0	0	0	0	0	0	0	0
1	0	0	0	0	0	0	0	0
0	0	0	0	0	0	0	0	0
1	0	0	0	0	0	0	0	0
0	0	0	0	0	0	0	0	0
1	0	0	0	0	0	0	0	0
0	0	0	0	0	0	0	0	0
0	0	0	0	0	0	0	0	0
0	0	0	0	0	0	0	0	0
1	0	0	0	0	0	0	0	0
0	0	0	0	0	0	0	0	0
0	0	0	0	0	0	0	0	0
0	0	0	0	0	0	0	0	1
0	0	0	0	0	0	1	0	0
0	0	0	0	0	0	0	0	0
0	0	0	0	0	0	0	0	0
0	0	0	0	0	0	0	0	0
0	0	0	0	0	0	0	0	0
0	0	0	0	0	0	1	0	0
0	0	0	0	0	0	0	0	0
1	0	0	0	1	0	0	0	0
0	0	0	0	0	0	0	0	0
1	0	0	0	0	0	0	0	0
1	0	0	0	0	0	0	0	0
1	0	0	0	0	0	0	0	0
1	0	0	0	0	0	0	0	0
0	0	0	0	0	0	0	0	0
1	0	0	0	0	0	1	0	0
0	0	0	1	0	0	0	0	0
0	0	0	0	0	0	0	0	0
0	0	0	0	0	0	0	0	0
1	0	0	0	0	0	0	0	0
0	0	0	0	0	0	0	0	0
1	0	0	0	0	0	0	0	0
0	0	0	0	0	0	0	0	0
0	0	0	0	1	0	0	0	0
1	0	0	0	0	0	0	0	0
0	0	0	0	0	0	0	0	0
0	0	0	0	1	0	0	0	0
0	0	0	0	0	0	0	0	0
1	0	1	0	0	0	0	1	0
1	0	0	0	0	0	0	0	0
0	0	0	0	0	0	0	0	0
0	0	0	0	0	0	0	0	0
1	1	0	0	0	0	0	0	0
0	0	0	0	0	0	0	0	0
1	0	0	0	0	0	0	1	0
0	0	0	0	0	0	0	0	0
0	0	0	0	0	0	0	0	0
0	0	0	0	0	0	0	0	0
0	0	0	0	0	0	0	0	0
0	0	0	0	0	0	0	0	0
0	0	0	0	0	0	0	0	0
0	1	0	0	0	0	0	0	0
0	0	0	0	0	0	0	0	0

0	0	0	0	0	0	0	0	0
0	0	0	0	0	0	0	0	0
0	0	0	0	1	0	0	0	0
1	0	0	0	0	0	0	0	0
0	0	0	1	0	0	0	0	0
0	0	0	0	1	0	0	1	0
0	0	0	0	0	0	0	0	0
0	0	0	0	0	0	0	1	0
0	0	0	0	0	0	0	1	0
0	0	0	0	0	0	0	1	0
0	1	0	0	0	0	0	0	0
0	0	0	0	0	0	0	0	0
0	0	0	0	0	0	0	0	0
0	0	1	0	0	0	0	0	1
0	0	0	0	0	0	0	0	0
0	0	0	0	0	0	0	0	0
1	0	0	0	0	0	1	0	0
0	0	0	0	0	0	0	1	0
0	0	0	0	0	0	0	0	0
0	0	0	0	0	0	0	0	0
0	0	0	0	0	0	0	0	0
0	1	0	0	0	0	0	0	0
0	1	0	0	0	0	1	0	0
1	0	0	0	0	0	0	0	0
1	0	0	0	0	0	0	0	0
0	0	0	0	0	0	0	0	0
0	0	0	0	0	0	0	0	0
0	0	0	0	0	0	0	0	0
0	0	0	0	0	0	0	0	0
0	0	0	0	0	0	0	0	0
0	0	0	0	0	0	0	0	0
0	0	0	0	0	0	0	0	0
0	0	0	0	0	0	0	1	0
0	0	0	0	0	0	0	0	0
0	0	0	0	0	0	0	0	0
0	0	1	0	0	0	0	1	0
0	0	0	0	0	0	0	0	0
1	0	0	0	0	0	0	0	0
0	0	0	0	0	0	0	0	0
0	0	0	0	1	0	0	0	0
0	0	0	0	0	0	0	0	0
0	0	0	0	0	0	0	0	0
1	1	0	0	0	0	0	0	0
1	0	0	0	0	0	0	0	0
1	1	0	0	0	0	0	0	0
1	0	0	0	0	0	0	0	0
0	0	0	0	0	0	0	0	0
0	0	0	0	0	0	0	0	0
0	0	0	0	0	0	0	0	0
0	0	0	0	0	0	0	0	0
1	0	0	0	0	0	0	0	0
0	0	0	0	0	0	0	0	0
0	0	0	0	0	0	0	0	0
0	0	0	0	0	0	0	1	0
1	0	0	0	0	0	0	0	0
1	0	0	0	0	0	0	0	0

0	0	1	0	0	0	0	0	0
1	1	0	0	0	0	0	0	0
1	0	0	0	0	0	0	0	0
0	0	0	0	0	0	0	0	0
0	0	0	0	0	0	0	0	0
1	0	0	0	0	0	0	0	0
0	0	1	0	0	0	1	0	0
0	0	0	0	0	0	0	0	0
1	0	0	0	1	0	0	0	0
0	0	0	0	0	0	0	0	0
0	0	0	0	0	0	0	0	0
1	0	0	0	0	0	0	1	0
0	0	0	0	0	0	0	0	0
0	0	0	0	0	0	0	0	0
0	0	0	0	0	0	0	1	0
0	0	0	0	0	0	0	0	0
0	0	0	0	0	0	0	0	0
1	0	0	0	0	0	0	0	0
0	0	0	0	0	0	0	0	0
0	0	0	0	0	0	0	0	0
1	0	1	0	0	0	0	0	0
0	1	0	0	0	0	1	0	0
0	0	0	0	0	0	0	0	0
0	0	0	0	0	0	0	1	0
1	0	0	0	0	0	0	0	0
0	0	0	0	0	0	0	0	0
0	0	0	0	0	0	0	1	0
0	0	0	0	0	0	1	0	1
0	0	0	0	0	0	0	0	0
1	1	0	0	0	0	0	0	0
0	0	0	0	0	0	0	0	0
0	0	0	0	0	0	0	0	0
0	0	0	0	0	0	0	0	0
1	0	0	0	0	0	0	0	0
1	0	0	0	0	0	0	0	0
1	0	1	0	0	0	0	0	0
0	0	0	0	0	0	0	0	0
0	0	0	0	0	0	0	0	0
0	0	0	1	0	0	0	0	0
1	0	1	0	0	0	0	0	0
1	0	0	0	0	0	0	0	0
0	0	0	0	0	0	0	0	0
0	0	0	1	0	0	0	0	0
1	0	0	0	0	0	0	0	0
0	0	0	0	0	0	0	0	0
0	0	1	0	0	0	0	1	0
0	0	0	0	0	0	0	0	0
0	0	0	0	0	0	0	0	0
0	0	0	0	0	0	0	0	0
0	0	0	0	0	0	0	0	0
1	0	0	0	0	0	0	1	0
0	0	0	0	0	0	0	0	0
0	0	0	0	0	0	0	1	0
0	0	0	0	0	0	0	0	0
1	0	0	0	0	0	0	0	0
1	0	0	0	0	0	0	0	0

1	0	0	0	0	0	0	0	0
0	0	0	0	0	0	0	0	0
1	0	0	0	0	0	0	0	0
0	0	0	0	0	0	0	0	0
0	0	0	0	0	0	0	0	0
0	0	0	0	0	0	0	0	0
0	0	0	0	0	0	0	0	0
0	0	0	0	0	0	0	1	0
1	0	0	0	0	0	0	0	0
1	0	0	1	0	0	0	0	0
0	1	0	0	0	0	0	0	0
1	0	0	0	0	0	0	0	0
0	0	0	0	0	0	0	0	0
0	0	0	1	0	0	0	0	0
0	0	0	0	0	0	0	0	0
1	0	1	0	0	0	0	1	0
0	0	0	0	0	0	0	0	0
0	0	0	0	0	0	0	0	0
0	0	0	0	0	0	0	0	0
1	0	0	0	0	0	0	0	0
1	0	0	0	0	0	0	0	0
1	0	0	0	0	0	0	0	0
1	0	0	0	0	0	0	0	0
0	0	0	0	0	0	0	0	0
0	0	0	0	0	0	0	0	0
1	0	0	0	1	0	0	0	0
0	0	0	0	0	0	0	0	0
1	0	1	0	0	0	0	0	0
0	0	0	0	0	0	0	1	0
0	0	0	0	0	0	0	0	0
0	0	0	0	0	0	0	0	0
0	1	0	0	0	0	0	1	0
0	0	0	0	0	0	0	0	0
0	0	0	0	0	0	0	0	0
0	0	0	0	0	0	0	0	0
0	0	0	0	0	0	0	0	0
0	0	0	0	0	0	0	1	0
0	0	0	0	0	0	0	0	0
1	0	0	0	0	0	0	0	0
1	0	0	0	0	0	0	0	0
0	0	0	0	0	0	0	0	0
0	0	0	0	0	0	0	0	0
0	0	0	0	0	0	0	1	0
1	0	0	0	1	0	0	0	0
0	0	0	0	0	0	0	0	0
0	0	0	0	0	0	0	0	0
0	0	0	0	0	0	0	0	0
1	0	0	0	0	0	0	0	0
0	0	0	0	0	0	0	0	0
0	0	0	0	0	0	0	0	0
0	0	0	0	0	0	0	0	0
0	0	0	0	0	0	0	1	0
0	0	0	0	0	0	0	0	0
0	0	0	0	0	0	0	0	0
1	0	0	0	0	0	0	0	0
0	0	0	0	0	0	0	0	0

0	0	0	0	0	0	0	0	0
0	0	0	0	0	0	0	0	0
0	1	0	0	0	0	0	0	0
0	0	0	0	0	0	0	0	0
1	0	0	0	0	0	0	0	0
0	0	0	0	0	0	0	0	0
0	0	0	0	0	0	0	0	0
0	0	0	0	0	0	0	0	0
1	0	0	0	0	0	0	0	0
0	0	0	0	0	0	0	1	0
0	0	0	0	0	0	0	0	0
0	0	0	0	0	0	0	0	0
1	0	0	0	0	0	0	0	0
1	0	0	0	0	0	0	0	0
0	0	0	0	0	0	0	0	0
0	0	0	0	1	0	0	0	0
1	0	0	0	0	0	0	1	0
0	0	0	0	0	0	0	0	0
1	0	0	0	0	0	0	0	0
0	0	0	0	0	0	0	0	0
0	1	0	0	0	0	0	0	0
0	0	0	0	0	0	0	0	0
0	0	0	0	0	0	0	0	0
1	0	0	0	0	0	0	0	0
0	0	0	0	0	0	0	0	0
0	1	0	0	0	0	0	0	0
0	0	0	0	0	0	0	0	0
0	0	0	0	0	0	0	1	0
0	1	0	0	0	0	0	0	1
0	0	1	0	0	0	0	0	0
0	0	0	0	0	0	0	0	0
0	0	0	1	0	0	0	0	0
0	0	0	0	0	0	0	0	0
0	0	0	0	0	0	0	0	0
0	0	0	0	0	0	0	0	0
0	0	0	0	0	0	0	0	0
0	0	0	0	0	0	1	0	0
1	0	0	0	0	0	0	0	0
0	0	0	0	0	0	0	1	0
0	0	0	0	0	0	0	1	0
1	0	0	0	0	0	0	0	0
0	1	0	0	0	0	0	0	0
0	0	0	0	1	0	0	0	0
0	0	0	0	0	0	0	0	0
0	1	0	0	0	0	0	0	0
0	0	0	0	0	0	0	0	0
0	0	0	0	0	0	0	0	0
0	0	0	0	0	0	0	0	0
0	0	0	0	0	0	0	0	0
1	0	0	0	0	0	0	1	0
0	0	0	0	1	0	0	1	0
0	0	0	0	0	0	0	0	0
1	0	0	0	0	0	0	0	0
1	0	0	0	0	0	0	0	0
0	0	0	0	0	0	0	0	0
0	0	0	0	0	0	0	0	0

0	0	0	0	0	0	1	0	0
1	1	0	0	0	0	0	0	0
0	0	0	0	0	0	1	0	0
0	0	0	0	0	0	0	0	0
1	0	0	0	0	0	0	0	0
0	0	0	0	0	0	0	0	0
0	0	0	0	0	0	0	0	0
0	0	0	0	0	0	0	0	0
0	0	0	0	0	0	1	0	0
0	0	0	0	0	0	0	0	0
1	0	0	0	0	0	0	0	0
0	0	0	0	0	0	0	1	0
0	0	0	0	0	0	0	0	0
0	0	0	1	0	0	0	0	0
0	0	0	0	0	0	0	0	0
0	1	0	0	0	0	0	1	0
1	0	0	0	1	0	0	0	0
1	0	0	0	0	0	0	0	0
0	0	0	0	0	0	0	0	0
1	0	0	0	0	0	0	0	0
1	0	0	0	1	0	0	0	0
0	1	0	0	0	0	1	0	0
1	0	0	0	0	0	0	0	0
1	0	0	0	0	0	0	0	0
1	0	0	0	0	0	0	0	0
0	0	0	0	0	0	0	0	0
1	0	0	0	0	0	0	0	0
1	0	0	0	0	0	0	0	0
0	1	0	0	0	0	0	0	0
0	0	0	0	0	0	0	0	0
1	0	0	0	0	0	0	0	0
1	0	0	0	0	0	0	0	0
0	0	0	0	1	0	0	0	0
1	0	0	0	0	0	0	0	0
0	0	0	0	0	0	0	0	0
0	0	0	0	1	0	0	0	0
1	0	0	0	0	0	0	0	0
0	0	0	0	1	0	0	0	0
1	0	0	0	0	0	0	0	0
0	1	0	0	0	0	0	0	0
1	0	0	0	0	0	0	0	0
0	0	0	0	0	0	1	0	0
1	0	0	0	0	0	0	0	0
1	0	0	0	1	0	0	0	0
0	0	0	0	0	0	0	1	0
0	0	0	0	0	0	0	0	0
0	1	0	0	0	0	0	0	0
0	0	0	0	0	0	0	0	0
0	0	0	0	0	0	0	0	0
0	0	0	0	0	0	0	0	0
1	0	0	0	0	0	0	0	0
1	0	0	0	0	0	0	0	0
1	0	0	0	0	0	0	0	0
1	0	0	0	0	0	0	0	0
0	0	0	0	0	0	0	0	0
0	0	0	0	1	0	0	0	0

0	1	0	0	0	0	0	0	0
1	1	0	0	0	0	0	0	0
1	0	0	0	0	0	0	0	0
1	0	0	0	0	0	0	0	0
0	0	0	0	0	0	0	0	0
0	0	0	0	0	0	0	1	0
1	0	0	0	0	0	0	0	0
0	0	0	0	0	0	0	0	0
0	0	0	0	0	0	0	0	0
0	0	0	1	0	0	0	0	0
0	0	0	0	0	0	0	1	0
0	0	0	0	0	0	0	0	0
0	0	0	0	0	0	0	1	0
1	0	0	0	0	0	0	0	0
0	0	0	0	0	0	0	0	0
1	0	0	0	0	0	0	0	0
0	0	0	0	0	0	0	0	0
0	0	0	0	0	0	0	0	0
1	0	0	0	0	0	0	0	0
1	0	0	0	0	0	0	0	0
0	0	0	1	0	0	0	0	0
0	0	0	0	0	0	0	0	0
0	0	0	0	0	0	0	0	0
0	0	0	0	0	0	0	1	0
0	0	0	0	0	0	0	0	0
0	0	0	0	0	0	0	0	0
0	0	0	0	0	0	0	0	0
1	0	0	0	0	0	0	0	0
0	0	0	0	0	0	0	0	0
0	0	0	0	0	0	0	0	0
0	0	0	0	0	0	0	0	0
1	0	0	0	0	0	0	0	0
0	0	0	0	0	0	0	0	0
0	0	0	0	0	0	0	0	0
0	0	0	0	0	0	0	0	0
0	0	0	0	0	0	0	0	0
1	0	0	0	0	0	0	0	0
0	1	0	0	0	0	0	1	0
1	0	0	0	0	0	0	0	0
0	0	0	0	0	0	0	1	0
0	0	0	0	0	0	1	0	0
0	0	0	0	0	0	0	0	0
1	0	0	0	0	0	0	0	0
1	0	0	0	0	0	0	0	0
1	0	0	0	0	0	0	0	0
0	0	0	0	0	0	0	0	0
1	0	0	0	0	0	0	0	0
1	0	0	0	0	0	0	0	0
1	0	1	0	0	0	0	1	0
1	0	0	0	0	0	0	0	0
1	0	0	0	0	0	0	0	0
0	0	0	0	0	0	0	0	0
0	0	0	0	0	0	0	0	0
0	0	0	0	0	0	0	0	0
0	1	0	0	0	0	0	0	0
1	1	0	0	0	0	1	0	0

0	0	0	0	0	0	0	1	1
1	0	0	0	0	0	1	0	0
0	0	0	0	0	0	0	0	0
0	0	0	0	0	0	0	0	0
0	0	0	0	0	0	0	0	0
0	0	0	0	0	0	0	0	0
0	0	0	0	0	0	0	0	0
0	0	0	0	0	0	0	0	0
0	0	0	0	0	0	0	0	0
0	0	0	0	0	0	0	0	0
1	1	0	0	0	0	0	0	0
1	0	1	0	0	0	0	0	0
0	0	0	0	0	0	0	0	0
1	0	0	0	0	0	0	0	0
0	0	0	1	0	0	0	0	0
0	0	0	0	0	0	0	0	0
1	0	0	0	0	0	0	0	0
1	0	0	0	0	0	0	0	0
0	0	0	0	0	0	0	0	0
0	0	0	0	0	0	0	0	0
0	0	0	0	0	0	0	0	0
0	0	0	0	0	0	0	0	0
1	0	1	0	0	0	0	0	0
0	0	0	0	0	0	0	1	0
0	0	0	0	0	0	0	0	0
0	0	0	0	0	0	0	0	0
1	1	0	0	0	0	0	1	0
0	1	0	0	0	0	0	0	0
0	0	0	0	0	0	0	0	0
1	0	0	0	0	0	0	0	0
0	0	0	1	0	0	0	0	0
1	0	0	0	0	0	0	0	0
0	0	0	0	0	0	0	0	0
1	0	0	0	0	0	0	0	0
0	0	0	0	0	0	0	0	0
0	0	0	0	0	0	0	0	0
0	0	0	0	0	0	0	0	0
0	0	0	0	0	0	0	0	0
0	0	0	0	0	0	0	0	0
0	0	0	0	0	0	0	0	0
0	0	0	0	0	0	0	0	0
0	0	0	0	0	0	0	0	0
0	0	0	0	0	0	0	0	0
0	0	0	0	0	0	0	1	0
0	0	0	0	0	0	0	0	0
0	0	0	0	0	0	0	1	0
0	0	0	0	0	0	0	0	0
1	0	0	0	0	0	0	0	0
1	0	0	0	0	0	0	0	0
0	1	0	0	0	0	0	0	0
0	0	0	1	0	0	0	0	0
0	0	0	0	0	0	0	1	0
0	0	0	0	0	0	0	0	0
0	0	0	0	0	0	0	0	0
0	0	0	0	0	0	0	0	0
1	0	0	0	0	0	0	0	0

0	0	0	0	0	0	0	0	0
0	1	0	0	0	0	0	0	0
1	1	0	0	0	0	0	0	0
0	0	0	0	0	0	0	0	0
0	0	0	0	0	0	0	1	0
1	0	0	0	0	0	0	0	0
1	0	1	0	0	0	0	0	0
1	0	0	0	0	0	0	0	0
0	0	0	0	0	0	0	0	0
1	0	0	1	0	0	0	0	0
1	0	1	0	0	0	0	1	1
0	0	1	0	0	0	0	0	0
0	0	1	0	0	0	0	0	0
0	1	0	0	0	0	1	0	0
0	0	0	0	0	0	0	0	0
1	0	0	0	0	0	0	0	0
0	0	0	0	1	0	0	0	0
0	0	0	0	0	0	0	0	0
0	0	0	0	0	0	0	1	0
1	0	0	0	0	0	0	0	0
1	0	0	0	0	0	0	0	0
0	0	0	1	0	0	0	0	0
0	0	0	0	0	0	0	0	0
1	0	0	0	0	0	0	0	0
1	0	0	0	0	0	0	0	0
0	0	0	0	0	0	0	0	0
1	0	0	0	0	0	0	0	0
0	0	0	0	0	0	0	0	0
0	0	0	0	0	0	0	0	0
1	0	0	0	0	1	0	1	0
1	0	0	0	0	0	0	0	0
1	0	0	0	0	0	0	0	0
0	0	0	0	0	0	0	0	0
0	0	0	0	0	0	0	0	0
0	0	0	0	0	0	0	0	0
0	0	0	0	0	0	0	0	0
0	0	0	0	0	0	0	0	0
0	0	0	0	0	0	0	0	0
0	0	0	0	0	0	0	0	0
0	0	0	0	0	0	0	0	0
0	0	0	0	0	0	0	0	0
1	0	0	0	0	0	0	0	0
0	1	0	0	0	0	0	0	0
1	0	0	0	0	0	0	0	0
1	0	0	0	1	0	0	0	0
1	0	0	0	0	0	0	0	0
1	0	0	0	0	0	1	0	0
0	0	0	0	0	0	0	0	0
1	0	0	0	0	0	0	0	0
1	0	1	0	0	0	0	0	0
0	0	0	0	0	0	0	0	0
0	0	0	0	0	0	0	0	0
0	0	0	0	0	0	0	0	0
0	0	0	0	0	0	0	0	0
1	1	1	0	0	0	0	0	0
0	0	0	0	0	0	0	0	0

0	0	0	0	0	0	0	0	0
0	0	0	0	0	0	0	0	0
0	1	0	0	0	0	0	0	0
1	0	0	0	0	0	0	0	0
0	0	0	0	0	0	0	0	0
1	0	0	0	1	0	0	0	0
0	0	0	0	0	0	0	0	0
0	0	0	0	0	0	0	0	0
1	0	0	0	0	0	0	0	0
1	0	0	0	0	0	0	0	0
1	0	0	0	0	0	0	0	0
0	0	0	0	0	0	0	0	0
0	0	0	0	0	0	0	0	0
1	0	0	0	0	0	0	0	0
0	0	0	0	0	0	1	1	0
0	0	0	0	0	0	0	0	0
0	0	0	0	0	0	0	0	0
1	0	0	0	0	0	0	0	0
1	1	0	0	0	0	0	0	0
0	0	0	0	1	0	0	0	0
0	0	0	0	0	0	0	0	0
0	0	0	0	0	0	0	0	0
1	0	0	0	0	0	0	0	0
0	0	0	0	0	0	0	0	0
1	0	0	0	0	0	0	0	0
0	0	0	0	0	0	0	0	0
0	0	0	0	0	0	1	1	0
0	0	0	0	0	0	0	0	0
0	0	0	0	0	0	0	0	0
1	0	0	0	0	0	0	0	0
0	0	0	0	0	0	0	0	0
1	0	0	0	0	0	0	0	0
0	0	0	0	0	0	0	0	0
1	0	0	0	0	0	0	0	0
1	0	0	0	0	0	0	0	0
0	0	0	0	0	0	0	1	1
0	0	0	0	0	0	0	0	0
0	0	0	0	0	0	0	0	0
1	0	0	0	0	0	0	0	0
0	0	0	0	0	0	0	0	0
0	0	0	0	0	0	0	1	0
0	0	0	0	0	0	0	0	0
0	0	0	0	0	0	0	0	0
1	0	0	0	0	0	0	0	0
0	0	0	0	0	0	0	0	0
0	0	0	0	0	0	0	0	0
1	0	0	0	0	0	0	0	0
0	0	0	0	0	0	0	0	0
1	0	0	0	0	0	0	0	0
0	0	0	0	0	0	0	0	0
0	0	0	0	0	0	0	0	0
0	0	0	0	0	0	0	0	0
1	0	0	0	0	0	0	0	0
0	0	0	0	0	0	0	1	0
1	0	0	0	0	0	0	0	0
0	0	0	0	0	0	0	1	0

1	0	0	0	0	0	0	0	0
0	0	0	0	0	0	1	0	0
0	0	0	0	0	0	0	0	0
1	0	0	0	0	0	0	0	0
0	0	0	0	0	0	0	0	0
0	0	0	1	0	0	0	0	0
0	0	0	0	0	0	0	0	0
0	0	0	0	0	0	0	0	0
0	0	0	0	0	0	0	1	0
0	0	0	0	0	0	0	0	0
0	0	0	0	0	0	0	0	0
0	1	0	0	0	0	0	0	0
0	0	0	0	0	0	0	0	0
0	1	0	0	0	0	0	0	0
0	0	0	0	0	0	0	0	0
1	0	0	0	0	0	0	0	0
0	0	0	0	0	0	0	0	0
0	0	0	0	0	0	0	0	0
0	0	0	0	0	0	0	0	1
0	0	0	0	0	0	0	0	0
0	0	0	0	0	0	0	0	0
0	0	0	0	0	0	0	0	0
0	0	0	0	0	0	0	0	0
0	0	0	0	0	0	0	0	0
0	0	0	0	0	0	0	0	0
0	0	0	0	0	0	0	0	0
0	0	0	0	0	0	0	0	0
1	0	0	0	0	0	0	0	0
0	0	0	0	0	0	0	0	0
0	0	0	0	0	0	0	0	0
0	0	0	0	0	0	0	0	0
0	0	0	0	0	0	0	0	0
1	0	0	0	0	0	0	0	0
0	0	0	0	0	0	0	0	0
0	0	0	0	0	0	0	0	0
0	0	0	0	0	0	0	0	0
0	0	0	0	0	0	0	0	0
1	0	0	0	0	0	0	0	0
0	0	0	0	0	0	0	0	0
1	0	0	0	0	0	0	0	0
1	0	0	0	0	0	0	0	0
0	0	0	0	0	0	0	1	0
0	0	0	0	0	0	0	0	0
1	0	0	0	0	0	0	1	0
0	0	0	0	0	0	0	0	0
1	0	0	0	0	0	0	1	0
1	0	0	0	0	0	0	0	0
0	0	0	0	0	0	0	0	0
0	0	0	0	0	0	0	0	0
0	0	0	0	0	0	0	0	0
1	0	0	0	0	0	0	0	0
0	0	0	0	0	0	0	0	0
0	0	0	0	0	0	0	0	0
1	0	0	0	0	0	0	0	0
0	0	0	0	0	0	0	0	0
1	0	1	0	0	0	0	1	0

0	0	0	0	0	0	0	0	0
0	0	0	0	0	0	0	0	0
1	0	0	0	0	0	0	0	0
0	0	0	0	0	0	0	0	0
0	0	0	0	0	0	0	0	0
1	0	0	0	0	0	0	0	0
1	0	0	0	0	0	0	1	0
1	0	0	0	0	0	0	0	0
0	0	0	0	0	0	0	0	0
1	0	0	0	0	0	0	0	0
0	0	0	0	0	0	0	1	0
0	0	0	0	0	0	0	0	0
0	0	0	0	0	0	0	0	0
0	0	0	0	0	0	0	0	0
0	0	0	0	0	0	0	0	0
1	0	0	0	0	0	0	0	0
0	0	0	0	0	0	0	0	0
0	0	0	0	0	0	0	0	0
0	0	0	0	0	0	1	0	0
0	0	0	0	0	0	0	0	0
0	0	0	0	0	0	0	0	0
0	0	0	0	0	0	0	0	0
0	0	0	0	0	0	0	0	0
1	0	0	0	0	0	1	0	0
1	0	0	0	0	0	0	0	0
0	0	0	0	0	0	0	0	0
1	0	0	0	0	0	0	0	0
0	0	0	0	0	0	0	1	0
0	0	0	0	0	0	0	0	0
0	0	0	1	0	0	0	0	0
0	0	0	0	0	0	0	0	0
0	0	0	0	0	0	0	0	0
0	0	0	0	0	0	0	0	0
0	0	0	0	0	0	0	0	0
1	0	0	0	0	0	0	0	0
0	0	0	0	0	0	0	0	0
0	0	0	1	0	0	0	0	0
0	0	0	0	0	0	0	0	0
0	0	0	0	0	0	0	0	0
0	0	0	0	0	0	1	0	0
1	0	0	0	0	0	0	0	0
0	0	0	0	0	0	0	0	0
1	0	0	0	0	0	0	0	0
0	1	0	0	0	0	0	0	0
1	0	0	0	0	0	0	0	0
0	0	0	0	0	0	0	0	0
0	1	0	0	0	0	0	0	0
1	0	0	0	1	1	0	0	0
0	0	0	1	0	0	0	0	0
1	0	0	0	0	0	0	0	0
0	0	0	0	0	0	0	0	0
1	0	0	0	0	0	0	1	0
0	0	0	0	0	0	0	0	0
0	0	0	0	0	0	0	0	0
0	0	0	0	0	0	0	0	0
0	0	0	0	0	0	0	0	0

0	0	0	0	0	0	0	0	0
0	0	0	0	0	0	1	0	0
1	0	0	0	0	0	0	0	0
0	0	0	0	0	0	0	0	0
0	0	0	0	1	0	0	0	0
0	0	0	0	0	0	0	0	0
1	0	1	0	0	0	0	1	0
0	0	0	0	0	0	0	1	0
1	0	0	0	0	0	0	0	0
0	0	0	0	0	0	0	0	0
1	0	0	0	0	0	0	0	0
1	0	0	0	0	0	0	0	0
0	0	0	0	0	0	0	0	0
0	0	0	0	0	0	0	0	0
0	0	0	0	0	0	0	0	0
0	0	0	0	0	0	0	0	0
1	0	0	0	0	0	0	0	0
0	0	0	0	0	0	0	1	0
0	0	0	0	0	0	0	0	0
0	0	0	0	0	0	0	0	0
0	0	1	0	0	0	0	0	0
1	0	0	0	0	0	0	0	0
1	1	0	0	0	0	0	0	0
1	0	1	0	0	0	0	0	0
0	0	0	0	0	0	0	0	0
0	0	0	0	0	0	0	1	0
0	0	0	0	0	0	0	1	0
0	0	0	0	0	0	0	0	0
0	1	1	0	0	0	0	0	0
0	0	0	0	1	0	0	0	0
0	0	0	0	0	0	0	0	0
1	0	0	0	0	0	0	0	0
0	0	0	0	0	0	0	0	0
1	0	0	0	0	0	0	0	0
0	0	0	0	0	0	0	0	0
0	0	0	0	0	0	0	0	0
0	0	0	0	1	0	0	0	0
0	0	1	0	0	0	0	0	0
1	0	0	0	1	0	0	0	0
0	0	0	0	0	0	0	0	0
1	0	0	0	1	0	0	0	0
1	0	0	0	0	0	0	0	0
0	0	0	0	0	0	0	0	0
0	0	0	0	0	0	0	0	0
1	0	0	0	0	0	0	0	0
0	0	0	0	0	0	0	0	0
0	1	0	0	0	0	0	0	0
0	0	0	0	0	0	0	0	0
0	0	0	0	0	0	0	0	0
0	0	0	0	0	0	0	0	0
0	1	1	0	0	0	0	0	0
0	0	0	0	0	0	0	0	0
0	1	0	0	0	0	0	0	0
0	0	0	0	0	0	0	0	0
0	0	0	0	0	0	0	0	0
1	0	1	0	0	0	0	0	0

0	0	0	0	0	0	0	0	0
0	0	0	0	0	0	0	1	1
0	0	0	0	0	0	0	0	0
0	0	0	0	0	0	0	0	0
0	0	0	0	0	0	0	0	0
0	0	0	0	0	0	0	0	0
1	0	0	0	0	0	0	0	0
1	0	0	0	0	0	0	0	0
0	0	0	0	0	0	0	0	0
0	0	0	0	0	0	1	0	0
0	0	0	0	0	0	0	0	0
1	0	0	0	0	0	0	0	0
0	1	0	0	0	0	0	0	0
1	0	0	0	0	0	0	0	0
1	0	0	0	0	0	0	0	0
0	0	0	0	0	0	0	0	0
1	0	0	0	0	0	0	0	0
0	0	0	0	0	0	0	0	0
0	0	0	0	0	0	0	0	0
0	0	0	0	0	0	0	0	0
1	0	0	0	0	0	0	0	0
1	0	0	0	0	0	0	0	0
0	0	0	0	0	0	0	0	0
0	0	0	0	0	0	0	0	0
0	0	0	0	0	0	0	0	0
0	0	0	0	0	0	0	1	0
0	0	0	0	0	0	0	0	0
0	0	0	0	0	0	0	0	0
0	0	0	0	0	0	0	0	0
0	0	0	0	0	0	0	0	0
0	0	0	0	0	0	0	0	0
1	0	0	0	0	0	0	0	0
1	0	0	0	0	0	0	0	0
1	0	0	0	0	0	0	0	0
1	0	0	0	0	0	0	0	0
0	0	0	0	0	0	1	0	0
0	0	0	0	0	0	0	1	0
0	0	0	0	0	0	0	0	0
0	0	0	0	0	0	0	0	0
0	0	0	0	0	0	0	0	0
0	0	0	0	0	0	0	0	0
1	0	0	0	0	0	0	0	0
0	0	0	0	0	0	0	0	0
0	1	0	0	0	0	0	1	0
0	0	0	0	0	0	0	1	0
0	0	0	0	0	0	0	0	1
0	0	0	0	0	0	0	0	0
0	0	0	0	0	0	0	0	0
0	0	0	0	0	0	0	0	0
1	0	0	0	0	0	0	0	0
0	0	0	0	0	0	0	0	0
1	0	0	0	1	0	0	0	0
1	0	0	0	0	0	0	0	0
0	0	0	0	0	0	0	0	0
0	0	0	0	0	0	0	0	0
1	0	0	0	0	0	0	1	0

0	1	0	0	0	0	0	0	0
1	0	0	0	0	0	0	0	0
0	1	0	0	1	0	0	0	0
0	0	0	0	0	0	0	1	0
0	0	0	0	0	0	0	1	0
0	0	0	0	0	0	0	0	0
1	0	0	0	0	0	0	0	0
0	0	0	0	0	0	0	0	0
0	0	0	0	0	0	0	0	0
0	0	0	0	0	0	0	1	0
0	0	0	0	0	0	0	0	0
0	0	0	0	0	0	0	1	0
0	0	0	1	0	0	0	0	0
1	0	0	0	0	0	0	0	0
0	0	0	0	0	0	1	0	0
0	0	0	0	0	0	0	0	0
1	0	0	0	0	0	0	0	0
1	0	0	0	0	0	0	0	0
0	1	0	0	0	0	0	0	0
0	0	0	0	1	0	0	0	0
0	0	0	0	0	0	0	0	0
0	0	0	0	0	0	0	0	0
0	0	0	0	0	0	0	0	0
1	0	0	0	0	0	0	0	0
0	0	0	0	0	0	0	0	0
0	0	0	0	0	0	0	0	0
0	0	0	0	0	0	0	0	0
1	0	0	0	0	0	0	1	0
0	0	0	0	0	0	0	1	0
0	0	0	0	0	0	0	0	0
1	0	1	0	0	0	0	0	0
0	0	0	0	0	0	0	0	0
0	0	0	0	0	0	0	0	1
0	0	0	0	0	0	0	0	0
0	0	0	0	0	0	0	1	0
0	0	0	0	0	0	0	0	0
0	0	0	0	0	0	0	0	0
0	0	0	0	0	0	0	0	0
0	0	0	0	0	0	0	0	0
1	0	0	0	0	0	0	0	0
1	0	0	0	0	0	0	0	0
1	0	0	0	0	0	0	0	0
0	0	0	0	0	0	1	0	0
0	0	0	0	0	0	1	0	0
0	0	0	0	0	0	1	0	0
1	0	0	0	0	0	0	0	0
1	0	0	0	0	0	0	0	0
1	0	0	0	0	0	0	0	0
0	0	0	0	0	0	0	0	0
0	0	0	0	0	0	0	0	0
0	0	0	0	0	0	0	0	0
0	1	0	0	0	0	0	0	0
0	0	0	0	0	0	0	0	0
1	0	0	0	1	0	0	0	0
1	0	0	0	0	0	0	0	0
0	0	0	0	0	0	0	0	0

0	0	0	0	0	0	0	0	0
0	0	0	0	0	0	0	0	0
0	0	0	0	0	0	0	0	0
0	0	0	0	0	0	0	0	0
0	0	0	0	0	0	0	0	0
0	0	0	0	0	0	0	0	0
0	0	0	0	0	0	0	0	0
0	0	0	0	0	0	0	0	0
0	0	0	0	0	0	0	0	0
1	0	0	0	0	0	0	0	0
0	0	0	0	0	0	0	1	0
0	0	0	0	0	0	0	1	0
0	0	0	0	0	0	0	0	0
0	0	0	0	0	0	0	0	0
0	0	0	0	0	0	0	0	0
0	0	0	0	0	0	0	0	0
0	1	0	0	0	0	0	0	0
1	0	0	0	0	0	0	0	1
0	0	0	0	0	0	0	0	0
0	0	0	0	0	0	0	0	0
0	0	0	1	0	0	0	0	0
0	0	0	0	0	0	0	0	0
0	0	0	0	0	0	0	0	0
1	0	0	0	0	0	0	0	0
0	0	0	0	0	0	0	0	0
1	0	0	0	0	0	0	0	0
0	0	0	0	0	0	0	0	0
0	0	0	0	0	0	0	0	0
1	0	0	0	0	0	0	0	0
0	0	0	0	0	0	0	0	0
0	0	0	0	0	0	0	0	0
0	0	0	0	0	0	0	0	0
0	0	0	0	0	0	0	1	0
0	0	0	0	0	0	0	0	0
0	0	0	0	0	0	0	0	0
1	0	0	0	0	0	0	0	0
0	0	0	0	0	0	0	1	0
0	0	0	0	0	0	0	0	0
0	0	0	0	0	0	0	0	0
0	0	0	0	0	0	0	0	0
1	0	0	0	0	0	0	0	0
0	0	0	0	0	0	0	0	0
0	0	0	0	0	0	0	0	0
1	0	0	0	0	0	0	0	0
0	0	0	0	0	0	0	0	0
0	0	0	0	0	0	0	0	0
1	0	0	0	0	0	0	0	0
0	0	0	0	0	0	0	0	0
0	0	0	0	0	0	0	0	0
0	0	0	0	0	0	0	0	0
0	0	0	0	0	0	0	0	0
0	0	0	0	0	0	0	0	0
0	0	0	0	0	0	0	1	0
1	0	0	0	0	0	1	0	0
1	0	0	0	0	0	1	0	0
0	0	0	0	0	0	0	0	0

0	0	0	0	0	0	0	0	0
0	0	0	0	0	0	0	0	0
1	0	1	0	1	0	0	0	0
1	1	1	0	0	0	0	0	0
0	0	0	0	0	0	0	0	0
0	0	0	0	0	0	0	0	0
0	0	0	1	0	0	0	0	0
0	0	0	0	0	0	0	0	0
0	0	0	0	0	0	0	1	0
0	0	0	0	0	0	0	0	0
1	0	0	0	0	0	0	0	0
0	0	0	0	0	0	0	0	0
0	0	0	0	0	0	0	0	0
0	0	0	0	0	0	0	0	0
0	0	0	0	0	0	0	0	0
0	0	0	0	0	0	0	0	0
1	0	0	0	0	0	0	0	0
0	0	0	0	0	0	0	0	0
0	0	0	0	0	0	0	0	0
0	0	0	1	0	0	0	0	0
0	0	0	0	0	0	0	0	0
0	0	0	0	0	0	0	0	0
0	0	0	0	0	0	0	0	0
0	0	0	0	0	0	0	0	0
0	0	0	0	0	0	0	0	0
1	0	0	0	0	0	0	0	0
0	0	0	0	0	0	0	0	0
0	1	0	0	0	0	0	0	0
1	0	0	0	0	0	0	0	0
0	0	0	0	0	0	0	0	0
0	0	0	0	0	0	0	0	0
0	0	0	0	0	0	0	0	0
0	0	0	0	0	0	0	0	0
0	0	0	0	0	0	0	0	0
0	0	0	0	0	0	0	0	0
0	0	0	0	0	0	0	0	0
1	0	0	0	0	0	0	0	0
0	0	0	0	0	0	0	0	0
0	0	0	0	0	0	0	0	0
0	0	0	0	0	0	0	0	0
1	0	0	0	0	0	0	1	0
0	0	0	0	0	0	0	0	0
0	0	0	0	0	0	0	0	0
1	0	0	0	0	0	0	0	0
0	0	0	0	0	0	0	0	0
0	0	0	0	0	0	0	0	0
1	0	0	0	0	0	0	0	0
0	0	0	0	0	0	0	0	0
1	0	0	0	0	0	0	0	0
1	0	0	0	0	0	0	0	0
1	0	1	0	0	0	0	0	0
1	0	0	0	0	0	0	0	0
1	0	0	0	0	0	0	0	0
0	0	0	0	0	0	0	0	0
0	0	0	0	0	0	0	0	0
0	0	0	0	0	0	0	0	1

0	0	0	0	0	0	0	0	0
0	1	0	0	0	0	0	0	0
0	0	0	0	0	0	0	0	0
0	0	0	0	0	0	0	0	0
0	0	0	0	0	0	0	0	0
0	0	0	0	0	0	0	0	0
1	0	0	0	1	0	0	0	0
0	0	0	0	0	0	0	0	0
0	0	0	0	0	0	0	0	0
0	0	0	0	0	0	0	0	0
1	0	0	0	0	0	0	0	0
0	0	0	0	0	0	0	0	0
1	0	0	0	0	0	0	0	0
0	0	0	0	0	0	0	0	0
0	0	0	0	0	0	0	0	0
1	0	0	0	0	0	0	0	0
0	0	0	0	0	0	0	0	0
0	0	0	0	0	0	0	0	0
0	0	0	0	0	0	0	0	0
1	0	0	0	0	1	0	0	0
0	0	0	0	0	0	0	0	0
1	0	0	0	0	0	0	0	0
0	0	0	0	1	0	0	0	0
1	0	1	0	0	0	0	1	0
1	0	0	1	0	0	0	0	0
1	0	0	0	0	0	0	0	0
1	0	0	0	0	0	0	0	0
1	0	0	0	0	0	0	0	0
1	0	0	0	0	0	0	0	0
1	0	0	0	0	0	0	0	0
0	0	0	0	0	0	0	0	0
0	0	0	0	0	0	0	0	0
1	0	1	0	0	0	0	0	0
0	0	0	0	0	0	0	1	0
0	0	0	0	0	0	0	0	0
1	0	0	0	0	0	0	0	0
0	0	0	1	0	0	0	0	0
0	0	0	0	0	0	0	0	0
0	0	0	0	0	0	1	0	0
0	0	0	0	0	0	0	0	0
0	0	0	0	0	0	0	0	0
1	0	0	0	0	0	0	0	0
1	0	0	0	0	0	0	0	0
0	0	0	0	0	0	0	0	0
0	0	0	0	0	0	0	0	0
0	0	0	0	0	0	0	1	0
1	0	0	0	0	0	0	0	0
0	0	0	0	0	0	0	0	0
0	0	0	0	0	0	0	1	0
1	0	0	0	0	0	0	1	0
0	0	0	0	0	0	0	0	0
0	0	0	0	0	0	0	0	0
1	0	0	0	0	0	0	0	0
0	0	0	0	0	0	0	1	0
0	0	0	0	0	0	0	0	0
0	0	0	0	0	0	0	0	0

1	0	0	0	1	0	0	0	0
0	0	0	0	1	0	0	0	0
0	0	0	0	0	0	0	0	0
0	0	0	0	0	0	0	0	0
0	0	0	0	0	0	0	0	0
0	1	0	0	0	0	0	0	0
1	0	0	0	0	0	0	0	0
0	0	0	0	0	0	0	0	0
0	0	0	0	0	0	0	0	0
1	0	0	0	0	0	0	1	0
0	0	0	0	0	0	0	0	0
0	0	0	0	0	0	0	0	0
1	1	0	0	0	0	0	0	0
0	0	0	0	0	0	0	0	0
0	0	0	0	0	0	0	0	0
0	0	0	0	0	0	0	0	0
0	0	0	0	0	0	0	0	0
0	0	0	0	0	0	0	0	0
1	0	0	0	0	0	0	0	0
0	0	0	0	0	0	0	0	0
1	0	0	0	0	0	0	0	0
1	0	1	0	0	0	0	0	0
0	0	0	0	0	0	0	0	0
1	0	0	0	0	0	0	0	0
0	0	0	0	0	0	0	0	0
0	0	0	0	0	0	0	0	0
0	0	0	0	0	0	0	0	0
0	0	0	1	0	0	0	0	0
0	0	0	0	0	0	0	0	1
0	0	0	0	0	0	0	0	0
1	0	0	0	0	0	0	0	0
0	0	0	0	0	0	0	0	0
0	0	0	0	0	0	0	0	0
1	0	0	0	0	0	0	0	0
0	0	0	0	0	0	0	0	0
0	0	0	0	0	0	0	0	1
1	0	0	0	0	0	0	0	0
0	0	0	0	0	0	0	0	0
0	0	0	0	0	0	0	0	0
0	0	0	0	0	0	0	0	0
0	1	0	0	0	0	0	0	0
0	0	0	0	0	0	1	0	0
0	1	0	0	0	0	0	1	0
0	0	0	0	0	0	0	0	0
0	0	0	0	0	0	0	0	0
0	0	0	0	0	0	0	0	0
0	0	0	0	0	0	0	0	0
1	0	0	0	1	0	0	0	0
0	0	0	0	0	0	0	0	0
0	0	0	0	0	0	0	1	0
0	0	0	0	0	0	0	0	0
0	0	0	0	0	0	0	0	0
0	0	0	0	0	0	0	0	0
0	0	0	0	0	0	1	0	0
0	0	0	0	0	0	0	0	0
0	0	0	0	0	0	0	0	0

0	0	0	0	0	0	0	0	0
1	0	0	0	0	0	0	0	1
0	1	0	0	0	0	0	0	0
0	0	0	0	0	0	0	0	0
0	0	0	0	0	0	0	0	0
1	0	0	0	1	0	0	0	0
0	0	0	0	0	0	0	0	0
0	0	0	0	0	0	0	0	0
1	0	0	0	0	0	0	0	0
0	0	0	0	0	0	0	0	0
0	0	0	0	0	0	0	0	0
0	0	0	0	0	0	0	0	0
0	0	0	0	0	0	0	1	0
0	0	0	0	0	0	0	0	0
0	0	0	0	0	0	0	0	0
0	0	0	0	0	0	0	0	0
0	0	0	0	0	0	1	0	0
0	0	0	0	0	0	0	0	0
1	0	0	0	0	0	0	0	0
0	1	0	0	0	0	0	1	0
1	0	0	0	0	0	0	0	0
0	0	0	0	0	0	0	0	1
0	0	0	0	0	0	0	0	0
0	0	0	0	0	0	1	0	0
0	0	0	0	0	0	0	0	0
1	0	1	0	0	0	0	0	0
0	0	0	0	0	0	1	0	0
0	0	0	0	0	0	0	0	0
0	0	0	0	0	0	0	0	0
0	0	0	0	0	0	0	1	0
0	0	0	0	0	0	0	0	0
1	0	0	0	0	0	0	0	0
0	0	0	0	0	0	0	0	0
0	0	0	0	0	0	0	0	0
0	0	0	0	0	0	0	0	0
1	0	0	0	0	0	0	0	0
0	0	0	0	0	0	0	0	0
0	0	0	0	0	0	1	0	0
0	0	0	0	0	0	0	0	0
1	0	0	0	0	0	0	0	0
0	0	0	0	0	0	0	0	0
0	0	0	0	0	0	1	1	0
1	0	0	0	0	0	0	0	0
0	0	0	0	0	0	0	0	0
0	0	0	0	0	0	0	0	0
0	0	0	0	0	0	0	0	0
0	0	0	0	0	0	0	0	0
1	0	1	0	0	0	0	0	0
0	0	0	0	0	0	0	0	0
1	0	0	0	0	0	0	0	0
0	0	0	0	0	0	0	1	0
0	0	0	0	0	0	0	0	0
0	0	0	0	0	0	0	0	0
0	0	0	0	0	0	0	0	0
0	0	0	0	0	0	0	0	0
0	0	0	0	0	0	0	0	0

0	0	0	0	0	0	0	0	0
0	0	0	0	0	0	0	0	0
1	0	0	0	0	0	0	0	0
1	0	0	0	0	0	0	0	0
0	0	0	0	0	0	0	0	0
1	0	0	0	0	0	0	0	0
0	0	0	0	0	0	0	0	0
0	0	0	0	0	0	0	0	0
1	0	0	0	0	0	0	0	0
0	0	0	0	0	0	0	0	0
0	0	0	0	0	0	0	0	0
0	0	0	0	0	0	0	0	0
1	0	0	0	0	0	0	0	0
0	0	0	0	0	0	0	0	0
0	0	0	0	0	0	0	0	0
0	0	0	0	0	0	0	0	0
0	0	0	0	0	0	0	0	0
0	0	0	0	0	0	0	0	0
0	0	0	0	0	0	0	0	0
0	0	0	0	0	0	0	1	0
0	1	0	0	0	0	0	0	0
1	0	0	0	0	0	0	0	0
0	0	0	0	0	0	0	0	0
0	0	0	0	0	0	0	0	0
1	0	0	0	0	0	0	1	0
1	0	1	0	0	0	0	0	0
1	0	1	0	0	0	0	1	0
0	0	0	0	0	0	0	1	0
0	0	0	0	0	0	0	0	0
0	0	0	0	0	0	0	0	0
0	0	0	0	0	0	0	0	0
0	0	0	0	0	0	0	0	0
1	0	0	0	0	0	0	0	0
1	0	0	0	0	0	0	0	0
0	0	0	0	0	0	0	0	0
0	0	0	0	0	0	0	0	0
0	0	0	0	0	0	0	0	0
1	1	0	0	0	0	0	0	0
1	0	0	0	0	0	0	0	0
1	0	0	0	0	0	0	0	0
0	0	0	0	0	0	0	0	0
1	0	0	0	0	0	0	0	0
0	0	0	0	0	0	0	0	0
0	1	0	0	0	0	0	0	0
1	0	0	0	0	0	0	0	0
0	0	0	0	0	0	0	0	0
0	0	0	0	0	0	1	0	0
1	0	0	0	0	0	0	0	0
0	1	0	0	0	0	0	1	0
0	0	0	0	0	0	0	0	0
1	0	0	0	0	0	0	0	0
1	0	0	0	0	0	0	0	0
0	1	0	0	0	0	0	0	0
1	0	0	0	0	0	0	0	0
0	0	0	0	0	0	0	0	0
1	0	0	0	0	0	0	0	0

1	0	1	0	0	0	0	1	0
0	1	0	0	0	0	0	0	0
1	0	0	0	0	0	0	0	0
0	0	0	0	0	0	0	0	0
0	0	0	0	0	0	0	0	0
0	0	0	0	0	0	0	0	0
0	0	0	0	0	0	0	1	0
1	0	0	0	0	0	0	0	0
0	0	0	0	0	0	0	0	0
0	1	0	0	0	0	0	0	0
0	0	0	0	0	0	0	0	0
0	0	0	0	0	0	0	0	0
0	0	0	0	0	0	0	1	0
0	0	0	0	1	0	0	0	0
0	0	0	0	0	0	0	0	0
1	0	0	0	0	0	0	0	0
0	0	0	0	0	0	0	0	0
1	0	0	0	0	0	0	0	0
0	1	0	0	0	0	0	0	0
0	0	0	0	0	0	0	0	0
0	0	0	0	0	0	0	0	0
0	0	0	0	0	0	0	0	0
1	0	0	0	0	0	0	0	0
0	0	0	0	0	0	0	0	0
0	0	0	0	0	0	0	0	0
1	0	0	0	0	0	0	0	0
1	0	0	0	0	0	0	0	0
0	0	0	0	0	0	0	0	0
1	0	0	0	0	0	0	0	0
0	0	0	0	0	0	0	0	0
0	0	0	0	0	0	0	0	0
1	0	0	0	0	0	0	1	0
0	0	0	0	0	0	0	0	0
0	0	0	0	0	0	0	0	0
0	0	0	0	0	0	1	0	0
0	0	0	0	0	0	0	0	0
0	0	0	0	0	0	0	0	0
0	0	0	0	0	0	0	0	0
1	0	0	0	0	0	0	0	0
0	0	0	0	0	0	0	0	0
1	0	0	0	0	0	0	0	0
1	0	0	0	0	0	0	0	0
0	0	0	0	0	0	0	0	0
1	0	0	0	0	0	0	0	0
1	0	0	0	0	0	0	0	0
1	0	0	0	0	0	0	0	0
1	0	0	0	0	0	0	0	0
0	0	0	0	0	0	0	0	0
0	0	0	0	0	0	0	0	0
1	0	0	0	0	0	0	0	0
0	0	0	0	0	0	0	0	0
0	0	0	0	0	0	0	1	0
1	0	0	0	0	0	0	0	0
0	0	0	0	0	0	0	1	0
0	0	0	0	0	0	0	0	0
0	0	0	0	0	0	0	0	0

0	0	0	0	0	0	0	0	0
0	0	0	0	0	0	0	0	0
0	0	0	0	0	0	0	0	0
1	0	0	0	0	0	0	0	0
0	0	0	0	0	0	0	0	0
1	0	0	0	0	0	0	0	0
0	0	0	0	0	0	0	0	0
0	0	0	0	0	0	0	0	0
1	0	0	0	0	0	0	0	0
0	0	0	0	0	0	0	0	0
0	0	0	0	0	0	0	0	0
1	0	0	0	0	0	0	0	0
0	0	0	0	0	0	0	0	0
1	0	0	0	0	0	0	0	0
0	0	0	0	0	0	0	0	0
0	0	0	0	0	0	0	0	0
0	0	0	0	0	0	0	0	0
0	0	0	0	0	0	0	0	0
1	0	0	0	0	0	0	0	0
0	0	0	0	0	0	0	0	0
1	0	1	0	0	0	0	0	0
0	0	0	0	0	0	0	0	0
1	0	0	0	0	0	0	0	0
0	0	0	0	0	0	0	0	0
0	0	0	0	0	0	0	0	0
1	0	0	0	0	0	0	0	0
0	0	0	0	0	0	0	0	0
0	0	0	0	0	0	0	0	1
0	0	0	0	0	0	0	0	0
1	0	0	0	0	0	0	1	1
1	0	0	0	0	0	0	0	0
0	0	0	0	0	0	0	0	0
1	0	0	0	0	0	0	0	0
0	1	0	0	0	0	0	0	0
0	0	0	0	0	0	0	0	0
1	0	0	0	0	0	0	0	0
0	0	0	0	0	0	0	1	0
1	0	0	0	0	0	0	0	0
1	0	0	0	0	0	0	0	0
1	0	0	0	0	0	0	0	0
0	0	1	0	0	0	0	0	0
0	0	0	0	0	0	0	0	1
0	0	0	0	0	0	0	0	0
0	0	0	0	0	0	0	0	0
1	0	0	0	0	0	0	0	0
0	0	0	0	0	0	0	0	0
0	1	0	0	0	0	0	0	0
0	0	0	0	0	0	0	0	0
1	0	0	0	0	0	0	0	0
1	0	0	0	0	0	0	0	0
0	0	0	0	0	0	0	0	0
0	0	0	0	0	0	0	0	0
1	0	0	0	0	0	0	0	0
1	0	0	0	0	0	0	0	0
0	0	0	0	0	0	0	0	0
1	0	0	0	0	0	0	0	0

0	0	0	0	0	0	0	0	0
0	0	0	0	0	0	0	0	0
1	0	0	0	0	0	0	0	0
0	0	0	0	0	0	0	0	0
0	0	0	0	0	0	0	0	0
0	0	0	0	0	0	0	0	0
1	0	0	0	0	0	0	0	0
0	0	0	0	0	0	0	0	0
0	0	0	0	0	0	0	0	0
0	0	0	0	0	0	0	0	0
0	0	0	0	0	0	1	0	0
1	0	0	0	0	0	0	0	0
1	0	0	0	1	1	0	0	0
0	0	0	0	0	0	0	0	0
1	0	0	0	0	0	0	0	0
1	0	0	0	0	0	0	1	0
0	0	0	0	0	0	0	0	0
0	1	0	0	0	0	0	0	0
1	0	0	0	0	0	0	0	0
0	0	0	0	0	0	0	0	0
1	0	0	0	0	0	0	0	0
1	0	0	0	0	0	0	0	0
0	0	0	0	0	0	0	0	0
0	0	0	0	0	0	0	0	0
0	0	0	0	0	0	0	1	0
0	0	0	0	0	0	0	0	0
0	0	0	0	0	0	0	0	0
1	0	0	0	0	0	0	0	0
1	0	0	0	0	0	0	0	0
0	0	0	0	0	0	0	0	0
0	0	0	0	0	0	0	0	0
0	1	0	0	0	0	0	0	0
0	0	0	0	0	0	0	0	0
1	0	0	0	0	0	0	0	0
0	0	0	0	0	0	0	0	0
0	0	0	0	0	0	0	0	0
1	0	0	0	0	0	0	0	0
0	0	0	0	0	0	0	0	0
1	0	0	0	0	0	0	0	1
0	0	0	0	0	0	0	0	0
0	0	0	0	0	0	0	0	0
1	0	0	0	0	0	0	0	0
0	0	0	0	0	0	0	0	0
0	0	0	0	0	0	0	0	0
0	0	0	0	0	0	0	0	0
0	0	0	0	0	0	1	0	0
0	0	1	0	0	0	0	0	0
0	0	0	0	0	0	0	0	0
0	0	0	0	0	0	0	0	0
0	0	0	0	0	0	0	0	0
0	0	0	0	0	0	0	0	0
1	0	0	0	0	0	0	0	0
0	0	0	0	0	0	0	0	1
0	0	0	0	0	0	0	0	0
0	0	0	0	0	0	0	0	0
1	0	0	0	0	0	0	0	0

1	0	0	0	0	0	0	0	0
0	0	0	0	0	0	0	0	0
0	0	0	0	0	0	0	0	0
0	1	0	0	0	0	0	0	0
0	0	0	0	0	0	0	1	0
0	0	0	0	0	0	0	0	0
0	0	0	0	0	0	0	0	0
1	0	0	0	0	0	0	0	0
1	0	0	0	0	0	0	0	0
1	0	0	0	0	0	0	0	0
1	0	0	0	0	0	0	0	0
0	0	0	0	0	0	0	0	0
0	0	0	0	0	0	0	0	0
1	0	0	0	0	0	0	0	0
0	0	0	0	0	0	1	1	0
0	0	0	0	0	0	0	0	1
0	0	0	0	0	0	0	1	0
0	0	0	0	0	0	0	0	0
0	0	0	0	0	0	0	0	0
1	0	0	0	0	0	0	0	0
0	0	0	0	0	0	0	0	0
0	0	0	0	0	0	0	0	0
0	0	0	0	0	0	0	0	0
0	0	0	0	0	0	0	0	0
0	0	0	0	0	0	0	0	0
0	0	0	0	0	0	0	1	0
0	0	0	0	0	0	0	0	0
0	0	0	0	0	0	0	0	0
0	0	0	0	0	0	0	0	0
0	0	0	0	0	0	0	0	0
1	0	0	0	0	0	0	0	0
0	0	0	0	0	0	0	0	0
0	0	0	0	0	0	0	0	0
0	0	0	0	0	0	0	0	0
0	0	0	0	0	0	0	0	0
1	0	0	0	0	0	0	0	0
0	0	0	0	0	0	0	0	0
0	0	0	0	0	0	0	0	0
1	0	1	0	0	0	0	0	0
0	0	0	0	0	0	0	0	0
0	0	0	0	0	0	0	0	0
1	0	0	0	0	0	0	0	0
0	0	0	0	0	0	0	0	0
1	0	0	0	0	0	0	0	0
0	0	0	0	0	0	0	0	0
0	0	0	0	0	0	0	0	0
0	0	0	0	0	0	0	1	0
1	0	0	0	1	0	0	0	0
0	0	0	0	0	0	0	0	0
0	1	0	0	0	0	1	0	0
1	0	0	0	0	0	0	0	0
1	0	0	0	0	0	0	0	0
1	0	0	0	0	0	0	0	0
0	0	0	0	0	0	0	0	0
0	0	0	0	0	0	0	0	0
0	0	0	0	0	0	0	0	0

0	0	0	0	0	0	0	0	0
1	0	0	0	0	0	0	0	0
0	1	0	0	0	0	0	0	0
1	0	1	0	0	0	0	0	0
1	0	0	0	0	0	0	0	0
0	0	0	0	0	0	0	0	0
0	0	0	0	0	0	0	0	0
0	0	0	0	0	0	0	1	0
1	0	0	0	0	0	0	0	0
0	0	0	0	0	0	0	0	0
1	0	0	0	0	0	0	0	0
0	0	0	0	0	0	0	0	0
1	0	0	0	0	0	0	1	0
0	0	0	0	0	0	0	0	0
0	0	0	0	0	0	0	0	0
0	0	0	0	0	0	0	1	0
0	0	0	0	0	0	0	0	0
0	0	0	0	0	0	0	0	0
0	0	0	0	0	0	0	0	0
0	0	0	0	0	0	0	0	0
0	0	0	0	0	0	0	0	0
1	0	0	0	0	0	0	0	0
0	0	0	0	0	0	0	0	0
1	0	0	0	0	0	0	0	0
0	0	0	0	0	0	0	0	0
0	0	0	0	1	0	0	0	0
0	0	0	0	0	0	0	0	0
1	0	0	0	0	0	0	0	0
1	0	0	0	0	0	0	0	0
0	0	0	0	0	0	0	0	0
0	0	0	0	0	0	0	0	0
0	1	0	0	0	0	0	0	0
0	0	0	0	0	0	0	0	0
0	0	0	0	0	0	0	0	0
0	0	0	0	0	0	0	0	0
0	0	0	0	0	0	0	0	0
0	0	0	0	0	0	0	0	0
0	0	0	0	0	0	0	0	0
1	0	0	0	0	0	0	0	0
0	0	0	0	0	0	0	0	0
0	0	0	0	0	0	0	0	0
0	0	0	0	0	0	0	1	0
0	0	0	0	0	0	0	1	0
0	0	0	0	0	0	0	0	0
0	1	0	0	0	0	0	0	0
1	0	1	0	0	0	0	0	0
1	0	0	0	0	0	0	0	0
0	0	1	0	0	0	0	0	0
1	0	0	0	0	0	0	0	0
1	0	0	0	0	0	0	0	0
0	0	0	0	0	0	0	0	0
0	0	0	0	0	0	0	0	0
0	0	0	0	0	0	0	0	0
0	0	0	0	0	0	0	0	0
0	0	0	0	0	0	0	0	0
0	0	0	0	1	0	0	0	0

0	0	0	0	0	0	0	0	0
0	0	0	0	0	0	0	0	0
0	0	0	0	0	0	0	0	0
1	0	0	0	0	0	0	0	0
0	0	0	0	0	0	0	0	0
0	0	0	0	0	0	0	0	0
0	0	0	0	0	0	0	0	0
0	0	0	0	0	0	0	0	0
0	0	0	0	0	0	0	0	0
1	0	0	0	0	0	0	0	0
0	0	0	0	0	0	0	1	0
0	0	0	0	0	0	0	0	0
0	0	0	0	0	0	0	0	0
0	1	0	0	0	0	0	0	0
0	0	0	0	0	0	0	0	0
0	0	0	0	0	0	0	0	0
0	0	0	0	0	0	0	0	0
0	0	0	0	0	0	0	0	0
0	0	0	0	0	0	0	0	0
1	0	0	0	0	0	0	0	0
1	0	0	0	0	0	0	0	0
0	0	0	0	0	0	0	1	0
0	0	0	0	0	0	0	0	0
0	0	0	0	0	0	0	0	0
1	0	0	0	0	0	0	0	0
1	0	0	0	0	0	0	0	0
0	1	1	0	0	0	0	0	0
0	0	0	0	0	0	0	1	0
0	0	0	0	0	0	0	0	0
1	0	0	0	0	0	0	0	0
0	0	0	0	0	0	0	1	0
1	0	0	0	0	0	0	0	0
1	0	0	0	0	0	0	0	0
0	0	0	0	0	0	0	0	0
0	0	0	0	0	0	0	0	0
0	0	0	0	0	0	0	0	0
1	0	0	0	0	0	0	0	0
0	0	0	0	0	0	0	0	0
0	0	0	0	0	0	0	1	0
0	0	0	0	0	0	0	0	0
0	1	0	0	0	0	0	0	0
1	0	0	0	0	0	0	0	0
1	0	0	0	0	0	0	0	0
0	0	0	0	0	0	0	0	0
0	0	0	0	0	0	0	1	0
1	0	0	0	0	0	0	0	0
0	0	0	0	0	0	0	1	0
0	0	0	0	0	0	0	0	0
0	0	0	0	0	0	0	0	0
0	0	0	0	0	0	0	0	0
0	0	0	0	0	0	0	0	0
0	0	0	0	0	0	0	1	0
0	0	0	0	0	0	0	0	0
0	0	0	0	0	0	0	0	0
0	0	0	0	0	0	0	0	0
1	0	0	0	0	0	0	0	0

0	0	0	0	0	0	0	1	0
1	0	0	0	0	0	0	0	0
0	0	0	0	0	0	0	0	0
0	0	0	0	0	0	0	1	0
0	0	0	0	0	0	0	0	0
0	0	0	0	0	0	0	0	0
0	0	0	0	0	0	0	0	0
0	0	0	0	0	0	0	0	0
1	0	0	0	0	0	0	0	0
0	1	0	0	0	0	0	1	0
1	0	0	0	0	0	0	1	0
0	0	0	0	0	0	0	0	0
0	0	0	0	0	0	0	0	0
0	0	0	0	0	0	0	0	0
0	0	0	0	0	0	0	0	0
0	0	0	0	0	0	0	0	0
0	1	0	0	0	0	0	0	0
0	0	0	0	0	0	0	0	0
1	0	0	0	0	0	0	0	0
1	0	0	0	0	0	0	0	0
1	1	0	0	0	0	0	0	0
1	0	0	0	1	0	0	0	0
0	0	0	0	0	0	0	0	0
0	1	0	0	0	0	0	0	0
0	0	0	1	0	0	0	0	0
0	0	0	0	0	0	0	0	0
1	0	0	0	1	0	0	0	0
0	0	0	0	0	0	0	0	0
0	0	0	0	0	0	0	0	0
0	0	0	0	0	0	0	0	0
1	0	0	0	0	0	0	0	0
1	0	0	0	0	0	0	0	0
0	0	0	0	0	0	0	0	0
0	0	0	0	0	0	0	0	0
0	0	0	0	0	0	0	0	0
0	0	0	0	0	0	0	0	0
0	0	0	0	0	0	0	0	0
0	0	0	0	0	0	0	0	0
0	0	0	0	0	0	0	0	0
0	0	0	0	0	0	0	0	0
0	0	0	0	0	0	0	0	0
0	0	0	0	0	0	0	0	0
0	0	0	0	1	0	0	0	0
1	0	0	0	0	0	0	0	0
0	0	0	0	0	0	0	0	0
0	0	0	0	0	0	0	0	0
0	0	0	0	0	0	0	0	0
0	0	0	0	0	0	0	0	0
0	0	0	0	0	0	0	0	0
1	0	0	0	0	0	0	0	0
0	0	0	0	0	0	0	0	0
0	0	0	0	0	0	0	0	0
0	0	0	0	0	0	0	0	0
0	0	0	0	0	0	0	0	0
1	0	0	0	0	0	0	0	0
0	0	0	0	0	0	0	0	0

0	0	0	0	0	0	0	0	0
0	0	0	0	0	0	0	0	0
0	0	0	0	0	0	0	0	0
0	1	0	0	0	0	0	1	0
0	0	0	0	0	0	0	0	0
1	0	0	0	0	0	0	0	0
0	0	0	0	0	0	0	0	0
1	0	0	0	0	0	0	0	0
0	0	0	0	0	0	0	0	0
0	0	0	0	0	0	0	0	0
0	0	0	0	0	0	0	0	0
1	0	1	0	0	0	0	0	0
0	0	0	0	0	0	0	0	0
1	1	0	0	0	0	0	0	0
0	0	0	0	0	0	0	0	0
0	0	0	0	0	0	0	1	0
0	0	0	0	1	0	0	0	0
1	0	0	0	0	0	0	0	0
0	0	0	1	0	0	0	0	0
1	0	0	0	1	0	0	0	0
0	0	0	0	0	0	0	0	0
0	0	0	0	0	0	0	0	0
1	0	0	0	0	0	0	0	0
0	0	0	0	0	0	0	0	0
1	0	0	0	0	0	0	0	0
0	0	0	0	0	0	0	0	0
0	0	0	0	0	0	0	0	0
0	0	0	0	0	0	0	0	0
0	0	0	0	0	0	0	0	0
1	0	0	0	0	0	0	0	0
0	0	0	0	0	0	0	0	0
0	0	0	0	0	0	0	0	0
0	0	0	0	0	0	0	0	0
0	0	0	0	0	0	0	0	0
0	0	0	0	0	0	0	0	0
1	0	0	0	0	0	0	0	0
1	0	0	0	0	0	0	1	0
0	0	0	0	0	0	0	0	0
0	0	0	0	0	0	0	0	0
0	0	0	0	0	0	0	1	0
1	0	0	0	0	0	0	0	0
1	0	0	0	0	0	0	0	0
0	0	0	0	0	0	0	0	0
1	0	0	0	0	0	0	0	0
0	0	0	0	0	0	0	0	0
1	0	0	0	0	0	0	0	0
1	0	0	0	1	0	0	0	0
0	0	0	0	0	0	0	0	0
0	1	0	0	0	0	1	0	0
0	0	0	0	0	0	0	0	0
0	1	0	0	0	0	0	1	0
0	0	0	0	0	0	0	0	0
1	0	0	0	0	0	0	0	0
0	0	0	0	0	0	0	1	0
0	0	0	0	1	0	0	0	0
0	0	0	0	0	0	0	0	0

0	0	0	0	0	0	1	0	0
0	0	0	0	0	0	0	0	0
0	0	0	0	0	0	0	0	0
1	0	0	0	0	0	0	0	0
0	0	0	0	0	0	0	0	0
0	0	0	0	0	0	0	0	0
0	0	0	0	0	0	0	1	0
0	0	0	0	0	0	0	0	0
1	0	0	0	0	0	0	0	0
0	0	0	0	0	0	0	0	0
1	0	0	0	0	0	0	0	0
1	0	0	0	0	0	0	0	0
0	0	0	0	0	0	0	0	0
1	0	0	0	0	0	0	0	0
0	0	0	0	0	0	0	0	0
1	0	0	0	0	0	0	0	0
1	0	0	0	0	0	0	0	0
0	0	0	1	0	0	0	0	0
0	1	0	0	0	0	1	0	0
0	0	0	0	0	0	1	0	0
1	0	0	0	1	0	0	0	0
1	0	0	0	0	0	0	0	0
1	0	0	0	0	0	0	0	0
0	0	0	0	0	0	0	0	0
0	0	0	0	0	0	0	0	0
0	1	0	0	0	0	1	0	0
1	0	0	0	1	0	1	0	0
1	0	0	0	0	0	0	0	0
1	0	0	0	1	0	0	1	0
0	0	0	0	0	0	0	0	0
0	0	0	0	0	0	0	0	0
0	0	0	0	0	0	0	0	0
0	0	0	0	0	0	0	0	0
1	0	0	0	0	0	0	0	0
0	0	0	0	0	0	0	0	0
1	0	0	0	0	0	0	0	0
1	0	0	0	0	0	0	0	0
0	0	0	0	0	0	0	0	0
0	0	0	0	0	0	0	0	0
0	0	0	0	0	0	1	1	0
0	0	0	0	0	0	0	1	0
1	0	0	0	0	0	0	0	0
0	0	0	0	0	0	0	0	0
0	0	1	0	0	0	0	0	0
1	0	0	0	0	0	0	0	0
0	0	0	0	0	0	0	0	0
0	0	0	0	0	0	0	1	0
1	0	0	0	0	0	0	1	0
1	0	0	0	0	0	0	1	1
0	0	0	0	0	0	0	0	0
0	0	0	0	0	0	0	0	0
1	0	0	0	0	0	0	0	0
0	0	0	0	0	0	0	1	0
0	0	0	0	0	0	0	0	0
1	0	0	0	0	0	0	0	0
0	0	0	0	0	0	0	0	0

0	0	0	0	0	0	0	0	0
0	0	0	0	0	0	0	0	0
1	0	0	0	0	0	0	0	0
0	0	0	0	0	0	0	0	0
0	0	0	0	0	0	0	1	0
0	0	0	0	0	0	0	0	0
0	0	0	0	0	0	0	0	0
1	0	0	0	0	0	0	0	0
0	0	0	0	0	0	0	0	0
0	0	0	0	0	0	0	0	0
0	0	0	0	0	0	0	0	0
0	0	0	0	0	0	0	0	0
0	0	0	0	0	0	0	0	0
0	0	0	0	0	0	0	0	0
0	0	0	0	0	0	0	0	0
0	0	0	0	0	0	0	0	0
1	0	0	0	1	0	0	0	0
0	0	0	0	0	0	0	0	0
1	0	0	0	0	0	0	0	0
0	0	0	0	0	0	0	1	0
0	0	0	0	0	0	0	0	0
1	0	0	0	0	0	0	0	0
0	0	0	0	0	0	0	0	0
0	0	0	0	0	0	0	0	0
0	0	0	0	0	0	0	0	0
0	1	0	0	0	0	0	0	0
1	0	0	0	0	0	0	0	0
0	0	0	0	0	0	0	0	1
1	0	0	0	0	0	0	0	0
1	0	0	0	0	0	0	0	0
0	0	0	0	0	0	0	0	0
0	0	0	0	0	0	0	0	0
0	0	0	0	1	0	0	0	0
0	0	0	0	0	0	0	0	0
0	0	0	0	0	0	0	1	0
1	0	0	0	0	0	0	0	0
0	0	0	0	0	0	0	0	0
0	1	0	0	0	0	0	0	0
0	0	0	0	0	0	0	0	0
0	0	0	0	0	0	0	1	0
0	0	0	1	0	0	0	0	0
0	0	0	0	0	0	0	0	0
0	0	0	0	0	0	0	1	0
1	0	0	0	0	0	0	0	0
0	0	0	0	0	0	0	0	0
0	0	0	0	0	0	0	0	0
0	0	0	0	0	0	0	0	0
0	0	0	0	0	0	0	0	0
0	0	0	0	0	0	0	1	0
0	0	0	0	1	0	0	0	0
0	1	0	0	0	0	0	0	0
0	0	0	0	0	0	0	0	0
0	0	0	0	0	0	0	0	0
0	0	0	0	0	0	0	0	0
0	0	0	0	0	0	0	0	0
0	0	0	1	0	0	0	0	0

0	0	0	0	0	0	0	0	0
0	1	0	0	0	0	0	0	0
0	0	0	0	0	0	0	0	0
1	0	0	0	0	0	0	0	0
0	0	0	0	0	0	0	0	0
0	0	0	0	0	0	0	0	0
1	0	0	0	0	0	0	0	0
0	0	0	0	0	0	0	0	0
0	0	0	0	0	0	0	0	0
0	0	0	0	0	0	1	0	0
0	0	0	0	0	0	0	0	1
1	0	0	0	0	0	0	0	0
1	0	0	0	0	0	0	0	0
0	1	0	0	0	0	0	0	0
1	0	0	0	0	0	0	0	0
0	0	0	0	0	0	1	1	1
0	0	0	0	0	0	0	0	0
0	0	0	0	0	0	0	0	0
0	0	0	0	0	0	1	0	0
1	0	0	0	0	0	0	0	0
0	0	0	0	1	0	0	0	0
0	0	0	0	0	0	0	0	0
0	0	0	0	0	0	0	1	0
0	0	0	0	0	0	0	0	0
1	0	1	0	0	0	0	0	0
0	0	0	0	0	0	0	0	0
0	0	0	0	0	0	0	0	0
1	0	0	0	0	0	0	0	0
0	1	0	0	0	0	0	0	0
0	0	0	0	0	0	0	1	0
0	0	0	1	0	0	1	0	0
0	0	0	0	0	0	0	0	0
0	0	0	0	0	0	0	0	0
0	0	0	0	0	0	0	0	0
0	0	0	0	0	0	0	0	0
1	0	0	0	0	0	0	0	0
0	0	0	0	0	0	0	0	0
1	0	0	0	1	0	0	0	0
0	0	0	0	0	0	0	0	0
0	0	0	0	0	0	0	0	0
1	0	0	0	0	0	0	1	0
1	0	0	0	0	0	0	1	0
0	0	0	0	0	0	0	0	0
0	0	0	0	0	0	0	0	0
0	0	0	0	0	0	0	1	0
0	0	0	0	0	0	0	0	0
0	0	0	0	0	0	0	0	0
0	0	0	0	0	0	0	0	0
0	0	0	0	0	0	0	0	0
1	1	0	0	1	0	0	0	0
0	0	0	0	0	0	0	0	0
1	0	0	0	0	0	0	1	0
0	0	0	0	0	0	0	0	0
0	0	0	0	0	0	0	1	0
0	0	0	0	0	0	0	0	0
0	0	0	1	0	0	0	0	0

1	0	0	0	0	0	0	0	0
0	0	0	0	0	0	0	0	0
0	0	0	0	0	0	0	1	0
0	0	0	0	0	0	0	0	0
0	0	0	0	0	0	0	0	0
0	0	0	0	0	0	0	0	0
0	0	0	0	0	0	0	0	0
0	0	0	0	0	0	0	0	0
0	0	0	0	0	0	0	1	0
0	0	0	0	0	0	0	0	0
1	0	0	0	0	0	0	1	0
0	0	0	0	0	0	0	0	0
0	0	0	0	0	0	0	0	0
1	0	0	0	0	0	0	1	0
1	0	0	0	0	0	0	0	0
0	0	0	0	0	0	0	0	0
0	0	0	0	0	0	0	1	0
0	0	0	0	0	0	0	0	0
0	0	0	0	0	0	0	0	0
1	0	0	0	0	0	0	0	0
0	0	0	0	0	0	0	0	0
0	0	0	0	0	0	0	0	0
0	0	0	0	0	0	0	0	0
0	0	0	0	0	0	0	0	0
0	0	0	0	0	0	0	1	0
1	0	0	0	0	0	0	0	0
0	0	0	0	0	0	0	0	0
0	0	0	0	0	0	0	0	0
0	0	0	0	0	0	0	0	0
1	1	0	0	1	0	0	0	0
0	0	0	0	0	0	0	0	0
0	0	0	0	0	0	0	0	0
0	1	0	0	0	0	0	0	0
0	0	0	0	0	0	0	0	1
0	0	0	0	0	0	0	0	0
0	0	0	0	0	0	0	0	0
0	0	0	0	0	0	0	1	0
0	0	0	0	0	0	0	0	0
0	0	0	0	0	0	0	0	0
0	0	0	0	0	0	0	0	0
0	0	0	0	0	0	0	0	0
0	0	0	0	0	0	0	0	0
1	0	0	0	0	0	0	0	0
0	0	0	0	0	0	0	0	0
1	0	0	0	0	0	0	0	0
0	0	0	0	0	0	0	1	0
0	0	1	0	0	0	0	0	0
1	0	0	0	0	0	0	0	0
0	0	0	0	0	0	0	1	0
0	0	0	0	0	0	0	0	0
0	0	0	0	0	0	0	1	0
0	0	0	0	0	0	0	0	0
0	0	0	0	0	0	0	0	0
0	0	0	0	0	0	0	0	0
0	0	0	0	0	0	0	0	0
0	1	0	0	0	0	0	0	0

1	0	0	0	0	0	0	0	0
0	0	0	0	0	0	1	1	0
0	0	0	0	0	0	0	0	0
0	0	0	0	0	0	0	0	0
1	0	0	0	0	0	0	0	0
1	0	0	0	0	0	1	0	0
0	0	0	0	0	0	0	0	0
0	0	0	0	0	0	0	1	0
0	0	0	0	0	0	0	1	0
0	0	0	0	0	0	1	0	0
1	0	0	0	0	0	0	0	0
0	0	0	0	0	0	0	0	0
0	1	0	0	0	0	0	0	0
0	0	0	0	0	0	0	1	0
0	0	0	0	0	0	0	0	0
0	0	0	0	0	0	0	0	0
1	0	0	0	0	0	0	0	0
1	0	0	0	0	0	0	1	0
0	0	0	0	0	0	0	1	0
0	0	0	0	0	0	0	0	0
1	0	0	0	0	0	0	0	0
0	0	0	0	0	1	0	0	0
0	0	0	0	0	0	0	0	0
1	0	0	0	1	0	0	0	0
0	0	0	0	0	0	0	0	0
0	0	0	0	0	0	0	0	0
1	0	0	0	0	0	0	1	0
0	0	0	0	0	0	0	0	0
0	0	0	0	0	0	0	0	0
0	1	0	0	0	0	0	1	0
0	0	0	0	0	0	0	0	0
0	0	0	0	0	0	0	0	0
0	0	0	0	0	0	0	0	0
1	0	0	0	0	0	1	0	0
0	0	0	0	0	0	0	0	0
0	1	0	0	0	0	0	0	0
0	0	0	0	0	0	0	0	0
0	0	0	0	1	0	0	0	0
0	0	0	0	0	0	0	0	0
0	0	0	0	0	0	0	1	0
0	0	0	0	0	0	0	0	0
0	0	0	0	0	0	0	0	0
0	0	0	0	0	0	0	1	1
0	0	0	0	0	0	0	0	0
0	0	0	0	0	1	0	0	0
0	0	0	0	1	0	0	0	0
0	0	0	0	0	0	0	0	1
0	0	0	0	0	0	0	0	0
0	0	0	0	0	0	0	0	0
0	0	0	0	0	0	0	0	0
0	0	0	1	0	0	0	0	0
0	0	0	0	0	0	0	0	0
0	0	0	0	0	0	0	0	0
0	0	0	1	0	0	1	0	0
0	0	0	0	1	0	0	0	0
1	0	0	0	0	0	0	0	0

1	0	0	0	0	0	1	0	0
1	0	0	0	0	0	0	0	0
0	0	0	0	0	0	0	0	0
0	0	0	0	0	0	0	0	0
0	0	0	0	1	0	0	1	0
0	1	0	0	0	0	0	0	0
0	0	0	0	0	0	0	0	0
0	0	0	0	0	0	0	0	0
0	0	0	0	0	0	0	0	0
0	0	0	0	0	0	0	0	0
1	0	0	0	0	0	0	0	0
0	0	0	0	0	0	0	0	0
0	0	0	0	0	0	0	0	0
0	0	0	0	0	0	0	0	0
0	0	0	0	0	0	0	0	0
0	0	0	0	0	1	0	1	0
0	0	0	0	0	0	0	0	0
0	0	0	0	0	0	0	0	0
0	0	0	0	0	0	0	0	0
1	0	0	1	0	0	0	1	0
0	0	0	0	0	0	0	0	0
0	0	0	0	0	0	0	0	0
0	0	0	0	0	0	0	0	0
0	0	0	0	0	0	0	0	0
0	0	0	0	0	0	0	0	0
0	0	0	0	0	0	0	0	0
1	0	0	0	0	0	0	0	0
1	0	0	0	0	0	0	0	0
1	0	0	0	0	0	0	0	0
0	1	0	0	0	0	0	0	0
0	0	0	0	0	0	0	1	0
0	1	0	0	0	0	0	1	0
0	0	0	0	1	0	0	0	0
1	0	0	0	0	0	0	0	0
0	0	0	0	0	0	0	0	0
0	0	0	0	0	0	0	0	0
0	1	0	0	0	0	0	0	0
1	0	0	0	0	0	0	0	0
1	0	0	0	0	0	0	0	0
0	0	0	0	0	0	0	0	0
0	0	0	0	0	0	0	0	0
0	0	0	0	0	0	0	0	0
0	0	0	0	0	0	0	0	0
0	0	0	0	0	0	0	0	0
1	0	0	0	0	0	0	0	0
0	0	0	0	0	0	0	0	0
1	0	0	0	0	0	0	0	0
0	0	0	0	0	0	0	0	0
0	0	0	0	1	0	0	0	0
1	0	0	0	0	0	0	0	0
1	0	0	0	0	0	0	1	0
1	1	0	0	0	0	0	0	0
0	0	0	0	0	0	0	0	0
0	0	0	0	0	0	0	0	0
0	0	0	0	0	0	0	0	0
0	0	0	0	0	0	0	0	0

1	0	0	0	0	0	0	1	0
0	1	0	0	0	0	0	0	0
0	0	0	0	0	0	0	0	0
0	0	0	0	0	0	0	0	0
0	0	0	0	0	0	0	0	0
0	1	0	0	0	0	0	0	0
1	0	0	0	0	0	0	0	0
0	0	0	0	0	0	0	0	0
0	1	0	0	0	0	0	0	0
0	0	0	0	0	0	0	0	0
0	0	0	0	0	0	0	0	0
0	0	0	0	0	0	0	0	0
0	0	0	0	0	0	0	0	0
0	0	0	0	0	0	0	0	0
1	0	0	0	0	0	0	0	0
0	0	0	0	0	0	0	0	0
0	0	0	0	0	0	1	0	0
0	0	0	0	0	0	0	0	0
0	0	0	0	0	0	0	0	0
0	0	0	0	0	0	0	0	0
0	0	0	0	0	0	0	0	0
1	0	0	0	0	0	0	0	0
0	0	0	0	0	0	1	0	0
0	0	0	0	0	0	0	0	0
0	0	0	1	0	0	0	0	0
1	0	0	0	0	0	0	1	0
0	0	0	0	0	0	0	0	0
0	0	0	0	0	0	0	0	0
0	0	0	0	1	0	0	0	0
0	1	0	0	0	0	0	0	0
0	0	0	0	0	0	0	0	0
1	0	0	0	0	0	0	0	0
0	0	0	0	0	0	0	0	0
0	0	0	0	0	0	0	0	0
0	0	0	0	0	0	0	0	0
1	0	0	0	0	0	0	0	0
0	0	1	0	0	0	0	0	0
0	0	0	0	0	0	0	0	0
0	0	0	0	0	0	0	1	0
0	0	0	0	0	0	0	0	0
0	0	0	0	0	0	0	1	0
1	0	0	0	0	0	0	0	0
0	0	0	0	0	0	0	0	0
1	0	0	0	1	0	0	0	0
0	0	0	0	0	0	0	0	0
1	1	0	0	0	0	0	0	0
0	0	0	0	0	0	0	0	0
0	0	0	0	0	0	0	1	0
1	0	0	0	0	0	0	0	0
0	0	0	0	0	0	0	1	0
0	0	0	0	0	0	0	0	0
1	0	0	1	0	0	0	0	0
1	0	0	1	0	0	0	0	0
1	0	0	1	0	0	0	0	0
0	1	0	0	0	0	0	0	0
0	0	0	0	0	0	0	0	0

0	1	0	0	0	0	0	0	0
1	0	0	0	0	0	0	0	0
1	0	0	0	0	0	0	0	0
0	0	0	0	0	0	0	0	0
0	0	0	0	0	0	0	0	0
0	0	0	0	0	0	0	0	0
0	0	0	0	0	0	0	0	0
0	0	0	0	1	0	0	0	0
0	1	0	0	0	0	0	0	0
0	0	0	0	0	0	0	0	0
1	0	0	0	0	0	0	1	0
0	0	0	0	0	0	0	1	0
1	0	0	0	0	0	0	0	0
0	0	0	0	0	0	0	0	0
0	0	0	0	0	0	0	0	0
0	0	0	0	0	0	0	0	0
0	0	0	0	0	0	0	0	1
0	0	0	0	0	0	0	0	0
0	1	0	0	0	0	0	0	0
0	0	1	0	0	0	0	0	0
1	0	0	0	0	0	0	0	0
0	0	0	0	0	0	0	0	0
0	0	0	0	0	0	0	0	0
0	0	0	0	0	0	0	0	0
0	0	0	0	0	0	1	0	0
0	0	0	0	0	0	0	0	0
0	0	0	0	0	0	0	0	0
0	0	0	0	0	0	0	0	0
0	0	0	0	0	0	0	0	0
0	0	0	0	0	0	0	0	0
0	0	0	0	0	0	0	0	0
0	0	0	0	0	0	0	0	0
1	0	0	0	0	0	0	0	0
0	0	0	0	0	0	0	0	0
0	0	0	0	0	0	0	0	0
0	0	0	0	0	0	0	0	0
0	0	0	0	0	0	0	0	0
0	0	0	0	0	0	1	0	0
1	0	0	0	0	0	0	0	0
0	0	0	0	0	0	0	0	0
1	0	0	0	1	0	0	0	0
1	0	0	0	0	0	0	1	0
0	0	0	0	0	0	0	0	0
0	0	0	0	0	0	0	1	0
0	0	0	0	0	0	0	0	0
0	0	0	0	0	0	0	0	0
1	0	0	0	0	0	0	0	0
1	0	0	0	0	0	0	0	0
1	0	0	1	0	0	0	0	0
0	0	0	0	0	0	0	0	0
0	0	0	0	0	0	0	0	0
0	0	0	0	0	0	0	0	0
0	0	0	0	0	0	0	0	0
1	0	0	0	0	0	0	0	0
0	0	0	0	0	0	0	0	0
1	0	0	0	0	0	0	0	0

1	0	0	0	0	0	0	1	0
0	0	0	0	1	0	0	0	0
1	0	0	0	1	0	0	0	0
0	0	0	0	0	0	0	0	0
0	0	0	0	1	0	0	0	0
0	0	0	0	0	0	0	0	0
0	0	0	0	0	0	0	0	0
0	0	0	0	0	0	0	1	0
0	0	0	0	0	0	0	0	0
1	0	0	0	0	0	0	0	0
1	0	0	0	0	0	0	1	0
0	0	0	0	0	0	0	0	0
0	0	0	0	0	0	0	0	1
0	0	0	0	0	0	0	0	0
0	0	0	0	0	0	0	0	0
0	0	0	1	0	0	1	0	0
0	0	0	0	0	0	1	0	1
1	0	0	0	0	0	0	0	0
0	0	0	0	0	0	0	0	0
0	0	0	0	0	0	0	0	0
1	0	0	0	0	0	0	0	0
1	0	0	0	0	0	0	0	0
0	1	0	0	0	0	0	0	0
1	0	0	0	0	0	0	0	0
0	0	0	0	0	0	0	1	1
0	0	0	0	0	0	0	0	0
0	0	0	0	0	0	0	0	0
0	0	0	0	0	0	0	1	0
0	0	0	0	0	0	0	1	0
1	0	0	0	0	0	0	0	0
0	0	0	0	0	0	0	0	0
0	0	0	0	0	0	0	0	0
1	1	0	0	0	0	0	0	0
0	0	0	0	0	0	0	0	0
1	0	0	0	0	0	0	0	0
0	0	0	0	0	0	0	1	0
0	0	0	0	0	0	0	0	0
0	0	0	0	0	0	1	0	0
1	0	0	0	0	0	0	1	0
0	0	0	0	0	0	0	0	0
0	0	0	0	0	0	0	0	0
0	0	0	1	0	0	0	1	0
0	0	0	0	0	0	0	0	0
0	0	0	0	0	0	0	0	0
1	0	0	0	0	0	0	0	0
0	0	0	0	0	0	0	1	0
1	0	0	0	0	0	0	0	0
0	0	0	0	1	0	0	0	0
0	0	0	0	0	0	0	0	0
0	0	0	0	0	0	0	0	0
0	0	0	0	0	0	0	0	0
0	0	0	0	0	0	0	0	0
0	0	0	0	0	0	0	1	0
0	0	0	0	0	0	0	1	0
0	0	0	0	0	0	0	0	0
0	1	0	0	0	0	0	0	0

1	0	0	0	0	0	0	1	0
1	0	0	0	0	0	0	0	0
0	0	0	0	0	0	0	0	0
0	0	0	0	0	0	0	0	0
1	0	0	0	0	0	0	0	0
0	0	0	0	0	0	0	0	0
0	0	0	0	0	0	0	0	0
1	0	0	0	0	0	0	0	0
0	0	0	0	0	0	0	1	0
0	0	0	0	0	0	0	0	0
0	0	0	0	0	0	0	0	0
0	1	0	0	0	0	0	0	0
0	0	0	0	0	0	0	0	0
1	0	0	0	0	0	0	0	0
0	0	0	0	0	0	0	0	0
0	0	0	0	0	0	0	0	0
1	0	0	0	0	0	0	0	0
0	0	0	0	0	0	0	0	0
1	0	0	0	0	0	0	0	0
0	0	0	0	0	0	0	0	0
0	0	0	0	1	0	1	0	0
0	0	0	0	0	0	0	0	0
0	0	0	0	0	0	0	0	0
1	0	0	0	0	0	0	0	0
0	0	0	0	0	0	0	0	0
1	0	0	0	0	0	0	0	0
0	0	0	0	0	1	0	1	0
0	0	0	0	0	0	0	0	0
1	0	0	0	0	0	0	0	0
0	0	0	0	1	0	0	1	0
1	0	0	1	0	0	0	0	0
1	0	0	0	0	0	0	0	0
0	0	0	0	0	0	0	0	0
0	0	0	0	0	0	0	0	0
0	0	0	0	0	0	0	0	0
0	0	0	0	0	0	0	0	0
0	0	0	0	0	0	1	0	0
0	0	0	0	0	0	0	0	0
0	0	0	0	0	0	0	0	0
0	0	0	0	0	0	0	0	0
0	0	0	0	0	0	0	0	0
0	0	0	0	0	0	0	0	0
0	0	0	0	0	0	0	0	0
0	0	0	0	0	0	0	1	0
1	0	0	0	1	0	0	0	0
0	0	0	0	0	0	0	0	0
1	0	0	0	0	0	0	1	0
1	1	0	0	0	0	0	0	0
0	0	0	0	0	0	0	0	0
0	0	0	0	0	0	0	0	0
0	0	0	0	0	0	0	0	0
0	1	0	0	0	0	0	0	0
0	0	0	0	0	0	0	0	0
0	0	0	0	0	0	0	1	0
0	0	0	0	0	0	0	0	0
0	0	0	0	0	0	0	0	0

0	0	0	0	0	0	0	0	0
1	0	0	0	0	0	0	0	0
1	0	0	1	0	0	0	0	0
0	0	0	0	0	0	0	0	0
0	0	0	0	1	0	0	0	0
0	0	0	0	0	0	0	0	0
0	0	0	0	0	0	0	0	0
0	0	0	0	0	0	0	1	0
0	0	0	0	0	0	1	1	0
1	0	0	0	0	0	0	0	0
1	0	0	0	0	0	0	1	0
0	0	0	0	1	0	0	0	0
0	0	0	0	0	0	0	1	1
0	0	0	0	0	0	0	0	0
0	0	0	0	0	0	0	1	0
1	0	0	1	0	0	0	0	0
1	0	0	0	0	0	0	1	0
0	0	0	0	0	0	0	0	0
0	0	0	0	0	0	0	0	0
0	0	0	0	0	0	0	1	1
0	0	0	0	0	0	0	0	0
0	0	0	0	0	0	0	0	0
0	0	0	0	0	0	0	0	0
1	0	0	0	0	0	0	1	0
0	0	0	0	0	0	0	0	0
0	0	0	0	0	0	0	0	0
0	0	0	0	0	0	0	0	0
0	0	0	0	0	0	0	0	0
1	0	0	0	0	0	0	0	0
0	0	0	0	0	0	0	0	0
0	0	0	0	0	0	0	0	0
0	0	0	0	1	0	0	0	0
0	0	0	0	0	0	0	0	0
0	0	0	0	0	0	0	0	0
0	0	0	0	0	0	0	0	0
1	0	0	0	0	0	0	0	0
0	0	0	0	0	0	0	0	0
0	0	0	0	0	0	0	0	0
0	0	0	0	0	0	0	0	0
0	0	0	0	0	0	0	0	0
0	0	0	0	0	0	0	0	0
1	0	0	0	0	0	1	0	0
1	0	0	0	0	0	0	0	0
0	0	0	0	0	0	0	0	0
1	0	0	0	0	0	0	0	0
1	0	0	0	0	0	0	0	0
0	0	0	0	0	0	0	0	0
0	0	0	0	1	0	0	0	0
0	0	0	0	0	0	0	1	0
0	0	0	0	0	0	0	0	0
1	0	0	0	0	0	0	1	0
0	0	0	0	0	0	0	0	0
1	0	0	0	0	0	0	0	0
0	1	0	0	0	0	0	0	0
0	0	0	0	0	0	0	0	0
0	0	0	0	0	0	0	0	0

0	0	0	0	1	0	0	0	0
0	0	0	0	0	0	0	0	0
0	1	0	0	0	0	0	0	0
0	0	0	0	0	0	0	0	0
0	0	0	0	0	0	0	0	0
0	0	0	0	0	0	0	0	0
0	0	0	0	0	0	0	0	0
1	0	0	1	0	0	0	0	0
0	0	0	0	0	0	0	0	0
0	0	0	0	0	0	0	0	0
0	0	0	0	0	0	0	0	0
0	0	0	0	0	0	0	0	0
0	0	0	0	0	0	0	0	1
0	0	0	0	0	0	0	0	0
0	0	0	0	0	0	0	0	0
1	0	0	0	0	0	0	0	0
0	0	0	0	0	0	0	0	0
1	0	0	0	0	0	0	1	0
0	0	0	0	0	0	0	0	0
0	1	0	0	0	0	1	0	0
0	1	0	0	0	0	0	0	0
0	0	0	0	0	0	0	0	0
0	0	0	0	0	0	0	0	0
0	0	0	0	0	0	0	1	0
0	0	0	0	0	0	0	0	0
0	0	0	0	0	0	0	1	0
1	0	0	0	0	0	0	0	0
1	0	0	0	0	0	0	0	0
1	0	0	0	0	0	0	0	0
0	0	0	0	0	0	0	0	0
0	0	0	0	0	0	0	0	0
0	0	0	0	0	0	0	0	0
0	0	0	0	0	0	0	0	0
0	0	0	0	0	0	0	0	0
0	0	0	1	0	0	1	0	0
1	0	0	0	0	0	1	0	0
0	0	0	0	0	0	0	0	0
0	0	0	0	0	0	0	0	0
0	1	0	0	0	0	0	0	0
0	0	0	0	1	0	0	1	0
0	0	0	0	0	0	0	0	0
0	0	0	0	1	0	0	0	0
0	0	0	0	0	0	0	0	0
0	1	0	0	0	0	0	0	0
0	0	0	0	1	0	0	0	0
0	0	0	0	0	0	0	0	0
0	0	0	0	0	0	0	1	0
0	0	0	0	0	0	0	0	0
0	0	0	0	0	0	0	0	0
0	1	0	0	0	0	0	0	0
0	0	0	0	0	0	0	0	0
0	0	0	0	0	0	0	0	0
0	0	0	0	0	0	0	1	0
0	0	0	0	0	0	1	0	0
0	0	0	0	0	0	0	0	0
0	0	0	0	0	0	0	0	0

0	0	0	0	0	0	0	0	0
0	0	0	1	0	0	0	0	0
0	0	0	0	0	0	0	0	0
0	0	0	0	0	0	0	0	0
0	0	0	0	0	0	0	0	0
0	0	0	0	0	0	0	1	0
0	0	0	0	0	0	0	0	0
0	0	0	0	0	0	0	0	0
0	0	0	0	0	0	0	0	0
0	0	0	0	0	0	0	0	0
0	0	0	0	0	0	0	1	0
0	0	0	0	0	0	0	0	0
0	0	0	0	0	0	0	0	0
0	0	0	0	0	0	0	0	0
0	0	0	0	0	0	0	1	0
0	0	0	0	0	0	0	0	0
0	0	0	0	0	0	0	0	0
0	0	0	0	0	0	0	1	0
0	0	0	0	0	0	0	0	0
0	0	0	0	0	0	0	0	0
0	0	0	0	0	0	0	0	0
0	0	0	0	0	0	0	0	0
0	0	0	0	0	0	0	0	0
0	1	0	0	0	0	0	0	0
0	0	0	0	0	0	0	0	0
0	0	0	0	0	0	0	1	0
0	0	0	0	0	0	0	0	0
0	0	0	0	0	0	0	1	0
1	0	0	0	0	0	0	0	0
0	0	0	0	0	0	0	0	0
0	0	0	0	0	0	0	0	0
0	0	0	0	0	0	0	0	0
0	0	0	0	0	0	0	0	0
0	0	0	0	0	0	0	0	0
0	0	0	0	1	0	0	0	0
0	0	0	0	0	0	0	0	0
0	0	0	0	0	0	0	0	0
0	0	0	0	0	0	0	0	0
0	0	0	0	0	0	0	0	0
0	0	0	0	0	0	0	0	0
0	0	0	0	0	0	0	0	0
0	0	0	0	0	0	0	0	0
0	0	0	0	0	0	0	1	0
0	0	0	0	0	0	0	0	0
0	0	0	0	0	0	0	0	0
0	0	0	0	0	0	0	0	0
0	0	0	0	1	0	0	0	0
0	0	0	0	0	0	0	0	0
0	0	0	0	0	0	0	0	0
0	0	0	0	0	0	0	0	0
0	0	0	0	0	0	1	0	0
0	0	0	0	0	0	0	1	1
0	0	0	0	0	0	0	0	0
0	0	0	0	0	0	0	1	0
1	0	0	0	0	0	0	0	0
0	0	0	0	0	0	0	0	0

0	0	0	0	0	0	0	0	0
1	0	0	0	0	0	0	0	0
0	0	0	0	0	0	0	0	0
0	0	0	0	0	0	0	0	0
0	1	0	0	0	0	0	1	0
0	0	0	0	0	0	0	0	0
0	0	0	0	1	0	0	0	0
0	0	0	0	0	0	0	0	0
0	1	0	0	0	0	0	1	0
0	0	0	0	0	0	0	0	0
0	0	0	0	0	0	0	0	0
0	0	0	0	0	0	0	0	0
0	0	0	0	0	0	0	0	0
1	0	0	0	0	0	0	0	0
0	0	0	0	0	0	0	0	0
0	0	0	0	0	0	0	0	0
0	0	0	0	0	0	0	0	0
0	0	0	0	0	0	0	0	0
1	1	0	0	0	0	0	0	0
0	0	0	0	0	0	0	0	0
0	0	0	0	0	0	0	0	0
0	0	0	0	0	0	0	0	0
0	0	0	0	0	0	0	0	0
0	0	0	0	0	0	0	1	0
0	0	0	0	0	0	0	1	0
0	0	1	0	0	0	0	0	0
0	0	0	0	0	0	0	0	0
0	0	0	0	0	0	0	0	1
0	0	0	0	0	0	0	0	0
0	0	0	0	0	0	0	0	0
1	0	0	0	0	0	0	0	0
1	0	0	0	0	0	0	0	0
0	0	0	0	0	0	0	0	0
0	0	0	0	0	0	0	0	0
0	0	0	0	0	0	0	1	0
0	0	0	0	0	0	0	0	0
0	0	0	0	0	0	0	0	0
0	0	0	0	0	0	0	0	0
0	0	0	0	0	0	0	0	0
0	0	0	0	0	0	0	1	0
0	0	0	0	0	0	0	0	0
0	0	0	0	0	0	0	0	0
0	0	0	0	0	0	0	0	0
0	1	0	0	0	0	0	0	0
0	0	0	0	0	0	0	0	0
0	0	0	0	0	0	0	1	0
0	0	0	0	0	0	0	0	0
0	0	0	0	0	0	0	0	0
0	0	0	0	0	0	0	0	0
0	1	0	0	0	0	0	0	0
0	0	0	0	0	0	0	0	0
0	0	0	0	1	0	0	0	0
0	0	0	0	0	0	0	1	0
0	0	0	0	0	0	0	0	0
0	0	0	0	0	0	0	0	0
0	0	0	0	0	0	0	0	0

0	0	0	0	0	0	0	0	0
0	0	0	0	0	0	0	1	0
0	0	0	0	1	0	0	0	1
0	0	0	0	0	0	0	0	0
0	0	0	0	0	0	0	0	0
0	0	0	0	1	0	0	0	0
0	0	0	0	0	0	0	0	0
0	0	0	0	0	0	0	0	0
0	0	0	0	0	0	0	0	0
0	0	0	0	0	0	0	0	0
0	0	0	0	0	0	0	0	0
0	0	0	0	0	0	0	0	0
0	0	0	0	0	0	0	0	0
0	0	0	0	0	0	0	0	0
1	0	0	0	0	1	0	1	0
0	1	0	0	1	0	0	0	0
0	0	0	0	0	0	0	0	0
0	1	0	0	0	0	0	0	0
0	0	0	0	0	0	1	1	0
0	0	0	0	0	0	0	0	0
0	0	0	0	0	0	0	0	0
0	0	0	0	0	0	0	0	0
0	0	0	0	0	0	0	0	0
0	0	0	0	0	0	0	0	0
0	0	0	0	0	0	0	0	0
0	0	0	0	0	0	0	0	0
0	0	0	0	0	0	0	0	0
0	0	0	0	0	1	0	0	0
0	0	0	0	0	0	0	0	0
0	0	0	0	0	0	0	0	0
0	0	0	0	0	0	0	0	0
0	0	0	0	0	0	0	0	0
1	0	0	0	0	0	0	0	0
0	0	0	0	0	0	0	1	0
0	0	0	0	0	0	0	0	0
0	0	0	0	0	0	0	0	0
0	0	0	0	1	0	0	0	0
0	0	0	0	0	0	0	0	0
0	0	0	0	0	0	0	0	0
0	0	0	0	1	0	0	0	0
0	0	0	0	0	0	1	1	0
0	0	0	0	0	0	0	0	0
0	0	0	0	0	0	0	0	0
0	0	0	0	0	0	0	1	0
0	0	0	0	0	0	0	0	0
0	0	0	0	0	0	0	0	0
0	0	0	0	0	0	0	0	0
0	1	0	0	0	0	0	0	0
0	0	0	0	1	0	0	0	0
0	0	0	0	0	0	0	0	0
0	0	0	0	0	0	0	0	0
0	0	0	0	0	0	0	0	0
0	0	0	0	0	0	0	0	0
1	0	0	0	0	0	0	0	0
1	0	0	0	0	0	0	0	0
0	0	0	0	0	0	0	0	0

0	0	0	0	0	0	0	0	0
0	0	0	0	0	0	0	1	0
0	0	0	0	0	0	0	0	0
0	0	0	0	0	0	0	0	0
0	0	0	0	0	0	0	0	0
0	0	0	0	0	0	0	0	0
0	0	0	0	0	0	0	0	0
0	0	0	0	0	0	0	0	0
0	0	0	0	0	0	0	0	0
0	1	0	0	0	0	1	0	0
0	0	0	0	0	0	0	0	0
0	0	0	0	0	0	0	0	0
0	0	0	0	0	0	0	0	1
0	0	0	0	0	0	0	0	0
0	0	0	0	0	0	0	0	0
0	0	0	0	0	0	0	0	0
0	0	0	0	0	0	0	0	0
0	0	0	0	0	0	0	0	0
0	0	0	0	0	0	0	0	0
0	0	0	0	1	0	0	1	0
0	0	0	0	0	0	0	0	0
1	0	0	0	0	0	0	0	0
0	1	0	0	0	0	0	0	0
0	0	0	0	0	0	0	0	0
0	0	0	0	0	0	0	0	0
0	0	0	0	0	0	0	1	0
0	0	0	0	0	0	0	0	0
0	0	0	0	0	0	0	0	0
0	0	0	0	0	0	0	0	0
0	0	0	0	0	0	0	0	0
0	0	0	0	0	0	0	1	0
0	0	0	0	0	0	0	0	0
0	0	0	0	0	0	0	0	0
1	0	0	0	0	0	0	0	0
0	1	0	0	0	0	0	0	0
0	0	1	0	0	0	0	0	0
0	0	0	0	0	0	0	0	0
0	0	0	1	0	0	0	0	0
0	0	0	0	0	0	0	1	0
0	0	0	0	0	0	0	0	0
0	0	0	0	0	0	0	0	0
0	0	0	0	0	0	0	0	0
0	0	0	0	0	0	1	0	0
1	0	0	0	0	0	0	0	0
0	0	0	0	0	0	0	0	0
1	0	0	0	0	0	0	0	0
0	0	0	0	0	0	0	0	0
0	0	0	0	0	0	0	0	0
0	0	0	0	0	0	0	1	0
0	0	0	0	0	0	0	0	0
0	0	0	0	0	0	0	0	0
0	0	0	0	0	0	0	0	0
0	0	0	0	1	0	0	0	0
0	0	0	0	0	0	0	0	0
1	0	0	0	0	0	0	1	0
0	0	0	0	0	0	0	0	0

0	0	0	0	0	0	0	1	0
0	0	0	0	0	0	0	0	0
0	0	0	0	0	0	0	0	0
0	0	0	0	1	0	0	0	0
1	0	0	0	0	0	0	0	0
0	1	0	0	0	0	0	1	0
1	0	0	0	0	0	0	0	0
0	0	0	0	0	0	0	0	0
1	0	0	0	0	0	0	0	0
0	0	0	0	0	0	0	1	0
0	0	0	0	0	0	0	0	0
0	0	0	0	0	0	0	0	0
0	0	0	0	0	0	0	0	0
0	0	0	0	0	0	0	0	0
1	0	0	0	0	0	0	0	0
0	0	0	0	0	0	0	0	0
0	0	0	0	0	0	0	0	0
0	0	0	0	0	0	0	0	0
1	0	0	0	1	0	0	0	0
1	0	0	0	0	0	0	1	0
1	0	0	0	0	0	0	0	0
0	0	0	0	0	0	0	0	0
0	0	0	0	0	0	0	0	0
0	0	0	0	0	0	0	0	0
0	0	0	0	1	0	0	0	0
1	1	0	0	0	0	0	0	0
0	0	0	0	0	0	0	0	0
0	0	0	0	0	0	0	0	0
1	0	0	0	0	0	0	0	0
0	0	0	0	0	0	0	0	0
1	0	0	0	0	0	0	0	0
0	0	0	0	0	0	0	0	0
1	0	0	0	0	0	0	0	0
0	0	0	0	0	0	0	0	0
0	0	0	0	0	0	0	1	0
1	0	0	0	0	0	0	0	0
0	1	0	0	0	0	0	0	0
0	0	0	0	0	0	0	1	0
0	1	0	0	0	0	0	0	0
0	0	0	0	0	0	0	1	0
0	0	0	0	0	0	0	0	0
0	0	0	0	0	0	0	0	0
0	0	0	0	0	0	0	0	0
1	0	0	0	1	0	0	0	0
0	0	0	0	0	0	1	0	0
0	0	0	0	0	0	0	0	0
0	0	0	0	0	0	0	0	1
1	0	0	0	1	0	0	0	0
0	0	0	0	0	0	0	0	0
0	0	0	0	0	0	0	0	0
0	0	0	0	0	0	0	1	0
0	0	0	0	0	0	0	0	0
0	0	0	0	0	0	0	0	0
0	0	0	0	0	0	0	0	0
0	0	0	0	0	0	0	0	0
0	0	0	0	0	0	0	0	0

0	0	0	0	0	0	0	0	0
1	0	0	0	0	0	0	0	0
0	0	0	1	0	0	0	0	0
0	0	0	0	0	0	0	0	0
1	0	0	0	0	0	0	0	0
0	0	0	0	0	0	0	0	0
0	0	0	0	0	0	0	0	0
0	0	0	0	0	0	0	1	0
0	0	0	0	0	0	0	1	0
1	0	0	0	0	0	0	0	0
0	0	0	0	0	0	0	1	0
0	0	0	0	0	0	0	0	0
1	0	0	0	0	0	0	0	0
1	0	0	0	1	0	0	0	0
0	0	0	0	0	0	0	0	0
1	0	0	0	0	0	0	0	0
0	0	0	0	0	1	0	0	0
0	0	0	0	0	0	1	0	0
1	0	0	0	0	0	0	0	0
0	0	0	0	0	0	0	0	0
0	0	0	0	0	0	0	0	0
0	0	1	0	0	0	0	0	0
0	1	0	0	0	0	0	0	0
0	0	0	0	0	0	0	0	0
0	0	0	0	1	0	0	0	0
0	0	0	0	0	0	0	0	0
1	0	0	0	0	0	1	0	0
1	0	0	0	0	0	0	1	0
0	0	0	0	0	0	0	0	0
0	0	0	0	0	0	0	1	0
0	0	0	0	0	0	0	0	0
0	1	0	0	0	0	0	1	0
0	0	0	0	0	0	0	0	0
0	0	0	0	0	0	0	0	0
0	0	0	0	0	0	0	0	0
0	0	0	0	0	0	1	0	0
1	0	0	0	0	0	0	0	1
1	0	0	0	0	0	0	0	0
0	0	0	0	0	0	0	0	0
1	0	0	0	0	0	0	1	0
0	0	0	0	0	0	0	0	0
0	0	0	0	0	0	0	0	0
0	0	0	0	0	0	0	0	0
0	0	0	0	0	0	0	0	0
1	0	0	0	0	0	0	0	0
0	0	0	0	0	0	0	0	0
0	1	0	0	0	0	0	0	0
0	0	0	0	0	0	0	0	0
0	1	0	0	0	0	0	0	0
0	0	0	0	1	0	0	0	0
1	0	0	0	0	0	0	0	0
0	0	0	0	0	0	0	0	0
0	0	0	0	0	0	0	1	0
0	0	0	0	0	0	0	0	0
0	0	0	0	0	0	0	0	0
0	0	0	0	0	0	0	0	0

0	0	0	0	0	0	0	0	0
0	0	0	0	0	0	0	0	0
0	0	0	0	0	0	0	0	0
0	0	0	0	0	0	0	0	0
0	0	0	0	0	0	0	0	0
0	0	0	0	0	0	0	0	0
0	0	0	0	0	0	0	0	0
0	0	0	0	0	0	0	1	0
0	0	0	0	0	0	0	0	0
0	0	0	0	1	0	0	0	0
0	0	0	0	0	0	0	0	0
0	0	0	0	0	0	0	1	0
1	1	0	0	0	0	0	0	0
0	0	0	0	0	0	0	0	0
0	0	0	0	0	0	0	0	0
0	0	0	0	0	0	0	0	0
1	0	0	0	0	0	0	0	0
0	0	0	0	0	0	0	0	0
0	0	0	0	0	0	0	0	0
1	0	0	0	0	0	0	0	0
0	1	0	0	0	0	0	0	0
1	0	0	0	0	0	0	0	0
0	0	0	0	0	0	0	0	0
0	0	0	0	0	0	0	0	0
1	0	0	0	0	0	1	0	0
1	0	0	0	1	0	0	0	0
1	0	0	0	0	0	0	0	0
1	0	0	0	0	0	0	0	0
1	0	0	0	0	0	0	0	0
1	0	0	0	0	0	0	0	0
0	0	0	0	0	0	1	0	0
1	0	0	0	1	0	0	1	0
0	0	0	0	0	0	0	1	0
0	0	0	0	0	0	0	0	0
1	0	0	0	0	0	0	0	0
1	0	0	0	0	0	0	0	0
0	0	0	0	0	0	0	1	0
0	0	0	0	0	0	0	0	0
0	1	0	0	0	0	0	1	0
0	0	0	0	0	0	0	0	0
1	0	0	0	0	0	0	1	0
0	0	0	0	0	0	0	0	0
1	0	0	0	0	0	0	0	0
0	0	0	0	0	0	1	0	0
0	0	0	0	0	0	0	0	0
1	0	0	0	0	0	0	0	0
0	0	0	0	0	0	0	0	0
1	0	0	0	0	0	0	0	0
0	0	0	0	1	0	0	0	0
0	1	0	0	0	0	0	0	0
1	0	0	0	1	0	0	0	0
0	0	0	0	0	0	0	1	0
0	0	0	0	0	0	0	0	0
0	0	0	0	0	0	0	0	0
0	0	0	0	0	0	0	0	0
1	1	0	0	0	0	0	0	0

0	0	0	0	0	0	0	0	0
0	0	0	0	0	0	0	0	0
1	0	0	0	0	0	0	0	0
0	0	0	0	0	0	0	0	0
1	0	0	0	0	0	0	0	0
1	0	0	0	0	0	0	0	0
1	0	0	0	0	0	0	0	0
0	0	0	0	0	0	0	0	0
1	0	0	0	0	0	0	0	0
1	0	0	0	0	0	0	0	0
1	0	0	0	0	0	0	0	0
1	0	0	0	0	0	0	1	0
1	0	0	0	0	0	0	0	0
1	0	0	0	0	0	0	0	0
1	1	0	0	0	0	0	0	0
0	0	0	0	0	0	0	0	0
1	0	0	0	0	0	1	0	0
0	0	0	0	0	0	0	1	0
0	0	0	0	0	0	0	0	0
0	0	0	0	0	0	0	1	0
1	0	0	0	0	0	0	0	0
1	0	0	0	0	0	0	0	0
1	0	0	0	0	0	0	0	0
0	0	0	0	0	0	0	1	0
1	0	0	0	1	0	0	0	0
0	0	0	0	0	0	0	0	0
0	1	0	0	0	0	0	0	0
1	0	0	0	0	0	0	0	0
0	1	0	0	0	0	0	0	0
1	0	0	0	0	0	0	0	0
1	0	0	0	0	0	0	0	0
0	0	0	0	1	0	0	0	0
0	0	0	0	0	0	0	0	0
0	0	0	0	0	0	0	0	0
0	0	0	0	0	0	0	0	0
0	0	0	1	0	0	0	0	0
1	0	0	0	0	0	0	0	0
1	0	0	1	0	0	0	1	0
0	0	0	0	1	0	0	0	0
1	0	0	0	0	0	0	0	0
1	0	0	0	0	0	0	0	0
0	0	0	0	0	0	0	0	0
0	0	0	0	0	0	0	0	0
0	0	0	0	0	0	0	0	0
0	0	0	0	0	0	0	0	0
1	0	0	0	0	0	0	0	0
0	0	0	0	0	0	0	0	0
0	0	0	0	0	0	0	0	0
0	0	0	0	0	0	0	0	0
0	0	0	0	0	0	0	0	0
0	0	0	1	0	0	0	0	0
0	0	0	0	0	0	0	0	0
0	0	0	0	0	0	0	0	1
0	0	0	0	0	0	0	0	0
1	0	0	0	0	0	0	0	0
0	0	0	0	0	0	1	0	0

1	0	0	0	1	0	1	0	0
1	0	0	0	0	0	0	0	0
1	0	0	0	0	0	0	0	0
0	0	0	0	0	0	0	0	0
0	0	0	0	0	0	0	0	0
0	1	0	0	0	0	0	0	0
0	0	0	0	1	0	0	0	0
0	0	0	0	0	0	0	0	0
0	1	0	0	0	0	0	0	0
0	0	0	0	0	0	0	0	0
0	0	0	0	0	0	0	0	0
1	0	0	0	0	0	0	0	0
1	0	0	0	0	0	0	0	0
0	0	0	0	0	0	0	0	0
0	0	0	0	0	0	0	1	0
0	0	0	0	0	0	0	0	0
0	0	0	0	0	0	0	0	0
1	1	0	0	0	0	0	0	0
1	0	0	0	0	0	0	0	0
0	0	0	0	0	0	0	0	0
1	0	0	0	0	0	0	0	0
0	0	0	0	0	0	0	0	0
0	0	0	0	0	0	0	0	0
0	0	0	0	0	0	0	0	0
1	0	0	0	0	0	0	0	0
0	1	0	0	0	0	0	0	0
0	0	0	0	0	0	0	0	0
0	0	0	0	0	0	0	0	0
0	0	0	0	0	0	0	0	0
0	0	0	0	1	1	0	0	0
0	0	0	0	0	0	0	0	0
0	1	0	0	0	0	0	0	0
0	0	0	0	0	0	0	0	0
0	0	0	0	0	0	0	0	0
0	0	0	0	0	0	0	0	0
0	0	0	0	0	0	0	0	0
0	1	0	0	0	0	0	0	0
0	0	0	0	0	0	0	0	0
0	0	0	0	0	0	0	0	0
0	0	0	0	0	0	0	0	0
0	0	0	1	0	0	0	0	0
1	0	0	0	0	0	0	0	0
0	1	0	0	0	0	0	1	0
0	0	0	0	0	0	0	0	0
0	0	0	0	0	0	0	0	0
0	0	0	0	0	0	0	0	0
1	0	1	0	0	1	0	1	0
0	0	0	0	0	0	0	0	0
0	0	0	1	0	0	1	0	0
0	0	0	0	0	0	0	0	0
0	0	0	0	1	0	0	0	0
1	0	0	0	0	0	0	0	0
0	0	0	0	1	0	0	0	0
1	0	0	0	0	0	0	0	0
1	0	1	1	0	0	0	0	1
1	1	1	0	0	1	0	1	1

0	0	0	0	0	0	0	1	0
0	0	0	0	0	0	0	0	0
0	0	0	0	0	0	0	0	0
0	0	0	0	0	0	0	1	0
0	0	0	0	0	0	0	0	0
0	0	0	0	0	0	0	0	0
0	0	0	0	0	0	0	0	0
0	0	0	0	0	0	0	0	0
0	0	0	0	0	0	1	0	0
0	0	0	0	0	0	0	0	0
0	0	0	0	0	0	0	0	0
0	0	0	0	0	0	0	0	0
0	0	0	0	0	0	0	0	0
0	0	0	0	0	0	0	0	0
0	0	0	0	0	0	0	0	0
0	0	0	0	0	0	0	0	0
0	0	0	0	0	0	0	0	0
0	0	0	0	0	0	0	0	0
0	0	0	0	0	0	0	0	0
1	0	0	0	0	0	0	0	0
0	0	0	0	0	0	0	0	1
0	0	0	0	0	0	0	0	0
0	0	0	0	0	0	0	0	0
0	0	0	0	0	0	0	0	0
0	0	0	0	0	0	0	0	0
0	0	0	0	0	0	0	0	0
0	0	0	0	0	0	0	0	0
0	0	0	0	0	0	0	0	0
1	0	0	0	0	0	0	0	0
0	0	0	0	0	0	0	0	0
0	0	0	0	0	0	0	0	0
0	0	0	0	0	0	0	0	0
0	0	0	0	0	0	0	0	0
0	0	0	0	0	0	0	0	0
0	0	0	0	0	0	0	1	0
0	0	0	0	0	0	0	0	0
0	0	0	0	1	0	0	1	1
0	0	0	0	0	0	0	0	0
0	0	0	0	0	0	0	0	0
0	0	0	0	1	0	0	0	0
0	0	0	0	0	0	1	0	0
0	0	0	0	0	0	0	0	0
0	0	0	0	0	0	0	0	0
0	0	0	0	0	0	0	0	0
0	0	0	0	0	0	0	0	0
0	0	0	0	0	0	0	0	0
0	0	0	0	0	0	0	0	0
0	0	0	0	0	0	0	0	0
0	0	0	0	0	0	0	0	0
0	0	0	0	0	0	0	0	0
0	0	0	0	0	0	0	0	0
0	0	0	0	0	0	0	0	0
0	0	0	0	0	0	0	0	0
0	0	0	0	0	0	0	0	0
0	0	0	0	0	0	0	0	0
0	0	0	0	0	0	0	0	0

0	0	0	0	0	0	0	0	0
0	0	0	0	0	0	0	0	0
1	0	0	0	0	0	0	1	0
0	1	0	0	0	0	0	0	0
0	0	0	0	0	0	0	0	0
0	0	0	0	0	0	0	0	0
0	0	0	0	0	0	0	0	0
0	0	0	0	0	0	0	0	0
0	0	0	0	0	0	0	0	0
0	1	1	0	1	0	0	0	0
0	0	0	0	0	0	0	0	0
0	0	0	0	0	0	0	0	0
0	1	0	0	0	0	0	0	0
0	0	0	0	0	0	0	0	0
1	1	0	0	0	0	0	0	0
0	0	0	0	0	0	0	0	0
0	0	0	0	0	0	0	0	0
0	0	0	0	0	0	0	0	0
0	0	0	0	0	0	0	0	0
0	0	0	0	0	0	1	0	0
0	0	0	0	0	0	0	0	0
0	0	0	0	0	0	0	0	0
0	0	0	0	0	0	0	0	0
0	0	0	0	0	0	0	0	0
0	0	0	0	0	0	0	0	0
0	0	0	0	0	0	0	0	0
0	0	0	0	0	0	0	0	0
0	0	0	0	0	0	0	0	0
0	0	0	0	0	0	0	0	0
1	0	0	0	0	0	0	0	0
0	0	0	0	0	0	0	0	0
0	1	0	0	0	0	0	0	0
0	0	0	0	0	0	0	0	0
0	0	0	0	0	0	0	0	1
0	0	0	0	0	0	0	0	0
1	0	0	0	0	0	0	0	0
0	0	0	0	0	0	0	0	0
0	0	0	0	0	0	0	0	0
0	0	0	0	0	0	0	0	0
0	0	0	0	0	0	0	0	0
1	0	0	0	0	0	0	1	0
0	0	0	0	0	0	0	0	0
0	0	0	0	0	0	0	0	0
0	0	0	0	0	0	0	1	0
0	0	0	0	0	0	0	1	0
0	0	0	0	0	0	0	1	0
0	0	0	0	0	0	0	0	0
0	0	0	0	0	0	0	0	0
0	0	0	0	0	0	0	0	0
0	0	0	0	0	0	0	0	0
0	0	0	0	0	0	0	0	0
0	1	0	0	0	0	0	0	0
0	0	0	0	0	0	0	0	0
0	0	0	0	0	0	0	0	0
0	0	0	0	0	0	0	0	0
0	0	0	0	0	0	0	0	0

0	0	0	0	0	0	0	0	0
0	0	0	1	0	0	0	0	0
0	1	0	0	0	0	0	0	0
0	0	0	0	0	0	0	0	0
0	0	0	0	0	0	0	0	0
0	0	0	0	0	0	0	0	0
0	0	0	0	0	0	1	0	0
0	0	0	0	0	0	0	1	0
0	0	0	0	0	0	0	0	0
0	1	0	0	1	0	0	0	0
0	0	0	0	0	0	0	0	0
1	0	0	0	0	0	0	0	0
0	0	0	0	0	0	0	0	0
0	0	0	0	0	0	0	0	0
0	0	0	0	0	0	0	0	0
0	0	0	0	0	0	0	0	0
0	0	0	0	0	0	0	0	0
0	0	0	0	0	0	0	0	0
0	0	0	0	0	0	0	0	0
0	0	0	0	0	0	0	0	0
0	0	0	0	0	0	0	0	0
0	1	0	0	0	0	0	1	0
1	0	0	0	1	0	0	0	0
0	0	0	0	0	0	0	0	0
0	0	0	0	0	0	0	0	0
0	0	0	0	0	0	0	0	0
0	0	0	0	0	0	0	0	0
1	0	0	0	0	0	1	1	0
0	0	0	0	0	0	0	0	0
0	0	0	0	0	0	0	0	0
0	0	0	0	0	0	0	0	0
0	0	0	0	0	0	0	0	0
0	1	0	0	0	0	0	1	0
0	0	0	0	0	0	0	0	0
0	0	0	0	0	0	0	0	0
0	0	0	0	0	0	0	0	0
0	0	0	0	0	0	0	0	0
0	0	0	0	0	0	0	0	0
0	0	0	0	0	0	0	1	0
0	0	0	0	0	0	0	0	0
0	0	0	0	0	0	0	0	0
0	0	0	0	0	0	0	0	0
0	0	0	0	0	0	0	0	0
0	0	0	0	0	0	0	0	0
0	0	0	0	0	0	0	0	0
0	0	0	0	0	0	0	0	0
0	0	0	0	0	0	0	0	0
0	0	0	0	0	0	0	0	0
0	0	0	0	0	0	0	0	0
0	0	0	0	0	0	0	0	0
0	0	0	0	1	0	0	0	0
0	0	0	0	0	0	0	0	0
0	0	0	0	1	0	0	0	0
0	0	0	0	0	0	0	0	0
0	0	0	0	0	0	0	0	0
0	0	0	0	0	0	0	0	0

1	0	0	0	0	0	0	0	0
0	0	0	0	0	0	0	0	0
0	0	0	0	0	0	0	0	0
0	0	0	0	0	0	0	0	0
0	0	0	0	1	0	0	0	0
0	0	0	0	0	0	0	0	0
0	0	0	0	0	0	0	0	0
1	0	0	0	0	0	0	1	0
0	0	0	0	0	0	0	0	0
0	0	0	0	0	0	0	0	0
0	0	0	0	0	0	0	0	0
0	0	0	0	0	0	0	0	0
0	0	0	0	0	0	0	0	0
0	0	0	0	0	0	0	0	0
0	0	0	0	0	0	0	0	0
0	0	0	0	0	0	0	0	0
0	0	0	0	0	0	0	0	0
0	0	0	0	0	0	0	0	0
0	0	0	0	0	0	0	0	0
0	1	0	0	0	0	0	0	0
1	0	0	0	0	0	0	0	0
0	0	0	0	0	0	0	0	0
0	1	0	0	0	0	0	0	0
0	0	0	0	0	0	0	0	0
0	0	0	0	0	0	0	0	0
0	0	0	0	0	0	1	1	0
0	0	0	0	0	0	0	0	0
0	0	0	0	0	0	0	0	0
0	0	1	0	0	0	0	0	0
0	0	0	0	1	0	0	0	0
0	0	0	0	0	0	0	0	0
0	0	0	0	0	0	0	0	0
0	0	0	0	0	0	0	0	0
0	0	0	0	0	0	0	0	0
0	0	0	0	0	0	0	0	0
0	0	0	0	0	0	0	0	0
0	0	0	0	0	0	0	0	0
0	0	0	0	0	0	0	0	0
1	0	0	0	0	0	0	0	0
0	0	0	0	0	0	0	1	0
0	0	0	0	0	0	0	0	0
0	0	0	0	0	0	0	0	0
0	0	0	0	0	0	0	0	0
0	1	0	0	0	0	0	0	0
0	0	0	0	0	0	0	0	0
1	0	0	0	0	0	0	0	0
0	0	0	0	0	0	0	0	0
0	0	0	0	0	0	0	0	0
0	0	0	0	1	1	0	0	0
0	0	0	0	0	0	0	0	0
0	0	0	0	0	0	0	1	0
0	0	1	0	1	0	0	1	0
0	0	0	0	0	0	0	1	0
0	1	0	0	0	0	0	0	0
0	0	0	0	0	0	0	0	0
1	0	0	0	0	0	0	0	0

1	1	0	0	0	0	0	0	0
0	0	0	0	0	0	0	0	0
0	0	0	0	0	0	1	0	0
1	0	0	0	0	0	0	0	0
1	0	0	0	0	0	1	0	0
1	0	0	0	0	0	0	0	0
1	1	0	0	0	0	0	0	0
0	0	0	0	0	0	0	0	0
0	0	0	0	0	0	0	0	0
0	0	0	0	0	0	0	0	0
0	0	0	0	0	0	0	0	0
0	0	0	0	0	0	0	0	0
0	0	0	0	0	0	0	0	0
0	0	0	0	0	0	0	0	0
0	0	0	0	0	0	0	0	0
1	1	0	0	0	0	0	0	0
1	0	0	0	0	0	0	0	1
1	0	0	0	0	0	0	0	0
1	0	0	0	0	0	0	0	0
0	1	0	0	0	0	0	0	0
0	0	0	0	0	0	0	0	0
0	0	0	0	0	0	0	1	0
1	0	0	1	0	0	0	0	0
1	0	0	0	0	0	0	0	0
0	0	0	0	0	0	0	0	0
0	0	0	0	0	0	0	0	0
0	0	0	0	0	0	0	0	0
0	0	0	0	0	0	0	1	0
0	0	0	0	0	0	0	0	0
1	0	0	0	0	0	0	1	0
0	0	0	0	0	0	0	0	0
0	0	0	0	0	0	0	1	0
0	0	0	0	0	0	0	0	0
1	0	0	0	0	0	0	0	0
0	0	0	0	1	1	0	1	0
1	0	0	0	0	0	0	0	0
0	0	0	0	0	0	0	0	0
0	0	0	0	0	0	0	0	0
0	0	0	0	0	0	0	0	0
1	0	0	0	1	0	0	0	0
0	0	0	0	0	0	0	0	0
1	0	0	0	0	0	0	0	0
0	0	0	0	0	0	0	0	0
1	1	0	0	0	0	0	0	0
0	0	0	0	1	0	0	0	0
1	1	0	0	0	0	0	0	0
0	1	0	0	0	0	0	0	0
0	0	0	0	0	0	0	0	0
0	0	0	0	0	0	0	0	0
1	0	0	0	0	0	0	0	0
1	0	0	0	0	0	0	0	0
0	0	0	0	0	0	0	0	0
1	0	0	0	0	0	0	0	0
0	0	0	0	0	0	0	0	0
1	0	0	0	0	0	0	0	0
1	0	0	0	0	0	0	0	0

1	0	0	0	0	0	0	0	0
0	0	1	0	1	0	0	1	0
0	0	0	0	0	0	0	1	0
0	1	0	0	0	0	0	0	0
1	0	0	0	0	0	0	0	0
1	0	0	0	0	0	0	0	0
0	0	0	0	0	0	0	0	0
0	0	0	0	0	0	1	0	0
1	1	1	0	0	0	0	0	0
1	0	0	0	1	0	0	0	0
1	0	0	0	0	0	0	1	0
1	0	0	1	0	0	0	1	0
0	1	0	0	0	0	0	0	0
0	0	0	0	1	1	0	0	0
0	0	0	0	0	0	0	0	0
0	0	0	0	0	0	0	0	0
0	0	0	0	0	0	0	0	0
0	0	0	0	0	0	0	0	0
0	0	0	0	1	0	0	0	0
0	0	0	0	0	0	0	0	0
0	0	0	0	1	0	0	0	0
0	0	0	0	0	0	0	0	0
0	0	0	0	1	0	0	0	0
0	0	0	0	0	0	0	0	0
0	0	0	0	0	0	0	0	0
0	0	0	0	1	0	0	0	0
0	0	0	0	0	0	0	0	0
0	0	0	0	0	0	0	0	0
0	0	0	0	0	0	0	0	0
0	0	0	0	0	0	0	0	0
0	0	0	0	0	0	0	0	0
0	0	0	0	0	0	0	0	0
0	0	0	0	0	0	0	0	0
0	0	0	0	0	0	0	0	0
0	0	0	0	0	0	0	0	0
0	0	0	0	1	0	0	1	0
0	0	0	0	0	0	0	0	0
0	0	0	0	0	0	0	0	0
0	0	0	0	0	0	0	0	0
1	0	0	0	0	0	0	0	0
0	0	0	0	0	0	0	0	0
0	0	0	0	0	0	0	0	0
0	0	0	0	0	0	0	0	0
0	0	0	0	0	0	0	0	0
0	0	0	0	0	0	0	0	0
0	0	0	0	0	0	0	0	0
0	0	0	0	0	0	0	0	0
0	0	0	0	0	0	0	0	0
0	0	0	0	0	0	0	0	0
0	0	0	0	0	0	0	0	0
0	0	0	0	0	0	0	0	0
0	0	0	0	0	0	0	0	0
0	0	0	0	0	0	0	0	0
0	0	0	0	0	0	0	1	0
0	0	0	0	0	0	0	0	0
0	0	0	0	0	0	0	0	0

0	0	0	0	0	0	0	0	0
0	0	0	0	0	0	0	0	0
0	0	0	0	0	0	0	0	0
0	0	0	0	0	0	0	0	0
0	0	0	0	0	0	0	0	0
0	0	0	0	0	0	0	0	0
0	0	0	0	0	0	0	0	0
0	0	0	0	0	0	0	0	0
0	0	0	0	1	0	0	0	0
0	0	0	0	0	0	0	0	0
0	0	0	0	0	0	0	0	0
0	0	0	0	0	0	0	0	0
0	0	0	0	0	0	0	0	0
0	0	0	0	0	0	0	0	0
0	0	0	0	0	0	0	0	0
0	0	0	0	0	0	0	0	0
0	0	0	0	0	0	0	0	1
0	0	0	1	0	0	0	0	0
0	0	0	0	0	0	0	0	0
0	0	0	0	0	0	0	0	0
0	0	0	0	0	0	0	1	0
0	0	0	0	0	0	0	0	0
0	0	0	0	1	0	0	0	0
0	0	0	0	0	0	0	0	0
0	0	0	0	0	0	0	0	0
0	1	0	0	0	0	0	0	0
0	0	0	0	0	0	0	0	0
0	0	0	0	0	0	0	0	0
0	0	0	0	0	0	0	0	0
0	0	0	0	0	0	0	0	0
0	0	0	0	0	0	0	0	0
0	0	0	0	0	0	0	0	0
0	0	0	0	0	0	0	0	0
0	0	0	0	0	1	0	0	0
0	0	0	0	0	0	0	0	0
0	0	0	0	1	0	0	1	0
0	0	0	0	0	0	0	0	0
0	0	0	0	0	0	0	0	0
0	0	0	0	0	0	0	0	0
0	0	0	0	1	0	0	0	0
0	0	0	0	0	0	0	0	0
0	0	0	0	0	0	0	1	0
0	0	0	0	0	0	0	0	0
0	1	0	0	0	0	0	0	0
0	0	0	0	0	0	0	0	0
0	0	0	0	0	0	0	0	0
0	0	0	0	0	0	0	0	0
0	0	0	0	0	0	0	0	0
0	0	0	0	0	0	0	0	0
0	0	0	0	0	0	0	0	0
0	0	0	0	0	0	0	0	0
0	0	0	0	0	0	0	1	1
0	0	0	0	0	0	0	0	0
0	0	0	0	0	0	0	0	0
0	0	0	0	0	0	0	0	0
0	0	0	0	0	0	0	0	0

0	0	0	0	0	0	0	0	0
0	0	0	0	0	0	0	0	0
0	0	0	0	0	1	0	0	0
0	0	0	0	0	0	0	0	0
0	0	0	0	0	0	0	0	0
0	0	0	0	0	0	0	0	0
0	0	0	0	0	0	0	0	0
0	0	0	0	0	0	0	0	0
0	0	0	0	0	0	0	0	0
0	0	0	0	0	0	0	0	0
0	0	0	0	0	0	0	0	0
0	0	0	0	0	0	0	0	0
0	0	0	0	0	0	0	0	0
0	0	0	0	0	0	0	0	0
0	0	0	0	0	0	0	1	0
0	0	0	0	0	0	0	0	0
0	1	1	0	0	0	0	0	0
0	0	0	0	0	0	0	0	0
0	0	0	0	0	0	0	0	0
0	0	0	0	0	0	0	0	0
0	0	0	0	0	0	0	0	0
0	0	0	0	0	0	0	0	0
0	0	0	0	0	0	0	0	0
0	0	0	0	0	0	0	0	0
0	0	0	0	0	0	0	0	0
0	0	0	0	1	0	0	0	0
0	0	0	0	0	0	0	0	0
0	0	0	0	0	0	0	0	0
0	0	0	0	0	0	0	0	0
0	1	0	0	0	0	0	0	0
0	0	0	0	0	0	1	0	0
0	0	1	0	0	0	0	0	0
0	0	0	0	0	0	0	0	0
0	0	0	0	0	0	0	0	0
0	0	0	0	0	0	0	0	0
0	0	0	0	0	0	0	0	0
0	0	0	0	0	0	0	0	0
0	0	0	0	0	0	0	0	0
0	0	0	0	0	0	0	0	0
0	0	0	0	0	0	0	0	0
0	0	0	0	0	0	0	0	0
0	0	0	0	1	0	0	0	0
0	0	0	0	0	0	0	0	0
0	0	0	0	0	0	0	0	0
0	0	0	0	0	0	0	0	0
0	0	0	0	0	0	0	1	1
0	0	0	0	0	0	0	0	0
0	0	0	0	0	0	0	0	0
0	0	0	0	0	0	0	0	0
0	0	0	0	0	0	0	0	1
0	1	0	0	0	0	0	0	0
0	0	0	0	0	0	0	0	0
0	0	1	0	0	0	0	1	0
0	0	0	0	0	0	0	0	0
0	0	1	0	0	0	0	0	0
0	0	0	0	0	0	0	0	0

0	0	0	0	0	0	0	0	0
0	0	0	0	0	0	0	1	0
0	0	0	0	0	0	0	0	0
0	0	0	0	0	0	0	0	0
0	0	0	0	0	0	0	0	0
0	0	0	0	1	0	0	0	0
0	0	0	0	1	0	0	0	0
0	0	0	0	0	0	0	0	0
0	0	0	0	0	0	0	0	0
0	0	0	0	0	0	0	0	0
0	0	0	0	0	0	0	0	0
0	0	0	0	0	0	0	0	0
0	1	0	0	0	0	0	0	0
0	0	0	0	0	0	0	0	0
0	0	0	0	0	0	0	0	0
1	0	0	0	0	0	0	0	0
0	0	0	0	0	0	0	0	0
0	0	0	0	0	0	0	0	0
0	0	0	0	0	0	0	1	0
0	0	0	0	0	0	0	1	0
0	1	0	0	0	0	0	0	0
0	0	0	0	0	0	0	0	0
0	0	0	0	0	0	0	0	0
0	0	0	0	0	0	0	0	0
0	0	0	0	0	0	0	0	0
0	0	0	0	0	0	0	0	0
0	0	0	0	0	0	0	0	0
0	0	0	0	0	0	0	0	0
0	0	0	0	1	0	0	0	0
0	0	0	0	0	0	0	0	0
0	0	0	0	0	0	0	0	0
0	0	0	0	0	0	0	0	0
0	0	0	0	0	0	0	0	0
0	0	0	0	0	0	0	0	0
0	0	0	0	0	0	0	0	0
0	1	0	0	0	0	0	0	0
0	0	0	0	1	0	0	0	0
0	0	0	0	0	0	0	0	0
1	0	0	0	0	0	0	0	0
0	0	0	1	0	0	0	0	0
0	0	0	0	0	0	0	0	0
0	0	0	0	0	0	0	0	0
1	0	0	0	0	0	0	0	0
0	0	0	0	1	0	0	0	0
0	0	0	0	0	0	0	0	0
0	0	0	0	0	0	0	0	0
0	0	0	0	0	0	0	0	0
0	0	0	0	0	0	0	0	0
0	0	0	0	0	0	0	0	0
0	0	0	0	0	0	0	0	0
0	0	0	0	0	0	0	0	0
0	0	0	1	0	0	0	0	0
0	0	0	0	0	0	0	0	0
0	1	0	0	0	0	0	0	0
0	0	0	0	0	0	0	0	0
0	0	0	0	0	0	0	0	0

0	0	0	0	0	0	0	0	0
0	0	0	0	0	0	0	0	0
0	0	0	0	0	0	0	0	0
0	0	0	0	0	0	0	0	0
0	0	0	0	0	0	0	0	0
0	0	0	0	0	0	0	0	0
0	0	0	0	1	0	0	0	0
0	0	0	0	0	0	0	0	0
0	0	0	0	0	0	0	0	0
0	0	0	0	0	0	0	0	0
0	0	0	0	0	0	0	0	0
0	0	0	0	0	0	0	0	0
0	0	0	0	0	0	0	0	0
0	1	1	0	0	0	0	0	0
0	0	0	0	0	0	0	0	0
0	0	0	0	0	0	0	0	0
0	0	0	0	0	0	0	0	0
1	1	0	1	0	0	0	0	0
1	0	0	0	0	0	0	0	0
1	0	0	0	0	0	0	0	0
0	0	0	0	0	0	0	1	0
0	0	0	0	0	0	1	0	1
0	0	0	0	0	0	0	0	0
0	0	0	0	0	0	0	0	0
0	0	0	0	0	0	0	0	0
0	0	0	0	0	0	0	0	0
1	0	0	0	0	0	0	0	0
0	0	0	0	0	0	0	1	0
0	0	0	0	0	0	0	0	0
0	0	0	0	0	0	0	0	0
0	0	0	1	0	0	0	0	1
0	0	0	0	0	0	0	1	0
0	0	0	0	0	0	0	1	0
1	0	0	0	0	0	0	0	0
0	0	0	0	0	0	0	1	0
1	1	0	0	0	0	0	0	0
0	0	0	0	0	0	0	0	0
0	0	0	0	0	0	0	0	0
0	0	0	0	0	0	0	0	0
1	0	0	0	0	0	0	0	0
0	0	0	0	1	0	0	0	0
0	1	0	1	0	0	0	1	1
0	0	0	0	0	0	0	0	0
0	1	0	0	0	0	0	1	0
0	0	0	0	0	0	0	0	0
0	1	0	0	0	0	0	0	0
0	0	0	0	0	0	0	0	0
1	0	0	0	0	0	0	0	0
0	0	0	0	0	0	0	0	0
0	0	0	0	0	0	0	0	0
0	0	0	0	0	0	0	0	0
0	0	0	0	0	0	0	0	0
1	0	0	0	0	0	0	0	0
0	0	0	0	0	0	0	1	0
0	0	0	0	0	0	0	0	0
0	0	0	0	0	0	0	0	0

0	0	0	0	0	0	0	0	0
0	0	0	0	0	0	0	0	0
0	0	0	0	0	0	0	0	0
0	0	0	0	0	0	0	0	0
1	0	0	0	0	0	0	0	0
0	0	0	0	0	0	0	0	0
0	1	0	1	0	0	0	0	0
0	0	0	0	0	0	0	0	0
0	0	0	0	0	0	0	1	0
0	0	0	0	0	0	0	0	0
1	0	0	0	0	0	0	0	0
0	0	0	0	1	0	0	0	0
0	0	0	0	0	0	0	0	0
0	0	0	0	0	0	0	0	0
1	0	0	0	1	0	0	1	0
0	0	0	0	0	0	0	1	0
0	0	0	0	0	0	0	0	0
1	0	0	0	0	0	0	1	0
0	0	0	0	0	0	0	0	0
1	0	0	0	0	0	0	0	0
0	0	0	0	0	0	0	0	0
0	0	0	0	0	0	0	0	0
0	1	0	0	0	0	0	1	0
0	0	0	0	0	0	0	0	0
1	0	0	0	0	0	0	0	0
0	0	0	0	0	0	0	0	0
1	0	0	0	0	0	0	0	0
0	0	0	0	0	0	0	0	1
0	0	0	0	0	0	0	0	0
0	0	0	0	0	0	0	0	0
0	0	0	0	0	0	0	0	0
0	0	0	0	0	0	0	0	0
0	0	0	0	1	0	0	0	0
0	0	0	0	0	0	0	0	0
0	0	0	0	0	0	0	0	0
0	0	0	0	1	0	0	0	0
0	1	0	0	0	0	0	0	0
0	0	1	0	0	0	0	1	0
0	0	0	0	0	0	0	0	0
0	0	0	0	0	0	0	0	0
0	0	0	0	0	0	0	1	0
0	0	0	0	0	0	0	0	0
0	0	0	0	1	0	0	0	0
0	0	0	0	0	0	0	0	0
0	0	0	0	0	0	0	0	0
0	0	0	0	0	0	1	1	0
0	0	0	0	0	0	0	0	0
1	0	0	0	1	0	0	0	0
1	1	0	0	0	0	0	1	0
0	0	0	0	0	0	0	0	0
1	0	0	0	0	0	0	0	0
0	0	0	0	0	0	0	0	0
0	0	0	0	0	0	0	0	0
0	0	0	0	0	0	0	1	0
0	0	0	0	0	0	0	0	0
0	0	0	0	0	0	0	0	0

0	0	0	0	0	0	0	0	0
0	0	0	0	0	0	0	0	0
0	0	0	0	0	0	0	0	0
0	0	0	0	0	0	1	1	0
0	0	0	0	0	0	0	0	0
0	0	0	0	0	0	0	0	0
0	0	0	0	0	0	0	1	0
0	0	0	0	0	0	0	0	0
1	0	0	0	0	0	0	0	0
0	0	0	0	0	0	0	0	0
0	0	0	0	0	0	0	0	0
0	1	0	0	0	0	0	0	0
0	0	0	0	0	0	0	0	0
0	0	0	0	0	0	1	1	0
0	0	0	0	0	0	0	1	0
0	0	0	0	0	0	1	1	0
1	0	0	0	0	0	0	0	0
0	1	0	0	0	0	0	0	0
0	0	0	0	0	0	0	0	0
0	0	0	0	1	0	0	0	0
0	1	0	0	0	0	1	1	0
0	0	0	0	0	0	0	0	0
0	0	0	0	0	0	0	0	0
0	0	0	0	0	0	0	0	0
0	0	0	0	0	0	0	0	0
0	0	0	0	0	0	1	1	0
0	0	0	0	0	0	0	0	0
0	0	0	0	1	0	0	0	0
0	0	0	0	0	0	0	0	0
1	0	0	1	0	0	0	0	0
0	0	0	0	0	0	0	0	0
0	0	0	0	0	0	0	0	0
0	0	0	0	0	0	0	1	0
0	0	0	0	0	0	0	1	0
0	0	0	0	1	0	0	0	0
0	0	0	0	1	0	0	0	0
0	0	0	0	0	0	0	0	0
0	0	0	0	0	0	0	0	0
1	0	0	0	0	0	0	0	0
0	0	0	0	0	0	0	1	0
0	0	0	0	0	0	0	0	0
0	0	0	0	0	0	0	0	0
0	0	0	0	0	0	0	0	0
0	0	0	0	0	0	0	0	0
0	0	0	0	0	0	0	0	0
0	0	0	0	0	0	0	0	0
0	0	0	0	1	0	0	1	0
0	1	0	0	1	0	0	0	0
1	0	0	0	0	0	0	0	0
0	0	0	0	0	0	0	0	0
0	0	0	0	0	0	0	0	0
0	0	0	0	0	0	0	0	0
0	0	0	0	0	0	0	0	0
0	0	0	0	1	0	0	0	0
0	0	0	0	0	0	0	0	0
0	1	0	0	0	0	0	0	0

0	0	0	0	0	0	0	0	0
0	0	0	0	1	0	0	0	0
0	1	0	0	0	0	0	0	0
0	0	0	0	0	0	0	0	0
0	0	0	0	1	0	0	1	0
0	1	0	0	0	0	0	0	0
0	1	1	0	0	0	0	1	0
0	0	0	0	0	0	0	1	0
0	0	0	0	0	0	0	0	0
0	0	0	0	0	0	0	1	0
1	0	0	0	0	0	0	0	0
0	0	0	0	0	0	0	0	0
0	0	0	0	0	0	0	0	0
0	0	0	0	0	0	0	0	0
0	1	0	0	0	0	0	0	0
0	0	0	0	0	0	0	0	0
0	0	0	0	0	0	0	0	0
1	0	0	0	0	0	0	0	0
0	0	0	0	1	0	0	0	0
0	0	0	0	1	0	0	1	0
1	0	0	0	0	0	0	0	0
1	1	0	1	0	0	0	0	0
0	0	0	0	0	0	0	0	0
0	1	0	0	0	0	0	0	0
1	0	0	0	0	0	0	0	0
0	0	0	0	0	0	0	0	0
0	0	0	0	0	0	0	1	0
0	0	0	0	0	0	0	1	0
0	0	0	0	0	0	0	0	0
1	0	0	0	1	0	0	0	0
0	0	0	0	0	0	0	0	0
1	0	0	0	0	0	0	0	0
0	0	0	0	0	0	0	0	0
0	0	1	0	0	0	0	0	0
0	0	0	0	0	0	0	0	0
0	0	1	0	0	0	0	0	0
1	1	0	0	0	0	0	0	0
0	0	0	0	0	0	0	0	0
0	0	0	0	0	0	0	0	0
0	0	0	0	0	0	0	0	0
0	0	0	0	0	0	0	0	0
0	0	1	0	1	1	1	0	0
0	0	0	0	0	0	0	0	0
0	0	0	0	0	0	0	1	0
0	0	0	0	0	0	0	0	0
0	0	0	0	0	0	0	1	0
0	0	0	0	0	0	0	0	0
0	0	0	0	0	0	0	0	0
0	0	0	0	0	0	0	0	0
0	0	0	0	0	0	0	0	0
0	0	0	0	0	0	0	0	0
0	1	0	0	0	0	0	0	0
0	0	0	0	0	0	1	0	0
0	0	0	0	0	0	0	0	0
0	0	0	0	0	0	0	0	0
0	0	0	0	0	0	1	0	0

1	0	0	0	0	0	0	0	0
0	0	0	0	0	0	0	0	0
0	1	0	0	0	0	0	0	0
0	0	0	0	0	0	0	0	0
1	0	0	0	1	0	0	0	0
0	0	0	0	0	0	0	0	1
0	0	1	0	0	0	0	1	0
0	0	1	0	0	0	0	0	0
1	0	0	0	0	0	0	1	0
0	0	0	0	0	0	1	0	0
0	0	0	0	0	0	0	0	0
0	0	0	0	0	0	0	1	0
0	0	0	0	0	0	0	0	0
0	0	0	0	0	0	0	0	0
0	0	0	0	0	0	0	0	0
0	0	0	0	0	0	0	0	0
0	0	0	0	1	0	0	0	0
0	0	0	0	0	0	0	0	0
0	0	0	0	0	0	0	0	0
0	0	0	0	0	0	0	0	0
0	0	0	0	0	0	0	0	0
0	0	0	0	0	0	0	0	0
0	0	0	0	0	0	0	0	0
0	0	0	0	0	0	0	0	0
0	0	0	1	0	0	0	1	0
0	0	0	0	0	0	0	0	0
0	0	0	0	0	0	0	0	0
0	0	0	0	0	0	0	0	0
0	0	0	0	0	0	0	0	0
0	0	0	0	0	0	0	0	0
0	0	0	0	0	0	0	0	0
1	0	0	0	0	0	0	0	0
1	0	0	0	0	0	0	0	0
1	0	0	0	0	0	0	0	0
0	0	0	0	0	0	0	0	0
1	0	0	0	0	0	0	0	0
0	0	0	0	0	0	0	1	0
0	1	0	0	0	0	0	0	0
0	0	0	0	0	0	0	0	0
0	0	0	0	0	0	0	0	0
0	0	0	0	0	0	0	0	0
0	0	0	0	0	0	0	0	0
0	0	0	0	0	0	0	0	0
0	0	0	0	0	0	0	0	0
0	0	0	0	0	0	0	0	0
1	0	0	0	0	0	0	0	0
0	0	0	0	0	0	0	0	0
1	0	1	0	0	0	0	0	0
0	0	0	0	0	0	0	0	0
0	0	0	0	0	0	0	0	0
0	0	0	0	0	0	0	0	0
0	0	0	0	0	0	0	0	0
0	0	0	0	0	0	0	0	0
0	0	0	0	0	0	0	1	0
0	1	0	0	0	0	0	0	0
0	0	0	0	0	0	0	0	0

0	0	0	0	0	0	0	0	0
0	0	0	0	0	0	0	0	0
0	0	0	0	0	0	0	0	0
1	0	0	0	0	0	0	0	0
0	0	0	0	0	0	0	0	0
0	0	0	0	0	0	0	0	0
0	0	0	0	0	0	0	0	0
0	0	0	0	0	0	1	1	0
0	0	0	0	0	0	0	0	0
0	0	0	0	0	0	0	0	0
0	0	0	0	0	0	0	0	0
0	0	0	0	0	0	0	1	0
0	0	0	0	0	0	0	0	0
0	0	0	0	0	0	0	0	0
0	0	0	0	0	0	0	1	0
0	0	0	0	0	0	0	0	0
1	0	0	0	0	0	0	0	0
0	0	0	0	0	0	0	0	0
0	0	0	0	0	0	0	1	0
0	0	0	0	0	0	0	0	0
0	0	0	0	0	0	0	0	0
0	0	0	0	0	0	0	0	0
0	0	0	0	0	0	0	0	0
0	0	0	0	0	0	0	0	0
0	0	0	0	0	0	0	0	0
0	0	0	0	1	0	0	0	0
0	0	0	0	0	0	1	0	0
0	0	0	0	0	0	1	0	0
1	0	0	0	0	0	0	0	0
0	0	0	0	0	0	0	0	0
1	0	0	0	0	0	0	0	0
0	0	0	0	0	0	0	0	0
0	0	0	0	0	0	0	0	0
0	0	0	0	0	0	0	0	0
0	0	0	0	0	0	0	0	0
0	0	0	0	0	0	0	0	0
0	0	0	0	0	0	0	1	1
0	0	0	0	0	0	0	0	0
1	0	0	0	0	0	0	0	0
0	0	0	0	0	0	0	0	0
0	0	0	0	0	0	0	0	0
0	0	0	0	0	0	0	0	0
0	0	0	0	0	0	0	0	0
0	1	0	0	0	0	0	0	0
0	0	0	0	0	0	0	1	0
0	0	0	0	0	0	0	0	0
0	0	0	0	0	0	0	0	0
0	0	0	0	0	0	0	0	0
0	0	0	0	0	0	0	1	0
0	0	0	0	0	0	0	0	0
0	0	0	0	0	0	0	0	0
0	0	0	0	0	0	0	0	0
0	0	0	0	0	0	0	0	0
0	0	0	0	0	0	0	1	0
0	1	0	0	0	0	0	0	0
0	0	0	0	0	0	0	0	0

0	0	0	0	1	0	0	0	0
0	0	0	0	0	0	0	0	0
0	0	0	0	0	0	0	0	0
0	0	0	0	0	0	0	0	0
0	0	0	0	0	0	0	0	0
0	0	0	0	0	0	0	1	0
0	0	0	0	1	0	0	0	0
0	0	0	0	0	0	0	0	0
0	0	0	0	0	0	0	0	0
0	0	0	0	0	0	0	0	1
0	0	0	0	0	0	0	0	0
0	0	0	0	1	0	0	0	0
0	0	0	0	0	0	0	0	0
0	0	0	0	0	0	0	0	0
0	0	0	1	0	0	1	0	0
0	0	0	0	0	0	0	0	0
0	0	0	0	0	0	0	0	0
0	0	0	0	0	0	0	0	0
0	0	0	0	0	0	0	0	0
0	0	0	0	0	0	0	0	0
0	1	0	0	0	0	0	0	0
0	0	0	0	0	0	0	0	0
0	0	0	0	0	0	0	0	0
0	0	0	0	0	0	0	0	0
0	0	1	0	0	0	0	0	0
0	1	0	0	0	0	0	0	0
0	0	0	0	0	0	0	1	0
0	0	0	0	1	0	0	0	0
0	0	0	0	0	0	0	0	0
0	0	0	0	0	0	0	1	0
0	0	0	0	0	0	0	1	0
0	0	0	0	0	0	0	0	0
0	0	0	0	0	0	0	0	0
0	1	0	0	0	0	0	0	0
0	0	0	0	0	0	0	0	0
0	0	0	0	0	0	0	0	0
0	0	0	0	0	0	0	0	0
0	0	0	0	0	0	0	0	0
0	0	0	0	0	0	0	0	0
0	0	0	0	0	0	0	0	0
0	0	0	0	0	0	0	0	0
0	0	0	0	0	0	0	0	0
0	0	0	0	0	0	1	0	0
0	0	0	0	0	0	0	1	0
0	0	0	0	0	0	0	0	0
0	0	0	0	0	0	0	0	0
0	0	0	0	0	0	0	0	0
1	0	0	0	0	0	0	0	0
0	1	0	0	0	0	0	0	0
0	0	0	0	0	0	0	0	0
0	0	0	0	0	0	0	0	0
0	0	0	0	0	0	0	0	0
0	0	0	0	0	0	0	1	0
0	0	0	0	0	0	0	0	0
0	0	0	0	1	0	0	0	0
0	0	0	0	0	0	0	0	0

0	0	0	0	0	0	0	0	0
0	0	0	1	1	0	0	0	0
0	0	0	0	0	0	0	0	0
0	0	0	0	0	0	0	1	0
0	0	0	0	0	0	0	0	0
0	0	1	0	1	0	0	1	0
0	0	0	0	0	0	0	0	0
0	0	0	0	0	0	0	0	0
0	0	0	0	0	0	0	0	0
0	0	0	0	0	0	0	0	0
0	0	0	0	0	0	0	0	0
0	0	0	0	0	0	0	0	0
0	0	0	0	0	0	0	0	0
0	0	0	0	0	0	0	0	0
0	0	0	0	1	0	0	0	0
0	0	0	0	1	0	0	0	0
0	0	0	0	0	0	0	0	0
0	0	0	0	0	0	0	0	0
0	0	0	0	0	0	0	0	0
0	0	0	0	1	0	0	0	0
0	1	0	0	0	0	0	1	0
0	0	0	0	0	0	1	1	0
0	0	0	0	0	0	0	0	0
1	1	0	0	0	0	1	0	0
0	0	0	0	0	0	0	0	0
0	0	0	0	0	0	0	0	0
0	0	0	0	0	0	0	0	0
0	0	0	0	0	0	0	0	0
0	0	0	0	0	0	0	0	0
0	1	0	0	0	0	0	0	0
0	0	0	0	0	0	0	0	0
0	0	0	0	0	0	0	0	1
1	1	0	0	0	0	0	0	0
0	0	0	0	0	0	0	0	0
0	1	0	0	0	0	0	0	0
0	0	0	0	0	0	0	0	0
0	0	0	0	0	0	0	1	0
0	0	0	0	0	0	0	0	0
0	0	0	0	0	0	0	0	0
0	0	0	0	0	0	0	0	0
0	0	0	0	0	0	0	1	0
0	0	0	0	1	0	0	0	0
0	0	0	0	0	0	0	0	0
0	0	0	0	0	0	1	1	0
0	1	0	0	0	0	0	0	0
0	0	0	0	0	0	0	0	0
0	0	0	0	0	0	0	0	0
0	0	0	0	0	0	1	0	0
0	0	0	0	0	0	0	0	0
0	0	0	0	1	0	0	0	0
0	0	0	0	0	0	1	0	0
0	0	0	0	0	0	0	0	0
0	0	0	0	0	0	0	0	0
0	0	0	0	0	0	0	0	0
1	0	0	0	0	0	0	0	0
0	0	0	0	0	0	0	0	0

0	0	0	0	0	0	0	0	0
0	0	0	0	0	0	0	0	0
0	0	0	0	0	0	0	0	0
0	0	0	0	0	0	0	0	0
0	0	0	0	0	0	0	0	0
0	0	0	0	0	0	0	1	0
0	0	0	0	0	0	0	0	0
0	0	0	0	0	0	0	0	0
0	0	0	0	1	0	0	0	0
1	0	0	0	0	0	0	0	0
0	0	0	0	0	0	0	0	0
0	0	0	0	0	0	0	0	0
0	0	0	0	0	0	0	0	0
0	0	0	0	0	0	0	0	0
0	0	0	0	0	0	0	0	0
0	0	0	0	0	0	0	0	0
0	0	0	0	0	0	0	0	0
1	0	0	0	0	0	0	0	0
0	0	1	0	0	0	0	0	0
0	1	0	0	0	0	0	0	0
1	0	0	0	0	0	0	0	0
0	0	0	0	0	0	0	0	0
0	0	0	0	0	0	0	0	0
0	0	0	0	0	0	0	0	0
0	0	0	0	0	0	0	0	0
0	0	0	0	0	0	0	0	0
0	0	0	0	0	0	0	0	0
0	0	0	0	0	0	0	0	0
0	0	0	0	0	0	0	0	0
0	0	0	0	0	0	0	0	0
0	0	0	0	0	0	0	1	0
0	0	0	0	0	0	0	0	0
0	0	0	0	1	0	0	0	0
0	0	0	0	0	0	0	0	0
1	0	0	0	0	0	0	0	0
0	0	0	0	0	0	0	0	0
0	1	0	0	0	0	0	0	0
0	0	0	0	0	0	0	0	0
0	0	0	0	0	0	0	0	0
0	0	1	0	1	0	0	0	0
0	0	0	0	0	0	0	1	0
1	0	0	0	0	0	0	0	0
0	0	0	0	0	0	0	0	0
0	0	0	0	0	0	0	0	0
0	1	0	0	0	0	0	0	0
1	0	0	0	0	0	0	0	0
0	0	0	0	0	0	0	0	0
0	0	0	0	0	0	0	0	0
0	0	0	0	1	0	0	0	0
0	0	0	0	0	0	0	0	0
0	0	0	0	0	0	0	0	0
0	0	0	0	1	0	0	0	0
1	0	0	0	0	0	0	0	0
0	0	0	0	0	0	0	0	0
0	0	0	0	0	0	0	0	0
0	0	0	0	0	0	0	0	0

0	0	0	0	0	0	0	0	0
0	0	0	0	0	0	0	1	0
0	0	0	0	0	0	0	0	0
1	0	0	0	0	0	0	0	0
0	0	0	0	0	0	0	0	0
0	0	0	0	0	0	0	0	0
0	0	0	0	0	0	0	0	0
0	0	0	0	0	0	0	0	0
0	0	0	0	0	0	0	0	0
0	0	0	0	0	0	0	0	0
0	0	0	0	0	0	0	0	0
0	0	0	0	0	0	0	0	0
0	0	0	0	0	0	0	0	0
0	0	0	0	0	0	0	0	0
0	0	0	0	0	0	0	1	0
0	0	0	0	0	0	0	0	0
1	0	0	0	0	0	0	0	1
0	0	0	0	0	0	0	0	0
0	0	0	0	0	0	0	0	0
0	0	0	0	0	0	0	0	0
0	0	0	0	0	0	0	0	0
0	0	0	0	0	0	0	0	0
0	0	0	0	0	0	0	0	0
1	0	0	0	0	0	0	0	0
0	0	0	0	0	0	0	0	0
0	0	0	0	0	0	0	0	0
1	0	0	0	0	0	0	0	0
1	1	0	0	0	0	0	0	0
0	0	0	0	0	0	0	0	0
0	0	0	0	0	0	0	0	0
0	0	0	0	0	0	0	0	0
0	0	0	0	0	0	0	0	0
0	1	0	0	0	0	0	0	0
1	0	0	0	0	0	0	0	0
1	0	0	0	0	0	0	0	0
0	0	0	0	0	0	0	0	0
1	0	0	0	0	0	0	1	0
0	0	0	0	0	0	0	0	0
1	0	0	0	0	0	0	0	0
0	0	0	0	0	0	0	0	0
0	0	0	0	0	0	0	1	0
0	0	0	0	0	0	0	0	0
1	0	0	0	0	0	0	0	0
0	0	0	0	0	0	0	1	0
0	0	0	0	0	0	0	0	0
0	1	0	0	0	0	0	0	0
0	0	0	0	0	0	0	0	0
0	0	0	0	1	0	0	0	0
1	0	0	0	0	0	0	0	0
0	0	0	0	0	0	0	0	0
0	0	0	0	0	0	0	0	0
0	0	0	0	0	0	0	0	0
0	0	0	0	0	0	0	0	0
0	1	0	0	0	0	0	0	0
1	0	0	0	0	0	0	0	0
0	0	0	0	0	0	0	0	0

1	0	0	0	0	0	0	0	0
0	0	0	0	0	0	0	0	0
0	0	0	0	0	0	0	0	0
1	0	0	0	0	0	0	0	0
0	0	0	0	0	0	0	0	0
0	0	0	0	0	0	0	0	0
0	0	0	0	0	0	0	0	0
0	0	0	0	0	0	0	0	0
0	0	0	1	0	0	0	1	0
1	0	0	0	0	0	0	1	0
0	0	0	0	0	0	0	0	0
0	0	0	0	0	0	0	0	0
0	0	0	0	0	0	0	0	0
0	0	0	0	0	0	0	0	0
1	0	0	0	0	0	0	0	0
0	0	0	0	0	0	0	0	0
1	1	0	0	0	0	0	0	0
1	0	0	0	0	0	0	0	0
0	0	0	0	0	0	0	0	0
0	0	0	0	0	0	0	0	0
1	0	0	0	0	0	0	0	0
0	0	0	0	0	0	0	0	0
0	0	0	0	0	0	0	0	0
0	0	0	0	0	0	0	0	0
0	0	0	0	0	0	0	0	0
0	0	0	0	0	0	0	0	0
1	0	0	0	0	0	0	0	0
1	0	0	0	0	0	0	0	0
0	0	0	0	0	0	0	1	0
0	0	0	0	0	0	0	0	0
0	0	0	0	0	0	0	0	0
1	0	0	0	0	0	0	0	0
1	0	0	0	0	0	0	0	0
0	0	0	0	0	0	0	0	0
0	1	0	0	0	0	0	0	0
0	0	0	0	0	0	0	0	0
0	0	0	0	0	0	0	0	0
0	0	0	0	0	0	0	0	0
0	0	0	0	0	0	0	0	0
0	0	0	0	0	0	0	0	0
0	0	0	0	0	0	0	0	0
0	0	0	0	0	0	0	0	0
0	0	0	0	0	0	0	0	0
1	0	0	0	0	0	0	0	0
1	0	0	0	0	0	0	0	0
0	0	0	0	0	0	0	0	0
0	0	0	0	0	0	0	1	0
0	0	0	0	0	0	0	0	0
0	0	0	0	0	0	0	1	0
0	0	0	0	0	0	0	0	0
0	0	0	0	0	0	0	0	0
1	0	0	0	0	0	0	0	0
0	0	0	0	1	0	0	0	0
0	0	0	0	0	0	0	0	0
0	0	0	0	0	0	0	1	0
0	0	0	0	0	0	0	0	0

0	0	0	0	0	0	0	0	0
0	0	0	0	0	0	0	1	0
1	0	0	0	0	0	0	0	0
0	0	0	0	0	0	0	0	0
0	0	0	0	0	0	0	0	0
0	0	0	0	0	0	0	0	0
0	0	0	0	0	0	0	0	0
0	0	0	0	0	0	0	0	0
1	0	0	0	0	0	0	0	0
0	0	0	0	0	0	0	0	0
0	0	0	0	0	0	0	0	0
0	0	0	0	0	0	1	0	0
1	0	0	0	0	0	0	0	0
0	0	0	0	0	0	0	0	0
0	0	0	0	0	0	0	0	0
0	0	0	0	1	0	0	0	0
0	0	0	0	0	0	0	0	0
0	0	0	0	0	0	0	0	0
0	0	0	0	0	0	0	0	0
0	0	0	0	0	0	0	0	0
1	0	0	0	0	0	0	0	0
0	0	0	0	0	0	0	0	0
0	0	0	0	0	0	0	0	0
0	0	0	0	0	0	0	0	0
0	0	0	0	0	0	0	0	0
0	0	0	0	0	0	0	0	0
0	0	0	0	0	0	0	0	0
0	0	0	0	0	0	0	0	0
0	0	0	0	0	0	0	0	0
0	0	0	0	0	0	0	0	0
0	0	0	0	0	0	0	0	0
0	0	0	0	0	0	0	0	0
0	0	0	0	0	0	0	0	0
0	0	0	0	0	0	0	0	0
0	0	0	0	0	0	0	0	0
0	0	0	0	0	0	0	0	0
0	0	0	0	0	0	0	0	0
0	0	0	0	0	0	0	0	0
0	0	0	0	0	0	0	0	0
0	0	0	0	0	0	0	0	0
1	0	0	0	0	0	0	0	0
0	0	0	0	0	0	0	0	0
0	0	0	0	0	0	0	0	0
1	0	0	0	0	0	0	0	0
0	1	0	0	0	0	0	1	0
0	0	0	0	0	0	0	0	0
1	0	0	0	0	0	0	0	0
0	0	0	0	0	0	0	0	0
1	0	0	0	1	0	0	0	0
1	0	0	0	0	0	0	0	0
0	0	0	0	1	0	0	0	0
1	0	0	0	0	0	0	0	0
0	0	0	0	0	0	0	0	0
0	0	0	0	0	0	0	0	0
0	0	0	0	0	0	0	0	0
0	0	0	0	0	0	0	0	0

0	0	0	0	0	0	1	0	0
0	0	0	0	0	0	0	0	0
0	0	0	0	0	0	0	0	0
0	0	0	0	0	0	0	0	0
0	0	0	0	0	0	0	0	0
0	0	0	0	0	0	0	0	0
0	0	1	0	0	0	1	0	0
1	0	0	0	0	0	0	0	0
1	1	0	0	0	0	0	1	0
1	0	0	0	0	0	0	0	0
0	0	0	0	0	0	0	0	0
0	0	0	0	0	0	0	0	0
0	0	0	0	0	1	0	0	0
0	0	0	0	0	0	0	1	0
0	0	0	0	0	0	0	0	0
0	0	0	0	0	0	0	1	0
0	0	0	0	0	0	0	0	0
1	0	0	0	0	0	0	0	0
0	0	0	0	0	0	0	0	0
0	1	0	0	0	0	0	0	0
0	0	0	0	0	0	0	0	0
0	0	1	0	0	0	0	0	0
0	0	0	0	0	0	0	0	0
0	0	0	0	0	0	0	0	0
1	0	0	0	0	0	0	0	0
0	0	1	0	0	0	0	0	0
0	0	0	0	0	0	0	0	0
0	0	0	0	0	0	0	1	0
0	0	0	0	0	0	0	0	0
1	0	0	0	0	0	1	0	0
0	0	0	0	0	0	0	0	0
0	0	0	0	0	0	0	0	0
0	0	0	0	0	0	0	0	0
0	0	0	0	0	0	0	0	0
0	1	0	0	0	0	0	1	0
0	0	0	0	0	0	0	0	0
0	0	0	0	0	1	0	0	0
1	0	1	0	0	0	0	0	0
0	0	0	0	0	0	0	0	0
1	0	0	0	0	0	0	0	0
0	0	0	0	0	0	0	0	0
0	0	0	0	0	0	0	0	0
0	0	0	0	0	0	0	0	0
0	0	0	0	0	0	0	0	0
0	1	0	0	0	0	0	0	0
1	0	0	0	0	0	0	1	0
0	0	0	0	0	0	0	0	0
0	0	0	0	0	0	0	0	0
1	0	0	0	0	0	0	0	0
0	0	0	0	0	0	0	0	0
1	0	0	0	0	0	0	0	0
1	0	0	0	0	0	0	0	0
0	0	0	0	0	0	0	0	0
0	0	0	0	0	0	0	1	0
0	0	0	0	0	0	0	0	0
0	0	0	0	0	0	0	1	0

0	0	0	0	0	0	0	0	0
0	0	0	0	0	0	0	1	0
1	0	0	1	0	0	0	0	0
0	0	0	0	0	0	0	0	0
0	0	0	0	0	0	0	0	0
1	0	0	0	0	0	0	0	0
0	1	0	0	0	0	0	0	0
0	0	0	0	0	0	0	0	0
1	0	0	0	0	0	0	0	0
0	0	0	0	0	0	0	0	0
0	0	0	0	0	0	1	0	0
1	0	0	0	0	0	0	0	0
0	0	0	0	0	0	0	0	0
0	0	0	0	0	0	0	0	0
0	0	0	0	0	0	0	0	0
0	0	0	0	0	0	0	0	0
0	0	0	0	0	0	0	0	0
1	0	0	0	0	0	0	0	0
0	0	0	0	0	0	0	0	0
0	0	0	0	0	0	0	0	0
1	0	0	0	0	0	0	0	0
1	1	0	0	0	0	0	0	0
0	0	0	0	0	0	0	0	0
0	0	0	0	0	0	0	0	0
0	0	0	0	0	0	0	0	0
0	0	0	0	0	0	0	1	0
0	0	0	0	0	0	0	0	0
0	0	0	0	0	0	0	0	0
0	0	1	0	0	0	0	0	0
0	0	0	0	0	0	0	0	0
0	0	0	0	0	0	0	0	0
0	1	0	0	0	0	0	0	0
0	0	0	0	0	0	1	0	0
0	0	0	0	0	0	0	0	0
0	0	0	0	0	0	0	0	0
0	0	0	0	0	0	0	0	0
0	0	0	0	0	0	0	0	0
0	0	0	0	0	0	0	0	0
0	0	0	0	0	0	0	0	0
0	0	0	0	0	0	0	0	0
0	0	0	0	0	0	0	0	0
0	0	0	0	0	0	0	1	0
1	0	0	0	0	0	0	0	0
0	0	0	0	0	0	0	0	0
1	0	0	0	0	0	0	0	0
0	0	0	0	0	0	0	0	0
0	0	0	0	0	0	0	0	0
0	0	0	0	0	0	0	0	0
0	0	0	0	0	0	0	0	0
1	0	0	0	0	0	0	0	0
0	1	0	0	0	0	0	0	0
0	0	0	0	0	0	0	0	0
0	0	0	0	0	0	0	1	0
0	0	0	0	0	0	0	0	0
1	0	0	0	0	0	0	0	0
0	0	0	0	0	0	0	0	0

0	0	0	0	0	0	0	0	0
0	0	0	0	0	0	0	0	0
0	0	0	0	0	0	0	1	0
0	0	0	0	0	0	0	0	0
0	0	0	1	0	0	0	0	0
0	0	0	0	0	0	0	0	0
0	0	0	0	0	0	0	0	0
0	0	0	0	0	0	0	0	0
0	0	0	0	0	1	0	0	0
0	0	0	0	0	0	0	0	0
0	0	0	0	0	0	0	0	0
0	0	0	0	0	0	0	0	0
0	0	0	0	0	0	0	0	0
0	0	0	0	0	0	0	0	0
1	0	0	0	0	0	0	0	0
0	0	0	0	1	0	0	0	0
0	0	0	0	0	0	0	0	0
0	0	0	0	0	0	0	0	0
1	0	0	0	0	0	0	0	0
0	0	0	0	0	0	0	0	0
0	0	0	0	0	0	0	0	0
0	0	0	0	0	0	0	0	0
1	0	0	0	0	0	0	0	0
0	0	0	0	0	0	0	0	0
0	0	0	0	0	0	0	0	0
0	0	0	0	0	0	0	0	0
1	0	0	0	0	0	0	0	0
0	1	0	0	0	0	0	0	0
0	0	0	0	0	0	0	0	0
0	0	0	0	0	0	0	0	0
1	0	0	0	0	0	0	0	0
0	0	0	0	0	0	0	0	0
0	0	0	0	0	0	0	0	0
0	0	0	0	0	0	0	0	0
0	0	0	0	0	0	0	0	0
0	0	0	0	0	0	0	0	0
0	0	0	0	0	0	0	0	0
0	0	0	0	0	0	0	0	0
0	0	0	0	0	0	0	0	0
0	0	1	0	0	0	0	0	0
0	0	0	0	0	0	0	0	0
1	0	0	0	0	0	0	0	0
1	0	0	0	0	0	0	0	0
1	0	0	0	1	0	0	0	0
1	0	1	0	0	0	0	0	0
0	1	0	0	0	0	0	0	0
1	0	1	0	0	0	0	0	0
0	0	0	0	0	0	0	0	0
0	0	0	0	0	0	0	0	0
1	0	0	0	0	0	0	0	0
0	1	0	0	0	0	0	0	0
0	0	0	0	0	0	0	0	0
0	0	0	0	0	0	0	0	0
0	0	0	0	0	0	0	0	0
1	0	0	0	1	0	0	0	0
0	0	0	0	0	0	0	0	0

0	0	0	0	0	0	0	0	0
0	0	0	0	0	0	0	0	0
0	1	0	0	0	0	0	0	0
0	0	0	0	0	0	0	0	0
0	0	1	0	0	0	0	0	0
0	0	0	0	0	0	0	0	0
0	0	0	0	0	0	0	0	0
0	0	0	0	0	0	0	0	0
0	0	0	0	0	0	0	0	0
0	0	0	0	0	0	0	0	0
1	0	0	0	0	0	0	0	0
0	0	0	0	0	0	0	0	0
0	0	0	0	0	0	0	0	0
0	0	0	0	0	0	0	0	0
0	0	0	0	0	0	0	0	0
0	0	0	0	0	0	0	0	0
0	0	0	0	0	0	0	0	0
0	0	0	0	0	0	0	0	0
0	0	0	0	0	0	0	0	0
0	0	0	0	1	0	0	0	0
0	0	0	0	0	0	0	0	0
0	0	0	0	0	0	0	0	0
1	0	0	0	0	0	0	1	0
0	0	0	0	0	0	0	1	0
0	0	0	0	0	0	0	0	0
0	0	0	0	0	0	0	0	0
1	0	0	0	0	0	0	0	0
0	0	0	0	0	0	0	1	0
1	0	0	0	1	0	0	0	0
0	0	0	0	0	0	0	0	0
1	0	0	0	0	0	0	0	0
0	0	0	0	0	0	1	0	0
0	0	0	0	0	0	0	0	0
0	0	0	0	0	0	0	0	0
0	0	0	0	0	0	0	0	0
0	0	0	0	0	0	0	0	0
0	0	0	0	0	0	0	0	0
1	0	0	0	0	0	0	0	0
0	0	0	0	0	0	0	0	0
0	0	0	0	0	0	0	1	0
0	0	0	1	0	0	0	0	0
0	0	0	0	0	0	0	1	0
1	0	0	0	0	0	0	0	0
1	0	0	0	0	0	0	0	0
0	0	0	0	0	0	0	0	0
0	0	0	0	0	0	0	1	0
1	1	0	0	0	0	0	0	0
1	0	0	0	0	0	0	0	0
0	0	0	0	0	0	0	0	0
1	0	0	0	0	0	0	0	0
0	0	0	0	0	0	0	0	0
0	0	0	0	0	0	0	0	0
0	0	0	0	0	0	0	0	0
0	0	0	0	0	0	0	0	0
0	0	0	0	0	0	0	0	0
0	0	0	0	0	0	0	0	0

0	0	0	0	0	0	0	0	0
0	0	0	0	0	0	0	0	0
0	0	0	1	0	0	0	0	0
0	0	0	0	0	0	0	0	1
0	0	0	0	0	0	0	0	0
0	0	0	0	0	0	0	0	0
0	0	0	0	0	0	0	0	0
0	0	0	0	0	0	0	0	0
0	0	0	0	0	0	0	0	0
0	0	0	0	0	0	0	0	0
0	0	0	0	0	1	0	0	0
1	0	0	0	0	0	0	0	0
0	0	0	0	0	0	0	0	0
0	0	0	0	0	0	0	0	0
0	0	0	0	0	0	0	0	1
0	0	0	0	0	0	0	0	0
0	0	0	0	0	0	0	0	0
0	0	0	0	0	0	0	0	0
0	0	0	0	0	0	0	1	0
1	1	0	0	0	0	0	0	0
0	0	0	0	0	0	0	0	0
0	0	0	1	0	0	0	1	0
0	0	0	0	0	0	0	0	0
0	0	0	0	0	0	0	0	0
1	0	0	0	0	0	0	0	0
0	0	0	0	0	0	0	0	0
0	0	0	0	0	0	0	0	0
1	0	0	0	0	0	0	0	0
0	0	0	0	0	0	0	1	0
0	0	0	0	0	0	0	0	0
0	0	0	0	0	0	0	0	0
0	0	0	0	0	0	0	0	0
1	1	1	0	0	0	0	0	0
0	0	0	0	0	0	0	0	0
0	0	0	0	0	0	0	0	0
0	0	0	0	0	0	0	0	0
0	0	0	0	0	0	0	0	0
1	0	0	0	0	0	0	0	0
1	0	0	0	0	0	0	0	0
0	0	0	0	0	0	0	1	0
0	0	0	0	0	0	0	0	0
0	0	0	0	0	0	1	0	0
1	0	0	0	0	0	0	0	0
0	0	0	0	0	0	0	0	0
0	0	0	0	0	0	0	0	0
1	0	0	0	0	0	0	0	0
0	0	0	0	0	0	0	0	0
0	0	0	0	0	0	0	0	0
0	0	0	0	0	0	0	0	0
0	0	0	0	0	0	0	0	0
0	0	0	0	0	0	0	1	0
1	0	0	0	0	0	0	0	0
0	0	0	0	0	0	0	0	0
0	0	0	0	0	0	0	0	0
0	0	0	0	0	0	0	0	0
0	0	0	0	0	0	0	0	0

0	0	0	0	1	0	0	0	0
0	0	0	0	0	0	0	0	0
0	0	0	0	0	0	0	0	0
0	0	0	0	0	0	0	0	0
0	0	0	0	0	0	0	0	0
0	0	0	0	0	0	0	0	0
0	0	0	0	0	0	0	0	0
1	0	0	0	0	0	0	0	0
0	0	0	0	0	0	0	0	0
1	0	0	0	0	0	0	0	0
0	0	0	0	0	0	0	1	0
0	0	0	0	0	0	0	0	0
0	0	0	0	0	0	0	0	0
0	0	0	0	0	0	0	0	0
0	0	0	0	0	0	0	0	0
0	0	0	0	0	0	0	0	0
0	0	0	0	1	0	0	0	0
0	1	0	0	0	0	0	0	0
0	0	0	0	1	0	0	1	0
0	0	0	0	0	0	0	0	0
0	0	0	0	0	0	0	0	0
0	0	0	0	0	0	0	0	0
0	0	0	0	0	0	0	0	0
0	0	0	0	0	0	0	0	1
0	1	0	0	0	0	0	1	0
0	0	0	0	0	0	0	0	0
1	1	0	0	0	0	0	0	0
0	0	0	0	0	0	0	0	0
0	0	0	0	1	1	0	0	0
1	0	0	0	1	0	0	0	0
0	0	0	0	0	0	0	0	0
0	0	0	0	0	0	0	0	0
0	0	0	0	0	0	0	0	0
0	0	0	0	0	0	0	0	0
0	0	0	0	0	0	0	0	0
1	0	0	0	0	0	0	0	0
0	0	0	0	0	0	0	0	0
0	0	0	0	0	0	0	0	0
0	0	0	0	0	0	0	0	0
0	0	0	0	0	0	0	0	0
0	0	0	0	0	0	1	0	0
1	0	1	0	0	0	0	0	0
1	0	1	0	0	0	0	0	0
0	0	0	0	0	0	0	0	0
1	0	0	0	0	0	0	0	0
1	0	0	0	0	0	0	0	0
0	0	0	0	0	0	0	0	0
0	0	0	0	0	0	0	1	0
0	0	0	0	1	0	0	0	0
0	0	0	0	0	0	0	0	0
0	0	0	0	0	0	0	1	0
1	0	0	0	0	0	0	0	0
1	0	0	0	0	0	0	1	0
0	0	0	0	0	0	0	0	0
0	0	0	0	0	0	0	0	0
1	0	0	0	0	0	0	0	0

0	0	0	0	0	0	0	0	0
1	0	0	0	0	0	0	0	0
0	0	0	0	0	0	0	0	0
0	0	0	0	0	0	0	0	0
0	0	0	0	0	0	0	0	0
0	0	0	0	0	0	0	0	0
0	0	0	0	0	0	0	0	0
0	0	0	0	0	0	0	0	0
0	0	0	0	0	0	0	0	0
0	0	0	0	0	0	0	0	0
0	0	0	0	0	0	0	0	0
0	0	0	0	0	0	0	0	0
0	0	0	0	0	0	0	0	0
0	0	0	1	0	0	0	1	0
0	0	0	1	0	0	0	0	0
0	0	0	0	0	0	0	0	0
0	0	0	0	0	0	0	0	0
0	0	0	0	0	0	0	0	0
0	0	0	0	0	0	0	0	0
0	0	0	0	0	0	0	0	0
1	0	0	0	0	0	0	0	0
1	0	0	0	0	0	0	0	0
0	0	0	0	0	0	0	0	0
0	0	0	0	0	0	0	0	1
0	0	0	0	0	0	0	0	0
0	0	0	0	0	0	0	0	0
1	0	0	0	0	0	0	0	0
0	0	0	0	0	0	0	1	0
0	0	0	0	0	0	0	0	0
0	0	0	0	0	0	0	0	0
1	0	0	0	1	0	0	0	0
0	0	0	0	0	0	0	0	0
0	0	0	0	0	0	0	0	0
1	0	0	0	0	0	0	0	0
0	0	0	0	0	0	0	0	0
0	0	0	0	0	0	0	1	0
0	0	0	0	0	0	0	0	0
0	1	0	0	0	0	0	0	0
0	0	0	0	0	0	0	0	0
0	0	0	0	0	0	0	0	0
0	0	0	0	0	0	0	0	0
0	0	0	0	0	0	0	0	0
0	0	0	0	0	0	0	0	0
0	0	0	0	0	0	0	0	0
1	0	0	0	0	0	0	0	0
0	0	0	0	0	0	0	0	0
0	0	0	0	0	0	0	0	0
1	0	0	0	0	0	0	0	0
0	0	0	0	0	0	0	0	0
0	0	0	0	0	0	0	0	0
0	0	0	0	0	0	0	0	0
0	1	0	0	0	0	0	0	0
0	0	0	0	0	0	0	0	0
0	0	0	0	0	0	0	0	0
0	0	0	0	0	0	0	0	0
0	0	0	0	0	0	0	0	0

0	0	0	0	0	0	0	0	0
0	0	0	0	1	0	0	0	0
0	0	0	0	0	0	0	0	0
0	0	0	0	0	0	0	1	0
0	0	0	0	0	0	0	0	0
0	0	0	0	0	0	0	0	0
0	0	0	0	0	0	0	0	0
0	0	0	0	0	0	1	0	0
0	0	0	0	0	0	0	0	0
0	0	0	0	0	0	0	0	0
0	0	0	0	0	0	0	0	0
1	1	0	0	0	0	0	0	0
1	0	0	0	1	0	0	0	0
0	0	0	0	0	0	0	0	0
0	0	0	0	1	0	0	1	0
0	0	0	0	0	0	0	0	0
0	0	0	0	0	0	0	0	0
0	0	0	0	0	0	0	0	0
0	0	0	0	0	0	0	1	0
0	0	0	0	0	0	0	0	0
1	0	0	0	0	0	0	0	0
0	0	0	0	0	0	0	1	0
0	0	0	0	0	0	0	0	0
1	0	0	0	0	0	0	0	0
0	0	0	0	0	0	0	0	0
0	0	0	0	0	0	0	1	0
0	0	0	0	0	0	0	0	0
1	0	0	0	0	0	0	0	0
0	0	0	0	0	0	0	0	0
0	1	0	0	0	0	0	0	0
0	0	0	0	0	0	0	0	0
0	0	0	0	0	0	0	0	0
0	0	0	0	0	0	0	1	0
1	0	0	0	0	0	0	0	0
0	0	0	0	0	0	0	0	0
0	0	0	0	0	0	0	0	0
0	0	0	0	0	0	0	0	0
0	1	0	0	0	0	0	0	0
0	0	0	0	0	1	0	0	0
0	0	0	0	0	0	0	0	0
1	0	0	0	0	0	0	0	0
0	0	0	0	0	0	0	1	0
0	0	0	0	0	0	0	0	0
1	0	0	0	0	0	0	0	0
0	0	0	0	0	0	0	0	0
0	0	0	0	0	0	0	0	0
0	0	0	0	0	0	0	0	0
0	0	0	0	0	0	0	0	0
0	0	0	0	0	0	0	0	0
0	0	0	0	0	0	0	0	0
1	0	0	0	0	0	0	0	0
0	0	0	0	0	0	0	0	0
0	0	0	0	0	0	0	0	0
0	0	0	0	0	0	0	0	0
1	0	0	0	0	0	0	0	0
0	0	0	0	0	0	0	0	0

0	0	0	0	0	0	0	0	0
0	0	0	0	0	0	0	0	0
0	0	0	0	0	0	0	0	0
0	0	0	0	0	0	0	0	0
0	0	0	0	0	0	0	0	0
0	0	0	0	0	0	0	0	0
0	0	1	0	0	0	0	0	0
0	0	0	0	0	0	0	0	0
0	0	0	0	0	0	0	0	0
0	0	0	0	0	0	0	0	0
0	0	0	0	0	0	0	0	0
0	1	0	0	0	0	0	1	0
0	0	0	0	0	0	0	0	0
0	0	0	0	0	0	0	0	0
1	0	0	0	0	0	0	0	0
0	0	0	0	0	0	0	0	0
0	1	0	0	0	0	0	0	0
0	0	0	0	0	0	0	0	0
1	0	0	0	0	0	0	0	0
0	0	0	0	0	0	0	0	0
1	0	0	0	0	0	0	0	0
0	0	0	0	1	0	0	0	0
0	0	0	0	0	0	0	0	0
1	0	0	0	0	0	0	1	0
0	1	0	0	0	0	0	0	0
0	0	0	0	0	0	0	0	0
0	0	0	0	0	0	0	0	0
0	0	0	0	1	0	0	0	0
0	0	0	0	1	0	0	0	0
0	1	0	0	0	0	0	0	0
0	0	0	1	0	0	0	0	0
0	0	0	0	0	0	0	1	0
0	0	0	0	0	0	0	1	0
0	0	0	0	0	0	0	0	0
0	0	0	0	0	0	0	0	0
0	0	0	0	0	0	0	0	0
0	0	0	0	0	0	0	0	0
0	0	0	0	0	0	0	0	0
0	0	0	0	0	0	0	1	0
0	0	0	0	0	0	0	0	0
0	0	0	0	0	0	0	0	0
0	0	0	0	0	0	0	1	0
0	0	0	0	0	0	0	0	0
0	0	0	0	0	0	0	0	0
0	0	0	0	0	0	1	0	0
1	0	0	0	0	0	0	0	0
1	0	0	0	0	0	0	0	0
0	0	0	0	0	0	0	0	1
0	0	0	0	0	0	0	0	0
0	0	0	0	0	0	0	0	0
0	0	0	0	0	0	0	0	0
0	0	0	0	0	0	0	0	0
0	0	0	0	0	0	0	0	0
0	0	0	0	0	0	0	0	0
0	0	1	0	0	0	0	0	0
0	0	0	0	0	0	0	0	0

0	0	0	0	0	0	0	0	0
0	0	0	0	0	0	0	0	0
0	0	0	0	0	0	0	1	0
0	0	0	0	0	0	0	0	0
0	0	0	0	0	0	0	0	0
0	0	0	0	0	0	0	0	0
0	0	1	0	0	0	0	0	0
0	1	0	0	0	0	0	1	0
0	0	0	0	0	0	0	0	0
0	0	0	0	0	0	0	0	1
0	0	0	0	0	0	0	0	0
0	1	0	1	0	0	0	0	0
0	0	0	0	0	0	0	0	0
0	0	0	0	0	0	0	0	0
0	0	0	0	0	0	0	0	0
0	0	0	1	0	0	0	0	0
0	0	0	0	0	0	0	0	0
0	0	0	0	0	0	0	0	0
0	0	0	0	0	0	0	0	0
1	0	0	0	0	0	0	0	0
0	0	0	0	0	0	0	0	0
0	0	0	0	0	0	0	0	0
0	0	0	0	0	0	0	1	0
0	0	0	0	0	0	0	0	0
0	0	0	0	0	0	0	0	0
0	1	1	0	0	0	0	0	0
0	0	0	0	0	0	0	0	0
1	0	0	0	0	0	0	0	0
0	0	0	0	0	0	0	0	0
0	0	0	0	0	0	0	0	0
0	0	0	0	0	0	0	0	0
0	0	0	0	0	0	0	0	0
0	0	0	0	0	0	0	0	0
0	0	0	0	0	0	0	0	0
0	0	0	0	0	0	0	0	0
0	0	0	0	0	0	0	0	0
0	0	0	0	0	0	0	0	0
0	0	0	0	0	0	0	0	0
1	0	0	0	0	0	0	0	0
0	0	0	0	0	0	0	0	0
0	0	0	0	0	0	0	0	0
0	0	0	0	1	0	0	0	0
0	0	0	0	0	0	0	0	0
0	0	0	0	0	0	0	1	0
1	0	0	0	0	0	0	0	0
0	0	0	0	0	0	0	0	0
0	1	0	0	0	0	0	0	0
0	0	0	0	0	0	0	0	0
1	0	0	0	0	0	0	0	0
1	0	0	0	0	0	0	0	0
0	0	0	0	0	0	0	0	0
1	0	0	0	0	0	0	0	0
0	0	0	0	0	0	1	0	0
0	0	0	0	0	0	0	0	0
0	0	1	0	0	0	0	1	0
0	0	0	0	0	0	0	0	0

1	0	0	0	0	0	0	1	0
0	0	0	0	0	0	0	0	0
0	0	0	0	0	0	0	1	0
1	0	0	0	0	0	0	0	0
1	0	0	0	0	0	0	0	0
0	0	0	0	0	0	0	0	0
0	0	0	0	0	0	0	0	0
0	0	0	1	0	0	0	0	1
0	0	0	0	0	0	0	0	1
0	0	0	0	0	0	0	0	0
0	0	0	0	0	0	0	0	0
0	0	0	0	0	0	0	0	0
1	0	0	0	0	0	0	0	0
0	0	0	0	0	0	0	0	0
0	0	0	0	0	0	0	0	0
1	0	0	0	1	0	0	0	0
0	0	0	0	0	0	0	0	0
0	1	0	0	0	0	0	0	0
1	0	0	0	0	0	0	0	0
0	0	0	0	0	0	0	0	0
0	0	0	0	0	0	0	1	0
1	0	0	0	0	0	0	0	0
0	0	0	0	0	0	0	0	0
0	0	0	0	0	0	0	0	0
0	1	0	0	0	0	0	0	0
1	0	0	0	0	0	0	1	0
1	0	0	0	0	0	0	0	0
1	0	0	0	0	0	0	0	0
0	0	0	0	0	0	0	0	0
1	0	0	0	0	0	0	0	0
0	0	0	0	0	0	0	0	0
0	0	0	1	0	0	1	0	0
1	0	0	0	0	0	0	0	0
1	0	0	0	0	0	0	0	0
0	0	0	0	0	0	0	0	0
1	0	0	0	0	0	0	0	0
0	0	0	0	0	0	0	0	0
0	0	0	0	0	0	0	1	0
0	0	0	0	0	0	0	0	0
0	0	0	0	0	0	0	0	0
0	0	0	0	0	0	0	0	0
0	0	0	0	0	0	0	0	0
0	0	0	1	0	0	0	0	0
0	0	0	0	0	0	0	0	0
0	1	0	0	0	0	0	0	0
0	0	0	0	0	0	0	0	0
0	0	0	0	0	0	0	0	0
0	0	0	0	1	0	1	0	0
0	0	0	0	0	0	0	0	0
0	0	0	0	0	0	0	0	0
0	0	0	0	0	0	0	0	0
0	0	0	0	0	0	0	0	0
0	0	0	0	0	0	0	0	0
0	0	0	0	0	0	0	1	0
1	0	0	0	0	0	0	0	0
0	0	0	0	0	0	0	0	0

0	0	0	0	0	0	0	0	0
0	0	0	0	0	0	0	0	0
0	0	0	0	0	0	0	0	0
0	0	0	0	0	0	0	0	0
0	0	0	0	0	0	0	0	0
0	0	0	0	0	0	0	0	0
0	0	0	0	0	0	0	0	0
0	0	0	0	0	0	0	1	0
0	1	0	0	0	0	0	0	0
0	0	0	0	0	0	0	1	0
0	0	0	0	0	0	0	0	0
0	0	0	0	0	0	0	0	0
0	0	0	0	0	0	0	0	0
0	0	0	0	0	0	0	0	0
0	0	0	0	0	0	0	0	0
0	0	0	0	0	0	0	0	0
0	0	0	0	0	0	0	0	0
0	0	0	0	0	0	0	0	0
0	0	0	0	0	0	0	1	0
0	0	0	0	0	0	0	0	0
0	0	0	0	0	0	0	0	0
0	0	0	0	0	0	1	0	0
1	0	0	0	0	0	0	0	0
0	0	0	0	0	0	0	0	0
0	0	0	0	0	0	0	0	0
1	0	0	0	0	0	0	0	0
0	0	0	0	0	0	0	0	0
0	0	0	0	0	0	0	0	0
0	0	0	0	0	0	0	0	0
0	0	0	0	0	0	0	1	0
1	0	0	0	0	0	0	0	0
0	0	0	0	0	0	0	1	0
0	0	0	0	0	0	0	0	0
1	0	0	0	0	0	0	0	0
1	0	0	0	0	0	0	0	0
0	0	0	0	0	0	0	0	0
0	0	1	0	0	0	0	0	0
0	0	0	0	0	0	0	0	0
0	0	0	0	1	0	0	0	0
0	0	0	0	0	0	0	0	0
0	0	0	0	0	0	1	0	0
0	0	0	0	0	0	0	0	0
0	1	0	0	0	0	0	0	0
1	0	0	0	0	0	0	0	1
0	0	0	0	0	0	0	0	0
0	0	0	0	0	0	0	1	0
0	0	0	0	0	0	0	1	0
0	0	0	0	0	0	0	0	1
0	0	0	0	0	0	0	0	0
0	0	0	0	0	0	0	0	0
0	0	0	0	0	0	0	0	0
0	0	0	0	0	0	0	0	0
1	0	0	0	0	0	0	0	0
0	0	1	0	0	0	0	0	0
0	0	0	0	0	0	0	0	0
1	0	0	0	0	0	0	0	0

0	0	0	0	0	0	0	0	0
0	0	0	0	0	0	0	0	0
0	0	0	0	0	0	0	0	0
0	0	0	0	0	0	0	0	0
1	0	0	0	0	0	0	0	0
0	0	0	0	0	0	0	0	0
0	0	0	0	0	0	1	0	0
1	0	0	0	0	0	0	0	0
0	1	0	0	0	0	0	0	0
0	0	0	0	0	0	0	0	0
0	0	0	0	0	0	0	0	0
0	1	0	0	0	0	1	0	0
1	0	0	0	0	0	1	0	0
0	0	0	0	1	0	0	0	0
0	1	0	0	0	0	0	0	0
0	1	0	0	0	0	0	0	1
0	0	0	0	0	0	0	0	0
0	0	0	0	0	0	0	0	0
0	0	0	0	0	0	0	0	0
0	0	0	0	0	0	0	0	0
0	0	0	0	0	0	0	1	0
0	0	0	0	0	0	0	0	0
0	0	0	0	0	0	0	0	0
0	0	0	0	0	0	0	0	0
0	0	0	0	0	0	0	0	0
0	0	0	0	0	0	0	0	0
0	0	0	0	0	0	1	0	0
0	0	0	0	1	0	0	0	0
0	0	0	0	0	0	0	0	0
1	0	0	0	0	0	0	0	1
0	0	0	0	0	0	0	0	0
0	0	0	0	0	0	0	0	0
0	0	0	0	0	0	0	0	0
1	1	0	0	0	0	0	0	0
0	0	0	0	0	0	0	1	0
0	0	0	0	0	0	0	0	0
1	0	0	0	1	0	0	0	0
1	0	0	0	0	0	0	0	0
0	1	0	0	0	0	0	0	1
0	0	0	0	0	0	0	0	0
0	0	0	0	0	0	0	0	0
0	0	0	0	0	0	0	0	0
0	0	0	0	1	0	0	0	0
0	0	0	0	0	0	0	0	0
0	0	0	0	0	0	0	1	0
0	0	0	0	0	0	0	0	0
0	0	0	0	0	0	0	1	0
0	0	0	0	0	0	0	0	0
1	0	0	0	0	0	0	0	0
0	0	0	0	0	0	0	0	0
1	0	0	0	0	0	0	0	0
1	0	0	0	0	0	0	1	0
0	0	0	0	0	0	0	0	0
0	0	0	0	0	0	0	0	0
1	0	0	0	0	0	0	0	0
0	0	0	0	0	0	0	0	0

0	0	0	0	0	0	0	0	0
1	0	0	0	0	0	0	1	0
1	0	0	0	0	0	0	0	0
1	0	0	0	0	0	0	0	0
0	0	0	0	0	0	0	0	0
0	0	0	0	0	0	0	0	0
0	0	0	0	0	0	0	0	0
0	0	0	0	0	0	0	0	0
0	0	0	0	0	0	0	0	0
0	0	0	0	0	0	0	0	0
1	0	0	0	0	0	0	0	0
0	0	0	0	0	0	0	0	0
0	0	0	0	0	0	0	0	0
1	0	0	0	0	0	0	0	0
0	0	0	0	0	0	0	0	0
0	0	0	0	0	0	0	0	0
0	0	1	0	0	0	0	0	0
1	0	0	0	1	0	0	0	0
0	0	0	0	0	0	0	0	0
0	0	0	0	0	0	0	0	0
0	0	0	0	0	0	0	0	0
0	0	0	0	0	0	0	0	0
1	0	0	0	0	0	0	0	0
0	0	0	0	0	0	0	0	0
0	0	0	0	0	0	0	0	0
0	0	0	0	0	0	0	0	0
0	0	0	0	0	0	0	0	0
0	0	0	0	0	0	1	0	0
0	0	0	0	0	0	0	0	0
0	0	0	0	0	0	0	0	0
0	0	0	0	0	0	0	1	0
1	0	0	0	0	0	0	0	0
0	0	0	0	0	0	0	0	0
0	0	0	0	0	0	0	0	0
0	1	0	0	0	0	0	0	0
1	0	0	1	0	0	0	0	0
1	0	0	0	0	0	0	0	0
0	0	0	0	0	0	0	0	0
0	0	0	0	0	0	0	1	0
1	0	0	0	0	0	0	0	0
1	0	0	0	0	0	0	0	0
0	0	0	0	0	0	0	0	0
0	0	0	0	0	0	0	1	0
0	0	0	0	0	0	0	0	0
0	1	0	0	0	0	0	0	0
0	0	0	0	0	0	0	1	0
0	0	0	0	0	0	0	0	0
0	1	0	0	0	0	0	0	0
0	0	0	0	0	0	0	0	0
0	0	0	0	0	0	0	0	0
1	0	0	0	0	0	0	0	0
0	0	0	0	0	0	0	1	0
0	0	0	0	0	0	0	0	0
0	0	0	0	0	0	0	0	0
0	0	0	0	0	0	0	0	0
1	0	0	0	0	0	1	0	0

0	0	0	0	0	0	0	1	0
1	0	0	0	0	0	0	0	0
0	0	0	0	0	0	0	1	0
1	0	0	0	0	0	0	0	0
1	0	0	0	0	0	0	0	0
0	0	0	1	0	0	0	0	0
1	0	0	0	0	0	0	0	0
0	0	0	0	0	0	0	0	0
1	0	0	0	0	0	0	0	0
0	0	0	0	1	0	0	0	0
0	0	0	0	0	0	0	0	0
0	0	0	0	0	0	0	0	0
0	0	0	0	0	0	0	0	0
0	0	0	0	0	0	0	0	0
0	0	0	0	0	0	0	0	0
0	0	0	0	0	0	1	0	0
0	0	0	0	0	0	0	0	0
0	1	0	0	0	0	0	0	0
1	0	0	0	0	1	0	0	0
0	1	0	0	0	0	0	0	0
0	0	0	0	0	0	0	0	0
0	0	0	0	0	0	0	0	0
1	0	0	0	0	0	0	0	0
0	0	0	0	0	0	0	1	0
0	0	0	0	0	0	0	0	0
1	0	0	0	0	0	0	0	0
0	0	0	0	0	0	0	0	0
0	0	0	0	0	0	0	1	0
0	0	0	0	0	0	0	0	0
0	0	0	0	0	0	0	0	0
0	0	0	0	0	0	1	0	0
1	0	0	1	0	0	0	0	0
0	0	0	0	0	0	0	0	0
0	0	0	0	0	0	0	0	0
0	0	0	0	0	0	0	0	0
0	1	0	0	0	0	0	0	0
0	1	0	0	0	0	0	0	0
0	0	1	0	0	0	0	0	0
0	0	0	0	0	0	1	0	0
0	0	0	0	0	0	0	0	0
0	0	0	0	0	0	0	1	0
0	0	0	0	0	0	0	0	0
0	0	0	0	0	0	0	0	0
0	0	0	0	0	1	0	0	0
0	1	0	0	0	0	0	0	0
0	0	0	0	0	0	0	0	0
0	0	0	0	0	0	0	0	0
0	0	0	0	0	0	0	0	0
0	0	0	0	0	0	0	0	0
0	0	0	0	1	0	0	0	0
1	0	0	0	0	0	0	1	0
0	0	0	0	0	0	0	0	0
0	0	0	0	0	0	0	0	0
0	0	0	0	0	0	0	0	0
0	0	0	0	0	0	0	0	0
0	0	0	0	0	0	1	0	0

0	0	0	0	0	0	0	0	0
1	1	0	0	1	0	0	0	0
0	0	0	0	0	0	0	0	0
1	0	0	0	0	0	0	0	0
0	0	0	0	0	0	0	0	0
0	0	0	0	0	0	0	0	0
0	0	0	1	0	0	0	0	0
0	1	0	0	0	0	0	0	0
0	0	1	0	0	0	0	0	0
0	0	0	0	0	0	0	0	0
1	0	0	0	0	0	0	0	0
1	0	0	0	1	0	0	0	0
0	0	0	0	0	0	0	0	0
0	0	0	0	0	0	0	0	0
0	0	0	0	0	0	0	0	0
0	0	0	0	0	0	0	1	0
0	0	0	0	0	0	0	0	0
0	0	0	0	0	0	0	0	0
0	0	0	0	0	0	0	0	0
1	0	0	0	0	0	0	0	0
0	0	1	0	0	0	0	0	0
1	0	0	0	0	0	0	0	0
0	0	0	0	0	0	0	1	0
0	0	0	0	0	0	0	0	0
0	0	0	0	0	0	0	0	0
0	0	0	0	0	0	0	0	0
0	0	0	0	0	0	0	0	0
1	0	0	0	1	0	0	0	0
0	0	0	0	0	0	0	0	0
0	0	0	0	0	0	0	1	0
0	0	0	0	0	0	0	0	0
0	0	0	0	0	0	1	0	0
0	0	1	0	0	0	0	1	0
0	0	0	0	0	0	0	0	0
0	0	0	0	0	0	0	0	0
0	0	0	0	1	0	0	0	0
0	0	0	0	0	0	0	0	0
0	0	0	0	0	0	0	0	0
0	0	0	0	0	0	0	1	0
0	0	0	0	0	0	0	0	0
0	0	1	0	0	0	0	0	0
0	0	0	0	0	0	0	0	0
0	0	0	0	0	0	0	0	0
0	0	0	0	0	0	0	0	0
0	0	0	0	0	0	0	0	0
0	0	0	0	0	0	0	0	0
0	0	0	0	0	0	0	1	0
0	0	0	1	0	0	0	0	0
0	0	0	0	0	0	0	1	1
0	1	0	0	0	0	0	0	0
1	0	1	0	0	0	0	0	0
0	0	0	0	0	0	0	0	0
0	0	0	0	0	0	0	0	0
1	0	0	0	0	0	0	0	0
0	0	0	0	0	0	1	0	0
0	0	0	0	0	0	0	0	0

0	0	0	0	0	0	0	0	0
0	0	0	0	0	0	0	0	0
0	0	0	0	0	0	0	0	0
1	0	0	0	0	0	0	0	0
0	0	0	0	0	0	0	0	0
0	0	0	0	0	0	0	1	0
1	0	0	0	0	0	1	0	0
0	0	0	0	0	0	0	0	0
1	1	0	0	0	0	0	0	0
0	1	0	0	0	0	0	1	0
1	0	1	0	0	0	0	0	0
1	0	0	0	0	0	0	0	0
0	0	0	0	0	0	0	0	0
0	0	0	0	0	0	0	0	0
0	0	0	0	0	0	0	0	0
0	0	0	0	0	0	0	0	0
1	0	0	0	0	0	0	0	0
0	0	0	0	0	0	0	0	0
0	0	0	0	0	0	0	0	0
0	0	0	0	0	0	0	0	0
0	0	0	0	0	0	0	0	0
0	0	0	0	0	0	0	0	0
0	0	0	0	0	0	0	0	0
0	0	0	0	0	0	0	0	0
0	0	0	0	0	0	0	0	0
0	0	0	0	0	0	0	0	0
0	0	0	0	0	0	0	0	0
0	0	0	0	0	0	1	0	0
0	0	0	0	0	0	0	0	0
0	0	0	1	0	0	0	0	0
1	0	0	0	0	0	0	0	0
0	0	0	0	0	0	0	0	0
0	0	0	0	0	0	0	0	0
0	0	0	0	0	0	0	0	0
0	1	0	0	0	0	0	0	0
1	0	0	0	0	0	0	1	0
0	0	0	0	0	0	0	0	0
0	1	0	0	0	0	0	0	0
0	0	0	0	0	0	1	0	0
1	0	0	0	0	0	0	0	0
0	0	0	0	0	0	0	0	0
0	0	0	0	0	0	0	0	0
0	0	0	0	0	0	0	0	0
0	0	0	0	0	0	0	0	0
0	0	0	0	0	0	0	1	0
0	0	0	0	0	0	0	0	0
0	0	0	0	0	0	0	1	0
1	0	0	0	0	0	0	0	0
0	0	0	0	0	0	0	0	0
0	0	0	0	0	0	0	0	0
0	0	0	0	0	0	0	0	0
1	0	0	0	0	0	0	0	0
0	0	0	0	0	0	0	0	0
1	0	0	1	0	0	0	0	0
0	0	0	0	0	0	0	0	0
0	0	0	0	0	0	0	0	0

0	0	0	0	0	0	0	0	0
0	0	0	0	0	0	0	0	0
0	0	0	0	0	0	0	0	0
0	1	0	0	0	0	0	0	0
0	0	0	0	0	0	0	0	0
0	0	0	0	0	0	0	0	0
0	0	0	0	0	0	0	0	0
1	0	0	0	0	0	0	0	0
0	0	0	0	0	0	0	0	0
0	0	0	0	0	0	0	0	0
1	0	0	1	0	0	0	0	0
1	0	0	0	0	0	0	0	0
1	0	0	0	0	0	0	0	0
1	0	0	0	0	0	0	0	0
1	0	0	0	0	0	0	0	0
0	1	0	0	0	0	0	0	0
0	0	0	0	0	0	0	0	0
0	0	0	0	0	0	0	0	0
1	0	0	0	0	0	0	0	0
0	0	0	0	0	0	0	0	0
0	0	0	0	0	0	0	0	0
0	0	0	0	0	0	1	0	0
0	0	0	0	0	0	0	0	0
0	0	0	0	0	0	0	0	0
0	0	0	1	0	0	0	0	0
0	0	0	0	0	0	0	0	0
0	0	0	0	0	0	0	0	0
0	0	0	1	0	0	0	1	0
0	0	0	0	0	0	0	0	0
1	0	0	0	0	0	0	0	0
0	1	0	0	0	0	0	0	0
0	0	0	0	0	0	0	0	0
0	0	0	0	0	0	0	0	0
0	1	0	0	0	0	0	0	0
0	0	0	0	0	0	0	1	0
0	0	0	0	0	0	0	0	0
0	0	0	0	1	0	0	0	0
0	0	0	0	1	0	0	0	0
0	0	0	0	0	0	0	0	0
1	0	0	0	0	0	0	0	0
0	0	0	0	0	0	0	0	0
0	0	0	0	0	0	0	0	0
1	0	0	0	1	0	0	1	0
0	0	0	0	0	0	0	0	0
0	0	0	0	0	0	0	0	0
1	0	0	0	0	0	0	0	0
0	0	0	0	0	0	0	0	0
0	0	0	0	0	0	0	0	0
0	0	0	0	0	0	0	0	0
0	0	0	0	0	0	0	1	0
0	0	0	1	0	0	0	0	0
1	0	0	0	0	0	0	0	0
0	0	0	0	0	0	0	0	0
0	0	0	0	0	0	0	0	0
1	0	0	0	0	0	0	0	0
0	0	0	0	0	0	0	1	0

0	0	0	0	0	0	0	0	0
1	0	0	0	0	0	0	0	0
0	0	0	0	0	0	0	0	0
0	0	0	0	0	0	0	0	0
0	0	0	0	0	0	0	0	0
0	0	0	0	0	0	0	0	0
1	0	0	0	0	0	0	0	1
0	1	0	0	0	0	0	0	0
0	0	0	0	0	0	0	0	0
0	0	0	0	0	0	0	0	0
0	0	0	0	0	0	0	0	0
0	0	0	0	0	0	0	0	0
0	1	0	0	0	0	0	0	0
0	0	0	0	0	0	0	0	0
0	0	0	0	0	0	0	0	0
1	0	0	0	0	0	0	0	0
0	0	0	0	0	0	0	0	0
0	0	0	0	0	0	0	0	0
0	0	0	0	0	0	0	0	0
0	0	0	0	0	0	0	1	0
0	0	0	0	0	0	0	0	0
0	0	0	0	0	0	0	0	0
0	0	0	0	1	0	0	0	0
0	0	0	0	0	0	0	0	0
0	0	0	0	0	0	0	0	0
0	0	0	0	0	0	0	0	0
0	0	0	0	0	0	0	0	0
0	0	0	0	0	0	0	0	0
0	0	0	0	0	0	0	0	0
0	0	0	0	0	0	0	0	0
0	0	0	0	0	0	0	0	0
0	0	0	0	0	0	0	0	0
0	0	0	0	0	0	0	0	0
0	0	0	0	0	0	0	0	0
0	0	0	0	0	0	1	0	0
0	0	0	0	0	0	0	0	0
0	1	0	0	0	0	0	0	0
1	0	0	0	0	0	0	0	0
0	0	0	0	0	0	0	0	0
0	0	0	0	0	0	0	0	0
0	0	0	0	0	0	0	0	0
0	0	0	0	0	0	0	0	0
0	0	0	0	0	0	0	0	0
0	0	0	0	0	0	0	0	0
0	0	0	0	0	0	0	0	0
0	0	0	0	0	0	0	0	0
1	0	0	0	1	0	0	0	0
0	1	0	0	0	0	0	0	0
0	0	0	0	0	0	0	0	0
0	0	0	0	0	0	0	0	0
0	0	0	0	0	0	0	0	0
1	0	0	0	0	0	0	0	0
0	0	0	1	0	0	0	1	0
0	0	0	0	0	0	0	0	0
1	0	0	0	0	0	0	0	0
1	0	0	0	0	0	0	0	0

0	0	0	0	0	0	0	0	0
0	0	0	0	0	0	0	0	0
1	0	0	0	0	0	0	0	0
0	0	0	0	0	0	0	0	0
0	0	0	0	0	0	0	0	0
0	0	0	0	0	0	0	0	0
0	0	0	0	0	0	0	0	0
0	0	0	0	0	0	0	0	0
0	0	0	0	1	0	0	0	0
0	0	0	0	0	0	0	0	0
1	0	0	0	0	0	0	0	0
1	0	0	0	0	0	0	0	0
0	0	0	1	0	0	0	1	0
0	0	0	0	0	0	0	0	0
0	0	0	0	0	0	0	0	0
0	0	0	0	0	0	0	1	0
0	0	0	0	0	0	0	0	0
0	0	0	0	0	0	0	1	0
0	0	0	0	0	0	0	1	0
1	0	0	0	0	0	0	0	0
0	0	0	0	0	0	0	0	0
0	0	0	0	0	1	0	0	0
1	0	0	0	0	0	0	0	0
0	0	0	0	0	0	0	0	0
1	0	0	0	0	0	0	0	0
0	0	0	0	0	0	0	0	0
0	0	0	0	0	0	0	0	0
0	0	0	0	0	0	0	0	0
0	0	0	0	0	0	0	0	0
0	0	0	0	0	0	0	0	0
0	0	0	0	0	0	0	0	0
0	0	0	0	0	0	0	0	0
0	0	0	0	0	0	0	0	0
1	0	0	0	0	0	0	0	0
0	0	0	0	0	0	0	1	0
0	1	0	0	0	0	0	0	0
0	0	0	1	0	0	0	0	0
0	0	0	0	0	0	1	0	0
0	0	0	0	0	0	0	0	0
0	0	0	0	0	0	0	0	0
0	0	0	0	0	0	0	0	0
0	0	0	0	0	0	0	0	0
0	0	0	0	0	0	0	0	0
0	0	0	0	0	0	0	0	0
0	0	0	0	1	0	0	0	0
0	0	0	0	0	0	0	0	0
0	0	0	0	0	0	0	0	0
1	0	0	0	0	0	0	0	0
0	0	0	0	0	0	0	0	0
0	0	0	1	0	0	0	0	1
0	0	0	0	0	0	0	0	0
1	0	0	0	0	0	0	1	0
0	0	0	0	0	0	0	0	0
0	1	0	0	0	0	0	0	1
0	0	0	0	0	0	0	0	0
0	0	0	0	0	0	0	0	0

1	0	0	0	0	0	0	0	0
0	0	0	0	1	0	0	0	0
0	0	0	0	0	0	0	0	0
0	0	0	0	0	0	0	0	0
0	0	0	0	0	0	0	0	0
0	0	0	0	0	0	0	0	0
0	0	0	0	0	0	0	0	0
0	0	0	0	0	0	0	0	0
0	0	0	0	0	0	0	0	0
1	0	0	0	1	0	0	0	0
1	1	0	0	0	0	0	0	0
0	0	0	0	0	0	0	0	0
0	0	0	0	0	0	0	0	0
0	0	0	0	0	0	0	0	0
0	0	0	0	0	0	0	0	0
1	0	0	0	0	0	1	0	0
0	0	0	0	0	0	0	0	0
0	0	0	0	0	0	0	0	0
0	0	0	0	0	0	0	0	0
0	0	0	0	0	0	0	0	0
1	0	0	0	0	0	0	0	0
0	0	0	0	0	0	0	0	0
0	0	0	0	0	0	0	0	0
0	0	0	0	0	0	0	0	0
0	0	0	0	0	0	0	0	0
0	0	0	0	0	0	0	0	0
0	0	0	0	0	0	1	0	0
0	0	0	0	0	0	1	0	0
0	0	0	0	0	0	0	0	0
0	0	0	0	0	0	0	0	0
0	0	0	0	0	0	0	1	0
0	0	0	0	0	0	0	0	0
0	0	0	0	0	0	0	0	0
0	0	0	0	0	0	0	1	0
0	0	0	0	0	0	0	0	0
1	0	0	0	0	0	0	0	0
0	1	0	0	0	0	0	0	0
0	0	0	0	0	0	0	1	0
0	0	0	0	0	0	0	0	0
0	0	0	0	0	0	0	0	0
0	0	0	0	0	0	0	0	0
0	0	0	0	0	0	0	0	0
0	0	0	0	0	0	0	0	0
0	0	0	0	0	0	0	0	0
1	0	0	0	0	0	0	0	0
1	0	0	0	0	0	0	1	0
0	0	0	0	0	0	0	0	0
0	0	0	0	0	0	0	0	0
1	0	0	0	0	0	0	0	0
0	0	0	0	0	0	0	0	0
0	0	0	0	0	0	0	0	0
0	0	0	0	0	0	0	0	0
1	0	0	0	0	0	0	0	0
0	0	0	0	0	0	0	0	0
1	0	0	0	0	0	0	0	0
0	0	0	0	0	0	0	0	0

0	1	0	0	0	0	0	0	0
0	0	0	0	0	0	0	0	0
1	0	1	0	0	0	0	0	0
0	0	0	0	0	0	0	0	0
0	0	0	0	1	0	0	0	0
0	0	0	0	0	0	0	0	0
1	0	0	0	0	0	0	0	0
0	0	0	0	0	0	0	0	0
0	0	0	0	0	0	0	0	0
0	0	0	0	0	0	0	0	0
0	0	0	0	0	0	0	0	0
0	0	0	0	1	0	0	0	0
0	0	0	0	0	0	0	0	0
0	0	0	0	0	0	0	0	0
0	0	0	0	1	0	0	0	0
0	0	0	0	0	0	0	0	1
1	0	0	0	0	0	0	0	0
0	0	0	0	0	0	1	0	0
0	0	0	0	0	0	0	0	0
0	0	0	0	0	0	0	0	0
0	0	0	0	0	0	0	0	1
0	0	0	0	0	0	0	0	0
0	0	0	0	0	0	0	0	0
0	1	0	0	0	0	0	0	0
1	0	0	0	0	0	0	1	0
0	0	0	0	0	0	0	0	0
0	0	0	0	0	0	0	0	0
0	0	0	0	0	0	0	0	0
0	0	0	0	0	0	0	0	0
0	0	0	0	0	0	0	0	0
0	0	0	0	0	0	0	0	0
0	0	0	0	0	0	0	0	0
1	0	1	0	0	0	0	0	0
0	0	0	1	0	0	0	1	0
1	0	0	0	0	0	0	1	0
0	0	0	0	0	0	0	0	0
0	0	0	0	0	0	0	0	0
0	0	0	0	0	0	0	0	0
0	1	0	0	0	0	0	0	0
0	0	0	0	0	0	0	0	0
0	0	0	0	0	0	0	0	0
1	0	0	0	0	0	0	0	0
0	0	0	0	0	0	0	0	0
0	0	0	0	0	0	0	0	0
0	0	0	0	0	0	0	0	0
1	0	0	0	1	0	0	0	0
0	0	0	0	0	0	0	1	0
0	0	0	0	0	0	0	0	0
0	0	0	0	0	0	0	0	0
0	0	0	0	0	0	0	0	0
0	0	0	0	0	0	0	0	0
0	0	0	0	0	0	0	0	0
1	0	0	0	0	0	0	0	0
0	1	0	0	0	0	0	0	0
1	0	0	0	0	0	0	0	0
0	0	0	0	0	0	0	0	0

0	0	0	0	0	0	0	0	0
1	0	0	0	0	0	0	0	0
1	0	1	0	0	0	0	0	0
0	0	0	0	0	0	0	0	0
0	0	0	0	0	0	0	0	0
0	0	0	0	0	0	0	0	0
1	0	0	0	0	0	0	0	0
0	0	0	0	0	0	0	0	0
0	0	0	0	0	0	0	0	0
0	0	0	0	0	0	0	0	0
0	0	0	0	0	0	0	1	0
0	0	0	0	0	0	0	0	0
0	0	0	0	0	0	0	0	0
1	0	1	0	0	0	0	0	0
0	1	0	0	1	0	0	0	0
1	0	0	0	0	0	0	0	0
0	0	0	0	0	0	0	0	0
0	0	0	0	0	0	0	0	0
0	0	0	0	0	0	0	0	0
0	0	0	0	0	0	0	0	0
0	0	0	0	0	0	0	0	0
0	0	0	0	0	0	0	0	0
0	0	0	1	0	0	0	0	0
0	0	0	0	0	0	0	0	0
0	0	0	0	0	0	0	0	0
0	1	0	0	0	0	0	0	0
0	0	0	0	0	0	0	0	0
1	0	0	0	0	0	0	0	0
0	0	0	0	0	0	0	0	0
0	0	0	0	0	0	0	0	0
0	0	0	0	0	0	0	0	0
1	0	0	0	0	0	0	0	0
0	0	0	0	0	0	0	0	0
0	0	0	0	0	0	0	0	0
0	0	0	0	0	0	0	0	0
0	0	0	0	0	0	0	1	0
0	0	0	0	0	0	0	0	0
0	0	0	0	0	0	0	0	0
0	0	0	0	0	0	0	0	0
0	0	0	0	0	0	0	0	0
0	0	0	0	0	0	0	0	0
0	0	1	0	0	0	0	0	0
0	1	0	0	0	0	0	0	0
0	0	0	0	0	0	0	0	0
1	0	0	0	0	0	0	0	0
0	1	0	0	0	0	0	0	0
0	0	0	0	0	0	0	0	0
0	0	0	0	0	0	0	0	0
0	1	0	0	0	0	0	0	0
0	1	0	0	0	0	0	0	0
0	0	1	0	0	0	0	1	0
0	1	0	0	0	0	0	0	0
0	0	0	0	0	0	0	0	0
0	0	0	0	0	0	0	1	0
0	0	0	0	0	0	0	0	0
0	0	0	0	0	0	0	0	0

0	0	0	0	0	0	0	0	0
0	0	0	0	0	0	1	0	0
1	0	0	0	0	0	1	0	0
0	0	0	0	0	0	0	0	0
0	0	0	0	0	0	0	0	0
1	0	0	0	0	0	0	0	0
0	0	0	0	0	0	0	0	0
0	0	0	0	0	0	0	0	0
0	0	0	0	0	0	0	0	0
0	0	0	0	0	0	0	0	0
0	0	0	0	0	0	0	0	0
0	0	0	0	0	0	0	0	0
1	0	0	0	0	0	0	1	0
1	0	0	0	0	0	0	0	0
1	0	0	0	0	0	0	0	0
0	0	0	0	0	0	0	0	1
0	0	0	0	0	0	0	0	0
1	0	0	0	0	0	0	0	0
0	0	0	0	0	0	0	0	0
0	0	0	0	0	0	0	1	0
0	0	0	0	1	0	0	0	0
0	0	0	0	0	0	0	0	0
0	0	0	0	0	0	0	0	0
1	0	0	0	0	0	0	0	0
0	0	0	0	0	0	0	0	0
1	0	0	0	0	0	0	0	0
0	0	0	0	0	0	0	0	0
1	0	0	0	0	0	0	0	0
0	0	0	0	0	0	0	0	0
0	0	0	0	0	0	0	0	0
0	0	0	0	0	0	0	0	0
0	0	0	0	0	0	0	0	0
0	0	0	0	0	0	0	0	0
0	0	0	0	0	0	0	1	0
0	0	0	0	0	0	0	0	0
0	0	0	0	0	0	0	0	0
0	0	0	0	0	0	0	0	0
0	0	0	0	0	0	0	0	0
0	0	0	0	0	0	0	0	0
0	0	0	0	0	0	0	0	0
0	0	0	0	0	0	0	1	0
0	0	0	0	0	0	0	1	0
0	0	0	0	0	0	0	0	0
0	0	0	0	0	0	0	0	0
1	0	0	0	0	0	0	0	0
0	0	0	0	0	0	0	0	0
0	0	0	0	0	0	0	0	0
0	0	0	0	0	0	0	0	0
0	1	0	0	0	0	0	0	0
0	0	0	0	0	0	0	0	0
1	0	1	0	0	0	0	0	0
0	0	0	0	0	0	0	0	0
0	0	0	0	0	0	0	0	0
0	0	0	0	0	0	0	0	0
0	0	0	0	0	0	0	1	0
0	0	0	0	1	0	0	0	0

0	0	0	0	0	0	0	0	0
0	0	0	0	0	0	0	0	0
0	0	0	0	0	0	0	0	0
1	0	0	0	0	0	0	0	0
0	0	0	0	0	0	0	0	0
1	0	0	0	0	0	0	0	0
0	0	0	0	0	0	0	0	0
0	0	0	0	0	0	0	0	0
1	0	0	0	0	0	0	0	0
0	0	0	0	0	0	0	0	0
0	0	0	0	0	0	0	0	0
0	0	0	0	0	0	0	0	0
0	0	0	0	0	0	0	0	0
1	0	0	0	0	0	0	0	0
1	0	0	0	0	0	0	0	0
0	0	1	0	0	0	0	0	0
0	0	0	0	0	0	0	0	0
0	0	0	0	0	0	1	0	0
0	0	0	0	0	0	0	1	0
0	0	0	0	0	0	0	0	0
0	0	0	0	0	0	0	0	0
0	0	0	0	0	0	0	0	0
0	0	0	0	0	0	0	0	0
1	1	0	0	0	0	0	0	0
1	0	1	0	0	0	0	0	0
0	0	0	0	0	0	0	0	1
1	0	0	0	0	0	0	0	0
0	0	0	0	0	0	0	0	0
0	0	0	0	0	0	0	0	0
0	0	0	0	0	0	0	0	0
1	0	0	1	0	0	0	0	0
0	0	0	0	0	0	0	0	0
1	0	0	0	0	0	0	0	0
1	0	0	0	0	0	0	0	0
0	0	0	0	0	0	0	0	0
0	0	0	0	0	0	0	0	0
1	0	0	0	0	0	0	0	0
0	0	0	0	0	0	0	0	0
1	1	0	0	0	0	0	0	0
0	0	0	0	0	0	0	0	0
0	0	0	0	0	0	0	0	0
0	0	0	0	0	0	0	0	0
0	0	0	0	0	0	0	0	0
0	0	0	0	0	0	0	0	0
0	0	0	0	0	0	0	1	0
0	0	0	0	0	0	0	1	0
0	0	0	0	0	0	0	0	1
0	0	0	0	0	0	0	0	0
0	0	0	0	0	0	0	0	0
1	0	0	0	0	0	0	0	0
0	0	0	0	0	0	0	0	0
0	0	0	0	0	0	0	0	0
0	0	0	0	0	0	0	0	0
0	0	0	0	0	0	0	0	0
0	0	0	0	0	0	0	0	0
0	0	0	0	0	0	0	0	0

1	0	0	0	0	0	0	0	0
1	0	1	0	0	0	0	0	0
0	0	0	0	0	0	0	0	0
1	0	0	0	1	0	0	0	0
0	0	0	0	0	0	0	0	0
0	0	0	0	0	0	0	0	0
1	0	0	0	1	0	0	1	0
1	0	0	0	0	0	0	0	0
1	0	0	0	0	0	0	0	0
0	0	0	0	0	0	0	0	0
0	0	0	0	0	0	0	0	0
1	0	0	0	0	0	0	0	0
0	1	0	0	0	0	0	1	0
0	0	0	0	0	0	1	0	0
0	0	0	0	0	0	0	0	0
0	0	0	0	0	0	0	0	0
0	0	0	0	0	0	0	0	0
0	0	0	0	0	0	0	1	0
0	0	0	0	0	0	0	0	0
0	0	0	0	0	0	0	0	0
0	0	0	0	0	0	0	0	0
1	0	0	0	0	0	0	0	0
0	0	0	0	0	0	0	0	0
0	1	0	0	0	0	0	0	0
0	0	0	0	0	0	0	0	0
0	0	0	0	0	0	0	0	0
1	0	0	0	0	0	0	0	0
1	0	0	0	0	0	0	0	0
0	0	1	0	0	0	0	0	0
0	0	0	0	0	0	0	0	0
0	0	0	0	0	0	0	0	0
1	0	0	0	0	0	0	0	0
0	0	0	0	0	0	0	0	0
0	0	0	0	0	0	0	1	0
0	0	0	0	0	0	0	0	0
0	0	0	0	0	0	0	0	0
0	0	0	0	0	0	0	0	0
1	0	0	0	0	0	0	0	0
0	0	0	0	0	0	0	0	0
0	0	0	0	0	0	0	0	0
1	0	0	0	0	0	0	0	0
0	0	0	0	0	0	0	1	0
0	0	0	0	0	0	0	0	0
0	0	0	0	0	0	0	0	0
0	0	0	0	0	0	0	0	0
0	0	0	0	0	0	0	0	0
1	0	0	0	0	0	0	0	0
0	0	0	0	0	0	0	0	0
0	0	0	0	0	0	0	0	0
0	0	0	0	0	0	0	0	0
0	0	0	0	0	0	0	0	0
0	0	0	0	0	0	0	0	0
1	0	0	0	0	0	0	0	0
0	0	0	0	0	0	0	0	0
1	0	0	0	0	0	0	0	0
0	0	0	1	0	0	0	0	0

0	0	0	0	0	0	0	0	0
0	0	0	0	0	0	0	0	0
0	0	0	0	0	0	0	0	0
1	0	0	0	0	0	0	0	0
0	0	0	0	0	0	0	0	0
0	0	0	0	0	0	0	0	0
0	0	0	0	0	0	0	0	0
1	0	0	0	0	0	0	0	0
0	0	0	0	0	0	0	0	0
0	0	0	0	0	0	0	0	0
0	0	0	0	0	0	0	0	0
0	0	0	0	0	0	1	0	0
0	0	0	0	0	0	0	0	0
0	0	0	0	0	0	0	0	0
0	0	0	0	0	0	0	0	0
1	0	0	0	0	0	0	1	0
1	0	0	1	0	0	0	0	0
0	0	0	0	0	0	0	0	0
0	0	0	0	0	0	0	0	0
0	0	0	0	0	0	0	0	0
0	0	0	0	0	0	0	0	0
0	0	0	0	0	0	0	0	0
0	0	0	0	0	0	0	0	0
0	0	0	0	0	0	0	0	0
1	0	0	0	0	0	0	0	0
0	1	0	0	0	0	0	0	0
0	0	0	0	0	0	0	0	0
0	0	0	0	0	0	0	0	0
0	0	0	0	0	0	0	0	0
0	0	0	0	0	0	0	0	0
0	0	1	0	0	0	0	0	0
0	0	0	0	0	0	0	0	0
0	0	0	0	0	0	0	1	0
0	0	0	0	0	0	0	0	0
0	0	0	0	0	0	0	0	0
0	0	0	1	0	0	0	0	0
0	0	0	0	0	1	0	0	0
0	0	0	0	0	0	0	0	0
0	0	0	0	0	0	0	0	0
0	0	0	0	0	0	0	0	0
0	0	0	0	0	0	0	0	0
1	0	0	0	1	0	0	0	0
0	0	0	0	0	0	0	1	1
1	0	0	0	0	0	0	0	0
0	0	0	0	0	0	0	0	0
0	0	0	0	0	0	0	0	0
1	0	0	0	0	0	0	0	0
0	0	0	0	0	0	0	0	0
0	0	0	0	0	0	0	0	0
0	1	1	0	0	0	0	0	0
0	0	0	0	0	0	0	0	0
0	0	0	0	0	0	0	0	0
0	0	0	0	1	1	0	0	0
0	1	0	0	0	0	0	0	0
1	0	0	0	0	0	1	0	0
0	0	0	0	0	0	0	0	0

1	0	1	0	0	0	0	1	0
0	0	0	0	0	0	0	0	0
1	0	0	0	0	0	0	0	0
0	0	0	1	0	0	0	0	0
0	0	0	0	0	0	0	0	0
0	0	0	0	0	0	0	0	0
0	0	0	0	0	0	0	0	0
1	0	0	0	0	0	0	0	0
1	0	0	0	0	0	0	0	0
0	0	0	0	0	0	0	0	0
0	0	0	0	0	0	0	0	1
1	0	0	0	0	0	0	0	0
1	0	0	0	0	0	0	0	0
0	0	0	0	0	0	0	0	0
1	0	0	0	0	0	0	0	0
1	0	0	0	0	0	0	0	0
0	0	0	0	0	0	0	1	0
0	1	0	0	0	0	0	0	1
0	0	0	0	0	0	0	0	0
0	0	0	1	0	0	0	0	0
0	0	0	0	0	0	0	0	0
0	1	0	0	0	0	0	0	0
0	0	0	0	0	0	0	0	0
0	0	0	0	0	0	0	0	0
0	0	0	0	0	0	0	0	0
0	0	0	0	0	0	0	0	0
0	0	1	0	0	0	0	0	0
1	0	0	0	0	0	0	0	0
1	0	0	0	0	0	0	0	0
0	0	0	0	0	0	0	0	0
0	0	0	0	0	0	0	1	0
0	0	0	0	0	0	0	0	0
1	0	0	0	0	0	0	0	0
0	0	0	0	0	0	0	0	0
0	0	0	0	0	0	0	0	0
0	0	0	0	0	0	0	0	0
0	0	0	0	0	0	0	0	0
1	0	0	0	0	0	0	0	0
0	0	0	0	0	0	0	1	0
0	0	0	0	0	0	0	0	0
0	0	0	0	0	0	0	0	0
0	0	0	0	0	0	0	0	0
0	0	0	0	0	0	0	0	0
1	0	0	0	0	0	0	0	0
1	0	0	0	0	0	0	0	0
0	0	0	0	0	0	0	0	0
0	0	0	0	0	0	0	0	0
0	1	0	0	0	0	0	0	0
0	0	0	0	0	0	0	0	0
0	0	0	0	0	0	0	0	0
0	0	0	0	0	0	0	0	0
0	0	0	0	0	0	0	0	0
1	0	0	0	0	0	0	0	0
0	0	0	0	0	0	0	0	0
0	0	0	0	0	0	0	0	0
0	0	0	0	0	0	0	0	0

0	0	0	0	0	0	0	0	0
0	0	1	0	0	0	0	0	0
1	0	0	0	0	0	0	0	0
0	0	0	0	0	0	0	0	0
0	0	0	0	0	0	0	0	0
0	0	0	0	0	0	0	0	0
0	0	0	0	0	0	0	0	0
1	0	0	0	0	0	0	0	0
0	0	0	0	0	0	0	0	0
0	1	0	0	0	0	0	0	0
1	0	0	0	0	0	0	0	0
0	0	0	0	0	0	0	1	1
0	0	0	0	0	0	0	0	0
0	1	0	0	0	0	0	0	0
1	0	0	0	0	0	0	0	0
0	0	0	0	0	0	0	0	0
1	0	0	0	0	0	0	0	0
0	0	0	0	0	0	0	0	0
0	0	0	0	0	0	0	0	0
0	1	0	0	0	0	0	0	0
1	0	0	0	0	1	0	0	0
0	0	1	0	0	0	0	0	0
0	0	0	0	0	0	0	0	0
0	0	0	0	0	0	0	0	0
0	0	0	0	0	0	0	0	0
0	0	0	0	0	0	0	0	0
0	0	0	0	0	0	0	0	0
0	0	0	0	0	0	0	0	0
1	0	0	0	0	0	0	0	0
0	0	0	0	0	0	0	0	0
0	0	0	0	0	0	0	0	0
0	0	0	0	0	0	0	0	0
1	1	0	0	0	0	0	0	0
0	0	0	0	0	0	0	0	0
0	0	0	0	0	0	0	0	0
0	0	0	0	0	0	0	0	0
0	0	0	0	0	0	0	0	0
0	0	0	0	1	0	0	0	0
1	0	0	0	0	0	0	0	0
1	0	0	0	0	0	0	0	0
0	0	0	0	0	0	0	0	0
1	0	0	0	0	0	0	0	0
0	0	0	0	0	0	0	0	1
1	0	0	0	0	0	0	1	0
0	0	0	0	0	0	0	1	0
0	0	0	1	0	0	0	0	0
0	0	0	0	0	0	0	0	0
0	0	0	0	0	0	0	0	0
0	1	0	0	0	0	0	0	0
0	0	0	0	0	0	0	0	0
0	0	0	0	0	0	0	0	0
0	0	0	0	0	0	1	0	0
0	0	0	0	0	0	0	1	0
1	0	0	0	0	0	0	0	0
0	0	0	0	0	0	0	0	0
1	0	0	0	0	0	0	0	0

0	0	0	0	0	0	0	0	0
0	0	0	0	0	0	0	0	0
0	0	0	0	1	0	0	0	0
0	0	0	0	0	0	0	0	0
0	0	0	0	0	0	0	0	0
0	0	0	0	0	0	0	1	0
1	0	0	0	0	0	0	0	0
0	0	0	0	0	0	0	0	0
0	0	0	0	0	0	0	0	0
0	0	0	0	0	0	0	0	0
0	0	0	0	0	0	0	0	0
0	0	0	0	0	0	0	0	0
1	0	0	0	0	0	0	0	0
0	0	0	0	0	0	0	0	0
0	0	0	0	0	0	0	0	0
0	0	0	1	0	0	0	0	0
0	0	0	0	0	0	0	0	0
0	1	0	0	0	0	0	0	0
1	0	0	0	0	0	0	0	0
0	0	0	0	0	0	0	0	0
0	0	0	0	0	0	0	0	0
0	0	0	0	0	0	0	0	0
0	0	0	0	0	0	0	0	0
1	1	0	0	0	0	0	0	0
0	0	0	0	0	0	0	0	0
0	0	0	0	0	0	0	0	0
1	0	0	0	0	0	0	1	0
0	1	0	1	0	0	0	0	0
1	0	0	0	0	0	0	0	0
0	0	0	0	0	0	0	0	0
0	0	0	0	0	0	0	1	0
0	0	0	0	0	0	0	0	0
0	0	0	0	0	0	0	0	0
0	0	0	0	0	0	0	0	0
0	0	0	0	0	0	0	0	0
1	0	0	0	0	0	0	0	0
0	0	0	0	0	0	0	0	0
0	0	0	0	0	0	0	0	0
0	0	0	0	0	0	0	0	0
0	1	0	0	0	0	0	0	0
0	0	0	0	0	0	0	0	0
0	0	0	0	0	0	0	0	0
0	0	0	0	0	0	0	0	0
1	0	0	0	0	0	0	0	0
0	0	0	0	0	0	0	0	0
1	0	0	0	0	0	0	0	0
0	0	0	0	0	0	0	0	0
0	0	0	0	0	0	0	0	0
0	0	0	0	0	0	0	0	0
1	1	0	0	0	0	0	0	0
0	0	0	0	0	0	0	0	0
0	1	0	0	0	0	0	0	0
1	0	0	0	0	0	0	0	0
0	0	0	0	0	0	0	0	0
0	0	0	0	0	0	0	0	0
0	0	0	0	0	0	0	0	0

0	0	0	0	0	0	0	0	0
0	0	0	0	0	0	0	1	0
0	0	0	0	0	0	0	0	0
1	0	1	0	0	0	0	0	0
0	0	0	0	0	0	0	1	0
0	0	0	0	0	0	0	0	0
0	0	0	0	0	0	0	0	0
0	0	0	0	0	0	0	0	0
0	0	0	0	0	0	0	0	0
1	0	0	0	0	0	0	0	0
1	0	0	0	0	0	0	0	0
0	0	0	0	0	0	0	0	0
0	0	0	0	0	0	0	0	0
0	0	0	0	0	0	0	0	0
0	0	0	0	0	0	0	0	0
0	0	0	0	0	0	0	0	0
0	0	0	0	0	0	0	0	0
0	0	0	0	0	0	0	0	0
0	0	0	0	0	0	0	0	0
0	0	0	0	0	0	0	0	0
0	1	0	0	0	0	0	0	0
0	1	0	0	0	0	0	0	0
1	0	0	0	0	0	0	0	0
0	0	0	0	0	0	0	0	0
0	0	0	0	0	0	0	0	0
0	0	0	0	0	0	0	0	0
0	0	0	0	0	0	0	0	0
1	0	0	0	0	0	0	0	0
0	0	0	0	0	0	0	1	0
0	0	0	0	0	0	0	1	0
1	0	0	0	0	0	0	0	0
0	1	0	1	0	0	0	0	0
1	0	0	0	0	0	1	0	0
0	0	0	0	0	0	0	0	0
0	0	0	0	0	0	0	0	0
0	0	0	0	0	0	0	0	0
0	0	0	0	0	0	0	0	0
1	0	0	0	0	0	0	0	0
0	0	0	0	0	0	0	0	0
0	0	0	0	0	0	0	0	0
0	0	0	0	0	0	0	0	0
0	0	0	0	0	0	0	0	0
1	0	0	0	0	0	0	0	0
0	1	0	0	0	0	0	0	0
0	0	0	0	0	0	0	0	0
0	0	1	0	0	0	0	1	0
0	0	0	1	0	0	0	1	0
0	0	0	0	0	0	0	1	0
0	0	0	0	0	0	0	0	0
0	0	0	1	0	0	0	0	0
0	0	0	0	0	0	0	0	0
0	0	0	0	0	0	0	0	0
0	0	0	0	0	0	0	0	0
0	0	0	0	0	0	0	0	0
0	0	0	0	0	0	0	0	0
0	0	0	0	0	0	0	1	0

0	0	0	0	0	0	0	0	0
0	0	0	0	0	0	0	0	0
0	0	0	0	0	0	0	0	0
1	0	0	1	0	0	0	1	0
0	0	0	0	0	0	0	0	0
0	1	0	0	0	0	0	0	0
0	0	0	0	0	0	0	0	0
0	0	0	0	0	0	0	0	0
0	0	0	0	0	0	0	0	0
0	0	0	0	0	0	0	0	0
0	0	0	0	0	0	0	1	0
0	0	0	0	0	0	0	0	0
0	0	0	0	0	0	1	0	0
0	0	0	0	0	0	0	0	0
0	0	0	0	0	0	0	0	0
1	0	0	0	0	0	0	0	0
1	0	0	0	0	0	0	0	0
0	0	0	0	0	0	0	0	0
1	0	0	0	0	0	0	0	0
0	0	0	0	0	0	0	0	0
1	0	0	0	1	0	0	0	0
0	0	0	0	1	0	0	0	0
0	0	0	0	0	0	0	0	0
0	0	0	0	0	0	0	0	0
0	0	0	0	0	0	0	1	0
0	0	0	0	0	0	0	0	0
0	1	0	0	0	0	0	0	0
0	0	0	0	0	0	0	0	0
0	1	1	0	0	0	0	0	0
1	0	0	0	0	0	0	0	0
0	0	0	0	0	0	0	0	0
0	0	0	0	0	0	0	0	0
0	0	0	0	0	0	0	0	0
0	0	0	0	0	0	0	0	0
0	0	0	0	0	0	0	0	0
1	0	0	0	0	0	0	0	0
0	0	0	0	0	0	1	0	0
0	0	0	0	0	0	0	0	0
0	0	0	0	0	0	0	0	0
0	0	0	0	0	0	0	0	0
0	0	0	0	0	0	0	0	0
1	0	0	0	0	0	0	0	0
0	0	0	0	0	0	0	0	0
0	0	1	0	0	0	0	0	0
0	0	0	0	0	0	0	0	0
0	0	0	0	0	0	0	0	0
0	0	0	0	0	0	0	0	0
0	0	0	1	0	0	0	1	0
0	0	0	0	0	0	0	0	0
0	0	0	1	0	0	0	0	0
1	0	0	0	0	0	0	0	0
0	1	0	0	0	0	0	0	0
0	0	0	0	0	0	0	0	0
1	0	0	0	0	0	1	0	0
0	0	0	0	0	0	0	1	0
0	1	0	0	0	0	0	0	0

0	0	0	0	0	0	0	0	0
1	0	0	0	0	0	0	0	0
0	0	0	0	0	0	0	1	0
0	0	0	0	0	0	0	0	0
0	0	0	0	0	0	0	0	0
1	0	0	0	0	0	0	0	0
0	0	0	0	0	0	0	0	0
0	0	0	0	1	0	0	0	0
0	0	0	0	0	0	0	0	0
0	0	0	0	0	0	0	0	0
0	0	0	0	0	0	0	0	0
0	0	0	0	0	0	0	0	0
0	0	0	0	0	0	0	0	0
0	0	0	0	0	0	0	0	0
0	0	0	0	0	0	0	0	0
1	0	0	0	0	0	0	0	0
0	0	0	0	0	0	0	0	0
0	0	0	0	0	0	0	0	0
0	0	0	0	0	0	0	0	0
1	0	0	0	0	0	0	0	0
0	0	0	0	0	0	0	0	0
1	0	0	0	0	0	0	0	0
0	0	0	0	0	0	0	0	0
0	0	0	0	0	0	0	0	0
0	1	0	0	0	0	0	0	0
0	0	0	0	0	0	0	0	0
0	0	0	0	0	0	1	0	0
0	0	0	0	0	0	0	1	0
1	0	1	0	0	0	0	0	0
0	0	0	0	1	0	0	0	0
1	0	0	0	0	0	0	0	0
0	0	0	0	0	0	0	0	0
0	0	0	0	0	0	0	0	0
0	0	0	0	0	0	0	0	0
0	0	0	0	0	0	0	0	0
0	0	0	0	0	0	0	0	0
0	0	0	0	0	0	0	1	0
0	0	0	0	1	0	0	0	0
0	0	0	0	0	0	0	0	0
0	0	0	0	0	0	0	0	0
0	0	0	0	0	0	0	0	0
1	0	0	0	0	0	0	0	0
0	0	0	0	0	0	0	0	0
0	0	0	0	0	0	0	0	0
0	0	0	0	0	0	0	0	0
0	0	0	0	0	0	0	0	0
0	0	0	0	0	0	0	0	0
0	0	0	0	0	0	1	0	0
0	0	0	0	0	0	0	0	0
0	0	0	0	0	0	0	0	0
0	0	0	0	0	0	0	0	0
0	0	0	0	0	0	0	0	0
0	0	0	0	0	0	0	0	0
0	0	0	0	0	0	0	0	0
0	1	0	0	0	0	0	0	0
0	0	0	0	0	0	0	0	0

0	0	0	0	0	0	0	0	0
0	0	0	0	0	0	0	0	0
0	0	0	0	0	0	0	1	0
0	1	0	0	0	0	0	1	0
0	0	0	0	0	0	0	0	0
0	0	0	0	0	0	0	0	0
0	1	0	0	0	0	0	0	0
0	0	0	0	0	0	0	0	0
0	0	0	0	0	0	0	0	0
0	0	0	0	0	0	0	0	0
0	0	0	0	0	0	0	0	0
1	0	0	0	0	0	0	0	0
0	0	0	0	0	0	0	0	0
0	0	0	0	0	0	0	0	0
0	0	0	0	0	0	0	0	0
0	0	0	0	0	0	0	0	0
0	0	0	0	0	0	0	0	0
0	0	0	0	0	0	0	1	0
0	0	0	0	0	0	0	0	0
0	0	0	0	0	0	0	0	0
0	0	0	0	0	0	0	0	0
0	0	0	0	0	0	0	0	0
0	0	0	0	0	0	0	0	0
0	0	0	0	0	0	0	0	0
0	0	0	0	0	0	0	0	0
0	0	0	0	0	0	0	0	0
0	0	0	0	0	0	0	0	0
0	0	0	0	0	0	0	0	0
1	0	0	0	0	0	0	1	0
1	0	0	0	0	0	0	1	0
0	0	0	0	0	0	0	0	0
0	0	0	0	0	0	0	0	0
0	0	0	0	0	0	0	0	0
0	0	0	0	0	0	0	0	0
0	0	0	0	0	0	0	0	0
1	0	0	0	1	1	0	0	0
0	0	0	0	0	0	0	0	0
1	0	0	0	0	0	0	0	0
1	0	0	0	0	0	0	0	0
0	0	0	0	0	0	0	0	0
0	0	0	0	0	0	0	0	0
0	0	0	0	0	0	0	0	0
0	0	0	0	0	0	0	0	0
0	0	0	0	0	0	0	0	0
0	0	0	0	0	0	0	0	0
1	0	0	0	0	0	0	1	0
0	0	0	0	0	0	0	0	0
0	0	0	0	0	0	0	0	0
0	0	0	0	0	0	0	0	0
0	1	0	0	0	0	0	1	0
1	0	0	0	0	0	0	0	0
1	0	0	0	0	0	0	0	0
0	0	1	0	0	0	0	0	0
0	0	0	0	0	0	0	0	0
0	0	0	0	0	0	0	0	0
0	0	0	0	0	0	0	0	0

0	0	0	1	0	0	0	0	0
0	0	0	0	0	0	0	0	0
0	0	0	0	0	0	0	0	0
0	0	0	0	0	0	0	0	0
0	0	0	0	0	0	0	0	0
0	0	0	0	0	0	0	0	0
0	0	0	0	0	0	0	0	0
0	0	0	0	0	0	1	0	0
1	0	0	0	0	0	0	0	0
0	0	0	0	0	0	0	1	0
0	0	0	0	0	0	0	0	0
0	0	0	0	0	0	0	0	0
0	0	0	0	0	0	0	0	0
0	0	0	0	0	0	0	0	0
0	1	0	0	0	0	0	0	0
0	0	0	0	0	0	0	1	0
1	0	0	0	0	0	0	0	0
0	0	0	0	0	0	0	0	0
0	0	0	0	0	0	0	0	0
0	0	0	0	0	0	0	1	0
0	0	0	1	0	0	0	0	0
1	0	0	0	0	0	0	1	0
0	0	0	0	0	0	0	0	0
1	0	0	0	0	0	0	1	0
0	0	0	0	0	0	0	0	0
0	0	0	0	0	0	0	0	0
0	0	0	0	0	0	0	0	0
0	0	0	0	0	0	0	0	0
0	0	0	0	0	0	0	0	0
0	0	0	0	0	0	0	0	0
1	1	0	0	0	0	0	0	0
0	0	0	0	0	0	0	0	0
0	0	0	0	0	0	0	0	0
0	0	0	0	0	0	0	1	0
0	1	0	0	0	0	0	0	0
0	0	0	0	0	0	0	0	0
0	1	0	0	0	0	0	0	0
0	0	0	0	0	0	0	0	0
0	0	0	0	0	0	0	0	0
0	0	0	0	0	0	0	0	0
0	0	0	0	0	0	0	0	0
1	0	0	0	0	0	0	0	0
0	0	0	0	0	0	1	0	0
1	0	0	0	1	0	0	0	0
1	0	0	0	0	0	0	0	0
0	0	0	0	0	0	0	0	0
0	0	0	0	0	1	0	0	0
0	0	0	1	0	0	0	0	0
0	0	0	0	0	0	0	0	0
0	0	0	0	0	0	0	0	0
0	1	0	0	0	0	0	0	0
0	0	0	0	0	0	0	0	0
0	0	0	0	0	0	0	0	0
0	0	0	0	0	0	0	0	0
0	0	0	0	0	0	0	0	0
0	0	0	0	0	0	0	0	0

0	0	0	0	0	0	0	0	0
0	0	0	0	0	0	0	0	0
1	0	0	0	0	0	0	0	0
0	0	0	0	0	0	0	0	0
1	0	0	0	0	0	0	0	0
1	0	0	0	0	0	0	0	0
1	0	0	0	0	0	0	1	0
0	0	0	0	0	0	0	0	0
0	0	0	0	0	0	0	0	0
0	0	0	0	0	0	0	0	0
0	0	0	0	0	0	0	0	0
0	0	0	0	0	0	0	1	0
0	0	0	0	0	0	0	0	0
0	0	0	0	0	0	0	0	0
0	0	0	0	0	0	0	0	0
0	0	0	0	0	0	0	0	0
0	0	0	0	0	0	0	0	0
0	0	0	0	0	0	0	0	0
0	0	0	0	0	0	1	0	0
0	0	0	0	0	0	0	0	0
0	0	0	0	0	0	0	0	0
0	0	0	0	0	0	0	0	0
0	0	0	0	0	0	0	0	0
0	0	0	0	0	0	0	0	0
0	0	0	0	0	0	0	1	0
0	0	0	0	0	0	0	0	0
1	0	0	0	0	0	0	0	0
0	0	0	0	0	0	0	0	0
0	0	0	0	0	0	0	0	0
0	0	0	0	0	0	0	0	0
0	0	0	0	0	0	0	1	0
0	1	0	0	0	0	0	0	0
0	1	0	0	0	0	0	0	0
0	1	0	0	0	0	0	1	0
1	0	0	0	0	0	0	0	1
0	0	0	0	0	0	0	0	0
0	0	0	0	0	0	0	1	0
1	0	0	0	0	0	0	0	0
0	0	0	0	0	0	0	0	0
0	0	0	0	0	0	0	0	0
0	0	0	0	0	0	0	0	0
0	0	0	0	0	0	0	0	0
0	0	0	0	0	0	0	0	0
0	0	1	0	0	0	0	0	0
0	0	0	0	0	0	0	0	0
0	0	0	0	0	0	0	0	0
0	0	0	0	0	0	0	0	0
0	0	0	0	0	0	1	0	0
0	0	0	0	0	0	0	0	0
0	0	0	0	0	0	0	0	0
0	0	0	0	0	0	0	1	0
0	0	0	0	0	0	0	0	1
0	0	0	0	0	0	0	1	0
0	0	0	0	0	0	0	0	0
0	0	0	0	0	0	0	0	0
0	0	0	0	0	0	0	0	0

0	1	0	0	0	0	0	0	0
0	0	0	0	0	0	0	0	0
0	0	0	0	0	0	0	0	0
0	0	0	0	0	0	0	0	0
0	0	0	0	0	0	0	0	0
0	0	0	1	0	0	0	1	0
1	0	0	0	0	0	0	0	0
0	0	0	0	0	0	0	0	0
0	0	0	0	0	0	0	0	0
0	0	0	0	0	0	0	1	0
0	0	0	0	0	0	0	0	0
0	1	0	0	0	0	0	1	0
0	0	0	0	0	0	0	0	0
1	0	0	0	0	0	0	0	0
0	0	0	0	0	0	0	0	0
0	0	0	0	0	0	0	0	0
1	0	0	0	0	0	0	0	0
0	0	0	0	0	0	0	0	0
0	0	0	0	0	0	0	0	0
1	0	0	0	0	0	0	1	0
1	0	0	0	0	0	0	0	0
1	1	0	0	0	0	0	0	0
0	1	0	0	0	0	0	1	0
0	0	0	0	0	0	0	0	0
0	0	0	0	0	0	0	0	0
0	0	0	0	0	0	0	0	0
0	0	0	0	0	0	0	0	0
0	0	0	0	0	0	0	1	0
0	0	0	0	0	0	0	1	0
1	0	0	0	0	0	0	1	0
0	0	0	0	0	0	0	0	0
0	0	0	0	0	0	0	1	0
0	0	0	0	0	0	0	0	0
0	1	0	0	0	0	0	0	0
0	0	0	0	0	0	0	1	0
0	0	0	0	0	0	1	0	0
0	0	0	0	0	0	0	0	0
1	0	0	0	0	1	0	0	0
0	0	0	0	0	0	0	0	0
0	0	0	0	0	0	0	0	0
0	0	0	0	0	0	0	0	0
0	0	0	0	0	0	0	0	0
1	0	0	0	0	0	0	0	0
0	0	0	0	0	0	0	0	0
1	0	0	0	0	0	0	0	0
1	1	0	0	0	0	0	0	0
0	0	0	0	0	0	0	0	0
0	0	0	0	0	0	0	0	0
0	0	0	0	0	0	0	0	0
0	0	0	0	0	0	0	0	0
0	0	0	0	0	0	0	0	0
0	0	0	0	0	0	0	0	0
0	0	0	0	0	0	0	0	0
0	0	0	0	0	0	0	0	0
1	0	0	0	0	0	0	0	0
0	0	0	0	0	0	0	0	0

0	1	0	0	0	0	0	0	0
0	0	0	0	0	0	0	0	0
0	0	0	0	0	0	0	0	0
1	0	0	0	0	0	0	0	0
0	0	0	0	0	0	0	0	0
0	0	0	0	0	0	0	0	0
0	0	0	0	0	0	0	0	0
0	0	0	0	0	0	0	0	0
0	0	0	0	0	0	0	0	0
0	0	0	0	0	0	0	0	0
0	0	0	0	0	0	0	0	0
0	0	0	0	0	0	0	0	0
0	0	0	0	0	0	0	0	0
1	0	0	0	0	0	0	0	0
0	0	0	0	0	0	0	0	0
0	0	0	0	0	0	0	0	0
1	0	0	0	0	0	0	0	0
0	0	0	0	0	0	0	0	0
0	1	0	0	0	0	0	1	0
0	0	0	0	0	0	0	1	0
1	0	0	0	0	0	0	0	0
0	0	0	0	0	0	0	0	0
0	0	0	0	1	0	0	1	0
0	0	0	0	0	0	0	0	0
0	0	0	0	0	0	0	0	0
0	0	0	0	0	0	0	0	0
0	0	0	0	0	0	0	0	0
0	0	0	0	0	0	0	0	0
0	0	0	0	0	0	0	0	0
1	0	0	0	0	0	0	0	0
0	0	0	0	0	0	0	0	0
0	0	0	0	0	0	0	0	0
0	0	0	0	0	0	0	0	0
0	0	0	0	0	0	0	0	0
0	0	0	0	0	0	0	1	0
1	0	0	0	0	0	0	0	0
0	0	0	0	0	0	0	1	0
0	0	0	0	0	0	0	0	0
0	0	0	0	0	0	0	1	0
0	0	0	0	0	0	0	0	0
0	0	0	0	1	0	0	0	0
0	0	0	0	0	0	0	0	0
0	0	0	0	0	0	0	0	0
0	0	1	0	0	0	1	0	0
1	0	0	0	0	0	0	0	0
1	0	0	0	0	0	0	0	0
0	0	0	0	0	0	0	0	0
1	0	0	0	0	0	0	0	0
0	1	1	0	0	0	0	0	0
0	0	0	0	0	0	0	0	0
0	0	0	0	0	0	0	0	0
0	0	0	0	0	0	0	0	0
0	0	0	0	0	0	0	0	0
0	0	0	0	0	0	0	0	0
0	0	0	0	0	0	0	0	1
0	0	0	0	0	0	0	0	0

0	1	1	0	0	0	0	0	0
0	0	0	0	0	0	0	0	0
0	0	0	1	0	0	0	0	0
0	0	0	0	0	0	0	0	0
0	0	1	0	0	0	0	0	0
0	0	1	0	0	0	0	0	1
0	0	0	0	0	0	0	0	0
0	0	0	0	0	0	0	1	0
0	0	0	0	0	0	0	0	0
0	0	0	0	0	0	0	0	0
1	0	0	0	0	0	0	0	0
0	0	0	1	0	0	0	0	0
0	0	0	0	0	0	0	0	0
1	0	0	0	0	0	0	0	0
0	0	0	0	0	0	0	0	0
1	0	0	0	0	0	0	0	0
0	0	0	0	0	0	0	1	0
0	0	0	0	0	0	0	0	0
0	0	0	0	1	0	0	0	0
0	0	0	0	0	0	0	0	0
0	0	0	0	0	0	0	0	0
0	0	0	0	0	0	0	0	0
0	0	0	0	0	0	0	0	0
0	0	0	0	0	0	0	0	0
0	0	0	1	0	0	0	1	0
0	0	0	0	0	0	0	0	0
0	0	0	0	0	0	0	0	0
0	0	0	0	0	0	0	1	0
0	1	0	0	0	0	0	0	0
0	0	0	0	0	0	0	0	0
1	0	0	0	0	0	0	0	0
0	0	0	0	0	0	0	0	0
0	0	0	0	0	0	0	1	1
0	0	0	0	0	0	0	0	0
0	0	0	0	0	0	0	0	0
0	0	0	0	0	0	0	0	0
0	0	0	0	0	0	0	0	0
0	0	0	0	0	0	0	0	0
0	0	0	0	0	0	0	0	0
0	0	0	0	0	0	0	0	0
0	0	0	0	0	0	0	0	0
0	1	0	0	0	0	0	0	0
0	0	0	0	0	0	0	0	0
0	0	0	0	0	0	1	1	0
0	0	0	0	0	0	0	1	0
0	0	0	0	0	0	0	0	0
0	0	0	1	0	0	0	0	0
1	0	0	0	0	0	0	0	0
0	0	0	0	0	0	0	1	0
0	0	0	0	0	0	0	0	0
0	0	0	0	0	0	0	0	0
0	0	0	0	0	0	0	0	0
0	0	0	0	0	0	0	0	0
0	1	0	0	0	0	0	0	0
0	0	0	0	0	0	1	0	0
0	0	0	0	0	1	0	1	0

0	0	1	0	0	0	0	0	0
1	0	0	0	0	0	0	0	0
0	0	0	0	0	0	0	0	0
0	0	0	0	1	0	0	0	0
0	0	0	0	0	0	0	0	0
0	0	0	0	0	0	0	0	0
0	0	0	0	0	0	0	0	0
0	0	0	0	0	0	0	0	0
1	0	0	0	0	0	0	0	0
0	1	0	0	0	0	0	0	0
1	0	1	0	0	0	0	0	0
1	0	0	0	0	0	0	0	0
0	0	0	0	0	0	0	0	0
0	0	0	0	0	1	0	1	0
0	0	0	0	0	0	0	0	0
0	0	0	0	0	0	0	0	0
0	0	0	0	0	0	0	0	0
0	0	0	1	0	0	0	0	0
0	0	0	0	0	0	0	1	0
0	0	0	0	0	0	0	0	0
0	0	0	0	0	0	0	1	0
0	0	0	0	0	0	0	0	0
1	0	0	0	0	0	0	0	0
0	0	0	0	0	0	0	0	0
0	0	0	0	0	0	0	0	0
0	0	0	0	0	0	0	0	0
0	0	0	0	0	0	0	0	0
0	0	0	0	0	0	0	0	0
0	0	0	0	0	0	0	0	0
0	0	0	0	0	0	0	0	0
0	0	0	0	0	0	0	0	0
0	0	0	0	0	0	0	1	0
1	0	0	0	0	0	0	0	0
0	0	0	0	0	0	0	0	0
0	0	0	0	0	0	0	0	0
0	0	0	0	0	0	0	0	0
0	0	0	0	0	0	0	0	0
0	0	0	0	0	0	0	0	0
0	0	1	0	0	0	0	0	0
0	0	0	0	0	0	0	0	0
0	0	0	0	0	0	0	0	0
0	0	0	0	0	0	0	0	0
0	0	0	0	0	0	0	0	0
0	0	0	0	0	0	0	0	0
0	0	0	0	0	0	0	1	0
0	0	0	0	0	0	0	0	0
0	0	0	0	0	0	0	0	0
0	0	0	0	0	0	0	0	0
0	0	0	0	0	0	0	0	0
0	0	0	0	0	0	0	0	0
1	0	0	0	0	0	0	0	0
0	0	0	0	0	0	0	0	0
0	0	0	0	0	0	0	0	0
0	0	0	1	0	0	0	0	0
0	0	0	0	0	0	0	0	0
0	0	0	0	1	0	0	0	0

0	0	0	0	0	0	0	0	0
0	0	0	0	0	0	0	1	0
0	0	0	1	0	0	0	0	0
0	0	0	0	0	0	0	0	0
0	0	0	0	0	0	0	1	0
0	0	0	0	0	0	0	1	0
1	0	0	0	0	0	0	0	0
0	0	0	0	0	0	0	0	0
0	0	0	0	0	0	0	0	0
0	0	0	0	1	0	0	0	0
0	0	0	0	0	0	0	0	0
0	0	0	0	0	0	0	0	1
0	0	0	0	0	0	0	0	0
0	0	0	0	0	0	0	0	0
0	0	0	1	0	0	0	0	0
0	0	0	0	0	0	0	0	0
0	0	0	0	0	0	0	0	0
0	0	0	0	0	0	0	0	0
0	1	0	0	0	0	0	0	0
0	0	0	0	0	0	0	0	0
0	0	0	0	0	0	0	0	0
0	0	0	0	0	0	0	0	0
0	0	0	0	0	0	0	1	0
0	0	0	0	0	0	0	0	0
0	0	0	0	0	0	0	0	1
0	0	0	0	0	0	0	1	0
1	0	0	0	0	0	0	0	0
0	0	0	0	0	0	0	0	0
0	0	0	0	0	0	0	0	0
0	0	0	0	0	0	0	0	0
0	0	0	0	0	0	0	1	0
0	0	0	0	0	0	0	0	0
0	0	0	0	0	0	0	0	0
1	0	0	0	0	0	0	1	0
0	0	0	0	0	0	0	0	0
0	0	0	0	0	0	0	0	0
1	0	0	0	1	0	0	0	0
0	0	0	0	0	0	0	0	0
0	0	0	0	0	0	0	0	0
0	0	0	0	0	0	0	0	0
1	0	0	0	0	0	0	0	0
0	1	0	0	0	0	0	0	0
0	0	0	0	0	0	0	0	0
0	0	0	0	0	1	0	0	0
0	0	0	0	0	0	0	0	0
0	0	0	0	0	0	0	0	0
1	0	0	0	0	0	0	0	0
0	0	0	0	0	0	0	0	0
0	0	0	0	0	0	0	0	0
0	0	0	0	0	0	0	0	0
0	0	0	0	0	0	1	0	0
1	0	0	0	0	0	0	0	0
0	0	0	1	0	0	0	0	0
0	0	0	0	0	0	0	0	0
0	0	0	0	0	0	0	0	0
0	0	0	0	0	0	0	0	0

0	0	0	0	0	0	0	0	0
0	0	0	0	0	0	0	0	0
1	0	0	0	0	0	0	0	0
0	0	0	0	0	0	0	0	0
0	0	0	0	0	0	0	0	0
0	0	0	0	0	0	0	0	0
0	0	0	0	0	0	0	0	0
0	0	0	0	0	0	0	0	0
0	1	0	0	0	0	0	1	0
0	0	0	0	0	0	1	0	0
0	0	0	0	0	0	0	0	0
0	0	0	0	0	0	0	0	0
0	1	0	0	0	0	1	0	0
0	0	0	0	0	0	0	0	0
0	0	0	0	0	0	0	0	0
1	0	0	0	0	0	0	0	0
0	0	0	0	0	0	1	0	1
0	0	0	0	1	0	0	0	0
1	0	0	0	0	0	0	0	0
0	0	0	0	0	0	0	0	0
0	0	0	0	0	0	0	0	0
0	0	0	0	0	0	0	1	0
0	0	0	0	0	0	0	0	0
0	0	0	0	0	0	0	1	0
0	0	0	0	0	0	0	0	0
0	0	0	0	0	0	0	0	0
0	0	0	0	0	0	0	0	0
0	0	0	0	0	0	0	0	0
0	0	0	0	0	0	0	1	0
0	0	0	0	0	0	0	0	0
0	0	0	0	0	0	0	0	0
0	0	0	0	0	0	0	0	0
0	0	0	0	1	0	0	0	0
0	0	0	0	0	0	0	0	0
0	0	0	0	0	0	0	1	0
0	0	0	0	0	0	0	0	0
0	0	0	0	0	0	0	0	0
0	0	0	0	0	0	0	1	0
0	0	0	0	0	0	0	0	0
0	0	0	0	0	0	0	0	0
0	0	0	0	0	0	0	0	0
0	0	0	0	0	0	0	0	0
0	0	0	0	0	0	0	0	0
0	0	0	0	0	0	0	0	1
1	0	0	0	0	0	0	0	0
0	0	0	0	0	0	0	0	0
0	0	0	0	0	0	0	0	0
0	0	0	0	0	0	0	0	0
0	0	0	0	0	0	0	0	0
0	0	0	0	0	0	0	0	0
0	0	0	0	0	0	0	0	0
0	1	0	0	0	0	0	0	0
0	0	0	0	0	0	0	1	0
0	0	0	0	0	0	0	0	0
0	0	0	0	0	0	0	0	0
0	0	0	0	0	1	0	0	0

0	0	0	1	0	0	0	0	0
0	0	0	0	0	0	0	0	0
0	0	0	0	0	0	0	0	0
0	0	0	0	0	0	0	0	0
1	0	0	0	0	0	0	0	0
0	0	0	0	0	0	0	0	0
0	0	0	0	0	0	0	0	0
0	0	0	0	0	0	0	0	0
1	0	0	0	0	0	0	0	0
1	0	0	0	0	0	0	0	0
0	0	0	0	0	0	0	0	0
0	0	0	0	0	0	0	0	0
1	0	0	0	0	0	0	0	0
0	0	0	0	1	0	0	0	0
0	0	0	0	0	0	0	0	0
0	0	0	0	0	0	0	0	0
1	0	0	0	0	0	0	0	0
1	0	0	0	0	0	0	0	0
0	0	0	0	0	0	0	0	0
1	0	0	0	0	0	0	0	0
0	0	0	0	0	0	0	0	0
1	0	0	0	0	0	0	0	0
0	0	0	0	0	0	0	0	0
0	0	0	0	0	0	0	0	0
1	0	0	0	0	0	0	0	0
0	0	1	0	0	0	0	0	0
1	0	0	0	0	0	0	0	0
0	0	0	0	0	0	0	1	0
0	0	1	0	0	0	0	0	0
0	0	0	0	0	0	0	0	0
0	0	0	0	0	0	0	1	0
0	0	0	0	0	0	0	0	0
1	0	0	0	0	0	0	0	0
0	0	0	0	0	0	0	0	0
1	0	1	0	0	0	0	0	0
1	0	0	0	0	0	1	0	0
0	0	0	0	0	0	0	0	0
1	0	0	0	0	0	0	0	0
0	0	0	0	0	0	0	0	0
0	0	0	0	0	0	0	0	0
0	0	0	0	0	0	0	0	0
1	0	0	0	0	0	0	0	0
0	0	0	0	0	0	0	0	0
0	0	0	0	0	0	0	0	0
0	0	0	0	0	0	1	0	0
1	0	0	0	0	0	0	0	0
0	0	0	0	0	0	0	0	0
0	0	0	0	0	0	0	0	0
0	0	0	0	0	0	0	0	0
0	0	0	0	0	0	0	0	0
0	0	0	0	0	0	0	0	0
0	0	0	0	0	0	0	0	0
0	1	0	0	0	0	0	0	0
1	0	0	0	0	0	0	0	0
0	1	0	0	0	0	0	0	0
1	0	0	0	0	0	0	0	0

0	0	0	0	0	0	0	0	0
0	0	0	0	0	0	0	0	0
0	0	0	0	0	0	0	0	0
0	0	0	0	0	0	0	0	0
1	0	0	0	0	0	0	0	0
0	0	0	0	0	0	0	0	0
0	0	0	0	0	0	1	0	0
1	0	0	0	0	0	0	0	0
1	0	0	0	0	0	0	0	0
0	0	0	0	0	0	0	0	0
0	0	0	0	0	0	0	0	0
0	0	0	0	0	0	0	0	0
0	0	0	0	0	0	0	0	0
0	0	0	0	0	0	0	1	0
1	0	0	0	0	0	0	0	0
0	0	0	0	0	0	0	0	0
0	0	0	0	0	0	0	0	0
0	0	0	0	0	0	1	0	0
0	0	0	0	0	0	0	1	0
0	0	0	0	0	0	0	0	0
1	0	0	0	0	0	0	0	0
1	0	0	0	0	0	0	0	0
1	0	0	1	0	0	0	0	0
0	0	0	0	0	0	0	1	0
0	0	1	0	0	0	0	0	0
0	0	0	0	0	0	0	0	0
0	0	0	0	0	0	0	0	0
1	1	0	0	0	0	0	0	0
0	0	0	0	0	0	0	0	0
1	0	0	0	0	0	0	0	0
0	0	0	0	0	0	0	0	0
0	0	0	0	0	0	0	0	0
0	0	0	0	0	0	0	0	0
0	1	0	0	0	0	1	1	1
0	0	0	0	0	0	0	0	0
0	0	0	0	0	0	0	0	0
0	0	0	0	0	0	0	0	0
0	0	0	0	0	0	0	0	0
0	0	0	0	0	0	0	0	0
0	0	0	0	0	0	0	0	0
0	0	0	0	0	0	0	0	0
0	0	0	0	0	0	0	0	0
0	0	0	0	1	0	0	0	0
0	0	0	1	0	0	0	1	0
0	0	0	0	0	0	0	0	0
0	0	0	0	0	1	0	0	0
1	0	0	0	0	0	0	0	0
0	0	0	0	0	0	0	0	0
0	0	0	0	0	0	0	0	0
0	0	0	0	0	0	0	0	0
0	0	0	0	0	0	0	0	0
0	0	0	0	0	0	0	0	0
1	0	0	0	0	0	0	0	0
0	0	0	0	0	0	0	0	0
0	0	0	0	1	0	0	0	0
0	0	0	0	0	0	0	0	0

0	0	0	0	0	0	0	0	0
0	0	0	0	0	0	0	0	0
0	1	0	0	0	0	0	0	0
0	0	0	0	0	0	0	0	0
0	0	0	0	0	0	0	0	0
0	0	0	0	0	0	0	0	0
0	0	0	0	0	0	0	0	0
0	0	0	0	0	0	0	0	0
1	0	0	0	0	0	0	0	0
1	0	0	0	0	0	0	0	0
0	0	0	0	0	0	0	0	0
0	0	0	0	0	0	0	0	0
1	1	0	0	0	0	0	0	0
0	0	0	0	0	0	0	0	0
0	0	0	0	0	0	0	0	0
1	0	0	0	0	0	0	0	0
0	0	0	0	0	0	0	0	0
0	0	0	0	0	0	0	0	0
0	0	0	0	0	0	0	0	0
0	0	0	0	0	0	0	1	0
0	0	0	0	0	0	0	0	0
1	0	0	0	0	0	0	0	0
1	0	0	0	0	0	0	1	0
1	0	0	0	0	0	0	0	0
0	0	0	0	0	0	0	0	0
0	0	0	0	0	0	0	0	0
0	0	0	0	0	0	0	0	0
0	0	0	0	0	0	0	0	0
0	0	0	0	0	0	0	0	0
0	0	1	0	0	0	0	0	0
1	0	0	0	0	0	0	0	0
0	0	0	0	0	0	0	0	0
0	0	0	0	1	0	0	0	0
1	0	0	0	0	0	0	0	0
0	0	0	0	0	0	0	1	0
0	0	1	0	0	0	0	1	0
0	0	0	0	0	0	0	0	0
0	0	0	0	0	0	0	0	0
1	0	0	0	0	0	0	0	0
0	0	0	0	0	0	0	0	0
0	0	0	0	0	0	0	0	0
1	0	0	0	0	0	0	0	0
0	0	1	0	1	0	0	0	0
0	0	0	0	0	0	0	0	0
0	0	0	0	0	0	0	0	0
0	0	0	0	0	0	0	0	0
0	0	0	0	0	0	0	0	0
0	0	0	0	0	0	0	0	0
0	0	0	0	0	0	0	1	0
0	0	0	0	1	0	0	0	0
0	0	0	0	0	0	0	1	0
0	0	0	0	0	0	0	1	0
0	0	0	0	0	0	0	0	0
0	0	0	0	0	0	0	0	0
0	0	0	0	0	0	0	0	0
0	0	0	0	0	0	0	0	0

0	0	0	0	0	0	0	0	0
0	0	0	0	0	0	0	0	0
1	0	0	0	0	0	0	0	0
0	0	0	0	0	0	0	1	0
0	0	0	0	0	0	0	0	0
0	0	0	0	0	0	0	0	0
0	0	0	0	0	0	0	0	1
0	0	0	0	0	0	0	0	0
0	0	0	0	0	0	0	0	0
0	0	0	1	0	0	0	0	0
0	0	1	0	1	0	0	0	0
1	0	0	0	0	0	0	0	0
0	0	0	0	0	0	0	0	0
0	0	0	0	0	0	0	1	0
0	0	0	0	0	0	0	0	0
0	0	0	0	0	0	0	0	0
0	0	0	0	0	0	0	0	0
0	0	0	0	0	0	0	0	0
0	0	0	0	0	1	0	0	0
0	0	0	0	0	0	0	0	0
0	0	0	0	0	0	0	0	0
0	0	0	0	0	0	0	0	0
0	0	0	0	0	0	0	0	0
0	0	0	0	1	0	0	0	0
1	0	0	0	0	0	0	0	0
0	1	0	0	0	0	0	0	0
0	0	0	0	0	0	0	0	0
0	0	0	0	1	0	0	0	0
0	0	0	0	0	0	0	0	0
0	0	0	0	1	0	0	0	0
0	0	0	0	0	0	0	0	0
1	0	0	0	0	0	0	0	0
0	0	0	0	0	0	0	0	0
1	0	0	0	0	0	0	0	0
0	0	0	0	0	0	0	0	0
0	0	0	0	0	0	0	0	0
0	0	0	0	0	0	0	0	0
0	0	0	0	0	0	0	0	0
0	1	0	0	0	0	0	0	0
0	0	0	0	0	0	0	0	0
0	0	0	0	0	0	0	0	0
0	0	0	0	0	0	0	0	0
0	0	0	0	0	0	0	0	0
0	0	0	0	0	0	0	0	0
1	0	0	0	0	0	0	0	0
0	0	0	0	0	0	0	0	0
0	0	0	0	0	0	0	0	0
0	0	0	0	0	0	0	0	0
0	0	0	0	0	0	0	1	0
0	0	0	0	0	0	0	0	0
0	1	0	0	0	0	0	0	0
0	0	0	0	0	0	0	0	0
0	0	0	0	0	0	0	0	0
0	0	0	0	0	0	0	0	0
1	0	0	0	0	0	0	0	0
0	0	0	0	0	0	0	0	0

0	0	0	0	0	0	0	0	0
0	0	0	0	0	0	0	0	0
0	0	0	0	0	0	0	0	0
1	0	0	0	0	0	0	0	0
0	0	0	0	0	0	0	0	0
1	0	0	0	0	0	0	0	0
0	0	0	0	0	0	0	0	0
0	0	0	0	0	0	0	0	0
0	0	0	0	0	0	0	0	0
0	0	0	0	1	0	0	0	0
0	0	0	0	0	0	0	0	0
1	0	0	0	0	0	0	0	0
0	0	0	0	0	0	0	0	0
0	0	0	0	0	0	0	0	0
0	0	0	0	0	0	0	0	0
0	0	0	0	0	0	0	0	0
1	0	0	0	0	0	0	0	0
0	1	0	0	0	0	0	0	0
0	0	0	0	0	0	0	0	0
0	0	0	0	0	0	0	0	0
0	0	0	0	0	0	0	0	0
1	0	0	0	0	0	0	0	0
0	0	0	0	0	0	0	0	0
0	0	0	0	0	0	0	0	0
0	0	0	0	0	0	0	1	0
0	0	0	0	0	0	0	0	0
1	0	0	0	0	0	0	0	0
0	0	0	0	0	0	0	0	0
0	0	0	0	0	0	0	0	0
0	0	0	0	0	0	0	0	0
0	0	0	0	0	0	0	0	0
0	0	0	0	0	0	0	0	0
0	0	0	0	0	0	0	0	1
1	0	0	0	0	0	0	1	0
0	0	0	0	0	0	0	0	0
0	0	0	0	0	0	0	0	0
0	0	0	0	0	0	0	0	0
0	0	0	0	0	0	0	0	0
0	0	0	0	0	0	0	0	0
0	0	0	0	0	0	0	0	0
0	0	0	0	0	0	0	0	0
0	0	0	0	0	0	0	0	0
0	0	0	0	0	0	1	0	0
0	0	0	0	0	0	0	0	0
0	0	0	0	0	0	0	0	0
0	0	0	0	0	0	0	0	0
1	0	0	0	0	0	0	0	0
0	0	0	0	0	0	0	1	0
0	0	0	0	1	0	0	0	0
0	0	0	0	1	0	0	0	0
0	0	0	0	0	0	0	0	0
1	0	0	0	0	0	0	0	0
0	0	0	0	0	0	0	0	0
0	0	0	0	0	0	0	0	0
0	0	0	0	0	0	0	0	0
0	0	0	0	0	0	0	0	0

0	0	0	0	0	0	0	0	0
0	0	0	0	0	0	0	0	0
0	0	0	0	0	0	0	0	1
0	0	0	0	0	0	0	0	0
1	0	0	0	0	0	0	0	0
0	0	0	0	0	0	0	0	0
1	0	0	0	0	0	0	0	0
0	0	0	0	0	0	0	0	0
0	0	0	0	0	0	1	0	0
1	0	1	0	0	0	0	0	0
0	1	0	0	0	0	0	0	0
0	0	0	0	0	0	0	1	0
1	0	0	0	0	0	0	0	0
1	0	0	0	0	0	0	0	0
1	0	0	0	0	0	0	0	0
0	0	0	0	0	0	0	0	0
0	0	0	0	0	0	0	0	0
0	0	0	0	0	0	0	0	0
0	1	0	0	0	0	0	0	0
0	0	0	0	0	0	0	0	0
0	0	0	0	1	1	0	0	0
0	0	0	0	0	0	0	0	0
0	1	0	0	0	0	0	0	0
0	0	0	0	0	0	0	0	0
0	1	0	0	0	0	0	0	0
1	0	0	0	0	0	0	0	0
0	0	0	0	0	0	0	0	0
0	0	0	0	0	0	0	1	0
0	0	0	0	0	0	1	0	0
1	0	0	0	0	0	0	0	0
0	0	0	0	0	0	0	0	0
0	0	0	0	0	0	0	0	0
1	0	0	0	0	0	0	0	0
0	0	0	0	0	0	0	0	0
1	0	1	0	0	0	0	0	0
1	0	0	0	0	0	0	0	0
0	0	0	0	0	0	0	0	0
0	1	0	0	0	0	0	0	0
0	0	0	0	0	0	0	1	0
0	0	0	0	0	0	0	0	0
0	0	0	0	0	0	0	0	0
0	0	0	0	0	0	0	0	0
0	0	0	0	0	0	0	0	0
0	0	0	0	0	0	0	0	0
0	0	0	0	0	0	0	0	0
0	0	0	0	0	0	0	0	0
0	0	0	0	0	0	0	0	0
1	0	0	0	0	0	0	0	0
0	0	0	0	0	0	0	0	0
0	0	0	0	0	0	0	0	0
0	0	0	0	0	0	0	0	0
0	0	0	0	0	0	0	0	0
0	0	0	0	0	0	0	0	0
0	0	0	0	0	0	0	0	0
0	0	0	0	0	0	0	0	0
0	0	0	0	0	0	0	0	0

0	0	1	0	0	0	0	0	0
0	0	0	0	1	0	0	0	0
0	0	0	0	0	0	0	0	0
0	0	0	0	0	0	0	0	0
1	0	0	0	0	0	0	0	0
0	0	1	0	0	0	0	0	0
0	0	0	0	0	0	0	0	0
1	0	0	0	0	0	0	0	0
1	0	0	0	0	0	0	0	0
0	0	0	0	0	0	0	0	0
1	0	0	0	0	0	0	0	0
0	0	0	0	0	0	0	0	1
1	0	0	0	0	0	0	0	0
0	0	0	0	0	0	0	0	0
0	0	0	0	0	0	0	0	0
1	0	0	0	1	0	0	0	0
0	0	0	0	1	0	0	0	0
0	0	0	0	0	0	0	0	0
0	0	0	0	0	0	0	0	0
0	0	0	0	0	0	0	0	0
0	0	0	0	0	0	0	0	0
0	0	0	0	0	0	0	1	0
0	0	0	0	0	0	0	0	0
0	0	0	0	0	0	0	0	0
0	0	0	0	0	0	0	0	0
1	0	0	0	0	0	0	0	0
0	0	0	0	0	0	0	0	1
0	0	0	0	0	0	0	0	0
0	0	0	0	0	0	0	0	0
1	0	0	0	0	0	0	0	0
0	0	0	0	0	0	0	0	0
0	0	0	0	0	0	0	1	0
1	0	0	0	0	0	0	0	0
1	0	0	0	0	0	0	0	0
0	1	0	0	0	0	0	0	1
0	0	0	0	0	0	0	0	0
0	0	0	0	0	0	0	0	0
0	0	0	0	0	0	0	0	0
0	0	0	0	0	0	0	0	0
0	0	0	0	0	0	0	0	0
0	0	0	0	0	0	0	0	0
1	0	0	0	0	0	0	0	0
0	0	0	0	0	0	0	0	0
0	0	0	0	0	0	0	0	0
0	0	0	0	0	0	0	0	0
0	0	0	0	0	0	0	0	0
0	0	0	0	0	0	1	0	0
0	0	0	0	0	0	0	0	0
0	0	0	0	0	0	0	0	0
0	0	0	0	0	0	0	0	0
0	0	0	0	0	0	0	1	0
0	0	0	0	0	0	0	0	0
0	0	0	0	0	0	0	0	0
0	0	0	1	0	0	0	0	0
0	0	0	0	0	0	0	0	0
0	0	0	0	0	0	0	0	0

0	0	0	0	0	0	0	0	0
0	0	0	0	0	0	0	0	0
1	0	0	0	0	0	0	0	0
0	0	0	0	0	0	0	0	0
0	0	0	0	0	0	0	0	0
0	0	0	0	0	0	0	0	0
1	0	0	0	0	0	0	0	0
0	0	0	0	0	0	0	0	0
0	0	0	0	0	0	0	0	0
0	0	0	0	1	0	0	0	0
0	0	0	0	0	0	0	0	0
0	0	0	0	1	0	0	0	0
0	0	0	0	1	0	0	0	0
0	1	0	0	0	0	0	0	0
0	0	0	0	0	0	0	0	0
0	0	0	0	0	0	0	0	0
0	0	0	0	0	0	0	0	0
0	0	0	0	0	0	0	0	0
0	0	0	0	0	0	0	0	0
1	0	0	0	0	0	0	0	0
0	0	0	0	0	0	0	0	0
0	0	0	0	0	0	0	0	0
0	0	0	0	0	0	0	0	0
0	0	0	0	0	0	0	0	0
0	0	1	0	0	0	0	0	0
0	0	0	0	0	0	0	0	0
0	0	0	0	0	0	0	0	0
0	0	0	0	0	0	0	0	0
0	0	0	0	0	0	0	0	0
0	0	0	0	1	1	0	0	0
0	0	0	0	0	0	0	1	0
0	0	0	0	0	0	0	0	0
0	0	0	0	0	0	0	0	0
1	0	0	0	0	0	0	0	0
0	1	1	0	0	0	0	0	0
0	0	0	0	0	0	0	0	0
0	0	0	0	0	0	0	0	0
1	0	0	0	0	0	0	0	0
1	0	0	0	0	0	0	0	0
0	0	0	0	0	0	0	0	0
1	0	0	0	0	0	0	0	0
0	0	0	0	0	0	0	0	0
0	0	0	0	0	0	0	1	0
0	0	0	0	0	0	0	0	0
1	0	0	0	0	0	0	0	0
1	0	0	0	0	0	0	1	0
0	0	0	0	0	0	0	0	0
0	0	0	0	0	0	0	0	0
1	0	0	0	0	0	0	0	0
0	0	0	0	0	0	0	0	0
0	0	0	0	0	0	0	0	0
0	0	0	0	0	0	0	0	0
0	0	0	0	0	0	0	1	0
0	1	0	1	0	0	0	0	0
0	0	0	0	0	0	0	0	0
1	0	0	0	0	0	0	0	0

0	0	0	0	0	0	0	0	0
1	0	0	0	0	0	1	0	0
0	0	1	0	0	0	0	0	0
1	0	0	0	0	0	0	0	0
1	0	0	0	0	0	0	0	0
0	0	0	0	0	0	0	0	0
0	0	0	0	0	0	0	0	0
0	0	0	0	0	0	0	0	0
0	0	0	0	0	0	0	0	0
0	1	0	0	0	0	0	0	0
1	0	0	0	0	0	0	0	0
0	0	0	0	0	0	0	0	0
0	1	0	0	0	0	0	0	0
0	1	0	0	0	0	0	0	0
0	0	0	0	0	0	0	0	0
0	0	0	0	0	0	0	1	0
1	0	0	0	0	0	0	0	0
0	0	1	0	0	0	0	0	1
0	0	0	0	0	0	0	0	0
0	0	0	0	0	0	0	0	0
0	0	0	0	0	0	0	0	0
0	0	0	1	0	0	0	0	0
0	1	0	0	0	0	1	0	0
0	0	0	0	0	0	0	0	0
0	0	0	0	0	0	0	0	0
0	0	0	0	0	0	0	0	0
0	0	0	0	0	0	0	0	0
0	0	0	0	0	0	1	0	0
1	0	0	0	0	0	0	0	0
0	0	1	0	0	0	0	1	0
1	0	0	0	0	0	0	0	0
0	0	0	0	0	0	0	0	0
1	0	0	0	0	0	0	0	0
0	0	0	0	0	0	0	0	0
0	0	0	0	0	0	0	0	0
0	0	0	0	0	0	0	0	0
0	0	0	0	0	0	0	0	0
0	0	0	0	0	0	0	0	0
0	0	0	0	0	0	0	0	0
1	0	0	0	0	0	0	0	0
1	0	0	0	0	0	0	0	0
0	0	0	0	0	1	0	0	0
1	0	0	0	0	0	0	0	0
0	0	0	0	0	0	0	0	0
1	0	0	0	0	0	0	0	0
0	1	0	0	0	0	0	0	0
0	0	0	0	0	0	0	0	0
0	0	0	0	0	0	0	0	0
1	0	0	0	0	0	0	0	0
0	0	0	0	0	0	0	1	0
1	1	0	0	0	0	0	0	0
0	0	0	0	0	0	0	0	0
0	0	0	0	0	0	0	0	0
0	0	0	0	0	0	0	1	0
0	0	0	0	0	0	0	0	0
0	0	1	0	0	0	0	0	0

0	0	0	0	1	0	0	0	0
1	0	0	0	0	0	0	1	0
0	0	0	0	0	0	0	0	0
0	1	0	0	0	0	0	0	0
1	0	0	0	0	0	0	0	0
0	0	0	0	0	0	0	0	0
0	0	0	0	0	0	0	0	0
0	0	0	0	0	0	0	0	0
0	0	0	0	0	0	0	0	0
0	0	0	0	0	0	0	0	0
0	0	0	0	0	0	0	0	0
0	0	0	0	0	0	0	1	0
0	0	1	0	0	0	0	0	0
1	0	0	0	0	0	0	0	0
1	0	0	0	0	0	0	0	1
0	0	0	0	0	0	0	0	0
0	0	0	1	0	0	0	0	0
0	0	0	0	0	0	0	1	0
0	0	0	1	0	0	0	0	0
0	0	0	0	0	0	0	0	0
0	0	0	0	0	0	0	0	0
0	0	0	0	0	0	0	0	0
0	0	0	0	1	0	0	0	0
0	0	0	0	1	1	0	0	0
1	0	0	0	0	0	0	0	0
0	0	0	0	0	0	0	0	0
0	0	0	0	0	0	0	0	0
0	0	0	0	0	0	0	0	0
0	0	0	0	0	0	0	0	0
0	0	0	0	0	0	0	0	0
0	0	0	0	0	0	0	0	0
0	0	0	0	0	0	0	0	0
0	0	0	0	0	0	0	0	0
1	0	0	0	0	0	0	0	0
0	0	0	0	0	0	0	0	0
0	0	0	0	0	0	0	0	0
0	0	0	0	0	0	0	0	0
1	0	0	0	0	0	0	0	0
0	0	0	0	0	0	0	0	0
0	0	0	0	0	0	0	0	0
0	0	0	0	0	0	0	1	0
0	0	0	0	0	0	0	0	0
0	0	0	0	0	0	0	0	0
0	0	0	0	0	0	0	0	0
0	0	0	0	0	0	0	0	0
1	0	0	0	0	0	0	0	0
0	0	0	0	0	0	0	1	0
0	0	0	0	0	0	0	0	0
0	0	0	0	0	0	0	0	0
0	0	0	0	0	0	0	1	0
0	0	0	0	0	0	0	0	0
0	0	0	0	0	0	0	0	0
0	0	0	0	0	0	0	0	0
0	0	0	0	0	0	0	0	0
0	0	0	0	0	0	0	0	0
1	0	0	0	0	0	0	0	0

1	0	0	0	0	0	0	0	0
0	1	0	0	0	0	0	0	0
0	0	0	0	0	0	0	0	0
0	0	0	0	0	0	0	0	1
0	0	0	0	0	0	0	0	0
0	0	0	0	0	0	0	0	0
1	0	0	0	0	0	0	0	0
0	0	0	0	0	0	0	0	0
0	0	0	0	0	0	0	0	0
0	0	0	0	0	0	0	0	0
0	0	0	0	0	0	0	0	0
0	0	0	0	0	0	0	0	0
0	0	0	0	0	0	0	0	0
0	0	0	0	0	0	0	0	0
0	0	0	0	0	0	0	0	0
0	0	0	0	0	0	0	0	0
0	0	0	0	0	0	0	0	0
0	0	0	0	0	0	0	0	0
0	0	0	0	0	0	0	1	0
1	0	0	0	0	0	0	0	0
0	0	0	0	1	0	0	1	0
0	0	0	0	0	0	0	0	0
0	0	0	0	0	0	0	0	0
0	0	0	0	0	0	0	0	0
0	0	0	0	0	0	0	0	0
0	0	0	0	0	0	0	0	0
1	0	0	0	0	0	0	0	0
0	0	0	0	0	0	0	1	0
0	0	0	0	0	0	0	0	0
0	0	0	0	0	0	0	0	0
0	0	0	0	0	0	0	0	0
0	0	0	0	0	0	0	0	0
0	0	0	0	0	0	0	0	0
0	0	0	0	0	0	0	0	0
1	0	0	0	0	0	0	0	0
0	0	0	0	0	0	0	0	0
1	0	0	0	0	0	0	0	0
0	0	0	0	0	0	0	0	0
0	0	0	1	0	0	1	0	0
0	0	0	0	0	0	0	0	0
0	0	0	0	0	0	0	0	0
0	0	0	0	0	0	0	0	0
0	0	0	0	0	0	0	0	0
1	0	0	0	0	0	0	1	0
0	0	0	0	0	0	0	0	0
0	0	0	0	1	0	0	0	0
0	0	0	0	0	0	0	0	0
0	0	0	0	0	0	0	0	0
0	0	0	0	0	0	0	0	0
0	0	0	0	0	0	0	1	0
0	0	0	0	0	0	0	0	0
0	0	0	0	0	0	0	0	0
0	0	0	0	0	0	0	0	0
0	0	0	0	0	0	0	0	0
0	0	0	0	0	0	0	0	0
0	0	1	1	0	0	0	0	0

0	0	0	0	0	0	0	0	0
0	0	0	0	0	0	0	0	0
0	0	0	1	0	0	0	0	0
1	0	0	0	1	0	0	0	0
0	0	0	0	0	1	0	0	0
1	0	0	0	0	0	0	0	0
0	0	0	0	0	0	0	0	0
0	0	0	0	0	0	0	0	0
0	1	0	0	0	0	1	0	0
0	0	0	0	0	0	0	0	0
0	0	0	0	0	0	0	0	0
1	0	0	0	0	0	0	0	0
0	0	0	0	0	0	0	0	0
0	0	0	1	0	0	0	0	0
1	0	0	0	0	0	0	0	0
0	0	0	0	0	0	0	0	0
0	0	0	0	0	0	0	0	0
0	0	0	0	0	0	0	0	0
1	0	0	0	0	0	0	0	0
0	0	0	0	0	0	0	0	0
0	0	0	0	0	0	0	0	0
1	0	0	0	0	0	0	0	0
0	0	0	0	0	0	0	0	0
0	0	0	0	0	0	0	0	0
0	0	0	0	0	0	0	1	0
0	0	0	0	0	0	0	0	0
0	0	0	0	0	0	0	0	0
0	0	0	0	0	0	0	0	0
0	0	0	0	0	0	0	0	0
0	0	0	0	0	0	0	0	0
0	0	0	0	1	1	0	0	0
0	0	0	0	0	0	0	1	0
0	0	0	0	0	0	0	0	0
0	0	0	0	0	0	0	0	0
0	0	0	0	0	0	0	0	0
0	0	0	0	0	0	0	0	0
1	0	0	0	0	0	0	0	0
0	0	0	0	0	0	0	0	0
0	0	0	0	0	0	1	0	0
0	0	0	0	0	0	0	0	0
0	0	0	0	0	0	1	0	0
0	0	0	0	0	0	0	0	0
1	0	0	0	0	0	0	0	0
0	0	0	0	0	0	0	0	0
0	0	0	0	1	0	0	0	0
0	0	0	0	0	0	0	0	0
0	0	0	0	0	0	0	0	0
0	0	0	0	0	0	0	0	0
0	0	0	0	0	0	0	0	0
1	0	0	0	0	0	0	0	0
1	0	0	0	0	0	0	0	0
0	0	0	0	0	0	0	0	0
0	1	0	0	0	0	0	0	0
0	0	0	0	0	0	0	0	0
0	0	0	0	0	0	0	0	0
0	0	0	0	0	0	0	0	0

1	0	0	0	0	0	0	0	0
0	0	0	0	0	0	0	0	0
1	1	0	0	0	0	0	0	0
0	0	0	0	0	0	0	0	0
0	0	0	0	0	0	0	0	0
0	0	0	0	0	0	0	0	0
0	0	0	0	0	0	0	0	0
0	0	0	0	0	0	0	0	0
0	0	0	0	0	0	0	0	0
0	0	0	0	0	0	0	0	0
0	0	0	0	0	0	0	0	0
0	0	0	0	0	0	0	0	0
0	0	0	0	0	0	0	0	0
0	0	0	0	0	0	0	0	0
1	0	0	0	0	0	0	0	0
0	0	0	0	0	0	1	0	0
0	0	0	0	0	0	0	0	0
0	0	0	0	0	0	0	0	0
1	0	0	0	0	0	0	1	0
1	0	0	0	0	0	0	0	0
0	0	0	0	0	0	0	0	0
0	0	0	0	0	0	0	0	0
0	0	0	0	0	0	0	0	0
1	0	0	0	0	0	0	0	0
0	0	0	0	0	0	0	1	0
0	0	0	0	0	0	0	0	0
1	0	1	0	0	0	0	0	0
1	0	0	0	0	0	0	0	0
0	0	0	0	0	0	0	0	0
1	0	0	0	0	0	0	0	0
0	0	0	0	0	0	0	0	0
0	0	0	0	0	0	0	0	0
0	0	0	0	0	0	0	0	0
0	0	0	0	0	0	0	0	0
0	0	0	0	0	0	0	0	0
0	0	0	0	0	0	0	0	0
0	0	0	0	0	0	0	0	0
0	1	0	0	0	0	0	0	0
1	0	0	0	0	0	0	0	0
0	0	1	0	0	0	0	0	0
0	0	0	0	0	0	0	0	0
1	1	0	0	0	0	0	0	0
0	0	0	0	0	0	0	0	0
0	0	1	0	0	0	0	0	0
0	0	0	0	0	0	0	0	0
0	0	0	0	0	0	0	0	0
0	0	0	0	0	0	0	1	0
1	0	0	0	0	0	0	0	0
0	0	0	0	0	0	0	0	0
0	0	0	0	0	0	0	0	0
0	0	0	0	0	0	0	0	0
0	0	0	0	0	0	0	0	0
0	0	0	0	0	0	0	0	0
0	0	0	0	0	0	0	1	0
0	0	0	0	0	0	0	0	0
0	0	0	0	0	0	0	0	0

0	0	1	1	0	0	0	1	0
1	0	0	0	0	0	0	0	0
1	0	0	0	0	0	0	1	0
0	0	0	0	0	0	0	0	0
0	0	0	0	0	0	0	0	0
0	0	0	0	0	0	0	0	0
0	0	0	0	0	0	0	0	0
0	0	0	0	0	0	0	0	0
0	0	0	0	0	0	0	0	0
1	0	0	0	0	0	0	0	0
0	1	0	0	0	0	0	0	0
0	0	0	1	0	0	0	0	0
0	0	0	0	0	0	0	0	0
0	0	0	0	0	0	0	0	0
0	0	0	0	0	0	0	0	0
0	0	0	0	0	0	0	0	0
1	0	0	0	0	0	0	0	0
1	0	0	0	0	0	0	0	0
0	0	0	0	0	0	0	0	0
0	0	0	0	0	0	0	0	0
0	0	0	0	0	0	0	0	0
0	1	0	0	0	0	0	0	0
0	0	1	0	0	0	0	0	0
1	0	0	0	0	0	0	0	0
0	0	0	0	0	0	0	0	0
1	0	0	0	0	0	0	0	0
1	0	0	0	0	0	0	0	0
0	0	0	0	0	0	0	0	0
0	0	0	0	0	0	0	0	0
0	0	0	0	0	0	0	0	0
0	0	0	0	0	0	0	0	0
0	0	0	0	0	0	0	0	0
0	0	0	0	0	0	0	0	0
0	0	0	0	0	0	0	0	0
0	0	0	0	0	0	0	0	0
0	0	0	0	0	0	0	0	0
0	0	0	0	0	0	0	0	0
0	0	0	0	0	0	0	0	0
0	0	0	0	0	0	0	0	0
0	0	0	0	0	0	0	0	0
0	0	0	0	0	0	0	0	0
0	0	0	0	0	0	0	0	0
0	0	0	0	0	0	0	0	0
0	0	0	0	0	0	0	0	0
0	0	0	0	0	0	0	0	0
0	0	0	0	0	0	0	0	0
0	0	0	0	0	0	0	0	0
0	0	0	0	0	0	0	0	0
0	0	0	0	0	0	0	0	0
0	0	0	0	0	0	0	0	0
0	0	0	0	0	0	0	0	0
0	0	0	0	0	0	0	0	0
0	0	0	0	0	0	0	0	0
0	0	0	0	0	0	0	0	0
0	1	0	0	0	0	0	0	0
0	0	0	0	0	0	0	0	0

0	1	0	0	0	0	0	0	0
0	0	0	0	0	0	0	0	0
0	0	0	0	0	0	0	0	0
0	0	0	0	0	0	0	0	0
0	0	0	0	0	0	0	0	0
0	0	0	0	0	0	0	0	0
0	0	0	0	0	0	0	0	0
0	0	0	0	0	0	0	0	0
0	0	0	0	0	0	0	0	0
0	0	0	0	0	0	0	0	0
0	0	0	0	0	0	0	0	0
0	0	0	0	0	0	0	0	0
0	0	0	0	0	0	0	0	0
0	0	0	0	0	0	0	0	0
0	0	0	0	0	0	0	0	0
0	0	0	0	0	0	0	0	0
0	0	0	0	0	0	0	0	0
0	0	0	0	0	0	0	0	0
0	0	0	0	0	0	0	1	0
0	0	0	0	0	0	0	0	0
0	0	0	0	0	0	0	0	0
0	0	0	0	0	0	0	0	0
0	0	0	0	0	0	0	0	0
0	0	0	0	0	0	0	1	1
0	0	0	0	0	0	0	0	0
0	0	0	0	0	0	0	0	0
0	0	0	0	0	0	0	0	0
0	0	0	0	0	0	0	0	0
0	0	0	0	0	0	0	0	0
0	0	0	0	0	0	0	0	0
0	0	0	0	0	0	0	0	0
0	0	0	0	0	0	0	0	0
0	0	0	0	0	0	0	0	0
0	0	0	0	0	0	0	0	0
0	0	0	0	0	0	0	0	0
0	0	0	0	0	0	0	0	0
0	0	0	0	0	0	0	0	0
0	0	0	0	0	0	0	0	0
0	0	0	0	0	0	0	0	0
0	0	0	0	0	0	0	0	1
0	0	0	0	0	0	0	0	0
0	0	0	0	0	0	0	0	0
0	0	0	0	0	0	0	0	0
0	0	0	0	0	0	0	0	0
0	0	0	0	0	0	0	0	0
0	0	0	0	0	0	0	0	0
0	0	0	0	0	0	0	1	0
0	0	0	0	0	0	0	1	0
0	0	0	0	0	0	0	0	0
0	0	0	0	1	0	0	0	0
0	0	0	0	0	0	0	0	0
0	0	0	0	0	0	0	0	0
0	0	0	0	0	0	0	0	0
0	0	0	0	0	0	0	0	0
0	0	0	0	0	0	0	0	0
0	0	0	0	0	0	0	0	0

0	0	0	0	0	0	0	0	0
0	0	0	0	0	0	0	0	0
0	0	0	0	0	0	0	0	0
0	1	0	0	0	0	0	0	0
0	0	0	0	0	0	0	0	0
0	0	0	0	0	0	0	0	0
0	0	0	0	0	0	0	0	0
0	1	0	0	0	0	0	0	0
0	0	0	0	0	0	0	0	0
0	1	0	0	0	0	0	1	0
0	0	0	0	0	0	0	0	0
0	0	0	0	0	0	0	0	0
0	0	0	0	0	0	0	0	0
0	0	0	0	0	0	0	0	0
0	0	0	0	0	0	0	0	1
0	0	0	0	0	0	0	0	0
0	0	0	0	0	0	0	0	0
0	0	0	0	0	0	0	0	0
0	0	0	0	0	0	0	0	0
0	0	0	0	0	0	0	0	0
0	0	0	0	0	0	0	0	0
0	0	0	0	0	0	0	0	0
0	0	0	0	0	0	0	0	0
0	0	0	0	0	0	0	0	0
0	0	0	0	0	0	0	1	0
0	1	0	0	0	0	0	0	1
0	0	0	0	0	0	0	0	0
0	0	0	0	0	0	0	0	0
0	0	0	0	0	0	0	0	0
0	0	0	0	0	0	0	0	0
0	0	0	0	0	0	0	0	0
0	0	0	0	0	0	0	0	0
0	0	0	0	0	0	0	0	0
0	0	0	0	0	0	0	0	0
0	0	0	0	0	0	0	0	0
0	0	0	0	0	0	0	0	0
0	0	0	1	0	0	0	0	0
0	0	0	0	0	0	0	0	0
0	0	0	0	0	0	0	0	0
0	0	0	0	0	0	0	0	0
0	0	0	0	0	0	0	0	0
0	0	0	0	0	0	0	0	0
0	1	0	0	0	0	0	0	0
0	0	0	0	0	0	0	0	0
0	0	0	0	0	0	0	0	0
0	0	0	0	0	0	0	0	0
0	0	0	0	0	0	0	0	0
0	0	0	0	0	0	0	1	0
0	0	0	0	0	0	0	0	0
0	0	0	0	0	0	0	0	0
0	0	0	0	0	0	0	0	0
0	0	0	0	0	0	0	0	0
0	1	0	0	0	0	0	1	0
0	0	0	0	0	0	0	0	0
0	0	0	0	0	0	0	0	0
0	0	0	0	0	0	0	0	0

0	0	0	0	0	0	0	0	0
0	0	0	0	0	0	0	0	0
0	0	0	0	0	0	0	0	0
0	0	0	0	0	0	0	0	0
0	0	0	0	0	0	0	0	0
0	0	0	0	0	0	0	0	0
0	0	0	0	0	0	0	0	0
0	0	0	0	0	0	0	0	0
0	0	0	0	0	0	0	0	0
0	0	0	0	0	0	0	0	0
0	0	0	0	0	0	0	0	0
0	0	0	0	0	0	0	0	0
0	0	0	0	0	0	0	0	0
0	0	0	0	0	0	0	0	0
0	0	0	0	0	0	0	0	0
0	0	0	0	0	0	0	0	0
0	0	0	0	0	0	0	0	0
0	0	0	0	0	0	0	0	0
0	0	0	0	0	0	0	0	0
0	0	0	0	0	0	0	0	0
0	0	0	0	0	0	0	0	0
0	0	0	0	0	0	0	0	0
0	0	0	0	0	0	0	0	0
0	0	0	0	0	0	0	0	0
0	0	0	0	0	0	0	0	0
1	0	0	0	0	0	0	0	0
0	0	0	0	1	0	0	0	0
0	0	1	0	0	0	0	0	0
0	0	0	0	0	0	0	0	0
0	0	0	0	0	0	0	1	0
0	0	0	0	0	0	0	0	0
0	0	0	0	0	0	0	0	0
0	0	0	0	0	0	0	0	0
0	1	0	0	0	0	0	0	0
0	0	0	0	0	0	0	0	0
0	0	0	0	0	0	0	0	0
0	0	0	0	0	0	0	0	0
0	0	0	0	0	0	0	0	0
0	0	0	0	0	0	0	0	0
0	0	0	0	0	0	0	0	0
0	0	0	0	0	0	0	0	0
0	0	0	0	0	0	0	0	0
0	0	0	0	0	0	0	0	0
0	0	0	0	0	0	0	0	0
0	0	0	0	0	0	0	0	0
0	0	0	0	0	0	0	0	0
0	0	0	0	0	0	0	0	0
0	0	0	0	0	0	0	0	0
0	1	0	0	0	0	0	0	0
0	0	0	0	1	0	0	0	0
0	0	0	0	0	0	0	0	0
0	0	0	0	0	0	0	0	0
0	0	0	0	0	0	0	0	0
1	0	0	0	0	0	0	0	0
0	0	0	0	0	0	0	1	0
0	0	0	0	0	0	0	0	0

0	0	0	0	0	0	0	0	0
0	1	0	0	0	0	0	0	0
0	0	0	0	0	0	0	0	0
0	0	0	0	0	0	0	0	0
0	0	0	0	0	0	0	1	0
0	0	0	0	0	0	0	0	0
0	0	0	0	0	0	0	0	0
0	0	0	0	0	0	0	0	0
0	0	0	0	0	0	0	0	0
0	0	0	0	0	0	0	0	0
1	0	0	0	0	0	0	0	0
0	0	0	0	0	0	0	0	0
0	0	0	0	0	0	0	0	0
0	0	0	0	0	0	1	0	0
0	0	0	0	0	0	0	0	0
0	0	0	0	0	0	0	0	1
0	0	0	0	0	0	0	0	0
0	0	0	0	0	0	0	0	0
1	0	0	0	0	0	0	1	0
0	0	0	0	0	0	0	0	0
0	0	0	0	0	0	0	1	0
0	0	0	0	0	0	0	0	0
0	0	0	0	1	0	0	0	0
0	0	0	0	0	0	0	0	0
0	1	0	0	0	0	0	1	0
1	0	0	0	0	0	0	0	0
0	0	0	0	0	0	0	0	0
0	0	0	0	0	0	0	0	0
0	1	0	0	0	0	0	0	0
0	0	0	0	0	0	0	0	0
0	0	0	0	0	0	0	0	0
0	1	1	0	0	0	0	1	0
0	0	0	1	1	0	0	1	0
0	0	0	0	0	0	0	0	0
1	0	0	0	1	0	0	1	0
0	0	0	0	0	0	0	0	0
1	0	0	0	0	0	0	0	0
0	0	0	0	1	0	0	0	0
0	0	0	0	0	0	0	0	0
0	0	0	0	0	0	0	1	0
1	0	0	0	0	0	0	0	0
0	0	0	0	0	0	0	0	0
0	0	0	0	1	0	0	0	0
0	0	0	0	0	0	0	0	0
0	0	0	0	0	0	0	0	0
0	0	0	0	0	0	0	0	0
0	0	0	0	0	0	0	1	0
0	0	0	0	0	0	0	0	0
0	0	0	0	0	0	0	0	0
0	0	0	0	0	0	0	0	0
0	0	0	0	0	0	0	0	0
0	0	0	0	0	0	0	0	0
1	0	0	0	1	0	0	0	0
1	0	0	0	0	0	0	0	0
1	0	0	0	0	0	1	1	0
0	0	0	0	0	0	0	0	0

0	0	0	0	0	0	0	0	0
0	0	0	1	0	0	0	0	0
0	0	0	0	0	0	0	0	0
0	0	0	0	0	0	0	0	0
0	0	0	0	0	0	0	0	0
0	0	0	0	0	0	0	0	0
0	0	0	0	0	0	0	0	0
0	0	0	0	1	0	0	0	0
0	0	1	0	0	0	0	0	0
0	0	0	0	0	0	0	0	0
0	0	0	0	0	0	0	0	0
0	0	0	0	0	0	0	0	0
0	0	0	0	0	0	0	0	0
0	0	0	0	0	0	0	0	0
0	0	0	0	0	0	0	0	1
0	0	0	0	0	0	0	0	0
0	0	0	0	0	0	0	0	0
0	0	0	0	0	0	0	0	0
1	0	0	0	1	0	0	0	0
0	0	0	0	0	0	0	0	0
0	0	0	0	0	0	0	0	0
0	0	0	0	0	0	0	0	0
0	0	0	0	0	0	0	1	0
0	0	0	0	0	0	0	0	0
0	0	0	0	1	0	0	0	0
0	0	0	0	0	0	0	0	0
0	0	0	0	0	0	0	0	0
0	0	0	0	0	0	1	0	0
1	0	0	0	0	0	0	0	0
0	0	0	0	0	0	0	0	0
0	0	0	0	0	0	0	0	0
0	0	0	0	0	0	0	0	0
0	0	0	0	0	0	0	0	0
0	0	0	0	0	0	0	0	0
0	0	0	0	1	1	0	0	0
0	0	0	0	0	0	0	0	0
1	0	0	0	0	0	0	0	0
0	0	0	0	0	0	0	0	0
1	0	0	0	1	0	0	0	0
0	0	0	0	0	0	0	0	0
0	0	0	0	0	0	1	0	0
0	0	0	0	0	0	0	0	0
0	0	0	0	0	0	0	0	0
0	0	0	0	0	0	0	0	0
0	0	0	1	0	0	0	0	0
0	0	0	0	0	0	0	0	0
0	0	0	0	0	0	0	0	0
0	0	0	0	0	0	0	0	0
0	0	0	0	0	0	0	0	0
0	1	0	0	0	0	0	0	0
0	0	0	0	0	0	0	0	0
0	0	0	0	0	0	0	0	0
0	0	0	0	0	0	0	1	0
0	0	0	0	0	0	0	0	0
0	0	0	0	0	0	0	0	0
1	0	0	0	0	0	0	1	0

0	0	0	0	0	0	0	0	0
0	0	0	0	0	0	0	0	0
1	0	0	0	0	0	0	0	0
0	0	0	0	0	0	0	0	0
0	0	0	0	0	0	0	0	0
0	0	0	0	0	0	0	0	0
1	0	0	0	0	0	0	0	0
0	0	0	0	0	0	0	0	0
0	0	0	0	0	0	0	1	0
0	0	0	0	0	0	0	0	0
0	0	0	0	0	0	0	0	0
0	0	0	0	0	0	0	0	0
1	0	0	0	0	0	0	0	0
1	0	0	0	0	0	0	0	0
0	0	0	0	0	0	0	0	0
1	0	0	0	0	0	0	0	0
0	0	0	0	0	0	0	0	0
0	1	0	0	0	0	0	0	0
1	0	0	0	0	0	0	0	0
0	0	0	0	0	0	0	0	0
0	0	0	0	0	0	0	0	0
0	0	0	0	0	0	0	0	0
1	0	0	0	0	0	0	0	0
0	1	0	0	0	0	0	0	0
0	0	0	0	0	0	0	0	0
0	0	0	0	0	0	0	0	0
0	0	0	0	0	0	0	0	0
1	0	0	0	0	0	0	0	0
0	0	0	0	0	0	0	0	0
0	0	0	0	0	0	0	0	0
1	1	0	0	0	0	0	0	0
0	0	0	0	0	0	0	0	0
0	1	0	0	0	0	0	0	0
0	0	0	0	0	0	0	0	0
0	0	0	0	0	0	0	0	0
1	0	0	0	0	0	0	0	0
1	0	0	0	0	0	0	0	0
1	0	0	0	0	0	0	0	0
1	0	0	0	0	0	0	0	0
1	0	0	0	1	0	0	0	0
0	0	0	0	0	0	0	1	0
0	0	0	0	0	0	0	1	0
0	0	0	1	0	0	0	0	0
0	0	0	0	0	0	0	0	0
0	0	0	0	0	0	0	0	0
0	0	0	0	0	0	0	0	0
0	0	0	0	0	0	0	0	0
1	0	0	0	0	0	0	0	0
0	0	0	0	0	0	0	0	0
0	0	0	0	0	0	0	0	0
0	0	0	0	0	0	0	0	0
0	0	0	0	0	0	0	1	0
1	0	0	0	0	0	0	0	0
0	0	0	0	0	0	0	0	0
0	0	0	0	0	0	0	0	0
0	0	0	0	0	0	0	0	0

1	0	0	0	0	0	0	1	0
0	0	0	0	0	0	0	0	0
0	0	0	0	0	0	0	0	0
0	0	0	0	0	0	0	0	0
0	0	0	0	0	0	0	0	0
0	0	0	0	0	0	0	0	0
0	0	0	0	0	0	0	0	0
1	0	0	0	0	0	1	0	0
0	0	0	0	0	0	0	0	0
0	0	0	0	0	0	0	0	0
0	0	0	0	0	0	0	0	0
1	0	0	0	0	0	0	0	0
0	0	0	0	0	0	0	0	0
0	0	0	0	0	0	0	0	0
0	0	0	0	0	0	0	0	0
0	0	0	0	0	0	0	0	0
0	0	0	0	0	0	0	0	0
1	0	0	0	0	0	0	0	0
0	0	0	0	0	0	0	0	0
0	0	0	0	0	0	0	0	0
0	0	0	0	0	0	0	1	0
0	0	0	1	0	0	0	1	0
0	0	0	0	0	0	0	0	0
1	1	0	0	0	0	0	1	0
0	0	0	0	1	0	0	1	0
0	0	0	0	0	0	0	0	0
0	1	0	0	0	0	0	0	0
0	0	0	0	0	0	0	0	0
0	0	0	0	0	0	0	0	0
0	0	0	0	0	0	0	0	0
0	0	0	0	0	0	0	0	0
0	0	0	0	0	0	0	1	0
0	0	0	0	0	0	0	0	0
0	0	0	0	0	0	0	0	0
1	0	0	0	0	0	0	0	0
0	0	0	0	0	0	0	0	0
0	0	0	0	0	0	0	0	0
0	0	0	0	0	0	0	1	0
0	0	0	0	0	0	0	0	0
1	0	0	0	0	0	0	0	0
0	0	0	0	0	0	0	0	0
0	0	0	0	0	0	0	0	0
0	0	0	0	0	0	0	0	0
0	0	0	0	0	0	0	0	0
0	0	0	0	0	0	0	0	0
1	0	0	0	0	0	0	0	0
0	0	0	0	0	0	0	0	0
0	0	0	0	0	0	0	0	0
0	0	0	0	0	0	0	1	0
0	0	0	0	0	0	0	0	0
0	0	0	0	0	0	0	0	0
0	0	0	0	0	0	0	0	0
0	0	0	0	0	0	0	0	0
0	0	0	0	0	0	0	0	0
0	1	0	0	0	0	0	0	0
0	0	0	0	0	0	0	0	0

0	0	0	0	0	0	1	0	0
1	0	0	0	0	0	0	0	0
1	0	0	0	0	0	0	0	0
1	0	0	0	0	0	0	0	0
0	0	0	0	0	0	0	0	0
0	1	0	0	0	0	0	0	0
0	0	0	0	0	0	0	1	1
0	0	0	0	0	0	0	0	0
1	0	0	0	0	0	0	0	0
0	0	0	0	0	1	0	0	0
0	0	0	0	0	0	0	0	0
1	0	0	0	0	0	0	0	0
0	0	0	0	0	0	0	0	0
0	0	0	0	0	0	0	0	0
0	0	0	0	0	0	0	0	0
0	0	0	0	0	0	1	0	0
0	0	0	0	0	0	0	0	0
1	0	0	0	0	0	0	0	0
0	0	0	0	0	0	0	0	0
0	0	0	0	0	0	0	0	0
0	0	0	1	0	0	0	0	0
0	0	0	0	0	0	0	0	0
0	0	0	0	0	0	0	0	0
1	0	0	0	0	0	0	0	0
0	0	0	0	0	0	0	0	0
0	0	0	0	0	0	0	0	0
1	0	0	0	1	0	0	0	0
1	0	0	0	0	0	0	0	0
0	0	0	0	0	0	0	0	0
0	0	0	0	0	0	0	0	0
1	0	0	0	0	0	0	0	0
1	0	0	0	0	0	0	0	0
0	1	0	0	0	0	0	0	0
0	0	0	0	0	0	0	0	0
0	0	0	0	0	0	0	0	0
0	1	0	0	0	0	0	0	0
0	0	0	0	0	0	0	0	0
0	1	0	0	0	0	0	0	0
0	0	0	0	0	0	0	0	0
1	0	0	0	0	0	0	0	0
0	0	0	0	0	0	0	0	0
0	1	0	0	0	0	0	0	0
0	0	0	0	0	0	0	0	0
0	0	0	0	0	0	0	0	0
0	0	0	0	0	0	0	0	0
0	0	0	0	0	0	0	0	0
0	0	0	0	0	0	0	0	0
1	0	0	0	0	0	1	0	0
1	0	0	0	0	0	0	0	0
0	0	0	0	0	0	0	0	0
0	0	0	0	0	0	0	1	0
0	0	0	0	0	0	0	0	0
0	0	0	0	0	0	0	0	0
0	0	0	0	0	0	0	0	0
0	0	0	0	0	0	0	0	0
0	1	0	0	0	0	0	0	0

0	0	0	0	0	0	0	0	0
1	0	0	0	0	0	0	0	0
0	0	0	0	0	0	0	0	0
0	0	0	0	0	0	0	0	0
0	0	0	0	0	0	0	0	0
0	0	0	0	0	0	0	0	0
0	0	0	0	0	0	0	0	0
0	0	0	0	0	0	0	0	0
0	0	0	0	0	0	0	0	0
0	0	0	0	0	0	0	0	0
0	0	0	0	0	0	0	0	0
0	0	0	0	0	0	0	0	0
0	0	0	0	0	0	0	0	0
0	0	0	0	0	0	0	0	0
0	0	0	0	0	0	0	0	0
1	0	0	0	0	0	0	0	0
1	1	0	0	0	0	0	0	0
0	0	0	0	0	0	0	0	0
1	0	0	0	0	0	0	0	0
1	0	0	0	0	0	0	0	0
0	0	0	0	0	0	0	0	0
0	0	0	0	0	0	0	0	0
0	0	0	0	0	0	0	1	0
0	0	0	0	0	0	0	1	0
1	0	0	0	0	0	0	0	0
1	0	0	0	0	0	0	0	0
0	0	0	0	0	0	0	0	0
0	0	0	0	0	0	0	0	0
1	0	0	0	0	0	0	1	0
0	0	0	0	0	0	0	0	0
0	0	0	0	0	0	0	0	0
0	0	0	0	0	0	0	0	0
0	0	0	0	0	0	0	0	0
0	0	0	0	0	0	0	0	0
1	0	0	0	0	0	0	0	0
1	0	0	0	0	0	0	0	0
0	0	0	0	0	0	0	0	0
1	0	0	0	0	0	0	0	0
0	0	0	0	0	0	0	0	0
0	0	0	0	0	0	0	0	0
0	0	0	0	0	0	0	0	0
1	0	0	0	0	0	0	0	0
0	0	0	0	0	0	0	0	0
1	0	0	0	0	0	0	0	0
1	0	0	0	0	0	0	0	0
0	0	0	0	0	0	0	0	0
0	0	0	0	0	0	1	0	0
0	0	0	0	0	0	0	0	0
0	0	0	0	0	0	0	0	0
1	0	0	0	0	0	0	1	0
0	1	0	0	0	0	0	0	0
1	0	0	0	1	0	0	0	0
0	0	0	0	0	0	0	0	0
0	0	0	0	0	0	0	0	0
1	0	0	0	0	0	0	0	0
0	1	0	0	0	0	0	0	0

0	0	0	0	0	0	0	0	0
1	1	0	0	0	0	0	0	0
0	0	0	0	0	0	0	0	0
1	1	0	0	0	0	0	0	0
1	0	0	0	0	0	0	0	0
1	0	0	0	0	0	0	0	0
1	0	0	0	0	0	0	0	0
1	0	0	0	0	0	0	0	0
1	0	0	0	0	0	0	0	0
0	0	0	0	0	0	0	0	0
0	1	0	0	0	0	1	0	0
0	0	0	0	0	0	0	0	0
0	0	0	0	0	0	0	0	0
0	0	0	0	0	0	0	0	0
0	0	0	0	0	0	0	0	0
1	0	0	0	0	0	0	0	0
0	0	0	0	0	0	0	0	0
1	0	0	0	0	0	0	0	0
0	0	0	0	0	0	0	1	0
0	0	0	0	0	0	0	0	0
1	0	0	0	0	0	0	0	0
0	0	0	0	0	0	0	1	0
0	0	0	0	0	0	0	0	0
1	1	0	0	0	0	0	0	1
0	0	0	0	0	0	0	0	0
0	0	0	0	0	0	0	0	0
0	0	0	0	0	0	0	0	0
0	0	0	0	0	0	0	1	0
0	1	0	0	0	0	0	0	0
0	0	0	0	0	0	0	0	0
0	0	0	1	0	0	0	0	0
0	0	0	0	0	0	0	0	0
0	0	0	0	0	0	0	0	0
1	0	0	0	0	0	0	0	0
0	0	0	0	0	0	0	0	0
0	0	0	0	0	0	0	0	0
0	0	0	0	0	0	0	0	0
0	0	0	0	0	0	0	0	0
0	0	0	0	0	0	0	0	0
1	0	0	0	0	0	0	0	0
0	0	0	0	0	0	0	0	0
0	0	0	0	0	0	0	0	0
0	0	0	0	0	0	0	0	0
1	0	0	0	0	0	1	0	0
1	0	0	0	1	0	0	0	0
0	0	0	0	0	0	0	0	0
1	0	0	0	0	0	1	0	0
1	0	0	0	0	0	0	0	0
0	0	0	0	0	0	0	0	0
0	0	0	0	0	0	0	0	0
0	0	0	0	0	0	0	0	0
0	0	0	0	0	0	0	0	0
0	0	0	0	0	0	0	0	0
0	0	0	0	0	0	0	0	0
0	0	0	0	0	0	0	0	0
0	0	0	0	0	0	0	0	0

0	0	0	0	0	0	0	0	0
1	0	0	0	0	0	0	0	0
0	1	0	1	0	0	1	0	0
1	0	0	0	0	0	0	0	0
1	0	0	0	0	0	0	0	0
1	0	0	0	0	0	0	1	0
1	0	0	0	0	0	0	0	0
1	0	0	0	0	0	0	1	0
0	1	0	0	0	0	0	0	0
0	0	0	0	0	0	0	0	0
0	0	0	0	0	0	0	1	0
0	0	0	0	0	0	0	1	0
0	1	0	0	0	0	0	1	0
0	1	0	0	0	0	0	0	0
0	0	0	0	0	0	0	0	0
0	0	0	0	0	0	0	0	0
0	0	0	0	0	0	1	0	0
0	1	0	0	0	0	0	0	0
0	1	0	0	0	0	0	1	0
1	0	0	0	0	0	0	0	0
0	0	0	0	0	0	0	0	0
0	0	0	0	0	0	0	1	0
0	0	0	0	0	0	0	1	0
0	1	0	0	0	0	0	0	0
0	1	0	0	0	0	0	0	0
0	0	0	0	0	0	0	0	0
0	0	0	0	0	0	0	0	0
0	0	0	0	0	0	0	0	0
0	0	0	0	0	0	0	0	0
1	0	0	0	0	0	0	0	0
0	1	0	0	0	0	0	0	0
1	0	0	0	0	0	0	0	0
0	0	0	0	0	0	0	0	0
1	0	0	0	0	0	0	1	0
1	0	0	0	0	0	0	0	0
0	0	0	0	0	0	0	0	0
0	0	0	0	0	0	0	0	0
1	0	0	0	0	0	0	0	0
1	0	0	0	0	0	0	0	0
0	0	0	0	0	0	0	0	0
0	0	0	0	0	0	0	0	0
0	0	0	0	0	0	1	0	0
1	0	0	0	0	0	0	0	0
0	0	0	0	0	0	0	0	0
0	0	0	0	0	0	0	0	0
0	1	0	0	0	0	0	0	0
0	0	0	0	0	0	0	1	0
0	0	0	0	0	0	0	0	0
0	0	0	0	0	0	0	0	0
0	0	0	0	0	0	0	0	0
0	0	0	0	0	0	0	0	0
0	0	0	0	0	0	1	1	0
0	0	0	0	0	0	0	0	0
1	0	0	0	1	0	0	0	0
0	0	0	0	0	0	0	0	0
1	0	0	0	0	0	0	0	0

0	0	0	0	0	0	0	0	0
0	0	0	0	0	0	0	0	0
0	0	0	0	0	0	0	0	0
0	0	0	0	0	0	0	0	0
0	0	0	0	0	0	0	0	0
0	0	0	0	0	0	0	0	0
1	0	0	0	1	0	0	0	0
0	0	0	0	0	0	0	0	0
0	0	0	0	0	0	0	0	0
1	0	0	0	0	0	0	0	0
0	0	0	0	0	0	0	0	0
1	0	0	0	0	0	0	0	0
1	0	0	0	0	0	1	1	0
0	0	0	0	0	0	0	0	0
0	0	0	0	0	0	1	0	0
0	0	0	0	0	0	0	0	0
0	0	0	0	0	0	0	0	0
0	0	0	0	0	0	0	1	1
0	0	0	0	0	0	0	0	0
0	0	0	0	0	0	0	0	0
0	0	0	0	0	0	0	0	0
0	0	0	0	0	0	0	0	0
1	1	0	0	0	0	0	0	0
1	0	0	0	0	0	0	0	0
1	0	0	0	0	0	0	0	0
0	0	0	0	0	0	0	0	0
0	0	0	0	0	0	0	0	0
0	0	0	0	0	0	0	1	0
0	0	0	0	0	0	1	0	0
0	0	0	0	0	0	0	0	0
0	0	0	0	1	0	0	0	0
1	0	0	0	0	0	0	1	0
1	1	0	0	0	0	0	0	0
0	0	0	0	0	0	0	0	0
0	0	0	0	0	0	0	0	0
0	0	0	0	0	0	0	1	0
0	0	0	0	0	0	0	0	0
0	0	0	0	0	0	0	0	0
0	1	0	0	0	0	0	0	0
0	0	0	0	0	0	0	0	0
0	0	0	0	0	0	0	0	0
0	0	0	0	0	0	0	0	0
0	0	0	0	0	0	0	0	0
1	0	0	0	0	0	0	0	0
0	0	0	0	0	0	0	0	0
0	0	0	0	0	0	0	0	0
0	1	0	0	0	0	0	0	0
0	0	0	0	0	0	0	0	0
0	0	0	0	0	0	0	0	0
1	0	0	0	0	0	0	0	0
0	0	0	0	0	0	0	0	0
0	0	0	0	0	0	0	0	0
0	0	0	0	0	0	0	0	0
0	0	0	0	0	0	0	0	0
0	1	0	0	0	0	0	0	0
0	0	0	0	0	0	0	0	0

1	0	0	0	0	0	0	0	0
0	0	0	0	0	0	0	0	0
0	0	0	0	0	0	0	0	0
1	0	0	0	0	0	0	0	0
1	0	0	0	0	0	0	0	0
1	0	0	0	0	0	0	0	0
0	0	0	0	0	0	0	0	0
0	0	0	0	0	0	0	0	0
0	0	0	0	0	0	0	0	0
0	1	0	0	0	0	0	0	0
0	0	0	0	0	0	0	1	0
0	0	0	0	0	0	0	0	0
0	0	0	0	0	0	0	0	0
0	0	0	0	0	0	0	1	0
0	0	0	0	0	0	0	1	0
0	0	0	0	0	0	0	0	0
0	0	0	0	0	0	0	0	0
0	0	0	0	0	0	0	0	0
1	0	0	0	0	0	0	0	0
0	0	0	0	0	0	0	0	0
0	0	0	0	0	0	0	0	0
0	0	0	0	0	0	0	0	0
0	0	0	0	0	0	0	0	0
1	0	0	0	0	0	0	0	0
0	0	0	0	0	0	0	1	0
0	0	0	0	0	0	0	0	0
0	0	0	0	0	0	0	1	0
0	0	0	0	0	0	0	1	0
0	0	0	0	0	0	0	0	0
0	0	0	0	0	0	0	0	0
0	0	0	0	0	0	0	0	0
0	1	0	0	0	0	0	0	0
1	0	0	0	0	0	0	0	0
0	1	0	0	0	0	0	0	0
0	0	0	0	0	0	0	0	0
0	0	0	0	0	0	0	0	0
0	0	0	0	0	0	0	0	0
0	0	0	0	0	0	0	0	0
0	0	0	0	0	0	0	0	0
0	0	0	0	0	0	0	0	0
0	0	0	0	0	0	0	1	1
0	0	0	0	0	0	0	0	0
0	0	0	0	0	0	1	0	0
1	0	0	0	1	1	0	0	0
0	0	0	0	0	0	0	1	1
0	0	0	0	0	0	0	0	0
0	0	0	0	0	0	0	0	0
0	0	0	0	0	0	1	0	0
1	0	0	0	0	0	0	0	0
0	0	0	0	0	0	0	1	0
0	0	0	0	0	0	0	1	1
1	0	0	1	0	0	0	0	0
0	0	0	0	0	0	0	0	0
0	0	0	0	0	0	0	0	0
0	0	0	0	0	0	0	0	0
0	0	0	0	0	0	0	0	0

0	0	0	0	0	0	1	0	0
0	0	0	0	0	0	0	0	0
0	0	0	0	0	0	0	0	0
0	0	0	0	0	0	0	0	1
0	0	0	0	0	0	0	0	0
0	0	0	0	0	0	1	0	0
1	0	0	0	0	0	0	1	0
0	0	0	0	0	0	0	0	0
1	0	0	0	0	0	0	0	0
0	1	0	0	0	0	0	0	0
0	0	0	0	0	0	0	0	0
0	0	0	0	0	0	0	1	0
1	0	0	0	0	0	0	0	0
0	0	0	0	0	0	0	0	0
0	0	0	0	0	0	0	0	0
0	0	0	0	0	0	0	1	0
0	0	0	0	0	0	0	0	0
0	0	0	0	0	0	0	0	0
0	0	1	0	0	0	0	1	0
0	0	0	0	0	0	0	0	0
0	0	0	0	0	0	0	0	0
1	0	0	0	0	0	0	0	0
0	0	0	0	0	0	0	1	0
0	0	0	0	0	0	0	0	0
0	0	0	0	0	0	0	0	0
0	0	0	0	0	0	0	0	0
0	0	0	0	0	0	0	0	0
1	0	0	0	0	0	0	0	0
0	0	0	0	0	0	0	0	0
0	0	0	0	0	0	0	0	0
0	0	0	0	0	0	0	0	0
0	0	0	0	0	0	0	0	0
0	0	0	0	0	0	0	0	0
0	0	0	0	1	0	0	0	0
0	0	0	0	0	0	0	0	0
0	0	0	0	0	0	0	1	0
1	0	0	0	0	0	0	0	0
0	0	0	0	0	0	0	0	0
0	0	0	0	0	0	0	0	0
0	0	0	0	0	0	0	0	0
0	0	0	0	0	0	0	0	0
1	0	0	0	0	0	0	1	0
1	0	0	0	0	0	0	0	0
0	0	0	0	0	0	0	0	0
1	0	0	0	0	0	0	0	0
0	0	0	0	0	0	0	0	0
0	1	0	0	0	0	0	0	0
0	1	0	0	0	0	0	0	0
0	0	0	0	0	0	0	1	0
0	0	0	0	1	0	1	0	0
0	0	0	1	0	0	0	0	0
1	0	0	0	0	0	0	0	0
0	1	0	0	0	0	0	0	0
0	1	0	0	0	0	0	1	0
0	0	0	0	0	0	0	0	0
0	0	0	0	0	0	0	0	0

0	0	0	0	0	0	0	0	0
0	0	0	0	0	0	0	0	0
0	0	0	0	0	0	0	0	0
0	1	0	0	0	0	0	0	0
1	0	0	0	0	0	0	0	0
0	0	0	0	0	0	0	0	0
0	0	0	0	0	0	0	0	0
0	0	0	0	0	0	0	0	0
0	0	0	0	0	0	0	0	0
0	0	0	0	1	0	0	1	0
1	0	0	0	1	0	0	0	0
0	0	0	0	0	0	0	0	0
0	0	0	0	0	0	0	0	0
0	0	0	0	0	0	0	0	0
0	0	0	0	0	0	0	0	0
0	0	0	0	0	0	0	0	0
0	0	0	0	0	0	0	0	0
0	1	0	0	0	0	0	0	0
0	0	0	0	0	0	0	0	0
0	0	0	0	0	0	1	0	0
0	0	0	0	1	0	0	0	0
0	0	0	0	0	0	0	0	0
0	1	0	0	0	0	0	0	0
0	0	0	0	0	0	0	0	0
0	0	0	0	0	0	0	0	0
1	0	0	0	0	0	0	1	0
0	0	0	0	0	0	0	0	0
0	0	0	0	0	0	0	0	0
0	0	0	0	0	0	0	0	0
0	1	0	0	0	0	1	0	0
0	0	0	0	0	0	0	0	0
0	0	0	0	0	0	0	0	0
0	0	0	0	0	0	0	0	0
0	0	0	0	0	0	0	0	0
0	0	0	0	0	0	0	0	0
0	0	0	0	0	0	0	1	0
0	0	0	0	0	0	0	0	0
0	0	0	0	0	0	0	0	0
0	0	0	0	0	0	0	0	0
0	0	0	0	0	0	0	0	0
0	0	0	0	0	0	0	0	1
0	0	0	0	0	0	1	0	0
0	0	0	0	0	0	1	1	0
0	1	0	0	0	0	0	0	0
0	1	0	0	1	0	0	0	0
0	0	0	0	0	0	1	0	0
0	0	0	0	0	0	0	1	0
0	0	0	0	0	0	0	0	0
0	0	0	0	0	0	0	0	0
1	0	0	0	0	0	0	0	0
0	0	0	0	1	0	0	0	0
0	0	0	0	0	0	0	1	0
0	0	0	0	0	0	0	1	0
0	1	0	0	0	0	0	0	0
0	0	0	0	0	0	0	0	0
0	0	0	0	0	0	0	0	0

0	0	0	0	0	0	0	0	0
0	0	0	0	0	0	0	0	0
1	0	0	0	0	0	0	0	0
0	0	0	0	0	0	0	0	0
0	0	0	0	0	0	0	0	0
0	0	0	0	0	0	0	0	0
0	0	0	0	0	0	0	1	0
0	0	0	0	0	0	1	1	0
1	0	0	0	0	0	0	0	0
0	0	0	0	1	0	0	1	0
0	0	0	0	0	0	0	0	0
0	0	0	0	0	0	0	0	0
0	0	0	0	0	0	0	1	0
0	0	0	0	0	0	0	0	0
0	0	0	0	0	0	0	0	0
0	0	0	0	0	0	0	0	0
0	0	0	0	0	0	0	0	0
0	0	0	0	0	0	0	0	0
0	0	0	0	0	0	0	0	1
0	0	0	0	0	0	0	0	0
0	1	1	0	0	0	0	0	0
0	1	0	0	0	0	0	0	0
0	0	0	0	0	0	0	0	0
0	0	0	0	0	0	0	0	0
0	0	0	0	0	0	0	0	0
0	0	0	0	0	0	0	0	0
0	0	0	0	1	0	0	0	0
0	0	0	0	0	0	0	1	0
0	0	0	0	0	0	0	0	0
0	0	0	0	0	0	0	0	0
0	0	0	0	0	0	0	0	0
0	0	0	0	0	0	0	0	0
0	0	0	0	0	0	0	0	0
0	0	0	0	0	0	0	0	0
0	0	0	0	0	1	0	0	0
0	0	0	0	1	0	0	0	0
0	0	0	0	0	0	0	0	0
1	0	0	0	0	0	0	0	0
0	0	0	0	0	0	0	0	0
0	0	0	0	0	0	0	0	0
0	0	0	0	0	0	0	0	0
0	0	0	0	0	0	0	0	0
0	0	0	0	0	0	0	0	0
0	0	0	0	0	0	0	0	0
0	0	0	0	0	0	0	0	0
0	1	0	0	0	0	0	0	0
0	0	0	0	0	0	0	0	0
0	0	0	0	1	0	0	0	0
0	0	0	0	0	0	0	0	0
0	0	0	0	0	0	0	0	0
0	0	0	0	0	0	0	0	0
0	0	0	0	0	0	0	0	0
1	0	0	0	0	0	0	0	0
1	0	0	0	0	0	0	0	0
0	0	0	0	0	0	0	0	0
0	0	0	0	0	0	0	0	0

0	0	0	0	0	0	0	0	0
0	0	0	0	0	0	0	0	0
0	0	0	0	1	0	0	0	0
0	0	0	0	0	0	0	1	0
0	0	0	0	0	0	0	0	0
0	0	0	0	0	0	0	0	0
0	0	0	0	0	0	0	0	0
0	0	0	0	0	0	0	0	0
0	0	0	0	1	0	1	0	0
0	0	0	0	0	0	0	0	0
1	0	0	0	0	0	0	0	0
0	0	0	0	0	0	0	0	0
0	0	0	0	1	0	0	0	0
0	0	0	0	0	0	0	0	0
0	0	0	0	1	0	0	1	0
0	0	0	0	0	0	0	0	0
0	0	0	0	0	0	0	0	0
0	0	0	0	1	0	0	0	0
1	0	0	0	0	0	0	0	0
0	0	0	0	0	0	0	0	0
0	0	0	0	0	0	0	0	0
0	0	0	0	1	0	0	1	0
0	0	0	0	0	0	0	1	0
0	0	0	0	0	0	0	0	0
0	0	0	0	0	0	0	0	0
0	0	0	0	0	0	0	0	0
0	0	0	0	0	0	0	1	0
0	0	0	0	0	0	0	0	0
0	1	0	0	0	0	0	0	0
0	0	0	0	0	0	0	0	0
0	0	0	0	1	0	0	0	0
0	0	0	0	0	0	0	0	0
0	0	0	0	0	0	1	0	0
0	0	0	0	0	0	0	0	0
1	0	0	0	0	0	1	0	0
0	0	0	0	0	0	0	0	0
0	0	0	0	0	0	0	0	0
0	1	0	0	0	0	0	0	0
0	0	0	0	0	0	0	0	0
0	0	0	0	0	0	0	0	0
0	0	0	0	0	0	0	0	0
1	0	0	0	0	0	0	0	0
0	0	0	0	0	0	0	0	0
0	0	0	0	0	0	0	0	0
0	0	0	0	0	0	0	0	0
0	0	0	0	0	0	0	0	0
0	0	0	0	0	0	0	0	0
0	0	0	0	0	0	0	1	0
0	0	0	0	0	0	0	0	0
1	0	0	0	0	0	0	1	0
0	0	0	0	1	0	0	0	0
0	0	0	0	0	0	0	0	0
0	0	0	0	0	0	0	1	0
0	0	0	0	0	0	0	0	0
0	0	0	0	0	0	0	0	0
0	0	0	0	0	0	0	0	0

0	0	0	0	0	0	0	0	0
0	0	0	0	0	0	0	1	0
0	0	0	1	0	0	0	0	0
1	0	0	0	0	0	0	0	0
0	0	0	0	0	0	0	0	0
0	1	0	0	0	0	0	0	0
0	0	0	0	0	0	0	0	0
1	0	0	0	0	0	0	0	0
0	0	0	0	0	0	0	0	0
1	0	0	0	0	0	0	0	0
0	1	0	0	0	0	0	1	0
0	0	0	0	0	0	0	0	0
0	0	0	0	0	0	0	0	0
0	0	0	0	0	0	0	1	0
0	0	0	0	0	0	0	0	0
0	0	0	0	0	0	0	0	0
1	0	0	1	0	0	0	0	0
0	0	0	0	0	0	0	0	0
0	0	0	0	0	0	0	0	0
0	0	0	0	0	0	0	0	0
0	0	0	0	0	0	0	0	0
0	0	0	0	0	0	0	0	0
0	0	0	0	0	0	0	0	0
0	0	0	0	0	0	0	0	0
0	0	0	0	0	0	0	0	0
0	0	0	0	0	0	0	0	0
0	0	0	0	0	0	0	0	0
0	0	0	0	0	0	0	0	0
0	0	0	0	0	0	0	0	0
0	0	0	0	0	0	0	0	0
1	0	0	0	0	0	0	0	0
0	0	0	0	0	0	0	0	0
1	0	0	0	0	0	0	1	0
0	0	0	0	0	0	0	0	0
0	0	0	0	0	0	0	0	0
1	0	0	0	0	0	0	0	0
0	0	0	0	0	0	0	0	0
1	1	0	0	0	0	0	0	0
1	0	0	0	0	0	0	0	0
1	0	0	0	0	0	0	1	0
1	0	0	0	0	0	0	0	0
0	0	0	0	0	0	0	0	0
0	0	0	0	0	0	0	0	0
0	0	0	0	0	0	0	0	0
1	0	0	1	0	0	0	0	0
0	0	0	0	0	0	0	0	0
0	0	0	0	0	0	0	0	0
0	1	0	0	0	0	0	0	0
0	0	0	0	0	0	0	0	0
0	0	0	0	0	0	0	0	0
0	0	0	0	0	0	0	0	0
1	0	0	0	0	0	0	0	0
0	0	0	0	0	0	0	0	0
0	0	0	1	0	0	0	1	0
0	0	0	0	0	0	0	0	0
0	0	0	0	0	0	0	0	0

0	0	0	0	0	0	0	0	0
0	0	0	0	0	0	0	1	0
1	0	0	0	1	0	0	0	0
1	0	0	0	1	0	0	0	0
0	0	0	0	0	0	0	1	0
0	0	0	0	0	0	0	0	0
0	0	0	0	1	0	0	0	0
0	0	0	0	0	0	0	0	0
0	0	0	0	0	0	0	0	0
0	0	0	0	0	0	0	0	0
0	0	0	0	0	0	0	0	0
1	0	0	0	0	0	0	0	0
0	0	0	0	1	0	0	0	0
0	0	0	0	0	0	0	0	0
0	0	0	0	0	0	0	0	0
0	0	0	0	0	0	0	0	0
1	0	0	0	0	0	0	0	0
0	0	0	0	0	0	0	0	0
0	0	0	0	0	0	0	0	0
0	0	0	0	0	0	0	0	0
0	0	0	0	0	0	0	0	0
0	0	0	0	0	0	0	0	0
1	0	1	0	0	0	0	0	0
1	0	0	0	0	0	0	0	0
0	0	0	0	0	0	0	0	0
1	0	0	0	0	0	0	0	0
1	0	0	0	0	0	0	1	0
0	0	0	0	0	0	0	0	0
1	0	0	0	0	0	0	0	0
0	0	0	0	0	0	0	0	0
1	0	0	0	0	0	0	0	0
0	0	0	0	0	0	0	0	0
0	0	0	0	0	0	0	1	0
0	0	0	0	0	0	0	0	0
1	0	0	0	0	0	0	0	0
1	0	0	0	0	0	0	0	0
1	0	0	0	0	0	0	0	0
0	0	0	0	0	0	0	0	0
0	0	0	0	0	0	0	0	0
1	0	0	0	0	0	0	1	0
0	0	0	0	0	0	0	0	0
0	0	0	0	0	0	0	0	0
0	0	0	0	0	0	0	0	0
0	1	0	0	0	0	0	0	0
0	0	0	0	0	0	0	0	0
1	0	0	0	0	0	0	0	0
0	0	0	0	0	0	0	0	0
0	0	0	0	0	0	0	1	0
0	0	0	0	0	0	0	0	0
1	0	0	0	0	0	0	0	0
0	0	0	0	0	0	0	0	0
1	0	0	0	0	0	0	0	0
0	0	0	0	0	0	0	0	0
0	0	0	0	0	0	0	0	0
0	1	0	0	0	0	0	0	0
0	0	0	0	0	0	0	0	0

0	0	0	0	1	0	0	0	0
0	0	0	0	0	0	0	0	0
0	0	0	0	0	0	0	0	0
0	0	0	0	0	0	0	0	0
0	0	0	0	0	0	0	0	0
0	0	0	0	0	0	0	0	0
0	0	0	0	0	0	0	0	0
0	0	0	0	0	0	0	0	0
0	0	0	0	1	0	0	0	0
0	1	0	0	0	0	0	0	0
0	0	0	0	0	0	0	1	0
0	0	0	0	0	0	0	0	0
0	0	0	0	0	0	0	0	0
0	1	0	0	0	0	0	0	0
0	0	0	0	0	0	0	0	0
0	0	0	0	0	0	0	0	0
0	1	1	0	0	0	0	0	0
1	0	0	0	0	0	0	0	0
0	0	0	0	1	0	0	0	0
0	0	0	0	0	0	0	0	0
0	0	0	0	0	0	0	0	0
0	0	1	0	0	0	0	0	0
0	0	0	0	0	0	0	1	0
0	0	0	0	0	0	0	0	0
0	0	0	0	0	0	0	0	0
0	0	0	0	0	0	0	0	0
0	0	0	0	0	0	0	0	0
0	0	0	0	0	0	0	0	0
0	0	0	0	0	0	0	0	0
0	0	0	0	0	0	0	0	0
0	0	0	0	0	0	0	0	0
0	0	0	0	0	0	0	0	0
0	0	0	0	0	0	0	0	0
0	0	0	0	0	0	0	0	0
1	0	0	0	0	0	0	0	0
0	0	0	0	0	0	0	0	0
0	0	0	0	0	0	0	1	0
0	0	0	0	0	0	0	0	0
0	0	0	0	0	0	0	0	0
0	0	0	0	0	0	0	0	0
0	0	0	0	0	0	0	0	0
1	0	0	0	0	0	0	0	0
0	0	0	0	0	0	0	0	0
0	0	0	0	0	0	0	0	0
0	0	0	0	0	0	0	0	0
0	0	0	0	1	0	0	0	0
0	0	0	0	0	0	0	0	0
0	0	0	0	0	0	0	0	0
1	1	0	0	0	0	0	0	0
0	0	0	0	0	0	0	0	0
0	0	0	0	0	0	0	0	0
0	0	0	0	1	0	0	1	0
0	0	0	0	0	0	0	0	0
0	0	0	0	0	0	0	0	0
0	1	0	0	0	0	0	0	0
0	0	0	0	1	0	0	0	0

0	0	0	0	0	0	0	0	0
0	0	0	0	0	0	0	0	0
0	0	0	0	0	0	0	0	0
0	0	0	0	0	0	0	0	0
0	0	0	0	0	0	0	1	0
0	0	0	0	1	0	0	1	0
0	0	0	0	0	0	0	0	0
0	0	0	0	0	0	0	1	0
1	0	0	0	0	0	0	0	0
0	0	0	0	0	0	0	0	0
0	0	0	0	0	0	0	0	0
0	0	0	0	0	0	0	0	0
0	0	0	0	0	0	0	0	0
1	0	0	0	0	0	0	0	0
0	0	0	0	0	0	0	0	0
1	0	0	0	0	0	0	0	0
0	0	0	0	0	0	0	0	0
0	0	0	0	0	0	0	0	0
0	0	0	0	0	0	0	0	0
0	0	0	0	0	0	0	0	0
0	0	0	0	1	0	0	0	0
1	0	0	0	0	0	0	0	0
0	0	0	0	0	0	1	0	0
0	0	0	0	0	0	0	0	0
0	0	0	0	0	0	0	0	0
0	0	0	0	0	0	0	0	0
1	0	0	0	0	0	0	0	0
0	1	0	0	0	0	0	0	0
0	1	0	0	0	0	0	0	0
0	0	0	0	0	0	0	0	0
0	0	0	0	0	0	0	0	0
0	0	0	0	0	0	0	0	0
0	0	0	0	0	0	0	0	0
0	0	0	0	0	0	0	0	0
0	0	0	0	0	0	1	0	0
1	0	0	0	0	0	0	0	0
0	0	0	0	0	0	0	0	0
0	0	0	1	0	0	0	0	0
0	0	0	0	0	0	0	0	0
0	0	0	1	0	0	0	0	0
0	0	0	0	0	0	0	0	0
0	0	0	0	0	0	0	0	0
0	0	0	0	0	0	0	0	0
1	1	0	0	0	0	1	0	0
0	0	0	0	0	0	0	0	0
0	0	0	0	0	0	0	0	0
0	0	0	0	1	0	0	0	0
0	0	0	0	0	0	0	0	0
0	1	0	0	0	0	0	0	0
0	0	0	0	0	0	0	1	0
1	1	0	0	0	0	0	1	0
0	0	0	0	1	0	0	0	0
0	0	0	0	0	0	0	0	0
0	0	0	0	0	0	0	0	0
0	0	1	0	0	0	0	0	0
0	0	0	0	0	0	0	0	0

0	1	0	0	0	0	0	0	0
0	0	0	0	0	0	0	0	0
0	0	0	0	0	0	0	0	0
0	0	0	0	0	0	0	0	0
0	0	0	0	0	0	0	0	0
0	0	0	0	0	0	0	0	0
0	1	0	0	0	0	0	0	0
0	0	0	0	0	0	0	0	0
0	0	0	0	0	0	0	0	0
0	0	0	0	0	0	0	0	1
0	0	0	0	0	0	0	0	0
0	0	0	0	0	0	0	0	0
1	0	0	0	0	0	0	0	0
0	1	0	0	0	0	0	0	0
0	0	0	0	0	0	0	0	0
0	0	0	0	0	0	0	0	0
0	0	0	0	0	0	0	0	0
0	0	0	0	0	0	0	0	0
1	1	0	0	0	0	0	0	0
0	0	0	0	0	0	1	0	0
0	0	0	0	0	0	1	1	0
0	0	0	0	0	0	0	0	0
0	0	0	0	0	0	0	0	0
0	0	0	0	0	0	0	0	0
0	0	0	0	1	1	0	0	0
0	0	0	0	0	0	0	0	0
0	0	0	0	0	0	0	0	0
0	0	0	0	1	0	0	0	0
0	0	0	0	0	0	0	0	0
1	0	1	0	0	0	0	0	0
0	0	0	0	0	0	0	0	0
0	0	0	0	0	0	0	0	0
0	0	0	0	1	0	0	0	0
0	0	0	0	0	0	0	0	0
0	0	0	0	0	0	0	0	0
0	0	0	0	0	0	0	0	0
0	0	0	0	0	0	0	0	0
1	0	0	0	0	0	0	0	0
0	0	0	0	0	0	0	0	0
0	0	0	0	1	0	0	1	0
0	0	0	0	0	0	0	0	0
0	0	0	0	0	0	0	0	0
0	0	0	0	0	0	0	0	0
0	0	0	1	0	0	0	0	1
0	0	0	0	0	0	0	0	0
0	0	0	0	0	0	0	0	0
0	0	0	0	0	0	0	0	0
0	0	0	0	0	0	0	0	0
0	0	0	0	0	0	0	0	0
0	0	0	0	0	0	0	0	0
0	0	0	0	0	0	0	0	0
0	0	0	0	0	0	0	0	0
0	0	0	0	0	0	0	0	0
0	0	0	0	1	0	0	0	0
0	0	0	0	0	0	0	1	0
0	0	0	0	0	0	0	0	0

0	0	0	0	0	0	0	0	0
0	0	0	0	0	0	0	0	0
0	0	0	0	0	0	0	0	0
0	0	0	0	0	0	0	0	0
1	0	0	0	0	0	0	1	0
1	0	0	0	0	0	1	0	0
0	0	0	0	0	0	0	0	0
0	0	0	0	0	0	0	0	0
0	0	0	0	1	0	0	0	0
1	0	0	0	1	0	0	0	0
0	0	0	0	0	0	0	0	0
0	0	0	0	0	0	0	0	0
0	0	0	0	0	0	0	0	0
0	0	0	0	0	0	0	0	0
0	1	0	0	0	0	0	0	0
0	0	0	0	0	0	0	0	0
0	0	0	0	0	0	0	0	0
0	1	0	1	0	0	0	0	0
0	0	0	0	0	0	0	0	0
1	0	0	0	0	0	0	0	0
0	0	0	0	0	0	0	0	0
0	0	0	0	0	0	0	1	0
0	0	0	0	0	0	0	0	0
0	0	0	0	0	0	0	0	0
1	0	0	0	1	0	0	0	0
0	0	0	0	0	0	0	0	0
0	0	0	0	0	0	0	0	0
0	1	0	1	0	0	0	0	0
0	0	0	0	0	0	0	0	0
0	0	0	0	0	0	0	0	0
1	0	0	0	0	0	0	0	0
0	0	0	0	0	0	0	0	0
0	0	0	0	0	0	0	0	0
0	0	0	0	0	0	0	0	0
0	0	0	0	0	0	0	0	0
0	0	0	0	0	0	0	0	0
0	0	0	0	0	0	0	0	0
1	0	0	0	0	0	0	1	0
0	0	0	0	0	0	0	0	0
0	0	0	0	0	0	0	0	1
0	0	0	0	0	0	0	0	0
0	0	0	0	1	0	0	0	0
0	0	0	0	0	0	0	0	0
0	0	0	0	0	0	0	0	0
0	0	0	0	0	0	0	0	0
0	0	0	0	0	0	0	0	0
0	0	0	0	0	0	0	0	0
0	0	0	0	0	0	0	0	0
0	0	0	0	0	0	0	0	0
0	0	0	0	0	0	0	0	0
1	0	0	0	0	0	0	0	0
0	0	0	1	0	0	0	0	0
1	0	0	0	0	0	0	0	0
0	1	0	0	0	0	0	0	0
0	0	0	0	0	0	0	0	0
0	0	0	0	0	0	1	0	0

0	0	0	0	0	0	0	0	1
0	0	0	0	0	1	0	1	0
1	0	0	0	0	0	0	0	0
0	0	0	0	1	0	0	0	0
0	0	0	0	0	0	0	0	0
0	1	0	0	0	0	0	0	0
0	0	0	0	0	0	0	0	0
1	0	0	0	0	0	0	0	0
0	0	0	0	0	0	0	0	0
0	0	0	0	0	0	0	0	0
0	0	0	0	0	0	0	0	0
0	0	0	0	0	0	0	0	0
0	0	0	0	0	0	0	0	0
0	0	0	0	0	0	0	0	0
0	0	0	0	0	0	0	0	0
0	0	0	0	0	0	0	0	0
0	0	0	0	0	0	0	1	0
0	0	0	0	0	0	0	0	0
0	0	0	0	0	0	0	1	0
0	0	0	0	0	0	0	0	0
0	0	0	0	0	0	0	0	0
0	0	0	0	0	0	0	0	0
0	1	0	0	0	0	1	0	0
0	0	0	0	0	0	0	0	0
0	0	0	0	0	0	0	0	0
0	0	0	0	0	0	0	0	0
0	0	0	0	0	0	0	0	0
0	0	0	0	0	0	0	1	0
0	0	0	0	0	0	0	0	0
0	0	0	0	1	0	0	0	0
0	0	0	0	0	0	1	0	0
1	0	0	0	0	0	0	0	0
0	0	0	0	0	0	0	0	0
0	0	0	0	0	0	0	0	0
0	0	0	0	0	0	0	0	0
0	0	0	0	0	0	0	0	0
0	0	0	0	0	0	0	0	0
0	0	0	0	0	0	0	0	0
0	0	0	0	0	0	0	0	0
0	0	0	0	0	0	0	0	0
0	0	0	0	0	0	0	0	0
0	0	0	0	0	0	0	0	0
0	0	0	0	0	0	0	0	0
0	0	0	0	0	0	0	0	0
0	0	0	0	0	0	0	0	0
0	0	0	0	0	0	0	0	0
0	0	0	0	0	0	0	0	1
0	0	0	0	0	0	0	1	0
0	0	0	0	1	0	0	0	0
0	0	0	0	0	0	0	0	0
1	0	0	0	1	0	0	1	0
0	0	0	0	0	0	0	0	0
0	0	0	0	0	0	0	0	0
1	0	0	0	0	0	0	1	0
0	0	0	0	0	0	0	0	0
0	0	0	0	0	0	0	0	0

0	0	0	0	0	0	0	0	0
0	0	0	0	1	0	0	0	0
1	0	0	0	0	0	0	0	0
0	1	0	0	0	0	0	0	0
0	0	0	0	0	0	0	0	0
0	1	0	0	0	0	0	0	0
0	0	0	0	0	0	0	0	0
0	0	0	0	0	0	0	0	0
0	0	0	0	0	0	0	0	0
0	0	0	0	0	0	0	0	0
0	0	0	0	0	0	0	0	0
0	1	0	0	0	0	0	1	0
0	0	0	0	0	0	0	0	0
0	0	0	0	0	0	0	0	0
0	0	0	0	0	0	0	0	1
0	0	0	0	0	0	0	0	0
0	0	0	0	0	0	0	0	0
0	0	0	1	0	0	0	0	0
0	0	0	0	0	0	0	0	0
0	0	0	0	0	0	0	1	0
0	0	0	0	0	0	0	0	0
0	0	0	0	0	0	0	0	0
0	0	0	0	0	0	0	0	0
0	0	0	0	0	0	0	0	0
0	0	0	0	1	0	0	1	0
0	0	0	0	0	0	0	1	0
0	0	0	0	0	0	0	1	0
0	0	0	0	0	0	0	0	0
0	0	0	0	1	0	0	1	0
0	0	0	0	0	0	0	0	0
0	0	0	0	0	0	0	0	0
0	0	0	0	0	0	0	0	0
0	0	0	0	0	0	0	0	0
0	0	0	0	0	0	0	0	0
0	0	0	0	0	0	0	0	0
0	0	0	0	0	0	0	0	0
0	0	0	0	0	0	0	1	0
1	0	0	0	0	0	0	0	0
1	0	0	0	0	0	0	0	0
0	0	0	0	0	0	0	0	0
0	0	0	0	0	0	0	0	0
0	0	0	0	0	0	0	0	0
0	0	0	0	0	0	0	0	0
0	0	0	0	0	1	0	0	0
1	0	0	0	0	0	0	0	0
0	0	0	0	0	0	0	0	0
0	1	0	0	0	0	0	0	0
0	0	0	0	0	0	0	1	0
0	0	0	0	0	0	0	0	0
0	0	0	0	1	0	0	0	0
0	0	0	0	0	0	0	0	0
0	0	0	0	0	0	0	0	0
1	0	0	0	0	0	0	0	0
1	0	0	0	0	0	0	0	0
0	0	0	0	0	0	0	0	0
0	0	0	0	0	0	0	0	0

0	1	0	0	0	0	0	0	0
0	0	0	0	0	0	0	0	0
0	0	0	0	0	0	0	0	0
0	0	0	0	0	0	0	0	0
1	0	0	0	0	0	0	0	0
0	0	0	0	0	0	0	0	0
1	1	0	0	0	0	0	0	0
0	0	0	0	0	0	0	0	0
0	0	0	0	0	0	0	0	0
0	0	0	0	0	0	0	0	0
1	0	0	0	0	0	0	1	0
0	1	0	0	0	0	0	1	0
0	0	0	0	0	0	0	0	0
1	0	0	0	0	0	0	0	0
0	0	0	0	0	0	0	0	0
0	0	0	0	0	0	0	0	0
0	0	0	0	0	0	0	1	0
0	0	0	0	0	0	0	0	0
0	0	0	0	0	0	0	0	0
1	0	0	0	0	0	0	0	0
0	0	0	0	0	0	0	0	0
0	0	0	0	0	0	0	0	0
0	0	0	0	0	0	0	0	0
0	0	0	0	0	0	0	0	0
0	0	0	0	0	0	0	0	0
0	0	0	0	0	0	0	0	0
0	0	0	0	0	0	0	0	0
0	0	0	0	0	0	0	0	0
0	0	0	0	0	0	0	0	0
0	0	0	0	1	0	0	0	0
0	0	0	0	0	0	0	1	0
0	0	0	0	0	0	0	0	0
0	0	0	0	0	0	0	0	0
0	0	0	0	0	0	0	0	0
0	0	0	0	1	0	0	1	0
0	0	0	0	1	0	0	1	0
0	1	0	0	0	0	0	0	0
0	0	0	0	0	0	0	0	0
1	0	0	0	0	0	0	0	0
0	0	0	0	0	0	0	0	0
0	0	0	0	0	0	0	0	0
0	0	0	0	0	0	0	0	0
0	0	0	0	0	0	0	0	0
0	0	0	0	0	0	0	0	0
0	0	0	0	0	0	0	0	0
0	0	0	0	0	0	0	0	0
0	1	0	0	0	0	0	1	0
1	0	0	0	0	0	0	0	0
0	0	0	0	0	0	0	0	0
0	0	0	0	0	0	0	0	0
0	0	0	0	0	0	0	0	0
0	0	0	0	0	0	0	0	0
1	0	0	0	0	0	0	0	0
0	0	0	0	0	0	0	0	0
0	0	0	0	0	0	0	0	0
1	0	0	0	0	0	0	0	0

0	0	0	0	0	0	0	0	0
0	0	0	0	0	0	0	1	0
1	0	0	0	1	0	0	0	0
0	0	0	0	0	0	0	1	0
1	0	0	0	0	0	0	0	0
0	0	0	0	0	0	0	0	0
0	1	0	0	0	0	0	0	0
0	0	0	0	0	0	0	0	0
0	0	0	0	0	0	0	0	0
0	0	0	0	0	1	0	1	0
0	0	0	0	0	0	0	0	0
0	0	0	0	0	0	0	0	0
0	0	0	0	1	0	0	0	0
0	1	0	0	0	0	0	0	0
0	0	0	0	0	0	0	0	0
0	0	0	1	0	0	0	0	0
1	0	0	0	0	0	0	1	0
0	0	0	0	0	0	0	0	0
0	0	0	0	0	0	0	0	0
0	0	0	0	0	0	0	0	0
0	0	0	0	0	0	0	0	0
0	0	0	0	0	0	0	0	0
0	0	0	0	0	0	0	0	0
0	0	0	0	0	0	0	0	0
0	0	0	0	0	0	0	1	0
0	0	0	0	0	0	0	0	0
0	0	0	0	0	0	0	0	0
0	0	0	0	0	0	0	0	0
0	0	0	0	0	0	0	0	0
1	0	0	0	0	0	0	0	0
0	0	0	0	0	0	0	0	0
1	0	0	0	0	0	0	0	0
0	0	0	0	0	0	0	0	0
0	0	0	0	0	0	1	0	0
0	0	0	0	0	0	0	0	0
0	0	0	0	0	0	0	0	0
0	0	0	0	0	0	0	0	0
0	0	0	0	0	0	0	0	0
0	1	0	0	0	0	0	0	0
1	0	0	0	0	0	0	0	0
0	0	0	0	0	0	0	0	0
1	0	0	0	0	0	0	0	0
0	0	0	0	0	0	0	0	0
1	0	0	0	0	0	0	0	0
0	0	0	0	0	0	0	0	0
0	0	0	0	1	0	0	0	0
1	0	0	0	0	0	0	0	0
0	0	0	0	0	0	0	0	0
0	1	0	0	0	0	0	0	0
0	0	0	0	0	0	0	1	0
0	0	0	0	0	0	0	0	0
0	0	0	0	0	0	0	1	0
0	0	0	0	0	0	0	0	0
0	0	0	0	0	0	0	0	0
0	0	0	0	0	0	0	0	0
0	0	0	0	0	0	0	0	0

0	0	0	0	0	0	0	0	0
0	0	0	0	0	0	0	0	0
0	0	0	0	0	0	0	0	0
0	0	0	0	0	0	0	0	0
0	0	0	0	0	0	0	0	0
0	0	0	0	0	0	0	0	0
1	0	0	0	0	0	0	0	0
0	0	0	0	0	0	0	0	0
1	0	0	0	0	0	0	0	0
0	0	0	0	0	0	0	0	0
0	0	0	0	0	0	0	0	0
0	1	0	0	0	0	0	1	1
0	0	0	0	0	0	0	0	0
0	1	0	0	0	0	0	0	0
0	1	0	0	0	0	0	0	0
0	1	0	0	0	0	0	0	0
0	0	0	0	0	0	0	0	0
0	0	0	0	0	0	0	0	0
0	0	0	0	0	0	0	0	0
0	0	0	0	0	0	1	0	0
1	0	0	0	0	0	0	0	0
1	0	0	0	0	0	0	0	1
0	0	0	0	0	1	0	0	0
0	0	0	0	0	0	0	0	0
0	1	0	1	0	0	0	0	0
0	0	0	0	0	0	0	0	0
1	0	0	0	0	0	0	0	0
0	0	0	0	0	0	0	0	1
1	1	0	1	0	0	0	0	0
0	0	0	0	0	0	0	0	0
0	0	0	0	0	0	0	0	0
0	0	0	0	0	0	0	0	0
0	0	0	0	0	0	0	0	0
0	0	0	0	0	0	0	0	0
0	0	0	0	0	0	0	0	0
0	0	0	0	0	0	0	0	0
0	0	0	0	0	0	0	0	0
0	0	0	0	0	0	0	0	0
0	0	0	0	0	0	0	0	0
0	0	0	0	0	0	0	0	0
0	0	0	0	0	0	0	0	0
1	0	0	0	0	0	0	0	0
0	0	0	0	0	0	0	0	0
0	0	0	1	0	0	0	0	0
0	0	0	0	0	0	0	0	0
0	0	0	0	0	0	0	1	0
0	0	0	0	0	0	0	0	0
1	0	0	0	0	0	0	0	0
0	0	0	0	0	0	0	1	0
0	0	0	0	0	0	0	0	0
1	0	0	0	0	0	0	0	0
1	0	0	0	0	0	0	0	0
0	0	0	0	0	0	0	0	1
0	0	0	0	0	0	0	0	0
0	0	0	0	0	0	0	0	0
0	0	0	0	0	0	0	0	0

0	0	0	0	0	0	0	0	0
0	0	0	0	0	0	0	0	0
0	0	0	0	0	0	0	0	0
1	0	0	0	1	0	0	0	0
0	0	0	0	0	0	0	0	0
0	0	0	0	1	0	0	0	0
0	0	0	1	0	0	0	0	0
0	0	0	0	0	0	0	0	0
0	0	0	0	0	0	0	0	0
0	0	0	0	0	0	0	0	0
1	0	0	0	0	0	0	0	0
0	0	0	0	0	0	0	0	0
0	0	0	0	0	0	0	0	0
0	0	0	0	0	0	0	0	1
0	0	0	0	0	0	0	0	0
0	0	0	0	0	0	0	0	0
0	0	0	0	0	0	0	0	0
0	0	0	0	0	0	0	1	0
1	0	0	0	0	0	0	0	0
0	0	0	0	0	0	0	0	0
0	0	0	0	1	0	0	0	0
0	0	0	0	0	0	0	0	0
0	1	0	0	0	0	0	0	0
0	0	0	0	1	0	0	0	0
0	0	0	0	0	0	1	0	0
0	0	0	0	0	0	0	0	0
1	0	0	0	0	0	0	0	0
0	0	0	0	1	0	0	1	0
1	0	0	0	0	0	0	0	0
0	0	0	0	0	0	0	1	0
0	0	0	0	0	0	0	0	0
0	0	0	0	0	0	0	0	0
0	0	0	0	0	0	0	1	0
0	0	0	0	1	0	0	0	0
0	0	0	0	0	0	0	0	0
0	0	0	0	0	0	0	0	0
0	0	0	0	0	0	0	0	0
0	0	0	0	0	0	0	0	0
1	0	0	0	1	0	0	0	0
1	0	0	0	0	0	0	0	1
1	0	0	0	0	0	0	0	0
1	0	0	0	0	0	0	0	0
0	0	0	0	0	0	0	0	0
0	0	0	0	0	0	0	0	0
0	0	0	0	0	0	0	0	0
0	0	0	0	0	0	0	0	0
0	0	0	0	1	0	0	0	0
0	0	0	0	0	0	0	0	0
0	0	0	0	0	0	0	0	0
0	0	0	0	0	0	0	0	0
0	0	0	0	0	0	0	0	0
0	0	0	0	1	0	0	0	0
0	0	0	0	0	0	0	0	0
0	0	0	0	0	0	0	0	0
0	0	0	0	0	0	0	0	0
0	0	0	0	0	0	0	0	0

0	0	0	0	1	0	0	0	0
0	0	0	0	1	0	0	0	0
0	0	0	0	0	0	0	0	0
1	0	0	0	0	0	0	0	0
1	0	0	0	0	0	0	0	0
0	0	0	0	0	0	0	0	0
0	0	0	0	0	0	0	0	0
0	1	0	0	0	0	0	0	0
0	0	0	0	0	0	0	0	0
0	0	0	0	0	0	0	0	0
0	0	0	0	0	0	0	0	0
0	0	0	0	0	0	0	0	0
0	0	0	0	0	0	0	0	0
0	0	0	0	0	0	0	0	0
0	0	0	0	0	0	0	0	0
0	0	0	0	0	0	0	0	0
1	0	0	0	0	0	0	0	0
0	0	0	0	0	0	0	0	0
0	0	0	0	0	0	0	0	0
0	0	0	0	0	0	0	0	0
0	0	0	0	0	0	0	0	0
0	0	0	0	0	0	0	0	0
1	0	0	0	1	0	0	0	0
0	0	0	0	0	0	0	1	0
0	0	0	0	0	0	0	0	0
1	0	0	0	0	0	0	0	0
1	0	0	0	0	0	0	1	0
0	0	0	0	0	0	0	0	0
0	0	0	0	0	0	0	0	0
1	0	0	0	0	0	0	0	0
0	1	0	0	0	0	0	0	0
0	0	0	0	0	0	0	0	0
0	0	0	0	0	0	0	0	0
0	0	0	0	0	0	0	0	0
0	0	0	0	0	0	0	0	0
0	0	0	0	0	0	0	0	0
0	0	0	0	0	0	0	0	0
0	0	0	0	0	0	0	0	0
0	0	0	0	0	0	0	0	0
1	0	0	0	0	0	0	0	0
0	0	0	0	0	0	0	0	0
0	0	0	0	0	0	0	0	0
0	0	0	0	0	0	1	0	0
1	0	0	0	0	0	0	0	0
0	0	0	0	0	0	0	0	0
0	1	0	0	0	0	0	0	0
1	1	0	0	0	0	0	0	0
1	0	0	0	0	0	0	0	0
0	0	0	0	0	0	0	0	0
1	0	0	0	0	0	0	0	0
0	0	0	0	0	0	0	0	0
0	1	0	0	0	0	0	0	0
1	0	0	0	0	0	0	0	0
0	0	0	0	0	0	0	0	0
1	0	0	0	0	0	0	0	0
0	0	0	0	0	0	0	0	0

0	0	0	0	0	0	0	0	0
0	0	0	0	0	0	0	0	0
1	1	0	0	0	0	0	0	0
1	1	0	0	0	0	0	0	0
0	0	0	0	0	0	1	0	0
0	0	0	0	0	0	0	0	0
0	0	0	0	0	0	0	0	0
0	0	0	0	0	0	0	0	0
0	0	0	0	0	0	0	1	1
0	1	0	0	0	0	0	0	0
0	0	0	0	0	0	0	0	0
0	0	0	0	0	0	0	0	0
0	0	0	0	0	0	0	0	0
1	0	0	0	0	0	0	0	0
0	0	0	0	0	0	0	0	0
0	0	0	1	0	0	0	0	0
0	0	0	0	0	0	0	0	0
0	0	0	0	0	0	1	0	0
0	0	0	0	0	0	0	0	0
0	0	0	0	0	0	0	0	0
0	1	0	0	0	0	0	1	0
0	0	0	0	0	0	0	0	0
0	0	0	0	0	0	0	0	0
0	0	0	0	0	0	0	0	0
0	0	0	0	0	0	0	0	0
1	0	0	1	0	0	0	0	0
0	0	0	0	0	0	1	0	0
1	0	0	0	1	0	0	0	0
0	0	0	0	0	0	0	1	0
0	0	0	0	0	0	0	0	0
0	0	0	0	0	0	0	0	0
0	0	0	0	0	0	1	0	0
0	0	0	0	0	0	0	0	0
0	0	0	0	0	0	0	1	0
0	0	0	0	0	0	1	0	0
0	0	0	0	0	0	0	1	0
0	0	0	0	0	0	0	0	0
0	0	0	0	0	0	0	1	0
1	1	0	0	0	0	0	0	0
0	0	0	1	0	0	0	0	0
0	0	0	0	0	0	0	1	0
0	0	0	0	0	0	0	0	0
0	0	0	0	0	0	0	0	0
1	0	0	0	0	0	0	0	0
0	0	0	1	0	0	0	0	0
0	0	0	0	0	0	0	0	0
0	0	0	0	0	0	0	1	0
0	0	0	0	0	0	0	0	0
0	0	0	0	0	0	0	0	0
1	0	0	0	0	0	0	1	0
1	1	0	0	0	0	0	0	0
1	0	0	0	0	0	0	1	0
0	1	0	0	0	0	0	0	0
0	0	0	0	0	0	1	0	0
0	0	0	0	0	0	0	0	0
1	1	0	0	0	0	0	0	0

0	0	0	0	0	0	0	0	0
1	1	0	0	0	0	0	0	0
0	0	0	0	0	0	1	0	0
0	0	0	0	0	0	0	0	0
0	0	0	1	0	0	0	0	0
1	0	0	0	0	0	1	0	0
1	0	0	0	0	0	0	1	0
1	0	0	0	0	0	0	1	0
0	0	0	0	0	0	1	0	0
1	0	0	0	0	0	0	0	0
1	0	0	0	0	0	0	0	0
0	0	0	0	0	0	0	0	0
0	0	0	0	0	0	0	0	0
0	0	0	0	0	0	0	0	0
0	0	0	0	0	0	0	0	0
0	0	0	0	0	0	0	0	0
1	0	0	0	0	0	1	0	0
0	0	0	0	0	0	0	1	0
0	0	0	0	0	0	0	0	0
0	0	0	0	0	0	0	0	0
0	0	0	0	0	0	0	0	0
0	0	0	0	0	0	0	0	0
0	0	0	0	0	0	0	0	0
0	0	0	1	0	0	0	0	0
0	0	0	0	0	0	0	0	0
0	0	0	0	0	0	0	0	0
1	0	0	1	0	0	1	0	0
0	0	0	0	0	0	0	1	0
0	0	0	0	0	0	0	0	0
0	0	0	0	0	0	0	1	1
0	0	0	0	0	0	0	0	0
0	0	0	0	0	0	0	0	0
0	0	0	0	0	0	0	1	0
0	0	0	0	0	0	0	0	0
0	0	0	0	0	0	1	0	0
0	0	0	0	0	0	0	0	0
0	0	0	0	1	1	0	0	0
0	0	0	0	0	0	0	0	0
0	0	0	0	0	0	0	0	0
0	0	0	0	1	0	0	0	0
0	0	0	0	0	0	0	0	0
1	1	0	0	0	0	0	0	0
0	0	0	0	0	0	0	0	0
1	0	0	0	0	0	0	0	0
1	0	0	0	1	0	0	0	0
0	0	0	0	0	0	0	0	0
1	0	0	0	0	0	0	0	0
0	0	0	0	0	0	0	0	0
1	0	0	0	0	0	1	0	0
1	0	0	0	1	0	1	0	0
0	0	0	0	0	0	0	0	0
0	0	0	0	0	0	0	0	0
1	0	0	0	0	0	0	1	0
1	1	0	0	0	0	0	0	0
0	0	0	0	0	0	1	0	0
0	1	0	0	0	0	0	0	0

1	0	0	0	1	0	0	0	0
0	0	0	0	0	0	0	0	0
0	0	0	0	0	0	0	0	0
0	0	0	0	0	0	0	0	0
0	0	0	0	0	0	0	0	0
1	0	0	0	0	0	0	0	0
0	0	0	0	0	0	0	0	0
0	0	0	0	0	0	0	0	0
0	0	0	0	0	0	0	0	0
0	0	0	0	0	0	0	0	0
0	0	0	0	0	0	0	0	0
0	0	0	0	0	0	0	0	0
0	0	0	0	0	0	0	0	0
0	0	0	0	0	0	0	0	0
1	0	0	0	0	0	0	0	0
0	0	0	0	0	0	0	1	0
0	0	0	0	0	0	1	0	0
0	0	0	0	0	0	0	0	0
0	0	0	0	0	0	0	0	0
0	1	0	0	0	0	0	1	0
0	0	0	0	0	0	0	0	0
0	0	0	0	0	0	0	0	0
1	0	0	0	0	0	0	0	0
0	0	0	0	0	0	0	0	0
0	0	0	0	0	0	0	1	0
0	0	0	0	1	0	0	0	0
0	0	0	0	0	0	0	0	0
0	0	0	0	0	0	0	0	0
0	0	0	0	0	0	0	0	0
0	0	0	0	0	0	0	0	0
0	0	0	0	0	0	0	0	0
1	0	0	0	0	0	0	0	0
0	0	0	0	0	0	0	0	0
0	0	0	0	0	0	0	0	0
0	1	0	0	1	0	0	1	0
1	0	0	0	0	0	0	0	0
0	0	0	0	0	0	0	0	0
0	0	0	0	0	0	0	0	0
0	0	0	0	0	0	0	0	0
1	0	0	0	0	0	0	0	0
0	0	0	0	0	0	0	0	0
0	0	0	0	1	0	0	0	0
1	0	0	0	0	0	0	0	0
0	1	0	0	0	0	0	0	0
1	0	0	0	0	0	0	0	0
0	0	0	0	0	0	0	1	0
1	0	0	0	0	0	0	0	0
0	0	0	0	0	0	0	0	0
0	0	0	0	0	0	0	0	0
0	0	0	0	1	0	0	0	0
0	0	0	0	0	1	0	0	0
1	0	0	0	0	0	0	0	0
0	0	0	0	0	0	0	0	0
1	0	0	0	0	0	1	1	0
1	0	0	0	0	0	0	0	0
1	0	0	0	0	0	0	0	0

0	0	0	0	0	0	0	0	0
0	0	0	0	0	0	0	0	0
1	0	0	0	0	0	0	0	0
0	0	0	0	0	0	0	0	0
0	0	0	0	0	0	0	0	0
1	0	0	0	0	0	0	0	0
0	0	0	0	0	0	0	0	0
0	0	0	0	1	0	0	0	0
0	0	0	0	0	0	0	1	0
1	1	0	0	0	1	0	0	0
1	0	0	0	0	0	0	0	0
0	0	0	0	0	0	0	0	0
1	0	0	0	0	0	0	0	0
1	0	0	0	0	0	0	0	0
1	0	0	0	0	0	0	0	0
0	0	0	0	0	0	0	0	0
0	0	0	0	0	0	0	0	0
0	1	0	0	0	0	0	0	0
1	0	0	0	0	0	0	0	0
1	0	0	0	0	0	0	1	0
0	0	0	0	0	0	0	0	0
0	0	0	0	0	0	0	0	0
1	0	0	0	0	0	0	0	0
0	0	0	0	0	0	0	0	0
0	0	0	0	0	0	0	0	0
1	0	0	0	0	0	0	0	0
0	0	0	0	0	0	0	0	0
0	0	0	0	0	0	1	0	0
0	0	0	0	0	0	0	0	0
0	0	0	0	0	0	0	0	0
0	0	0	0	0	0	0	0	0
0	0	0	0	0	0	0	0	0
0	0	0	0	0	0	0	0	0
0	0	0	0	0	0	0	0	0
0	0	0	0	0	0	0	0	0
0	0	0	0	0	0	0	0	0
1	0	0	0	0	0	0	0	0
1	0	0	0	0	0	0	0	0
0	0	0	0	0	0	1	0	0
0	0	0	0	0	0	0	0	0
0	0	0	0	0	0	0	0	0
0	0	0	0	0	0	0	0	0
0	0	0	0	0	0	0	0	0
0	0	0	0	1	0	0	1	0
0	0	0	0	0	0	0	0	0
0	0	0	0	0	0	0	0	0
0	0	1	0	0	0	0	1	0
0	0	0	0	0	0	0	0	0
0	0	0	0	0	0	0	0	0
0	0	0	0	0	0	0	0	0
1	0	0	0	0	0	0	0	0
0	0	0	0	1	0	0	0	0
1	0	0	0	0	0	0	0	0
0	0	0	0	0	0	0	0	0
0	0	0	0	0	0	0	0	0
1	0	0	0	0	0	0	1	0

0	0	0	0	0	0	0	0	0
1	0	0	0	1	0	0	0	0
0	0	0	0	0	0	0	0	1
1	0	0	0	0	0	0	0	0
1	0	0	0	0	0	0	0	0
0	0	0	0	0	0	0	0	0
0	0	0	0	0	0	0	1	0
1	0	0	1	0	0	0	0	0
0	0	0	0	0	0	0	0	0
0	0	0	0	0	0	0	0	0
0	1	0	0	0	0	0	0	0
1	0	0	0	0	0	0	0	0
0	0	0	0	0	0	0	0	0
0	0	0	0	1	0	0	0	0
1	1	0	0	0	0	0	0	0
0	0	0	0	0	0	0	0	0
0	0	0	0	0	0	0	0	0
0	0	0	0	0	0	0	0	0
0	1	0	0	0	0	0	0	0
0	0	0	0	0	0	0	0	0
1	0	0	0	0	0	0	1	0
0	0	0	0	0	0	0	0	0
0	0	0	0	0	0	0	0	0
0	0	0	0	0	0	0	1	0
1	1	0	0	0	0	0	0	0
1	0	0	0	0	0	0	0	0
0	1	0	0	0	0	0	0	0
1	0	0	0	0	0	0	0	0
0	0	0	0	0	0	1	0	0
0	0	0	0	0	0	0	0	0
0	0	0	0	1	0	0	0	0
0	0	0	0	0	0	1	0	0
0	0	0	0	0	0	0	0	0
1	0	0	0	0	0	0	0	0
0	0	0	0	0	0	0	0	0
1	0	0	0	0	0	0	0	0
1	0	0	0	0	0	1	0	0
0	0	0	0	0	0	0	1	0
0	0	0	0	0	0	0	0	0
0	0	0	0	0	0	0	0	0
0	0	0	0	0	0	0	0	0
0	0	0	0	0	0	0	0	0
0	0	0	0	0	0	0	0	0
0	0	0	0	0	0	0	0	0
1	0	0	0	0	0	0	0	0
1	0	0	0	0	0	0	0	0
0	0	0	0	0	0	0	0	0
1	1	0	1	0	0	0	0	0
0	0	0	0	0	0	0	0	0
0	1	0	0	0	0	0	0	0
0	0	0	0	0	0	0	0	0
1	0	0	0	0	0	0	0	0
0	1	0	0	0	0	0	0	0
1	0	0	0	0	0	0	0	0
0	0	0	0	0	0	0	0	0
0	0	0	0	0	0	0	0	0

0	0	0	0	0	0	0	0	0
0	0	0	0	0	0	0	0	0
0	0	0	0	0	0	0	0	0
0	1	0	0	0	0	0	1	0
0	0	0	0	0	0	0	0	0
1	0	0	0	0	0	0	0	0
0	0	0	0	0	0	0	0	0
1	0	0	0	0	0	0	0	0
0	0	0	0	0	0	0	1	0
0	0	0	0	0	0	0	0	0
1	0	0	0	0	0	0	0	0
0	0	0	0	0	0	0	0	0
0	0	0	0	1	0	0	0	0
0	1	0	0	0	0	0	1	0
0	0	0	0	0	0	0	0	0
1	0	0	0	0	0	0	0	0
0	0	0	0	0	0	0	1	1
0	0	0	0	0	0	0	0	0
0	0	0	1	0	0	0	0	0
0	0	0	1	0	0	0	0	0
1	0	0	0	0	0	0	0	0
1	0	0	0	0	0	0	0	0
1	0	0	0	0	0	0	0	0
1	0	0	0	0	0	0	0	0
0	0	0	0	0	0	0	0	0
0	0	0	0	0	0	0	1	0
0	0	0	0	0	0	0	0	0
1	1	0	0	0	0	0	0	0
0	0	0	0	0	0	0	0	0
0	0	0	0	0	0	0	1	0
0	0	0	0	0	0	0	0	0
0	0	0	0	0	0	0	0	0
0	0	0	0	0	0	0	0	0
0	0	0	0	0	0	1	0	0
0	0	0	0	0	0	0	0	0
1	0	0	0	0	0	0	0	0
1	0	0	0	0	0	0	0	0
0	0	0	0	0	0	0	0	0
0	0	0	0	0	0	0	0	0
1	0	0	0	0	0	0	0	0
0	0	0	0	0	0	0	0	0
0	0	0	0	0	0	0	0	0
0	0	0	0	0	0	0	0	0
0	1	0	0	0	0	0	0	0
0	0	0	0	0	0	0	0	0
1	0	0	0	0	0	0	0	0
1	0	0	0	0	0	0	0	0
0	0	0	0	0	0	0	0	0
0	0	0	0	0	0	0	0	0
0	0	0	0	0	0	0	0	0
0	0	0	0	0	0	0	0	0
1	0	0	0	0	0	0	0	0
1	0	0	0	0	0	0	0	0
1	1	0	0	0	0	0	1	0
1	0	0	0	0	0	0	0	0
1	0	0	1	0	0	0	0	0

0	1	0	0	0	0	0	0	0
0	1	0	0	0	0	0	0	0
1	0	0	0	0	0	0	0	0
1	1	0	0	0	0	0	1	0
1	0	0	0	0	0	0	0	0
0	0	0	0	0	0	0	0	0
1	1	0	0	0	0	0	0	0
0	0	0	0	1	0	0	0	0
0	0	0	0	0	0	0	0	0
0	0	0	0	0	0	0	0	0
1	0	0	0	0	0	0	0	0
0	0	0	0	0	0	0	0	0
1	1	0	0	0	0	0	0	0
0	0	0	0	0	0	0	0	0
0	1	0	0	0	0	0	1	0
0	0	0	0	0	0	0	0	0
0	0	0	0	0	0	0	0	0
1	0	0	0	0	0	0	0	0
0	0	0	0	0	0	1	0	0
0	0	0	0	0	0	0	0	0
0	0	0	0	0	0	0	0	0
0	1	0	0	0	0	0	0	0
1	0	0	0	0	0	0	0	0
1	0	0	0	0	0	0	0	0
1	0	0	0	0	0	0	0	0
0	0	0	0	0	0	0	0	0
0	0	0	0	0	0	0	0	0
1	0	0	0	0	0	0	0	0
0	0	0	0	0	0	0	0	0
0	0	0	0	0	0	0	0	0
0	0	0	0	0	0	0	0	0
0	0	0	0	0	0	0	0	0
0	0	0	0	0	0	0	0	0
0	0	0	0	0	0	0	0	0
0	0	0	0	0	0	0	0	0
1	0	0	0	0	0	0	0	0
1	0	0	0	0	0	0	0	0
1	0	0	0	0	0	0	0	0
0	1	0	0	0	0	0	0	0
0	0	0	1	0	0	0	0	0
0	0	0	1	0	0	0	0	0
1	0	0	0	0	0	0	0	0
1	0	0	0	0	0	0	0	0
0	0	0	0	0	0	0	1	0
0	0	0	0	0	0	0	0	0
0	0	0	0	0	0	0	0	0
0	0	0	0	0	0	0	0	0
0	0	0	0	0	0	0	0	0
0	0	0	0	0	0	0	0	0
0	0	0	0	0	0	0	0	0
0	1	0	0	0	0	0	1	0
0	0	0	0	0	0	0	0	0
1	0	0	0	0	0	0	0	0
1	0	0	0	0	0	0	0	0
0	0	0	0	0	0	0	0	0
1	0	0	0	0	0	0	0	0

1	0	0	0	0	0	0	0	0
1	0	0	1	0	0	0	0	0
0	0	0	0	0	0	0	0	0
1	0	0	0	0	0	0	0	0
0	0	0	0	0	0	0	0	0
0	0	0	0	0	0	0	0	0
0	0	0	0	0	0	0	0	0
0	0	0	0	0	0	0	0	0
1	0	0	0	0	0	0	0	0
1	0	0	0	0	0	1	0	0
1	0	0	0	0	0	0	0	0
0	0	0	0	0	0	0	0	0
0	0	0	0	0	0	0	0	0
0	0	0	0	0	0	0	0	0
0	1	0	0	0	0	0	0	0
0	0	0	0	0	0	0	0	0
0	0	0	0	0	0	0	0	0
1	0	0	0	0	0	0	0	0
0	0	0	0	0	0	0	0	0
0	0	0	0	0	0	0	0	0
0	0	0	0	0	0	0	0	0
0	0	0	0	0	1	0	0	0
0	0	0	0	0	0	0	0	0
0	0	0	0	0	0	0	0	0
1	1	0	0	0	0	0	0	0
0	0	0	0	0	0	0	0	0
0	1	0	0	0	0	0	0	0
0	0	0	0	0	0	0	0	0
0	0	0	0	1	0	0	0	0
0	0	0	0	0	0	0	0	0
0	0	0	0	0	0	0	1	0
0	0	0	0	0	0	0	0	1
0	0	0	0	0	0	0	0	0
1	0	0	0	0	0	0	0	0
1	0	0	0	0	0	0	0	0
0	0	0	0	0	0	0	1	0
0	0	0	0	0	0	0	0	0
1	0	0	0	0	0	0	0	0
0	0	0	0	0	0	0	0	0
0	0	0	1	0	0	0	0	0
0	0	0	0	0	0	0	0	0
1	0	0	0	0	0	0	0	0
0	0	0	0	0	0	0	0	0
1	0	0	0	0	0	0	0	0
1	0	1	0	0	0	0	0	0
0	0	0	0	0	0	0	1	0
0	0	0	0	0	1	0	1	0
1	0	0	0	0	0	0	0	0
0	0	0	0	0	0	0	0	0
0	1	0	0	0	0	1	0	0
1	0	0	0	0	0	0	1	0
1	0	0	0	0	0	0	0	0
0	0	0	0	0	0	0	0	0
0	1	1	0	0	0	0	0	0
0	0	0	0	0	0	0	0	0
1	0	0	0	0	0	0	0	0

0	0	0	0	0	0	0	0	0
0	0	0	0	0	0	0	0	0
0	0	0	0	0	0	0	0	0
0	0	0	0	0	0	0	0	0
0	0	0	0	0	0	0	0	0
0	0	0	0	0	0	0	0	0
0	0	0	0	0	0	0	0	0
0	0	0	0	0	0	0	0	0
0	0	0	0	0	0	0	0	0
0	0	0	0	0	0	0	0	0
0	0	0	0	0	0	0	0	0
1	0	0	0	0	0	0	0	0
1	1	0	0	0	0	0	0	0
0	0	0	0	0	0	0	1	0
0	0	0	0	0	0	0	0	0
1	0	0	0	0	0	0	0	0
1	0	0	0	0	0	0	1	0
0	0	0	0	0	0	0	0	0
1	0	0	0	0	0	0	0	0
1	0	0	0	0	0	0	0	0
0	0	0	0	1	0	0	0	0
0	0	0	0	0	0	0	0	0
0	0	0	0	0	0	0	0	0
0	0	0	0	0	0	0	0	0
1	0	0	0	0	0	0	0	0
0	0	0	0	0	0	0	1	0
0	0	0	0	0	0	0	0	0
0	1	0	0	0	0	1	0	0
0	0	0	0	0	0	0	0	0
0	1	0	0	0	0	0	0	0
1	0	0	0	1	0	1	0	0
0	0	0	0	0	0	0	0	0
0	0	0	0	0	0	0	0	0
0	0	0	0	0	0	0	1	0
0	0	0	0	0	0	0	0	0
0	0	0	0	0	0	0	0	0
0	0	0	0	0	0	0	0	0
0	0	0	0	0	0	0	0	0
1	0	0	0	0	0	0	0	0
0	0	0	0	0	0	0	0	0
0	0	0	0	0	0	0	0	0
0	0	0	0	0	0	0	0	0
0	0	0	0	0	0	0	0	0
0	0	0	0	0	0	0	0	0
0	0	0	0	0	0	0	0	0
0	0	0	0	0	0	0	1	0
1	0	0	0	0	0	0	0	0
0	0	0	0	0	0	0	0	0
0	0	0	0	0	0	0	0	0
0	0	0	0	0	0	0	0	0
0	0	0	0	0	0	0	0	0
0	0	0	0	0	0	0	0	0
1	0	0	0	0	0	0	0	0
0	0	0	0	0	0	0	0	0
0	0	0	0	0	0	0	1	0
0	0	0	0	0	0	0	0	0

0	0	0	0	0	0	0	1	1
0	0	0	0	0	0	0	0	0
1	0	0	0	0	0	0	0	0
1	0	0	0	0	0	0	0	0
0	0	0	0	0	0	0	0	0
0	0	0	0	0	0	0	0	0
0	0	0	0	0	0	0	0	0
0	1	1	0	0	0	0	0	0
0	0	0	0	0	0	0	1	0
1	0	0	0	0	0	0	0	0
0	0	0	0	0	0	0	0	0
0	0	0	0	0	0	0	0	0
0	0	0	0	0	0	0	0	0
0	0	0	0	0	0	0	0	0
1	0	0	0	0	0	0	0	0
1	0	0	0	0	0	0	1	0
0	0	0	0	0	0	0	0	0
0	0	0	0	0	0	0	0	0
1	0	0	0	0	0	0	0	0
0	0	0	0	1	0	0	0	0
0	1	0	0	0	0	0	0	0
1	0	0	0	0	0	0	0	0
1	0	0	0	0	0	0	0	0
0	0	0	0	0	0	0	0	0
1	0	0	0	0	0	0	0	0
0	0	0	0	0	0	0	0	0
0	0	1	0	0	0	0	0	0
0	0	0	0	0	0	0	0	0
0	0	0	0	0	0	0	0	0
1	0	0	0	0	0	0	0	0
1	0	0	0	0	0	0	0	0
0	0	0	0	0	0	0	0	0
0	0	0	0	0	0	0	0	0
0	0	0	0	0	0	0	1	0
0	0	0	0	0	0	0	0	0
1	0	0	0	0	0	0	0	0
1	0	0	0	0	0	0	0	0
1	1	0	0	0	0	0	0	0
0	0	0	0	0	0	0	0	0
0	0	0	0	0	0	0	0	0
0	0	0	0	0	0	0	0	0
0	0	0	0	0	0	0	0	0
0	1	0	0	0	0	0	0	0
0	0	0	0	0	0	0	0	0
1	0	0	0	0	0	0	0	0
1	0	0	0	0	0	0	0	0
0	0	0	0	0	0	0	0	0
0	0	0	0	0	0	0	0	0
0	1	0	0	0	0	0	0	0
1	0	0	0	0	0	0	0	0
1	0	0	0	0	0	0	0	0
1	0	0	0	0	0	0	0	0
0	0	0	0	0	0	0	0	0
0	0	0	0	0	0	0	0	0
1	0	0	1	0	0	0	0	0
1	0	0	0	0	0	0	0	0

1	0	0	0	0	0	0	0	0
1	0	0	0	0	0	0	0	0
1	0	0	0	0	0	0	0	1
0	0	0	0	0	0	0	0	0
0	0	0	0	0	0	0	0	0
0	0	0	0	0	0	0	0	0
0	1	0	0	0	0	0	0	0
0	0	0	0	0	0	0	0	0
0	0	1	0	0	0	0	0	0
0	0	0	0	0	0	0	0	0
0	0	0	0	0	0	0	0	0
0	0	0	0	0	0	0	0	0
0	0	0	0	0	0	0	1	0
0	0	0	0	0	0	0	0	0
0	0	0	0	0	0	0	0	0
0	0	0	0	0	0	0	0	0
0	0	0	0	0	0	0	0	0
0	0	0	0	1	0	0	0	0
0	0	0	0	0	0	0	0	0
0	0	0	0	0	0	0	0	0
0	0	0	1	0	0	0	0	0
0	0	0	0	0	0	0	0	0
0	0	0	0	0	0	0	0	0
0	0	0	0	0	0	0	0	0
0	0	0	0	0	0	0	0	0
1	0	0	0	1	0	0	0	0
0	0	0	0	0	0	0	0	0
1	0	0	0	0	0	0	0	0
0	0	0	0	0	0	1	0	0
0	0	0	0	0	0	0	0	0
0	0	0	0	0	0	0	0	0
1	0	0	0	0	0	0	0	0
1	0	0	0	0	0	0	0	0
0	0	0	0	0	0	0	0	0
1	0	0	0	0	0	0	0	0
1	0	0	0	0	0	1	0	0
1	0	0	0	0	0	0	0	0
0	0	0	0	0	0	0	0	0
0	0	0	0	0	0	0	0	0
0	1	0	0	0	0	0	0	0
1	0	0	0	0	0	0	0	0
0	0	0	0	0	0	0	0	0
1	0	0	0	0	0	0	0	0
0	0	0	0	0	0	0	0	0
0	0	0	0	0	0	0	0	0
1	0	0	0	0	0	0	0	0
0	0	0	0	0	0	0	1	1
0	0	0	0	0	0	0	0	0
0	0	0	0	0	0	0	0	0
1	0	0	0	0	0	0	0	0
0	0	0	0	0	0	0	0	0
0	1	0	0	1	0	0	1	0
0	0	0	0	0	0	0	0	0
1	0	0	0	0	0	0	1	0
0	0	0	1	0	0	0	0	0
0	0	0	0	0	0	0	0	0

1	0	0	0	0	0	0	0	0
0	1	0	0	0	0	0	0	0
0	1	0	0	0	0	0	1	0
0	0	0	0	0	0	0	0	0
1	0	0	0	0	0	0	0	0
0	0	0	0	0	0	0	0	0
0	0	0	0	0	0	1	0	0
1	0	0	0	0	0	0	0	0
0	0	0	0	0	0	1	0	0
1	0	0	0	0	0	0	0	0
0	0	0	0	0	0	0	1	0
0	0	0	0	0	0	0	0	0
0	0	0	0	0	0	0	0	0
1	0	0	0	0	0	0	0	0
1	0	0	0	0	0	0	0	0
0	0	0	0	0	0	0	0	0
0	0	0	0	0	0	0	0	0
0	0	0	0	0	0	0	0	0
1	0	0	0	0	0	1	0	0
0	0	0	0	0	0	0	0	0
0	1	0	1	0	0	0	0	0
1	0	0	0	0	0	0	0	0
0	0	0	0	0	0	0	0	0
0	0	0	0	0	0	0	0	0
0	0	0	0	0	0	0	0	0
0	0	0	0	0	0	0	0	0
0	0	0	0	0	0	0	0	0
1	0	0	1	0	0	0	0	0
0	0	0	0	0	0	0	0	0
1	0	0	0	0	0	0	0	0
0	1	0	0	0	0	0	0	0
0	0	0	0	0	0	0	0	0
1	0	0	0	0	0	0	0	0
1	0	0	0	0	0	0	0	0
0	0	0	0	0	0	0	0	0
1	0	0	0	0	0	0	0	0
0	0	0	0	0	0	0	0	0
0	0	0	0	0	0	0	0	0
0	0	0	1	0	0	0	0	0
0	0	0	0	0	0	0	0	0
0	0	0	0	0	0	0	0	0
0	0	0	0	0	0	0	0	0
1	0	0	0	0	0	0	0	0
0	1	0	0	0	0	0	0	0
1	0	0	0	0	0	0	0	0
0	0	0	0	0	0	0	0	0
0	0	0	0	0	0	0	0	0
1	0	0	0	0	0	0	0	0
0	0	0	0	0	0	0	0	0
0	0	0	0	0	0	0	0	0
0	0	0	0	0	0	0	0	0
0	0	0	0	0	0	0	0	0
0	0	0	0	0	0	1	0	0
0	0	0	0	0	0	0	0	0
0	0	0	0	0	0	0	0	0
1	0	0	0	0	0	0	0	0

0	0	0	0	0	0	0	0	0
0	0	0	1	0	0	0	0	0
0	0	0	0	0	0	0	0	0
0	0	0	0	0	0	0	0	0
0	0	0	0	0	0	0	0	0
0	0	0	0	0	0	0	0	0
1	0	0	0	0	0	0	0	0
0	0	0	0	0	0	0	0	0
0	0	0	0	0	0	0	0	0
0	0	0	0	0	0	0	0	0
0	1	0	0	0	0	0	1	0
1	0	0	0	0	0	0	0	0
0	0	0	0	0	0	0	0	0
0	0	0	0	0	1	0	0	0
0	0	0	1	0	0	1	0	0
0	0	0	0	0	0	0	0	0
1	0	0	0	0	0	0	0	0
1	0	0	0	0	0	0	0	0
0	0	0	0	0	0	0	0	0
1	1	0	0	0	0	0	0	0
0	1	0	0	0	0	0	0	0
1	0	0	0	0	0	0	0	0
1	0	0	0	0	0	0	1	0
0	0	0	0	0	0	0	0	0
1	0	0	1	0	0	0	0	0
0	0	0	0	0	0	0	1	0
1	0	0	0	0	0	1	0	0
0	1	0	0	0	0	0	0	0
0	0	0	0	0	0	0	0	0
1	0	0	0	0	0	0	0	0
0	0	0	0	0	0	0	0	0
0	1	0	0	0	0	1	0	0
0	0	0	0	0	0	0	0	0
0	0	0	0	0	0	0	0	0
0	0	0	0	0	0	0	0	0
0	0	0	0	0	0	0	1	0
0	0	0	0	0	0	0	0	0
0	0	0	0	0	0	1	0	0
1	0	0	0	0	0	0	0	0
1	0	0	0	0	0	0	0	0
0	0	0	0	0	0	0	0	0
0	0	0	0	0	0	0	0	0
0	0	0	0	0	0	0	0	0
0	0	0	0	0	0	0	0	0
1	0	0	0	0	0	0	0	0
0	0	0	0	0	0	0	1	0
0	0	0	0	0	0	0	0	0
0	0	0	0	0	0	0	1	0
1	0	0	0	0	0	0	0	0
0	0	0	0	0	0	0	1	0
1	0	0	0	0	0	0	0	0
0	0	0	0	0	0	0	1	0
0	0	0	0	0	0	0	0	0
0	0	0	0	0	0	0	0	0
0	0	0	0	0	0	0	0	0
0	0	0	0	0	0	0	0	0

0	0	0	0	0	0	0	0	0
1	0	0	0	0	0	0	0	0
0	0	0	0	0	0	0	1	0
1	0	0	0	0	0	0	1	0
1	0	0	0	0	0	0	0	0
1	1	0	0	0	0	0	1	0
0	0	0	0	0	0	0	0	0
0	0	0	0	0	0	0	0	0
0	0	0	0	0	0	0	0	0
0	0	0	0	0	0	0	1	0
0	0	1	0	0	0	0	0	1
0	0	0	0	0	0	0	0	0
0	0	0	0	0	0	0	1	0
0	0	0	0	0	0	0	0	0
0	0	0	0	0	0	1	0	0
0	0	0	0	0	0	0	0	0
0	0	0	0	0	0	0	0	0
0	0	0	0	0	0	0	0	0
0	0	0	0	0	0	0	0	0
0	0	0	0	0	0	0	0	0
0	0	0	0	0	0	0	0	0
0	1	0	0	0	0	0	0	0
0	0	0	0	0	0	0	0	0
0	0	0	0	0	0	0	0	0
0	0	0	0	0	0	0	0	0
1	0	0	0	0	0	0	0	0
0	0	0	0	0	0	0	0	0
1	0	0	0	0	0	0	0	0
0	0	0	0	0	0	0	0	0
0	0	0	0	0	0	0	0	0
0	0	0	0	0	0	0	0	0
1	0	0	0	0	0	0	0	0
0	0	0	0	0	0	0	0	0
1	0	0	0	0	0	0	0	0
0	0	0	0	0	0	0	0	0
0	0	0	0	0	0	0	0	0
1	1	1	0	0	0	0	0	0
0	0	0	1	0	0	0	1	0
0	0	0	0	0	0	0	0	0
1	0	0	0	0	0	0	1	0
1	0	0	0	0	0	0	0	0
0	0	0	0	1	0	0	0	0
0	0	0	0	0	0	0	0	0
0	0	0	0	0	0	0	0	0
0	0	0	0	0	0	0	1	0
1	0	0	0	0	0	0	0	0
0	0	0	0	0	0	0	1	0
0	0	0	0	0	0	0	0	0
0	0	0	0	0	0	0	0	0
0	0	0	0	0	0	0	0	0
0	0	0	0	0	0	0	0	0
0	0	0	0	0	0	0	0	0
0	0	0	0	0	0	0	0	0
0	0	0	0	0	0	0	0	0
0	0	0	0	0	0	0	1	0
0	0	0	0	0	0	0	0	0

0	0	0	0	0	0	0	0	0
0	0	0	0	0	0	0	0	0
0	0	0	0	0	0	0	0	0
1	0	0	0	0	0	0	0	0
0	0	0	0	0	0	0	0	0
0	0	0	0	0	0	0	0	0
1	0	0	0	0	0	0	0	0
1	0	0	0	0	0	0	0	0
0	0	0	0	0	0	0	0	0
0	0	0	0	0	0	0	0	0
1	0	0	0	0	0	0	1	0
0	0	0	0	0	0	0	0	0
0	0	0	0	1	0	0	0	0
0	0	0	0	0	0	0	0	0
0	0	0	0	0	0	0	0	0
0	0	0	0	0	0	0	0	0
0	0	0	0	0	0	0	0	0
1	1	0	0	0	0	0	0	0
0	0	0	0	0	0	0	0	0
0	0	0	0	0	0	0	1	0
0	0	0	0	0	0	0	0	0
0	0	0	0	0	0	0	0	0
1	0	0	0	0	0	0	0	0
0	0	0	0	0	0	0	1	0
0	0	0	0	0	0	0	0	0
0	0	0	0	0	0	0	0	0
0	0	0	0	0	0	0	0	0
0	0	0	0	0	0	1	1	0
0	0	0	0	0	0	0	0	0
0	0	0	0	0	0	0	0	0
0	0	0	0	0	0	0	0	0
0	0	0	0	0	0	0	0	0
0	0	0	0	0	0	0	0	0
0	0	0	0	0	0	0	0	0
0	0	0	0	0	0	0	0	0
0	0	0	0	0	0	0	0	0
0	0	0	0	0	0	0	0	0
0	0	0	0	0	0	0	0	0
0	0	0	0	0	0	0	0	0
0	0	0	0	0	0	0	0	1
0	0	0	0	0	0	0	0	0
0	0	0	0	0	0	0	0	0
0	1	0	0	0	0	0	0	0
0	0	0	0	0	0	0	0	0
0	0	0	0	0	0	0	0	0
0	0	0	0	0	0	0	0	0
0	0	0	0	0	0	0	0	0
1	0	0	0	0	0	0	0	0
0	1	0	0	0	0	0	0	0
0	0	0	0	0	0	0	0	0
0	0	0	0	0	0	0	0	0
0	0	0	0	0	0	0	0	0
0	0	0	0	0	0	0	0	0
1	0	0	0	0	0	0	0	0
0	0	0	0	0	0	0	1	0
0	0	0	0	0	0	0	0	0

1	0	0	0	0	0	0	0	0
1	0	0	0	0	0	0	0	0
0	0	0	0	0	0	0	0	0
1	0	0	0	0	0	0	1	0
0	0	0	0	0	0	0	0	0
0	0	0	0	0	0	0	0	0
0	0	0	0	0	0	0	0	0
0	0	0	0	0	0	0	0	0
0	0	0	0	0	0	0	0	0
0	0	0	0	0	0	0	0	1
0	0	0	0	0	0	0	0	0
0	0	0	0	0	0	0	0	0
0	0	0	0	0	0	0	0	0
0	0	0	0	0	0	0	0	0
1	0	0	0	0	0	0	0	0
0	0	0	0	0	0	0	0	0
1	0	0	0	0	0	0	0	0
0	0	0	0	0	0	0	0	0
0	0	0	0	0	0	0	0	0
0	0	0	0	0	0	0	0	0
0	0	0	0	0	0	0	0	0
0	0	0	0	0	0	0	0	0
0	0	0	0	0	0	0	1	1
0	0	0	0	0	0	0	0	0
0	0	0	0	0	0	0	0	0
0	0	0	0	0	0	0	0	0
0	0	0	0	0	0	0	0	0
0	0	0	0	0	0	0	0	0
0	0	0	0	0	0	0	0	0
1	0	0	0	0	0	0	0	0
0	0	0	0	0	0	0	0	0
0	0	0	0	0	0	1	0	0
1	0	0	0	0	0	0	0	0
0	0	0	0	0	0	0	0	0
0	0	0	0	0	0	0	0	0
0	0	0	0	0	0	0	0	0
0	0	0	0	0	0	0	0	0
0	0	0	0	0	0	0	0	0
0	0	0	0	0	0	0	0	0
0	0	0	0	0	0	0	0	0
0	0	0	0	0	0	0	0	0
0	0	0	0	0	0	0	1	0
0	0	1	0	0	0	0	0	0
0	0	0	0	0	0	0	0	0
0	0	0	0	0	0	0	0	0
0	0	0	0	0	0	0	0	0
0	0	0	0	0	0	0	0	0
0	0	0	0	0	0	0	1	0
0	0	0	0	0	0	0	0	0
0	0	0	0	0	0	0	0	0
0	0	0	0	0	0	0	1	0
0	0	0	0	0	0	0	0	0
0	0	0	0	0	0	0	0	0
0	0	0	0	0	0	0	0	0
0	0	0	0	0	0	0	0	0
1	0	0	0	0	0	0	0	0

0	0	0	0	0	0	0	0	0
0	0	0	0	1	1	0	0	0
0	0	0	0	0	0	0	0	0
1	0	0	0	0	0	0	0	0
0	0	0	0	0	0	0	0	0
0	0	0	0	0	0	0	0	0
0	0	0	0	0	0	0	0	0
0	0	0	0	0	0	0	0	0
0	0	0	0	0	0	0	0	0
0	0	0	0	0	0	0	1	0
0	0	0	0	0	0	0	0	0
0	0	0	0	0	0	0	0	0
1	0	0	0	0	0	0	0	0
0	0	0	0	0	0	0	0	0
0	0	0	0	0	0	0	1	0
0	0	0	0	0	0	0	1	0
0	0	0	0	0	0	0	0	0
0	0	0	0	0	0	0	1	0
1	0	0	0	0	0	0	0	0
0	0	0	0	0	0	0	0	0
0	0	0	0	0	0	0	1	0
0	0	0	0	0	0	0	0	0
1	0	0	0	0	0	0	0	0
1	0	0	0	0	0	0	0	0
0	0	0	0	0	0	0	1	0
1	0	0	0	0	0	0	0	0
0	0	0	0	0	0	0	0	0
0	0	0	0	0	0	0	0	0
0	0	0	0	0	0	0	0	0
0	0	0	0	0	0	0	0	0
0	0	0	0	0	0	0	0	0
0	0	0	0	0	0	0	0	0
0	0	0	0	1	0	0	0	0
0	0	0	0	0	0	1	0	0
0	0	0	0	0	0	0	0	0
0	0	1	0	0	0	0	0	0
0	0	0	0	0	0	0	0	0
0	0	0	0	0	0	0	1	0
1	0	0	0	0	0	0	0	0
0	0	0	0	0	0	0	0	0
0	0	0	0	0	0	0	0	0
1	1	0	0	0	0	0	0	0
0	0	0	0	0	0	0	0	0
0	0	0	0	0	0	0	0	0
0	0	0	0	0	0	0	0	0
0	0	0	0	0	0	0	0	0
0	0	0	0	0	0	0	0	0
0	0	0	0	0	0	0	0	0
0	0	0	0	0	0	0	0	0
1	0	0	0	0	0	0	0	0
0	0	0	0	0	0	0	1	0
0	0	0	0	0	0	0	0	0
0	0	0	0	0	0	0	0	0
0	0	0	0	0	0	0	0	0
0	0	0	0	0	0	0	0	0
0	0	0	0	0	0	0	0	0

0	0	0	1	0	0	0	0	0
0	0	0	0	0	0	0	0	0
0	0	0	0	0	0	0	0	0
0	0	0	0	0	0	0	0	0
0	1	0	0	0	0	0	0	0
0	0	0	1	0	0	0	0	0
0	0	0	0	0	0	0	1	0
0	0	0	0	0	0	0	0	0
0	0	0	0	0	0	0	0	0
1	0	0	0	0	0	0	1	0
0	0	0	0	0	0	0	0	0
1	0	0	0	0	0	0	1	0
0	0	0	0	0	0	0	0	0
0	0	0	0	0	0	0	0	0
1	0	0	0	0	0	0	0	0
0	0	0	0	0	0	0	1	0
0	0	0	0	0	0	0	0	0
0	0	0	0	0	0	0	1	0
0	0	0	0	0	0	1	1	0
0	1	0	0	0	0	0	0	0
0	0	0	0	0	0	0	0	0
0	0	0	0	0	0	0	0	0
0	0	0	0	0	0	0	0	0
0	0	0	0	0	0	0	0	0
0	0	0	0	0	0	0	0	0
0	0	0	0	0	0	0	0	0
0	0	0	1	0	0	0	0	0
1	0	0	0	0	0	0	0	0
1	0	0	0	0	0	0	1	0
1	0	0	0	0	0	0	0	0
0	0	0	0	0	0	0	0	0
0	0	0	0	0	0	0	1	0
0	0	0	0	0	0	0	0	0
1	0	0	0	0	0	0	0	0
0	0	0	0	0	0	0	0	0
0	0	0	0	0	0	0	0	0
0	0	0	0	0	0	0	0	0
0	0	0	0	0	0	0	0	0
0	0	0	0	0	0	0	0	0
0	0	0	0	0	0	0	0	0
0	0	0	0	1	0	0	0	0
0	0	0	0	0	0	0	0	0
0	0	0	0	0	0	0	0	0
0	0	0	0	0	0	0	0	0
0	0	0	0	0	0	0	0	0
0	0	0	0	0	0	0	0	0
0	0	0	0	0	0	0	0	0
0	0	0	0	0	0	1	1	0
0	0	0	0	0	0	0	1	0
1	0	0	0	0	0	0	1	0
0	0	0	0	0	0	1	0	0
0	0	0	0	0	0	0	0	0
0	0	0	0	0	0	0	1	0
0	0	0	0	0	0	0	0	0
0	0	0	0	0	0	0	0	0
0	0	1	0	0	0	0	1	0

0	0	0	0	0	0	0	0	0
0	0	0	0	0	0	0	0	0
0	0	0	0	0	0	0	0	0
0	0	0	0	1	0	0	0	0
0	0	0	0	0	0	0	1	0
0	0	0	0	0	0	0	0	0
1	0	0	0	0	0	0	0	0
0	0	0	0	0	0	0	0	0
0	0	0	0	0	0	0	0	0
0	0	0	0	0	0	0	0	0
0	0	0	0	0	0	0	0	0
1	0	0	0	0	0	0	0	0
1	0	0	0	0	0	0	0	0
0	0	0	0	0	0	0	0	0
0	0	0	0	0	0	0	1	0
1	0	0	0	0	0	0	1	0
1	0	0	0	0	0	0	0	0
0	0	0	0	0	0	0	0	0
0	0	0	0	0	0	0	0	0
0	0	0	0	0	0	0	0	0
0	0	0	0	0	0	0	1	0
0	0	0	0	0	0	0	0	0
0	0	0	0	0	0	0	0	0
0	0	0	0	0	0	0	0	0
0	0	0	0	0	0	0	1	0
0	0	0	0	0	0	0	0	0
1	0	0	0	0	0	0	0	0
0	0	0	0	0	0	0	0	0
0	0	0	0	0	0	0	0	0
0	0	0	0	0	0	0	0	0
0	0	0	0	0	0	0	0	0
0	0	0	0	0	0	0	0	0
0	0	0	0	0	0	0	0	0
0	1	0	0	0	0	0	0	0
0	0	0	0	0	0	0	0	0
1	0	0	0	0	0	1	0	0
0	0	0	0	1	0	0	0	0
0	0	0	0	0	0	0	0	0
0	0	0	0	0	0	0	1	0
0	0	0	0	0	0	0	0	0
1	0	0	0	0	0	0	0	0
0	0	0	0	0	0	0	0	0
1	0	0	0	0	0	0	0	0
0	1	0	0	0	0	0	0	1
1	0	0	0	0	0	0	0	0
0	0	0	0	0	0	0	0	0
0	0	0	0	0	0	0	0	0
1	0	0	0	0	0	0	0	0
1	0	0	0	0	1	0	0	1
0	0	0	0	0	0	0	0	0
0	0	0	0	0	0	0	0	0
0	0	0	0	0	0	0	0	0
0	0	0	0	0	0	0	0	0
0	0	0	0	0	0	0	0	0
1	0	0	0	0	0	0	0	0
0	0	0	0	0	0	0	0	0

0	0	0	0	0	0	1	0	0
0	0	0	0	0	0	0	0	0
0	0	0	0	0	0	0	0	0
0	0	0	0	0	0	0	0	0
0	1	0	0	0	0	0	1	0
0	0	0	0	0	0	0	0	0
0	0	0	0	1	0	0	0	0
0	0	0	0	0	0	0	1	0
0	0	0	0	0	0	0	0	0
1	0	0	0	0	0	0	0	0
0	0	0	0	0	0	0	0	0
0	1	0	0	0	0	0	0	0
1	0	0	0	0	0	0	0	0
0	0	0	0	0	0	0	0	0
0	1	0	0	0	0	1	0	0
1	0	0	0	0	0	0	0	0
1	0	0	0	0	0	0	0	0
0	1	0	0	0	0	0	0	0
0	0	0	0	0	0	0	0	0
0	1	0	0	0	0	0	0	0
1	0	0	0	0	0	0	0	0
1	0	0	0	0	0	1	0	0
1	0	0	0	0	0	0	0	0
0	1	0	0	0	0	0	0	0
0	0	0	0	0	0	0	0	0
0	0	0	0	0	0	0	0	1
0	0	0	0	0	0	0	0	0
0	0	0	0	0	0	0	0	0
1	0	0	0	0	0	0	0	0
0	0	0	0	0	0	0	0	0
0	0	0	0	0	0	0	0	0
0	0	0	0	0	0	0	0	0
0	0	0	0	0	0	0	0	0
0	0	0	0	0	0	0	0	0
1	0	0	0	0	0	0	0	0
0	0	0	0	0	0	0	0	0
0	0	0	0	0	0	0	0	0
0	0	0	0	0	0	0	0	0
0	0	0	0	0	0	0	0	0
0	0	0	0	0	0	0	0	0
0	0	0	0	0	0	0	0	0
0	0	0	0	0	0	0	0	0
0	1	0	0	0	0	0	0	0
1	0	0	0	0	0	0	0	0
0	0	0	0	1	0	0	0	0
0	1	0	0	0	0	0	0	0
0	0	0	0	0	0	0	0	0
0	0	0	0	0	0	0	0	0
1	0	0	0	0	0	0	0	0
0	0	0	0	0	0	0	0	0
0	0	0	0	0	0	0	0	0
0	0	0	0	0	0	0	0	0
0	0	0	0	0	0	0	0	0
0	0	0	0	0	0	0	0	0
0	0	0	0	0	0	0	0	0
0	0	0	0	0	0	0	1	0

1	0	0	0	0	0	0	0	0
0	0	0	0	0	0	0	1	0
0	0	0	0	0	0	0	0	0
0	0	0	0	0	0	0	0	0
0	0	0	0	0	0	0	0	0
0	1	0	0	0	0	0	0	0
1	0	0	0	0	0	0	0	0
0	1	0	0	0	0	0	0	0
0	1	0	0	0	0	0	0	0
0	0	0	0	0	0	0	0	0
0	0	0	0	0	0	0	1	0
0	0	0	0	0	0	0	0	0
1	0	0	0	0	0	0	0	0
1	0	0	0	0	0	0	0	0
1	0	0	0	0	0	0	0	0
1	0	0	0	0	0	0	0	0
1	1	0	1	0	0	0	0	0
1	0	0	0	0	0	0	0	0
1	0	0	0	0	0	0	0	0
0	0	0	0	0	0	0	1	0
0	0	0	0	0	0	0	0	0
0	0	0	0	0	0	0	0	0
0	0	0	0	0	0	0	0	0
0	0	0	0	0	0	0	0	0
0	0	0	0	0	0	0	0	0
0	1	0	0	0	0	0	0	0
1	0	0	0	1	0	0	0	0
0	0	0	0	1	0	0	0	0
0	0	0	0	1	0	0	0	0
0	0	0	0	0	0	0	0	0
0	0	0	0	0	0	0	0	0
0	1	0	0	0	0	0	0	0
0	0	0	0	0	0	0	1	0
0	0	0	0	0	0	0	0	0
0	0	0	0	0	0	0	0	0
0	0	0	0	0	0	0	0	0
0	0	0	0	0	0	0	0	0
0	0	0	0	0	0	0	0	0
0	0	0	0	0	0	0	0	0
0	0	0	0	0	0	0	0	0
0	0	0	0	1	0	0	0	0
1	0	0	0	0	0	0	0	0
0	0	0	0	0	0	0	0	0
0	0	0	0	0	0	0	0	0
0	0	0	0	0	0	0	0	0
0	0	0	0	0	0	0	0	0
0	0	0	0	0	0	0	0	0
0	0	0	0	0	0	0	1	0
0	0	0	0	1	0	0	0	0
0	0	0	0	0	0	0	1	0
0	0	0	0	0	0	0	0	0
0	0	0	1	0	0	0	0	0
0	0	0	0	0	0	0	0	0
1	0	0	0	0	0	0	0	0
0	0	0	0	0	0	0	0	0
0	0	0	0	0	0	0	1	0

0	0	0	0	0	0	0	1	0
0	0	0	0	0	0	1	0	0
1	0	0	0	0	0	1	0	0
0	0	0	0	0	0	0	0	0
0	0	0	0	0	0	0	0	0
0	0	0	0	1	0	0	0	0
0	0	0	0	0	0	0	0	0
0	1	0	0	0	0	0	1	0
0	0	0	0	0	0	0	0	0
1	0	0	0	0	0	0	1	0
0	0	0	0	0	0	0	0	0
0	0	0	0	0	0	0	0	0
0	0	0	0	0	0	0	0	0
1	0	0	0	1	0	0	0	0
0	0	0	0	0	0	0	0	0
0	0	0	0	0	0	0	1	0
0	0	0	0	0	0	0	0	0
0	0	0	0	0	0	0	0	0
0	0	0	0	0	0	0	0	0
0	0	0	0	0	0	0	0	0
0	0	0	0	0	0	0	0	0
0	1	0	0	0	0	0	0	0
0	0	0	0	0	0	1	0	0
0	0	0	0	0	0	0	0	0
0	0	0	0	0	0	0	0	0
0	0	0	0	0	0	0	0	0
0	0	0	0	0	0	0	0	0
0	0	0	0	0	0	0	0	0
0	1	0	0	0	0	0	1	0
0	1	0	0	0	0	0	0	0
0	0	0	0	0	0	0	0	0
0	0	0	0	0	0	0	0	0
0	0	0	0	1	0	0	0	0
0	0	0	0	1	0	0	0	0
0	0	0	0	0	0	0	0	0
0	0	0	0	0	0	0	0	0
0	0	0	0	0	0	0	0	0
0	0	0	0	0	0	0	0	0
0	0	0	0	0	0	0	0	0
0	0	0	0	0	0	0	0	0
1	0	0	0	0	0	0	0	0
1	0	0	0	0	0	1	0	0
0	0	0	0	0	0	0	0	0
0	0	0	0	0	0	0	0	0
0	0	0	0	0	0	0	0	0
0	0	0	0	0	0	0	0	0
0	0	0	0	0	0	0	1	0
0	0	0	0	0	0	0	0	0
0	0	0	0	0	0	0	0	0
1	0	0	0	0	0	0	0	0
1	0	0	0	0	0	0	0	0
0	0	0	0	0	0	0	0	0
0	0	0	0	0	0	0	0	0
0	1	0	0	0	0	0	0	0
1	0	0	0	0	0	0	0	0
0	0	0	0	0	0	0	0	0

0	0	0	1	0	0	0	0	0
1	0	0	0	0	0	0	0	0
0	0	0	0	0	0	0	0	0
0	0	0	0	0	0	0	0	0
0	0	0	0	0	0	0	0	0
0	0	0	0	1	0	0	1	0
0	0	0	0	0	0	0	0	0
0	0	0	0	0	0	0	0	0
0	1	0	0	0	0	0	0	0
1	0	0	0	1	0	0	0	0
1	0	0	0	0	0	0	0	0
0	0	0	0	0	0	0	0	0
0	0	0	0	0	0	0	0	0
0	0	0	0	0	0	0	0	0
0	0	0	0	0	0	0	0	0
0	0	0	0	0	0	0	0	0
0	0	0	0	0	0	0	0	0
0	0	0	0	0	0	0	0	0
0	1	0	0	0	0	0	0	0
0	0	0	0	0	0	0	0	0
0	0	0	0	0	0	0	0	0
0	0	0	0	0	0	0	0	0
0	0	0	0	0	0	0	0	0
0	1	0	0	0	0	0	1	0
0	0	0	0	0	0	0	0	0
0	1	0	0	0	0	0	0	0
1	0	0	0	1	0	0	0	0
0	0	0	0	0	0	0	0	0
0	0	0	0	0	0	0	0	0
0	0	0	0	0	0	0	0	0
0	0	0	0	0	0	0	0	0
0	0	0	0	0	0	0	0	0
0	0	0	0	1	0	0	0	0
0	0	0	0	0	0	0	0	0
0	1	0	0	0	0	0	0	0
0	0	0	0	0	0	0	0	0
0	0	0	0	0	0	0	0	0
1	0	0	0	0	0	0	0	0
0	0	0	0	0	0	0	1	0
0	0	0	0	0	0	0	0	0
0	0	0	0	0	0	0	0	0
0	0	0	0	0	0	0	0	0
0	0	0	0	0	0	0	0	0
0	0	0	0	0	0	0	1	0
0	0	0	0	0	0	0	0	0
0	0	0	0	0	0	0	0	0
0	0	0	0	0	0	0	0	0
0	0	0	0	0	0	0	0	0
0	0	0	0	0	0	0	0	0
0	0	0	0	0	0	0	0	0
0	0	0	0	0	0	0	0	0
0	0	0	0	0	0	0	0	0
0	0	0	0	0	0	0	0	0
0	0	0	0	0	0	0	0	0
0	0	0	0	0	0	0	0	0
0	0	0	0	0	0	0	0	0

0	0	0	0	0	0	0	0	0
1	0	0	0	0	0	0	0	0
0	0	0	0	1	0	0	0	0
0	0	0	0	0	0	0	0	0
0	1	0	0	0	0	0	1	0
0	0	0	0	0	0	0	0	0
0	0	0	0	1	0	0	1	0
0	0	0	0	0	0	0	0	0
0	0	0	0	0	0	0	0	0
0	0	0	0	1	0	0	0	0
0	0	0	0	0	0	0	0	0
0	0	0	0	0	0	0	0	0
0	0	0	0	0	0	0	0	0
0	0	0	0	0	0	0	0	0
0	0	0	0	0	0	0	0	0
0	1	0	0	0	0	0	0	0
0	0	0	0	0	0	0	0	0
0	0	0	0	0	0	0	0	0
0	0	0	0	0	0	0	0	0
0	0	0	0	0	0	0	0	0
1	0	0	0	0	0	0	0	0
0	0	0	0	1	0	0	0	0
0	0	0	0	1	0	0	0	0
1	0	0	0	1	0	0	0	0
1	0	0	1	0	0	0	0	0
0	0	0	0	0	0	0	0	0
0	0	0	0	0	0	0	0	0
0	0	0	0	0	0	0	0	0
0	0	0	0	0	0	0	0	0
1	0	0	0	0	0	0	0	0
0	0	0	0	0	0	0	0	0
0	0	0	0	0	0	0	0	0
0	0	0	0	0	0	0	0	0
0	0	0	0	0	0	0	0	0
0	0	0	0	0	0	0	0	0
0	0	0	0	0	0	0	0	0
0	0	0	0	0	0	0	0	0
0	0	0	0	0	0	0	0	0
0	0	0	0	0	0	0	0	0
0	1	0	0	0	0	0	0	0
0	0	0	0	0	0	0	0	0
0	0	0	0	0	0	0	0	0
0	0	0	0	0	0	0	0	0
0	0	0	0	0	0	0	0	0
0	0	0	0	0	0	0	0	0
0	0	0	0	0	0	0	0	0
0	0	0	0	0	0	0	0	0
0	0	0	0	0	0	0	0	0
0	1	1	0	0	0	0	0	1
0	0	0	0	0	0	0	0	0
1	0	0	0	0	0	0	0	0
1	0	0	0	0	0	0	0	0
0	1	0	0	0	0	0	0	0
0	0	0	0	0	0	0	0	0
0	0	0	0	0	0	0	0	0
0	0	0	0	0	0	0	0	0

0	0	0	0	0	0	0	0	0
1	0	0	0	1	0	0	1	0
0	0	0	0	0	0	0	0	0
0	0	0	0	0	0	0	1	0
1	0	0	0	0	0	0	0	0
0	0	0	0	0	0	0	0	0
0	0	0	0	0	0	0	0	0
0	0	0	0	0	0	0	1	0
0	0	0	0	0	0	0	0	0
1	0	0	0	0	0	0	0	0
0	0	0	0	0	0	0	0	0
0	0	0	0	0	0	0	0	0
0	0	0	0	1	0	0	0	0
0	0	0	0	0	0	0	0	0
0	0	0	0	0	0	0	1	0
0	0	0	0	0	0	0	0	0
0	0	0	0	0	0	0	0	0
0	0	0	0	0	0	0	0	0
0	0	0	0	0	0	0	0	0
0	0	0	0	0	0	0	0	0
0	0	0	0	0	0	0	0	0
0	0	0	0	0	0	0	0	0
0	0	0	0	0	0	0	0	0
1	0	0	0	0	0	0	1	0
0	0	0	0	0	0	0	0	0
0	0	0	1	0	0	0	1	1
1	0	0	0	0	0	0	0	0
0	0	0	0	0	0	0	0	0
0	0	0	0	0	0	0	0	0
0	0	0	0	1	0	0	0	0
0	0	0	0	0	0	0	0	0
0	0	0	0	0	0	0	0	0
0	0	0	0	0	0	0	0	0
0	0	0	0	0	0	0	0	0
1	0	0	0	1	0	0	0	0
0	0	0	0	0	0	0	0	0
0	0	0	0	0	0	0	0	0
1	0	0	0	0	0	0	0	0
0	0	0	0	0	0	0	0	0
0	0	0	0	0	0	0	0	0
0	0	0	0	0	0	0	0	0
0	0	0	0	0	0	0	0	1
0	0	0	0	0	0	0	0	0
0	0	0	0	0	0	0	0	0
0	0	0	0	0	0	0	0	0
0	0	0	0	0	0	0	0	0
1	0	0	0	0	0	0	0	0
0	0	0	0	0	0	0	0	0
1	1	0	0	0	0	0	0	0
0	0	0	0	0	0	0	0	0
0	0	0	0	0	0	0	0	0
0	0	0	0	0	0	0	1	0
0	0	0	0	0	0	0	0	0
0	0	0	0	0	0	0	0	0
0	0	0	0	0	0	0	0	0
1	0	0	0	0	0	0	0	0

0	0	0	0	0	0	0	0	0
0	0	0	0	0	0	0	0	0
1	0	0	0	0	0	0	0	0
1	0	0	0	0	0	0	0	0
0	0	0	0	0	0	0	0	0
0	0	0	0	0	0	0	0	0
1	0	0	0	0	0	0	0	0
0	0	0	0	0	0	0	0	0
0	0	0	0	0	0	0	0	0
0	0	0	0	0	0	0	1	1
1	0	0	0	0	0	0	0	0
0	0	0	0	0	0	0	0	0
1	0	0	0	0	0	0	0	0
0	0	0	0	0	0	0	0	0
0	1	0	0	0	0	0	0	0
0	0	0	0	0	0	0	0	0
1	0	0	0	0	0	0	0	0
0	0	0	0	0	0	0	0	0
0	0	0	0	0	0	0	0	0
0	0	0	0	0	0	0	0	0
0	0	0	0	0	0	0	0	0
1	0	0	0	0	0	0	0	0
0	0	0	0	0	0	0	0	0
0	0	0	0	0	0	0	0	0
0	0	0	0	0	0	0	0	0
0	0	0	0	0	0	0	0	0
0	0	0	0	0	0	0	0	0
1	0	1	0	0	0	0	0	0
0	0	0	0	0	0	0	0	0
0	0	0	0	0	0	0	0	0
0	0	0	0	0	0	0	0	0
0	0	0	0	0	0	0	0	0
0	0	0	0	0	0	0	0	0
0	0	0	0	0	0	0	0	0
0	0	0	0	0	0	0	0	0
0	0	0	0	0	0	0	0	0
0	0	0	0	0	0	0	0	0
0	0	0	0	0	0	0	0	0
1	0	1	0	0	0	0	0	0
0	0	0	0	0	0	0	0	0
0	0	0	0	0	0	0	0	0
0	0	0	0	0	0	0	0	0
0	0	0	0	0	0	0	0	0
0	0	0	0	0	0	0	0	0
0	0	0	0	0	0	0	0	0
0	0	0	0	0	0	0	0	0
0	0	0	0	0	0	0	0	0
1	0	0	0	0	0	0	0	0
0	0	0	0	0	0	0	0	0
0	0	0	0	0	0	0	0	0
0	0	0	0	0	0	0	0	0
0	0	0	0	0	0	0	0	0
0	0	0	0	0	0	0	1	0
0	0	0	0	0	0	0	0	0
0	0	0	0	0	0	0	0	0
0	0	1	0	0	0	0	1	0

0	0	0	0	0	0	0	0	0
1	0	0	0	0	0	0	0	0
0	0	0	0	0	0	0	0	0
0	0	0	0	0	0	0	0	0
0	0	0	0	0	0	0	0	0
0	0	1	0	0	0	0	1	0
0	0	0	0	0	0	0	0	0
0	0	0	0	0	0	0	0	0
0	0	1	0	0	0	0	0	0
1	0	0	0	0	0	0	0	0
1	0	0	0	0	0	0	1	0
0	0	0	0	0	0	0	0	0
0	0	0	0	0	0	0	0	0
0	0	0	0	0	0	0	0	0
0	0	0	0	0	0	1	1	0
0	0	0	0	0	0	0	0	0
0	0	0	0	0	0	0	0	0
0	0	0	0	0	0	0	0	0
0	0	0	0	0	0	0	0	0
0	0	0	0	0	0	0	0	0
1	0	0	0	0	0	0	0	0
0	0	0	0	0	0	0	0	0
0	0	0	0	0	0	0	0	0
0	1	0	0	1	0	0	0	0
0	0	0	0	0	0	0	0	0
1	0	0	0	0	0	0	0	0
0	0	0	0	0	0	0	0	0
0	0	0	0	0	0	0	0	0
0	0	0	0	0	0	0	0	0
0	0	0	0	0	0	0	0	0
1	0	1	0	0	0	0	0	0
0	0	0	0	0	0	0	0	0
0	0	0	0	0	0	0	0	0
0	0	0	0	0	0	0	0	0
0	0	0	0	0	0	0	0	0
0	0	0	0	0	0	0	0	0
0	1	1	0	0	0	0	0	0
1	0	0	0	0	0	0	0	0
0	1	0	0	0	0	0	0	0
0	0	0	0	0	0	0	0	0
0	0	0	0	1	0	0	0	0
0	0	0	0	0	0	1	0	0
0	0	0	0	0	0	0	0	0
0	0	0	0	0	0	0	0	0
0	0	0	0	0	0	0	0	0
0	0	0	0	0	0	0	0	0
1	0	0	0	0	0	0	0	0
0	0	1	0	0	0	0	0	0
0	0	0	0	0	0	0	0	0
1	1	0	0	0	0	0	0	0
0	0	0	0	0	0	0	0	0
0	0	0	0	0	0	0	0	0
1	0	0	0	0	0	0	0	0
0	0	0	0	0	0	0	1	0
0	0	0	0	0	0	0	0	0
0	0	0	0	0	0	0	0	0

0	0	0	0	0	0	0	0	0
1	0	0	0	0	0	0	0	0
0	0	0	0	1	0	0	0	0
0	0	0	0	0	0	0	0	0
0	0	0	0	0	0	0	0	0
0	0	0	0	0	0	0	0	0
1	0	0	0	0	0	0	0	0
1	0	0	0	0	0	0	0	0
0	0	0	0	0	0	0	0	0
0	1	0	0	0	0	0	0	0
1	0	0	0	0	0	0	0	0
0	1	0	0	0	0	0	0	0
0	0	0	0	0	0	0	0	0
1	0	0	0	0	0	0	0	0
0	0	0	0	0	0	0	0	0
0	0	0	0	0	0	0	0	0
0	0	0	0	1	0	0	1	0
1	1	0	0	0	0	0	0	0
0	0	0	0	0	0	1	0	0
0	0	0	0	0	0	0	0	0
0	0	0	0	0	0	0	0	0
1	0	0	0	0	0	0	0	0
0	0	0	0	0	0	0	1	0
0	0	0	0	0	0	0	0	0
0	0	0	0	0	0	0	0	0
1	0	0	0	0	0	0	0	0
0	0	0	0	1	0	0	1	0
0	0	0	0	0	0	0	1	0
0	0	0	0	0	0	0	0	0
0	0	0	0	0	0	0	0	0
0	0	0	0	0	0	0	0	0
0	0	0	0	0	0	0	0	0
0	0	0	0	0	0	0	0	0
1	0	0	0	0	0	0	0	0
0	0	0	0	0	0	0	0	0
1	0	0	0	0	0	0	0	0
0	0	0	0	0	0	0	0	0
0	1	0	0	0	0	0	0	0
0	0	0	0	0	0	0	0	0
0	0	0	0	0	0	0	1	0
0	0	0	0	0	0	0	0	0
0	0	0	0	0	0	0	0	0
0	1	0	0	0	0	0	0	0
1	0	0	0	0	0	0	1	0
0	0	0	0	0	0	0	0	0
0	0	0	0	0	0	0	0	0
0	0	0	0	0	0	1	0	0
0	0	0	0	0	0	0	0	0
0	0	0	0	0	0	0	0	0
0	0	0	0	0	0	0	0	0
0	0	0	0	0	0	0	0	0
0	0	0	0	0	0	0	0	0
0	0	0	0	0	0	0	0	0
0	0	0	0	0	0	0	0	0
0	0	0	0	0	0	0	0	0
0	0	0	0	0	0	0	0	0

0	0	0	0	0	0	0	0	0
0	0	0	0	0	0	0	0	0
0	0	0	0	0	0	0	0	0
0	0	0	0	0	0	0	0	0
0	0	0	0	0	0	0	0	0
0	0	0	0	0	0	0	0	0
0	0	0	0	0	0	0	0	0
0	0	0	0	0	0	0	0	0
0	0	0	0	0	0	0	0	0
0	0	0	0	0	0	0	1	0
0	0	0	0	0	0	0	0	0
0	0	0	0	0	0	0	0	0
0	0	0	0	0	0	0	0	0
0	0	0	0	0	0	0	0	0
0	1	0	0	0	0	0	0	0
0	0	0	0	0	0	0	0	0
1	0	0	0	0	0	0	0	0
0	1	0	0	0	0	0	0	0
0	0	0	0	0	0	0	0	0
0	0	0	0	0	0	0	0	0
0	0	0	0	0	0	0	0	0
0	0	0	0	0	0	0	0	0
0	0	0	0	0	0	0	0	0
0	0	0	0	0	0	0	0	0
0	0	0	0	0	0	0	0	0
0	0	0	0	0	0	0	0	0
0	0	0	0	0	0	0	0	0
0	0	0	0	0	0	0	0	0
0	0	0	0	0	0	0	0	0
0	0	0	0	0	0	0	0	0
0	0	0	0	0	0	0	0	0
0	0	0	0	0	0	0	0	0
0	0	0	0	0	0	0	0	0
0	0	0	0	0	0	0	1	0
0	0	0	0	0	0	0	0	0
0	0	0	0	0	0	0	0	0
0	0	0	0	0	0	0	0	0
0	0	0	0	0	0	0	0	0
1	0	0	0	0	0	0	0	0
1	0	0	0	0	0	0	0	0
0	0	0	0	0	0	0	0	0
0	0	0	0	0	0	0	0	0
0	0	0	0	0	0	0	0	0
0	0	0	0	0	0	0	0	0
0	0	0	0	0	0	0	0	0
0	0	0	0	0	0	0	0	0
0	0	0	0	0	0	0	0	0
0	1	0	0	0	0	0	0	0
0	1	0	0	0	0	1	0	0
0	0	0	0	0	0	0	0	0
0	0	0	0	0	0	0	0	0
0	0	0	0	0	0	0	0	0
0	1	0	0	0	0	0	0	0
0	0	1	0	0	0	0	0	0
0	0	0	0	0	0	0	0	0
0	0	0	0	0	0	0	0	0

0	0	0	0	0	0	0	0	0
0	0	0	0	0	0	0	0	0
0	0	1	0	0	0	0	0	0
0	0	0	0	0	0	0	0	0
0	0	0	0	0	0	0	0	0
1	0	0	0	0	0	0	0	0
0	0	0	0	0	0	0	1	0
0	0	0	0	0	0	0	0	0
0	0	0	0	0	0	0	0	0
0	0	0	0	0	0	0	0	0
0	0	0	0	0	0	0	0	0
0	0	0	0	0	0	0	0	0
0	0	0	0	0	0	0	0	0
0	0	0	0	0	0	0	0	0
0	1	0	0	0	0	0	0	0
1	0	0	0	0	0	0	0	0
0	0	0	0	0	0	0	0	0
1	0	0	0	0	0	0	0	0
0	0	0	0	0	0	0	0	0
0	0	0	0	0	0	1	0	0
0	0	0	0	0	0	0	0	0
1	1	0	0	0	0	0	0	0
0	1	0	0	0	0	0	0	0
0	0	0	0	0	0	0	0	0
0	0	0	0	0	0	0	0	0
0	0	0	0	0	0	0	0	0
0	0	0	0	0	0	0	0	0
0	0	0	0	0	0	0	0	0
0	0	0	0	0	0	0	0	0
0	0	0	0	0	0	0	0	0
0	0	0	0	0	0	0	0	0
0	0	0	0	0	0	0	0	0
0	0	0	0	0	0	0	0	0
0	0	0	0	0	0	0	0	0
0	0	0	0	0	0	0	0	0
0	0	0	0	0	0	0	0	0
0	0	0	0	1	0	0	0	0
0	0	0	0	0	0	0	0	0
0	0	0	0	0	0	0	0	0
0	0	0	0	0	0	0	0	0
0	0	0	0	0	0	0	0	0
0	0	0	0	0	0	0	0	0
1	0	0	0	0	0	0	0	0
0	0	0	0	0	0	0	0	0
1	0	0	0	0	0	0	1	0
0	0	0	0	0	0	1	0	0
1	0	0	0	0	0	0	0	0
0	0	0	0	0	0	0	0	0
0	0	0	0	0	0	0	0	0
0	0	0	0	0	0	1	1	0
0	0	0	0	0	0	0	0	1
0	0	0	0	0	0	0	0	0
0	0	0	0	0	0	0	0	0
0	0	0	0	0	0	0	0	0
1	0	0	0	0	0	0	0	0
0	0	0	0	0	0	0	1	0

0	0	0	0	0	0	0	0	0
0	0	0	0	0	0	0	0	0
0	0	0	0	0	0	0	0	0
0	0	0	0	0	0	0	0	0
0	0	0	0	0	0	0	0	0
0	0	0	0	0	0	0	0	0
0	0	0	0	0	0	0	0	0
0	0	0	0	0	0	0	0	0
0	0	0	0	0	0	0	0	0
1	0	0	0	0	0	0	0	0
0	0	0	0	0	0	0	0	1
0	0	0	1	0	0	0	0	0
0	0	0	0	0	0	0	0	0
0	0	0	0	0	0	0	0	0
0	0	0	0	0	0	0	0	0
0	0	0	0	0	0	0	0	0
0	0	0	0	0	0	0	0	0
0	0	0	0	0	0	0	0	0
0	0	0	0	0	0	0	0	0
0	0	0	0	0	0	0	0	0
0	0	0	0	0	0	0	0	0
1	0	0	0	0	0	0	1	0
1	1	0	0	0	0	0	0	0
0	0	0	0	0	0	0	0	0
0	0	0	0	0	0	0	0	0
0	0	0	0	0	0	0	0	0
0	0	0	0	0	0	0	0	0
0	0	0	0	0	0	0	0	0
0	0	0	0	0	0	0	0	0
0	1	0	0	0	0	0	1	0
0	1	0	0	0	0	1	1	0
0	0	0	0	0	0	0	0	0
0	0	0	0	0	0	0	0	0
0	0	0	0	0	0	0	0	0
0	0	0	0	0	0	0	0	0
0	0	0	0	0	0	0	0	0
0	0	0	0	0	0	0	0	0
0	1	0	0	0	0	0	0	0
0	0	0	0	0	0	0	0	0
0	0	0	0	0	0	1	0	0
1	0	0	0	0	0	0	0	0
0	0	0	0	0	0	0	0	0
1	0	0	0	0	0	0	0	0
0	0	0	0	0	0	0	1	0
1	0	0	0	0	0	0	0	0
1	0	0	0	0	0	0	0	0
0	0	0	0	0	0	0	0	0
0	0	0	0	0	0	0	0	0
0	0	0	0	0	0	0	0	0
0	0	0	0	0	0	0	0	0
0	0	0	0	0	0	0	0	0
0	0	0	0	0	0	0	0	0
0	0	0	0	0	0	0	0	0
0	0	0	0	0	0	0	0	0
0	0	0	0	0	0	0	0	0
0	0	0	0	0	0	0	0	0

1	0	0	0	0	0	0	0	0
0	0	0	0	0	0	0	0	0
1	0	0	0	0	0	0	0	0
0	0	0	0	0	0	0	0	0
1	0	0	0	0	0	0	0	0
0	0	0	0	0	0	0	0	0
0	0	0	0	0	0	0	0	0
0	0	0	0	0	0	0	0	0
0	0	0	0	0	0	0	0	0
0	0	0	0	0	0	0	0	0
0	0	0	1	0	0	0	0	0
0	0	0	0	0	0	0	0	0
0	0	0	0	0	0	0	1	0
0	0	0	0	0	0	0	0	0
0	0	0	0	0	0	0	0	0
0	0	0	0	0	0	0	0	0
0	0	0	0	0	0	0	0	0
1	0	0	0	0	0	0	1	0
0	0	0	0	0	0	1	0	0
0	0	0	0	0	0	0	0	0
0	0	0	0	0	0	0	0	0
0	0	0	0	0	0	0	1	0
0	0	0	0	0	0	0	0	0
0	0	0	0	0	0	0	0	0
0	0	0	0	0	0	0	0	0
0	0	0	0	0	0	0	0	0
1	0	0	0	0	0	0	0	0
0	0	1	0	1	0	0	0	0
0	0	0	0	0	0	0	0	0
0	0	0	0	0	0	0	0	0
0	0	0	0	0	0	0	1	0
0	0	0	0	0	0	0	0	0
1	0	0	0	0	0	0	0	0
1	0	0	0	0	0	0	0	0
0	0	0	0	0	0	0	0	0
0	0	0	0	0	0	0	0	0
0	0	0	0	0	0	0	0	0
0	0	0	0	1	0	0	0	0
0	0	0	0	0	0	0	0	0
0	0	0	0	0	0	0	0	0
0	0	0	0	0	0	0	0	0
0	0	0	0	0	0	0	0	0
0	0	0	0	0	0	0	0	0
0	0	0	0	0	0	0	0	0
0	1	0	0	0	0	0	0	0
0	0	0	0	0	0	0	0	0
0	0	0	0	0	0	0	1	0
1	0	0	0	0	0	0	0	0
0	0	0	0	0	0	0	0	0
0	0	0	0	0	0	0	0	0
1	0	0	0	0	0	0	0	0
0	0	0	0	0	0	0	0	0
1	0	0	0	0	0	0	0	0
0	0	0	0	0	0	0	0	0
1	0	0	0	0	0	0	0	0
0	0	0	0	0	0	0	0	0

0	0	0	0	0	0	0	0	0
0	0	0	0	0	0	0	0	0
0	0	0	0	0	0	0	0	0
0	0	1	0	0	0	0	1	0
0	0	0	0	0	0	0	0	0
0	0	0	0	0	0	0	0	0
0	0	0	0	0	0	0	0	0
1	0	0	0	0	0	0	0	0
1	0	0	0	0	0	0	0	0
0	0	0	0	0	0	0	0	0
0	0	0	0	0	0	0	0	0
0	0	1	0	0	0	0	0	0
0	0	0	0	0	0	0	0	0
1	0	0	0	0	0	0	0	0
0	0	0	0	0	0	0	0	0
0	0	0	0	0	0	0	0	0
0	0	0	0	0	0	0	0	0
0	0	0	0	0	0	0	0	0
0	0	0	0	0	0	0	0	0
0	0	0	0	0	0	0	1	0
0	0	0	0	0	0	0	0	0
0	0	0	0	0	0	0	0	0
1	0	0	0	0	0	0	0	0
0	0	0	0	0	0	1	0	0
0	0	0	0	0	0	0	0	0
0	0	0	0	0	0	0	0	0
0	0	0	0	0	0	0	0	0
0	0	0	0	0	0	0	0	0
0	0	0	0	0	0	0	0	0
0	0	0	0	0	0	0	0	0
0	0	0	0	0	0	0	1	0
0	0	0	0	0	0	0	0	0
0	0	0	0	0	0	0	1	0
0	0	0	0	0	0	0	0	0
0	0	0	0	0	0	0	0	0
0	0	0	0	0	0	0	0	0
0	0	0	0	0	0	0	0	0
0	0	0	0	0	0	0	0	0
0	0	0	0	0	0	0	0	0
0	0	0	0	0	0	0	0	0
0	0	0	0	0	0	0	1	0
0	0	0	0	0	0	0	0	0
0	0	0	0	0	0	0	0	0
0	1	0	0	0	0	0	0	0
1	0	0	0	0	0	0	0	0
0	0	0	0	0	0	0	0	0
1	0	0	0	0	0	0	0	0
1	1	0	0	0	0	0	1	0
0	0	0	0	0	0	0	0	0
1	0	0	0	0	0	0	0	0
1	0	0	0	0	0	0	1	0
0	0	1	0	0	0	0	0	0
0	0	0	0	0	0	0	0	0
1	1	0	0	0	0	0	0	0
0	0	0	0	0	0	0	0	0
0	0	0	0	0	0	0	0	0

0	0	0	0	0	0	0	0	0
0	0	0	0	0	0	0	0	0
0	1	0	0	1	0	0	0	0
1	0	0	0	0	0	0	1	0
0	0	0	0	0	0	0	0	0
0	0	0	0	0	0	0	0	0
0	0	0	0	0	0	0	0	0
0	0	0	0	0	0	1	1	0
0	0	0	0	0	0	0	0	0
0	0	0	0	0	0	0	0	0
0	0	0	0	0	0	0	0	0
0	0	0	0	0	0	0	0	0
1	0	0	0	0	0	0	0	0
0	0	1	0	0	0	0	0	0
1	0	0	0	0	0	0	1	0
1	0	0	0	0	0	0	0	0
0	1	0	0	0	0	0	0	0
0	0	0	0	0	0	0	0	0
0	0	0	0	0	0	0	0	0
0	0	0	0	0	0	0	0	0
0	0	0	0	0	0	0	0	0
0	0	0	0	0	0	0	0	0
0	0	0	0	0	0	0	0	0
0	0	0	0	0	0	0	0	0
0	0	0	0	0	0	0	0	0
0	0	0	0	0	0	0	0	0
1	0	0	0	0	0	0	0	0
0	0	0	0	0	0	0	0	0
0	0	0	0	0	0	0	0	0
1	1	0	0	0	0	0	1	0
0	0	0	0	0	0	0	0	0
0	0	0	0	0	0	0	0	0
1	0	0	0	0	0	0	0	0
0	0	0	0	0	0	0	0	0
0	0	0	0	0	0	0	0	0
0	0	0	0	0	0	0	0	0
1	0	0	0	0	0	0	0	0
0	0	0	0	0	0	0	1	0
0	0	0	0	0	0	0	0	0
0	0	0	0	0	0	0	0	0
0	0	0	0	0	0	0	0	0
1	0	0	0	0	0	0	0	0
0	0	0	0	0	0	0	0	0
0	0	0	0	0	0	0	0	0
0	0	0	0	0	0	0	0	0
0	1	1	0	0	0	0	1	0
0	0	0	0	0	0	0	0	0
0	0	0	0	0	0	0	0	0
0	0	0	0	0	0	0	0	0
1	0	0	0	0	0	0	0	0
0	0	1	0	0	0	0	0	0
1	0	0	0	0	0	0	0	0
0	0	0	0	0	0	0	1	0
0	0	0	0	0	0	0	0	0
0	0	0	0	0	0	0	0	0
0	0	0	0	0	0	0	0	0

0	1	0	0	0	0	0	0	0
0	0	0	0	0	0	0	0	0
0	0	0	0	0	0	0	0	0
0	0	0	0	0	0	0	0	0
0	0	0	0	0	0	0	0	0
0	0	0	0	0	0	0	0	0
0	0	0	0	0	0	0	0	0
0	0	0	0	0	0	0	0	0
0	1	0	0	0	0	0	0	0
0	0	0	0	0	0	0	0	0
0	0	0	0	0	0	0	1	0
0	0	0	0	0	0	0	0	0
1	0	0	0	0	0	0	0	0
0	0	0	0	0	0	0	1	0
1	0	0	0	1	0	0	0	0
0	0	0	0	0	0	0	1	0
0	0	0	0	0	0	0	0	0
0	0	0	0	0	0	0	0	0
1	0	0	0	0	0	0	0	0
0	0	0	0	0	0	0	0	0
0	0	0	0	0	0	0	0	0
0	0	0	0	0	0	0	0	0
0	0	0	0	0	0	0	1	0
0	1	0	0	0	0	1	0	0
0	1	0	0	0	0	1	0	0
0	0	0	0	0	0	1	0	0
0	0	0	0	0	0	0	0	0
0	0	0	0	0	0	1	0	1
0	0	0	0	0	0	0	0	0
0	0	0	0	0	0	0	0	0
1	0	0	0	0	0	0	0	0
0	0	0	0	0	0	0	0	0
0	0	0	0	0	0	0	0	0
0	0	0	0	0	0	0	0	0
0	0	0	0	0	0	0	0	1
0	0	0	0	0	0	0	0	0
0	0	0	0	1	0	0	0	0
0	0	0	0	0	0	0	0	0
0	0	0	0	0	0	0	0	0
0	0	0	0	0	0	0	1	0
0	0	0	0	0	0	0	0	0
0	0	0	0	0	0	0	0	1
0	0	0	0	0	0	0	0	0
1	0	0	0	0	0	0	0	0
1	0	0	0	0	0	0	0	1
0	0	0	0	0	0	0	0	0
0	0	0	0	0	0	0	1	0
0	0	0	0	1	0	0	0	0
0	0	0	0	0	0	0	0	0
0	0	0	0	0	0	0	0	0
0	0	0	0	0	0	0	1	0
0	0	0	0	0	0	0	0	0
0	0	0	0	0	0	1	0	0
0	0	0	0	1	0	0	0	0
0	1	0	0	0	0	0	0	0
0	0	0	0	0	0	0	1	0

0	0	0	0	0	0	0	0	0
0	0	0	0	0	0	0	0	0
0	0	0	0	0	0	0	0	0
0	0	0	0	1	0	0	0	0
0	0	0	0	0	0	0	0	0
0	0	0	0	0	0	0	0	0
0	0	0	0	0	0	0	0	0
0	0	0	0	1	0	0	0	0
1	0	0	0	0	0	0	0	0
0	0	0	0	0	0	0	0	0
0	0	0	0	0	0	0	0	0
0	0	0	0	0	0	0	0	0
0	0	0	0	0	0	0	0	0
0	0	0	0	1	0	0	0	0
0	0	0	0	1	0	0	0	0
0	0	0	0	0	0	0	0	0
0	0	0	0	0	0	0	0	0
0	0	0	0	0	0	0	0	0
0	0	0	0	0	0	0	1	0
0	0	0	0	1	0	0	0	0
0	0	0	0	0	0	0	0	0
0	0	0	0	0	0	0	0	0
0	0	0	0	0	0	0	0	0
0	0	0	0	0	0	0	0	0
0	0	0	0	0	0	0	1	0
0	0	0	0	0	0	0	0	0
0	0	0	0	0	0	0	0	0
1	0	0	0	1	0	0	0	0
0	0	0	0	0	0	0	0	0
0	0	0	0	0	0	0	1	0
1	0	0	0	1	0	0	0	0
0	0	0	0	0	0	0	0	0
0	0	0	0	0	0	0	0	0
0	0	0	0	0	0	0	0	0
0	0	0	0	0	0	0	0	0
0	0	0	0	0	0	0	1	0
0	0	0	0	0	0	0	1	0
0	0	0	0	0	0	0	0	0
0	0	0	0	0	0	0	0	0
0	0	0	0	0	0	0	0	0
0	0	0	0	0	0	0	0	1
0	0	0	0	0	0	0	0	0
0	0	0	0	1	0	0	0	0
0	0	0	0	0	0	0	1	0
0	0	0	0	0	0	0	0	0
0	0	0	0	0	0	0	1	0
0	0	0	0	0	0	0	0	0
0	0	0	0	0	0	0	0	0
0	0	0	0	0	0	0	0	0
0	0	0	0	1	0	0	0	0
0	0	0	0	0	0	0	0	0
0	0	0	0	0	0	0	0	0
1	0	0	0	0	0	0	0	0
0	0	0	0	0	0	1	0	0
0	0	0	0	0	0	0	0	0
0	0	0	0	0	0	0	0	0

0	0	0	0	0	0	0	0	0
0	0	0	0	0	0	0	0	0
0	0	0	0	0	0	0	0	0
0	0	0	0	0	0	1	0	0
0	0	0	0	0	0	0	0	0
0	0	0	0	0	0	0	0	0
0	0	1	0	0	0	0	0	0
0	0	0	0	0	0	0	0	0
0	0	0	0	0	0	0	0	1
0	0	0	0	0	0	0	0	0
0	0	0	0	0	0	0	0	0
0	0	0	0	0	0	0	0	0
0	0	0	0	0	0	0	0	0
0	0	0	0	0	0	0	0	0
0	0	0	0	0	0	0	0	0
0	0	0	0	0	0	0	0	0
0	0	0	0	0	0	0	0	0
0	0	0	0	0	0	0	0	0
1	0	0	0	1	0	0	0	0
0	0	0	0	0	0	0	0	0
0	0	0	0	0	0	0	0	0
1	0	0	0	0	0	0	1	0
0	0	0	0	0	0	0	0	0
0	0	0	0	0	0	0	0	0
1	0	0	0	0	0	0	0	0
0	0	0	0	0	0	0	0	0
0	0	0	0	0	0	0	0	0
0	1	0	0	0	0	1	0	1
0	0	0	0	0	0	1	0	0
0	0	0	0	0	0	0	0	0
0	0	1	0	0	0	0	1	0
0	0	0	0	0	0	0	0	0
0	0	0	0	0	0	0	0	0
0	0	0	0	0	0	0	0	0
0	1	0	0	0	0	0	0	0
1	0	0	0	1	0	0	0	0
0	0	0	0	0	0	0	0	0
0	0	0	0	0	0	0	0	1
0	0	0	0	0	0	0	0	0
0	0	0	0	0	0	0	0	0
0	0	0	0	0	0	0	0	0
0	0	0	0	0	0	0	0	0
0	0	0	0	0	0	0	0	0
0	1	0	0	0	0	0	0	0
0	0	0	0	0	0	0	0	0
0	0	0	0	0	0	0	0	0
1	0	0	0	0	0	0	0	0
0	0	0	0	0	0	0	0	0
0	0	0	0	0	0	0	0	0
0	0	0	0	0	0	0	0	0
0	0	0	0	0	0	0	1	0
0	0	0	0	0	0	0	0	0
1	1	0	0	0	0	0	0	0
0	0	0	0	0	0	0	0	0
0	0	0	0	0	0	0	0	0
0	0	1	0	0	0	0	0	0

0	0	0	0	0	0	0	0	0
0	0	0	0	0	0	0	0	0
1	0	0	0	0	0	0	1	0
0	0	1	0	0	0	0	0	0
1	1	0	0	0	0	0	0	0
0	0	0	0	0	0	0	0	0
0	0	0	0	0	0	0	0	0
0	0	0	0	0	0	0	0	0
0	1	0	0	0	0	0	0	0
1	0	0	0	0	0	0	0	0
1	0	0	0	0	0	0	1	0
0	0	0	0	0	0	1	0	0
1	0	1	0	0	0	0	0	0
0	0	0	0	0	0	0	0	0
0	0	0	0	0	0	0	0	0
0	0	0	0	0	0	0	0	0
0	0	0	0	0	0	0	0	0
0	0	0	0	0	0	0	0	0
0	0	0	0	0	0	0	0	0
0	0	0	0	0	0	0	1	0
0	0	0	0	0	0	0	0	0
0	0	0	0	0	0	0	1	0
0	0	0	0	0	0	0	0	0
0	0	0	0	0	0	0	0	0
0	0	0	0	0	0	0	0	0
0	0	0	0	0	0	0	0	0
0	1	0	0	0	0	0	0	0
0	0	0	0	0	0	0	0	1
0	0	0	0	0	0	0	0	0
0	0	0	0	0	0	0	0	0
0	0	0	0	0	0	0	0	0
0	0	0	0	0	0	0	0	0
0	0	0	0	1	0	0	0	0
0	0	0	0	0	0	0	0	0
0	0	0	0	0	0	0	0	0
0	0	0	0	0	0	0	0	0
1	0	0	0	0	0	0	0	0
1	1	0	0	1	0	0	0	0
0	0	0	0	0	0	0	0	0
0	0	0	0	0	0	0	0	0
0	0	0	0	0	0	0	0	0
0	0	0	0	0	0	0	0	0
0	0	0	0	0	0	0	0	0
1	0	0	0	0	0	0	0	0
0	0	0	0	0	0	0	0	0
0	0	0	0	0	0	0	0	0
0	0	0	0	0	0	0	1	0
0	0	0	0	0	0	0	0	0
0	0	0	0	0	0	0	0	0
0	0	0	0	0	0	0	0	0
0	0	0	0	0	0	0	1	0
0	0	0	0	0	0	0	0	0
0	0	0	0	0	0	1	0	0
1	0	0	0	0	0	0	0	0
1	0	0	0	0	0	0	1	0
1	0	0	0	0	0	0	0	0

0	0	0	0	0	0	0	1	0
1	0	0	0	0	0	0	0	0
0	0	0	0	0	0	0	0	0
0	0	0	0	0	0	0	0	0
0	0	0	0	0	0	0	0	0
0	0	0	0	0	0	0	0	0
1	0	0	0	0	0	0	0	0
0	0	0	0	0	0	0	0	0
0	0	0	0	0	0	0	0	0
0	0	0	0	0	0	0	0	0
0	0	0	0	0	0	0	0	0
0	0	0	0	0	0	0	0	0
0	0	0	0	0	0	0	0	0
1	0	0	0	0	0	0	0	0
0	0	0	0	0	0	0	0	0
0	0	0	0	0	0	0	0	0
0	0	0	0	0	0	0	0	0
0	0	0	0	0	0	0	0	0
0	0	0	0	0	0	0	0	0
0	0	0	0	0	0	0	0	0
1	0	0	0	0	0	0	0	0
0	0	0	0	0	0	0	0	0
1	0	0	0	0	0	0	0	0
0	0	0	1	0	0	1	0	0
0	0	0	0	0	0	0	0	0
0	0	0	0	0	0	0	0	0
0	0	0	0	0	0	0	1	0
0	0	0	0	0	0	0	0	0
0	1	0	0	0	0	0	0	0
0	0	0	0	0	0	0	0	0
0	0	0	0	0	0	0	0	0
0	0	0	0	0	0	0	0	1
0	0	0	0	0	0	0	1	0
0	0	0	0	1	0	1	0	0
0	0	0	0	0	0	0	0	0
0	0	0	0	0	0	0	1	0
0	0	0	0	0	0	0	0	0
0	0	0	0	0	0	0	0	0
0	0	0	0	0	0	0	0	0
0	0	0	0	0	0	0	0	0
0	0	0	0	0	0	0	1	0
0	0	0	0	0	0	0	0	0
0	0	0	0	0	0	0	0	0
0	0	0	0	0	0	0	0	0
0	0	0	0	0	0	0	0	0
0	0	0	0	0	0	0	0	0
0	0	0	0	0	0	0	0	0
0	0	0	0	0	0	0	0	0
0	0	0	0	0	0	0	0	0
0	0	0	0	0	0	0	0	0
1	0	0	0	0	0	0	0	0
0	0	0	0	0	0	0	0	0
0	0	0	0	0	0	0	0	0
1	0	0	0	0	0	0	0	0
0	0	0	0	0	1	0	1	0
0	0	0	0	0	0	0	0	0

0	0	0	0	0	0	0	1	0
0	0	0	0	0	0	0	0	0
0	0	0	0	0	0	0	0	0
0	0	0	0	0	0	0	0	0
0	0	0	0	0	0	0	0	0
1	0	0	0	0	0	0	0	0
0	0	0	0	0	0	0	0	0
1	0	0	0	0	0	0	0	0
0	0	0	0	0	0	0	0	0
0	0	0	0	0	0	0	0	0
0	0	0	0	0	0	0	0	0
0	0	0	0	0	0	0	0	0
0	0	0	0	0	0	0	0	0
0	0	0	0	0	0	0	0	0
0	0	0	0	0	1	0	0	0
0	1	0	0	0	0	1	0	0
0	0	0	0	0	0	0	1	0
0	0	0	0	0	0	0	0	0
0	0	0	0	0	0	0	0	0
0	0	0	0	0	0	0	0	0
0	0	0	0	0	0	0	0	0
0	0	0	0	0	0	0	0	0
1	0	0	0	0	0	0	0	0
0	0	1	0	0	0	0	0	0
0	0	0	0	0	0	0	0	0
1	0	0	0	0	0	0	1	0
0	0	0	0	0	0	0	0	0
0	0	0	0	0	0	0	0	0
0	0	0	0	0	0	0	0	0
0	0	0	0	0	0	0	0	0
0	0	0	0	0	0	0	0	0
0	0	0	0	0	0	0	0	0
0	0	0	0	0	0	0	0	0
0	0	0	0	0	0	0	0	0
0	0	0	0	0	0	1	0	0
1	0	0	0	0	0	0	0	0
0	0	0	0	0	0	0	0	0
0	0	0	0	0	0	0	0	0
0	0	0	0	0	0	0	0	0
0	0	0	0	0	0	0	1	0
0	0	0	0	0	0	0	0	0
0	0	0	0	0	0	0	0	0
0	0	0	0	0	0	0	0	0
0	0	0	0	0	0	0	0	0
1	0	0	0	0	0	0	0	0
1	0	0	0	0	0	0	0	0
1	0	0	0	0	0	0	0	0
1	0	0	0	0	0	0	0	0
0	0	0	0	0	0	0	1	0
1	0	0	0	0	0	0	0	0
0	0	0	0	0	0	0	0	0
0	0	0	0	0	0	0	0	0
0	0	0	0	0	0	0	0	0
0	0	0	0	0	0	0	0	0
0	0	0	0	0	0	0	0	0
0	0	0	0	0	0	0	0	0

0	0	0	0	0	0	0	0	0
0	0	0	0	0	0	0	0	0
0	0	0	0	0	0	0	0	0
0	0	0	0	0	0	0	0	0
0	0	0	0	0	0	0	0	0
1	0	0	0	0	0	0	0	0
0	0	0	0	0	0	0	0	0
0	0	0	0	0	0	0	0	0
0	0	0	0	0	0	0	0	0
0	1	0	0	0	0	0	0	0
1	0	0	0	0	0	0	0	0
0	0	0	0	0	0	0	0	0
0	0	0	0	0	0	0	1	0
0	0	0	0	0	0	0	0	0
0	0	0	0	0	0	0	0	0
0	0	0	0	0	0	0	0	0
0	0	0	0	0	0	0	0	0
0	0	0	0	0	0	0	0	0
0	0	0	0	0	0	0	0	0
0	0	0	0	0	0	0	0	0
0	0	0	0	0	0	0	0	0
0	0	0	0	0	0	0	0	0
0	0	0	0	0	0	0	0	0
0	0	0	0	1	0	0	0	0
1	0	0	0	0	0	0	0	0
1	0	0	0	0	0	0	0	0
1	0	0	0	0	0	0	0	0
0	0	0	0	0	0	0	0	0
0	0	0	0	0	0	0	0	0
0	1	0	0	0	0	0	0	0
0	0	0	0	0	0	0	0	0
0	0	0	0	0	0	0	0	0
0	0	0	0	0	0	0	0	0
0	0	0	0	0	0	0	0	0
1	0	0	0	0	0	0	0	0
0	0	0	0	0	0	0	0	0
1	0	0	0	0	0	0	0	0
1	0	0	0	0	0	0	0	0
0	0	0	0	0	0	0	0	0
0	0	0	0	0	0	0	0	0
0	0	0	0	0	0	0	0	0
0	0	0	0	0	0	0	0	0
0	0	0	0	0	0	0	0	0
1	0	0	0	0	0	0	0	0
0	0	0	0	0	0	0	1	0
1	0	0	0	0	0	0	0	0
0	0	0	0	0	0	0	0	0
0	0	0	0	1	0	0	0	0
0	0	0	0	0	0	0	0	0
0	0	0	0	0	0	0	0	0
0	0	0	0	0	0	0	1	0
1	0	0	0	0	0	0	0	0
0	0	0	0	1	0	0	0	0
0	0	0	0	0	0	0	0	0
1	0	0	0	0	0	0	0	0
0	0	0	0	0	0	0	0	0

0	0	0	0	0	0	0	1	0
0	0	0	0	0	0	1	0	0
0	0	1	0	1	0	0	0	0
0	0	0	0	0	0	0	0	0
1	0	0	0	1	0	0	0	0
1	0	0	0	0	0	0	0	0
0	0	0	0	0	0	0	0	1
1	0	0	0	0	0	0	0	0
0	0	0	0	0	0	0	0	0
0	0	1	0	0	0	0	1	0
0	0	0	0	0	0	0	1	0
0	0	0	0	0	0	0	0	0
0	0	0	0	0	0	0	0	0
0	0	0	0	1	0	0	0	0
1	0	0	0	0	0	0	0	0
0	0	0	0	0	0	0	0	0
0	0	0	0	0	0	0	1	0
1	1	0	0	0	0	0	1	0
1	0	0	0	1	0	0	0	0
0	0	0	0	0	0	0	0	0
0	0	0	0	0	0	0	0	1
0	0	0	0	0	0	0	0	0
1	0	0	0	0	0	0	0	0
0	0	0	0	0	0	0	0	0
0	0	0	0	1	0	0	0	0
0	0	0	0	0	0	0	0	0
0	0	0	0	0	0	0	0	0
0	0	0	0	0	0	0	0	0
0	0	0	0	0	0	0	0	0
1	0	0	0	0	0	0	0	0
0	0	0	0	0	0	0	0	0
1	0	0	0	0	0	0	0	0
0	0	0	0	0	0	0	0	0
0	0	0	0	0	0	0	0	0
1	0	0	0	0	0	0	0	0
0	0	0	0	0	0	0	1	0
0	1	0	0	0	0	0	0	0
0	0	0	0	0	0	0	0	0
0	0	0	0	0	0	0	0	0
0	0	0	0	1	0	0	0	0
0	1	0	0	0	0	0	0	0
0	0	0	0	1	0	0	0	0
0	0	0	0	0	0	0	0	0
0	0	0	0	0	0	1	0	0
1	0	0	0	0	0	0	0	0
0	0	0	1	1	1	0	1	0
1	0	0	0	0	0	1	0	0
1	0	0	0	0	0	0	0	0
0	0	0	0	0	0	0	0	0
0	0	0	0	0	0	0	0	0
0	0	0	0	0	0	0	0	0
1	0	0	0	0	0	0	0	0
0	1	1	0	0	0	0	0	0
0	0	0	0	0	0	0	1	0
0	0	0	0	0	0	0	0	0
1	0	0	0	1	0	0	0	0

0	1	0	0	0	0	0	0	0
0	0	0	0	0	0	0	0	0
0	0	0	0	0	0	0	0	0
0	0	0	0	0	0	0	0	0
1	0	0	0	0	0	0	0	0
0	0	0	0	0	0	0	0	0
1	0	0	0	0	0	0	0	0
0	0	0	0	0	0	0	0	0
0	0	0	0	0	0	0	0	0
0	0	0	0	0	0	0	0	0
0	0	0	0	0	0	0	0	1
1	0	0	0	0	0	0	0	0
0	0	0	0	0	0	1	0	0
0	0	0	0	0	0	0	0	0
0	0	0	0	0	0	0	0	0
0	0	0	0	0	0	0	0	0
0	0	0	0	0	0	0	0	0
0	0	0	0	0	0	0	0	0
0	0	0	0	0	0	0	0	0
0	1	0	0	0	0	0	0	0
1	0	0	0	0	0	0	0	0
0	0	0	0	0	0	1	0	0
1	0	0	0	0	0	0	0	0
0	0	0	0	0	0	0	0	0
1	0	0	0	0	0	0	0	0
1	0	0	0	0	0	0	0	0
1	0	0	0	0	0	0	0	1
0	0	0	0	0	0	0	0	0
1	0	0	0	0	0	0	0	0
1	0	0	0	0	0	0	1	1
0	0	0	0	0	0	0	0	0
0	0	0	0	0	0	0	0	0
1	1	0	0	0	0	1	0	1
0	0	0	0	1	0	0	0	0
1	0	0	0	0	1	0	1	0
0	0	0	0	0	0	1	0	0
0	0	0	0	0	0	0	0	0
0	1	0	0	0	0	0	0	1
1	0	0	0	0	0	0	1	0
1	1	0	0	0	0	1	0	0
0	0	0	0	0	0	0	0	0
1	0	0	0	0	0	0	0	0
0	1	0	0	0	0	0	1	1
0	0	0	0	0	0	0	0	0
0	0	0	0	0	0	0	1	1
1	0	0	0	0	0	0	0	0
0	0	0	0	0	0	0	0	0
0	0	0	0	0	0	0	0	0
0	0	0	0	0	0	0	0	0
1	0	0	0	0	0	0	0	0
0	0	0	0	0	0	1	1	0
0	0	0	0	0	0	0	1	0
1	0	0	0	0	0	0	0	0
0	0	0	0	0	0	0	0	0
0	0	0	0	0	0	0	1	0
0	0	0	0	0	0	0	0	0

1	1	0	0	1	0	0	0	1
1	0	0	0	0	0	0	0	0
1	1	0	0	0	0	0	1	0
0	0	0	0	0	0	0	0	0
0	0	0	0	0	0	1	1	0
0	0	0	0	1	0	0	0	0
0	0	0	0	0	0	0	1	0
0	0	0	0	0	0	0	0	0
0	0	0	0	0	0	0	0	0
1	0	0	0	0	0	0	0	0
0	1	0	0	0	0	0	0	0
0	0	0	1	0	0	0	0	0
0	0	0	0	0	0	0	0	0
1	0	0	0	0	0	0	1	0
0	0	0	0	0	0	0	0	0
0	0	0	0	0	0	0	0	0
0	0	0	0	0	1	0	0	1
1	1	0	0	0	0	0	0	0
0	0	0	0	0	0	0	0	0
1	1	0	0	0	0	0	0	0
0	0	0	0	0	0	0	0	0
1	0	0	0	0	0	0	0	0
0	0	0	0	0	0	1	1	0
0	0	0	0	0	0	0	0	0
0	0	0	1	0	0	0	0	0
0	0	0	0	0	0	0	0	0
0	0	0	0	0	0	0	0	0
0	0	0	0	0	0	0	0	0
1	0	0	0	1	0	0	1	0
0	0	0	0	0	0	0	0	0
0	0	0	0	0	0	0	1	0
0	0	0	0	0	0	0	0	0
0	0	0	0	0	0	0	0	0
0	0	0	0	0	0	0	0	0
0	0	0	0	1	0	0	0	0
0	0	0	0	0	0	0	0	0
0	0	0	0	0	0	0	0	0
0	0	0	0	0	0	0	0	0
0	0	0	0	0	0	0	1	0
1	0	0	0	0	0	0	0	0
0	0	0	0	0	0	0	0	0
0	0	0	0	0	0	0	0	0
1	0	0	0	0	0	0	1	0
1	0	0	0	0	0	0	0	0
0	0	0	0	0	0	0	0	0
1	0	0	0	0	0	0	0	0
0	0	0	0	0	0	0	0	0
0	0	0	0	0	0	0	1	0
0	0	0	0	1	0	0	0	0
1	1	0	0	0	0	0	0	0
0	0	0	0	0	0	0	0	0
0	0	0	0	0	0	0	0	0
0	0	0	0	0	0	0	0	0
1	0	0	0	1	0	0	0	0
0	0	0	0	0	0	0	0	0
1	0	0	0	0	0	0	0	0

0	0	1	0	1	0	0	0	0
0	0	0	0	0	0	0	0	0
1	0	0	0	0	0	1	0	0
0	0	0	0	0	0	0	1	0
1	0	0	0	0	0	0	0	0
1	1	0	0	1	0	0	0	0
0	0	0	0	0	0	0	0	0
0	0	0	0	0	0	0	0	0
0	1	0	0	0	0	0	1	0
0	0	0	0	0	0	0	0	0
0	0	0	0	0	0	0	0	0
0	0	0	0	0	0	0	0	0
0	0	0	0	0	0	0	1	0
0	0	0	0	0	0	0	0	0
1	0	0	0	0	0	0	0	0
1	0	0	0	0	0	0	0	0
1	1	0	0	0	0	0	0	0
0	0	0	0	0	0	0	0	0
1	0	0	0	0	0	0	0	0
0	0	0	0	0	0	0	0	0
0	0	0	0	0	0	0	0	0
0	0	0	0	0	0	1	0	0
0	0	0	0	0	0	0	0	0
0	0	0	0	0	0	0	1	0
0	0	0	0	0	0	0	0	0
0	0	0	0	0	0	0	0	0
0	0	0	0	1	0	0	0	0
0	0	0	0	0	1	0	1	0
1	0	0	0	0	0	0	0	0
0	0	0	0	0	0	0	0	0
0	0	0	0	0	0	0	0	0
0	0	0	0	0	0	0	0	0
1	0	0	0	0	0	0	0	0
0	0	0	0	0	0	0	0	0
1	0	0	0	0	0	0	0	0
0	1	0	0	0	0	0	0	0
1	0	0	0	0	0	0	0	0
0	1	0	0	0	0	0	1	1
0	1	0	0	0	0	1	0	0
0	0	0	0	0	0	0	0	0
0	0	0	0	0	0	0	1	0
0	0	0	0	0	0	0	0	0
0	0	0	0	0	0	0	0	0
0	0	0	0	0	0	0	0	0
1	0	0	0	0	0	0	1	0
0	1	0	0	0	0	0	0	0
0	0	0	0	0	0	0	1	0
0	0	0	0	0	0	0	0	0
0	0	0	0	0	0	0	0	0
0	0	0	0	0	0	0	0	0
0	0	0	0	0	0	0	0	0
0	0	0	0	1	0	0	0	0
0	0	0	0	0	0	0	0	0
1	0	0	0	0	0	0	0	0
0	0	0	0	0	0	0	0	0
0	0	0	0	1	0	0	0	0

0	0	0	0	1	0	0	0	0
1	0	0	0	0	0	0	0	0
0	1	0	0	0	0	1	0	0
1	0	0	0	0	0	1	0	0
0	0	0	0	0	0	0	0	0
1	0	0	0	0	0	0	0	0
0	0	0	0	0	0	0	0	0
0	0	0	0	0	0	0	0	0
0	0	0	0	0	0	0	0	0
0	0	0	0	0	0	0	0	0
0	0	0	0	0	0	0	0	0
0	0	0	0	0	0	0	0	0
1	0	0	0	0	0	0	0	0
0	1	0	0	0	0	0	1	0
0	0	0	0	0	0	0	0	0
0	0	0	0	0	0	1	0	0
0	0	0	0	0	0	0	0	0
0	0	0	0	0	0	0	0	0
0	0	0	0	0	0	0	0	0
0	0	0	0	0	0	0	0	0
0	0	0	0	0	0	0	0	0
0	0	0	0	0	0	0	0	0
1	1	0	0	0	0	1	0	0
0	1	0	0	0	0	0	0	0
0	0	0	0	0	0	0	0	0
0	0	0	0	0	0	0	0	0
0	0	0	0	0	0	0	0	0
1	0	0	0	0	0	0	1	0
0	0	0	0	0	0	0	0	0
0	0	0	0	0	0	0	0	0
0	0	0	0	0	0	0	0	0
0	0	0	0	0	0	0	1	0
0	0	0	0	0	0	0	0	0
0	0	0	0	0	0	0	0	0
1	0	0	0	0	0	0	0	0
0	0	0	0	0	0	0	1	0
0	0	0	0	0	0	0	0	0
0	0	0	0	0	0	1	0	0
0	0	0	0	0	0	0	1	0
1	0	0	0	0	0	0	0	0
1	0	0	0	0	0	0	0	0
1	0	0	0	0	0	0	0	0
0	0	0	0	0	0	0	0	0
0	0	0	0	0	0	0	0	0
0	0	0	0	0	0	0	0	0
0	0	0	0	0	0	0	0	0
1	0	0	0	0	0	0	0	0
0	0	0	0	0	0	0	0	0
0	0	0	0	0	0	0	0	0
1	0	0	0	0	0	0	0	0
1	0	0	0	1	0	0	0	0
0	0	0	0	1	0	0	0	0
0	0	0	0	0	0	0	1	0
1	0	0	0	0	0	0	0	0
0	0	0	0	0	0	0	1	0
0	0	0	0	1	0	0	0	0

0	0	0	0	0	0	0	0	0
0	0	0	0	0	0	0	0	0
0	0	0	0	0	0	0	0	0
0	0	0	0	0	0	0	0	0
0	0	0	0	0	0	0	0	0
0	0	0	0	0	0	0	0	0
0	0	0	0	0	0	0	0	0
1	0	0	0	0	0	0	0	0
0	0	0	0	0	0	0	0	0
0	0	1	0	1	0	0	1	0
0	1	0	0	0	0	0	0	0
0	0	0	0	0	0	0	0	0
0	0	0	0	0	0	0	1	0
0	0	0	0	0	0	0	0	0
0	0	0	0	0	0	0	0	0
1	0	0	0	0	1	0	0	0
1	0	0	0	0	0	0	0	0
0	0	0	0	1	0	0	0	0
0	0	0	0	0	0	0	0	0
0	0	0	0	0	0	0	0	0
0	0	0	0	0	0	0	1	0
0	1	0	0	0	0	0	0	0
0	0	0	0	0	0	0	0	0
0	0	0	0	0	0	0	0	0
0	1	0	0	0	0	0	0	0
0	0	0	0	0	0	0	0	0
0	0	0	0	0	0	0	0	0
0	0	0	0	0	0	0	0	0
1	0	0	0	0	0	0	0	0
0	0	0	0	0	0	0	0	0
0	0	0	0	0	0	0	1	0
0	0	0	0	0	0	1	0	0
1	0	0	0	0	0	0	0	0
0	1	0	0	0	0	0	0	0
0	0	0	0	0	0	0	0	0
0	0	0	0	0	0	0	0	0
0	0	0	0	0	0	0	0	0
0	1	0	0	0	0	0	0	0
0	0	0	0	0	0	0	0	0
0	0	0	0	0	0	0	0	0
0	0	0	0	0	0	0	0	0
0	0	0	0	0	0	0	1	0
1	0	0	0	0	0	0	0	0
0	0	0	0	1	0	0	0	0
0	0	0	0	0	0	0	1	0
0	0	0	0	0	0	0	0	0
0	0	0	0	0	0	0	0	0
0	0	0	0	0	0	0	0	0
0	0	0	0	0	0	0	0	0
1	0	0	0	0	0	0	0	0
0	0	0	0	1	0	0	1	0
1	0	0	0	0	0	0	1	0
0	0	0	0	0	0	0	0	0
0	1	0	0	0	0	0	0	0
0	0	0	0	0	0	0	0	0
0	0	0	0	0	0	0	0	0

1	0	0	0	1	1	0	0	0
1	0	0	0	1	0	0	0	0
1	0	0	0	0	0	0	0	0
0	1	0	0	0	0	0	0	0
1	0	0	0	0	0	0	0	0
0	0	0	0	0	0	0	0	0
1	1	0	0	0	0	0	0	0
1	0	0	0	0	0	0	0	0
1	0	0	0	0	0	0	0	0
1	0	0	0	0	0	0	0	0
0	1	0	0	0	0	1	0	0
1	0	0	0	0	0	0	0	0
0	0	0	0	0	0	1	1	0
0	0	0	0	0	0	0	0	0
0	0	0	0	0	0	1	0	0
0	0	0	0	1	0	0	0	0
0	0	0	0	0	0	0	0	0
1	0	0	0	1	0	0	0	0
0	0	1	0	0	0	0	1	0
0	0	0	0	0	0	0	1	0
0	0	0	0	0	0	0	0	0
0	0	0	0	0	0	0	0	0
0	0	0	0	0	0	0	0	0
0	0	0	0	0	0	0	1	0
0	0	0	0	0	0	0	0	0
1	0	0	0	1	0	0	1	0
1	0	0	0	1	0	0	0	0
1	0	0	0	0	0	0	0	0
1	0	0	0	0	0	0	0	0
0	0	0	0	1	0	0	0	0
1	0	0	0	0	0	0	0	0
0	1	0	0	0	0	0	0	0
0	0	0	0	0	0	0	0	0
1	0	0	0	1	0	0	0	0
0	0	0	0	0	0	0	0	0
1	0	0	0	0	0	0	0	0
0	0	0	0	0	0	0	0	0
0	0	0	0	0	0	0	0	0
0	1	0	0	0	0	0	0	0
0	0	0	0	0	0	0	0	0
0	0	0	0	0	1	1	1	0
1	0	0	0	0	0	0	0	0
0	0	0	0	0	0	0	0	0
0	0	0	0	0	0	0	0	0
0	0	0	0	0	0	0	0	0
0	0	0	0	0	0	0	0	0
0	0	0	0	0	0	0	1	0
0	0	0	0	1	0	0	0	0
1	0	0	0	0	0	1	0	0
1	1	0	0	0	0	0	0	0
0	0	0	0	0	0	0	0	0
1	0	0	0	0	0	0	0	0
1	0	0	0	0	0	0	1	0
1	1	0	0	0	0	0	0	0
0	0	0	0	0	0	0	0	0
0	1	0	0	0	0	1	1	0

0	1	0	0	0	0	0	0	0
0	0	0	0	0	0	0	0	0
0	0	0	0	0	0	0	0	0
0	0	0	0	0	0	0	0	0
0	0	0	0	0	0	0	0	0
0	0	0	0	0	0	0	0	0
0	0	0	0	0	0	0	0	0
0	0	0	0	0	0	0	0	0
0	0	0	0	0	0	0	0	0
0	0	0	0	0	0	0	0	0
0	0	0	0	1	0	0	0	0
0	0	0	0	0	0	0	0	0
0	0	0	0	0	0	0	0	1
1	0	0	0	0	0	0	0	0
0	0	0	0	1	0	0	0	0
0	1	0	0	0	0	0	0	0
0	0	0	0	0	0	0	0	0
0	0	0	0	0	0	0	0	0
0	0	0	0	0	0	0	0	0
0	0	0	0	0	0	0	0	0
0	0	0	0	0	0	0	0	0
1	0	0	0	0	0	0	0	0
0	0	0	0	0	0	0	0	0
0	0	0	0	0	0	0	1	0
0	0	0	0	0	0	0	0	0
0	0	0	0	0	0	0	1	0
0	1	0	0	0	0	0	0	0
0	0	0	0	0	0	0	0	0
0	0	0	0	0	0	0	0	0
0	0	0	0	0	0	0	0	0
0	0	0	0	0	0	0	0	0
1	0	0	0	0	0	0	0	0
0	0	0	0	0	0	0	0	0
0	0	0	0	0	0	0	0	0
0	0	0	0	0	0	0	0	0
0	0	0	0	0	0	0	0	0
0	0	0	0	0	0	0	0	0
0	0	0	0	1	0	0	0	0
0	0	0	0	0	0	0	0	0
0	0	0	0	0	0	0	0	0
0	0	0	0	0	0	0	0	0
0	0	0	0	0	0	0	0	0
0	0	0	0	0	0	0	0	0
0	0	0	0	0	0	0	0	0
0	0	0	0	0	0	1	0	0
1	0	0	0	0	0	0	0	0
0	0	0	0	0	0	0	0	0
1	0	0	0	0	0	0	0	0
0	0	0	0	0	0	0	0	1
1	0	0	0	0	0	0	0	0
0	0	0	0	0	0	0	0	0
1	0	0	0	0	0	0	0	0
0	0	0	0	0	0	0	0	0
0	0	0	0	0	0	0	0	0
0	0	0	0	0	0	0	0	0
0	0	0	0	0	0	0	0	0

0	0	0	0	0	0	0	0	0
0	0	0	0	0	0	0	0	0
0	0	0	0	0	0	0	0	0
0	0	0	0	0	0	0	0	1
1	0	0	1	0	0	0	0	0
0	0	0	1	0	0	0	0	0
0	0	0	0	0	0	0	0	0
0	0	0	0	0	0	0	0	0
0	0	0	0	0	0	0	0	0
0	0	0	0	1	0	0	0	0
0	0	0	0	0	0	0	0	0
0	0	0	0	0	0	0	0	0
0	0	0	0	0	0	0	0	0
1	0	0	0	0	0	0	0	0
0	0	0	0	0	0	0	0	0
0	0	0	0	0	0	0	0	0
0	1	0	0	0	0	0	0	0
0	0	0	0	0	0	0	0	0
0	0	0	0	0	0	0	0	0
0	0	0	0	0	0	0	0	0
0	0	0	0	0	0	0	0	0
0	0	0	0	0	0	0	0	0
1	0	0	0	0	0	0	0	0
0	0	0	0	0	0	0	0	0
0	0	0	0	0	0	0	0	0
0	0	0	0	0	0	0	0	0
0	1	0	0	0	0	0	0	0
0	0	0	0	0	0	0	1	0
0	0	0	0	0	0	0	0	0
1	0	0	0	0	0	0	0	0
0	0	0	0	0	0	0	0	0
0	0	0	0	0	0	0	0	0
0	1	0	1	0	0	0	1	0
0	0	0	0	0	0	0	0	0
0	0	0	0	0	0	0	0	0
0	0	0	0	0	0	0	0	0
1	1	0	0	0	0	0	0	1
1	0	0	0	0	0	0	1	0
1	0	0	0	0	0	0	0	0
0	0	0	0	0	0	0	1	0
0	0	0	0	0	0	0	0	0
0	0	0	0	1	1	0	0	0
0	0	0	0	0	0	0	0	0
0	0	0	0	0	0	0	0	0
0	1	0	0	0	0	0	0	0
0	0	0	0	0	0	0	0	0
0	0	0	0	0	0	0	0	0
0	0	0	0	0	0	0	0	0
0	0	0	0	0	0	0	0	1
0	0	0	0	0	0	0	0	0
0	0	0	0	0	0	0	0	0
0	0	0	0	0	0	0	0	0
0	0	0	0	0	0	0	0	0
1	0	0	0	0	0	0	0	0
0	0	0	0	0	0	0	0	0
0	0	0	0	0	0	0	0	0

0	0	0	0	0	0	0	0	0
0	0	0	0	0	0	0	0	0
0	0	0	0	0	0	0	0	0
0	0	0	0	0	0	0	0	0
0	0	0	0	0	0	0	0	1
1	0	0	0	0	0	0	0	0
0	0	0	0	0	0	0	0	0
0	0	0	0	0	0	0	0	0
0	0	0	0	0	0	0	0	0
0	0	0	0	0	0	0	0	0
0	0	0	0	0	0	0	0	0
0	0	0	0	0	0	0	0	0
0	0	0	0	0	0	0	0	1
0	0	0	0	0	0	0	0	0
0	1	0	0	0	0	0	0	0
0	0	0	0	0	0	0	0	0
1	0	0	0	0	0	0	0	0
0	0	0	0	0	0	0	0	0
0	0	0	0	0	0	0	0	0
0	0	0	0	0	0	0	0	0
0	0	0	0	0	0	0	0	1
0	0	0	0	1	1	0	0	0
1	0	0	0	0	0	1	0	0
0	0	0	0	0	0	0	0	0
0	0	0	0	0	0	0	0	0
0	1	0	0	0	0	0	0	0
0	0	0	0	0	0	0	1	0
0	0	0	0	0	0	0	0	0
0	0	0	0	0	0	0	0	0
0	0	0	0	0	0	0	0	0
0	0	0	0	0	0	0	0	0
0	0	0	0	0	0	0	0	0
1	0	0	1	0	0	0	0	0
0	0	0	0	0	0	0	0	0
0	0	0	0	0	0	0	0	0
0	0	0	0	0	0	0	0	0
0	0	0	0	0	0	0	0	0
0	0	0	0	0	0	0	0	0
1	0	0	0	0	0	0	0	0
0	0	0	0	0	0	0	0	0
1	0	0	0	0	0	0	0	0
0	0	0	0	0	0	0	0	0
0	0	0	0	0	0	0	1	0
1	0	0	0	0	0	0	0	0
0	0	0	0	0	0	0	0	0
1	0	0	0	0	0	0	0	0
0	0	0	0	0	0	0	1	0
1	0	0	0	0	0	0	0	0
1	0	0	0	0	0	0	0	0
1	0	0	0	0	0	0	0	0
0	0	0	0	0	0	0	0	0
0	0	0	0	0	0	0	0	0
0	0	0	0	0	0	0	0	0
0	0	0	0	0	0	0	0	0
0	1	0	0	0	0	0	0	0
0	0	0	0	0	0	0	0	0

0	0	0	1	0	0	0	0	0
0	0	0	0	1	0	0	0	0
0	0	0	0	0	0	0	0	0
0	0	0	0	0	0	0	0	0
1	0	0	0	0	0	0	0	0
0	1	0	0	0	0	0	0	0
0	0	0	0	1	0	0	0	0
0	0	0	0	0	0	0	1	0
0	0	0	0	0	0	0	0	0
1	0	0	0	1	0	0	0	0
0	0	0	0	1	0	0	1	0
0	0	0	0	1	0	0	1	0
0	0	0	0	1	0	0	0	0
0	0	1	1	0	0	0	0	0
0	0	0	0	0	0	0	0	0
0	0	0	0	0	0	0	0	0
0	1	0	0	0	0	0	1	0
0	0	0	0	0	0	1	1	0
0	0	0	0	0	0	0	0	0
0	0	0	0	0	0	0	0	0
0	0	1	0	0	0	0	0	0
0	1	0	0	0	0	0	0	0
0	0	0	0	0	0	0	0	0
0	0	0	0	0	0	0	0	0
0	1	0	0	0	0	0	0	0
0	1	0	0	0	0	0	1	0
0	0	0	0	0	0	0	1	0
0	0	0	0	0	0	0	0	0
0	1	0	0	0	0	0	1	0
0	0	0	0	0	0	0	0	0
0	0	0	0	0	0	0	0	0
0	0	0	0	0	0	0	0	0
0	0	0	0	0	0	0	0	0
1	0	0	0	0	0	0	0	0
0	0	0	0	0	0	0	1	0
0	1	0	0	0	0	0	1	0
0	0	0	0	0	0	0	0	0
0	1	0	0	0	0	0	0	0
0	0	0	0	0	0	0	0	0
0	0	0	0	0	0	0	0	0
0	0	0	0	0	0	0	0	0
0	0	0	0	0	0	0	0	0
0	0	0	0	0	0	0	0	0
0	0	0	1	0	0	0	1	0
0	1	0	0	1	0	0	1	0
0	0	0	0	0	0	0	0	0
0	0	0	0	0	0	0	0	0
0	0	0	0	0	0	0	0	0
0	0	0	0	1	0	0	0	0
0	0	0	0	0	0	0	0	0
0	0	0	0	0	0	0	1	0
1	0	0	0	0	0	0	0	0
0	0	0	0	0	0	0	1	0
0	0	0	0	0	0	0	0	0
0	0	0	0	0	0	0	0	0
0	0	0	0	1	0	0	0	0

0	0	0	0	0	0	0	0	0
0	0	0	0	0	0	0	0	0
0	0	0	0	1	0	0	0	0
1	0	0	0	0	0	0	0	0
0	0	0	0	0	0	0	0	0
1	0	0	0	0	0	0	0	0
0	0	0	0	0	0	0	0	0
1	0	0	0	0	0	0	0	0
0	1	0	0	0	0	0	0	0
1	0	0	0	0	0	0	0	0
0	0	0	0	0	0	0	0	0
0	0	0	0	0	0	0	0	0
0	1	0	0	0	0	0	0	0
0	0	0	0	1	0	0	0	0
0	0	0	0	1	0	0	0	0
0	0	0	0	0	0	0	0	1
0	0	0	0	0	0	0	0	0
0	0	0	0	0	0	0	0	0
0	0	0	0	0	0	0	0	0
0	0	0	0	1	0	0	0	0
0	0	0	0	0	0	0	0	0
0	0	0	0	0	0	0	0	0
0	1	0	0	0	0	0	1	0
0	0	0	0	0	0	0	0	0
0	0	0	0	1	0	0	0	0
1	0	0	0	0	0	0	1	0
0	1	0	0	0	0	0	1	0
0	0	0	0	0	0	0	0	0
0	0	0	0	0	0	0	0	0
1	0	0	0	0	0	0	0	0
0	0	0	0	0	0	0	1	0
0	0	0	0	0	0	0	0	0
0	0	0	0	1	0	0	0	0
0	0	0	0	0	0	0	0	0
0	1	1	0	0	0	1	0	0
0	0	0	0	0	0	0	0	0
0	0	0	1	0	0	0	0	0
0	1	0	0	0	0	0	0	0
0	0	0	0	0	0	0	0	0
0	1	1	0	0	0	0	0	0
1	0	0	0	0	0	0	0	0
0	0	0	0	1	0	0	0	0
1	0	0	0	0	0	1	0	0
0	0	0	0	0	0	0	1	0
1	0	0	0	1	0	0	1	0
0	0	0	1	0	0	0	0	0
0	0	0	0	0	0	0	0	0
1	0	0	0	0	0	0	1	0
0	0	0	0	0	0	0	0	0
0	0	0	0	0	0	0	0	0
0	0	0	0	0	0	0	0	0
0	0	0	0	0	0	0	0	0
0	0	0	0	0	0	0	0	0
1	0	0	0	0	0	0	0	0
0	0	0	0	0	0	0	0	0
0	0	0	1	1	0	0	0	0

0	0	0	0	0	0	0	0	0
0	0	0	0	1	0	1	0	0
0	0	1	0	0	0	0	0	0
1	0	0	0	0	0	0	0	0
0	0	0	0	0	0	0	0	0
0	0	0	0	0	0	0	0	0
0	0	0	0	0	0	0	0	0
0	0	0	1	0	0	0	0	0
0	0	1	0	1	0	0	0	0
1	0	0	0	0	0	0	0	0
0	1	0	0	0	1	0	1	0
0	0	0	0	0	0	0	0	0
0	0	0	0	0	0	0	0	0
0	1	0	0	0	0	0	0	0
0	0	0	0	0	0	0	0	0
0	0	0	0	0	0	0	1	0
0	0	0	0	0	0	0	0	0
0	0	0	0	0	0	0	1	0
0	0	0	0	0	0	0	0	0
0	0	0	0	1	0	0	0	0
0	0	0	0	0	0	0	0	0
1	0	0	0	0	0	0	0	0
0	0	0	0	0	0	0	0	0
0	0	0	0	0	0	0	0	0
0	0	0	0	0	0	0	0	0
0	0	0	0	0	0	0	0	0
1	0	0	0	0	0	0	0	0
0	0	0	0	0	0	0	0	0
0	0	0	0	0	0	0	0	0
0	0	0	0	0	0	0	0	0
0	0	0	0	0	0	0	0	0
1	0	0	0	0	0	0	0	0
0	0	0	0	0	0	0	0	0
0	0	0	0	0	0	0	0	0
0	0	0	0	0	0	0	0	0
1	0	0	0	0	0	0	0	0
0	0	0	0	0	0	0	0	0
0	0	0	0	0	0	0	0	0
0	0	0	0	0	0	0	0	0
0	0	0	0	0	0	0	0	0
0	0	0	0	0	0	0	0	0
0	0	0	0	0	0	0	0	0
0	0	0	0	1	0	0	0	0
0	0	0	0	0	0	0	0	0
0	0	0	0	0	0	0	0	0
0	0	0	0	0	0	0	0	0
0	0	0	0	0	0	0	0	0
0	0	0	0	0	0	0	0	0
0	1	0	0	0	0	0	0	0
0	0	0	0	0	0	0	1	0
0	0	0	0	0	0	0	0	0
1	0	0	0	0	0	0	0	0
0	0	0	0	0	0	0	0	0
1	0	0	0	0	0	0	0	0
0	0	0	0	0	0	0	0	0
0	0	0	0	0	0	0	0	0

0	0	0	0	0	0	0	0	0
0	0	0	0	0	0	0	0	0
1	0	0	0	1	0	0	0	0
0	0	0	0	1	0	0	0	0
1	0	0	0	0	0	0	0	0
0	0	0	0	0	0	0	0	0
1	0	0	0	0	0	0	0	0
0	0	0	0	0	0	0	0	0
0	0	0	0	0	0	0	0	0
0	0	0	0	0	0	0	0	0
0	0	0	0	0	0	0	0	0
0	0	0	0	0	0	0	0	0
0	0	0	0	0	0	0	0	0
0	0	0	0	0	0	0	0	0
0	0	0	0	0	0	0	0	0
1	0	0	1	0	0	0	0	0
0	0	0	0	0	0	0	0	0
0	0	0	0	0	0	0	0	0
0	0	0	0	0	0	0	0	0
0	0	0	0	0	0	0	0	0
0	0	0	0	0	0	0	0	0
0	0	0	0	0	0	0	0	0
1	0	0	0	0	0	0	0	0
1	0	0	0	0	0	0	0	0
0	0	0	0	0	0	0	0	0
0	0	0	0	0	0	0	0	0
0	0	0	0	1	0	0	0	0
1	0	0	0	0	0	0	0	0
1	0	0	0	0	0	0	0	0
0	0	0	0	0	0	0	0	0
0	0	0	0	0	0	0	0	0
0	0	0	0	0	0	0	0	0
0	0	0	0	0	0	0	0	0
0	0	0	0	0	0	0	0	0
1	0	0	0	0	0	0	0	0
0	0	0	0	0	0	0	0	0
0	0	0	0	0	0	0	0	0
0	0	0	0	0	0	0	0	0
0	0	0	0	0	0	0	0	0
1	0	0	0	0	0	0	0	0
0	0	0	0	0	0	0	0	0
0	0	0	0	0	0	0	0	0
0	0	0	0	0	0	0	0	0
0	0	0	0	0	0	0	0	0
0	0	0	0	0	0	0	0	0
0	0	0	0	0	0	0	0	0
0	0	0	0	0	0	0	0	0
1	0	0	0	0	0	0	0	0
0	0	0	0	0	0	0	0	0
0	0	0	0	0	0	0	0	0
0	0	0	0	0	0	0	0	1
0	0	0	0	1	0	1	0	0
0	0	0	0	0	0	0	0	0
0	0	0	0	0	0	0	0	0
0	0	0	0	0	0	0	0	0
0	0	0	0	0	0	0	0	0

0	1	0	0	0	0	0	0	0
1	0	0	0	0	0	0	0	0
1	0	0	0	0	0	0	0	0
0	0	0	0	0	0	0	0	0
1	0	0	0	0	0	0	0	0
0	0	0	0	0	0	0	0	0
0	0	0	0	0	0	0	0	0
0	0	1	0	0	0	0	0	0
1	0	0	0	0	0	0	0	0
0	0	0	0	0	0	0	0	0
0	0	0	0	0	0	0	0	0
0	1	0	0	0	0	0	1	0
0	0	0	0	0	0	0	0	0
1	0	0	0	0	0	0	0	0
0	0	0	0	0	0	0	0	0
0	0	0	0	0	0	0	0	0
0	0	0	0	0	0	0	0	0
0	0	0	0	0	0	0	0	0
1	0	0	0	0	0	0	0	0
1	0	0	0	0	0	0	0	0
0	0	0	0	0	0	0	0	0
0	0	0	0	0	0	0	1	0
0	0	0	0	0	0	0	0	0
0	0	0	0	0	0	0	0	0
0	0	0	0	0	0	0	0	0
1	0	0	0	0	0	0	0	0
0	0	0	0	0	0	0	0	0
0	0	0	0	0	0	0	0	0
0	0	0	0	0	0	0	0	0
0	1	0	0	0	0	0	0	0
0	0	0	0	0	0	0	0	0
0	0	0	0	0	0	0	0	0
0	0	0	0	0	0	0	0	0
0	0	0	0	0	0	0	0	0
0	0	0	0	0	0	0	1	0
0	0	0	0	0	0	0	0	0
0	0	0	0	0	0	0	0	0
0	0	0	0	0	0	0	0	0
0	0	0	0	0	0	0	0	0
0	0	0	0	0	0	0	0	0
1	0	0	0	1	1	0	0	0
0	0	0	0	0	0	0	0	0
0	0	0	0	0	0	0	0	0
0	0	0	0	0	0	0	0	0
0	0	0	0	0	0	0	0	0
0	0	0	0	1	0	0	1	0
0	0	0	0	0	0	0	0	0
0	0	0	0	1	0	0	0	0
0	0	0	0	0	0	0	0	0
0	0	0	0	1	0	0	0	0
0	0	0	0	0	0	0	0	0
0	0	0	0	0	0	0	0	0
0	0	0	0	0	0	0	0	0
0	0	0	0	0	0	0	0	0
1	0	0	0	0	0	0	0	0
0	0	0	0	0	0	0	0	0

0	0	0	0	0	0	0	0	0
0	0	0	0	0	0	0	0	0
1	0	0	0	0	0	0	0	0
0	0	0	0	0	0	0	0	0
0	0	0	0	0	0	0	0	0
0	0	0	0	0	0	0	0	0
0	0	0	0	0	0	0	0	0
0	0	0	0	0	0	0	0	0
0	0	0	0	0	0	0	0	0
0	0	0	0	0	0	0	0	0
0	0	0	0	0	0	0	0	0
0	0	0	0	0	0	0	0	0
1	0	0	0	0	0	0	0	0
1	0	0	0	0	0	0	0	0
0	0	0	0	0	0	0	0	0
0	0	0	0	0	0	0	0	0
1	0	0	0	0	0	0	0	0
0	0	0	0	0	0	0	0	0
0	0	0	0	0	0	0	0	0
0	0	0	0	0	0	0	0	0
0	0	0	0	0	0	0	0	0
0	0	0	0	0	0	0	1	0
0	0	0	0	0	0	0	0	0
0	0	0	0	0	0	0	0	0
0	0	0	0	0	0	0	0	0
0	0	0	0	0	0	0	0	0
0	0	0	0	0	0	0	0	0
0	0	0	0	0	0	0	0	0
1	0	0	0	0	0	0	0	0
1	0	0	0	0	0	0	0	0
0	0	0	0	0	0	0	0	0
0	0	0	0	0	0	0	0	1
0	0	0	0	0	0	0	0	0
0	0	0	0	0	0	0	0	0
0	0	0	0	0	0	0	1	0
0	0	0	0	0	0	0	0	0
0	0	0	0	0	0	0	0	0
0	0	0	0	0	0	0	0	0
0	0	0	0	0	0	0	1	0
0	0	0	0	0	0	0	0	0
0	0	0	0	0	0	0	0	0
0	1	0	1	0	0	0	0	1
1	0	0	0	0	0	0	0	0
1	0	0	0	0	0	0	0	0
0	0	0	0	0	0	0	0	0
1	0	0	0	0	0	0	0	0
0	0	0	0	0	0	0	0	0
0	0	0	0	0	0	0	0	0
0	0	0	0	0	0	0	0	0
1	0	0	0	0	0	0	0	0
1	1	0	0	0	0	0	0	0
0	0	0	0	0	0	0	0	1
0	0	0	0	0	0	0	0	0
0	0	0	0	0	0	0	0	0
0	0	0	0	0	0	0	0	0
0	0	0	0	0	0	0	0	0

0	0	0	0	1	0	0	0	0
0	0	0	0	0	0	0	0	0
0	0	0	0	0	0	0	0	0
0	0	0	0	0	0	0	0	0
1	0	0	0	0	0	0	0	0
0	0	0	0	0	0	0	0	0
0	0	0	0	0	0	0	0	0
0	0	0	0	0	0	0	0	0
0	0	0	0	0	0	0	0	0
0	0	0	0	0	0	0	0	0
0	0	0	0	0	0	0	0	0
0	0	0	0	0	0	0	0	0
0	0	0	0	0	0	0	0	0
0	0	0	0	0	0	0	0	0
0	0	0	0	0	0	0	0	0
0	0	0	0	0	0	0	0	0
1	0	0	0	0	0	0	0	0
1	0	0	0	0	0	0	0	0
0	0	0	0	0	0	0	0	0
0	0	0	0	0	0	0	0	0
0	0	0	0	0	0	0	0	0
0	1	0	0	0	0	0	0	0
0	0	0	0	0	0	0	0	0
0	0	0	0	0	0	0	0	0
0	0	0	0	1	0	0	0	0
0	0	0	0	0	0	0	0	0
0	0	0	0	0	0	0	0	0
0	0	0	0	0	0	0	0	0
0	0	0	0	0	0	0	0	0
0	0	0	0	0	0	0	0	0
0	0	0	0	0	0	0	0	0
0	0	0	0	0	0	0	0	0
0	0	0	0	0	0	0	0	0
0	0	0	0	1	0	0	0	0
0	0	0	0	0	0	0	0	0
0	0	0	0	0	0	0	0	0
0	0	0	0	0	0	0	0	0
0	0	0	0	0	0	0	0	0
0	0	0	0	0	0	0	0	0
0	0	0	0	0	0	0	0	0
1	0	0	0	0	0	0	0	0
0	0	0	0	0	0	0	0	0
1	0	0	0	0	0	0	0	0
0	0	0	0	1	0	0	0	0
0	1	0	0	0	0	0	0	0
0	0	0	0	0	0	0	0	0
0	0	0	0	0	0	0	0	0
0	0	0	0	0	0	0	0	0
1	0	0	0	1	0	0	0	0
0	0	0	0	0	0	0	0	0
0	0	0	0	1	0	0	0	0
0	0	0	0	0	0	0	0	0
0	0	0	0	0	0	0	0	0
0	0	0	0	0	0	0	0	0
0	0	0	0	0	0	0	0	1
0	0	0	0	0	0	0	0	0

0	0	0	0	0	0	0	0	0
0	0	0	0	0	0	1	0	0
0	0	0	0	0	0	0	0	0
0	0	0	0	0	0	0	0	0
0	0	0	0	1	1	0	0	0
0	0	0	0	0	0	0	0	0
0	0	0	0	1	0	0	0	0
0	0	0	0	0	0	0	0	0
0	0	0	0	0	0	0	0	0
0	0	0	0	0	0	0	0	0
0	0	0	0	0	0	0	0	0
0	0	0	0	0	0	0	0	0
0	0	0	0	1	0	0	0	0
0	0	0	0	0	0	0	0	0
0	0	0	0	0	0	0	0	0
0	0	0	0	0	0	0	0	0
0	0	0	0	0	0	0	0	0
0	0	0	0	0	0	0	0	0
0	0	0	0	0	0	0	0	0
0	1	0	0	0	0	0	0	0
0	0	0	0	0	0	0	0	0
0	0	0	0	0	0	0	1	0
0	0	0	0	0	0	1	0	0
0	0	0	0	0	0	0	0	0
0	0	0	0	0	0	0	0	0
0	0	0	0	0	0	0	0	0
0	0	0	0	0	0	0	0	0
0	0	1	0	0	0	0	0	0
1	0	0	0	0	0	0	0	0
0	0	0	0	0	0	0	0	0
0	0	0	0	0	0	0	0	0
1	0	0	0	0	0	0	0	0
0	0	0	0	0	0	0	0	0
0	0	0	0	1	0	0	1	0
0	0	0	0	0	0	0	0	0
0	0	0	0	0	0	0	0	0
0	0	0	0	0	0	1	0	0
1	0	0	0	0	0	0	0	0
0	0	0	0	1	0	0	0	0
0	0	0	0	0	0	0	0	0
0	0	0	0	0	0	0	1	0
0	0	0	0	0	0	0	0	0
0	0	0	0	0	0	0	0	0
0	0	0	0	0	0	0	0	0
0	0	0	0	0	0	0	0	0
0	0	0	0	0	0	0	0	0
1	0	0	0	0	0	0	0	0
0	0	0	0	0	0	0	0	0
0	0	0	0	1	0	0	0	0
0	0	1	0	0	0	0	0	0
1	0	0	0	0	0	0	0	0
0	0	0	0	0	0	0	0	0
0	0	0	0	0	0	0	0	0
0	0	0	0	0	0	0	1	0
0	0	0	0	0	0	0	0	0
1	0	0	0	0	0	0	0	0

1	0	0	0	0	0	0	1	0
0	0	0	0	0	0	0	0	0
0	0	0	0	0	0	0	1	0
0	0	0	0	0	0	0	1	0
0	0	0	0	0	0	0	0	0
1	0	0	1	0	0	0	0	0
0	0	0	0	0	0	0	0	0
0	0	0	0	0	0	0	0	0
1	0	0	0	0	0	0	1	0
1	0	0	0	0	0	0	0	0
1	0	0	0	0	0	0	1	0
1	0	0	0	0	0	0	1	0
1	0	0	0	0	0	0	0	0
0	0	0	0	0	0	0	0	0
1	0	0	0	0	0	0	0	0
0	0	1	0	0	0	0	1	0
1	0	0	0	0	0	0	0	0
1	0	0	0	0	0	0	0	0
0	0	0	0	0	0	0	0	0
0	0	0	0	0	0	0	0	0
0	0	0	0	0	0	0	0	0
1	0	0	0	0	0	0	0	0
1	0	0	0	1	0	0	0	0
0	0	0	0	0	0	0	0	0
0	1	0	0	0	0	0	0	0
0	0	0	0	0	0	0	0	0
0	0	0	0	0	0	0	0	0
0	0	0	0	0	0	0	0	0
0	0	0	0	0	0	0	0	0
0	0	0	0	0	0	0	0	0
0	0	0	0	0	0	0	0	0
0	0	0	0	0	0	0	0	0
0	0	0	0	0	0	1	0	0
0	0	0	0	0	0	0	0	0
0	0	0	0	0	0	0	0	0
1	0	0	0	0	0	0	0	0
0	0	0	0	0	0	0	1	1
0	0	0	0	0	0	0	0	0
0	0	0	0	0	0	0	0	0
0	0	0	0	0	0	0	0	0
0	0	0	0	0	0	0	0	0
0	0	0	0	0	0	1	0	0
0	0	0	0	0	0	0	0	0
0	0	0	0	0	0	0	0	0
1	0	0	0	1	0	1	0	0
0	0	0	0	0	0	0	0	0
0	0	0	0	0	0	0	0	0
0	0	0	0	0	0	0	0	0
0	0	0	0	0	0	0	1	0
0	0	0	0	0	1	0	0	0
0	0	0	0	0	0	0	0	0
0	0	0	0	0	0	0	0	0
0	0	0	1	0	0	0	0	0
1	0	0	0	0	0	0	0	0
0	0	0	0	0	0	0	0	0
1	0	0	0	0	0	0	0	0

1	0	0	0	0	0	0	0	0
0	0	0	0	0	0	0	0	0
0	0	0	0	0	0	0	0	0
0	0	0	0	0	0	0	0	0
0	0	0	0	0	0	0	0	0
0	0	0	0	0	0	0	0	0
0	0	0	0	0	0	0	0	0
1	0	0	0	0	0	0	0	0
0	0	0	0	0	0	0	0	0
0	0	0	0	0	0	0	0	0
1	0	0	0	0	0	0	0	0
0	0	0	0	0	0	0	0	0
0	0	0	0	0	0	0	0	0
0	1	0	0	0	0	0	0	0
1	1	0	0	0	0	0	0	0
0	0	0	0	0	0	0	0	0
1	0	0	0	1	0	0	0	0
0	0	0	0	0	0	0	0	0
0	0	0	0	0	0	0	0	0
0	0	0	0	0	0	0	0	0
0	0	0	0	0	0	0	1	0
0	0	0	0	0	0	0	0	0
0	0	0	0	0	0	0	0	0
0	0	0	0	0	0	0	0	0
0	0	0	0	0	0	0	0	0
0	0	0	0	0	0	0	0	0
0	0	0	0	1	0	0	0	0
0	0	0	0	0	0	0	0	0
1	0	0	0	0	0	0	0	0
0	1	0	0	0	0	0	0	0
0	0	0	0	0	0	0	0	0
1	1	0	0	0	0	0	0	0
0	0	0	0	0	0	0	0	0
1	0	0	0	0	0	0	0	0
0	0	0	0	0	0	0	0	0
1	0	0	0	0	0	0	0	0
0	0	1	0	0	0	0	0	0
0	0	0	0	1	0	0	0	0
0	0	0	0	1	0	0	0	0
0	0	0	0	1	0	0	0	0
0	0	0	0	0	0	0	0	0
0	0	0	0	0	0	0	0	0
0	0	0	0	0	0	0	0	0
0	0	0	0	0	0	0	0	0
0	0	0	0	0	0	0	0	0
0	0	0	0	0	0	0	0	0
1	0	0	0	0	0	0	1	0
0	0	0	0	0	0	0	0	0
0	0	0	0	0	0	0	0	0
0	0	0	0	1	0	0	0	0
0	0	0	0	0	0	0	0	0
1	0	1	0	0	0	0	0	0
0	0	0	0	0	0	0	0	0
1	0	0	0	1	0	1	0	0
0	0	0	0	0	0	0	1	0
0	0	0	0	1	0	0	1	0

1	0	0	0	0	0	0	0	0
0	0	0	0	0	0	0	0	0
0	0	0	0	1	0	0	0	0
0	0	0	0	0	0	0	0	0
0	0	0	0	0	0	0	0	0
0	0	0	0	0	0	0	0	0
1	0	0	0	0	0	0	0	0
0	0	0	0	0	0	0	0	0
1	0	0	0	0	0	0	0	0
0	0	0	0	0	0	1	0	0
1	0	0	0	0	0	0	0	0
0	0	0	0	0	0	0	0	0
0	0	0	0	0	0	0	0	0
0	0	0	0	0	0	0	0	0
0	0	0	0	0	0	0	0	0
0	0	0	0	0	0	0	0	0
0	0	0	0	0	0	0	0	0
0	0	0	0	0	0	0	0	0
0	0	0	0	0	0	0	0	0
0	0	0	0	0	0	0	0	0
0	0	0	0	0	0	0	0	0
0	0	0	0	0	0	0	0	0
0	0	0	0	0	0	0	0	0
1	0	0	0	0	0	0	0	0
0	0	0	0	0	0	0	0	0
0	0	0	0	0	0	0	0	0
1	0	0	1	0	0	0	0	0
0	0	0	0	0	0	0	0	0
0	0	0	0	0	0	0	0	0
0	0	0	0	0	0	0	0	0
0	0	0	0	0	0	0	0	0
0	0	0	0	0	0	0	0	0
0	0	0	0	0	0	0	0	0
0	0	0	0	0	0	0	0	0
0	0	0	0	0	0	0	0	0
1	0	0	0	0	0	0	0	0
0	0	0	0	0	0	0	0	0
0	0	0	0	0	0	0	0	0
0	0	0	0	0	0	0	0	0
1	0	0	0	1	0	0	0	0
1	0	0	0	0	0	0	0	0
0	0	0	0	0	0	1	0	0
0	0	0	0	0	0	0	0	0
0	1	0	0	0	0	0	0	0
0	0	1	0	0	0	0	0	0
0	0	0	0	0	0	0	0	0
0	0	0	0	1	0	0	0	0
0	0	0	0	0	0	0	0	0
0	0	0	0	0	0	0	0	0
0	0	0	0	0	0	0	0	0
0	0	0	0	0	0	0	0	0
1	0	0	0	0	0	0	0	0
1	0	0	0	0	0	0	0	0
0	0	0	0	0	0	0	0	0
0	0	0	0	0	0	0	0	0
0	0	0	0	0	0	0	0	0

1	0	0	0	0	0	0	0	0
0	0	0	0	0	0	0	0	0
0	0	0	0	0	0	0	0	0
0	0	0	0	0	0	0	0	0
0	0	0	0	0	0	0	0	0
1	0	0	0	0	0	0	0	0
0	0	0	0	0	0	0	0	0
0	0	0	0	0	0	0	0	0
0	0	0	0	0	0	0	0	0
0	0	0	0	0	0	0	0	0
0	0	0	0	0	0	0	0	0
0	0	0	0	0	0	0	0	0
0	0	0	0	1	0	0	0	0
0	0	0	0	0	0	0	0	0
0	0	0	0	1	0	0	0	0
1	0	0	0	0	0	0	0	0
1	0	0	0	0	0	0	0	0
1	0	0	0	0	0	0	0	0
0	0	0	0	0	0	0	0	0
0	0	0	0	0	0	0	0	0
0	0	0	0	0	0	1	0	0
1	0	0	0	0	0	0	0	0
0	0	0	0	0	0	0	0	0
0	0	0	0	0	0	0	0	0
0	0	0	0	0	0	0	0	0
0	0	0	0	0	0	0	0	0
0	0	0	0	0	0	0	0	0
0	0	0	0	0	0	0	0	0
0	0	0	0	0	0	0	0	0
0	0	0	0	0	0	0	0	0
0	0	0	0	1	0	0	0	0
0	0	0	0	0	0	0	0	0
0	0	0	0	0	0	0	1	0
0	0	0	0	0	0	0	0	0
1	0	0	0	0	0	0	0	0
1	0	0	0	1	0	0	0	0
0	0	0	0	0	0	1	1	0
1	0	0	0	0	0	0	1	0
0	0	0	0	0	0	0	0	0
0	0	0	0	0	0	0	0	0
1	0	0	0	0	0	0	1	0
0	0	0	0	0	0	0	0	0
0	0	0	0	0	0	0	0	0
0	0	0	0	1	0	0	0	0
0	0	0	0	0	0	0	0	0
0	0	0	0	0	0	0	0	0
0	0	0	0	0	0	0	0	0
0	1	0	0	1	0	0	0	0
0	0	0	0	0	0	0	0	0
0	0	0	0	0	0	0	0	0
0	0	0	0	0	0	0	0	0
0	0	0	0	0	0	0	0	0
0	0	0	0	0	0	0	0	0
0	0	0	0	0	0	0	0	0
0	1	0	0	0	0	0	0	0
0	0	0	0	0	0	0	0	0

0	0	0	0	0	0	0	1	0
0	0	0	0	0	0	0	0	0
0	0	0	0	1	0	0	0	0
0	0	0	0	0	0	0	0	0
1	0	0	0	0	0	0	0	0
0	0	0	0	0	0	0	0	0
0	0	0	0	0	0	0	0	0
0	0	0	0	0	0	0	0	0
0	0	0	0	0	0	0	0	0
1	0	0	0	0	0	0	0	0
0	0	0	0	0	0	0	1	0
0	0	0	0	0	0	0	0	0
1	0	0	0	0	0	0	0	0
0	0	0	0	0	0	0	1	0
0	0	0	0	0	0	0	0	0
1	0	0	0	0	0	0	0	0
1	0	0	0	0	0	0	1	0
0	0	0	0	1	0	0	0	0
0	0	0	0	0	0	0	0	0
0	0	0	0	0	0	0	0	0
0	0	0	0	0	0	0	0	0
0	0	0	0	0	0	0	0	0
0	0	0	0	0	0	0	0	0
1	0	0	0	1	0	0	0	0
0	0	0	0	0	0	0	0	0
0	0	0	0	0	1	0	0	0
1	0	0	0	0	0	0	1	0
1	0	0	0	0	0	0	1	0
0	0	0	0	0	0	0	0	1
0	0	0	0	0	0	0	0	0
0	0	0	0	0	0	0	0	0
1	0	0	1	0	0	0	1	0
0	0	0	0	0	0	0	0	0
1	0	1	0	0	0	0	0	0
0	0	0	0	1	0	0	0	0
0	0	0	0	0	0	0	1	0
0	0	0	0	0	0	0	1	0
0	0	0	0	0	0	0	0	0
0	0	0	0	0	0	0	0	0
0	0	0	1	0	0	0	0	0
0	0	0	0	0	0	0	0	0
0	0	0	0	0	0	0	0	0
0	0	0	0	0	0	0	0	0
0	0	0	0	0	0	0	0	0
0	0	0	0	0	0	0	0	0
0	0	0	0	0	0	0	0	0
0	0	0	0	0	0	0	0	0
0	0	0	0	0	0	0	0	0
0	0	0	0	0	0	0	0	0
0	0	0	0	0	0	0	0	0
0	0	0	0	0	0	0	0	0
0	0	0	0	1	0	1	0	0
0	1	0	0	0	0	0	0	0
0	0	0	1	0	0	0	0	0
0	0	0	0	0	0	0	0	0
1	0	0	0	0	0	0	0	0

0	0	0	0	0	0	0	0	0
0	0	0	0	0	0	0	1	0
0	1	0	0	0	0	0	0	0
1	0	0	0	0	0	0	1	0
0	1	0	0	0	0	0	0	0
0	0	0	0	0	0	0	1	0
0	0	0	0	0	0	0	1	0
0	0	0	0	0	0	0	0	0
0	0	0	0	0	0	0	0	0
0	0	0	0	0	0	0	0	0
0	0	0	0	1	0	0	0	0
0	0	0	0	0	0	0	0	0
1	0	0	0	0	0	0	0	0
0	0	0	0	0	0	0	0	0
0	0	0	0	0	0	0	0	0
0	0	0	0	1	0	0	0	0
0	0	0	0	0	0	0	0	0
0	0	0	0	0	0	0	1	0
0	0	0	0	0	0	0	0	0
0	1	0	0	0	0	0	0	0
0	0	0	0	0	0	0	0	0
0	0	0	0	1	0	0	0	0
0	0	1	1	0	0	0	0	0
0	0	1	0	0	0	0	0	0
0	0	0	0	0	0	0	0	0
0	0	0	0	0	0	0	0	0
0	0	0	0	0	0	0	0	0
0	1	0	0	0	0	0	0	1
0	0	0	0	0	0	0	0	0
1	0	0	0	0	0	0	0	0
0	0	0	0	0	0	0	1	0
0	0	0	0	0	0	0	0	0
0	1	0	0	0	0	0	0	0
1	0	0	0	0	0	0	0	0
0	0	0	0	0	0	0	1	0
0	0	0	0	0	0	0	0	0
0	0	0	0	1	0	0	1	0
0	0	0	0	0	0	0	1	0
0	0	0	0	0	0	0	0	0
0	0	0	0	0	0	0	0	0
0	0	0	0	0	0	0	0	0
0	0	0	0	0	0	0	0	0
0	0	0	0	0	0	0	1	0
1	0	0	0	1	0	0	0	0
0	0	0	0	0	0	0	1	0
1	1	0	0	0	0	0	0	0
0	0	0	0	0	0	0	0	0
0	0	0	0	0	0	0	0	0
1	1	0	0	0	0	0	0	0
0	0	0	0	0	0	0	1	0
0	0	0	0	0	0	0	0	0
0	0	0	0	0	0	0	0	0
0	0	0	0	0	0	1	0	0
0	0	0	0	0	0	0	0	0
0	0	0	0	0	0	0	0	0
1	0	0	0	0	0	0	0	0

0	0	0	0	0	0	0	0	0
0	0	0	0	1	0	0	0	0
0	0	0	0	0	0	1	0	0
0	0	0	0	1	0	0	0	0
0	0	0	0	0	0	0	0	0
0	0	0	0	0	0	1	0	0
0	0	0	0	1	0	0	0	0
0	0	0	0	0	0	0	0	0
1	0	0	0	0	0	0	0	0
1	0	0	0	0	0	0	0	0
0	0	0	0	0	0	0	0	0
0	0	0	0	0	0	0	0	0
0	0	0	0	0	0	0	0	0
0	0	0	0	0	0	0	0	0
0	0	0	1	0	0	0	0	0
0	0	0	0	0	0	0	0	0
0	0	0	0	1	0	0	0	0
0	0	0	0	0	0	0	0	0
0	0	0	0	1	0	0	1	0
1	0	0	0	0	0	0	0	0
0	0	0	1	0	0	0	0	0
0	0	0	0	0	0	0	0	0
0	0	0	0	0	0	0	0	0
1	0	0	0	0	0	0	0	0
0	0	0	0	0	0	0	0	0
0	0	0	0	0	0	0	0	0
1	0	0	0	0	0	0	0	0
1	0	0	0	0	0	0	0	1
0	0	0	0	0	0	0	1	0
0	0	0	0	0	0	0	0	0
0	0	0	0	0	0	0	0	0
0	0	0	0	0	0	0	0	0
0	0	0	0	0	0	0	0	0
0	0	0	0	0	0	0	0	0
0	0	0	0	0	0	0	0	0
0	0	0	0	0	0	0	0	0
0	0	0	0	0	0	0	0	0
0	0	0	0	0	0	0	0	0
0	0	0	0	0	0	0	0	0
0	0	0	0	0	0	0	0	0
1	0	0	0	0	0	0	0	0
0	0	0	0	0	0	0	0	0
0	0	0	0	0	0	0	0	0
0	0	1	0	0	0	0	0	0
0	0	0	0	0	0	0	0	0
0	0	0	0	0	0	0	0	0
0	0	0	0	0	0	0	0	0
1	0	0	0	0	1	0	0	0
0	0	0	0	0	0	0	0	0
0	0	0	0	0	0	0	0	0
0	0	0	0	0	0	0	0	0
1	0	0	0	0	0	0	0	0
0	0	0	0	0	0	0	0	0
0	1	0	0	0	0	0	0	0
0	0	0	0	0	0	0	0	0
0	0	0	0	0	0	0	0	0

0	0	0	0	0	0	0	0	0
0	0	0	0	0	0	0	0	0
1	0	0	0	0	0	0	0	0
1	0	0	0	0	0	0	0	0
0	0	0	0	0	0	0	0	0
0	0	0	0	0	0	0	0	0
1	0	0	0	0	0	0	0	0
0	0	0	0	0	0	0	0	0
1	0	0	0	0	0	0	0	0
0	0	0	0	0	0	0	0	0
0	0	0	0	0	0	0	0	0
0	0	0	0	0	0	0	0	0
1	1	0	0	0	0	0	0	0
0	0	0	0	0	0	0	0	0
0	0	0	0	0	0	0	0	0
0	0	0	0	0	0	0	0	0
0	0	0	0	0	0	0	0	0
0	0	0	0	0	0	0	0	0
0	0	0	0	0	0	0	0	0
0	0	0	0	0	0	0	0	0
0	0	0	0	0	0	0	0	0
0	0	0	0	0	0	0	0	0
1	0	0	0	0	0	0	0	0
0	0	0	0	0	0	0	0	0
0	0	0	0	0	0	0	0	0
0	0	0	0	0	0	0	0	0
0	0	0	0	0	0	0	0	0
0	0	0	0	0	0	0	0	0
0	0	0	0	0	0	0	0	0
0	0	0	0	0	0	0	0	0
0	0	0	0	0	0	0	0	0
0	0	0	0	0	0	0	0	0
0	0	0	0	0	0	0	0	0
0	0	0	0	0	0	0	0	0
0	0	0	0	0	0	0	0	0
0	0	0	0	1	0	0	0	0
0	0	0	0	0	0	0	1	1
1	0	0	0	0	0	0	0	0
1	0	0	0	0	0	0	0	0
0	0	0	0	0	0	0	0	0
1	0	0	0	1	1	0	0	0
0	0	0	0	0	0	0	0	0
0	0	0	0	0	0	0	1	0
0	0	0	0	0	0	0	0	0
0	0	0	0	1	0	0	0	0
0	0	0	0	0	0	0	1	0
0	0	0	0	0	0	0	0	0
1	0	0	0	0	0	0	0	0
1	0	0	0	0	0	0	0	0
0	0	0	0	0	0	0	0	0
0	0	0	0	0	0	0	0	0
0	0	0	0	0	0	0	0	0
0	0	0	0	0	0	0	0	0
0	0	0	0	1	0	0	0	0
0	0	0	0	0	0	0	0	0
1	0	0	0	0	0	0	0	0

1	1	0	0	0	0	0	0	0
0	0	0	0	0	0	0	0	0
0	0	0	0	0	0	0	0	0
0	0	0	0	0	0	0	0	0
0	1	0	0	0	0	0	0	0
0	0	0	0	0	0	0	0	0
0	1	0	0	0	0	0	0	0
0	1	0	0	0	0	0	0	0
0	0	0	0	0	0	0	0	1
1	0	0	0	0	0	0	0	0
0	0	0	0	0	0	0	0	0
1	0	0	0	0	0	0	0	0
1	0	0	0	0	0	0	0	0
0	0	0	0	0	0	0	0	0
1	0	0	0	1	0	0	0	0
0	0	0	0	0	0	0	1	1
0	0	0	0	0	0	0	0	0
0	0	0	0	0	0	0	0	0
0	1	0	0	0	0	0	0	0
0	0	0	0	0	0	0	0	0
0	0	0	0	0	0	0	0	0
0	0	0	0	0	0	0	0	0
0	0	0	0	0	0	0	0	0
0	0	0	0	0	0	0	0	0
0	0	0	0	0	0	0	0	0
0	0	0	0	0	0	0	0	0
0	0	0	0	0	0	0	0	0
0	0	0	0	0	0	0	1	1
0	0	0	0	0	0	0	0	0
0	0	0	0	0	0	0	0	0
1	0	0	0	0	0	0	0	0
0	0	0	0	0	0	0	0	0
0	0	0	0	0	0	0	0	0
0	0	0	0	0	0	0	0	0
0	0	0	0	0	0	0	0	0
0	0	0	0	0	0	0	0	0
0	0	0	0	0	0	0	0	0
0	0	0	0	0	0	0	0	0
0	0	0	0	0	0	0	1	0
0	0	0	0	0	0	0	0	0
0	0	0	0	0	0	0	0	0
0	0	0	0	0	0	0	0	0
0	0	0	0	0	0	0	0	0
0	0	0	0	0	0	0	0	0
0	0	0	0	0	0	0	0	0
0	0	0	0	0	0	0	1	0
0	0	0	0	0	0	0	0	0
0	0	0	0	0	0	0	0	0
0	0	0	0	0	0	1	0	0
0	0	0	0	0	0	0	0	0
0	0	0	0	0	0	0	1	0
0	0	0	0	0	0	0	0	0
0	0	0	0	0	0	0	0	0
0	0	0	0	0	0	0	0	0
0	0	0	0	0	0	0	0	0
0	0	0	0	0	0	0	0	0

0	0	0	0	0	0	0	0	0
0	0	0	0	0	0	1	0	0
0	0	0	0	0	0	0	0	0
0	1	0	0	0	0	0	0	0
0	0	0	0	0	0	0	0	0
0	0	0	0	1	0	0	0	0
0	0	0	0	0	0	0	0	0
0	0	0	0	0	0	0	0	0
0	0	0	0	0	0	0	0	0
0	1	0	0	0	0	0	0	1
0	0	0	0	0	0	0	0	0
0	0	0	0	0	0	0	0	0
0	0	0	0	0	0	0	0	0
0	0	0	0	0	0	0	0	0
0	0	0	0	0	0	0	1	0
0	0	0	0	1	0	0	0	0
0	0	0	0	0	0	0	0	0
0	0	0	0	0	0	0	0	0
0	0	0	0	0	0	0	1	0
0	0	0	0	0	0	0	0	0
0	0	0	0	0	0	0	0	0
0	0	0	0	0	0	0	0	0
0	0	0	0	0	0	0	0	0
0	0	0	0	0	0	0	0	0
0	0	0	0	0	0	0	0	0
0	0	0	0	0	0	0	0	0
0	0	0	0	1	0	0	0	0
0	0	1	0	0	1	0	1	0
0	0	0	0	0	0	0	0	0
0	0	0	0	0	0	0	0	0
0	0	0	0	0	0	0	0	0
0	0	0	0	0	0	0	0	0
0	0	0	0	0	0	0	0	0
0	0	0	0	0	0	0	0	0
0	0	0	0	0	0	0	0	0
0	0	0	0	0	0	0	0	0
0	0	0	0	0	0	0	0	0
0	0	0	0	1	0	0	0	0
0	0	0	0	0	0	0	1	0
0	0	0	0	0	0	0	0	0
0	0	0	0	0	0	0	0	0
0	0	0	0	0	0	0	0	0
0	0	0	0	0	0	0	0	0
0	0	0	0	0	0	0	1	0
0	0	0	0	0	0	0	0	0
0	0	0	0	0	0	0	0	0
0	0	0	0	0	0	0	0	0
0	0	0	1	0	0	0	0	0
1	0	0	0	0	0	0	0	0
0	0	0	1	0	0	0	0	0
0	0	0	0	0	0	0	1	0
0	0	0	0	0	0	0	0	0
0	0	0	0	0	0	0	0	0
1	0	0	0	0	0	0	0	0
0	0	0	0	0	0	0	0	0
0	0	0	0	0	0	0	0	0

0	0	0	0	0	0	0	0	0
0	0	0	0	0	0	0	0	0
0	0	0	0	0	0	0	0	0
0	0	0	0	0	0	0	0	0
0	1	0	0	0	0	0	0	0
0	0	0	0	0	0	0	0	0
0	0	0	0	1	0	0	0	0
0	0	0	0	0	0	0	0	0
0	0	0	0	0	0	0	1	0
0	0	0	0	0	0	0	1	0
0	0	0	0	0	0	0	0	0
0	0	0	0	0	0	0	0	0
0	0	0	0	0	0	0	1	0
1	0	0	0	0	0	0	0	0
0	0	0	0	0	0	0	1	0
0	0	0	0	0	0	0	1	0
0	0	0	0	0	0	0	0	0
0	0	0	0	0	0	0	0	0
0	0	0	0	0	0	0	1	0
0	1	0	0	0	0	0	1	0
0	0	0	0	0	0	0	0	0
0	0	0	0	0	0	0	0	0
0	0	0	0	0	0	0	1	0
0	0	0	0	0	0	0	1	0
0	0	0	0	0	0	0	0	0
0	0	0	0	0	0	0	1	0
0	0	0	0	0	0	0	0	0
0	0	0	0	0	0	0	0	0
0	0	0	0	0	0	0	0	0
1	0	0	0	0	0	0	0	0
1	0	0	0	0	0	0	0	0
0	0	0	0	0	0	0	0	0
1	0	0	0	0	0	0	0	0
0	0	0	0	0	0	0	0	0
0	0	0	0	0	0	0	0	0
0	0	0	0	0	0	0	0	0
0	0	0	0	0	0	0	0	0
1	0	0	0	0	0	0	0	0
1	0	0	0	0	0	0	0	0
0	0	0	0	0	0	0	0	0
1	0	0	0	0	0	0	0	0
1	0	0	0	0	0	0	0	0
0	0	0	0	0	0	0	0	0
0	0	0	0	0	0	0	0	0
0	0	0	0	0	0	0	0	0
0	0	0	0	0	0	0	0	0
0	0	0	0	0	0	0	0	0
0	0	0	0	0	0	0	0	0
1	0	0	0	0	0	0	0	0
1	1	0	0	0	0	0	0	0
0	0	0	0	0	0	0	0	0
1	0	0	0	0	0	0	0	0
1	0	0	0	0	0	0	0	0
0	0	0	0	0	0	0	0	0
0	0	0	0	0	0	0	0	0
0	0	0	0	0	0	0	0	0

0	0	0	0	0	0	0	0	0
0	0	0	0	0	0	0	0	0
0	0	0	0	0	0	0	0	0
0	0	0	0	0	0	0	0	0
1	0	0	0	0	0	1	0	0
0	0	0	0	0	0	0	0	0
1	0	0	0	0	0	0	0	0
1	0	0	0	0	0	0	0	0
0	0	0	0	0	0	0	0	0
0	0	0	0	0	0	0	0	0
1	0	0	0	0	0	0	0	0
0	0	0	0	0	0	0	0	0
1	0	0	0	0	0	0	0	0
0	0	0	0	0	0	0	0	0
0	0	0	0	0	0	0	0	0
1	0	0	0	1	0	0	0	0
0	0	0	0	0	0	0	1	0
0	0	0	0	0	0	0	0	0
0	0	0	0	0	0	0	0	0
0	0	0	0	0	0	0	0	0
0	0	0	0	0	0	0	0	0
0	0	0	0	0	0	0	0	0
0	0	0	0	0	0	0	0	0
0	0	0	0	0	0	0	1	0
0	0	0	0	0	0	0	1	0
1	0	0	0	0	0	0	0	0
1	0	0	0	0	0	0	0	0
1	0	0	0	0	0	0	0	0
1	0	0	0	0	0	0	0	0
0	0	0	0	0	0	0	0	0
1	0	0	0	0	0	0	0	0
1	0	0	0	0	0	0	0	0
1	0	0	0	0	0	0	0	0
0	0	0	0	0	0	0	0	0
0	0	0	0	0	0	0	0	0
0	0	0	0	0	0	0	0	0
0	0	0	0	0	0	0	0	0
0	0	0	0	0	0	0	0	0
0	0	0	0	0	0	0	0	0
0	0	0	0	0	0	0	0	0
0	0	0	0	0	0	0	1	0
0	0	0	0	0	0	0	0	0
1	0	0	0	0	0	0	0	0
0	1	0	0	0	0	0	0	0
0	0	0	0	0	0	0	0	0
0	0	0	0	0	0	0	1	0
0	0	0	0	0	0	0	0	0
0	0	0	0	0	0	0	1	0
0	0	0	0	0	0	0	1	0
0	0	0	0	0	0	0	0	0
0	1	0	0	0	0	0	0	0
0	0	0	0	0	0	0	0	0
0	0	0	0	0	0	0	0	0
0	0	0	0	0	0	0	0	0
1	0	0	0	0	0	0	0	0
0	0	0	0	0	0	0	1	0

1	0	0	0	0	0	0	0	0
1	0	0	0	0	0	0	0	0
0	0	0	0	0	0	0	0	0
0	0	0	0	0	0	0	0	0
0	0	0	0	0	0	0	0	0
0	0	0	0	0	0	0	0	0
1	0	0	0	0	0	0	0	0
1	0	0	0	0	0	1	0	1
1	0	0	0	0	0	1	0	0
0	0	0	0	0	0	0	0	0
1	0	0	0	1	0	0	0	0
1	0	0	0	0	0	0	0	0
0	0	0	0	0	0	0	0	0
0	0	0	0	0	0	0	0	0
1	0	0	0	0	0	0	0	0
0	0	0	0	0	0	0	0	0
0	0	0	0	0	0	0	0	0
0	0	0	0	0	0	0	0	0
0	0	0	0	0	0	0	0	0
0	0	0	0	0	0	0	0	0
0	0	0	0	0	0	0	0	0
0	0	0	0	0	0	0	0	0
0	0	0	0	0	0	0	0	0
0	0	0	0	0	0	0	0	0
1	0	0	0	0	0	0	0	0
0	0	1	0	0	0	0	0	0
1	0	0	0	0	0	0	0	0
0	0	0	0	0	0	0	0	0
0	1	0	0	0	0	0	0	0
0	0	0	0	1	0	0	0	0
0	0	0	0	0	0	0	0	0
0	0	0	0	0	0	0	0	0
0	1	1	0	0	0	0	0	0
0	0	0	0	0	0	0	1	0
0	1	0	0	0	0	0	0	0
0	0	0	0	0	0	0	0	0
0	0	0	0	0	0	0	0	0
0	0	0	0	0	0	0	0	0
0	0	0	0	0	0	0	0	0
0	0	0	0	0	0	0	0	0
0	0	0	0	0	0	0	0	0
0	0	0	0	1	0	0	0	0
1	0	0	0	0	0	0	0	0
1	0	0	0	0	0	0	0	0
0	0	0	0	0	0	0	0	0
0	0	0	0	0	0	0	0	0
0	0	0	0	0	0	0	0	0
1	1	0	0	0	0	0	0	0
1	0	0	0	0	0	0	0	0
0	1	0	0	0	0	0	1	0
0	0	0	0	0	0	1	0	0
0	0	0	0	0	0	0	0	0
0	0	0	0	0	0	0	0	0
0	0	0	0	0	0	0	0	0
0	0	0	0	0	0	0	1	0
0	0	0	0	0	0	0	0	0

0	0	0	0	0	0	0	0	0
0	0	0	0	0	0	0	1	0
0	0	0	0	0	0	0	0	0
0	0	0	0	0	0	0	0	0
0	0	0	0	0	0	0	0	0
0	0	0	0	0	0	0	0	0
0	0	0	0	0	0	0	0	0
0	0	0	0	0	0	0	0	0
0	0	0	0	0	0	0	0	0
1	0	0	1	0	0	0	0	0
0	0	0	0	0	0	0	1	0
0	0	0	0	0	0	0	0	0
0	0	0	0	0	0	0	0	0
1	0	0	0	0	0	0	1	0
1	0	0	0	0	0	0	0	0
1	0	0	0	0	0	0	0	0
0	0	0	0	0	0	0	0	0
0	0	0	0	0	0	0	0	0
0	0	0	0	0	0	0	0	0
0	0	0	0	0	0	0	0	0
0	0	0	0	0	0	0	0	0
0	0	0	0	0	0	0	0	0
0	0	0	0	0	0	0	0	0
0	0	0	0	0	0	0	0	0
0	0	0	0	0	0	0	0	0
0	0	0	0	0	0	0	0	0
0	0	0	0	0	0	0	0	0
0	0	0	0	0	0	0	0	0
0	0	0	1	0	0	0	0	0
0	1	0	0	0	0	0	0	0
0	0	0	0	0	0	0	0	0
1	0	0	0	1	0	0	0	0
0	1	0	0	0	0	0	0	0
0	0	0	0	0	0	0	0	0
0	0	0	0	0	0	0	0	0
0	0	0	0	0	0	0	0	0
0	0	0	0	0	0	0	0	0
0	0	0	0	0	0	0	0	0
0	0	0	0	0	0	0	0	0
0	0	0	0	0	0	0	0	0
0	0	0	0	0	0	0	0	0
0	0	0	0	0	0	0	0	0
0	0	0	0	0	0	0	0	0
0	0	0	0	0	0	0	0	0
0	0	0	0	0	0	0	0	1
0	0	0	0	0	0	0	0	0
0	0	0	0	0	0	0	0	0
0	0	0	0	0	0	0	0	0
0	0	0	0	0	0	0	0	0
0	0	1	0	0	0	0	0	0
0	0	0	0	0	0	0	0	0
0	0	0	0	0	0	0	1	0
0	0	0	0	0	0	0	0	0
0	0	0	0	0	0	0	0	0
0	1	0	0	0	0	0	0	0
0	1	0	0	0	0	0	0	0

0	0	0	0	0	0	1	0	0
0	0	0	0	0	0	0	0	0
0	0	0	0	0	0	0	0	0
1	0	0	0	0	0	0	0	0
0	0	0	0	0	0	0	0	0
0	0	0	0	0	0	0	0	0
1	0	0	0	0	0	0	0	0
0	0	0	0	0	0	0	0	0
0	0	0	0	0	0	0	0	0
0	0	0	0	0	0	0	0	0
0	1	0	0	0	0	0	0	0
0	0	0	0	0	0	0	0	0
0	0	0	0	0	0	0	0	0
1	0	1	0	0	0	0	1	0
0	0	0	0	0	0	0	0	0
0	0	0	0	0	0	0	0	0
0	0	0	0	0	0	0	0	0
0	0	0	0	0	0	0	0	0
0	0	0	0	0	0	0	0	0
0	0	0	0	0	0	0	0	0
0	0	0	0	0	0	0	0	0
0	0	0	0	0	0	0	0	0
0	0	0	0	0	0	0	0	0
1	0	0	0	0	0	0	0	0
0	0	0	0	0	0	0	0	0
0	0	0	1	0	0	0	0	0
0	0	0	0	0	0	0	0	0
0	0	0	0	0	0	0	0	0
0	0	0	0	0	0	0	0	0
0	0	0	0	0	0	0	0	0
0	0	0	0	0	0	0	0	0
1	0	0	0	0	0	0	0	0
0	0	0	0	0	0	0	0	0
0	0	0	0	0	0	0	0	0
0	0	0	0	0	0	0	0	0
0	0	0	0	0	0	0	0	1
0	1	0	0	0	0	0	0	0
0	0	0	0	0	0	0	0	0
1	0	0	0	0	0	0	0	0
0	0	0	0	0	0	0	0	0
0	0	0	0	0	0	0	0	0
0	0	0	0	0	0	0	0	0
0	0	0	0	0	0	0	0	0
0	0	0	0	0	0	0	0	0
0	0	1	0	0	0	0	1	0
0	0	0	0	0	0	0	0	0
0	0	0	0	0	0	0	0	0
0	0	0	0	0	0	0	0	0
0	0	0	0	0	0	0	0	0
0	0	0	0	0	0	0	0	0
0	0	0	0	0	0	0	0	0
0	0	0	0	0	0	0	0	0
0	0	0	0	0	0	1	0	0
0	0	0	0	0	0	0	0	0
1	0	0	0	0	0	1	0	0
1	0	0	0	0	0	0	0	0

1	0	0	0	0	0	0	0	0
0	0	0	0	0	0	0	0	0
0	0	0	0	0	0	0	0	0
0	0	0	0	0	0	0	0	1
1	0	0	0	0	0	0	0	0
1	0	0	0	0	0	0	0	0
0	0	0	0	0	0	0	0	0
1	0	0	0	0	0	0	0	0
0	0	0	0	0	0	0	0	0
1	0	0	0	0	0	0	0	0
0	0	0	0	0	0	0	0	0
0	0	0	0	0	0	0	0	0
0	0	1	0	0	0	0	0	0
0	0	0	0	0	0	0	0	0
0	0	0	0	0	0	0	0	0
0	0	0	0	0	0	0	0	0
0	0	0	0	0	0	0	1	0
1	0	0	0	0	0	0	0	0
0	0	0	0	0	0	0	0	0
0	0	0	0	0	0	0	1	0
0	0	0	0	1	0	0	0	0
0	0	0	0	0	0	0	0	0
0	0	0	0	0	0	1	0	0
0	0	0	0	0	0	0	0	0
0	0	0	0	0	0	0	0	0
0	0	0	0	0	0	0	1	0
0	0	0	0	0	0	0	0	0
0	0	0	0	0	0	0	0	0
1	0	0	0	0	0	0	0	0
1	0	0	0	0	0	0	0	0
0	0	0	0	0	0	0	1	0
0	0	0	0	1	0	0	0	0
1	0	0	0	0	0	0	0	0
0	0	0	0	0	0	0	0	1
1	0	0	0	0	0	0	0	0
0	0	0	0	0	0	0	1	0
0	0	0	0	0	0	0	1	0
0	0	0	0	0	0	0	0	0
1	0	0	0	0	0	0	0	0
0	0	0	0	0	0	0	0	0
0	0	0	0	0	0	0	0	0
0	0	0	0	0	0	0	0	0
0	0	0	0	0	0	0	0	0
1	0	0	0	0	0	0	1	0
0	0	0	0	0	0	0	0	0
1	0	0	0	1	0	0	0	0
0	0	0	0	0	0	0	0	0
0	0	0	0	0	0	0	0	0
1	0	0	0	0	0	0	0	0
0	0	0	0	0	0	0	0	0
0	0	0	0	0	0	1	0	0
0	0	1	0	0	0	0	0	0
0	0	0	0	0	0	0	0	0
0	0	0	0	0	0	0	1	0
1	0	0	0	0	0	0	1	0
0	0	1	0	0	0	0	0	0

0	0	0	0	0	0	0	0	0
0	0	0	0	0	0	0	0	0
0	0	0	0	0	0	0	0	0
1	0	0	0	0	0	0	0	0
1	0	0	0	0	0	0	0	0
0	0	0	0	0	0	0	0	0
1	0	0	0	0	0	0	0	0
0	0	0	0	0	0	0	0	0
1	0	0	0	0	0	0	0	0
0	0	0	0	0	0	0	0	0
1	0	0	0	0	0	0	0	0
0	0	0	0	0	0	0	0	0
1	0	0	0	1	0	0	0	0
1	0	0	0	0	0	0	0	0
0	0	1	1	0	0	0	0	0
0	0	0	0	0	0	0	0	0
0	0	0	0	0	0	0	0	0
0	0	0	0	0	0	0	1	0
0	0	0	0	0	0	0	0	0
0	0	0	0	0	0	0	0	0
0	0	0	0	0	0	0	0	0
1	0	0	0	0	0	0	0	0
0	0	0	0	0	0	0	0	0
0	0	0	0	0	0	0	0	0
0	0	0	0	0	0	0	0	0
0	0	0	1	0	0	0	0	0
1	0	0	0	0	0	0	0	0
1	0	0	0	0	0	0	1	0
1	0	0	0	0	0	0	0	0
0	0	0	0	0	0	0	0	0
1	0	0	0	0	0	0	0	0
0	0	0	0	0	0	0	0	0
0	0	0	0	0	0	0	0	0
0	0	0	0	0	0	0	0	0
0	0	0	0	0	0	0	0	0
0	0	0	0	0	0	0	0	0
1	0	0	0	0	0	0	0	0
1	0	0	0	1	0	0	0	0
1	0	0	0	0	0	0	0	0
1	0	0	0	0	0	0	0	0
1	0	0	0	0	0	0	0	0
0	0	0	0	0	0	0	0	0
0	0	0	0	0	0	0	0	0
0	0	0	0	0	0	0	0	0
0	0	0	0	0	0	0	0	0
1	0	0	0	0	0	0	0	0
0	0	0	0	0	0	0	0	1
0	0	0	0	0	0	0	0	0
1	0	0	0	0	0	0	1	0
1	0	0	0	0	0	0	0	0
0	0	0	0	0	0	0	0	0
0	0	0	0	0	0	0	0	0
0	0	0	0	0	0	0	0	0
1	0	0	0	0	0	0	1	0
1	0	0	0	0	0	0	0	0
1	0	0	0	0	0	0	1	0

0	0	0	0	0	0	0	0	0
0	0	0	0	0	0	0	0	0
0	0	0	0	0	0	0	0	0
0	0	0	0	0	0	0	0	0
0	0	0	0	0	0	0	1	0
1	0	0	0	0	0	0	0	0
0	0	0	0	0	0	0	1	0
0	0	0	0	0	0	0	0	0
1	0	0	0	0	0	0	0	0
0	0	0	0	0	0	0	0	0
1	0	0	0	1	0	0	0	0
1	0	0	0	0	0	0	0	0
0	0	0	0	0	0	0	0	0
1	0	0	0	0	0	0	0	0
1	0	0	0	0	0	0	0	0
0	0	0	0	0	0	0	0	0
0	0	0	0	0	0	0	0	0
0	0	0	0	0	0	0	1	0
1	0	0	0	0	0	0	0	0
0	0	0	0	0	0	0	0	0
0	0	0	0	0	0	0	0	0
1	0	0	0	0	0	0	0	0
0	0	0	0	0	0	0	0	0
0	0	0	0	0	0	0	0	0
1	0	0	0	0	0	1	0	0
0	0	0	0	0	0	0	0	0
0	0	0	0	0	0	0	0	0
1	0	0	0	0	0	0	0	1
1	0	0	0	0	0	0	0	0
0	0	0	0	0	0	0	0	0
0	0	0	0	0	0	0	0	0
0	0	0	0	0	0	0	0	0
0	0	0	0	0	0	0	0	0
0	0	0	0	0	0	0	1	0
1	0	0	0	0	0	0	0	0
0	0	0	0	0	0	0	0	0
1	0	0	0	0	0	0	0	0
0	0	0	0	0	0	0	0	0
0	0	0	0	0	0	0	1	0
0	0	0	0	0	0	0	0	0
0	0	0	0	0	0	0	0	0
0	0	0	0	0	0	0	0	0
0	0	0	0	0	0	0	0	0
0	0	0	0	0	0	0	0	0
0	0	0	0	0	0	0	0	0
1	0	0	0	0	0	0	0	0
0	0	0	0	0	0	0	0	0
0	0	0	1	0	0	0	0	0
0	0	0	0	0	0	0	0	0
0	0	0	0	1	0	0	0	0
0	0	0	0	0	0	0	0	0
0	0	0	0	0	0	0	0	0
0	0	0	0	0	0	0	0	0
0	0	0	0	0	0	0	1	0
1	0	0	0	0	0	0	1	0
0	0	0	0	0	0	0	0	0

0	0	0	0	0	0	0	0	0
1	0	0	0	1	0	0	0	0
0	0	0	0	0	0	0	1	0
1	0	0	0	0	0	0	0	0
1	0	0	0	0	0	0	0	0
0	0	0	0	0	0	0	0	0
0	0	0	0	0	0	0	0	0
1	0	0	0	0	0	0	0	0
1	0	0	0	0	0	0	1	0
0	0	0	0	0	0	0	0	0
1	0	0	0	0	0	0	0	0
0	0	0	0	0	0	0	0	0
0	0	0	0	0	0	0	0	0
1	1	0	0	0	0	0	0	0
0	0	0	0	0	0	0	0	0
0	0	0	0	0	0	0	0	0
1	0	0	0	0	0	0	0	0
0	1	0	0	0	0	0	0	0
0	0	0	0	0	0	0	0	0
0	0	0	0	0	0	0	0	0
0	0	0	0	0	0	1	0	0
0	0	0	0	0	0	0	0	0
0	0	0	0	0	0	0	0	0
0	0	0	0	0	0	0	0	0
0	0	0	0	0	0	0	0	0
0	0	0	1	0	0	0	1	0
0	0	0	0	0	0	0	0	0
0	0	0	0	0	0	0	0	0
0	0	1	0	0	0	0	0	0
0	0	0	0	0	0	0	0	0
0	0	0	0	0	0	0	0	0
0	0	0	0	0	0	1	0	0
0	0	0	0	0	0	0	0	0
1	0	0	0	0	0	0	0	0
0	0	0	0	0	0	0	0	0
0	0	0	1	0	0	0	0	0
0	1	0	0	0	0	0	1	0
0	0	0	0	0	0	0	0	0
1	0	0	0	0	0	0	0	0
0	0	0	0	0	0	0	0	0
0	0	0	0	0	0	0	0	0
0	0	0	0	0	0	0	0	0
0	0	0	1	0	0	0	0	0
1	0	0	0	0	0	0	0	0
0	0	1	0	0	0	0	1	0
0	0	0	0	0	0	0	0	0
0	0	0	1	0	0	0	0	0
0	0	0	0	0	0	0	1	0
1	0	0	0	0	0	0	0	0
0	0	0	0	0	0	0	0	0
0	0	0	1	0	0	0	0	0
0	0	0	0	0	0	0	0	0
1	0	0	0	0	0	0	1	0
0	0	0	0	0	0	0	1	0
0	0	0	0	0	0	0	0	0
0	0	0	0	0	0	0	0	0

0	0	0	1	0	0	0	0	0
0	0	0	0	0	0	0	0	0
0	1	0	0	0	0	0	0	0
0	0	0	0	0	0	0	0	0
0	0	0	0	0	0	0	0	0
1	0	0	0	0	0	0	0	0
0	0	0	0	0	0	0	0	0
1	0	0	0	0	0	0	0	0
1	0	0	0	0	0	0	0	0
0	0	0	0	0	0	0	0	0
1	0	0	0	0	0	0	0	0
0	0	0	0	0	0	0	0	0
1	0	0	0	0	0	0	0	0
1	0	0	0	0	0	0	1	0
0	0	0	0	0	0	0	1	0
0	0	0	0	0	0	0	1	0
0	0	0	0	0	0	0	0	0
0	0	0	0	0	0	0	0	0
0	0	0	0	0	0	0	0	0
0	0	0	0	0	0	0	1	0
0	0	0	0	1	0	0	0	0
1	0	0	0	0	0	0	0	0
0	0	0	0	0	0	0	0	0
0	0	1	0	0	0	0	0	0
0	0	0	0	0	0	0	0	0
0	0	0	0	0	0	0	0	0
0	0	0	0	0	0	0	0	0
0	1	1	0	1	0	0	1	0
0	0	1	0	0	0	0	0	0
0	0	0	0	0	0	0	0	0
1	0	0	0	0	0	0	0	1
0	0	0	0	0	0	0	0	0
1	0	0	0	0	0	0	0	0
0	0	0	0	0	0	0	1	0
0	0	0	0	0	0	0	0	0
0	0	0	0	0	0	0	0	0
0	0	0	0	0	0	0	0	0
0	0	0	0	1	0	0	0	0
1	0	0	0	1	0	0	0	0
0	0	0	0	0	0	0	0	0
0	0	0	0	0	0	0	0	0
0	0	0	0	0	0	0	1	0
0	0	0	0	0	0	0	0	0
0	0	0	0	0	0	0	0	0
1	0	0	0	0	0	0	0	0
0	0	0	0	0	0	0	0	0
0	0	0	0	0	0	0	0	0
1	0	0	0	0	0	0	0	0
0	0	0	0	0	0	0	0	0
0	0	0	0	0	0	0	0	0
0	0	0	0	0	0	0	0	0
1	0	0	0	0	0	0	0	0
0	0	0	0	0	0	1	0	0
0	0	0	0	0	0	0	0	0
1	0	0	0	0	0	0	0	0
0	0	0	0	1	0	0	0	0

1	0	0	0	0	0	0	0	0
0	0	0	0	0	0	0	0	0
1	0	0	0	0	0	0	0	0
0	0	0	0	0	0	0	0	0
0	0	0	0	0	0	0	0	0
0	0	0	0	0	0	0	1	0
0	0	0	0	0	0	0	1	0
0	0	0	0	0	0	0	0	0
0	0	0	0	0	0	0	0	0
0	0	0	0	0	0	0	0	0
0	0	0	0	1	0	0	0	0
0	0	0	0	1	0	0	0	0
0	0	0	0	0	0	0	0	0
0	0	0	0	0	0	0	0	0
0	0	0	0	0	0	0	0	0
0	0	0	0	0	0	0	0	0
0	0	0	0	0	0	0	0	0
0	0	0	0	0	0	0	0	0
0	0	0	0	0	0	0	0	0
0	0	0	0	0	0	0	1	0
0	0	0	0	0	0	0	1	0
0	0	0	0	0	0	0	0	0
0	0	0	0	0	0	0	0	0
0	0	0	1	0	0	0	0	0
1	0	0	0	0	0	0	0	0
0	0	0	0	0	0	0	0	1
1	0	0	0	0	0	0	0	0
0	0	0	0	0	0	0	0	0
1	0	1	0	0	0	0	0	0
0	0	0	1	0	0	0	0	0
0	0	0	1	0	0	0	0	0
0	0	0	0	0	0	0	0	0
0	0	0	0	0	0	0	0	0
0	0	0	0	0	0	0	0	0
0	0	0	0	0	0	0	0	0
1	0	0	0	0	0	0	0	0
1	0	0	0	0	0	0	0	0
0	0	0	0	0	0	0	0	0
0	0	0	0	0	0	0	0	0
0	0	0	0	0	0	0	0	0
0	0	0	0	0	0	0	0	0
0	0	0	0	0	0	0	0	0
1	0	0	0	0	0	0	0	0
1	0	0	0	0	0	0	1	0
0	0	0	0	0	0	0	0	0
0	0	0	0	0	0	0	0	0
0	0	0	0	0	0	0	0	0
0	0	0	0	0	0	0	0	0
0	0	0	0	0	0	0	0	0
0	0	0	0	0	0	0	0	0
1	0	0	0	0	0	0	0	0
0	1	0	0	0	0	0	0	0
0	0	0	0	0	0	0	0	0
1	0	0	0	0	0	0	0	0
0	0	0	0	0	0	0	0	0
0	1	0	0	0	0	0	0	0

0	0	0	0	0	0	0	0	0
0	0	0	0	0	0	0	0	0
0	0	0	0	0	0	0	0	0
0	0	0	0	0	0	0	1	0
0	0	1	0	0	0	0	0	0
0	0	0	0	0	0	0	0	0
0	0	0	0	0	0	0	0	0
1	0	0	0	0	0	0	0	0
0	1	0	0	0	0	0	0	0
0	1	0	0	0	0	0	1	0
0	0	0	0	0	0	0	0	0
1	0	0	0	1	0	0	0	0
0	0	0	0	0	0	0	1	0
1	0	0	0	0	0	0	0	0
0	0	0	0	0	0	0	1	0
0	0	0	0	0	0	0	1	0
1	0	0	0	0	0	0	0	0
0	0	0	0	0	0	0	0	0
1	0	0	0	0	0	0	0	0
1	0	0	0	1	0	0	0	0
0	0	0	0	0	0	0	0	0
0	0	0	0	0	0	0	0	0
0	1	0	0	0	0	0	1	0
1	0	0	0	0	0	0	0	0
0	0	0	0	1	0	0	0	0
0	0	0	0	0	0	0	0	0
0	0	0	0	0	0	0	0	1
1	0	0	0	0	0	0	0	0
1	0	0	0	0	0	0	0	0
0	0	0	0	0	0	0	0	0
0	0	0	0	0	0	0	0	0
0	0	0	0	0	0	0	1	0
1	0	0	0	0	0	0	0	0
0	0	0	0	0	0	0	0	0
0	0	0	0	0	0	0	0	0
0	0	0	0	0	0	0	0	0
1	0	0	0	1	0	0	0	0
1	0	0	0	0	0	0	0	0
0	0	0	0	0	0	1	0	0
0	0	0	0	0	0	0	0	0
0	1	0	0	0	0	0	0	0
0	0	0	0	0	0	0	0	0
0	0	1	0	0	0	0	1	0
0	1	0	0	0	0	0	0	0
1	0	0	0	0	0	0	1	0
0	0	0	0	1	0	0	0	0
0	0	0	0	0	0	0	0	0
1	0	0	0	0	0	0	0	0
0	0	0	0	0	0	0	0	0
0	0	0	0	0	0	0	0	0
1	0	0	0	0	0	0	0	0
0	0	0	0	0	0	1	0	0
0	0	0	0	0	0	0	0	0
0	0	0	0	0	0	0	0	0
1	0	0	0	0	0	0	0	0
0	0	0	0	1	0	0	0	0

0	0	0	0	1	1	0	0	0
0	0	0	0	0	0	0	0	0
1	0	0	0	0	0	0	0	0
1	0	0	0	0	0	0	0	0
0	0	0	0	0	0	0	0	0
0	0	0	0	0	0	0	1	0
0	0	0	0	0	0	0	0	0
1	0	0	0	0	0	0	0	0
1	0	0	0	0	0	0	0	0
0	1	0	0	0	0	0	0	0
0	0	0	0	0	0	0	0	0
0	0	0	0	0	0	0	0	0
0	0	1	0	0	0	0	0	0
0	0	0	0	0	0	0	0	0
0	0	0	0	0	0	0	0	0
0	0	0	0	0	0	0	0	0
1	0	0	0	0	0	0	0	0
0	0	0	0	0	0	0	0	0
0	0	0	0	0	0	0	0	0
1	0	0	0	0	0	0	0	0
0	0	0	0	0	0	0	0	0
0	0	0	0	0	0	0	0	0
0	1	1	0	0	0	0	1	0
0	0	0	0	0	0	0	0	0
0	0	0	0	0	0	0	0	0
0	0	0	0	0	0	0	0	0
1	0	0	0	0	0	0	0	0
0	0	0	0	0	0	0	0	0
1	0	0	0	0	0	0	0	0
0	0	0	0	0	0	0	1	0
0	0	0	0	0	0	0	0	0
0	0	0	0	0	0	0	0	0
1	0	0	0	0	0	0	0	0
0	0	0	0	0	0	0	0	0
0	0	0	0	0	0	0	0	0
1	0	0	0	0	0	0	0	0
0	0	0	0	0	0	0	1	0
1	0	0	0	0	0	0	0	0
0	1	1	0	0	0	0	1	0
0	0	1	0	0	0	0	0	0
1	0	0	0	0	0	0	0	0
1	0	0	0	1	0	0	0	0
1	0	0	0	0	0	0	0	0
1	0	0	0	0	0	0	0	0
0	0	1	0	0	0	0	0	0
1	0	0	0	0	0	0	1	0
0	0	0	0	0	0	0	0	0
0	0	0	0	0	0	1	0	0
0	0	0	0	0	0	0	0	0
0	0	0	0	0	0	0	0	0
1	0	0	0	0	0	0	0	0
0	0	0	0	0	0	0	0	0
0	0	0	0	0	0	0	0	0
0	0	0	0	0	0	0	0	0
0	0	0	0	0	0	0	0	0
0	0	0	0	0	0	0	1	0

0	0	0	0	0	0	0	0	0
1	0	0	0	0	0	0	0	0
0	0	0	0	0	0	0	0	0
1	0	0	0	0	0	0	0	0
1	0	1	0	1	0	0	0	0
0	0	0	0	0	0	0	0	0
0	0	0	1	0	0	0	0	0
1	0	1	0	0	0	0	0	0
0	0	0	0	0	0	0	0	0
1	0	0	0	1	0	0	0	0
0	0	0	0	0	0	0	0	0
0	0	0	0	0	0	0	0	0
1	0	0	0	0	0	0	0	0
0	0	0	0	0	0	0	0	0
0	0	0	0	0	0	1	0	0
0	0	0	0	0	0	0	0	0
0	0	0	0	0	0	0	1	0
0	0	0	0	0	0	0	0	0
0	0	0	0	0	0	0	0	0
0	0	0	0	0	0	0	0	0
0	0	0	0	0	0	0	0	0
0	0	0	0	0	0	0	1	0
0	0	0	0	0	0	0	0	0
0	0	0	0	0	0	0	0	0
0	0	0	0	0	0	0	0	0
0	0	0	0	0	0	0	0	0
0	0	0	0	0	0	1	0	0
0	0	0	0	0	0	0	0	0
1	0	0	0	0	0	0	0	0
0	0	0	0	0	0	0	0	0
0	0	0	0	0	0	0	0	0
0	0	0	0	0	0	0	0	0
0	0	0	0	0	0	0	0	0
0	0	0	0	0	0	0	0	0
1	0	0	0	0	0	1	0	0
0	1	0	0	0	0	0	1	0
0	0	0	0	0	0	0	0	0
1	0	0	0	1	0	0	0	0
0	0	0	0	0	0	0	0	0
0	0	0	0	0	0	0	0	0
0	0	0	0	0	0	0	0	0
0	0	0	0	0	0	0	0	0
0	0	0	0	0	0	0	0	0
1	0	0	0	0	0	0	0	1
1	0	0	0	0	0	0	0	0
0	0	0	0	1	0	0	0	0
0	0	0	0	0	0	0	0	0
0	0	0	0	0	0	0	0	0
0	0	0	0	0	0	0	0	0
1	0	0	0	0	0	0	0	0
1	0	0	0	0	0	0	0	0
0	0	0	0	0	0	0	0	0
1	0	0	0	0	0	0	0	0
0	0	0	0	0	0	0	0	0
0	0	0	0	0	0	1	0	0
0	0	0	0	0	0	0	0	0

0	0	0	0	0	0	0	0	0
1	0	0	0	0	0	0	0	0
0	0	0	0	0	0	0	0	0
0	0	0	0	0	0	0	0	0
0	0	0	0	0	0	0	0	0
0	0	0	0	0	0	0	1	0
0	0	0	0	0	0	0	0	0
0	0	0	0	0	0	0	0	0
0	0	0	0	0	0	0	0	0
0	0	1	0	0	0	0	0	0
0	0	0	0	0	0	0	0	0
1	0	0	0	0	0	0	0	0
1	0	0	0	0	0	0	0	0
1	0	0	0	0	0	0	0	0
1	0	0	0	0	0	0	0	0
0	0	0	0	0	0	0	0	0
1	0	0	0	0	0	0	1	0
0	0	0	0	0	0	0	0	0
1	0	1	0	0	0	0	0	0
0	0	0	0	0	0	0	1	0
0	0	0	0	0	0	0	0	0
1	0	0	0	0	0	0	0	0
0	0	0	0	0	0	0	0	0
0	0	0	0	0	0	1	0	0
1	0	0	0	1	0	0	0	0
1	0	0	0	0	0	0	0	0
0	0	0	1	0	0	0	0	0
0	1	0	0	0	0	0	0	0
0	0	0	0	0	0	0	0	0
1	0	0	0	0	0	0	0	0
1	0	0	0	1	0	0	0	0
0	0	0	0	0	0	0	0	0
0	0	1	0	0	0	0	0	0
0	0	0	0	0	0	0	0	0
0	0	0	0	0	0	1	0	0
0	0	0	0	1	0	0	0	0
0	0	0	0	0	0	0	0	0
0	0	0	0	0	0	0	0	0
0	0	0	0	0	0	0	0	0
0	0	0	0	0	0	0	0	0
1	0	0	0	0	0	0	0	0
1	0	0	0	0	0	0	0	0
0	0	0	0	0	0	0	0	0
0	0	0	0	1	0	1	0	0
0	1	0	0	0	0	1	0	0
0	0	0	0	1	0	0	0	0
0	0	0	0	0	0	0	1	0
0	0	0	0	0	0	0	0	0
1	0	0	0	0	0	0	0	0
1	0	0	0	0	0	0	0	0
0	0	0	0	0	0	0	0	0
0	0	0	0	0	0	0	0	0
0	0	0	0	0	0	0	0	0
1	0	0	0	0	0	0	0	0
0	0	0	0	0	0	0	0	0
0	0	0	0	0	0	0	0	0

0	0	0	1	0	0	0	0	0
0	0	0	0	0	0	0	0	0
1	0	0	0	0	0	0	0	0
1	0	0	0	0	0	1	1	0
0	0	0	0	0	0	0	0	0
0	0	0	0	0	0	0	0	0
0	0	0	0	0	0	0	0	0
1	0	0	0	0	0	0	0	0
0	0	0	0	0	0	0	0	0
0	0	0	0	0	0	0	0	0
0	0	0	1	0	0	0	0	0
0	0	0	0	0	0	0	0	0
0	0	0	0	0	0	0	0	0
0	0	0	0	0	0	0	0	0
1	0	0	0	0	0	0	0	0
0	0	0	0	0	0	0	0	0
0	0	1	0	1	0	0	0	0
1	0	0	0	0	0	0	0	0
0	0	0	1	0	0	0	0	0
1	0	0	0	1	1	0	0	0
0	0	0	0	0	0	0	0	0
0	0	0	0	1	0	0	0	0
1	0	1	0	0	0	0	0	0
0	0	0	0	0	0	0	0	0
0	0	0	0	0	0	0	0	0
0	0	0	1	0	0	0	0	0
0	0	0	0	0	0	0	0	0
1	0	0	0	0	0	0	0	0
0	0	0	0	0	0	0	0	0
0	0	0	0	0	0	0	0	0
0	0	0	0	0	0	0	0	0
0	0	1	0	0	0	0	0	0
0	0	0	0	0	0	0	0	0
1	0	0	0	0	0	0	0	0
0	0	0	0	0	0	0	1	0
0	0	1	0	0	0	0	0	0
0	0	0	0	0	0	0	0	0
1	0	0	0	0	0	0	0	0
0	0	0	0	0	0	0	0	0
0	1	0	0	0	0	0	0	1
1	0	0	0	0	0	0	0	0
0	0	0	0	0	0	1	0	0
0	0	0	0	0	0	0	0	0
0	0	0	0	0	0	0	0	0
1	0	0	0	0	0	0	0	0
0	0	0	0	0	0	0	0	0
0	0	0	0	0	0	0	0	0
1	0	0	0	0	0	0	0	0
0	0	0	0	0	0	0	1	0
0	0	0	0	0	0	0	0	0
0	0	0	0	0	0	0	0	0
1	0	0	0	0	0	0	0	0
0	0	0	0	0	0	0	0	0
0	1	0	0	0	0	0	0	0
0	0	0	0	0	0	0	0	0
0	0	0	0	0	0	0	0	0

0	1	0	0	0	0	0	0	0
1	0	0	0	0	0	0	0	0
1	0	0	0	1	0	0	0	0
0	0	0	0	1	0	0	0	0
0	0	0	0	0	0	0	0	0
0	1	0	0	0	0	0	0	0
0	0	0	1	0	0	0	0	0
1	0	0	0	0	0	0	0	0
0	0	0	0	0	0	0	0	0
1	0	0	0	0	0	0	0	0
0	0	0	0	0	0	0	0	0
0	0	0	0	0	0	0	1	0
0	0	0	0	0	0	0	0	0
1	0	0	0	0	0	0	0	0
1	0	0	0	0	0	0	0	0
0	0	0	0	0	0	0	0	0
0	0	0	0	0	0	0	0	0
0	0	0	0	0	0	0	1	0
1	0	0	0	0	0	0	0	0
1	0	0	0	0	0	0	0	0
1	0	1	0	0	0	0	0	0
0	0	0	0	0	0	1	0	0
0	0	0	0	0	0	0	0	0
0	0	0	0	0	0	0	0	0
0	0	0	0	0	0	0	0	0
0	0	0	0	0	0	0	0	0
1	0	0	0	0	0	0	0	0
0	0	0	0	0	0	0	0	0
0	0	0	0	0	0	0	0	0
0	0	0	0	0	0	0	1	0
0	0	0	0	0	0	0	0	0
1	0	0	0	0	0	0	0	0
0	0	0	0	0	0	0	0	0
0	0	0	0	0	0	0	0	0
1	0	0	0	0	0	0	0	0
0	0	0	0	1	0	0	0	0
0	1	0	0	0	0	0	0	0
0	0	0	0	0	0	0	0	0
0	0	0	0	0	0	0	0	0
0	0	0	0	0	0	0	0	0
0	0	0	0	0	0	0	0	0
0	0	0	0	0	0	0	0	0
1	0	0	0	0	0	0	0	0
0	0	0	0	0	0	0	0	0
1	0	0	0	0	0	0	0	0
0	1	0	0	0	0	0	0	0
1	0	0	0	0	0	0	0	0
0	0	0	0	0	0	0	0	0
1	0	0	0	0	0	0	0	0
0	0	0	0	0	0	0	0	0
1	0	0	0	0	0	1	1	0
0	0	0	0	0	0	0	0	0
0	0	0	0	0	0	0	0	0
1	0	0	0	0	0	0	0	0
0	0	0	0	1	0	0	0	0
0	0	0	0	0	0	0	1	0

0	0	0	0	0	0	0	0	0
1	0	0	0	0	0	0	0	0
1	0	0	0	0	0	0	0	0
0	0	0	0	0	0	0	0	0
1	0	0	0	0	0	0	1	0
0	0	0	0	0	0	0	0	0
0	0	0	0	0	0	0	1	0
0	0	0	0	0	0	0	0	0
1	0	0	0	0	0	0	0	0
0	0	0	0	0	0	0	1	0
0	0	0	0	0	0	0	0	0
0	0	0	0	0	0	0	0	0
0	0	0	0	0	0	0	0	0
0	0	0	0	0	0	0	0	0
0	0	0	0	0	0	0	0	0
1	0	0	0	0	0	0	0	0
0	0	0	0	0	0	0	0	0
0	0	0	0	0	0	0	0	0
0	0	0	0	0	0	0	0	0
0	0	0	0	0	0	0	0	0
0	0	0	0	0	0	0	0	0
0	0	0	0	0	0	0	0	0
1	0	0	0	0	0	0	0	0
0	0	0	0	0	1	0	1	0
0	0	1	0	0	0	0	0	0
0	0	0	0	0	0	0	1	0
1	0	0	0	0	0	0	0	0
0	0	0	0	0	0	0	0	0
0	0	0	0	0	0	0	0	0
0	0	0	0	0	0	0	0	0
0	0	0	0	0	0	0	0	0
0	0	0	0	0	0	0	0	0
1	0	0	0	0	0	0	0	0
0	0	0	0	1	0	0	0	0
0	0	0	0	0	0	1	0	0
0	0	0	0	0	0	0	0	0
0	0	0	0	0	0	0	0	0
1	0	0	0	0	0	0	0	0
0	1	0	0	0	0	0	0	0
1	0	0	0	0	0	0	0	0
0	0	0	0	0	0	0	0	0
0	0	0	0	0	0	0	1	0
1	0	0	0	0	0	0	0	0
0	0	0	0	1	0	0	0	0
0	0	0	0	0	0	0	0	0
0	0	0	0	0	0	0	0	0
0	0	0	0	0	0	0	0	0
1	0	0	0	0	0	0	0	0
0	0	0	0	0	0	0	0	0
0	0	0	0	0	0	0	0	0
1	0	0	0	0	0	0	0	0
0	0	0	0	0	0	0	0	0
0	0	0	0	0	0	0	0	0
0	0	0	0	0	0	0	1	0
0	0	0	0	0	0	0	0	0
1	0	0	0	1	0	0	0	0

0	0	0	0	0	0	0	0	0
1	0	0	0	1	0	0	0	0
0	0	0	0	0	0	1	1	0
1	0	0	0	0	0	0	0	0
0	0	0	0	0	0	0	0	0
0	0	0	0	0	0	0	0	0
0	0	0	0	0	0	0	0	0
0	0	0	0	0	0	0	0	0
1	0	0	0	0	0	0	0	0
0	0	0	0	0	0	0	0	0
0	0	0	0	0	0	0	0	0
1	0	0	0	0	0	0	1	0
0	0	0	0	0	0	0	0	0
0	0	0	0	0	0	0	0	0
0	0	0	0	0	0	0	0	0
0	0	0	0	0	0	0	0	0
1	0	0	0	0	0	0	0	0
0	0	0	0	0	0	0	0	0
0	0	0	1	0	0	0	1	0
0	0	0	0	1	0	0	0	0
1	0	0	0	0	0	0	0	0
1	0	0	0	0	0	0	0	0
1	0	0	0	0	0	0	1	0
0	0	0	0	0	0	0	0	0
0	0	0	0	0	0	0	0	0
0	0	0	0	0	0	0	0	0
0	0	0	0	0	0	0	1	1
0	0	0	0	0	0	0	0	0
0	0	0	0	0	0	1	0	0
0	0	0	0	0	0	0	0	0
0	0	0	0	0	0	0	0	0
0	0	0	0	0	0	0	0	0
0	0	0	0	0	0	0	0	0
0	0	0	0	0	0	0	0	0
0	0	0	0	0	0	0	1	0
1	0	0	0	0	0	0	0	0
1	0	0	0	1	0	0	0	0
0	0	0	0	0	0	0	0	0
0	0	0	0	0	0	0	0	0
1	0	0	0	0	0	0	0	0
0	0	0	0	0	0	1	0	0
0	0	0	0	0	0	0	0	0
1	0	0	0	0	0	0	0	0
1	0	0	0	1	0	0	0	0
0	0	0	0	0	0	0	0	0
1	0	0	0	0	0	0	0	0
0	0	0	0	0	0	0	0	0
0	0	0	0	0	0	0	0	0
0	0	0	0	0	0	0	0	0
0	0	0	0	0	0	0	0	0
1	0	1	0	0	0	0	0	0
0	0	0	0	0	0	0	0	0
1	0	0	0	0	0	0	0	0
0	0	0	0	0	0	0	0	0
1	0	0	0	0	0	0	0	0
0	0	0	0	0	0	0	0	0

1	0	0	0	0	0	0	0	0
1	0	0	0	0	0	0	1	0
1	0	0	0	0	0	0	1	1
0	0	0	0	0	0	0	0	0
0	0	0	0	0	0	0	0	0
0	0	0	0	0	0	0	1	0
1	0	0	0	0	0	0	0	0
0	0	0	0	0	0	0	1	0
0	0	0	0	0	0	0	0	0
0	0	0	0	0	0	1	1	0
0	0	0	0	0	0	0	0	0
0	0	0	0	0	0	0	0	0
1	0	0	0	0	0	0	0	0
1	0	0	0	0	0	0	0	0
0	0	0	0	0	0	0	0	0
1	0	0	0	0	0	0	0	0
1	0	0	0	0	0	0	1	0
1	0	0	0	0	0	0	0	0
0	1	0	0	0	0	0	1	0
1	0	0	0	0	0	0	0	0
0	0	0	1	0	0	1	0	0
1	0	1	0	0	0	0	1	0
0	0	0	0	0	0	0	1	0
0	0	0	0	0	0	0	1	0
0	0	0	0	0	0	0	0	0
0	0	0	0	1	0	0	0	0
0	0	0	0	0	0	0	0	0
0	0	0	0	0	0	0	0	0
0	0	0	0	0	0	0	0	0
0	0	0	0	0	0	0	1	0
0	0	0	0	0	0	0	0	0
1	0	0	0	0	0	0	0	0
0	1	0	0	0	0	0	0	0
1	0	0	0	0	0	0	1	0
0	0	0	0	0	0	0	0	0
0	0	0	0	0	0	0	0	0
0	0	0	0	1	0	0	0	0
1	0	0	0	0	0	0	0	0
1	0	0	0	0	0	0	0	0
0	0	0	0	0	0	0	0	0
0	0	0	0	0	0	0	0	0
1	0	1	0	0	0	0	0	0
1	0	0	0	0	0	0	0	0
1	0	0	0	0	0	0	0	0
0	0	0	0	0	0	0	0	0
0	0	0	0	0	0	0	0	0
0	0	0	0	0	0	0	0	0
1	0	0	0	0	0	0	0	0
0	0	0	0	0	0	0	0	0
1	0	0	0	0	0	0	0	0
0	0	0	0	0	0	0	0	0
0	0	0	0	0	0	0	0	0
0	0	0	0	0	0	0	0	0
0	1	0	0	0	0	0	0	0
0	0	0	0	0	0	0	1	0
0	0	0	0	0	0	0	1	0

0	0	0	0	0	0	0	0	0
0	0	0	0	0	0	0	0	0
1	0	0	0	0	0	0	0	0
0	0	0	0	0	0	0	0	0
1	1	0	0	0	0	0	0	0
0	0	0	0	0	0	0	0	0
0	0	0	0	0	0	0	1	0
1	0	0	0	0	0	0	0	0
0	0	0	1	0	0	0	0	0
0	0	0	0	0	0	0	0	0
1	0	0	0	0	0	0	0	0
0	0	0	0	0	0	0	0	0
1	0	0	0	0	0	0	0	0
1	1	0	0	0	0	0	0	0
0	0	0	0	0	0	0	0	0
0	0	0	0	0	0	0	0	0
0	0	0	0	0	0	0	0	0
0	0	0	0	0	0	0	0	0
0	0	0	0	0	0	0	0	0
1	0	0	0	0	0	0	0	0
0	0	0	0	0	0	0	0	0
0	0	0	0	0	0	0	0	0
0	0	0	0	0	0	0	0	0
1	0	0	0	1	0	0	1	0
0	0	0	0	0	0	0	0	0
0	0	0	0	1	0	0	0	0
1	0	0	0	0	0	0	0	0
0	0	0	0	0	0	1	0	0
0	0	1	0	0	0	0	0	0
0	0	0	0	0	0	0	0	0
0	0	0	0	0	0	0	0	0
1	0	1	0	0	0	0	0	0
0	0	0	0	0	0	0	1	0
0	0	0	0	0	0	0	0	0
0	0	0	0	0	0	0	0	0
1	0	0	0	0	0	0	0	0
1	0	0	0	0	0	0	0	0
0	0	0	0	0	0	0	0	0
0	0	0	0	0	0	0	0	1
0	0	1	0	0	0	0	0	0
0	0	0	0	0	0	0	0	0
1	0	1	0	1	0	0	0	0
0	0	0	0	0	0	0	0	0
1	0	0	0	0	0	0	0	0
0	0	0	0	0	0	0	1	0
1	0	0	0	0	0	0	0	0
0	0	0	0	0	0	0	0	0
0	0	0	0	0	1	0	0	0
0	0	0	0	0	0	0	0	0
0	0	0	1	0	0	0	0	0
0	0	0	0	0	0	0	0	0
0	0	0	0	0	0	0	0	0
0	0	0	0	0	0	0	0	0
0	0	0	0	0	0	0	0	0
0	0	0	0	0	0	0	1	0
1	0	0	0	1	0	0	0	0

0	0	0	0	0	0	0	0	0
0	0	0	0	0	0	0	0	0
0	0	0	0	0	0	0	0	1
1	0	0	0	0	0	0	0	0
0	0	0	0	0	0	0	0	0
0	0	0	0	0	0	0	0	0
0	0	0	0	0	0	0	1	0
1	0	0	0	0	0	0	0	0
0	0	0	0	0	0	0	0	0
0	0	0	0	0	0	0	0	0
1	0	0	0	0	0	0	0	0
0	0	0	0	0	0	0	0	0
0	0	0	0	0	0	0	0	0
1	0	0	0	0	0	0	0	0
0	0	0	0	0	0	0	0	0
0	0	0	0	0	0	0	1	0
1	1	0	0	1	0	0	1	0
0	0	0	0	0	0	0	0	0
1	0	0	0	0	0	0	0	0
1	0	0	0	0	0	0	0	0
0	0	0	0	1	0	0	0	0
1	0	0	0	0	0	0	0	0
1	0	0	0	0	0	0	0	0
0	0	0	0	0	0	0	0	0
0	0	0	0	0	0	0	1	0
0	1	0	0	0	0	0	0	0
0	0	0	0	0	0	0	1	0
0	0	0	0	0	0	0	0	0
1	0	0	0	0	0	0	1	0
0	0	0	0	0	0	0	1	0
1	0	0	0	0	0	0	0	0
0	0	1	0	0	0	0	1	0
0	0	0	0	0	0	0	0	0
0	0	0	0	0	0	0	0	0
0	0	0	0	0	0	0	0	0
0	0	0	0	0	0	0	0	0
0	1	0	0	0	1	0	0	0
0	0	0	0	0	0	0	1	0
0	0	0	0	0	0	0	0	0
0	0	0	0	0	0	0	0	0
0	1	0	0	0	0	0	1	0
1	0	0	0	0	0	0	1	0
1	0	0	0	0	0	0	0	0
0	1	0	0	0	0	0	0	0
0	0	0	0	0	0	0	0	0
0	0	0	0	0	0	0	0	0
0	0	0	0	0	0	0	0	0
0	0	0	0	0	0	0	1	0
1	0	0	0	0	0	0	0	0
1	0	0	0	0	0	0	0	0
0	0	0	0	0	0	0	0	0
0	0	0	0	0	0	0	0	0
1	0	0	0	0	0	0	0	0
0	0	0	0	0	0	0	0	0
0	0	0	0	0	0	0	0	0
0	0	0	0	0	0	0	0	0

0	0	0	0	0	0	0	0	0
0	1	1	0	0	0	0	0	0
0	0	0	0	0	0	1	0	0
0	0	0	0	0	0	0	0	0
1	0	0	0	0	0	0	1	0
0	0	0	0	0	0	0	0	0
0	0	0	0	0	0	0	0	0
0	0	0	0	0	0	0	0	0
0	0	0	0	0	0	0	0	0
0	0	0	0	0	0	0	0	0
0	0	0	0	0	0	0	0	0
0	0	0	0	0	0	0	0	0
0	0	0	0	0	0	0	1	0
0	0	0	0	0	0	0	0	0
0	0	0	0	0	0	0	0	0
0	0	0	0	0	0	0	0	0
1	0	0	0	0	0	0	0	0
1	0	0	0	0	0	0	1	0
1	0	0	0	0	0	0	0	0
0	1	0	0	0	0	0	0	0
0	0	0	0	0	0	0	0	0
0	0	0	0	0	0	0	0	0
0	0	0	0	0	0	0	0	0
0	0	0	0	0	0	0	0	0
0	0	1	0	0	0	0	1	0
1	0	1	0	1	0	0	0	0
0	0	0	0	0	0	0	0	0
0	0	0	0	0	0	0	0	0
0	0	0	0	0	0	0	0	0
1	0	0	0	0	0	1	0	0
1	0	0	0	0	0	0	0	0
0	0	0	0	0	0	0	0	0
1	1	0	0	0	0	0	0	0
0	0	0	0	0	0	0	0	0
0	0	0	0	0	0	0	0	0
1	0	0	0	0	0	0	0	0
1	0	0	0	0	0	0	0	0
0	0	0	0	0	0	0	0	0
0	0	0	0	0	0	0	0	0
0	0	0	0	0	0	0	0	0
0	0	0	0	0	0	0	0	0
0	0	0	0	0	0	0	0	0
0	1	0	0	0	0	0	0	0
0	0	0	0	0	0	0	0	0
0	0	0	0	0	0	0	0	0
0	0	0	0	0	0	0	0	0
0	0	0	0	0	0	0	0	0
0	0	0	0	0	0	0	0	0
1	0	0	0	0	0	0	0	0
0	0	0	0	0	0	0	0	0
0	0	0	0	0	0	0	0	0
1	0	0	0	0	0	0	0	0
0	0	0	0	1	0	0	0	0
0	0	0	0	0	0	0	0	0
1	0	0	0	0	0	0	0	0
0	0	0	0	0	0	0	0	0

0	0	0	0	0	0	0	0	0
0	0	0	0	0	0	0	0	0
0	0	0	0	0	0	0	1	0
1	0	0	0	0	0	0	1	0
1	0	0	0	0	0	1	0	0
0	0	0	0	0	0	0	0	0
1	0	0	0	0	0	0	0	0
0	0	0	0	0	0	0	0	0
1	0	0	0	0	0	0	0	0
0	0	0	0	0	0	0	0	0
0	0	0	0	0	0	0	0	0
0	0	0	0	0	0	0	0	0
0	0	0	0	0	0	0	0	0
1	0	0	0	0	0	0	0	0
0	0	0	0	0	0	0	1	1
0	0	0	0	0	0	0	0	0
0	1	0	0	0	0	0	1	0
0	0	0	0	0	0	0	1	0
0	0	0	0	1	0	0	1	0
0	0	0	0	0	0	0	0	0
0	0	0	0	0	0	0	0	0
0	0	0	0	0	0	0	0	0
0	0	0	0	0	0	0	1	0
0	0	0	0	0	0	0	1	0
1	0	0	0	0	0	0	0	0
0	0	0	0	0	0	0	0	0
0	0	0	0	0	0	0	0	0
0	0	0	0	0	0	0	0	0
0	0	0	0	1	0	0	0	0
0	0	0	0	0	0	0	0	0
0	0	0	0	0	0	0	0	0
0	0	0	0	0	0	0	0	0
0	0	0	0	0	0	0	1	0
0	0	0	0	0	0	0	0	0
0	0	0	0	0	0	0	0	0
0	0	0	0	0	0	0	0	0
0	0	0	0	0	0	0	0	0
0	0	0	0	0	0	0	0	0
0	0	0	0	0	0	0	0	0
0	0	0	0	0	0	0	0	0
0	0	0	0	0	0	0	0	0
1	0	0	0	0	0	0	0	0
0	1	0	0	0	0	0	0	0
0	0	0	0	0	0	0	0	0
0	0	0	0	0	0	0	0	0
0	0	0	0	0	0	0	0	0
0	0	1	0	0	0	0	1	0
1	0	0	0	0	0	0	0	0
0	0	0	0	0	0	1	0	0
0	0	0	0	0	0	0	0	0
0	0	0	0	0	0	0	0	0
0	0	0	0	0	0	0	0	0
0	0	0	0	0	0	0	0	0
0	0	0	0	0	0	0	0	0
0	0	0	0	0	0	0	0	0
0	0	0	0	0	0	0	0	0

0	0	0	0	0	0	0	0	0
0	1	0	0	0	0	0	0	0
1	0	0	0	0	0	0	0	0
0	0	0	0	0	0	0	0	0
0	0	0	0	1	0	0	0	0
0	1	0	0	0	0	0	1	0
1	0	0	0	1	0	0	0	0
1	0	0	0	0	0	0	0	0
1	0	0	0	1	0	0	0	0
0	0	0	0	0	0	0	0	0
0	0	0	0	0	0	0	1	0
0	0	0	0	0	0	0	0	0
1	1	0	0	0	0	1	0	1
0	0	0	0	0	0	0	0	0
0	0	0	0	0	0	0	0	0
0	0	0	0	0	0	0	0	0
1	0	1	0	0	0	0	1	0
0	0	0	0	0	0	0	0	0
0	0	0	0	0	0	0	0	0
1	0	0	0	0	0	0	0	0
1	0	0	0	0	0	0	0	0
0	0	0	0	0	0	0	0	0
0	0	1	0	0	0	0	0	1
1	0	0	0	1	0	0	0	0
0	0	0	0	0	0	0	0	0
1	0	0	0	0	0	0	0	0
1	0	0	0	0	0	0	0	0
1	1	0	0	0	0	0	0	0
0	0	0	0	0	0	0	0	0
0	0	0	0	0	0	0	0	0
1	0	0	0	0	0	0	0	0
0	0	0	0	0	0	0	1	0
1	0	1	0	0	0	0	0	0
0	0	0	0	0	0	0	0	0
1	0	0	0	0	0	0	0	0
0	0	0	0	0	0	0	0	0
0	0	0	0	0	0	0	0	0
1	0	0	0	0	0	0	0	0
0	0	0	0	0	0	0	0	0
1	0	0	0	0	0	0	0	1
1	0	0	0	0	0	0	0	0
0	0	0	0	0	0	0	0	0
0	0	0	0	0	0	0	0	0
0	0	0	0	0	0	0	0	0
1	0	0	0	0	1	0	0	0
0	0	0	0	0	0	0	0	0
1	0	0	0	0	0	0	0	0
0	0	0	0	0	0	0	0	0
0	0	0	0	0	0	0	0	0
1	0	0	0	0	0	0	0	0
1	0	1	0	0	0	0	0	0
1	0	0	0	0	0	0	1	0
0	0	0	0	0	0	0	0	0
0	0	0	0	0	0	0	1	0
1	0	0	0	0	0	0	0	0
0	0	0	0	0	0	0	0	0

1	0	0	0	0	0	0	0	0
0	0	0	0	0	0	0	0	0
0	0	0	0	0	0	0	0	0
0	0	0	0	0	0	0	0	0
1	0	0	0	0	0	0	0	0
0	0	0	0	0	0	0	0	1
0	0	0	0	0	0	0	0	0
0	1	0	0	0	0	0	0	0
0	0	1	0	0	0	0	0	1
0	0	0	0	0	0	0	0	0
1	0	0	0	0	0	0	0	0
0	0	0	0	0	0	0	0	0
0	0	0	0	0	0	0	0	0
0	0	0	0	0	0	0	0	0
1	1	0	0	0	0	0	1	0
1	0	0	0	0	0	0	0	0
1	0	0	0	0	0	0	0	0
1	0	0	0	0	0	0	0	0
0	0	0	0	0	0	1	1	0
0	0	0	0	0	0	0	0	0
0	0	0	0	0	0	0	1	0
1	0	0	0	0	0	0	0	0
1	0	0	0	0	0	0	0	0
0	0	0	0	0	0	0	0	0
0	0	0	0	0	0	0	0	0
1	0	0	0	0	0	0	0	0
0	0	0	0	0	0	0	0	0
0	0	0	0	0	0	0	0	0
1	0	0	0	0	0	0	1	0
0	0	0	0	0	0	0	0	0
0	0	0	0	0	0	0	0	0
0	0	0	0	0	0	0	0	0
0	0	0	0	0	0	0	0	0
0	0	0	0	0	0	0	0	0
0	0	0	0	0	0	0	0	0
0	0	0	0	0	0	0	0	0
1	0	0	0	0	0	0	0	0
0	0	0	0	0	0	0	0	0
0	1	0	0	0	0	0	0	0
1	0	1	0	0	0	0	0	0
0	0	0	0	0	0	0	0	0
0	0	0	0	0	0	0	0	0
1	0	0	0	0	0	0	0	0
1	0	0	0	0	0	0	0	0
1	0	0	0	0	0	0	1	0
1	0	1	0	0	0	0	1	0
1	0	0	0	0	0	0	0	0
0	0	0	0	0	0	0	0	0
0	0	0	0	0	0	0	0	0
1	0	0	0	0	0	0	0	0
1	1	1	0	0	0	0	0	0
0	0	0	0	0	0	0	0	0
0	0	0	0	0	0	0	1	0
0	0	0	0	0	0	0	0	0
0	0	0	0	0	0	0	0	0
0	0	0	0	1	0	0	0	0

0	0	0	0	0	0	0	1	0
1	0	0	0	0	0	0	0	0
0	0	0	0	0	0	0	0	0
1	0	0	0	0	0	0	1	0
0	0	0	0	0	0	0	0	0
0	0	0	0	0	0	0	1	0
0	0	0	0	0	0	0	0	0
0	0	0	0	0	0	0	1	0
0	0	0	0	0	0	0	0	0
0	0	0	0	0	0	0	0	0
0	0	0	0	0	0	0	1	0
1	0	0	0	0	0	0	0	0
0	0	0	0	0	0	0	0	0
1	0	0	0	0	0	1	0	0
0	0	0	0	1	1	0	0	0
0	0	0	0	0	0	0	0	0
0	0	0	0	0	0	0	0	0
0	0	0	0	0	0	0	0	0
1	0	0	0	0	0	0	0	0
1	0	0	0	0	0	1	0	0
0	0	0	0	0	0	1	0	0
1	0	1	0	0	0	0	0	0
0	0	0	0	0	0	0	0	0
0	0	0	0	0	0	0	1	0
0	0	0	0	0	0	0	0	0
0	0	0	0	0	0	0	0	0
0	0	0	0	0	0	0	0	0
0	0	0	0	0	0	0	0	0
0	0	0	0	0	0	0	1	0
0	0	0	0	0	0	0	0	0
0	0	0	0	0	0	0	0	0
1	0	0	0	0	0	0	0	0
0	0	0	0	1	0	0	0	0
1	0	0	0	0	0	0	0	0
0	0	0	0	0	0	0	0	0
1	1	0	0	0	0	0	0	0
0	0	0	0	0	0	0	0	0
1	0	0	0	0	0	0	0	0
0	0	0	0	0	0	0	0	0
1	0	0	0	0	0	0	1	0
1	0	0	0	0	0	0	0	0
0	0	0	0	0	0	0	1	0
0	0	0	0	0	0	0	0	0
1	0	1	0	0	0	0	0	0
0	0	0	0	0	0	0	0	0
0	0	0	0	0	0	0	0	0
1	0	0	0	0	0	0	0	0
0	0	0	0	0	0	0	0	0
1	0	0	0	0	0	0	0	0
0	1	0	0	0	0	0	0	0
0	0	0	0	0	0	0	0	0
0	0	0	0	0	0	1	0	0
0	1	0	0	0	0	0	0	0
1	0	0	0	0	0	0	0	0
0	0	0	0	0	0	0	0	0
0	0	0	0	0	0	0	0	0

0	0	0	0	0	0	0	0	0
0	0	0	0	0	0	0	0	0
0	0	0	0	0	0	0	0	0
0	0	0	1	0	0	0	0	0
1	1	0	0	0	0	0	0	0
0	1	0	0	0	0	0	0	0
0	0	0	0	0	0	0	0	0
0	0	0	0	0	0	0	0	0
0	0	0	0	0	0	0	0	0
1	0	0	0	1	0	0	0	0
1	0	0	0	0	0	0	0	0
0	0	0	0	0	0	1	0	0
0	0	0	0	0	0	0	1	0
0	0	0	0	0	0	0	0	0
1	0	0	0	0	0	0	0	0
0	0	0	0	1	0	0	1	0
0	0	0	0	0	0	0	0	0
0	0	0	0	0	0	0	0	0
0	0	0	0	0	0	0	1	0
1	0	0	0	0	0	0	0	0
0	0	0	0	0	0	0	0	0
0	0	0	0	0	0	0	0	0
0	0	0	0	0	0	0	1	0
0	0	0	0	0	0	0	0	0
0	0	0	0	0	0	0	0	0
0	0	0	0	0	0	0	1	0
0	0	0	0	0	0	0	0	0
1	0	0	0	0	0	0	0	0
0	0	0	0	0	0	0	0	0
0	0	0	0	0	0	0	1	0
1	0	0	0	0	0	0	0	0
1	0	1	0	0	0	0	0	0
0	0	0	0	0	0	0	0	0
0	1	0	0	0	0	0	0	0
1	0	0	0	0	0	0	0	0
0	0	0	0	0	0	0	0	0
0	0	0	0	0	0	0	0	0
1	0	0	0	0	0	0	0	0
1	0	0	0	0	0	0	1	0
1	0	0	0	0	0	0	0	0
0	0	0	0	0	0	0	0	1
0	0	0	0	0	0	0	0	0
0	0	0	0	0	0	0	0	0
0	0	0	0	0	0	1	0	0
0	0	0	0	0	0	0	0	0
1	0	0	0	1	0	0	0	0
0	0	0	0	0	0	0	1	0
0	0	0	0	0	0	0	0	0
0	0	0	0	0	0	0	1	0
0	0	0	0	0	0	0	0	0
1	0	0	0	0	0	0	0	0
0	0	0	0	0	0	0	0	0
1	0	0	0	0	0	0	0	0
1	0	0	0	0	0	0	0	0
0	0	0	0	0	0	0	0	0
0	0	0	0	0	0	0	0	0

0	0	0	0	0	0	0	0	0
0	0	0	0	0	0	0	0	0
0	0	1	0	0	0	0	1	0
0	0	0	0	0	0	0	0	0
0	0	0	0	0	0	0	0	0
0	0	0	0	0	0	0	0	0
0	0	0	0	0	0	0	0	0
0	0	0	0	1	0	0	0	0
0	0	0	0	0	0	0	0	0
0	0	0	0	0	0	0	0	0
1	0	0	0	1	0	0	0	0
1	0	0	0	1	0	0	0	0
1	0	0	0	0	0	1	0	0
0	0	0	0	0	0	0	0	0
0	0	0	0	0	0	0	1	0
0	0	0	0	0	0	0	0	0
0	0	1	0	0	0	0	0	0
0	0	0	0	0	0	0	0	0
0	0	0	0	0	0	0	0	0
0	0	0	0	0	0	0	0	0
0	0	0	0	0	0	0	0	0
0	0	0	0	0	0	0	0	0
1	0	0	0	0	0	0	0	0
1	0	0	0	0	0	0	0	0
0	0	1	0	0	0	0	0	0
0	0	0	0	0	0	0	0	0
1	0	0	0	0	0	0	0	0
1	0	0	0	0	0	0	0	0
0	0	0	0	0	0	0	0	0
1	0	0	0	0	0	0	0	0
0	0	0	0	0	0	0	0	0
0	0	0	0	0	0	0	0	0
0	0	0	0	0	0	1	0	0
0	0	0	0	0	0	0	0	0
0	0	0	0	0	0	0	0	0
0	0	0	0	0	0	0	0	0
1	0	0	0	0	0	0	0	0
1	0	0	0	0	0	0	0	0
1	0	0	0	0	0	0	0	0
1	0	0	0	0	0	0	0	0
1	0	0	0	0	0	0	0	0
1	0	0	0	0	0	0	0	0
0	0	0	0	0	0	0	1	0
1	0	0	0	0	0	0	1	0
0	0	0	0	0	0	0	0	0
0	0	0	1	0	0	0	0	0
0	1	0	0	0	0	0	0	0
1	1	1	0	0	0	0	0	0
1	0	0	0	0	0	0	0	0
0	0	0	0	0	0	0	0	0
0	0	0	0	0	0	0	0	0
1	0	0	0	0	0	0	0	0
0	0	0	0	0	0	0	0	0
0	0	0	0	1	0	0	0	0
0	0	0	0	0	0	0	0	0
1	0	0	0	0	0	0	0	0

1	0	0	0	0	0	0	0	0
0	0	0	0	0	0	0	1	0
0	0	0	0	0	0	0	0	0
0	0	0	0	0	0	0	0	0
0	0	0	0	0	0	0	0	0
0	0	0	0	0	0	0	0	0
0	0	0	0	0	0	0	0	0
0	0	0	1	0	0	0	0	0
0	0	0	0	0	0	0	1	0
0	0	0	0	0	0	0	0	0
0	0	0	0	1	0	0	0	0
0	0	0	1	1	0	0	0	0
0	0	0	0	0	0	0	0	0
0	0	0	0	0	0	0	0	0
0	0	0	0	0	0	0	0	0
0	0	0	0	0	0	0	0	0
0	0	0	0	0	0	0	0	0
0	0	0	0	0	0	0	0	0
0	0	0	0	0	0	0	0	0
1	0	0	0	0	0	0	0	0
0	0	0	0	0	0	0	0	0
1	0	0	0	1	0	0	0	0
1	0	0	0	0	0	0	0	0
0	0	0	0	0	0	0	0	0
0	0	0	0	0	0	0	0	0
0	0	0	0	0	0	0	0	0
0	0	0	0	0	0	0	0	0
0	0	0	0	0	0	0	0	0
0	0	0	0	0	0	0	0	0
0	0	0	0	0	0	0	0	0
0	0	0	0	0	0	0	0	0
0	0	0	0	0	0	0	0	0
1	0	0	0	0	0	0	0	0
0	0	0	0	0	0	0	0	0
1	0	0	0	0	0	0	1	0
0	0	0	0	0	0	1	0	0
0	0	0	0	0	0	0	0	0
1	1	0	0	0	0	0	1	0
0	0	0	0	0	0	0	0	0
1	0	0	0	1	0	0	0	0
1	0	0	0	0	0	1	0	0
0	0	0	0	0	0	0	0	0
0	0	0	0	1	0	0	0	0
0	0	0	0	0	0	0	0	0
0	0	1	0	0	0	0	0	0
0	0	0	0	0	0	0	0	0
0	0	0	0	0	0	0	0	0
0	1	0	0	0	0	0	1	0
0	1	0	0	0	0	1	0	0
0	0	0	0	0	0	0	0	0
0	0	0	0	0	0	0	0	0
0	0	0	0	0	0	0	0	0
0	1	0	0	0	0	0	1	0
0	1	0	0	0	0	0	0	0
0	0	0	0	0	0	0	0	0
1	0	0	0	0	0	0	0	0

0	0	0	1	0	0	0	1	0
0	0	0	0	0	0	0	0	0
0	0	0	0	1	0	0	0	0
0	0	0	0	0	0	0	0	0
0	0	0	0	0	0	1	0	0
1	0	0	0	0	0	0	0	0
1	0	0	0	0	0	0	0	0
0	0	0	0	0	0	0	0	0
0	0	0	0	0	0	0	0	0
1	0	0	0	0	0	0	0	0
0	0	0	0	0	0	0	0	0
0	0	0	0	0	0	0	0	0
0	1	0	0	0	0	0	0	0
1	0	0	0	0	0	0	0	0
0	0	0	0	0	0	0	0	0
1	0	0	1	0	0	0	0	0
1	0	0	0	0	0	0	0	0
0	0	0	0	0	0	0	0	0
0	1	0	0	0	0	0	1	0
0	0	0	0	0	0	0	0	0
0	0	0	0	1	0	0	0	0
1	0	0	0	0	0	0	0	0
0	0	0	0	0	0	0	0	0
0	0	0	0	0	0	0	0	0
0	0	0	0	0	0	1	0	0
0	0	0	0	0	0	0	0	0
1	0	0	0	1	0	0	0	0
0	0	0	0	0	0	1	0	0
1	0	0	0	0	0	0	1	0
0	0	0	0	0	0	0	0	0
0	1	0	0	0	0	1	1	0
1	0	0	0	0	0	0	0	0
0	0	0	0	1	0	0	1	0
0	0	0	0	0	0	0	0	0
0	0	0	0	0	0	0	0	0
0	0	0	0	0	0	0	0	0
0	1	0	0	0	0	0	0	0
1	0	0	0	0	0	0	0	0
0	0	0	0	0	0	0	0	0
0	0	0	0	0	0	0	0	0
0	0	0	0	0	0	0	1	0
0	0	0	0	0	0	0	0	0
0	0	0	0	0	0	0	0	0
0	0	0	0	0	0	0	0	0
0	0	0	0	0	0	0	0	0
0	0	0	0	0	0	0	0	0
0	0	0	0	0	0	0	0	0
0	0	0	0	0	0	0	0	0
0	0	0	0	0	0	0	0	0
0	0	0	0	0	0	0	0	0
0	0	0	0	0	0	0	0	0
1	0	0	0	0	0	0	0	0
1	0	0	0	0	0	0	0	0
0	0	0	0	0	0	0	0	0
1	0	0	0	0	0	0	0	0
0	0	0	0	0	0	0	0	0

0	0	0	0	0	0	0	0	0
1	0	1	0	0	0	0	0	0
0	0	0	0	0	0	0	0	0
1	0	0	0	0	0	0	0	0
0	0	0	0	0	0	0	0	0
0	0	1	0	0	0	0	0	0
1	0	0	0	0	0	0	0	0
0	0	0	0	0	0	0	0	0
0	0	0	0	0	0	0	0	0
0	0	1	0	0	0	0	0	0
0	0	0	0	0	0	1	0	0
0	0	0	0	0	0	0	0	0
1	0	0	0	0	0	0	0	0
0	0	0	0	0	0	0	0	1
1	1	0	0	0	0	0	0	0
0	0	1	0	0	0	0	0	0
0	0	0	0	0	0	0	0	0
0	0	0	0	1	0	0	0	0
1	0	1	0	0	0	0	0	0
0	0	0	0	0	0	0	0	0
0	0	0	0	0	0	0	0	0
0	0	0	0	0	0	0	0	0
0	0	0	0	0	0	0	0	0
0	0	0	0	0	0	0	1	0
1	0	0	0	0	0	0	1	0
0	0	0	0	0	0	0	0	0
0	0	0	0	0	0	0	0	0
1	0	0	0	0	0	0	0	0
0	0	0	0	0	0	0	0	0
0	0	0	0	0	0	0	0	0
0	0	0	0	0	0	0	0	0
0	0	0	0	0	0	0	0	0
0	0	0	0	1	0	0	0	0
0	0	0	0	0	0	0	0	0
0	0	0	0	0	0	0	0	0
0	0	0	0	1	0	0	0	0
1	0	0	0	1	0	0	0	0
0	0	0	0	0	0	0	0	0
0	1	0	0	0	0	0	0	0
1	0	0	0	0	0	0	0	0
0	0	0	0	0	0	0	0	0
0	0	0	0	0	0	0	0	0
0	0	0	0	0	0	0	0	0
1	0	0	0	0	0	0	0	0
0	0	0	0	1	0	0	0	0
0	0	0	0	0	0	0	0	0
0	0	0	0	0	0	0	0	0
0	0	0	0	0	0	0	0	0
0	0	0	0	0	1	0	0	0
1	0	0	0	0	0	0	0	0
0	0	0	0	0	0	0	0	0
0	0	0	0	0	0	0	0	0
0	0	0	0	0	0	0	0	0
0	0	0	0	0	0	0	0	0
0	0	0	0	0	0	0	0	0
1	0	0	0	0	0	0	0	0

0	0	0	0	0	0	0	0	0
0	0	0	0	0	0	0	0	0
0	0	0	0	1	0	0	0	0
0	0	0	0	0	0	0	0	0
1	0	0	0	0	0	0	0	0
1	0	0	0	1	0	0	0	0
1	0	0	0	0	0	0	0	0
1	0	0	0	0	0	0	0	0
1	0	0	0	0	0	0	0	0
0	0	0	0	0	0	0	0	0
0	0	0	0	0	0	0	0	0
1	0	0	0	0	0	0	1	0
0	0	0	0	0	0	0	0	0
1	0	0	0	0	0	0	0	0
0	0	0	0	0	0	0	0	0
0	0	0	0	0	0	0	1	0
0	0	0	0	0	0	0	0	0
0	0	0	0	0	0	0	0	0
0	0	0	0	0	0	0	0	0
0	0	0	0	0	0	0	0	0
0	0	0	0	0	0	0	0	0
1	0	0	0	0	0	0	0	0
1	0	0	0	0	0	0	1	0
0	0	0	0	0	0	0	0	0
1	0	0	0	0	0	0	0	0
0	0	0	0	0	0	0	0	0
1	0	0	0	0	0	0	0	0
0	0	0	0	0	0	0	0	0
0	0	0	0	0	0	0	0	0
0	0	0	0	0	0	0	0	0
0	0	0	0	0	0	0	0	0
0	0	0	0	0	0	0	0	0
0	0	0	0	0	0	0	0	0
0	0	0	0	0	0	0	0	0
0	0	0	0	1	0	0	0	0
0	0	0	0	0	0	0	1	0
1	0	0	0	0	0	0	0	0
0	0	0	0	0	0	0	0	0
0	0	0	0	0	0	0	0	0
1	0	0	0	0	0	0	0	0
1	0	0	0	0	0	0	0	0
1	0	0	0	0	0	0	0	0
0	0	0	0	0	0	0	0	1
0	0	0	0	1	0	0	0	0
0	0	0	0	0	0	0	0	0
0	0	0	0	0	0	0	0	0
0	0	0	0	0	0	0	0	0
0	1	0	0	0	0	0	0	0
0	0	0	0	0	0	0	0	0
0	0	0	0	0	0	0	1	0
0	0	0	0	0	0	0	0	0
1	0	0	0	0	0	0	0	0
1	0	0	0	0	0	0	0	0
0	0	0	0	0	0	0	0	0
1	0	0	0	0	0	0	0	0
0	0	0	0	0	0	0	0	0

0	0	0	0	0	0	0	1	0
0	0	1	0	0	0	0	1	0
0	0	1	0	0	0	0	0	0
0	0	0	0	0	0	0	0	0
1	0	0	0	0	0	0	0	0
0	0	0	0	0	0	0	0	0
0	0	0	0	0	0	0	0	0
0	0	0	0	0	0	0	1	0
0	0	0	0	0	0	0	0	0
1	0	0	0	0	0	0	0	1
1	0	0	0	1	0	0	0	0
0	0	0	0	0	0	0	0	0
0	0	0	0	0	0	0	1	0
0	0	0	0	0	0	0	0	0
0	0	0	0	0	0	0	0	0
0	0	0	0	0	0	0	1	0
0	0	0	0	1	0	0	0	0
0	0	0	0	0	0	0	0	0
0	0	0	0	0	0	0	0	0
0	0	0	0	0	0	0	0	0
0	0	0	0	0	0	0	0	0
0	0	0	0	0	0	0	0	0
0	0	1	1	0	0	0	1	0
0	0	0	0	0	0	0	0	0
1	0	0	0	0	0	0	0	0
0	0	0	0	0	0	0	0	0
0	0	0	0	0	0	0	0	0
0	0	0	0	0	0	0	0	0
0	0	0	0	0	0	0	0	0
1	0	0	0	0	0	0	0	0
0	0	0	0	0	0	0	1	0
0	0	0	0	0	0	0	0	1
0	0	0	0	0	0	0	1	0
0	0	0	0	0	0	0	0	0
0	0	0	0	0	0	0	0	0
0	0	0	0	0	0	0	0	0
1	0	0	0	0	0	0	1	0
0	0	0	0	0	0	0	1	0
1	0	0	0	0	0	0	0	0
0	0	0	0	0	0	0	0	0
0	0	0	0	0	0	0	0	0
0	0	0	0	0	0	0	0	0
0	0	0	0	0	0	0	0	0
0	0	1	0	0	0	0	0	0
0	0	0	0	0	0	0	1	1
1	0	0	0	0	0	1	0	0
0	0	0	0	0	0	0	0	0
1	0	0	0	0	0	0	0	0
0	0	0	0	0	0	0	0	0
1	0	0	0	0	0	1	0	0
0	0	0	0	0	0	0	1	0
0	0	0	0	0	0	0	0	0
0	0	0	0	0	0	0	0	0
0	0	0	0	0	0	0	0	0
1	0	0	0	0	0	0	0	0
0	0	0	0	1	0	0	0	0

1	0	0	0	0	0	0	0	0
0	1	0	0	0	0	0	1	0
1	0	0	0	0	0	0	0	0
1	0	0	0	0	0	0	1	0
0	0	0	1	0	0	0	1	0
0	0	0	0	1	0	0	0	0
0	0	0	0	1	0	0	0	0
1	0	1	0	0	0	0	0	0
0	1	0	0	0	0	0	0	0
0	1	0	0	0	0	0	1	0
0	0	0	0	0	0	0	0	0
0	0	0	0	0	0	0	0	0
0	0	0	0	0	0	0	0	0
0	0	0	0	0	0	1	1	0
0	0	0	0	0	0	0	0	0
0	0	0	0	0	0	0	0	0
0	0	0	0	0	0	0	0	0
1	0	0	0	0	0	0	0	0
0	0	0	0	0	0	0	0	0
0	0	0	0	0	0	0	0	0
0	0	0	0	0	0	0	0	0
0	0	0	0	0	0	0	0	0
0	0	0	0	0	0	0	0	0
0	0	0	0	0	0	0	0	0
1	0	0	0	0	0	0	0	0
0	0	0	0	0	0	0	1	0
0	0	0	0	0	0	0	0	0
0	0	0	0	0	0	1	0	0
1	0	0	0	0	0	0	0	0
0	0	0	0	0	0	0	0	0
0	0	0	0	0	0	0	0	0
1	0	0	0	0	0	0	0	0
1	0	0	0	0	0	0	0	0
0	0	0	0	0	0	0	0	0
0	0	0	0	0	0	0	0	0
1	0	0	0	0	0	0	0	0
1	0	0	0	0	0	0	0	0
0	0	0	0	0	0	0	0	0
0	0	0	0	0	0	0	0	0
0	0	0	0	0	0	0	0	0
0	0	0	0	0	0	0	0	0
0	0	0	0	0	0	0	1	0
1	0	0	0	1	0	0	0	0
0	0	0	0	0	0	0	0	0
0	0	0	0	0	0	0	0	0
0	0	0	0	0	0	0	0	0
0	0	0	0	1	0	0	0	0
0	0	0	0	0	0	0	0	0
0	0	0	0	0	0	0	0	0
0	0	0	0	0	0	0	0	0
0	1	0	0	0	0	0	0	0
0	0	0	0	0	0	0	0	0
0	1	0	0	0	0	0	0	0
0	0	0	0	0	0	0	0	0
0	0	0	0	0	0	0	1	0
0	0	0	0	0	0	0	0	0

0	0	0	0	0	0	0	0	0
0	0	0	0	0	0	0	0	0
0	0	0	0	0	0	1	0	0
0	0	0	0	0	0	0	0	0
0	0	0	0	0	0	0	0	0
0	0	0	0	0	0	0	0	0
0	0	0	0	0	0	0	0	0
1	0	0	0	0	0	0	0	0
0	0	0	0	0	0	0	0	0
0	0	0	0	0	0	0	0	0
0	1	0	0	0	0	0	1	0
1	0	0	0	0	0	0	0	0
0	1	0	0	0	0	0	0	0
0	0	0	0	0	0	0	0	0
0	0	0	0	1	1	0	0	0
0	0	0	0	0	0	0	0	0
1	0	0	0	0	0	0	0	0
0	0	0	0	0	0	0	0	0
0	0	0	0	0	0	0	0	0
1	0	0	0	0	0	0	0	0
0	0	0	0	0	0	0	0	0
0	0	0	0	0	0	0	0	0
1	0	0	0	0	0	0	0	0
1	0	0	0	0	0	0	0	0
0	0	0	0	0	0	0	0	0
0	0	0	0	0	0	0	1	0
1	0	0	0	0	0	0	0	0
1	0	0	0	1	0	0	0	0
0	0	0	0	0	0	0	1	0
1	0	0	0	0	0	0	0	0
0	0	0	0	0	0	0	0	0
0	1	0	0	0	0	0	0	0
0	0	0	0	0	0	1	0	0
0	0	0	0	0	0	0	0	0
0	0	0	0	0	0	0	0	0
0	0	0	0	0	0	0	0	0
0	0	0	1	0	0	0	0	0
0	0	0	0	0	0	0	0	0
0	0	0	0	0	0	0	0	0
0	0	0	0	0	0	0	1	1
0	0	0	0	0	0	0	0	0
0	0	0	0	0	0	0	0	0
1	0	0	0	0	0	0	0	0
0	0	0	0	0	0	0	0	0
0	0	0	0	0	0	0	0	0
0	0	0	0	0	0	0	0	0
0	0	0	0	0	0	0	0	0
0	0	0	0	0	0	0	0	0
1	0	0	0	0	0	0	0	1
0	0	0	0	0	0	0	0	0
0	0	0	0	0	0	0	0	0
0	0	0	0	0	0	0	0	0
1	0	0	0	0	0	0	1	0
0	0	0	0	0	0	0	1	0
0	0	0	0	0	0	0	0	0
0	0	0	0	0	0	0	0	0

0	0	0	0	1	0	0	0	0
0	0	0	0	0	0	0	0	0
1	1	0	0	0	0	0	0	0
0	0	0	0	0	1	0	0	0
1	0	0	0	0	0	0	0	0
0	0	0	0	0	0	0	0	0
0	0	0	0	0	0	0	0	1
0	0	0	0	0	0	0	0	0
0	0	0	0	0	0	0	0	0
0	0	0	0	0	0	0	0	0
0	0	0	0	0	0	0	0	0
0	0	0	0	1	0	0	0	0
1	0	0	0	1	0	0	0	0
1	0	0	0	0	0	0	0	0
0	0	0	0	0	0	0	0	0
0	1	0	0	0	0	0	0	0
0	0	0	0	0	0	0	1	0
1	1	0	0	1	0	0	0	0
0	0	0	0	1	0	0	0	0
0	0	0	0	0	0	0	0	0
1	0	0	0	0	0	0	0	0
1	0	0	0	0	0	1	0	0
0	0	0	0	0	0	0	0	0
0	0	0	0	0	0	0	0	0
0	0	0	0	0	0	0	0	0
1	1	1	0	0	0	0	0	0
0	0	0	0	0	0	0	0	0
1	0	0	0	0	0	0	0	0
0	1	0	0	0	0	0	0	0
0	0	0	0	0	0	0	0	0
0	0	0	0	0	0	0	0	0
0	0	0	0	0	0	0	0	0
0	0	0	0	0	0	0	0	0
0	0	0	0	0	0	0	0	0
0	0	0	0	0	0	0	1	0
0	0	0	0	0	0	0	0	0
1	0	0	0	0	0	0	1	0
0	0	0	0	0	0	0	0	0
0	0	0	0	0	0	0	0	0
0	0	0	0	0	0	0	1	0
0	0	0	0	0	0	0	1	0
0	0	0	0	1	0	0	1	0
1	0	0	0	0	0	0	0	0
0	0	1	0	0	0	0	0	0
0	0	0	0	0	0	0	0	0
0	0	0	0	0	0	0	0	0
0	0	0	0	0	0	0	0	0
1	0	0	0	0	0	0	0	0
0	0	0	0	0	0	0	1	0
0	0	0	0	0	0	0	0	0
0	0	0	0	0	0	0	0	0
0	0	0	0	0	0	0	0	0
0	0	0	0	0	0	0	0	0
0	0	1	0	0	0	0	0	0
0	0	0	0	0	0	0	0	0
1	0	0	0	0	0	0	1	0

0	0	0	0	0	0	0	0	0
0	0	0	0	0	0	0	0	0
0	0	0	0	0	0	0	0	0
0	0	0	0	0	0	0	1	0
0	0	0	0	0	0	0	1	0
0	0	0	0	0	0	0	0	0
0	0	1	0	0	0	0	0	0
1	0	0	0	0	0	0	1	0
0	0	0	0	0	0	0	0	0
0	0	0	0	0	0	0	0	0
0	0	0	0	0	0	0	0	0
0	0	0	0	0	0	0	0	0
0	0	0	0	0	0	0	0	0
0	0	0	0	0	0	1	0	0
1	0	0	0	0	0	1	0	0
1	0	0	0	0	0	0	0	0
1	0	0	0	0	0	0	0	0
0	0	0	0	0	0	0	0	0
0	0	0	0	0	0	0	0	0
1	0	0	0	0	0	0	0	0
0	0	0	0	0	0	0	0	0
0	0	0	0	0	0	0	0	0
1	0	0	0	0	0	0	0	0
0	0	0	0	0	0	0	0	0
0	0	0	1	0	0	0	1	0
0	0	0	0	0	0	0	0	0
1	0	0	0	0	0	0	0	0
0	0	0	0	0	0	0	0	0
0	0	0	0	0	0	0	0	0
0	0	0	0	0	0	0	0	0
1	0	1	0	0	0	0	0	0
0	0	0	0	0	0	0	0	0
1	0	1	0	0	0	0	0	0
0	0	0	0	0	0	0	0	0
0	0	0	0	0	0	0	0	0
0	0	0	0	0	0	0	0	0
0	0	0	0	1	0	0	1	0
1	0	0	1	0	0	0	0	0
0	0	0	0	0	0	0	0	0
1	0	0	0	0	0	0	0	0
0	0	0	0	0	0	0	0	0
0	0	0	0	0	0	0	0	0
1	0	0	0	0	0	0	0	0
1	0	0	0	0	0	0	0	0
1	1	0	0	0	0	0	0	0
1	0	0	0	0	0	0	0	0
0	0	0	0	0	0	0	0	0
1	0	0	0	1	0	0	0	0
0	0	0	0	0	0	0	0	0
1	0	0	0	0	0	0	1	0
1	0	0	0	0	0	0	1	0
0	0	0	0	0	0	0	1	0
0	0	0	0	0	0	0	0	0
0	0	0	0	0	0	0	0	0
0	0	0	0	0	0	0	0	0
0	0	0	0	0	0	0	1	0

0	0	0	0	0	0	0	0	0
1	0	0	0	0	0	0	0	0
0	0	0	0	0	0	0	0	0
0	0	0	0	0	0	1	0	0
0	0	0	0	0	0	0	0	0
0	0	0	0	0	0	0	1	0
0	0	0	0	0	0	0	0	0
1	0	0	0	0	0	1	0	0
0	0	0	0	0	0	0	0	0
1	0	0	0	0	0	0	1	0
0	0	0	0	0	0	0	0	0
1	1	0	0	0	0	0	0	0
0	0	0	0	0	0	0	0	0
0	0	0	0	0	0	0	0	0
0	0	0	0	0	0	0	1	0
0	0	0	0	0	0	0	1	0
1	0	0	0	0	0	0	0	0
0	0	0	0	0	0	0	0	0
0	0	0	0	0	0	0	0	0
1	0	0	0	0	0	0	0	0
0	0	0	0	0	0	0	0	0
1	0	0	0	0	0	0	0	0
0	0	0	0	0	0	0	1	0
0	0	0	0	0	0	0	0	0
0	0	0	0	0	0	0	0	0
0	0	0	0	0	0	0	0	0
1	0	0	0	0	0	0	0	0
0	0	0	0	0	0	0	1	0
0	0	0	0	0	0	0	0	0
0	0	0	0	0	0	0	0	0
0	0	0	0	1	0	0	0	0
0	0	0	0	0	0	0	0	0
0	0	0	0	0	0	0	0	0
0	0	0	0	0	0	0	0	0
0	0	0	0	0	0	0	1	0
0	0	0	0	0	0	0	0	0
0	0	0	0	0	0	0	0	0
0	0	0	0	0	0	0	0	0
1	0	0	0	0	0	0	0	0
0	0	0	0	0	0	0	0	0
0	0	1	0	0	0	0	0	0
0	0	0	0	0	0	0	0	0
0	0	0	0	0	0	0	0	0
0	0	0	0	0	0	1	0	0
0	1	0	0	0	0	0	0	0
1	0	0	0	0	0	0	1	0
0	0	0	0	0	0	0	0	0
0	0	0	0	0	0	0	0	0
0	0	0	0	0	0	0	0	0
1	0	0	0	0	0	0	0	0
0	0	0	0	0	0	1	0	0
0	0	0	0	0	0	0	0	1
0	0	0	0	0	0	0	0	0
1	0	0	0	1	0	0	0	0
0	0	0	0	0	0	0	0	0
0	0	0	0	0	0	0	0	0

1	0	0	0	0	0	0	0	0
0	0	0	0	0	0	0	0	0
0	0	0	0	0	0	0	0	0
1	0	0	0	0	0	0	0	0
0	0	0	0	0	0	0	0	0
1	0	0	0	0	0	0	0	0
0	0	0	0	0	0	0	0	0
1	0	0	0	0	0	0	0	0
1	0	0	0	0	0	0	0	0
1	0	0	0	0	0	0	0	0
0	0	0	0	0	0	0	0	0
0	0	0	0	0	0	0	0	0
0	0	0	0	0	0	0	0	1
0	0	0	0	0	0	0	1	0
0	0	0	0	0	0	0	0	0
0	0	0	0	0	0	0	0	0
0	0	0	0	0	0	0	0	0
0	0	0	0	0	0	0	0	0
1	0	0	0	0	0	1	0	0
0	0	0	0	0	0	0	0	0
0	0	0	0	0	0	1	0	0
0	0	0	0	0	0	0	0	0
0	0	0	0	0	0	0	0	0
0	0	0	1	0	0	0	0	0
0	1	0	0	0	0	0	0	0
0	0	0	0	0	0	0	1	0
0	0	1	0	0	0	0	0	0
0	0	0	0	0	0	0	0	0
0	0	0	0	0	0	0	0	0
0	0	0	0	0	0	0	0	0
1	0	0	0	0	0	0	0	0
0	0	0	0	0	0	0	1	0
0	0	0	0	0	0	0	0	0
1	0	0	0	0	0	0	0	0
1	0	0	0	0	0	0	0	0
0	0	0	0	0	0	0	0	0
1	0	0	0	0	0	0	0	0
1	0	0	0	0	0	0	0	0
0	0	0	0	0	0	0	0	0
1	0	0	0	1	0	0	0	0
0	0	0	0	0	0	0	0	0
1	0	0	0	0	0	0	0	0
0	0	0	0	0	0	0	0	0
0	0	0	0	0	0	0	0	0
0	0	0	0	0	0	0	0	0
0	0	0	0	0	0	0	0	0
1	0	0	0	1	0	0	0	0
0	0	0	0	0	0	0	0	0
0	0	0	0	0	0	0	0	0
0	0	0	0	0	0	0	0	0
1	0	1	0	0	0	0	0	0
1	0	0	0	0	0	0	1	0
1	0	0	0	0	0	0	0	0
1	0	0	0	0	0	0	0	0
1	0	0	0	0	0	0	0	0
0	0	0	0	0	0	0	0	0

0	0	0	0	0	0	0	0	0
0	0	0	0	0	0	0	0	0
0	0	0	0	0	0	0	1	1
1	0	0	0	0	0	0	0	0
0	0	0	0	0	0	0	1	0
0	0	0	0	0	0	0	0	0
0	0	0	0	0	0	0	1	0
1	0	0	0	1	0	0	0	0
1	0	0	0	0	0	0	0	0
0	0	0	0	0	0	0	1	0
0	0	0	0	0	0	0	0	0
1	0	0	0	0	0	0	0	0
0	0	0	0	0	0	0	0	0
0	0	0	0	0	0	0	0	0
0	0	0	0	0	0	0	0	0
0	0	0	0	0	0	0	0	0
0	0	0	0	0	0	0	0	0
1	0	0	0	0	0	0	0	0
0	0	0	0	0	0	0	0	0
0	0	0	0	0	0	0	0	0
1	0	0	0	1	0	0	0	0
0	0	0	0	0	0	0	0	0
0	0	0	0	0	0	0	1	0
0	0	0	0	1	0	0	0	0
0	0	0	0	0	0	0	0	0
0	0	0	0	0	0	0	0	0
0	0	0	0	0	0	0	0	0
0	0	0	0	0	0	0	0	0
0	0	0	0	0	0	0	0	0
0	0	0	0	0	0	0	0	0
1	0	0	0	0	0	0	0	0
1	0	0	0	0	0	0	0	0
0	0	0	0	0	0	0	1	0
0	0	0	0	0	0	0	0	0
1	0	0	0	0	0	0	0	0
0	0	0	0	0	0	0	0	0
0	0	0	0	1	0	0	0	0
0	0	0	0	0	0	0	0	0
0	0	0	0	0	0	0	0	0
0	0	0	0	0	0	0	0	0
0	1	1	0	0	0	0	0	0
0	0	0	0	0	0	0	1	0
0	0	0	0	0	0	0	0	0
0	0	0	0	0	0	0	0	0
0	0	0	0	0	0	0	0	0
1	0	1	0	0	0	0	0	0
0	0	0	0	1	0	0	0	0
1	0	0	0	0	0	0	0	0
1	0	0	0	0	0	0	0	0
0	0	0	0	0	0	0	0	0
0	0	0	0	0	0	0	0	0
0	0	0	0	0	0	0	0	0
1	0	0	0	0	0	1	0	0
1	0	0	0	0	0	0	0	0
0	0	0	0	0	0	0	0	0
0	0	0	0	0	0	0	0	0

0	0	0	0	0	0	0	0	0
0	0	0	0	0	0	0	0	0
0	0	1	0	0	0	0	0	0
0	0	0	0	0	0	0	0	0
0	0	0	0	0	0	0	1	0
1	0	0	0	1	0	0	0	0
1	0	0	0	1	0	0	0	0
0	0	0	0	0	0	0	0	0
1	0	0	0	0	0	0	0	0
0	0	0	0	0	0	0	0	0
0	0	0	0	0	0	0	0	0
1	0	0	0	0	0	0	0	0
0	0	0	0	0	0	0	0	0
0	0	0	0	0	0	0	1	0
1	0	0	0	0	0	0	0	0
1	0	0	0	0	0	0	0	0
0	0	0	0	0	0	0	0	0
0	0	0	0	0	0	0	0	0
0	0	0	0	0	0	0	0	0
1	0	0	0	0	0	0	0	0
0	0	1	0	0	0	0	0	0
1	0	0	0	0	0	0	0	0
0	0	0	0	0	0	1	0	0
0	0	0	0	0	0	0	0	0
0	0	0	0	0	0	0	0	0
0	0	0	0	0	0	0	0	0
0	0	0	0	0	0	0	0	0
0	0	0	0	0	0	1	0	0
0	0	0	0	0	0	0	0	0
1	0	1	0	0	0	0	0	0
1	0	0	0	0	0	0	0	0
1	0	0	0	0	0	0	0	0
0	0	0	0	0	0	0	0	0
0	0	0	0	0	0	0	0	0
0	0	0	0	0	0	1	0	1
0	0	0	0	0	0	0	0	0
0	0	0	0	0	0	0	1	0
0	0	0	0	0	0	0	0	0
0	1	0	0	0	0	1	0	0
1	0	0	0	0	0	0	0	0
0	0	0	0	0	0	0	0	0
0	0	0	0	0	0	0	0	0
0	0	0	0	0	0	0	1	0
0	0	0	0	0	0	0	0	0
0	0	0	0	0	0	0	0	0
0	0	0	0	0	0	0	0	0
0	0	0	0	0	0	0	0	0
0	0	0	0	0	0	0	0	0
0	1	0	0	0	0	0	0	0
0	0	0	1	0	0	0	0	0
0	0	0	0	0	0	0	0	0
0	0	0	0	0	0	0	0	0
1	0	0	0	0	0	0	0	0
1	0	0	0	0	0	0	0	0
1	0	0	0	0	0	0	0	0
0	0	0	0	0	0	0	0	0

0	0	0	0	0	0	0	1	0
0	0	0	0	0	0	0	0	0
0	0	0	0	0	0	0	0	0
1	0	0	0	0	0	0	0	0
1	0	0	0	0	0	0	0	0
1	0	0	0	0	0	0	0	0
0	0	0	0	0	0	0	0	0
1	0	0	0	0	0	0	0	0
0	0	0	0	0	0	0	0	0
0	0	0	0	0	0	0	0	0
0	0	0	0	0	0	0	0	0
0	0	0	0	0	0	0	1	0
1	0	0	0	1	0	0	0	0
1	0	0	0	0	0	0	0	0
1	0	0	0	0	0	0	0	0
0	0	0	0	0	0	0	0	0
1	0	0	0	0	0	0	0	0
0	0	0	0	0	0	0	1	0
1	0	0	0	0	0	0	0	0
0	0	0	0	0	0	0	0	0
1	0	0	0	0	0	0	0	0
0	0	0	0	0	0	0	1	0
0	0	0	0	0	0	0	0	0
0	0	0	0	0	0	0	0	0
0	0	0	0	0	0	0	0	0
0	0	0	0	1	0	0	0	0
0	0	0	0	0	0	0	0	0
1	0	0	0	0	0	0	0	0
0	0	0	0	0	0	0	0	0
0	0	0	0	0	0	0	1	0
0	0	0	0	0	0	0	1	0
1	0	0	0	0	0	0	0	0
0	0	0	0	0	0	0	0	0
0	0	0	0	0	0	0	0	0
0	0	0	0	0	0	0	0	0
0	0	0	0	0	0	0	0	0
0	0	0	0	0	0	0	0	0
0	0	0	0	0	0	0	0	0
1	0	0	0	0	0	0	0	0
0	0	0	0	0	0	0	0	0
0	0	1	0	0	0	0	0	0
0	0	0	0	0	0	0	0	0
0	0	0	0	0	0	0	0	0
0	0	1	0	0	0	0	0	0
0	0	0	0	0	0	0	0	0
1	0	0	1	0	0	0	0	0
0	0	0	0	1	0	0	0	0
1	0	0	0	0	0	0	0	1
0	0	0	0	0	0	0	0	0
1	0	1	0	0	0	0	0	0
1	0	0	0	0	0	0	0	0
0	0	0	0	0	0	0	0	0
0	0	0	0	0	0	0	0	0
0	0	0	0	0	0	0	0	0
0	0	0	0	0	0	0	0	0
0	0	0	0	0	0	0	0	0

1	0	0	0	0	0	0	0	0
0	0	0	0	0	0	0	0	0
1	0	0	0	0	0	0	0	0
0	0	0	0	0	0	0	0	0
1	0	0	0	0	0	0	0	0
0	0	0	0	0	0	0	0	0
0	0	0	0	0	0	0	0	0
1	0	0	0	0	0	0	0	0
0	0	0	0	0	0	0	0	0
0	0	0	0	0	0	0	0	0
0	0	0	0	0	0	0	1	1
0	0	0	0	0	0	0	0	0
0	0	0	0	0	0	0	0	0
0	0	0	0	0	0	0	0	0
0	1	0	0	0	0	0	0	0
0	0	0	0	0	0	0	1	0
0	0	0	0	0	0	0	0	0
0	0	0	0	0	0	0	0	0
0	0	0	0	0	0	0	1	0
1	0	0	0	0	0	0	0	0
0	0	0	0	0	0	0	0	0
0	0	0	0	0	0	0	0	0
1	0	0	0	1	0	0	0	0
0	0	0	0	0	0	0	0	0
0	0	0	0	0	0	0	0	0
0	0	0	0	0	0	0	0	0
0	0	0	0	0	0	0	0	0
0	0	0	0	0	0	0	1	0
0	0	0	0	0	0	0	0	0
0	0	0	0	0	0	0	0	0
0	0	0	0	0	0	0	0	0
0	0	0	0	0	0	0	0	0
0	0	0	0	0	0	0	0	0
0	0	0	0	0	0	0	0	0
1	0	0	0	0	0	0	0	0
0	0	0	0	0	0	0	0	0
0	0	0	0	0	0	0	0	0
0	0	0	0	0	0	0	0	0
0	0	0	0	0	0	0	0	0
1	0	0	0	0	0	1	0	0
1	0	0	0	0	0	0	0	0
0	0	0	0	0	0	0	0	0
0	0	0	0	0	0	0	0	0
0	0	0	0	0	0	0	0	0
0	0	0	0	0	0	0	0	0
0	0	0	0	0	0	0	0	0
0	0	0	0	0	0	0	0	0
0	0	0	0	0	0	0	0	0
0	0	0	0	0	0	0	0	0
1	0	0	0	0	0	0	0	0
0	0	0	0	0	0	0	0	0
1	0	0	0	0	0	1	0	0
1	0	0	0	0	0	0	0	0
0	0	0	0	1	0	0	0	0
0	0	0	0	0	0	0	0	0
1	0	0	0	0	0	0	0	0

1	0	0	0	0	0	0	0	0
0	0	0	0	0	0	0	0	0
0	0	0	0	0	0	0	0	0
1	0	0	0	0	0	0	0	0
0	0	1	0	0	0	0	0	0
0	0	0	1	0	0	0	0	0
0	0	0	0	1	0	0	0	0
1	0	0	0	0	0	0	0	0
1	0	0	0	0	0	0	0	0
0	0	0	0	0	0	0	0	0
0	0	0	1	1	0	0	1	0
0	0	0	0	0	0	0	0	0
0	0	1	0	0	0	0	0	0
1	1	0	0	0	0	0	0	0
1	0	0	0	1	0	0	0	0
1	0	0	0	0	0	0	0	0
1	0	0	0	0	0	0	1	0
0	0	0	0	0	0	0	0	0
0	0	0	0	0	0	0	1	0
1	0	0	0	0	0	0	0	0
1	0	0	0	0	0	0	0	0
1	1	0	0	0	0	0	0	0
0	0	0	0	0	0	0	0	0
0	0	0	0	0	0	1	0	0
0	0	0	0	0	0	0	0	0
0	0	0	0	0	0	0	0	0
0	0	0	0	0	0	0	0	0
0	0	0	0	0	0	0	0	0
0	0	0	0	0	0	0	0	0
0	0	0	0	0	0	0	0	0
1	0	0	0	1	0	0	0	0
0	0	0	0	0	0	0	0	0
0	0	0	0	0	0	0	0	0
0	0	0	0	0	0	0	0	0
0	0	0	0	0	0	0	0	0
0	0	0	0	0	0	0	0	0
1	0	0	0	0	0	0	0	0
0	0	0	0	0	0	1	1	0
0	0	0	0	0	0	0	0	0
0	0	0	0	0	0	0	0	0
0	0	0	0	0	0	0	0	0
1	0	0	0	0	0	0	0	0
1	0	0	0	1	0	0	0	0
0	0	0	0	0	0	0	0	0
0	0	0	0	0	0	0	1	0
0	0	0	0	0	0	0	1	0
1	0	0	0	0	0	0	0	0
0	0	0	0	0	0	0	0	0
1	0	0	1	0	0	0	0	0
0	0	0	0	0	0	0	0	0
0	0	0	0	0	0	0	1	0
1	0	0	0	0	0	0	1	0
0	0	0	0	0	0	0	0	0
1	1	0	0	0	1	0	0	0
0	0	0	0	0	0	0	0	0
0	0	0	0	0	0	0	1	0

0	0	0	0	0	0	0	1	0
1	0	0	0	0	0	0	1	0
0	0	0	0	0	0	0	0	0
0	0	0	0	0	0	0	1	0
0	1	0	0	0	0	0	0	0
0	0	0	0	0	0	0	0	0
0	0	0	0	1	0	0	0	0
0	0	0	0	0	0	0	0	0
1	0	0	0	0	0	0	0	0
0	0	0	0	0	0	0	0	0
0	0	0	0	0	0	0	0	0
0	0	0	0	0	0	0	0	0
0	0	0	0	0	0	0	0	0
0	0	0	0	0	0	0	0	0
0	0	0	0	0	0	0	1	1
0	0	0	0	0	0	0	1	0
0	0	0	0	0	0	0	0	0
1	0	0	0	0	0	0	0	0
0	0	0	0	0	0	0	1	0
0	0	0	0	0	0	0	0	0
0	0	0	0	0	0	0	0	0
1	0	0	0	1	0	0	0	0
0	0	0	0	0	0	0	1	0
0	0	0	0	0	0	0	0	0
1	0	0	0	0	0	0	0	0
0	0	0	0	0	0	0	1	1
0	0	0	0	0	0	0	0	0
0	0	0	0	0	0	0	0	0
1	0	0	0	0	0	0	0	0
0	0	0	0	0	0	0	0	0
0	0	0	0	0	0	0	0	0
1	0	0	0	1	0	0	0	0
0	0	0	0	0	0	0	0	0
0	1	0	0	0	0	0	0	0
0	0	0	0	0	0	0	1	0
1	0	0	0	0	0	0	0	0
0	0	0	0	0	0	0	0	0
0	0	0	0	0	0	0	0	0
1	0	0	0	0	0	0	0	0
0	0	0	0	0	0	0	0	0
0	1	0	0	0	0	0	0	0
0	0	0	0	0	0	0	0	0
0	0	0	0	0	0	0	0	0
0	0	0	0	0	0	0	0	0
1	0	1	0	0	0	0	0	0
0	1	0	0	0	0	0	0	0
0	0	0	0	0	0	0	0	0
0	0	0	0	0	0	0	1	0
0	0	0	0	0	0	0	0	0
0	0	0	0	0	0	0	0	0
1	0	0	0	0	0	0	0	0
0	0	0	0	0	0	0	0	0
0	0	0	0	0	0	1	0	0
1	0	0	0	0	0	1	0	0
0	0	0	0	0	0	0	0	0
0	0	0	0	0	0	0	0	0

0	0	1	0	0	0	0	0	0
0	0	0	0	0	0	0	0	0
0	0	0	0	0	0	0	0	0
1	0	0	0	0	0	0	0	0
0	0	1	0	0	0	0	0	0
0	0	0	0	0	0	0	1	0
1	0	0	0	0	0	0	0	0
1	0	0	0	0	0	0	1	0
0	0	1	0	0	0	0	0	0
0	0	0	0	0	0	0	0	0
0	0	0	0	0	0	1	0	0
0	0	0	0	0	0	0	0	0
0	0	0	0	0	0	0	0	0
1	0	0	0	0	0	0	0	0
1	0	0	0	0	0	0	0	1
0	0	0	0	0	0	1	1	0
0	0	0	0	0	0	0	1	0
1	0	0	0	0	0	0	0	0
0	0	0	0	0	0	0	0	0
0	0	0	0	0	0	0	0	0
1	0	0	0	0	0	0	0	0
1	0	0	0	0	0	0	0	0
1	0	0	0	0	0	0	0	0
0	0	0	0	0	0	0	0	0
0	0	0	0	0	0	0	0	0
0	0	0	0	0	0	0	0	0
0	1	0	0	0	0	0	0	0
0	0	0	0	0	0	0	0	0
1	0	0	0	0	0	0	0	0
1	0	0	0	0	0	0	0	0
0	0	0	0	1	0	0	0	0
0	0	0	0	1	0	0	0	0
0	0	0	0	0	0	0	0	0
1	0	0	0	0	0	0	0	0
0	0	0	0	0	0	0	0	0
1	0	0	0	0	0	1	0	0
0	0	0	0	0	0	0	0	0
0	0	0	0	0	0	0	0	0
0	0	0	0	0	0	0	0	0
1	0	0	0	0	0	0	0	0
0	0	0	0	0	0	0	0	0
0	0	0	0	0	0	0	0	0
0	0	0	0	0	0	0	1	0
0	0	1	0	0	0	0	0	0
0	0	0	0	0	0	1	0	0
0	0	0	0	0	0	0	0	0
1	0	0	0	0	0	0	0	0
0	0	0	0	0	0	0	0	0
1	0	0	0	0	0	0	1	0
1	0	0	0	0	0	0	0	0
0	0	0	0	0	0	0	0	0
1	0	0	0	0	0	0	0	0
0	0	0	0	0	0	0	0	0
0	0	0	0	0	0	0	0	0
0	0	0	0	0	0	0	0	0
0	0	0	0	0	0	0	1	0

0	0	0	0	0	0	0	0	0
0	0	0	0	0	0	0	0	0
0	0	0	0	0	0	0	0	0
0	0	0	0	0	0	0	1	0
1	0	0	0	0	0	0	0	0
0	0	0	0	0	0	0	0	0
0	0	0	0	0	0	0	0	0
1	0	0	0	0	0	0	0	0
0	0	0	0	0	0	0	0	0
0	0	0	0	0	0	0	0	0
0	0	0	0	0	0	0	0	0
0	0	0	0	0	0	0	0	0
0	0	0	0	0	0	0	0	0
1	0	1	0	0	0	0	0	0
0	0	0	0	0	0	0	0	0
1	1	0	0	0	0	0	0	0
0	0	0	0	0	0	0	0	0
0	0	0	0	1	0	0	0	0
0	0	0	0	0	0	0	0	0
0	0	0	0	0	0	0	1	0
1	0	0	0	0	0	0	0	0
0	0	0	0	0	0	0	0	0
0	0	0	0	0	0	0	0	0
0	0	0	0	0	0	0	0	0
0	0	0	0	1	0	0	0	0
0	0	0	0	0	0	0	0	0
0	0	0	0	0	0	0	0	0
0	0	0	0	0	0	0	1	0
0	0	0	0	0	0	0	0	0
0	0	0	0	0	0	0	0	0
0	0	0	0	0	0	0	0	0
0	0	0	0	0	0	0	0	0
0	0	0	0	1	0	0	0	0
0	0	0	0	0	0	0	0	0
0	0	0	0	0	0	0	0	0
0	0	0	0	0	0	0	0	0
0	0	0	0	0	0	0	0	0
0	0	0	0	0	0	0	0	0
0	0	0	0	0	0	0	0	0
0	0	0	0	0	0	0	0	0
0	0	0	1	0	0	0	0	0
0	0	0	0	0	0	0	0	0
0	0	0	0	0	0	0	0	0
0	0	0	0	0	0	0	0	0
0	0	0	0	0	0	0	0	0
0	0	0	1	0	0	0	0	0
0	0	0	0	0	0	0	0	0
0	0	0	0	0	0	0	0	0
0	0	0	0	0	0	0	0	0
0	0	0	0	0	0	0	0	0
0	0	0	0	0	0	0	1	0
0	0	0	0	0	0	0	0	0
0	0	0	0	0	0	0	0	0
0	0	0	0	0	0	0	0	0
0	0	0	0	0	0	1	0	0
0	0	0	0	0	0	0	0	0

0	0	0	0	0	0	0	0	0
0	0	0	0	0	0	0	0	0
0	0	0	0	0	0	0	0	0
0	0	0	0	0	0	0	0	0
0	0	0	0	0	0	0	0	0
0	0	0	0	0	0	0	0	0
0	0	0	0	0	0	0	0	0
0	0	0	1	0	0	0	0	0
0	0	0	0	1	0	0	0	0
0	0	0	0	0	0	0	0	0
1	0	0	0	0	0	1	0	0
0	1	0	0	0	0	0	0	0
0	0	0	0	0	0	0	0	0
0	1	0	1	0	0	1	0	0
0	0	0	0	0	0	0	0	0
0	0	0	0	0	0	0	1	0
0	0	0	0	0	0	0	1	0
0	0	0	0	0	0	0	1	0
0	0	0	0	0	0	0	0	0
0	1	0	0	0	0	0	0	0
0	1	0	0	0	0	0	0	0
0	0	0	0	0	0	0	0	0
0	0	0	0	0	0	0	0	0
0	0	0	0	0	0	0	0	0
0	0	0	0	0	0	0	0	0
0	0	0	0	1	0	0	0	0
0	0	0	0	0	0	0	0	0
1	0	0	0	1	0	0	0	0
0	0	0	0	0	0	0	0	0
0	0	0	0	0	0	0	0	0
0	1	0	0	0	0	0	0	0
0	0	0	0	0	0	0	0	0
0	1	0	0	0	0	0	0	0
0	0	0	0	0	0	1	1	0
0	0	0	0	0	0	0	0	1
0	0	0	0	0	0	0	0	0
0	0	0	0	0	0	1	1	0
0	0	0	0	0	0	0	0	0
0	0	0	0	0	0	0	0	0
0	0	0	0	0	0	0	0	0
0	0	0	0	0	0	0	0	0
0	0	0	0	0	0	0	0	0
0	0	0	0	0	0	0	0	0
0	0	0	0	0	0	0	0	0
0	0	0	0	0	0	0	0	0
0	1	1	0	0	0	0	0	0
0	0	0	0	1	0	0	0	0
0	0	0	0	0	0	0	0	0
0	0	0	0	1	0	0	0	0
0	1	0	0	0	0	0	0	0
0	0	0	0	0	0	0	0	0
0	0	0	0	0	0	0	0	0
1	0	0	0	0	0	0	0	0
0	0	0	0	0	0	0	0	0
0	0	0	0	0	0	0	0	0
1	0	0	0	0	0	0	0	0

0	0	0	0	0	0	0	0	0
0	0	0	0	0	0	0	1	0
0	0	0	0	0	0	0	0	0
0	0	0	0	0	0	0	0	0
0	0	0	0	0	0	0	0	0
0	0	0	0	0	0	0	0	0
0	0	0	0	0	0	0	0	0
0	0	0	0	1	0	1	1	0
0	0	0	0	0	0	0	0	0
0	0	0	0	0	0	0	0	0
0	0	0	0	0	0	0	0	0
1	1	0	0	0	0	0	0	0
0	0	0	0	0	0	0	0	0
0	0	0	0	0	0	1	0	0
0	0	0	0	1	0	0	0	0
0	0	0	0	0	0	0	0	1
0	0	0	0	0	0	0	0	0
1	0	0	0	0	0	0	0	0
0	0	0	0	0	0	0	0	0
0	0	0	0	0	0	0	0	0
0	1	0	0	0	0	0	0	0
0	0	0	0	0	0	0	0	0
0	0	0	0	0	0	0	0	0
0	1	0	0	0	0	0	0	0
0	0	0	0	1	0	0	0	0
0	0	0	0	0	0	0	0	0
0	0	0	0	0	0	1	1	0
0	0	0	0	1	0	0	0	0
0	0	0	0	0	0	0	0	0
0	0	0	0	1	0	0	0	0
0	0	0	0	1	0	0	0	0
0	0	0	0	0	0	0	0	0
1	0	0	0	1	0	1	1	0
0	0	0	0	0	0	0	0	0
0	0	0	0	0	0	0	1	0
0	0	0	0	0	0	0	1	0
0	0	0	0	0	0	0	0	0
0	0	0	0	0	0	0	1	0
0	0	0	0	0	0	0	0	0
0	0	0	0	0	0	0	0	0
0	0	0	0	0	0	0	0	0
0	0	0	0	1	0	0	0	1
0	0	0	0	0	0	0	0	0
0	0	0	0	0	0	0	0	0
0	0	0	0	0	0	0	0	0
0	0	0	0	0	0	0	0	0
0	0	0	0	0	0	0	0	0
0	0	0	0	0	0	1	0	1
0	0	0	0	0	0	0	0	0
0	0	0	0	0	0	0	0	0
0	0	1	0	0	0	0	0	0
0	0	0	0	0	0	0	0	0
0	0	0	0	0	0	0	0	0
0	0	0	1	0	0	0	0	0
0	0	0	0	0	0	0	0	0
0	0	0	0	0	0	0	0	0

0	0	0	0	0	0	0	0	0
0	0	0	0	0	0	0	0	0
0	0	0	0	0	0	0	0	0
0	0	0	0	0	0	0	0	0
0	0	0	0	0	0	0	1	0
0	0	0	0	0	0	0	0	0
0	0	0	0	0	0	0	0	0
0	0	0	0	0	0	0	0	0
0	0	0	0	0	0	0	0	0
0	0	0	0	0	0	0	0	0
0	0	0	0	0	0	0	0	0
0	0	0	0	0	0	0	0	0
0	0	0	0	0	0	0	0	0
0	0	0	0	0	0	0	0	0
0	0	0	0	0	0	0	0	0
0	0	0	0	0	0	0	0	0
0	0	0	0	0	0	0	0	0
0	0	0	0	0	0	0	0	0
0	0	0	0	0	0	0	0	0
0	1	0	0	0	0	0	0	0
0	0	0	0	0	0	0	0	0
0	0	0	1	0	0	0	0	0
0	0	0	0	0	0	0	0	0
0	0	0	0	0	0	0	0	0
0	0	0	0	0	0	0	0	0
0	0	0	0	0	0	0	0	0
0	0	0	0	0	0	0	0	0
0	0	0	0	0	0	0	0	0
0	0	0	0	0	0	0	1	0
0	0	0	0	0	0	0	0	0
0	0	0	0	0	0	0	0	0
0	0	0	0	0	0	0	0	0
0	0	0	0	1	0	0	0	0
1	0	0	0	0	0	0	0	0
0	0	0	1	0	0	0	0	1
0	0	0	0	0	0	0	0	0
0	0	1	0	0	0	0	0	0
0	0	0	0	1	0	0	0	0
0	0	0	0	1	0	0	0	0
0	1	0	0	0	0	0	1	0
0	0	0	0	0	0	0	1	0
0	1	0	0	0	0	0	1	0
0	0	0	0	0	0	0	0	0
0	0	0	0	0	0	0	0	0
0	0	0	0	0	0	0	0	0
0	0	0	0	0	0	0	0	0
0	0	0	0	0	0	0	0	0
0	0	0	0	0	0	0	1	0
0	0	0	0	0	0	0	0	0
0	1	1	0	1	0	0	0	0
1	1	0	0	0	0	0	0	0
1	0	0	0	0	0	0	0	0
0	0	0	0	0	0	0	0	1
1	0	0	0	0	0	0	0	0
0	0	0	0	0	0	0	0	0
0	0	0	0	0	0	0	0	0

0	0	0	0	0	0	0	0	0
0	1	0	0	0	0	1	0	0
1	0	0	0	0	0	0	0	0
0	1	0	0	0	0	0	0	0
0	0	0	0	0	0	0	0	0
0	0	0	0	0	0	0	0	0
0	0	0	0	0	0	0	0	0
0	0	0	0	0	0	0	1	0
0	1	0	0	0	0	0	1	0
0	0	0	0	0	0	0	0	0
0	0	0	0	0	0	0	0	0
0	0	0	0	0	0	0	0	0
1	0	0	0	0	0	0	0	0
0	0	0	0	0	0	0	0	0
0	0	0	0	0	0	0	0	0
0	0	0	0	0	0	0	0	0
0	0	0	0	0	0	0	0	0
0	0	0	0	0	0	0	0	0
0	0	0	0	0	0	0	0	0
0	0	0	0	0	0	0	0	0
0	0	0	0	0	0	0	0	0
0	0	0	0	0	0	0	0	0
1	0	0	0	0	0	0	0	1
0	0	0	0	0	0	1	0	0
0	0	0	0	0	0	0	0	0
0	0	0	0	0	0	0	0	0
0	0	0	0	0	0	0	0	0
0	0	0	0	0	0	0	0	0
0	0	1	0	0	0	0	0	0
0	0	0	0	0	0	0	0	0
0	1	1	0	1	0	0	0	0
0	0	0	0	0	0	0	0	0
0	0	0	0	0	0	0	0	0
0	0	0	0	0	0	0	0	0
1	0	0	0	0	0	0	0	0
0	0	0	0	0	0	0	0	0
0	1	0	0	0	0	1	0	0
0	0	1	0	0	0	0	0	0
0	0	0	0	0	0	0	0	0
0	0	0	0	0	0	0	0	0
0	0	0	0	0	0	0	0	0
0	0	0	0	0	0	0	0	0
1	0	0	0	0	0	0	0	0
0	0	0	0	0	0	0	0	0
0	0	0	0	0	0	0	0	0
0	0	0	0	0	0	0	0	0
1	0	0	0	0	0	0	0	0
0	0	0	0	0	0	0	0	0
0	0	0	0	0	0	0	0	0
0	0	0	1	0	0	0	0	1
0	0	0	0	0	0	0	0	0
1	0	0	0	0	0	0	0	0
1	0	0	0	0	0	0	0	0
1	0	0	0	0	0	0	1	0
0	0	0	0	0	0	0	0	0
0	0	0	0	0	0	0	0	0

0	0	0	0	0	0	0	0	0
0	0	0	0	0	0	0	1	0
1	0	0	0	0	0	0	0	0
0	0	0	0	0	0	0	0	0
0	0	0	0	0	0	0	0	0
0	0	0	0	1	0	0	0	0
1	0	0	0	0	0	0	0	0
0	0	0	0	0	0	0	0	0
1	1	0	0	0	0	0	0	0
1	0	0	0	0	0	0	0	0
0	0	0	0	0	0	0	0	0
0	0	0	0	0	0	1	0	0
0	0	0	0	0	0	0	0	0
1	0	0	0	0	0	0	0	0
0	0	0	0	0	0	0	0	0
1	0	0	0	0	0	0	0	0
1	0	0	0	0	0	0	0	0
0	0	0	0	0	0	0	0	0
0	0	0	0	0	0	0	0	0
0	0	1	0	0	0	0	0	0
0	0	0	0	1	0	0	0	0
0	0	0	0	0	0	0	0	0
0	0	0	0	0	0	0	0	0
1	0	0	0	0	0	0	0	0
0	0	1	1	0	0	0	0	0
0	0	0	0	0	0	0	0	0
0	0	0	0	0	0	0	0	0
0	0	0	0	0	0	0	0	0
0	0	0	0	0	0	0	0	0
0	1	0	0	0	0	0	0	0
1	0	0	0	0	0	0	0	0
0	0	0	0	0	0	0	0	0
0	0	0	0	0	0	0	0	0
0	0	0	0	0	0	0	0	0
0	0	0	0	0	0	0	0	1
1	0	0	0	0	0	0	0	0
0	0	0	0	0	0	0	1	0
0	0	0	0	0	0	0	1	0
0	0	0	0	0	0	0	0	0
0	0	0	0	0	0	0	0	0
1	0	0	0	0	0	0	0	0
1	0	0	0	0	0	0	0	0
0	1	0	0	0	0	0	0	0
1	0	0	0	1	0	0	0	0
0	0	0	0	0	0	0	0	0
1	0	0	0	0	0	0	0	0
0	0	0	0	0	0	0	0	0
0	0	0	0	0	0	0	0	0
1	0	0	0	0	0	0	0	0
0	0	0	0	0	0	0	0	0
0	0	0	0	0	0	1	0	0
0	1	0	1	0	0	0	0	0
0	0	0	0	0	0	0	1	0
0	0	0	0	0	0	0	0	0
1	0	0	0	0	0	0	0	0
1	0	0	0	0	0	0	0	0

1	0	0	0	0	0	0	0	0
0	0	0	0	0	0	0	0	0
0	1	0	0	0	0	0	0	0
0	0	0	0	0	0	0	0	0
0	1	0	0	0	0	0	0	0
0	0	0	0	0	0	0	0	0
1	1	0	0	0	0	0	0	0
0	0	0	0	0	0	0	0	0
0	0	0	0	0	0	0	0	0
0	0	0	0	0	0	0	0	0
0	1	0	0	0	0	0	0	0
0	1	0	0	0	0	0	0	0
0	0	0	0	0	0	0	0	0
1	0	0	0	0	0	0	0	0
0	0	0	0	0	0	0	0	0
1	0	0	0	0	0	0	0	0
1	0	0	0	0	0	0	0	0
0	0	0	0	0	0	0	0	0
1	0	0	0	0	0	0	0	0
0	0	0	0	0	0	0	1	0
0	0	0	0	0	0	0	1	0
0	0	0	0	0	0	0	0	0
1	0	0	0	0	0	0	0	0
0	0	0	0	0	0	0	1	0
1	0	0	0	0	0	0	1	0
0	0	0	0	0	0	0	0	0
0	0	0	0	0	0	0	1	0
1	0	0	0	1	0	0	0	0
0	0	0	0	0	0	0	0	0
1	0	0	0	0	0	0	0	0
0	0	0	0	0	0	0	0	0
1	0	0	0	0	0	0	0	0
1	0	0	0	0	0	0	1	0
1	0	0	0	0	0	0	0	0
0	0	0	1	0	0	0	1	1
0	0	0	0	0	0	1	0	1
0	1	0	0	0	0	0	0	0
1	0	0	0	0	0	0	0	0
1	0	0	0	0	0	0	0	0
0	1	0	0	0	0	0	0	0
0	0	0	0	0	0	1	0	0
0	0	0	0	0	0	0	0	0
0	1	0	0	0	0	0	0	0
0	0	0	0	0	0	1	0	0
0	0	0	0	0	0	0	0	0
0	0	0	0	0	0	0	0	0
0	0	0	0	0	0	1	0	0
1	0	0	0	0	0	0	0	0
0	1	0	0	0	0	0	0	0
1	0	0	0	0	0	1	1	0
1	0	0	0	0	0	0	0	0
0	0	0	0	0	0	0	0	0
0	0	0	0	1	1	0	0	0
0	0	0	0	0	0	0	0	0
1	0	0	0	0	0	0	0	0
1	0	0	0	0	0	0	0	0

1	0	0	0	0	0	0	0	0
0	0	0	0	0	0	0	0	0
1	0	0	0	0	0	0	0	0
0	0	0	0	0	0	0	0	0
0	0	0	0	0	0	0	0	0
0	0	0	0	0	0	0	0	0
0	0	0	0	0	0	0	0	0
0	0	0	0	0	0	0	0	0
0	0	0	0	0	0	0	0	0
0	0	0	0	1	0	0	0	0
0	1	0	0	0	0	0	0	0
0	0	0	0	0	0	0	0	1
1	0	0	0	0	0	0	0	0
1	0	0	0	0	0	0	0	0
0	0	0	0	0	0	1	0	0
0	0	0	0	0	0	0	0	0
0	0	0	0	0	0	0	0	0
0	0	0	0	0	0	0	0	0
0	0	0	0	0	0	0	0	0
0	0	0	0	0	0	0	0	0
0	0	0	0	0	0	0	1	0
0	0	0	0	0	0	0	0	0
0	0	0	0	0	0	0	0	0
0	0	0	0	0	0	0	0	0
1	0	0	0	1	0	0	0	0
1	0	0	0	0	0	0	0	0
1	0	0	0	0	0	0	0	0
0	1	0	0	0	1	0	0	0
0	0	0	0	0	0	0	0	0
0	0	0	0	0	0	0	0	0
0	0	0	0	0	0	0	0	0
1	0	0	0	0	0	0	1	0
1	0	0	0	0	0	0	1	0
0	0	0	0	0	0	0	1	0
0	0	0	0	0	0	0	0	0
0	0	0	0	1	0	1	0	0
0	0	0	0	0	0	0	1	0
1	0	0	0	0	0	0	0	0
1	0	0	0	0	0	0	0	0
0	0	0	0	0	0	0	0	0
0	0	0	0	1	0	0	0	0
1	0	0	0	0	0	0	0	0
1	0	0	0	0	0	0	0	0
0	0	0	0	0	0	0	0	0
0	0	0	0	0	0	0	0	0
0	0	0	0	0	0	0	0	0
0	0	0	0	0	0	0	0	0
0	0	0	0	0	0	0	0	0
0	0	0	0	0	0	0	1	0
0	0	1	0	0	0	0	0	0
0	0	0	0	0	0	0	0	0
0	0	0	0	0	0	0	0	0
0	0	0	0	0	0	0	0	0
1	0	0	0	0	0	0	0	0
0	0	0	0	0	0	0	0	0
0	0	0	0	0	0	0	0	0

1	0	0	0	0	0	0	0	0
0	0	0	0	0	0	0	0	0
0	0	0	0	0	0	0	0	0
0	0	0	0	0	0	0	0	0
0	0	0	0	0	0	0	0	0
0	0	0	0	1	1	0	0	0
0	0	0	0	0	0	0	0	0
0	0	0	0	0	0	0	0	0
0	0	0	0	0	0	0	0	0
0	0	0	0	0	0	1	0	0
0	0	0	0	0	0	0	0	0
0	0	0	0	0	0	0	0	0
0	0	0	0	0	0	0	0	0
0	0	0	0	0	0	0	0	0
0	0	0	0	0	0	0	0	0
0	0	0	0	0	0	0	0	0
0	0	0	0	0	0	0	0	0
0	0	0	0	0	0	0	0	0
0	0	0	0	0	0	0	0	0
0	0	0	0	0	0	0	0	0
1	0	0	0	0	0	0	0	0
1	0	1	1	0	0	0	0	0
1	0	0	0	0	0	0	1	0
0	0	0	0	1	0	0	0	0
0	1	0	0	0	0	1	0	0
0	0	0	0	0	0	0	0	0
1	0	0	0	0	0	0	0	0
1	0	0	0	0	0	0	0	0
0	0	0	0	0	0	0	0	0
0	0	0	0	0	0	0	0	0
0	0	0	0	0	0	0	0	0
0	0	0	0	0	0	0	0	0
0	0	0	0	0	0	0	0	0
0	0	0	0	1	0	1	0	0
1	0	0	0	1	0	0	0	0
0	0	0	0	0	0	0	0	0
0	0	0	0	0	0	0	0	0
1	0	0	0	0	0	0	0	0
0	0	0	0	0	0	0	0	0
0	0	0	0	0	0	0	1	0
1	1	0	0	0	0	0	0	0
0	0	0	0	0	0	0	0	0
0	0	0	0	0	0	0	0	0
0	0	0	0	0	0	0	0	0
0	0	0	0	0	0	0	0	0
1	0	0	0	0	0	0	0	0
0	0	0	0	0	0	0	0	0
0	0	0	0	0	0	0	0	0
1	0	0	0	0	0	0	1	0
1	0	0	0	1	0	0	0	0
1	0	0	0	0	0	0	0	0
0	0	0	0	0	0	0	0	0
1	0	0	0	0	0	0	1	0
0	0	0	0	0	0	0	0	0
0	1	0	1	0	0	0	0	0
0	0	0	0	0	0	0	0	0

0	0	0	0	0	0	0	0	0
0	0	0	0	0	0	0	0	0
0	0	0	0	0	0	0	0	0
1	0	1	0	0	0	0	0	0
0	0	0	0	0	0	0	0	0
1	0	0	0	0	0	0	0	0
0	0	0	0	0	0	0	0	0
1	0	0	0	0	0	0	0	0
1	0	0	0	0	0	0	0	0
1	0	0	0	0	0	0	0	0
0	0	0	0	0	0	0	0	0
0	0	0	0	0	0	0	1	0
0	1	0	0	0	0	0	0	0
0	1	0	0	0	0	0	0	0
0	0	0	0	0	0	0	0	0
1	0	0	0	0	0	0	0	0
0	0	0	0	0	0	0	0	0
0	0	0	0	0	0	0	0	0
0	0	0	0	0	0	0	1	0
1	0	0	0	0	0	0	0	0
0	0	0	0	0	0	1	0	0
0	0	0	0	0	0	0	0	0
0	0	0	0	0	0	0	0	0
0	0	0	0	0	0	0	0	0
0	0	0	0	0	0	0	0	0
0	0	0	1	0	0	0	0	0
0	0	0	0	0	0	0	0	0
0	0	0	0	0	0	0	0	0
0	0	0	0	0	0	0	0	0
0	1	0	0	0	0	0	0	0
1	0	0	0	0	0	0	0	0
0	0	0	0	0	0	0	0	0
0	0	0	0	1	0	0	0	0
0	1	0	0	0	0	0	0	0
0	0	0	0	0	0	0	0	0
1	0	0	0	0	0	0	0	0
1	0	0	0	0	0	0	0	0
0	0	0	0	0	0	0	0	0
0	0	0	0	0	0	0	0	0
1	0	0	0	0	0	0	0	0
1	0	0	0	1	0	0	0	0
0	0	0	0	0	0	0	0	0
1	0	0	0	0	0	0	0	0
0	0	0	0	0	0	0	0	0
1	0	0	0	0	0	0	1	0
0	0	0	0	0	0	0	0	0
1	1	0	0	0	0	0	0	0
0	0	0	0	0	0	0	0	0
0	0	0	0	0	0	0	0	0
0	1	1	0	0	0	0	0	0
1	0	0	0	0	0	0	0	0
0	0	0	0	0	0	0	1	0
0	0	0	0	0	0	0	0	0
1	0	0	0	0	0	0	1	0
0	0	0	0	0	0	0	1	0
1	0	0	0	0	0	0	0	0

0	0	0	0	0	0	0	0	0
0	0	0	0	0	0	0	0	0
0	0	0	0	0	0	0	0	0
0	0	0	0	0	0	0	1	0
1	0	0	0	0	0	0	0	0
1	0	0	0	0	0	0	0	0
1	0	0	0	0	0	0	0	0
0	0	0	0	1	0	0	0	0
1	0	0	0	0	1	0	1	0
0	0	0	0	0	0	1	0	0
0	0	0	0	0	0	0	0	0
0	0	0	0	1	0	0	0	0
1	1	0	0	0	0	0	0	0
0	0	0	0	0	0	0	0	0
1	0	0	0	0	0	0	0	1
0	0	0	0	0	0	0	1	0
0	0	0	0	0	0	0	0	0
0	0	0	0	0	0	0	0	0
0	0	0	0	0	0	0	0	0
0	0	0	0	0	0	0	0	0
0	0	0	0	0	0	0	0	0
0	0	0	0	0	0	0	0	0
0	0	0	0	0	0	0	0	0
0	0	0	0	0	0	0	0	0
1	0	0	0	0	0	0	0	0
0	0	0	0	0	0	0	0	0
1	0	0	0	0	0	0	0	0
0	1	0	0	0	0	0	0	0
1	1	0	0	0	0	1	0	0
1	0	0	0	0	0	0	0	0
0	0	0	0	0	0	0	0	0
0	0	0	0	0	0	0	0	0
0	0	0	0	0	0	0	0	0
1	0	0	0	0	0	0	0	0
0	0	0	0	0	0	0	0	0
1	0	0	0	0	0	0	0	0
0	0	0	0	0	0	0	0	0
1	0	0	0	0	0	0	0	0
0	0	0	0	0	0	0	0	0
1	0	0	0	0	0	0	0	1
0	0	0	0	0	0	0	0	0
0	0	0	0	0	0	0	0	0
0	0	0	0	0	0	0	1	0
0	0	0	0	0	0	0	0	0
0	0	0	0	0	0	0	1	0
0	1	0	0	0	0	0	0	0
1	0	0	0	0	0	0	1	1
1	0	0	0	0	0	0	0	0
1	0	0	0	0	0	0	0	0
0	0	0	1	0	0	0	0	0
1	0	0	0	0	0	0	0	0
0	0	1	0	0	0	0	1	0
0	0	0	0	0	0	0	0	0
1	1	0	0	0	0	0	0	0
0	0	0	0	0	0	0	0	0
0	0	0	0	0	0	1	1	1

0	0	0	0	0	0	0	0	0
1	0	0	0	0	0	0	0	0
0	0	0	0	0	0	0	0	0
0	0	0	0	0	0	0	0	0
0	0	0	0	0	0	0	0	0
0	0	0	1	0	0	0	0	0
1	0	0	0	0	0	0	0	0
0	0	0	0	0	0	0	0	0
0	0	0	0	0	0	0	0	0
0	0	0	0	1	0	0	0	0
1	0	0	0	0	0	0	0	0
0	0	0	0	1	0	0	0	0
0	1	0	0	0	0	1	1	0
0	0	0	0	0	0	0	0	0
0	0	0	0	0	0	0	0	0
0	1	0	0	0	0	1	0	0
1	0	0	0	0	0	0	0	0
1	0	0	0	0	0	1	0	0
0	0	0	0	0	0	0	0	0
0	1	0	0	1	0	0	0	0
0	0	0	0	0	0	0	0	0
0	0	0	0	0	0	0	0	0
0	0	0	0	0	0	0	0	0
0	0	0	0	0	0	0	0	0
1	0	0	0	0	0	0	0	0
0	0	0	0	0	0	0	0	0
0	0	0	0	0	0	0	0	1
1	0	0	1	0	0	0	0	0
0	0	0	0	0	0	0	0	0
0	1	0	0	0	0	0	0	0
0	0	0	0	0	0	0	0	0
1	0	0	0	0	0	0	1	0
0	0	0	0	1	0	0	0	0
0	0	0	0	0	0	0	0	0
0	1	0	0	0	0	1	0	0
0	0	0	0	0	0	0	0	0
0	0	0	0	0	0	0	1	0
0	0	0	0	0	0	0	0	0
1	0	0	0	0	0	0	0	0
0	0	0	0	0	0	0	0	0
1	0	0	0	0	0	0	0	0
1	0	0	0	0	0	0	0	0
0	0	1	0	0	0	1	0	0
1	0	0	0	0	0	0	0	0
1	0	0	0	1	0	0	0	0
1	0	0	1	0	0	1	0	0
1	0	0	0	1	1	0	1	0
0	0	0	0	0	0	0	0	0
0	0	0	0	0	0	0	0	0
0	0	0	0	0	0	0	0	0
0	0	0	0	0	0	0	0	0
0	0	0	0	0	0	0	0	0
0	0	0	0	0	0	0	0	0
0	0	0	0	0	0	0	0	0
0	0	0	0	0	0	0	0	0
0	0	0	0	0	0	0	0	0

0	0	0	0	0	0	0	1	0
0	1	0	0	0	0	0	0	0
1	0	0	0	0	0	0	1	0
1	0	0	0	0	0	0	1	0
0	0	0	0	0	0	0	0	0
0	0	0	0	1	0	0	0	0
0	0	0	0	0	0	0	1	0
0	0	0	0	0	0	0	0	0
0	0	0	0	0	0	0	0	0
0	0	0	0	0	0	0	0	0
0	0	0	0	0	0	0	0	1
0	0	0	0	0	0	0	0	0
0	0	0	0	0	0	0	0	0
0	0	0	0	1	0	0	0	0
1	0	0	0	0	0	1	0	0
1	1	0	0	0	0	0	0	0
0	0	0	0	0	0	0	1	0
0	1	0	0	0	0	0	0	1
1	0	0	0	0	0	0	0	0
0	0	0	0	0	0	0	0	0
1	0	0	0	0	0	0	0	0
0	0	0	0	0	0	0	0	0
1	0	0	0	0	0	0	0	0
0	0	0	0	0	0	0	0	0
0	0	0	0	0	0	0	1	0
0	0	0	0	0	0	0	0	0
0	0	0	0	0	0	0	0	0
0	0	0	0	1	0	0	0	1
0	0	0	0	0	0	1	0	0
0	0	0	0	0	0	0	1	0
0	0	0	0	0	0	0	0	0
1	1	0	0	0	0	0	0	0
0	0	0	0	1	0	0	0	0
0	0	0	0	0	0	0	0	0
0	0	0	0	0	0	1	0	0
1	0	0	0	0	0	0	0	0
0	0	0	0	0	0	1	0	0
0	0	0	0	0	0	0	0	0
0	0	0	0	0	0	0	0	0
0	0	0	0	0	0	0	0	0
1	0	0	0	0	0	0	0	0
0	0	0	0	0	0	1	0	0
0	0	0	0	0	0	0	0	0
1	0	0	0	0	0	0	0	0
1	0	0	0	0	0	0	0	0
0	0	0	0	0	0	0	0	0
1	1	0	0	0	0	0	0	0
0	0	0	0	0	0	0	0	0
0	0	0	0	0	0	0	0	0
0	0	0	0	0	0	0	0	0
0	0	0	0	0	0	0	0	0
1	0	0	0	0	0	0	0	0
0	0	0	0	0	0	0	0	0
0	0	0	0	0	0	0	0	0
1	1	0	0	0	0	0	0	0
1	0	0	0	0	0	0	0	0

1	0	0	0	0	0	0	0	0
0	0	0	0	0	0	0	0	0
0	0	0	0	0	0	0	1	0
0	0	0	0	1	0	0	0	0
0	1	0	0	0	0	0	0	0
0	0	0	0	0	0	0	0	0
0	0	0	0	0	0	1	0	0
1	1	0	0	0	0	0	0	0
0	0	0	0	0	0	0	0	0
0	0	0	0	0	0	0	0	0
0	0	0	1	0	0	0	0	0
1	0	0	0	0	0	0	1	0
0	0	0	0	0	0	0	0	0
0	0	0	0	0	0	0	0	0
0	0	0	0	0	0	0	0	1
0	0	0	0	0	0	0	0	0
0	0	0	0	1	1	0	1	0
0	0	0	0	0	0	0	0	0
1	0	0	1	0	0	0	0	0
0	0	0	1	0	0	0	0	0
1	0	0	0	0	0	0	0	0
0	0	0	0	0	0	0	0	0
0	0	0	0	0	0	0	0	0
0	0	0	0	0	0	0	0	0
1	0	0	0	0	0	0	0	0
0	0	0	0	0	0	0	0	0
0	0	0	0	0	0	0	0	0
0	0	0	0	0	0	0	0	1
0	0	0	0	0	0	0	0	0
0	0	0	0	0	0	0	0	0
0	0	0	0	0	0	0	0	0
0	0	0	0	0	0	0	0	0
1	0	0	0	0	0	0	0	0
0	0	0	1	0	0	0	0	0
0	0	0	0	0	0	0	0	0
0	0	0	0	0	0	0	0	0
0	0	0	0	0	0	0	1	0
0	0	0	0	0	0	0	0	0
0	0	0	0	0	0	0	0	0
1	0	0	0	0	0	0	0	0
0	0	0	0	0	0	0	0	0
0	0	0	0	0	0	0	0	0
1	0	0	0	0	0	0	0	0
0	0	0	0	0	0	1	0	0
0	0	0	0	0	0	0	0	0
0	0	0	0	0	0	0	0	0
1	0	0	0	0	0	0	0	0
1	0	0	0	0	0	0	0	0
0	0	0	0	0	0	0	0	0
0	0	0	0	1	0	1	0	0
0	0	0	0	0	0	0	1	0
0	0	0	0	1	0	0	0	0
0	0	0	0	0	0	0	0	0
0	1	0	0	0	0	0	0	1
0	1	0	0	0	0	0	0	0
1	0	0	0	0	0	0	0	0

0	0	0	0	0	0	0	0	0
0	1	0	1	0	0	0	0	0
0	0	0	0	0	0	0	0	0
0	0	0	0	0	0	0	0	0
0	0	0	0	0	0	0	0	0
0	0	0	0	0	0	0	0	0
1	0	0	0	0	0	0	0	0
1	0	0	0	0	0	0	0	0
0	0	0	0	0	0	0	1	0
0	0	0	0	0	0	0	0	0
0	0	0	0	0	0	0	0	0
0	0	0	0	0	0	0	0	0
0	1	0	0	0	0	0	0	0
1	0	0	0	0	0	0	0	0
0	0	0	0	0	0	0	0	0
0	0	0	0	0	0	0	0	0
0	0	0	0	1	0	0	0	0
0	0	0	0	0	0	0	0	0
0	0	0	0	0	0	0	1	0
0	0	0	0	0	0	0	0	0
1	0	0	0	0	0	0	0	0
0	0	0	0	0	0	0	0	0
0	0	0	0	0	0	0	0	0
0	0	0	0	0	0	0	0	0
1	0	0	0	0	0	0	0	0
0	0	0	0	0	0	0	0	0
0	1	0	1	0	0	0	0	0
0	0	0	0	1	0	0	0	0
1	0	0	0	0	0	0	0	0
0	0	0	0	0	0	0	0	0
0	0	0	0	0	0	1	1	0
0	0	0	0	0	0	0	1	0
0	0	0	0	0	0	0	0	0
0	0	0	0	0	0	0	0	0
0	0	0	0	0	0	0	0	0
0	0	0	0	0	0	0	0	0
0	0	0	0	0	0	0	0	0
0	0	0	0	0	0	0	0	0
1	0	0	0	1	0	0	0	0
0	0	0	0	0	0	0	1	0
1	0	0	0	0	0	0	1	0
0	0	0	0	0	0	0	0	0
0	0	0	0	0	0	0	0	0
0	0	0	0	0	0	0	1	0
0	0	0	0	0	0	0	0	0
0	0	0	0	0	0	0	0	0
0	1	0	0	0	0	0	0	0
0	0	0	0	0	0	0	0	0
0	0	0	0	0	0	0	0	0
1	0	0	0	0	0	0	0	0
0	0	0	0	0	0	1	0	0
0	0	0	0	0	0	0	0	1
0	0	0	0	0	0	0	0	0
0	0	0	0	0	0	0	0	0
0	0	1	0	1	0	0	0	0
0	1	0	0	0	0	1	1	1

0	0	0	0	0	0	0	0	0
0	0	0	0	0	0	0	0	0
0	0	0	0	0	0	0	0	0
0	0	0	0	0	0	0	0	0
0	0	0	0	0	0	0	0	0
0	0	0	0	0	0	0	0	0
0	0	0	0	0	0	0	0	0
0	1	0	0	0	0	0	0	0
0	0	0	0	1	0	0	0	0
0	1	0	0	0	0	0	1	0
0	0	0	0	0	0	0	0	0
0	1	0	0	0	0	0	1	0
0	0	0	0	0	0	0	0	0
1	0	0	0	0	0	0	0	0
0	0	0	0	0	0	0	1	0
0	0	0	0	0	0	0	0	0
0	0	0	0	0	0	0	0	0
0	0	0	0	0	0	0	0	0
0	0	0	0	0	0	0	0	0
0	0	0	0	0	0	0	0	0
0	0	0	0	0	0	0	0	0
0	0	0	0	0	0	0	0	0
0	0	0	0	0	0	0	0	0
0	0	0	0	0	0	0	0	0
0	0	0	0	0	0	0	0	0
0	0	0	0	0	0	0	0	0
0	0	0	0	0	0	0	0	0
0	0	0	0	0	0	0	0	0
0	0	0	0	0	0	0	0	0
0	0	0	0	0	0	0	0	0
0	0	0	0	0	0	0	0	0
1	0	0	0	0	0	0	0	1
0	0	0	0	0	0	0	0	0
1	0	0	0	0	0	0	0	0
0	0	0	0	0	0	0	1	0
0	0	0	0	0	0	0	0	0
0	0	0	0	0	0	0	0	0
0	0	0	0	0	0	0	0	0
0	0	0	0	0	0	0	0	0
0	0	0	0	0	0	0	0	0
1	0	0	0	0	0	0	0	0
0	0	0	0	0	0	0	0	0
0	0	0	0	0	0	0	0	0
0	1	0	0	0	0	0	0	0
0	0	0	0	0	0	0	0	0
1	0	0	0	0	0	0	0	0
0	0	0	1	0	0	0	0	0
0	0	0	0	0	0	0	0	0
0	0	0	0	0	0	0	0	0
0	0	0	0	1	0	0	1	0
0	0	0	0	0	0	0	0	0
0	0	0	0	0	0	0	0	0
0	0	0	0	0	0	0	0	0
0	0	0	0	0	0	0	0	0
0	1	0	0	0	0	0	0	0
0	1	0	0	0	0	0	1	0

0	0	0	0	0	0	0	0	0
0	0	0	0	0	0	0	1	0
1	0	1	0	0	0	0	0	0
0	1	0	0	0	0	0	0	0
0	0	0	0	0	0	0	0	0
1	0	0	0	0	0	0	0	0
1	0	0	0	0	0	0	0	0
0	0	0	0	0	0	0	0	0
0	0	0	0	0	0	0	0	0
0	0	0	0	0	0	0	0	0
0	0	0	0	0	0	0	0	0
1	0	0	0	0	0	0	0	0
0	0	0	0	0	0	0	0	0
0	0	0	0	0	0	0	0	0
0	0	1	0	0	0	0	1	0
0	1	0	0	0	0	0	0	0
0	0	0	0	0	0	0	0	0
1	0	0	0	0	0	0	0	0
0	0	0	0	0	0	0	0	0
0	0	0	0	0	0	0	0	0
0	0	0	0	0	0	0	0	0
0	0	0	0	0	0	0	1	0
0	0	0	0	1	0	0	0	0
0	0	0	0	0	0	0	0	0
0	0	0	0	0	0	0	0	0
0	0	0	0	0	0	0	0	0
0	0	0	0	1	0	0	0	0
0	0	0	0	0	0	0	0	0
0	0	0	0	0	0	0	0	0
0	0	1	0	0	0	0	0	0
0	0	0	0	0	0	1	1	0
0	0	0	0	0	0	0	0	0
0	0	0	0	0	0	0	0	0
0	0	0	0	0	0	0	0	0
0	1	0	0	0	0	0	0	0
0	0	0	0	0	0	0	0	0
0	1	0	0	0	0	0	0	0
0	0	0	0	0	0	0	0	0
1	0	1	0	0	0	0	0	0
0	0	0	0	0	0	0	0	0
0	0	0	0	0	0	0	1	0
0	0	0	0	0	0	0	0	0
0	1	0	0	0	0	0	0	1
0	0	0	0	0	0	0	0	0
0	0	0	0	0	0	0	0	0
0	0	0	0	1	0	0	0	0
1	0	0	0	0	0	0	0	0
0	0	0	0	0	0	0	0	0
0	0	0	0	0	0	0	0	0
0	0	0	0	0	0	0	0	0
0	0	0	0	0	0	0	1	0
0	0	0	0	0	0	0	1	0
0	0	0	0	0	0	0	1	0
0	0	0	0	0	0	1	0	0
0	0	0	0	0	0	0	0	0
0	0	0	0	0	0	0	0	0

0	0	0	0	0	0	0	0	0
0	0	0	0	0	0	0	0	0
0	0	0	0	0	0	0	0	0
0	0	0	0	0	0	0	0	0
0	0	0	0	0	0	0	0	0
1	0	0	0	0	0	0	0	1
0	0	0	0	0	0	0	0	0
1	0	0	0	1	0	0	0	0
1	0	0	0	0	0	0	1	0
1	1	0	0	0	0	0	0	0
0	0	0	0	0	0	0	0	0
0	0	0	0	0	0	0	0	0
0	0	0	0	0	0	0	0	0
1	0	0	0	0	0	0	0	0
0	0	0	0	0	0	0	0	0
0	0	0	0	0	0	0	0	0
0	0	0	0	0	0	0	0	0
0	0	0	0	0	0	0	0	0
0	0	0	0	0	0	0	1	0
0	0	0	0	0	0	0	0	0
0	1	0	0	0	0	0	0	0
0	0	0	0	0	0	0	0	0
1	1	0	0	0	0	0	0	0
0	0	0	0	0	0	0	0	0
0	0	0	0	0	0	0	0	0
0	0	0	0	0	0	0	0	0
0	0	0	0	0	0	0	0	0
0	0	0	0	0	0	0	0	0
0	0	0	0	0	0	0	0	0
0	0	0	0	0	0	0	0	0
0	0	0	0	0	0	0	0	0
1	0	0	0	0	0	0	0	0
0	0	0	0	0	0	0	0	0
0	0	0	0	0	0	0	0	0
0	0	0	0	0	0	0	0	0
1	0	0	0	0	0	0	0	0
0	0	0	0	0	0	0	1	0
0	0	0	0	0	0	0	0	0
0	0	0	0	0	0	0	0	0
0	0	0	0	0	0	0	0	0
0	0	0	0	0	0	0	0	0
0	0	0	0	0	0	0	0	0
0	0	0	0	0	0	0	0	0
0	0	0	0	0	0	0	0	0
0	0	0	0	0	0	0	0	0
0	0	0	0	0	0	0	0	0
0	0	0	0	0	0	0	0	0
0	1	1	0	1	0	0	0	0
0	0	0	0	0	0	0	0	0
0	0	0	0	0	0	0	0	0
0	0	0	0	0	0	0	0	0
0	0	0	0	0	0	0	0	0
0	0	0	0	0	0	0	0	0
1	0	0	0	0	0	0	0	0
0	0	0	0	0	0	0	0	0
0	0	0	0	0	0	0	0	0

0	0	0	0	0	0	0	0	0
0	0	0	0	0	0	0	1	0
0	0	0	0	0	0	0	0	0
0	0	0	0	0	0	0	0	0
0	0	0	0	0	0	0	0	0
0	0	0	0	0	0	0	0	0
0	0	0	0	0	0	0	0	0
0	0	0	0	0	0	0	1	0
0	0	0	0	0	0	0	0	0
0	0	0	0	0	0	0	0	0
0	0	0	0	0	0	0	0	0
0	0	0	0	1	0	0	0	0
0	0	0	0	0	0	0	0	0
1	0	0	0	0	0	0	0	0
0	0	0	0	0	0	0	0	0
0	0	0	0	0	0	0	0	0
0	0	0	0	0	0	0	1	0
0	0	0	0	0	0	0	0	0
1	0	0	1	0	0	0	0	0
0	0	0	0	0	0	0	0	0
0	0	0	0	0	0	0	0	0
0	0	0	0	0	0	0	1	0
0	0	0	0	0	0	0	0	0
0	0	0	0	0	0	0	0	0
1	0	0	0	0	0	0	0	0
0	0	0	0	0	0	0	0	0
0	0	0	0	0	0	0	0	0
0	0	0	0	0	0	0	1	0
0	0	0	0	0	0	0	0	0
0	0	0	0	0	0	0	0	0
0	0	0	0	0	0	0	1	0
1	0	0	0	0	0	0	0	0
0	0	0	0	0	0	0	0	0
0	0	0	0	0	0	0	1	0
0	0	0	0	0	0	0	0	0
0	0	0	0	0	0	0	0	0
0	0	0	0	0	0	0	0	0
0	0	0	0	0	0	0	0	0
0	0	0	0	0	0	0	1	0
0	0	0	0	0	0	0	0	0
0	0	0	0	0	0	0	0	0
0	0	1	0	0	0	0	1	0
0	0	0	0	0	0	0	0	0
1	0	0	0	0	0	0	0	0
1	0	0	0	0	0	0	0	0
0	0	0	0	0	0	1	0	0
0	0	0	0	0	0	0	0	0
0	0	0	0	0	0	0	0	0
0	0	0	0	0	0	0	0	0
0	0	0	0	0	0	0	0	0
0	0	0	0	0	0	0	0	0
1	0	0	0	0	0	0	0	0
0	0	0	0	0	0	0	0	0
1	0	0	0	0	0	0	0	0
0	0	0	0	0	0	0	0	0
0	0	0	0	0	0	0	0	0

1	0	0	0	0	0	0	0	0
0	0	0	0	0	0	0	0	0
0	0	0	0	0	0	0	0	0
0	0	0	0	0	0	0	0	0
0	1	0	0	0	0	0	0	0
1	0	0	0	0	0	0	0	0
0	1	0	0	0	0	0	0	0
0	1	0	0	0	0	0	0	0
1	0	0	0	0	0	0	1	0
0	0	0	0	0	0	0	0	0
0	0	0	0	1	0	0	0	0
0	0	0	0	0	0	0	0	0
0	1	0	0	0	0	0	0	0
0	0	0	0	0	0	0	0	0
1	0	0	0	0	0	0	0	0
0	0	0	0	0	0	0	0	0
0	0	0	0	0	0	0	1	0
0	0	0	0	0	0	0	0	0
1	0	0	0	0	0	0	0	1
1	0	0	0	0	0	0	0	0
1	0	0	0	0	0	0	1	0
0	0	0	0	0	0	0	0	0
1	0	0	0	0	0	0	0	0
0	0	0	0	0	0	0	1	0
0	0	0	0	0	0	0	0	0
0	0	0	0	0	0	0	0	0
0	0	0	0	0	0	0	1	0
0	0	0	0	0	0	0	0	1
0	0	0	0	0	0	0	0	0
0	0	0	0	0	0	0	0	0
0	1	0	0	0	0	0	1	0
0	0	0	0	0	0	0	0	0
1	0	0	0	0	0	0	0	0
0	0	0	0	0	0	0	0	0
0	0	0	0	0	0	0	0	0
0	0	0	0	0	0	0	0	0
0	0	0	0	0	0	0	0	0
1	0	0	0	0	0	0	0	0
1	0	0	0	0	0	0	0	0
0	0	0	0	0	0	0	1	0
0	0	0	1	0	0	0	0	0
1	0	0	0	0	0	0	1	0
1	0	0	0	0	0	0	0	0
1	0	0	0	0	0	0	0	0
0	1	0	0	0	0	0	0	0
0	0	0	0	0	1	0	0	0
0	0	0	0	0	0	0	0	0
1	0	0	0	0	0	0	1	0
0	0	0	0	0	0	0	0	0
1	0	0	0	0	0	0	0	0
0	0	0	0	0	0	0	1	0
0	0	0	0	0	0	0	0	0
1	0	0	0	0	0	0	0	0
0	0	0	0	0	1	0	0	0
0	0	0	0	0	0	0	0	0
0	0	0	0	0	0	0	0	0

0	0	0	0	0	0	0	0	0
0	0	0	0	0	0	0	0	0
1	0	0	0	1	0	0	0	0
0	0	0	0	0	0	0	0	0
0	0	0	0	0	1	0	0	0
0	0	0	0	1	0	0	1	0
0	0	0	0	0	0	0	0	0
0	0	0	0	0	0	0	0	0
1	0	0	0	0	1	0	1	0
1	0	0	0	0	1	0	0	0
0	0	0	0	0	0	0	0	0
0	1	0	0	0	0	0	1	0
1	0	0	0	0	0	0	0	0
0	0	0	0	0	0	0	0	0
0	0	0	0	0	0	0	0	0
0	0	0	0	0	0	0	1	0
0	0	0	0	0	0	0	0	0
0	0	0	0	0	0	0	0	0
0	0	0	0	0	0	0	0	0
0	0	0	0	0	0	0	0	0
1	0	0	0	0	0	0	0	0
0	1	0	0	0	0	0	0	0
0	0	0	0	0	0	0	0	0
0	0	0	0	0	0	0	0	0
0	0	0	0	0	0	0	0	0
1	0	0	1	1	0	0	0	0
1	0	0	0	0	0	0	0	0
1	1	0	0	0	0	0	0	0
0	0	0	0	0	0	0	0	0
0	0	0	0	0	0	0	1	0
0	0	0	0	0	0	0	0	0
0	0	0	0	1	0	0	0	0
0	0	0	0	0	0	0	0	0
0	0	0	0	0	0	0	1	0
0	0	0	0	0	0	0	0	0
0	0	0	0	0	0	0	0	0
1	1	0	0	0	0	1	1	0
0	0	0	0	0	0	0	0	0
0	0	0	0	0	0	0	0	0
0	0	0	0	0	0	0	0	0
0	0	0	0	0	0	0	0	0
0	1	0	0	0	0	0	0	0
0	0	0	0	0	0	0	0	0
0	0	0	0	0	0	0	0	0
0	0	0	0	0	0	0	0	0
0	0	0	0	0	0	0	0	0
0	0	0	0	0	0	0	1	0
0	0	0	0	0	0	0	0	0
0	0	0	0	0	0	0	0	0
0	1	0	0	0	0	0	0	0
0	0	0	0	0	0	0	0	0
0	0	0	0	0	0	0	0	0
1	0	0	0	0	0	0	0	0
0	0	0	0	0	0	0	0	0
1	0	0	0	0	0	0	0	0
0	0	0	0	0	0	0	0	0

0	0	0	0	0	0	1	1	0
0	0	0	0	0	0	0	0	0
0	0	0	1	0	0	0	0	0
1	0	0	0	0	0	0	0	0
1	0	0	0	0	0	0	1	0
1	0	0	0	0	0	0	0	0
0	0	0	0	0	0	0	0	0
0	0	0	0	0	0	0	0	0
0	0	0	0	0	0	0	0	0
0	0	0	0	0	0	0	0	0
0	0	0	0	0	0	0	0	0
0	0	0	0	1	0	0	0	0
1	0	0	0	1	1	0	0	0
0	1	0	0	0	0	0	0	0
1	0	0	0	0	0	0	0	0
0	0	0	0	0	0	0	0	0
0	0	0	0	1	0	0	0	0
1	0	0	0	0	0	0	0	0
0	0	0	0	0	0	0	0	0
0	0	0	0	0	0	0	0	0
0	0	0	0	0	0	0	0	0
0	0	0	0	0	0	0	1	0
0	0	0	0	0	0	0	1	0
1	0	0	0	0	0	0	0	0
0	0	0	0	0	0	0	0	0
1	0	0	0	0	0	0	1	0
0	0	0	0	1	0	0	0	0
0	0	0	0	0	0	0	1	0
0	0	1	0	0	0	0	0	0
0	0	0	0	0	0	0	0	0
0	0	0	0	0	0	0	0	0
0	0	0	0	0	0	0	0	0
0	0	0	0	0	0	0	0	0
0	0	0	0	1	0	0	0	0
0	0	0	0	0	0	0	0	0
0	0	0	0	0	0	0	0	0
0	0	0	0	0	0	0	0	0
0	0	0	0	0	0	0	0	0
0	0	0	0	0	0	0	0	0
1	0	0	0	0	0	0	0	0
0	0	0	0	0	0	0	0	0
0	0	0	0	0	0	0	0	0
0	0	0	0	0	0	0	0	0
0	0	0	0	0	0	0	0	0
0	0	0	0	0	0	0	0	0
0	0	0	0	0	0	0	0	0
0	0	0	0	0	0	0	0	0
0	1	0	0	0	0	0	0	0
0	0	0	0	0	0	0	0	0
0	0	0	0	0	0	0	0	0
0	1	0	0	0	0	0	0	0
0	0	0	0	0	0	0	0	0
0	0	0	0	0	0	0	0	0
1	0	0	0	0	0	0	0	0
0	0	0	0	0	0	0	0	0
0	1	0	0	0	0	0	0	0

1	0	0	0	0	0	1	0	0
0	0	0	0	0	0	0	0	0
0	0	0	0	0	1	0	0	0
0	0	0	0	0	0	0	0	0
0	0	0	0	0	0	0	0	0
0	0	0	0	0	0	0	0	0
0	0	0	0	0	0	0	0	0
0	0	0	0	0	0	0	0	0
0	0	0	0	0	0	0	0	0
0	0	0	0	0	0	0	0	0
0	0	0	0	0	0	0	0	0
1	0	0	0	0	0	0	0	0
0	0	0	0	0	0	0	0	0
0	0	0	0	0	0	0	0	0
0	0	0	0	0	0	0	0	0
0	1	0	0	0	0	0	0	0
0	0	0	0	0	0	0	1	0
0	0	0	0	0	0	0	0	0
0	0	0	0	0	0	0	0	0
1	0	0	0	0	0	0	0	0
1	0	0	0	0	0	0	0	0
0	0	0	0	0	0	0	0	0
0	0	0	0	0	0	0	0	0
0	0	0	0	0	0	0	0	0
0	0	0	0	0	0	0	0	0
0	0	0	0	0	0	0	0	0
0	0	0	0	0	0	0	0	0
1	0	0	0	0	0	0	0	0
0	0	0	0	0	0	0	0	0
0	0	0	0	0	1	0	0	0
1	0	0	0	0	0	0	0	0
1	0	0	0	0	0	0	0	0
0	0	0	0	0	0	0	0	0
1	0	0	0	0	0	0	0	0
0	0	0	0	0	0	0	0	0
1	0	0	0	0	0	0	0	0
0	0	0	0	0	0	0	0	0
0	0	0	0	0	0	0	0	0
0	0	0	0	0	0	0	0	0
0	0	0	0	0	0	0	0	0
1	0	0	0	0	0	0	0	0
1	0	0	0	0	0	0	0	0
0	1	0	0	0	0	0	0	0
0	0	0	0	0	0	0	0	0
0	0	0	1	0	0	0	1	0
0	0	0	0	0	0	0	0	0
0	0	0	0	0	0	0	0	0
0	0	0	0	0	0	0	0	0
0	0	0	0	0	0	0	0	0
0	0	0	0	0	0	0	0	0
0	1	0	0	0	0	0	0	0
0	0	0	0	0	0	0	0	0
1	0	0	0	0	0	0	0	0
0	0	0	0	0	0	0	0	0
0	0	0	0	0	0	0	0	0
0	0	0	0	1	0	0	0	0

0	0	0	0	0	0	0	0	0
1	0	0	0	0	0	0	0	0
1	0	0	0	0	0	0	0	0
0	0	0	0	0	0	0	0	0
1	0	0	0	0	0	0	0	0
1	0	0	0	0	0	0	0	0
0	0	0	0	0	0	0	0	0
0	0	0	0	0	0	0	0	0
0	0	0	0	0	0	0	1	0
1	0	0	0	0	0	0	0	0
1	0	0	0	0	0	0	0	0
1	0	0	0	0	0	0	0	0
1	0	0	0	0	0	0	0	0
0	0	0	0	0	0	0	1	0
0	0	0	0	0	0	0	0	0
1	0	0	0	0	0	0	0	0
0	0	0	0	0	0	0	0	0
0	0	0	0	0	0	0	0	0
1	0	0	0	0	0	0	1	0
0	0	0	0	1	0	0	0	0
1	0	0	0	0	0	0	0	0
1	0	0	0	0	0	0	0	0
0	0	0	0	0	0	0	0	0
0	0	0	0	0	0	0	0	0
0	0	0	0	0	0	0	0	0
0	0	0	0	0	0	0	0	0
0	0	0	0	0	0	0	0	0
0	0	0	0	0	0	0	0	0
0	1	0	0	0	0	0	1	0
1	0	0	0	0	0	0	0	0
1	0	0	0	0	0	0	0	0
1	0	0	0	0	0	0	0	0
1	0	0	0	0	0	0	0	0
0	0	0	0	0	0	0	0	0
0	0	0	0	0	0	0	0	0
1	0	0	0	0	0	0	0	0
0	0	0	0	0	0	0	1	0
0	0	0	0	0	0	0	0	0
0	0	0	0	0	0	0	1	1
0	0	0	0	0	0	0	0	0
0	0	0	0	0	0	0	0	0
1	0	0	0	0	0	0	0	0
1	0	0	0	0	0	0	1	0
0	0	0	0	0	0	0	0	0
1	0	0	0	0	0	0	0	0
0	0	0	0	0	0	0	0	0
0	0	0	0	0	0	0	0	0
1	0	0	0	0	0	0	0	0
1	0	0	0	0	0	0	0	0
0	0	0	1	0	0	0	0	0
0	0	0	0	0	0	0	0	0
1	0	0	0	0	0	0	0	0
1	0	0	0	0	0	0	0	0
1	0	0	0	0	0	0	0	0
0	0	0	0	0	0	0	0	0
1	0	0	0	0	0	0	0	0

0	0	0	0	0	0	0	1	0
1	1	1	0	0	0	0	0	0
1	0	0	0	0	0	0	0	0
1	0	0	0	0	0	0	0	0
1	0	0	0	0	0	0	0	0
1	0	0	0	0	0	0	0	0
1	0	0	0	0	0	0	0	0
1	0	1	0	0	0	0	0	0
0	0	0	0	0	0	0	1	0
0	0	0	0	0	0	0	0	0
0	0	0	0	0	0	0	0	0
0	0	0	0	1	0	0	0	0
0	0	0	0	0	0	0	0	0
1	0	0	0	0	0	0	0	0
0	0	0	0	0	0	0	0	0
1	0	0	0	0	0	0	0	0
0	0	0	0	0	0	0	0	0
0	0	0	0	0	0	0	0	0
1	0	0	0	0	0	0	0	0
1	0	0	0	0	0	0	0	0
1	0	0	0	0	0	0	1	0
0	0	0	0	0	0	0	0	0
1	0	0	0	0	0	0	0	0
0	0	0	0	0	0	0	0	0
0	0	0	0	0	0	0	0	0
0	0	0	0	0	0	0	0	0
0	0	0	0	0	0	0	0	0
0	0	0	0	0	0	0	0	0
1	0	0	0	0	0	0	0	0
1	0	0	0	0	0	0	0	0
0	0	0	0	0	0	0	0	0
1	0	0	0	0	0	0	0	0
0	0	0	0	0	0	0	0	0
0	0	0	0	0	0	0	0	0
1	0	0	0	0	0	0	0	0
0	0	0	0	0	0	0	0	0
1	0	0	0	0	0	0	0	0
1	0	0	0	0	0	0	0	0
0	0	0	0	0	0	0	0	0
1	0	0	0	0	0	0	0	0
1	0	0	0	0	0	0	0	0
0	1	0	0	0	0	0	0	0
0	0	0	0	0	0	0	0	0
0	0	0	0	0	0	0	0	0
0	0	0	0	0	0	0	0	0
1	0	0	0	0	0	0	0	0
1	0	0	0	1	0	0	0	0
1	0	0	0	0	0	1	0	0
0	0	0	0	0	0	1	0	0
1	0	0	0	0	0	0	0	0
1	0	0	0	0	0	0	0	0
0	0	0	0	0	0	0	0	0
0	0	0	0	0	0	0	0	0
1	0	0	0	0	0	0	0	0
0	1	0	0	0	0	0	0	0
1	0	0	0	0	0	0	0	0

0	0	0	0	0	0	1	1	0
0	0	0	0	0	0	0	1	0
0	0	0	0	0	0	0	1	0
0	0	0	0	0	0	0	0	0
0	0	0	0	0	0	1	1	0
0	0	0	0	0	0	0	0	0
1	0	0	0	0	0	0	0	0
1	0	0	0	1	0	0	0	0
0	0	0	0	0	0	0	0	0
0	0	0	0	0	0	0	0	0
1	0	0	0	0	0	0	0	0
0	0	1	0	0	0	0	0	1
0	0	0	0	0	0	0	0	0
0	0	0	0	0	0	0	0	0
1	1	0	0	0	0	0	0	0
0	0	0	0	0	0	0	0	0
1	1	0	0	0	0	0	0	0
1	0	0	0	0	0	0	0	0
0	0	0	0	0	0	0	0	0
0	0	0	0	0	0	0	0	0
1	0	0	0	0	0	0	0	0
0	0	0	0	0	0	0	1	0
0	0	0	1	0	0	0	0	0
0	0	0	0	0	0	0	0	0
0	0	0	0	0	0	1	0	0
0	0	0	0	0	0	0	0	0
1	0	0	0	1	0	0	0	0
1	0	0	0	1	0	0	0	0
0	0	0	0	0	0	0	0	0
1	0	0	0	0	0	0	0	0
1	0	0	0	0	0	0	0	0
1	0	0	0	0	0	0	0	0
0	0	0	0	0	0	1	0	0
1	0	0	0	0	0	0	0	0
0	0	0	0	0	0	0	0	0
1	0	0	0	0	0	0	0	0
0	1	0	0	0	0	0	0	0
0	0	0	0	0	0	0	0	1
0	1	0	0	0	0	0	0	0
1	0	0	0	0	0	0	0	0
1	0	0	0	0	0	0	0	0
0	0	0	0	0	0	0	1	0
0	0	0	0	0	0	0	0	0
1	0	0	0	0	0	0	0	0
1	0	0	0	0	0	0	0	0
1	0	0	0	0	0	0	1	0
0	1	1	0	0	0	0	0	0
1	0	0	0	0	0	0	0	0
0	0	0	0	0	0	0	0	0
0	0	0	0	0	0	0	0	0
0	0	0	0	0	0	0	0	0
1	0	0	0	1	0	0	0	0
0	0	0	0	0	0	1	0	0
0	0	0	0	0	0	0	0	0
0	0	0	0	0	0	0	0	0
0	0	0	0	0	0	0	0	0

0	0	0	0	0	0	0	0	0
0	0	0	0	0	0	0	0	0
1	0	0	0	0	0	0	0	0
0	1	0	1	0	0	0	0	0
1	0	0	0	0	0	0	0	0
1	0	0	0	0	0	0	0	0
1	0	0	0	0	0	0	0	0
0	0	0	0	0	0	0	0	0
0	0	0	0	0	0	0	0	0
1	0	0	0	1	0	0	0	0
0	0	0	0	0	0	0	0	0
0	0	0	0	0	0	0	0	0
0	0	0	0	0	0	0	1	0
0	0	0	0	0	0	0	0	0
1	0	0	0	1	0	0	0	0
1	0	0	0	0	0	0	0	0
0	0	0	0	0	0	0	0	0
0	0	0	0	0	0	0	1	1
0	0	0	0	0	0	0	0	0
1	0	0	0	0	0	0	0	0
0	0	0	0	0	0	0	0	0
0	0	0	0	0	0	0	0	0
1	0	0	0	0	0	0	0	0
1	0	0	0	0	0	0	0	0
1	0	0	0	0	0	0	0	0
0	0	0	0	0	0	0	0	0
0	0	0	0	0	0	0	0	0
1	0	0	0	0	0	1	0	0
0	0	0	0	0	0	0	0	0
1	0	0	0	0	0	0	0	0
1	0	0	0	0	0	0	0	0
1	0	0	0	0	0	0	1	0
0	0	0	0	0	0	0	0	0
1	0	0	0	0	0	0	0	0
1	0	0	0	0	0	0	0	0
0	0	0	0	0	0	1	0	0
1	0	0	0	0	0	0	0	0
1	0	0	0	1	0	0	0	0
0	0	0	0	0	0	0	0	0
1	0	0	0	0	0	0	0	0
0	0	0	0	0	0	0	0	0
1	0	1	0	0	0	0	0	0
0	0	0	0	0	0	0	0	0
0	0	0	0	0	0	0	1	0
1	0	0	0	0	0	0	0	0
1	0	0	0	1	0	0	0	0
0	0	0	0	0	0	0	0	0
1	0	0	0	0	0	0	0	0
1	0	0	0	0	0	0	0	0
1	0	0	0	1	0	0	0	0
0	0	0	0	0	0	0	0	0
1	0	0	0	0	0	0	0	0
1	0	0	0	0	0	0	0	0
1	0	0	0	1	0	0	0	0
0	0	0	0	1	0	0	0	0
0	0	0	0	0	0	0	0	0

1	1	0	0	0	0	0	0	0
1	0	0	0	0	0	0	0	0
0	0	0	0	0	0	0	1	0
0	0	0	0	0	0	0	0	0
0	0	0	0	0	0	0	0	0
1	0	0	0	0	0	0	0	0
1	0	0	0	0	0	0	0	0
1	0	0	0	0	0	0	0	0
0	0	0	0	0	0	0	0	0
0	0	0	0	0	1	0	0	0
1	0	0	0	0	0	0	0	0
0	0	0	0	0	0	0	0	0
1	1	0	0	0	0	0	0	0
0	0	0	0	0	0	0	0	0
0	1	0	0	0	0	0	0	0
0	0	0	0	0	0	0	0	0
1	0	0	0	0	0	0	0	0
0	0	0	0	0	0	0	0	0
1	0	0	0	0	0	0	0	0
1	0	0	0	0	0	0	0	0
0	0	0	0	0	0	0	0	0
0	0	0	0	0	0	0	0	0
0	0	0	0	0	0	0	0	0
0	0	0	0	0	0	0	0	0
0	0	1	0	0	0	0	0	0
1	0	0	0	0	0	0	0	0
0	0	0	0	0	0	0	0	0
0	0	0	0	0	0	0	0	0
0	0	0	0	0	0	0	1	0
0	0	0	0	0	0	0	0	0
1	0	0	0	0	0	0	0	0
0	0	0	0	0	0	0	1	0
1	0	0	0	0	0	0	0	0
1	0	0	0	0	0	0	0	0
0	0	0	0	0	0	1	0	0
1	0	0	0	0	0	0	0	0
0	0	0	0	0	0	0	1	0
1	0	0	0	1	0	0	0	0
0	0	0	0	0	0	0	0	0
0	0	0	0	0	0	0	0	0
1	0	0	0	0	0	0	0	0
0	0	0	0	0	0	0	0	0
1	0	0	0	0	0	0	0	0
0	0	0	0	0	0	0	0	0
1	0	0	0	0	0	0	0	0
1	0	0	0	0	0	0	0	0
1	0	0	0	0	0	0	0	0
1	0	0	0	0	0	0	0	0
0	0	0	0	1	0	0	0	0
0	0	0	0	0	0	0	0	0
0	1	0	0	0	0	1	0	0
1	0	0	0	0	0	0	0	0
1	0	0	0	0	0	0	0	0
0	0	0	0	0	0	0	0	0
0	0	0	0	0	0	0	1	0
0	0	0	0	0	0	0	0	0

0	0	0	0	0	0	0	1	0
0	0	0	0	0	0	0	0	0
0	0	0	0	0	0	0	1	0
0	0	0	0	0	0	0	0	0
0	0	0	0	0	0	0	0	0
1	0	0	0	0	0	0	0	0
0	0	0	0	0	0	0	0	0
0	0	0	0	0	0	0	0	0
1	0	0	0	0	0	0	0	0
1	0	0	0	0	0	0	0	0
0	0	0	0	0	0	0	0	0
0	0	0	0	0	0	0	0	0
1	0	0	0	0	0	0	0	0
0	0	0	0	0	0	0	0	0
1	0	0	0	0	0	0	0	0
1	0	0	0	0	0	0	1	0
0	0	0	0	0	0	0	0	0
0	0	0	0	0	0	0	0	0
0	0	0	0	0	0	0	0	0
0	0	0	0	0	0	0	0	0
1	0	0	0	1	0	0	0	0
0	0	0	0	0	0	0	1	0
0	0	0	0	0	0	0	0	0
0	0	0	0	0	0	0	1	0
0	0	0	0	0	0	0	0	0
1	0	0	0	0	0	0	0	0
0	0	0	0	0	0	0	0	0
0	0	0	0	0	0	1	1	0
0	0	0	0	0	0	0	0	0
1	0	0	0	1	0	0	0	0
1	0	0	0	0	0	0	0	0
0	0	0	0	0	0	0	0	0
0	1	0	0	0	0	0	0	0
1	0	0	0	0	0	0	0	0
0	0	0	0	0	0	0	0	0
0	0	0	0	0	0	1	1	0
0	0	0	0	0	0	0	1	0
1	0	0	0	0	0	0	0	0
1	0	0	0	0	0	0	0	0
0	0	0	0	0	0	0	0	0
0	0	0	0	0	0	0	0	0
0	0	0	0	0	0	0	0	0
0	0	0	0	0	0	0	0	0
0	0	0	0	0	0	0	0	0
0	0	0	0	0	0	0	0	0
0	0	0	0	0	0	0	1	0
1	0	0	0	0	0	0	0	0
0	0	0	0	0	0	0	1	0
0	0	0	0	0	0	0	0	0
1	0	0	0	0	0	0	0	0
0	0	0	0	0	0	0	0	0
1	0	0	0	0	0	0	0	0
0	0	0	0	0	0	0	0	0
0	0	0	0	0	0	0	0	0

coagulatio	hematolog	history of r	surgical his	severe der	pulmonary	valvulopath	neurologic	psychiatric
0	0	1	0	0	0	0	0	0
0	0	0	0	0	0	0	0	0
0	0	1	0	0	0	0	0	0
0	0	0	0	0	0	0	0	0
0	0	1	0	0	0	0	0	0
0	0	0	0	0	0	0	0	0
0	0	0	0	0	0	0	0	0
0	0	0	0	0	0	0	0	0
0	0	0	0	0	0	0	0	0
0	0	0	0	0	0	0	0	0
0	0	0	0	0	0	0	0	0
0	0	0	0	0	0	0	0	0
0	0	1	0	0	0	0	0	0
0	0	0	0	0	0	1	0	0
0	0	1	1	0	0	0	0	0
0	0	0	0	0	0	0	0	0
0	0	0	0	0	0	0	0	0
0	0	0	0	0	0	0	0	0
0	0	0	0	0	0	0	0	0
0	0	1	0	0	0	0	0	0
0	0	0	0	0	0	0	0	0
0	0	0	0	0	0	0	0	0
0	0	0	0	0	0	0	0	0
0	0	0	0	0	0	0	0	0
0	0	0	0	0	0	0	0	0
0	0	0	0	0	0	0	0	0
0	0	0	0	0	0	0	0	0
0	0	0	0	0	0	0	0	0
0	0	0	0	0	0	0	0	0
0	0	1	0	0	0	0	0	0
0	0	0	0	0	0	0	0	0
0	0	0	0	0	0	0	0	0
0	0	0	0	0	0	0	0	0
0	0	0	0	0	0	0	0	0
0	0	0	0	0	0	0	0	0
0	0	0	0	0	0	0	0	0
0	0	0	0	0	0	0	0	0
0	0	0	0	0	0	0	0	0
0	0	1	0	0	0	0	0	0
0	0	0	0	0	0	0	0	0
0	0	0	0	0	0	0	0	0
0	0	0	0	0	0	0	0	0
0	0	0	0	0	0	0	0	1
0	0	0	0	0	0	0	0	0
0	0	0	0	0	0	0	0	0
0	0	0	0	0	0	0	0	0
1	0	1	0	0	0	0	0	0
0	0	0	0	0	0	0	0	0
0	0	0	0	0	0	0	0	0
0	0	0	0	0	0	0	0	0
0	0	0	0	0	0	0	0	0
0	0	1	0	0	0	0	0	0
0	0	1	0	0	0	0	0	0
0	0	0	0	0	0	0	0	0
0	0	1	1	0	0	0	0	0

0	0	0	0	0	0	0	0	0
0	0	0	0	0	0	0	0	0
0	0	1	0	0	0	0	0	0
0	0	1	0	0	0	0	0	0
0	0	1	0	0	0	0	0	0
0	0	0	0	0	0	0	0	0
1	0	0	0	0	0	0	0	1
0	0	0	0	0	0	0	0	0
0	0	0	0	0	0	0	0	0
1	0	0	0	0	0	0	0	0
0	0	0	0	0	0	0	1	0
0	0	0	0	0	0	0	0	0
0	0	0	0	0	0	0	0	0
0	0	0	0	0	0	0	0	1
0	0	0	0	0	0	0	0	0
0	0	0	0	0	0	0	0	0
0	0	1	0	0	0	0	0	0
0	0	0	0	0	0	0	0	0
0	0	0	0	0	0	0	0	0
0	0	0	0	0	0	0	0	0
0	0	0	0	0	0	0	0	0
0	0	1	0	0	0	0	0	0
0	0	0	0	0	0	0	0	0
0	0	0	0	0	0	0	0	0
0	0	0	0	0	0	0	0	0
0	0	0	0	0	0	0	0	0
0	0	0	0	0	0	0	0	0
0	0	0	0	0	0	0	0	0
0	0	0	0	0	0	0	0	0
0	0	1	0	0	0	0	0	0
0	0	0	0	0	0	0	0	0
0	0	0	0	0	0	0	0	0
0	0	0	0	0	0	0	0	0
0	0	1	0	0	0	0	0	0
0	0	0	0	0	0	0	0	0
0	0	0	0	0	0	0	0	0
0	0	1	0	0	0	0	1	0
0	0	1	0	0	0	0	0	0
0	0	0	0	0	0	0	0	0
0	0	0	0	0	0	0	0	0
0	0	0	0	0	0	0	0	0
0	0	0	0	0	0	0	0	0
0	0	0	0	0	0	0	0	1
0	0	0	0	0	0	0	0	0
1	0	0	0	0	0	0	0	0
0	0	0	0	0	0	0	0	0
0	0	1	0	1	0	0	0	1
0	0	1	0	0	0	0	0	0
0	0	1	0	0	0	0	0	0
0	0	0	0	0	0	0	0	0
0	0	1	0	0	0	0	0	0
1	0	0	0	0	0	0	0	0
0	0	0	0	0	0	0	0	1
0	0	1	0	0	0	0	0	0
0	0	0	0	0	0	0	0	0
1	0	0	0	0	0	0	0	0

0	0	1	0	0	0	0	0	0
0	0	1	0	0	0	0	0	0
1	0	0	0	0	0	0	0	0
0	0	0	0	0	0	0	0	0
1	0	1	0	0	0	0	0	0
0	0	0	0	0	0	0	0	0
0	0	0	0	0	0	0	0	0
0	0	0	0	1	0	0	0	0
0	0	0	0	0	0	0	0	1
1	0	0	0	0	0	0	0	0
0	0	0	0	0	0	0	0	0
0	0	0	0	0	0	0	0	0
0	0	1	0	0	0	0	0	0
0	0	0	0	0	0	0	0	0
0	0	1	0	0	0	0	0	0
0	0	0	0	0	0	0	0	0
0	0	0	0	0	0	0	0	0
0	0	0	0	0	0	0	0	0
0	0	0	1	0	0	0	0	0
0	0	1	0	0	0	0	0	0
0	0	0	0	0	0	0	0	0
0	0	1	0	0	0	0	0	0
0	0	0	0	0	0	0	0	0
0	0	1	0	0	0	0	0	0
0	0	0	0	0	0	0	0	0
0	0	0	0	0	0	0	0	0
0	0	0	0	0	0	0	0	0
0	0	0	0	0	0	0	0	0
0	0	0	0	0	0	0	0	0
0	0	0	0	0	0	0	0	0
1	0	0	0	0	0	0	0	0
0	0	0	0	0	0	0	0	0
0	0	0	0	0	0	0	0	0
0	0	1	0	0	0	0	0	0
0	0	0	0	0	0	0	0	0
0	0	0	0	0	0	0	0	0
0	0	0	0	0	0	0	0	0
0	0	0	0	0	0	0	0	0
0	0	0	0	0	0	0	0	0
1	0	1	0	0	0	0	0	0
0	0	0	0	0	0	0	0	0
0	0	1	0	0	0	0	0	0
0	0	0	0	0	0	0	0	0
0	0	0	0	0	0	0	0	0
0	0	1	0	0	0	0	0	0
0	0	0	0	0	0	0	0	0
0	0	0	0	0	0	0	0	0
0	0	0	0	0	0	0	0	0
0	0	0	0	0	0	0	0	0
0	0	1	0	0	0	0	0	0
0	0	0	0	0	0	0	0	0
0	0	0	0	0	0	0	0	0
1	0	0	0	0	0	0	0	0
0	0	0	0	0	0	0	0	0
1	0	0	0	0	0	0	0	0
0	0	1	0	0	1	0	0	0

0	0	0	0	0	0	0	0	0
0	0	0	1	0	0	0	0	0
0	0	0	0	0	0	0	0	0
0	0	0	0	0	0	0	0	0
0	0	1	0	0	0	0	0	0
0	0	1	0	0	0	0	0	0
0	0	0	0	0	0	0	0	0
0	0	0	1	0	0	0	0	0
0	0	0	0	0	0	0	0	1
0	0	0	0	0	0	0	0	0
0	0	0	0	0	0	0	0	0
0	0	0	0	0	0	0	0	0
0	0	0	0	0	0	0	0	0
0	0	1	1	0	0	0	0	0
0	0	1	0	0	0	0	0	0
0	0	0	0	0	0	0	0	0
0	0	0	0	0	0	0	0	0
0	0	0	0	0	0	0	0	0
0	0	0	0	0	0	0	0	0
0	0	1	0	0	0	0	0	0
0	0	1	0	0	0	0	0	0
0	0	0	0	0	0	0	0	0
0	0	0	0	0	0	0	0	1
0	0	0	0	0	0	0	0	0
0	0	0	0	0	0	0	0	0
0	0	0	0	0	0	0	0	0
0	0	0	0	0	0	0	0	0
0	0	0	0	0	0	0	0	0
0	0	1	0	0	0	0	0	0
0	0	0	0	0	0	0	0	0
0	0	0	0	0	0	0	0	0
0	0	1	0	0	0	0	0	0
0	0	0	0	0	0	0	0	0
0	0	1	0	0	0	0	0	0
0	0	0	0	0	0	0	0	0
0	0	1	1	0	0	0	0	0
0	0	0	0	0	0	0	0	0
0	0	1	0	0	0	0	0	0
0	0	0	0	0	0	0	0	0
0	0	0	1	0	1	0	0	0
0	0	0	0	0	0	0	0	0
0	0	0	0	0	0	0	0	0
0	0	1	0	0	0	0	0	0
0	0	0	0	0	0	0	0	0
0	0	1	0	0	0	0	0	0
0	0	0	0	0	0	0	0	0
0	0	0	0	0	0	0	0	0
0	0	1	0	0	0	0	0	0
0	0	0	0	0	0	0	0	0
0	0	0	0	0	0	0	0	0
0	0	0	1	0	0	0	0	0
0	0	0	0	0	0	0	0	0
0	0	1	0	0	0	0	0	0
0	0	0	0	0	0	0	0	0
0	0	0	0	0	0	0	0	0
0	0	1	0	0	0	0	0	0

0	0	0	0	0	0	0	0	0
0	1	0	0	0	0	0	0	0
0	0	1	0	0	0	0	0	1
0	0	0	0	0	0	0	0	0
0	0	0	0	0	0	0	0	0
0	0	0	0	0	0	0	0	0
0	0	1	0	0	0	0	0	0
0	0	0	0	0	0	0	0	0
0	0	1	0	0	0	0	0	0
0	0	0	0	0	0	0	0	0
0	0	0	0	0	0	0	0	0
0	0	0	0	0	0	0	0	0
0	0	1	0	0	0	0	0	0
0	0	0	0	0	0	0	0	0
0	0	1	0	0	0	0	0	0
0	0	0	0	0	0	0	0	0
0	0	0	0	0	0	0	0	0
0	0	1	0	0	0	0	0	0
0	0	0	0	0	0	0	0	0
0	0	0	0	0	0	0	0	0
0	0	0	0	0	0	0	0	0
0	0	0	0	0	0	0	0	0
0	0	0	0	0	0	0	0	0
0	0	0	0	0	0	0	0	0
0	0	0	0	0	0	0	0	0
0	0	1	0	0	0	0	0	0
0	0	1	0	0	0	0	0	0
0	0	0	0	0	0	0	0	0
0	0	0	0	0	0	0	0	0
0	0	1	1	0	0	0	0	0
0	0	1	0	0	0	0	0	0
0	0	0	0	0	0	0	0	0
0	0	0	0	0	0	0	0	0
0	0	1	0	0	0	0	0	0
0	0	1	0	0	0	0	0	0
0	0	1	0	0	0	0	0	0
0	0	0	0	0	0	0	0	0
0	0	1	0	0	0	0	0	0
0	0	0	0	0	0	0	0	0
0	0	0	0	0	0	0	0	0
0	0	1	0	0	0	0	0	0
0	0	0	0	0	0	0	0	1
0	0	1	0	0	0	0	0	0
0	0	0	0	0	0	0	0	0
0	0	0	0	0	0	0	0	0
0	0	1	1	0	0	0	0	0
1	0	0	0	0	0	0	0	0
0	0	1	0	0	0	0	0	0
0	0	0	0	0	0	0	0	0
0	0	0	0	0	0	0	0	0
0	0	0	0	0	0	0	0	0
0	0	1	1	0	0	0	0	0
0	0	0	0	0	0	0	0	0
0	0	0	0	0	0	0	0	0
0	0	0	0	0	0	0	0	0
0	0	1	0	0	0	0	0	0

0	0	0	0	0	0	0	0	0
0	0	0	1	0	0	0	0	0
0	0	0	0	0	0	0	0	0
0	0	1	0	0	0	0	0	0
0	0	0	0	0	0	0	0	0
0	0	1	1	0	0	0	0	0
0	0	0	0	0	0	0	0	0
0	0	1	0	0	0	0	0	0
0	0	0	0	0	0	0	0	0
0	0	0	0	0	0	0	0	0
0	0	0	0	0	0	0	0	0
0	0	0	0	0	0	0	0	0
0	0	0	1	0	0	0	0	0
1	0	0	0	0	0	0	0	0
0	0	0	0	0	0	0	0	0
0	0	1	0	0	0	0	0	0
1	0	1	0	0	0	0	0	0
0	0	0	0	0	0	0	0	0
0	0	0	0	0	0	0	0	0
0	0	0	0	0	0	0	0	0
0	0	1	0	0	0	0	0	0
0	0	1	1	0	0	1	0	0
0	0	1	0	0	0	0	0	0
0	0	0	0	0	0	0	0	0
0	0	1	1	1	0	0	0	0
1	0	1	0	0	0	0	0	0
0	0	0	0	0	0	0	0	0
0	0	0	0	0	0	0	0	0
0	0	1	1	0	0	0	0	0
0	0	0	1	0	0	0	0	0
0	0	0	0	0	0	0	0	0
0	0	0	0	0	0	0	0	0
0	0	0	1	0	0	0	0	0
0	0	0	0	0	0	0	0	0
0	0	0	1	0	0	0	0	0
0	0	0	0	0	0	0	0	0
0	0	0	0	0	0	0	0	0
0	0	0	0	0	0	0	0	0
0	0	0	0	0	0	0	0	0
0	0	1	0	0	0	0	0	0
0	0	1	0	0	0	1	0	0
0	0	0	0	0	0	1	0	0
0	0	0	0	0	0	0	0	0
0	0	0	0	0	0	0	0	0
0	0	1	0	0	0	0	0	0
0	0	0	1	0	0	0	0	0
0	0	1	0	0	0	0	0	0
0	0	0	0	0	0	0	0	0
0	0	0	0	0	0	0	0	0
0	0	1	0	0	0	0	0	0
0	0	0	0	0	0	0	0	0
0	0	1	0	0	0	0	0	0
0	0	0	0	0	0	0	0	0
0	0	0	0	0	0	0	0	0
0	0	0	0	0	0	0	0	0
0	0	1	0	0	0	0	0	0

0	0	0	0	0	0	0	0	0
0	0	0	0	0	0	0	0	0
0	0	0	0	0	0	0	0	0
0	0	0	0	0	0	0	0	0
0	0	1	0	0	0	0	0	0
0	0	1	0	0	0	0	0	0
0	0	0	0	0	0	0	0	0
0	0	0	0	0	0	0	0	0
0	0	0	0	0	0	0	0	0
0	0	0	0	0	0	0	0	0
0	0	0	0	0	0	0	0	0
0	0	0	0	0	0	0	0	0
0	0	1	0	0	0	0	0	0
0	0	1	0	0	0	0	0	0
0	0	0	0	0	0	0	0	0
0	0	0	0	0	0	0	0	0
0	0	0	0	0	0	0	0	0
0	0	0	0	0	0	0	0	0
0	0	0	0	0	0	0	1	0
0	0	1	0	0	0	0	0	0
0	0	1	0	0	0	0	0	0
0	0	0	1	0	0	0	0	0
0	0	0	1	0	0	0	0	0
0	0	0	0	0	0	0	0	0
0	0	0	0	0	0	0	0	0
0	0	0	0	0	0	0	0	0
0	0	1	0	0	0	0	0	0
0	0	1	0	0	0	0	0	0
0	0	1	0	0	1	0	0	0
0	0	0	0	0	0	0	0	0
0	0	1	0	0	0	0	0	0
0	0	0	0	0	0	0	0	0
0	0	0	0	0	0	0	0	1
0	0	0	0	0	0	0	1	0
0	0	0	0	0	0	0	0	0
0	0	1	0	0	0	0	0	0
0	0	0	0	0	0	0	0	0
0	0	0	0	0	0	0	0	0
0	0	1	0	0	0	0	0	0
0	0	0	0	0	0	0	0	0
0	0	0	0	0	0	0	0	0
0	0	0	0	0	0	0	0	0
0	0	0	0	0	0	0	0	0
0	0	0	0	0	0	0	0	0
0	0	1	0	0	0	0	0	0
0	0	0	0	0	0	0	0	0
0	0	0	0	0	0	0	0	0
0	0	0	0	0	0	0	0	0
0	0	1	0	0	0	0	0	0
0	0	0	0	0	0	0	0	0
0	0	0	0	0	0	0	0	0
0	0	0	0	0	0	0	0	0
0	0	0	0	0	0	0	0	0
0	0	1	0	0	0	0	0	0
0	0	0	0	0	0	0	0	0
0	0	0	0	0	0	0	0	0

0	0	1	0	0	0	0	0	0
0	0	1	0	0	0	0	0	0
0	0	0	0	0	0	0	0	0
0	0	1	0	0	0	0	0	0
0	1	1	0	0	0	0	0	0
0	0	1	1	0	0	0	0	0
0	0	1	0	0	0	0	0	0
0	0	0	0	0	0	0	0	0
0	0	1	0	0	0	0	0	0
0	0	0	0	0	0	0	0	0
0	0	0	0	0	0	0	0	1
0	0	0	0	0	0	0	0	0
0	0	0	0	0	0	0	0	0
0	0	0	0	0	0	0	0	0
0	0	1	0	0	0	0	0	0
0	0	0	0	0	0	0	0	0
0	0	1	0	0	0	0	0	0
0	0	0	0	0	0	0	0	0
0	0	1	0	0	0	0	0	0
0	0	1	0	0	0	0	0	1
0	0	0	0	0	0	0	0	0
0	0	1	0	0	0	0	0	0
0	0	1	0	0	0	0	0	0
0	0	1	0	0	0	0	1	1
0	0	0	0	0	1	0	0	0
0	0	0	0	0	0	0	0	0
0	0	1	0	0	0	0	0	0
0	0	0	0	0	0	0	0	0
0	0	0	0	0	1	0	0	0
0	0	0	0	0	0	0	0	0
0	0	0	0	0	0	0	0	0
0	0	1	0	0	0	0	0	0
0	0	1	0	0	0	0	0	0
0	0	1	0	0	0	0	0	0
0	0	0	0	0	0	0	0	0
0	0	0	0	0	0	0	0	0
0	0	1	0	0	0	0	0	0
0	0	0	0	0	0	0	0	0
0	0	0	1	0	0	0	0	0
0	0	1	0	0	0	0	0	0
0	0	0	0	0	0	0	0	0
0	0	0	0	0	0	0	0	0
0	0	0	0	0	0	0	0	0
0	0	0	0	0	0	0	0	0
0	0	0	0	0	0	0	0	0
0	0	1	0	0	0	0	0	0
0	0	0	0	0	0	0	0	0
0	0	0	0	0	0	0	0	0
0	0	0	0	0	0	0	0	0
0	1	0	0	0	0	0	0	0
0	0	0	1	0	0	0	0	0
0	0	1	0	0	0	0	0	0
0	0	0	0	0	0	0	0	0
0	0	0	0	0	0	0	0	0
0	0	1	0	0	0	1	0	0
0	0	0	0	0	0	0	0	0

0	0	1	0	0	0	0	0	0
0	0	1	0	0	0	0	0	0
0	0	0	0	0	0	0	0	0
0	0	0	0	0	0	0	0	1
0	0	0	0	0	0	0	0	0
0	0	0	0	0	0	0	0	0
0	0	0	0	0	0	0	0	0
0	0	1	0	0	0	0	0	0
0	0	0	0	0	0	0	0	0
0	0	0	0	0	0	0	0	0
0	0	0	0	0	0	0	0	0
0	0	0	0	0	0	0	0	0
0	0	0	0	0	0	0	0	1
0	0	0	0	0	0	0	0	0
0	0	0	0	0	0	0	0	0
0	0	0	0	0	0	0	0	0
0	0	0	0	0	0	0	0	0
0	0	0	0	0	0	0	0	0
0	0	0	1	0	0	0	0	0
0	0	0	0	0	0	0	0	0
0	0	1	0	0	0	0	0	0
0	0	1	0	0	0	0	0	0
0	0	0	0	0	0	0	0	0
0	0	0	0	0	0	0	0	0
0	0	0	0	0	0	0	0	0
0	0	1	0	0	0	0	0	0
0	0	1	0	0	0	0	0	0
0	0	0	0	0	0	0	0	0
0	0	0	0	0	0	0	0	0
0	0	0	0	0	0	0	0	0
0	0	0	0	0	0	0	0	1
0	0	0	0	0	0	0	0	0
0	0	0	0	0	0	0	0	0
0	0	1	0	0	0	0	0	0
0	0	1	0	0	0	0	0	0
0	0	0	0	0	0	0	0	0
0	0	0	0	0	0	0	0	0
0	0	1	0	0	0	0	0	1
0	0	0	0	0	0	0	0	0
0	0	1	0	0	0	0	0	0
0	0	1	0	0	0	0	0	0
0	0	1	0	0	0	0	0	0
0	0	0	0	0	0	1	0	0
0	0	0	0	0	0	0	0	0
0	0	0	0	0	0	0	0	0
0	0	1	0	0	0	0	0	0
0	0	0	0	0	0	0	0	0
0	0	0	0	0	0	0	0	0
0	0	1	0	0	0	0	0	0
0	0	1	0	0	0	0	0	0
0	0	0	0	0	0	0	0	0
0	0	0	0	0	0	0	0	0
0	0	0	0	0	0	0	0	0
0	0	0	0	0	0	0	0	0
0	0	0	0	0	0	0	0	0
0	0	1	0	0	0	0	0	0

0	0	0	0	0	0	0	0	0
0	0	0	0	0	0	0	0	0
0	0	0	0	0	0	0	0	0
0	0	0	0	0	0	0	0	0
0	0	1	0	0	0	0	0	0
0	0	0	0	0	0	0	0	0
0	1	0	0	0	0	0	0	0
0	0	0	0	0	0	0	0	0
0	0	0	1	0	0	0	0	0
0	0	0	0	0	0	0	0	0
0	0	0	0	0	0	0	0	0
0	0	0	0	0	0	0	0	0
0	0	0	0	0	0	0	0	1
0	0	0	0	0	0	0	0	0
0	0	0	0	0	0	0	0	0
0	0	0	0	0	1	0	0	0
0	0	0	0	0	1	0	0	0
0	0	0	0	0	0	0	0	0
0	0	0	0	0	0	0	0	0
0	0	1	0	0	0	0	0	0
0	0	0	0	0	0	0	0	1
0	0	0	0	0	0	0	0	0
0	0	1	0	0	0	0	0	0
0	0	0	0	0	0	0	0	0
0	0	0	0	0	0	0	1	0
0	0	0	0	0	0	0	0	1
0	0	0	0	0	0	0	0	0
0	0	0	0	0	0	0	0	0
0	0	1	0	0	0	0	0	0
0	0	0	0	0	0	0	0	0
0	0	0	0	0	0	0	0	0
0	0	0	0	0	0	0	0	0
0	0	0	0	0	0	0	0	0
0	1	0	0	0	0	0	0	0
0	0	0	0	0	0	0	0	0
0	0	0	0	0	0	0	0	0
0	0	1	0	0	0	0	0	0
0	0	0	0	0	0	0	0	0
0	0	1	0	0	0	0	0	0
0	0	0	0	0	0	0	0	0
0	0	1	0	0	0	0	0	0
0	0	0	0	0	0	0	0	0
0	0	0	0	0	0	0	0	0
0	0	0	0	0	0	0	0	0
0	0	1	0	0	0	0	0	0
0	0	0	0	0	0	0	0	0
0	0	1	0	0	0	0	0	0
0	0	0	0	0	0	0	0	0
0	0	0	0	0	0	0	0	0
0	0	1	0	0	0	0	0	0
0	0	0	0	0	0	0	0	0
0	0	0	0	0	0	0	0	0
0	0	0	0	0	0	0	0	0
0	0	0	0	0	0	0	0	0
0	0	0	0	0	0	0	0	0
0	0	0	0	0	0	0	0	0

0	0	0	0	0	0	0	0	0
0	0	0	0	0	0	0	0	0
0	0	0	0	0	0	0	0	0
0	0	1	0	0	0	0	0	0
0	0	0	0	0	0	0	0	1
0	0	0	0	0	0	0	0	0
0	0	0	0	0	0	0	0	0
0	0	1	0	0	0	0	0	0
0	0	0	0	0	0	0	0	0
0	0	1	0	0	0	0	0	0
0	0	0	1	0	0	0	0	0
0	0	0	0	0	0	0	0	0
0	0	1	1	0	0	0	0	0
0	0	1	0	0	1	0	0	0
0	0	1	0	0	0	0	0	0
0	0	1	0	0	0	0	0	0
0	0	0	0	0	0	0	0	0
0	0	1	0	0	0	0	0	0
0	0	0	0	0	0	0	0	0
0	0	0	0	0	0	0	0	0
0	0	0	0	0	0	0	0	0
0	0	0	0	0	0	0	0	0
0	0	0	0	0	0	0	0	0
0	0	0	0	0	0	0	0	0
0	0	0	0	0	0	0	0	0
0	0	0	0	0	0	0	0	0
0	0	0	0	0	0	0	0	0
0	0	0	0	0	0	0	0	0
0	0	0	0	0	0	0	0	0
0	0	0	0	0	0	0	0	0
0	0	0	0	0	0	0	0	0
0	0	1	0	0	0	0	0	0
0	0	0	0	0	0	0	0	0
0	0	0	0	0	0	0	0	0
0	0	0	1	0	0	0	0	0
0	0	0	0	0	0	0	0	0
0	0	0	0	0	0	0	0	0
0	0	0	0	0	0	0	0	0
0	0	0	0	0	0	0	0	1
0	0	0	0	0	0	0	0	0
0	0	0	0	0	0	0	0	0
0	0	0	0	0	0	0	0	0
0	0	0	0	0	0	0	0	0
0	0	0	0	0	0	0	0	0
0	0	0	0	0	0	0	0	0
0	0	0	0	0	0	0	0	0
0	0	0	0	0	0	0	0	0
0	0	0	0	0	0	0	0	1
0	0	0	0	0	0	0	0	0
0	0	0	0	0	0	0	0	0
0	0	0	0	0	0	0	0	0
0	0	1	0	0	0	0	0	0
0	0	0	0	0	0	0	0	0
0	0	1	0	0	0	0	0	0
0	0	0	0	0	0	0	0	0
1	0	0	0	0	0	0	0	0

0	0	0	0	0	0	0	0	0
0	0	0	0	0	0	0	0	0
0	0	0	0	0	0	0	0	1
0	0	1	0	0	0	0	0	0
0	0	0	0	0	0	0	0	0
0	0	0	0	0	0	0	0	0
0	0	0	0	0	0	0	0	0
0	0	0	0	0	0	0	0	0
0	0	1	0	0	0	0	0	0
0	0	0	0	0	0	0	0	0
0	0	1	0	0	0	0	0	0
0	0	0	0	0	0	0	0	0
0	0	1	1	0	0	0	0	0
0	0	0	0	0	0	0	0	0
0	0	0	0	0	0	0	0	0
0	0	0	0	0	0	0	0	0
0	0	0	0	0	0	0	0	0
0	0	0	0	0	0	0	0	0
0	0	0	0	0	0	0	0	0
1	0	0	0	0	0	0	0	0
0	0	0	0	0	0	0	0	0
0	0	0	0	0	0	0	0	0
0	0	1	0	0	0	0	0	0
0	0	0	0	0	0	0	0	0
0	0	1	0	0	0	0	0	0
0	0	1	0	0	0	0	0	0
0	0	1	0	0	0	0	0	0
0	0	0	0	0	0	0	0	0
0	0	0	0	0	0	0	0	0
0	0	0	0	0	0	0	0	0
0	0	0	0	0	0	0	0	0
0	0	0	0	0	0	0	0	0
0	0	0	0	0	0	0	0	0
0	0	0	0	0	0	0	0	0
0	0	0	0	0	0	0	0	0
0	0	1	0	0	0	0	0	0
0	0	0	0	0	0	0	0	0
0	0	0	0	0	0	0	0	0
0	0	0	0	0	0	0	0	0
0	0	1	0	0	0	0	0	0
0	0	0	0	0	0	0	0	0
0	0	0	0	0	0	0	0	0
0	0	0	0	0	0	0	0	0
0	0	1	0	0	0	0	0	0
0	0	0	0	0	0	0	0	0
0	0	1	0	0	0	0	0	0
0	0	0	0	0	0	0	0	0
0	0	0	0	0	0	0	0	0
1	0	0	0	0	0	0	0	0
0	0	0	0	0	0	0	0	0
0	0	0	0	0	0	0	0	0
0	0	0	0	0	0	0	0	0
0	0	0	0	0	0	0	0	0
0	0	0	0	0	0	0	0	0
0	0	0	0	0	0	0	0	0
0	0	1	0	0	0	0	0	0

0	0	0	0	0	0	0	0	0
0	0	0	0	0	0	0	0	0
0	0	0	0	0	0	0	0	0
0	0	1	0	0	0	0	0	0
0	0	0	0	0	0	0	0	0
0	0	1	0	0	0	0	0	0
0	0	0	0	0	0	0	0	0
0	0	0	0	0	0	0	0	0
0	0	0	0	0	0	0	0	0
0	0	0	0	0	0	0	0	0
0	0	0	0	0	0	0	0	0
0	0	0	0	0	0	0	0	0
0	0	1	0	0	0	0	0	0
0	0	0	0	0	0	0	0	0
0	0	0	0	0	0	0	0	0
0	0	1	0	0	0	0	0	0
0	0	1	0	0	0	0	0	0
0	0	0	0	0	0	0	0	0
0	0	0	0	0	0	0	0	0
0	0	0	0	0	0	0	0	0
0	0	0	0	0	0	0	0	0
0	0	0	0	0	0	0	0	0
0	0	0	0	0	0	0	0	1
0	0	0	0	0	0	0	0	0
0	0	0	0	0	0	0	0	0
0	0	0	0	0	0	0	0	0
0	0	0	0	0	0	0	0	0
0	0	0	0	0	0	0	0	0
0	0	0	0	0	0	0	0	0
0	0	0	0	0	0	0	0	0
0	0	0	0	0	0	0	0	0
0	0	0	0	0	0	0	0	0
0	0	1	1	0	0	0	0	0
0	0	0	0	0	0	0	0	0
0	0	1	0	0	0	0	1	0
0	0	1	0	0	0	0	0	0
0	0	0	0	0	0	0	0	0
0	0	0	0	0	0	0	0	0
0	0	0	0	0	0	0	0	0
0	0	0	0	0	0	0	0	0
0	0	0	0	0	0	0	0	1
0	0	1	0	0	0	0	0	0
0	0	0	0	0	0	0	0	0
0	0	0	0	0	0	0	0	0
0	0	0	0	0	0	0	0	0
0	0	0	1	0	0	0	0	0
0	0	0	1	0	0	0	0	0
0	0	0	0	0	0	0	0	0
0	0	1	0	0	0	0	0	0
0	0	0	0	0	0	0	0	0
0	0	0	0	0	0	0	0	0
0	0	1	0	0	0	0	0	0
0	0	0	0	0	0	0	0	0
0	0	0	0	0	0	0	0	0
0	0	1	0	0	0	0	0	0
1	0	0	0	0	0	0	0	0

0	0	0	0	0	0	0	0	0
0	0	0	0	0	0	0	0	0
0	0	0	0	0	0	0	0	0
0	0	1	0	0	0	0	0	0
0	0	0	0	0	0	0	0	0
0	0	0	0	0	0	0	0	0
0	0	0	0	0	0	0	0	0
0	0	0	0	0	0	0	0	0
0	0	0	0	0	0	0	0	0
0	0	1	0	0	0	0	0	0
0	0	0	1	0	0	0	0	0
0	0	0	0	0	0	0	0	0
0	0	0	0	0	0	0	0	0
0	0	1	0	0	0	0	0	0
0	0	0	0	0	0	0	0	1
0	0	0	0	0	0	0	0	0
0	0	0	0	0	0	0	0	0
0	0	0	0	0	0	0	0	0
0	0	0	0	0	0	0	0	0
0	0	0	0	0	0	0	0	0
0	0	0	0	0	0	0	0	0
0	0	0	0	0	0	0	0	0
0	0	0	0	0	0	0	0	0
0	0	0	0	0	0	0	0	0
0	0	0	0	0	0	0	0	0
0	0	0	0	0	0	0	0	0
0	0	1	0	0	0	0	0	0
0	0	0	0	0	0	0	0	0
0	0	0	0	0	0	0	0	0
0	0	1	0	0	0	0	0	0
0	0	0	0	0	0	0	0	0
0	0	1	1	0	0	0	0	0
0	0	0	0	0	0	0	0	0
0	0	0	1	0	0	0	0	0
0	0	0	0	0	0	0	0	0
0	0	0	0	0	0	0	0	0
0	0	1	0	0	0	0	0	0
1	0	0	0	0	0	0	0	0
0	0	0	0	0	0	0	0	0
0	0	0	0	0	0	0	0	0
0	0	0	0	0	0	0	0	0
0	0	1	0	0	0	0	0	0
0	0	1	0	0	0	0	0	0
0	0	0	1	0	0	0	0	0
0	0	0	0	0	0	0	0	0
0	0	0	0	0	0	0	0	0
0	0	0	0	0	0	0	0	0
0	0	0	0	0	0	0	0	0
0	0	0	0	0	0	0	0	0
0	0	0	0	0	0	0	0	0
0	0	1	0	0	0	0	0	0
0	0	1	0	0	0	0	0	0
0	0	1	0	0	0	0	0	0
0	0	0	0	0	0	0	0	0
0	0	1	0	0	0	0	0	0
0	0	1	0	0	0	0	0	0

0	0	1	0	0	0	0	0	0
0	0	0	0	0	0	0	0	0
0	0	0	0	0	0	0	0	0
0	0	0	0	0	0	0	0	0
0	0	0	0	0	0	0	0	0
0	0	0	0	0	0	0	0	0
0	0	0	0	0	0	0	0	0
0	0	0	0	0	0	0	0	0
0	0	0	0	0	0	0	0	0
0	0	0	0	0	0	0	0	0
0	0	0	0	0	0	0	0	0
0	0	0	0	0	0	0	0	0
0	0	1	1	0	0	0	0	0
0	0	0	0	0	0	0	0	0
0	0	0	0	0	0	0	0	0
0	0	1	0	0	0	0	0	0
0	0	0	0	0	0	0	0	0
0	0	0	0	0	0	0	0	0
0	0	0	0	0	0	0	0	0
0	0	1	0	0	0	0	0	0
0	0	1	0	0	0	0	0	0
0	0	1	0	0	0	0	0	0
0	0	1	0	0	0	0	0	0
0	0	0	0	0	0	0	0	0
0	1	0	0	0	0	0	0	0
0	0	0	0	0	0	0	0	0
0	0	0	0	0	0	0	0	0
0	0	1	0	0	0	0	0	0
0	0	0	0	0	0	0	0	0
0	0	1	0	0	0	0	0	0
0	0	1	0	0	0	0	0	0
0	0	0	0	0	0	0	0	0
0	0	1	0	0	0	0	0	0
0	0	0	0	0	0	0	0	0
0	0	0	0	0	0	0	0	0
0	0	0	0	0	0	0	0	0
0	0	0	0	0	0	0	0	0
0	0	0	0	0	0	0	0	0
0	0	1	0	0	0	0	0	0
0	0	0	0	0	0	0	0	0
0	0	1	0	1	0	0	0	0
0	0	1	0	0	0	0	0	0
0	0	0	0	0	0	0	0	0
0	1	0	0	0	0	0	0	0
0	0	1	0	0	0	0	0	0
0	0	0	0	0	0	0	0	0
0	0	1	0	0	0	0	0	0
0	0	0	0	0	0	0	0	0
0	0	0	0	0	0	1	0	0
0	0	0	0	0	0	0	0	0
0	0	0	0	0	0	0	0	0
0	0	0	1	0	0	0	0	0
0	0	0	1	0	1	0	0	0
0	1	0	0	0	0	0	0	0
0	0	0	1	0	0	0	0	0
0	0	0	0	0	0	0	0	0

0	0	1	1	0	0	0	0	0
0	0	1	0	0	0	0	0	0
0	0	0	0	0	0	1	0	0
0	0	0	0	0	0	0	0	0
0	1	0	0	0	0	0	0	0
0	0	0	0	0	0	0	0	0
0	0	0	0	0	0	0	0	0
0	0	0	0	0	0	0	0	0
0	0	1	0	0	0	0	0	0
0	0	0	0	0	0	0	0	0
0	0	0	0	0	0	0	0	0
0	0	0	0	0	0	0	0	0
0	0	1	0	0	0	0	0	1
0	0	1	0	0	0	0	0	0
0	0	0	0	0	0	0	0	0
0	0	0	0	0	0	0	0	0
0	0	0	0	0	0	0	0	0
0	0	0	0	0	0	0	0	0
0	0	0	0	0	0	0	0	0
0	0	0	0	0	0	0	0	0
0	0	0	0	0	0	0	0	0
0	0	0	0	0	0	0	0	0
0	0	0	0	0	0	0	0	0
0	0	0	0	0	0	0	0	0
0	0	1	0	0	0	0	0	0
0	0	0	0	0	0	0	0	0
0	0	1	0	0	0	0	0	0
0	0	0	0	0	0	0	0	0
0	0	0	0	0	0	0	1	0
0	0	0	0	0	0	0	0	0
0	0	1	1	0	0	0	0	0
0	0	0	0	0	0	0	0	0
0	0	1	0	0	0	0	0	0
0	0	0	0	0	0	0	0	0
0	0	0	0	0	0	0	0	0
0	0	0	0	0	0	0	0	0
0	0	1	0	0	0	0	0	0
0	0	0	1	0	0	0	0	0
0	0	0	0	0	0	0	0	0
0	0	0	0	0	0	0	0	0
0	0	1	0	0	0	0	0	0
1	0	0	0	0	0	0	0	0
0	0	0	0	0	0	0	0	0
0	0	0	0	0	0	0	0	0
0	0	0	0	0	0	0	0	0
0	0	0	0	0	0	0	0	0
0	0	1	0	0	0	0	0	0
0	0	0	0	0	0	0	0	0
0	0	0	0	0	0	0	0	0
0	0	0	0	0	0	0	0	0
0	0	1	0	0	0	0	0	0
0	0	0	0	0	0	0	0	0
0	0	0	0	0	0	0	0	0
0	0	1	0	0	0	0	0	0
0	0	0	0	0	0	0	0	0
0	0	0	0	0	0	0	0	0

0	0	0	0	0	0	0	0	0
0	0	0	0	0	0	0	0	0
0	0	1	0	0	0	0	0	0
1	0	0	0	0	1	0	0	0
0	1	0	0	0	0	0	0	0
0	0	0	0	0	0	0	0	0
0	0	0	0	0	0	0	0	0
0	0	0	0	0	0	0	0	0
0	0	1	0	0	0	0	0	0
0	0	0	0	0	0	0	0	0
0	0	0	0	0	0	0	0	0
0	0	1	0	0	0	0	0	0
0	0	0	0	0	0	0	0	0
0	0	0	0	0	0	0	0	0
0	0	0	0	0	0	0	0	0
0	0	0	0	0	0	0	0	0
0	0	1	0	0	0	0	0	0
1	0	0	0	0	0	0	0	0
0	0	0	0	0	0	0	0	0
1	0	0	1	0	0	0	0	0
0	0	0	0	1	0	0	0	0
0	0	0	0	0	0	0	0	0
1	0	0	0	0	0	0	0	0
0	0	0	0	0	0	0	0	0
1	0	0	0	0	0	0	0	0
1	0	1	0	0	0	1	0	0
1	0	0	0	0	0	1	0	0
1	0	0	0	0	0	0	0	0
0	0	0	0	0	0	0	0	0
1	0	0	0	0	0	0	0	0
0	0	1	0	0	0	0	0	0
1	0	1	0	0	0	0	0	0
0	0	1	0	0	0	0	0	0
0	0	1	0	0	0	0	0	0
1	0	0	0	0	0	0	0	1
0	0	0	1	0	0	0	0	0
0	0	0	0	0	0	0	0	0
0	0	1	0	0	0	0	0	0
1	0	0	0	0	0	0	0	0
0	0	1	1	0	0	0	0	0
0	1	0	0	0	0	0	0	0
1	0	0	0	1	0	0	0	0
1	0	0	0	0	0	0	1	0
0	0	1	0	0	0	0	0	0
0	0	0	0	0	0	0	0	0
0	0	1	0	0	0	0	1	0
0	0	0	0	0	0	0	0	0
0	0	1	1	0	0	0	0	0
1	0	1	0	0	0	0	0	1
0	0	0	1	0	0	0	0	1
0	0	1	0	0	0	0	0	0
0	0	0	0	0	0	0	0	0
1	0	0	0	0	0	0	0	0
0	0	0	0	0	0	0	0	0
1	0	0	0	0	0	0	0	0
0	0	0	0	0	0	0	0	0

1	0	0	0	0	0	0	0	0
1	0	0	0	0	0	0	0	0
0	0	0	0	0	0	0	0	0
1	0	1	0	0	0	0	0	0
1	0	0	0	0	0	0	0	0
0	0	0	0	0	0	0	0	0
0	0	0	0	0	0	0	0	0
1	0	0	0	0	0	0	0	0
0	0	0	0	0	0	0	0	0
0	0	1	0	0	0	0	0	0
0	0	1	0	0	0	0	0	0
0	0	0	0	0	0	0	0	0
0	0	1	0	0	0	0	0	0
0	0	0	0	0	0	0	0	0
1	0	0	0	0	0	0	0	0
0	0	0	0	0	0	0	0	0
0	0	1	0	0	0	0	0	1
0	0	0	0	0	0	0	0	0
0	0	0	0	0	0	0	0	0
0	0	1	0	0	0	0	0	0
0	0	0	0	0	0	0	0	0
0	0	0	0	0	0	0	0	0
0	0	0	0	0	0	0	0	0
0	0	0	0	0	0	0	0	1
0	0	0	0	0	0	0	0	0
1	0	1	0	0	0	0	0	0
1	0	0	0	0	0	0	0	0
1	0	1	0	0	0	0	0	0
0	0	1	0	0	0	0	0	0
0	0	0	0	0	0	0	0	0
0	0	0	0	0	0	0	0	1
0	0	1	0	0	0	0	0	0
1	0	0	0	0	0	0	0	0
0	0	0	0	0	0	1	0	0
0	0	1	0	0	0	0	0	0
1	0	0	0	0	0	0	0	0
0	0	0	0	0	0	0	0	1
0	0	0	0	0	0	0	0	0
1	0	1	0	0	0	1	0	0
0	0	0	0	0	0	0	0	0
1	0	0	0	0	0	1	0	0
0	0	0	0	0	0	0	0	0
1	0	1	1	0	0	0	0	0
0	0	1	0	0	0	0	0	0
0	0	0	1	0	0	0	0	0
0	0	0	0	0	0	0	0	0
0	0	0	0	0	0	0	0	0
0	0	0	0	0	0	0	0	0
1	1	0	0	0	0	0	0	0
1	0	0	0	0	0	0	0	0
0	0	1	0	0	0	0	0	0
0	0	0	0	0	0	0	0	0
0	0	0	0	0	0	0	0	0
0	0	1	0	0	0	0	0	0
1	0	0	0	0	0	0	0	0
0	0	0	0	0	0	0	0	1

0	0	0	0	0	0	0	0	0
0	0	0	0	0	0	0	0	0
0	0	1	0	0	0	0	0	0
0	0	0	0	0	0	0	0	0
0	0	0	0	0	0	0	0	0
0	0	0	0	0	0	0	0	1
0	0	1	0	0	0	0	0	0
0	0	0	0	0	0	0	0	0
1	0	1	0	0	0	0	0	0
0	0	0	0	0	0	0	0	0
0	0	0	0	0	0	0	0	0
0	0	0	0	0	0	0	0	0
0	0	0	0	0	0	0	0	0
0	0	0	0	0	0	0	0	0
0	0	0	0	0	0	0	0	0
0	0	0	0	0	0	0	0	0
0	0	0	0	0	0	0	0	0
0	0	1	0	0	0	0	0	0
0	0	1	0	0	0	0	0	0
0	0	0	0	0	0	0	0	0
0	0	0	0	0	0	0	0	0
0	0	0	0	0	0	0	0	0
0	0	0	0	0	0	0	0	0
0	0	0	0	0	0	0	0	0
0	0	0	0	0	0	0	0	0
0	0	1	0	0	0	0	0	0
0	0	0	0	0	0	0	0	0
0	0	0	0	0	1	0	0	0
0	0	0	0	0	0	0	0	0
0	0	1	0	0	0	0	0	0
0	0	1	0	0	0	0	0	0
0	0	0	0	0	0	0	0	0
0	0	0	0	0	0	0	0	0
0	0	0	0	0	0	0	0	0
0	0	0	0	0	0	0	0	0
0	0	1	0	0	0	0	0	0
0	0	1	0	0	0	0	0	0
0	0	0	0	0	0	0	0	0
0	0	1	0	0	0	0	0	0
0	0	0	0	0	0	0	0	0
0	0	0	0	0	0	0	1	0
0	0	0	0	0	0	0	0	0
0	0	0	0	0	0	0	0	0
0	0	0	0	0	0	0	0	0
0	0	0	0	0	0	0	0	0
0	0	1	0	0	0	0	0	0
0	0	0	0	0	0	0	0	0
0	0	0	0	0	0	0	0	0
0	0	1	0	0	0	0	0	0
0	0	0	0	0	0	0	0	0
0	0	0	0	0	0	0	0	0
0	0	0	0	0	0	0	0	0
0	0	0	0	0	0	0	0	0
0	0	0	0	0	0	0	0	0
0	0	0	0	0	0	0	0	0
0	0	0	0	0	0	0	0	0

0	0	0	0	0	0	0	0	0
0	0	1	0	0	1	0	0	0
0	0	0	0	0	0	0	0	0
0	0	1	0	0	0	0	0	0
0	0	1	0	0	0	0	0	1
0	0	0	0	0	0	0	0	0
0	0	1	0	0	0	0	0	0
0	0	1	0	0	0	0	0	0
0	0	0	0	0	0	0	0	0
0	0	1	0	0	0	0	0	0
0	0	0	0	0	0	0	0	0
0	0	1	0	0	1	0	0	0
0	0	1	0	0	0	0	0	0
0	0	0	0	0	0	0	0	0
0	0	0	0	0	0	0	0	0
0	0	0	0	0	0	0	0	0
0	0	0	1	0	0	0	0	0
0	0	0	0	0	0	0	0	0
0	0	0	0	0	0	0	0	0
0	0	0	0	0	0	0	0	0
0	0	0	0	0	0	0	0	0
0	0	0	0	0	0	0	1	0
0	0	1	0	1	0	0	0	0
0	0	0	0	0	0	0	0	0
0	0	0	1	0	0	0	0	0
0	0	0	0	0	0	0	0	0
0	0	1	0	0	0	0	0	0
0	0	1	1	0	0	0	0	0
0	0	1	0	0	0	0	0	0
0	0	1	0	0	0	0	0	0
0	0	0	0	0	0	0	0	0
0	0	0	0	0	0	0	0	0
0	0	0	0	1	0	0	0	0
0	0	0	0	1	0	0	0	0
0	0	0	0	0	0	0	0	1
0	0	0	0	0	0	0	0	0
0	0	1	0	0	0	0	0	0
0	0	0	0	0	0	0	0	0
0	0	1	0	0	0	0	0	0
0	0	0	1	0	0	0	0	0
0	0	0	0	0	0	0	0	0
0	0	1	0	0	0	0	0	0
0	0	0	1	0	0	0	0	0
0	0	0	0	0	0	0	0	0
0	0	0	0	1	0	0	0	0
0	0	1	0	0	0	0	0	0
0	0	0	0	0	0	0	0	0
0	0	0	0	0	0	0	0	0
0	0	1	0	1	0	0	0	0
0	0	1	0	0	0	0	1	0
0	0	0	0	0	0	0	0	0
0	0	0	0	0	0	0	0	0
1	0	0	0	0	0	0	0	0
0	0	1	0	1	0	0	0	0
0	0	0	1	0	0	0	0	0
0	0	0	1	0	0	0	0	0

0	0	0	0	0	0	0	0	0
1	0	0	1	0	0	0	0	0
0	0	0	1	0	0	0	0	0
0	0	1	0	0	0	0	0	0
0	0	0	0	0	0	0	0	0
0	0	0	0	0	0	0	0	0
0	0	1	0	0	0	0	0	0
0	0	0	1	0	0	0	0	0
1	0	0	0	0	0	1	0	0
1	0	0	0	0	1	0	1	0
0	0	0	0	0	1	0	0	1
0	0	0	0	0	0	0	0	0
1	0	0	0	0	0	0	0	0
0	0	0	0	0	0	0	0	0
0	0	0	0	0	0	0	0	1
1	0	0	0	0	0	0	0	0
1	0	0	0	0	0	0	0	0
0	0	0	0	0	0	0	0	0
0	0	0	0	0	0	0	0	0
0	0	0	0	0	0	0	0	0
0	0	0	0	0	0	0	0	0
0	0	0	0	0	0	0	0	0
0	0	0	1	0	0	0	0	0
1	0	1	0	1	1	0	0	0
0	0	1	0	1	0	0	0	0
0	0	0	0	0	0	0	0	0
0	0	1	0	0	0	0	0	0
0	0	0	0	0	0	0	0	0
0	0	0	0	0	0	0	0	0
0	0	0	0	0	0	0	0	0
1	0	0	0	0	0	0	0	0
0	0	0	0	1	0	0	0	0
1	0	0	0	0	0	0	0	0
0	0	1	1	0	1	0	0	0
0	0	0	0	0	0	0	0	0
0	0	0	0	0	0	0	0	0
0	0	1	0	0	0	0	0	0
0	0	1	0	1	0	0	0	0
0	0	0	0	0	0	0	0	0
1	0	0	0	0	0	0	0	0
0	0	0	0	0	0	0	0	0
0	0	0	0	0	0	0	0	0
0	0	1	0	0	0	0	0	0
0	0	0	0	1	0	0	0	0
0	0	0	0	0	0	0	0	0
0	0	1	0	0	0	0	0	0
1	0	1	0	0	0	0	0	0
1	0	0	0	0	0	0	0	0
0	0	0	0	0	0	0	0	0
0	0	0	1	0	0	0	0	0
0	0	0	0	0	0	0	0	0
1	1	1	0	0	0	0	0	0
0	0	0	0	0	0	0	0	0
0	0	0	0	0	1	0	0	0
1	0	1	0	0	0	0	0	0
0	0	0	0	0	0	0	0	0

0	0	1	0	0	0	0	0	0
0	0	0	0	0	0	0	0	0
0	0	0	0	0	0	0	0	0
0	0	0	0	0	0	0	0	0
0	0	0	0	0	0	0	0	0
0	0	0	0	0	0	0	0	0
0	0	0	0	0	0	0	0	1
0	0	0	0	0	0	1	0	0
0	0	0	0	0	0	0	0	0
0	0	0	0	0	0	0	0	0
0	0	1	0	0	0	0	0	0
0	0	0	0	0	0	0	0	0
0	0	0	0	0	0	0	0	0
0	0	0	0	0	0	0	0	0
0	0	0	0	0	0	0	0	0
1	0	0	0	0	0	0	0	0
0	0	0	0	0	0	0	0	1
1	0	0	0	0	0	0	0	0
0	0	1	1	0	0	0	0	0
0	0	0	0	0	0	0	0	0
0	0	0	1	0	0	0	0	0
0	0	0	0	0	0	0	0	0
0	0	0	0	0	0	0	0	0
0	0	0	0	0	0	0	0	0
0	0	0	0	0	0	0	0	0
0	0	0	0	0	0	0	0	0
0	0	0	0	0	0	0	0	0
0	0	0	0	0	0	0	0	1
0	0	0	0	0	0	0	0	0
1	0	0	0	0	0	0	0	0
0	0	0	0	0	0	0	0	0
0	0	0	0	0	0	0	1	0
0	0	1	0	0	0	0	0	0
0	0	0	0	0	0	0	0	0
0	0	0	0	0	0	0	0	0
0	0	0	0	0	0	0	0	0
0	0	1	1	0	0	0	0	0
0	0	1	0	0	0	0	0	0
0	0	1	0	0	0	0	0	0
0	0	0	0	0	0	0	0	0
0	0	0	0	0	0	0	0	0
0	0	0	0	0	0	0	0	0
0	0	0	0	0	0	0	0	0
0	0	0	0	0	0	0	0	0
0	1	1	0	0	0	0	0	0
0	0	0	0	0	0	0	0	0
0	0	0	0	0	0	0	0	0
0	0	0	0	0	0	0	0	1
0	0	1	0	0	0	0	0	0
0	0	0	0	0	0	0	0	0
0	0	0	0	0	0	0	0	0
0	0	0	0	0	0	1	0	0
0	0	0	0	1	0	0	0	0
0	0	0	0	0	0	0	0	0
0	0	0	0	0	0	0	0	0
0	0	1	0	0	0	0	0	0

0	0	1	0	0	0	0	0	0
0	0	0	0	0	0	0	0	1
0	0	0	0	0	0	0	0	0
0	0	1	0	0	0	0	0	0
0	0	0	0	0	0	0	0	0
0	0	0	0	0	0	0	0	0
0	0	0	1	0	0	0	0	0
0	0	0	0	0	0	0	0	0
0	0	0	0	0	0	0	1	0
0	0	0	0	0	0	0	0	0
0	0	0	0	0	0	0	0	0
0	0	1	0	0	0	0	0	0
0	0	0	0	0	0	0	0	0
0	0	0	0	0	0	0	0	0
0	0	0	0	0	0	0	0	0
0	0	0	0	0	0	0	1	0
0	0	0	0	0	0	0	1	0
0	0	0	0	0	0	0	0	0
0	0	1	0	0	0	0	0	0
0	0	0	0	0	0	0	0	0
0	0	0	0	0	0	0	0	0
0	0	0	0	0	0	0	0	0
0	0	1	0	0	0	0	0	0
0	0	0	0	0	0	0	0	0
0	0	0	0	0	0	0	0	0
0	0	0	0	0	0	0	0	0
0	0	0	0	0	0	0	0	0
0	0	0	0	0	0	0	0	0
0	0	0	0	0	0	0	0	0
0	0	0	0	0	0	0	0	0
0	0	0	0	0	0	0	0	0
0	0	0	0	0	0	0	0	0
0	0	0	0	0	0	0	0	0
0	0	1	0	0	0	0	0	0
0	0	1	0	0	0	0	0	0
0	0	1	0	0	0	0	0	1
0	0	0	0	0	0	0	0	0
0	0	0	0	0	0	0	0	0
0	0	0	0	0	0	0	0	0
0	0	0	0	0	0	0	0	0
0	0	1	0	0	0	0	0	0
0	0	0	0	0	0	0	0	0
0	0	0	0	0	0	0	0	0
0	1	0	0	0	0	0	0	0
0	0	1	0	0	0	0	0	0
0	0	0	0	0	0	0	0	0
0	1	0	0	0	0	0	0	0
0	0	1	0	0	0	0	0	1
0	0	1	0	0	0	0	0	0
0	0	0	0	0	0	0	0	0
0	0	1	0	0	0	0	0	0
0	0	0	0	0	0	0	0	0
0	0	0	0	0	0	0	0	0
0	0	0	0	0	0	0	0	0
0	0	0	0	0	0	0	0	0
0	0	1	0	0	0	0	0	0

0	0	1	0	0	0	0	0	0
0	0	0	0	0	0	0	0	0
0	0	0	0	0	0	0	0	0
0	0	0	0	0	0	0	0	0
0	0	0	0	0	0	0	0	0
0	0	0	0	0	0	0	0	0
0	0	0	0	0	0	0	0	0
0	0	0	0	0	0	0	0	0
0	0	0	0	0	0	0	0	0
0	0	0	0	0	0	0	0	0
0	0	0	0	0	0	0	0	0
0	0	0	0	0	0	0	0	0
0	0	1	0	0	0	0	0	0
0	0	0	0	0	0	0	0	1
0	0	1	1	0	0	0	0	0
0	0	1	0	0	0	0	0	0
0	0	0	0	0	0	0	0	0
0	0	0	0	0	0	0	0	0
0	0	1	0	0	0	0	0	0
0	0	0	0	0	0	0	1	0
0	0	0	0	0	0	0	0	0
0	0	1	0	0	0	0	0	0
0	0	0	0	0	0	0	0	0
0	0	0	0	0	0	0	0	0
0	0	0	0	0	0	0	0	0
0	0	0	0	0	0	0	0	0
0	0	1	0	0	0	0	0	0
0	0	0	0	0	0	0	0	0
0	0	0	0	0	0	0	0	0
0	0	0	1	0	0	0	0	0
0	0	0	0	0	0	0	0	0
0	0	0	0	0	0	0	0	0
0	0	0	0	0	0	0	0	0
0	0	0	0	0	0	0	0	0
0	0	0	0	0	0	0	0	0
0	0	0	0	0	0	0	0	0
0	0	0	0	0	0	0	0	0
0	0	1	0	0	0	0	0	1
1	0	0	0	0	0	0	0	0
0	0	0	0	0	0	0	0	0
0	0	0	0	0	0	0	0	0
0	0	0	0	0	0	0	0	0
0	0	0	0	1	0	0	0	0
0	0	0	0	0	0	0	0	0
0	0	0	0	0	0	0	0	0
0	0	0	0	0	0	0	0	0
0	0	0	0	0	0	0	0	0
0	0	0	0	0	0	0	0	0
0	0	0	0	0	0	0	0	0
0	0	0	0	0	0	0	0	0
0	0	0	0	0	0	0	0	0
0	0	0	0	0	0	0	0	0
0	0	1	0	0	0	0	0	0
0	0	1	0	0	0	0	0	0
0	0	0	0	0	0	0	0	0
0	0	0	0	0	0	0	0	0

0	0	0	0	0	0	0	0	0
0	0	0	0	0	0	0	0	0
0	0	1	0	0	0	0	0	0
0	0	0	0	0	0	0	0	0
0	0	0	0	0	0	0	0	0
0	0	0	0	0	0	0	0	1
0	0	0	0	0	0	0	0	0
0	0	0	0	0	0	0	0	0
0	0	0	0	0	0	0	0	0
0	0	1	0	0	0	0	0	0
0	0	0	0	0	0	0	0	0
0	0	1	0	0	0	0	0	0
0	0	0	0	0	0	0	0	0
0	0	1	0	0	0	0	0	0
0	0	0	0	0	0	0	0	0
0	0	0	0	0	0	0	0	0
0	0	1	0	0	0	0	0	0
0	0	0	0	0	0	0	0	0
0	0	0	0	0	0	0	0	0
0	0	1	0	0	0	0	0	0
0	0	1	0	0	0	0	0	0
0	0	1	0	0	0	0	0	0
0	0	0	0	0	0	0	0	0
0	0	0	0	0	0	0	0	1
0	0	1	0	0	0	0	0	0
0	0	0	0	0	0	0	0	0
0	0	0	0	0	0	0	0	0
0	0	0	0	0	0	0	0	0
0	0	0	0	0	0	0	0	0
0	0	0	0	0	0	0	0	0
0	0	0	0	0	0	0	0	0
0	0	0	0	0	0	0	0	0
0	0	1	0	0	0	0	0	0
0	0	1	0	0	0	0	0	0
0	0	0	0	0	0	0	0	0
0	0	0	0	0	0	0	0	0
0	0	0	0	0	0	0	0	0
0	0	0	0	0	0	0	0	0
0	0	0	0	0	0	0	0	0
0	0	1	0	0	0	0	0	0
0	0	0	0	0	0	0	0	0
0	0	0	0	0	0	0	0	0
0	1	0	0	0	0	0	0	0
0	0	0	0	0	0	0	0	0
0	0	0	0	0	0	0	0	0
0	0	0	0	0	0	0	0	0
0	0	0	0	0	0	0	0	0
0	0	1	0	0	0	0	0	0
0	0	0	0	0	0	0	0	0
0	0	0	0	0	0	0	0	0
0	0	0	0	0	0	0	0	0
0	0	0	0	0	0	0	0	0
0	0	0	0	0	0	0	0	0
0	0	0	0	0	0	0	0	0
0	0	0	0	0	0	0	0	0
0	1	0	0	0	0	0	0	0

0	0	1	0	0	0	0	0	0
0	0	0	0	0	0	0	0	0
0	0	0	0	0	0	0	0	0
0	0	0	0	0	0	0	0	0
0	0	0	0	0	0	0	0	0
0	0	0	0	0	0	0	0	1
0	0	0	0	0	0	0	0	1
0	0	0	0	0	0	0	0	0
0	0	0	0	0	0	0	0	0
0	0	0	0	0	0	0	0	0
0	0	0	1	0	0	0	0	0
0	0	0	0	0	0	0	0	0
0	0	0	0	0	0	0	0	0
0	0	0	0	0	0	0	0	0
0	0	1	0	0	0	0	0	0
0	0	1	1	0	0	0	0	0
0	0	0	0	0	0	0	0	0
0	0	0	0	0	0	0	0	0
0	0	0	0	0	0	0	0	1
0	0	1	0	0	0	0	0	0
0	0	0	0	0	0	0	0	0
0	0	1	0	0	0	0	0	0
0	0	0	0	0	0	0	0	0
0	0	0	0	0	0	0	0	0
0	0	0	0	0	0	0	0	0
0	0	0	0	0	0	0	0	0
0	0	0	0	0	0	0	0	0
0	0	0	0	0	0	0	0	0
0	0	0	0	0	0	0	0	0
0	0	0	0	0	0	0	0	0
0	0	0	0	0	0	0	0	0
0	0	0	0	0	0	0	0	0
0	0	0	0	0	0	0	0	0
0	0	0	0	0	0	0	0	0
0	0	1	0	0	0	0	0	0
0	0	0	0	0	0	0	0	0
0	0	0	0	0	0	0	0	0
0	0	1	1	0	0	0	0	0
0	0	1	0	0	1	0	0	0
0	0	0	0	0	0	0	0	0
0	0	0	0	0	0	0	0	0
0	0	0	0	0	0	0	0	0
0	0	0	0	0	0	0	0	0
0	0	0	0	0	0	0	0	0
0	0	0	1	0	0	0	0	0
0	0	0	0	0	0	0	0	0
0	0	0	0	0	0	0	0	0
0	0	0	0	0	0	1	0	0
0	0	0	0	0	0	0	0	0
0	0	0	0	0	0	0	0	0
0	0	0	0	0	0	0	0	0
0	0	0	0	0	0	0	0	0
0	0	0	0	0	0	0	0	0
0	0	0	0	0	0	0	0	0
0	0	1	0	0	0	0	0	0
0	0	0	0	0	0	0	0	0

0	0	1	1	0	0	0	0	0
0	0	1	0	0	0	0	0	0
0	0	0	0	0	0	0	0	0
0	0	1	0	0	0	0	0	0
0	0	0	0	0	0	0	0	0
0	0	0	0	0	0	0	0	0
0	0	0	0	0	0	0	0	0
0	1	0	0	0	0	0	0	0
0	0	1	0	0	0	0	0	0
0	0	0	0	0	0	0	0	0
0	0	0	0	0	0	0	0	0
0	0	0	0	0	0	0	0	0
0	0	0	0	0	0	0	0	0
0	0	0	0	0	0	0	0	0
0	0	1	0	0	0	0	0	0
0	0	1	0	0	0	0	0	0
0	0	0	0	0	0	0	0	0
0	0	1	0	0	0	0	0	0
0	0	0	0	0	0	0	0	0
0	0	0	0	0	0	0	0	0
0	0	0	0	0	0	0	0	0
0	0	0	0	0	0	0	0	0
0	0	0	0	0	0	0	0	0
0	0	1	0	0	0	0	0	0
0	0	1	0	0	0	0	0	0
0	0	0	0	0	0	0	0	0
0	0	0	0	0	0	0	0	0
0	0	1	0	0	0	0	0	0
0	0	0	0	0	0	0	0	0
0	0	0	0	0	0	0	0	0
0	0	0	0	0	0	0	0	0
0	0	1	0	0	0	0	0	1
0	0	0	0	0	0	0	0	1
0	0	1	0	0	0	0	0	0
0	0	0	0	0	0	0	0	0
0	0	0	0	0	0	0	0	0
0	0	0	0	0	0	0	0	1
0	0	1	0	0	0	0	0	0
0	0	1	0	0	0	0	0	0
0	0	0	0	0	0	0	0	0
0	0	0	0	0	0	0	0	0
0	0	0	0	1	0	0	0	0
0	0	0	0	0	0	0	0	0
0	0	0	0	0	0	0	0	0
0	0	0	0	0	0	0	0	0
0	0	0	0	0	0	0	0	0
0	0	0	0	0	0	0	0	0
0	0	1	0	0	0	0	0	0
0	0	0	0	0	0	0	0	0
0	0	0	0	0	0	0	0	0
0	0	0	0	0	0	0	0	0
0	0	0	0	0	0	0	0	0
0	0	1	0	0	0	0	0	0
0	0	0	0	0	0	0	0	0
0	0	1	0	0	0	0	0	0
0	0	0	0	0	0	0	0	0

0	0	1	0	0	0	0	0	0
0	0	0	0	0	0	0	0	0
0	0	1	0	0	0	0	0	0
0	0	1	0	0	0	0	0	0
0	0	1	0	0	0	0	0	0
0	0	0	0	0	0	0	0	0
0	0	0	0	0	0	0	0	0
0	0	1	0	0	0	0	0	0
0	0	0	0	0	0	0	0	0
0	0	1	0	0	0	0	0	0
0	0	0	0	0	0	0	0	0
0	0	0	0	0	0	0	0	0
0	0	0	0	0	0	0	0	0
0	0	0	0	0	0	0	1	0
0	0	0	0	0	0	0	0	0
0	0	1	0	0	0	0	0	0
0	0	0	0	0	0	0	0	0
0	0	1	0	0	0	0	0	0
0	0	0	0	0	0	0	0	0
0	0	0	0	0	0	0	0	0
0	0	1	0	0	0	0	0	0
0	0	0	0	0	0	0	0	0
0	0	0	0	0	0	0	0	0
0	0	0	0	0	0	0	0	0
0	0	0	0	0	0	0	0	0
0	0	0	0	0	0	0	0	0
0	0	0	0	0	0	0	0	0
0	0	0	0	0	0	0	0	0
0	0	1	0	0	0	0	0	0
0	0	0	0	0	0	0	0	0
0	0	1	0	0	0	0	0	0
0	0	1	0	0	0	0	0	0
0	0	1	0	0	0	0	0	0
0	0	1	0	0	0	0	0	0
0	0	1	0	0	0	0	0	0
0	0	0	0	0	0	0	0	0
0	0	1	0	0	0	0	0	0
0	0	1	0	0	0	0	0	0
0	0	1	0	0	0	0	0	0
0	0	0	0	0	0	0	0	0
0	0	0	0	0	0	0	0	0
0	0	0	0	0	0	0	0	0
0	0	0	0	0	0	0	0	0
0	0	1	0	0	0	0	0	0
0	0	0	0	0	0	0	0	0
0	0	1	0	0	0	0	0	0
0	0	1	0	0	0	0	0	0
0	0	1	0	0	0	0	0	0
0	0	0	0	0	0	0	0	0
0	0	1	0	0	0	0	0	0
0	0	0	0	0	0	0	0	0
0	0	0	0	0	0	0	0	0
0	0	0	0	0	0	0	0	0
0	0	0	0	0	0	0	0	0
0	0	0	0	0	0	0	0	0
0	0	1	0	0	0	0	0	0

0	0	0	0	0	0	0	0	0
0	0	1	0	0	0	0	0	0
0	0	0	0	0	0	0	0	0
0	0	0	0	0	0	0	0	0
0	0	1	0	0	0	0	0	0
0	0	0	0	0	0	0	0	0
0	0	1	0	0	0	0	0	0
0	0	0	0	0	0	0	0	0
0	0	0	0	0	0	0	0	0
0	0	0	0	0	0	0	0	0
0	0	0	0	0	0	0	0	0
0	0	0	0	0	0	0	0	0
0	0	1	0	0	0	0	0	0
0	0	0	0	0	0	0	0	0
0	0	0	0	0	0	0	0	0
1	0	1	0	0	0	0	0	0
0	0	0	0	0	0	0	0	0
0	0	0	0	0	0	0	0	0
0	0	0	0	0	0	0	0	0
0	0	1	0	0	0	0	0	1
1	0	1	0	0	0	0	0	0
1	0	0	0	0	0	0	0	0
0	0	0	0	0	0	0	0	0
0	0	0	0	0	0	0	0	0
0	0	0	0	0	0	0	0	0
0	0	0	0	0	0	0	0	0
1	0	0	0	0	0	0	0	0
0	0	1	0	0	0	0	0	0
0	0	0	0	0	0	0	0	0
0	0	0	0	0	0	0	0	0
0	0	1	0	0	0	0	0	0
0	0	1	0	0	0	0	0	0
0	0	1	1	0	0	0	0	0
1	0	0	0	0	0	0	0	0
0	0	0	0	0	0	0	0	0
0	0	0	0	0	0	0	0	0
0	0	0	0	0	0	0	0	0
1	0	0	0	0	0	0	0	0
0	0	0	0	0	0	0	0	0
0	0	0	0	0	0	0	0	0
0	0	0	0	0	0	0	0	0
0	0	1	0	0	0	0	0	0
0	0	0	0	0	0	0	0	0
1	0	0	0	0	0	0	0	0
0	0	0	0	0	0	0	0	0
0	0	0	0	0	0	0	0	1
1	0	0	0	0	0	0	0	0
1	0	1	0	0	0	0	0	0
0	0	0	0	0	0	0	0	0
1	0	0	0	0	0	0	0	0
0	0	0	0	0	0	0	0	0
0	0	1	0	0	0	0	0	0
0	0	1	0	0	0	0	0	0
0	0	0	0	0	0	0	0	0
0	0	1	0	0	0	0	0	0
1	0	0	0	0	0	0	0	0

0	0	0	0	0	0	0	0	0
0	0	0	0	0	0	0	0	0
0	0	0	0	0	0	0	0	0
0	0	0	0	0	0	0	0	1
0	0	0	0	0	0	0	0	0
0	0	0	0	0	0	0	0	0
0	0	0	0	0	0	0	0	0
1	0	0	0	0	0	0	0	0
1	0	1	0	0	0	0	0	0
0	0	0	0	0	0	0	0	0
1	0	0	0	0	0	0	0	0
1	0	0	0	0	0	0	0	0
0	0	0	0	0	0	0	1	0
0	0	1	0	0	0	0	0	0
0	0	1	0	0	0	0	0	0
0	0	0	0	0	0	0	0	0
1	0	0	0	0	0	0	0	0
1	0	0	0	0	0	0	0	1
0	0	0	1	0	0	0	0	0
0	0	1	0	0	0	0	0	0
0	0	1	0	0	0	0	0	0
0	0	0	0	0	0	0	0	0
0	0	0	0	0	0	0	0	0
0	0	0	0	0	0	0	0	0
0	0	0	0	0	0	0	0	0
1	0	0	0	0	0	0	0	0
0	0	1	0	0	0	0	0	0
0	0	0	0	0	0	0	0	0
0	0	0	0	0	0	0	0	0
0	0	0	0	0	0	0	0	0
1	0	0	0	0	0	0	0	0
0	0	1	0	0	0	0	0	0
0	0	0	0	0	0	0	0	0
0	0	0	0	0	0	0	0	0
1	0	0	0	0	0	0	0	0
0	0	0	0	0	0	0	0	0
1	0	0	0	0	0	0	0	1
0	0	1	0	0	0	0	0	0
0	0	0	0	0	0	0	0	0
1	0	1	0	0	0	1	0	0
0	0	0	0	0	0	0	0	0
0	0	0	0	0	0	0	0	0
1	0	0	0	0	0	0	0	0
0	0	0	0	0	0	0	0	0
1	0	0	0	0	0	0	0	0
0	0	0	0	0	0	0	0	0
0	0	0	0	0	0	0	0	0
0	0	0	0	0	0	0	0	0
0	0	0	0	0	0	0	0	0
0	0	0	0	0	0	0	0	1
0	0	0	0	0	0	0	0	0
0	0	0	0	0	0	0	0	0
0	0	0	0	0	0	0	0	0
0	0	0	0	0	0	0	0	0
0	0	0	0	0	0	0	0	0
1	0	1	0	0	0	0	0	0

0	0	0	0	0	0	0	0	0
0	0	0	0	0	0	0	0	0
0	0	1	0	0	0	0	0	0
0	0	0	1	0	0	0	0	0
0	0	0	0	0	0	0	0	0
0	0	1	0	0	0	0	0	0
0	0	0	0	0	0	0	0	0
0	0	0	0	0	0	0	0	0
0	0	1	0	0	0	0	0	0
0	0	0	0	0	0	0	0	0
0	0	0	0	0	0	0	0	1
0	0	0	0	0	0	0	0	0
0	0	0	0	0	0	0	0	0
0	0	0	0	0	0	0	0	0
0	0	0	0	0	0	0	0	0
0	0	0	0	0	0	0	0	0
0	0	0	0	0	0	0	0	0
0	0	0	0	0	0	0	0	0
0	0	1	0	0	0	0	0	0
0	0	0	0	0	0	0	0	0
0	0	0	0	0	0	0	0	0
0	0	0	0	0	0	0	0	0
1	0	0	0	0	0	0	0	0
0	0	0	0	0	0	0	0	0
0	0	0	0	0	0	0	0	0
0	0	0	0	0	0	0	0	0
0	0	0	0	0	0	0	0	0
0	0	0	0	0	0	0	0	0
0	0	0	0	0	0	0	0	1
0	0	0	0	0	0	0	0	0
0	1	0	0	0	0	0	0	0
1	0	1	0	0	0	0	0	0
0	0	0	0	0	0	0	0	0
0	0	0	0	0	0	0	0	0
0	0	0	0	0	0	0	0	0
0	0	0	0	0	0	0	0	0
0	0	0	1	0	0	1	0	0
0	0	0	0	0	0	0	0	0
0	0	0	0	0	0	0	0	0
1	0	0	0	0	0	0	0	0
0	0	0	0	0	0	0	0	0
0	0	0	0	0	0	0	0	0
0	0	1	0	0	0	0	0	0
0	0	0	0	0	0	0	0	0
1	0	0	1	0	0	0	0	0
0	0	0	0	0	0	0	0	0
1	0	0	0	0	0	0	0	0
0	0	0	0	0	0	0	0	0
0	0	1	0	0	0	0	0	0
0	0	0	0	0	0	0	0	0
1	0	0	0	0	0	0	0	0
0	0	0	0	0	0	0	0	0
0	0	0	0	0	0	0	0	0
0	0	0	0	0	0	0	0	0
1	0	0	0	0	0	0	0	0
1	0	0	0	0	0	0	0	0

0	0	1	0	0	0	0	0	0
1	0	0	0	0	0	0	0	0
0	0	0	0	0	0	0	0	0
0	0	0	0	0	0	0	0	0
0	0	0	0	0	0	0	0	0
0	0	1	0	0	0	0	0	0
0	1	0	0	0	0	0	0	0
0	0	0	0	0	0	0	0	0
0	0	0	0	0	0	0	0	0
1	0	0	0	0	0	0	0	0
0	0	0	0	0	0	0	0	0
1	0	1	0	0	0	0	0	0
0	0	0	0	0	0	0	0	0
0	0	1	0	0	0	0	0	0
1	0	0	0	0	0	0	0	0
0	0	0	0	0	0	0	0	0
0	0	1	0	0	0	0	0	0
1	0	0	0	0	0	0	0	0
0	0	0	0	0	0	0	0	0
0	0	1	0	0	1	0	0	0
0	0	1	0	0	0	0	0	0
0	0	0	0	0	0	0	0	0
0	0	0	0	0	0	0	0	0
0	0	0	0	0	0	0	0	0
0	0	0	0	0	0	0	0	0
0	0	1	0	0	0	0	0	0
0	0	0	0	0	0	0	0	0
0	0	0	0	0	0	0	0	0
0	0	0	0	0	0	0	0	0
0	0	0	0	0	0	0	0	0
0	0	1	0	1	0	0	0	0
0	0	0	0	0	0	0	0	0
0	0	0	0	0	0	0	0	0
0	0	0	0	0	0	0	0	0
0	0	1	0	0	0	0	0	0
0	0	1	0	0	0	0	0	0
0	0	0	0	0	0	0	0	0
0	0	0	0	0	0	0	0	0
0	0	0	0	0	0	0	0	0
0	0	0	0	0	0	0	0	0
0	0	0	0	0	0	0	0	0
0	0	0	0	0	0	0	0	0
0	0	1	0	0	0	0	0	0
0	0	0	0	0	0	0	0	0
0	0	1	0	0	0	0	0	0
0	0	0	0	0	0	0	0	0
0	0	0	0	0	0	0	0	0
0	0	0	0	0	0	0	0	0
0	0	0	0	0	0	0	0	0
0	0	0	0	0	0	0	0	0
0	0	1	0	0	0	0	0	0
0	0	0	0	0	0	0	0	0
0	0	0	0	0	0	0	0	0
0	0	0	0	0	0	0	0	0
0	0	0	0	0	0	0	0	0
0	0	0	0	0	0	0	0	0

0	0	0	0	0	0	0	0	0
0	0	0	0	0	0	0	0	0
0	0	1	0	0	0	0	0	0
0	0	1	0	0	0	0	0	0
0	0	0	0	0	0	0	0	0
0	0	0	0	0	0	0	0	0
0	0	0	0	0	0	0	0	0
0	0	0	0	0	0	0	0	0
0	0	0	0	0	0	0	0	0
0	0	0	0	0	0	0	0	0
0	0	0	0	0	0	0	0	0
0	0	1	0	0	0	0	0	0
0	0	0	0	0	0	0	0	0
0	0	0	0	0	0	0	0	0
0	0	0	0	0	0	0	0	0
0	0	0	0	0	0	0	0	0
0	0	0	0	0	0	0	0	0
0	0	1	0	0	0	0	0	0
0	0	1	0	0	0	0	0	0
0	0	0	0	0	0	0	0	0
0	0	1	0	0	0	0	0	0
0	0	0	0	0	0	0	0	0
0	0	0	0	0	0	1	0	0
0	0	1	0	0	0	0	0	0
0	0	1	0	0	0	0	0	0
0	0	1	0	0	0	0	0	0
0	0	0	0	0	0	0	0	0
0	0	0	0	0	0	0	0	0
0	0	0	0	0	0	0	0	0
0	0	0	0	0	0	0	0	0
0	0	0	0	0	0	0	0	0
0	0	1	0	0	0	0	0	0
0	0	0	0	0	0	0	0	0
0	0	1	0	0	0	0	0	0
0	0	0	0	0	0	0	0	0
0	0	0	0	0	0	0	0	0
0	1	0	0	0	0	0	0	0
0	0	1	0	0	0	0	0	0
0	1	1	0	0	0	0	0	0
0	0	0	0	0	0	0	0	0
0	0	0	0	0	0	0	0	0
0	0	0	0	0	0	0	0	0
0	0	0	0	0	0	0	0	0
0	0	0	0	0	0	0	0	0
0	0	1	0	0	0	0	0	0
0	0	1	0	0	0	0	0	0
0	0	0	0	0	0	0	0	0
0	0	1	0	0	0	0	0	0
0	0	0	1	0	0	0	0	0
0	0	0	0	0	0	0	0	0
0	0	1	1	0	0	0	0	0
0	0	0	0	0	0	0	0	1
0	0	1	0	0	0	0	0	0
0	0	0	0	0	0	0	0	1
0	0	0	0	0	0	0	0	0
0	0	0	0	0	0	0	0	0

0	0	0	0	0	0	0	0	0
0	0	0	0	0	0	0	0	0
0	0	0	0	0	0	0	0	0
0	0	0	0	0	0	0	0	0
0	0	0	0	0	0	0	0	0
0	0	0	0	0	0	0	0	0
0	0	0	0	0	0	0	0	0
0	0	0	0	0	0	0	0	0
0	0	0	0	0	0	0	0	0
0	0	0	0	1	0	0	0	0
0	0	0	0	0	0	0	0	0
0	0	1	0	0	0	0	0	0
0	0	0	0	0	0	0	0	0
1	0	0	0	0	0	0	0	0
0	0	0	1	1	0	0	0	0
0	0	0	0	0	0	0	0	0
0	0	0	0	0	0	0	0	1
0	0	0	0	0	0	0	0	0
0	0	0	0	1	0	0	0	0
0	0	0	0	0	0	0	0	0
0	0	0	0	0	0	0	0	0
0	0	0	0	0	0	0	0	0
0	0	0	0	0	0	0	0	0
0	0	0	0	0	0	0	0	0
0	0	0	0	0	0	0	0	0
0	0	0	0	0	0	0	0	0
0	0	0	0	0	0	0	0	0
0	0	0	0	0	0	0	0	0
0	0	0	0	0	0	0	0	0
0	0	0	0	0	0	0	0	0
0	0	0	0	0	0	0	0	0
0	0	0	0	0	0	0	0	0
0	0	0	1	0	0	0	0	0
0	0	0	0	0	0	0	0	0
0	0	0	0	0	0	0	0	0
0	0	0	0	0	0	0	0	0
0	0	0	0	0	0	0	0	0
0	0	0	0	0	0	0	0	0
0	0	0	0	0	0	0	0	0
0	0	0	0	0	0	0	0	0
0	0	0	0	0	0	0	0	0
0	0	0	1	0	0	0	0	1
0	0	0	0	0	0	0	0	0
0	0	0	0	0	0	0	0	1
0	0	0	0	0	0	0	0	0
0	0	0	0	0	0	0	0	1
0	0	0	0	0	0	0	0	0
0	0	0	0	0	0	0	0	0
0	0	0	0	0	0	0	0	0
0	0	0	0	0	0	0	0	0
0	0	1	0	0	0	0	0	0
0	0	1	0	0	0	0	0	0
0	0	0	1	0	0	0	0	0
0	0	0	0	0	0	0	0	0
0	0	0	0	0	0	0	0	0
0	0	0	0	0	0	0	0	0

0	0	0	0	0	0	0	0	0
0	0	0	0	0	0	0	0	0
0	0	0	0	0	0	0	0	0
0	0	0	0	0	0	0	0	0
0	1	0	0	0	0	0	0	0
0	0	0	0	0	0	1	0	0
0	0	0	1	0	0	0	0	0
0	0	1	0	0	0	0	0	0
0	0	0	0	0	0	0	0	1
0	0	1	1	0	0	0	0	0
0	0	0	0	0	0	0	0	0
0	0	0	0	0	0	0	0	0
0	0	1	0	0	0	0	0	0
0	0	1	0	0	0	0	0	0
0	0	0	0	0	0	0	0	0
0	0	0	1	0	0	0	0	0
0	0	0	0	0	0	0	0	0
0	0	1	0	0	0	0	0	0
0	0	0	0	0	0	0	0	0
0	0	0	0	0	0	0	0	0
0	0	0	0	0	0	0	0	0
0	0	0	0	0	0	0	0	0
0	0	1	0	0	0	0	0	0
0	0	1	0	0	0	0	0	0
0	0	0	0	0	0	0	0	0
0	0	1	0	0	0	0	0	0
0	0	0	0	0	0	0	0	0
0	0	1	1	0	0	0	0	0
0	0	1	0	0	0	0	0	0
0	0	0	0	0	0	0	0	0
0	0	0	0	0	0	0	0	1
0	0	0	0	0	0	0	0	0
0	0	0	0	0	0	0	0	0
0	0	0	0	0	0	0	0	0
0	0	0	0	0	0	0	0	0
0	0	1	0	0	0	0	0	0
0	0	0	1	0	0	0	0	0
0	0	0	0	0	0	0	0	0
0	0	0	0	0	0	0	0	0
0	0	0	0	0	0	0	0	0
0	0	0	0	0	0	0	0	1
0	0	0	0	0	0	0	1	0
0	0	0	0	0	0	0	0	0
0	0	0	1	0	0	0	0	0
0	0	0	0	0	0	0	0	0
0	0	0	0	0	0	0	0	0
0	0	0	0	0	0	0	0	0
0	0	0	0	0	0	0	0	0
0	0	0	0	0	0	0	1	1
0	0	0	0	0	0	0	0	0
0	0	1	0	0	0	0	0	1
0	0	1	0	0	0	0	0	1
0	0	0	0	0	0	0	0	0
0	0	0	0	0	0	0	0	0
0	0	0	0	0	0	0	0	0
0	0	0	0	0	0	0	1	0

0	0	0	0	0	0	0	0	0
0	0	0	0	0	0	0	0	0
0	0	0	0	0	0	0	1	1
0	0	0	0	0	0	0	0	0
0	0	0	0	0	0	0	0	0
0	0	0	0	0	0	0	0	0
0	0	0	0	0	0	0	0	0
0	0	0	0	0	0	0	0	0
0	0	0	0	0	0	0	1	0
0	0	0	0	0	0	0	0	0
1	0	0	0	0	0	0	0	0
0	0	0	0	0	0	0	0	0
0	0	1	0	0	0	0	0	0
0	0	0	0	0	0	0	0	0
0	0	0	0	0	0	0	0	0
0	0	0	0	0	0	0	0	0
0	0	0	0	0	0	0	0	0
0	0	1	0	0	0	0	0	0
0	0	0	0	0	0	0	0	0
0	0	0	0	0	0	0	0	0
0	0	1	0	0	0	0	0	0
0	0	0	0	0	0	0	0	0
1	0	0	0	0	0	0	0	0
0	0	1	0	1	0	0	0	0
0	0	0	0	0	0	0	0	0
0	0	0	1	0	0	0	0	0
0	0	0	0	0	0	0	0	0
0	0	0	0	0	0	0	0	0
0	0	0	0	0	0	0	0	0
0	0	0	0	0	0	0	0	0
0	0	1	0	0	0	0	0	0
0	0	0	0	0	0	0	0	0
0	0	1	0	0	0	0	0	0
0	0	0	0	0	0	0	0	0
0	0	0	0	0	0	0	0	0
0	0	1	0	0	0	0	0	1
0	0	0	0	0	0	0	0	0
0	0	1	0	0	0	0	0	0
0	0	0	1	0	0	0	0	0
0	0	0	0	0	0	0	0	0
0	0	1	0	0	0	0	0	0
0	0	0	0	0	0	0	0	0
0	0	0	0	0	0	0	0	0
0	0	0	0	0	0	0	0	0
0	0	0	0	0	0	0	0	0
0	0	0	0	0	0	0	0	0
0	0	0	0	0	0	0	0	0
0	0	1	0	0	0	0	0	0
0	0	0	0	0	0	0	0	1
0	0	0	0	0	0	0	0	0
0	0	1	0	0	0	0	1	0
0	0	0	0	0	0	1	0	0
0	0	0	0	0	0	0	0	0
0	0	0	0	0	0	0	0	1
0	0	0	0	0	0	0	0	0
0	0	0	0	0	0	0	0	0

0	0	0	0	0	0	0	0	0
0	0	0	1	0	0	0	0	0
0	0	0	0	0	0	0	0	0
0	0	1	0	0	0	0	0	0
0	0	0	0	0	0	0	0	0
0	0	0	0	0	0	0	0	1
0	0	0	0	0	0	0	0	0
0	0	1	0	0	0	0	0	0
0	0	0	0	0	0	0	0	0
0	0	0	0	0	0	0	0	1
0	0	0	0	0	0	0	0	0
0	0	0	0	0	0	0	0	0
0	0	0	0	0	0	0	0	0
0	0	0	0	0	0	0	0	0
0	0	1	0	0	0	0	0	0
0	0	0	1	0	0	1	0	0
0	0	0	0	0	0	0	0	0
0	0	0	0	0	0	0	0	0
0	0	0	0	0	0	0	0	0
0	0	0	1	0	0	0	0	0
0	0	0	1	0	0	0	0	0
0	0	0	0	0	0	0	0	0
0	0	0	0	0	0	0	0	0
0	0	0	0	0	0	0	0	0
0	0	0	0	0	0	0	0	0
0	0	0	0	0	0	0	0	0
0	0	0	0	0	0	0	0	0
0	0	1	0	0	0	0	0	0
0	0	0	0	0	0	0	0	0
0	0	0	0	0	0	0	0	0
0	0	0	0	0	0	0	1	1
0	0	0	0	0	0	0	0	0
0	0	0	0	0	0	0	0	0
0	0	1	0	0	0	0	0	0
0	0	0	0	1	0	0	0	0
0	0	0	1	0	0	0	0	0
0	0	0	0	0	0	0	0	0
0	0	1	0	0	0	0	0	0
0	0	0	0	0	0	0	0	0
0	0	0	1	0	0	0	0	0
0	0	1	0	0	0	0	0	0
0	0	1	1	0	0	0	0	0
0	0	0	0	0	0	0	0	0
0	0	1	0	1	0	0	0	0
0	0	1	1	0	0	0	0	0
0	0	0	1	0	0	0	0	0
1	0	0	0	0	0	0	0	0
1	0	0	0	0	0	0	0	0
0	0	0	0	0	0	0	0	0
1	0	0	0	0	0	0	0	0
0	0	0	1	0	0	0	0	0
0	0	0	0	0	0	0	0	0
0	0	0	0	0	0	0	0	0
0	0	0	0	0	0	0	0	0
0	0	1	0	0	0	0	0	0
0	0	0	0	0	0	0	0	0

0	0	1	0	0	0	0	0	0
0	0	0	0	0	0	0	0	0
1	0	0	0	0	0	0	0	0
1	0	0	1	0	0	0	0	0
0	0	0	0	0	0	0	0	0
1	0	1	0	0	0	0	0	0
1	0	1	0	0	1	0	0	0
0	0	0	0	0	0	0	0	0
0	1	0	0	0	0	0	0	0
0	0	1	0	0	0	0	0	0
0	0	0	0	0	0	0	0	0
1	0	1	0	0	0	0	1	0
1	0	0	0	0	0	0	0	0
0	0	0	0	0	0	1	0	0
0	0	0	1	0	0	0	0	0
0	0	0	0	0	0	0	0	0
1	0	0	0	0	0	0	0	0
1	0	0	1	0	0	0	0	0
1	0	0	0	0	0	0	0	0
1	0	0	1	0	0	0	0	0
0	0	1	0	0	0	0	0	0
1	0	0	0	0	0	0	0	0
0	0	0	0	0	0	0	0	0
1	0	0	0	0	0	0	0	0
0	0	0	0	0	0	0	0	0
0	0	0	0	0	0	0	0	0
1	0	0	0	0	0	1	0	0
0	1	0	0	0	0	0	1	0
0	0	0	0	0	0	0	0	0
0	0	0	0	0	0	1	0	0
0	0	1	0	0	0	0	0	0
0	0	0	0	0	0	0	0	0
0	0	1	0	0	0	0	0	0
0	0	1	0	0	0	0	0	0
0	0	0	0	0	0	0	0	0
0	0	1	0	0	0	0	0	0
0	0	0	0	1	0	0	0	0
1	0	0	0	0	0	0	0	0
1	0	0	0	0	0	0	0	0
1	0	0	0	0	0	0	0	0
1	0	0	0	0	0	0	0	0
0	0	0	0	0	0	0	0	0
0	0	0	0	0	0	0	0	0
0	0	0	0	0	0	0	0	0
0	0	0	0	0	0	0	0	0
1	0	0	0	0	0	0	0	0
1	0	0	1	0	0	0	0	0
0	0	0	0	0	0	0	0	0
1	0	0	0	0	0	0	0	0
0	0	0	0	0	0	0	0	0
0	0	1	0	0	0	0	0	0
0	0	0	0	0	0	0	0	1
0	0	0	0	0	0	0	0	0
1	0	0	0	0	0	0	0	0
1	0	0	0	0	0	0	0	0
1	0	0	0	0	0	0	0	0

0	0	0	0	0	0	0	0	0
0	0	0	0	0	0	0	0	0
0	0	0	0	0	0	0	0	0
1	0	1	0	0	0	0	0	0
0	0	1	0	0	0	0	0	0
0	0	1	0	0	1	0	0	0
1	0	0	0	0	0	0	0	0
1	0	1	0	0	0	0	0	0
1	0	0	1	0	0	0	0	0
0	0	1	0	0	0	0	0	0
0	0	0	0	0	0	0	0	0
1	0	0	0	0	0	0	0	0
1	0	0	0	0	0	0	0	0
0	0	0	0	0	0	0	0	0
0	0	0	0	0	0	0	0	0
1	0	0	0	0	0	0	0	0
0	0	0	0	0	0	0	0	0
0	0	0	0	0	0	0	0	0
0	0	0	0	0	0	0	0	1
0	0	1	0	0	0	0	0	0
0	0	0	0	0	0	0	0	0
0	0	0	0	0	0	0	0	0
1	0	0	0	0	0	0	0	0
0	0	0	0	0	0	0	0	1
0	0	0	0	0	0	0	0	0
0	0	0	0	0	0	0	0	0
0	0	0	0	0	0	0	0	0
1	0	0	0	0	0	0	0	0
0	0	0	0	0	0	0	0	0
0	0	1	0	0	0	0	0	0
0	0	0	0	1	0	0	0	0
0	0	0	0	0	0	0	0	0
1	0	0	0	0	0	0	0	0
0	0	1	0	0	0	0	0	0
0	0	0	0	0	0	0	0	0
1	0	0	0	0	1	0	0	0
0	0	1	0	0	0	0	0	0
0	0	1	0	0	0	0	0	0
0	0	0	0	0	0	0	0	0
0	0	0	0	0	0	0	0	0
0	0	0	0	0	0	0	0	0
0	0	0	0	0	0	0	0	0
1	0	0	0	0	1	1	0	0
0	0	1	0	0	0	0	0	0
0	0	0	1	0	0	0	0	0
1	0	0	0	0	0	0	0	0
0	0	1	0	0	0	0	0	0
0	0	1	0	0	0	0	0	0
0	0	0	0	0	0	0	0	0
0	0	0	0	0	0	0	0	0
0	0	1	1	0	0	0	0	0
0	0	0	0	0	0	0	0	0
1	0	1	0	0	0	0	0	0
0	0	0	0	0	0	0	0	0
0	0	0	0	0	0	0	0	0
0	0	0	0	0	0	0	0	0

1	0	0	0	0	0	0	0	0
1	0	0	0	0	0	0	0	0
0	0	1	0	0	0	0	0	0
0	0	1	1	0	0	0	0	0
0	0	0	0	0	0	0	1	0
1	0	1	0	0	0	0	0	0
0	0	1	0	0	0	0	0	0
0	0	0	0	0	0	0	0	0
0	0	1	0	0	0	0	0	0
0	0	1	0	0	0	0	0	0
0	0	0	0	0	0	0	0	0
0	0	0	0	0	0	0	0	1
0	0	0	0	0	0	0	0	0
0	0	1	0	0	0	0	0	0
1	0	1	0	0	0	0	0	0
0	0	0	0	0	0	0	0	0
0	0	0	0	0	0	0	0	0
0	0	0	1	0	0	0	0	0
0	0	0	0	0	0	0	0	0
1	0	0	0	0	0	0	0	0
0	0	0	0	0	0	0	1	1
0	0	0	0	0	0	0	0	0
0	0	0	0	0	0	0	0	0
1	0	0	0	0	0	0	0	0
0	0	1	0	0	0	0	0	0
1	0	0	0	0	0	0	0	0
0	0	0	0	0	0	0	0	0
0	0	0	0	0	0	0	0	0
0	0	0	0	0	0	0	0	0
0	0	0	0	0	0	0	0	0
0	0	0	0	0	0	0	0	0
0	0	0	0	0	0	0	0	0
0	0	0	0	0	0	0	0	0
0	0	0	0	0	0	0	0	0
0	0	1	0	0	0	0	0	0
0	0	1	0	0	0	0	0	0
0	0	1	0	0	0	0	0	0
0	0	0	0	0	0	0	0	0
0	0	0	0	0	0	0	0	0
1	0	0	0	0	0	0	0	0
0	0	1	1	0	0	0	0	0
0	0	0	0	0	0	0	0	0
0	0	0	0	0	0	0	0	0
1	0	0	0	0	0	0	0	0
0	0	0	0	0	0	0	0	0
0	0	0	0	0	0	0	0	0
0	0	0	0	0	0	0	0	0
1	0	0	0	0	0	0	0	0
1	0	0	0	0	0	0	0	0
0	0	0	0	0	0	0	0	0
0	0	1	0	0	0	0	0	0
0	0	0	0	0	0	0	0	0
0	0	0	0	0	0	0	0	0
0	0	0	0	1	0	0	0	0
1	0	0	0	0	1	0	0	0
0	0	1	0	0	0	0	0	0

0	0	1	0	0	0	0	0	0
0	0	0	0	0	0	0	0	0
0	0	0	0	0	0	0	0	0
1	0	0	0	0	0	0	0	0
0	0	1	1	0	0	0	0	0
0	0	0	0	0	0	0	0	0
0	0	0	0	0	0	0	0	0
0	0	0	0	0	0	0	0	0
0	0	0	0	0	0	0	0	0
1	0	0	0	0	0	0	0	0
0	0	0	0	0	0	0	0	0
0	0	0	0	0	0	0	0	0
0	0	0	0	0	0	0	0	0
0	0	1	0	0	0	0	0	0
1	0	0	0	0	0	0	0	0
0	0	1	0	0	0	0	0	0
0	0	0	1	0	0	0	0	0
0	0	1	0	0	0	0	0	0
0	0	0	0	0	0	0	0	0
0	0	0	0	0	0	0	0	0
0	0	1	0	0	0	0	0	0
0	0	1	0	0	0	0	0	0
0	0	0	1	0	0	0	0	0
0	0	0	0	0	0	0	0	0
0	0	0	0	0	0	0	0	0
1	0	0	1	0	0	0	0	0
0	0	0	1	0	0	0	0	0
0	0	1	0	0	0	0	0	0
0	0	0	0	0	0	0	0	0
0	0	1	0	0	0	0	0	0
0	0	0	0	0	0	0	0	0
0	0	0	0	0	0	0	0	0
1	0	0	1	0	0	0	0	0
1	0	0	0	0	0	0	0	0
0	0	0	0	0	0	0	0	0
1	0	0	0	0	0	0	0	0
0	0	1	0	0	0	0	0	0
0	0	0	0	0	0	0	0	0
1	0	1	0	0	1	0	0	0
0	0	0	0	0	0	0	0	0
0	0	0	0	0	0	0	0	0
1	0	0	1	0	0	1	0	0
0	0	0	0	0	0	0	0	0
0	0	0	0	0	0	0	0	0
0	0	0	0	0	0	0	0	0
1	0	0	0	0	0	0	0	0
1	0	0	0	0	0	0	0	0
0	0	1	0	0	0	0	0	0
0	0	0	0	0	0	0	0	0
0	0	0	0	0	0	0	0	0
0	0	0	0	0	0	0	0	0
0	0	0	1	0	0	0	0	0
1	0	0	0	0	0	0	0	0
0	0	0	0	0	0	0	1	0
1	0	0	0	0	0	0	0	0
0	0	0	0	0	0	0	0	0

0	0	1	0	0	0	0	0	0
0	0	1	0	0	0	0	0	0
0	0	0	0	0	0	0	0	0
1	0	0	0	0	0	0	0	0
0	0	0	0	0	0	0	0	0
1	0	1	0	0	0	0	0	0
0	0	0	0	0	0	0	0	0
0	0	0	0	0	0	0	0	0
1	0	0	0	0	0	0	0	0
0	0	0	0	0	0	0	0	0
0	0	0	1	0	0	0	0	0
1	0	0	1	0	0	0	0	0
0	0	0	0	0	0	0	0	0
0	0	0	1	0	0	0	0	0
0	0	1	1	0	0	0	0	0
1	0	0	0	0	0	0	0	0
0	0	0	0	0	0	0	0	0
1	0	0	1	0	0	0	0	0
0	0	0	0	0	0	0	0	1
0	0	0	0	0	0	0	0	0
0	0	0	0	0	0	0	0	0
1	0	0	0	0	0	0	0	0
0	0	0	1	0	0	0	0	0
1	0	0	1	0	0	0	0	0
0	0	0	0	0	0	0	0	0
0	0	1	0	0	0	0	0	0
1	0	0	0	0	0	0	0	0
0	0	0	0	0	0	0	0	0
0	0	0	0	0	0	0	0	0
0	0	1	0	0	0	0	0	0
0	0	0	0	0	0	0	1	0
0	0	0	0	0	0	0	0	0
0	0	0	1	0	0	0	0	0
0	0	0	1	0	0	0	0	0
0	0	0	0	0	0	0	0	0
0	0	1	0	0	0	0	0	0
0	0	0	1	0	0	0	0	0
0	0	0	0	0	0	0	0	0
0	0	1	1	0	0	0	0	0
0	0	1	0	0	0	0	0	0
0	0	1	0	0	0	0	0	0
0	0	0	0	0	0	0	0	0
0	0	0	0	0	0	0	0	0
0	0	0	0	0	0	0	0	0
1	0	0	0	0	0	0	0	0
0	0	0	0	0	0	0	0	0
0	0	0	0	0	0	0	0	0
0	0	0	1	0	0	0	0	0
0	1	1	0	0	0	0	0	0
0	0	0	0	0	0	0	0	0
0	0	0	0	0	0	0	0	0
0	0	0	0	0	0	0	0	0
0	0	0	0	0	0	0	0	0
0	0	0	0	0	0	0	0	0
1	0	1	0	0	1	0	0	0
0	0	0	0	0	0	0	0	0

0	0	1	0	0	0	0	0	0
1	0	0	0	0	0	0	0	0
0	0	0	0	0	0	0	0	0
0	0	1	0	0	0	0	0	0
0	0	1	0	0	0	0	0	0
1	0	0	0	0	0	0	0	0
1	0	0	0	0	0	0	0	0
0	0	0	0	0	0	0	0	0
0	0	1	0	0	0	0	0	0
0	0	0	0	0	0	0	0	0
1	0	1	0	0	1	0	0	0
0	0	1	0	0	0	0	0	0
0	0	0	0	0	0	0	0	0
0	0	0	0	0	0	0	0	0
0	0	0	0	0	0	0	0	0
0	0	0	0	0	0	0	0	0
1	0	0	0	0	0	0	0	0
0	0	0	0	0	0	0	0	0
0	0	0	0	0	0	0	0	0
0	0	0	0	0	0	0	0	0
0	0	0	0	0	0	0	0	0
0	0	0	0	0	0	0	0	0
0	0	0	1	0	0	0	0	0
0	0	0	0	0	0	0	0	0
0	0	1	0	0	0	0	0	0
0	0	1	0	0	0	0	0	0
0	0	0	0	0	0	0	0	0
0	0	0	0	0	0	0	0	0
0	0	0	0	0	1	0	0	0
0	0	0	0	0	0	0	0	0
0	0	1	0	0	0	0	0	0
1	0	0	0	0	0	1	0	0
0	0	0	0	0	0	0	0	0
0	0	0	0	0	0	0	0	0
0	0	1	0	0	0	0	0	0
0	0	1	0	0	0	0	0	0
0	0	1	0	0	0	0	0	0
0	0	0	0	0	0	0	0	0
1	0	0	1	0	0	0	0	0
0	0	0	0	0	0	0	0	0
0	0	1	0	0	0	0	0	0
0	0	1	0	0	0	0	0	0
0	0	0	0	0	0	0	0	0
0	0	0	0	0	0	0	0	0
0	0	0	0	0	0	0	0	0
1	0	0	0	0	0	0	0	0
0	0	0	0	0	0	0	0	0
1	0	0	0	0	0	0	0	0
1	0	1	1	0	0	0	0	1
0	0	0	0	0	0	0	0	1
1	0	0	1	0	0	0	0	0
1	0	0	1	0	0	0	0	0
0	0	0	0	0	0	0	0	0
0	0	0	0	0	0	0	0	0
0	0	0	0	0	0	0	0	0
0	0	0	1	0	0	0	0	0

0	0	0	0	0	0	0	0	0
0	0	1	0	0	0	0	0	0
0	0	0	0	0	0	0	0	0
0	0	0	0	0	0	0	0	0
0	0	1	0	0	0	0	0	0
0	0	0	0	0	0	0	0	0
0	0	0	0	0	0	0	0	0
1	0	1	0	0	0	0	0	0
0	0	0	0	0	0	0	0	0
1	0	1	0	0	0	0	0	0
1	0	1	1	0	0	0	0	0
1	0	1	0	0	0	0	0	0
1	0	0	1	0	0	0	0	0
1	0	0	0	0	0	0	0	0
0	0	1	0	0	0	0	0	0
0	0	0	0	0	0	0	0	1
0	0	0	0	0	0	0	0	0
0	0	1	1	0	0	0	0	1
1	0	0	0	0	0	0	0	0
0	0	0	0	0	0	0	0	1
0	0	0	0	0	0	0	0	0
0	0	1	0	0	0	0	0	0
1	0	0	1	0	0	1	0	0
0	0	0	0	0	0	0	0	0
0	0	0	0	0	0	0	0	0
1	0	0	0	0	0	0	0	0
0	0	0	0	0	0	0	0	0
0	0	0	0	0	0	0	0	0
1	0	1	0	0	0	0	0	0
0	0	0	0	0	0	0	0	0
1	0	0	0	0	0	0	0	0
1	0	1	0	0	0	0	0	0
0	0	0	0	0	0	0	0	0
0	0	0	0	0	0	0	1	0
0	1	0	0	0	0	0	0	0
0	0	0	0	0	0	0	0	0
1	0	1	1	0	0	0	0	0
0	0	1	1	0	0	0	0	0
1	0	1	0	0	0	0	0	0
0	0	0	0	0	0	0	0	0
0	0	0	0	0	0	0	0	0
0	0	0	0	0	0	0	0	1
0	0	1	0	0	0	0	0	0
1	0	0	0	0	0	0	0	0
0	0	0	0	0	0	0	0	0
0	0	1	0	0	0	0	0	0
1	0	0	0	0	1	0	0	1
0	0	0	0	0	0	0	0	1
1	0	0	0	0	0	0	0	0
1	0	1	0	0	0	0	0	0
0	0	1	0	0	0	0	0	0
0	0	0	0	0	0	0	0	0
0	0	0	0	0	0	0	0	0
1	0	0	0	0	0	0	0	0
1	0	0	0	0	0	0	0	0
0	0	1	0	0	0	0	0	1

0	0	0	1	0	0	0	0	0
0	0	1	0	0	0	0	0	0
0	0	0	0	0	0	0	0	0
0	0	1	0	0	0	0	0	0
1	0	1	0	0	0	0	0	0
0	0	1	0	0	0	0	0	0
0	0	0	0	0	0	0	0	0
0	0	0	0	0	0	0	0	0
0	0	0	0	0	0	0	0	0
0	0	0	0	0	0	0	0	0
0	0	1	0	0	0	0	0	0
1	0	0	0	0	0	0	0	0
0	0	0	0	0	0	1	0	0
0	0	0	0	0	0	0	0	0
0	0	0	0	0	0	0	0	0
1	0	1	0	0	0	0	0	0
0	0	0	0	0	0	0	0	0
0	0	1	0	0	0	0	0	0
0	0	1	0	0	0	0	0	0
0	0	0	0	0	0	0	0	0
0	0	0	1	0	0	1	0	0
0	0	0	0	0	0	0	0	1
0	0	0	0	0	0	0	0	0
0	0	0	0	0	0	0	0	0
0	0	0	0	0	0	0	0	0
1	0	1	0	0	0	0	0	0
0	0	1	0	0	0	0	0	0
0	0	0	0	0	0	0	0	0
1	0	1	1	0	0	0	0	0
1	0	0	0	0	0	0	0	0
1	0	0	0	0	0	0	0	0
0	0	1	0	0	0	0	0	0
1	0	0	0	0	0	0	0	0
1	0	0	0	0	0	0	0	0
0	0	0	0	0	0	0	0	0
0	0	0	0	0	0	0	0	0
0	0	1	0	0	0	0	0	1
1	0	0	0	0	0	0	0	0
0	0	0	0	0	0	0	0	0
0	0	0	0	0	0	0	0	0
1	0	0	0	0	0	0	0	0
0	0	1	0	0	0	0	0	0
1	0	0	0	0	0	0	0	0
0	0	0	0	0	0	0	0	0
1	0	0	0	0	1	0	0	0
0	0	0	0	0	0	0	0	0
0	0	0	0	0	0	0	0	0
0	0	0	0	0	0	0	0	0
0	0	1	0	0	0	0	0	0
0	0	0	0	0	0	0	0	1
1	0	0	1	0	0	0	0	0
1	0	0	0	0	0	0	0	0
0	0	0	0	0	0	0	0	1
0	0	1	1	0	0	0	0	0
0	0	0	0	0	0	0	1	0
0	0	1	1	0	0	0	0	0

0	0	0	0	0	0	0	0	0
0	0	1	1	0	0	0	0	0
1	0	0	0	0	0	0	0	0
1	0	1	0	0	0	0	0	0
0	0	0	0	0	0	0	0	0
0	0	1	0	0	0	0	0	0
0	0	1	0	0	0	0	0	0
0	0	1	0	0	0	0	0	0
1	0	1	0	0	0	0	0	0
0	1	0	0	0	0	0	0	0
0	0	0	1	0	0	0	0	0
1	0	0	0	0	0	0	0	0
1	0	1	0	0	0	0	0	0
1	0	1	1	0	0	0	0	0
0	0	0	0	0	0	0	0	0
0	0	1	0	0	0	0	0	0
1	0	1	0	0	0	0	0	0
1	0	0	1	0	0	0	0	0
0	0	0	0	0	0	0	0	0
1	0	1	0	0	0	0	0	0
0	0	0	0	0	0	0	0	0
0	0	0	0	0	0	0	0	0
0	0	0	0	0	0	0	0	0
0	0	0	0	0	0	0	0	0
0	0	0	0	0	0	0	0	1
1	0	0	0	0	0	0	0	0
0	1	1	0	0	0	0	0	0
1	0	0	0	0	0	0	0	0
0	0	1	0	0	0	0	0	0
0	0	0	0	0	0	0	0	0
0	0	0	0	0	0	0	0	0
0	0	1	0	0	0	0	0	0
1	0	1	0	0	0	0	0	0
1	0	1	0	0	0	0	0	0
0	0	0	0	0	0	0	0	1
1	0	0	1	0	0	0	0	0
1	0	0	0	0	0	0	0	1
1	0	1	1	0	0	0	0	0
1	0	0	0	0	0	0	0	0
1	0	1	1	0	0	0	0	0
0	0	0	0	0	0	0	0	0
0	0	1	0	0	0	0	0	0
1	0	1	0	0	0	0	0	0
1	0	0	0	0	0	0	0	0
0	0	1	0	0	0	0	0	0
0	0	0	0	0	0	0	0	0
1	0	1	0	0	0	0	0	0
0	0	0	0	0	0	0	0	0
1	0	0	0	0	0	0	0	0
0	0	0	0	0	0	0	0	0
0	0	0	0	0	0	0	0	0
0	0	1	0	0	0	0	0	0
0	0	1	0	0	0	0	0	0
0	0	0	0	0	0	0	0	0
0	0	0	0	0	0	0	0	0
0	0	0	0	0	0	0	0	0

0	0	0	0	0	0	0	0	1
1	0	0	0	0	0	0	0	0
0	0	0	0	0	0	0	0	0
0	0	0	0	0	0	0	0	0
0	0	0	0	0	0	0	0	0
0	0	0	0	0	0	0	0	0
0	0	0	0	0	0	0	0	0
0	0	0	0	0	0	0	0	0
0	0	0	0	0	0	0	0	0
0	0	0	0	0	0	0	0	0
0	0	0	0	0	0	0	0	0
0	0	0	0	0	0	0	0	0
0	0	0	0	0	0	0	0	0
1	0	1	0	0	0	0	0	0
0	0	0	0	0	0	0	0	0
0	0	0	0	0	0	0	0	0
0	0	0	1	0	0	0	0	0
0	0	0	0	0	0	0	0	0
0	0	0	1	0	0	0	0	0
0	0	1	0	0	0	0	0	0
0	0	0	0	0	0	0	0	0
0	0	0	0	0	0	0	0	0
0	0	0	1	0	0	0	0	0
0	0	0	0	0	0	0	0	0
0	0	0	0	0	0	0	0	0
0	0	0	0	0	0	0	0	0
0	1	0	0	0	0	0	0	0
0	0	0	0	0	0	0	0	1
0	0	0	0	0	0	0	0	0
0	0	0	0	0	0	0	0	0
0	0	1	0	0	0	0	0	0
0	0	0	0	0	0	0	0	0
0	0	1	1	0	0	0	0	0
0	0	0	0	0	0	0	0	0
0	0	0	1	0	0	0	0	0
0	0	0	0	0	0	0	0	0
0	0	1	0	0	0	0	0	0
0	0	0	1	0	0	0	0	0
0	0	0	0	0	0	0	0	0
0	0	0	0	0	0	0	0	0
0	0	0	0	0	0	0	0	0
0	0	0	0	0	0	0	0	0
0	0	0	0	0	0	0	0	0
0	0	0	0	0	0	0	0	0
0	0	0	0	0	0	0	1	0
0	0	0	0	0	0	0	0	0
0	0	0	0	0	0	0	0	0
0	0	0	0	0	0	0	0	0
0	0	1	0	0	0	0	0	0
0	0	0	0	0	0	0	0	0
0	0	0	0	0	0	0	0	0
0	0	0	0	0	0	0	0	0
0	0	0	0	0	0	0	0	0
0	0	0	1	0	0	0	0	0
0	0	0	0	0	1	0	0	0
0	0	1	0	0	0	0	0	0

0	0	0	1	0	0	0	0	0
0	0	0	0	0	0	0	1	0
0	0	0	0	0	0	0	0	0
0	0	0	0	1	0	0	0	0
0	0	0	1	0	0	0	0	0
0	1	0	0	0	0	0	0	0
0	0	0	0	0	0	0	0	1
0	0	0	0	0	0	1	0	0
0	0	0	0	0	0	0	0	0
0	0	0	0	0	0	0	0	1
0	0	0	0	0	0	0	0	0
0	0	0	0	0	0	0	0	0
0	0	0	0	0	0	0	0	0
0	0	1	0	0	0	0	0	0
0	0	1	0	0	0	0	0	0
0	0	0	0	0	0	0	0	0
0	0	0	0	0	0	0	0	0
0	0	1	1	0	0	0	0	0
0	0	1	0	0	0	0	0	0
0	0	0	0	0	0	0	0	0
0	0	0	0	0	0	0	0	0
0	0	1	0	0	0	0	0	0
0	0	0	1	0	0	0	0	0
0	0	1	1	0	0	0	0	0
0	0	0	0	0	0	0	0	0
0	0	1	0	0	0	0	0	0
0	0	0	0	0	0	0	0	0
0	0	0	0	0	0	0	0	0
0	0	1	0	0	0	0	1	0
0	0	0	0	0	0	0	0	0
0	0	0	0	0	0	0	0	0
0	0	0	0	0	0	0	0	0
0	0	0	0	0	0	0	0	0
0	0	0	0	0	0	0	0	0
0	0	0	0	0	0	0	0	0
0	0	0	0	0	0	0	0	0
0	0	0	0	0	0	0	0	0
0	0	1	1	0	0	0	0	0
0	0	0	0	0	0	0	0	0
0	0	0	0	0	0	0	0	0
0	0	0	0	0	0	0	0	0
0	0	1	0	0	0	0	0	0
0	0	0	0	0	0	0	0	0
0	0	0	0	0	0	0	0	0
0	0	0	1	0	0	0	0	0
0	0	0	1	0	0	0	0	0
0	0	1	0	0	0	0	0	0
0	0	0	0	0	0	0	1	1
0	0	1	1	0	0	0	0	0
0	0	0	0	0	0	0	0	1
0	0	0	0	0	0	0	0	0
0	0	1	0	0	0	0	0	0
0	0	0	0	0	0	0	0	0
0	0	0	0	0	0	0	0	0
0	0	0	0	0	0	0	0	1
0	0	0	0	0	0	0	0	0

0	0	0	0	0	0	0	0	0
0	0	0	0	0	0	0	0	0
0	0	0	0	0	0	0	0	0
0	0	0	0	0	0	0	0	0
0	0	0	0	0	0	0	0	0
0	0	0	0	0	0	0	0	0
0	0	0	0	0	0	0	0	0
0	0	0	0	0	0	0	0	0
0	0	1	0	0	0	0	0	0
0	0	0	0	0	0	0	0	0
0	0	0	0	0	0	0	1	0
0	0	1	0	0	0	0	0	0
0	0	0	0	0	0	0	0	0
0	0	0	0	0	0	0	0	0
0	0	0	0	0	0	0	0	0
0	0	0	0	0	0	0	0	0
0	0	0	0	1	0	0	0	0
0	0	0	0	0	0	0	0	0
0	0	0	0	0	0	0	0	0
0	0	0	1	0	0	0	0	0
0	0	0	0	0	0	0	0	0
0	0	0	0	0	0	0	0	0
0	0	0	0	0	0	0	0	0
0	0	0	0	0	0	0	0	1
0	0	0	0	0	0	0	0	0
0	0	0	0	0	0	0	0	0
0	0	0	0	0	0	0	0	0
0	0	1	0	0	0	0	0	0
0	0	0	0	0	0	0	0	0
0	0	0	0	0	0	0	0	0
0	0	0	0	0	0	0	1	0
0	0	0	0	0	0	0	1	0
0	0	0	0	0	0	0	0	0
0	0	0	0	0	0	0	0	0
0	0	0	0	0	0	0	0	0
0	0	0	0	0	0	0	0	0
0	0	1	0	0	0	0	0	0
0	0	0	0	0	0	0	0	0
0	0	0	0	0	0	0	0	0
0	0	0	0	0	0	0	0	0
0	0	1	0	0	0	0	0	0
0	0	1	0	0	0	0	0	0
0	0	1	0	0	0	0	0	0
1	0	0	1	0	0	0	0	0
1	0	0	0	0	0	0	0	0
0	0	1	0	0	0	0	0	0
0	0	0	1	0	0	0	0	0
0	0	0	0	0	0	0	0	0
0	0	0	0	0	0	0	0	0
1	0	0	1	0	0	0	0	0
0	0	1	1	0	0	0	0	0
0	0	0	0	0	0	0	0	0
0	0	1	0	0	0	0	0	0
1	0	0	0	0	0	0	0	0
0	0	0	0	0	0	0	0	0
1	0	0	0	0	0	0	0	0

0	0	0	0	0	0	0	0	0
0	0	0	0	0	0	0	0	0
0	0	1	0	0	0	0	0	0
0	0	0	0	0	0	0	0	0
1	0	0	0	0	0	0	0	0
0	0	0	0	0	0	0	0	0
1	0	0	0	0	0	0	0	0
1	0	1	0	0	0	0	0	0
1	0	0	0	0	0	0	0	0
0	0	1	0	0	0	0	0	0
0	0	0	0	0	0	0	0	0
0	0	0	0	0	0	0	0	0
0	0	0	0	0	0	0	0	0
0	0	1	1	0	0	0	0	0
0	0	0	0	0	0	0	0	0
0	0	0	0	0	0	0	0	0
0	0	1	1	0	0	0	0	0
0	0	1	0	0	0	0	0	0
0	0	0	1	0	0	0	0	0
0	0	1	0	0	0	0	0	0
0	0	1	0	0	0	0	0	0
0	0	1	0	0	0	0	0	0
0	0	0	0	0	0	0	0	0
0	0	1	0	0	0	0	0	0
1	0	1	0	0	0	0	0	0
0	0	1	1	0	0	0	0	0
[truncated: 455,998 more chars]
